# Measuring routine childhood vaccination coverage in 204 countries and territories, 1980–2019: a systematic analysis for the Global Burden of Disease Study 2020, Release 1

**DOI:** 10.1016/S0140-6736(21)00984-3

**Published:** 2021-08-07

**Authors:** GBD 2020, Release 1, Vaccine Coverage Collaborators, Natalie C Galles, Patrick Y Liu, Rachel L Updike, Nancy Fullman, Jason Nguyen, Sam Rolfe, Alyssa N Sbarra, Megan F Schipp, Ashley Marks, Gdiom Gebreheat Abady, Kaja M Abbas, Sumra Wajid Abbasi, Hedayat Abbastabar, Foad Abd-Allah, Amir Abdoli, Hassan Abolhassani, Akine Eshete Abosetugn, Maryam Adabi, Abdu A Adamu, Olatunji O Adetokunboh, Qorinah Estiningtyas Sakilah Adnani, Shailesh M Advani, Saira Afzal, Seyed Mohammad Kazem Aghamir, Bright Opoku Ahinkorah, Sohail Ahmad, Tauseef Ahmad, Sepideh Ahmadi, Haroon Ahmed, Muktar Beshir Ahmed, Tarik Ahmed Rashid, Yusra Ahmed Salih, Yonas Akalu, Addis Aklilu, Chisom Joyqueenet Akunna, Hanadi Al Hamad, Fares Alahdab, Luciana Albano, Yosef Alemayehu, Kefyalew Addis Alene, Ayman Al-Eyadhy, Robert Kaba Alhassan, Liaqat Ali, Syed Mohamed Aljunid, Sami Almustanyir, Khalid A Altirkawi, Nelson Alvis-Guzman, Hubert Amu, Catalina Liliana Andrei, Tudorel Andrei, Adnan Ansar, Alireza Ansari-Moghaddam, Ippazio Cosimo Antonazzo, Benny Antony, Jalal Arabloo, Morteza Arab-Zozani, Kurnia Dwi Artanti, Judie Arulappan, Asma Tahir Awan, Mamaru Ayenew Awoke, Muluken Altaye Ayza, Ghasem Azarian, Ahmed Y Azzam, Darshan B B, Zaheer-Ud-Din Babar, Senthilkumar Balakrishnan, Maciej Banach, Simachew Animen Bante, Till Winfried Bärnighausen, Hiba Jawdat Barqawi, Amadou Barrow, Quique Bassat, Narantuya Bayarmagnai, Diana Fernanda Bejarano Ramirez, Tariku Tesfaye Bekuma, Habtamu Gebrehana Belay, Uzma Iqbal Belgaumi, Akshaya Srikanth Bhagavathula, Dinesh Bhandari, Nikha Bhardwaj, Pankaj Bhardwaj, Sonu Bhaskar, Krittika Bhattacharyya, Sadia Bibi, Ali Bijani, Antonio Biondi, Archith Boloor, Dejana Braithwaite, Danilo Buonsenso, Zahid A Butt, Paulo Camargos, Giulia Carreras, Felix Carvalho, Carlos A Castañeda-Orjuela, Raja Chandra Chakinala, Jaykaran Charan, Souranshu Chatterjee, Soosanna Kumary Chattu, Vijay Kumar Chattu, Fazle Rabbi Chowdhury, Devasahayam J Christopher, Dinh-Toi Chu, Sheng-Chia Chung, Paolo Angelo Cortesi, Vera Marisa Costa, Rosa A S Couto, Omid Dadras, Amare Belachew Dagnew, Baye Dagnew, Xiaochen Dai, Lalit Dandona, Rakhi Dandona, Jan-Walter De Neve, Meseret Derbew Molla, Behailu Tariku Derseh, Rupak Desai, Abebaw Alemayehu Desta, Deepak Dhamnetiya, Mandira Lamichhane Dhimal, Meghnath Dhimal, Mostafa Dianatinasab, Daniel Diaz, Shirin Djalalinia, Fariba Dorostkar, Bassey Edem, Hisham Atan Edinur, Sahar Eftekharzadeh, Iman El Sayed, Maysaa El Sayed Zaki, Muhammed Elhadi, Shaimaa I El-Jaafary, Aisha Elsharkawy, Shymaa Enany, Ryenchindorj Erkhembayar, Christopher Imokhuede Esezobor, Sharareh Eskandarieh, Ifeanyi Jude Ezeonwumelu, Sayeh Ezzikouri, Jawad Fares, Pawan Sirwan Faris, Berhanu Elfu Feleke, Tomas Y Ferede, Eduarda Fernandes, João C Fernandes, Pietro Ferrara, Irina Filip, Florian Fischer, Mark Rohit Francis, Takeshi Fukumoto, Mohamed M Gad, Shilpa Gaidhane, Silvano Gallus, Tushar Garg, Biniyam Sahiledengle Geberemariyam, Teshome Gebre, Birhan Gebresillassie Gebregiorgis, Ketema Bizuwork Gebremedhin, Berhe Gebremichael, Bradford D Gessner, Keyghobad Ghadiri, Mansour Ghafourifard, Ahmad Ghashghaee, Syed Amir Gilani, Ionela-Roxana Glăvan, Ekaterina Vladimirovna Glushkova, Mahaveer Golechha, Kebebe Bekele Gonfa, Sameer Vali Gopalani, Houman Goudarzi, Mohammed Ibrahim Mohialdeen Gubari, Yuming Guo, Veer Bala Gupta, Vivek Kumar Gupta, Reyna Alma Gutiérrez, Emily Haeuser, Rabih Halwani, Samer Hamidi, Asif Hanif, Shafiul Haque, Harapan Harapan, Arief Hargono, Abdiwahab Hashi, Shoaib Hassan, Mohamed H Hassanein, Soheil Hassanipour, Hadi Hassankhani, Simon I Hay, Khezar Hayat, Mohamed I Hegazy, Golnaz Heidari, Kamal Hezam, Ramesh Holla, Mohammad Enamul Hoque, Mostafa Hosseini, Mehdi Hosseinzadeh, Mihaela Hostiuc, Mowafa Househ, Vivian Chia-rong Hsieh, Junjie Huang, Ayesha Humayun, Rabia Hussain, Nawfal R Hussein, Segun Emmanuel Ibitoye, Olayinka Stephen Ilesanmi, Irena M Ilic, Milena D Ilic, Sumant Inamdar, Usman Iqbal, Lalu Muhammad Irham, Seyed Sina Naghibi Irvani, Sheikh Mohammed Shariful Islam, Nahlah Elkudssiah Ismail, Ramaiah Itumalla, Ravi Prakash Jha, Farahnaz Joukar, Ali Kabir, Zubair Kabir, Rohollah Kalhor, Zul Kamal, Stanley M Kamande, Himal Kandel, André Karch, Getinet Kassahun, Nicholas J Kassebaum, Patrick DMC Katoto, Bayew Kelkay, Andre Pascal Kengne, Yousef Saleh Khader, Himanshu Khajuria, Ibrahim A Khalil, Ejaz Ahmad Khan, Gulfaraz Khan, Junaid Khan, Maseer Khan, Moien AB Khan, Young-Ho Khang, Abdullah T Khoja, Jagdish Khubchandani, Gyu Ri Kim, Min Seo Kim, Yun Jin Kim, Ruth W Kimokoti, Adnan Kisa, Sezer Kisa, Vladimir Andreevich Korshunov, Soewarta Kosen, Barthelemy Kuate Defo, Vaman Kulkarni, Avinash Kumar, G Anil Kumar, Nithin Kumar, Alexander Kwarteng, Carlo La Vecchia, Faris Hasan Lami, Iván Landires, Savita Lasrado, Zohra S Lassi, Hankil Lee, Yeong Yeh Lee, Miriam Levi, Sonia Lewycka, Shanshan Li, Xuefeng Liu, Stany W Lobo, Platon D Lopukhov, Rafael Lozano, Ricardo Lutzky Saute, Muhammed Magdy Abd El Razek, Alaa Makki, Ahmad Azam Malik, Fariborz Mansour-Ghanaei, Mohammad Ali Mansournia, Lorenzo Giovanni Mantovani, Francisco Rogerlândio Martins-Melo, Philippa C Matthews, John Robert Carabeo Medina, Walter Mendoza, Ritesh G Menezes, Endalkachew Worku Mengesha, Tuomo J Meretoja, Amanual Getnet Mersha, Mohamed Kamal Mesregah, Tomislav Mestrovic, Bartosz Miazgowski, George J Milne, Andreea Mirica, Erkin M Mirrakhimov, Hamid Reza Mirzaei, Sanjeev Misra, Prasanna Mithra, Masoud Moghadaszadeh, Teroj Abdulrahman Mohamed, Karzan Abdulmuhsin Mohammad, Yousef Mohammad, Mokhtar Mohammadi, Abdollah Mohammadian-Hafshejani, Arif Mohammed, Shafiu Mohammed, Archisman Mohapatra, Ali H Mokdad, Mariam Molokhia, Lorenzo Monasta, Mohammad Ali Moni, Ahmed Al Montasir, Catrin E Moore, Ghobad Moradi, Rahmatollah Moradzadeh, Paula Moraga, Ulrich Otto Mueller, Sandra B Munro, Mohsen Naghavi, Mukhammad David Naimzada, Muhammad Naveed, Biswa Prakash Nayak, Ionut Negoi, Sandhya Neupane Kandel, Trang Huyen Nguyen, Rajan Nikbakhsh, Dina Nur Anggraini Ningrum, Molly R Nixon, Chukwudi A Nnaji, Jean Jacques Noubiap, Virginia Nuñez-Samudio, Vincent Ebuka Nwatah, Bogdan Oancea, Chimedsuren Ochir, Felix Akpojene Ogbo, Andrew T Olagunju, Babayemi Oluwaseun Olakunde, Obinna E Onwujekwe, Nikita Otstavnov, Stanislav S Otstavnov, Mayowa O Owolabi, Jagadish Rao Padubidri, Keyvan Pakshir, Eun-Cheol Park, Fatemeh Pashazadeh Kan, Mona Pathak, Rajan Paudel, Shrikant Pawar, Jeevan Pereira, Mario F P Peres, Arokiasamy Perianayagam, Marina Pinheiro, Majid Pirestani, Vivek Podder, Roman V Polibin, Richard Charles G Pollok, Maarten J Postma, Faheem Hyder Pottoo, Mohammad Rabiee, Navid Rabiee, Amir Radfar, Alireza Rafiei, Vafa Rahimi-Movaghar, Mosiur Rahman, Amir Masoud Rahmani, Setyaningrum Rahmawaty, Aashish Rajesh, Rebecca E Ramshaw, Priyanga Ranasinghe, Chythra R Rao, Sowmya J Rao, Priya Rathi, David Laith Rawaf, Salman Rawaf, Andre M N Renzaho, Negar Rezaei, Mohammad Sadegh Rezai, Maria Rios-Blancas, Emma L B Rogowski, Luca Ronfani, Godfrey M Rwegerera, Anas M Saad, Siamak Sabour, Basema Saddik, Mohammad Reza Saeb, Umar Saeed, Amirhossein Sahebkar, Mohammad Ali Sahraian, Nasir Salam, Hamideh Salimzadeh, Mehrnoosh Samaei, Abdallah M Samy, Juan Sanabria, Francesco Sanmarchi, Milena M Santric-Milicevic, Benn Sartorius, Arash Sarveazad, Brijesh Sathian, Monika Sawhney, Deepak Saxena, Sonia Saxena, Abdul-Aziz Seidu, Allen Seylani, Masood Ali Shaikh, Morteza Shamsizadeh, Pavanchand H Shetty, Mika Shigematsu, Jae Il Shin, Negussie Boti Sidemo, Ambrish Singh, Jasvinder A Singh, Smriti Sinha, Valentin Yurievich Skryabin, Anna Aleksandrovna Skryabina, Amin Soheili, Eyayou Girma Tadesse, Animut Tagele Tamiru, Ker-Kan Tan, Yohannes Tekalegn, Mohamad-Hani Temsah, Bhaskar Thakur, Rekha Thapar, Aravind Thavamani, Ruoyan Tobe-Gai, Hamid Reza Tohidinik, Marcos Roberto Tovani-Palone, Eugenio Traini, Bach Xuan Tran, Manjari Tripathi, Berhan Tsegaye, Gebiyaw Wudie Tsegaye, Anayat Ullah, Saif Ullah, Sana Ullah, Brigid Unim, Marco Vacante, Diana Zuleika Velazquez, Bay Vo, Sebastian Vollmer, Giang Thu Vu, Linh Gia Vu, Yasir Waheed, Andrea Sylvia Winkler, Charles Shey Wiysonge, Vahit Yiğit, Birhanu Wubale Yirdaw, Dong Keon Yon, Naohiro Yonemoto, Chuanhua Yu, Deniz Yuce, Ismaeel Yunusa, Mohammad Zamani, Maryam Zamanian, Dejene Tesfaye Zewdie, Zhi-Jiang Zhang, Chenwen Zhong, Alimuddin Zumla, Christopher J L Murray, Stephen S Lim, Jonathan F Mosser

## Abstract

**Background:**

Measuring routine childhood vaccination is crucial to inform global vaccine policies and programme implementation, and to track progress towards targets set by the Global Vaccine Action Plan (GVAP) and Immunization Agenda 2030. Robust estimates of routine vaccine coverage are needed to identify past successes and persistent vulnerabilities. Drawing from the Global Burden of Diseases, Injuries, and Risk Factors Study (GBD) 2020, Release 1, we did a systematic analysis of global, regional, and national vaccine coverage trends using a statistical framework, by vaccine and over time.

**Methods:**

For this analysis we collated 55 326 country-specific, cohort-specific, year-specific, vaccine-specific, and dose-specific observations of routine childhood vaccination coverage between 1980 and 2019. Using spatiotemporal Gaussian process regression, we produced location-specific and year-specific estimates of 11 routine childhood vaccine coverage indicators for 204 countries and territories from 1980 to 2019, adjusting for biases in country-reported data and reflecting reported stockouts and supply disruptions. We analysed global and regional trends in coverage and numbers of zero-dose children (defined as those who never received a diphtheria-tetanus-pertussis [DTP] vaccine dose), progress towards GVAP targets, and the relationship between vaccine coverage and sociodemographic development.

**Findings:**

By 2019, global coverage of third-dose DTP (DTP3; 81·6% [95% uncertainty interval 80·4–82·7]) more than doubled from levels estimated in 1980 (39·9% [37·5–42·1]), as did global coverage of the first-dose measles-containing vaccine (MCV1; from 38·5% [35·4–41·3] in 1980 to 83·6% [82·3–84·8] in 2019). Third-dose polio vaccine (Pol3) coverage also increased, from 42·6% (41·4–44·1) in 1980 to 79·8% (78·4–81·1) in 2019, and global coverage of newer vaccines increased rapidly between 2000 and 2019. The global number of zero-dose children fell by nearly 75% between 1980 and 2019, from 56·8 million (52·6–60·9) to 14·5 million (13·4–15·9). However, over the past decade, global vaccine coverage broadly plateaued; 94 countries and territories recorded decreasing DTP3 coverage since 2010. Only 11 countries and territories were estimated to have reached the national GVAP target of at least 90% coverage for all assessed vaccines in 2019.

**Interpretation:**

After achieving large gains in childhood vaccine coverage worldwide, in much of the world this progress was stalled or reversed from 2010 to 2019. These findings underscore the importance of revisiting routine immunisation strategies and programmatic approaches, recentring service delivery around equity and underserved populations. Strengthening vaccine data and monitoring systems is crucial to these pursuits, now and through to 2030, to ensure that all children have access to, and can benefit from, lifesaving vaccines.

**Funding:**

Bill & Melinda Gates Foundation.

## Introduction

The development and mass distribution of childhood vaccines has been one of the greatest public health achievements in history, underpinning marked progress in child survival and health outcomes worldwide.^1^, ^2^, ^3^, ^4^ Initiated by WHO in 1974, the Expanded Programme on Immunisation (EPI) spurred coordinated, country-level progress in routine vaccination (eg, diphtheria, tetanus, pertussis, measles, polio, and BCG), and laid the foundation for efforts to introduce new vaccines and further increase coverage over the following decades.^5^ National governments and global organisations continue to dedicate substantial resources to vaccines, with total spending on immunisation exceeding US$107 billion in low-income and middle-income countries alone from 2000 to 2017.^6^ The 2011–20 Global Vaccine Action Plan (GVAP) set forth various targets for childhood vaccination, such as reaching 90% coverage across all vaccines in national immunisation programmes by 2020.^7^ GVAP's successor, the Immunization Agenda 2030 (IA2030), further calls for increased and equitable access to all routine vaccines for everyone, proposing to halve the number of zero-dose children missed by current vaccination programmes in each country by 2030.^8^, ^9^

**Research in context**

**Evidence before this study**

Rigorous, comparable, and timely estimates of vaccine coverage are needed to inform vaccination policies, programmes, and investments. WHO and UNICEF gather country-reported administrative and household survey data each year, among other immunisation indicators, through the Joint Reporting Form for immunisation, and annually produce the WHO–UNICEF Estimates of National Immunization Coverage (WUENIC) for member states. This estimation process, which has been described as a rule-based approach combining heuristics with expert assessment and decisions, has some strengths, including familiarity for key stakeholders and the ability to integrate expert opinion on vaccine coverage and its drivers. Compared to statistical models, however, the WUENIC approach does not produce quantitative estimates of uncertainty and adjusts only for relatively large discrepancies between country-reported data and household survey coverage estimates. To the best of our knowledge, no other study provides systematic, internally consistent analyses of global, regional, and national vaccine coverage trends based on a statistical framework, by vaccine and over time.

**Added value of this study**

Drawing from the Global Burden of Diseases, Injuries, and Risk Factors Study (GBD) 2020, Release 1, our analysis provides annual estimates of routine vaccine coverage for 11 vaccine-dose combinations from 1980 to 2019 in 204 countries and territories. Our modelling approach incorporates time-varying and location-varying bias adjustments, leverages temporal trends and covariate relationships to estimate vaccine coverage in the absence of country-specific data, and quantifies uncertainty for all estimates. We use these coverage estimates and GBD population estimates to quantify the number of zero-dose children (ie, children who have never received a dose of a diphtheria-tetanus-pertussis [DTP] vaccine as a proxy) over time; measure progress towards the Global Vaccine Action Plan (GVAP) 2020 targets of at least 90% coverage across all childhood vaccines by 2019; and analyse the relationships between national-level vaccine coverage and sociodemographic development.

**Implications of all the available evidence**

Over the past four decades, global coverage of both longstanding and more newly available vaccines improved, and the number of zero-dose children declined by nearly 75% since 1980. Yet from 2010 to 2019, much of the world saw progress stagnate or even reverse course. Most locations fell below the 2020 GVAP target of achieving at least 90% coverage across vaccines in 2019, signalling the need to further expand programme reach of unvaccinated or under-vaccinated children. Associations between sociodemographic development and vaccine coverage varied, underscoring the importance of how vaccine programmes operate and reach target populations above and beyond development alone. Continuing to strengthen vaccine data systems and measurement approaches—and leveraging these inputs to inform programme investments and implementation—is crucial to ensure that all children have access to lifesaving vaccines.

Yet as the GVAP era ends and IA2030 begins, acute service delivery challenges have emerged, with the COVID-19 pandemic substantially affecting routine immunisation throughout the world in 2020.^10^, ^11^, ^12^ At this pivotal juncture, it is important to clearly understand where—and for which vaccines—gains and gaps in coverage occurred before the onset of COVID-19. Robust and comparable estimates of vaccine coverage over time are thus key inputs for evaluating progress towards GVAP targets and serve as a baseline for IA2030's ambitions.

Vaccination data can be sparse, subject to bias, and inconsistent, complicating coverage estimation at both national and global levels. Since 2000, the WHO–UNICEF Estimates of National Immunization Coverage (WUENIC)^13^ have compiled available data sources (ie, country-reported data and household surveys gathered through the Joint Reporting Form [JRF]) for all member states and produced annual coverage estimates, by vaccine and dose, based on prespecified heuristics and expert judgement.^13^, ^14^, ^15^ Although WUENIC's approach has its strengths, such as incorporating qualitative knowledge and engagement via country consultation processes, statistical models offer important advantages. For instance, in the WUENIC method, country-reported data are only calibrated to survey-based estimates when both datapoints are available for a given vaccine-country-year and a discrepancy of ten percentage points or more is observed between survey data and country-reported data.^15^ Statistical models can account more fully for trends in reporting bias, as well as synthesise discrepant data sources while accounting for data quality and precision, quantify uncertainty, and leverage trends in time and other predictors to improve estimates where data are sparse. Previous research has used statistical methods to quantify discrepancies in administrative versus survey-based coverage over time;^16^, ^17^ however, such work is generally limited to a subset of vaccines, locations, or years. To date, no past research has, to our knowledge, systematically estimated coverage across vaccines, over multiple decades, and by location for all countries within a cohesive statistical modelling framework.

Drawing from the Global Burden of Diseases, Injuries, and Risk Factors Study (GBD) 2020, Release 1 (GBD 2020 R1), we estimated coverage for 11 vaccine-dose combinations in 204 countries and territories from 1980 to 2019. These include the well established original EPI vaccines (diphtheria-tetanus-pertussis, first dose [DTP1] and third dose [DTP3] vaccines; measles-containing vaccine, first dose [MCV1]; BCG, first dose; and polio vaccine, third dose [Pol3]), alongside newer vaccines introduced into national immunisation schedules over the past four decades (hepatitis B vaccine, third dose [HepB3]; *Haemophilus influenzae* type b vaccine, third-dose [Hib3]; measles-containing vaccine, second dose [MCV2]; pneumococcal conjugate vaccine, third dose [PCV3]; rubella-containing vaccine, first dose [RCV1]; and completed rotavirus series, two or three doses [RotaC]). We utilised survey and administrative data on vaccine coverage via a multi-step modelling approach that includes bias adjustments for discordance between survey data and administrative data, and propagated uncertainty through each estimation step. Last, we did secondary analyses to further examine relationships between changes in sociodemographic development and vaccine coverage, and explored trends in those children who never received a DTP dose (referred to as zero-dose children,^9^, ^18^, ^19^ proxying for children currently not reached by routine immunisation programmes). This study offers a crucial benchmark for global patterns in vaccine-specific and overall childhood vaccination trends before COVID-19, strengthening our collective understanding of past progress and challenges as the world pursues greater equity in immunisation access and delivery. This report was produced as part of the GBD Collaborator Network and in accordance with the GBD protocol.

## Methods

### Overview

This analysis is part of the broader GBD 2020 R1, an update from GBD 2019.^20^, ^21^, ^22^ The Global Health Data Exchange (GHDx) will be updated simultaneously with the release of new GBD rounds; content in these resources will always be synchronous. This analysis complies with the Guidelines for Accurate and Transparent Health Estimates Reporting (GATHER) statement,^23^ with further information provided in the appendix (section 1). All data processing and modelling were done in R statistical software, with more details provided in the appendix (sections 2–4) by analytical step; the source code will be made accessible upon publication and data are available on the GHDx website.

### Data

We defined vaccine coverage as the proportion of children who received at least the stated vaccine dose (eg, DTP1) through a routine immunisation programme; we excluded campaign doses when possible. Of 3118 total sources reviewed, 975 unique sources from 1980 to 2019 were used in this analysis, resulting in 55 326 country-cohort-year-vaccine-dose-specific datapoints across vaccines (appendix; tables S1–S3). We primarily used the GHDx to collate available coverage data sources as described in the appendix (sections 2.1–2.3; figure S2). These sources included household surveys (eg, Demographic and Health Surveys, Multiple Indicator Cluster Surveys, other multi-country survey series, and country-specific surveys) and official country-reported coverage data from the JRF. We excluded sources without data on children aged 12–59 months (aside from country-reported data, which reflect target population ages) and sources that were not nationally representative (ie, geographically or focused on a subgroup of the target population) or did not include dose-specific vaccine coverage from at least one vaccine in or after the country-reported national introduction year. We then reviewed all vaccine coverage observations from sources meeting these criteria and excluded data obtained before introduction of each vaccine or judged to be implausible. Complete inclusion and exclusion criteria and details about all reviewed data sources are summarised in the appendix (section 2.3; figure S2, tables S1–S3). For survey data, children with either home-based records or parental recall indicating vaccine receipt were considered vaccinated. Where individual-level microdata were available, we estimated coverage as the proportion of vaccinated children by vaccine, dose, and age in years, accounting for survey design (appendix section 2.4). We extracted survey report tabulations if microdata were unavailable.

Age-cohort-specific coverage data from children aged 12–59 months were assigned to the year of expected vaccine receipt using country-specific vaccine schedules and vaccine introduction years reported through the JRF.^24^, ^25^ This approach aligns survey-based coverage estimates with those from country-reported data by cohort, facilitating adjustment for administrative bias.

### Administrative bias adjustment

To incorporate administrative data while accounting for potential biases,^26^ we implemented bias adjustments for official country-reported data from the JRF for DTP3, MCV1, BCG, and Pol3. We first estimated the magnitude of potential bias by location-year, using paired observations of survey-based coverage estimates and JRF data for the same year, vaccine, location, and target population cohort. We then modelled administrative bias using a multi-step approach: we first predicted bias using the Healthcare Access and Quality (HAQ) Index (a summary measure of health-care performance^27^) as a covariate in a two-stage random spline model, and then, for locations with available bias observations, we used spatiotemporal Gaussian process regression (ST-GPR)^20^ to better account for trends in bias (appendix section 3.4).

### Vaccine-specific coverage estimation

We modelled vaccine-specific coverage using ST-GPR,^20^ a statistical method that enables non-linear trend estimation and incorporates data uncertainty into final estimates (appendix section 3.2). We used the HAQ Index and GBD mortality estimates from conflict and terrorism per capita as covariates in the first stage of each model, along with a covariate based on country-reported stockouts or other disruptions derived from discontinuities in administrative data (appendix section 3.7).

Relative to DTP3, country-reported data on DTP1 were sparse or not routinely collected from 1980 to 2000. We developed a time-varying model to impute DTP1 from reported DTP3 coverage and trends in DTP1–3 dropout, and used continuation-ratio ordinal regression to ensure internal consistency between DTP1 and DTP3 for the full time period (appendix section 3.3).^28^

Since more recently introduced vaccines had comparatively less available data, we used the more data-rich DTP3 and MCV1 models to inform estimation of HepB3, Hib3, PCV3, RotaC, MCV2, and RCV1 coverage (appendix section 3.6). We modelled the coverage ratio of newer vaccines relative to reference vaccines (DTP3 or MCV1) on the basis of schedule similarity, using observations from both survey data and unadjusted country-reported data. We used ST-GPR to estimate full time series for each scale-up ratio, then multiplied these ratios by corresponding DTP3 or MCV1 estimates to produce final estimates for all newer vaccines while propagating uncertainty.

We assumed 0% coverage for each vaccine before its formal introduction in national immunisation schedules, with the exception of Hib3, PCV3, and RotaC in China. For these vaccines, which were available in private markets but not yet included in the national immunisation schedule, we constrained estimates using lot release data (China National Institutes for Food and Drug Control^29^ and Y Teng, Linksbridge SPC, personal communication); further details are provided in the appendix (section 3.8). For DTP3, MCV1, BCG, and Pol3, we used EPI onset information to indicate the introduction of these vaccines and assumed no children were vaccinated with these vaccines before their formal introduction. Where applicable (eg, for BCG), we also assumed 0% coverage for eligible cohorts after removal of a vaccine from national immunisation schedules.

To compute 95% uncertainty intervals (UIs) for location-year-vaccine estimates, we sampled 1000 random draws from the modelled posterior distribution and took the ordinal 2·5th and 97·5th percentile of draws for each measure. National estimates were aggregated to GBD super-regions, groupings based on geographical proximity and epidemiological similarity,^30^ using GBD 2020 R1 estimates of target age group populations (updated from GBD 2019^22^ as part of the GBD continuous update cycle).

### Assessing coverage trends in relation to sociodemographic development, GVAP target attainment, and zero-dose children

Using these coverage estimates, we did three additional analyses. First, we sought to examine relationships between vaccine coverage and sociodemographic development, a type of benchmarking exercise used to identify potential performance outliers and where improvements in health are occurring faster or more slowly than parallel changes in development.^22^, ^31^, ^32^ For our development metric we used the GBD's Socio-demographic Index (SDI), a summary measure on a scale of 0–100 based on average income per capita, educational attainment, and fertility rates in a given location and year.^22^ We applied a constrained mixed-effects meta-regression^33^ to quantify global averages of expected coverage—estimated levels for any value of SDI—for DTP3, MCV1, and Pol3 across all location-years. We then compared country-level vaccine coverage and SDI estimates from 1980 to 2019 relative to these global averages of expected coverage on the basis of SDI alone. Further details are provided in the appendix (section 4.1).

Second, we evaluated progress towards the 2020 GVAP target of at least 90% national-level coverage across vaccines.^7^ We defined target attainment as a mean coverage estimate of 90% or higher, by vaccine and for all assessed vaccines, in 2010 and 2019. To ensure comparability across vaccines and years, our main analysis included nine vaccines for each location and year, irrespective of vaccine introduction. We then repeated this analysis considering only vaccines included in each location's national immunisation schedule in the year of evaluation (appendix section 4.2).

Third, we estimated the number of zero-dose children as a function of DTP1:

$$DTPO_{iy}=(1-DTP1_{iy})\times p_{iy}$$

where *DTP*0 is the number of children younger than 1 year of age without any DTP doses for location *i* and year *y, DTP*1 is DTP1 coverage, and *p* is the GBD 2020 R1 population estimate of children younger than 1 year. We used children who did not receive a first dose of a DTP-containing vaccine as a proxy for zero-dose children not reached by routine immunisation services, following the draft IA2030 implementation framework (which has since been published)^9^ and previous studies;^18^ additional details are provided in the appendix (section 4.3).

### Comparison with WUENIC estimates

We calculated concordance correlation coefficients for each vaccine, comparing coverage estimates to those produced by WUENIC for each location-year. We also aggregated country-level WUENIC coverage estimates to the global level (weighted means using GBD 2020 R1 target population estimates, to provide more direct comparisons with this study's global estimates; appendix section 4.4, table S8).

### Role of the funding source

The funder of this study had no role in study design, data collection, data analysis, data interpretation, or the writing of the report.

## Results

### Overview

In this section, we present global results and results by GBD super-region. Owing to BCG phase-out or non-introduction in 46 countries as of 2019, the results presented here focus on the remaining vaccines. We present DTP3 coverage estimates for consistency with other vaccines delivered as a three-dose primary series, while using DTP1 coverage to estimate counts of zero-dose children. Coverage estimates by vaccine, including BCG and DTP1, are available for each location from 1980 to 2019, with corresponding WUENIC estimates, in the appendix (figures S18–S28, S31, table S7) and on the GHDx website.

### Historical trends and progress in routine vaccination

**Figure 1:**
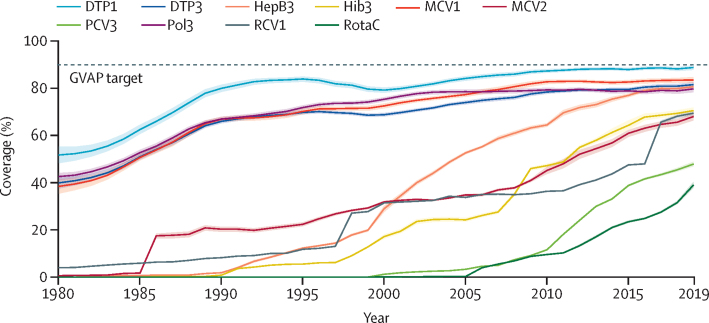
Global vaccine coverage by vaccine, 1980–2019 The dotted line represents the GVAP target of reaching at least 90% coverage by 2020. The solid lines represent the mean estimates for each vaccine. The lighter-coloured shading surrounding the solid lines represents the 95% uncertainty intervals. GVAP=Global Vaccine Action Plan. DTP1=diphtheria-tetanus-pertussis vaccine, first dose. DTP3=diphtheria-tetanus-pertussis vaccine, third dose. HepB3=hepatitis B vaccine, third dose. Hib3=*Haemophilus influenzae* type b vaccine, third dose. MCV1=measles-containing vaccine, first dose. MCV2=measles-containing vaccine, second dose. PCV3=pneumococcal conjugate vaccine, third dose. Pol3=polio vaccine, third dose. RCV1=rubella-containing vaccine, first dose. RotaC=completed rotavirus series.

Globally, vaccine coverage markedly increased from 1980 to 2019 (figure 1). MCV1 coverage more than doubled, rising from 38·5% (95% UI 35·4–41·3) in 1980 to 83·6% (82·3–84·8) in 2019. During this time, DTP3 coverage also increased from 39·9% (37·5–42·1) to 81·6% (80·4–82·7), and Pol3 coverage rose from 42·6% (41·4–44·1) to 79·8% (78·4–81·1).

**Figure 2:**
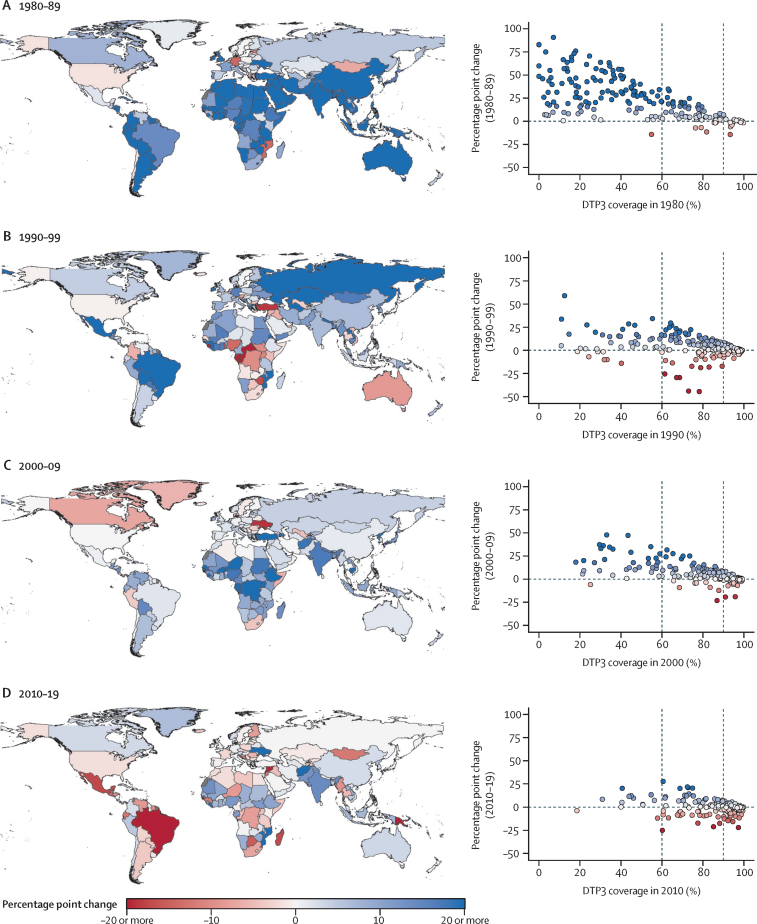
Changes in DTP3 coverage, by decade, from 1980 to 1989 (A), 1990 to 1999 (B), 2000 to 2009 (C), and 2010 to 2019 (D) For each decade, maps (left) and scatterplots (right) are colour-coded to reflect absolute changes in DTP3 coverage. Locations not estimated for GBD are shown in grey. Each circle on the scatterplot represents a country or territory, and the same colour is reflected on the corresponding map. For the scatterplots, the horizontal dashed line at 0 indicates no coverage change within the decade; the vertical dashed lines mark 60% and 90% coverage, with the latter being the national GVAP target for 2020. DTP3=diphtheria-tetanus-pertussis vaccine, third dose. GBD=Global Burden of Diseases, Injuries, and Risk Factors Study. GVAP=Global Vaccine Action Plan.

Overall, progress for these routine vaccines was most rapid from 1980 to 1989 (figure 2). For DTP3 specifically, global coverage increased from 39·9% (95% UI 37·5–42·1) to 64·2% (63·3–65·1). All GBD super-regions saw DTP3 coverage rise, with universal country-level gains in Latin America and the Caribbean, north Africa and the Middle East, and south Asia. These improvements were concentrated among locations starting with lower coverage: for instance, of 123 countries with DTP3 coverage lower than 60% in 1980, 106 (86·2%) had more than a ten-percentage-point gain by 1989. Similar patterns occurred in the next two decades, with 19 (48·7%) of 39 countries with DTP3 coverage lower than 60% increasing by at least ten percentage points from 1990 to 1999, as did 26 (76·5%) of 34 countries from 2000 to 2009.

From 2010 to 2019, however, progress on DTP3, MCV1, and Pol3 coverage stalled or reversed in many locations. For DTP3, 25 countries with at least 90% coverage in 2010 saw levels fall below this threshold in 2019, and 16 countries with coverage ranging from 60% to 90% in 2010 saw declines of five percentage points or more by 2019. Globally, 94 countries and territories recorded decreasing DTP3 coverage since 2010, with countries in Latin America and the Caribbean among those with the largest reductions (figure 2; appendix figure S14). Results were similar for MCV1 and Pol3 coverage (appendix figure S14).

### Introduction and scale-up of newer vaccines

Since the early 2000s, vaccines such as HepB, Hib, MCV2, PCV, RCV, and RotaC were introduced into many countries' national immunisation schedules and scaled up in an effort to expand protection against these vaccine-preventable diseases. By 2019, global coverage of these newer additions began to approach that of more established vaccines, reaching 80·7% (95% UI 79·5–81·8) for HepB3, 70·6% (69·2–71·9) for Hib3, 69·5% (68·6–70·3) for RCV1, and 68·1% (66·5–69·5) for MCV2 (figure 1). Although global PCV3 and RotaC coverage were somewhat lower in 2019 (47·9% [47·0–48·9] and 39·1% [38·0–40·4], respectively), countries that introduced these vaccines often rapidly increased coverage. For instance, of the 125 countries that introduced PCV into their routine immunisation schedules before 2015, 104 (83·2%) reached PCV3 coverage within five percentage points of DTP3 coverage by 2019; 57 (45·6%) of 79 achieved the equivalent for RotaC coverage.

### Progress towards GVAP targets

**Figure 3:**
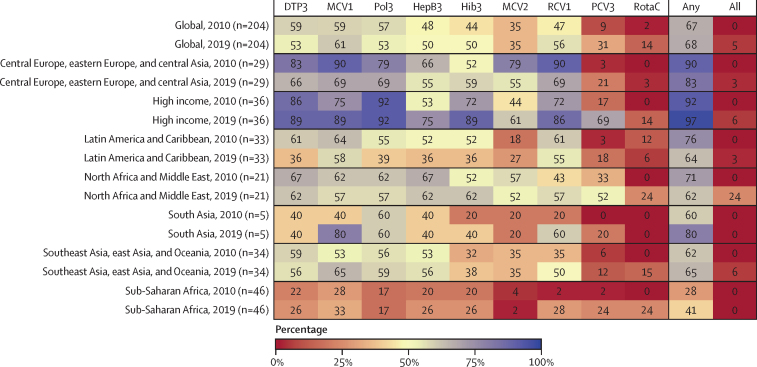
Percentage of locations reaching the GVAP national coverage target in 2010 and 2019, globally and by GBD super-region Each cell represents the percentage of locations, globally and by GBD super-region, that have reached the GVAP 90% national coverage target in 2010 and 2019 for the assessed vaccines. Percentages are shown for each vaccine separately meeting the target, for at least any single vaccine meeting the target, and for all assessed vaccines listed as meeting the target. Percentages are calculated on the basis of the total number of locations in the GBD super-region, irrespective of whether locations included the vaccine in their national schedule in 2010 or 2019. GVAP=Global Vaccine Action Plan. GBD=Global Burden of Diseases, Injuries, and Risk Factors Study. DTP3=diphtheria-tetanus-pertussis vaccine, third dose. MCV1=measles-containing vaccine, first dose. Pol3=polio vaccine, third dose. HepB3=hepatitis B vaccine, third dose. Hib3=*Haemophilus influenzae* type b vaccine, third dose. MCV2=measles-containing vaccine, second dose. RCV1=rubella-containing vaccine, first dose. PCV3=pneumococcal conjugate vaccine, third dose. RotaC=completed rotavirus series.

Benchmarking country performance from 2010 to 2019 provides insight into GVAP trajectories and the likelihood of achieving the 2020 target (figure 3). In 2010, 121 (59·3%) of 204 countries and territories reached at least 90% mean coverage for DTP3, as did 120 (58·8%) of 204 countries and territories for MCV1, and 117 (57·4%) of 204 countries and territories for Pol3; by contrast, in 2019, 109 (53·4%) of 204 countries and territories reached at least 90% mean coverage for DTP3, as did 124 (60·8%) of 204 countries and territories for MCV1, and 109 (53·4%) of 204 countries and territories for Pol3. Worsening performance was particularly striking for two GBD super-regions (central Europe, eastern Europe, and central Asia; and Latin America and the Caribbean), whereas south Asia saw sustained improvement for MCV1. Sub-Saharan Africa had the lowest proportion of countries that met the 90% mean GVAP target in 2019, ranging from 2·2% to 32·6% by vaccine, and with 19 (41·3%) of 46 locations meeting this mean target for any vaccine. Globally, 11 countries and territories reached 90% or higher average coverage across nine assessed vaccines in 2019: Armenia, Australia, Bahrain, Mauritius, Morocco, Nicaragua, Niue, Norway, Palestine, Qatar, and Saudi Arabia. Target attainment conditional on vaccine introduction is summarised in the appendix (figure S29).

### Changes in vaccine coverage relative to sociodemographic development

**Figure 4:**
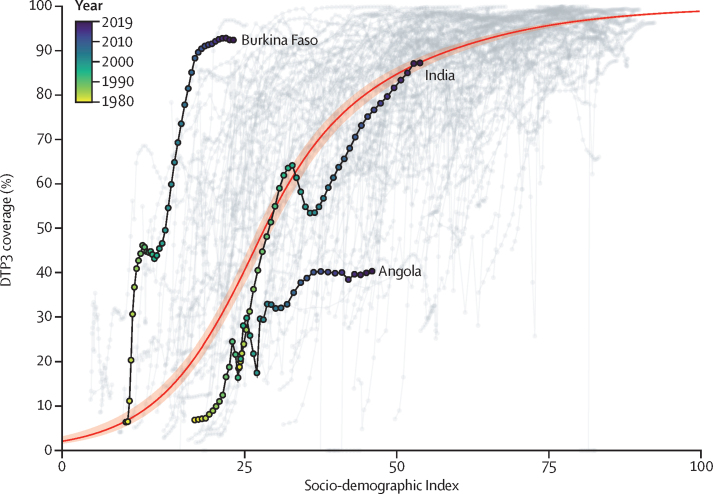
Relationships between national DTP3 coverage and SDI, 1980–2019 The solid red line represents the global average relationship between DTP3 coverage and SDI alone, as estimated across locations and over time; the shading represents the 95% uncertainty interval. Observed SDI estimates and DTP3 coverage estimates for each location are shown in light grey. For selected countries (ie, Burkina Faso, India, and Angola), the colour of each point represents the estimation year, from yellow (1980) to purple (2019). Each location's vaccine coverage estimates relative to SDI can be found in the appendix (figure S15). DTP3=diphtheria-tetanus-pertussis vaccine, third dose. SDI=Socio-demographic Index.

On average, higher vaccine coverage was associated with higher SDI (figure 4); however, this relationship was far from linear. For instance, an increase in SDI values from 25 to 35 (on a 0–100 scale) was associated with a greater than 25-percentage-point difference in DTP3 coverage (ie, from expected global averages of 30·3% [95% UI 27·7–32·8] with an SDI of 25 to 58·5% [55·0–61·5] with an SDI of 35). At higher levels of SDI, similar changes in SDI were associated with far less pronounced changes in coverage: changing from an SDI of 75 to 85, for example, corresponded to a 2·0-percentage-point difference in DTP3 coverage (from 95·3% [94·7–95·8] to 97·3% [97·0–97·6]).

Comparing these average global relationships between DTP3 coverage and SDI also helps to identify locations that have outpaced—or are lagging behind—changes in development. Burkina Faso, for example, emerged as far exceeding its expected DTP3 coverage given its SDI, reaching 92·4% (87·3–95·9) coverage in 2019 when its expected DTP3 coverage was 35·6% (32·9–38·2) relative to SDI. By contrast, Angola had among the largest gaps between estimated DTP3 coverage and expected levels on the basis of SDI alone: in 2019, Angola's DTP3 coverage was 40·3% (33·7–47·3), whereas its expected coverage was 81·6% (79·6–83·3). While this gap grew in Angola over time, by 2019 other countries such as India saw observed coverage track more closely to expected estimates on the basis of SDI alone. Similar patterns occurred for MCV1 and Pol3 coverage, with country-by-country comparisons for each vaccine provided in the appendix (figures S15–S16).

### Trends in the number of zero-dose children

**Figure 5:**
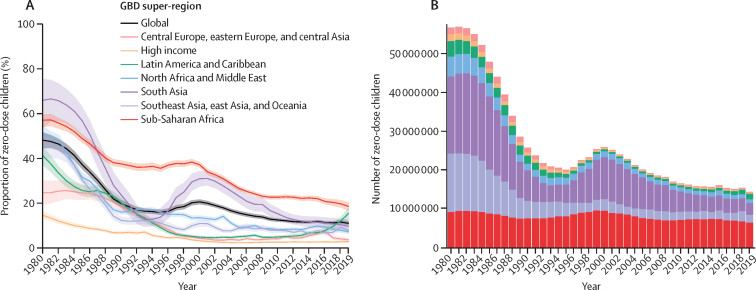
Proportion (A) and total number (B) of zero-dose children, globally and by GBD super-region, 1980–2019 The solid lines represent the proportion of zero-dose children for each GBD super-region, and the lighter-coloured shading surrounding the solid lines represents the 95% uncertainty intervals. Zero-dose children are approximated by subtracting estimates of DTP1 coverage from 100%, and then multiplying percentages by population estimates from GBD. Bar colours denote each GBD super-region. GBD=Global Burden of Diseases, Injuries, and Risk Factors Study. DTP1=diphtheria-tetanus-pertussis vaccine, first dose.

Globally, the number of zero-dose children fell from 56·8 million (95% UI 52·6–60·9) in 1980 to 14·5 million (13·4–15·9) in 2019, a decrease of nearly 75% (figure 5). Southeast Asia, east Asia, and Oceania had among the largest reductions during this time (86·3% [83·0–89·1]), as did south Asia (84·4% [78·3–89·1]). Trends in counts of zero-dose children involved both changes in DTP1 coverage and population growth. For example, in sub-Saharan Africa, the total number of zero-dose children decreased by 30·1% (21·8–37·8) from 1980 to 2019, with 6·5 million (5·8–7·1) zero-dose children remaining in 2019. Yet the percentage of zero-dose children in sub-Saharan Africa decreased even more drastically, from 57·1% (54·1–59·9) in 1980 to 18·6% (16·8–20·5) in 2019. Conversely, the number and proportion of zero-dose children increased in Latin America and the Caribbean from 2000 onwards, rising from 0·52 million (0·46–0·58) in 2000 to 1·5 million (1·2–1·7) in 2019 and from 5·0% (4·4–5·6) in 2000 to 15·6% (13·2–18·1) in 2019. In 2019, 75% of zero-dose children lived in 14 countries: Angola, Brazil, Chad, China, Democratic Republic of the Congo, Ethiopia, India, Indonesia, Mexico, Nigeria, Pakistan, Philippines, Somalia, and South Africa.

### Comparison with WUENIC estimates

Concordance between GBD-based and WUENIC-based estimates was generally high (ie, from ρ_c_=0·74 for RCV1 to ρ_c_=0·92 for DTP3 and MCV1; appendix table S8). Globally, similar coverage trends were observed for DTP3, MCV1, and Pol3, with gains stagnating from 2010 to 2019,^13^, ^18^ and more variation occurred at the country level, as well as by vaccine, over time. Additional comparisons are shown in the appendix (figures S30–S31).

## Discussion

### Summary of the main findings

This study provides a comprehensive assessment of global patterns in coverage for 11 vaccines from 1980 to 2019, a 40-year period of both notable progress and enduring disparities for routine vaccination. Coverage of longstanding and more recently introduced vaccines improved in much of the world between 1980 and 2010, protecting more children against vaccine-preventable diseases than ever before. Yet from 2010 to 2019, a period in which the introduction and scale-up of new vaccines was largely successful, gains for more established vaccines were minimal; in some locations, particularly in Latin America and the Caribbean, vaccine coverage faltered. These trends imply that, while the GVAP era broadened global access to more vaccines, less progress was made in ensuring routine immunisation services reach all children. In 2019, 14·5 million children worldwide still lacked one dose of DTP, a key indicator of zero-dose children and thus those who are at greatest risk of being left behind. Timely, disaggregated data on the evolving needs for routine immunisation programmes are crucial to target resources to those who need them the most, particularly if the global ambitions encompassed by IA2030 are to be realised in the next decade.

### Global gains and challenges in routine immunisation

The GVAP era followed years of sizeable progress for childhood vaccination: from 1980 to 2010, global coverage of original EPI vaccines such as DTP3 and MCV1 more than doubled, the number of zero-dose children fell steadily, and from the early 2000s initiatives such as Gavi, the Vaccine Alliance, supported the introduction and scale-up of new vaccines.^18^, ^19^, ^34^ Access to HepB and Hib vaccines, as well as PCV and RotaC, vastly improved, and many countries, especially those in sub-Saharan Africa, rapidly increased coverage of these newer vaccines to approach that of more established vaccines. Yet expanding these gains hinges upon increasing the reach of routine immunisation to all children—and the world has been far less successful in this endeavour, as evidenced by the stagnating or even faltering coverage of long-established vaccines between 2010 and 2019. Such trends correspond with other global analyses, such as those from WUENIC, underscoring challenges in further improving and expanding vaccine coverage in the past decade.^18^, ^19^ Although countries across the sociodemographic spectrum have shown these concerning trends, this trajectory has been particularly striking for Latin America and the Caribbean. Past research suggests that compounding factors, including large subnational disparities in access to vaccines and shifting perceptions of vaccine risk, could have contributed to regional declines.^35^, ^36^, ^37^ By 2019, only 109 of 204 countries and territories reached at least 90% mean estimated coverage for DTP3, while only 11 countries and territories met this threshold across nine of the assessed vaccines.

Improving and sustaining advances in vaccination requires a constellation of local and global factors, and our analysis further emphasises that progress in routine immunisation is far from inevitable. Key determinants that drive childhood vaccination trends are complex and inter-related, ranging from individual and community-level characteristics (eg, parental knowledge, vaccine confidence, and physical and financial access to immunisation services) to health-system capacity and enabling macro-level forces (eg, general health-system strength, political commitment to vaccine programmes, reliable funding, and supportive policies).^38^, ^39^ Longstanding challenges in supply chain or distribution channels can constrain further scale-up and outreach services,^40^ while societal vaccine confidence patterns have been strongly linked to uptake.^41^ Prolonged conflict or surges of unrest can contribute to persistently low coverage or abrupt declines in vaccination rates.^42^, ^43^ Widespread infectious disease outbreaks, as underscored by Ebola virus disease and now COVID-19,^44^, ^45^ can strain already fragile health systems and disrupt usual modes of vaccine delivery. How these factors affect vaccine services differs across locations and over time, and thus warrants further examination to better understand the pathways towards strengthening routine immunisation services.

In light of recent trends, coupled with the effects of COVID-19 on routine immunisation services,^10^, ^11^, ^12^ accelerating global vaccination gains will require more than a continuation of current programme strategies.^41^, ^42^ By quantifying uncertainty and synthesising multiple sources of coverage data while adjusting for biases, statistically derived coverage estimates provide a robust platform to track progress towards global targets. Moreover, IA2030 marks an evolution in global immunisation priorities, recognising the limitations of the more top-down GVAP^46^ and championing locally tailored approaches for each community. IA2030 also emphasises the need to identify and reach zero-dose children historically missed by routine immunisation services, and that strengthening of such services must occur in tandem with bolstering service access and primary care integration.^47^ To support these efforts, the action-based IA2030 Monitoring and Evaluation Framework supports the use of data not only to track progress but also to continuously improve routine immunisation programmes at all levels of implementation.^9^ This shift in strategy aims to further develop the reach, equity, and sustainability of global immunisation systems within the contexts of primary care and universal health coverage. For these efforts to be successful, however, data must be of sufficient quality to inform policy decisions. As such, our estimates can serve as a useful comparator to those produced by WUENIC:^48^ areas of divergence might indicate low data availability or quality, resulting in high sensitivity to methodological assumptions.

Countries where coverage gains have outpaced the average pace of progress could provide valuable insights for breaking through coverage plateaus. For example, studies of vaccine coverage in Burkina Faso emphasise the importance of leadership and communication from vaccination centres in promoting effective services.^49^, ^50^ Consistency in programme review, funding, and country-led initiatives targeting traditionally marginalised or hard-to-reach populations has been positively associated with vaccine uptake and success.^51^ Amid ongoing challenges to equitably improve routine immunisation, our estimates of vaccine coverage aim to augment the evidence base from which more data-driven and strategic planning can occur for global initiatives and national programmes alike.

### Limitations

This study is subject to several limitations. First, we could not account for all potential sources of bias in survey data. For example, displaced or otherwise marginalised populations could be under-represented in surveys that base sampling frames on official census data. Parental recall of vaccination is also subject to bias. We did not apply a recall adjustment, as previous efforts to quantify recall biases show substantial variation in both direction and magnitude.^52^, ^53^, ^54^ We were also unable to systematically adjust for differences in methodological quality between surveys, as such descriptions were not available for all sources. Second, our approach to leveraging multiple age cohorts maximises the use of available data, but assumes negligible effects of migration, survival bias due to differential mortality by vaccination status, and catch-up vaccination that might not be well captured in survey data. These limitations could result in over-estimation of coverage in some locations (ie, marginalised groups generally have lower access to routine immunisation services than the general population; unvaccinated children might have higher mortality at young ages than vaccinated children and thus are not represented in survey data); however, the precise effects are likely to vary by location and over time. Third, in order to incorporate both administrative and survey data into our current modelling framework, we did not account for the timeliness of vaccination. Future studies should develop methods to estimate age-specific coverage rates where data permit, as such estimates could better reflect trends in schedule adherence and when delays in vaccination are occurring. Fourth, our estimates do not explicitly account for vaccines administered through private markets (aside from selected vaccines in China), vaccines introduced only for certain at-risk populations, or vaccine doses administered through campaigns; as such, coverage might be under-estimated in locations where these modes of delivery are common. Fifth, although also utilised elsewhere,^9^, ^18^ using DTP1 coverage to inform zero-dose estimates could over-estimate the total number of children who have never received any vaccine. Future analyses could examine the correlation structures across vaccine-dose combinations at the individual level and ascertain the likelihood of children without DTP1 receiving any other vaccine over time.^55^ Sixth, our study focuses on national-level vaccine coverage, which might obscure important subnational inequalities.^56^, ^57^ To reach children left behind by current vaccination programmes, within-country disparities in childhood vaccination coverage across factors transcending geography (eg, wealth and education, race and ethnicity, and refugee status) must continue to be identified and addressed.

### Conclusions

Childhood vaccine coverage has markedly improved since the 1980s, cementing efforts to expand original EPI vaccines and initiatives to scale up new vaccines as among the most important success stories in global health. Yet, stagnation and, in some cases, reversal of gains from 2010 to 2019 serve as warning signs that staying the course today will not deliver universal access to immunisation in the future. The complementary visions of GVAP and now IA2030 conceive of a world where all children benefit from the protection of safe and effective vaccines and have the opportunity to live full, healthy lives. To meet these goals, it is crucial to address both enduring and new challenges facing childhood vaccination efforts and use evidence-informed strategies for strengthening routine immunisation programmes throughout the world.

## Data sharing

To download the data used in these analyses and corresponding results, please visit the Global Health Data Exchange at http://ghdx.healthdata.org.

## Declaration of interests

Quique Bassat reports participation on a data safety monitoring board (DSMB) or advisory board as a member of the Independent Data Monitoring Committee for Respiratory Syncytial Virus vaccine development for the protection of infants (since October, 2015) (GlaxoSmithKline [GSK]) and as DSMB chair for the research project “Phase IV study to evaluate the effectiveness of the inactivated adsorbed vaccine against COVID-19 CoronaVac, among public safety and education workers with risk factors for severity, in Manaus (Amazonas)” outside the submitted work. Sonu Bhaskar reports a leadership or fiduciary role in other board, society, committee or advocacy groups, unpaid with the Rotary Club of Sydney Board of Directors, outside the submitted work. Irina Filip reports payment or honoraria for lectures, presentations, speakers' bureaus, manuscript writing, or educational events from Avicenna Medical and Clinical Research Institute, outside the submitted work. Bradford D Gessner reports participation on a DSMB or advisory board at Sanofi Pasteur with participation on a dengue vaccine and general immunisation advisory board that included honoraria; stock or stock options in Pfizer; and other financial or non-financial interests as an employee of Pfizer, all outside the submitted work. Sheikh Mohammed Shariful Islam reports grants or contracts from the National Health and Medical Research Council (NHMRC) and National Heart Foundation of Australia, outside the submitted work. Nicholas J Kassebaum reports grant support for the present manuscript from the Bill & Melinda Gates Foundation; support for attending meetings or travel support, or both, to the Gates Grand Challenges and Group B Strep Vaccine meeting from the Bill & Melinda Gates Foundation; and travel support to the Group B Strep Vaccine meeting from WHO, outside the submitted work. Philippa C Matthews reports grants or contracts from the Wellcome Intermediate Fellowship (Ref 110110Z/15/Z) and the BRC Fellowship NIHR British Research Council (Oxford) senior fellowship, outside the submitted work. Jonathan F Mosser reports support for the present manuscript from the Bill & Melinda Gates Foundation; and support for attending meetings or travel support, or both, from the Bill & Melinda Gates Foundation, outside the submitted work. Maarten J Postma reports grants and personal fees from MSD, GSK, Pfizer, Boehringer Ingelheim, Novavax, BMS, Astra Zeneca, Sanofi, IQVIA, and Seqirus; personal fees from Quintiles, Novartis, and Pharmerit; grants from Bayer, DIKTI, LPDP, Budi, WHO, Antilope, FIND, EU, and BioMerieux; stock ownership in Health-Ecore, and PAG; and is an adviser to Asc Academics, outside the submitted work. Amir Radfar reports payment or honoraria for lectures, presentations, speakers' bureaus, manuscript writing, or educational events from the Avicenna Medical and Clinical Research Institute, outside the submitted work. Alyssa N Sbarra reports support for the present manuscript from the Bill & Melinda Gates Foundation; direct consultancy fees from the Pan American Health Organization for a short-term consulting project from January to December, 2018; and support for attending meetings or travel support, or both, from the Bill & Melinda Gates Foundation for costs of travel (September, 2019, and April, 2018) and reimbursements (August, 2019). Jasvinder A Singh reports consulting fees from Crealta/Horizon, Medisys, Fidia, Two Labs, Adept Field Solutions, Clinical Care Options, Clearview Healthcare Partners, Putnam Associates, FocusForward, Navigant Consulting, Spherix, MedIQ, UBM, Trio Health, Medscape, WebMD, and Practice Point communications; and the National Institutes of Health and the American College of Rheumatology; payment or honoraria for lectures, presentations, speakers' bureaus, manuscript writing, or educational events from Simply Speaking; support for attending meetings or travel support, or both, from OMERACT, an international organisation that develops measures for clinical trials and receives arm's length funding from 12 pharmaceutical companies, when travelling bi-annually to OMERACT meetings; participation on a DSMB or advisory board as a US Food and Drug Administration (FDA) Arthritis Advisory Committee member; a leadership or fiduciary role in other board, society, committee, or advocacy groups, paid or unpaid, with OMERACT as a member of the steering committee, with the Veterans Affairs Rheumatology Field Advisory Committee as a member, and with the UAB Cochrane Musculoskeletal Group Satellite Center on Network Meta-analysis as a director and editor; and stock or stock options in TPT Global Tech, Vaxart pharmaceuticals, and Charlotte's Web Holdings, and previously owned stock options in Amarin, Viking, and Moderna pharmaceuticals, all outside the submitted work. Andrea Sylvia Winkler reports grants or contracts from the German Federal Ministry of Education and Research for various global health projects where payments were made to her institution, the Technical University of Munich. All other authors declare no competing interests.
