# Supplementary material for: Measuring routine childhood vaccination coverage in 204 countries and territories, 1980–2019: a systematic analysis for the Global Burden of Disease Study 2020, Release 1
Source: Lancet. 2021 Aug 7;398(10299):503–21. doi: 10.1016/S0140-6736(21)00984-3 (PMC8358924; doi:10.1016/S0140-6736(21)00984-3)
Supplement: Supplementary appendix [file mmc1.pdf]

THE LANCET

Supplementary appendix

This appendix formed part of the original submission and has been peer reviewed. We post it as supplied by the authors.

Supplement to: GBD 2020, Release 1, Vaccine Coverage Collaborators. Measuring routine childhood vaccination coverage in 204 countries and territories, 1980–2019: a systematic analysis for the Global Burden of Disease Study 2020, Release 1. *Lancet* 2021; published online July 15. http://dx.doi.org/10.1016/S0140-6736(21)00984-3.

Appendix: Supplementary appendix to "Measuring routine childhood vaccination coverage in 204 countries and territories, 1980-2019: a systematic analysis for the Global Burden of Disease Study 2020, Release 1"

This appendix provides more detailed methodology and supplemental figures, tables, and results for "*Measuring routine childhood vaccination coverage in 204 countries and territories, 1980-2019: a systematic analysis for the Global Burden of Disease Study 2020, Release 1*." Portions of this appendix have been adapted from Vos et al.[1] References are provided for adapted sections.

Table of Contents

1 Compliance with Guidelines for Accurate and Transparent Health Estimates Reporting (GATHER)....4
2 Data processing....6
2.1 Overview of data search strategy and use of the Global Health Data Exchange (GHDx)....6
2.2 Input data types....6
2.3 Data exclusions and outliers....7
2.4 Input data processing....8
2.4.1 Survey data processing....8
2.4.2 Official country-reported JRF coverage data processing....9
2.5 Ancillary data....9
3 Supplementary coverage estimation methods....9
3.1 Overview of modelling process....9
3.2 Spatiotemporal Gaussian process regression specifications....10
3.2.1 Mean function....11
3.2.2 Error variance spatiotemporal smoothing....11
3.2.3 Gaussian process regression....11
3.3 DTP1 estimation....11
3.3.1 DTP1 administrative data imputation....11
3.3.2 Continuation ratio ordinal regression DTP1 estimation model....12
3.4 Administrative bias adjustment....13
3.4.1 MR-BRT global cascade....13
3.4.2 ST-GPR location-specific temporal smoothing....14
3.4.3 Bias integration and uncertainty propagation....14
3.5 EPI coverage estimation....14
3.6 Ratio coverage estimation....15
3.7 Stockout adjustment....16
3.7.1 Stockout detection model....16
3.7.2 Reported stockouts....16
3.7.3 Generation of a stockout magnitude covariate for ST-GPR modelling....16
3.8 China Hib3, PCV3, and RotaC coverage estimation....17
3.9 Global and GBD super-region coverage aggregation....18
4 Additional analyses....18
4.1 Relating coverage to development....18
4.2 GVAP progress....19
4.3 Zero-dose children estimation....20
4.4 GBD and WUENIC estimate comparisons....20
4.4.1 Global and GBD super-region estimate comparisons....20

4.4.2 Key estimation methodology differences 20
5 Author contributions 22
6 Supplementary references 26
7 Supplementary figures and tables 29

1 Compliance with Guidelines for Accurate and Transparent Health Estimates Reporting (GATHER)

Item #	Checklist item	Description of Compliance
**Objectives and funding**		
**1**	Define the indicator(s), populations (including age, sex, and geographic entities), and time period(s) for which estimates were made.	Introduction (main text)
**2**	List the funding sources for the work.	Summary (main text)
**Data Inputs**		
For all data inputs from multiple sources that are synthesized as part of the study:		
**3**	Describe how the data were identified and how the data were accessed.	Methods (main text); supplementary section 2. Data processing
**4**	Specify the inclusion and exclusion criteria. Identify all ad-hoc exclusions.	Supplementary section 2. Data processing; Supplementary figure 2
**5**	Provide information on all included data sources and their main characteristics. For each data source used, report reference information or contact name/institution, population represented, data collection method, year(s) of data collection, sex and age range, diagnostic criteria or measurement method, and sample size, as relevant.	Supplementary section 2. Data processing; Supplementary tables 1, 2, 3, 6; Included data sources available through http://ghdx.healthdata.org/record/ihme-data/gbd-2020-routine-childhood-vaccination-coverage-1980-2019
**6**	Identify and describe any categories of input data that have potentially important biases (eg, based on characteristics listed in item 5).	Supplementary section 3.4. Administrative bias adjustment; Supplementary figures 5, 6, 7, 8
For data inputs that contribute to the analysis but were not synthesized as part of the study:		
**7**	Describe and give sources for any other data inputs.	Supplementary section 2. Data processing; Supplementary table 2
For all data inputs:		
**8**	Provide all data inputs in a file format from which data can be efficiently extracted (eg, a spreadsheet rather than a PDF), including all relevant meta-data listed in item 5. For any data inputs that cannot be shared because of ethical or legal reasons, such as third-party ownership, provide a contact name or the name of the institution that retains the right to the data.	Available through http://ghdx.healthdata.org/record/ihme-data/gbd-2020-routine-childhood-vaccination-coverage-1980-2019
**Data analysis**		
**9**	Provide a conceptual overview of the data analysis method. A diagram may be helpful.	Methods (main text); Supplementary figure 2
**10**	Provide a detailed description of all steps of the analysis, including mathematical formulae. This description should cover, as relevant, data cleaning, data pre-processing, data adjustments and weighting of data sources, and mathematical or statistical model(s).	Methods (main text); Supplementary section 3. Supplementary coverage estimation methods

**11**	Describe how candidate models were evaluated and how the final model(s) were selected.	Supplementary section 3.1 Overview of the modelling process; Supplementary figure 3
**12**	Provide the results of an evaluation of model performance, if done, as well as the results of any relevant sensitivity analysis.	Results (main text); Supplementary section 3. Supplementary coverage estimation methods
**13**	Describe methods for calculating uncertainty of the estimates. State which sources of uncertainty were, and were not, accounted for in the uncertainty analysis.	Methods (main text); Supplementary section 3.4.3. Bias integration and uncertainty propagation
**14**	State how analytic or statistical source code used to generate estimates can be accessed.	Available through http://ghdx.healthdata.org/record/ihme-data/gbd-2020-routine-childhood-vaccination-coverage-1980-2019
**Results and Discussion**		
**15**	Provide published estimates in a file format from which data can be efficiently extracted.	Available through http://ghdx.healthdata.org/record/ihme-data/gbd-2020-routine-childhood-vaccination-coverage-1980-2019
**16**	Report a quantitative measure of the uncertainty of the estimates (eg, uncertainty intervals).	Supplementary section 3.4.3. Bias integration and uncertainty propagation
**17**	Interpret results in light of existing evidence. If updating a previous set of estimates, describe the reasons for changes in estimates.	Discussion (main text)
**18**	Discuss limitations of the estimates. Include a discussion of any modelling assumptions or data limitations that affect interpretation of the estimates.	Introduction (main text); Limitations (main text)

2 Data processing

2.1 Overview of data search strategy and use of the Global Health Data Exchange (GHDx)

The identification and collation of data sets on vaccine coverage presents particular challenges. Traditional systematic reviews of peer-reviewed literature would only return a small fraction of available data, as many of the most common sources of coverage data – such as nationally-representative household surveys, administrative data, and official country-reported data – are variably published in academic journals. Due to these limitations, we instead undertook an effort to comprehensively identify and catalogue available coverage data sources using the Global Health Data Exchange (GHDx).

The GHDx is a data cataloguing platform containing a wide variety of household survey series (eg, Demographics and Health Survey [DHS], Multiple Indicator Cluster Survey [MICS], and other country-specific survey series), country-reported data, peer-reviewed literature, and other kinds of health data types on myriad health topics. It is regularly updated and maintained as new data are published, released, made publicly available and/or supplemented by collaborators. Data are further organized by keyword, facilitating researcher ability to filter and search by specific topic areas of interest. The GHDx aims to be "the world's most comprehensive catalogue of surveys, censuses, vital statistics, and other health related data",[2] providing a platform through which data can be catalogued and tracked, particularly in cases where traditional systematic literature reviews may be insufficient.

As described below, our search strategy was multifaceted. We searched the GHDx for available data pertaining to vaccination coverage, adding newly identified data sources to this catalogue through ongoing review of releases from major survey series and contributions from our collaborator network. We cross-checked our list of catalogued sources with those identified annually through the WUENIC process[3] (see supplementary section 2.2 below), adding any sources present in the WUENIC input data set to the GHDx as needed. In addition to survey data sources, we also catalogued relevant data sources collected through the Joint Reporting Form (JRF, ie, administrative coverage data and official country-reported data). Through this process, we aim to comprehensively catalogue publicly-available data on global vaccine coverage, with results available and updated on an ongoing basis at http://ghdx.healthdata.org/.

2.2 Input data types

We searched for available survey data pertaining to vaccination coverage (including keywords: *BCG vaccines* OR *Diphtheria vaccines* OR *DTP vaccines* OR *Hepatitis B vaccines* OR *Hepatitis vaccines* OR *Hib vaccines* OR *Measles vaccines* OR *Meningococcal vaccines* OR *MMR vaccines* OR *Mumps vaccines* OR *Pentavalent vaccines* OR *Pneumococcal vaccines* OR *Polio vaccines* OR *Rotavirus vaccines* OR *Rubella vaccines* OR *Vaccination cards* OR *Pentavalent vaccines* OR *Immunization*) in the GHDx across all 204 estimated locations and 40 years (1980-2019) to generate our input dataset. We considered data on inactivated polio vaccine (IPV) and oral polio vaccine (OPV) equally to inform estimates of third-dose polio vaccine (Pol3); further, we did not consider data related to hepatitis B birth-dose (HepB0), yellow fever, human papillomavirus (HPV) vaccine, Japanese encephalitis, or any other vaccine that may be included in some national vaccination schedules.

In our study, we also used the published WHO-UNICEF Estimates of National Immunization Coverage (WUENIC) coverage survey data source list[4] to compare our input data to the sources used in the 2020 WUENIC release[5] to identify any additional sources not listed in the GHDx. We extracted the individual-level survey microdata whenever available from each source, and when microdata was not available, we extracted the national-level coverage aggregates from the survey reports. When neither microdata nor survey reports were available, we used the coverage values extracted and reported by WHO. Our methods

to process and aggregate these data are described below. Supplementary table 1 details the vaccine coverage sources included to produce our final estimates.

In addition to survey data, we also used official country-reported estimates of vaccine coverage from the 2020 Joint Reporting Form (JRF) for 195 WHO Member States between 1980 and 2019.[6] The JRF includes both the official country-reported data – which contains one single reported coverage value per location, vaccine, and year – and the administrative data – which contains the number of doses delivered and the number of children in the target population per location, vaccine, and year. Coverage via the administrative data can also be calculated over 100%, if the number of doses reported to have been delivered exceeds the estimated target population size, whereas as a 99% coverage cap is imposed on all of the official country-reported data collated through the JRF. If a similar cap is applied to administrative data, the resulting coverage proportions between both official country-reported and administrative coverage data in the JRF are harmonious for the majority of location-vaccine-years: 78.3% of all location-vaccine-years are within 1 percentage-point difference of each other across both data sets, as illustrated in supplementary figure 1.

Remaining differences between official country-reported and administrative estimates may be present for multiple reasons. For instance, in some cases, countries may choose to leverage additional data sources, perform adjustments on the underlying administrative data, or otherwise produce different estimates that represent their official best estimate of coverage within the country for that year. Such possible adjustments may differ by country and are not systematically catalogued, preventing us from directly reconciling the official and administrative estimates. Furthermore, the administrative data only provides estimates beginning in the year 2000; as our estimation series begins in 1980, the temporal detail offered by the official country-reported data – which has reporting as early as 1966 – is more valuable for spanning our entire estimation year range. Last, our use of the official country-reported JRF data also aligns with the type of country-reported data used in the WUENIC process.

Before using the official country-reported data from the JRF directly, in select location-years, we replaced the official country-reported estimates available through the WHO website with country estimates reported directly to UNICEF, as reported in the 1980-2008 WUENIC country profiles.[7] This is because prior to the implementation of the JRF process, countries may have reported discrepant official coverage values to WHO and UNICEF. Most often, this occurred between 1980 and 1990. This substitution was made in cases when the estimates reported to UNICEF were judged to be more plausible. We generally aligned the input data sources used in this model with those used historically in the WUENIC process.

Additional auxiliary data including country-reported vaccine introductions and vaccine-specific stockouts were also incorporated (see supplementary section 2.5). These data were obtained from the 2020 JRF and supplemented by custom location-specific review.

2.3 Data exclusions and outliers

Supplementary figure 2 illustrates our data screening and inclusion/exclusion process. First, we evaluated each data source identified through the process detailed above according to the following inclusion criteria:

1. Source includes data on children aged 12-59 months (or, if country-reported data, data from the relevant vaccine-specific target population age range)
2. Source is nationally representative of the general target population and captures doses delivered through routine immunisation services (ie, source cannot be only representative of a subnational region; cannot only represent a subgroup such as travellers or immunocompromised patients;

cannot report doses delivered in the private sector [with the exception of specific vaccines in China, see supplementary section 3.8])

3. Source includes dose-specific vaccine coverage data from at least one vaccine of interest in or after the country-reported national introduction year

Sources not meeting these initial criteria were fully excluded from the analysis.

Second, we evaluated each vaccine- and cohort-specific observation of coverage from each data source meeting the initial inclusion criteria. If an observation of vaccine coverage occurred before the introduction of that vaccine into the national schedule, that data point was outliered. In addition, coverage observations highly incongruent with the preponderance of other available data in surrounding years or otherwise considered to be implausible were outliered, after a detailed review of all relevant data sources for comparison and, where possible, review of survey reports and/or survey microdata to investigate potential methodological reasons for discrepancies. Supplementary table 2 details all sources meeting the initial inclusion criteria for which at least one observation of vaccine coverage was excluded from our model, along with reasons for exclusion.

2.4 Input data processing

We incorporated data from children ages 12 months through 59 months in our models, grouped by birth cohort (12-23 months, 24-35 months, 36-47 months, 48-59 months). We used the national immunisation schedules from the JRF to assign each cohort to the calendar year of expected vaccine delivery. Rather than only using children from the youngest cohort, we included all cohorts as available to integrate more data across the time series. This approach requires assumptions of negligible influence from catch-up vaccination, migration, and differential mortality in older age cohorts.

To accommodate and avoid national data attribution inconsistencies due to historical country name changes, survey data were associated with national locations using ISO alpha-3 country codes throughout the extraction, data processing, and modelling pipeline. Therefore, the underlying data stayed attributed to the correct geographic location although the mapped country name may be updated over time. We handled changes in national boundaries on a case-by-case basis. When possible from geolocated microdata or survey report data with detailed sampling descriptions, the original coverage estimate representative of the historical, singular geography was split and recalculated for each new component geography using available doses and target populations and/or sampling design. Alternatively, when this level of necessary detail was not provided, we assigned the singular, reported coverage value to all component locations but redistributed the survey sample size using national population weights estimated back in time.[8] All data included in the model were reviewed in detail. If redistributed survey data no longer satisfied our data inclusion criteria requiring national representativeness or the data were highly incongruent with other available sources for the geography, these data were subject to further review and potential exclusion (described in supplementary section 2.3).

2.4.1 Survey data processing

In the microdata, we considered children vaccinated if they had evidence of vaccine receipt from either home-based record (HBR) or parental recall. Where we had data on conjugate vaccine coverage and its component vaccines separately, we used the larger coverage proportion of the two for each component vaccine in our analysis. We did not implement an adjustment for recall bias, as the direction and magnitude of bias between reporting methods does not appear to be consistent[9]. Microdata were systematically excluded from our analysis if children were missing age assignment information or were outside our included age range (eg, children under 12 months or over 59 months of age), required indicators were not present (eg, year, child age, survey design information), or dose information was not

recorded; additional details on the data exclusion and outliers screening process can be found in supplementary section 2.3. Using this survey microdata and associated sampling weights, we calculated mean coverage at the GBD location level for each vaccine and birth cohort along with associated uncertainty while accounting for survey design in R (3.6.1)[10] using the “survey” package.[11]

For surveys without available microdata, national-level vaccine coverage data were extracted from survey reports. Survey report data variances were calculated using the binomial approximation when sample size was provided. When the sample size was not provided, we assumed a standard sample population of 100 people to also generate variance using the binominal approximation. If reported coverage data represented a combination of multiple birth cohorts, we assigned the datum to the cohort aligning with the age range midpoint. Extracted report data were systematically excluded if outside our study years or age range.

2.4.2 Official country-reported JRF coverage data processing

To account for partial-year or geographically-targeted roll-outs of newly introduced vaccines in the country-reported JRF estimates, we compared the country-reported denominators for PCV3, Hib3, HepB3, and RotaC to the denominator reported for DTP3 in the same year in the JRF administrative data.[12] In years where target populations were discrepant, coverage of the above vaccines were recalculated using vaccine-specific, country-reported doses delivered and the DTP3 target population. This approach aims to recalculate coverage for newer vaccines as a proportion of all children in the country and year of the target age for vaccination, using the assumption that DTP3 target populations would have greater stability over time given its longstanding inclusion in national immunisation schedules. Most denominator adjustments occurred within five years of national PCV3, Hib3, HepB3, and RotaC introduction in the country, and generally resulted in lower national coverage in the years surrounding introduction (as expected).

We calculated the variance for each official country-reported data point using the binomial approximation. Rather than using country-provided denominators, which would result in an implausibly low variance estimate, we assigned a sample size of 50 when the location had no survey data available for the vaccine or 10 when survey data was available.

2.5 Ancillary data

In addition to the vaccine coverage data described above, we incorporated data on location-vaccine-specific national introduction years, any years vaccines were removed from the national schedule, and data on country-reported vaccine stockouts or other delivery disruptions (described more in supplementary section 3.7). These ancillary data primarily came from the 2020 JRF, including national vaccine introduction years[13] and system indicators series.[14] Where absent in the JRF reports, custom searches were completed to fill in missing information. Supplementary table 3 details the additional ancillary data used to support these modelling features. Current immunisation schedules were used to inform assumptions about the recommended age of vaccination across the entire historical time series of vaccine coverage, due to the lack of verified data on historical national recommendations.

3 Supplementary coverage estimation methods

3.1 Overview of modelling process

Our annual, country-specific estimates of coverage reflect routine vaccination for the childhood vaccines included in each country’s national immunisation program (with the exception of China as described in supplementary section 3.8). In this analysis, we modelled estimates of first-dose Bacillus Calmette-Guérin (BCG); first- and third-dose diphtheria-tetanus-pertussis (DTP1, DTP3); third-dose hepatitis B (HepB3);

third-dose *Haemophilus influenzae* type b (Hib3); first- and second-dose measles-containing vaccine (MCV1, MCV2); third-dose pneumococcal conjugate vaccine (PCV3); third-dose polio (Pol3); first-dose rubella-containing vaccine (RCV1); and complete rotavirus (RotaC, either Rota2 or Rota3 by country). We estimated overall coverage of key vaccination coverage indicators, but did not account for differential vaccination formulations, including serotype-specific pneumococcal vaccines. Additionally, this work did not currently capture all other vaccinations administered via national, routine infrastructures, excluding Japanese encephalitis, meningococcal, human papillomavirus, and yellow fever vaccines, among others. Spatiotemporal Gaussian process regression (ST-GPR) was the primary modelling approach used in the estimation process. First, we used ST-GPR (in combination with a meta-regression framework called "meta-regression – Bayesian, regularised, trimmed" [MR-BRT]) to generate location- and time-varying estimates of bias in country-reported data, which were used to adjust the time series of official country-reported data (see supplementary section 3.4.1). Second, using the survey data and bias-adjusted country-reported data, ST-GPR was used to estimate annual vaccine coverage and associated uncertainty using Healthcare Access and Quality (HAQ) Index,[15] location-specific vaccine stockouts, and years since vaccine introduction as covariates. BCG, MCV1, DTP3, and Pol3 were modelled directly in ST-GPR while estimates of HepB3, Hib3, PCV3, RotaC, MCV2, and RCV1 were modelled as ratios against a reference vaccine (DTP3 or MCV1, see supplementary section 3.63.6). To model DTP1 coverage while ensuring internal consistency with DTP3 coverage, we used a conditional ordinal regression method, which has been described elsewhere in detail[16] and is summarized in supplementary section 3.3.2. From each model, we obtained 1,000 samples (draws) from the posterior distribution of coverage, which were then summarized to produce 95% uncertainty intervals for each annual estimate. Supplementary figure 3 illustrates an overview of our modelling process with the key sections described in further detail below. Modelling was done using R version 4.0.3.[10] Throughout our modelling process, we used versions of covariates that have been updated for GBD 2020, Release 1 (GBD 2020 R1).

3.2 Spatiotemporal Gaussian process regression specifications

Spatiotemporal Gaussian process regression (ST-GPR) is a flexible, stochastic modelling tool designed to accommodate diverse input data and make predictions over time and space. Combining linear mixed-effects models and nonparametric Gaussian process estimation frameworks, ST-GPR interpolates nonlinear trends based on interactions of model hyperparameters defined by the user. In this analysis, we ran logit-transformed ST-GPR models at several steps in the modelling process: to impute DTP1 JRF data where absent in years 1980-2000, to produce ratios of administrative bias corrections, and finally to generate estimates of location-specific vaccine coverage. The methodology and rationale underlying this modelling framework has previously been described in depth;[1] key features specific to our vaccine coverage evaluation are described here:

In general, if $P_{c,t}$ is logit-transformed vaccination coverage in country c and time t, the following notation describes the ST-GPR prediction framework:

$$P_{c,t} = g_c(t) + \epsilon_{c,t}$$

where

$$\epsilon_{c_t} \sim Normal\left(0, \sigma_p^2\right)$$
$$g_c(t) \sim GP\left(m_c(t), Cov\left(g_c(t)\right)\right).$$

The term $m_c(t)$ defines the components of the mean function. Independently, we expressed $m_c(t)$ as:

$$logit\left(P_c(t)\right) = X_c\beta + h\left(r_{c,t}\right)$$

where $X_c\beta$ summarizes a mixed-effects linear model, and $h(r_{c,t})$ the linear model residual smoothing function.

3.2.1 Mean function

The selected linear expression for each ST-GPR model serves as the basis for prediction, able to incorporate a suite of user-specified covariates. The linear model specification for each ST-GPR model run in our estimation process is described in their respective supplementary sections (DTP1-3 dropout: supplementary section 3.3.1; administrative bias adjustment: supplementary section 3.4.23.4.2; Expanded Programme on Immunisation (EPI) coverage estimation: supplementary section 3.5; ratio coverage estimation: supplementary section 3.6). Supplementary table 4 describes the strength of the covariate relationship in each model.

3.2.2 Error variance spatiotemporal smoothing

Following linear regression prediction, the linear model residuals become inputs for a locally-weighted regression (LOESS) model to further smooth and minimize prediction error over time and space. The spatiotemporal smoothing process is guided by user-specified hyperparameters (supplementary table 5) for time and space weights. In locations with high data density, smoothing across time and space is minimized so the model follows all country-specific data, while in no-data or data-sparse locations the model is more flexible. In addition, the smoothing framework leverages existing data correlation patterns to generate data weights. In countries with direct input data, space-time weights and subsequent model fit are derived entirely from the country-specific data; in the absence of country-specific data, model parameters were defined so that regional data available from neighbouring countries in proximal years guided model prediction. The weighted, smoothed sum of residuals calculated from this function get added back into the linear model estimates.

3.2.3 Gaussian process regression

Last, Gaussian process regression (GPR) using a Matérn-Euclidian covariance function is used to predict smoothed estimates over all countries and years. GPR deviates from the spatiotemporal model predictions most often in locations with high data density and high data variation, and predicted model uncertainty also increases under those circumstances. All predicted residuals from the linear and spatiotemporal models are included in GPR to produce the final smoothed estimates. 1000 predictions are sampled and summarized, using the 2.5^{th} and 97.5^{th} ordinal draws to produce 95% uncertainty intervals around every location-year estimate of vaccine coverage.

3.3 DTP1 estimation

3.3.1 DTP1 administrative data imputation

The original JRF indicators primarily focused on coverage of BCG, DTP3, MCV1, and Pol3 in the official country coverage data reports, with no required nor standardized inquiry about any other vaccines or doses in the national schedule, including DTP1. As such, estimates of DTP1 were not routinely reported by countries before the year 2000. Producing accurate representations of early years of DTP1 coverage is particularly significant in order to more effectively examine counts and proportions of zero-dose children over time. In order to better estimate DTP1 between 1980-2000, we modelled location-specific DTP1,3 absolute dropout (calculated as $DTP1\ coverage - DTP3\ coverage$) using all available pairs of unadjusted, official country-reported data to impute unadjusted DTP1 data where missing from the 2020 JRF report between years 1980-2000.

Our model of DTP1,3 absolute dropout was run in ST-GPR to produce dropout estimates correlated over space and time. It was informed by the following linear model:

$$X_{c,t} = \beta_0 + \beta_1 SDI_{c,t} + \epsilon_{c,t}$$

where $X_{c,t}$ is the absolute magnitude of dropout for country c year t; and $SDI_{c,t}$ is value of the Socio-demographic Index (SDI) for country c and year t. Supplementary table 4 summarizes the covariate weight observed in this model.

With a complete time series of absolute dropout estimated from ST-GPR, we imputed DTP1 coverage for country-years between 1980 and 2000 where official country reported data on DTP1 coverage were missing. To impute DTP1 coverage, we added the DTP1,3 absolute dropout estimate from the ST-GPR model to the country-reported, unadjusted value for DTP3 coverage by location and year. No DTP1 estimate was imputed if the location was also missing a reported estimate of DTP3 coverage in the same year. All unadjusted, imputed DTP1 data were capped at 99.9% coverage. Variance were assigned to these data similarly to all other official country-reported data, as described in supplementary section 2.4.2.

We evaluated the validity of this imputation approach by comparing the actual, official country-reported DTP1 data to data predicted by the imputation model when all DTP1 data pre-2000 (ie, 1980-1999) were withheld from the ST-GPR model. Out of all 204 countries and territories we estimate, only 31 locations had DTP1 coverage data reported prior to the year 2000 to inform this validation analysis, and the majority of available data were from the years 1990 to 1999 (76.6% [157/205] total location-years); all but one of the seven GBD super-regions were represented (south Asia).

We calculated the mean error and mean absolute error to evaluate model performance after re-creating imputed DTP1 data in these 31 locations and capping estimates at 99% if imputations exceeded 100% DTP1 coverage. Across all years 1980-1999 combined, the mean error was -3.39 percentage-points and the mean absolute error was 4.35 percentage-points. Supplementary figure 4 visualises how the imputed data and actual country-reported data compare.

3.3.2 Continuation ratio ordinal regression DTP1 estimation model

DTP1 coverage was estimated using a continuation ratio ordinal regression modelling framework which has been described elsewhere in detail.[16] This method leverages all available DTP1 and DTP3 data to model the probabilities of vaccination with 3+ doses, exactly 1 or 2 doses, or 0 doses by location and year, following:

DTP3 coverage:	$p(d \geq 3)$
DTP1 *or* DTP2 coverage:	$p(1 \leq d \leq 2)$
DTP0 coverage:	$p(d = 0)$

where d is the number of doses received per child, given that the sum of all probabilities equals 100%. We modelled DTP3 coverage in ST-GPR as described above, then additionally modelled DTP1 or DTP2 coverage conditional on the receipt of fewer than 3 doses:

$$DTP1{,}2_{conditional} = p(1 \leq d \leq 2 \mid d < 3)$$

Using draw-level estimates of DTP3 coverage and DTP1,2 conditional coverage from ST-GPR and that the sum of all probabilities for the three coverage categories listed above is 100%, we calculated DTP1 *or* DTP2 coverage and DTP0 coverage at the draw level. This method ensures internal consistency between DTP1 and DTP3 estimates at the draw-level.

3.4 Administrative bias adjustment

Administrative data on vaccine coverage are subject to multiple limitations, resulting from inaccurate or incomplete reporting of services delivered, inaccurate or out-of-date target population size estimates, and or difficulties in data collection and management.[17] Survey data are also subject to limitations, but are often considered to be a more accurate and less biased source of information on vaccine coverage.[18] However, most household surveys only occur every few years at best, while administrative data offer more continuous information on vaccine coverage over time. Notably, vaccine coverage estimates derived from administrative and survey data may show considerable differences, and the direction and magnitude of discrepancies varied over time and by location in this analysis. Until reliable and accurate reporting systems are available across all countries, addressing variation in administrative data reporting and benchmarking against surveys as available remains important.

In our study, we systematically addressed time- and location-varying biases in country-reported data using statistical frameworks to quantify the observed relationships between administrative and survey data pairs. For the purposes of this analysis, we used the official country-reported data reported through the JRF in place of administrative data. Country-reported data collected through the JRF represents the official country estimate of coverage in a given year, and is often – but not always – identical to administrative data reported in the same year (supplementary section 2.2, supplementary figure 1). While directly using the underlying administrative data to inform a vaccine coverage model would avoid this limitation, administrative data time series are notably incomplete for most countries, particularly in years prior to 2000, when compared to country-reported data collected through the JRF. We therefore estimated bias of, adjusted, and incorporated the official country-reported data in this model.

3.4.1 MR-BRT global cascade

To compute the magnitude of bias in country-reported data by country and vaccine, first we identified the overlapping location-vaccine-years where observations of both survey and administrative coverage were available, and we calculated the ratio between each available survey and country-reported data pair for BCG, DTP3, MCV1, and Pol3. Using these data pairs, we modelled the ratio of survey-derived to country-reported coverage – or administrative bias – in a multi-step process. First, we fit a two-stage random spline model using a Bayesian meta-regression technique (meta-regression–Bayesian, regularised, trimmed; MR-BRT)[19,20] with Healthcare Access and Quality (HAQ) Index as a predictor to estimate global bias direction and magnitude. MR-BRT is a powerful statistical tool which allows for capture of non-parametric relationships between covariates and outcomes. In this model, we utilized its trimming feature to algorithmically outlier 10% of all ratio data, and we evenly spaced knots at intervals of 0.2 across the 0-1 distribution of HAQ Index, following:

First stage (global): $\log(bias\ ratio_a) \sim spline(HAQ_a) * \beta_a$

Second stage (country-specific): $\log\left(bias\ ratio_{a,c}\right) \sim spline\left(HAQ_{a,c}\right) * \beta_{a,c}$

$$\beta_{a,c} \sim N(\beta_a, \sigma^2)$$
$$\text{with } \beta_a \text{ from the global model as prior}$$

where $\log(bias\ ratio_{a,c})$ is the log-transformed bias ratio of survey data to administrative data by vaccine a and country c, and $HAQ_{a,c}$ is the is value of the Healthcare Access and Quality (HAQ) Index for vaccine a and country c. The first stage MR-BRT model produced singular, global predictions of bias across the spectrum of HAQ Index. In locations without survey data, these global estimates were used adjust all the available country-reported data. Supplementary figures 5 through 8 show the global relationships calculated for these four vaccines.

The second stage of the MR-BRT cascade used the global predictions as a prior for location-specific fits. In locations with survey data, the predictions from the second stage of this cascading spline model were used as a prior in ST-GPR, described below in supplementary section 3.4.2. For locations without survey data, the predictions from the cascading spline model – based on a country's HAQ Index – were directly used to estimate bias, without an additional ST-GPR step.

3.4.2 ST-GPR location-specific temporal smoothing

To better capture temporal trends in bias in locations with paired observations of coverage from survey data and official country-reported data, we used an ST-GPR modelling step. We fit an ST-GPR model to the administrative bias ratio derived above, using the location-specific MR-BRT predictions as a prior in place of a linear mean function. We excluded extreme bias ratios (> 2) from this modelling process, as well as other implausible bias ratios (supplementary table 6). This additional ST-GPR step accounted for additional country-specific temporal trends in bias not captured by trends in HAQ Index. The outputs from ST-GPR were smoothed adjustment ratios for each location, vaccine, and year, correlated over time.

3.4.3 Bias integration and uncertainty propagation

After modelling the bias adjustment ratios, we multiplied each year of official reported coverage data by its corresponding predicted bias ratio to produce adjusted country-reported coverage estimates. Where survey data were systematically lower than official reported coverage, this approach aims to adjust for overestimation in the reported coverage data; where survey data were systematically higher than official reported coverage, this approach adjusts for underestimation in the reported coverage data. These bias adjustments were calculated and applied directly for BCG, DTP3, MCV1, and Pol3. For other vaccines, the bias adjustments for vaccines given on the same schedule (DTP3 or MCV1) were propagated via the ratio estimation method, assuming that any bias in country-reported data will be similar between each new vaccine and its corresponding reference vaccine. Overall, this method allowed us to preserve reported coverage trends in locations without survey data while also adjusting for systematic differences between administrative and survey data in locations with surveys.

3.5 EPI coverage estimation

The Expanded Programme on Immunization (EPI) developed in 1974 chiefly targeted the uptake and adherence to DTP, MCV, Pol, and BCG schedules around the world.[21,22] These longstanding vaccines have been robustly measured over time by countries and independent survey series, resulting in a wealth of data to facilitate direct modelling of these entities in our analysis.

Using the household survey microdata, survey report data, and adjusted official country-reported data as described elsewhere (supplementary section 2), we modelled coverage of BCG, DTP3, MCV1, and Pol3 directly in ST-GPR. The following mixed effects linear model was used in the prediction of DTP3, Pol3, BCG, MCV1, and DTP1,2-conditional coverage:

$$logit(P_c(t)) = \beta_0 + \beta_1 HAQ_{c,t} + \beta_2 war_{c,t} + \beta_3 stockout_{c,t} + \alpha_c + \gamma_{R[c]} + \omega_{SR[c]} + \epsilon_{c,t}$$

where $P_c(t)$ is vaccination coverage for country c year t; $HAQ_{c,t}$ is the value of the Healthcare Access and Quality (HAQ) Index for country c and year t; $war_{c,t}$ is log-transformed mortality rate due to acute war and terror events for country c and year t; $stockout_{c,t}$ is the value of the vaccine stockout covariate for country c and year t; and α_c, $\gamma_{R[c]}$, and $\omega_{SR[c]}$ are nested country, region, and GBD super-region random intercepts, respectively. Supplementary table 4 summarizes the covariate weights observed in each vaccine-specific ST-GPR model, and supplementary table 5 the specified hyperparameters for error variance spatiotemporal smoothing and Gaussian process regression in ST-GPR.

After modelling, estimates prior to national introduction or in periods of temporary removal from the national schedule were forced to zero. Of the EPI vaccines, this primarily affected the BCG vaccine, which has been removed from 46 country schedules by 2019.[23] Changes in EPI coverage over time were evaluated using mean annual estimates (main text figure 2, supplementary figures 9 through 10).

3.6 Ratio coverage estimation

Given their more recent global introduction, there are less available data on coverage of HepB3, Hib3, PCV3, RotaC, RCV1, and MCV2 than is available for the EPI vaccines listed above. To leverage the comparatively data-rich DPT3 and MCV1 estimates, we modelled the scale-up of HepB3, Hib3, PCV3, and RotaC in ST-GPR as a ratio against DPT3 coverage and the scale-up of RCV1 and MCV2 as a ratio against MCV1 coverage. We calculated these ratios using paired observations of the vaccine of interest and the reference vaccines (DTP3 or MCV1) by country-year from the same source. Then, we modelled the full time series of the ratio using ST-GPR. The following linear models were used in the mean function for these ratios, respectively, equation (A) describing the ratio vaccines with DTP3 and equation (B) the ratio vaccines with MCV1:

(A)

$$logit\left(P_c(t)\right) = \beta_0 + \beta_1 HAQ_{c,t} + \beta_2 stockout_{c,t} + \beta_3 introduction_{c,t} * DTP3_{c,t} + \alpha_c + \gamma_{R[c]} + \omega_{SR[c]} + \epsilon_{c,t}$$

(B)

$$logit(P_c(t)) = \beta_0 + \beta_1 HAQ_{c,t} + \beta_2 stockout_{c,t} + \beta_3 introduction_{c,t} * MCV1_{c,t} + \alpha_c + \gamma_{R[c]} + \omega_{SR[c]} + \epsilon_{c,t}$$

where $P_{c,t}$ is vaccination coverage for country c year since introduction i; $HAQ_{c,i}$ is the value of the HAQ Index for country c and year t; $stockout_{c,t}$ is the value of the vaccine stockout deflection weight for country c and year t; $introduction_{c,t}$ is number of years since introduction of the vaccine in the national immunisation schedule for country c and year t; $DTP3_{c,t}$ is the proportion of estimated DTP3 vaccine coverage for country c and year t; $MCV1_{c,t}$ is the proportion of estimated MCV1 vaccine coverage for country c and year t; and α_c, $\gamma_{R[c]}$, and $\omega_{SR[c]}$ are nested country, region, and GBD super-region random intercepts, respectively. Through this design, we constrained the coverage of all ratios to be less than one, assuming the coverage of each newer vaccine to be less than its corresponding reference EPI vaccine. Supplementary table 4 summarizes the covariate weights observed in each vaccine-specific ST-GPR ratio model, and supplementary table 5 the specified hyperparameters for error variance spatiotemporal smoothing and Gaussian process regression in ST-GPR.

We estimated final vaccine-specific coverage by multiplying the modelled ratios by the complementary DPT3 or MCV1 coverage values by location and year. Estimates prior to national introduction or in periods of temporary removal from the national schedule were forced to zero, with the exception of China RotaC, Hib3, and PCV3 (described in more detail in supplementary section 3.8). In estimated GBD subnational locations,[1] we re-aggregated national estimates taking into account subnational-varying vaccine introduction years, if applicable.

3.7 Stockout adjustment

3.7.1 Stockout detection model

To capture the magnitude of disruptions due to stockouts and other disruptions in vaccine coverage, we used a leave-one-out generalized additive model (GAM) approach to assess the relationship between observed and expected coverage in the official country-reported data. First, we fit location-, vaccine-specific GAMs for each year of available country-reported data, each time excluding one year from the model. When complete for all years of available data, we compiled the out-of-sample predicted coverage values for all country-reported data years in sequence to produce a reference time series of expected coverage.

Next, we calculated the difference between the country-reported coverage value and the GAM-predicted out-of-sample data value for each country and year. We then identified all location-vaccine-years in which country-reported coverage was lower than expected from the leave-one-out GAM model, as these negative deflections in the official country-reported coverage time serious could represent stockouts or other supply disruptions. Such deflections could also represent stochastic noise in the official country-reported data, however. We therefore matched the location-vaccine-years with negative deflections identified through the leave-one-out GAM model with identified as potential stockouts against a list of country-reported stockouts (see below).

3.7.2 Reported stockouts

We used the Indicator Series from the 2020 JRF to develop our reference list of unique country-vaccine stockouts.[14] We used affirmative country responses to the question "*Is there a stockout at national level*" by vaccine to indicate occurrence of a stockout. These reported data were only available beginning in 2003, though few countries have a complete time series of annual responses since that date. In years prior to 2003 and for location-years without a response to the national-level stockout question in the JRF, we supplemented this JRF source by reviewing location-specific stockouts reported to WUENIC and included in their annual country coverage descriptions[5] and performing targeted searches of ministry of health websites and news sources for evidence of stockouts or other vaccination delivery disruptions. These targeted searches were performed for all locations and years for which no JRF stockout response was available and with negative deflections 10 percentage-points or greater in the country-reported data compared to estimated non-stockout coverage from the leave-one-out GAM approach described above. Location-vaccine-years were considered to represent stockouts if there were both a negative deflection in observed coverage as compared to predicted coverage and documented evidence of a stockout or other disruption to the vaccine delivery system. Supplementary table 3 details the location-vaccine-year-specific stockouts not captured from the 2020 JRF.

3.7.3 Generation of a stockout magnitude covariate for ST-GPR modelling

The magnitude of each matched stockout deflection was calculated using the adjusted official country-reported coverage value and the expected country-reported value for the same vaccine and year from the leave-one-out GAM model:

$$V_{c,t} = invlogit\left(logit(E_{c,t}) - logit(R_{c,t})\right)$$

where $V_{c,t}$ is the value of the stockout covariate; $E_{c,t}$ is the expected bias-adjusted country-reported coverage from the leave-one-out GAM model, and $R_{c,t}$ is the bias-adjusted country-reported coverage for country c and year t. In all location-years without stockouts, the stockout covariate was assigned a value corresponding to no of the stockout value of was assigned to allow the model to differentially follow the adjusted data in stockout years, following:

$$V_{c,t} = invlogit(0)$$

where $V_{c,t}$ is the value of the stockout covariate in country c and year t.

This stockout covariate was then used as a covariate in the when fitting ST-GPR coverage models (see supplementary sections 3.5 and 3.6). Constructed in this way, the stockout covariate reflects the magnitude of decrease in coverage in the modelling (logit) space for the linear prior in the ST-GPR model that would be expected from a given stockout, under the assumption that the deflection observed in the time series of adjusted official country-reported data reflects the true magnitude of the stockout.

3.8 China Hib3, PCV3, and RotaC coverage estimation

In our analysis, we estimated vaccine coverage for all locations, vaccines, and years for which the vaccine was introduced into the national immunisation schedule. However, due to China's large, annual birth cohort size, we also estimated Hib3, PCV3, and RotaC coverage, although these vaccines were not included in China's national immunisation schedule as of 2019.

For estimation of Hib3, PCV3, and RotaC in China, we were unable to identify reliable, geographically representative data on coverage from either survey or administrative data. In the absence of data, our ST-GPR modelling framework produces predictions of coverage from covariate and spatiotemporal trends, as well as from the DTP3 coverage estimates used as the denominator in the ratio estimation process (supplementary sections 3.2 and 3.6). These modelled results can be interpreted as expected coverage of Hib3, PCV3, and RotaC in China had these vaccines been introduced into the national schedule in their year of first introduction (see supplementary figures 11 through 13). Modelled results under such an assumption, however, are highly likely to overestimate coverage. We therefore used lot release data from the China National Institutes for Food and Drug Control (NIFDC; China NIFDC website[24] and Y Teng, Linksbridge SPC, personal communication) to constrain our time series within theoretical maximums of coverage per year since any introduction.

We estimated the maximum number of doses of Hib3 and PCV3 by dividing the total number of annual reported doses by three. For RotaC, we had to account for concurrent availability of the *RotaTeq* vaccine and Lanzhou lamb rotavirus (LLR) vaccine. The *RotaTeq* vaccine is recommended on a three-dose schedule to be completed by children 12 months,[25] so we computed completed *RotaTeq* series as was done for PCV3; the recommended LLR vaccine schedule is four doses delivered to children aged two months through three years,[26] so we assumed that one-fourth of reported doses were delivered to children under 12 months and considered reception of one dose of the LLR vaccine to serve as complete vaccination for the under-12-month age group. We divided each count of completed PCV3 and RotaC series, respectively, by the under 12-month population in China to generate theoretical maximum levels of coverage, assuming no wastage and no dropout.

We then transformed the PCV3 and RotaC modelled output distributions from ST-GPR in logit space such that 97.5% of all draws were below the maximum coverage value per year (in other words, so that

the upper limit of the 95% uncertainty interval for our coverage estimates equalled the theoretical maximum coverage), following:

$$logit(maximum\ coverage_y) = logit(97.5\%\ target\ quantile\ distribution_y) + k$$

where $maximum\ coverage_y$ is the theoretical maximum coverage obtained per year based on available stock data for vaccine y; 97.5% $target\ quantile\ distribution_y$ is the percent of all coverage draws per year modelled in ST-GPR for vaccine y; and k is a year-specific factor used to transform the distribution of vaccine coverage draws obtained from the ST-GPR model so that 97.5% of the distribution is located at that theoretical maximum coverage line. All transformations were computed on draw-level estimates of Hib3, PCV3, and RotaC coverage, respectively, to preserve and recalculate uncertainty effectively.

This process has several key limitations. This approach ensures that the modelled distribution of coverage does not exceed a theoretical maximum, but requires the assumption that that the relationship between maximum possible coverage (as reflected by the 97.5th percentile of the distribution) and the mean estimated coverage is similar in the original modelled estimates and constrained coverage estimates. This approximation may not reflect true drop-out or wastage rates. Additional data on drop-out and wastage rates for Hib, PCV, and Rota in China and similar countries and comprehensive data are needed to inform models of the relationship between supply data and survey data. Such data and models would permit better estimation of coverage for Hib3, PCV3, and RotaC in China and for vaccines not yet introduced into national schedules elsewhere.

3.9 Global and GBD super-region coverage aggregation

To produce global- and GBD super-region-specific estimates of coverage, we leveraged the country-reported national immunisation schedules from the JRF[27] to match the appropriate location-vaccine-specific target populations to modelled vaccine coverage estimates. All population estimates used were updated from GBD 2019[8] as part of our continuous update cycle that will be made available with GBD 2020, Release 1 (GBD 2020 R1). Once location-vaccine-specific coverage was matched to the appropriate target population, coverage was converted to counts of vaccinated children to produce population-weighted GBD super-region and global aggregates. The seven GBD super-regions represent groupings of countries based on geographic proximity and epidemiologic similarity.[28] Most-detailed populations and geographic estimates of coverage were used in locations modelled subnationally for GBD 2019.[1] Supplementary figure 14 shows vaccine-specific estimates aggregated to GBD super-region aggregations, complementing figure 1 in the main manuscript.

4 Additional analyses

4.1 Relating coverage to development

In order to explore the relationship between vaccination coverage and development, we analysed the relationship between modelled estimates of vaccine coverage and the Socio-demographic Index (SDI), a summary measure of a country's position on the spectrum of development.[8] SDI is a composite average of three key drivers of development: lag-distributed income per capita, average educational attainment, and total fertility rates among females under 25 years of age (TFU25), expressed on a scale of 0 to 100.

Using MR-BRT[1,19] in R version 3.6.1[10] we modelled the relationship between SDI and coverage for three EPI vaccines (MCV1, DTP3, and Pol3); we excluded BCG due to its frequent removal from national immunisation settings in low-burden settings. We fit a MR-BRT ensemble spline model, which accepts both a range and count of possible splines to use in the model, to this relationship. This approach

iteratively samples possible model predictions at random and weights the final component models by model fit, as measured by the log-likelihood, and total variation, as measured using the second order derivative. This model contained five knots across the range of SDI, our single covariate input. Knot placement was limited to the bounds of 10 and 90, and the minimum distance between knots was 10 units of SDI change. Coverage inputs were transformed into logit space to ensure no prediction of expected coverage would exceed 100%.

For each of these three vaccines, this MR-BRT model produced an estimate of the average coverage expected for every unit level of SDI. In order to illustrate how a country's development and coverage trajectory over time compares to this average relationship, we produced country-specific plots of the relationship between coverage and development for each country. Supplementary figures 15 through 17 illustrates each country-specific relationship between expected and observed coverage for DTP3, MCV1, and Pol3, respectively. Of note, these modelled results show coverage as a function of SDI alone, and do not reflect the other social, political, and behavioural factors which differentially drive immunisation trends around the world.

4.2 GVAP progress

All country-, year-, and vaccine-specific mean estimates were compared to the threshold of 90% coverage to evaluate which country-year-vaccine combinations met the national Global Vaccine Action Plan (GVAP) 2020 target. To calculate the percentage of locations by GBD super-region that had met the target for each vaccine, we counted the number of locations meeting or exceeding the target and divided into the total number of locations in the GBD super-region, disregarding country-specific vaccine introduction status. To calculate the global percentage, we counted how many countries had met the threshold compared to all 204 evaluated. The percentage of "Any" vaccine by geography level was evaluated by counting the number of unique locations with any assessed vaccine greater or equal to the 90% threshold in each respective year and dividing by the appropriate denominator. The percentage of "All" vaccines by geography level was evaluated by counting the countries with all assessed vaccines at or above the 90% threshold and dividing by the appropriate denominator. BCG was excluded from this analysis given the non-introduction or phase-out of BCG in 46 countries by 2019, and DTP1 because DTP3 is considered the primary indicator from the DTP series.[29] The 11 countries with national average coverage at or above 90% for all 9 remaining vaccines in 2019 were: Armenia, Australia, Bahrain, Mauritius, Morocco, Nicaragua, Niue, Norway, Palestine, Qatar, and Saudi Arabia. Supplementary figures 18 through 28 show national-level coverage by vaccine in select years, complemented by supplementary table 7.

We repeated this analysis taking location-varying immunisation schedules into account (see supplementary section 2.5). The above calculations are the same, although for each vaccine, geography, and year the denominator changed according to how many countries had each vaccine introduced. To determine the number of countries that met or exceeded the 90% national GVAP target for all vaccines in the national schedule, we limited the vaccines we evaluated per year using the country-reported national introduction years available in the 2020 JRF (supplementary figure 29).[13] The 46 countries with national average coverage at or above 90% for all vaccines introduced in the national schedule by 2019 were: Andorra, Armenia, American Samoa, Australia, Bahrain, Bermuda, Brunei, Chile, Cook Islands, Dominica, Egypt, Spain, Greenland, Guam, Croatia, Hungary, Iran, Iceland, Jamaica, Japan, Saint Kitts and Nevis, South Korea, Sri Lanka, Morocco, Malta, Northern Mariana Islands, Mauritius, Malaysia, Nicaragua, Niue, Netherlands, Norway, Nauru, Oman, North Korea, Portugal, Palestine, Qatar, Saudi Arabia, Slovakia, Tokelau, Tonga, Tunisia, Taiwan (province of China), Uruguay, and Saint Vincent and the Grenadines.

4.3 Zero-dose children estimation

Analyses of zero-dose children support a global effort to target locations and populations where it is estimated that children have never received a dose of any vaccine, approximated by those having never received a DTP dose (referred to as zero-dose children).[30,31] To calculate estimates of zero-dose children, we converted our DTP1 coverage proportion estimates to counts using the 0-11 month population age group – the target age group to which the DTP series is provided – which was updated from GBD 2019[8] as part of our continuous update cycle that will be made available with GBD 2020 R1. Then, the number of zero-dose children was calculated as the difference between the total target population of 0-11 month children eligible for vaccination per location and year minus the number of children that received DTP1.

4.4 GBD and WUENIC estimate comparisons

4.4.1 Global and GBD super-region estimate comparisons

The primary extant mechanism for estimating national, regional, and global trends in vaccine coverage comes in the form of the WHO-UNICEF Estimates of National Immunization Coverage (WUENIC).[5] This UN-based collaboration collates country-reported vaccination data and provides annual updates of coverage trends at the country, regional, and global levels. As described elsewhere, the WUENIC estimation process follows a systematic framework combining a series of rules and heuristics with expert opinion to produce annual, vaccine-specific estimates of coverage for WHO Member States.[3] In contrast, the GBD framework leverages a statistical approach described in detail above (supplementary section 3). Although there are key estimation methodology differences described below in supplementary section 4.4.2, both GBD and WUENIC aim to use similar types of input data and capture acute, country-reported disruptions due to vaccine stockouts or other delivery system disturbances (see supplementary sections 2.1 and 3.7.2, respectively). WUENIC estimates are released annually and tabulated elsewhere.[5]

To broadly evaluate the similarities and differences between GBD and WUENIC (2020 release) vaccine coverage estimates, we computed concordance correlation coefficients (ρ_c) between all overlapping location-vaccine-years with estimates produced by both GBD and WUENIC. Coefficients were produced individually for each vaccine and ranged from $\rho_c = 0.74$ (RCV1) to $\rho_c = 0.92$ (DTP3, MCV1). Supplementary table 8 details the coefficient for each estimated vaccine.

In order to compare the GBD and WUENIC estimates at the global level, we aggregated national-level WUENIC estimates following the process described in supplementary section 3.9. In order to increase comparability between the WUENIC aggregates and aggregated GBD estimates, we used the national mean estimates from WUENIC matched to GBD 2020 R1-produced target populations. Of note, official WUENIC global estimates will differ from these estimates due to the use of different target population estimates. Supplementary figure 30 compares the aggregated GBD and WUENIC estimates globally by vaccine. National-level comparisons of GBD and WUENIC estimates are available in supplementary figure 31.

4.4.2 Key estimation methodology differences

Key methodology differences likely drive many of the observed coverage differences between GBD and WUENIC although many high-level findings are the same at the global level (eg, stalled coverage progress between 2010 and 2019 particularly for DTP3, MCV1, and Pol3 and reductions in the number of zero-dose children).[32] Further detail on specific methodological considerations underpinning the WUENIC estimates have been previously published elsewhere.[3]

WUENIC incorporates a parental recall bias adjustment for third doses of DTP, polio, HepB, and Hib vaccines. This adjustment has the largest effect in locations containing survey data where the proportion of coverage reported by parental recall is high, and is based on the assumption that parental recall of the

first-dose in a multi-dose series is more reliable than their recall of a later dose. To calibrate survey input data following this assumption, WUENIC process re-calculates third-dose coverage among all those missing a home-based record (HBR) using drop-out rates from those with HBR. This requires the assumption that vaccine dropout rates among children with a HBR and without a HBR are similar. If children with a HBR have a lower probability of vaccine dropout between their first and third dose, this process may lead to an overestimate of third-dose coverage. Because past efforts to quantify recall biases show substantial variation in terms of bias direction and magnitude,[9,33,34] our GBD estimation framework does not apply a recall bias adjustment. These different assumptions regarding recall may explain, at least in part, the observation that estimates of DTP3 coverage in the GBD estimates tend to be lower than those from WUENIC, and that less of a discrepancy is observed for DTP1.

In addition, WUENIC generally bases its inaugural estimation year per vaccine on country-reported data availability from the JRF. In contrast, the GBD framework aims to leverage data on vaccine introduction year reported through the JRF or elsewhere (supplementary section 2.5; supplementary table 3); in the absence of the availability of specific vaccine introduction information, we estimate coverage beginning in 1980 for all vaccines. These different approaches to estimation of vaccine coverage in years prior to the availability of data likely underpin differences in aggregate coverage between IHME and WUENIC in many cases, (eg, for MCV2, for which no WUENIC estimates are available prior to 2000).

A third key methodological difference between GBD and WUENIC estimates is how differences between the country-reported and survey data are reconciled. The WUENIC approach limits adjustments of the country-reported data only when the difference between the country-reported and available survey data is greater or equal to 10 percentage-points. The country-reported bias adjustment approach used by GBD (described in detail in supplementary section 3.4) allows for application of bias adjustments smaller in scale. In locations where official country-reported data are higher than corresponding survey estimates, but where discrepancies are smaller than 10 percentage points, GBD estimates will therefore tend to be lower than those produced by WUENIC.

Understanding the broad differences and implications of both approaches is important to validate and strategically compare the produced estimates by country and at higher geographic aggregations. For policymakers, additional value is derived by having two independently produced sets of estimates, particularly as the Immunization Agenda 2030 (IA2030) monitoring and evaluation framework is currently being developed,[31] and thus decisions on how to best assess IA2030 targets across routine immunisation metrics remain in-progress. In 2020, the Immunization and Vaccines-related Implementation Research Advisory Committee, organized by WHO, compared the WUENIC estimates with modelled estimates, noting that “estimates from the Institute [for] Health Metrics and Evaluation (IHME), external work to provide coverage estimates in the context of the Global Burden of Disease (GBD) initiative, could serve as good comparators” to the estimates produced by WUENIC.[35] For instance, identification of situations in which coverage estimates are highly sensitive to methodological differences can illustrate greater fundamental uncertainties about the state of existing knowledge about vaccine coverage. Further work should aim to specifically identify the key reasons for specific divergences in estimates between GBD and WUENIC processes, in order to identify opportunities for further methodological refinement and gaps in existing information about vaccine coverage at national, regional, and global scales.

5 Author contributions

Natalie Galles, Patrick Liu, Rachel Updike, Nancy Fullman, Megan Schipp, Ashley Marks, Stephen Lim, and Jonathan Mosser managed the estimation or publications process. Patrick Liu, Rachel Updike, and Nancy Fullman wrote the first draft of the manuscript. Natalie Galles and Jonathan Mosser conceptualized and drafted additional original sections during subsequent revisions. Natalie Galles, Patrick Liu, Rachel Updike, and Alyssa Sbarra had primary responsibility for applying analytical methods to produce estimates. Natalie Galles, Patrick Liu, Rachel Updike, Jason Nguyen, Sam Rolfe, and Jonathan Mosser had primary responsibility for seeking, cataloguing, extracting, or cleaning data; and designing or coding figures and tables. Natalie Galles, Patrick Liu, Rachel Updike, Alyssa Sbarra, Gdiom Abady, Sumra Abbasi, Hedayat Abbastabar, Amir Abdoli, Hassan Abolhassani, Akine Abosetugn, Maryam Adabi, Olatunji Adetokunboh, Qorinah Adnani, Saira Afzal, Bright Ahinkorah, Sohail Ahmad, Tauseef Ahmad, Sepideh Ahmadi, Haroon Ahmed, Muktar Ahmed, Tarik Ahmed Rashid, Addis Aklilu, Chisom Akunna, Hanadi Al Hamad, Fares Alahdab, Yosef Alemayehu, Kefyalew Alene, Robert Alhassan, Liaqat Ali, Syed Aljunid, Sami Almustanyir, Nelson Alvis-Guzman, Hubert Amu, Adnan Ansar, Benny Antony, Jalal Arabloo, Morteza Arab-Zozani, Kurnia Artanti, Asma Awan, Senthilkumar Balakrishnan, Maciej Banach, Simachew Bante, Amadou Barrow, Diana Bejarano Ramirez, Dinesh Bhandari, Sonu Bhaskar, Till Bärnighausen, Archith Boloor, Dejana Braithwaite, Carlos Castañeda-Orjuela, Raja Chandra Chakinala, Souranshu Chatterjee, Soosanna Kumary Chattu, Vijay Kumar Chattu, Dinh-Toi Chu, Vera Costa, Lalit Dandona, Rakhi Dandona, Meseret Derbew Molla, Rupak Desai, Mandira Dhimal, Meghnath Dhimal, Mostafa Dianatinasab, Maysaa El Sayed Zaki, Ryenchindorj Erkhembayar, Sayeh Ezzikouri, Jawad Fares, Berhanu Feleke, João Fernandes, Mark Francis, Takeshi Fukumoto, Mohamed Gad, Shilpa Gaidhane, Tushar Garg, Teshome Gebre, Birhan Gebregiorgis, Ketema Gebremedhin, Keyghobad Ghadiri, Mansour Ghafourifard, Ahmad Ghashghaee, Ionela-Roxana Glăvan, Mahaveer Golechha, Dr Kebebe Gonfa, Houman Goudarzi, Vivek Gupta, Rabih Halwani, Asif Hanif, Shafiul Haque, Arief Hargono, Shoaib Hassan, Mohamed Hassanein, Soheil Hassanipour, Hadi Hassankhani, Mohamed Hegazy, Kamal Hezam, Mehdi Hosseinzadeh, Mowafa Househ, Segun Ibitoye, Sumant Inamdar, Usman Iqbal, Seyed Sina Irvani, Sheikh Mohammed Shariful Islam, Nahlah Elkudssiah Ismail, Farahnaz Joukar, Zubair Kabir, Rohollah Kalhor, Zul Kamal, André Karch, Nicholas J Kassebaum, Yousef Khader, Himanshu Khajuria, Ibrahim Khalil, Gulfaraz Khan, Maseer Khan, Tawfik Khoja, Jagdish Khubchandani, Gyu Ri Kim, Min Seo Kim, Yun Jin Kim, Adnan Kisa, Sezer Kisa, Vladimir Korshunov, Soewarta Kosen, Barthelemy Kuate Defo, Savita Lasrado, Hankil Lee, Yeong Yeh Lee, Miriam Levi, Xuefeng Liu, Muhammed Magdy Abd El Razek, Fariborz Mansour-Ghanaei, Mohammad Ali Mansournia, Lorenzo Mantovani, Francisco Martins-Melo, Walter Mendoza, Ritesh Menezes, Endalkachew Mengesha, Mohamed Mesregah, Bartosz Miazgowski, George Milne, Erkin Mirrakhimov, Masoud Moghadaszadeh, Teroj Mohamed, Karzan Mohammad, Abdollah Mohammadian-Hafshejani, Shafiu Mohammed, Ali Mokdad, Mariam Molokhia, Lorenzo Monasta, Ghobad Moradi, Ulrich Mueller, Mohsen Naghavi, Ionut Negoi, Rajan Nikbakhsh, Chukwudi Nnaji, Jean Jacques Noubiap, Vincent Nwatah, Bogdan Oancea, Chimedsuren Ochir, Felix Ogbo, Andrew Olagunju, Obinna Onwujekwe, Jagadish Rao Padubidri, Keyvan Pakshir, Eun-Cheol Park, Fatemeh Pashazadeh Kan, Mona Pathak, Shrikant Pawar, Jeevan Pereira, Mario Peres, Arokiasamy Perianayagam, Marina Pinheiro, Vivek Podder, Richard Pollok, Maarten Postma, Amir Radfar, Alireza Rafiei, Vafa Rahimi-Movaghar, Priyanga Ranasinghe, Chythra Rao, Sowmya J Rao, Priya Rathi, Salman Rawaf, Andre Renzaho, Luca Ronfani, Godfrey Rwegerera, Basema Saddik, Umar Saeed, Nasir Salam, Abdallah Samy, Francesco Sanmarchi, Milena Santric-Milicevic, Benn Sartorius, Arash Sarveazad, Brijesh Sathian, Monika Sawhney, Deepak Saxena, Abdul-Aziz Seidu, Allen Seylani, Masood Shaikh, Morteza Shamsizadeh, Pavanchand Shetty, Jae Il Shin, Negussie Sidemo, Ambrish Singh, Jasvinder Singh, Anna Skryabina, Amin Soheili, Animut Tamiru, Ruoyan Tobe-Gai, Marcos Tovani-Palone, Bach Tran, Berhan Tsegaye, Gebiyaw Tsegaye, Anayat Ullah, Saif Ullah, Sana Ullah, Yasir Waheed, Charles Wiysonge, Birhanu Yirdaw, Naohiro Yonemoto, Chuanhua Yu, Ismaeel Yunusa, Dejene Zewdie, Zhi-Jiang Zhang, and Jonathan Mosser provided data or critical feedback on data sources. Natalie Galles, Patrick Liu, Rachel Updike, Alyssa Sbarra, Muktar

Ahmed, Tarik Ahmed Rashid, Ahmed Azzam, Behailu Derseh, Mandira Dhimal, Meghnath Dhimal, Mostafa Dianatinasab, Iman El Sayed, Berhanu Feleke, Shilpa Gaidhane, Mehdi Hosseinzadeh, Mowafa Househ, Nicholas J Kassebaum, Adnan Kisa, Sezer Kisa, Endalkachew Mengesha, Masoud Moghadaszadeh, Ali Mokdad, Mohammad Ali Moni, Mohsen Naghavi, Siamak Sabour, Abdallah Samy, Deepak Saxena, Allen Seylani, Negussie Sidemo, and Jonathan Mosser developed methods or computational machinery. Natalie Galles, Patrick Liu, Rachel Updike, Nancy Fullman, Alyssa Sbarra, Gdiom Abady, Kaja Abbas, Sumra Abbasi, Hedayat Abbastabar, Foad Abd-Allah, Hassan Abolhassani, Akine Abosetugn, Abdu Adamu, Olatunji Adetokunboh, Qorinah Adnani, Shailesh Advani, Saira Afzal, Seyed Mohammad Kazem Aghamir, Bright Ahinkorah, Sohail Ahmad, Tauseef Ahmad, Haroon Ahmed, Muktar Ahmed, Tarik Ahmed Rashid, Yonas Akalu, Addis Aklilu, Chisom Akunna, Hanadi Al Hamad, Fares Alahdab, Yosef Alemayehu, Kefyalew Alene, Robert Alhassan, Liaqat Ali, Syed Aljunid, Sami Almustanyir, Khalid Altirkawi, Nelson Alvis-Guzman, Hubert Amu, Tudorel Andrei, Alireza Ansari-Moghaddam, Jalal Arabloo, Morteza Arab-Zozani, Kurnia Artanti, Judie Arulappan, Asma Awan, Mamaru Awoke, Muluken Ayza, Ghasem Azarian, Ahmed Azzam, Darshan B, Zaheer-Ud-Din Babar, Senthilkumar Balakrishnan, Maciej Banach, Simachew Bante, Hiba Barqawi, Amadou Barrow, Quique Bassat, Diana Bejarano Ramirez, Tariku Bekuma, Uzma Belgaumi, Akshaya Bhagavathula, Dinesh Bhandari, Nikha Bhardwaj, Pankaj Bhardwaj, Sonu Bhaskar, Till Bärnighausen, Ali Bijani, Archith Boloor, Dejana Braithwaite, Danilo Buonsenso, Zahid Butt, Carlos Castañeda-Orjuela, Raja Chandra Chakinala, Jaykaran Charan, Souranshu Chatterjee, Soosanna Kumary Chattu, Vijay Kumar Chattu, Fazle Chowdhury, Devasahayam Christopher, Dinh-Toi Chu, Sheng-Chia Chung, Paolo Cortesi, Vera Costa, Omid Dadras, Amare Dagnew, Baye Dagnew, Xiaochen Dai, Lalit Dandona, Rakhi Dandona, Jan-Walter De Neve, Meseret Derbew Molla, Behailu Derseh, Rupak Desai, Abebaw Desta, Deepak Dhamnetiya, Mandira Dhimal, Meghnath Dhimal, Mostafa Dianatinasab, Daniel Diaz, Shirin Djalalinia, Fariba Dorostkar, Bassey Edem, Hisham Edinur, Sahar Eftekharzadeh, Iman El Sayed, Maysaa El Sayed Zaki, Muhammed Elhadi, Aisha Elsharkawy, Christopher Esezobor, Sharareh Eskandarieh, Ifeanyi Ezeonwumelu, Sayeh Ezzikouri, Jawad Fares, Pawan Faris, Berhanu Feleke, João Fernandes, Pietro Ferrara, Florian Fischer, Mark Francis, Takeshi Fukumoto, Mohamed Gad, Shilpa Gaidhane, Tushar Garg, Biniyam Geberemariyam, Teshome Gebre, Birhan Gebregiorgis, Ketema Gebremedhin, Berhe Gebremichael, Bradford Gessner, Mansour Ghafourifard, Ahmad Ghashghaee, Syed Amir Gilani, Mahaveer Golechha, Dr Kebebe Gonfa, Sameer Gopalani, Houman Goudarzi, Mohammed Gubari, Yuming Guo, Vivek Gupta, Reyna Gutiérrez, Emily Haeuser, Rabih Halwani, Samer Hamidi, Asif Hanif, Shafiul Haque, Harapan Harapan, Arief Hargono, Abdiwahab Hashi, Shoaib Hassan, Mohamed Hassanein, Soheil Hassanipour, Hadi Hassankhani, Simon Hay, Khezar Hayat, Mohamed Hegazy, Golnaz Heidari, Ramesh Holla, Mohammad Hoque, Mostafa Hosseini, Mehdi Hosseinzadeh, Mihaela Hostiuc, Mowafa Househ, Vivian Chia-rong Hsieh, Rabia Hussain, Nawfal Hussein, Segun Ibitoye, Olayinka Ilesanmi, Irena Ilic, Sumant Inamdar, Usman Iqbal, Lalu Irham, Seyed Sina Irvani, Sheikh Mohammed Shariful Islam, Nahlah Elkudssiah Ismail, Ramaiah Itumalla, Ravi Jha, Farahnaz Joukar, Ali Kabir, Zubair Kabir, Rohollah Kalhor, Himal Kandel, André Karch, Getinet Kassahun, Nicholas J Kassebaum, Patrick Katoto, Bayew Kelkay, Andre Kengne, Yousef Khader, Himanshu Khajuria, Ibrahim Khalil, Ejaz Khan, Junaid Khan, Maseer Khan, Moien Khan, Young-Ho Khang, Tawfik Khoja, Jagdish Khubchandani, Gyu Ri Kim, Min Seo Kim, Yun Jin Kim, Ruth Kimokoti, Adnan Kisa, Sezer Kisa, Vladimir Korshunov, Barthelemy Kuate Defo, Vaman Kulkarni, Avinash Kumar, Nithin Kumar, Alexander Kwarteng, Carlo La Vecchia, Savita Lasrado, Zohra Lassi, Yeong Yeh Lee, Sonia Lewycka, Xuefeng Liu, Stany Lobo, Ricardo Lutzky Saute, Muhammed Magdy Abd El Razek, Ahmad Malik, Fariborz Mansour-Ghanaei, Mohammad Ali Mansournia, Lorenzo Mantovani, Francisco Martins-Melo, Philippa Matthews, John Robert Medina, Walter Mendoza, Ritesh Menezes, Endalkachew Mengesha, Tuomo Meretoja, Amanual Mersha, Mohamed Mesregah, Andreea Mirica, Erkin Mirrakhimov, Hamid Reza Mirzaei, Sanjeev Misra, Prasanna Mithra, Masoud Moghadaszadeh, Teroj Mohamed, Yousef Mohammad, Abdollah Mohammadian-Hafshejani, Arif Mohammed, Shafiu Mohammed, Archisman Mohapatra, Ali Mokdad, Mariam Molokhia, Mohammad Ali Moni, Catrin Moore, Ghobad Moradi, Rahmatollah Moradzadeh, Ulrich Mueller, Mohsen Naghavi, Muhammad Naveed, Biswa Nayak,

Duduzile Ndwandwe, Ionut Negoi, Sandhya Neupane Kandel, Rajan Nikbakhsh, Dina Ningrum, Molly Nixon, Chukwudi Nnaji, Jean Jacques Noubiap, Vincent Nwatah, Bogdan Oancea, Felix Ogbo, Andrew Olagunju, Babayemi Olakunde, Obinna Onwujekwe, Nikita Otstavnov, Mayowa Owolabi, Jagadish Rao Padubidri, Eun-Cheol Park, Mona Pathak, Rajan Paudel, Shrikant Pawar, Jeevan Pereira, Mario Peres, Arokiasamy Perianayagam, Majid Pirestani, Vivek Podder, Maarten Postma, Faheem Pottoo, Navid Rabiee, Amir Radfar, Alireza Rafiei, Vafa Rahimi-Movaghar, Mosiur Rahman, Setyaningrum Rahmawaty, Aashish Rajesh, Priyanga Ranasinghe, Chythra Rao, Sowmya J Rao, Priya Rathi, Salman Rawaf, Andre Renzaho, Negar Rezaei, Mohammad Sadegh Rezai, Maria Rios-Blancas, Emma Rogowski, Godfrey Rwegerera, Anas Saad, Siamak Sabour, Basema Saddik, Umar Saeed, Mohammad Ali Sahraian, Mehrnoosh Samaei, Abdallah Samy, Juan Sanabria, Francesco Sanmarchi, Milena Santric-Milicevic, Benn Sartorius, Arash Sarveazad, Brijesh Sathian, Monika Sawhney, Deepak Saxena, Sonia Saxena, Allen Seylani, Masood Shaikh, Morteza Shamsizadeh, Pavanchand Shetty, Jae Il Shin, Negussie Sidemo, Ambrish Singh, Jasvinder Singh, Smriti Sinha, Anna Skryabina, Amin Soheili, Eyayou Tadesse, Animut Tamiru, Ker-Kan Tan, Yohannes Tekalegn, Mohamad-Hani Temsah, Aravind Thavamani, Ruoyan Tobe-Gai, Marcos Tovani-Palone, Eugenio Traini, Bach Tran, Manjari Tripathi, Berhan Tsegaye, Gebiyaw Tsegaye, Anayat Ullah, Saif Ullah, Sana Ullah, Sebastian Vollmer, Yasir Waheed, Charles Wiysonge, Birhanu Yirdaw, Dong Keon Yon, Naohiro Yonemoto, Chuanhua Yu, Deniz Yuce, Ismaeel Yunusa, Mohammad Zamani, Maryam Zamanian, Dejene Zewdie, Zhi-Jiang Zhang, Alimuddin Zumla, and Jonathan Mosser provided critical feedback on methods or results. Natalie Galles, Patrick Liu, Rachel Updike, Nancy Fullman, Kaja Abbas, Foad Abd-Allah, Hassan Abolhassani, Abdu Adamu, Olatunji Adetokunboh, Qorinah Adnani, Shailesh Advani, Saira Afzal, Bright Ahinkorah, Sepideh Ahmadi, Haroon Ahmed, Muktar Ahmed, Yonas Akalu, Fares Alahdab, Luciana Albano, Kefyalew Alene, Ayman Al-Eyadhy, Robert Alhassan, Liaqat Ali, Sami Almustanyir, Nelson Alvis-Guzman, Hubert Amu, Ippazio Cosimo Antonazzo, Benny Antony, Jalal Arabloo, Morteza Arab-Zozani, Kurnia Artanti, Judie Arulappan, Muluken Ayza, Ahmed Azzam, Darshan B, Zaheer-Ud-Din Babar, Senthilkumar Balakrishnan, Maciej Banach, Hiba Barqawi, Amadou Barrow, Quique Bassat, Tariku Bekuma, Akshaya Bhagavathula, Dinesh Bhandari, Sonu Bhaskar, Till Bärnighausen, Antonio Biondi, Dejana Braithwaite, Danilo Buonsenso, Paulo Camargos, Giulia Carreras, Felix Carvalho, Carlos Castañeda-Orjuela, Souranshu Chatterjee, Soosanna Kumary Chattu, Vijay Kumar Chattu, Fazle Chowdhury, Devasahayam Christopher, Dinh-Toi Chu, Paolo Cortesi, Vera Costa, Amare Dagnew, Baye Dagnew, Jan-Walter De Neve, Meseret Derbew Molla, Behailu Derseh, Rupak Desai, Deepak Dhamnetiya, Daniel Diaz, Bassey Edem, Sahar Eftekharzadeh, Iman El Sayed, Maysaa El Sayed Zaki, Muhammed Elhadi, Shaimaa El-Jaafary, Aisha Elsharkawy, Shymaa Enany, Christopher Esezobor, Sharareh Eskandarieh, Sayeh Ezzikouri, Jawad Fares, Berhanu Feleke, Tomas Ferede, João Fernandes, Pietro Ferrara, Florian Fischer, Mark Francis, Takeshi Fukumoto, Mohamed Gad, Shilpa Gaidhane, Silvano Gallus, Tushar Garg, Biniyam Geberemariyam, Teshome Gebre, Berhe Gebremichael, Bradford Gessner, Sameer Gopalani, Yuming Guo, Vivek Gupta, Reyna Gutiérrez, Emily Haeuser, Rabih Halwani, Shafiul Haque, Harapan Harapan, Abdiwahab Hashi, Shoaib Hassan, Mohamed Hassanein, Simon Hay, Golnaz Heidari, Kamal Hezam, Ramesh Holla, Mohammad Hoque, Mostafa Hosseini, Junjie Huang, Nawfal Hussein, Segun Ibitoye, Olayinka Ilesanmi, Irena Ilic, Sumant Inamdar, Seyed Sina Irvani, Sheikh Mohammed Shariful Islam, Nahlah Elkudssiah Ismail, Ramaiah Itumalla, Ravi Jha, Ali Kabir, Himal Kandel, André Karch, Getinet Kassahun, Patrick Katoto, Yousef Khader, Himanshu Khajuria, Ejaz Khan, Gulfaraz Khan, Junaid Khan, Maseer Khan, Moien Khan, Tawfik Khoja, Jagdish Khubchandani, Gyu Ri Kim, Yun Jin Kim, Adnan Kisa, Sezer Kisa, Barthelemy Kuate Defo, Vaman Kulkarni, Avinash Kumar, Alexander Kwarteng, Carlo La Vecchia, Faris Lami, Iván Landires, Savita Lasrado, Yeong Yeh Lee, Xuefeng Liu, Platon Lopukhov, Muhammed Magdy Abd El Razek, Ahmad Malik, Lorenzo Mantovani, Francisco Martins-Melo, Philippa Matthews, Walter Mendoza, Ritesh Menezes, Endalkachew Mengesha, Tuomo Meretoja, Tomislav Mestrovic, Prasanna Mithra, Masoud Moghadaszadeh, Teroj Mohamed, Karzan Mohammad, Yousef Mohammad, Abdollah Mohammadian-Hafshejani, Arif Mohammed, Shafiu Mohammed, Archisman Mohapatra, Ali Mokdad, Mariam Molokhia, Lorenzo Monasta, Mohammad Ali Moni, Ahmed Montasir, Paula Moraga, Ulrich Mueller, Sandra Munro, Biswa Nayak, Ionut Negoi,

Sandhya Neupane Kandel, Rajan Nikbakhsh, Molly Nixon, Jean Jacques Noubiap, Virginia Nuñez-Samudio, Vincent Nwatah, Felix Ogbo, Andrew Olagunju, Obinna Onwujekwe, Nikita Otstavnov, Mayowa Owolabi, Jagadish Rao Padubidri, Eun-Cheol Park, Mona Pathak, Shrikant Pawar, Jeevan Pereira, Arokiasamy Perianayagam, Richard Pollok, Maarten Postma, Navid Rabiee, Amir Radfar, Alireza Rafiei, Vafa Rahimi-Movaghar, Rebecca Ramshaw, Priyanga Ranasinghe, Chythra Rao, Sowmya J Rao, Salman Rawaf, Andre Renzaho, Emma Rogowski, Luca Ronfani, Anas Saad, Basema Saddik, Umar Saeed, Amirhossein Sahebkar, Mohammad Ali Sahraian, Nasir Salam, Hamideh Salimzadeh, Mehrnoosh Samaei, Abdallah Samy, Juan Sanabria, Francesco Sanmarchi, Milena Santric-Milicevic, Arash Sarveazad, Deepak Saxena, Allen Seylani, Negussie Sidemo, Jasvinder Singh, Smriti Sinha, Anna Skryabina, Amin Soheili, Eyayou Tadesse, Animut Tamiru, Ker-Kan Tan, Mohamad-Hani Temsah, Hamid Reza Tohidinik, Marcos Tovani-Palone, Bach Tran, Anayat Ullah, Saif Ullah, Brigid Unim, Yasir Waheed, Andrea Winkler, Charles Wiysonge, Vahit Yiğit, Naohiro Yonemoto, Deniz Yuce, Maryam Zamanian, Chenwen Zhong, Alimuddin Zumla, and Jonathan Mosser drafted the work or revised it critically for important intellectual content. Natalie Galles, Patrick Liu, Rachel Updike, Nancy Fullman, Jason Nguyen, Sam Rolfe, Saira Afzal, Muktar Ahmed, Liaqat Ali, Behailu Derseh, Berhanu Feleke, Takeshi Fukumoto, Shilpa Gaidhane, Tushar Garg, Stanley Kamande, Junaid Khan, Masoud Moghadaszadeh, Arif Mohammed, Ali Mokdad, Mohsen Naghavi, Shrikant Pawar, Abdallah Samy, Deepak Saxena, Negussie Sidemo, and Vahit Yiğit contributed to extracting, cleaning, or cataloging data; and designing or coding figures and tables. Lalit Dandona, Simon Hay, Nicholas J Kassebaum, Rafael Lozano, Ali Mokdad, Mohsen Naghavi, Molly Nixon, Christopher Murray, Stephen Lim, and Jonathan Mosser managed the overall research enterprise. The corresponding authors had full access to all the data in the study and had final responsibility for the decision to submit for publication. Members of the core IHME research team for this topic area had full access to the underlying data used to generate estimates presented in this paper. All other authors had access to, and reviewed, estimates as part of the Global Burden of Disease Study and research evaluation process, which includes additional stages of internal IHME and external formal collaborator review.

6 Supplementary references

1 Vos T, Lim SS, Abbafati C, et al. Global burden of 369 diseases and injuries in 204 countries and territories, 1990–2019: a systematic analysis for the Global Burden of Disease Study 2019. *The Lancet* 2020; **396**: 1204–22. doi: https://doi.org/10.1016/S0140-6736(20)30925-9.

2 Institute for Health Metrics and Evaluation (IHME). Global Health Data Exchange. 2020 http://ghdx.healthdata.org/.

3 Burton A, Monasch R, Lautenbach B, *et al.* WHO and UNICEF estimates of national infant immunization coverage: Methods and processes. *Bull World Health Organ* 2009; **87**: 535–41.

4 UNICEF, WHO. WHO and UNICEF Survey Data for Immunization Coverage Time Series 2019. July 15, 2020. https://data.unicef.org/topic/child-health/immunization/ (accessed July 15, 2020).

5 UNICEF, WHO. WHO and UNICEF reported estimates of immunization coverage time series 2019. July 15, 2020. https://immunizationdata.who.int/listing.html?topic=coverage&location= (accessed July 15, 2020).

6 UNICEF, WHO. WHO and UNICEF Reported Official Target Population, Number of Doses Administered and Official Coverage 1966-2019. July 15, 2020. https://immunizationdata.who.int/listing.html?topic=coverage&location= (accessed July 15, 2020).

7 UNICEF, WHO. WHO and UNICEF Review of National Immunization Coverage, 1980-2008.

8 Wang H, Abbas KM, Abbasifard M, et al. Global age-sex-specific fertility, mortality, healthy life expectancy (HALE), and population estimates in 204 countries and territories, 1950–2019: a comprehensive demographic analysis for the Global Burden of Disease Study 2019. *The Lancet* 2020; **396**: 1160–203. doi: https://doi.org/10.1016/S0140-6736(20)30977-6.

9 Dansereau E, Brown D, Stashko L, Danovaro-Holliday MC. A systematic review of the agreement of recall, home-based records, facility records, BCG scar, and serology for ascertaining vaccination status in low and middle-income countries. *Gates Open Res.*, 2020 DOI:10.12688/gatesopenres.12916.2.

10 R Core Team (2020). R: A language and environment for statistical computing. R Foundation for Statistical Computing, Vienna, Austria. URL https://www.R-project.org/.

11 Package ‘survey’. Lumley, T. https://cran.r-project.org/web/packages/survey/survey.pdf.

12 UNICEF, WHO. WHO and UNICEF Reported Administrative Data for Immunization Coverage Time Series 2000-2019. July 15, 2020. https://immunizationdata.who.int/listing.html?topic=coverage&location= (accessed July 15, 2020).

13 UNICEF, WHO. WHO and UNICEF Year of Introductions for Selected Vaccines Database 2019. July 15, 2020. https://immunizationdata.who.int/listing.html?topic=vaccine-intro&location= (accessed July 15, 2020).

14 UNICEF, WHO. WHO and UNICEF Reported Data on System Indicators 2019. July 15, 2020. https://immunizationdata.who.int/listing.html?topic=indicators&location= (accessed July 15, 2020).

15 Fullman N, Yearwood J, Abay SM, et al. Measuring performance on the Healthcare Access and Quality Index for 195 countries and territories and selected subnational locations: a systematic analysis from the Global Burden of Disease Study 2016. *The Lancet* 2018; **391**: 2236–71. doi: https://doi.org/10.1016/S0140-6736(18)30994-2.

16 Mosser JF, Gagne-Maynard W, Rao PC, *et al.* Mapping diphtheria-pertussis-tetanus vaccine coverage in Africa, 2000 – 2016 : a spatial and temporal modelling study. *The Lancet* 2019; **393**: 1843–55.

17 Bloland P, MacNeil A. Defining & assessing the quality, usability, and utilization of immunization data. *BMC Public Health* 2019; **19**. DOI:10.1186/s12889-019-6709-1.

18 Lim SS, Stein DB, Charrow A, Murray CJL. Tracking progress towards universal childhood immunisation and the impact of global initiatives: a systematic analysis of three-dose diphtheria, tetanus, and pertussis immunisation coverage. *The Lancet* 2008; **372**: 2031–46.

19 Zheng P, Barber R, Sorensen RJD, Murray CJL, Aravkin AY. Trimmed Constrained Mixed Effects Models: Formulations and Algorithms. *J Comput Graph Stat* 2021; **2021**: 1–13.

20 Murray CJL, Aravkin AY, Zheng P, et al. Global burden of 87 risk factors in 204 countries and territories, 1990–2019: a systematic analysis for the Global Burden of Disease Study 2019. *The Lancet* 2020; **396**: 1223–49. doi: https://doi.org/10.1016/S0140-6736(20)30752-2.

21 Keja K, Chan C, Hayden G, Henderson RH. Expanded programme on immunization. *World Health Stat Q Rapp Trimest Stat Sanit Mond* 1988; **41**: 59–63.

22 WHO | The Expanded Programme on Immunization. https://www.who.int/teams/immunization-vaccines-and-biologicals/essential-programme-on-immunization (accessed Feb 22, 2021).

23 Aeras (United States), McGill University (Canada), McGill University Health Centre (Canada), University of Ottawa. The BCG World Atlas 2nd Edition 2017. Canada: McGill University (Canada), 2017.

24 Chinese National Institutes for Food and Drug Control. Summary of the publicity status of the batch issuance of biological products. 2021. https://bio.nifdc.org.cn/pqf/search.do?formAction=pqfGs (accessed June 21, 2021).

25 Dosage and Administration for RotaTeq® (Rotavirus Vaccine, Live, Oral, Pentavalent). https://www.merckvaccines.com/rotateq/dosage-and-administration/ (accessed Jan 14, 2021).

26 Kirkwood CD, Steele AD. Rotavirus Vaccines in China: Improvement Still Required. *NLM (Medline)*, 2018 DOI:10.1001/jamanetworkopen.2018.1579.

27 UNICEF, WHO. WHO and UNICEF reported immunization schedules by vaccine 2019. July 15, 2020. https://immunizationdata.who.int/listing.html?topic=vaccine-schedule&location= (accessed July 15, 2020).

28 Murray CJL, Ezzati M, Flaxman AD, *et al.* GBD 2010: Design, definitions, and metrics. *The Lancet*, 2012 DOI:10.1016/S0140-6736(12)61899-6.

29 MacDonald N, Mohsni E, Al-Mazrou Y, *et al.* Global vaccine action plan lessons learned I: Recommendations for the next decade. *Vaccine* 2020; **38**: 5364–71.

30 The equity goal (phase 5). https://www.gavi.org/our-alliance/strategy/phase-5-2021-2025/equity-goal (accessed Jan 14, 2021).

31 WHO. Implementing the immunization agenda 2030. 2021 https://www.who.int/publications/m/item/implementing-the-immunization-agenda-2030 (accessed June 18, 2021).

32 Chard AN, Gacic-Dobo M, Diallo MS, Sodha SV, Wallace AS. Routine Vaccination Coverage — Worldwide, 2019. *MMWR Morb Mortal Wkly Rep* 2020; **69**: 1706–10.

33 Miles M, Ryman TK, Dietz V, Zell E, Luman ET. Validity of vaccination cards and parental recall to estimate vaccination coverage: A systematic review of the literature. *Vaccine* 2013; **31**: 1560–8.

34 Murray CJ, Shengelia B, Gupta N, Moussavi S, Tandon A, Thieren M. Validity of reported vaccination coverage in 45 countries. *The Lancet* 2003; **362**: 1022–7.

35 World Health Organization (WHO). Weekly epidemiological record, 4 December 2020. *Wkly Epidemiol Rec* 2020; **95**: 609–28.

7 Supplementary figures and tables

Supplementary figure 1. Comparing official country-reported coverage estimates and country-reported administrative estimates from the JRF across vaccines, 2000-2019. Each data point represents official country-reported coverage for a given location-year (x-axis) and coverage derived from country-reported administrative data (y-axis) from the JRF across all vaccines and available years. Country-reported administrative data have been collected via the JRF since 2000. JRF=Joint Reporting Form.

Supplementary figure 2. Vaccine coverage data source identification and screening process. Flow chart object shapes and colours correspond to different data review processes and data categorisations, as described above (supplementary section 2). GHDx=Global Health Data Exchange. WUENIC=WHO-UNICEF Estimates of National Immunization Coverage.

Supplementary figure 3. Modelling flow chart. Flow chart object shapes and colours correspond to different modelling processes and input types, as described above (supplementary section 3). Estimation begins with data preparation steps (upper light blue box) and ends with coverage modelling (lower light blue box). JRF=Joint Reporting Form. SDI=Socio-demographic Index. ST-GPR=spatiotemporal Gaussian process regression. HAQ Index=Healthcare Access and Quality Index. MR-BRT=meta-regression—Bayesian, regularised, trimmed. GAM=generalize additive model. UI=uncertainty interval. BCG=Bacillus Calmette-Guérin. DTP1=diphtheria-tetanus-pertussis, first dose. DTP3=diphtheria-tetanus-pertussis, third dose. HepB3=hepatitis B vaccine, third dose. Hib3=*Haemophilus influenzae* type b vaccine, third dose. MCV1=measles-containing vaccine, first dose. MCV2=measles-containing vaccine, second dose. PCV3=pneumococcal conjugate vaccine, third dose. Pol3=polio vaccine, third dose. RCV1=rubella-containing vaccine, first dose. RotaC=completed rotavirus series.

Supplementary figure 4. Comparing official country-reported and imputed DPT1 coverage, 1980-1999. For countries with official country-reported DPT1 coverage from the JRF between 1980-1999, each data point represents the unadjusted, reported country-year coverage level (x-axis) plotted against the coverage imputed from the modelling approach described in supplementary section 3.3.1. Data points are shaded by year of coverage represented and labelled with ISO3 country codes when the difference between country-reported and imputed coverage exceeded 10 percentage-points. DTP1=diphtheria-tetanus-pertussis, first-dose. JRF=Joint Reporting Form.

Supplementary figure 5. Global predictions of administrative bias, BCG. Survey-to-administrative ratio data included in the global MR-BRT model of bias are shown in blue, and the data trimmed out of the model are shown in red. Points are scaled by the inverse of the data variance weights. The log of the global bias prediction is shown as the solid black line. The dashed grey line illustrates a no-bias adjustment relationship. Larger distances between each point and the dashed line indicates a larger absolute magnitude of observed survey-to-administrative data bias. Increases in HAQ Index represent higher estimated country HAQ capacity. BCG=Bacillus Calmette-Guérin. MR-BRT=meta-regression—Bayesian, regularised, trimmed. HAQ=Healthcare Access and Quality.

Supplementary figure 6. Global predictions of administrative bias, DTP3. Survey-to-administrative ratio data included in the global MR-BRT model of bias are shown in blue, and the data trimmed out of the model are shown in red. Points are scaled by the inverse of the data variance weights. The log of the global bias prediction is shown as the solid black line. The dashed grey line illustrates a no-bias adjustment relationship. Larger distances between each point and the dashed line indicates a larger absolute magnitude of observed survey-to-administrative data bias. Increases in HAQ Index represent

higher estimated country HAQ capacity. DTP3=diphtheria-tetanus-pertussis, third dose. MR-BRT=meta-regression—Bayesian, regularised, trimmed. HAQ=Healthcare Access and Quality.

Supplementary figure 7. Global predictions of administrative bias, MCV1. Survey-to-administrative ratio data included in the global MR-BRT model of bias are shown in blue, and the data trimmed out of the model are shown in red. Points are scaled by the inverse of the data variance weights. The log of the global bias prediction is shown as the solid black line. The dashed grey line illustrates a no-bias adjustment relationship. Larger distances between each point and the dashed line indicates a larger absolute magnitude of observed survey-to-administrative data bias. Increases in HAQ Index represent higher estimated country HAQ capacity. MCV1=measles-containing vaccine, first dose. MR-BRT= meta-regression—Bayesian, regularised, trimmed. HAQ=Healthcare Access and Quality.

Supplementary figure 8. Global predictions of administrative bias, Pol3. Survey-to-administrative ratio data included in the global MR-BRT model of bias are shown in blue, and the data trimmed out of the model are shown in red. Points are scaled by the inverse of the data variance weights. The log of the global bias prediction is shown as the solid black line. The dashed grey line illustrates a no-bias adjustment relationship. Larger distances between each point and the dashed line indicates a larger absolute magnitude of observed survey-to-administrative data bias. Increases in HAQ Index represent higher estimated country HAQ capacity. Pol3=polio vaccine, third dose. MR-BRT= meta-regression—Bayesian, regularised, trimmed. HAQ=Healthcare Access and Quality.

Supplementary figure 9. Changes in MCV1 coverage by decade, 1980-2019. For each decade, maps (left) and scatterplots (right) are colour-coded to reflect absolute changes in MCV1 coverage. Every circle on the scatterplot represents a country or territory, and then the same colour is reflected on the corresponding map. Locations not estimated for GBD are coloured in grey. For the scatterplots, the horizontal dashed line at 0% indicates no coverage change within the decade; the vertical dashed lines mark 60% and 90% coverage, with latter being the national GVAP target for 2020. GVAP=Global Vaccine Action Plan. MCV1=measles-containing vaccine, first dose.

Supplementary figure 10. Changes in Pol3 coverage by decade, 1980-2019. For each decade, maps (left) and scatterplots (right) are colour-coded to reflect absolute changes in Pol3 coverage. Every circle on the scatterplot represents a country or territory, and then the same colour is reflected on the corresponding map. Locations not estimated for GBD are coloured in grey. For the scatterplots, the horizontal dashed line at 0% indicates no coverage change within the decade; the vertical dashed lines mark 60% and 90% coverage, with latter being the national GVAP target for 2020. GBD=Global Burden of Disease. GVAP=Global Vaccine Action Plan. Pol3=polio vaccine, third dose.

Supplementary figure 11. Transformation of estimates in China using stock data, Hib3. The unadjusted estimate of coverage predicted from ST-GPR is shown as the red line. The 95% unadjusted uncertainty interval is represented by the shaded red band around the mean estimate. The transformed estimates of coverage are shown as the blue line. The stock data used to transform the estimates are shown as the green points in all available years. The upper shaded blue band around the blue transformed estimate of coverage represents the theoretical maximum coverage. Hib3=*Haemophilus influenzae* type b vaccine, third dose. ST-GPR=spatiotemporal Gaussian process regression.

Supplementary figure 12. Transformation of estimates in China using stock data, PCV3. The unadjusted estimate of coverage predicted from ST-GPR is shown as the red line. The 95% unadjusted uncertainty interval is represented by the shaded red band around the mean estimate. The transformed estimates of coverage are shown as the blue line. The stock data used to transform the estimates are shown as the green points in all available years. The upper shaded blue band around the blue transformed

estimate of coverage represents the theoretical maximum coverage. PCV3=pneumococcal conjugate vaccine, third dose. ST-GPR=spatiotemporal Gaussian process regression.

Supplementary figure 13. Transformation of estimates in China using stock data, RotaC. The unadjusted estimate of coverage predicted from ST-GPR is shown as the red line. The 95% unadjusted uncertainty interval is represented by the shaded red band around the mean estimate. The transformed estimates of coverage are shown as the blue line. The stock data used to transform the estimates are shown as the green points in all available years. The upper shaded blue band around the blue transformed estimate of coverage represents the theoretical maximum coverage. RotaC=completed rotavirus series. ST-GPR=spatiotemporal Gaussian process regression.

Supplementary figure 14. Time series of vaccination coverage by vaccine and GBD super-region, 1980–2019. GVAP target of reaching at least 90% coverage by 2020 is represented by the dotted line. 95% uncertainty intervals are represented the lighter-coloured shading surrounding the darker mean estimates. GBD=Global Burden of Disease. GVAP=Global Vaccine Action Plan. DTP1=diphtheria-tetanus-pertussis, first dose. DTP3=diphtheria-tetanus-pertussis, third dose. HepB3=hepatitis B vaccine, third dose. Hib3=*Haemophilus influenzae* type b vaccine, third dose. MCV1=measles-containing vaccine, first dose. MCV2=measles-containing vaccine, second dose. PCV3=pneumococcal conjugate vaccine, third dose. Pol3=polio vaccine, third dose. RCV1=rubella-containing vaccine, first dose. RotaC=completed rotavirus series.

Supplementary figure 15. Observed coverage trend versus expected coverage given SDI, by country, DTP3. Bolded red curve with associated 95% uncertainty represents the mean expected relationship between coverage and SDI from a MR-BRT model. The observed SDI and DTP3 coverage trajectories for each country are shown in light grey. In each location-specific panel, the colour of each point represents the year of estimation, from yellow (1980) to purple (2019), for that country or territory. SDI=Socio-demographic Index. DTP3=diphtheria-tetanus-pertussis, third dose. MR-BRT=meta-regression—Bayesian, regularised, trimmed.

Supplementary figure 16. Observed coverage trend versus expected coverage given SDI, by country, MCV1. Bolded red curve with associated 95% uncertainty represents the mean expected relationship between coverage and SDI from a MR-BRT model. The observed SDI and MCV1 coverage trajectories for each country are shown in light grey. In each location-specific panel, the colour of each point represents the year of estimation, from yellow (1980) to purple (2019), for that country or territory. SDI=Socio-demographic Index. MCV1=measles-containing vaccine, first dose. MR-BRT=meta-regression—Bayesian, regularised, trimmed.

Supplementary figure 17. Observed coverage trend versus expected coverage given SDI, by country, Pol3. Bolded red curve with associated 95% uncertainty represents the mean expected relationship between coverage and SDI from a MR-BRT model. The observed SDI and Pol3 coverage trajectories for each country are shown in light grey. In each location-specific panel, the colour of each point represents the year of estimation, from yellow (1980) to purple (2019), for that country or territory. SDI=Socio-demographic Index. Pol3=polio vaccine, third dose. MR-BRT=meta-regression—Bayesian, regularised, trimmed.

Supplementary figure 18. Global vaccination coverage in select years, BCG. Maps are ordered top to bottom chronologically by year, featuring estimates from 1980, 1990, 2000, 2010, and 2019, respectively. Countries with higher estimated coverage in each year are coloured in dark blue, and countries with lower estimated coverage are coloured in dark red. Locations where the vaccine is not nationally recommended

or where estimates are not produced for GBD are coloured in grey. GBD=Global Burden of Disease. BCG=Bacillus Calmette-Guérin vaccine.

Supplementary figure 19. Global vaccination coverage in select years, DTP1. Maps are ordered top to bottom chronologically by year, featuring estimates from 1980, 1990, 2000, 2010, and 2019, respectively. Countries with higher estimated coverage in each year are coloured in dark blue, and countries with lower estimated coverage are coloured in dark red. Locations where the vaccine is not nationally recommended or where estimates are not produced for GBD are coloured in grey. GBD=Global Burden of Disease. DTP1=diphtheria-tetanus-pertussis, first dose.

Supplementary figure 20. Global vaccination coverage in select years, DTP3. Maps are ordered top to bottom chronologically by year, featuring estimates from 1980, 1990, 2000, 2010, and 2019, respectively. Countries with higher estimated coverage in each year are coloured in dark blue, and countries with lower estimated coverage are coloured in dark red. Locations where the vaccine is not nationally recommended or where estimates are not produced for GBD are coloured in grey. GBD=Global Burden of Disease. DTP3=diphtheria-tetanus-pertussis, third dose.

Supplementary figure 21. Global vaccination coverage in select years, HepB3. Maps are ordered top to bottom chronologically by year, featuring estimates from 1980, 1990, 2000, 2010, and 2019, respectively. Countries with higher estimated coverage in each year are coloured in dark blue, and countries with lower estimated coverage are coloured in dark red. Locations where the vaccine is not nationally recommended or where estimates are not produced for GBD are coloured in grey. GBD=Global Burden of Disease. HepB3=hepatitis B vaccine, third dose.

Supplementary figure 22. Global vaccination coverage in select years, Hib3. Maps are ordered top to bottom chronologically by year, featuring estimates from 1980, 1990, 2000, 2010, and 2019, respectively. Countries with higher estimated coverage in each year are coloured in dark blue, and countries with lower estimated coverage are coloured in dark red. Locations where the vaccine is not nationally recommended or where estimates are not produced for GBD are coloured in grey. GBD=Global Burden of Disease. Hib3=*Haemophilus influenzae* type b vaccine, third dose.

Supplementary figure 23. Global vaccination coverage in select years, MCV1. Maps are ordered top to bottom chronologically by year, featuring estimates from 1980, 1990, 2000, 2010, and 2019, respectively. Countries with higher estimated coverage in each year are coloured in dark blue, and countries with lower estimated coverage are coloured in dark red. Locations where the vaccine is not nationally recommended or where estimates are not produced for GBD are coloured in grey. GBD=Global Burden of Disease. MCV1=measles-containing vaccine, first dose.

Supplementary figure 24. Global vaccination coverage in select years, MCV2. Maps are ordered top to bottom chronologically by year, featuring estimates from 1980, 1990, 2000, 2010, and 2019, respectively. Countries with higher estimated coverage in each year are coloured in dark blue, and countries with lower estimated coverage are coloured in dark red. Locations where the vaccine is not nationally recommended or where estimates are not produced for GBD are coloured in grey. GBD=Global Burden of Disease. MCV2=measles-containing vaccine, second dose.

Supplementary figure 25. Global vaccination coverage in select years, PCV3. Maps are ordered top to bottom chronologically by year, featuring estimates from 1980, 1990, 2000, 2010, and 2019, respectively. Countries with higher estimated coverage in each year are coloured in dark blue, and countries with lower estimated coverage are coloured in dark red. Locations where the vaccine is not nationally recommended

or where estimates are not produced for GBD are coloured in grey. GBD=Global Burden of Disease. PCV3=pneumococcal conjugate vaccine, third dose.

Supplementary figure 26. Global vaccination coverage in select years, Pol3. Maps are ordered top to bottom chronologically by year, featuring estimates from 1980, 1990, 2000, 2010, and 2019, respectively. Countries with higher estimated coverage in each year are coloured in dark blue, and countries with lower estimated coverage are coloured in dark red. Locations where the vaccine is not nationally recommended or where estimates are not produced for GBD are coloured in grey. GBD=Global Burden of Disease. Pol3=polio vaccine, third dose.

Supplementary figure 27. Global vaccination coverage in select years, RCV1. Maps are ordered top to bottom chronologically by year, featuring estimates from 1980, 1990, 2000, 2010, and 2019, respectively. Countries with higher estimated coverage in each year are coloured in dark blue, and countries with lower estimated coverage are coloured in dark red. Locations where the vaccine is not nationally recommended or where estimates are not produced for GBD are coloured in grey. GBD=Global Burden of Disease. RCV1=rubella-containing vaccine, first dose.

Supplementary figure 28. Global vaccination coverage in select years, RotaC. Maps are ordered top to bottom chronologically by year, featuring estimates from 1980, 1990, 2000, 2010, and 2019, respectively. Countries with higher estimated coverage in each year are coloured in dark blue, and countries with lower estimated coverage are coloured in dark red. Locations where the vaccine is not nationally recommended or where estimates are not produced for GBD are coloured in grey. GBD=Global Burden of Disease. RotaC=completed rotavirus series.

Supplementary figure 29. Percentage of locations reaching the 2020 GVAP target in 2010 and 2019, globally and by GBD super-region, given country introduction. Each cell represents the percentage of countries, globally and by GBD super-region, that have reached the GVAP 90% national coverage target in 2010 and 2019 accounting for variable national introduction schedules per vaccine. Percentages are shown for each vaccine separately meeting the target, for at least any single vaccine meeting the target (represented by the column labelled "Any"), and for all assessed vaccines as listed meeting the target including all countries with the vaccine introduced in the given year ("All"). Cells shown in white have no countries with the vaccine in their national schedule for that vaccine-year combination. GVAP=Global Vaccine Action Plan. GBD=Global Burden of Disease. DTP3=diphtheria-tetanus-pertussis, third dose. HepB3=hepatitis B vaccine, third dose. Hib3=*Haemophilus influenzae* type b vaccine, third dose. MCV1=measles-containing vaccine, first dose. MCV2=measles-containing vaccine, second dose. PCV3=pneumococcal conjugate vaccine, third dose. Pol3=polio vaccine, third dose. RCV1=rubella-containing vaccine, first dose. RotaC=completed rotavirus series.

Supplementary figure 30. Comparing global coverage estimates, by vaccine, from WUENIC and GBD, 1980-2019. Country-level WUENIC estimates were aggregated to the global level as described in supplementary section 4.4.1. WUENIC=WHO-UNICEF Estimates of National Immunization Coverage. GBD=Global Burden of Disease. BCG=Bacillus Calmette-Guérin. DTP1=diphtheria-tetanus-pertussis, first dose. DTP3=diphtheria-tetanus-pertussis, third dose. HepB3=hepatitis B vaccine, third dose. Hib3=*Haemophilus influenzae* type b, third dose. MCV1=measles-containing vaccine, first dose. MCV2=measles-containing vaccine, second dose. PCV3=pneumococcal conjugate vaccine, third dose. Pol3=polio vaccine, third dose. RCV1=rubella-containing vaccine, first dose. RotaC=rotavirus, complete series (two or three doses).

Supplementary figure 31. Time series of location-specific vaccination coverage by vaccine, 1980-2019. Pages show country- and vaccine-specific estimates of coverage proportions, 1980-2019. Data used

in the model are coloured in black. Data excluded from the model are coloured in light blue. Point shapes correspond to different input data types. The solid green line illustrates estimated annual coverage. 95% uncertainty intervals are represented by the shaded, light green band around each mean estimate. WUENIC estimates released July 2020 are shown in light blue. WUENIC=WHO-UNICEF Estimates of National Immunization Coverage. Original Country-reported=raw, unadjusted official country-reported data from the Joint Reporting Form (JRF). GBD=Global Burden of Disease. Country-reported=official country-reported data from the JRF, after bias adjustment. DHS=Demographics and Health Survey. MICS=Multiple Indicator Cluster Survey. Other microdata=Non-DHS, non-MICS survey microdata. Other tabulation=survey report tabulation. Imputed=statistically imputed as described in supplementary section 3.3.1.

Supplementary table 1. Data sources included in vaccine coverage modelling. List of sources with GHDx identification number included in the final analysis is provided. Vaccines with an “I” were included, vaccines with a “E” were excluded, and vaccines with “-” were not present. These sources can also be explored following this link to the GHDx input data sources tool: http://ghdx.healthdata.org/record/ihme-data/gbd-2020-routine-childhood-vaccination-coverage-1980-2019. BCG=Bacillus Calmette-Guérin. DTP1=diphtheria-tetanus-pertussis, first dose. DTP3=diphtheria-tetanus-pertussis, third dose. HepB3=hepatitis B vaccine, third dose. Hib3=*Haemophilus influenzae* type b vaccine, third dose. MCV1=measles-containing vaccine, first dose. MCV2=measles-containing vaccine, second dose. PCV3=pneumococcal conjugate vaccine, third dose. Pol3=polio vaccine, third dose. RCV1=rubella-containing vaccine, first dose. RotaC=completed rotavirus series.

Supplementary table 2. Data sources excluded from vaccine coverage modelling. Citations, series, country, years, and vaccines are provided for each excluded dataset along with rationale for their exclusion. BCG=Bacillus Calmette-Guérin. DTP1=diphtheria-tetanus-pertussis, first dose. DTP3=diphtheria-tetanus-pertussis, third dose. HepB3=hepatitis B vaccine, third dose. Hib3=*Haemophilus influenzae* type b vaccine, third dose. MCV1=measles-containing vaccine, first dose. MCV2=measles-containing vaccine, second dose. PCV3=pneumococcal conjugate vaccine, third dose. Pol3=polio vaccine, third dose. RCV1=rubella-containing vaccine, first dose. RotaC=completed rotavirus series.

Supplementary table 3. Supplementary ancillary data used in vaccine coverage modelling. These data supplement the country-reported data obtained from the JRF (see supplementary section 2.5). The Ancillary Source Type field denotes which kind of data the source provides (Introduction, meaning vaccine-specific introduction; Removal, meaning vaccine-specific phase-out or non-introduction; and Disruption, meaning vaccine-specific acute disruption to vaccine delivery). These sources can also be explored following this link to the GHDx input data sources tool: http://ghdx.healthdata.org/record/ihme-data/gbd-2020-routine-childhood-vaccination-coverage-1980-2019. BCG=Bacillus Calmette-Guérin. DTP1=diphtheria-tetanus-pertussis, first dose. DTP3=diphtheria-tetanus-pertussis, third dose. HepB3=hepatitis B vaccine, third dose. Hib3=*Haemophilus influenzae* type b vaccine, third dose. MCV1=measles-containing vaccine, first dose. MCV2=measles-containing vaccine, second dose. PCV3=pneumococcal conjugate vaccine, third dose. Pol3=polio vaccine, third dose. RCV1=rubella-containing vaccine, first dose. RotaC=completed rotavirus series.

Supplementary table 4. ST-GPR covariate weights. Tables a-c describe the covariates used in each ST-GPR linear model component. Asterisks denote covariate significance (p-value) in each model. ST-GPR=spatiotemporal Gaussian process regression. BCG=Bacillus Calmette-Guérin. DTP1=diphtheria-tetanus-pertussis, first dose. DTP3=diphtheria-tetanus-pertussis, third dose. HepB3=hepatitis B vaccine, third dose. Hib3=*Haemophilus influenzae* type b vaccine, third dose. MCV1=measles-containing vaccine,

first dose. MCV2=measles-containing vaccine, second dose. PCV3=pneumococcal conjugate vaccine, third dose. Pol3=polio vaccine, third dose. RCV1=rubella-containing vaccine, first dose. RotaC=completed rotavirus series. GBD=Global Burden of Disease.

Supplementary table 5. ST-GPR hyperparameters. Descriptions and values of each ST-GPR hyperparameter are provided for each ST-GPR model. Parameter values are included separately by location data density as applicable. ST-GPR=spatiotemporal Gaussian process regression. BCG=Bacillus Calmette-Guérin. DTP1=diphtheria-tetanus-pertussis, first dose. DTP3=diphtheria-tetanus-pertussis, third dose. HepB3=hepatitis B vaccine, third dose. Hib3=*Haemophilus influenzae* type b vaccine, third dose. MCV1=measles-containing vaccine, first dose. MCV2=measles-containing vaccine, second dose. PCV3=pneumococcal conjugate vaccine, third dose. Pol3=polio vaccine, third dose. RCV1=rubella-containing vaccine, first dose. RotaC=completed rotavirus series.

Supplementary table 6. ST-GPR administrative bias excluded ratios. Citations, series, country, years, and vaccines provided for each excluded survey-to-country-reported bias ratio along with rationale for their exclusion. ST-GPR=spatiotemporal Gaussian process regression. BCG=Bacillus Calmette-Guérin. DTP3=diphtheria-tetanus-pertussis, third dose. MCV1=measles-containing vaccine, first dose. Pol3=polio vaccine, third dose.

Supplementary table 7. National vaccination coverage in select years, by location and vaccine. Estimated coverage proportions and 95% uncertainty intervals for each country and vaccine in select years are provided. Vaccines not included in the national schedule in each respective year are indicated with a dash. BCG=Bacillus Calmette-Guérin. DTP1=diphtheria-tetanus-pertussis, first dose. DTP3=diphtheria-tetanus-pertussis, third dose. HepB3=hepatitis B vaccine, third dose. Hib3=*Haemophilus influenzae* type b vaccine, third dose. MCV1=measles-containing vaccine, first dose. MCV2=measles-containing vaccine, second dose. PCV3=pneumococcal conjugate vaccine, third dose. Pol3=polio vaccine, third dose. RCV1=rubella-containing vaccine, first dose. RotaC=completed rotavirus series.

Supplementary table 8. Concordance correlation coefficients, by vaccine, for WUENIC- and GBD-based coverage estimates. Reported coefficient values are based on concordance estimated between matched location-year estimates from 1980 to 2019 from each source. WUENIC=WHO-UNICEF Estimates of National Immunization Coverage. GBD=Global Burden of Disease. CCC=Concordance correlation coefficient. BCG=Bacillus Calmette-Guérin. DTP1=diphtheria-tetanus-pertussis, first dose. DTP3=diphtheria-tetanus-pertussis, third dose. HepB3=hepatitis B vaccine, third dose. Hib3=*Haemophilus influenzae* type b vaccine, third dose. MCV1=measles-containing vaccine, first dose. MCV2=measles-containing vaccine, second dose. PCV3=pneumococcal conjugate vaccine, third dose. Pol3=polio vaccine, third dose. RCV1=rubella-containing vaccine, first dose. RotaC=completed rotavirus series.

Supplementary figure 1. Comparing official country-reported coverage estimates and country-reported administrative estimates from the JRF across vaccines, 2000-2019. Each data point represents official country-reported coverage for a given location-year (x-axis) and coverage derived from country-reported administrative data (y-axis) from the JRF across all vaccines and available years. Country-reported administrative data have been collected via the JRF since 2000. JRF=Joint Reporting Form.

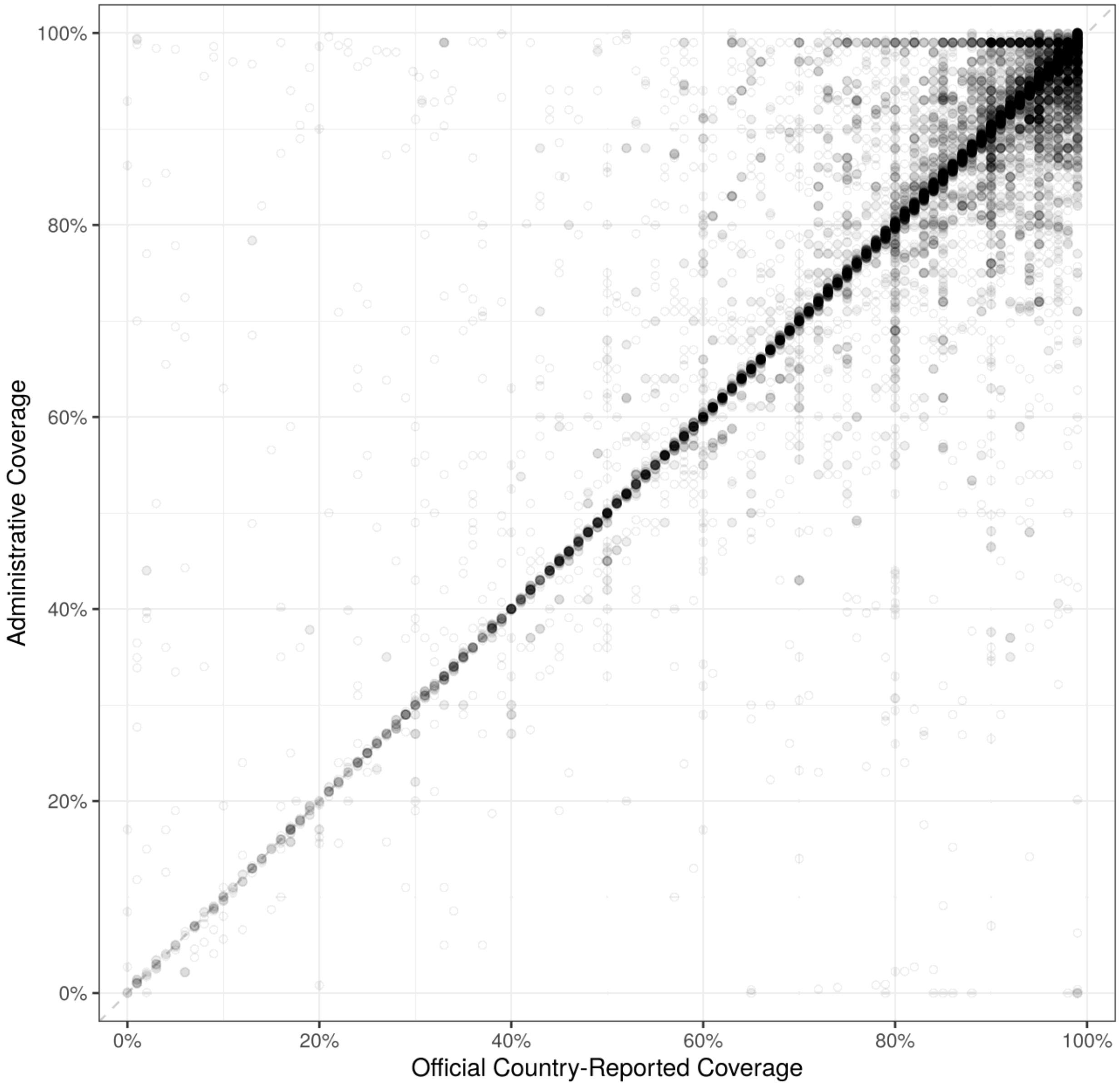

Supplementary figure 2. Vaccine coverage data source identification and screening process. Flow chart object shapes and colours correspond to different data review processes and data categorisations, as described above (supplementary section 2). GHDx=Global Health Data Exchange. WUENIC=WHO-UNICEF Estimates of National Immunization Coverage.

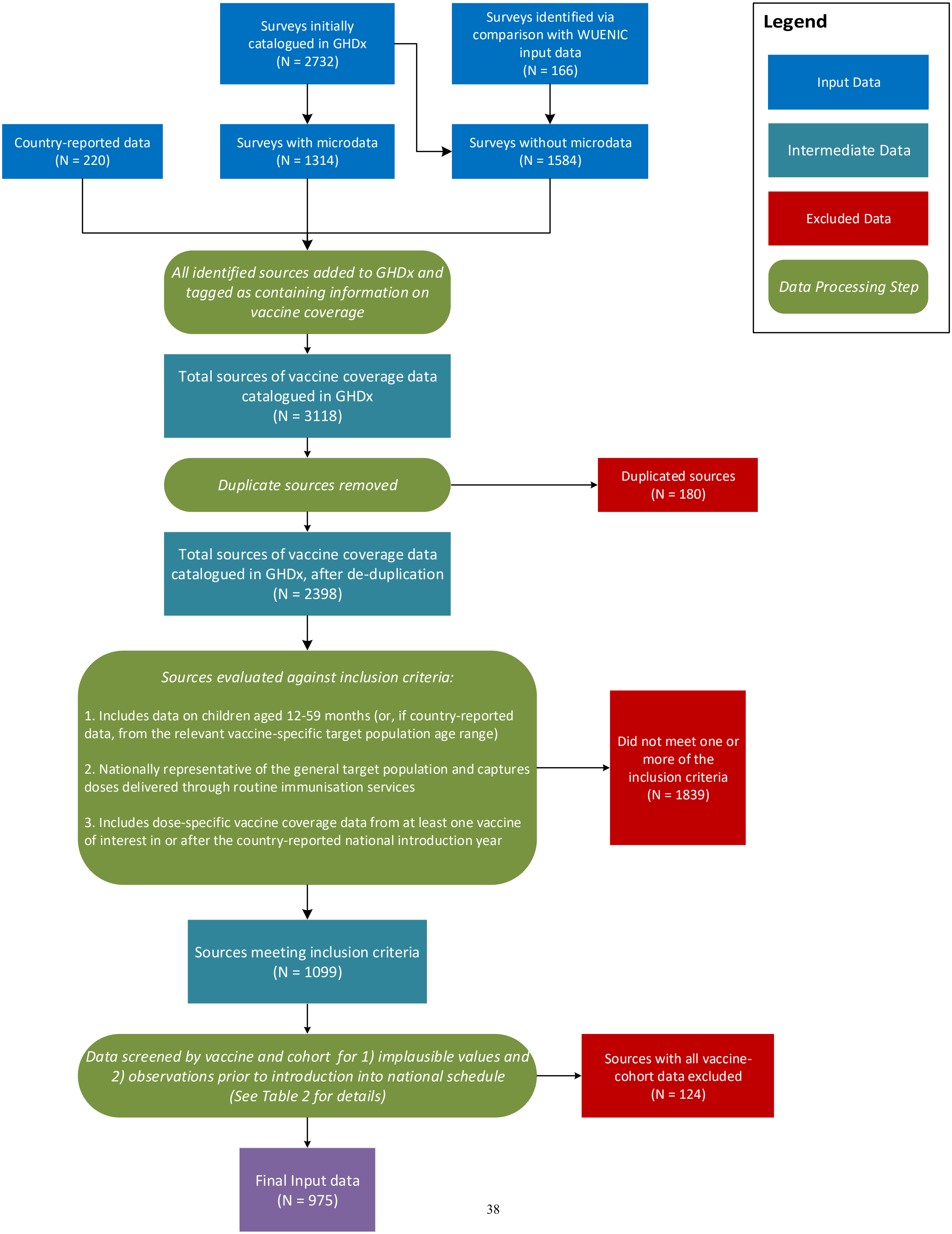

Legend
Input Data
Intermediate Data
Excluded Data
Data Processing Step
Surveys initially catalogued in GHDx (N = 2732)
Surveys identified via comparison with WUENIC input data (N = 166)
Country-reported data (N = 220)
Surveys with microdata (N = 1314)
Surveys without microdata (N = 1584)
All identified sources added to GHDx and tagged as containing information on vaccine coverage
Total sources of vaccine coverage data catalogued in GHDx (N = 3118)
Duplicate sources removed
Duplicated sources (N = 180)
Total sources of vaccine coverage data catalogued in GHDx, after de-duplication (N = 2398)
Sources evaluated against inclusion criteria:
1. Includes data on children aged 12-59 months (or, if country-reported data, from the relevant vaccine-specific target population age range)
2. Nationally representative of the general target population and captures doses delivered through routine immunisation services
3. Includes dose-specific vaccine coverage data from at least one vaccine of interest in or after the country-reported national introduction year
Did not meet one or more of the inclusion criteria (N = 1839)
Sources meeting inclusion criteria (N = 1099)
Data screened by vaccine and cohort for 1) implausible values and 2) observations prior to introduction into national schedule (See Table 2 for details)
Sources with all vaccine-cohort data excluded (N = 124)
Final Input data (N = 975)

Supplementary figure 3. Modelling flow chart. Flow chart object shapes and colours correspond to different modelling processes and input types, as described above (supplementary section 3). Estimation begins with data preparation steps (upper light blue box) and ends with coverage modelling (lower light blue box). JRF=Joint Reporting Form. SDI=Socio-demographic Index. ST-GPR=spatiotemporal Gaussian process regression. HAQ Index=Healthcare Access and Quality Index. MR-BRT=meta-regression—Bayesian, regularised, trimmed. GAM=generalize additive model. UI=uncertainty interval. BCG=Bacillus Calmette-Guérin. DTP1=diphtheria-tetanus-pertussis, first dose. DTP3=diphtheria-tetanus-pertussis, third dose. HepB3=hepatitis B vaccine, third dose. Hib3=*Haemophilus influenzae* type b vaccine, third dose. MCV1=measles-containing vaccine, first dose. MCV2=measles-containing vaccine, second dose. PCV3=pneumococcal conjugate vaccine, third dose. Pol3=polio vaccine, third dose. RCV1=rubella-containing vaccine, first dose. RotaC=completed rotavirus series.

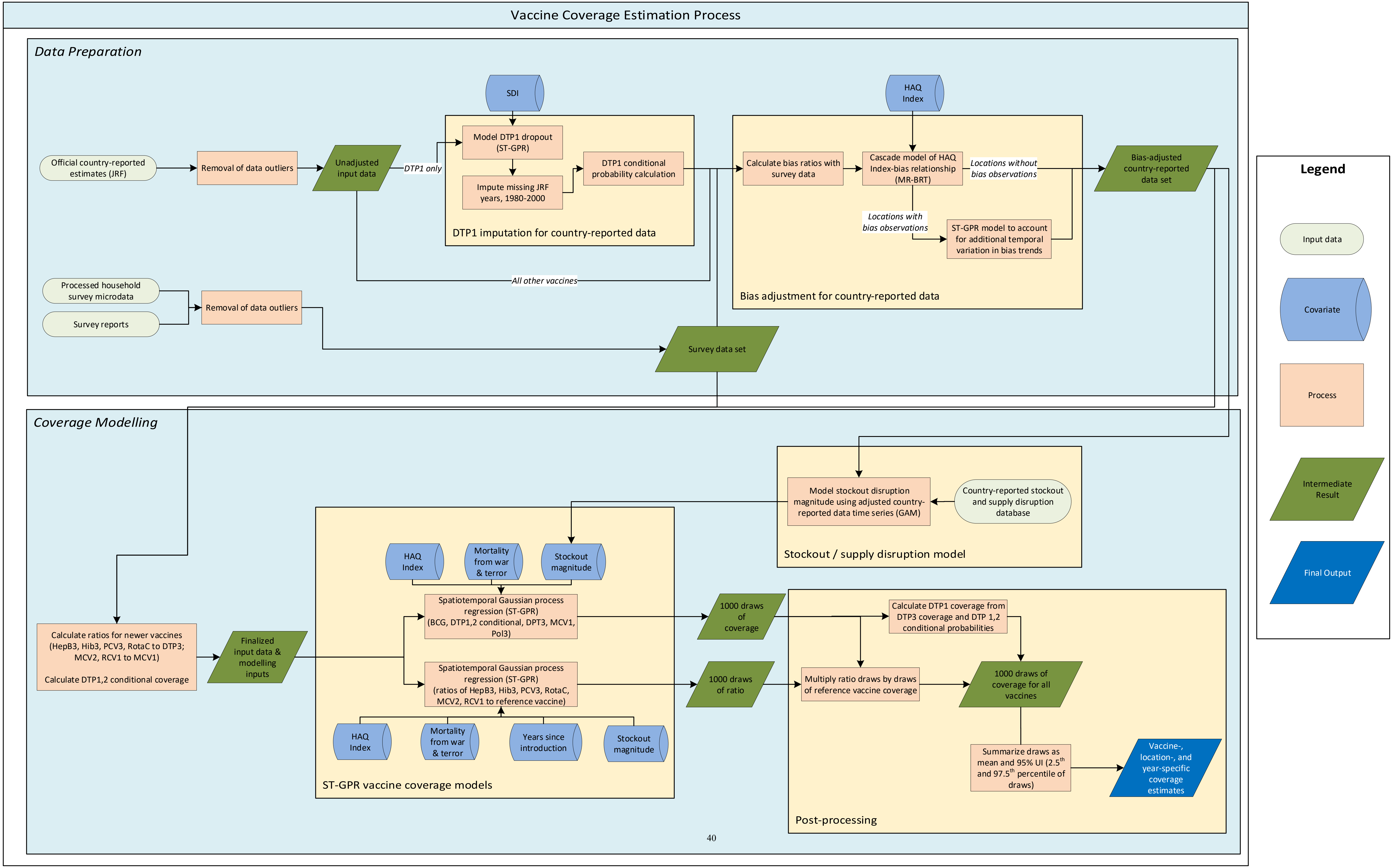

Vaccine Coverage Estimation Process
Data Preparation
SDI
HAQ Index
Official country-reported estimates (JRF)
Removal of data outliers
Unadjusted input data
DTP1 only
Model DTP1 dropout (ST-GPR)
Impute missing JRF years, 1980-2000
DTP1 conditional probability calculation
DTP1 imputation for country-reported data
Calculate bias ratios with survey data
Cascade model of HAQ Index-bias relationship (MR-BRT)
Locations without bias observations
Locations with bias observations
ST-GPR model to account for additional temporal variation in bias trends
Bias adjustment for country-reported data
Bias-adjusted country-reported data set
All other vaccines
Processed household survey microdata
Survey reports
Removal of data outliers
Survey data set
Coverage Modelling
Model stockout disruption magnitude using adjusted country-reported data time series (GAM)
Country-reported stockout and supply disruption database
Stockout / supply disruption model
HAQ Index
Mortality from war & terror
Stockout magnitude
Spatiotemporal Gaussian process regression (ST-GPR) (BCG, DTP1,2 conditional, DPT3, MCV1, Pol3)
Spatiotemporal Gaussian process regression (ST-GPR) (ratios of HepB3, Hib3, PCV3, RotaC, MCV2, RCV1 to reference vaccine)
HAQ Index
Mortality from war & terror
Years since introduction
Stockout magnitude
ST-GPR vaccine coverage models
Calculate ratios for newer vaccines (HepB3, Hib3, PCV3, RotaC to DTP3; MCV2, RCV1 to MCV1)
Calculate DTP1,2 conditional coverage
Finalized input data & modelling inputs
1000 draws of coverage
1000 draws of ratio
Calculate DTP1 coverage from DTP3 coverage and DTP 1,2 conditional probabilities
Multiply ratio draws by draws of reference vaccine coverage
1000 draws of coverage for all vaccines
Summarize draws as mean and 95% UI (2.5th and 97.5th percentile of draws)
Vaccine-, location-, and year-specific coverage estimates
Post-processing
Legend
Input data
Covariate
Process
Intermediate Result
Final Output

Supplementary figure 4. Comparing official country-reported and imputed DPT1 coverage, 1980-1999. For countries with official country-reported DPT1 coverage from the JRF between 1980-1999, each data point represents the unadjusted, reported country-year coverage level (x-axis) plotted against the coverage imputed from the modelling approach described in supplementary section 3.3.1. Data points are shaded by year of coverage represented and labelled with ISO3 country codes when the difference between country-reported and imputed coverage exceeded 10 percentage-points. DTP1=diphtheria-tetanus-pertussis, first-dose. JRF=Joint Reporting Form.

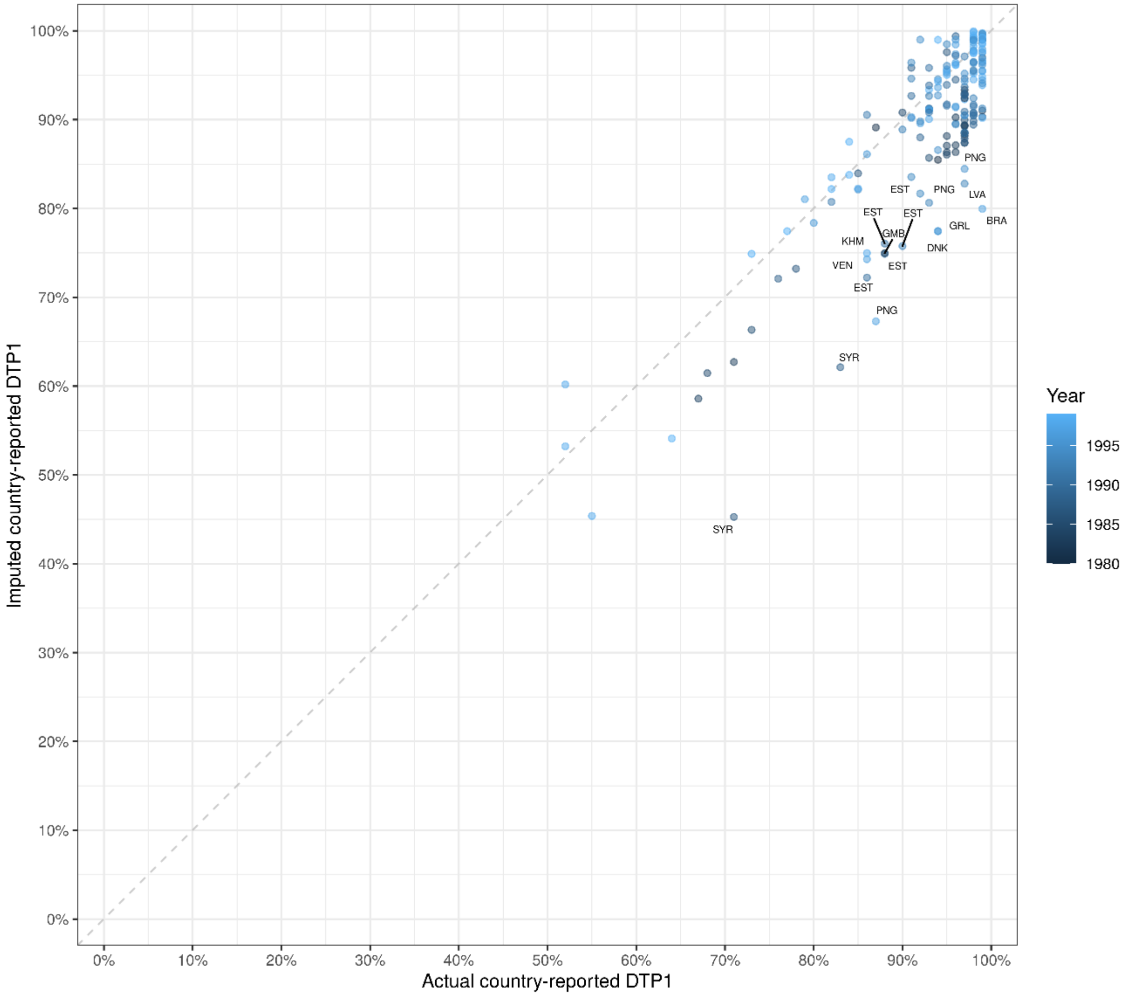

Supplementary figure 5. Global predictions of administrative bias, BCG. Survey-to-administrative ratio data included in the global MR-BRT model of bias are shown in blue, and the data trimmed out of the model are shown in red. Points are scaled by the inverse of the data variance weights. The log of the global bias prediction is shown as the solid black line. The dashed grey line illustrates a no-bias adjustment relationship. Larger distances between each point and the dashed line indicates a larger absolute magnitude of observed survey-to-administrative data bias. Increases in HAQ Index represent higher estimated country HAQ capacity. BCG=Bacillus Calmette-Guérin. MR-BRT=meta-regression—Bayesian, regularised, trimmed. HAQ=Healthcare Access and Quality.

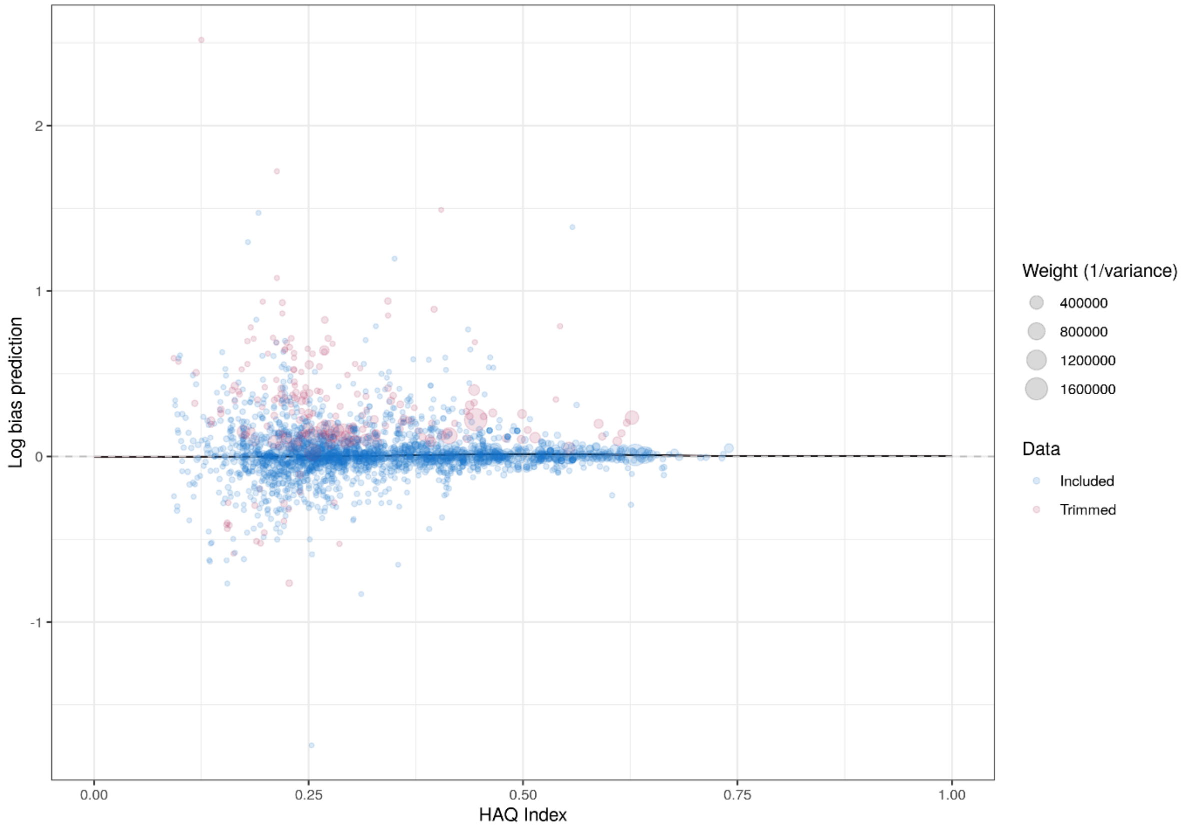

Supplementary figure 6. Global predictions of administrative bias, DTP3. Survey-to-administrative ratio data included in the global MR-BRT model of bias are shown in blue, and the data trimmed out of the model are shown in red. Points are scaled by the inverse of the data variance weights. The log of the global bias prediction is shown as the solid black line. The dashed grey line illustrates a no-bias adjustment relationship. Larger distances between each point and the dashed line indicates a larger absolute magnitude of observed survey-to-administrative data bias. Increases in HAQ Index represent higher estimated country HAQ capacity. DTP3=diphtheria-tetanus-pertussis, third dose. MR-BRT=meta-regression—Bayesian, regularised, trimmed. HAQ=Healthcare Access and Quality.

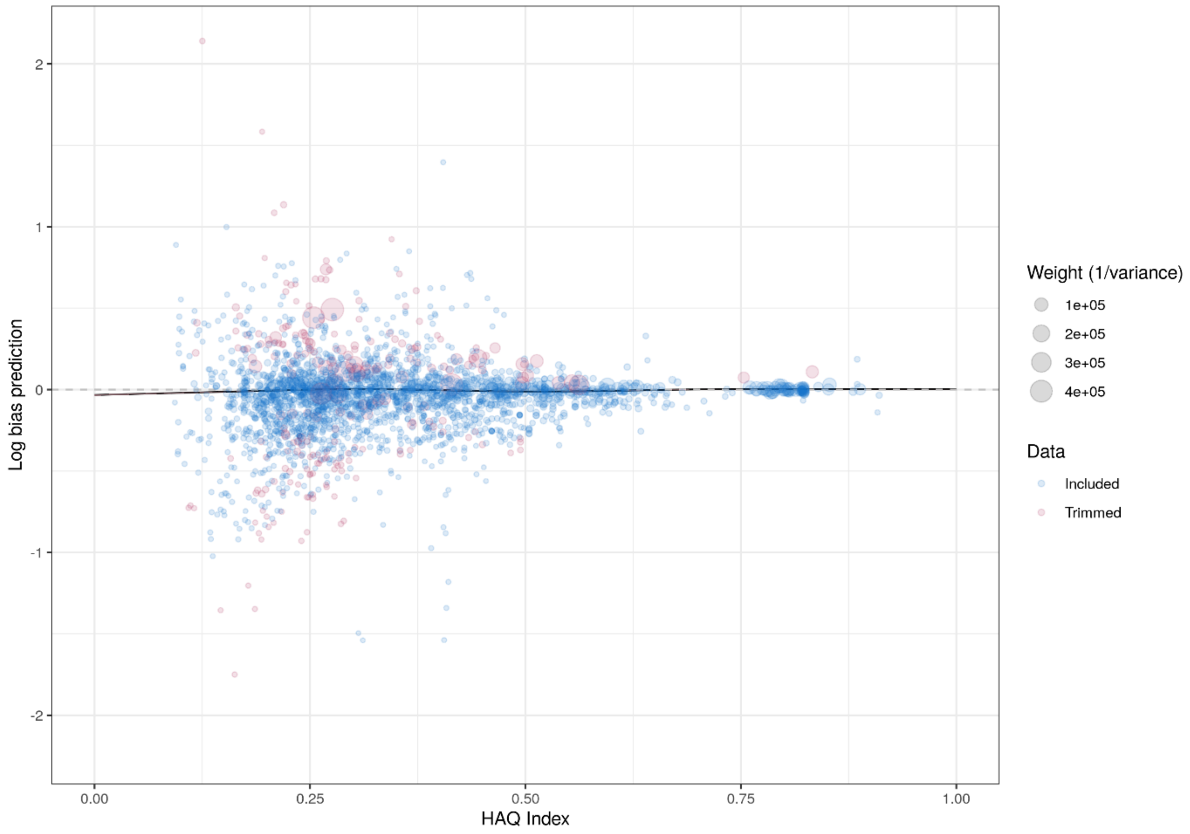

Supplementary figure 7. Global predictions of administrative bias, MCV1. Survey-to-administrative ratio data included in the global MR-BRT model of bias are shown in blue, and the data trimmed out of the model are shown in red. Points are scaled by the inverse of the data variance weights. The log of the global bias prediction is shown as the solid black line. The dashed grey line illustrates a no-bias adjustment relationship. Larger distances between each point and the dashed line indicates a larger absolute magnitude of observed survey-to-administrative data bias. Increases in HAQ Index represent higher estimated country HAQ capacity. MCV1=measles-containing vaccine, first dose. MR-BRT= meta-regression—Bayesian, regularised, trimmed. HAQ=Healthcare Access and Quality.

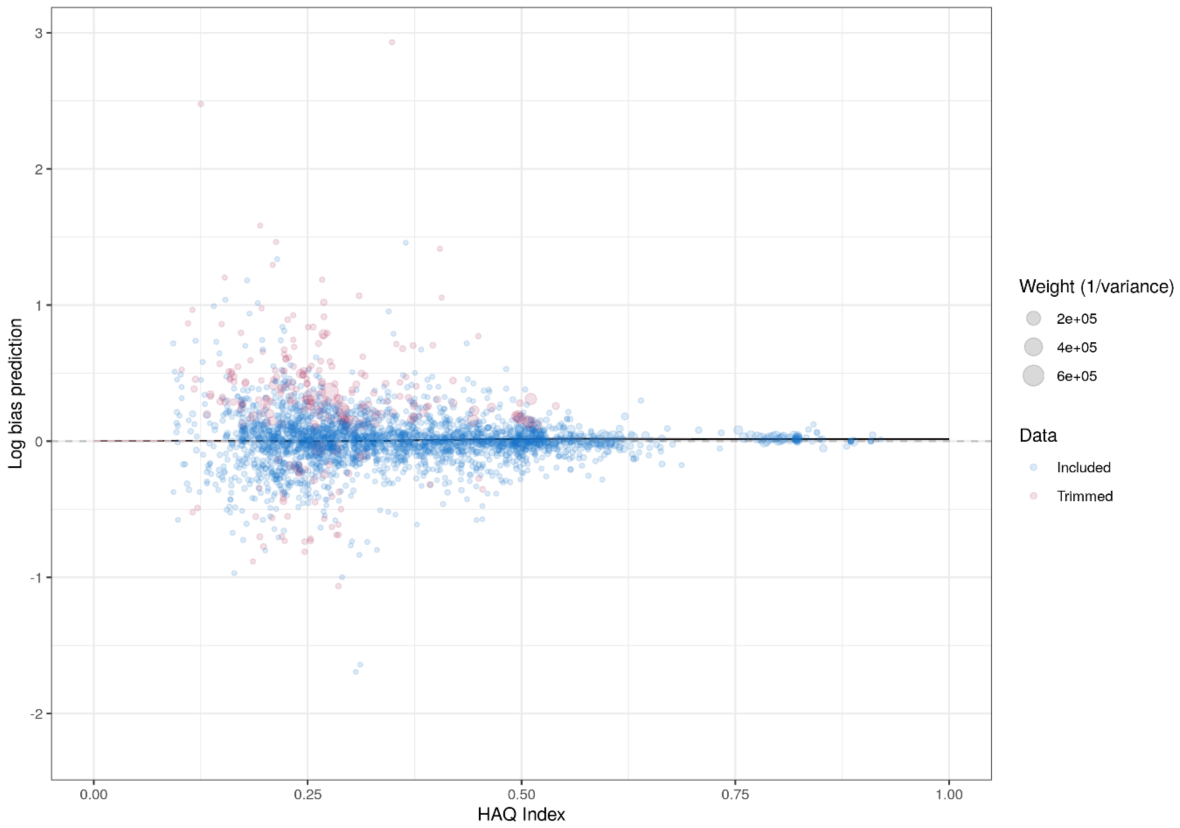

Supplementary figure 8. Global predictions of administrative bias, Pol3. Survey-to-administrative ratio data included in the global MR-BRT model of bias are shown in blue, and the data trimmed out of the model are shown in red. Points are scaled by the inverse of the data variance weights. The log of the global bias prediction is shown as the solid black line. The dashed grey line illustrates a no-bias adjustment relationship. Larger distances between each point and the dashed line indicates a larger absolute magnitude of observed survey-to-administrative data bias. Increases in HAQ Index represent higher estimated country HAQ capacity. Pol3=polio vaccine, third dose. MR-BRT= meta-regression—Bayesian, regularised, trimmed. HAQ=Healthcare Access and Quality.

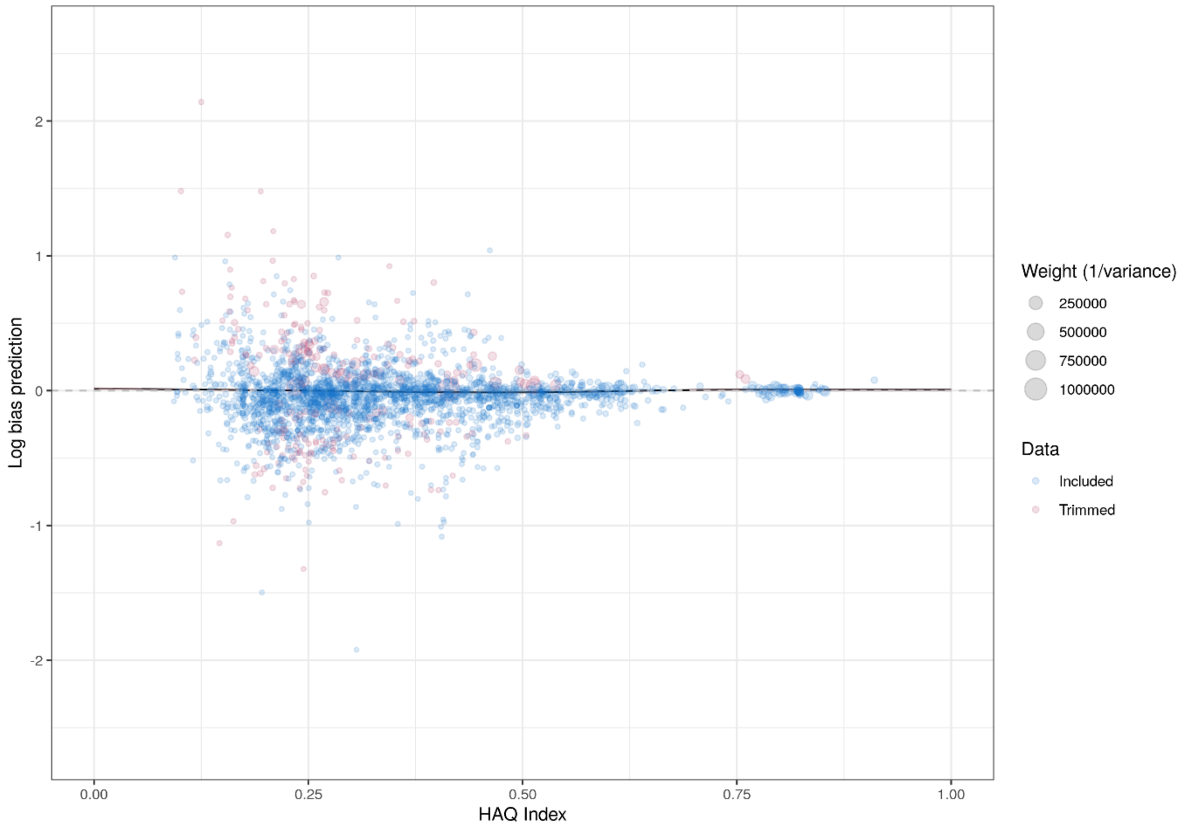

Supplementary figure 9. Changes in MCV1 coverage by decade, 1980-2019. For each decade, maps (left) and scatterplots (right) are colour-coded to reflect absolute changes in MCV1 coverage. Every circle on the scatterplot represents a country or territory, and then the same colour is reflected on the corresponding map. Locations not estimated for GBD are coloured in grey. For the scatterplots, the horizontal dashed line at 0% indicates no coverage change within the decade; the vertical dashed lines mark 60% and 90% coverage, with latter being the national GVAP target for 2020. GVAP=Global Vaccine Action Plan. MCV1=measles-containing vaccine, first dose.

Changes in MCV1 coverage, by decade, from 1980 to 1989 (A), 1990 to 1999 (B), 2000 to 2010 (C), and 2010 to 2019 (D).

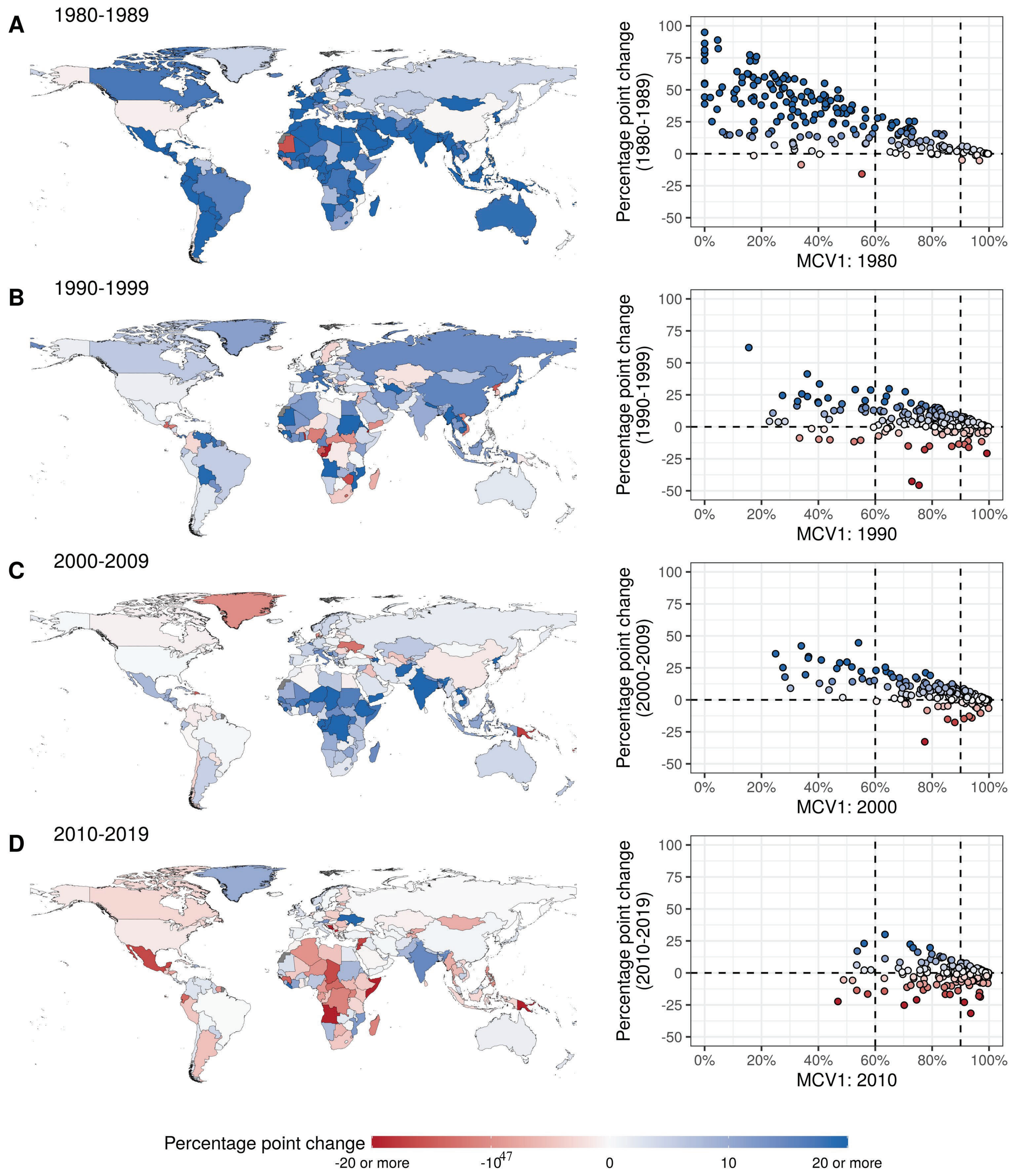

Supplementary figure 10: Changes in Pol3 coverage by decade, 1980-2019. For each decade, maps (left) and scatterplots (right) are colour-coded to reflect absolute changes in Pol3 coverage. Every circle on the scatterplot represents a country or territory, and then the same colour is reflected on the corresponding map. Locations not estimated for GBD are coloured in grey. For the scatterplots, the horizontal dashed line at 0% indicates no coverage change within the decade; the vertical dashed lines mark 60% and 90% coverage, with latter being the national GVAP target for 2020. GBD=Global Burden of Disease. GVAP=Global Vaccine Action Plan. Pol3=polio vaccine, third dose.

Changes in Pol3 coverage, by decade, from 1980 to 1989 (A), 1990 to 1999 (B), 2000 to 2010 (C), and 2010 to 2019 (D).

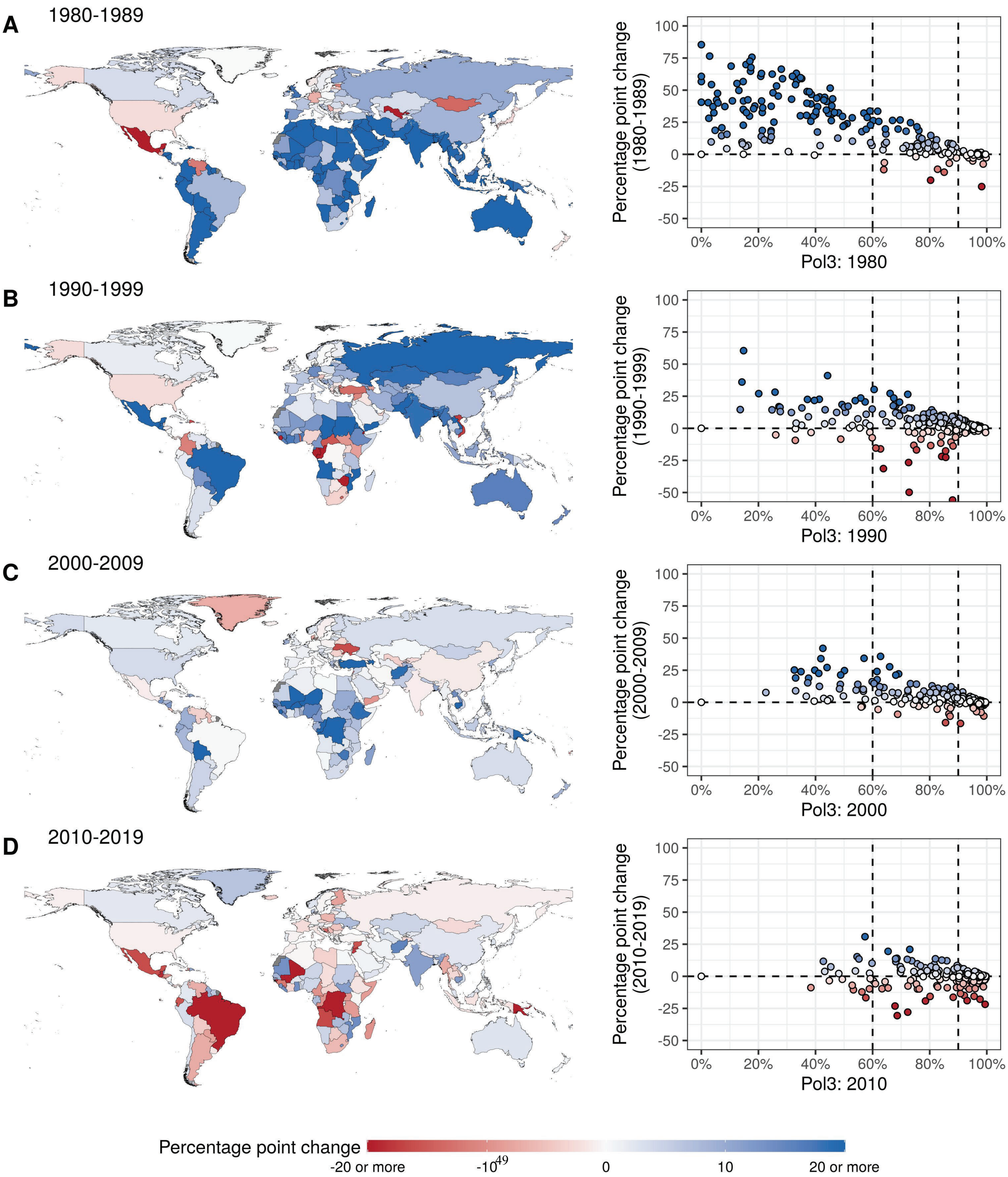

Supplementary figure 11. Transformation of estimates in China using stock data, Hib3. The unadjusted estimate of coverage predicted from ST-GPR is shown as the red line. The 95% unadjusted uncertainty interval is represented by the shaded red band around the mean estimate. The transformed estimates of coverage are shown as the blue line. The stock data used to transform the estimates are shown as the green points in all available years. The upper shaded blue band around the blue transformed estimate of coverage represents the theoretical maximum coverage. Hib3=*Haemophilus influenzae* type b vaccine, third dose. ST-GPR=spatio-temporal Gaussian process regression.

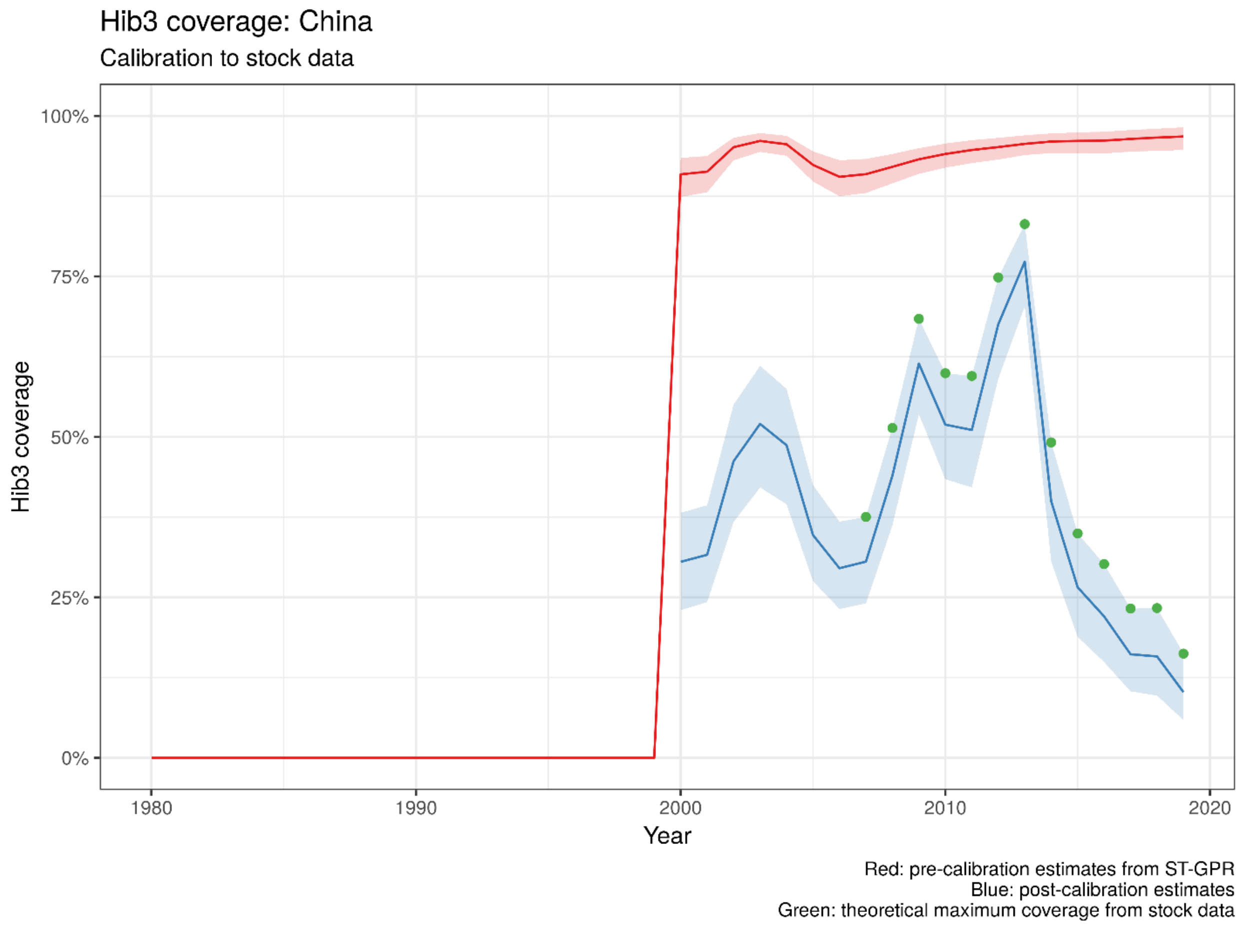

Supplementary figure 12. Transformation of estimates in China using stock data, PCV3. The unadjusted estimate of coverage predicted from ST-GPR is shown as the red line. The 95% unadjusted uncertainty interval is represented by the shaded red band around the mean estimate. The transformed estimates of coverage are shown as the blue line. The stock data used to transform the estimates are shown as the green points in all available years. The upper shaded blue band around the blue transformed estimate of coverage represents the theoretical maximum coverage. PCV3=pneumococcal conjugate vaccine, third dose. ST-GPR=spatio-temporal Gaussian process regression.

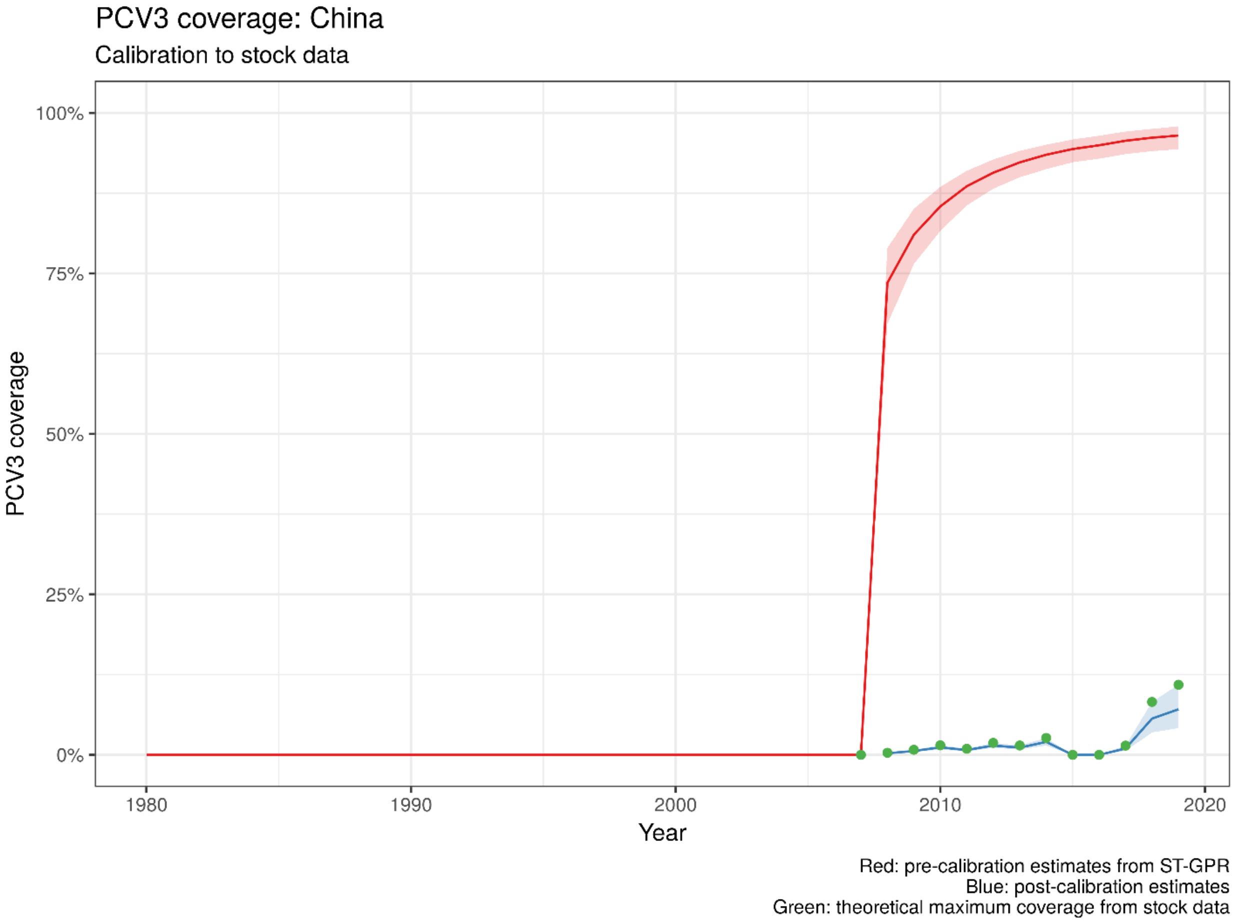

Supplementary figure 13. Transformation of estimates in China using stock data, RotaC. The unadjusted estimate of coverage predicted from ST-GPR is shown as the red line. The 95% unadjusted uncertainty interval is represented by the shaded red band around the mean estimate. The transformed estimates of coverage are shown as the blue line. The stock data used to transform the estimates are shown as the green points in all available years. The upper shaded blue band around the blue transformed estimate of coverage represents the theoretical maximum coverage. RotaC=completed rotavirus series. ST-GPR=spatio-temporal Gaussian process regression.

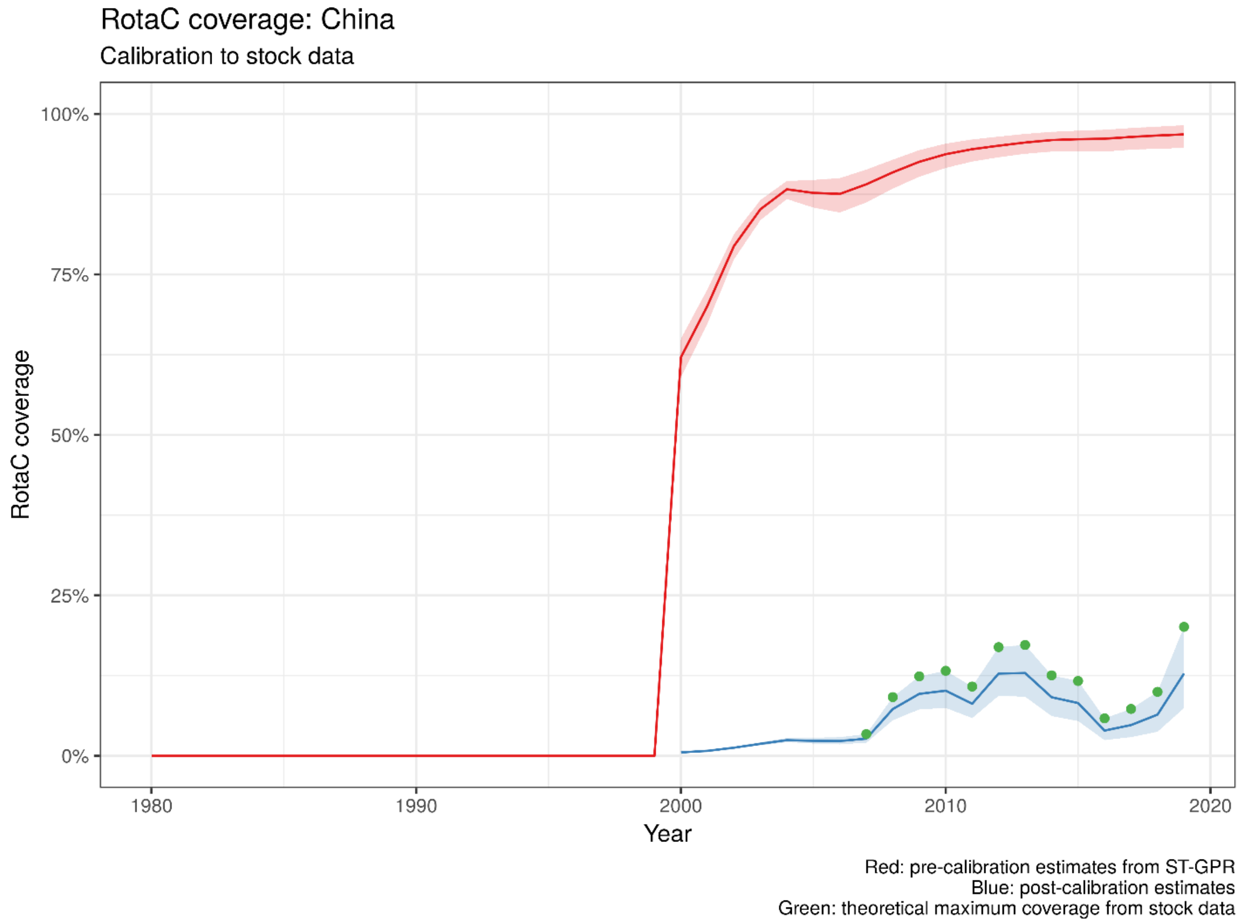

Supplementary figure 14. Time series of vaccination coverage by vaccine and GBD super-region, 1980–2019. GVAP target of reaching at least 90% coverage by 2020 is represented by the dotted line. 95% uncertainty intervals are represented the lighter-coloured shading surrounding the darker mean estimates. GVAP=Global Vaccine Action Plan. DTP1=diphtheria-tetanus-pertussis, first dose. DTP3=diphtheria-tetanus-pertussis, third dose. HepB3=hepatitis B vaccine, third dose. Hib3=*Haemophilus influenzae* type b vaccine, third dose. MCV1=measles-containing vaccine, first dose. MCV2=measles-containing vaccine, second dose. PCV3=pneumococcal conjugate vaccine, third dose. Pol3=polio vaccine, third dose. RCV1=rubella-containing vaccine, first dose. RotaC=completed rotavirus series.

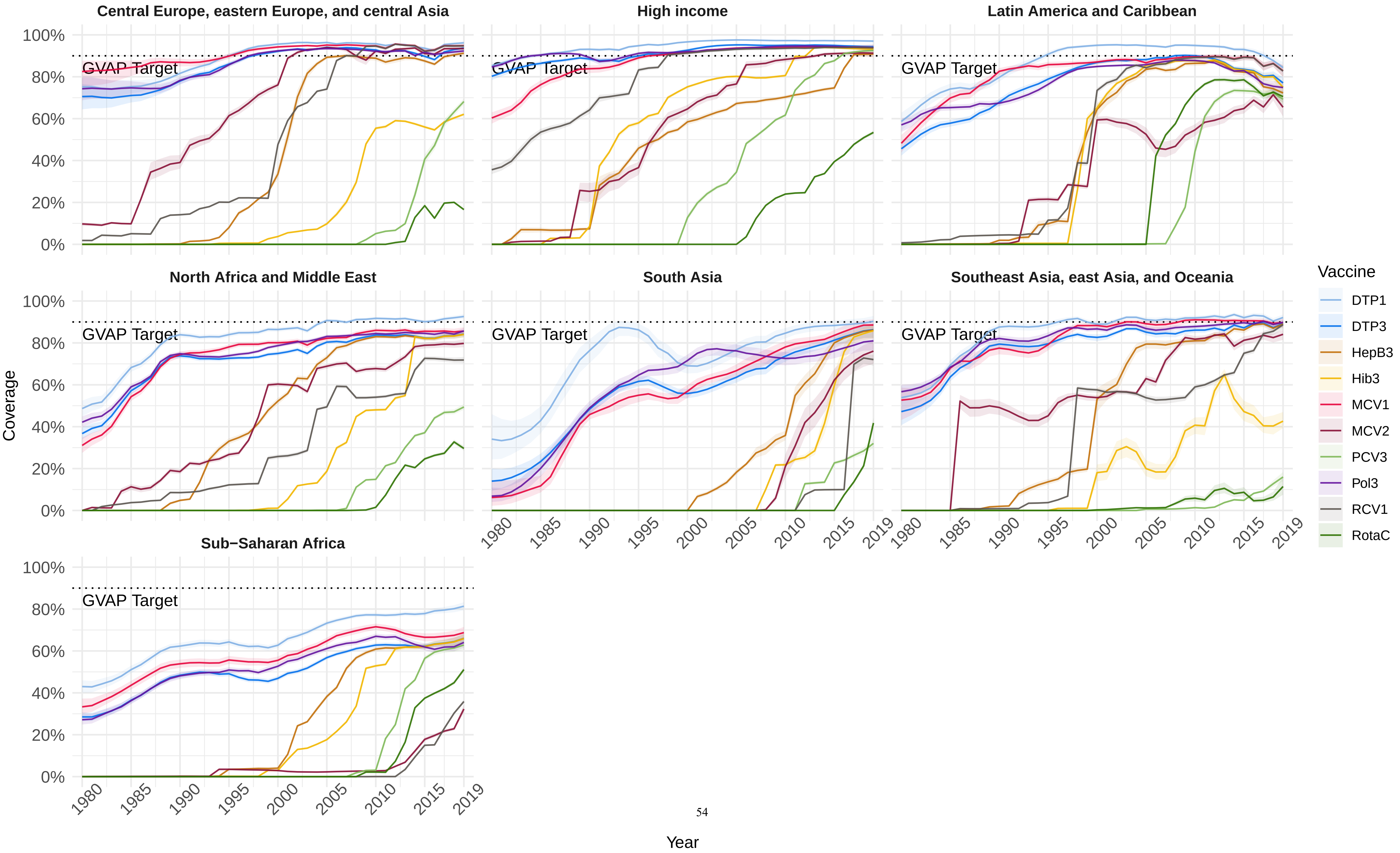
Central Europe, eastern Europe, and central Asia
High income
Latin America and Caribbean
North Africa and Middle East
South Asia
Southeast Asia, east Asia, and Oceania
Sub−Saharan Africa
GVAP Target
Coverage
100%
80%
60%
40%
20%
0%
1980
1985
1990
1995
2000
2005
2010
2015
2019
Year
Vaccine
DTP1
DTP3
HepB3
Hib3
MCV1
MCV2
PCV3
Pol3
RCV1
RotaC

Supplementary figure 15. Observed coverage trend versus expected coverage given SDI, by country, DTP3. Bolded red curve with associated 95% uncertainty represents the mean expected relationship between coverage and SDI from a MR-BRT model. The observed SDI and DTP3 coverage trajectories for each country are shown in light grey. In each location-specific panel, the colour of each point represents the year of estimation, from yellow (1980) to purple (2019), for that country or territory. SDI=Socio-demographic Index. DTP3=diphtheria-tetanus-pertussis, third dose. MR-BRT=meta-regression—Bayesian, regularised, trimmed.

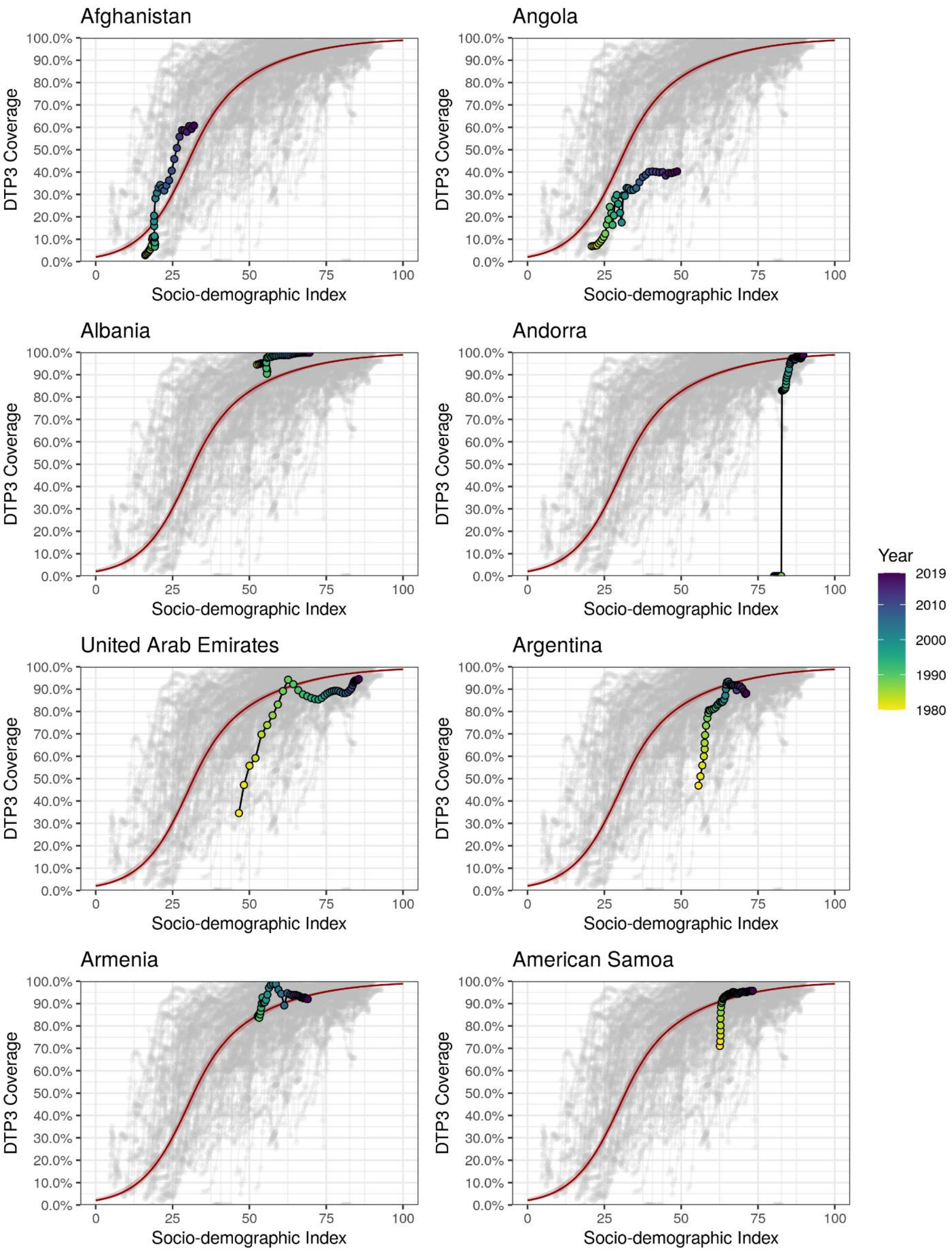

Afghanistan
Angola
Albania
Andorra
United Arab Emirates
Argentina
Armenia
American Samoa
DTP3 Coverage
0.0%
10.0%
20.0%
30.0%
40.0%
50.0%
60.0%
70.0%
80.0%
90.0%
100.0%
Socio-demographic Index
0
25
50
75
100
Year
2019
2010
2000
1990
1980

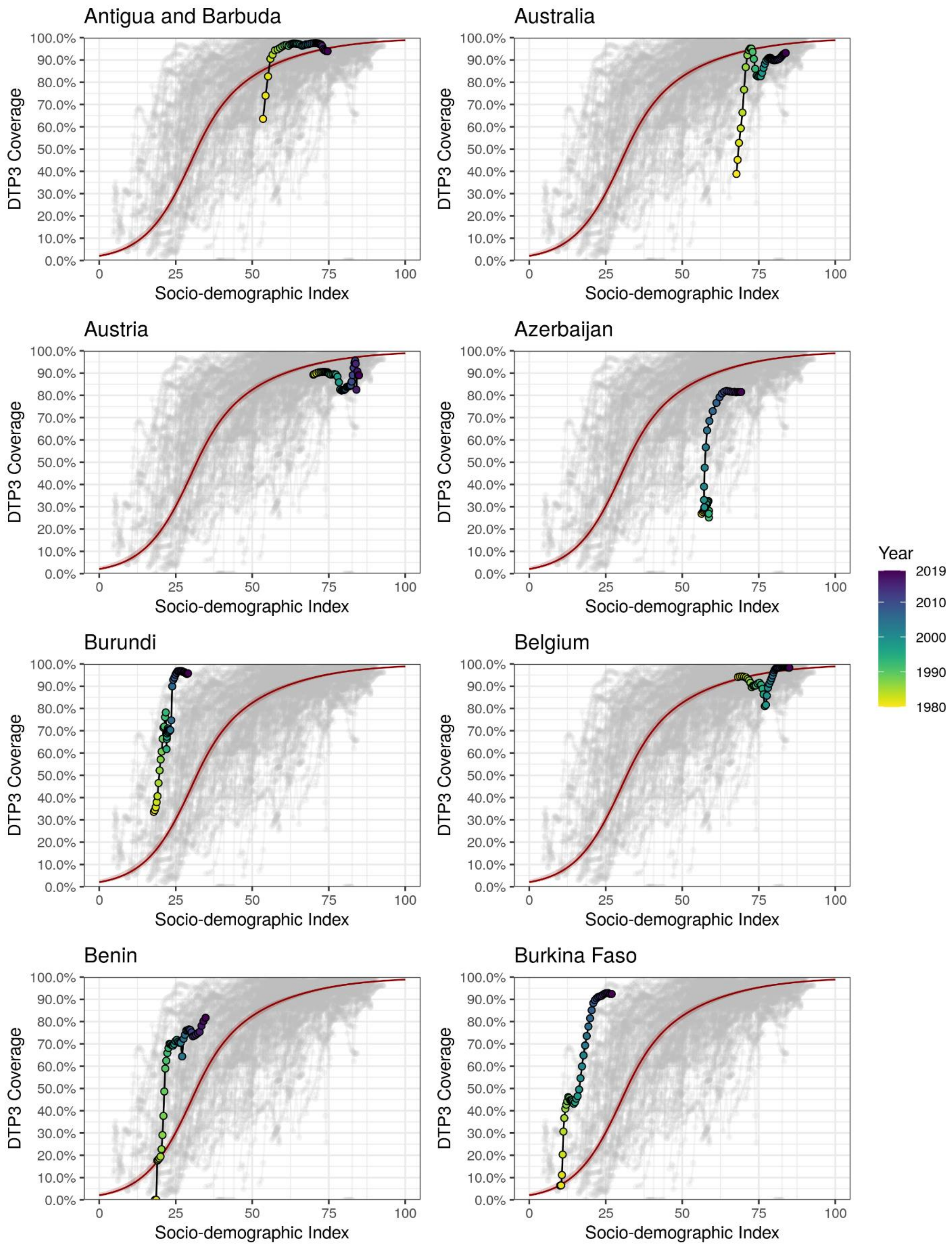

Antigua and Barbuda
Australia
Austria
Azerbaijan
Burundi
Belgium
Benin
Burkina Faso
DTP3 Coverage
Socio-demographic Index
100.0%
90.0%
80.0%
70.0%
60.0%
50.0%
40.0%
30.0%
20.0%
10.0%
0.0%
0
25
50
75
100
Year
2019
2010
2000
1990
1980

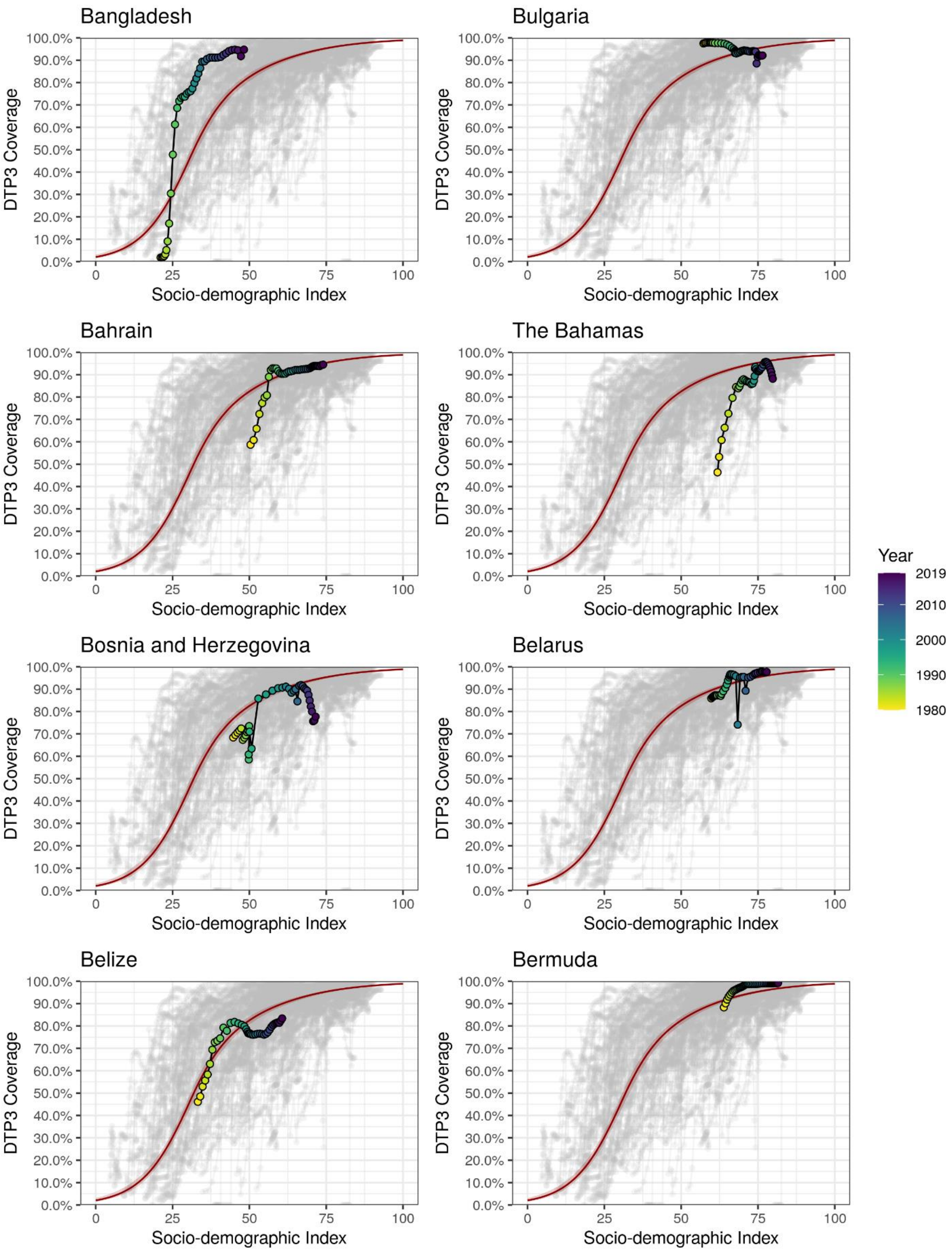
Bangladesh
Bulgaria
Bahrain
The Bahamas
Bosnia and Herzegovina
Belarus
Belize
Bermuda
DTP3 Coverage
Socio-demographic Index
0.0%
10.0%
20.0%
30.0%
40.0%
50.0%
60.0%
70.0%
80.0%
90.0%
100.0%
0
25
50
75
100
Year
2019
2010
2000
1990
1980

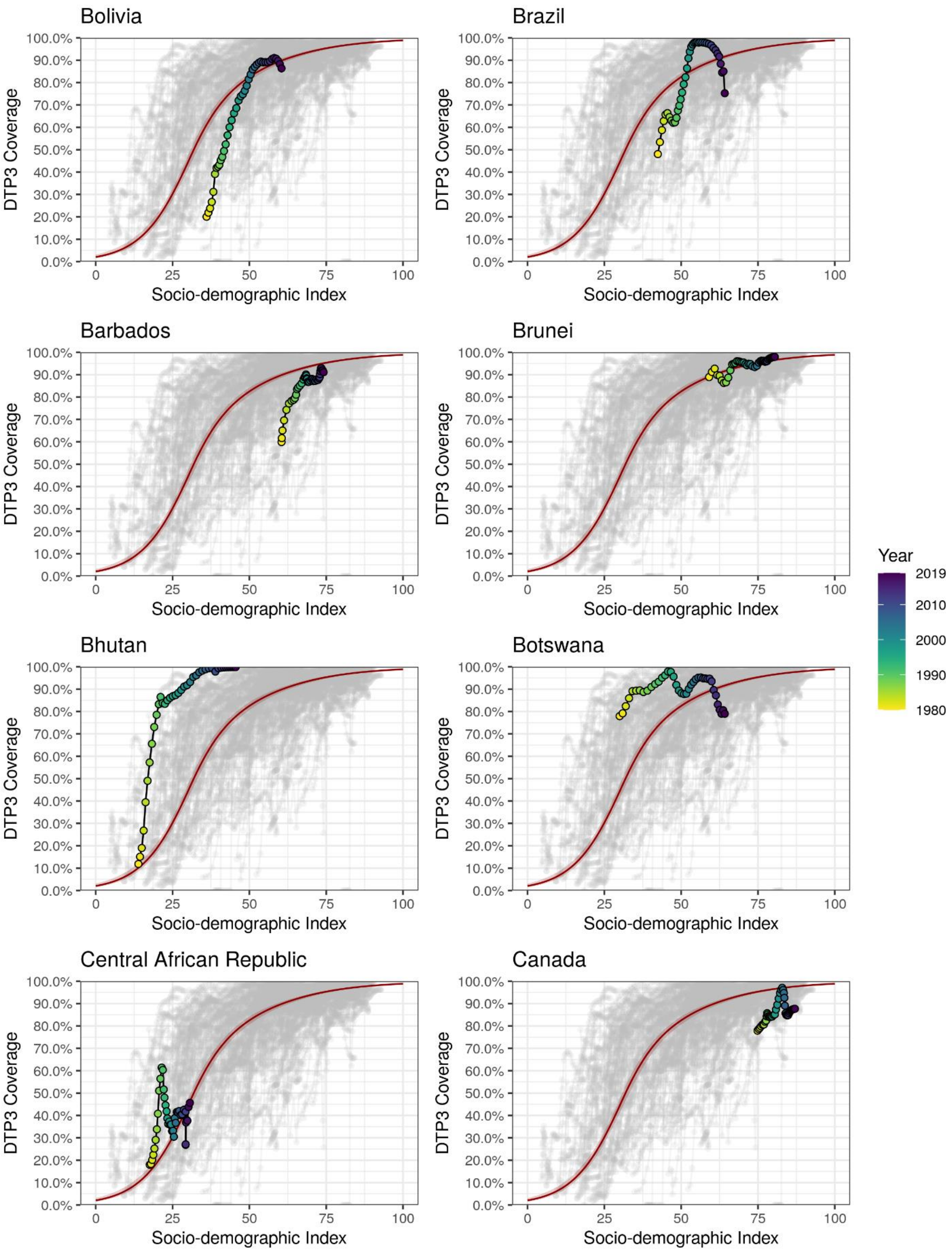
Bolivia
Brazil
Barbados
Brunei
Bhutan
Botswana
Central African Republic
Canada
DTP3 Coverage
Socio-demographic Index
100.0%
90.0%
80.0%
70.0%
60.0%
50.0%
40.0%
30.0%
20.0%
10.0%
0.0%
0
25
50
75
100
Year
2019
2010
2000
1990
1980

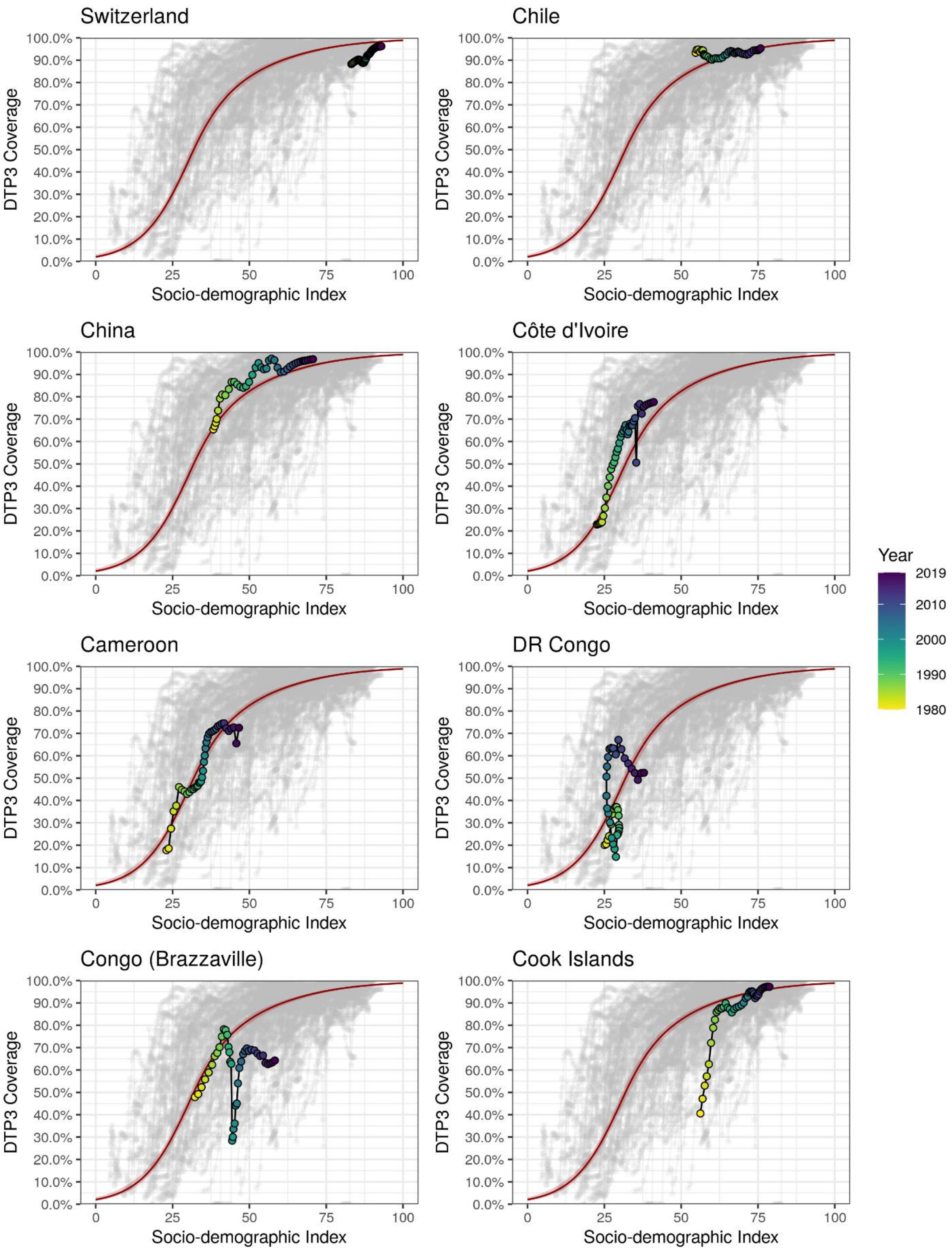
Switzerland
Chile
China
Côte d'Ivoire
Cameroon
DR Congo
Congo (Brazzaville)
Cook Islands
DTP3 Coverage
Socio-demographic Index
Year
2019
2010
2000
1990
1980

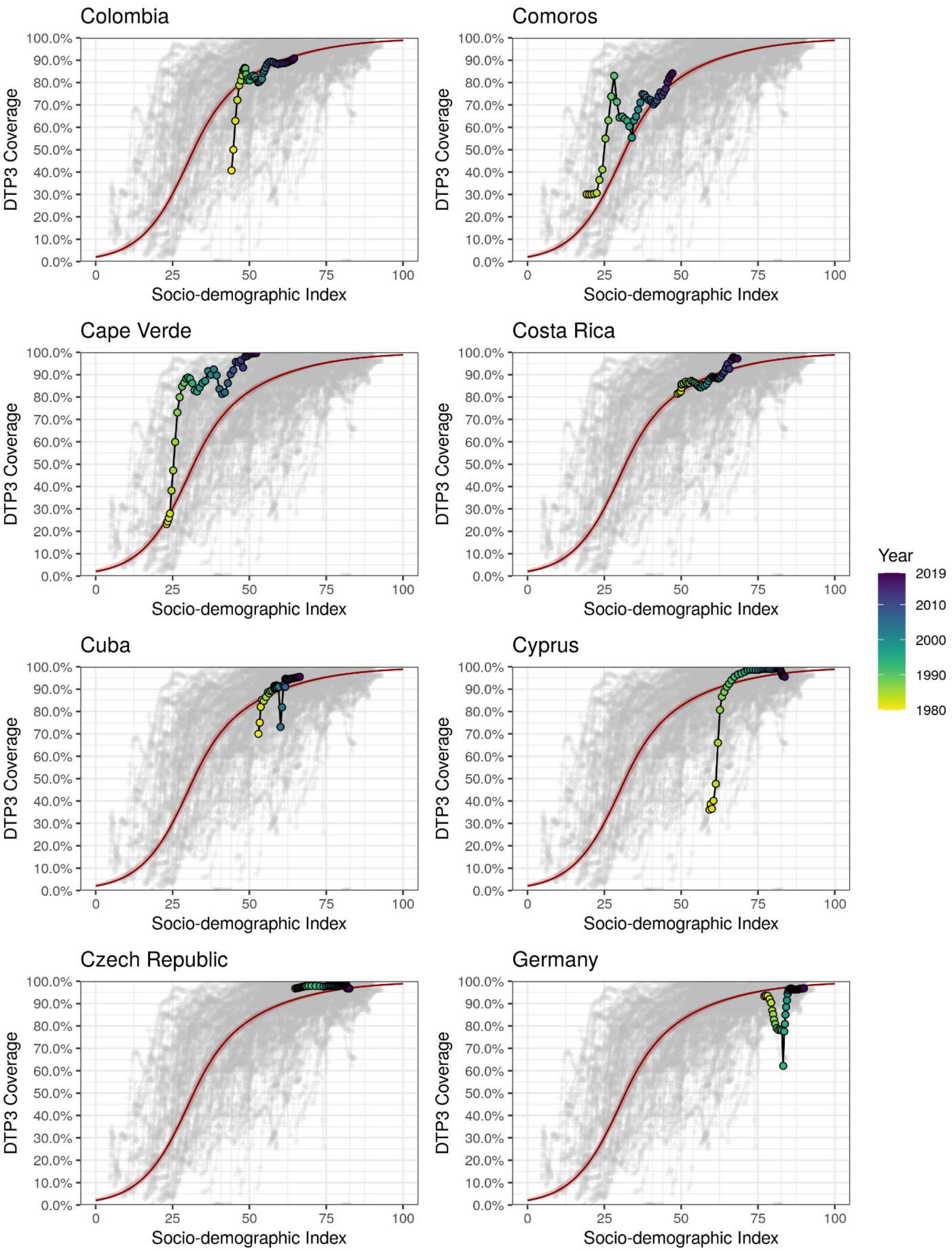
Colombia
Comoros
Cape Verde
Costa Rica
Cuba
Cyprus
Czech Republic
Germany
DTP3 Coverage
Socio-demographic Index
100.0%
90.0%
80.0%
70.0%
60.0%
50.0%
40.0%
30.0%
20.0%
10.0%
0.0%
0
25
50
75
100
Year
2019
2010
2000
1990
1980

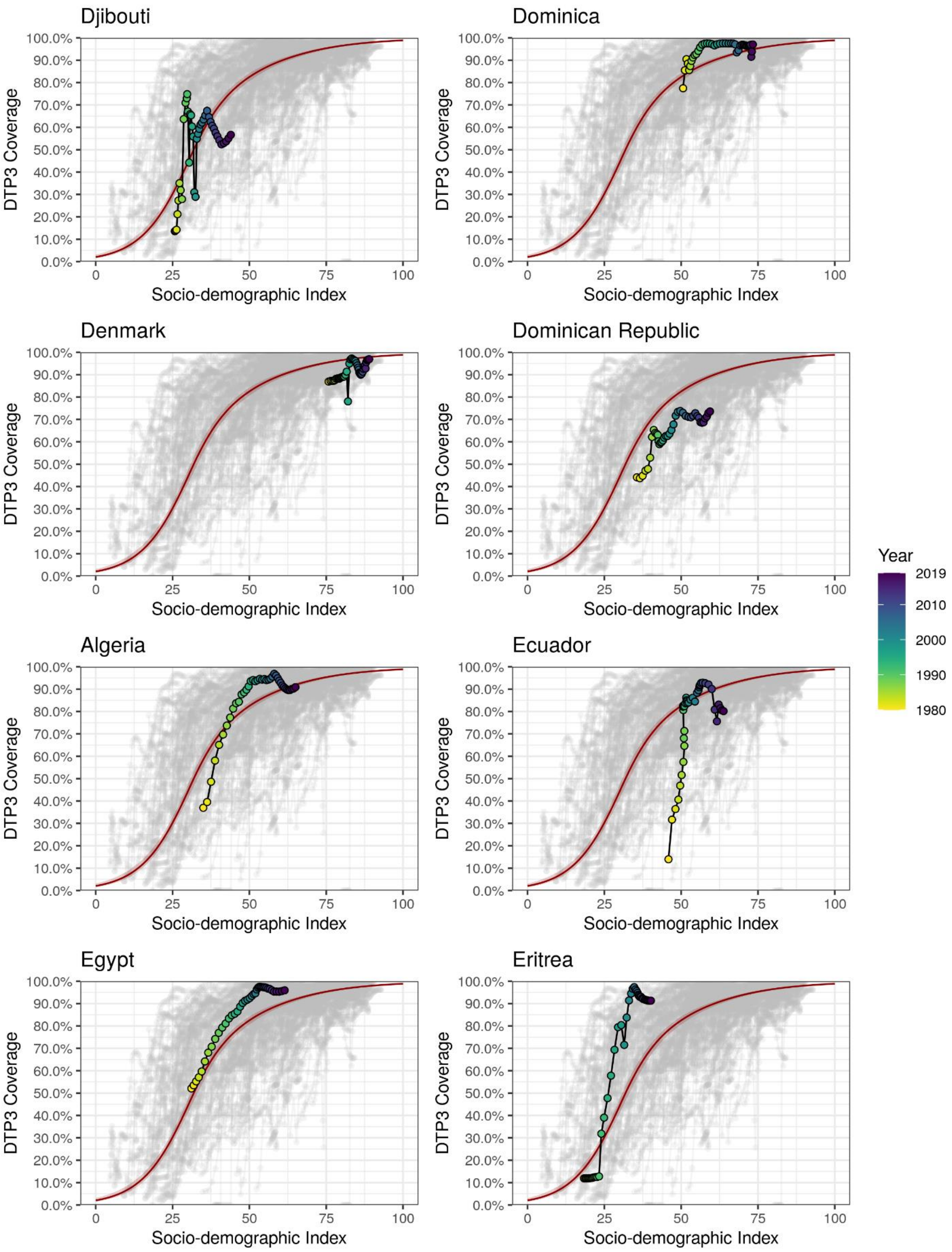
Djibouti
Dominica
Denmark
Dominican Republic
Algeria
Ecuador
Egypt
Eritrea
DTP3 Coverage
100.0%
90.0%
80.0%
70.0%
60.0%
50.0%
40.0%
30.0%
20.0%
10.0%
0.0%
0
25
50
75
100
Socio-demographic Index
Year
2019
2010
2000
1990
1980

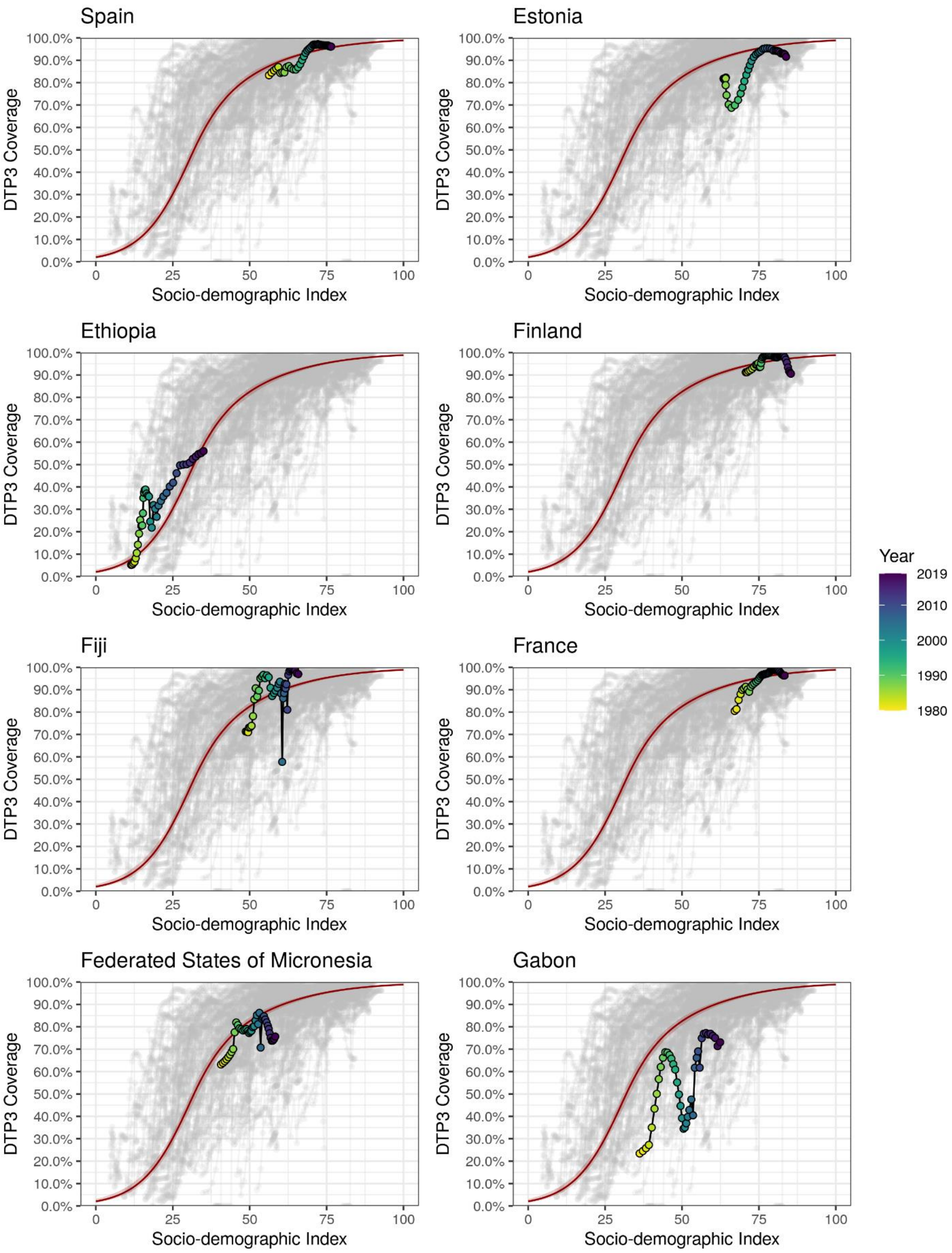
Spain
Estonia
Ethiopia
Finland
Fiji
France
Federated States of Micronesia
Gabon
DTP3 Coverage
100.0%
90.0%
80.0%
70.0%
60.0%
50.0%
40.0%
30.0%
20.0%
10.0%
0.0%
0
25
50
75
100
Socio-demographic Index
Year
2019
2010
2000
1990
1980

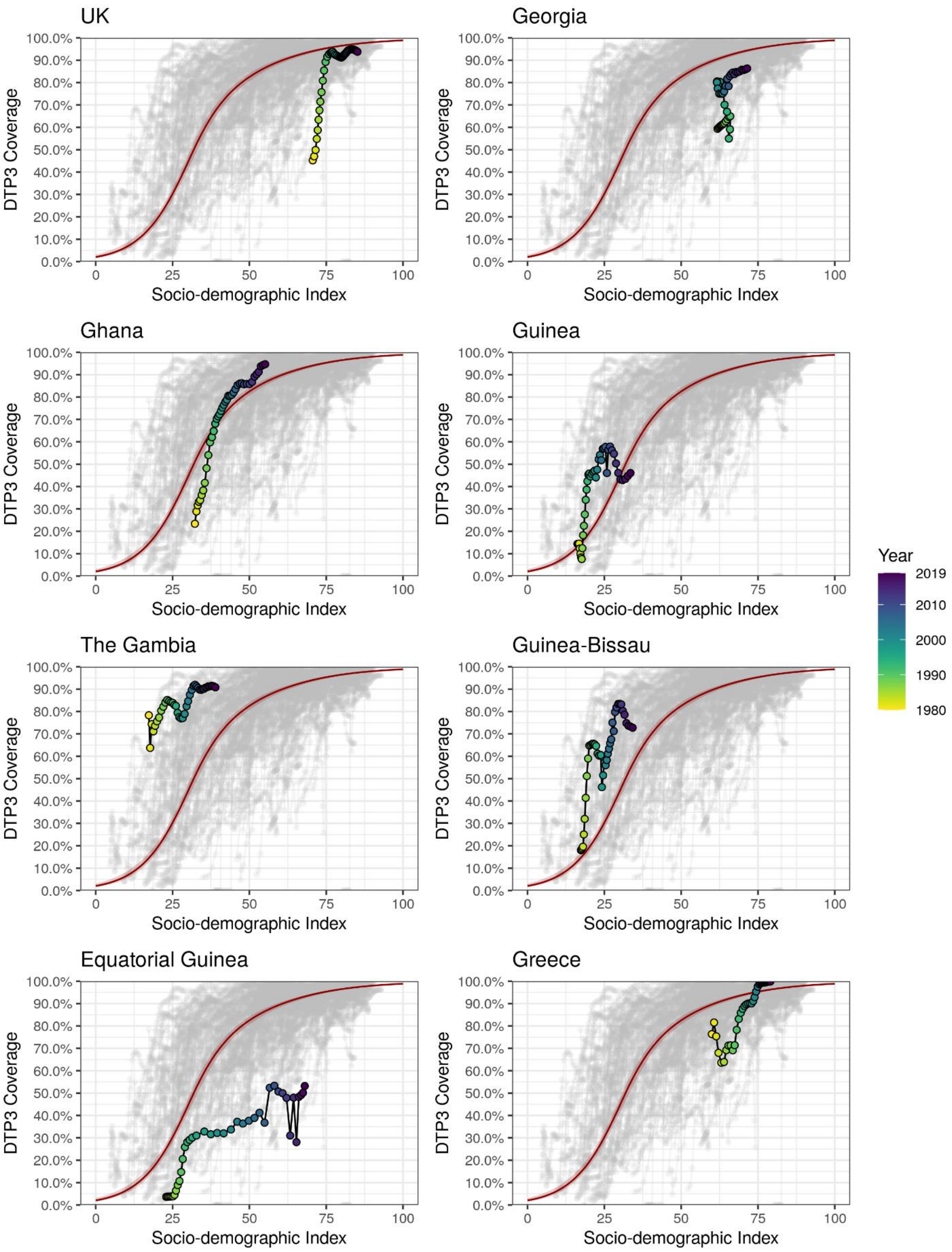
UK
Georgia
Ghana
Guinea
The Gambia
Guinea-Bissau
Equatorial Guinea
Greece
DTP3 Coverage
Socio-demographic Index
0.0%
10.0%
20.0%
30.0%
40.0%
50.0%
60.0%
70.0%
80.0%
90.0%
100.0%
0
25
50
75
100
Year
2019
2010
2000
1990
1980

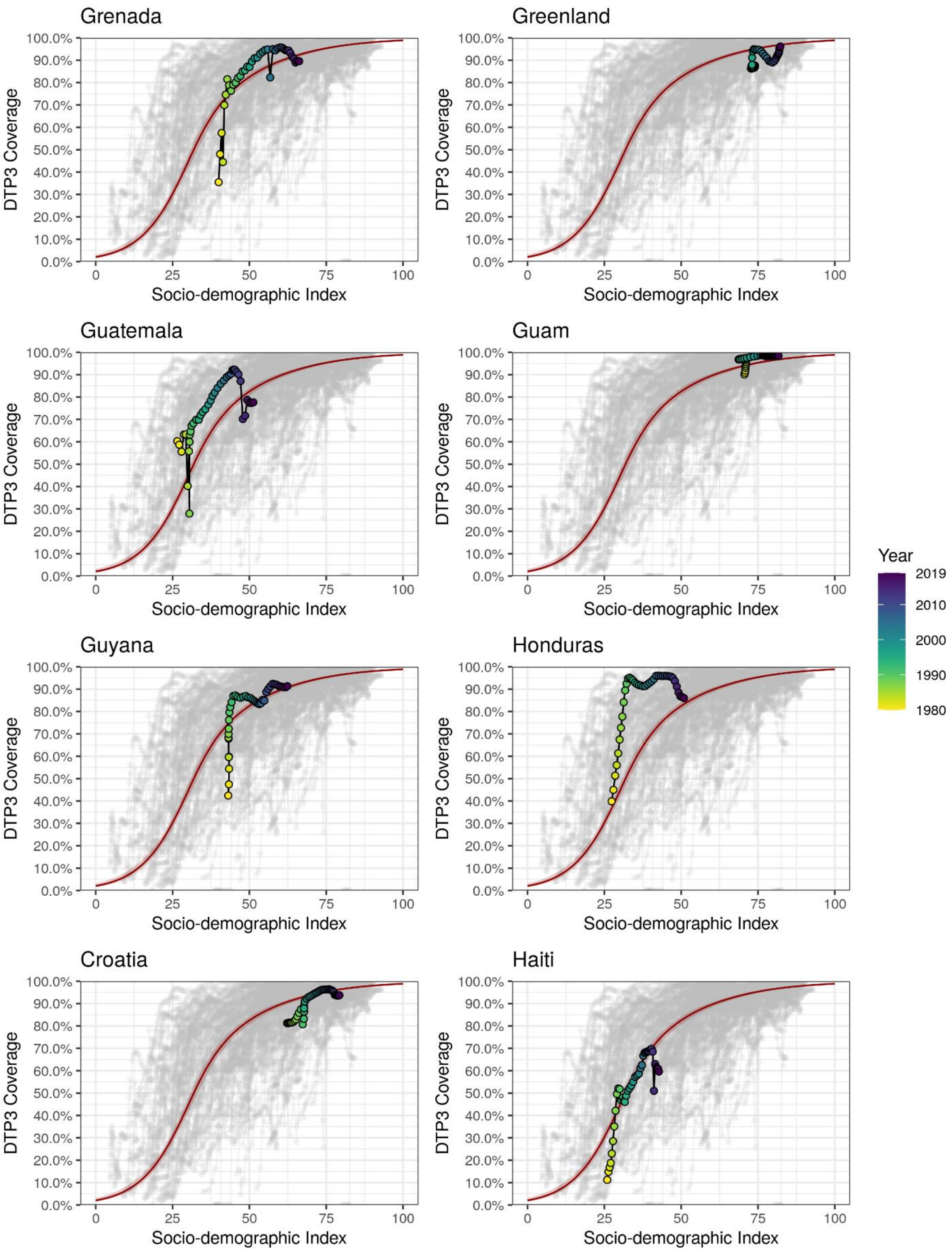
Grenada
Greenland
Guatemala
Guam
Guyana
Honduras
Croatia
Haiti
DTP3 Coverage
Socio-demographic Index
0.0%
10.0%
20.0%
30.0%
40.0%
50.0%
60.0%
70.0%
80.0%
90.0%
100.0%
0
25
50
75
100
Year
2019
2010
2000
1990
1980

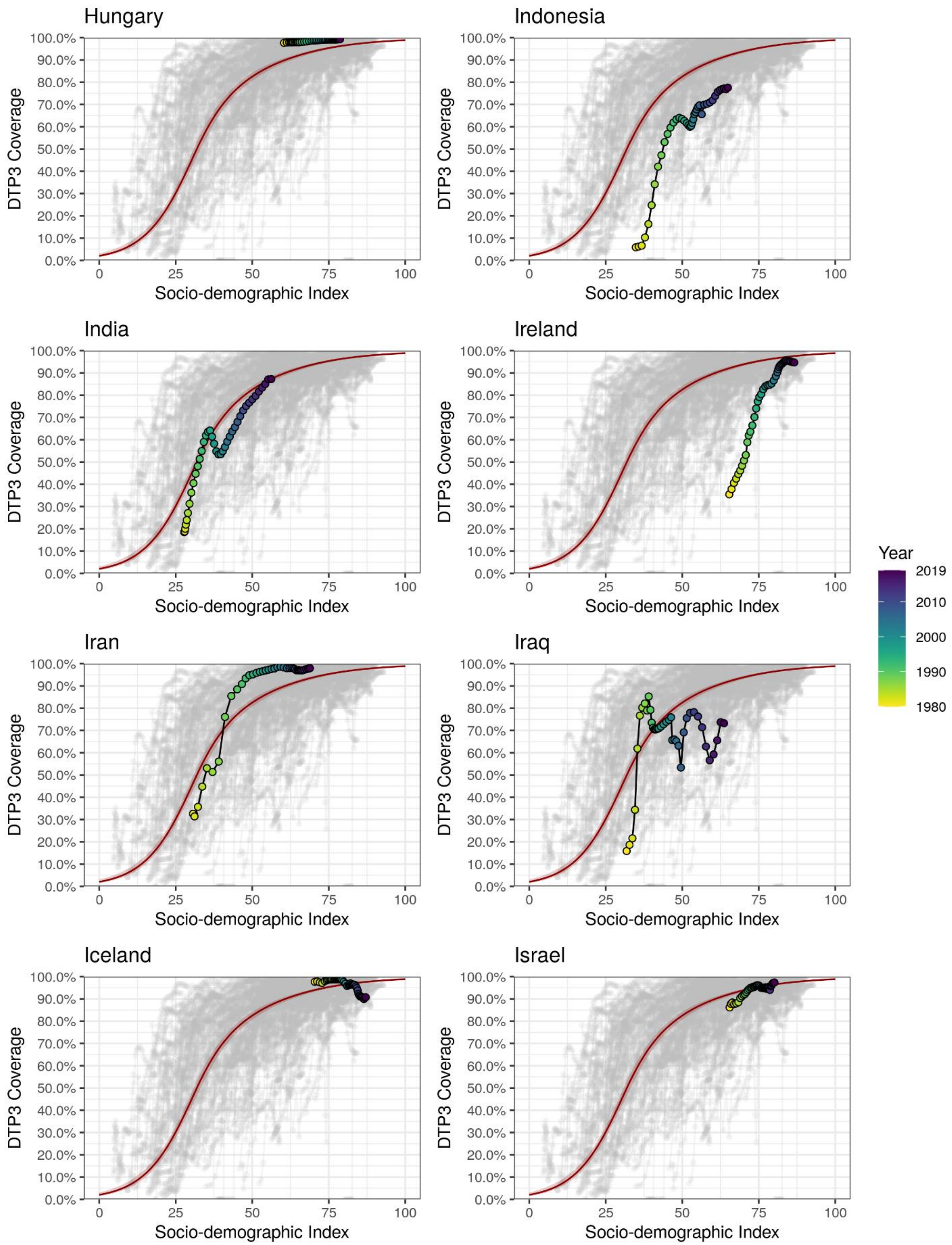
Hungary
Indonesia
India
Ireland
Iran
Iraq
Iceland
Israel
DTP3 Coverage
Socio-demographic Index
0.0%
10.0%
20.0%
30.0%
40.0%
50.0%
60.0%
70.0%
80.0%
90.0%
100.0%
0
25
50
75
100
Year
2019
2010
2000
1990
1980

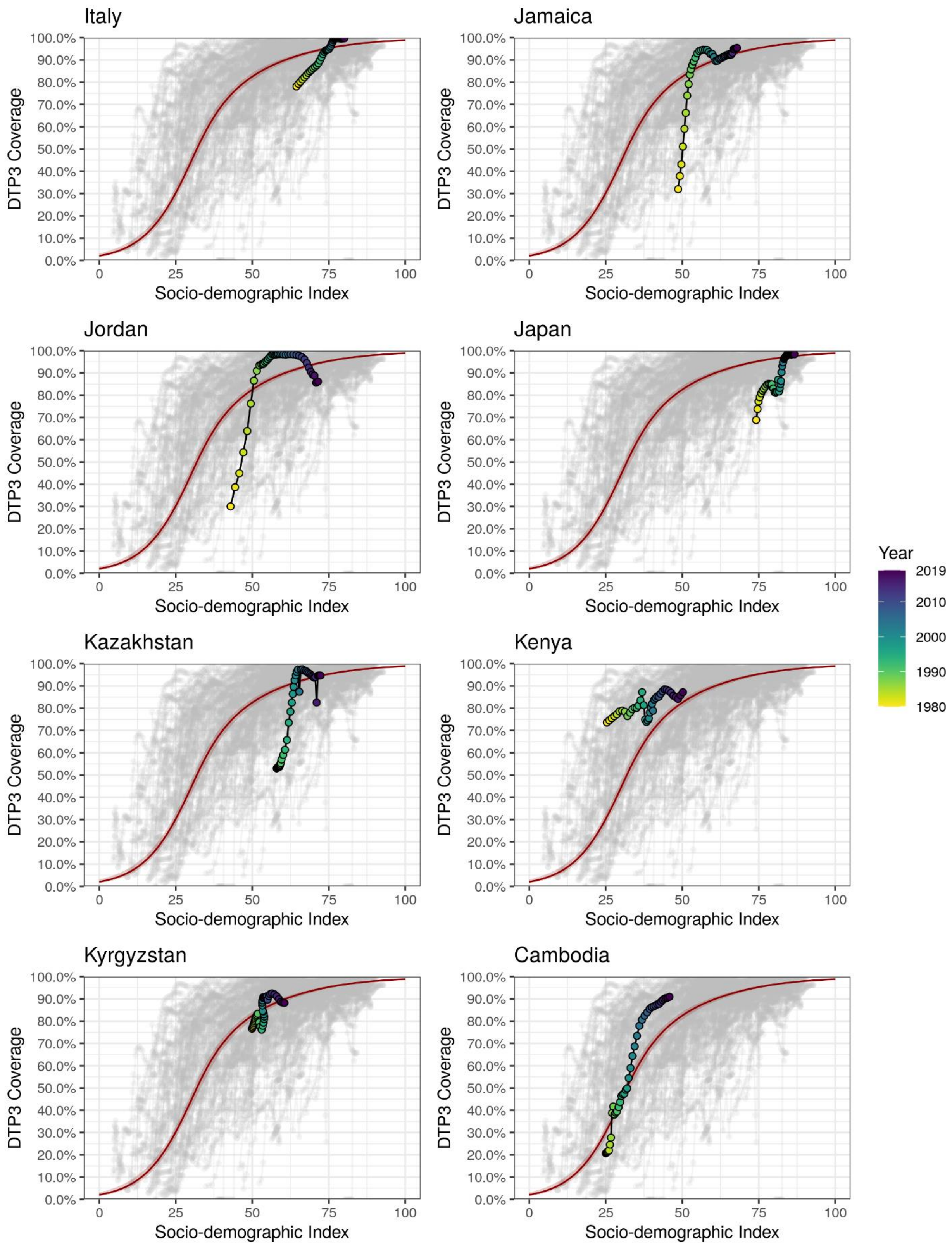
Italy
Jamaica
Jordan
Japan
Kazakhstan
Kenya
Kyrgyzstan
Cambodia
DTP3 Coverage
Socio-demographic Index
0.0%
10.0%
20.0%
30.0%
40.0%
50.0%
60.0%
70.0%
80.0%
90.0%
100.0%
0
25
50
75
100
Year
2019
2010
2000
1990
1980

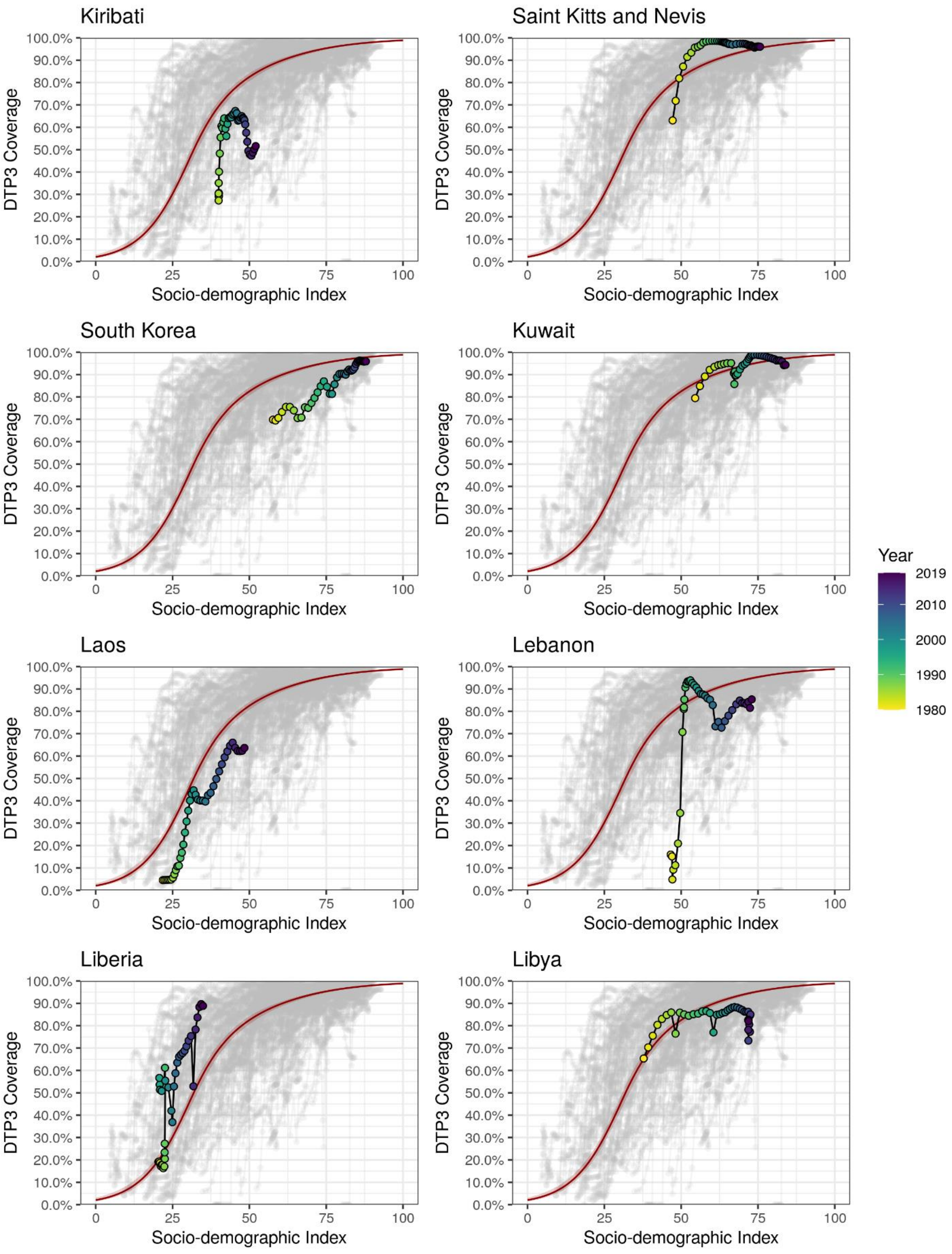

Kiribati
Saint Kitts and Nevis
South Korea
Kuwait
Laos
Lebanon
Liberia
Libya
DTP3 Coverage
Socio-demographic Index
100.0%
90.0%
80.0%
70.0%
60.0%
50.0%
40.0%
30.0%
20.0%
10.0%
0.0%
0
25
50
75
100
Year
2019
2010
2000
1990
1980

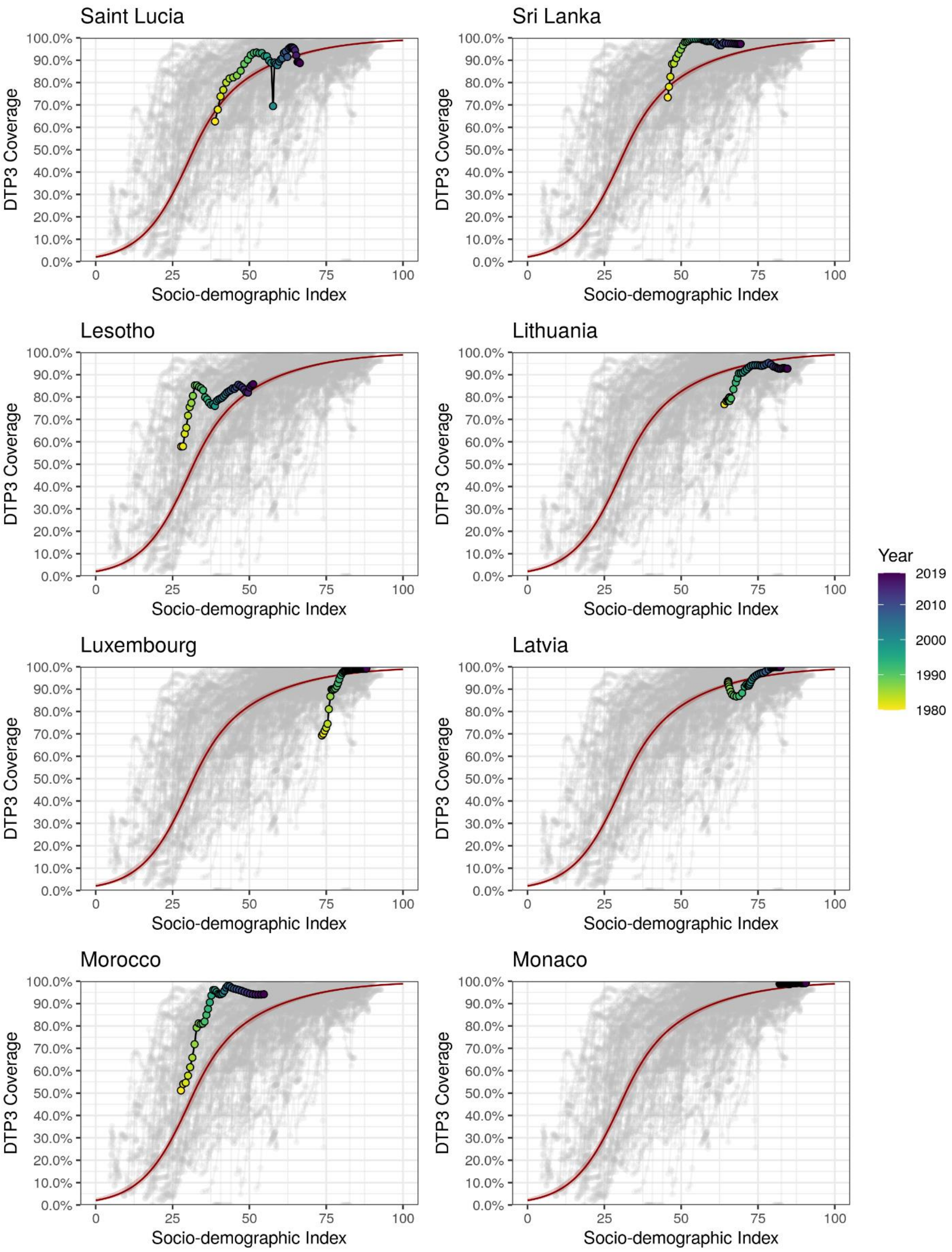
Saint Lucia
Sri Lanka
Lesotho
Lithuania
Luxembourg
Latvia
Morocco
Monaco
DTP3 Coverage
0.0%
10.0%
20.0%
30.0%
40.0%
50.0%
60.0%
70.0%
80.0%
90.0%
100.0%
Socio-demographic Index
0
25
50
75
100
Year
2019
2010
2000
1990
1980

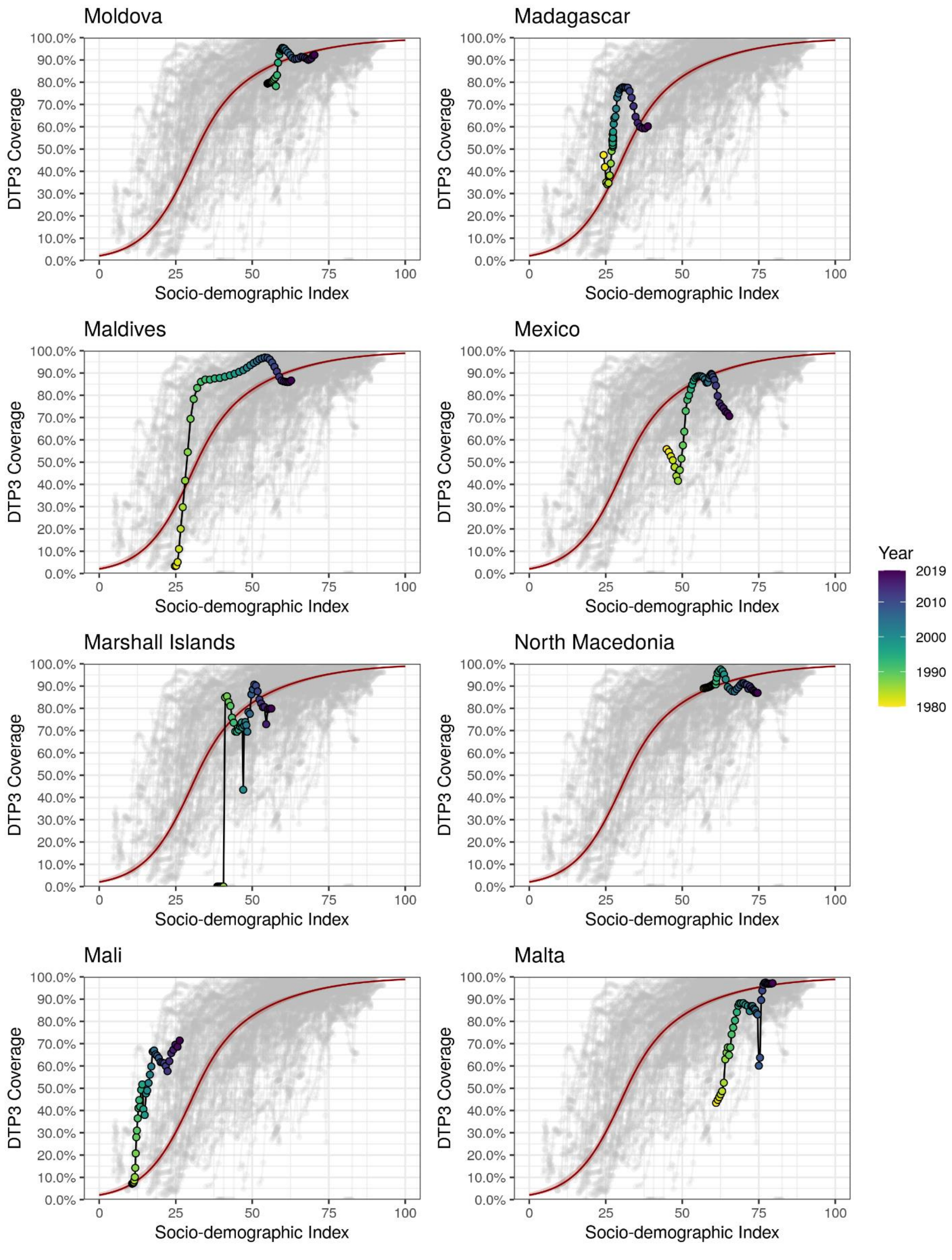

Moldova
Madagascar
Maldives
Mexico
Marshall Islands
North Macedonia
Mali
Malta
DTP3 Coverage
Socio-demographic Index
100.0%
90.0%
80.0%
70.0%
60.0%
50.0%
40.0%
30.0%
20.0%
10.0%
0.0%
0
25
50
75
100
Year
2019
2010
2000
1990
1980

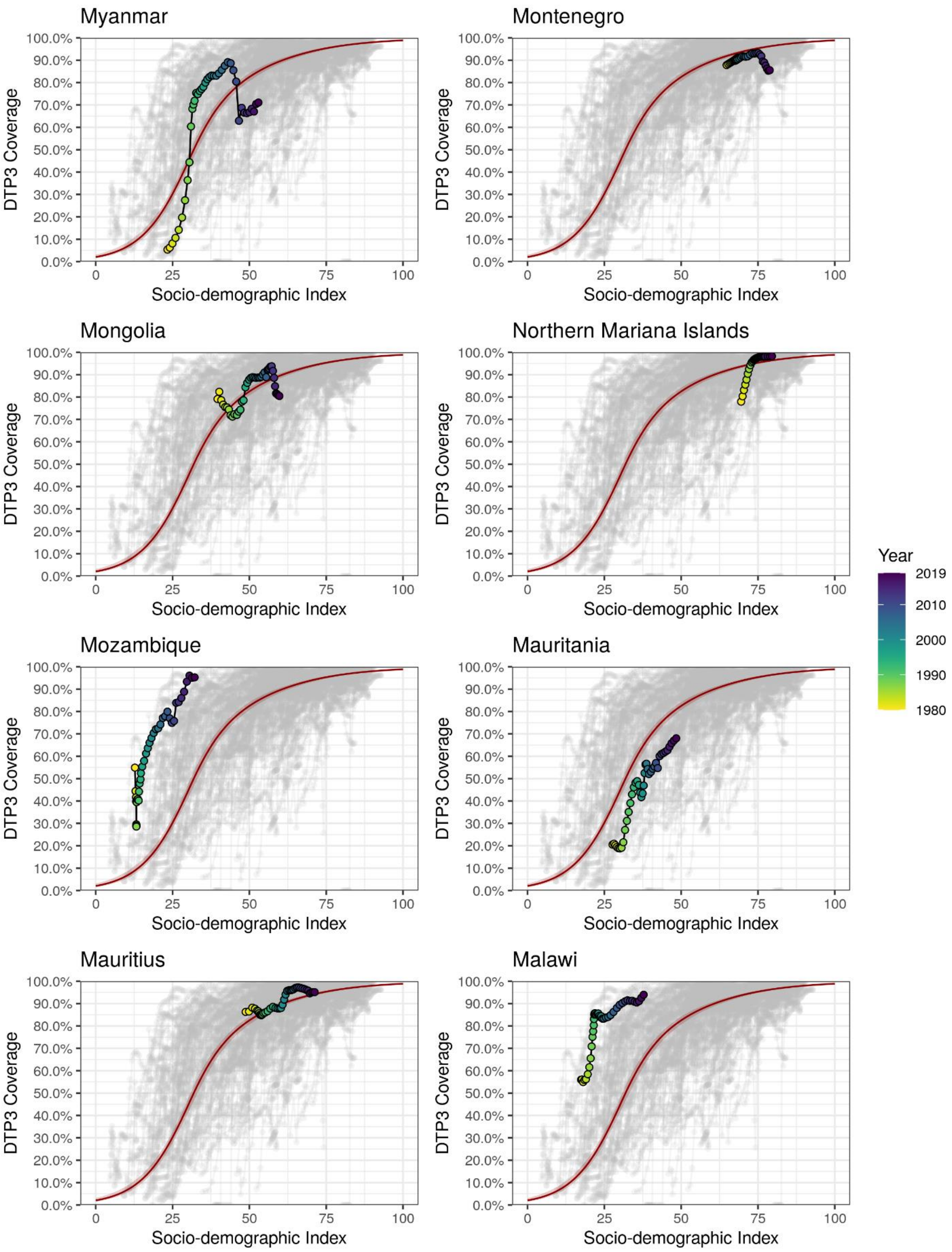
Myanmar
Montenegro
Mongolia
Northern Mariana Islands
Mozambique
Mauritania
Mauritius
Malawi
DTP3 Coverage
Socio-demographic Index
0.0%
10.0%
20.0%
30.0%
40.0%
50.0%
60.0%
70.0%
80.0%
90.0%
100.0%
0
25
50
75
100
Year
2019
2010
2000
1990
1980

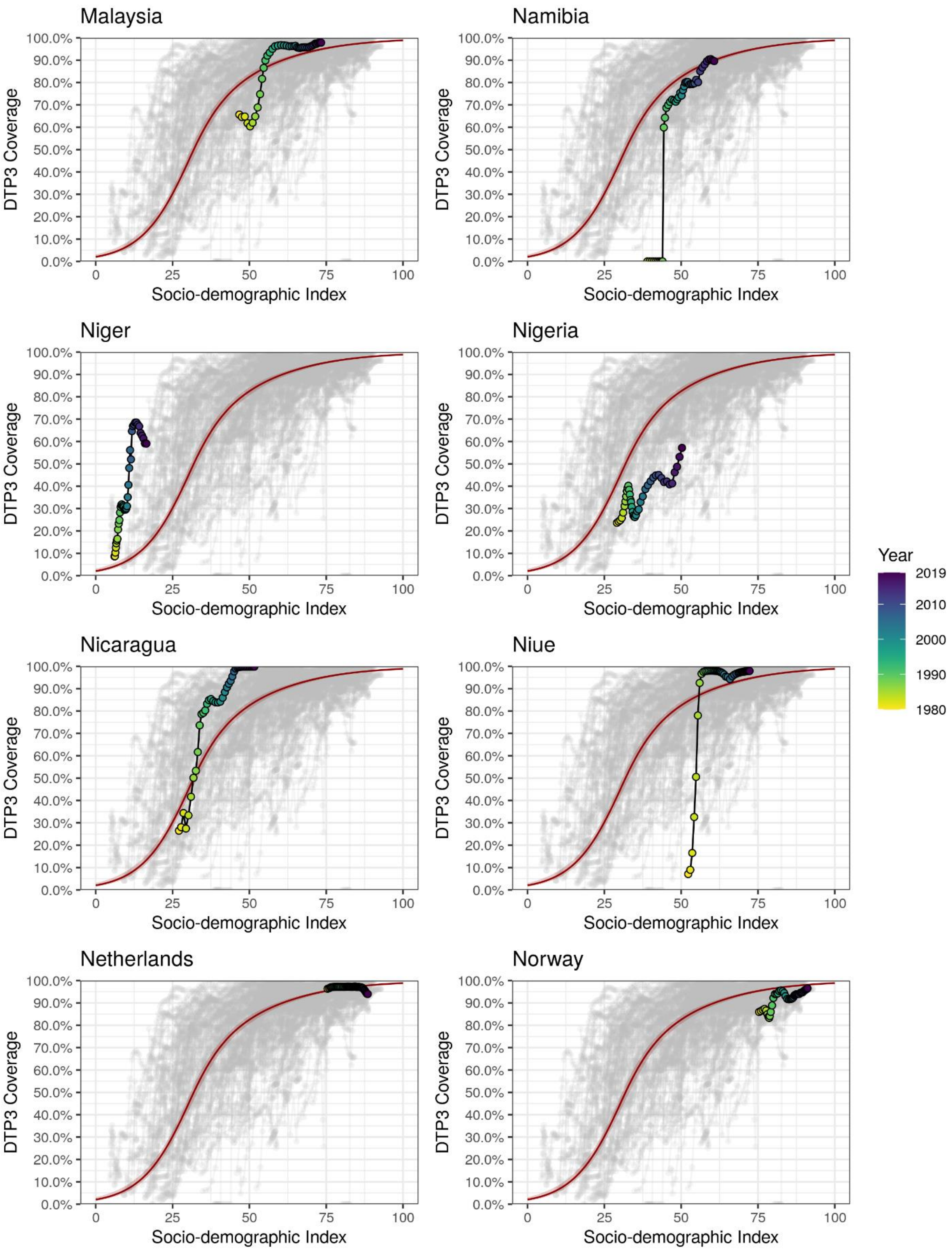
Malaysia
Namibia
Niger
Nigeria
Nicaragua
Niue
Netherlands
Norway
DTP3 Coverage
100.0%
90.0%
80.0%
70.0%
60.0%
50.0%
40.0%
30.0%
20.0%
10.0%
0.0%
Socio-demographic Index
0
25
50
75
100
Year
2019
2010
2000
1990
1980

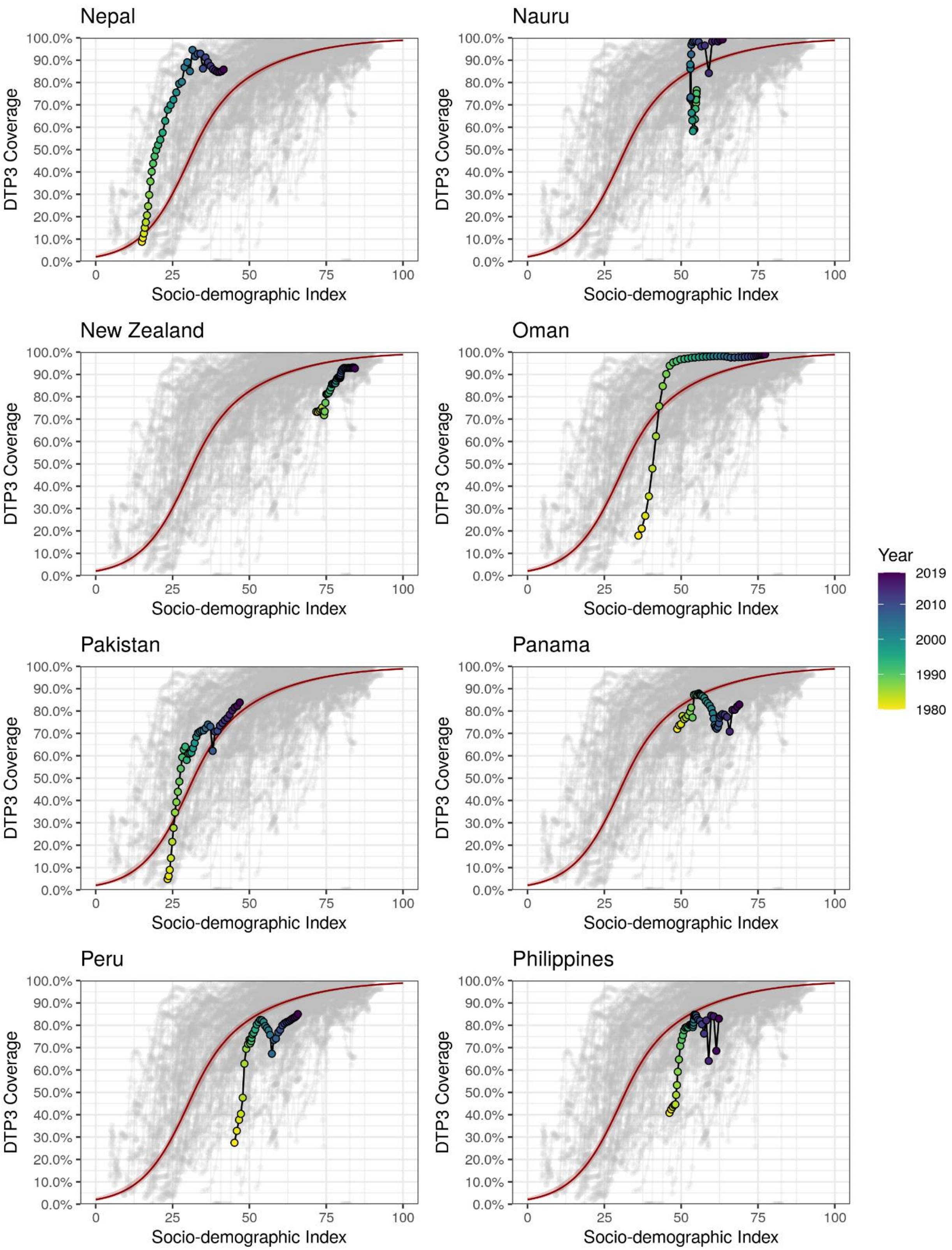
Nepal
Nauru
New Zealand
Oman
Pakistan
Panama
Peru
Philippines
DTP3 Coverage
Socio-demographic Index
0.0%
10.0%
20.0%
30.0%
40.0%
50.0%
60.0%
70.0%
80.0%
90.0%
100.0%
0
25
50
75
100
Year
2019
2010
2000
1990
1980

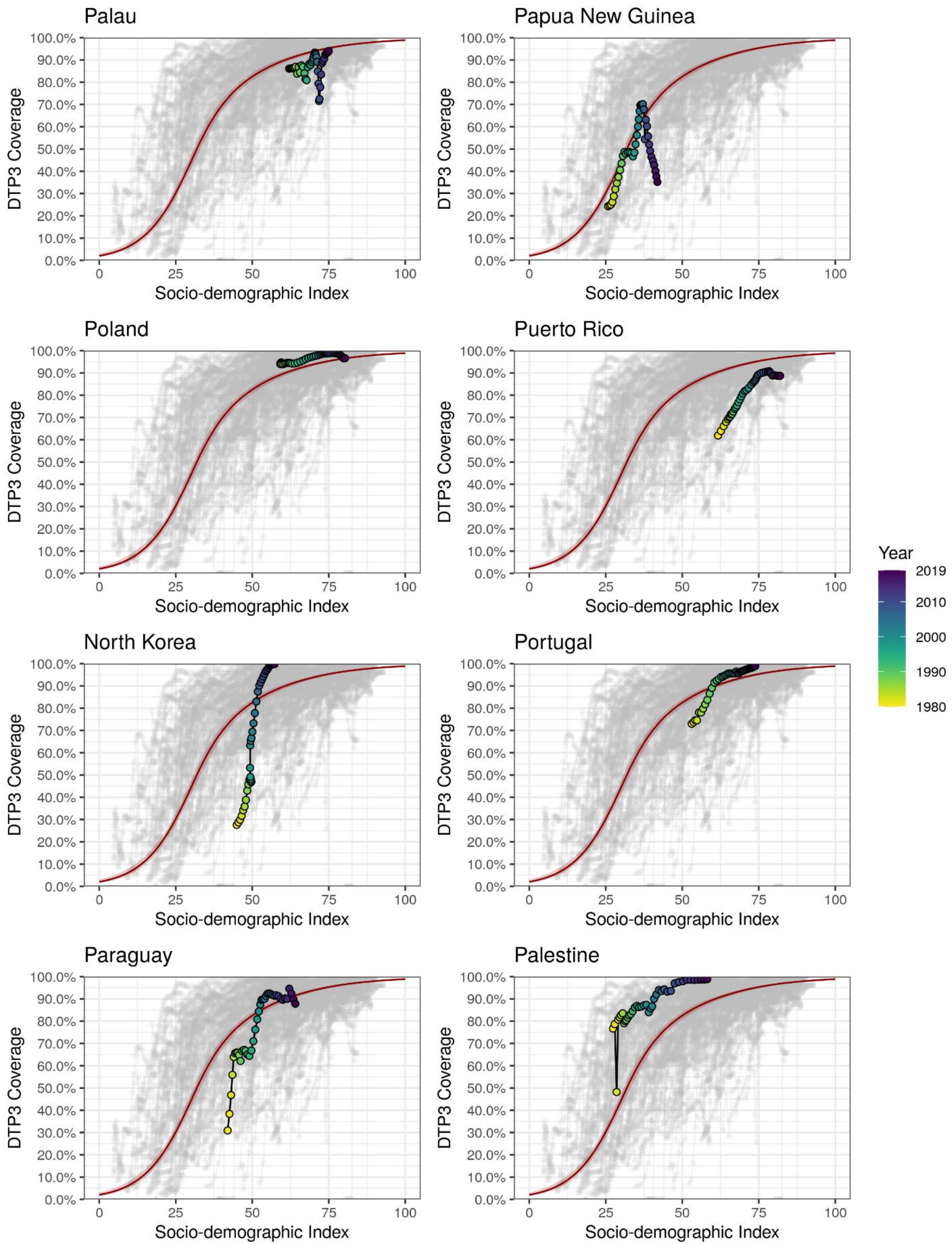
Palau
Papua New Guinea
Poland
Puerto Rico
North Korea
Portugal
Paraguay
Palestine
DTP3 Coverage
Socio-demographic Index
Year
2019
2010
2000
1990
1980

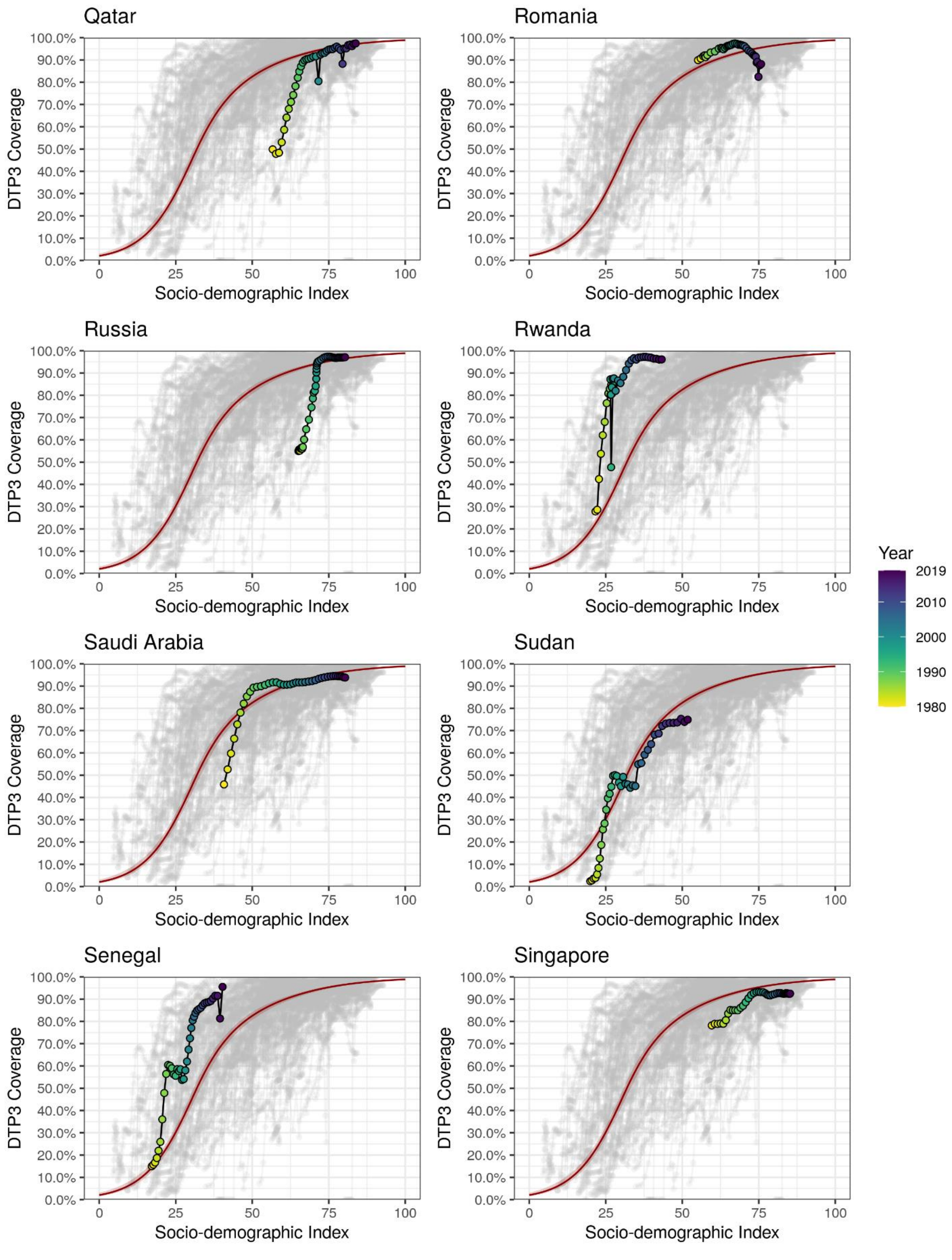
Qatar
Romania
Russia
Rwanda
Saudi Arabia
Sudan
Senegal
Singapore
DTP3 Coverage
Socio-demographic Index
0.0%
10.0%
20.0%
30.0%
40.0%
50.0%
60.0%
70.0%
80.0%
90.0%
100.0%
0
25
50
75
100
Year
2019
2010
2000
1990
1980

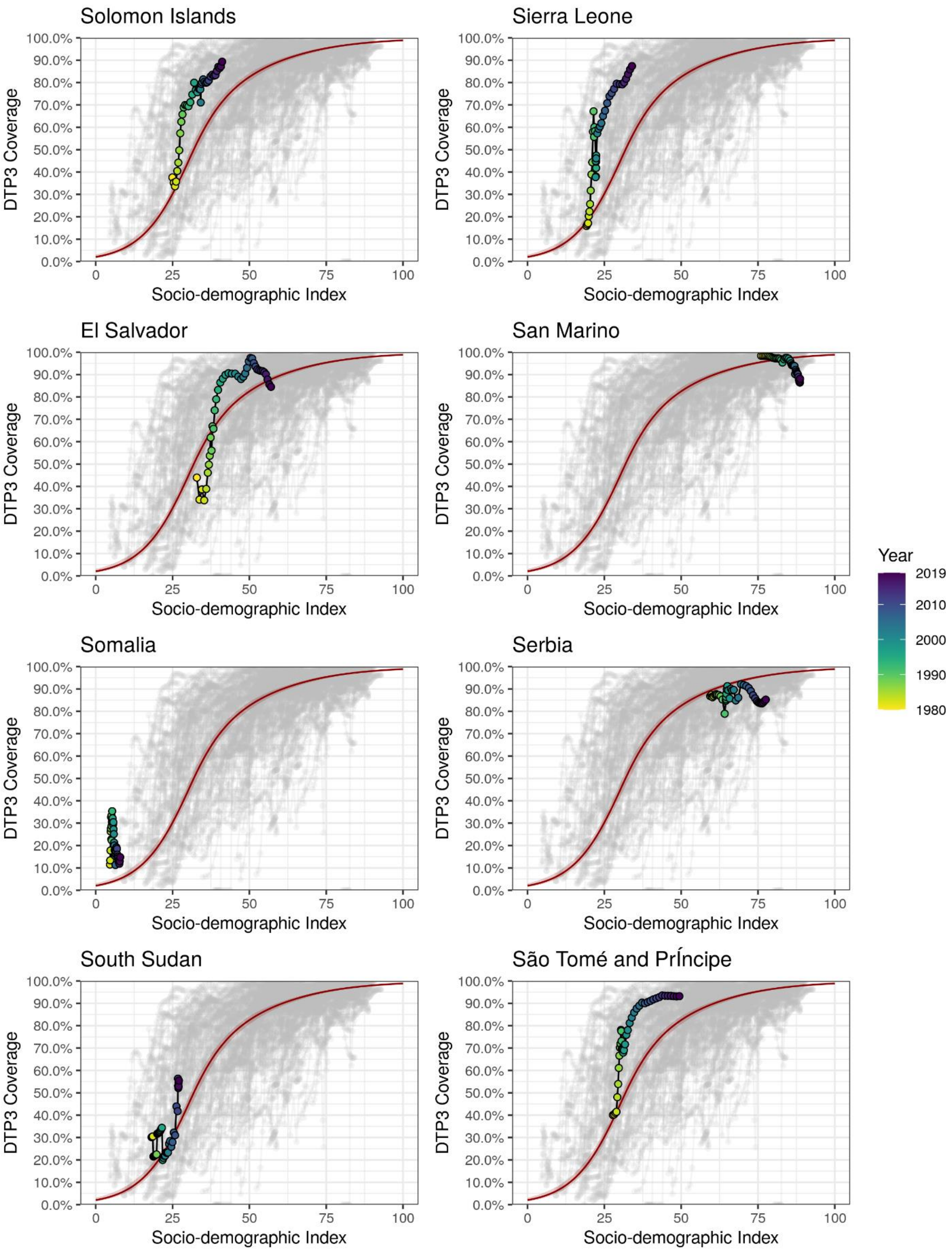

Solomon Islands
Sierra Leone
El Salvador
San Marino
Somalia
Serbia
South Sudan
São Tomé and PrÍncipe
DTP3 Coverage
Socio-demographic Index
0.0%
10.0%
20.0%
30.0%
40.0%
50.0%
60.0%
70.0%
80.0%
90.0%
100.0%
0
25
50
75
100
Year
2019
2010
2000
1990
1980

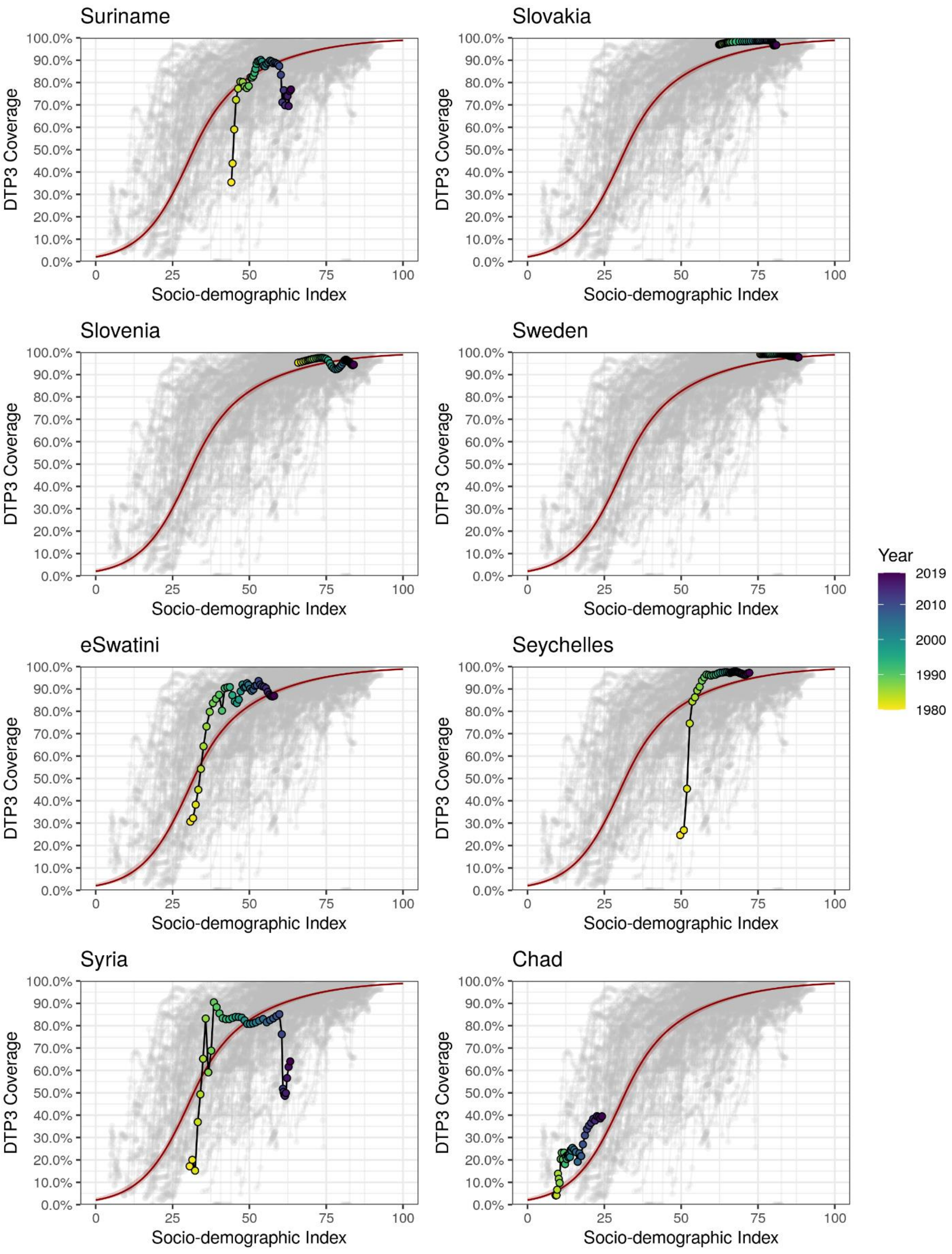
Suriname
Slovakia
Slovenia
Sweden
eSwatini
Seychelles
Syria
Chad
DTP3 Coverage
Socio-demographic Index
0.0%
10.0%
20.0%
30.0%
40.0%
50.0%
60.0%
70.0%
80.0%
90.0%
100.0%
0
25
50
75
100
Year
2019
2010
2000
1990
1980

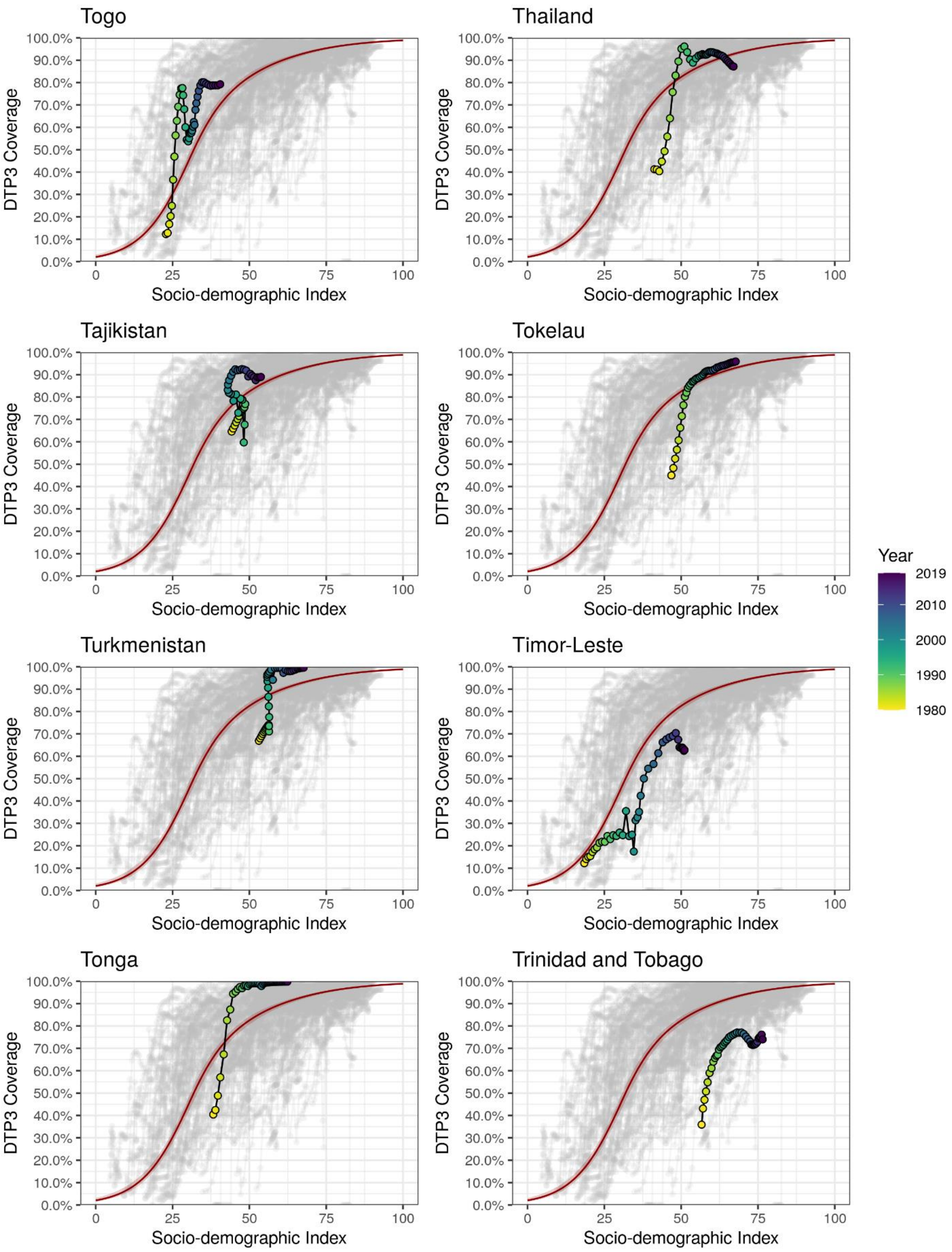
Togo
Thailand
Tajikistan
Tokelau
Turkmenistan
Timor-Leste
Tonga
Trinidad and Tobago
DTP3 Coverage
Socio-demographic Index
0.0%
10.0%
20.0%
30.0%
40.0%
50.0%
60.0%
70.0%
80.0%
90.0%
100.0%
0
25
50
75
100
Year
2019
2010
2000
1990
1980

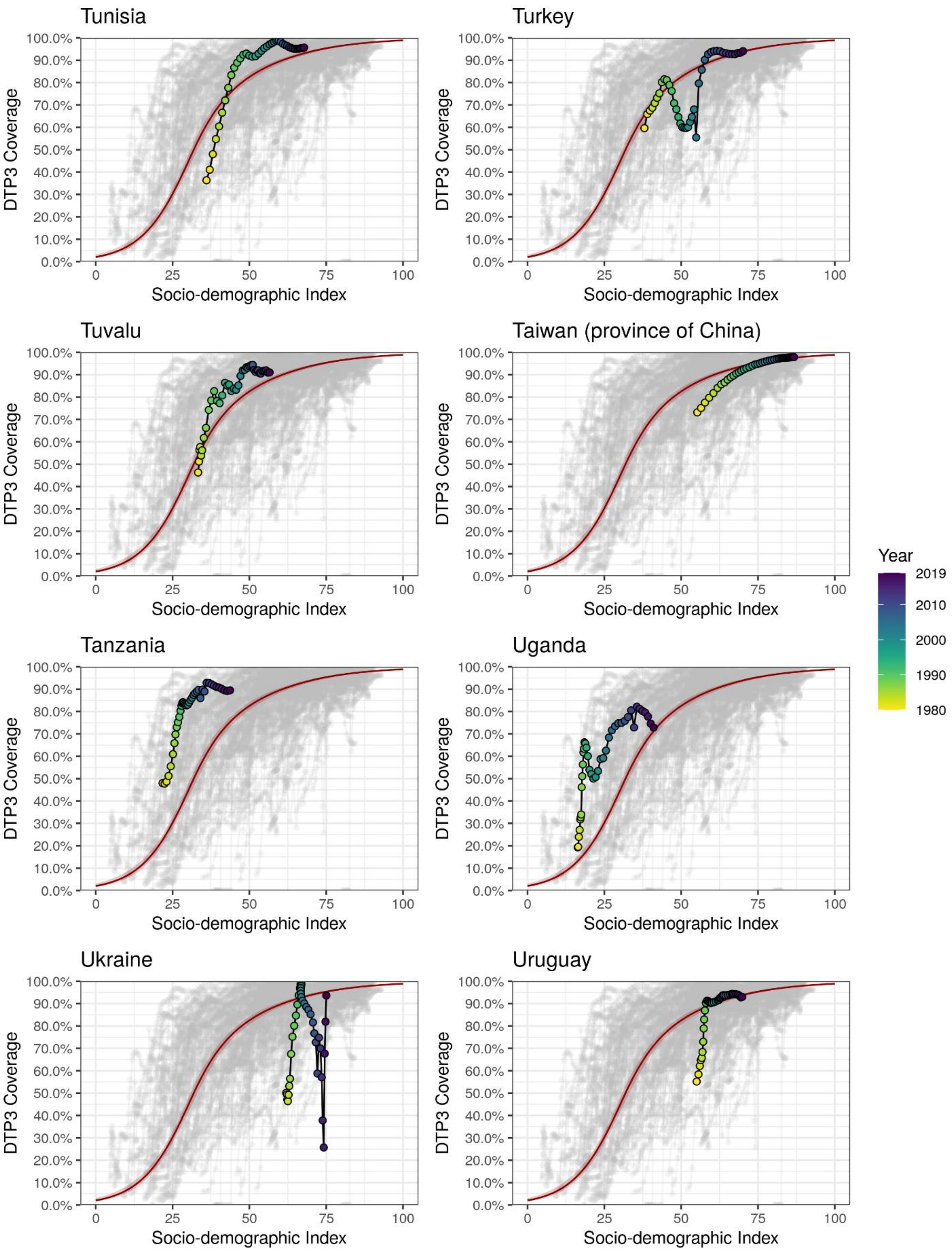
Tunisia
Turkey
Tuvalu
Taiwan (province of China)
Tanzania
Uganda
Ukraine
Uruguay
DTP3 Coverage
Socio-demographic Index
100.0%
90.0%
80.0%
70.0%
60.0%
50.0%
40.0%
30.0%
20.0%
10.0%
0.0%
0
25
50
75
100
Year
2019
2010
2000
1990
1980

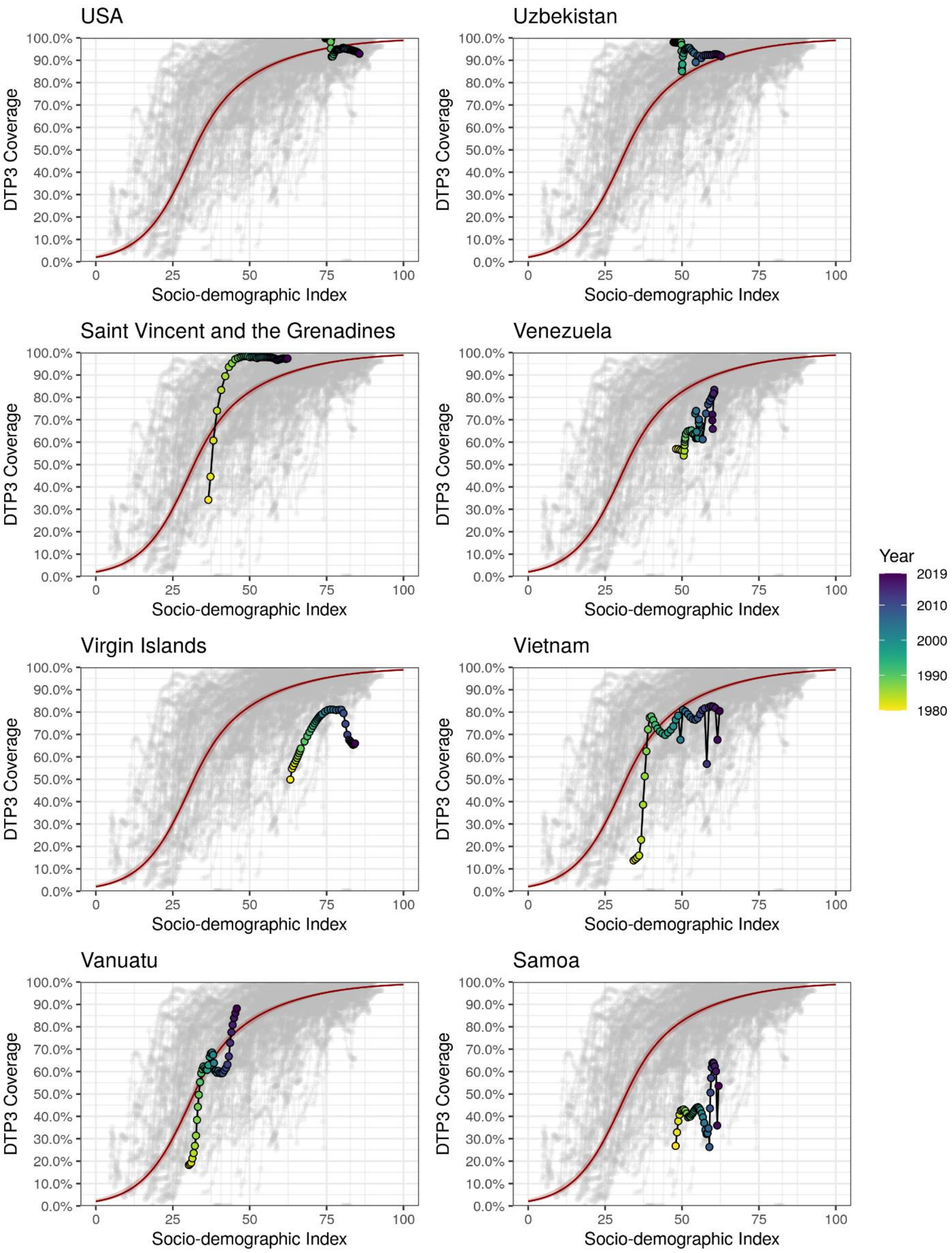
USA
Uzbekistan
Saint Vincent and the Grenadines
Venezuela
Virgin Islands
Vietnam
Vanuatu
Samoa
DTP3 Coverage
Socio-demographic Index
0.0%
10.0%
20.0%
30.0%
40.0%
50.0%
60.0%
70.0%
80.0%
90.0%
100.0%
0
25
50
75
100
Year
2019
2010
2000
1990
1980

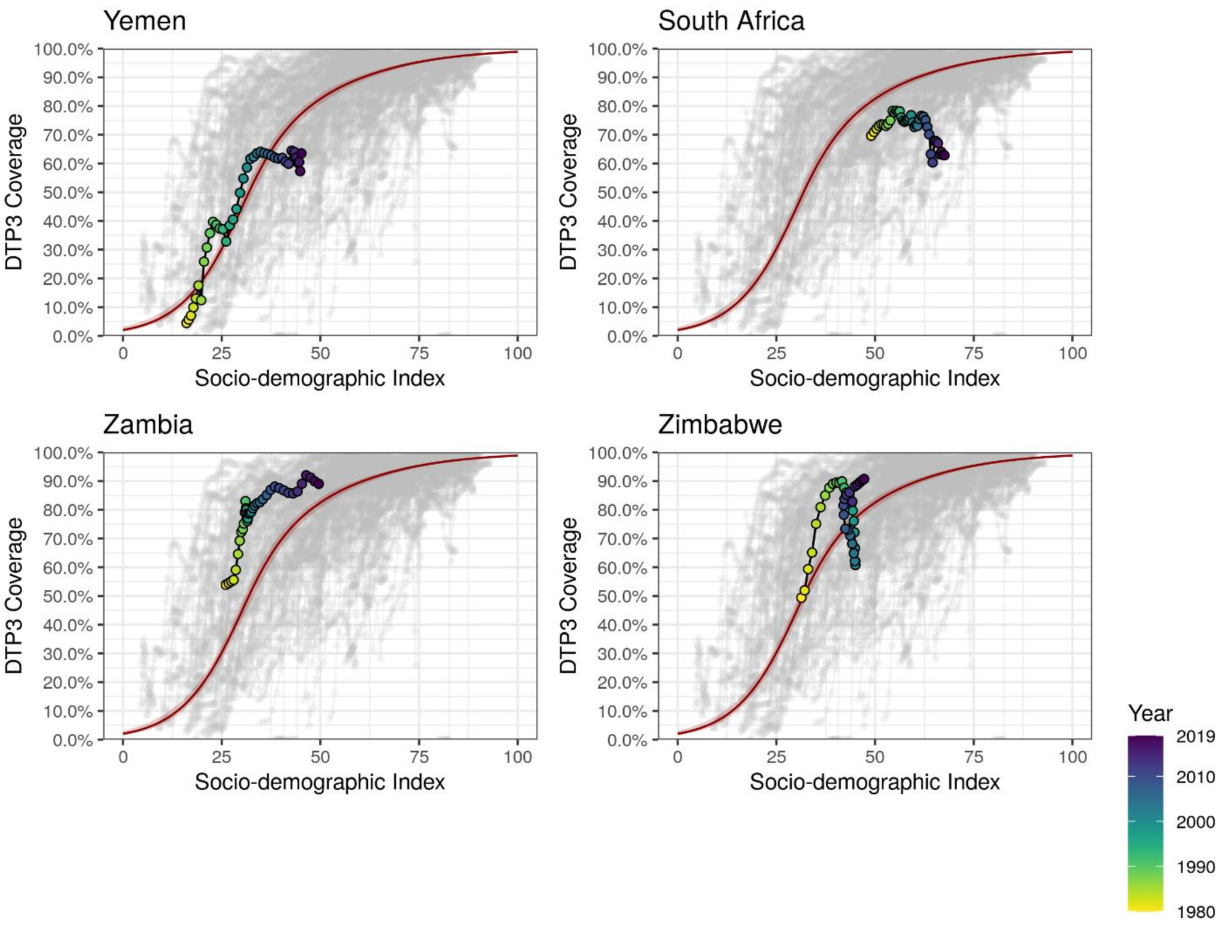
Yemen
South Africa
Zambia
Zimbabwe
DTP3 Coverage
Socio-demographic Index
100.0%
90.0%
80.0%
70.0%
60.0%
50.0%
40.0%
30.0%
20.0%
10.0%
0.0%
0
25
50
75
100
Year
2019
2010
2000
1990
1980

Supplementary figure 16. Observed coverage trend versus expected coverage given SDI, by country, MCV1. Bolded red curve with associated 95% uncertainty represents the mean expected relationship between coverage and SDI from a MR-BRT model. The observed SDI and MCV1 coverage trajectories for each country are shown in light grey. In each location-specific panel, the colour of each point represents the year of estimation, from yellow (1980) to purple (2019), for that country or territory. SDI=Socio-demographic Index. MCV1=measles-containing vaccine, first dose. MR-BRT=meta-regression—Bayesian, regularised, trimmed.

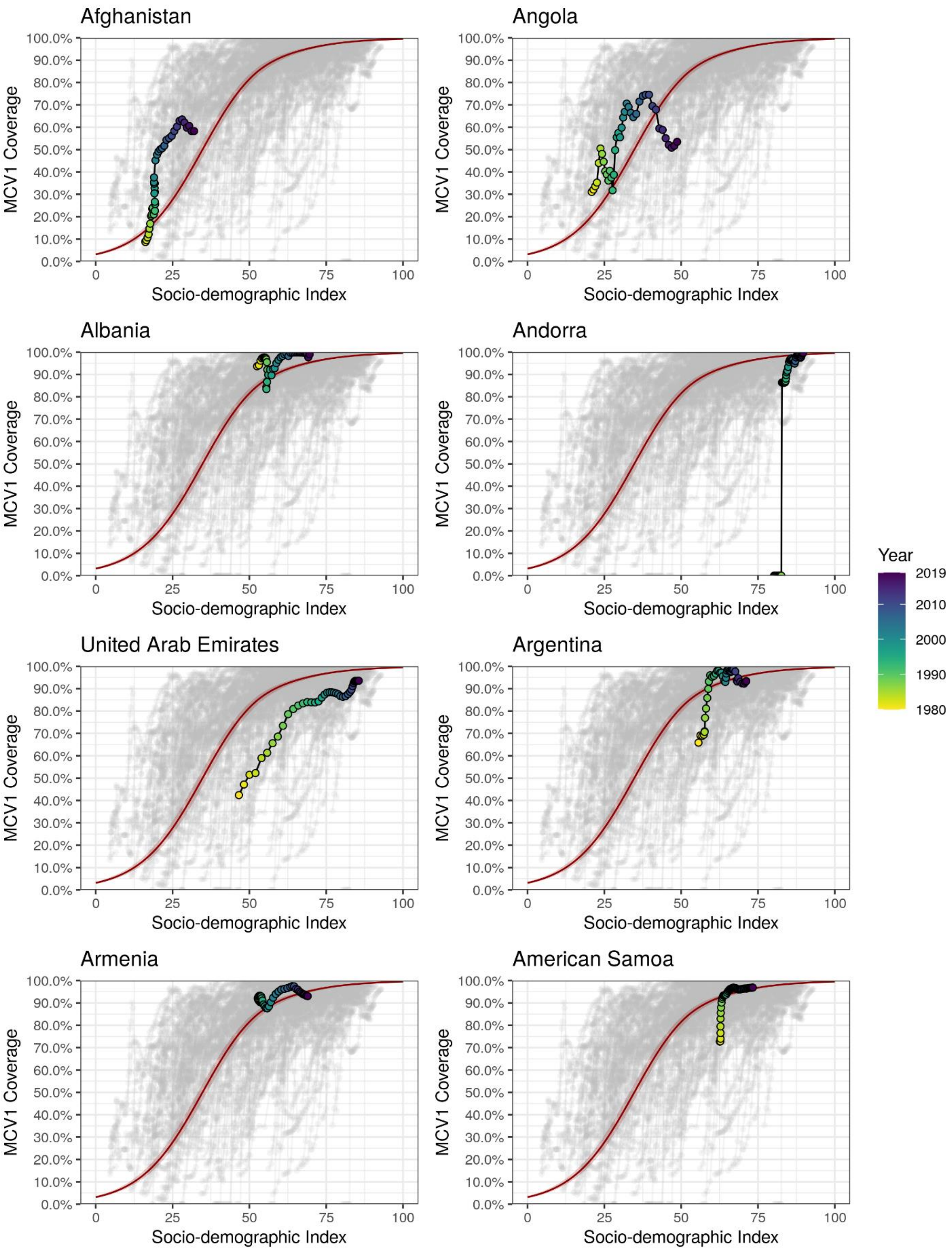
Afghanistan
Angola
Albania
Andorra
United Arab Emirates
Argentina
Armenia
American Samoa
MCV1 Coverage
Socio-demographic Index
100.0%
90.0%
80.0%
70.0%
60.0%
50.0%
40.0%
30.0%
20.0%
10.0%
0.0%
0
25
50
75
100
Year
2019
2010
2000
1990
1980

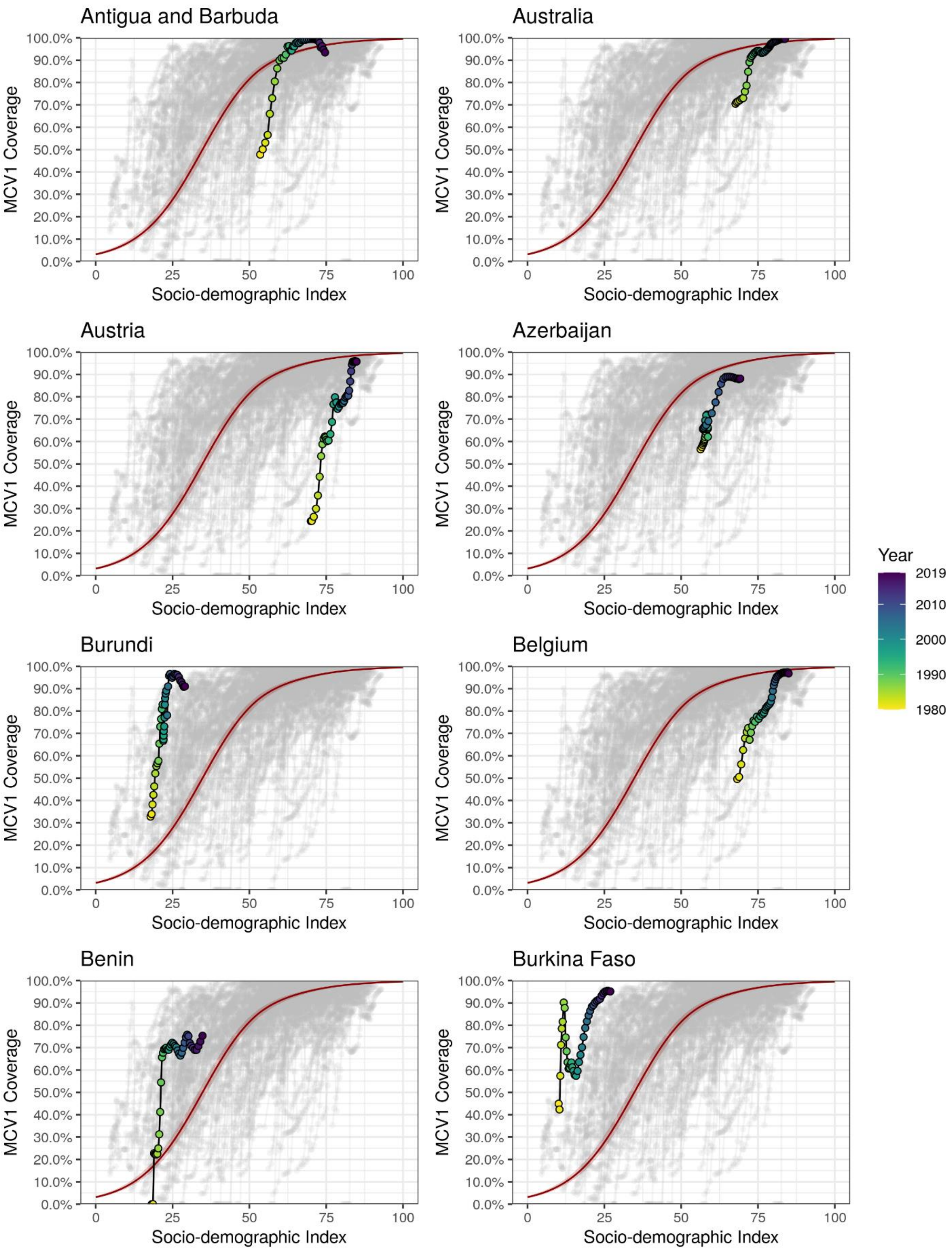
Antigua and Barbuda
Australia
Austria
Azerbaijan
Burundi
Belgium
Benin
Burkina Faso
MCV1 Coverage
Socio-demographic Index
Year
2019
2010
2000
1990
1980

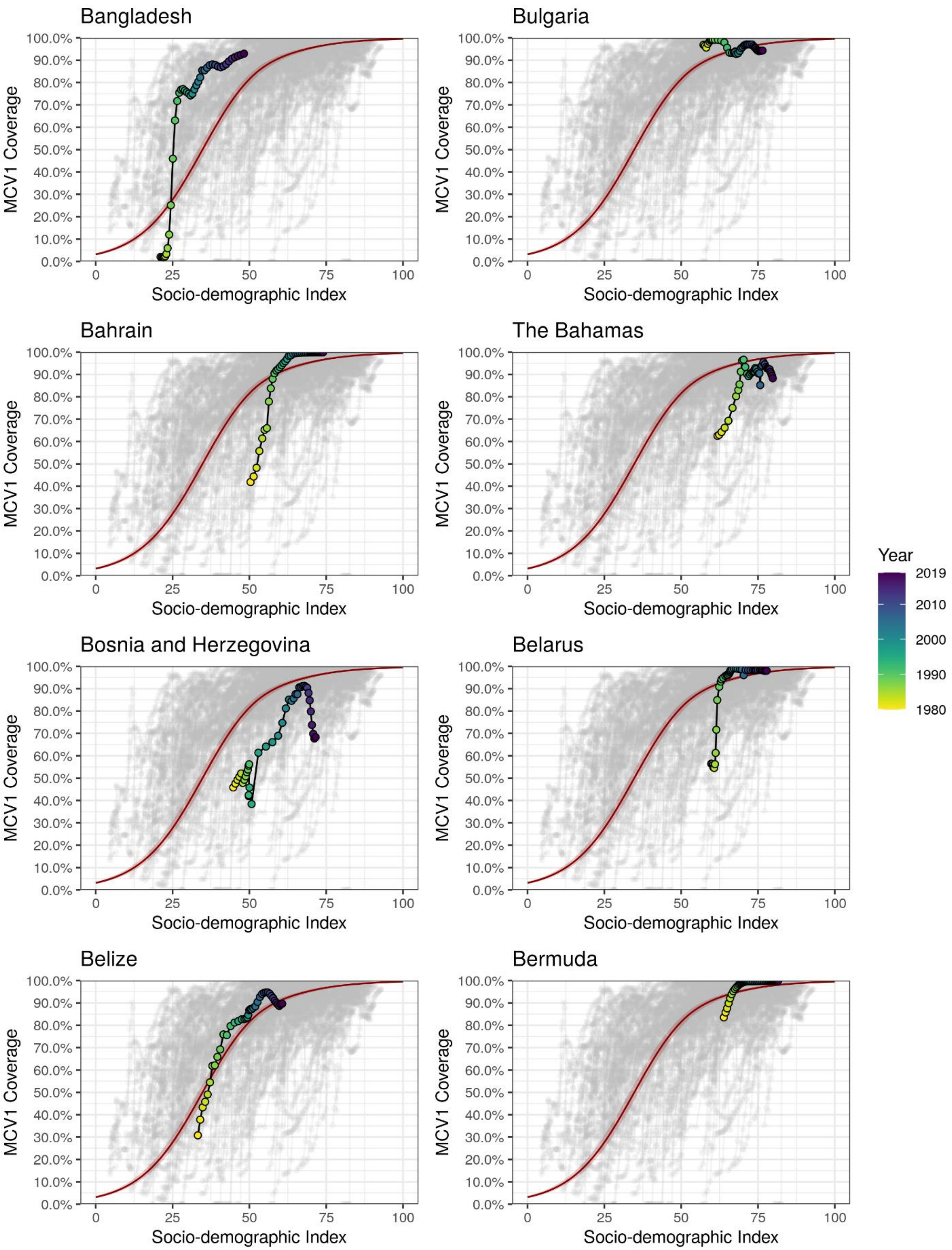
Bangladesh
Bulgaria
Bahrain
The Bahamas
Bosnia and Herzegovina
Belarus
Belize
Bermuda
MCV1 Coverage
0.0%
10.0%
20.0%
30.0%
40.0%
50.0%
60.0%
70.0%
80.0%
90.0%
100.0%
Socio-demographic Index
0
25
50
75
100
Year
2019
2010
2000
1990
1980

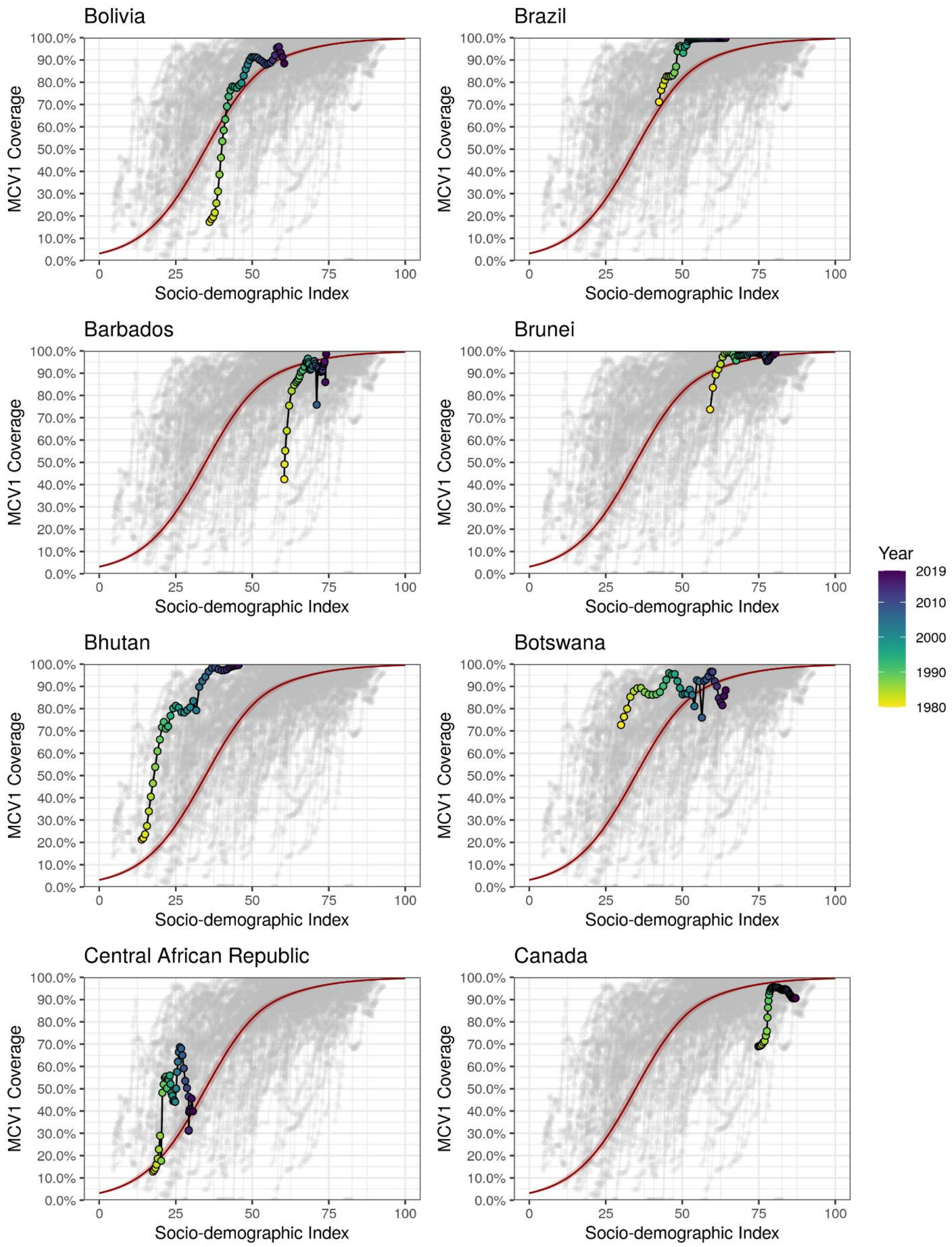
Bolivia
Brazil
Barbados
Brunei
Bhutan
Botswana
Central African Republic
Canada
MCV1 Coverage
Socio-demographic Index
0.0%
10.0%
20.0%
30.0%
40.0%
50.0%
60.0%
70.0%
80.0%
90.0%
100.0%
0
25
50
75
100
Year
2019
2010
2000
1990
1980

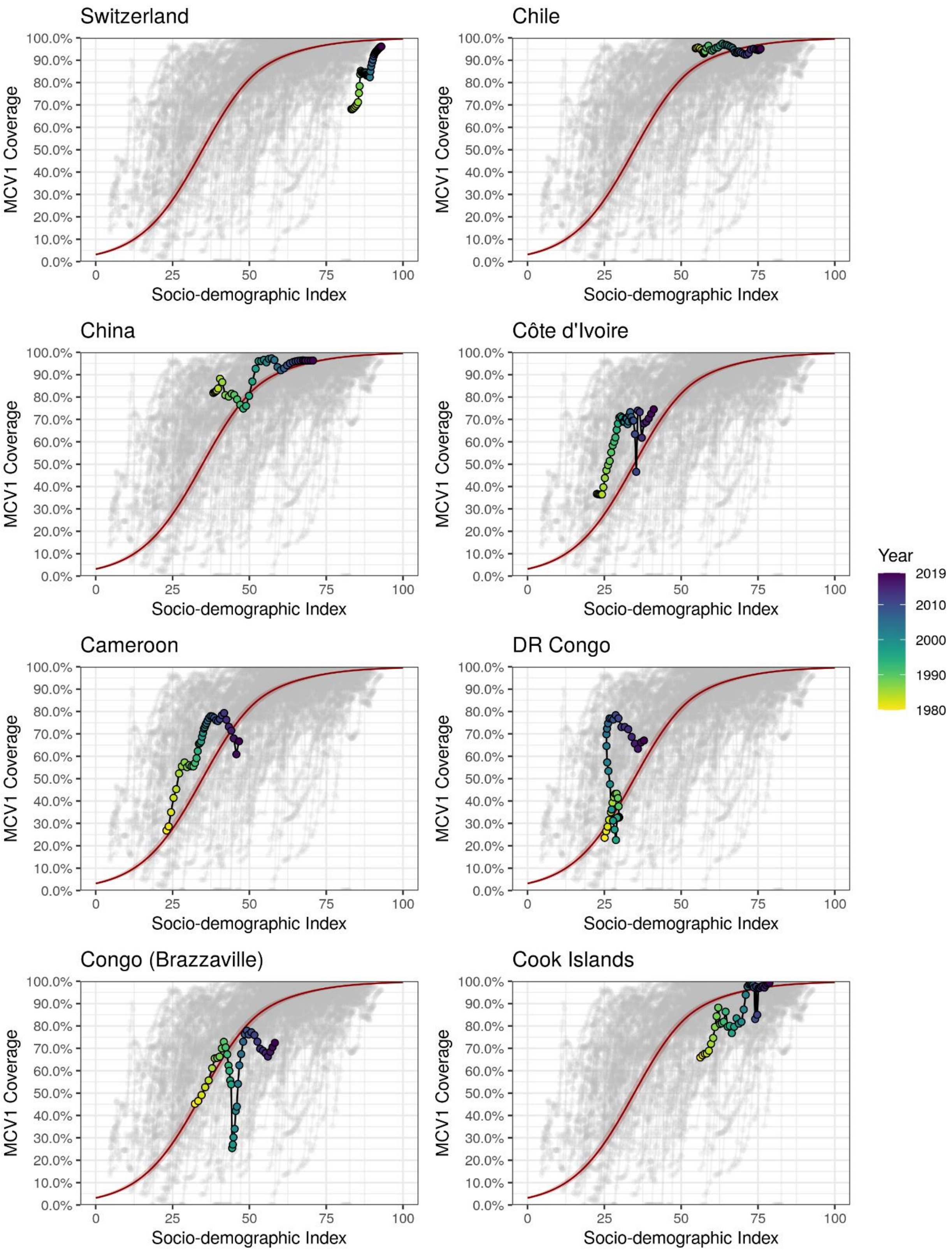
Switzerland
Chile
China
Côte d'Ivoire
Cameroon
DR Congo
Congo (Brazzaville)
Cook Islands
MCV1 Coverage
Socio-demographic Index
0.0%
10.0%
20.0%
30.0%
40.0%
50.0%
60.0%
70.0%
80.0%
90.0%
100.0%
0
25
50
75
100
Year
2019
2010
2000
1990
1980

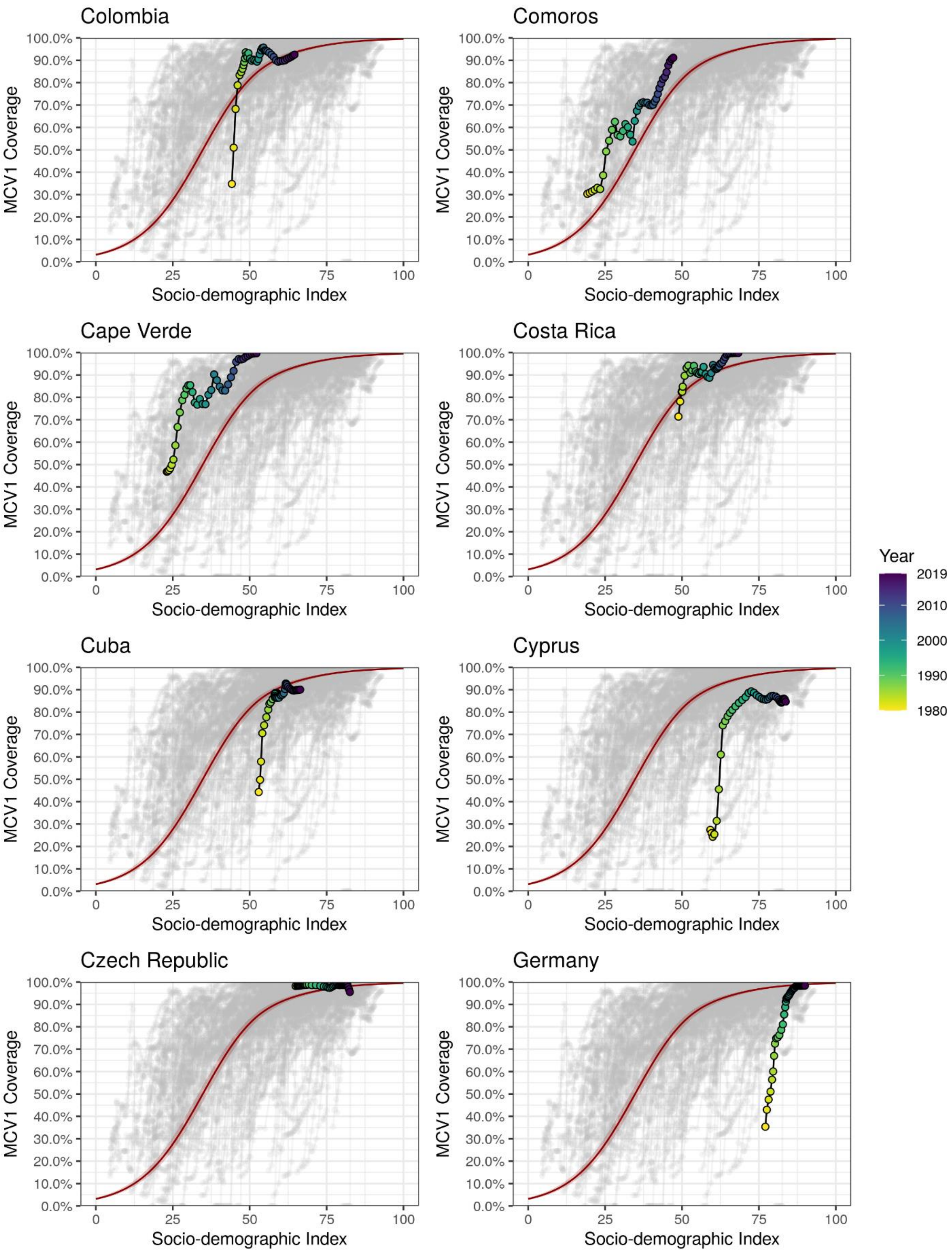

Colombia
Comoros
Cape Verde
Costa Rica
Cuba
Cyprus
Czech Republic
Germany
MCV1 Coverage
100.0%
90.0%
80.0%
70.0%
60.0%
50.0%
40.0%
30.0%
20.0%
10.0%
0.0%
0
25
50
75
100
Socio-demographic Index
Year
2019
2010
2000
1990
1980

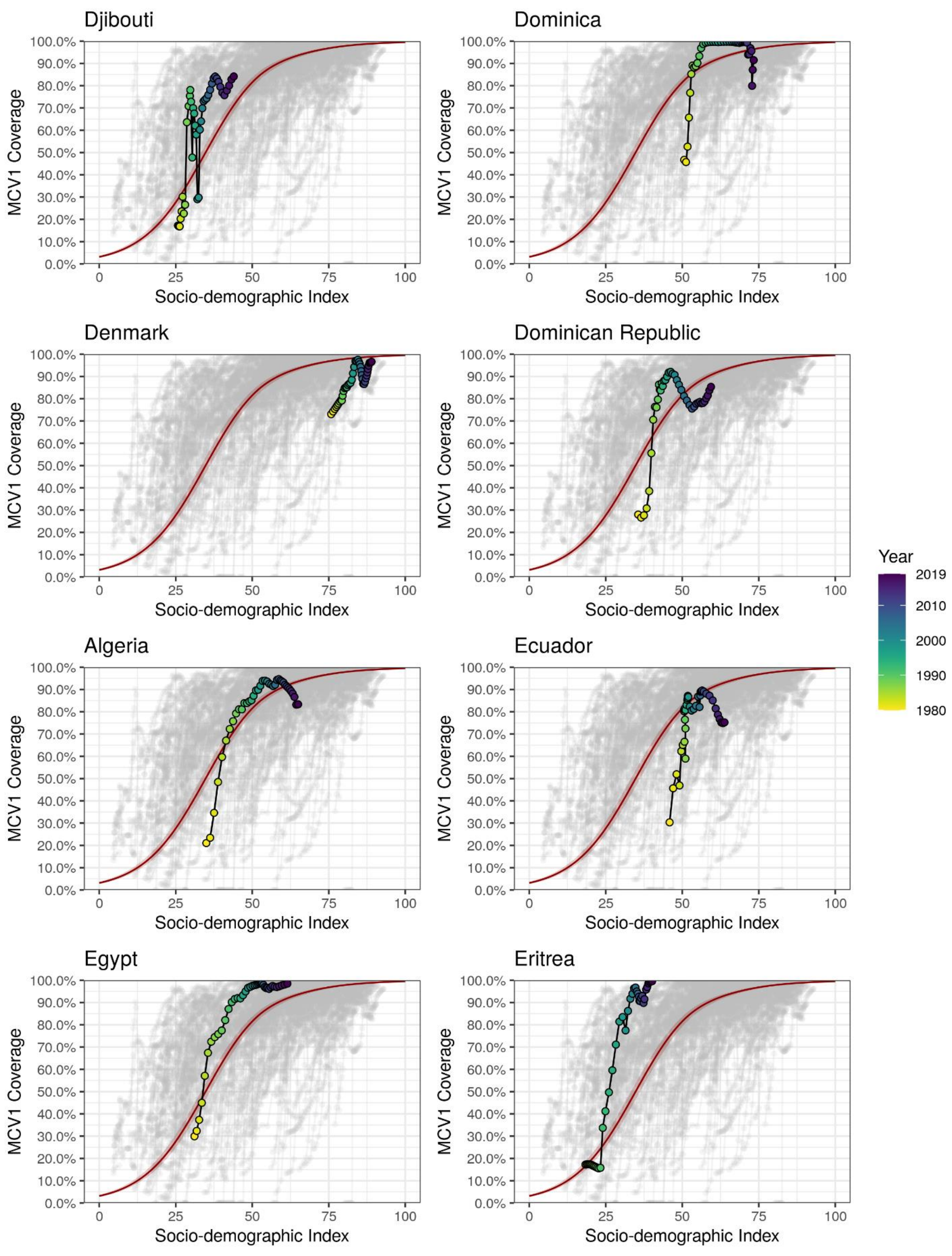
Djibouti
Dominica
Denmark
Dominican Republic
Algeria
Ecuador
Egypt
Eritrea
MCV1 Coverage
Socio-demographic Index
100.0%
90.0%
80.0%
70.0%
60.0%
50.0%
40.0%
30.0%
20.0%
10.0%
0.0%
0
25
50
75
100
Year
2019
2010
2000
1990
1980

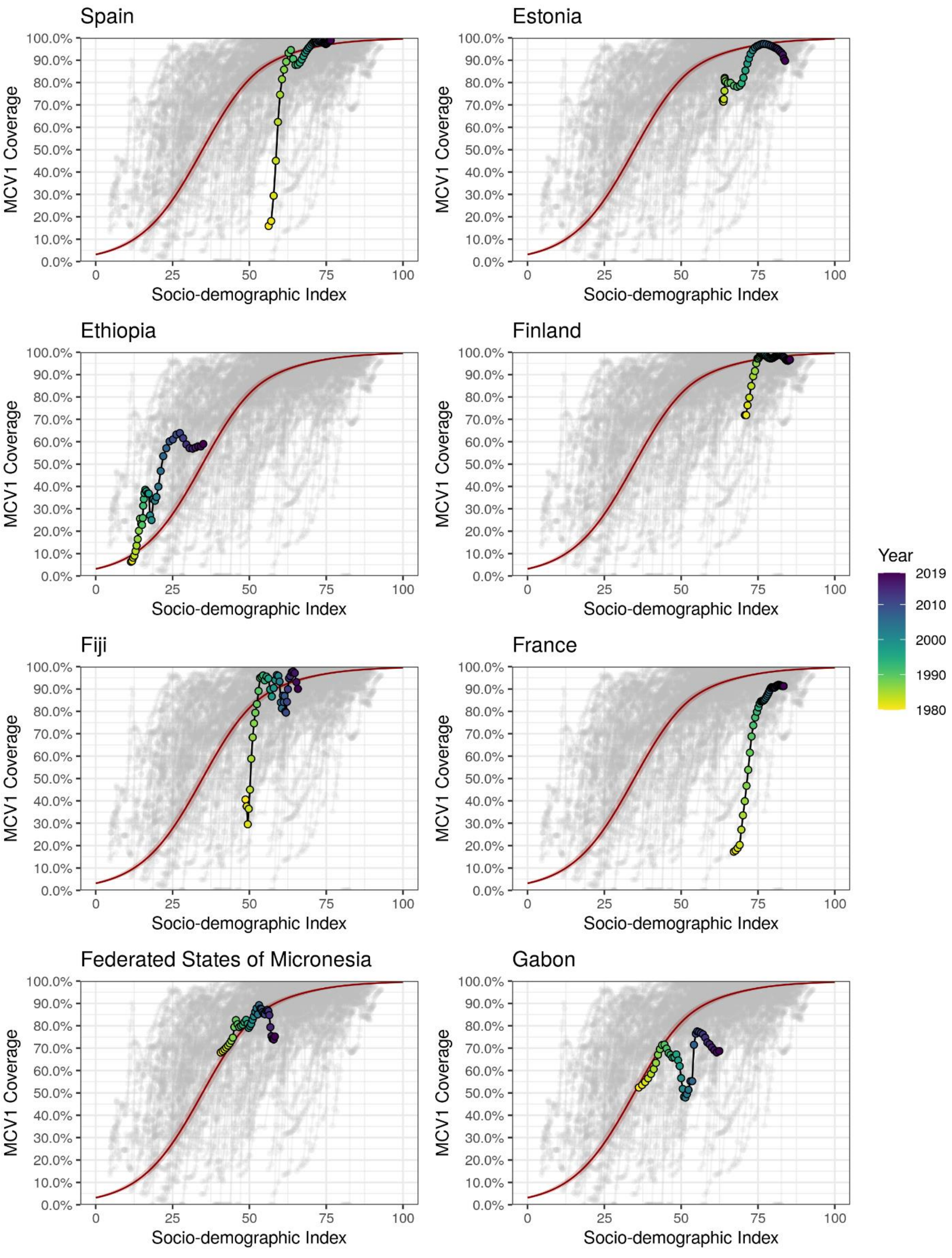
Spain
Estonia
Ethiopia
Finland
Fiji
France
Federated States of Micronesia
Gabon
MCV1 Coverage
100.0%
90.0%
80.0%
70.0%
60.0%
50.0%
40.0%
30.0%
20.0%
10.0%
0.0%
0
25
50
75
100
Socio-demographic Index
Year
2019
2010
2000
1990
1980

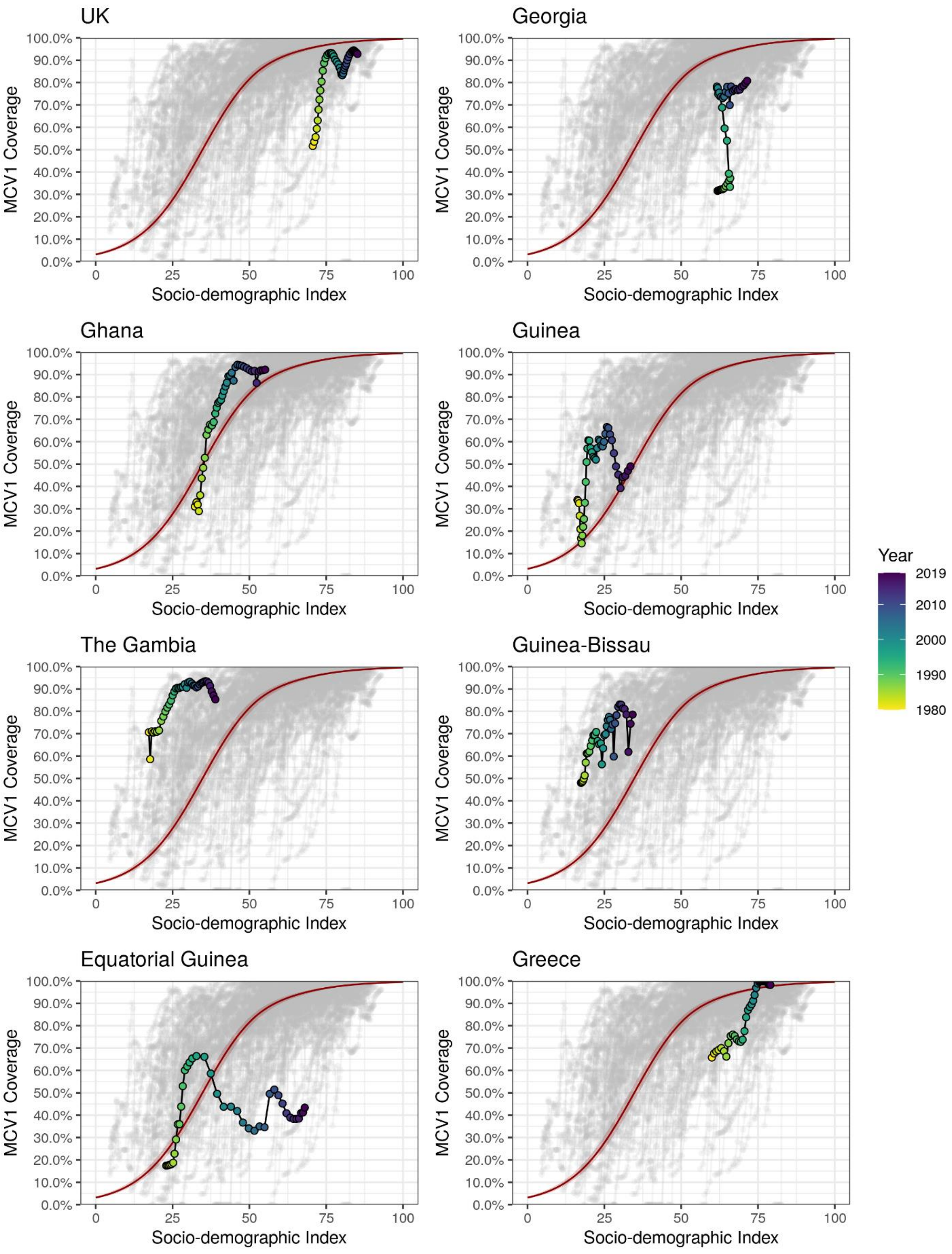
UK
Georgia
Ghana
Guinea
The Gambia
Guinea-Bissau
Equatorial Guinea
Greece
MCV1 Coverage
Socio-demographic Index
0.0%
10.0%
20.0%
30.0%
40.0%
50.0%
60.0%
70.0%
80.0%
90.0%
100.0%
0
25
50
75
100
Year
2019
2010
2000
1990
1980

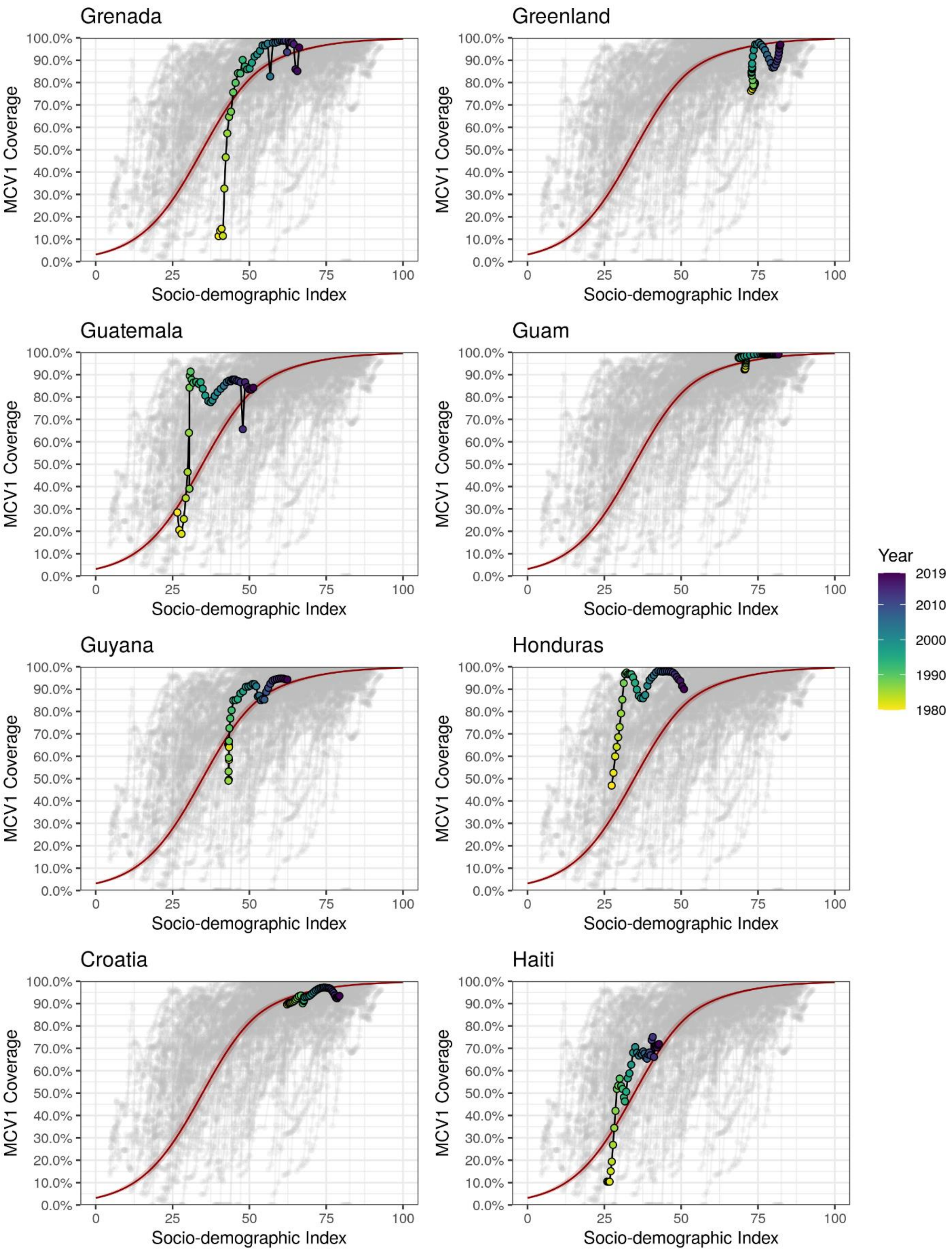
Grenada
Greenland
Guatemala
Guam
Guyana
Honduras
Croatia
Haiti
MCV1 Coverage
Socio-demographic Index
0.0%
10.0%
20.0%
30.0%
40.0%
50.0%
60.0%
70.0%
80.0%
90.0%
100.0%
0
25
50
75
100
Year
2019
2010
2000
1990
1980

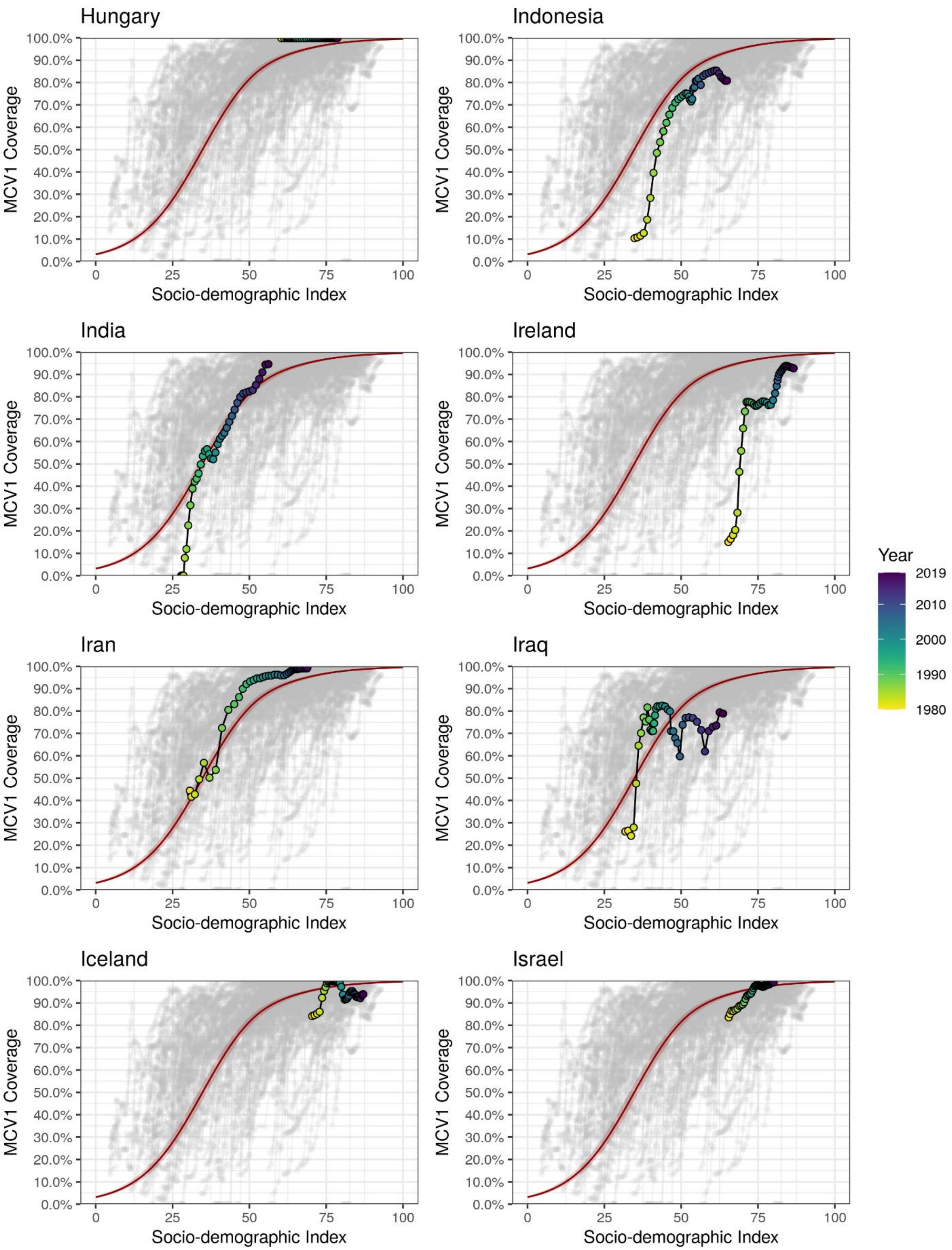
Hungary
Indonesia
India
Ireland
Iran
Iraq
Iceland
Israel
MCV1 Coverage
Socio-demographic Index
0.0%
10.0%
20.0%
30.0%
40.0%
50.0%
60.0%
70.0%
80.0%
90.0%
100.0%
0
25
50
75
100
Year
2019
2010
2000
1990
1980

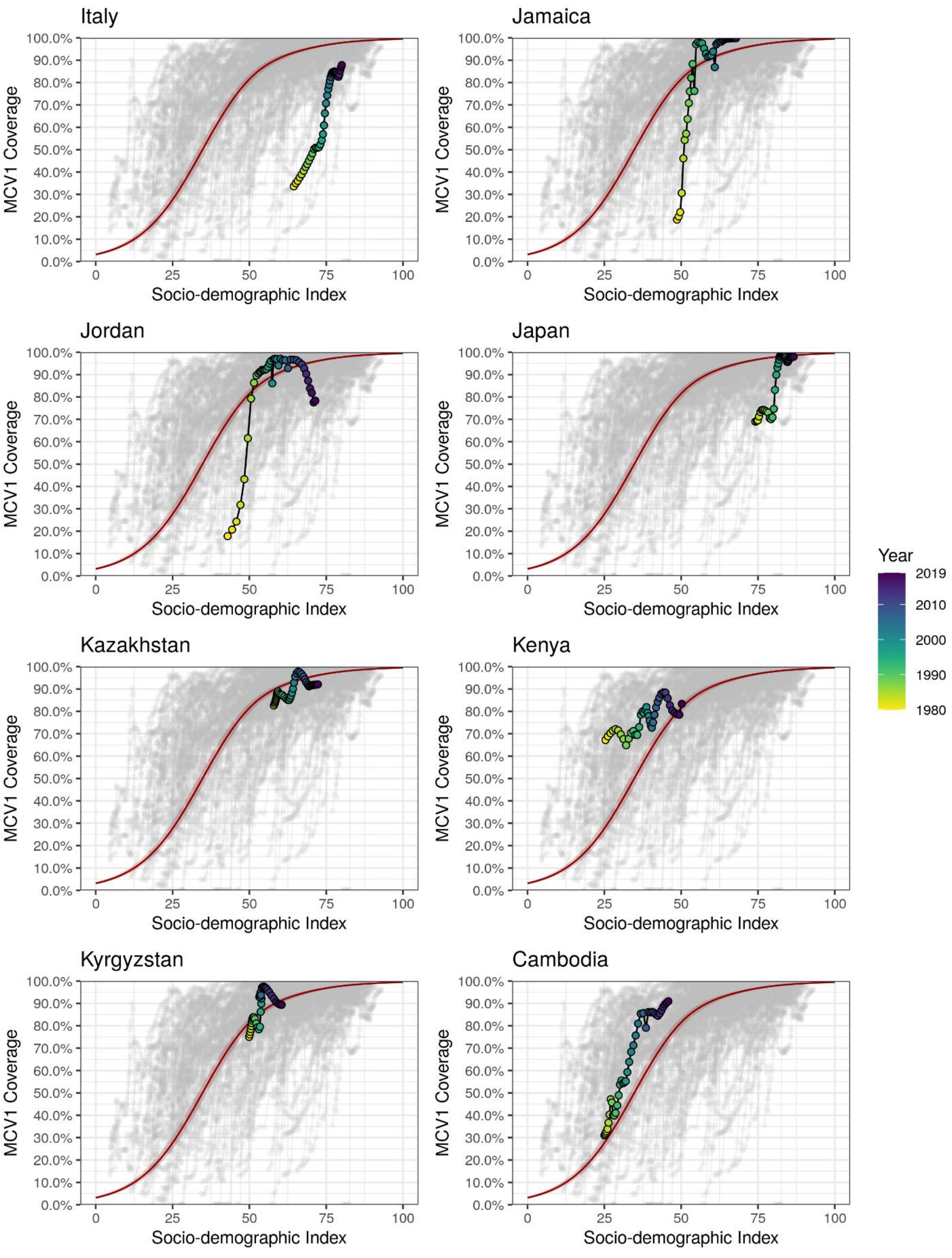
Italy
Jamaica
Jordan
Japan
Kazakhstan
Kenya
Kyrgyzstan
Cambodia
MCV1 Coverage
Socio-demographic Index
100.0%
90.0%
80.0%
70.0%
60.0%
50.0%
40.0%
30.0%
20.0%
10.0%
0.0%
0
25
50
75
100
Year
2019
2010
2000
1990
1980

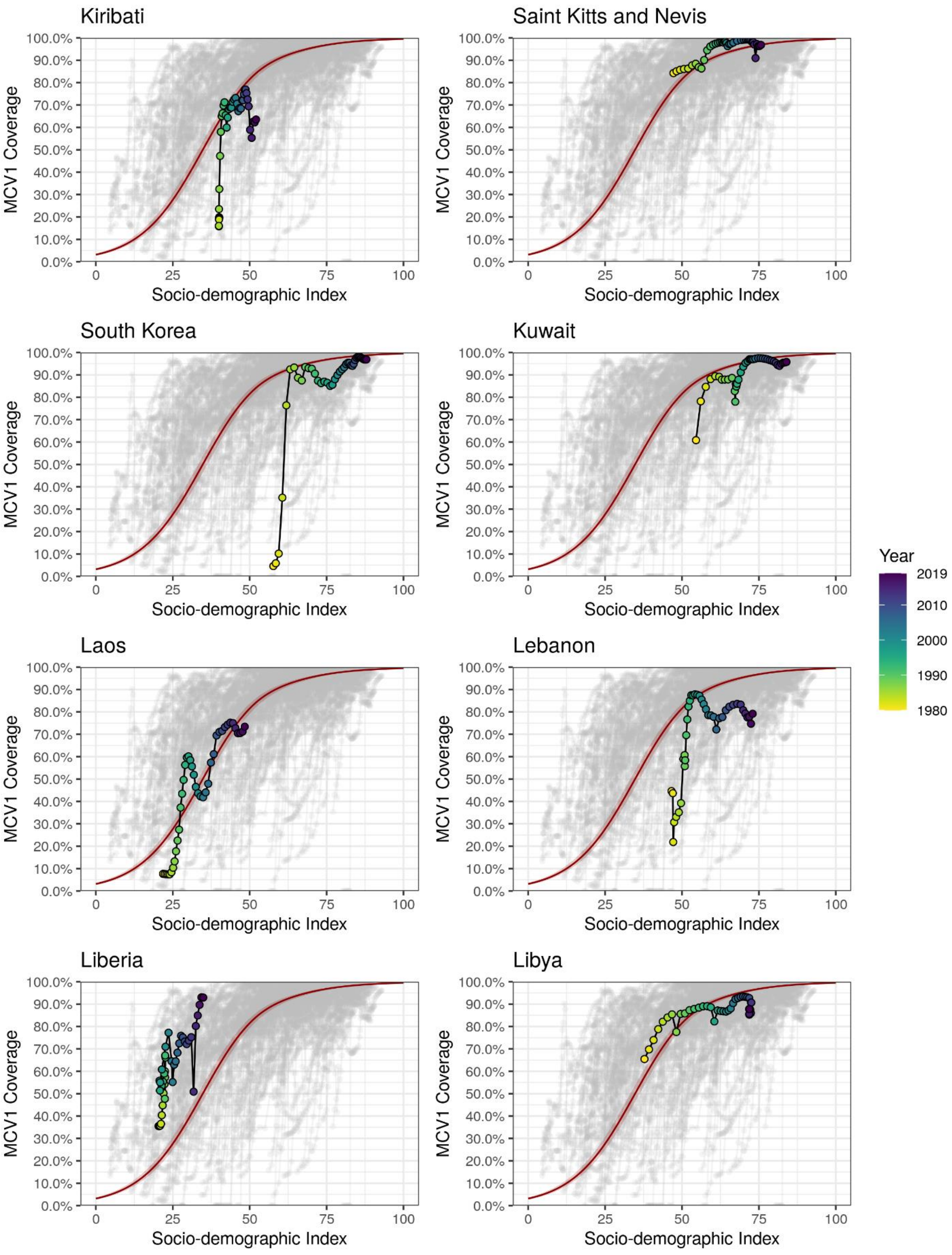
Kiribati
Saint Kitts and Nevis
South Korea
Kuwait
Laos
Lebanon
Liberia
Libya
MCV1 Coverage
Socio-demographic Index
0.0%
10.0%
20.0%
30.0%
40.0%
50.0%
60.0%
70.0%
80.0%
90.0%
100.0%
0
25
50
75
100
Year
2019
2010
2000
1990
1980

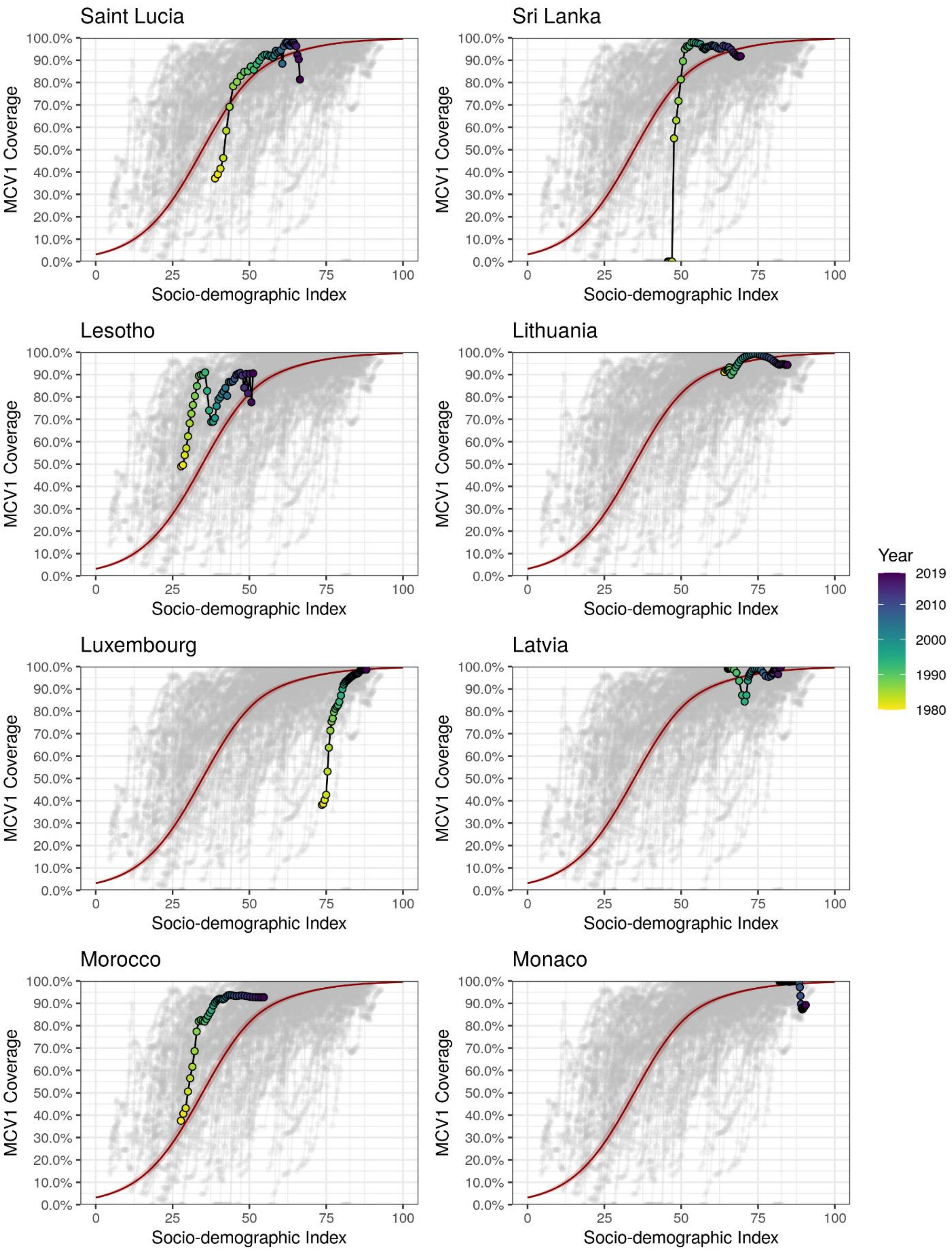

Saint Lucia
Sri Lanka
Lesotho
Lithuania
Luxembourg
Latvia
Morocco
Monaco
MCV1 Coverage
Socio-demographic Index
0.0%
10.0%
20.0%
30.0%
40.0%
50.0%
60.0%
70.0%
80.0%
90.0%
100.0%
0
25
50
75
100
Year
2019
2010
2000
1990
1980

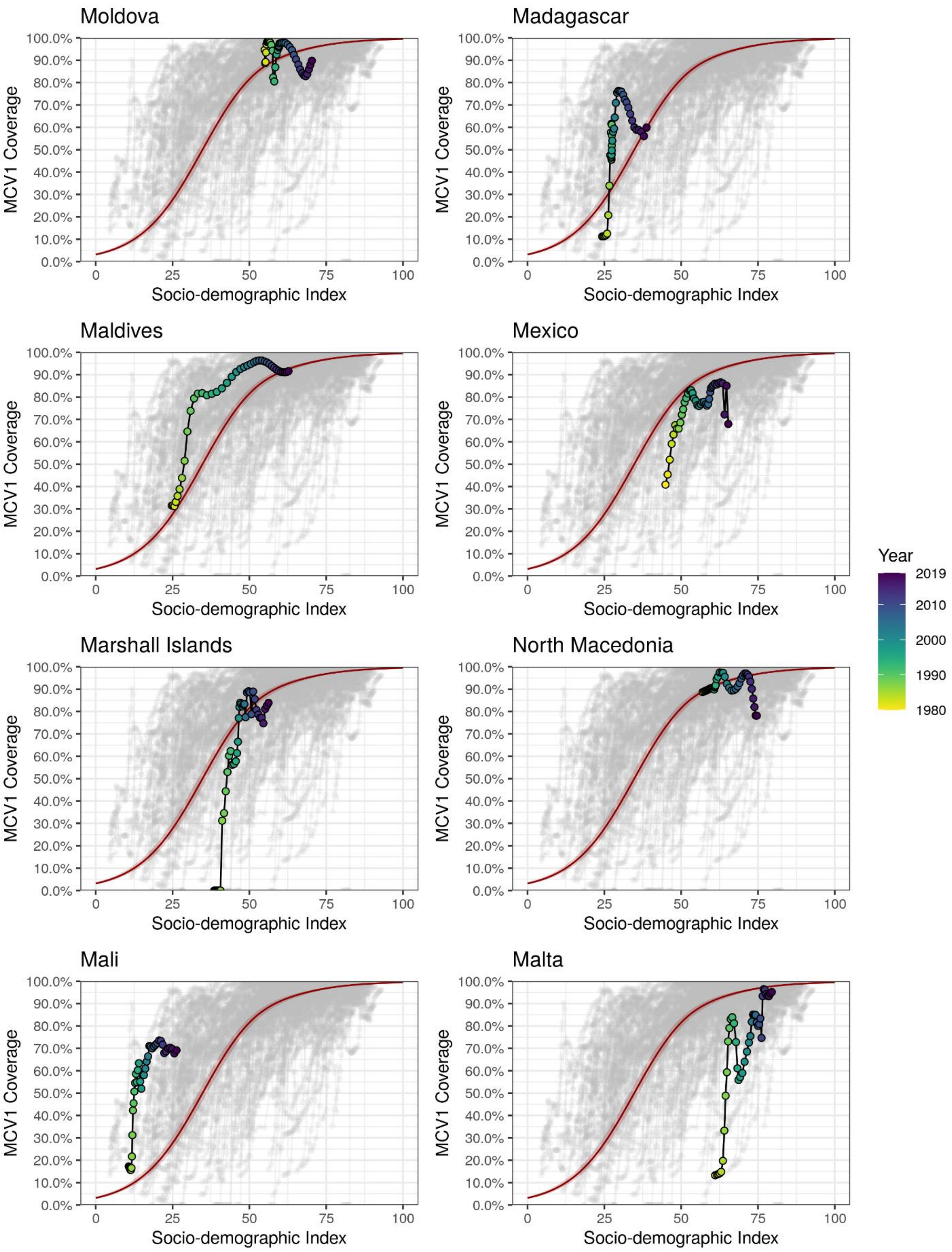
Moldova
Madagascar
Maldives
Mexico
Marshall Islands
North Macedonia
Mali
Malta
MCV1 Coverage
Socio-demographic Index
0.0%
10.0%
20.0%
30.0%
40.0%
50.0%
60.0%
70.0%
80.0%
90.0%
100.0%
0
25
50
75
100
Year
2019
2010
2000
1990
1980

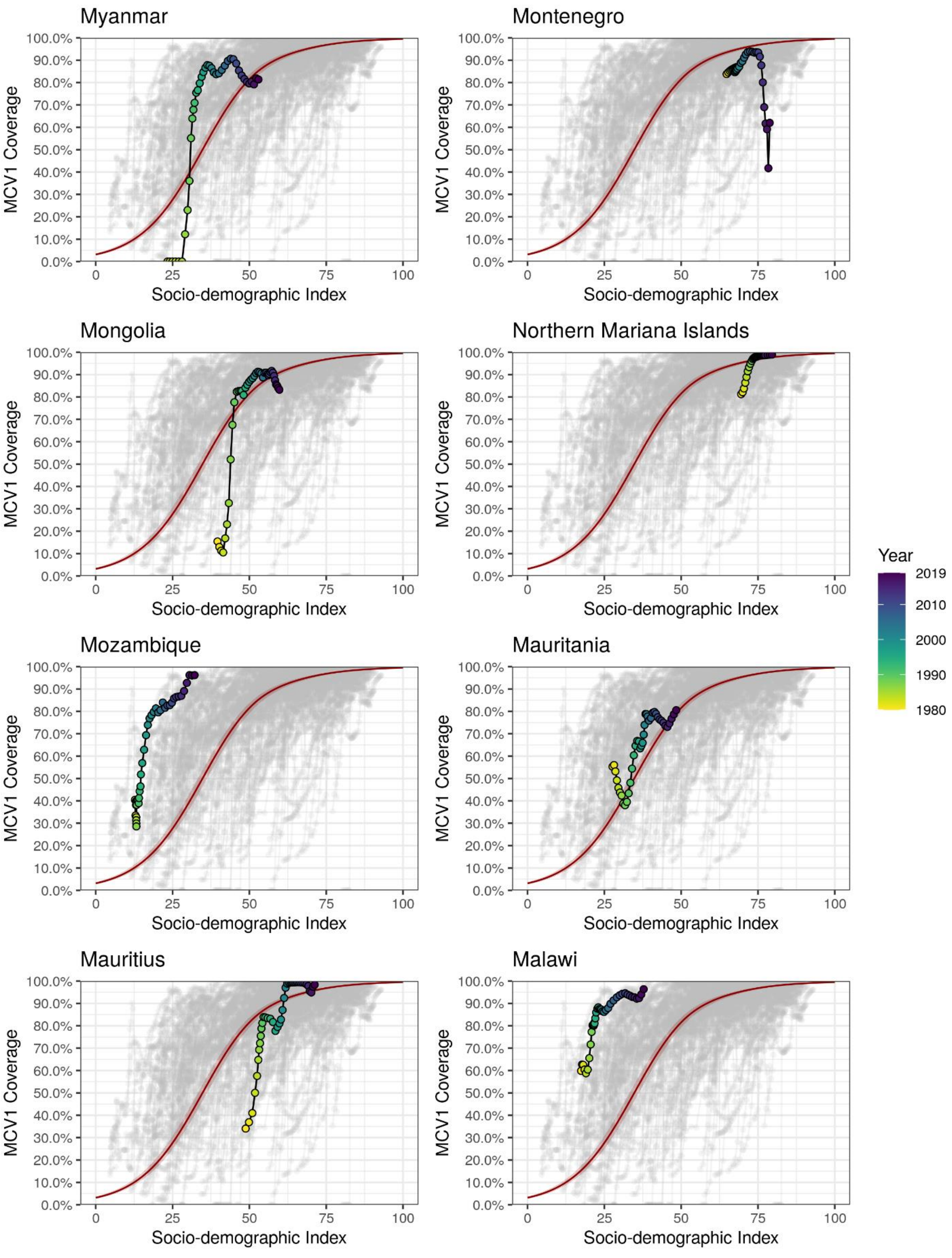
Myanmar
Montenegro
Mongolia
Northern Mariana Islands
Mozambique
Mauritania
Mauritius
Malawi
MCV1 Coverage
Socio-demographic Index
0.0%
10.0%
20.0%
30.0%
40.0%
50.0%
60.0%
70.0%
80.0%
90.0%
100.0%
0
25
50
75
100
Year
2019
2010
2000
1990
1980

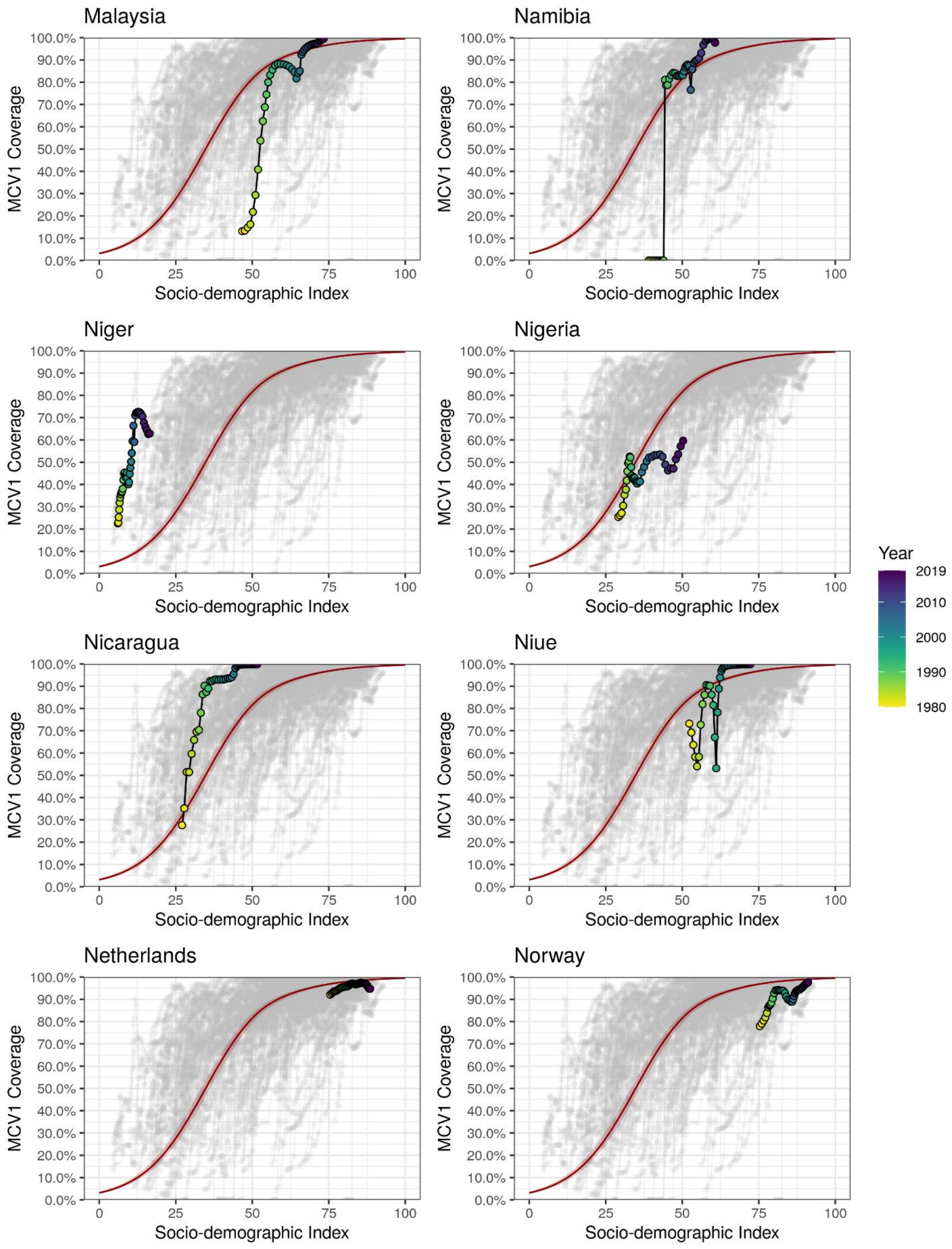
Malaysia
Namibia
Niger
Nigeria
Nicaragua
Niue
Netherlands
Norway
MCV1 Coverage
Socio-demographic Index
0.0%
10.0%
20.0%
30.0%
40.0%
50.0%
60.0%
70.0%
80.0%
90.0%
100.0%
0
25
50
75
100
Year
2019
2010
2000
1990
1980

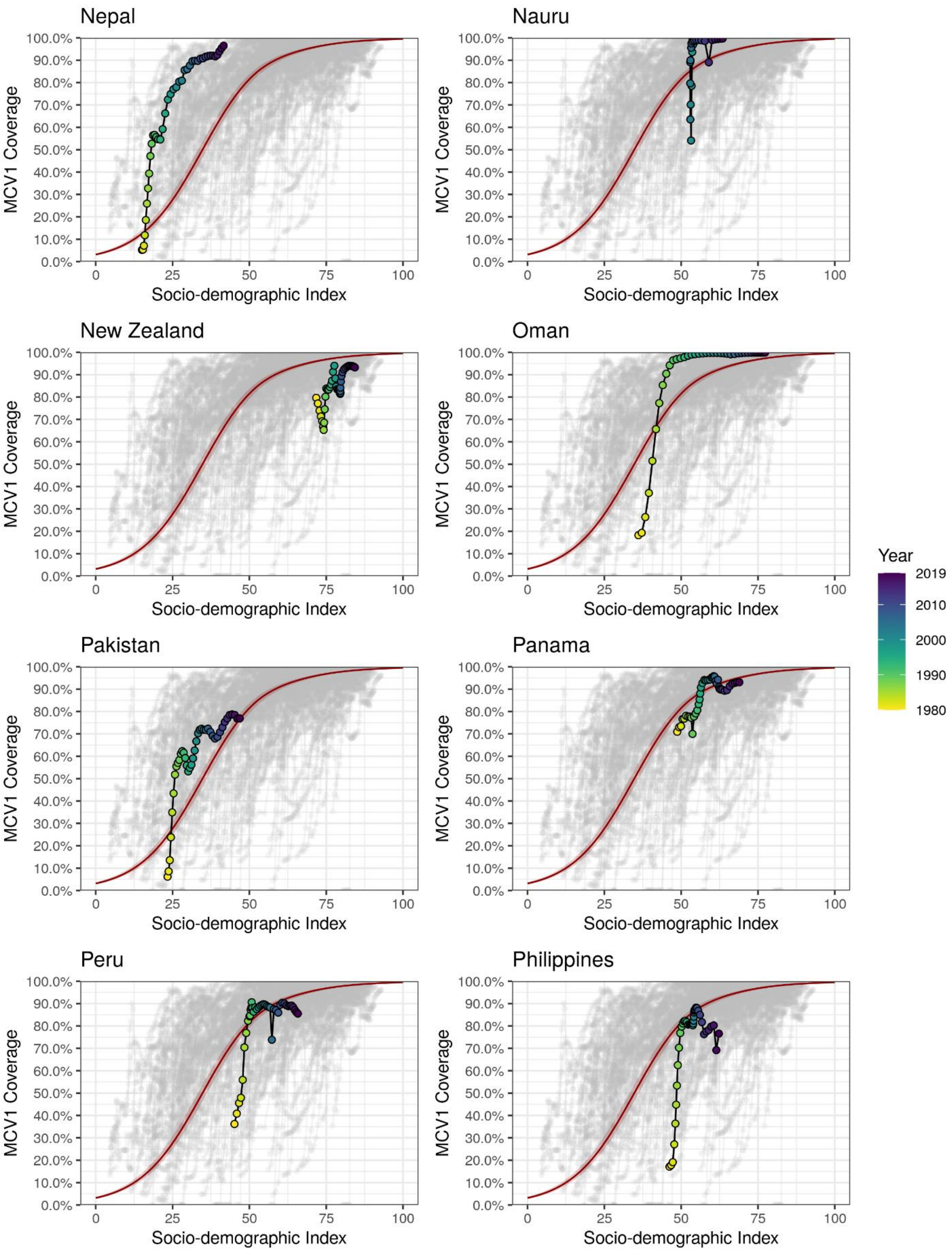
Nepal
Nauru
New Zealand
Oman
Pakistan
Panama
Peru
Philippines
MCV1 Coverage
0.0%
10.0%
20.0%
30.0%
40.0%
50.0%
60.0%
70.0%
80.0%
90.0%
100.0%
Socio-demographic Index
0
25
50
75
100
Year
2019
2010
2000
1990
1980

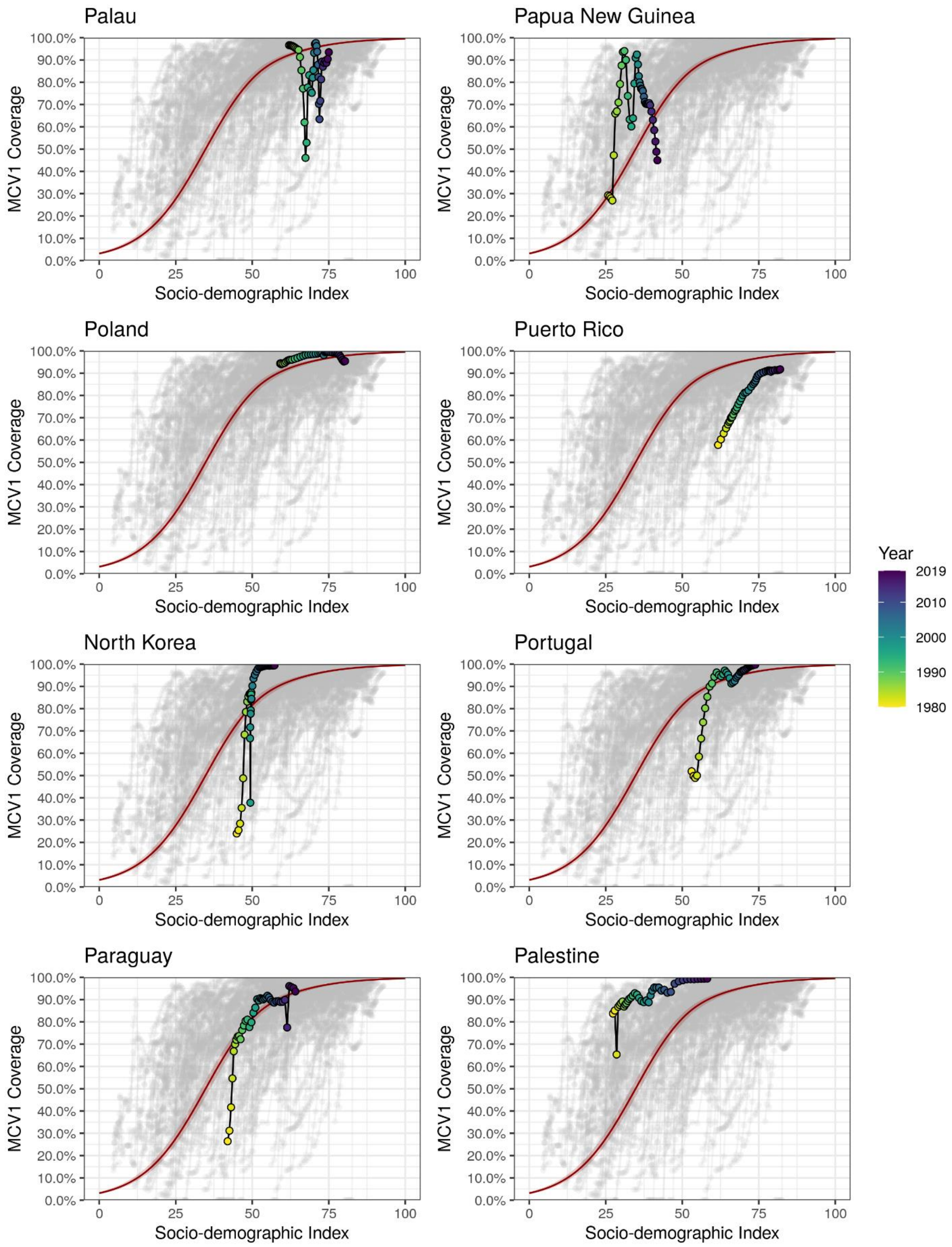
Palau
Papua New Guinea
Poland
Puerto Rico
North Korea
Portugal
Paraguay
Palestine
MCV1 Coverage
Socio-demographic Index
0.0%
10.0%
20.0%
30.0%
40.0%
50.0%
60.0%
70.0%
80.0%
90.0%
100.0%
0
25
50
75
100
Year
2019
2010
2000
1990
1980

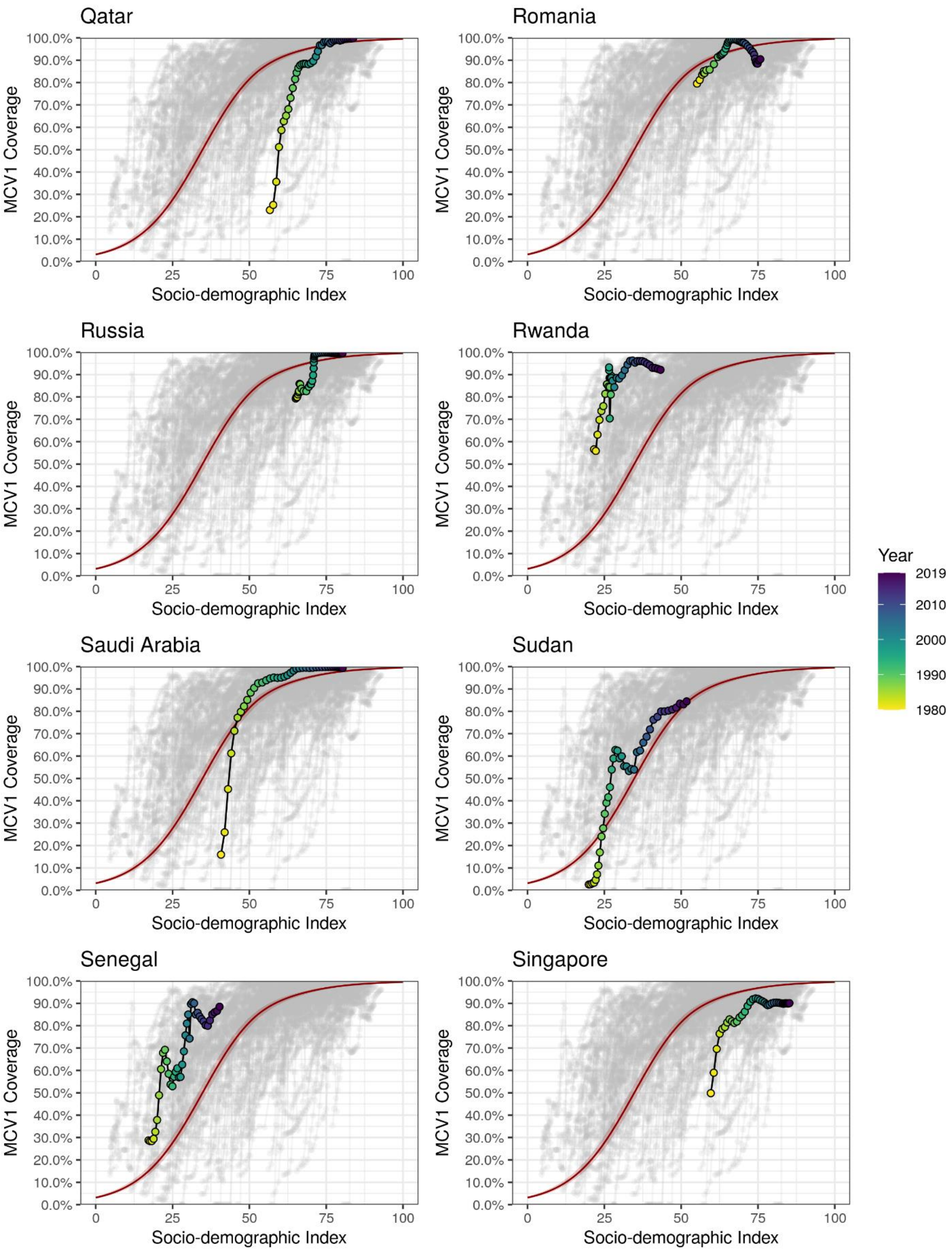
Qatar
Romania
Russia
Rwanda
Saudi Arabia
Sudan
Senegal
Singapore
MCV1 Coverage
Socio-demographic Index
0.0%
10.0%
20.0%
30.0%
40.0%
50.0%
60.0%
70.0%
80.0%
90.0%
100.0%
0
25
50
75
100
Year
2019
2010
2000
1990
1980

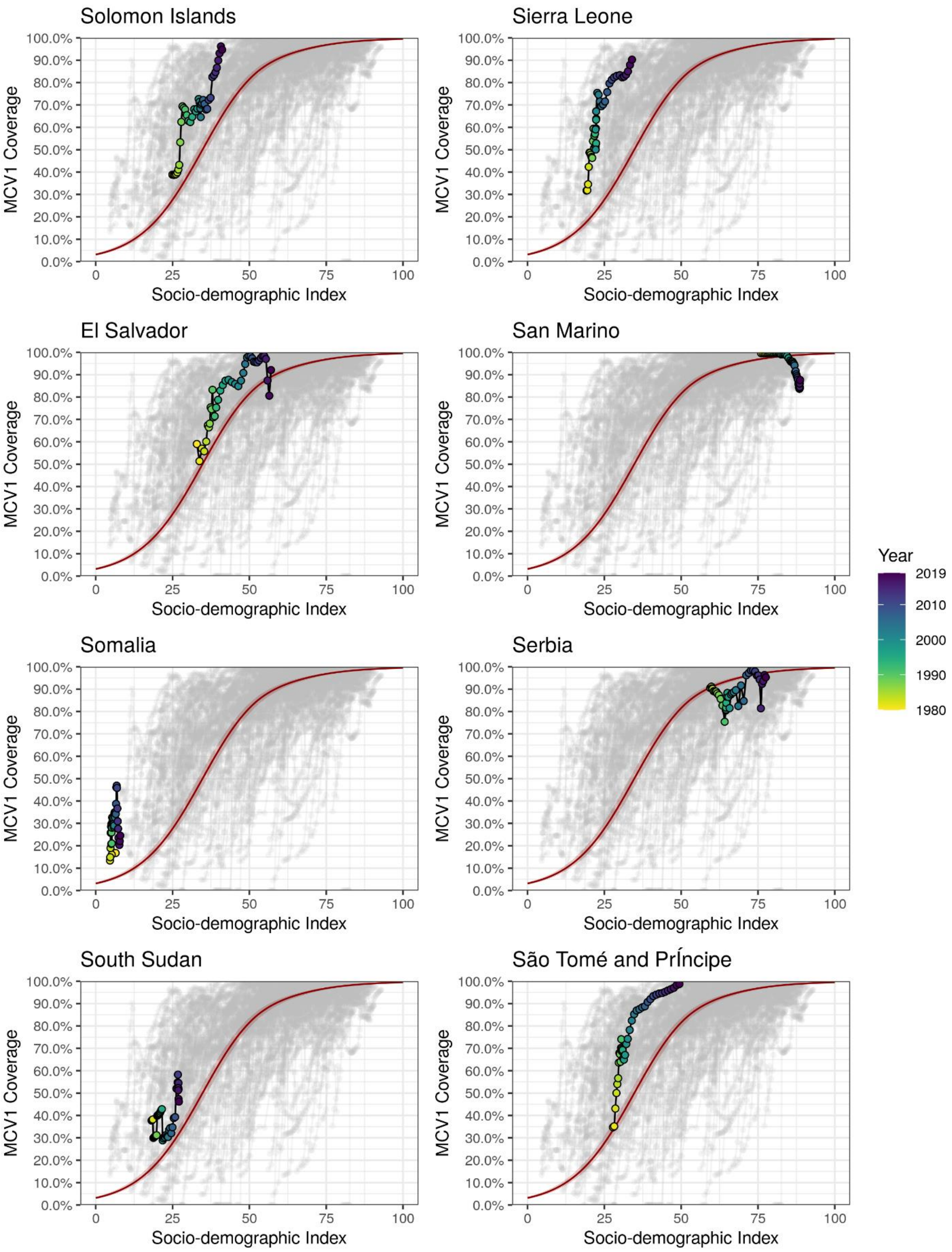
Solomon Islands
Sierra Leone
El Salvador
San Marino
Somalia
Serbia
South Sudan
São Tomé and PrÍncipe
MCV1 Coverage
Socio-demographic Index
0.0%
10.0%
20.0%
30.0%
40.0%
50.0%
60.0%
70.0%
80.0%
90.0%
100.0%
0
25
50
75
100
Year
2019
2010
2000
1990
1980

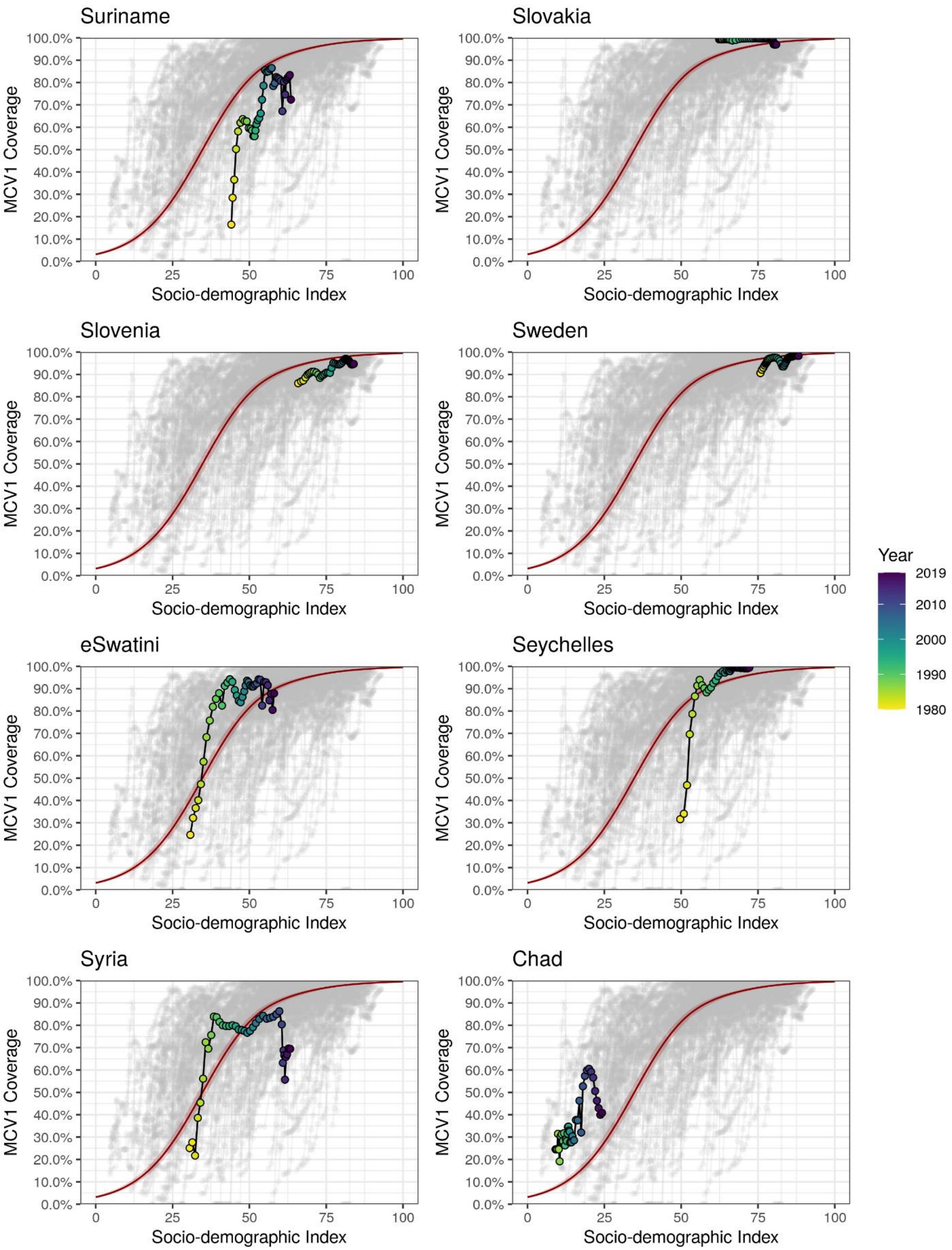
Suriname
Slovakia
Slovenia
Sweden
eSwatini
Seychelles
Syria
Chad
MCV1 Coverage
Socio-demographic Index
0.0%
10.0%
20.0%
30.0%
40.0%
50.0%
60.0%
70.0%
80.0%
90.0%
100.0%
0
25
50
75
100
Year
2019
2010
2000
1990
1980

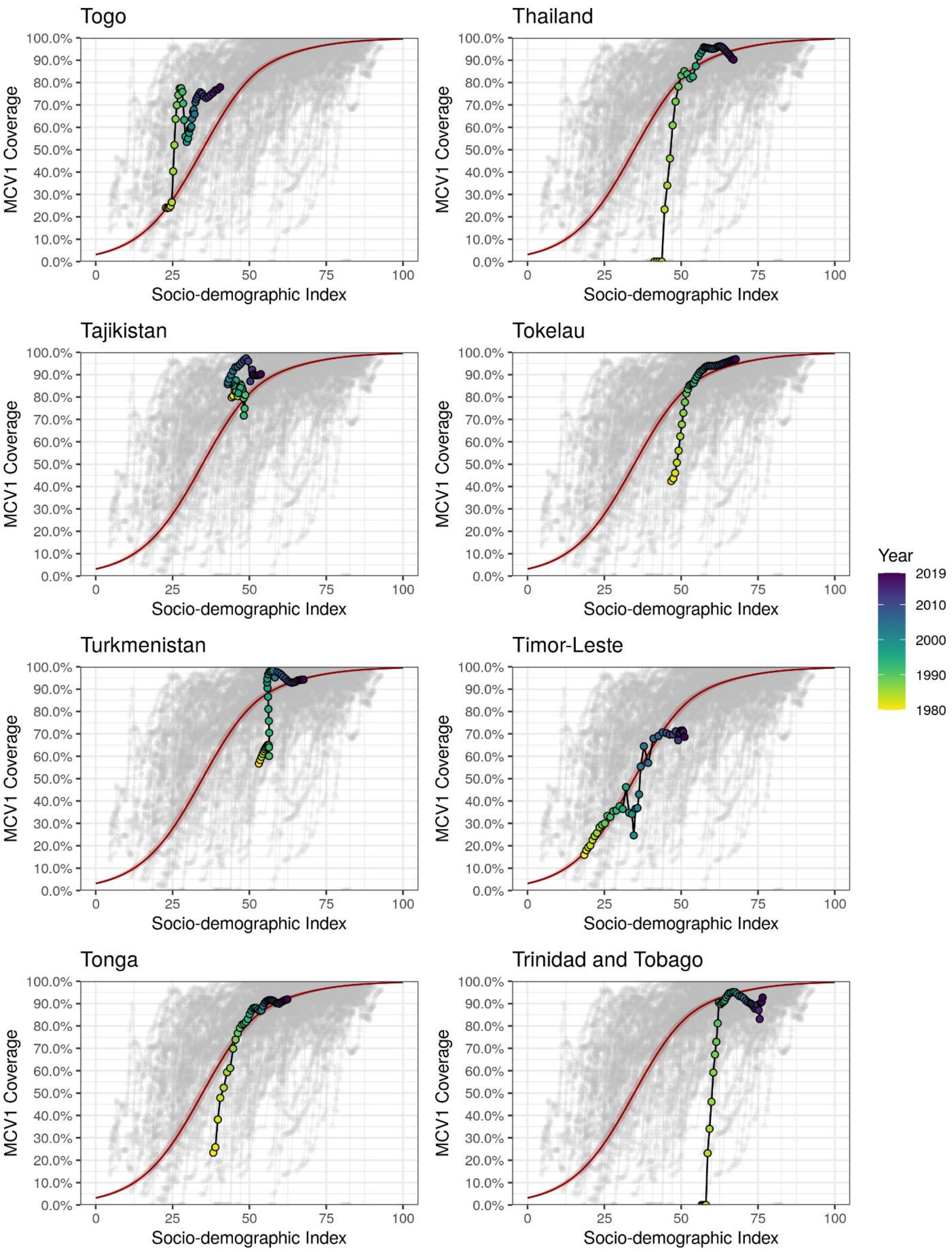
Togo
Thailand
Tajikistan
Tokelau
Turkmenistan
Timor-Leste
Tonga
Trinidad and Tobago
MCV1 Coverage
Socio-demographic Index
0.0%
10.0%
20.0%
30.0%
40.0%
50.0%
60.0%
70.0%
80.0%
90.0%
100.0%
0
25
50
75
100
Year
2019
2010
2000
1990
1980

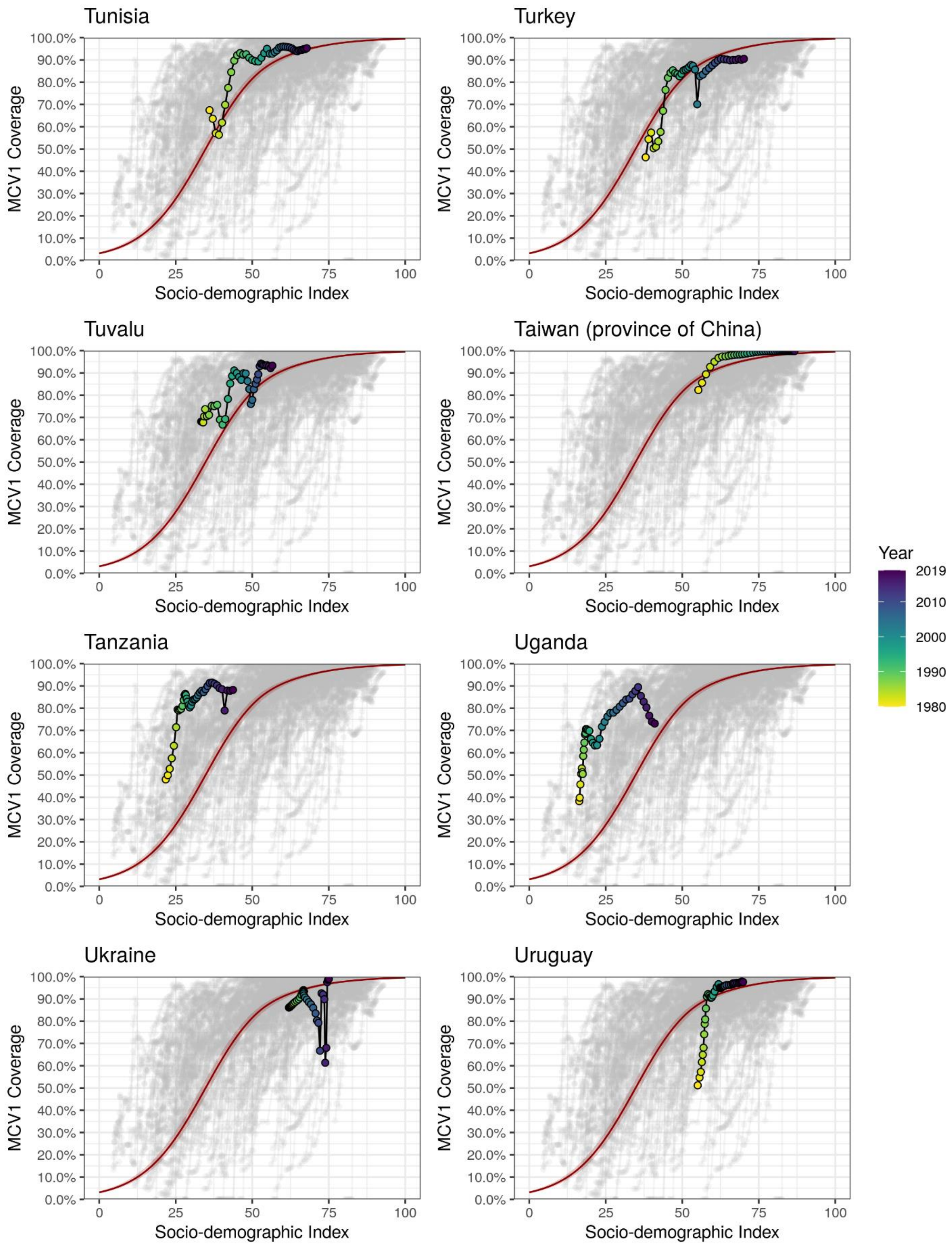
Tunisia
Turkey
Tuvalu
Taiwan (province of China)
Tanzania
Uganda
Ukraine
Uruguay
MCV1 Coverage
Socio-demographic Index
100.0%
90.0%
80.0%
70.0%
60.0%
50.0%
40.0%
30.0%
20.0%
10.0%
0.0%
0
25
50
75
100
Year
2019
2010
2000
1990
1980

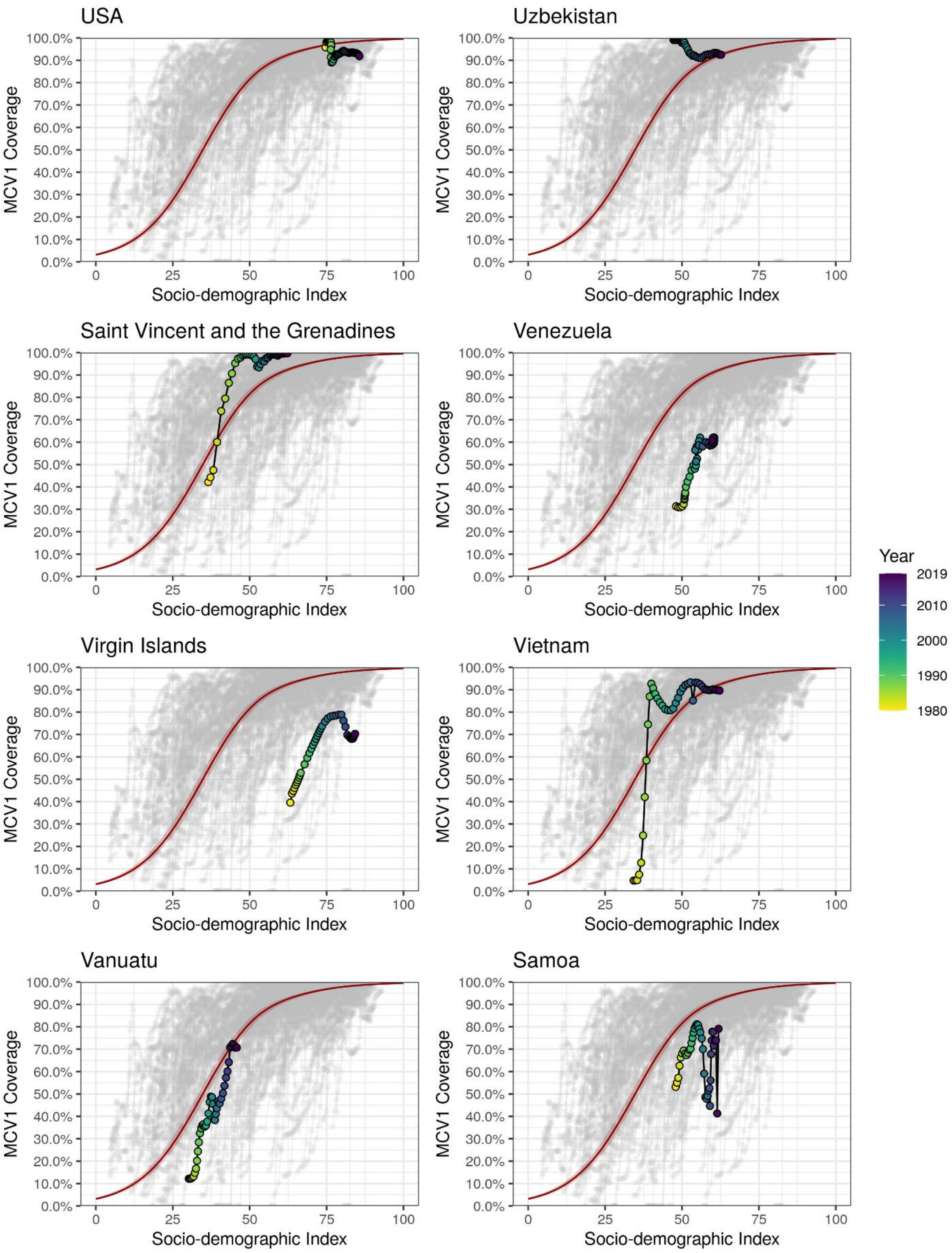
USA
Uzbekistan
Saint Vincent and the Grenadines
Venezuela
Virgin Islands
Vietnam
Vanuatu
Samoa
MCV1 Coverage
Socio-demographic Index
0.0%
10.0%
20.0%
30.0%
40.0%
50.0%
60.0%
70.0%
80.0%
90.0%
100.0%
0
25
50
75
100
Year
2019
2010
2000
1990
1980

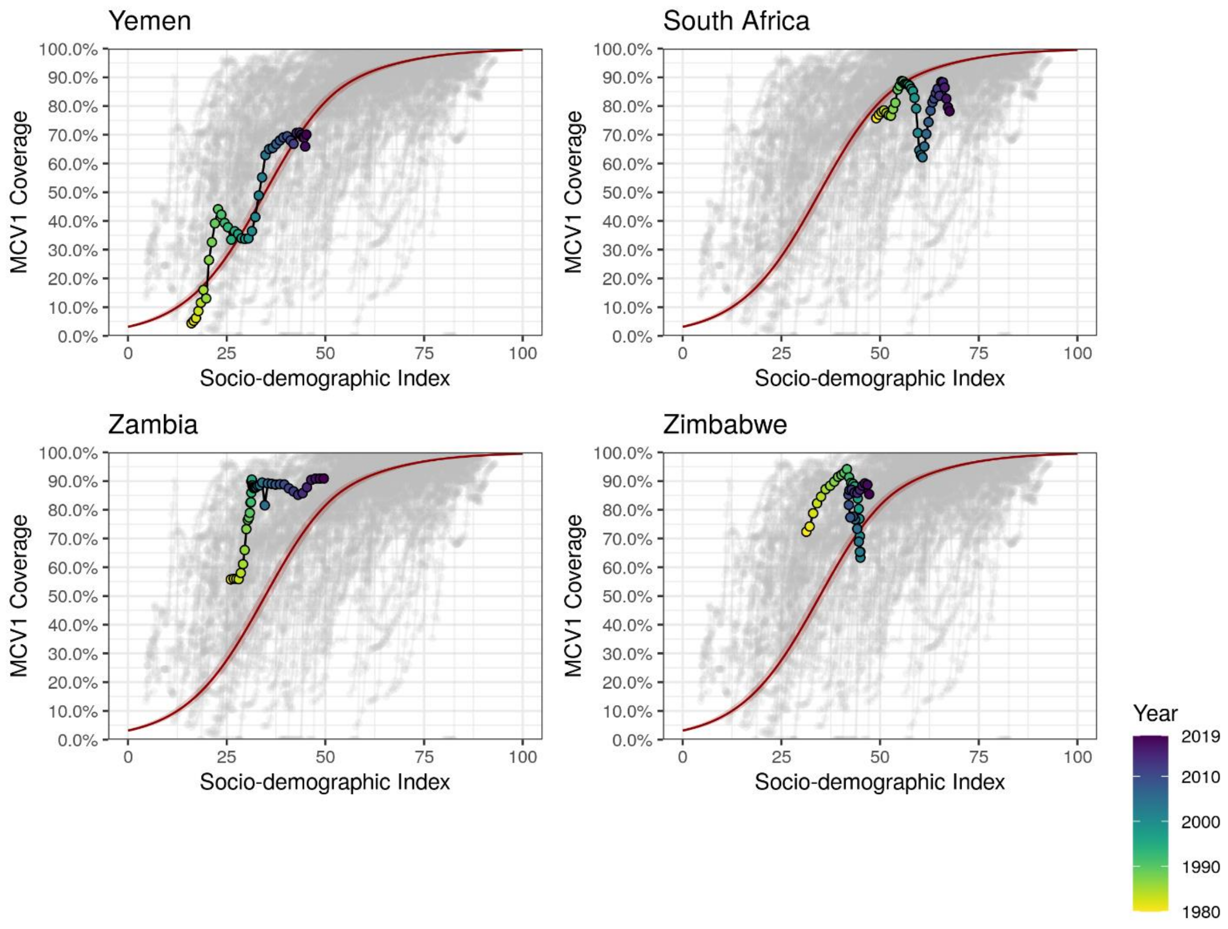
Yemen
South Africa
Zambia
Zimbabwe
MCV1 Coverage
Socio-demographic Index
0.0%
10.0%
20.0%
30.0%
40.0%
50.0%
60.0%
70.0%
80.0%
90.0%
100.0%
0
25
50
75
100
Year
2019
2010
2000
1990
1980

Supplementary figure 17. Observed coverage trend versus expected coverage given SDI, by country, Pol3. Bolded red curve with associated 95% uncertainty represents the mean expected relationship between coverage and SDI from a MR-BRT model. The observed SDI and Pol3 coverage trajectories for each country are shown in light grey. In each location-specific panel, the colour of each point represents the year of estimation, from yellow (1980) to purple (2019), for that country or territory. SDI=Socio-demographic Index. Pol3=polio vaccine, third dose. MR-BRT=meta-regression—Bayesian, regularised, trimmed.

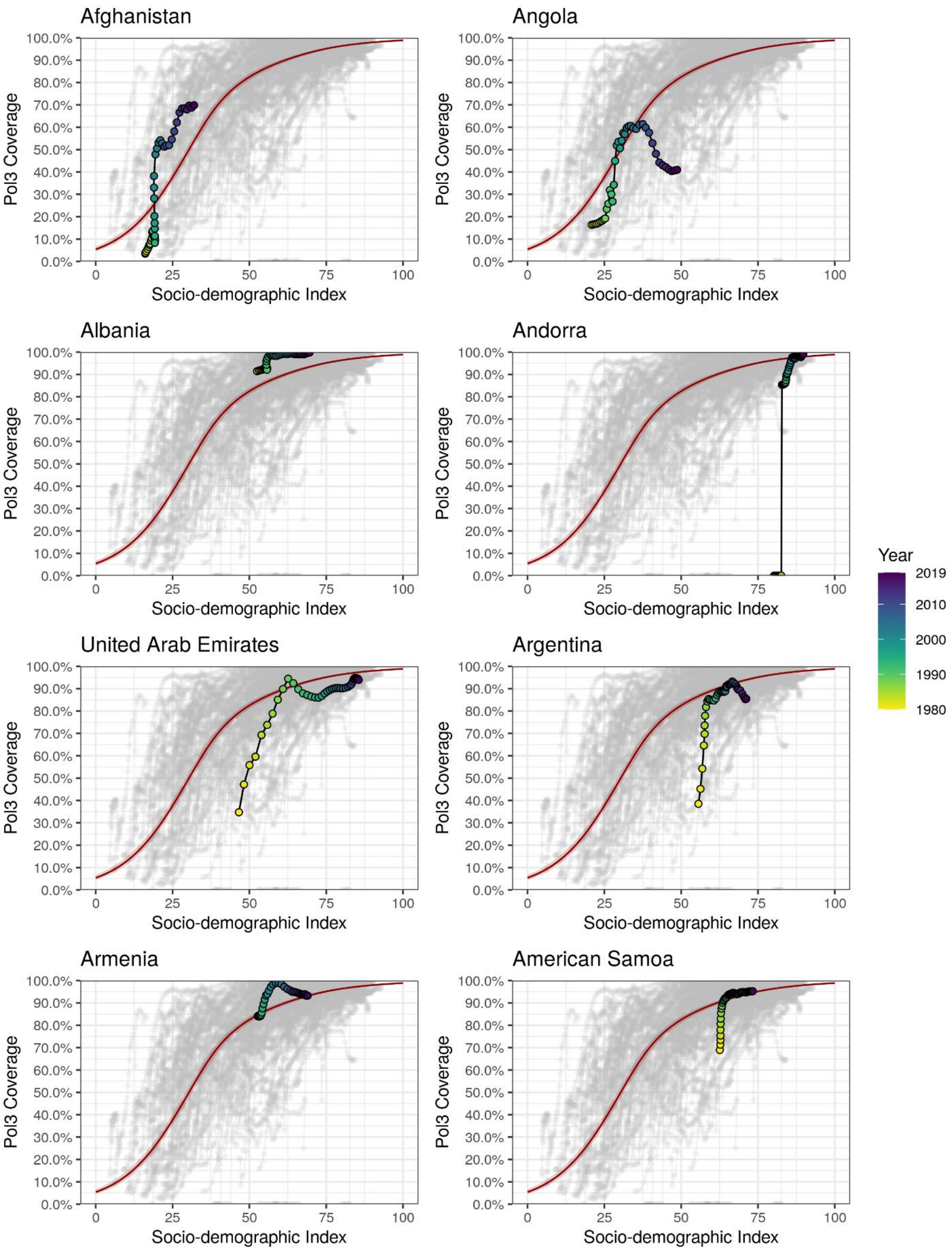
Afghanistan
Angola
Albania
Andorra
United Arab Emirates
Argentina
Armenia
American Samoa
Pol3 Coverage
Socio-demographic Index
100.0%
90.0%
80.0%
70.0%
60.0%
50.0%
40.0%
30.0%
20.0%
10.0%
0.0%
0
25
50
75
100
Year
2019
2010
2000
1990
1980

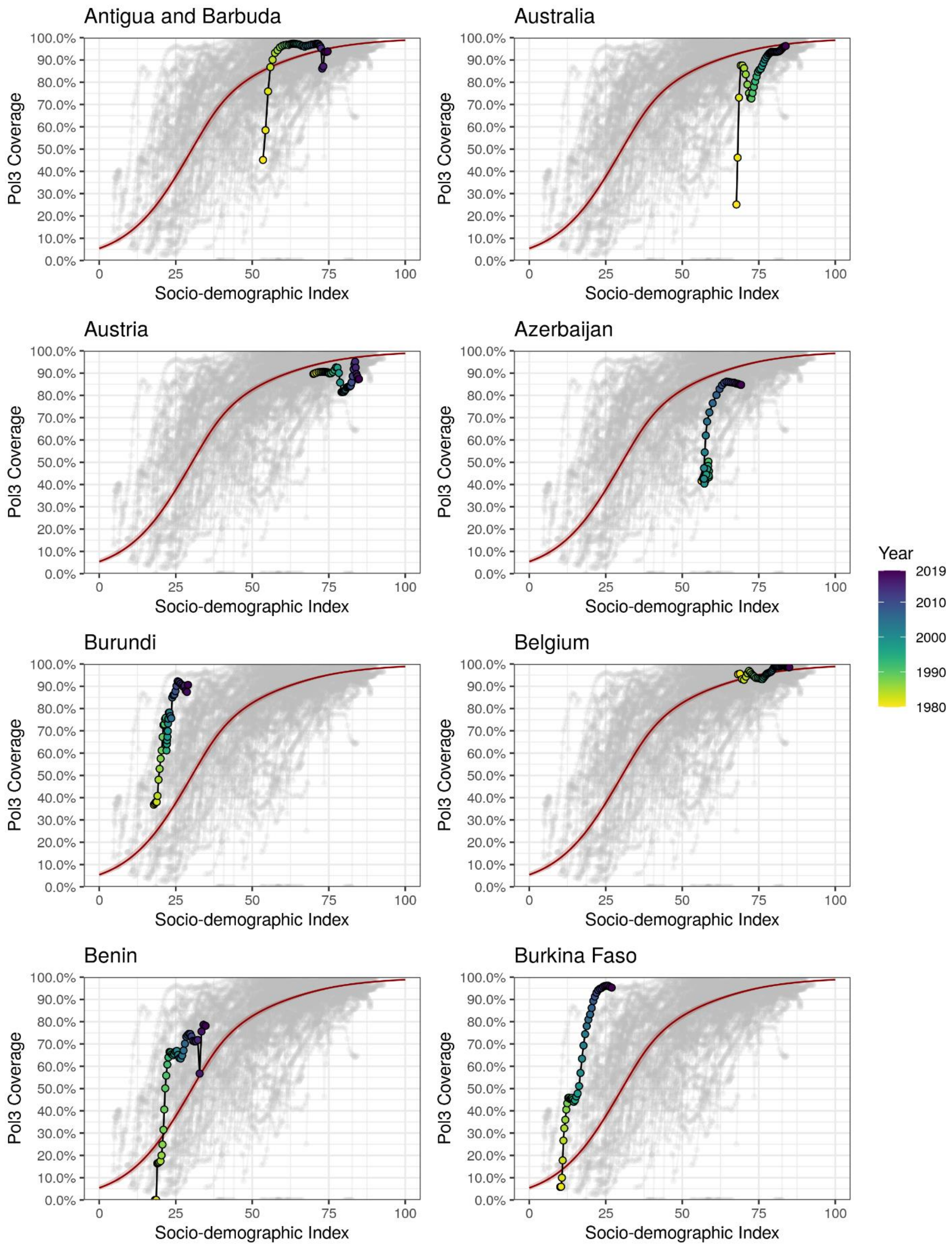
Antigua and Barbuda
Australia
Austria
Azerbaijan
Burundi
Belgium
Benin
Burkina Faso
Pol3 Coverage
Socio-demographic Index
Year
2019
2010
2000
1990
1980

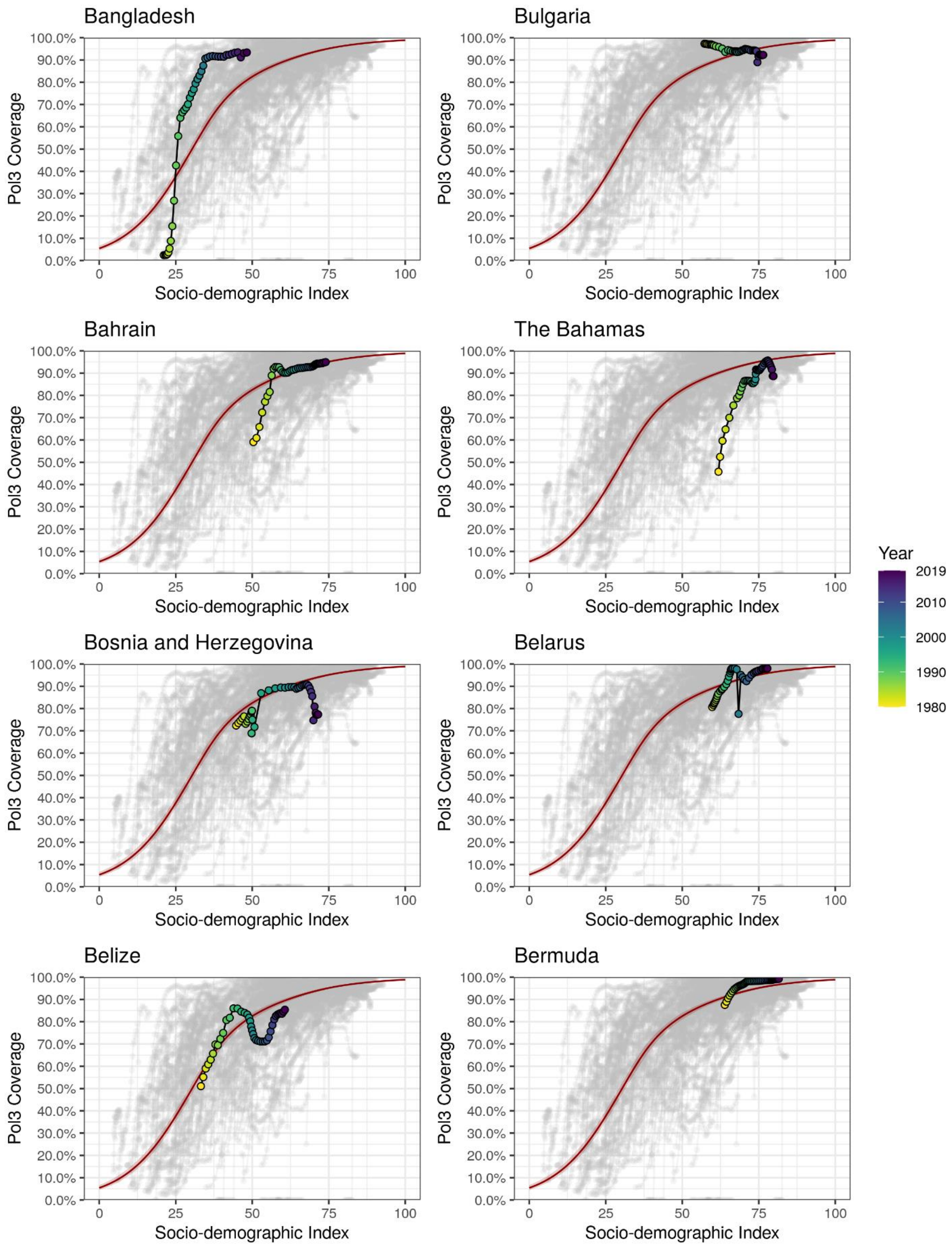

Bangladesh
Bulgaria
Bahrain
The Bahamas
Bosnia and Herzegovina
Belarus
Belize
Bermuda
Pol3 Coverage
Socio-demographic Index
Year
2019
2010
2000
1990
1980

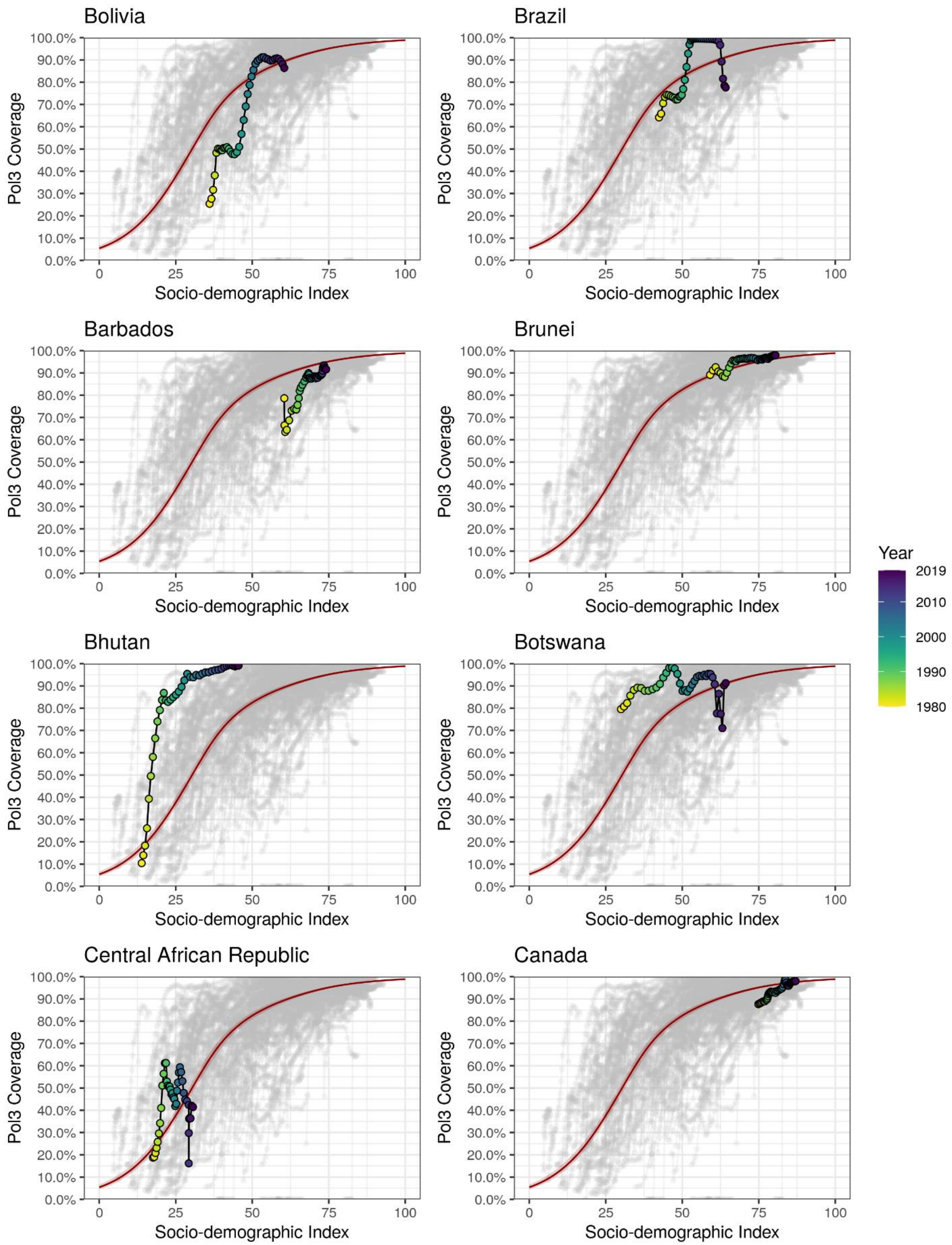
Bolivia
Brazil
Barbados
Brunei
Bhutan
Botswana
Central African Republic
Canada
Pol3 Coverage
Socio-demographic Index
Year
2019
2010
2000
1990
1980

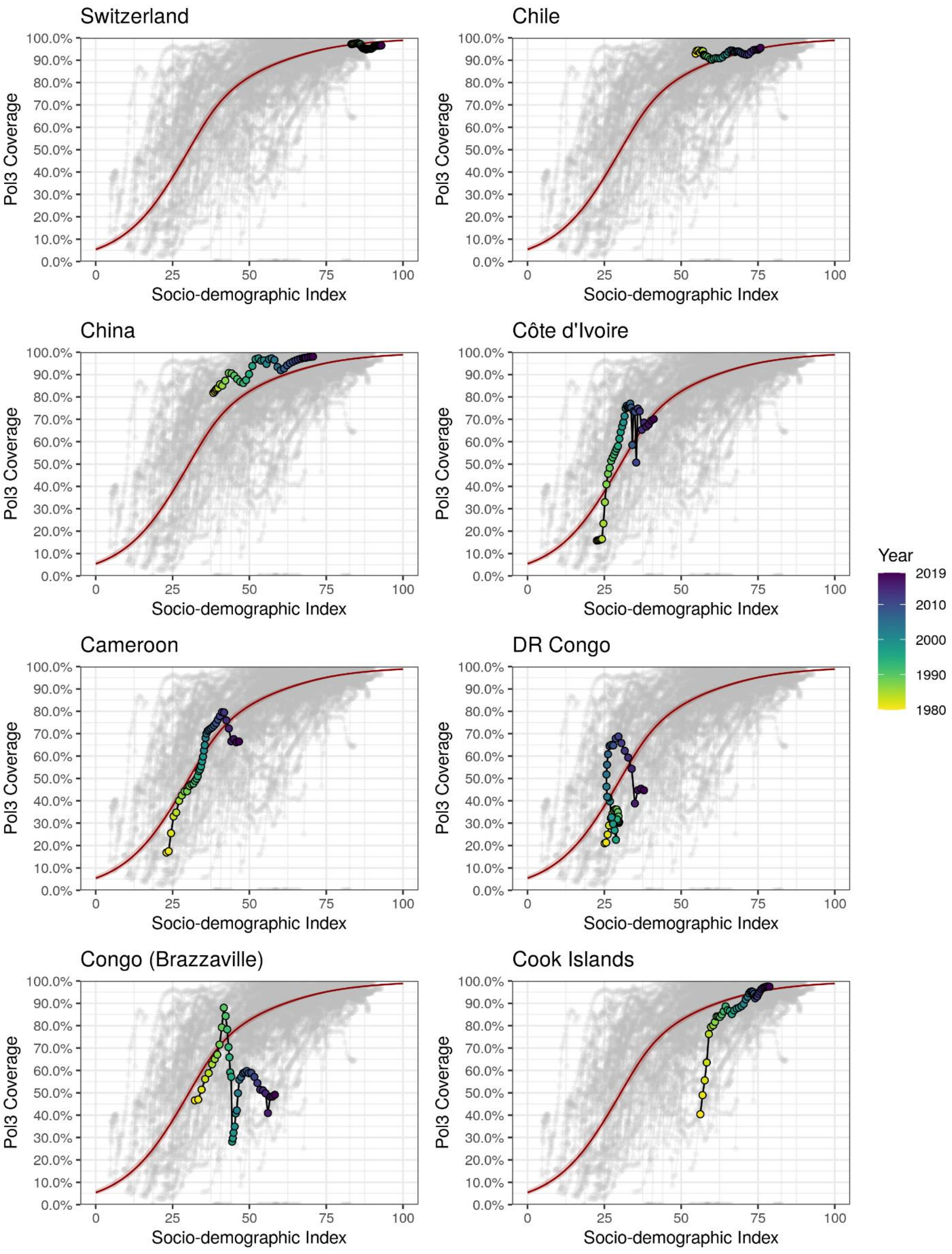
Switzerland
Chile
China
Côte d'Ivoire
Cameroon
DR Congo
Congo (Brazzaville)
Cook Islands
Pol3 Coverage
Socio-demographic Index
0.0%
10.0%
20.0%
30.0%
40.0%
50.0%
60.0%
70.0%
80.0%
90.0%
100.0%
0
25
50
75
100
Year
2019
2010
2000
1990
1980

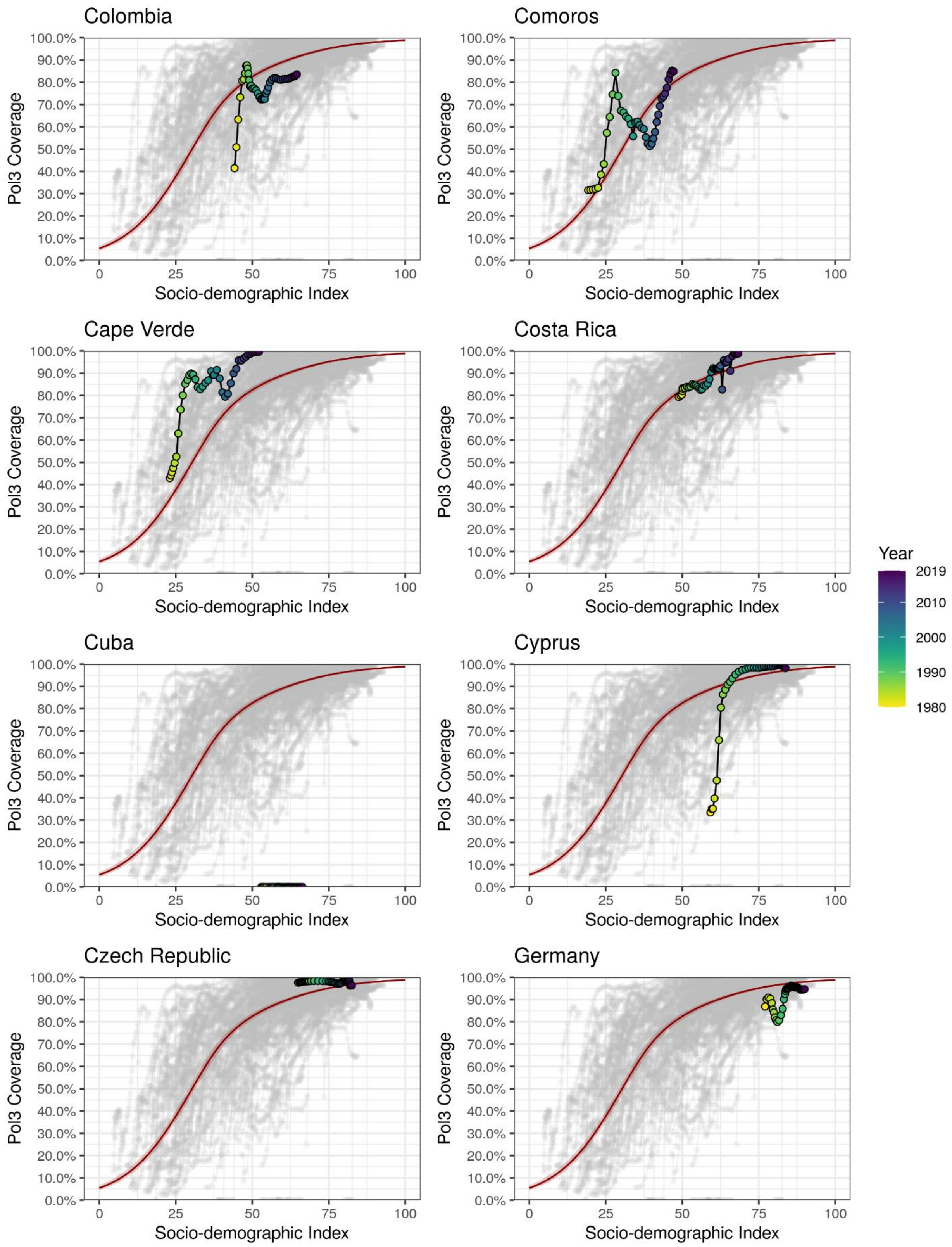
Colombia
Comoros
Cape Verde
Costa Rica
Cuba
Cyprus
Czech Republic
Germany
Pol3 Coverage
Socio-demographic Index
100.0%
90.0%
80.0%
70.0%
60.0%
50.0%
40.0%
30.0%
20.0%
10.0%
0.0%
0
25
50
75
100
Year
2019
2010
2000
1990
1980

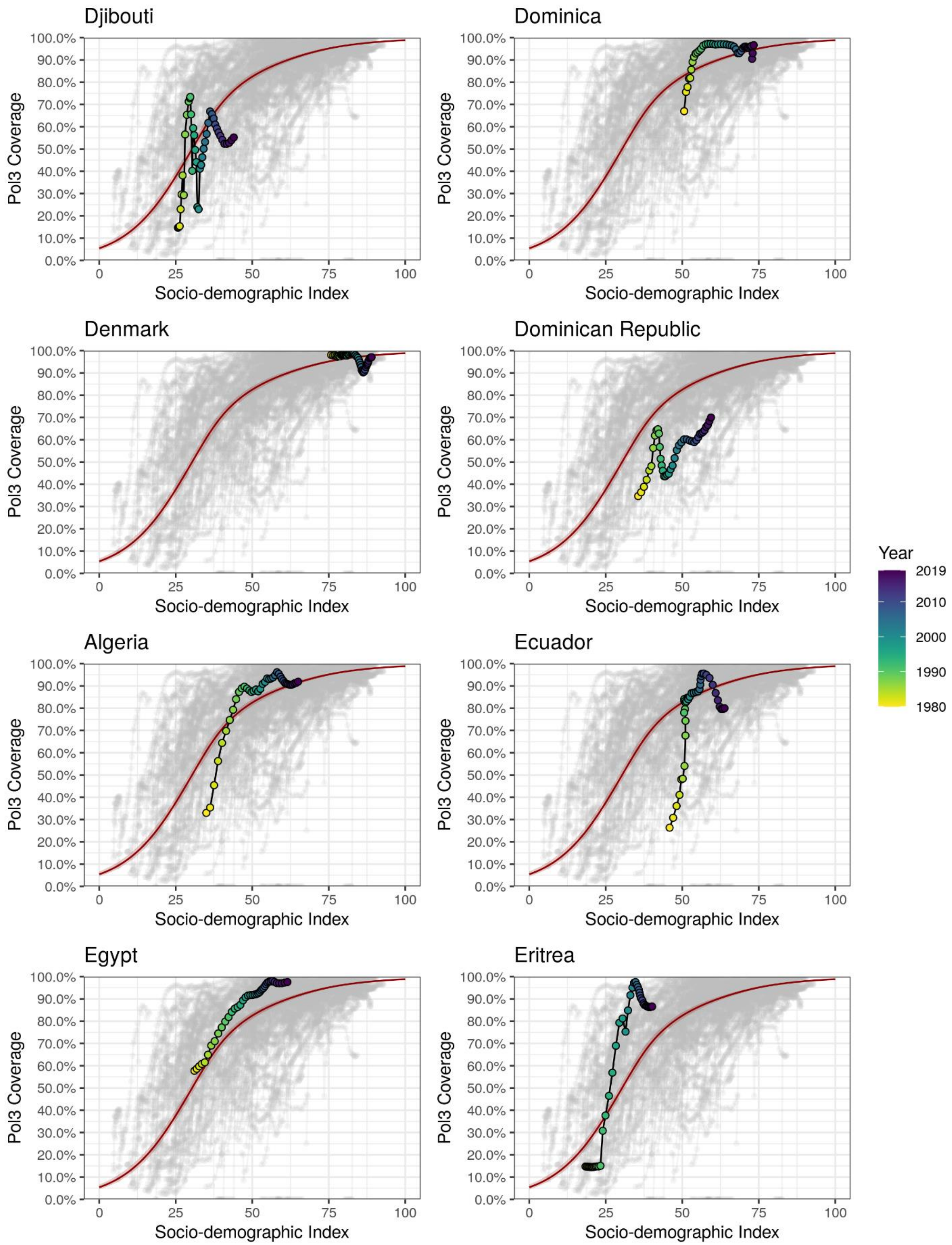
Djibouti
Dominica
Denmark
Dominican Republic
Algeria
Ecuador
Egypt
Eritrea
Pol3 Coverage
Socio-demographic Index
100.0%
90.0%
80.0%
70.0%
60.0%
50.0%
40.0%
30.0%
20.0%
10.0%
0.0%
0
25
50
75
100
Year
2019
2010
2000
1990
1980

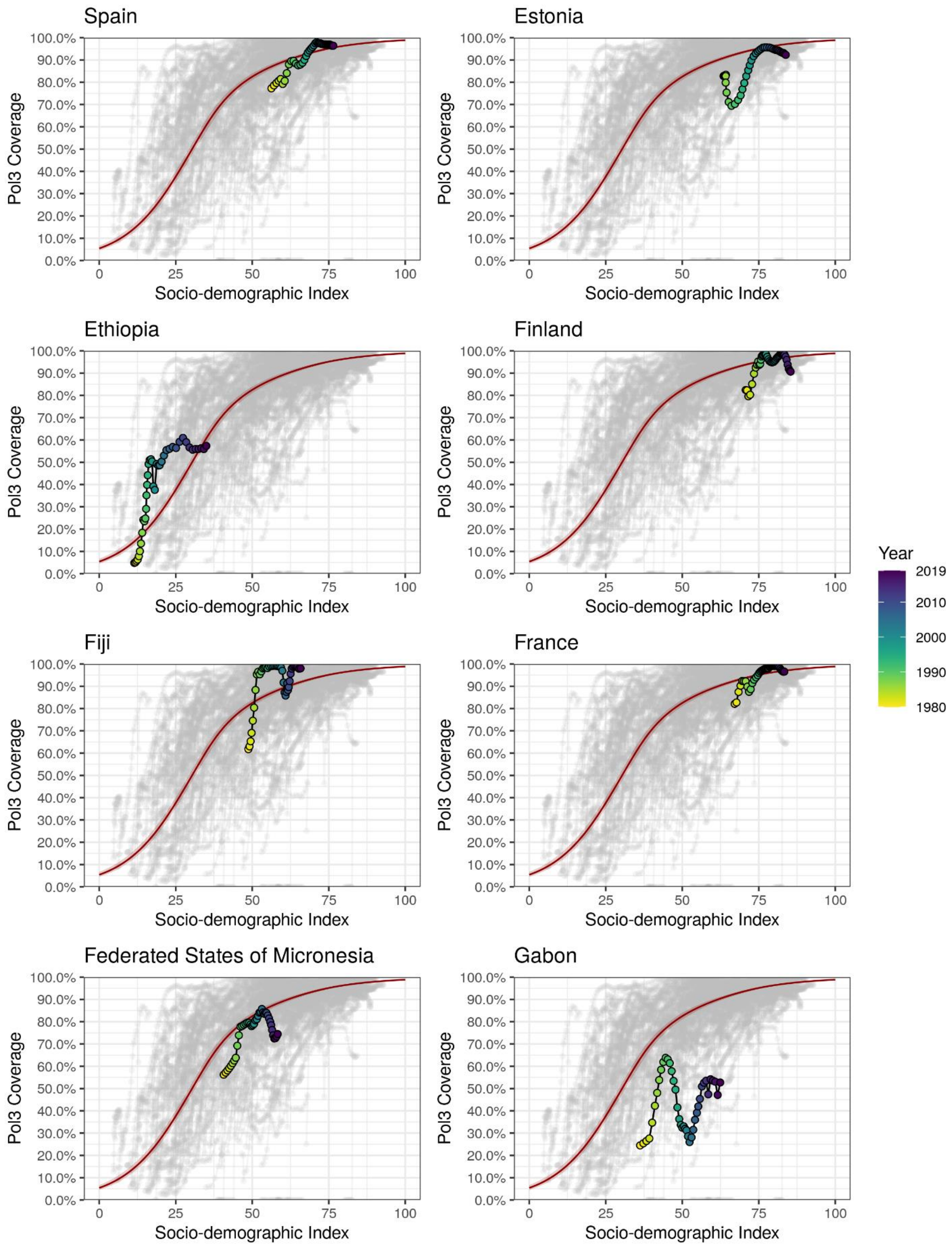
Spain
Estonia
Ethiopia
Finland
Fiji
France
Federated States of Micronesia
Gabon
Pol3 Coverage
Socio-demographic Index
0.0%
10.0%
20.0%
30.0%
40.0%
50.0%
60.0%
70.0%
80.0%
90.0%
100.0%
0
25
50
75
100
Year
2019
2010
2000
1990
1980

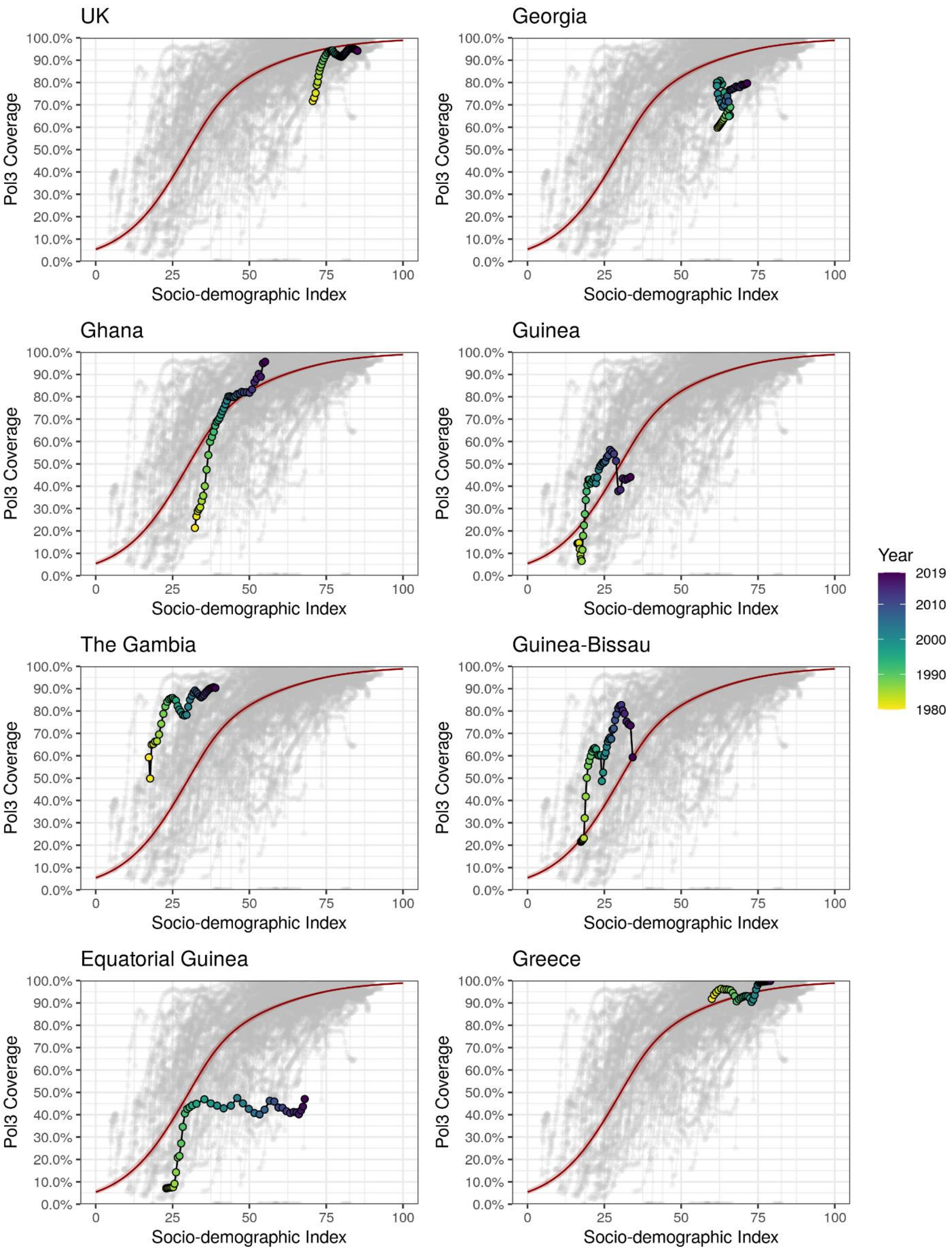
UK
Georgia
Ghana
Guinea
The Gambia
Guinea-Bissau
Equatorial Guinea
Greece
Pol3 Coverage
Socio-demographic Index
100.0%
90.0%
80.0%
70.0%
60.0%
50.0%
40.0%
30.0%
20.0%
10.0%
0.0%
0
25
50
75
100
Year
2019
2010
2000
1990
1980

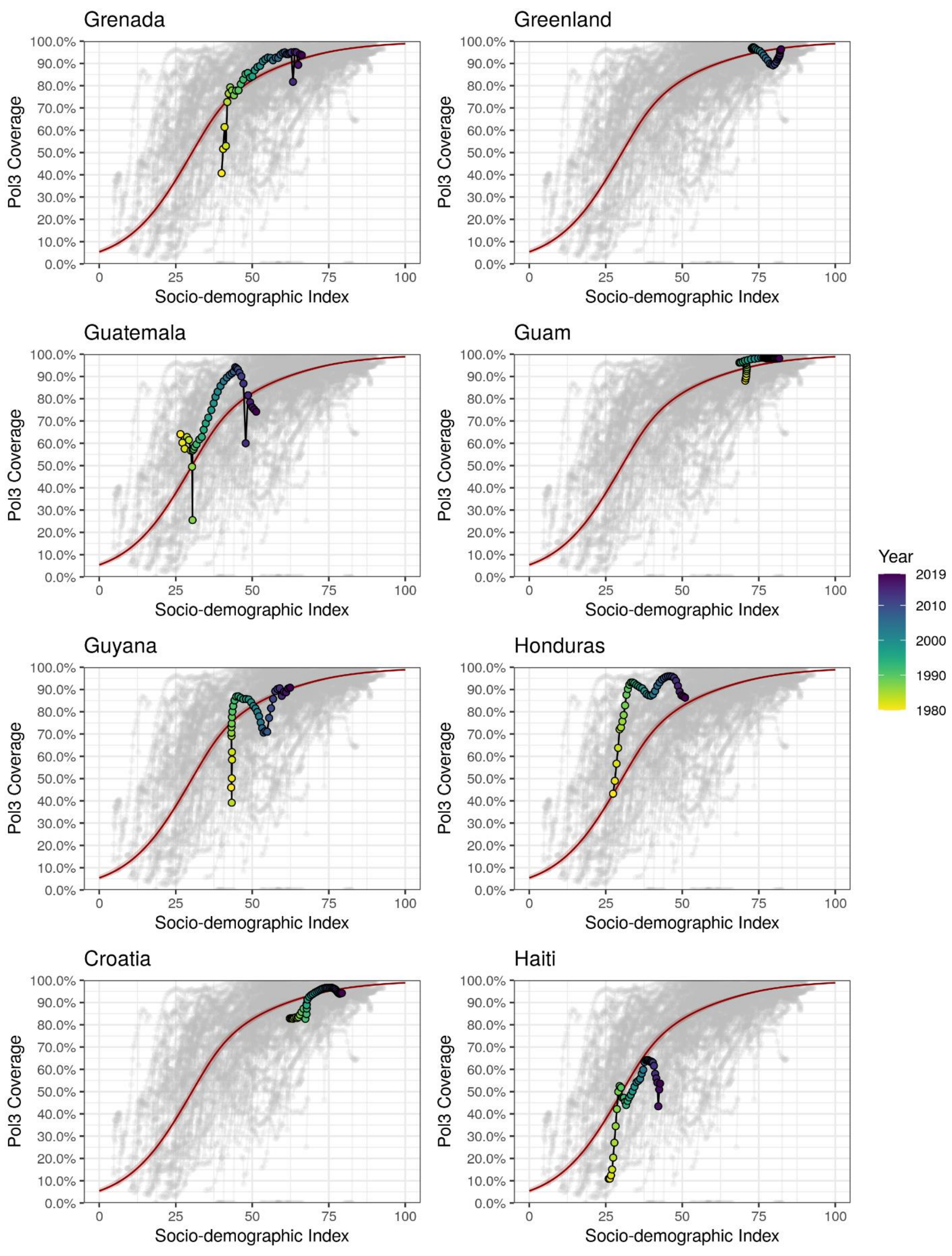
Grenada
Greenland
Guatemala
Guam
Guyana
Honduras
Croatia
Haiti
Pol3 Coverage
Socio-demographic Index
0.0%
10.0%
20.0%
30.0%
40.0%
50.0%
60.0%
70.0%
80.0%
90.0%
100.0%
0
25
50
75
100
Year
2019
2010
2000
1990
1980

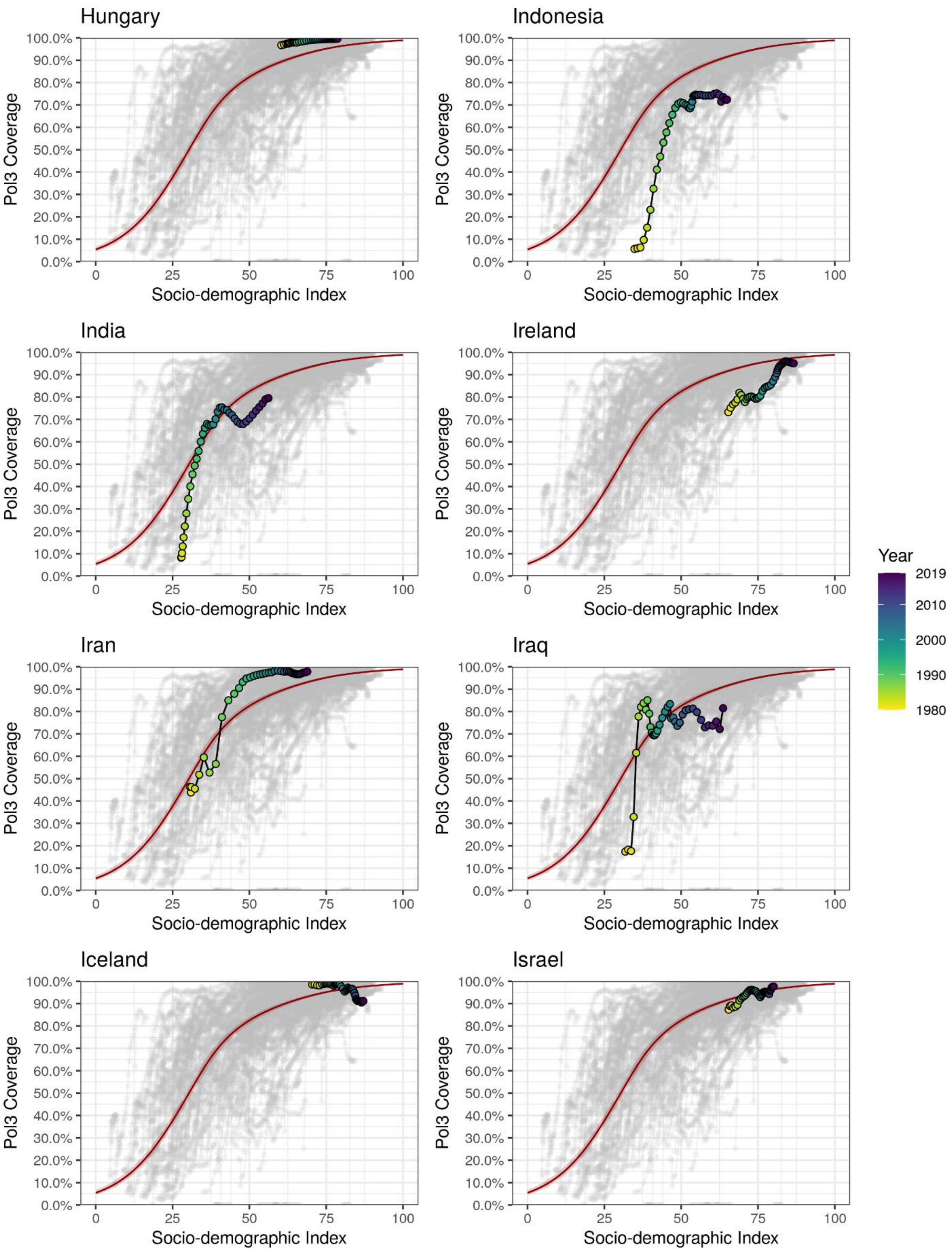
Hungary
Indonesia
India
Ireland
Iran
Iraq
Iceland
Israel
Pol3 Coverage
Socio-demographic Index
100.0%
90.0%
80.0%
70.0%
60.0%
50.0%
40.0%
30.0%
20.0%
10.0%
0.0%
0
25
50
75
100
Year
2019
2010
2000
1990
1980

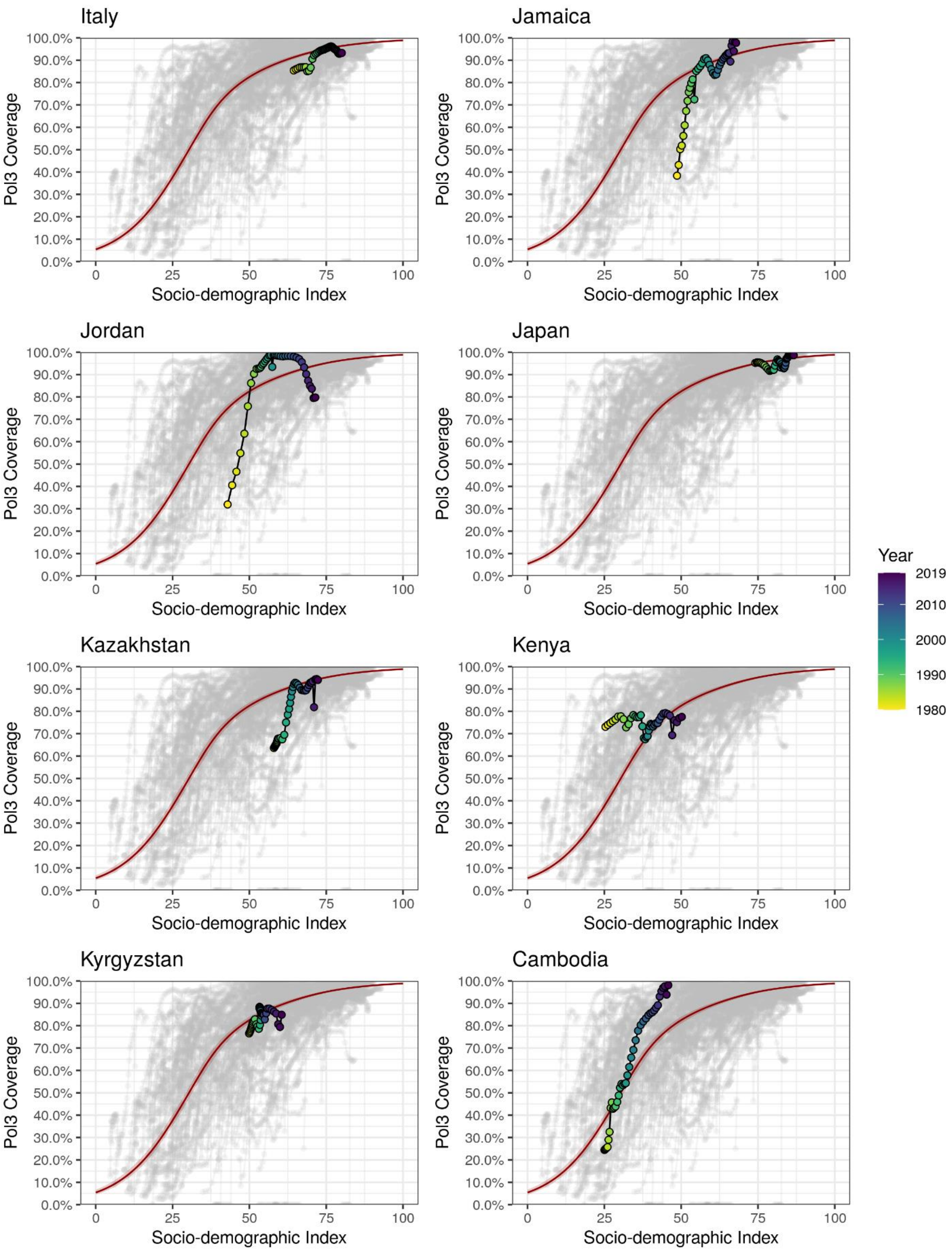
Italy
Jamaica
Jordan
Japan
Kazakhstan
Kenya
Kyrgyzstan
Cambodia
Pol3 Coverage
Socio-demographic Index
0.0%
10.0%
20.0%
30.0%
40.0%
50.0%
60.0%
70.0%
80.0%
90.0%
100.0%
0
25
50
75
100
Year
2019
2010
2000
1990
1980

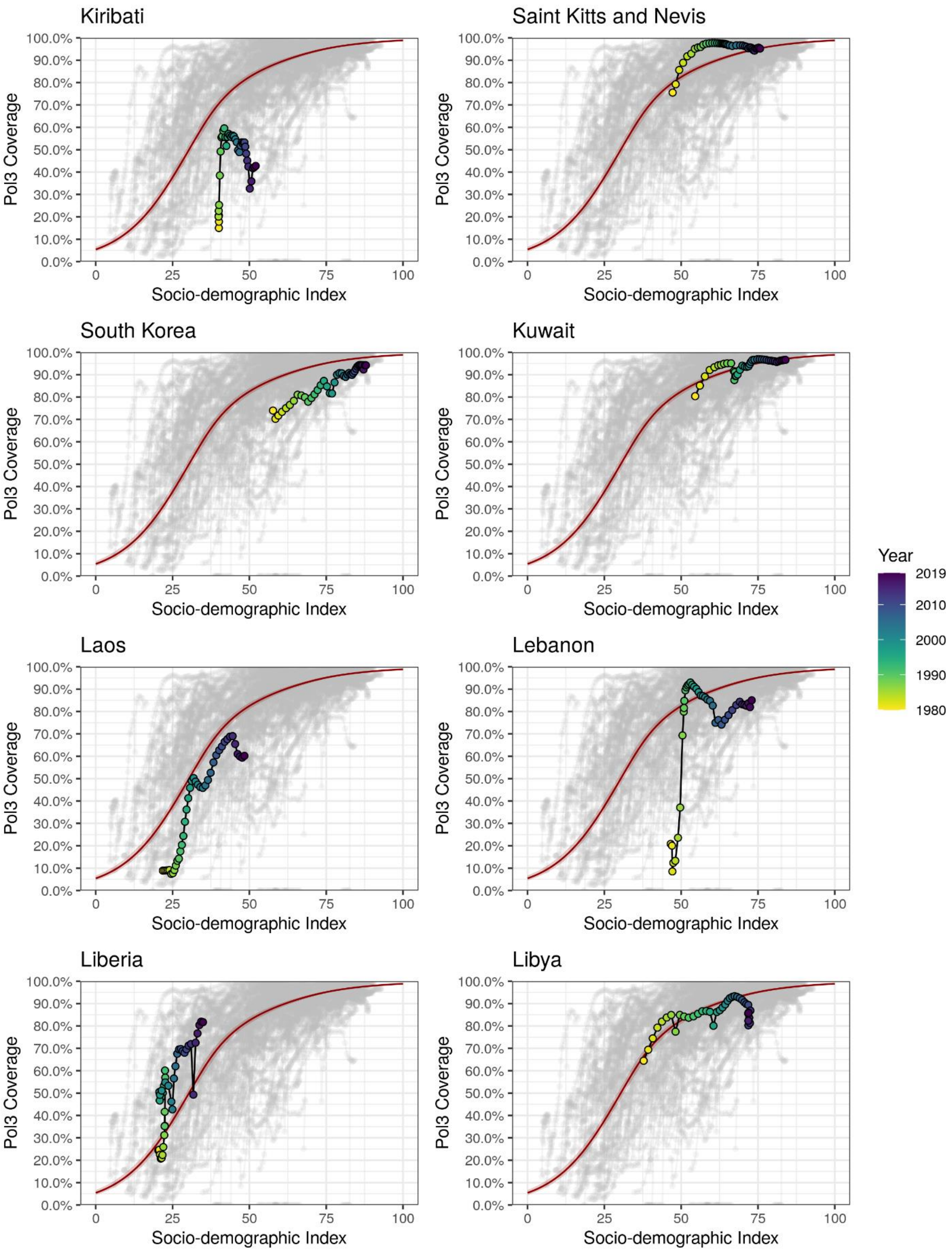
Kiribati
Saint Kitts and Nevis
South Korea
Kuwait
Laos
Lebanon
Liberia
Libya
Pol3 Coverage
Socio-demographic Index
0.0%
10.0%
20.0%
30.0%
40.0%
50.0%
60.0%
70.0%
80.0%
90.0%
100.0%
0
25
50
75
100
Year
2019
2010
2000
1990
1980

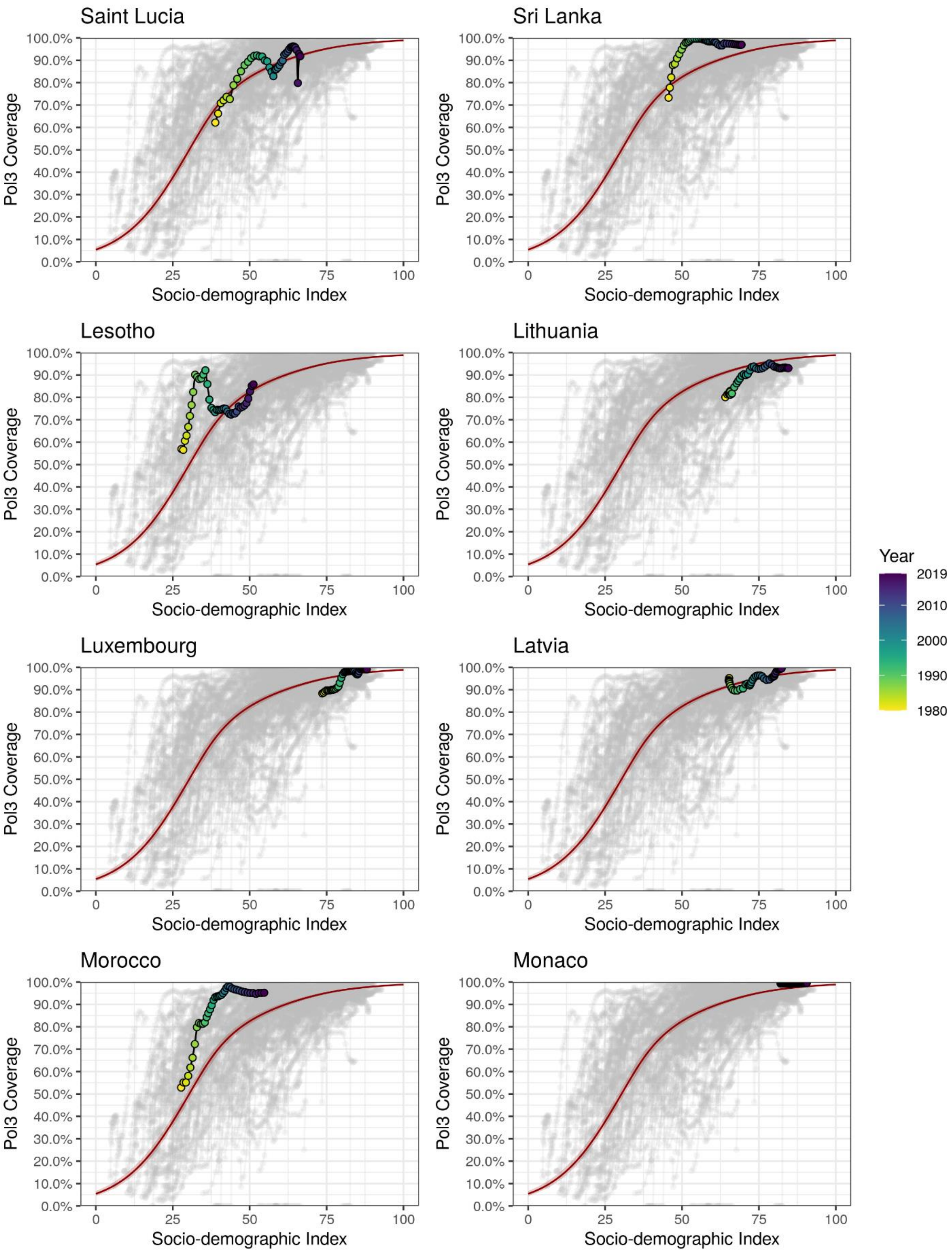
Saint Lucia
Sri Lanka
Lesotho
Lithuania
Luxembourg
Latvia
Morocco
Monaco
Pol3 Coverage
100.0%
90.0%
80.0%
70.0%
60.0%
50.0%
40.0%
30.0%
20.0%
10.0%
0.0%
0
25
50
75
100
Socio-demographic Index
Year
2019
2010
2000
1990
1980

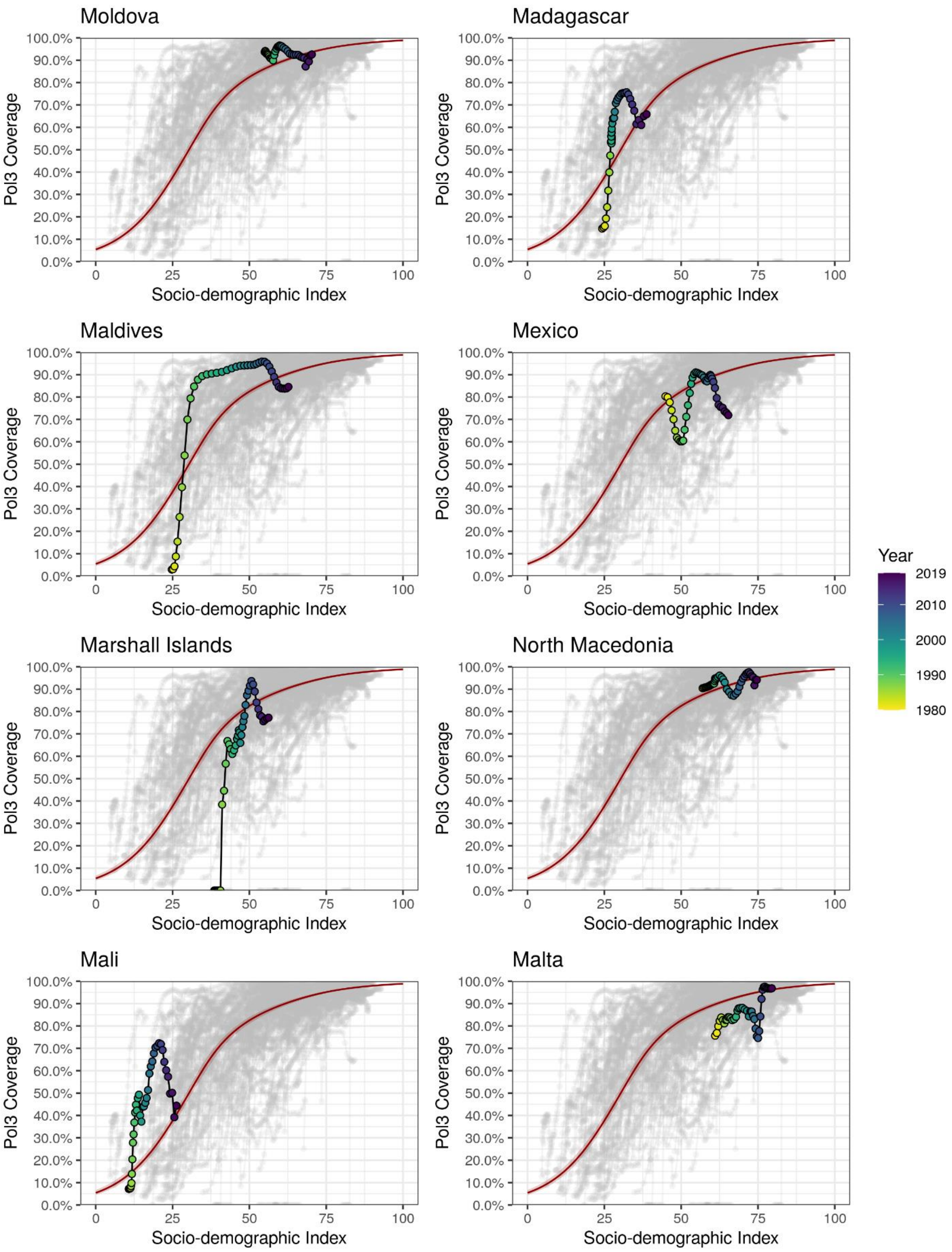

Moldova
Madagascar
Maldives
Mexico
Marshall Islands
North Macedonia
Mali
Malta
Pol3 Coverage
Socio-demographic Index
0.0%
10.0%
20.0%
30.0%
40.0%
50.0%
60.0%
70.0%
80.0%
90.0%
100.0%
0
25
50
75
100
Year
2019
2010
2000
1990
1980

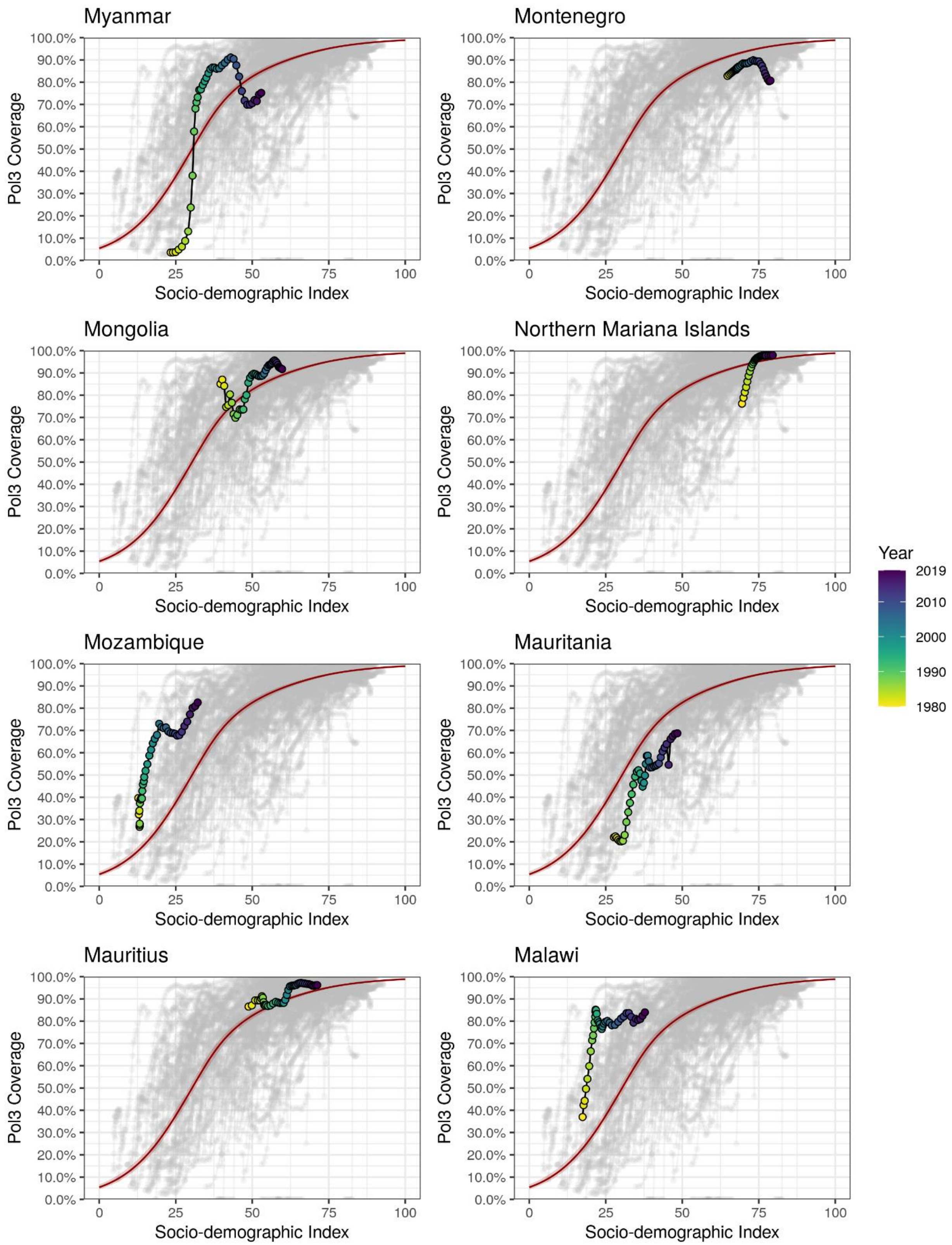

Myanmar
Montenegro
Mongolia
Northern Mariana Islands
Mozambique
Mauritania
Mauritius
Malawi
Pol3 Coverage
Socio-demographic Index
Year
2019
2010
2000
1990
1980

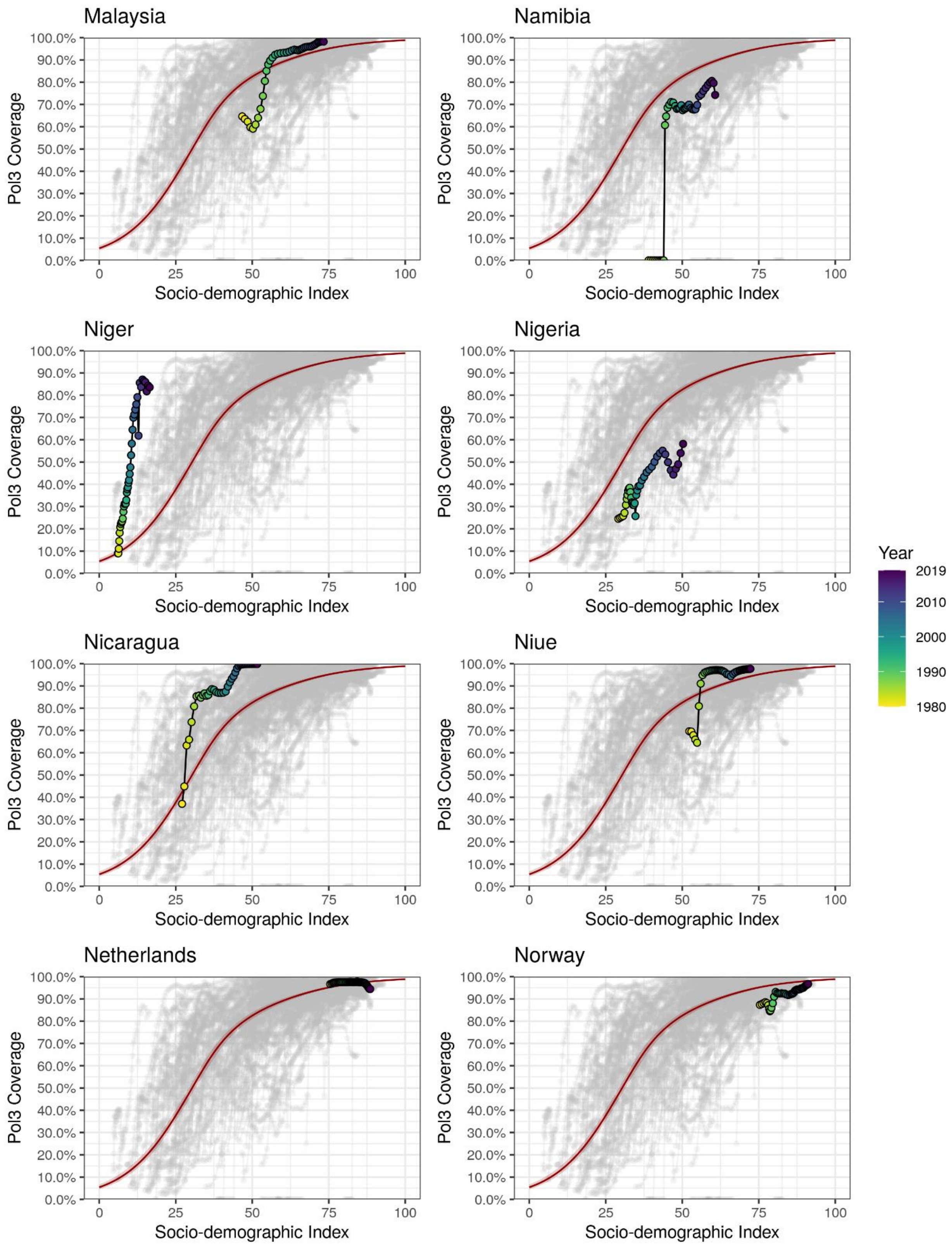

Malaysia
Namibia
Niger
Nigeria
Nicaragua
Niue
Netherlands
Norway
Pol3 Coverage
Socio-demographic Index
Year
2019
2010
2000
1990
1980

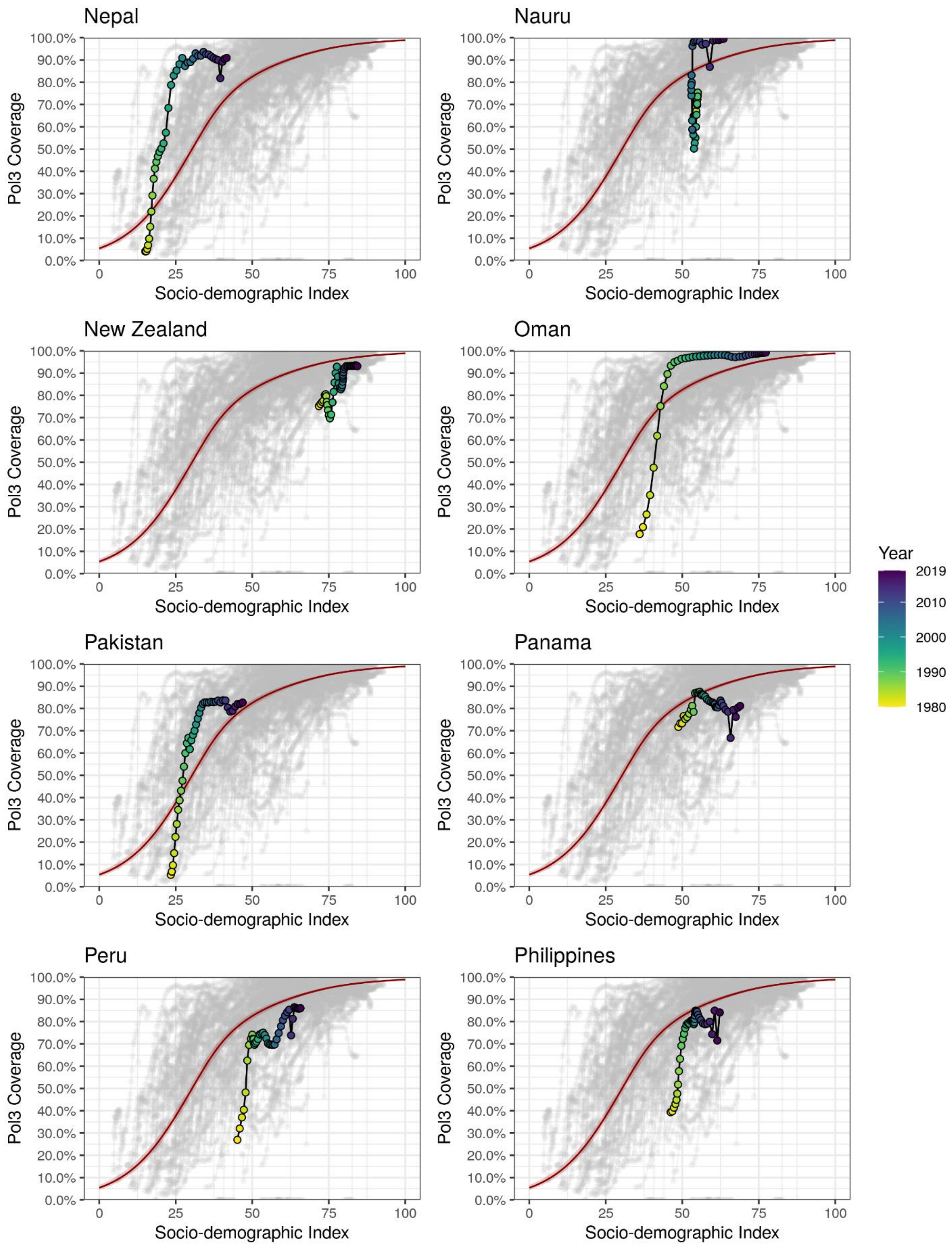
Nepal
Nauru
New Zealand
Oman
Pakistan
Panama
Peru
Philippines
Pol3 Coverage
Socio-demographic Index
100.0%
90.0%
80.0%
70.0%
60.0%
50.0%
40.0%
30.0%
20.0%
10.0%
0.0%
0
25
50
75
100
Year
2019
2010
2000
1990
1980

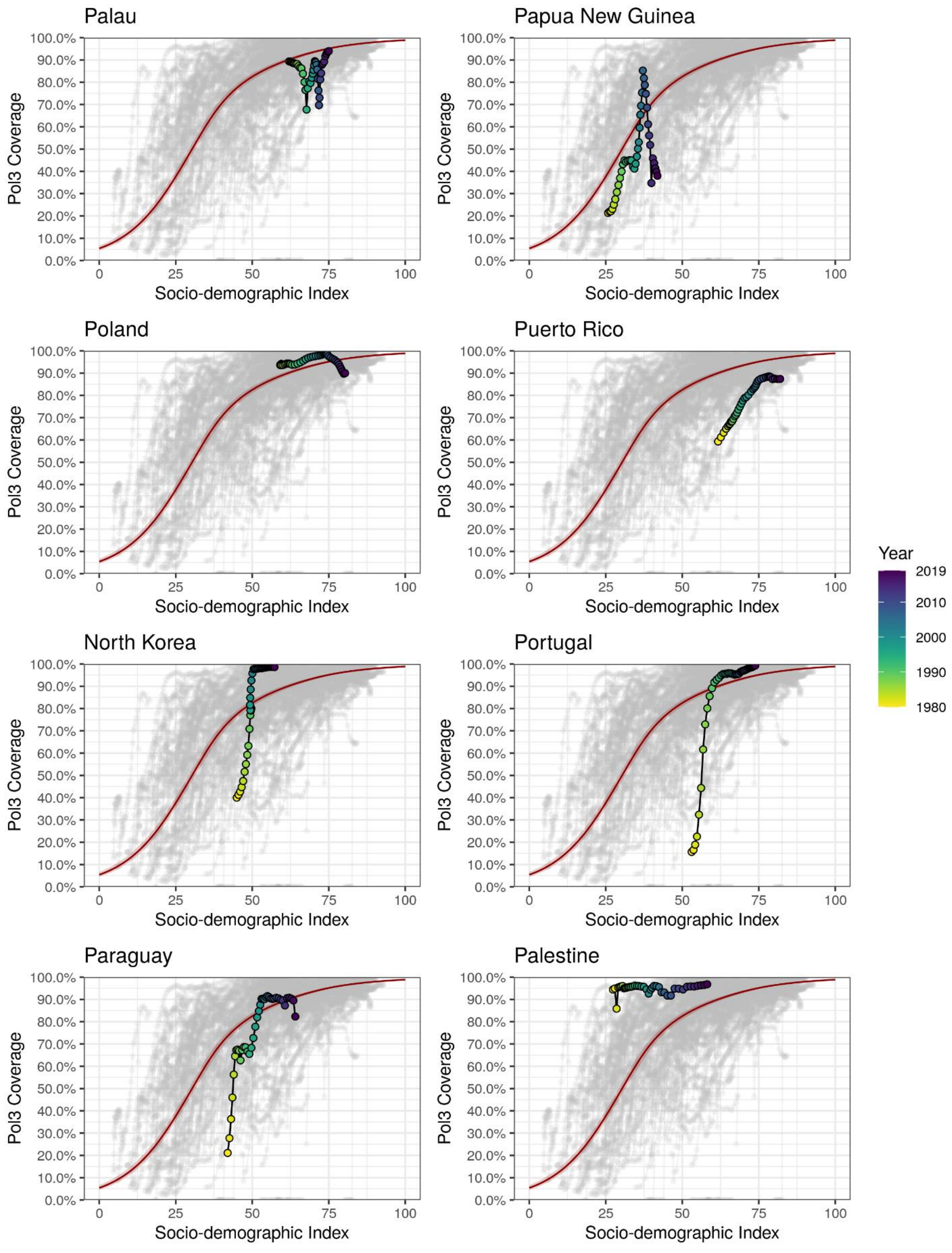

Palau
Papua New Guinea
Poland
Puerto Rico
North Korea
Portugal
Paraguay
Palestine
Pol3 Coverage
Socio-demographic Index
100.0%
90.0%
80.0%
70.0%
60.0%
50.0%
40.0%
30.0%
20.0%
10.0%
0.0%
0
25
50
75
100
Year
2019
2010
2000
1990
1980

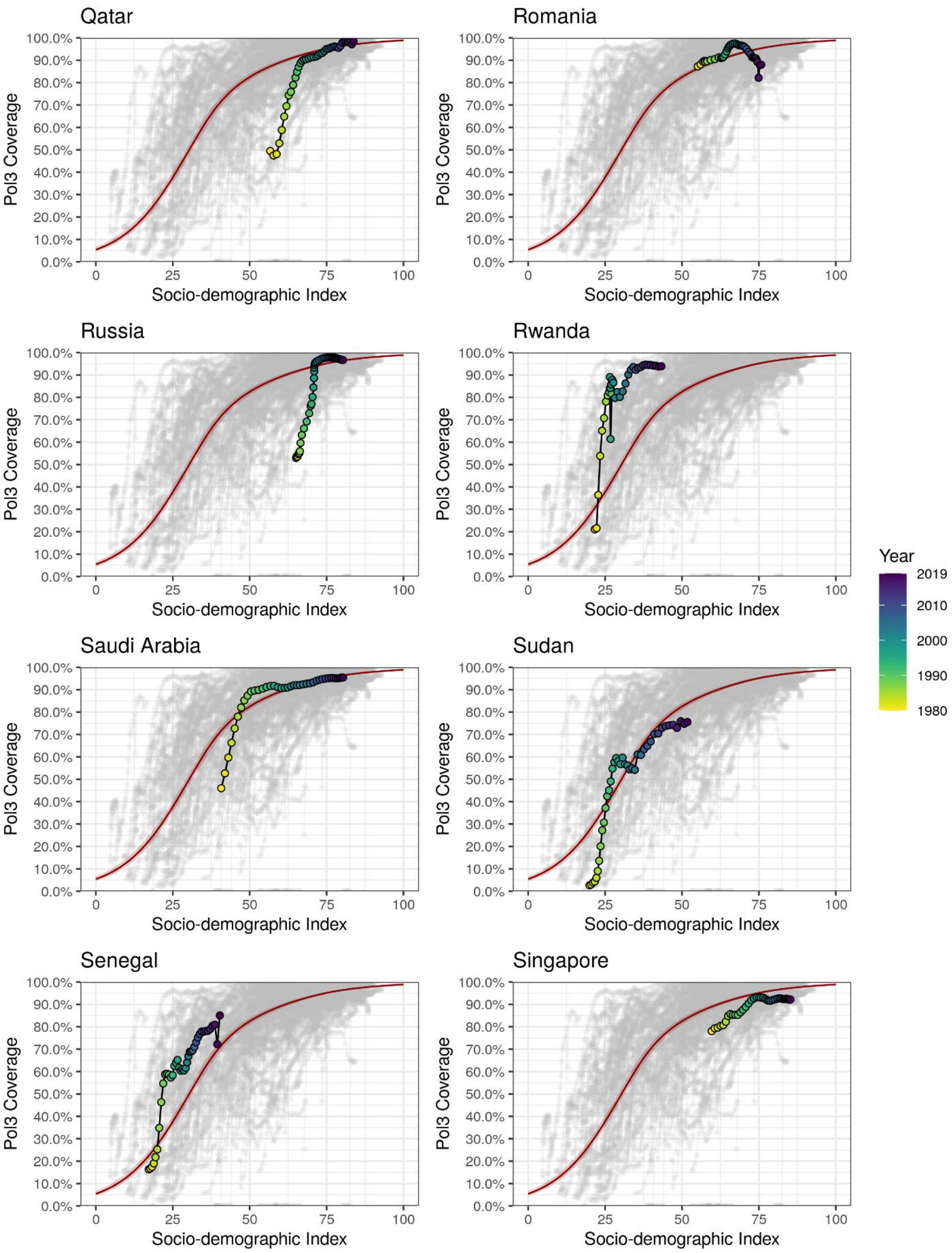
Qatar
Romania
Russia
Rwanda
Saudi Arabia
Sudan
Senegal
Singapore
Pol3 Coverage
Socio-demographic Index
0.0%
10.0%
20.0%
30.0%
40.0%
50.0%
60.0%
70.0%
80.0%
90.0%
100.0%
0
25
50
75
100
Year
2019
2010
2000
1990
1980

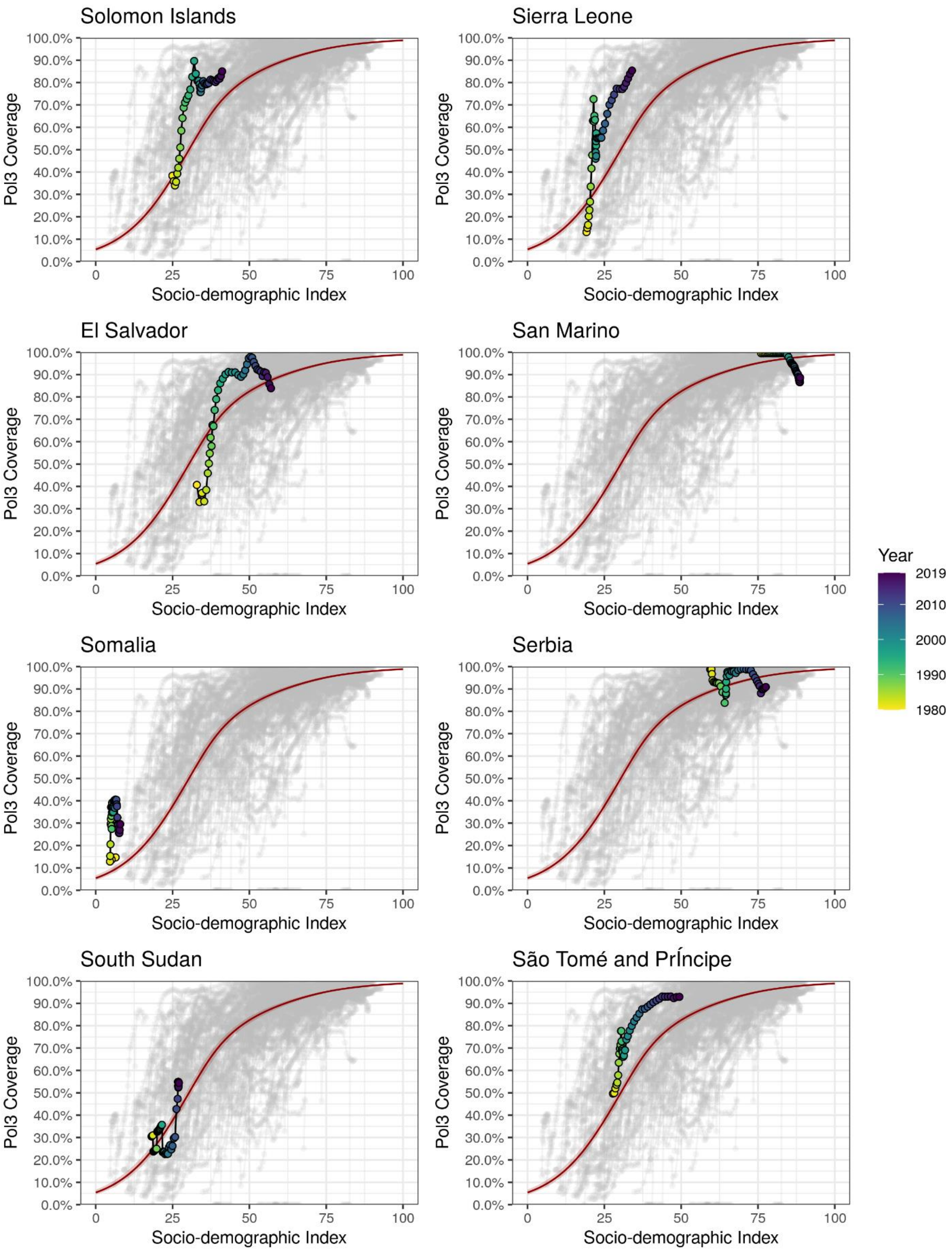
Solomon Islands
Sierra Leone
El Salvador
San Marino
Somalia
Serbia
South Sudan
São Tomé and PrÍncipe
Pol3 Coverage
Socio-demographic Index
100.0%
90.0%
80.0%
70.0%
60.0%
50.0%
40.0%
30.0%
20.0%
10.0%
0.0%
0
25
50
75
100
Year
2019
2010
2000
1990
1980

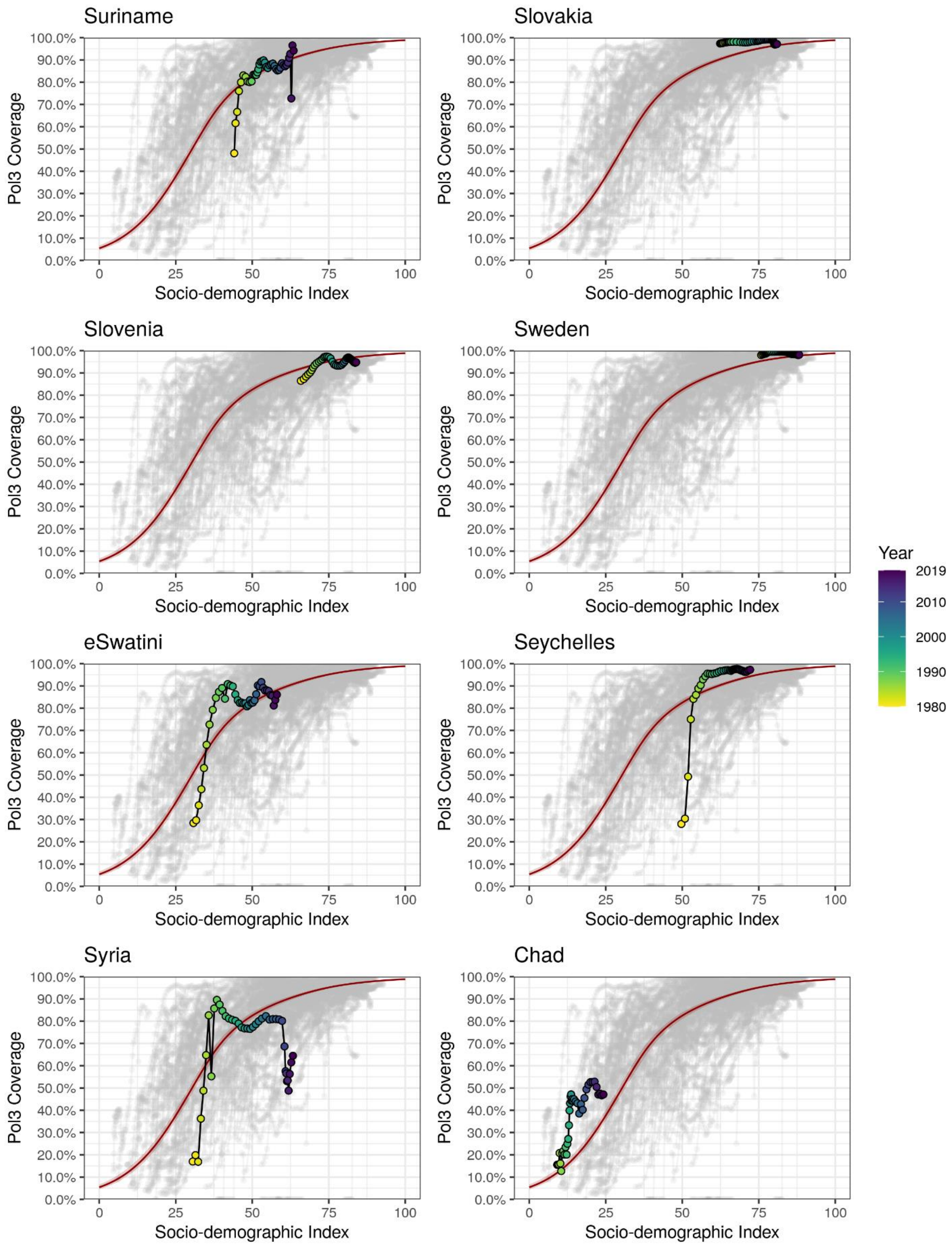
Suriname
Slovakia
Slovenia
Sweden
eSwatini
Seychelles
Syria
Chad
Pol3 Coverage
Socio-demographic Index
100.0%
90.0%
80.0%
70.0%
60.0%
50.0%
40.0%
30.0%
20.0%
10.0%
0.0%
0
25
50
75
100
Year
2019
2010
2000
1990
1980

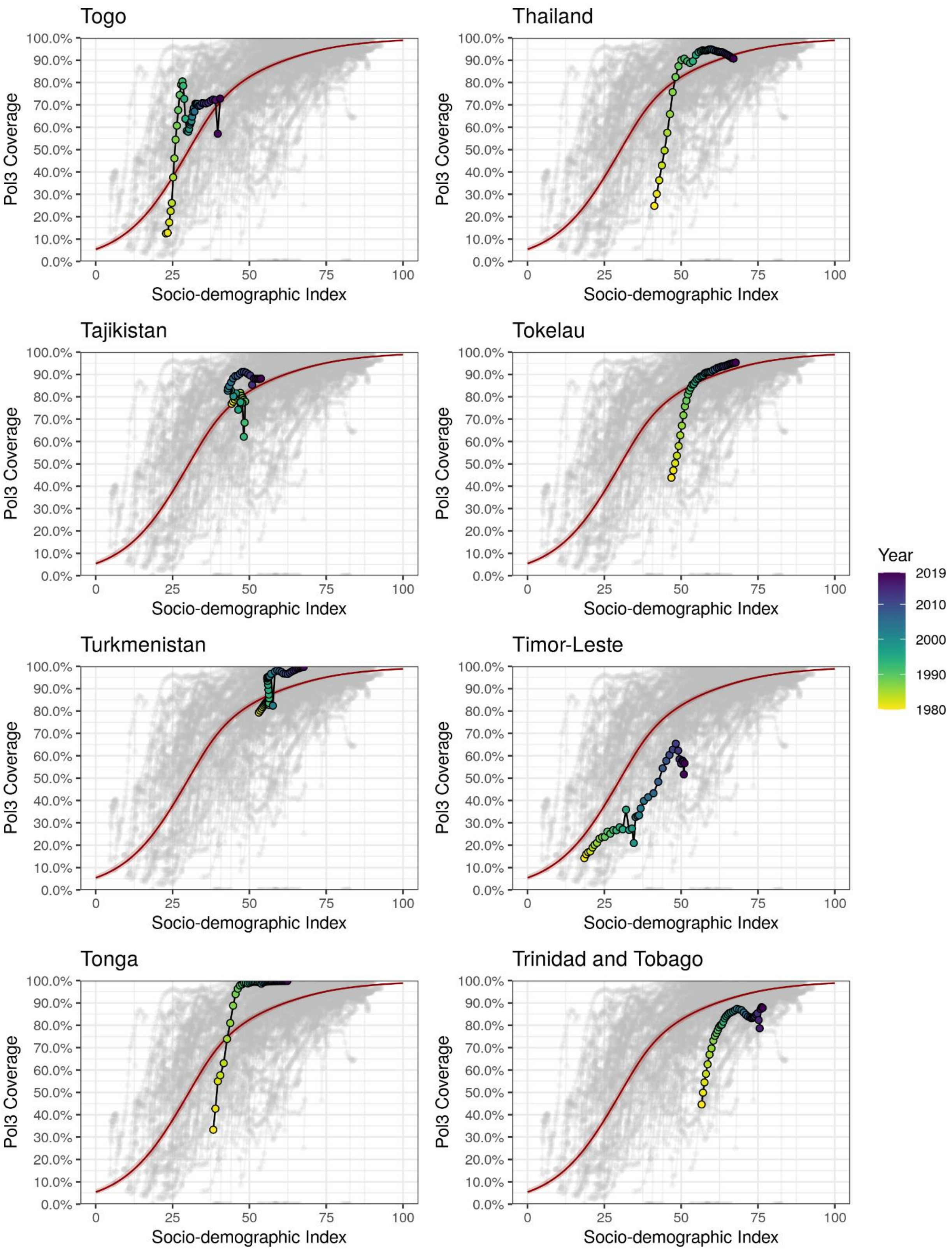
Togo
Thailand
Tajikistan
Tokelau
Turkmenistan
Timor-Leste
Tonga
Trinidad and Tobago
Pol3 Coverage
Socio-demographic Index
100.0%
90.0%
80.0%
70.0%
60.0%
50.0%
40.0%
30.0%
20.0%
10.0%
0.0%
0
25
50
75
100
Year
2019
2010
2000
1990
1980

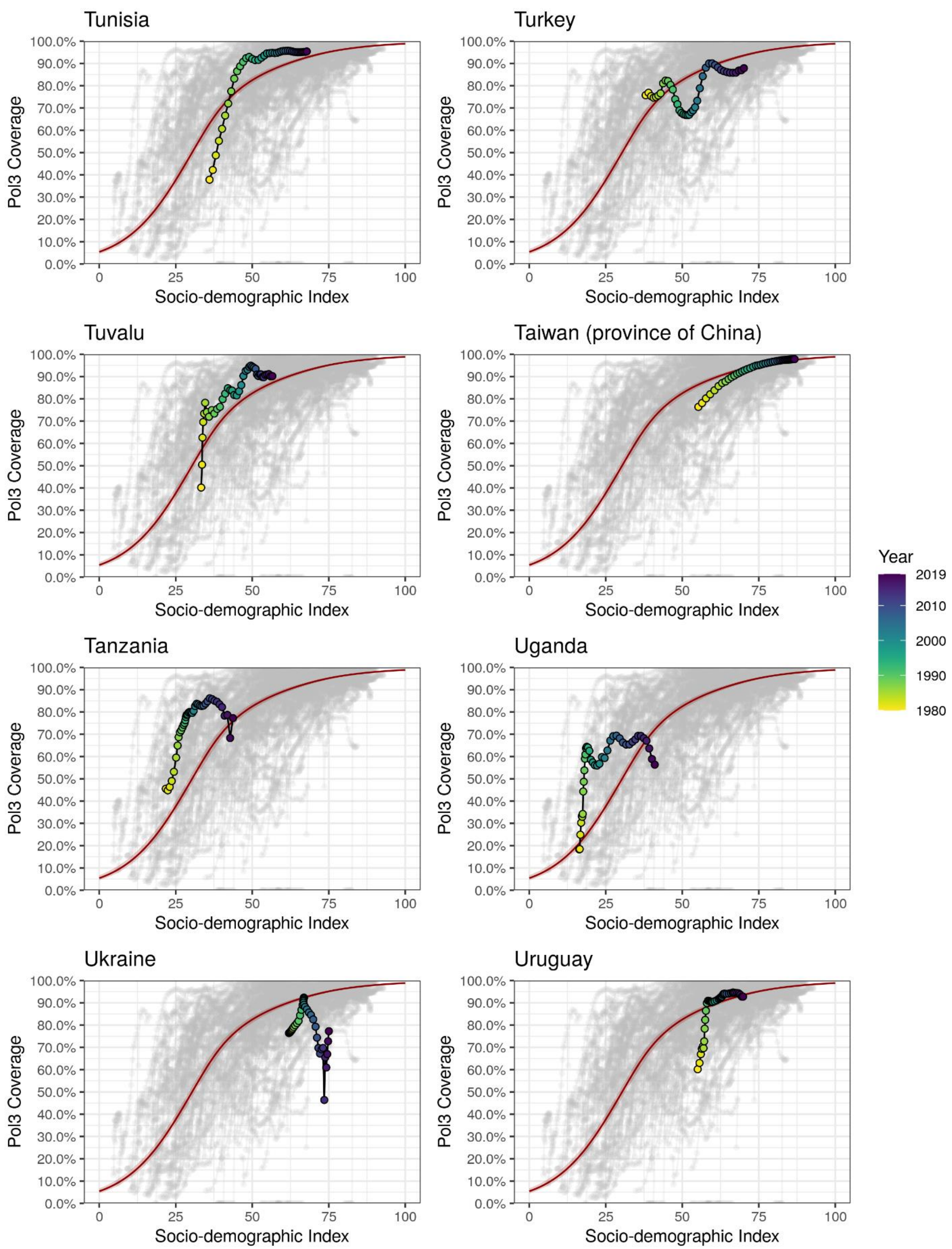
Tunisia
Turkey
Tuvalu
Taiwan (province of China)
Tanzania
Uganda
Ukraine
Uruguay
Pol3 Coverage
Socio-demographic Index
Year
2019
2010
2000
1990
1980

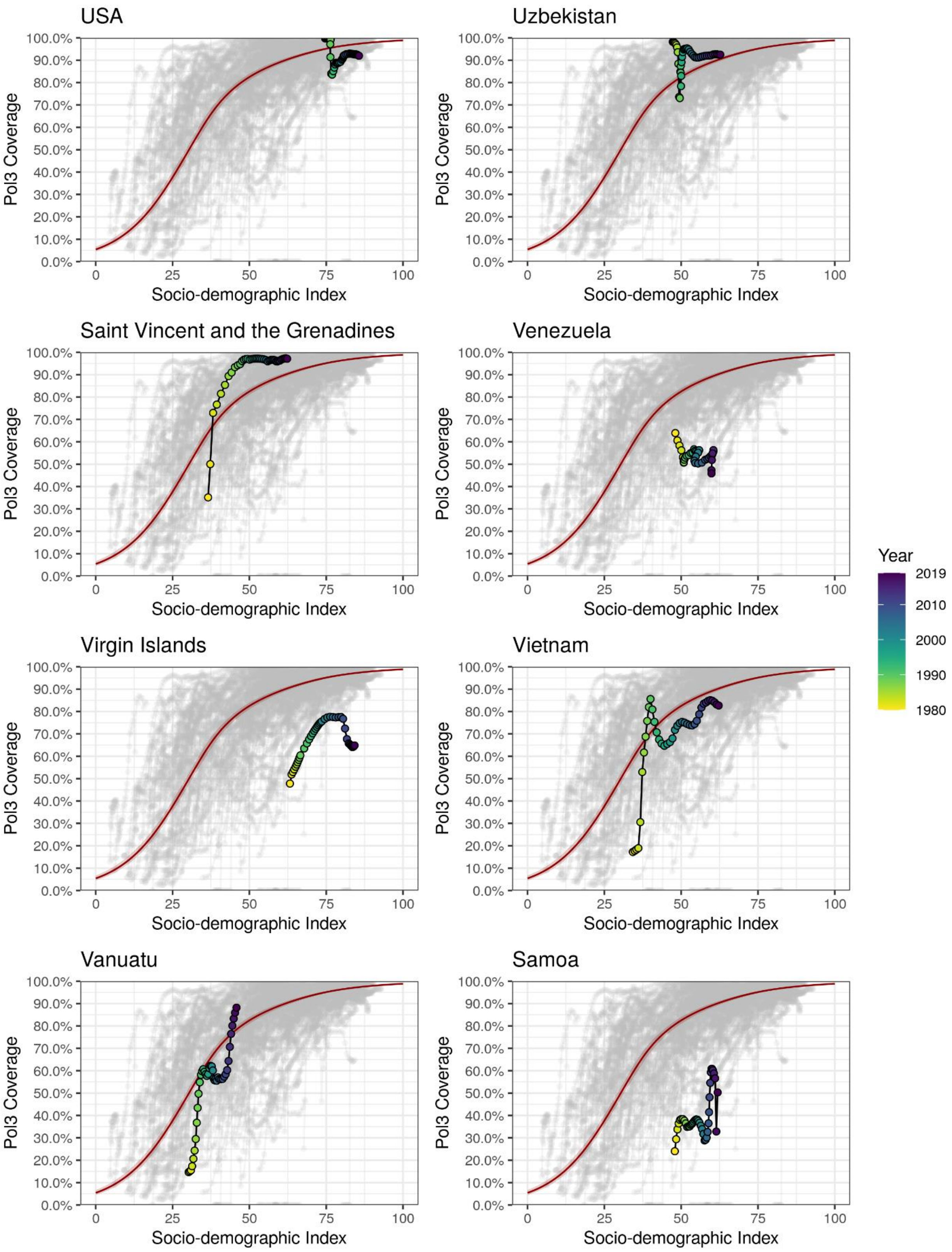
USA
Uzbekistan
Saint Vincent and the Grenadines
Venezuela
Virgin Islands
Vietnam
Vanuatu
Samoa
Pol3 Coverage
Socio-demographic Index
0.0%
10.0%
20.0%
30.0%
40.0%
50.0%
60.0%
70.0%
80.0%
90.0%
100.0%
0
25
50
75
100
Year
2019
2010
2000
1990
1980

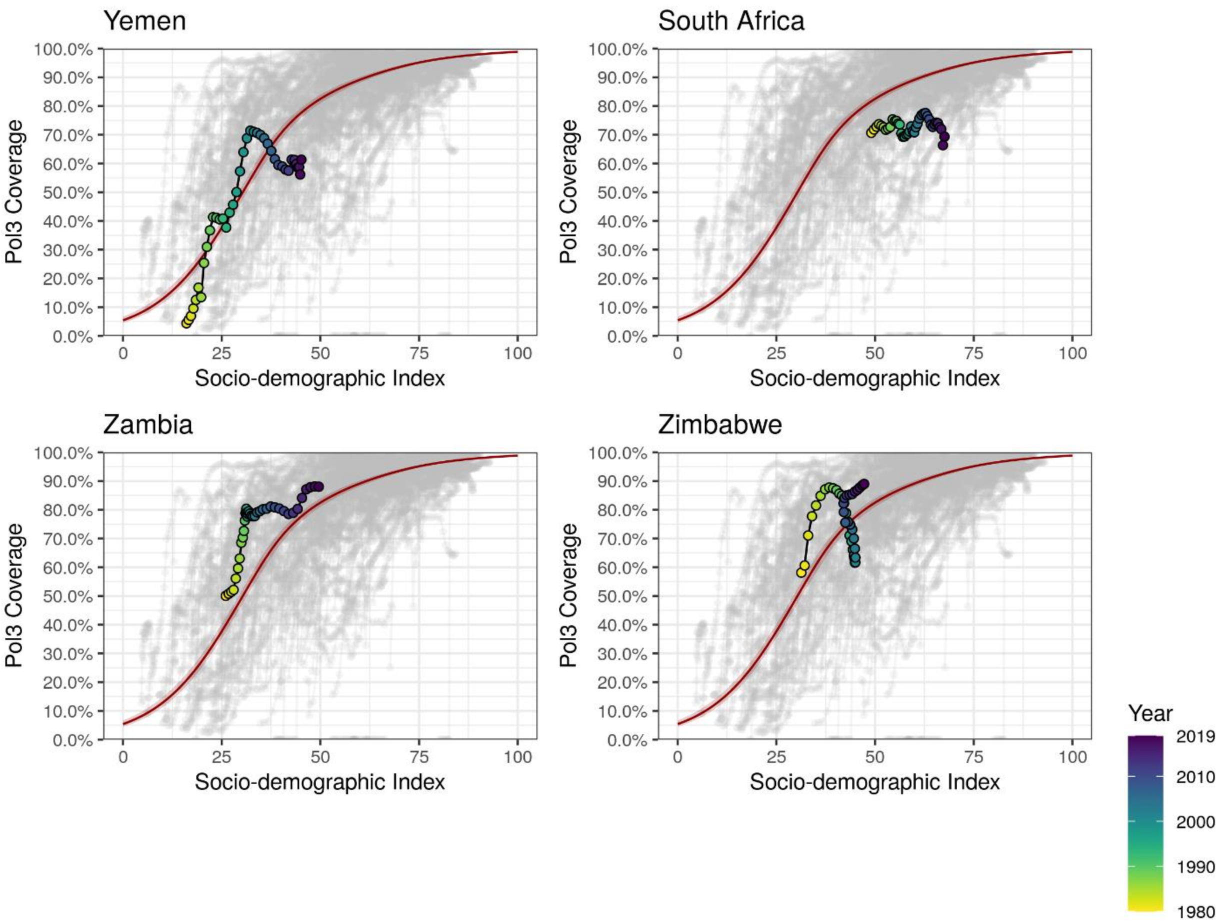
Yemen
South Africa
Zambia
Zimbabwe
Pol3 Coverage
Socio-demographic Index
100.0%
90.0%
80.0%
70.0%
60.0%
50.0%
40.0%
30.0%
20.0%
10.0%
0.0%
0
25
50
75
100
Year
2019
2010
2000
1990
1980

Supplementary figure 18. Global vaccination coverage in select years, BCG. Maps are ordered top to bottom chronologically by year, featuring estimates from 1980, 1990, 2000, 2010, and 2019, respectively. Countries with higher estimated coverage in each year are coloured in dark blue, and countries with lower estimated coverage are coloured in dark red. Locations where the vaccine is not nationally recommended or where estimates are not produced for GBD are coloured in grey. GBD=Global Burden of Disease. BCG=Bacillus Calmette-Guérin vaccine.

1980

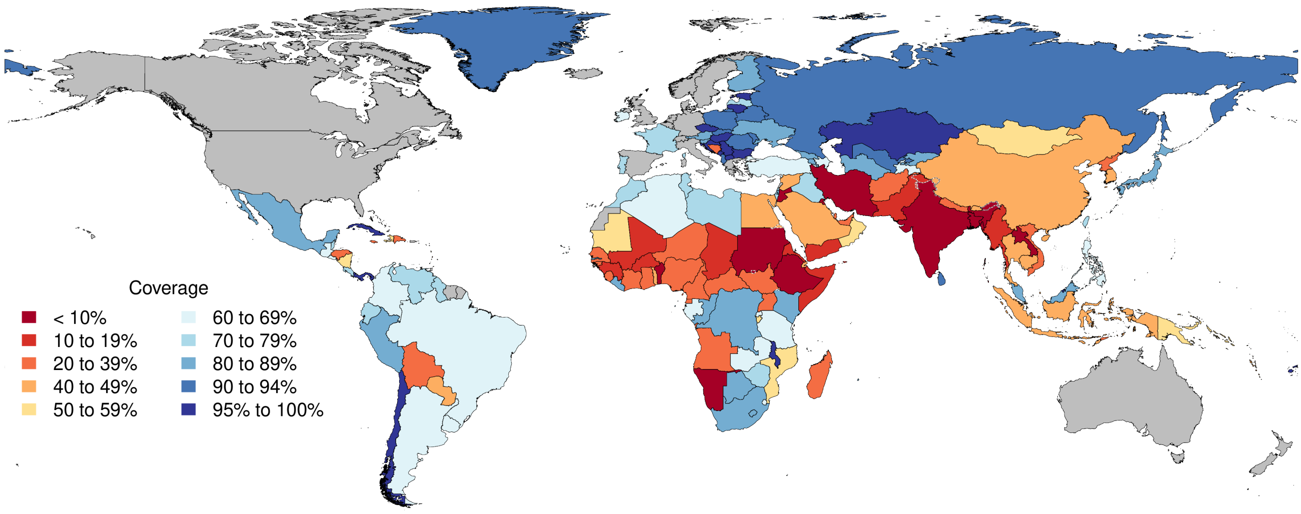

1990

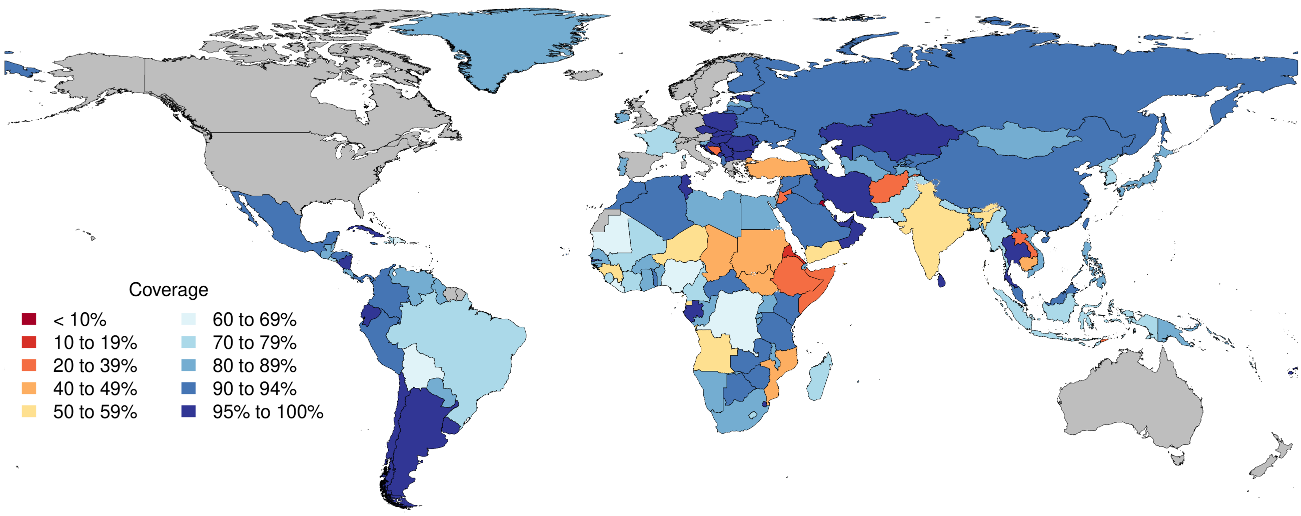

2000

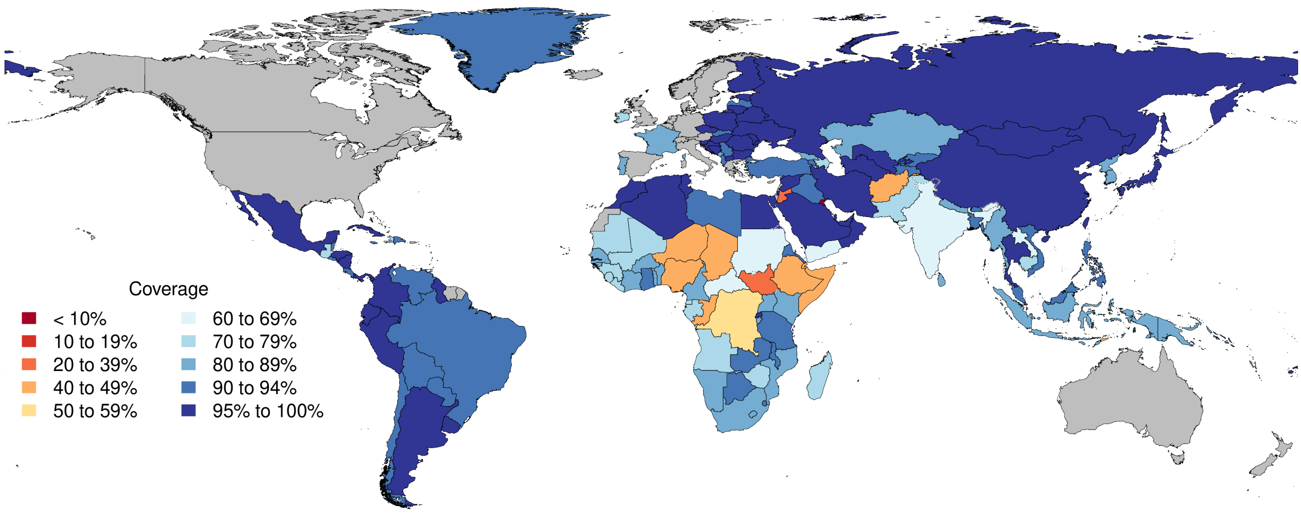

2010

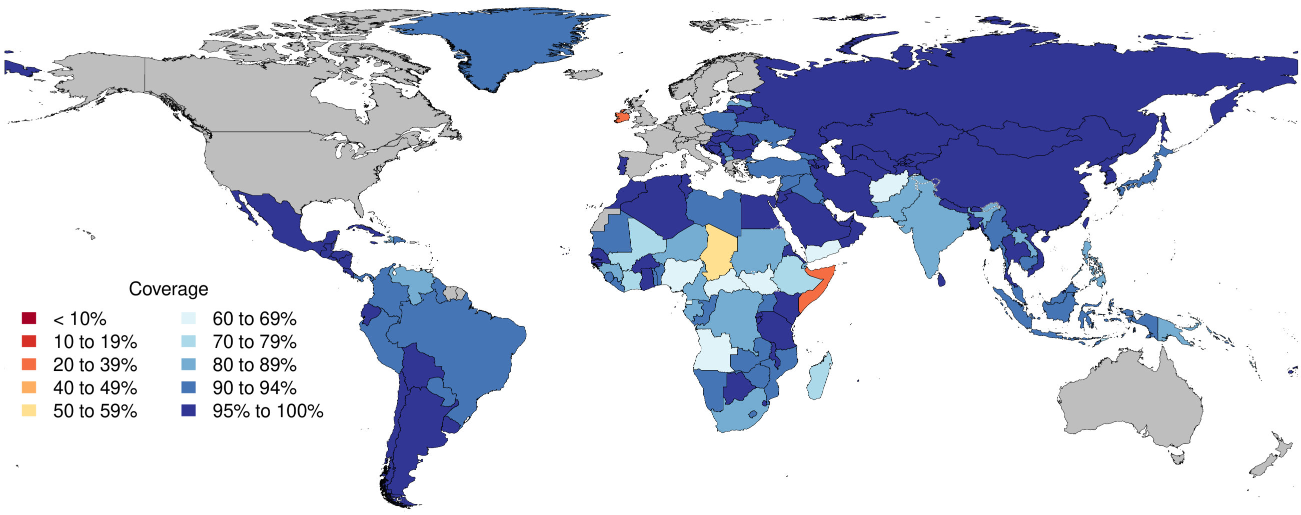

2019

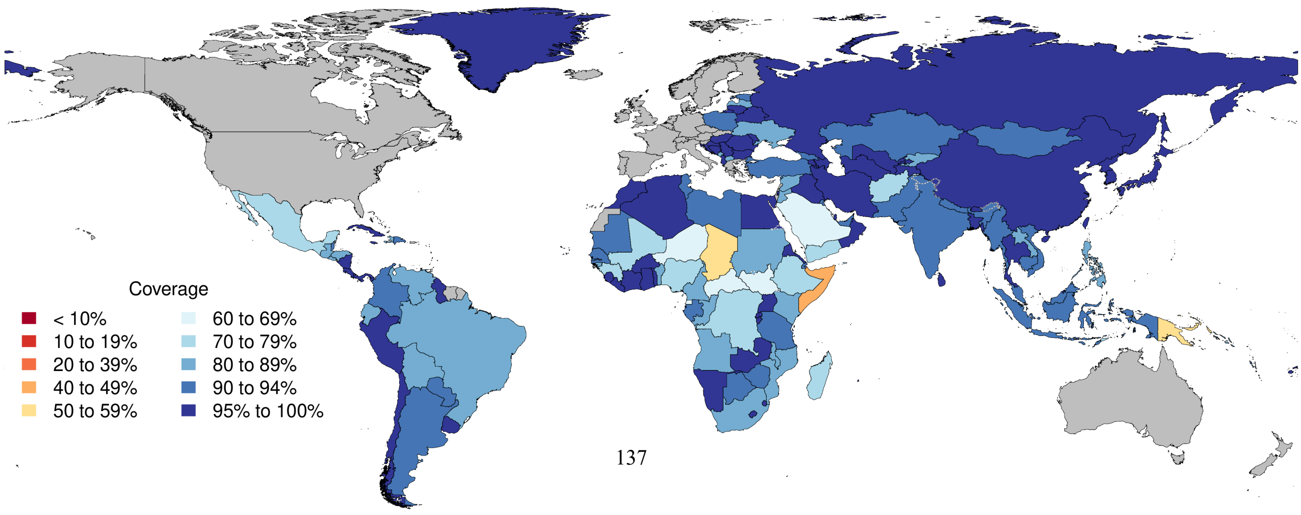

Supplementary figure 19. Global vaccination coverage in select years, DTP1. Maps are ordered top to bottom chronologically by year, featuring estimates from 1980, 1990, 2000, 2010, and 2019, respectively. Countries with higher estimated coverage in each year are coloured in dark blue, and countries with lower estimated coverage are coloured in dark red. Locations where the vaccine is not nationally recommended or where estimates are not produced for GBD are coloured in grey. GBD=Global Burden of Disease. DTP1=diphtheria-tetanus-pertussis, first dose.

1980

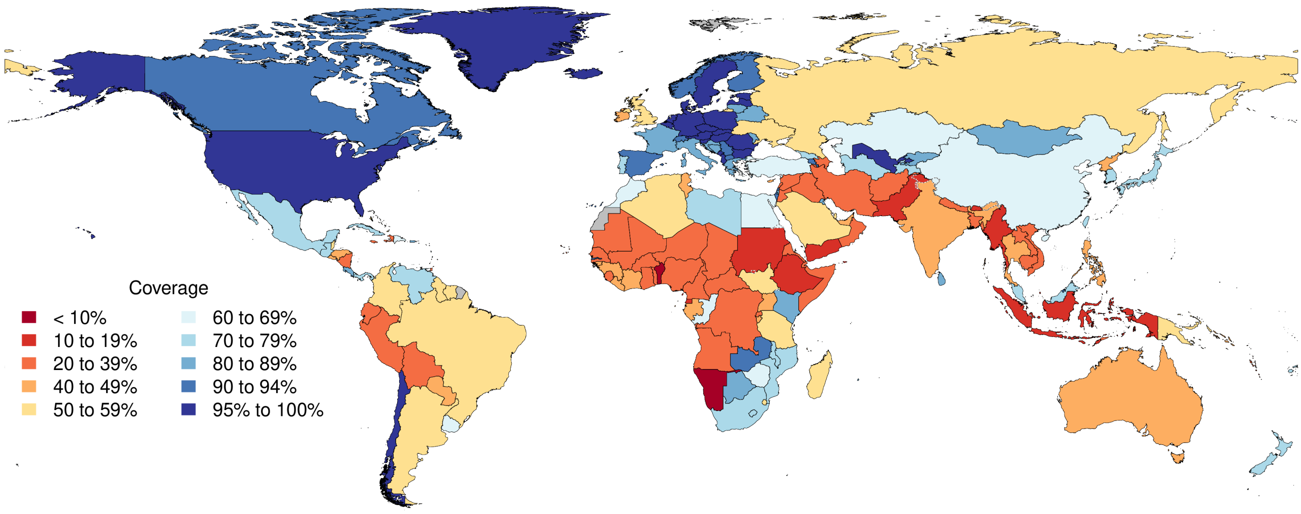

1990

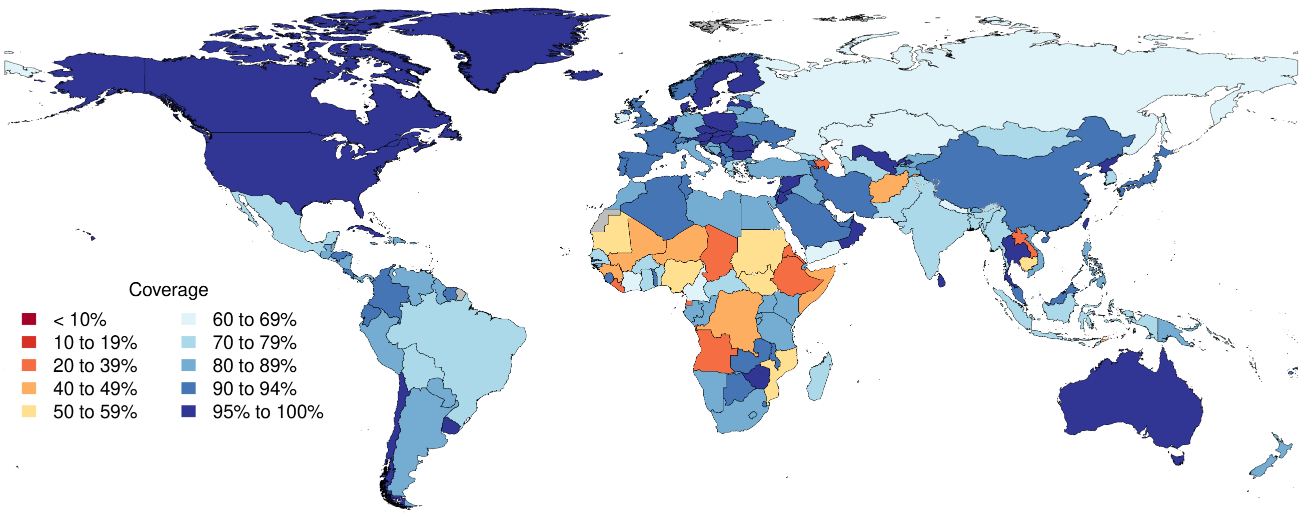

2000

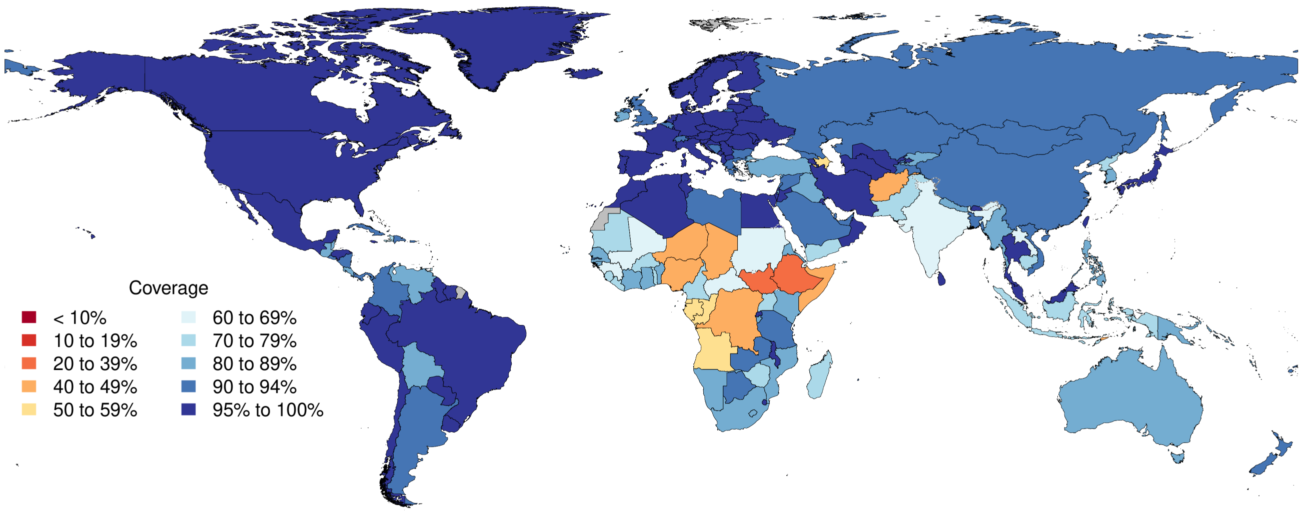

2010

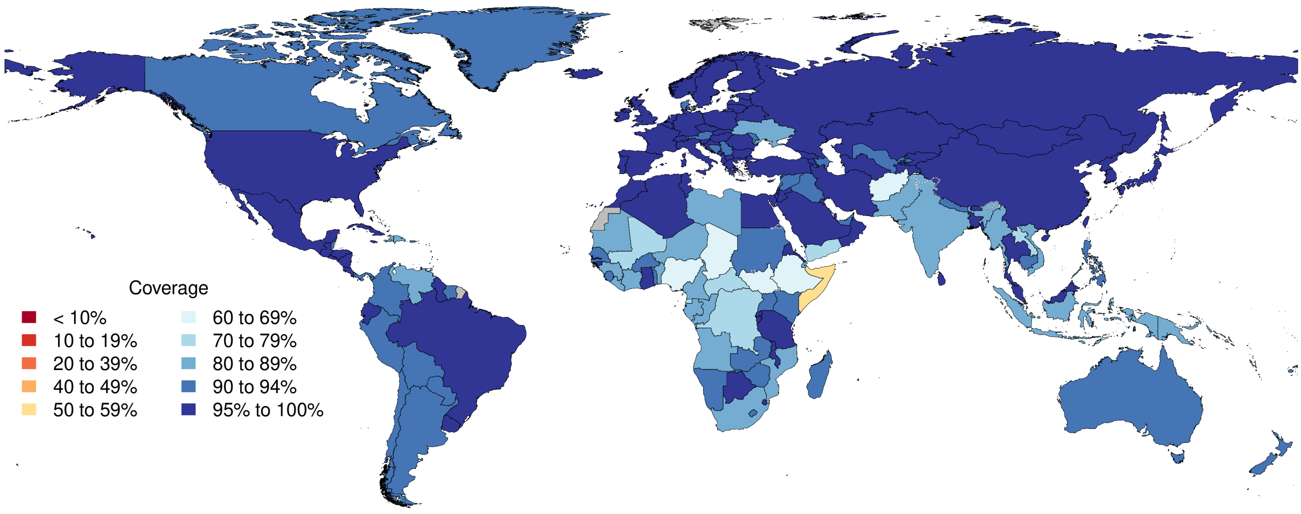

2019

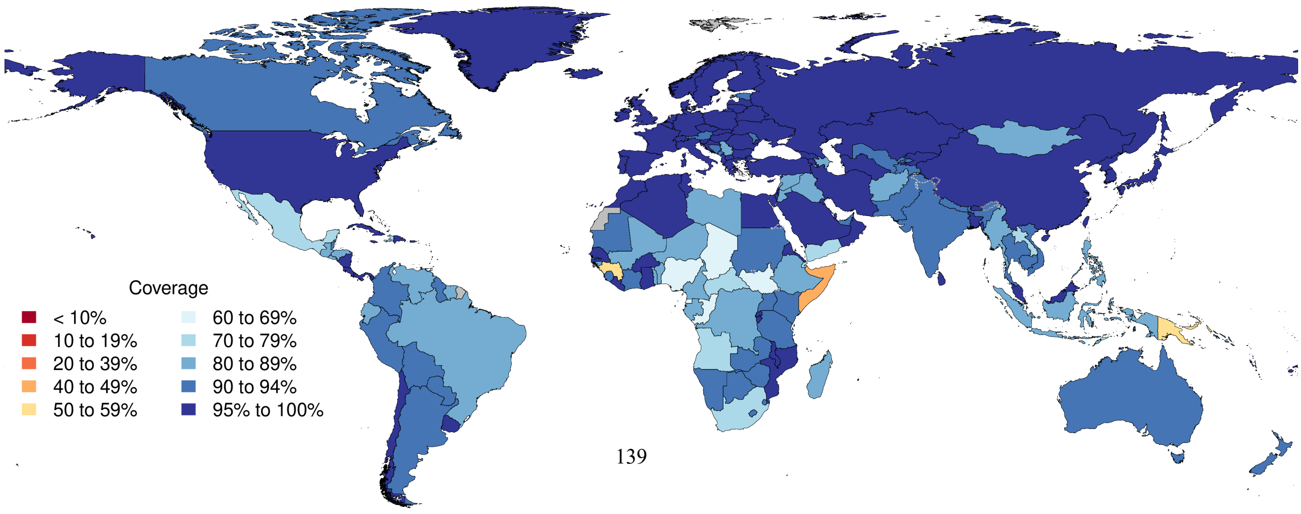

Supplementary figure 20. Global vaccination coverage in select years, DTP3. Maps are ordered top to bottom chronologically by year, featuring estimates from 1980, 1990, 2000, 2010, and 2019, respectively. Countries with higher estimated coverage in each year are coloured in dark blue, and countries with lower estimated coverage are coloured in dark red. Locations where the vaccine is not nationally recommended or where estimates are not produced for GBD are coloured in grey. GBD=Global Burden of Disease. DTP3=diphtheria-tetanus-pertussis, third dose.

1980

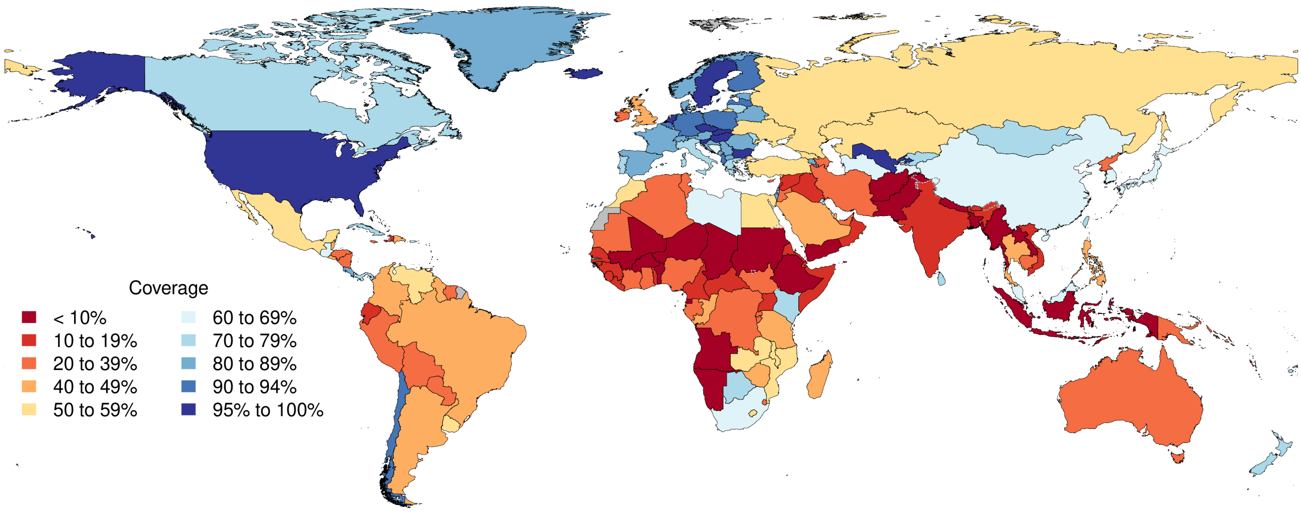

1990

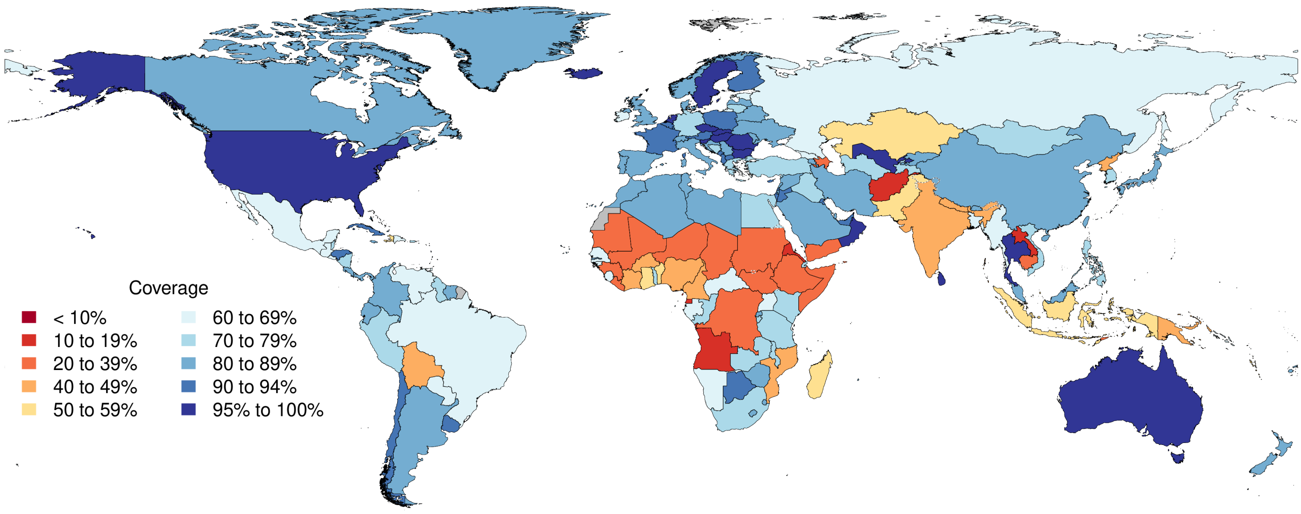

2000

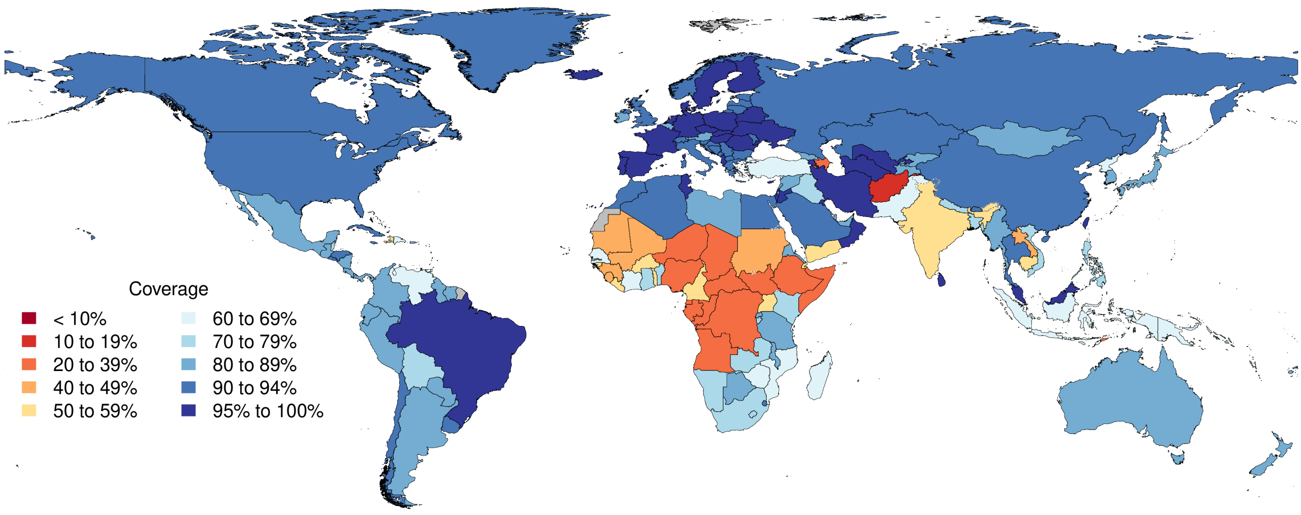

2010

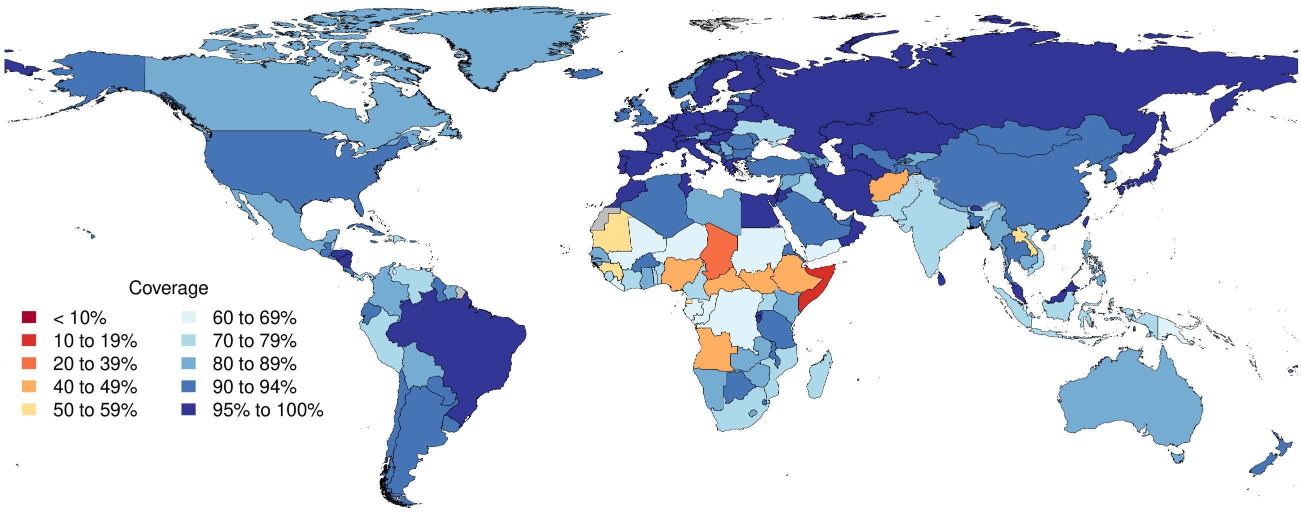

2019

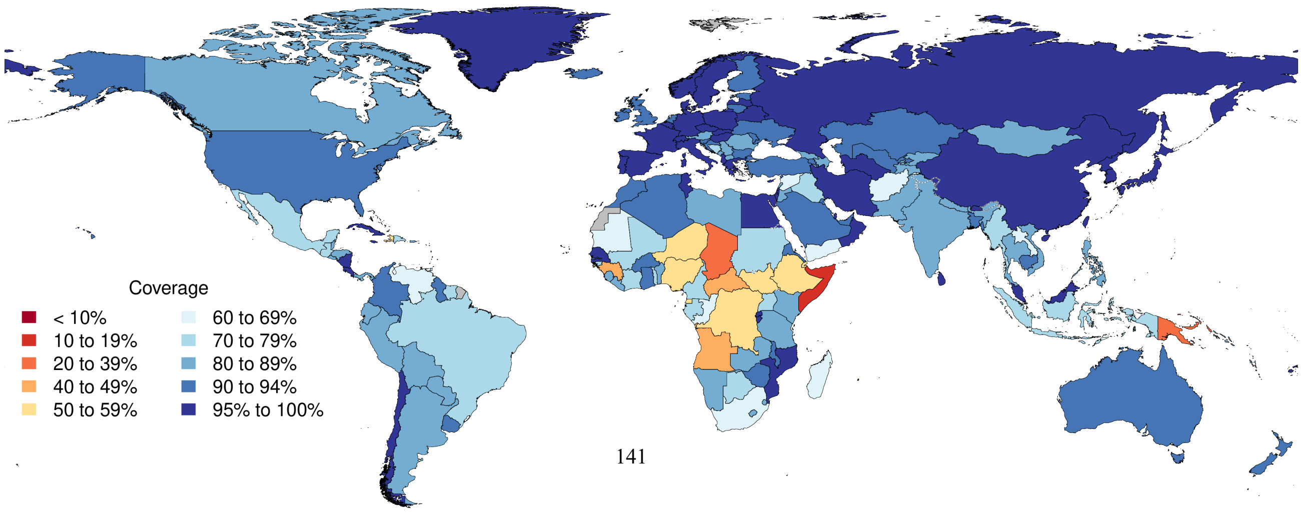

Supplementary figure 21. Global vaccination coverage in select years, HepB3. Maps are ordered top to bottom chronologically by year, featuring estimates from 1980, 1990, 2000, 2010, and 2019, respectively. Countries with higher estimated coverage in each year are coloured in dark blue, and countries with lower estimated coverage are coloured in dark red. Locations where the vaccine is not nationally recommended or where estimates are not produced for GBD are coloured in grey. GBD=Global Burden of Disease. HepB3=hepatitis B vaccine, third dose.

1980

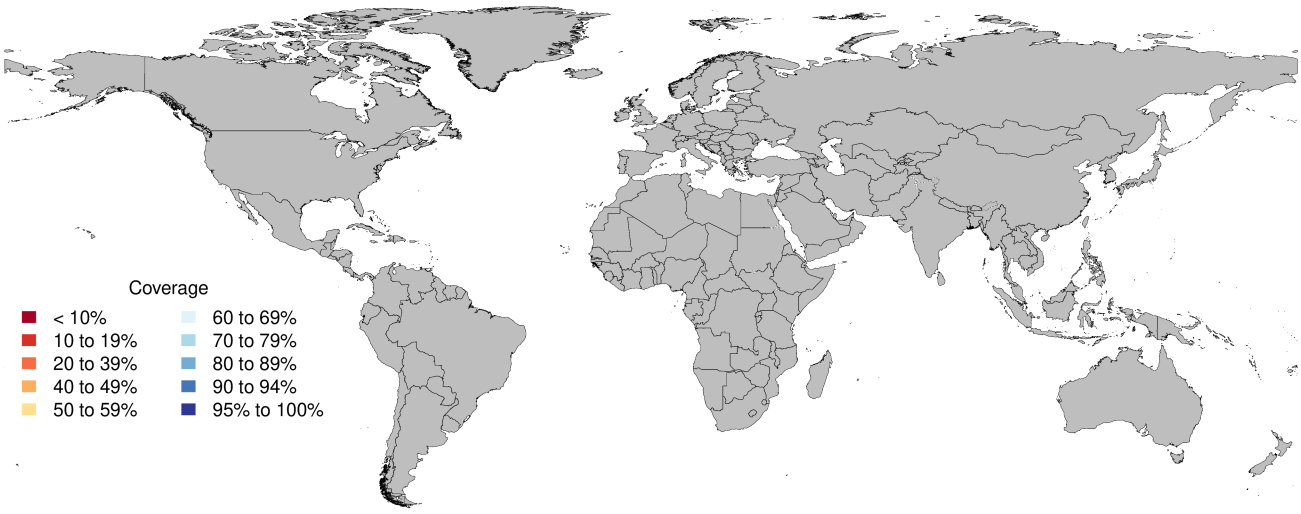

1990

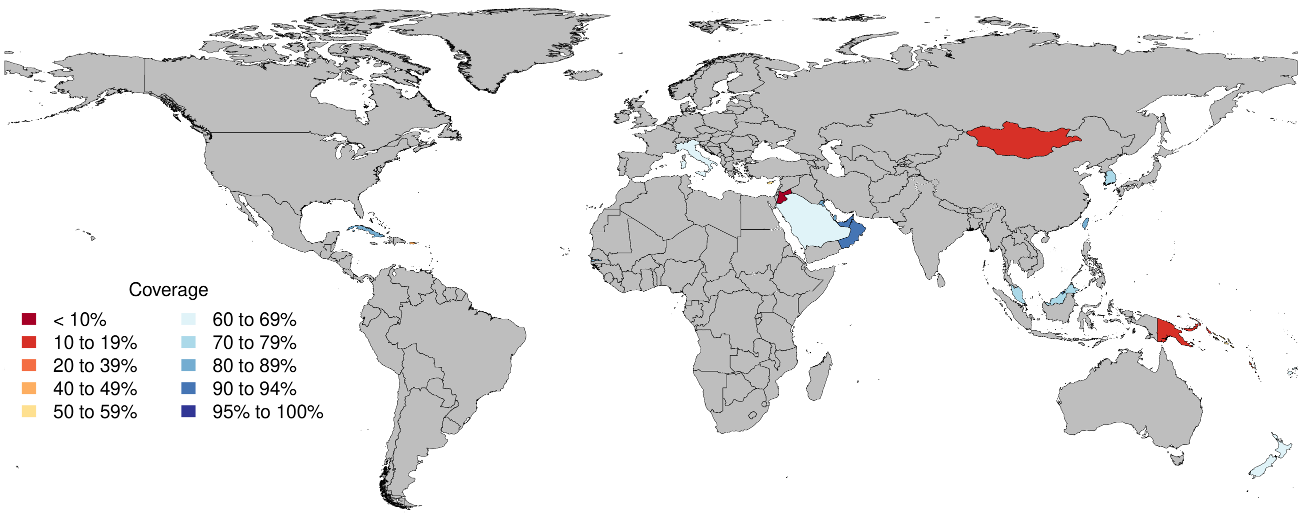

2000

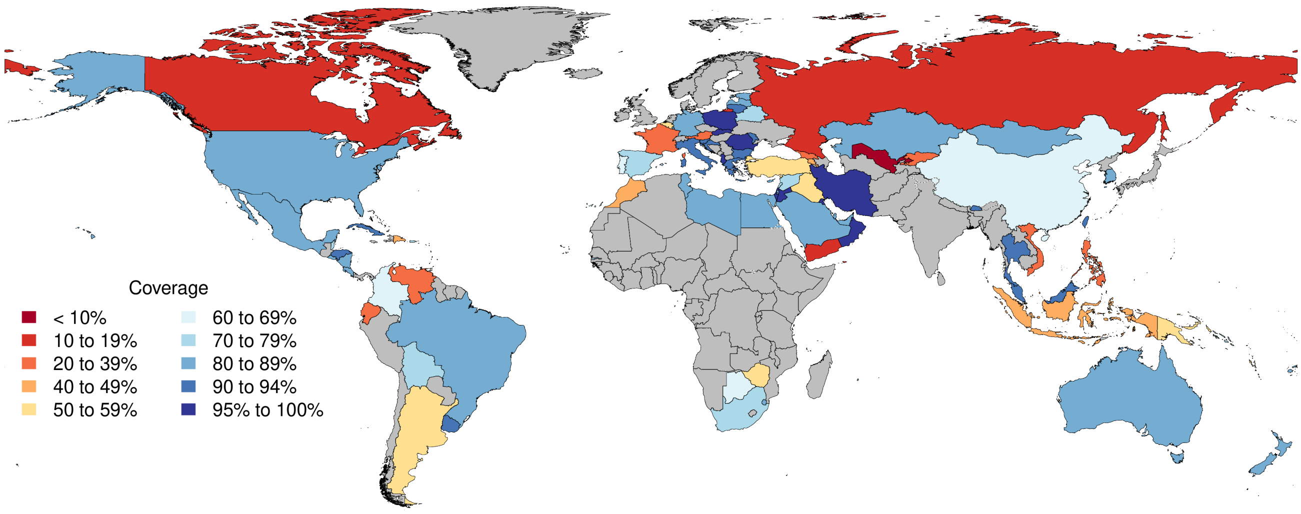

2010

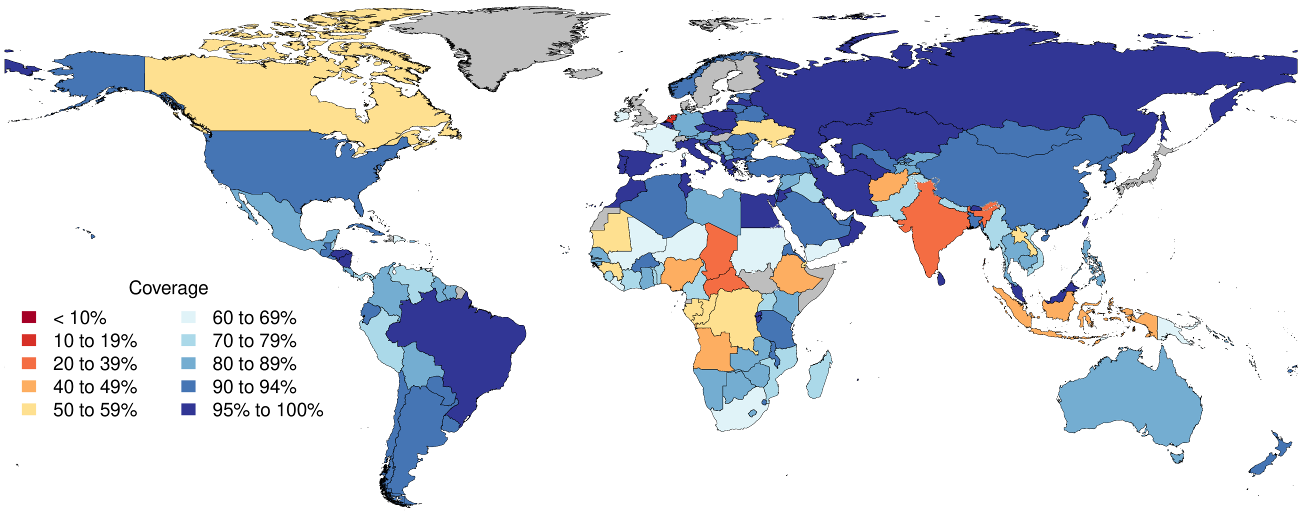

2019

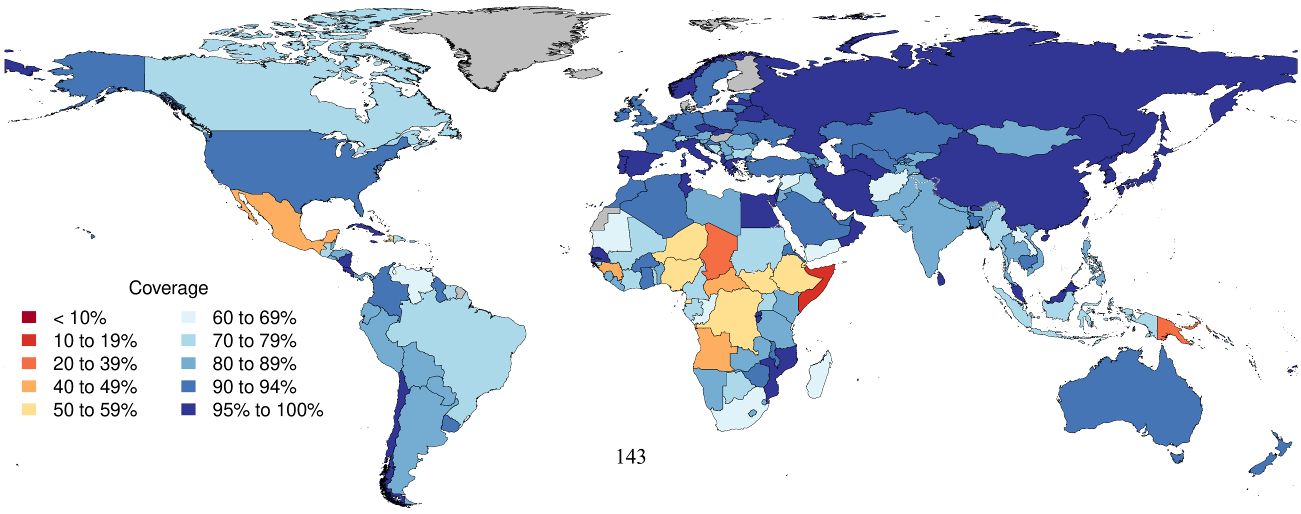

Supplementary figure 22. Global vaccination coverage in select years, Hib3. Maps are ordered top to bottom chronologically by year, featuring estimates from 1980, 1990, 2000, 2010, and 2019, respectively. Countries with higher estimated coverage in each year are coloured in dark blue, and countries with lower estimated coverage are coloured in dark red. Locations where the vaccine is not nationally recommended or where estimates are not produced for GBD are coloured in grey. GBD=Global Burden of Disease. Hib3=*Haemophilus influenzae* type b vaccine, third dose.

1980

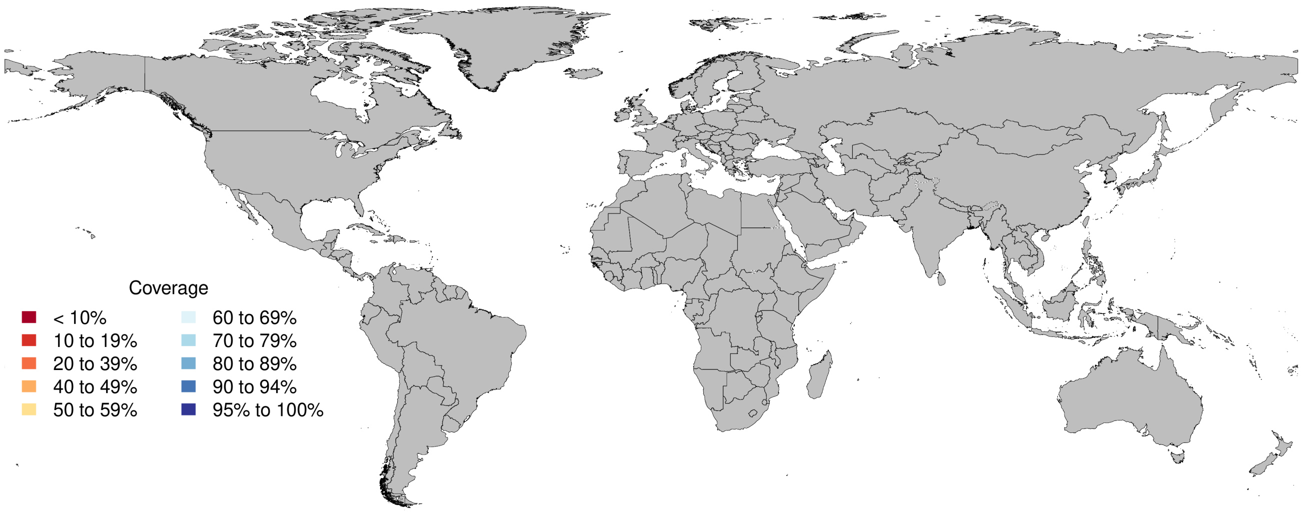

1990

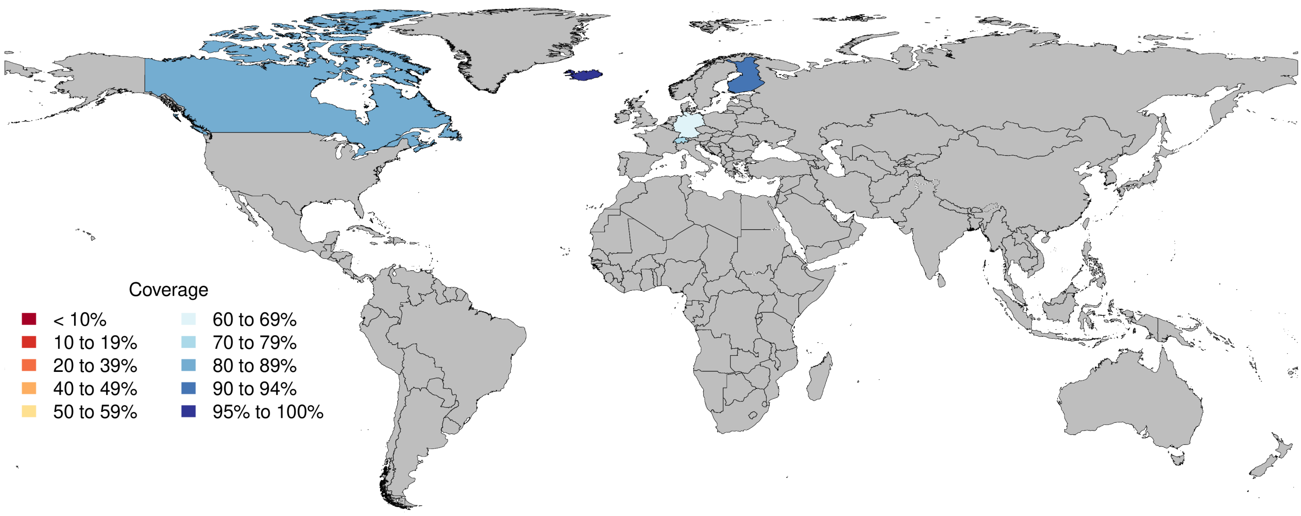

2000

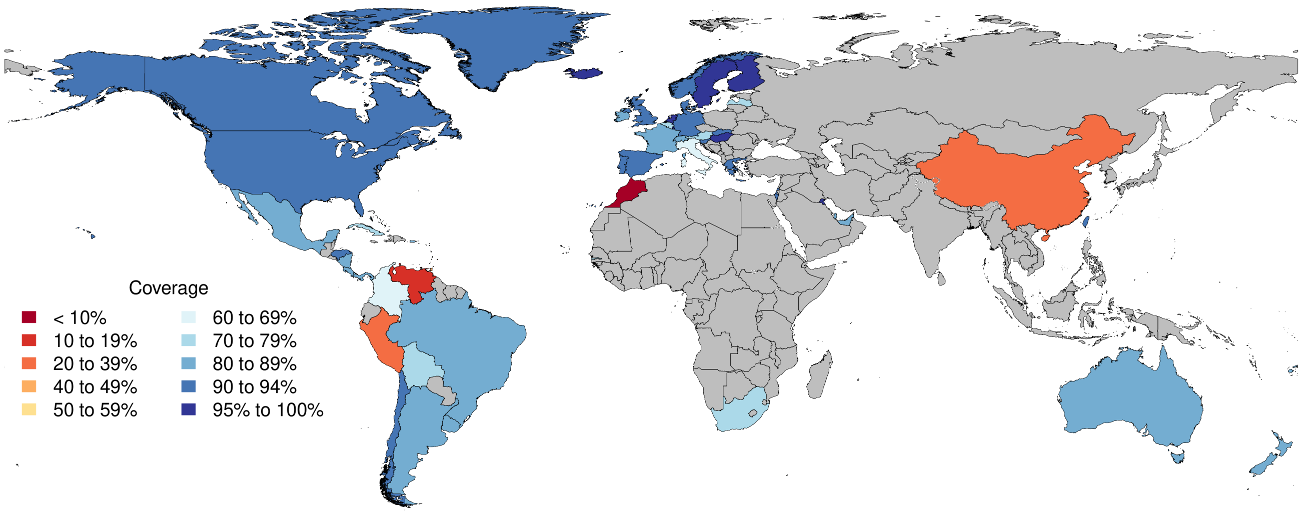

2010

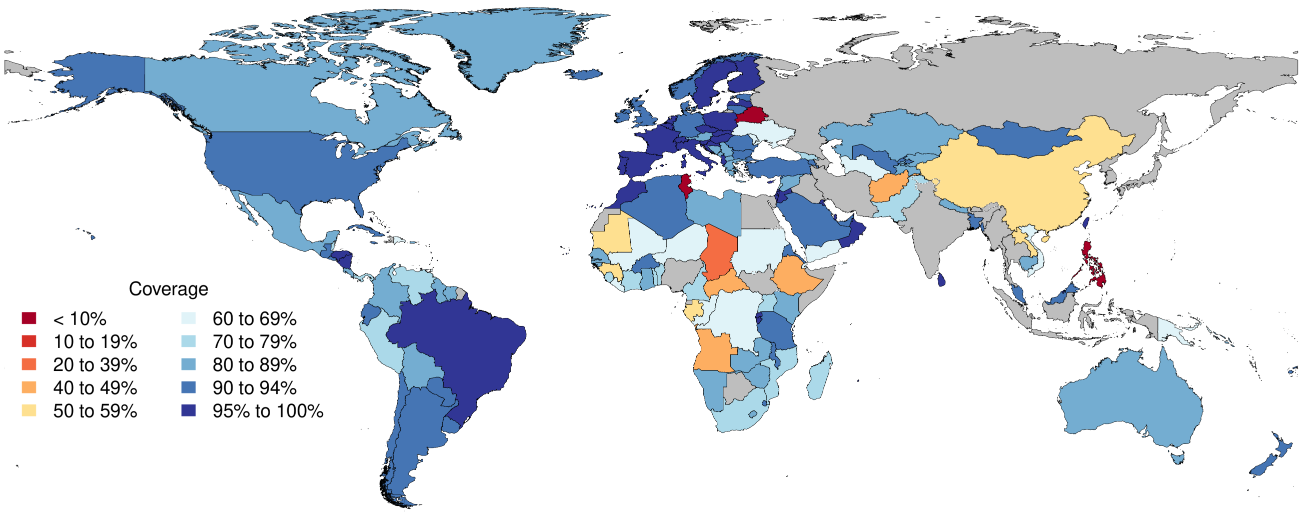

2019

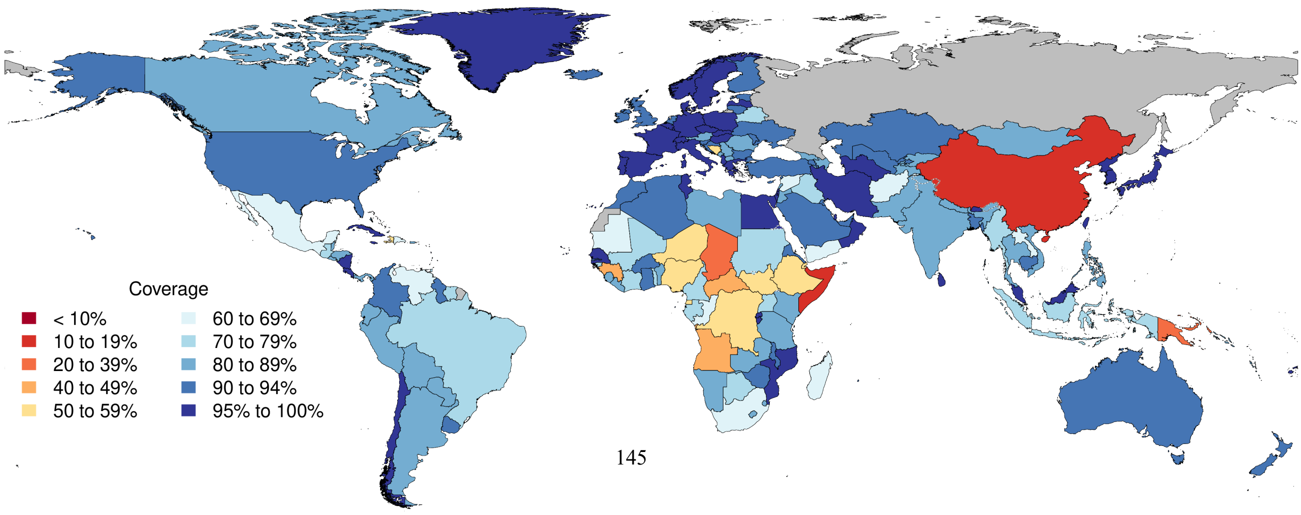

Supplementary figure 23. Global vaccination coverage in select years, MCV1. Maps are ordered top to bottom chronologically by year, featuring estimates from 1980, 1990, 2000, 2010, and 2019, respectively. Countries with higher estimated coverage in each year are coloured in dark blue, and countries with lower estimated coverage are coloured in dark red. Locations where the vaccine is not nationally recommended or where estimates are not produced for GBD are coloured in grey. GBD=Global Burden of Disease. MCV1=measles-containing vaccine, first dose.

1980

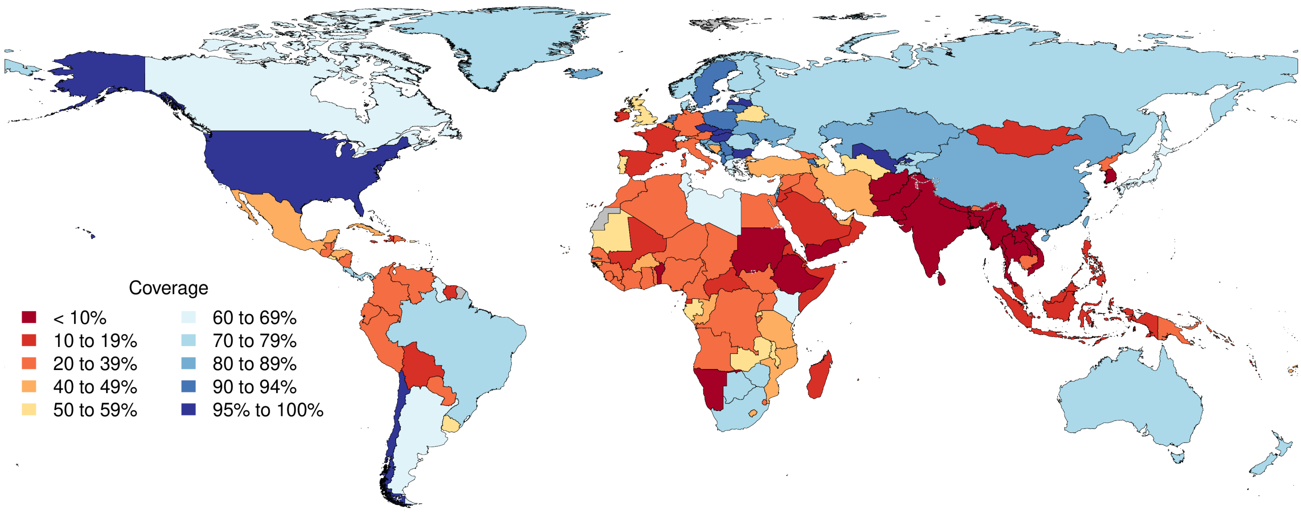

1990

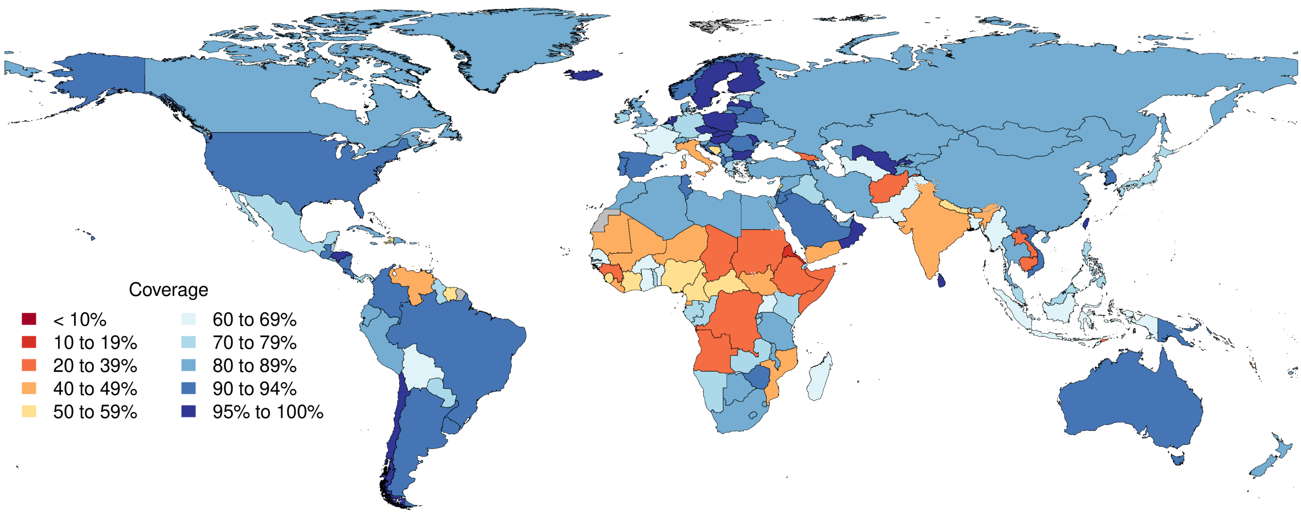

2000

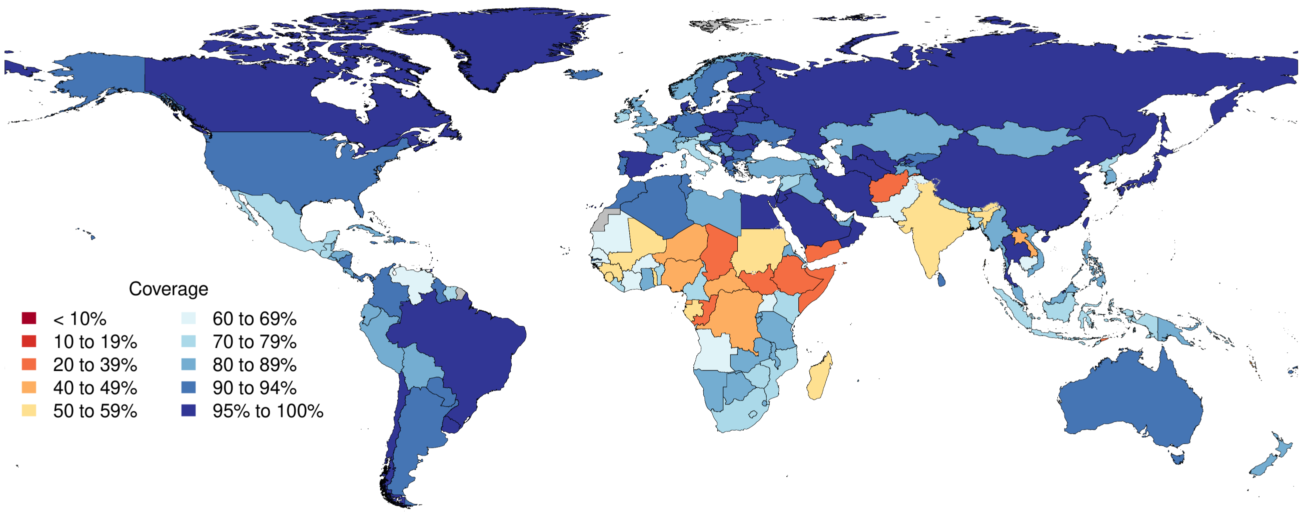

2010

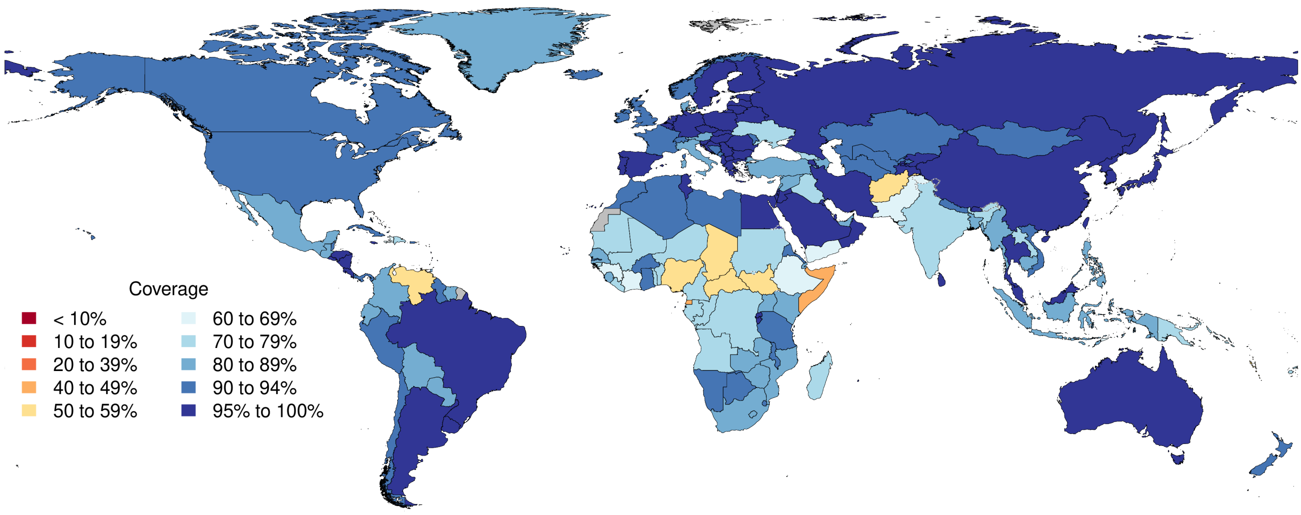

2019

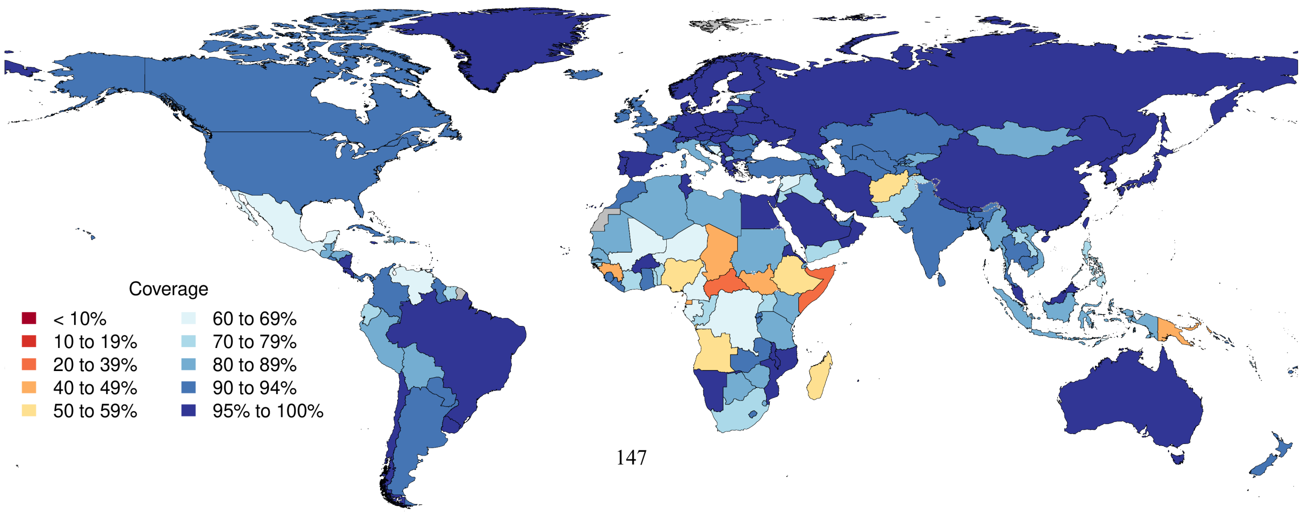

Supplementary figure 24. Global vaccination coverage in select years, MCV2. Maps are ordered top to bottom chronologically by year, featuring estimates from 1980, 1990, 2000, 2010, and 2019, respectively. Countries with higher estimated coverage in each year are coloured in dark blue, and countries with lower estimated coverage are coloured in dark red. Locations where the vaccine is not nationally recommended or where estimates are not produced for GBD are coloured in grey. GBD=Global Burden of Disease. MCV2=measles-containing vaccine, second dose.

1980

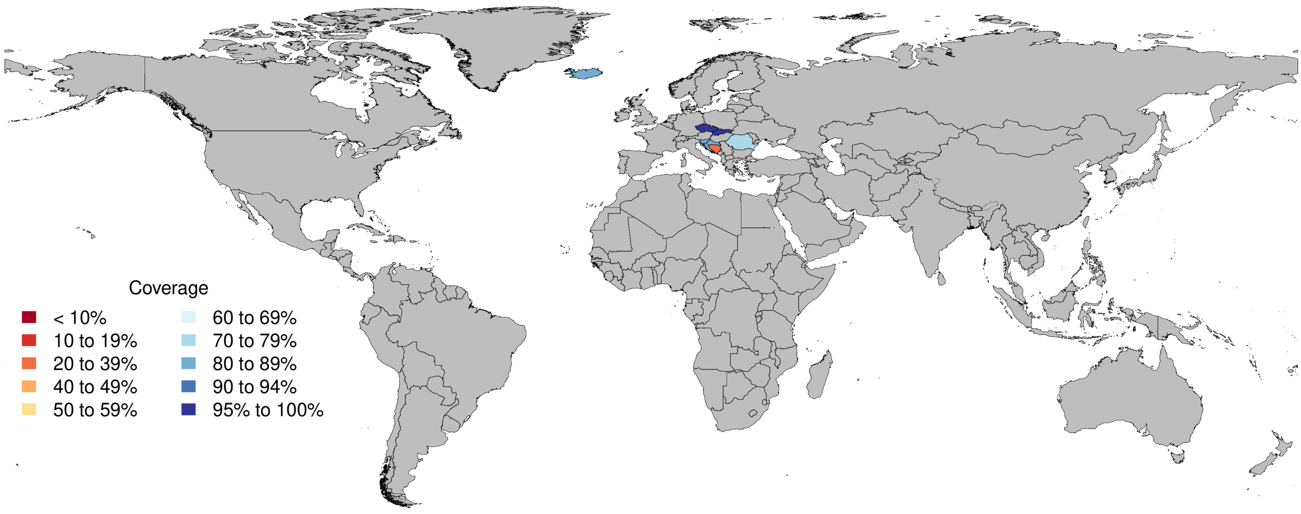

1990

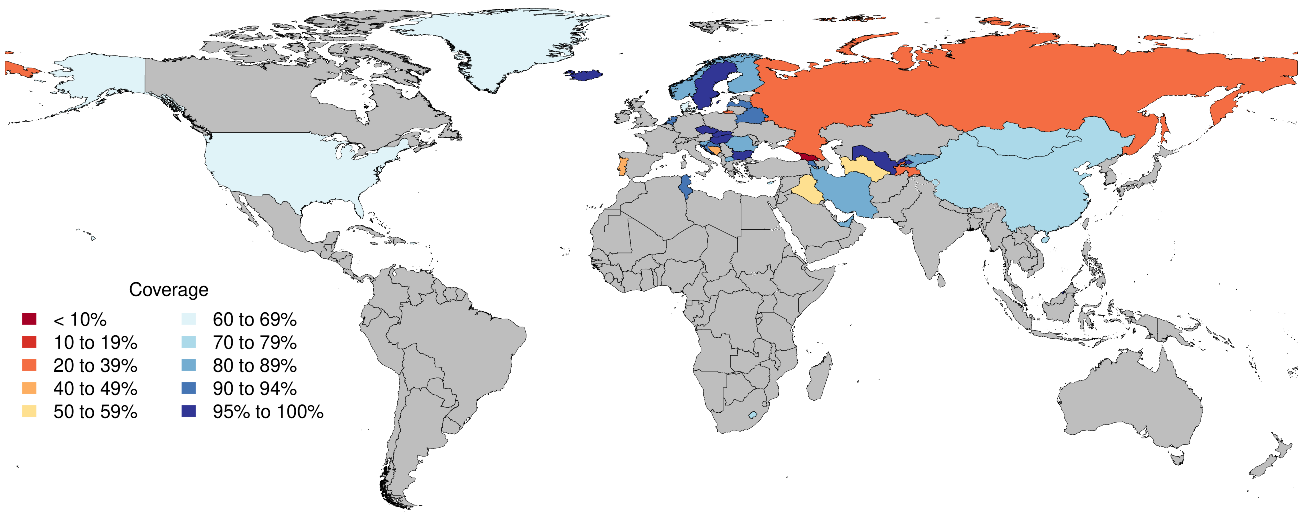

2000

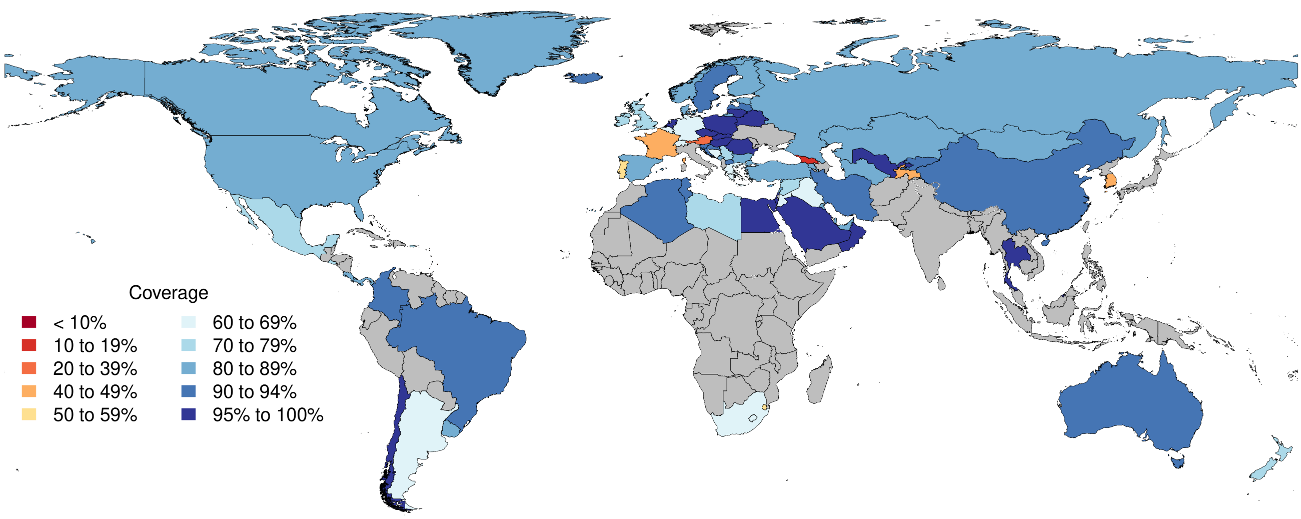

2010

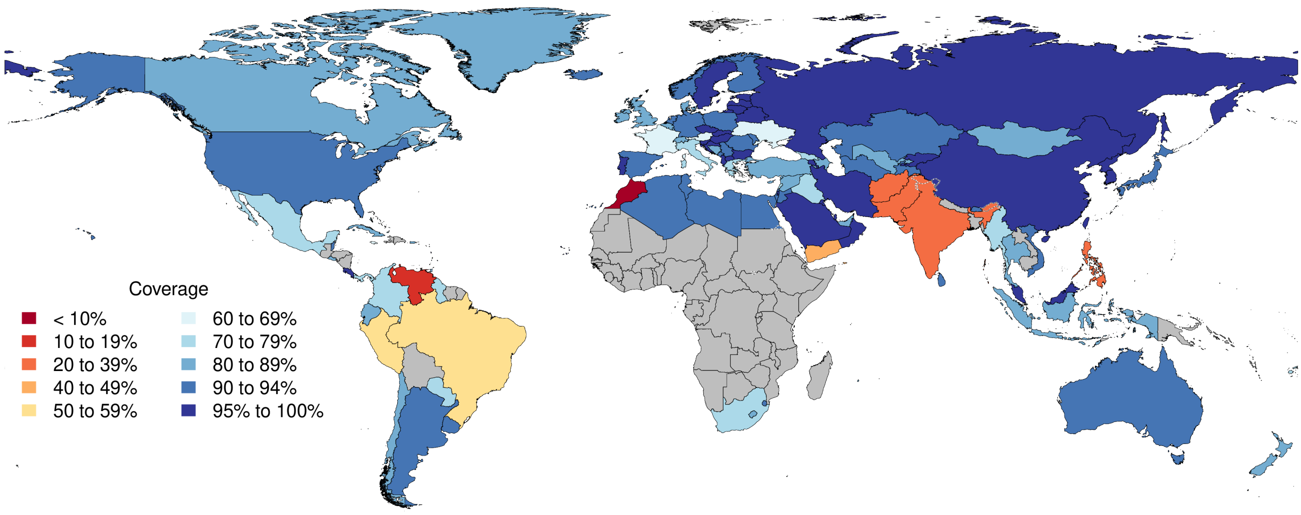

2019

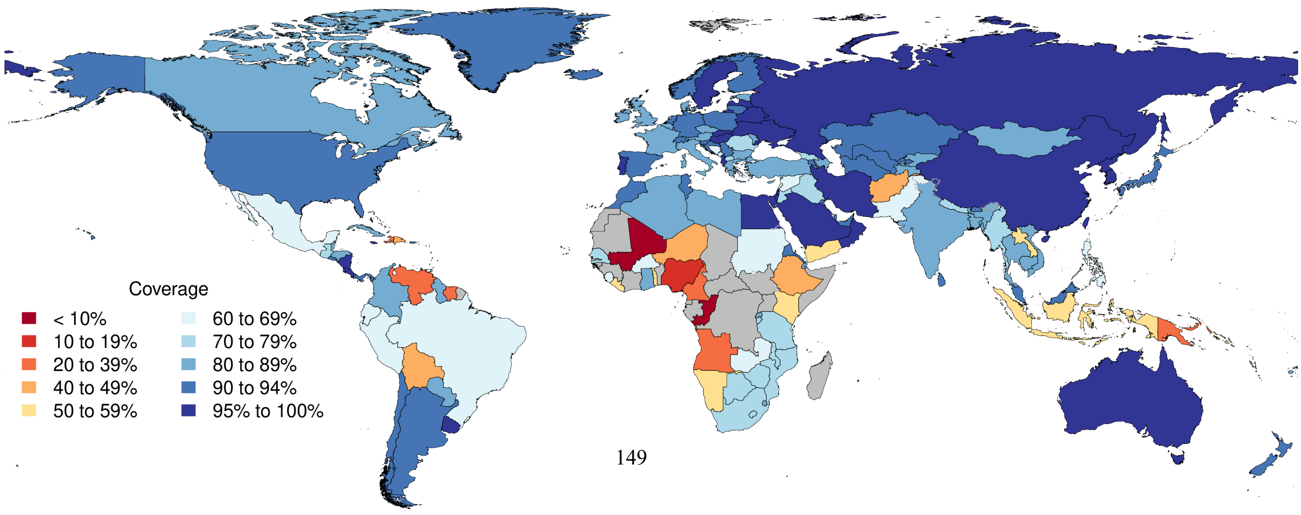

Supplementary figure 25. Global vaccination coverage in select years, PCV3. Maps are ordered top to bottom chronologically by year, featuring estimates from 1980, 1990, 2000, 2010, and 2019, respectively. Countries with higher estimated coverage in each year are coloured in dark blue, and countries with lower estimated coverage are coloured in dark red. Locations where the vaccine is not nationally recommended or where estimates are not produced for GBD are coloured in grey. GBD=Global Burden of Disease. PCV3=pneumococcal conjugate vaccine, third dose.

1980

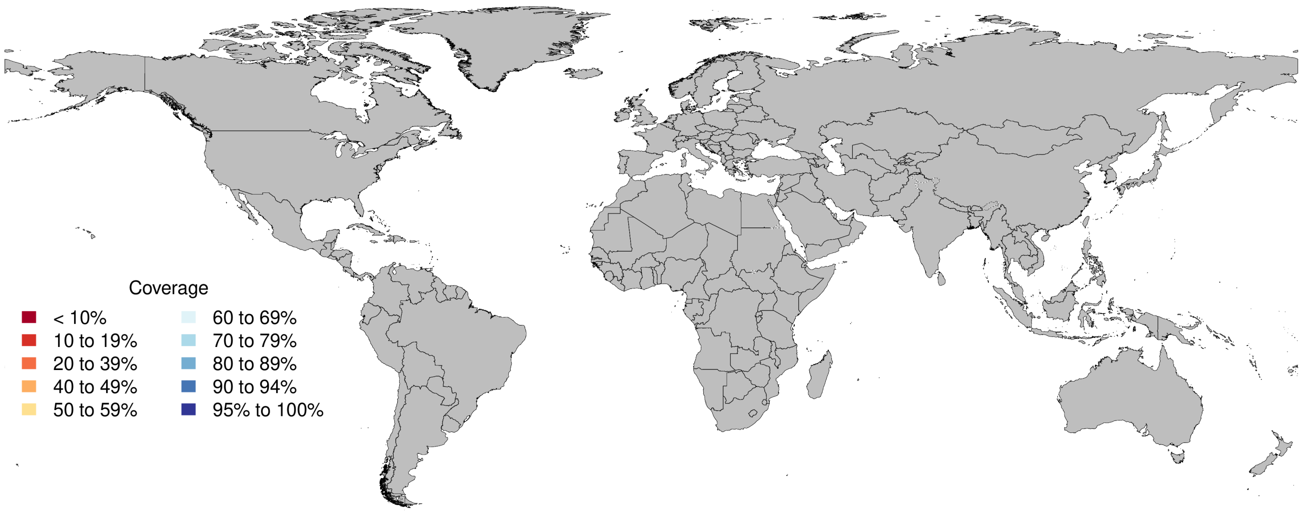

1990

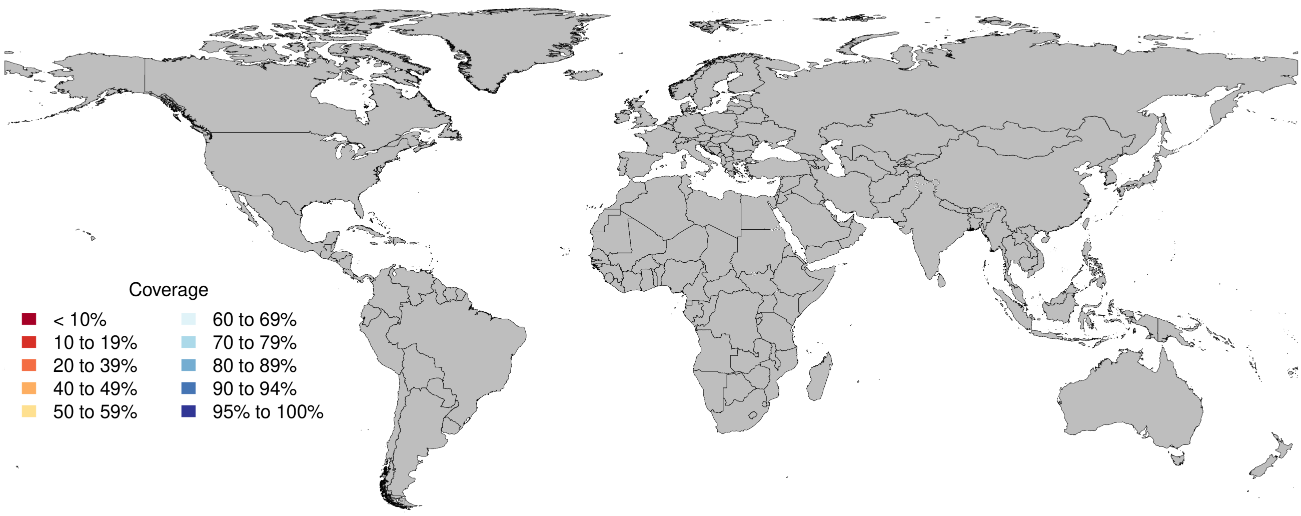

2000

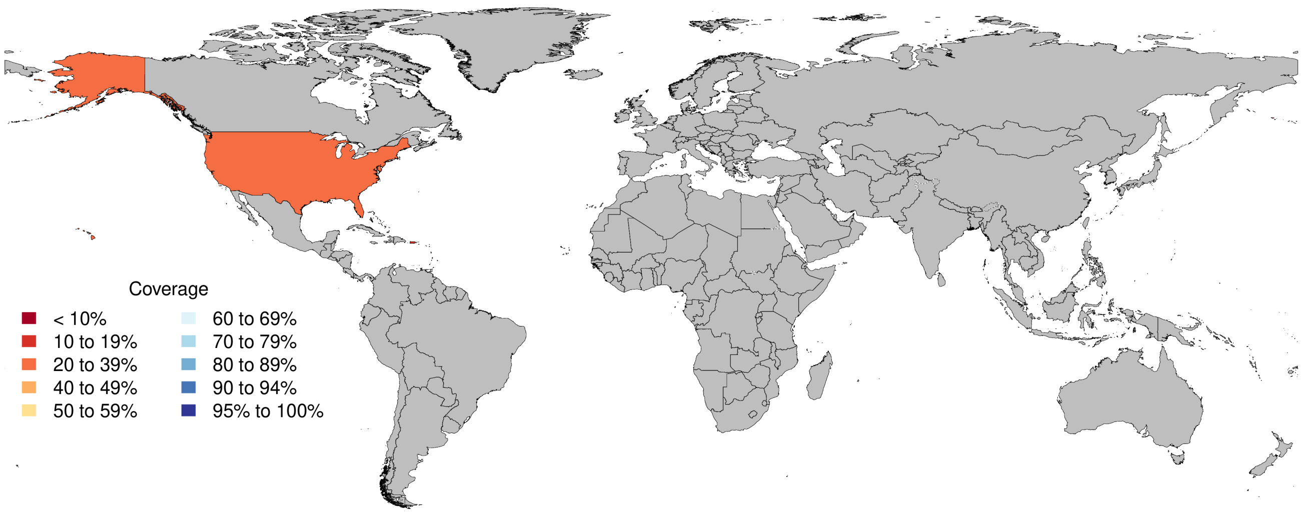

2010

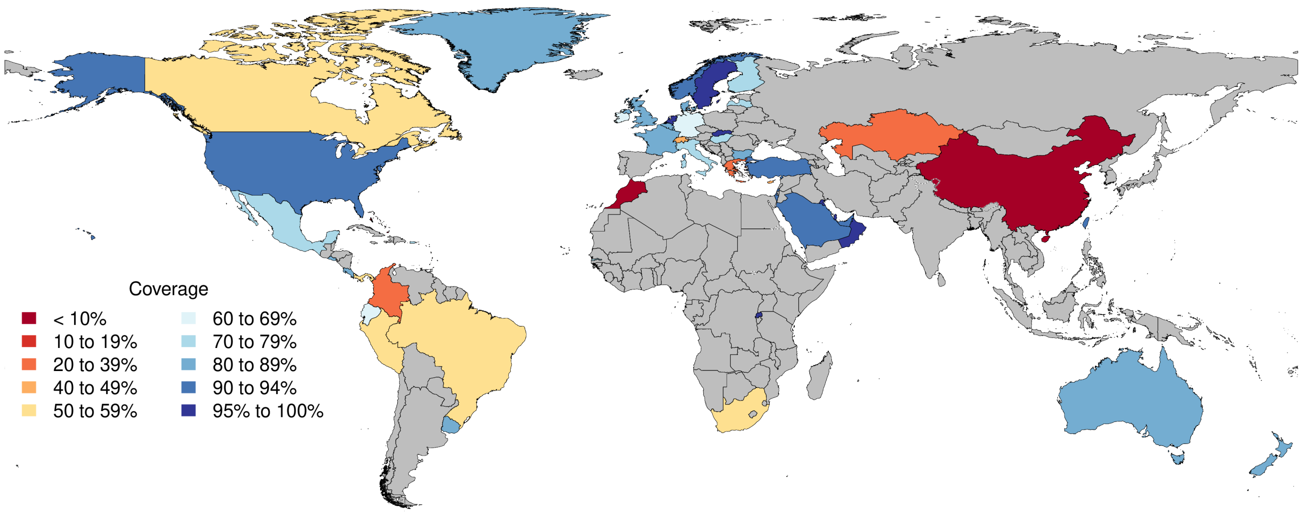

2019

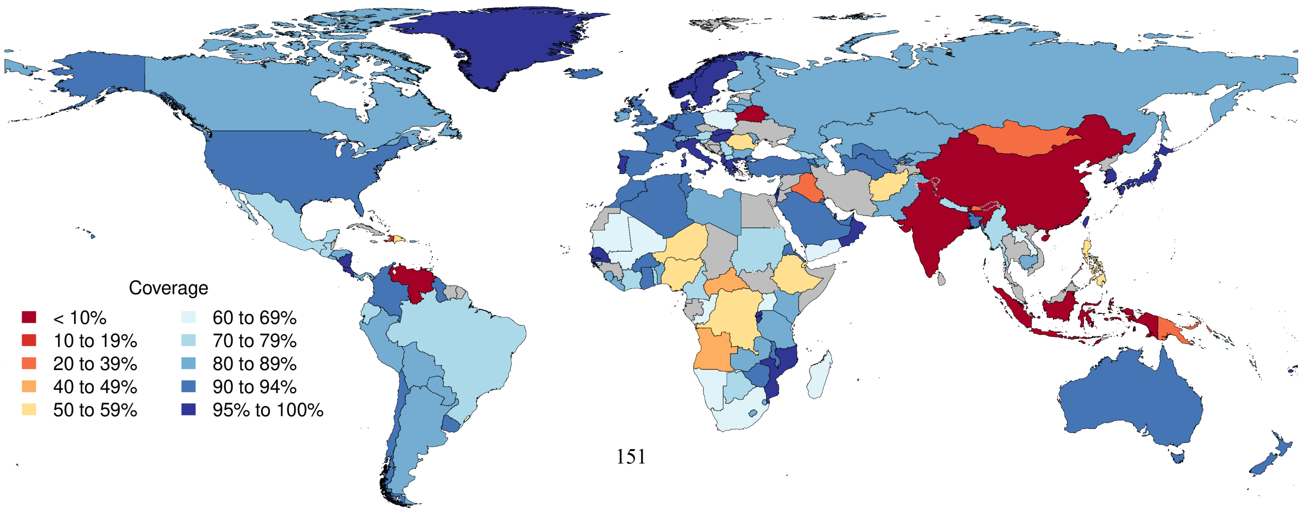

Supplementary figure 26. Global vaccination coverage in select years, Pol3. Maps are ordered top to bottom chronologically by year, featuring estimates from 1980, 1990, 2000, 2010, and 2019, respectively. Countries with higher estimated coverage in each year are coloured in dark blue, and countries with lower estimated coverage are coloured in dark red. Locations where the vaccine is not nationally recommended or where estimates are not produced for GBD are coloured in grey. GBD=Global Burden of Disease. Pol3=polio vaccine, third dose.

1980

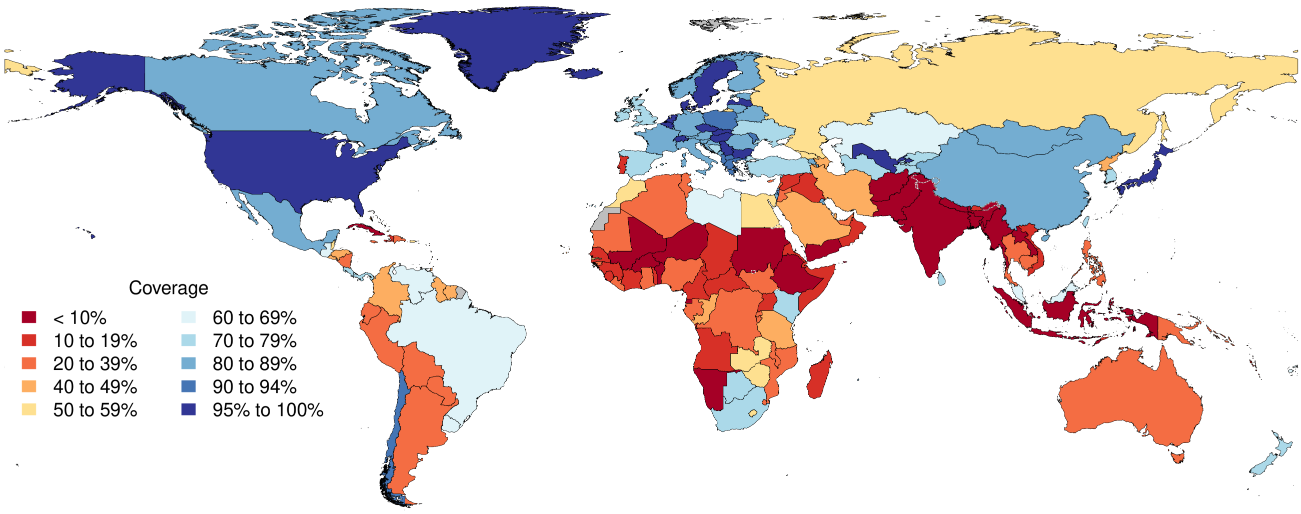

1990

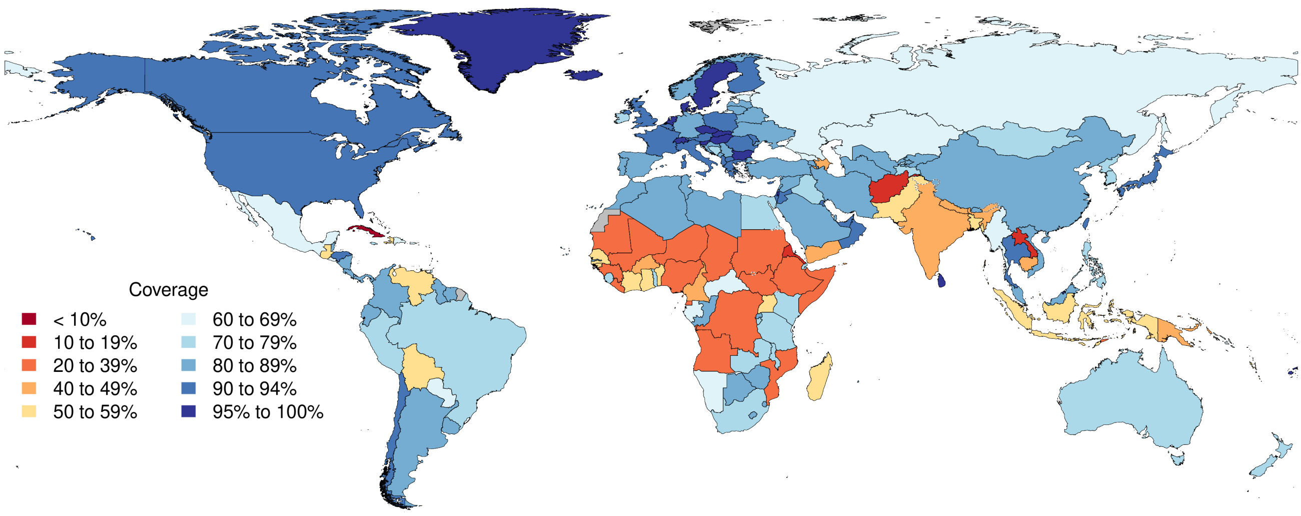

2000

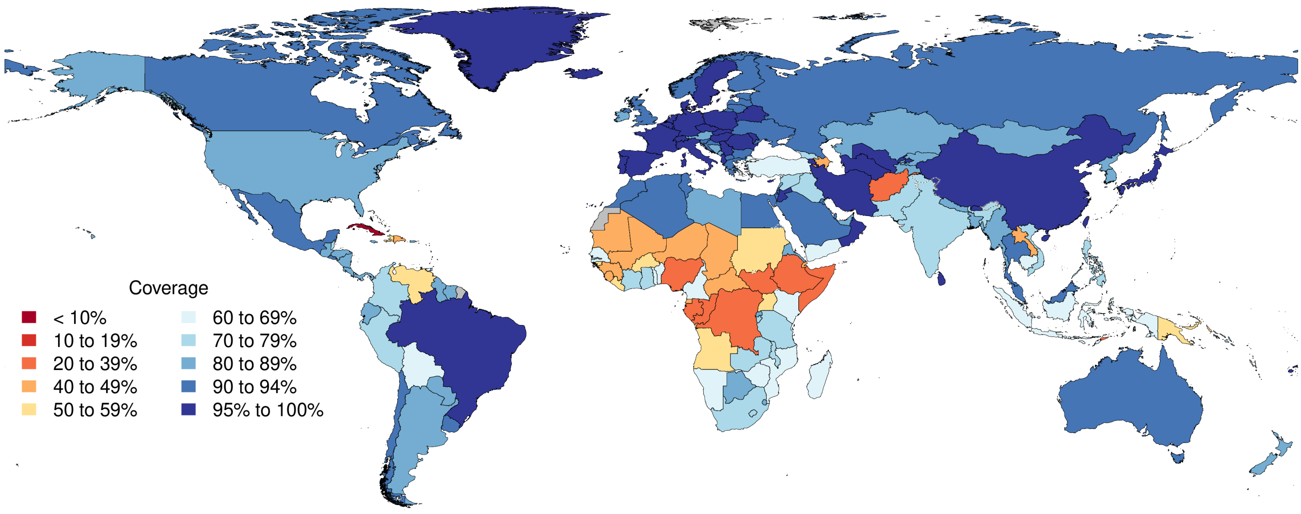

2010

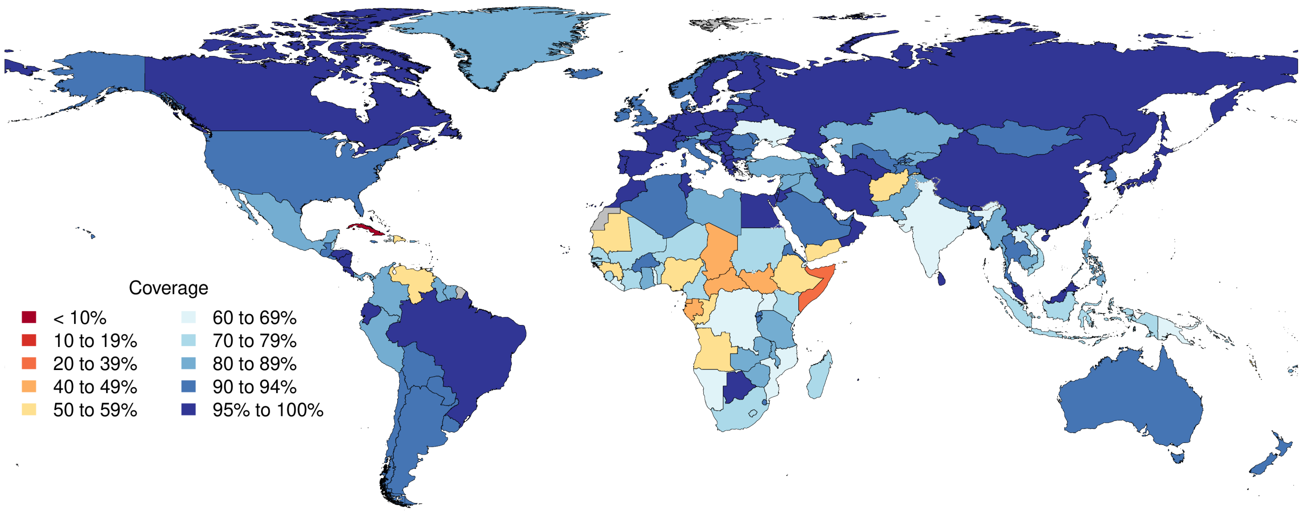

2019

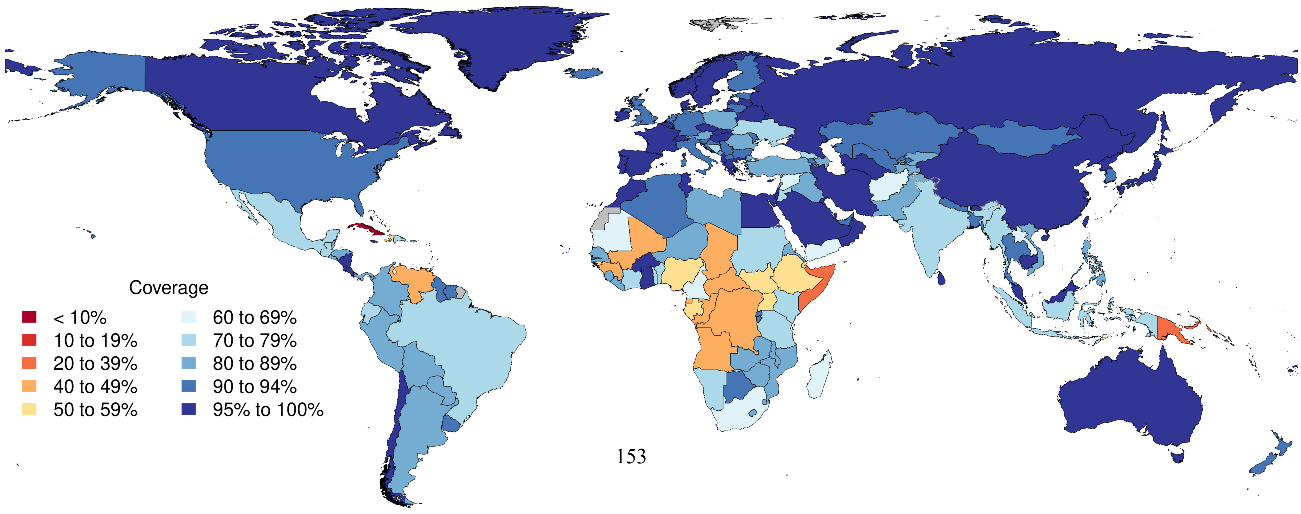

Supplementary figure 27. Global vaccination coverage in select years, RCV1. Maps are ordered top to bottom chronologically by year, featuring estimates from 1980, 1990, 2000, 2010, and 2019, respectively. Countries with higher estimated coverage in each year are coloured in dark blue, and countries with lower estimated coverage are coloured in dark red. Locations where the vaccine is not nationally recommended or where estimates are not produced for GBD are coloured in grey. GBD=Global Burden of Disease. RCV1=rubella-containing vaccine, first dose.

1980

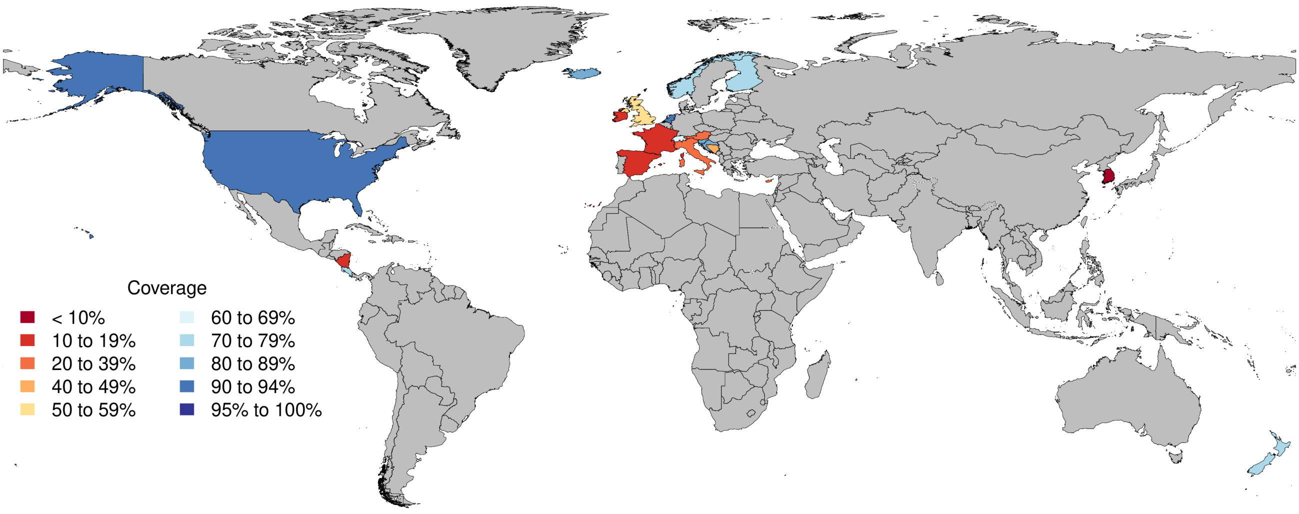

1990

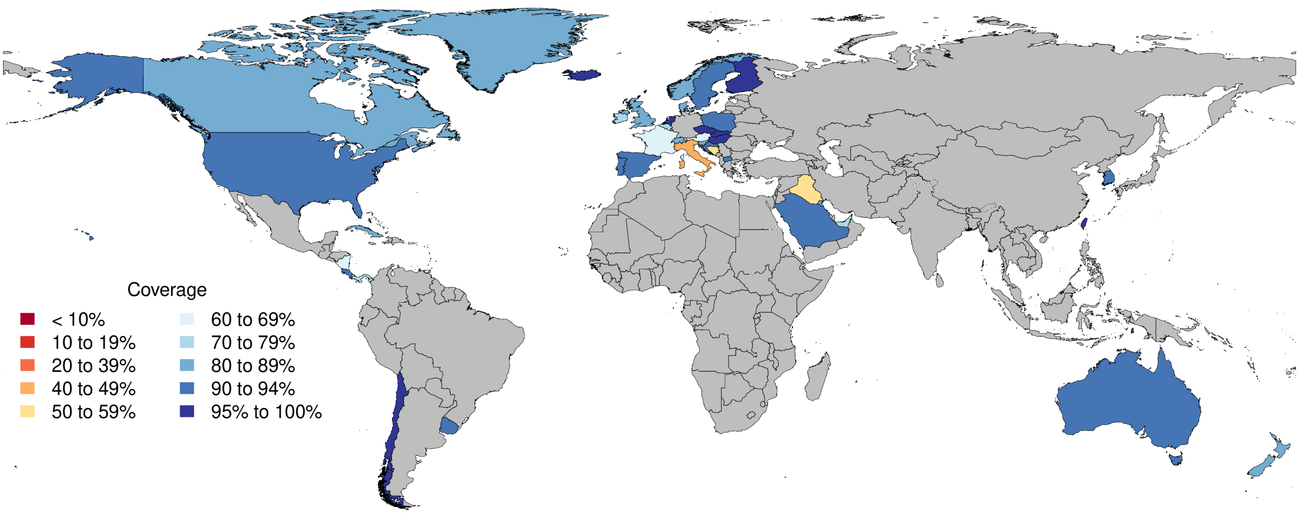

2000

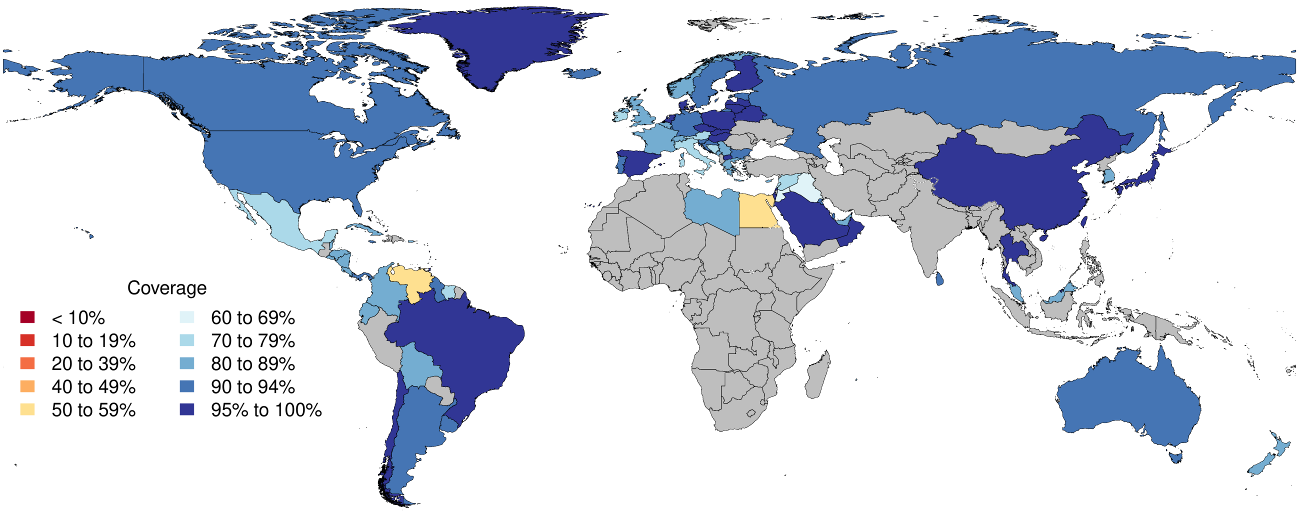

2010

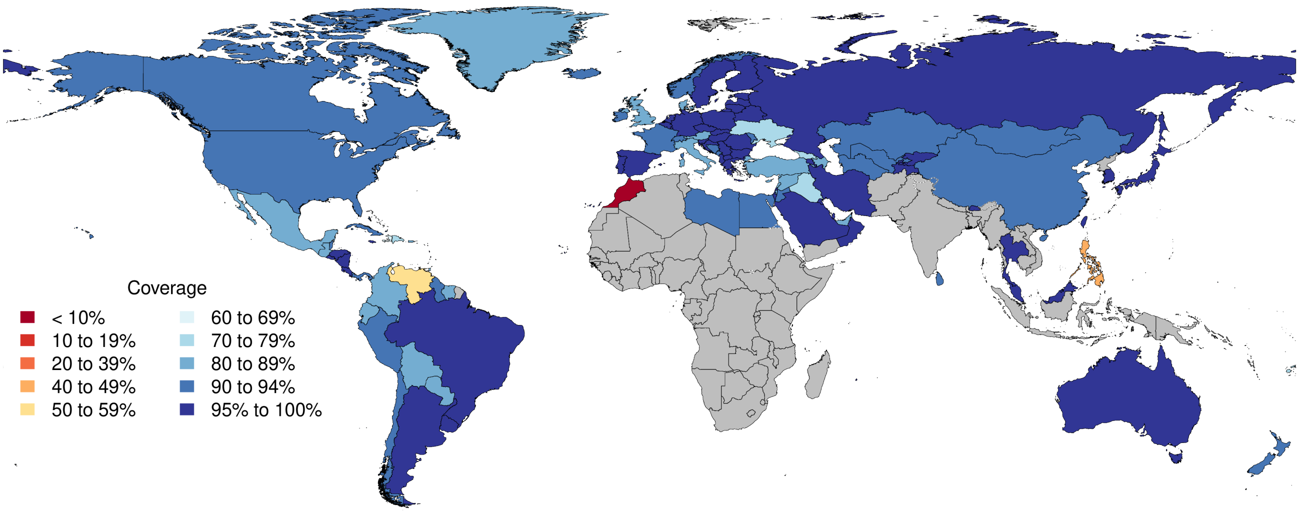

2019

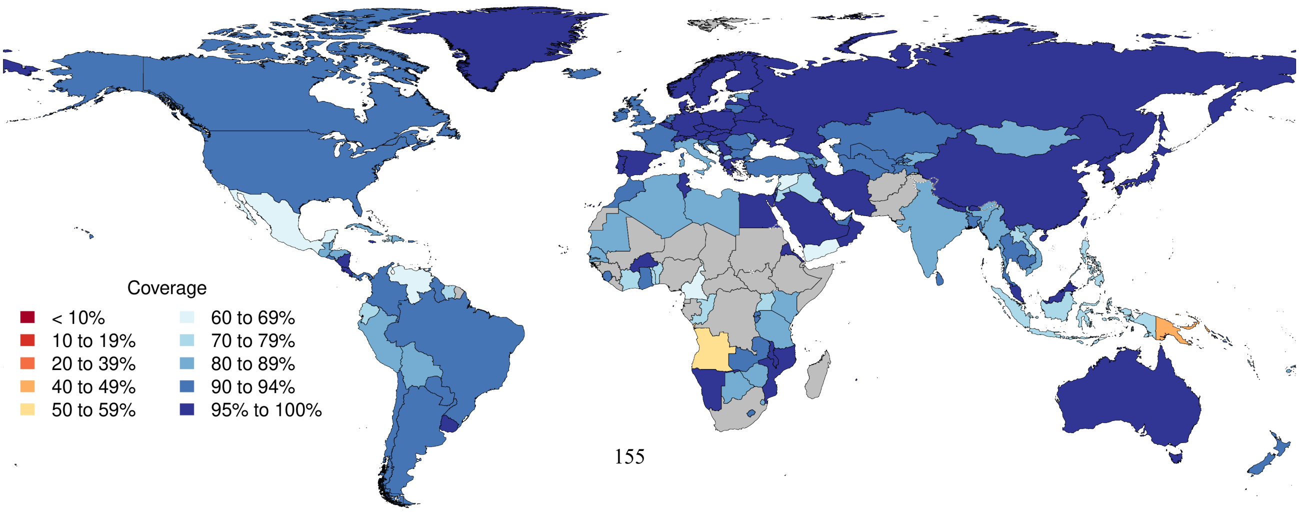

Supplementary figure 28. Global vaccination coverage in select years, RotaC. Maps are ordered top to bottom chronologically by year, featuring estimates from 1980, 1990, 2000, 2010, and 2019, respectively. Countries with higher estimated coverage in each year are coloured in dark blue, and countries with lower estimated coverage are coloured in dark red. Locations where the vaccine is not nationally recommended or where estimates are not produced for GBD are coloured in grey. GBD=Global Burden of Disease. RotaC=completed rotavirus series.

1980

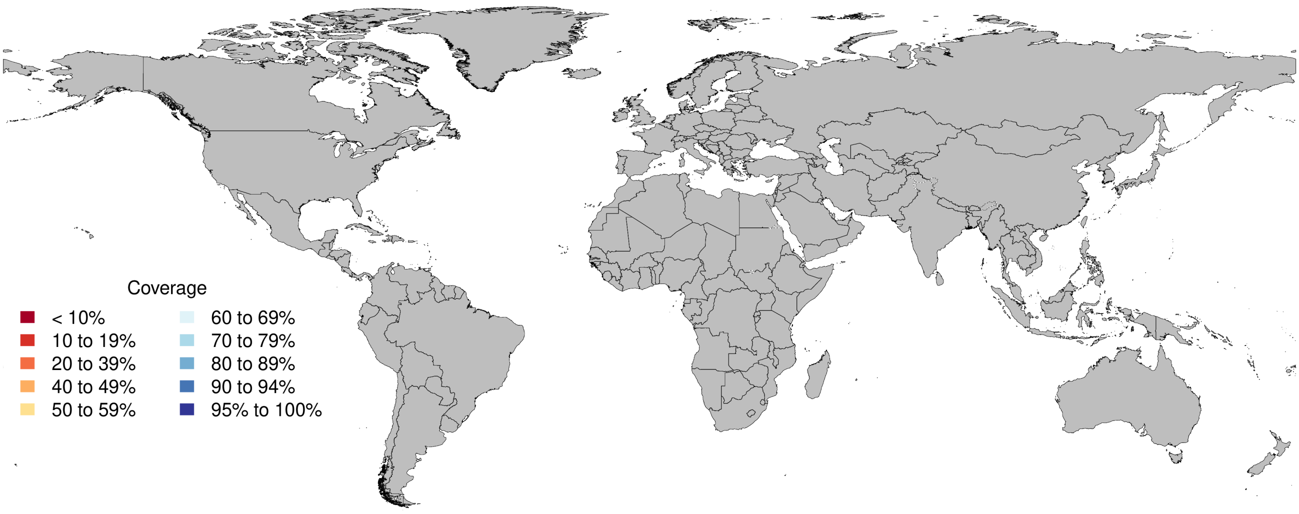

1990

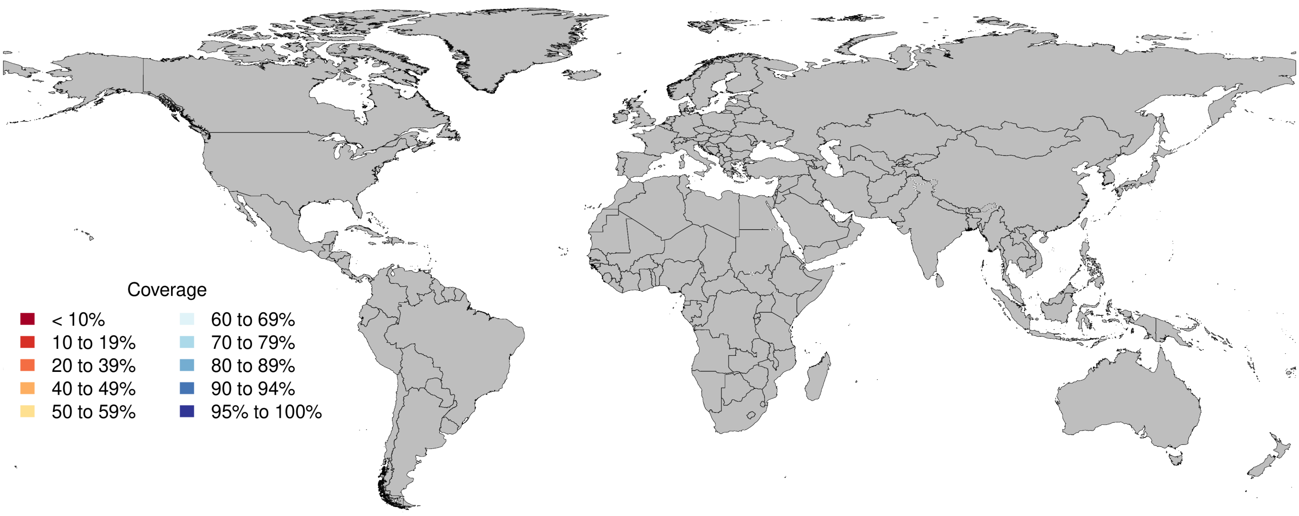

2000

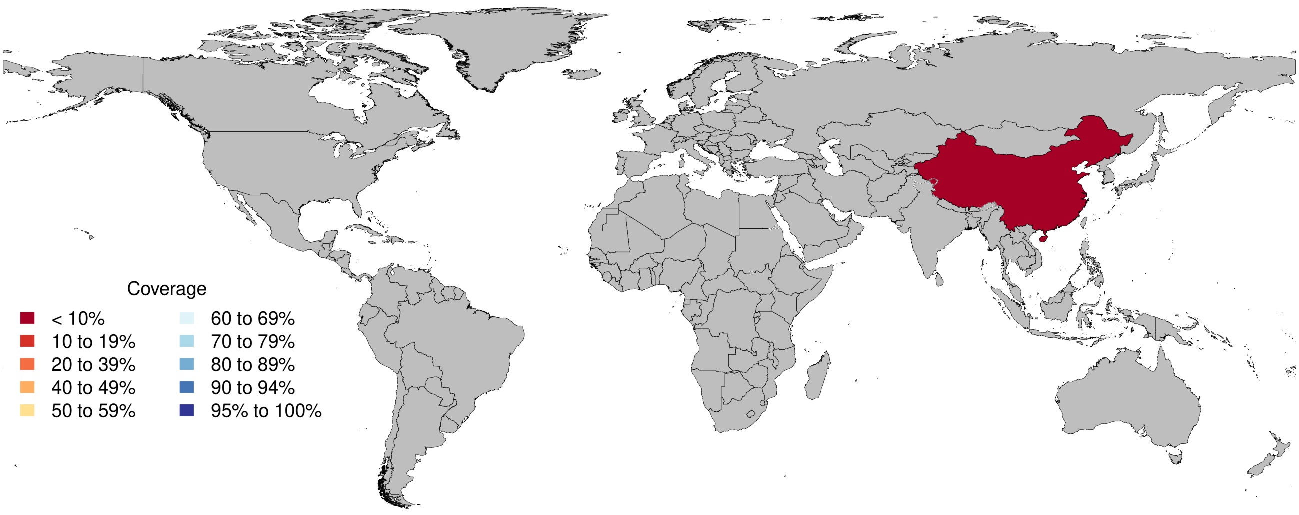

2010

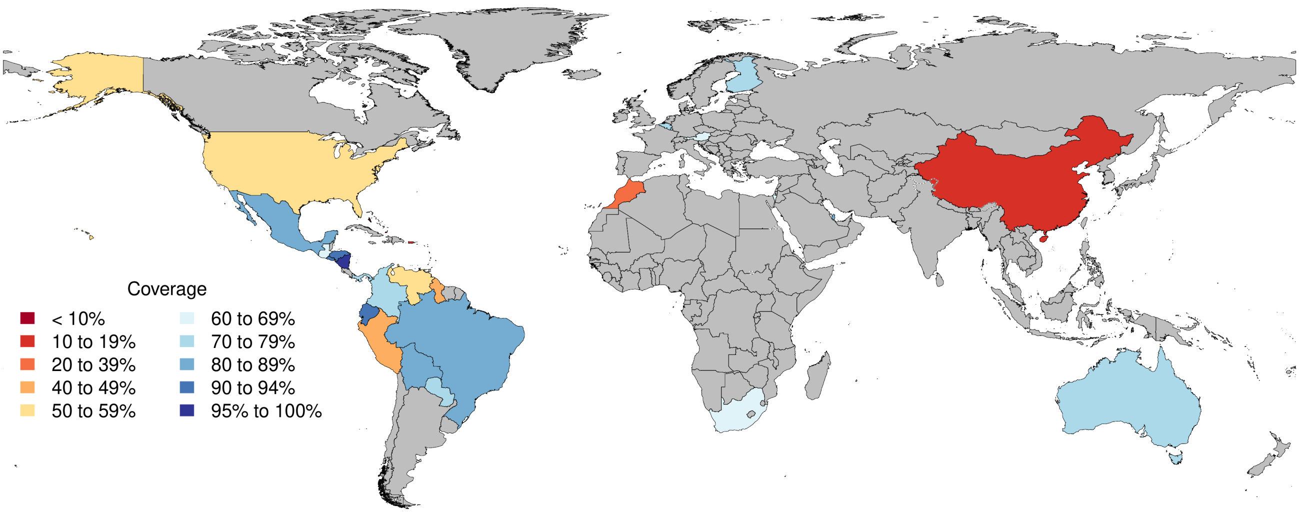

2019

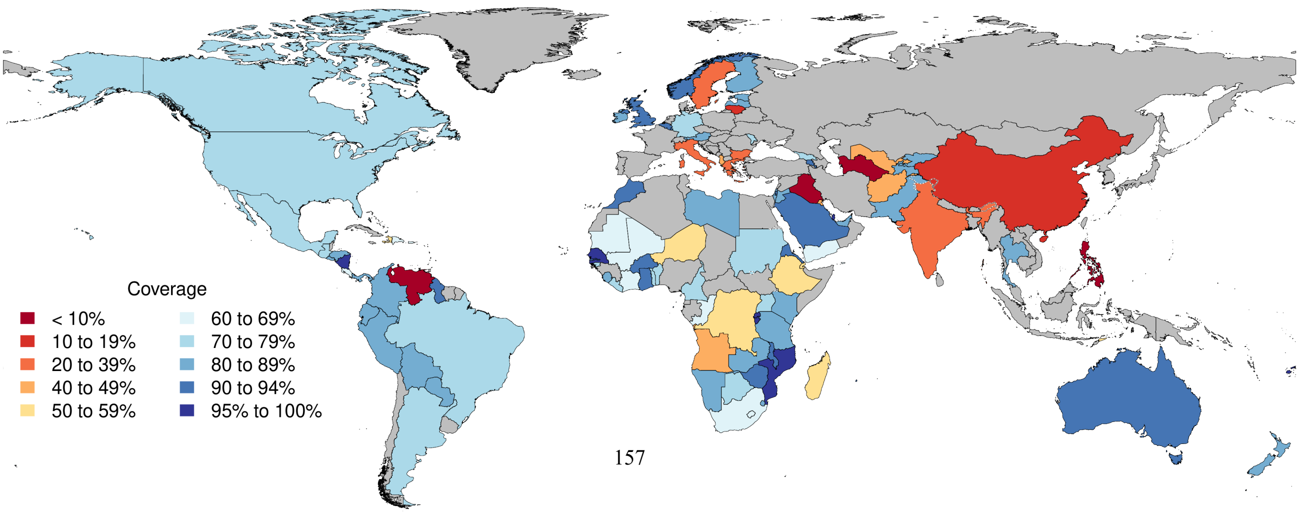

Supplementary figure 29. Percentage of locations reaching the 2020 GVAP target in 2010 and 2019, globally and by GBD super-region, given country introduction. Each cell represents the percentage of countries, globally and by GBD super-region, that have reached the GVAP 90% national coverage target in 2010 and 2019 accounting for variable national introduction schedules per vaccine. Percentages are shown for each vaccine separately meeting the target, for at least any single vaccine meeting the target (represented by the column labeled "Any"), and for all assessed vaccines as listed meeting the target including all countries with the vaccine introduced in the given year ("All"). Cells shown in white have no countries with the vaccine in their national schedule for that vaccine-year combination. GVAP=Global Vaccine Action Plan. GBD=Global Burden of Disease. DTP3=diphtheria-tetanus-pertussis, third dose. HepB3=hepatitis B vaccine, third dose. Hib3=*Haemophilus influenzae* type b vaccine, third dose. MCV1=measles-containing vaccine, first dose. MCV2=measles-containing vaccine, second dose. PCV3=pneumococcal conjugate vaccine, third dose. Pol3=polio vaccine, third dose. RCV1=rubella-containing vaccine, first dose. RotaC=completed rotavirus series.

	DTP3	MCV1	Pol3	HepB3	Hib3	MCV2	RCV1	PCV3	RotaC	Any	All
Global, 2010	59	59	57	51	51	51	70	30	12	67	27
Global, 2019	53	61	53	51	51	40	64	41	26	68	23
Central Europe, Eastern Europe, and Central Asia, 2010	83	90	79	68	56	79	90	20		90	38
Central Europe, Eastern Europe, and Central Asia, 2019	66	69	69	57	61	55	69	27	8	83	14
High-income, 2010	86	75	92	68	79	44	72	24	0	92	25
High-income, 2019	89	89	92	84	89	61	86	74	29	97	39
Latin America and Caribbean, 2010	61	64	55	53	53	25	62	8	24	76	18
Latin America and Caribbean, 2019	36	58	39	36	36	29	56	26	10	64	18
North Africa and Middle East, 2010	67	62	62	67	61	60	56	88	0	71	33
North Africa and Middle East, 2019	62	57	57	62	62	52	63	65	38	62	43
South Asia, 2010	40	40	60	40	33	33	100			60	20
South Asia, 2019	40	80	60	40	40	20	75	20	0	80	0
Southeast Asia, East Asia, and Oceania, 2010	59	53	56	53	48	50	60	33	0	62	41
Southeast Asia, East Asia, and Oceania, 2019	56	65	59	56	43	41	57	24	42	65	38
Sub-Saharan Africa, 2010	22	28	17	21	22	50	100	33	0	28	15
Sub-Saharan Africa, 2019	26	33	17	26	26	3	46	29	31	41	0

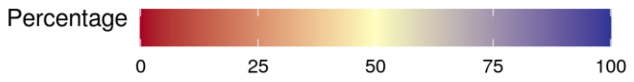

Supplementary figure 30. Comparing global coverage estimates, by vaccine, from WUENIC and GBD, 1980-2019. Country-level WUENIC estimates were aggregated to the global level as described in supplementary section 4.4.1. WUENIC=WHO-UNICEF Estimates of National Immunization Coverage. GBD=Global Burden of Disease. BCG=Bacillus Calmette-Guérin. DTP1=diphtheria-tetanus-pertussis, first dose. DTP3=diphtheria-tetanus-pertussis, third dose. HepB3=hepatitis B vaccine, third dose. Hib3=*Haemophilus influenzae* type b, third dose. MCV1=measles-containing vaccine, first dose. MCV2=measles-containing vaccine, second dose. PCV3=pneumococcal conjugate vaccine, third dose. Pol3=polio vaccine, third dose. RCV1=rubella-containing vaccine, first dose. RotaC=rotavirus, complete series (two or three doses).

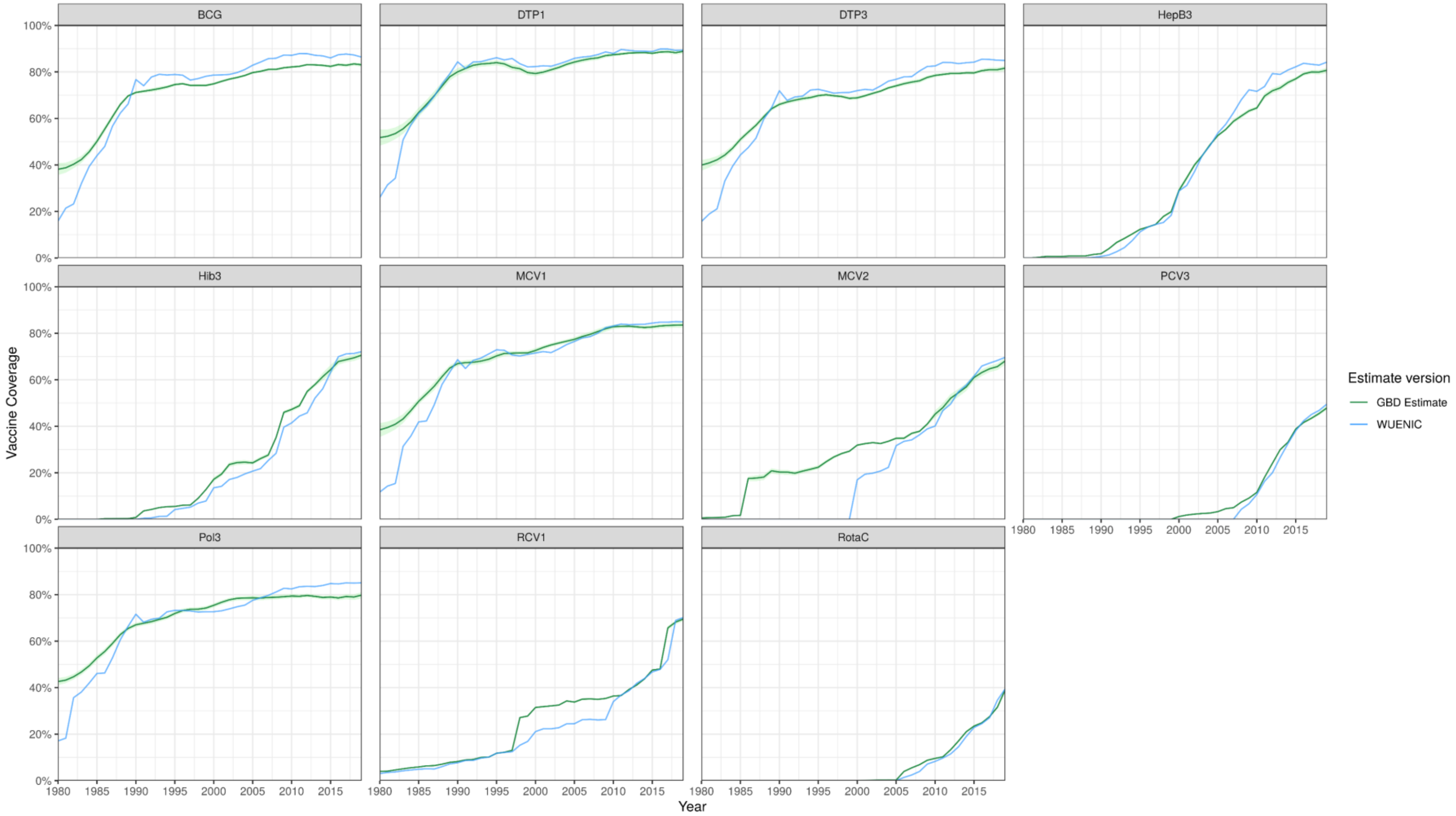

Supplementary figure 31. Time series of location-specific vaccination coverage by vaccine, 1980-2019. Pages show country- and vaccine-specific estimates of coverage proportions, 1980-2019. Data used in the model are coloured in black. Data excluded from the model are coloured in light blue. Point shapes correspond to different input data types. The solid green line illustrates estimated annual coverage. 95% uncertainty intervals are represented by the shaded, light green band around each mean estimate. WUENIC estimates released July 2020 are shown in light blue. WUENIC=WHO-UNICEF Estimates of National Immunization Coverage. Original Country-reported=raw, unadjusted official country-reported data from the Joint Reporting Form (JRF). GBD=Global Burden of Disease. Country-reported=official country-reported data from the JRF, after bias adjustment. DHS=Demographics and Health Survey. MICS=Multiple Indicator Cluster Survey. Other microdata=Non-DHS, non-MICS survey microdata. Other tabulation=survey report tabulation. Imputed=statistically imputed as described in supplementary section 3.3.1.

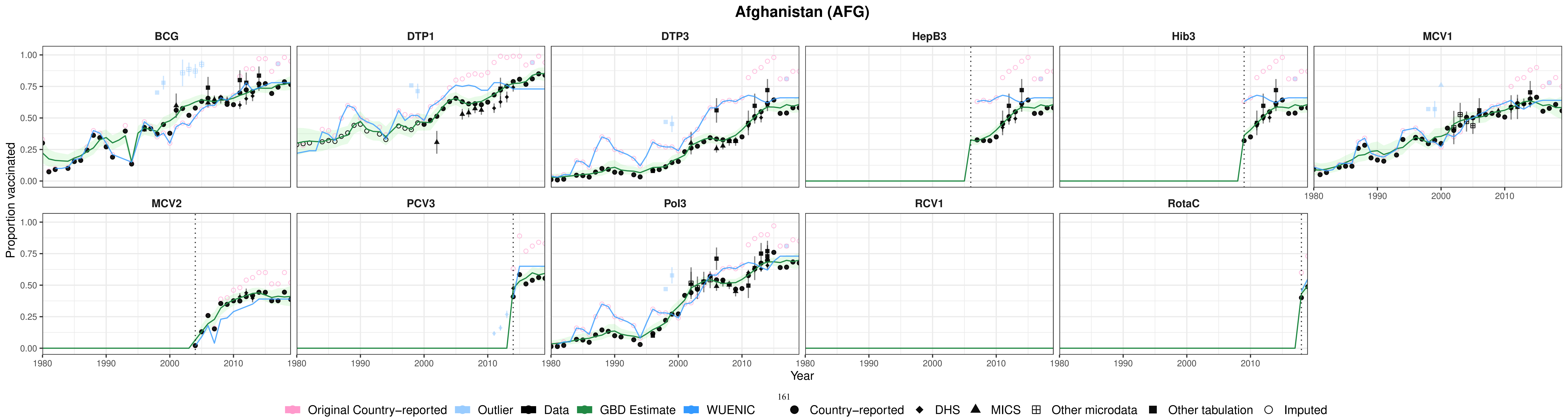
Afghanistan (AFG)
BCG
DTP1
DTP3
HepB3
Hib3
MCV1
MCV2
PCV3
Pol3
RCV1
RotaC
Proportion vaccinated
Year
Original Country−reported
Outlier
Data
GBD Estimate
WUENIC
Country−reported
DHS
MICS
Other microdata
Other tabulation
Imputed

Angola (AGO)

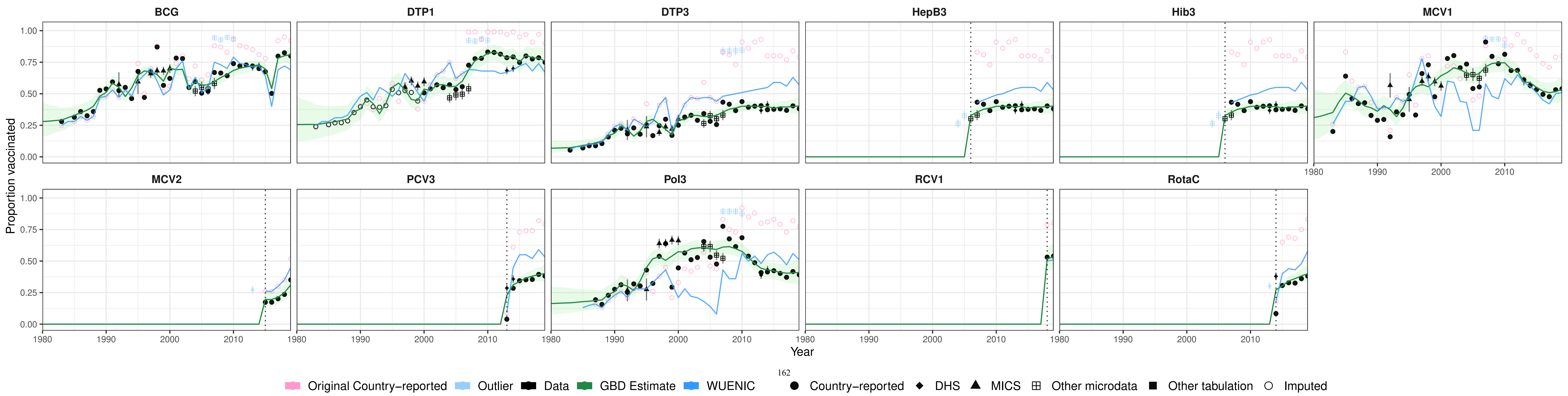

BCG
DTP1
DTP3
HepB3
Hib3
MCV1
MCV2
PCV3
Pol3
RCV1
RotaC
Proportion vaccinated
Year
1.00
0.75
0.50
0.25
0.00
1980
1990
2000
2010
Original Country−reported
Outlier
Data
GBD Estimate
WUENIC
Country−reported
DHS
MICS
Other microdata
Other tabulation
Imputed

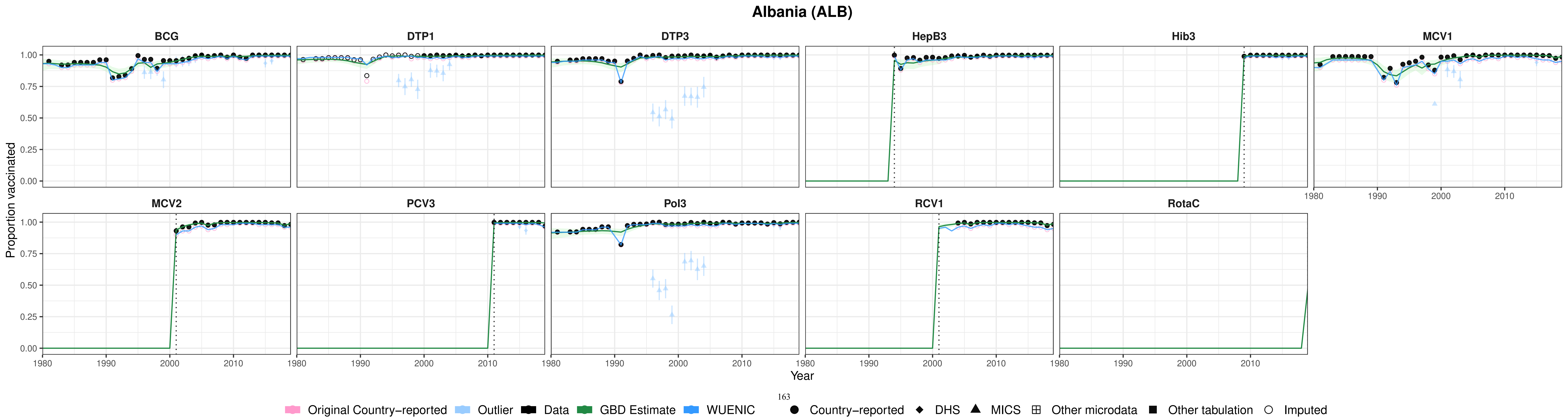
Albania (ALB)
BCG
DTP1
DTP3
HepB3
Hib3
MCV1
MCV2
PCV3
Pol3
RCV1
RotaC
Proportion vaccinated
Year
Original Country−reported
Outlier
Data
GBD Estimate
WUENIC
Country−reported
DHS
MICS
Other microdata
Other tabulation
Imputed

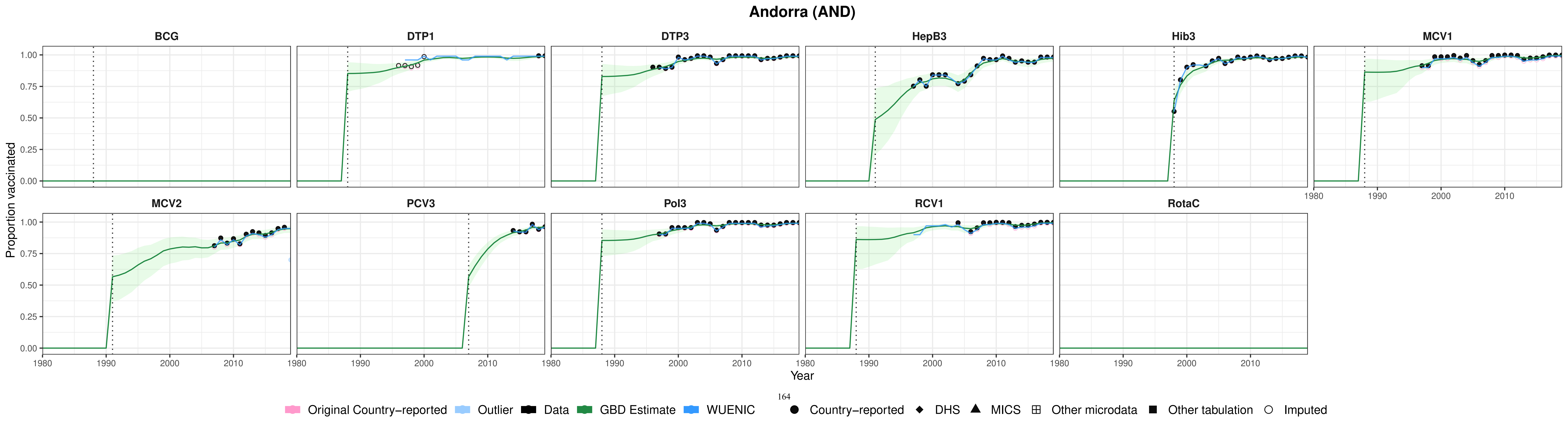

Andorra (AND)
BCG
DTP1
DTP3
HepB3
Hib3
MCV1
MCV2
PCV3
Pol3
RCV1
RotaC
Proportion vaccinated
0.00
0.25
0.50
0.75
1.00
1980
1990
2000
2010
Year
Original Country−reported
Outlier
Data
GBD Estimate
WUENIC
Country−reported
DHS
MICS
Other microdata
Other tabulation
Imputed

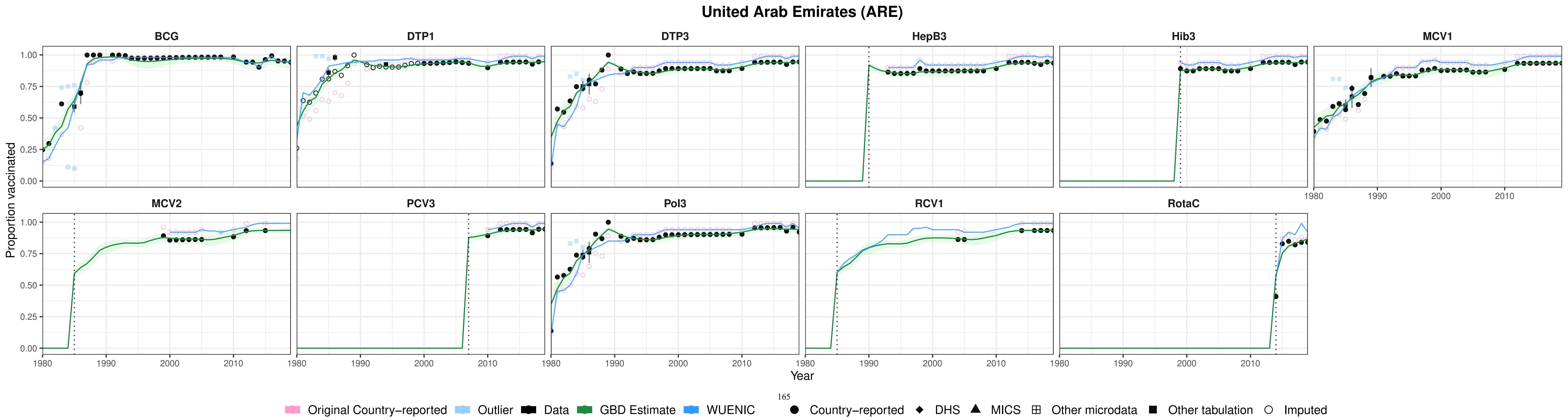
United Arab Emirates (ARE)
BCG
DTP1
DTP3
HepB3
Hib3
MCV1
MCV2
PCV3
Pol3
RCV1
RotaC
Proportion vaccinated
Year
Original Country−reported
Outlier
Data
GBD Estimate
WUENIC
Country−reported
DHS
MICS
Other microdata
Other tabulation
Imputed

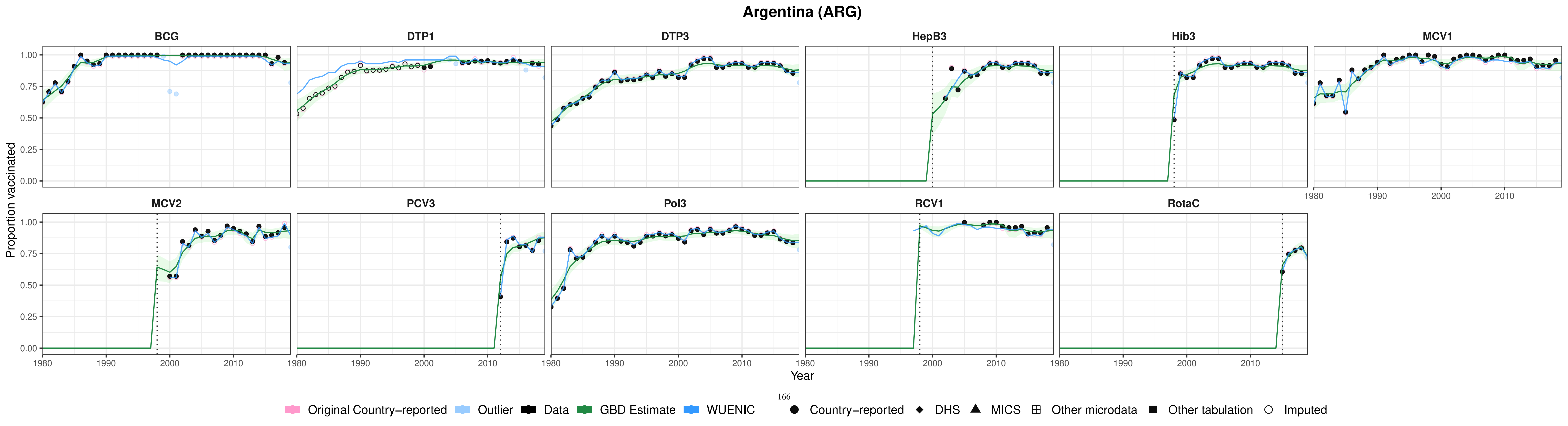
Argentina (ARG)
BCG
DTP1
DTP3
HepB3
Hib3
MCV1
MCV2
PCV3
Pol3
RCV1
RotaC
Proportion vaccinated
Year
Original Country−reported
Outlier
Data
GBD Estimate
WUENIC
Country−reported
DHS
MICS
Other microdata
Other tabulation
Imputed

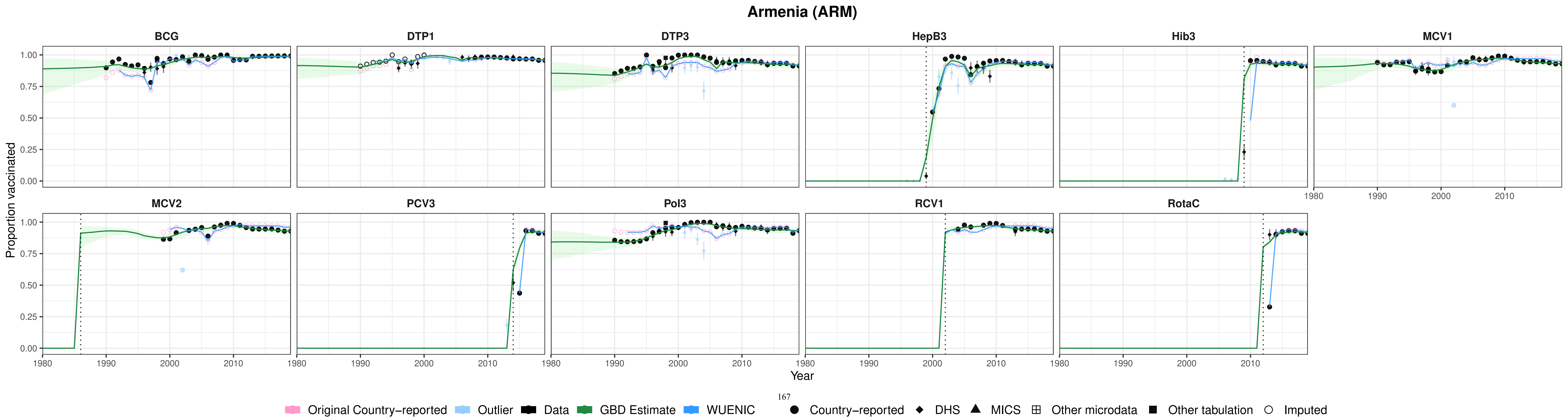

Armenia (ARM)
BCG
DTP1
DTP3
HepB3
Hib3
MCV1
MCV2
PCV3
Pol3
RCV1
RotaC
Proportion vaccinated
Year
1.00
0.75
0.50
0.25
0.00
1980
1990
2000
2010
Original Country−reported
Outlier
Data
GBD Estimate
WUENIC
Country−reported
DHS
MICS
Other microdata
Other tabulation
Imputed

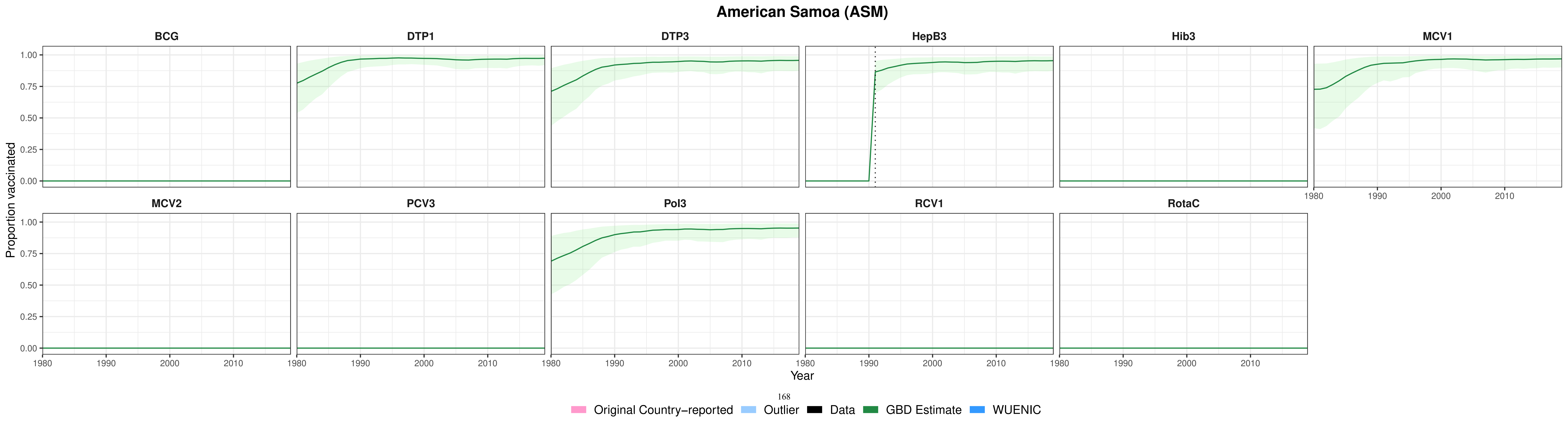

American Samoa (ASM)
BCG
DTP1
DTP3
HepB3
Hib3
MCV1
MCV2
PCV3
Pol3
RCV1
RotaC
Proportion vaccinated
Year
0.00
0.25
0.50
0.75
1.00
1980
1990
2000
2010

Original Country−reported
Outlier
Data
GBD Estimate
WUENIC

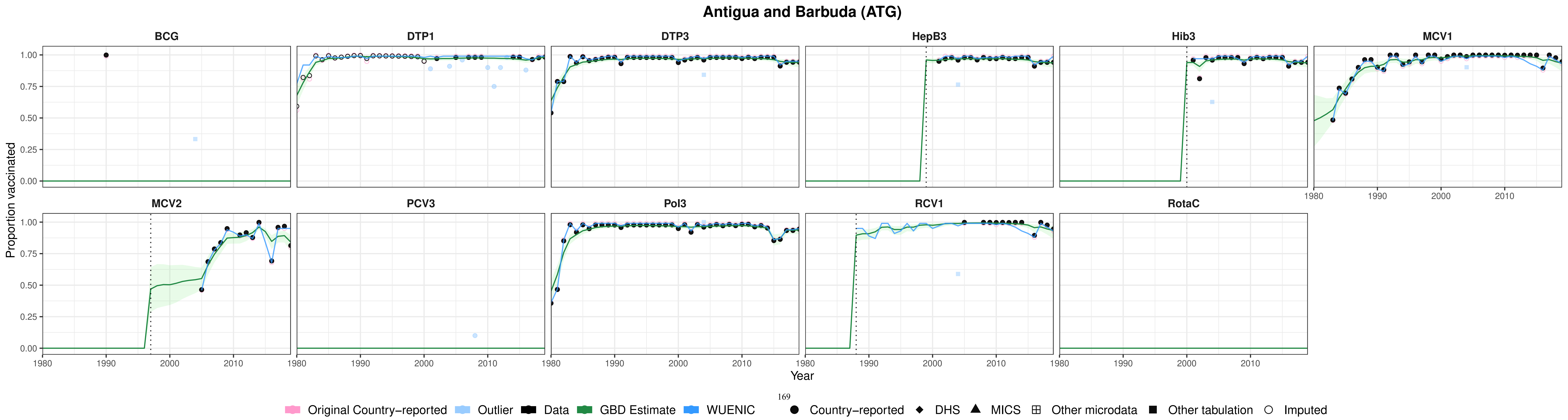
Antigua and Barbuda (ATG)
BCG
DTP1
DTP3
HepB3
Hib3
MCV1
MCV2
PCV3
Pol3
RCV1
RotaC
Proportion vaccinated
Year
Original Country−reported
Outlier
Data
GBD Estimate
WUENIC
Country−reported
DHS
MICS
Other microdata
Other tabulation
Imputed

Australia (AUS)

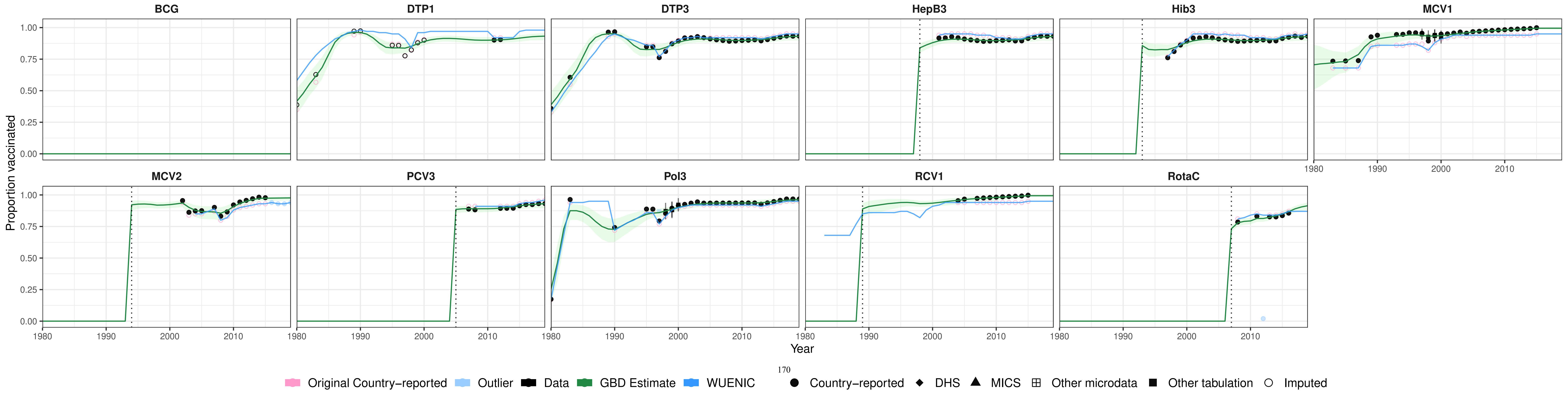

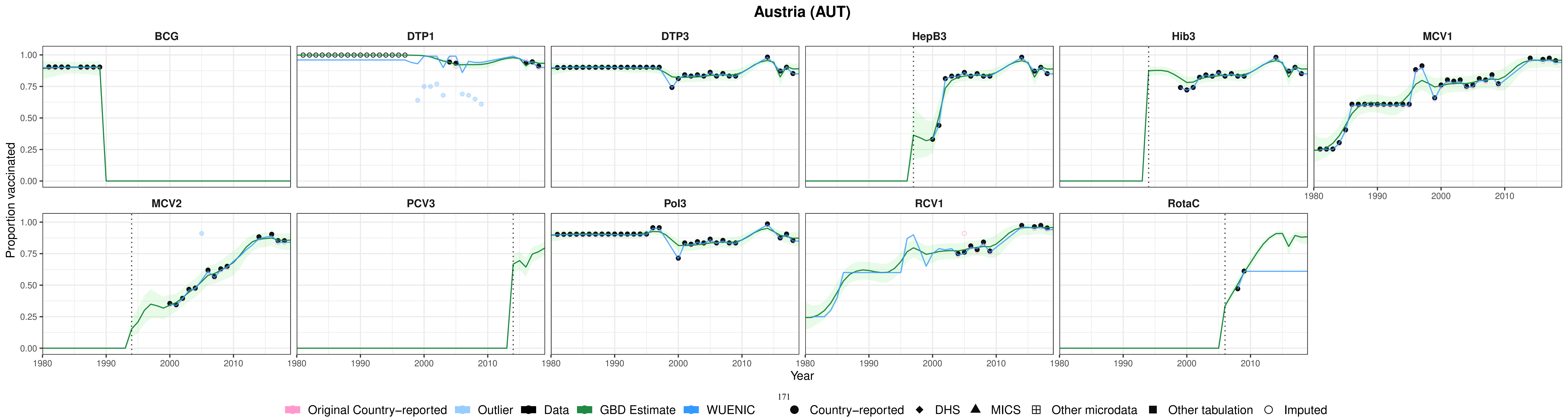

Austria (AUT)
BCG
DTP1
DTP3
HepB3
Hib3
MCV1
MCV2
PCV3
Pol3
RCV1
RotaC
Proportion vaccinated
Year
1.00
0.75
0.50
0.25
0.00
1980
1990
2000
2010
Original Country−reported
Outlier
Data
GBD Estimate
WUENIC
Country−reported
DHS
MICS
Other microdata
Other tabulation
Imputed

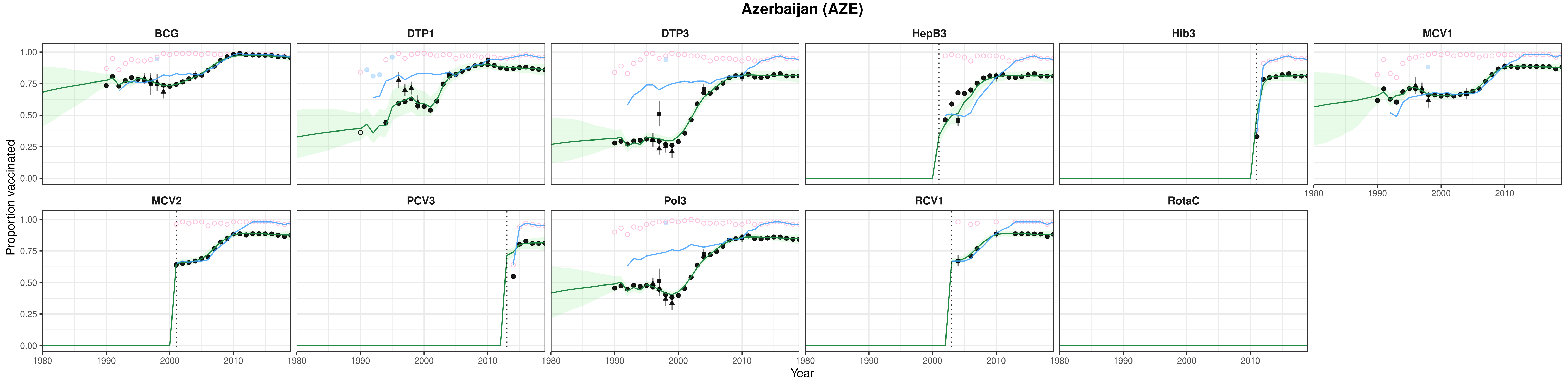
Azerbaijan (AZE)
BCG
DTP1
DTP3
HepB3
Hib3
MCV1
MCV2
PCV3
Pol3
RCV1
RotaC
Proportion vaccinated
Year
Original Country−reported
Outlier
Data
GBD Estimate
WUENIC
Country−reported
DHS
MICS
Other microdata
Other tabulation
Imputed

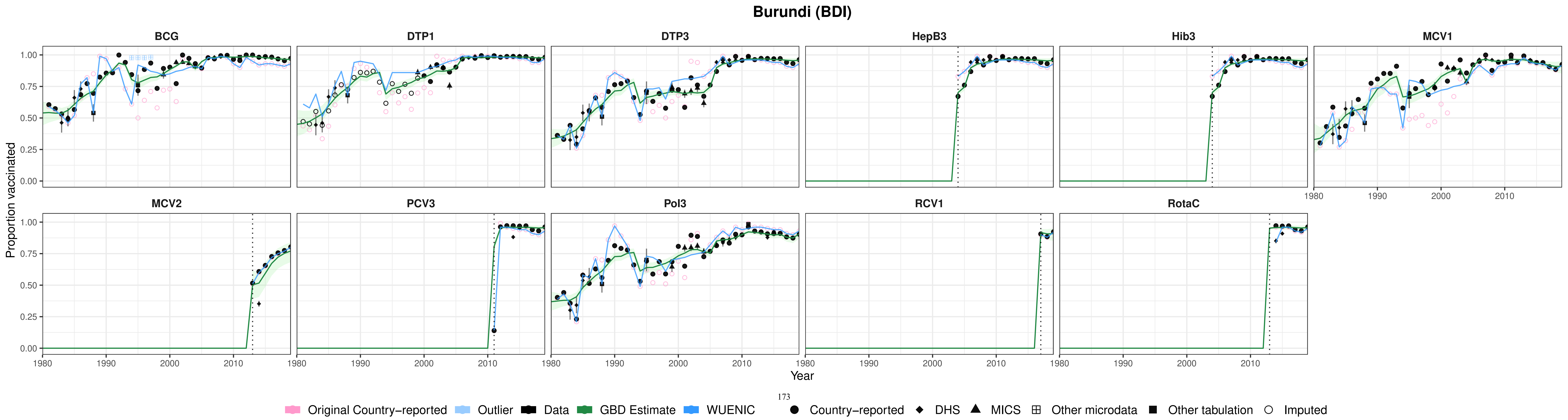

Burundi (BDI)
BCG
DTP1
DTP3
HepB3
Hib3
MCV1
MCV2
PCV3
Pol3
RCV1
RotaC
Proportion vaccinated
Year
1.00
0.75
0.50
0.25
0.00
1980
1990
2000
2010
Original Country−reported
Outlier
Data
GBD Estimate
WUENIC
Country−reported
DHS
MICS
Other microdata
Other tabulation
Imputed

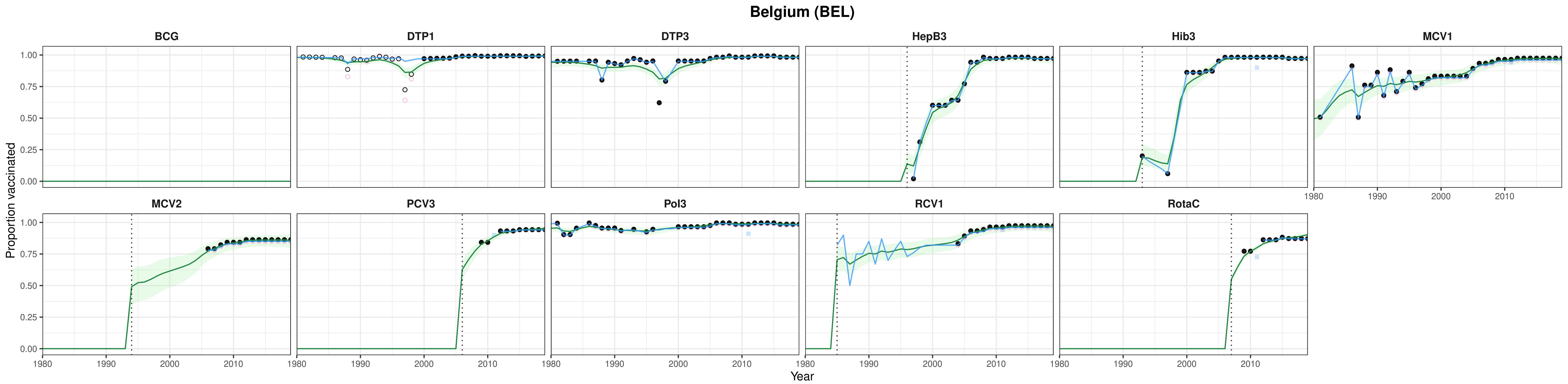

Belgium (BEL)
BCG
DTP1
DTP3
HepB3
Hib3
MCV1
MCV2
PCV3
Pol3
RCV1
RotaC
Proportion vaccinated
Year
0.00
0.25
0.50
0.75
1.00
1980
1990
2000
2010

Original Country−reported
Outlier
Data
GBD Estimate
WUENIC
Country−reported
DHS
MICS
Other microdata
Other tabulation
Imputed

Benin (BEN)

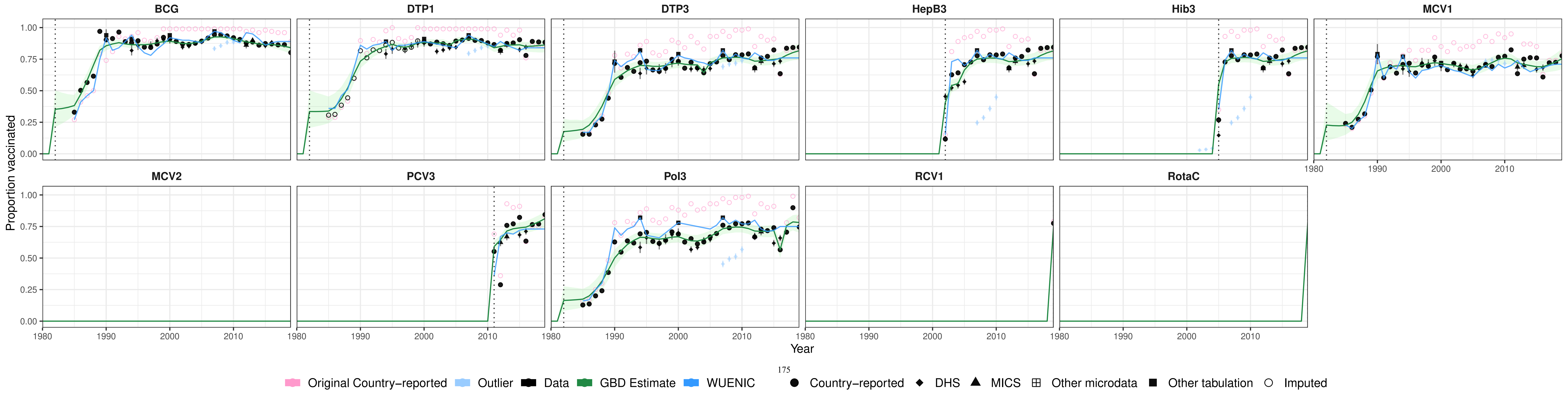

BCG
DTP1
DTP3
HepB3
Hib3
MCV1
MCV2
PCV3
Pol3
RCV1
RotaC
Proportion vaccinated
Year
Original Country−reported
Outlier
Data
GBD Estimate
WUENIC
Country−reported
DHS
MICS
Other microdata
Other tabulation
Imputed

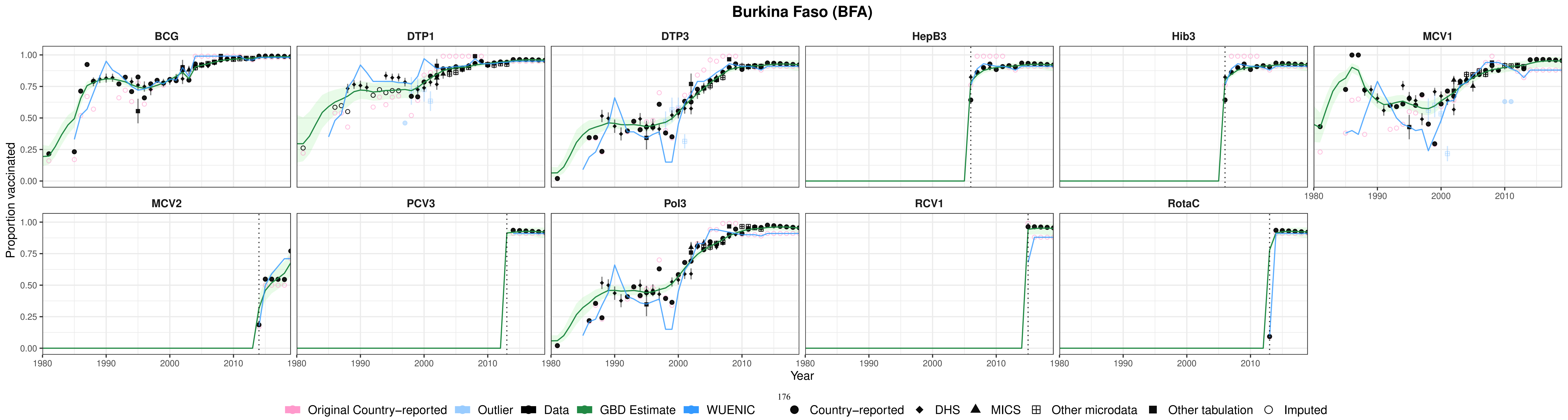

Burkina Faso (BFA)
BCG
DTP1
DTP3
HepB3
Hib3
MCV1
MCV2
PCV3
Pol3
RCV1
RotaC
Proportion vaccinated
Year
1.00
0.75
0.50
0.25
0.00
1980
1990
2000
2010
Original Country−reported
Outlier
Data
GBD Estimate
WUENIC
Country−reported
DHS
MICS
Other microdata
Other tabulation
Imputed

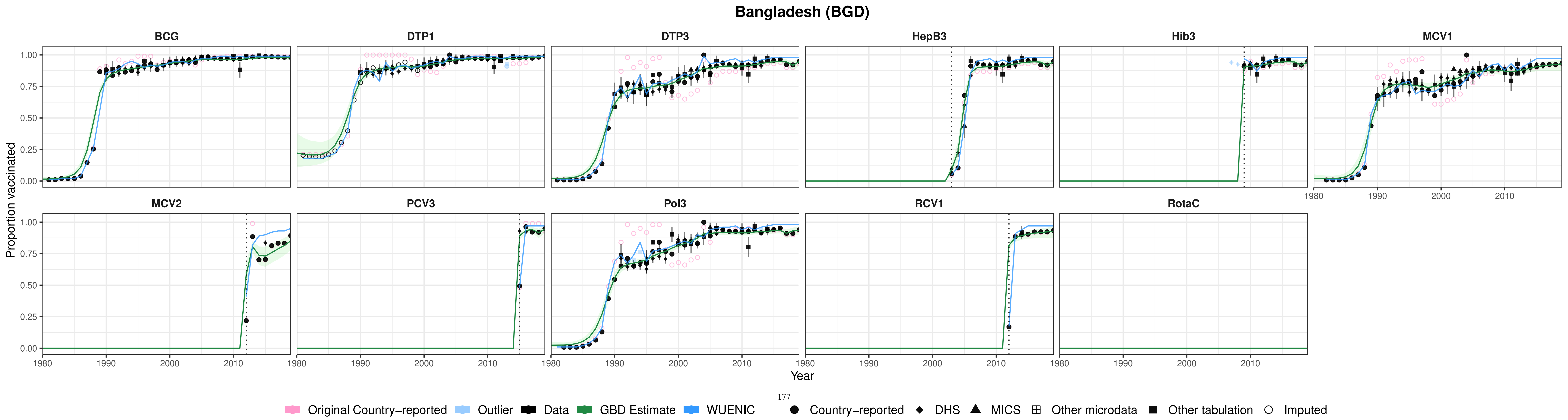

Bangladesh (BGD)
BCG
DTP1
DTP3
HepB3
Hib3
MCV1
MCV2
PCV3
Pol3
RCV1
RotaC
Proportion vaccinated
Year
Original Country−reported
Outlier
Data
GBD Estimate
WUENIC
Country−reported
DHS
MICS
Other microdata
Other tabulation
Imputed

Bulgaria (BGR)

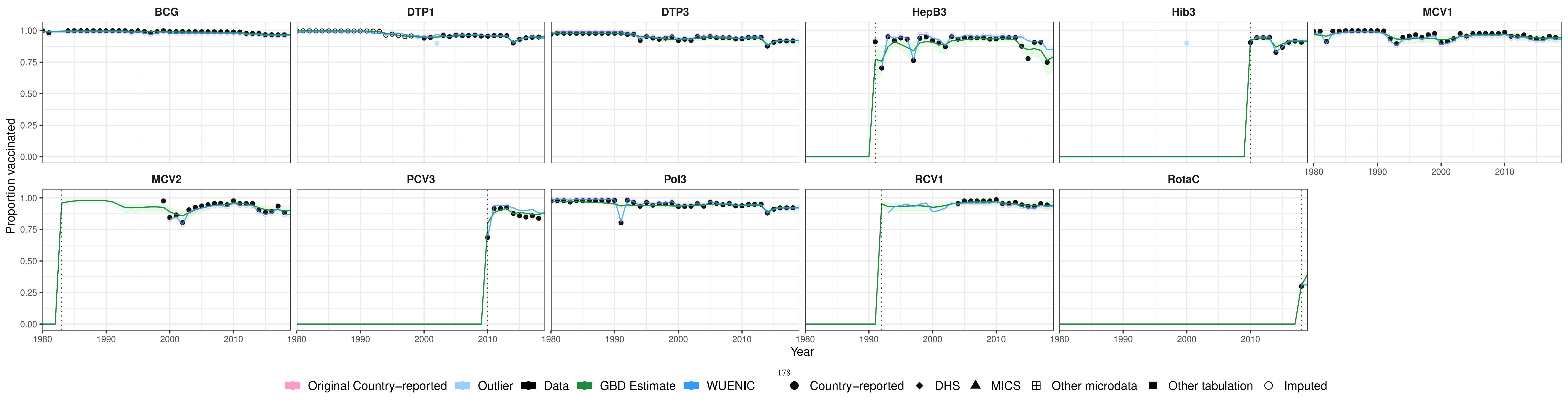

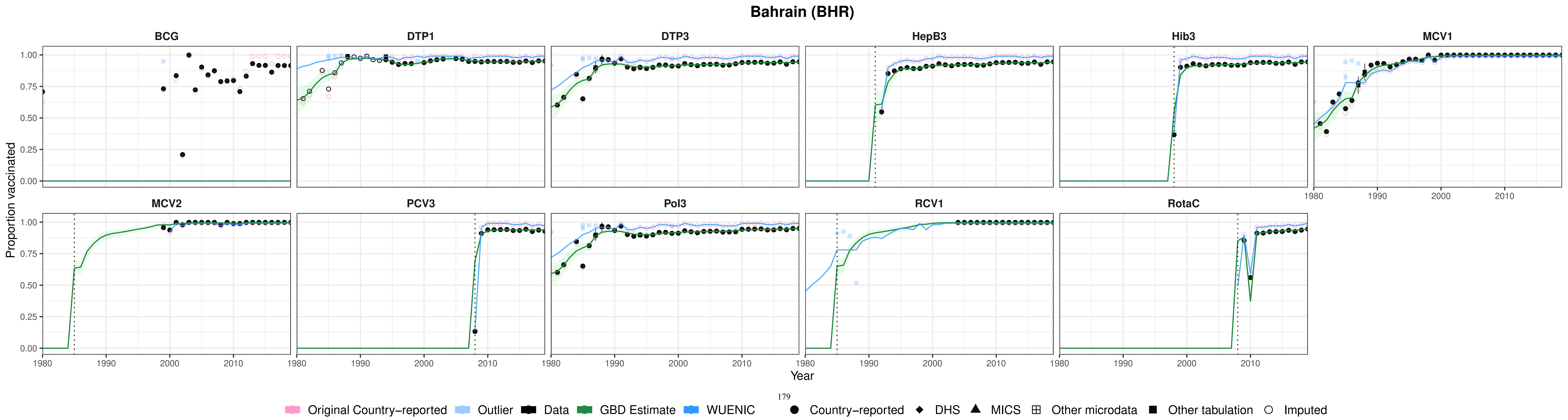
Bahrain (BHR)
BCG
DTP1
DTP3
HepB3
Hib3
MCV1
MCV2
PCV3
Pol3
RCV1
RotaC
Proportion vaccinated
0.00
0.25
0.50
0.75
1.00
1980
1990
2000
2010
Year
Original Country−reported
Outlier
Data
GBD Estimate
WUENIC
Country−reported
DHS
MICS
Other microdata
Other tabulation
Imputed

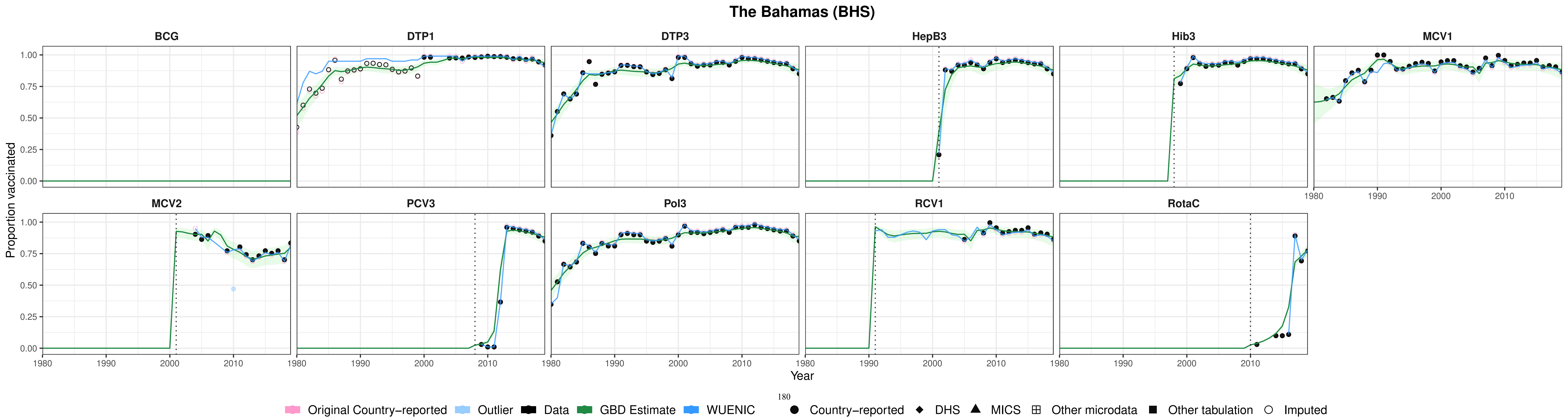
The Bahamas (BHS)
BCG
DTP1
DTP3
HepB3
Hib3
MCV1
MCV2
PCV3
Pol3
RCV1
RotaC
Proportion vaccinated
Year
Original Country−reported
Outlier
Data
GBD Estimate
WUENIC
Country−reported
DHS
MICS
Other microdata
Other tabulation
Imputed

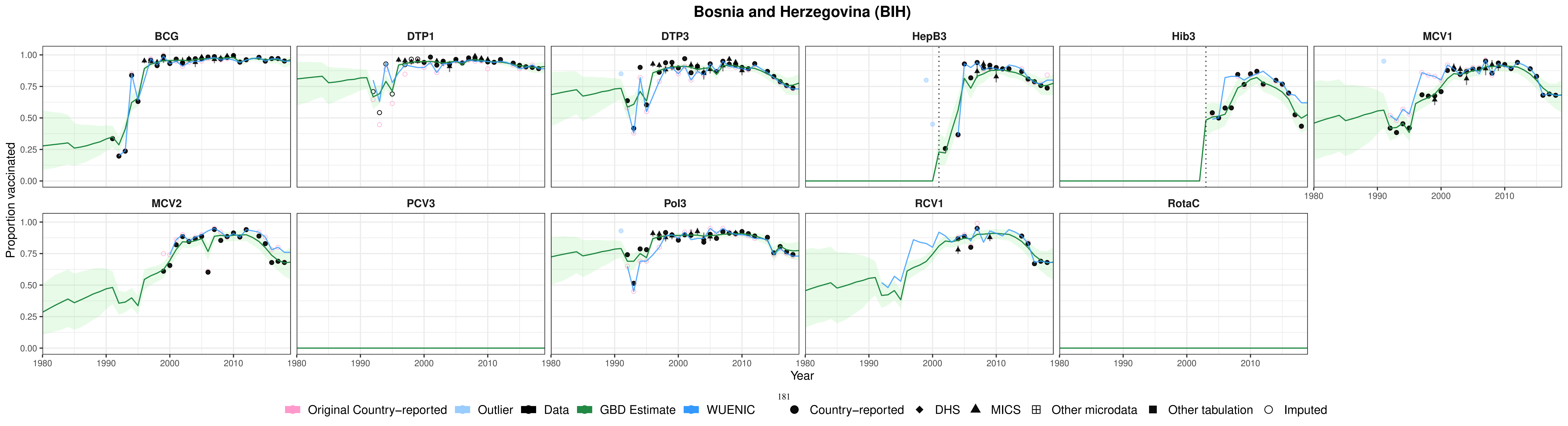
Bosnia and Herzegovina (BIH)
BCG
DTP1
DTP3
HepB3
Hib3
MCV1
MCV2
PCV3
Pol3
RCV1
RotaC
Proportion vaccinated
Year
0.00
0.25
0.50
0.75
1.00
1980
1990
2000
2010
Original Country−reported
Outlier
Data
GBD Estimate
WUENIC
Country−reported
DHS
MICS
Other microdata
Other tabulation
Imputed

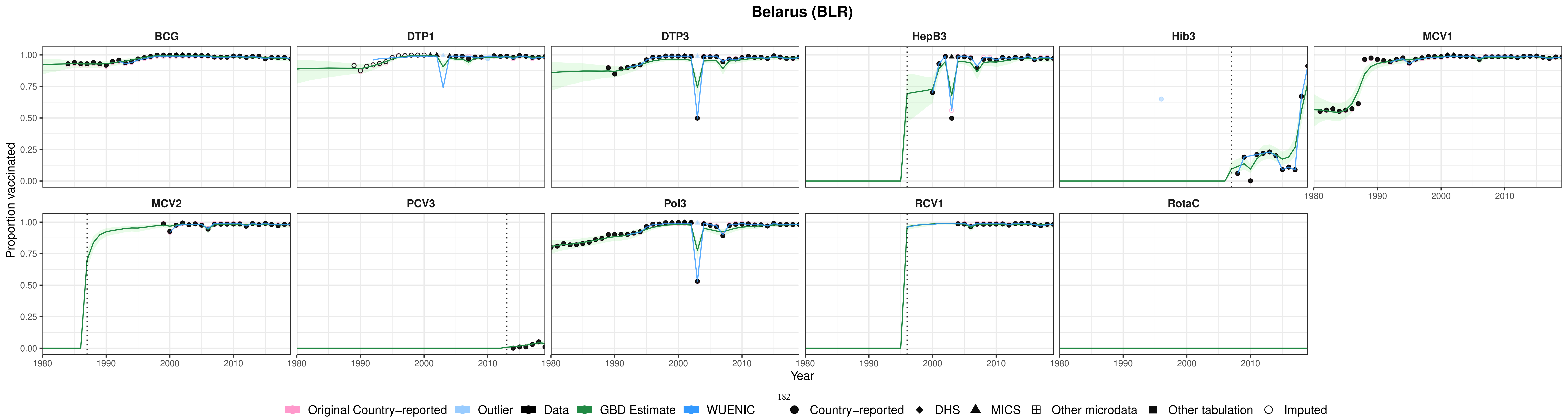

Belarus (BLR)
BCG
DTP1
DTP3
HepB3
Hib3
MCV1
MCV2
PCV3
Pol3
RCV1
RotaC
Proportion vaccinated
Year
Original Country−reported
Outlier
Data
GBD Estimate
WUENIC
Country−reported
DHS
MICS
Other microdata
Other tabulation
Imputed

Belize (BLZ)

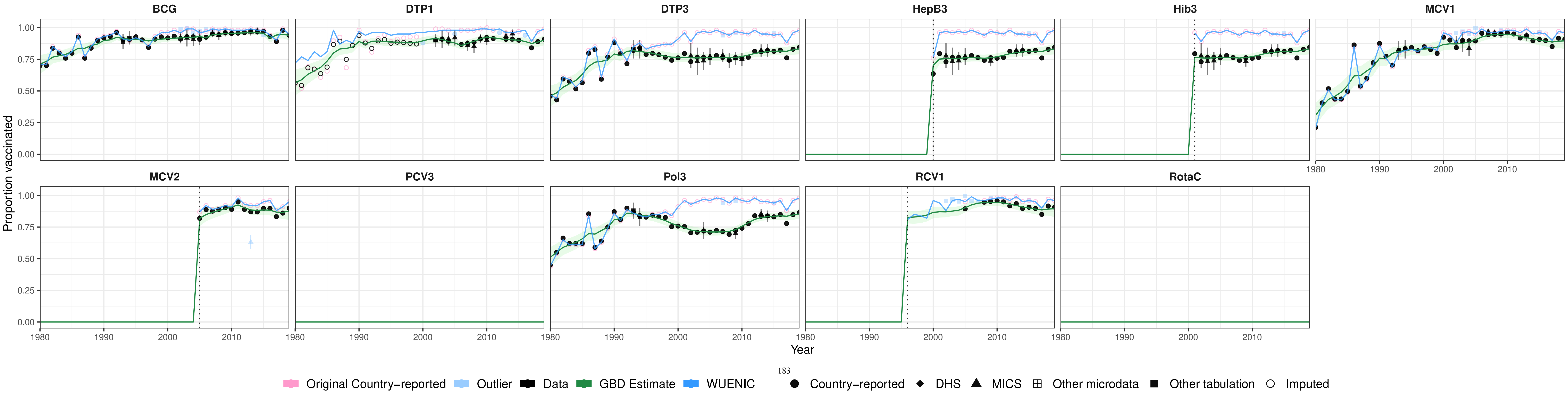

BCG
DTP1
DTP3
HepB3
Hib3
MCV1
MCV2
PCV3
Pol3
RCV1
RotaC
Proportion vaccinated
Year
0.00
0.25
0.50
0.75
1.00
1980
1990
2000
2010
Original Country−reported
Outlier
Data
GBD Estimate
WUENIC
Country−reported
DHS
MICS
Other microdata
Other tabulation
Imputed

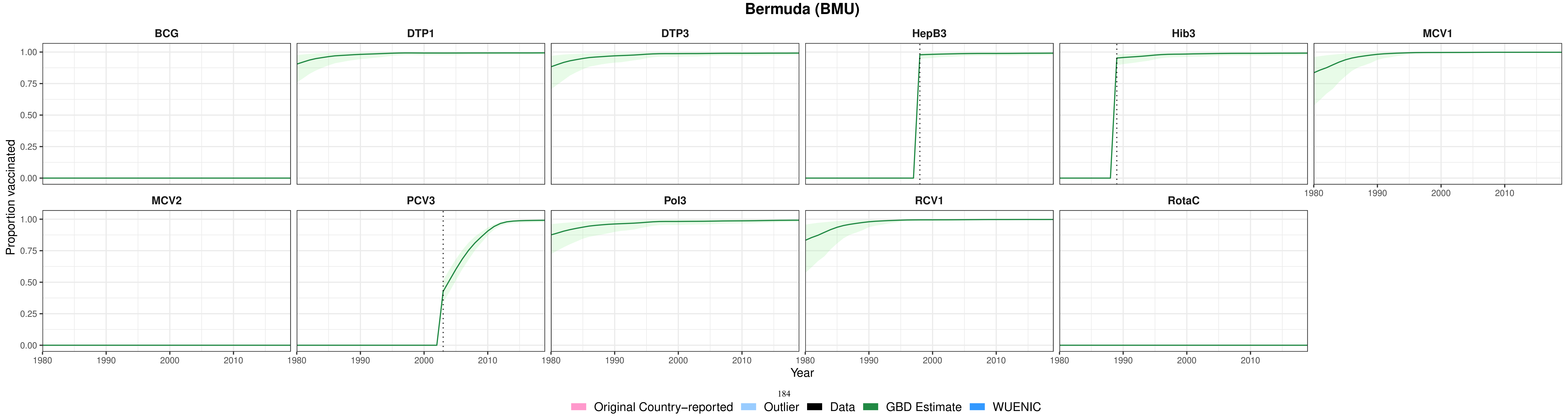

Bermuda (BMU)
BCG
DTP1
DTP3
HepB3
Hib3
MCV1
MCV2
PCV3
Pol3
RCV1
RotaC
Proportion vaccinated
0.00
0.25
0.50
0.75
1.00
1980
1990
2000
2010
Year
Original Country−reported
Outlier
Data
GBD Estimate
WUENIC

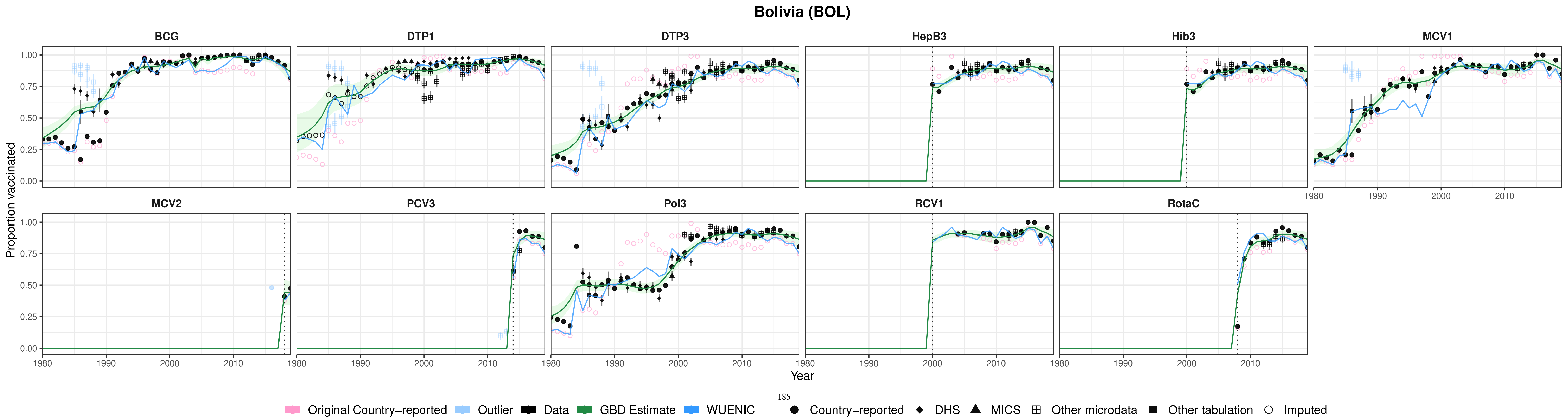
Bolivia (BOL)
BCG
DTP1
DTP3
HepB3
Hib3
MCV1
MCV2
PCV3
Pol3
RCV1
RotaC
Proportion vaccinated
Year
0.00
0.25
0.50
0.75
1.00
1980
1990
2000
2010
Original Country−reported
Outlier
Data
GBD Estimate
WUENIC
Country−reported
DHS
MICS
Other microdata
Other tabulation
Imputed

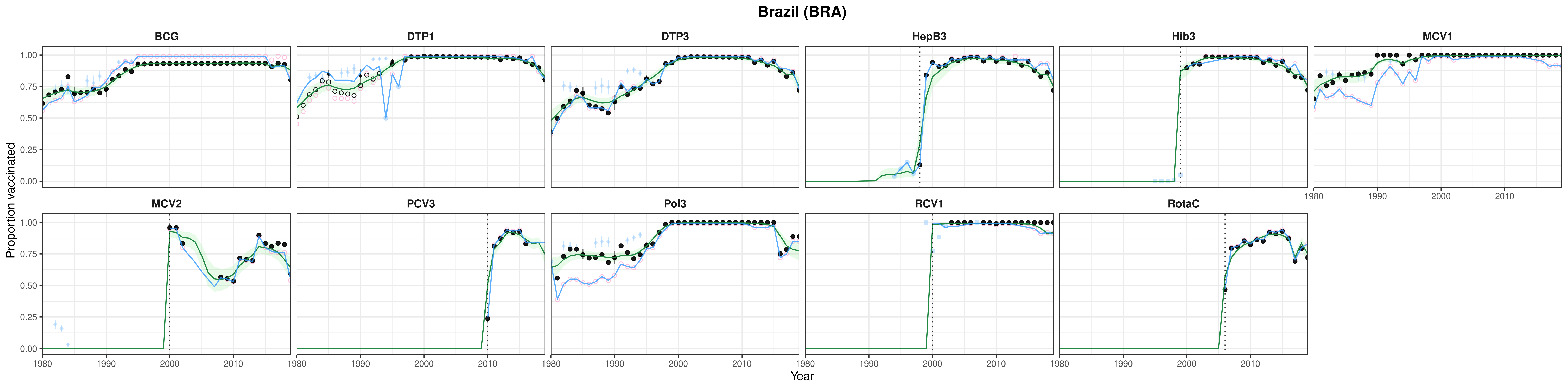
Brazil (BRA)
BCG
DTP1
DTP3
HepB3
Hib3
MCV1
MCV2
PCV3
Pol3
RCV1
RotaC
Proportion vaccinated
Year
Original Country−reported
Outlier
Data
GBD Estimate
WUENIC
Country−reported
DHS
MICS
Other microdata
Other tabulation
Imputed

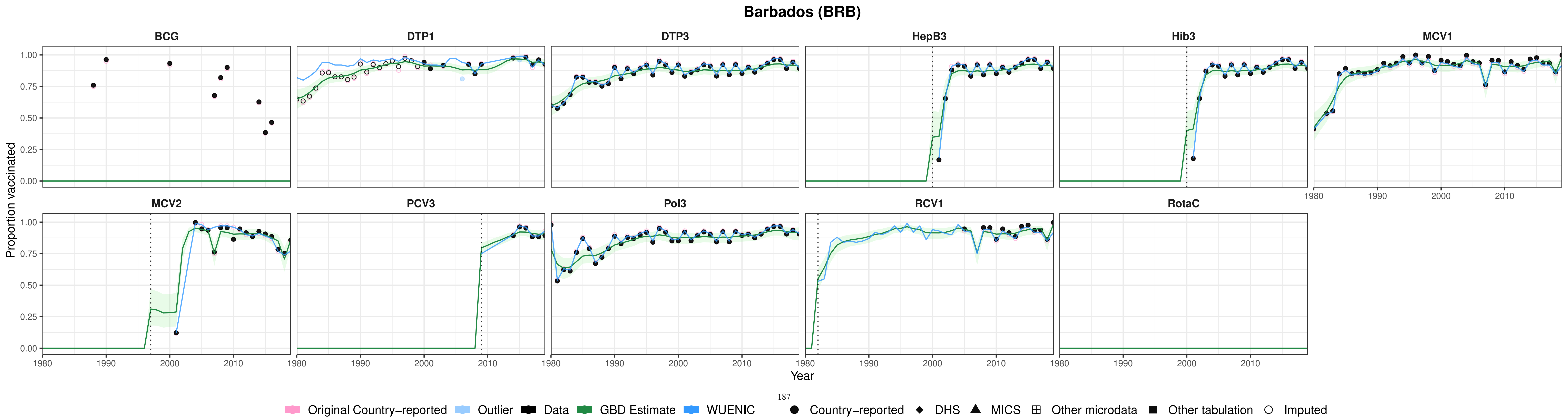

Barbados (BRB)
BCG
DTP1
DTP3
HepB3
Hib3
MCV1
MCV2
PCV3
Pol3
RCV1
RotaC
Proportion vaccinated
Year
Original Country−reported
Outlier
Data
GBD Estimate
WUENIC
Country−reported
DHS
MICS
Other microdata
Other tabulation
Imputed

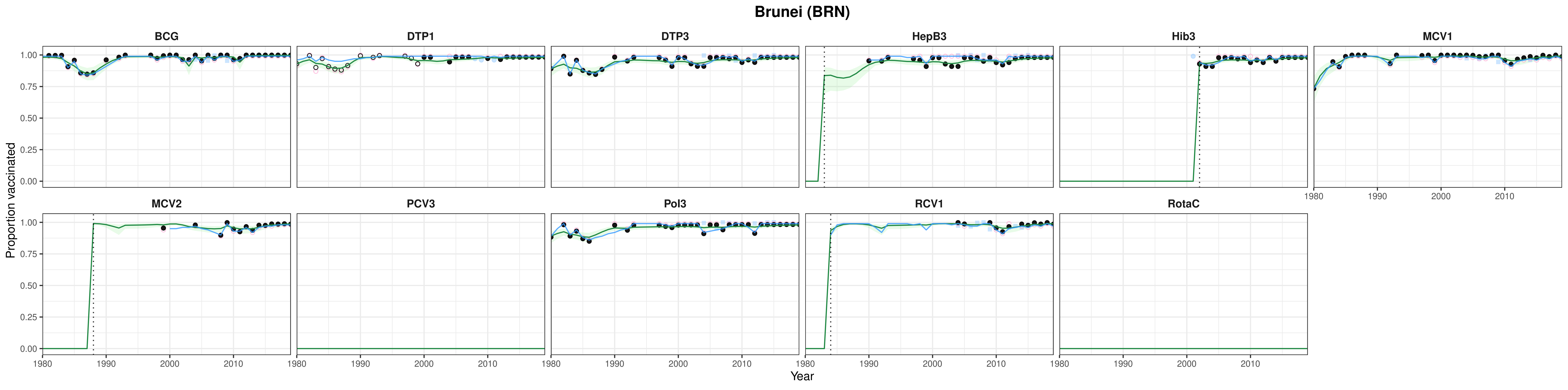
Brunei (BRN)
BCG
DTP1
DTP3
HepB3
Hib3
MCV1
MCV2
PCV3
Pol3
RCV1
RotaC
Proportion vaccinated
0.00
0.25
0.50
0.75
1.00
1980
1990
2000
2010
Year
Original Country−reported
Outlier
Data
GBD Estimate
WUENIC
Country−reported
DHS
MICS
Other microdata
Other tabulation
Imputed

Bhutan (BTN)

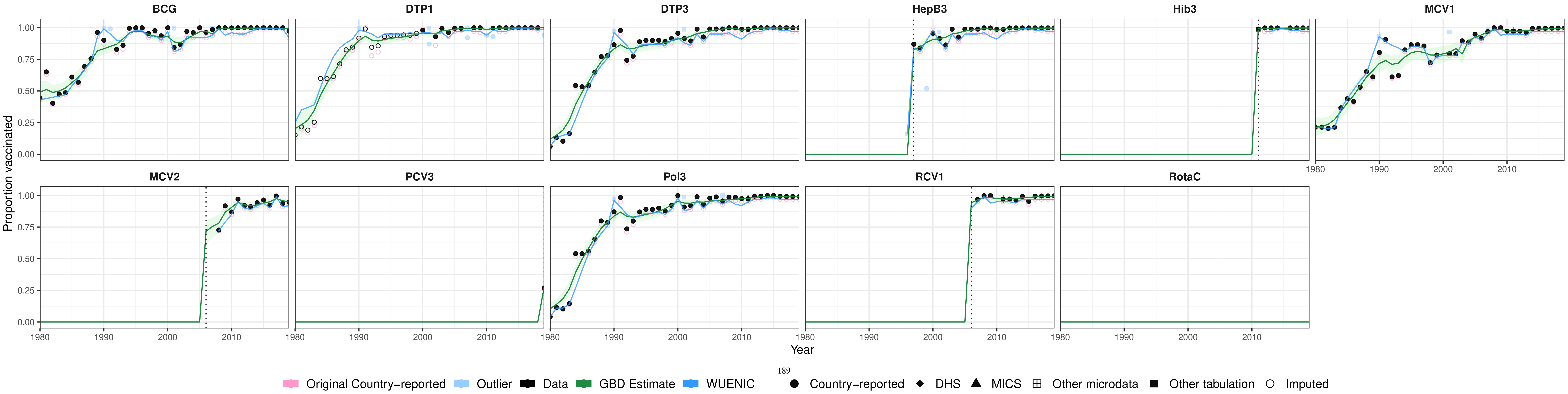
BCG
DTP1
DTP3
HepB3
Hib3
MCV1
MCV2
PCV3
Pol3
RCV1
RotaC
Proportion vaccinated
Year
1.00
0.75
0.50
0.25
0.00
1980
1990
2000
2010
Original Country−reported
Outlier
Data
GBD Estimate
WUENIC
Country−reported
DHS
MICS
Other microdata
Other tabulation
Imputed

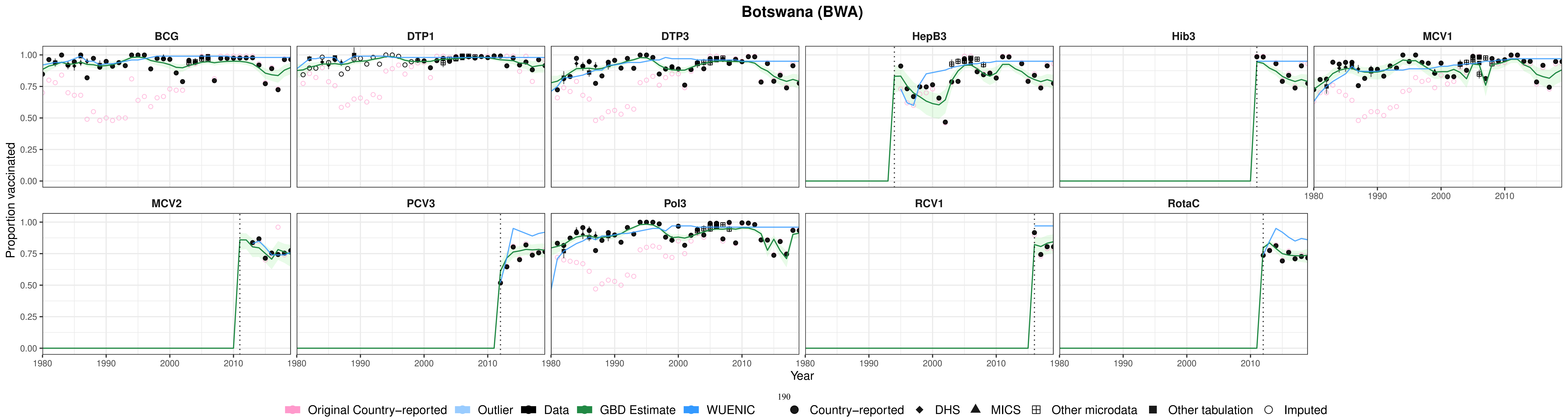

Botswana (BWA)
BCG
DTP1
DTP3
HepB3
Hib3
MCV1
MCV2
PCV3
Pol3
RCV1
RotaC
Proportion vaccinated
Year
Original Country−reported
Outlier
Data
GBD Estimate
WUENIC
Country−reported
DHS
MICS
Other microdata
Other tabulation
Imputed

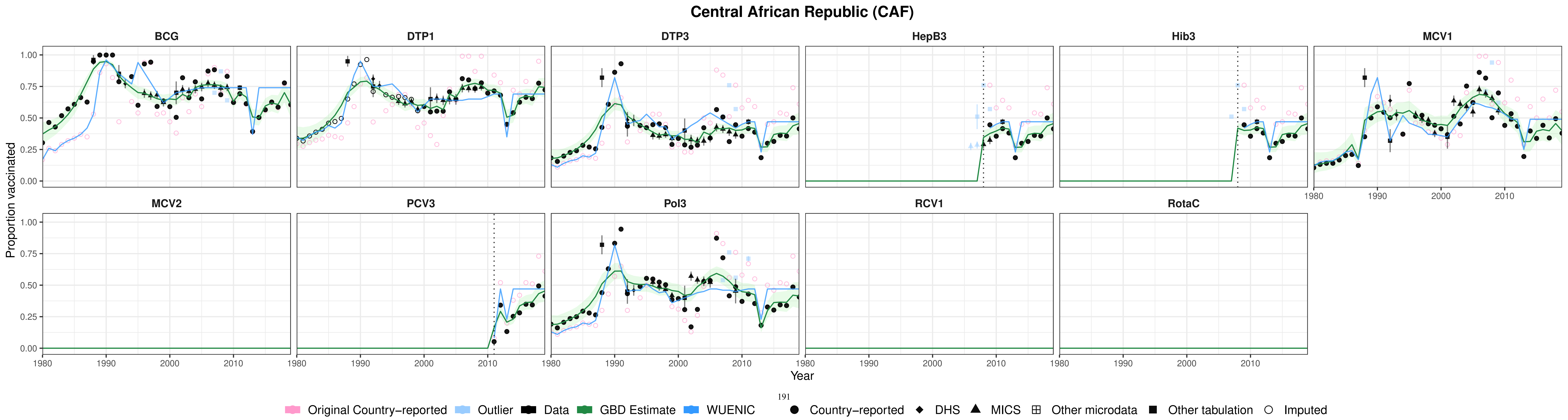
Central African Republic (CAF)
BCG
DTP1
DTP3
HepB3
Hib3
MCV1
MCV2
PCV3
Pol3
RCV1
RotaC
Proportion vaccinated
1.00
0.75
0.50
0.25
0.00
1980
1990
2000
2010
Year
Original Country−reported
Outlier
Data
GBD Estimate
WUENIC
Country−reported
DHS
MICS
Other microdata
Other tabulation
Imputed

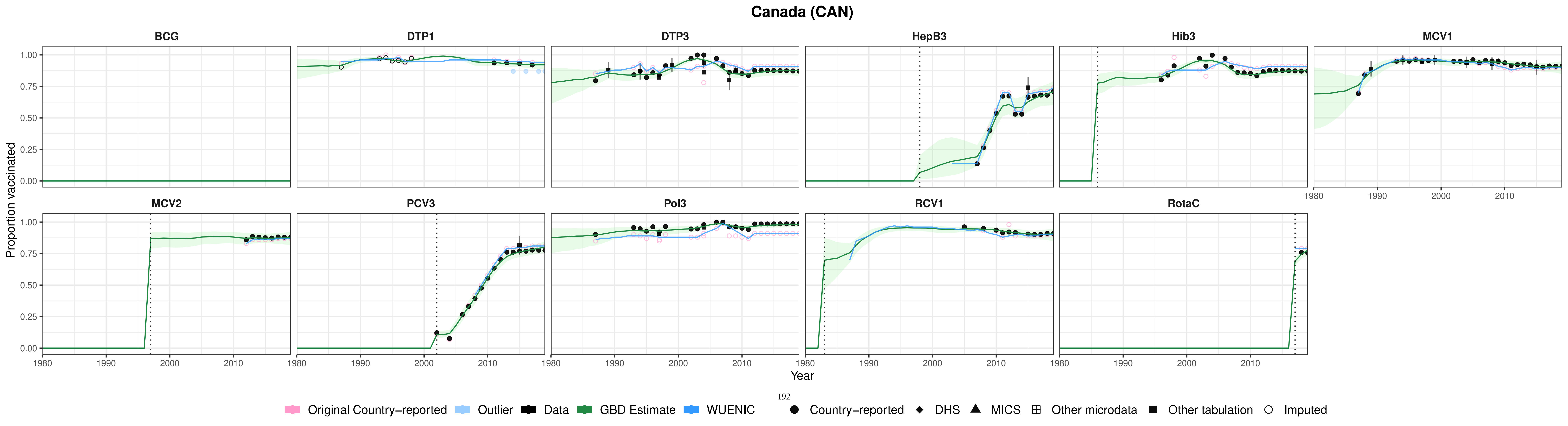

Canada (CAN)
BCG
DTP1
DTP3
HepB3
Hib3
MCV1
MCV2
PCV3
Pol3
RCV1
RotaC
Proportion vaccinated
Year
Original Country−reported
Outlier
Data
GBD Estimate
WUENIC
Country−reported
DHS
MICS
Other microdata
Other tabulation
Imputed

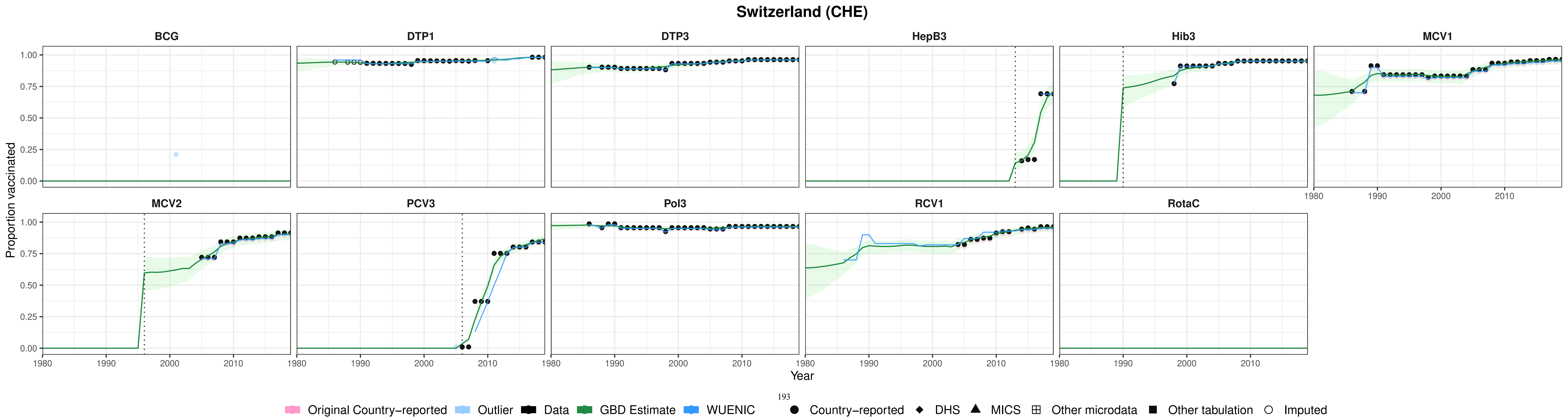
Switzerland (CHE)
BCG
DTP1
DTP3
HepB3
Hib3
MCV1
MCV2
PCV3
Pol3
RCV1
RotaC
Proportion vaccinated
Year
Original Country−reported
Outlier
Data
GBD Estimate
WUENIC
Country−reported
DHS
MICS
Other microdata
Other tabulation
Imputed

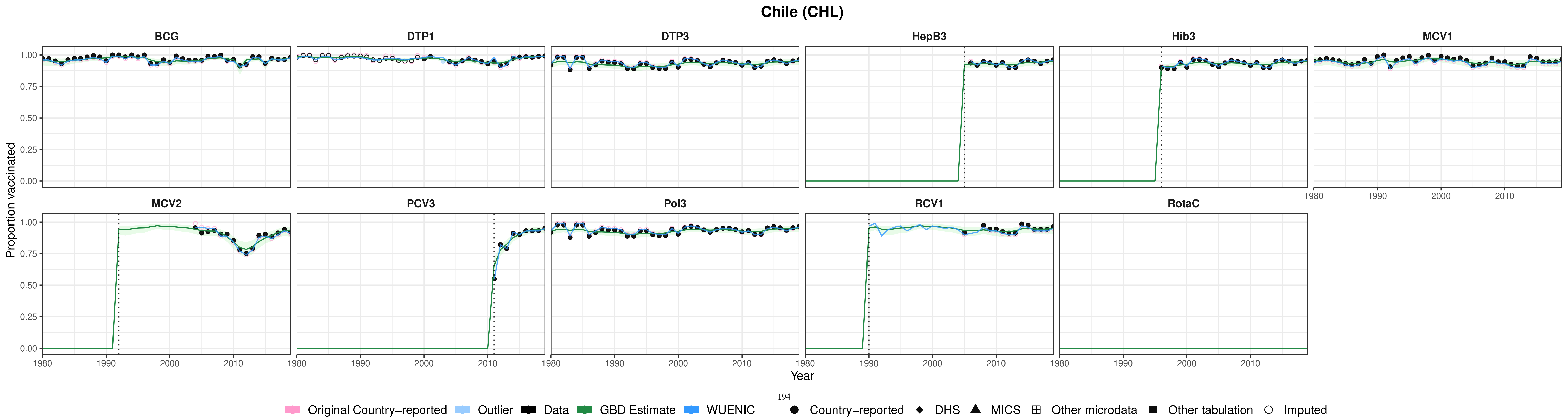

Chile (CHL)
BCG
DTP1
DTP3
HepB3
Hib3
MCV1
MCV2
PCV3
Pol3
RCV1
RotaC
Proportion vaccinated
Year
1.00
0.75
0.50
0.25
0.00
1980
1990
2000
2010
Original Country−reported
Outlier
Data
GBD Estimate
WUENIC
Country−reported
DHS
MICS
Other microdata
Other tabulation
Imputed

China (CHN)

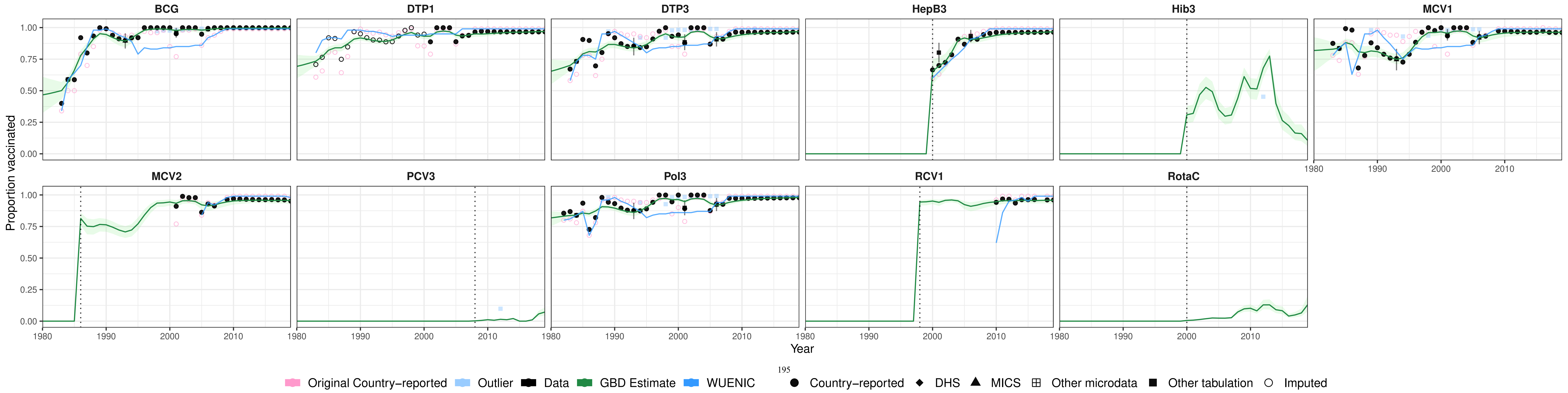

BCG
DTP1
DTP3
HepB3
Hib3
MCV1
MCV2
PCV3
Pol3
RCV1
RotaC
Proportion vaccinated
Year
Original Country−reported
Outlier
Data
GBD Estimate
WUENIC
Country−reported
DHS
MICS
Other microdata
Other tabulation
Imputed

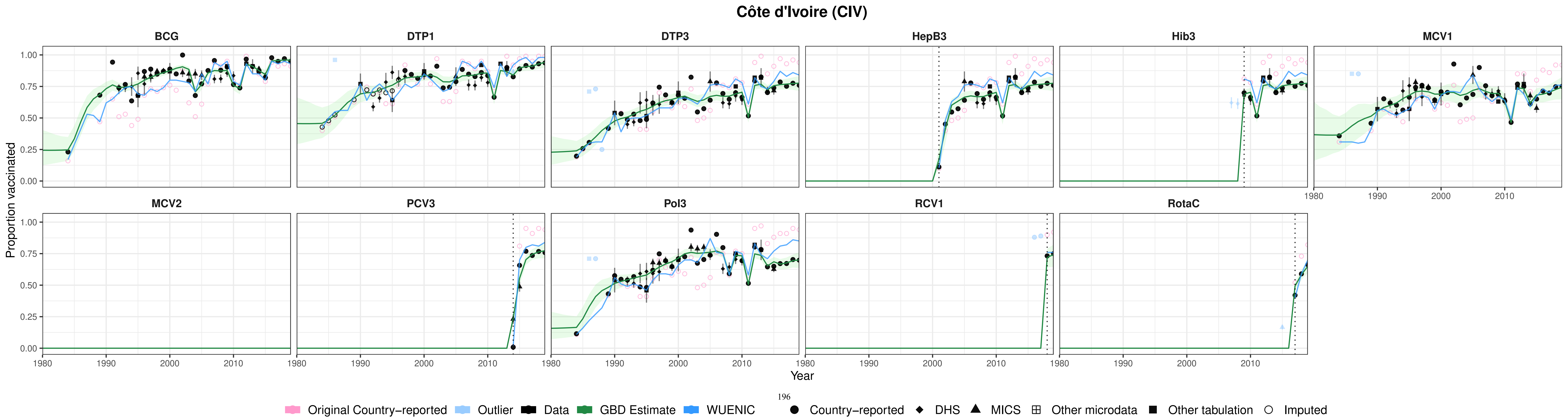

Côte d'Ivoire (CIV)
BCG
DTP1
DTP3
HepB3
Hib3
MCV1
MCV2
PCV3
Pol3
RCV1
RotaC
Proportion vaccinated
Year
Original Country−reported
Outlier
Data
GBD Estimate
WUENIC
Country−reported
DHS
MICS
Other microdata
Other tabulation
Imputed

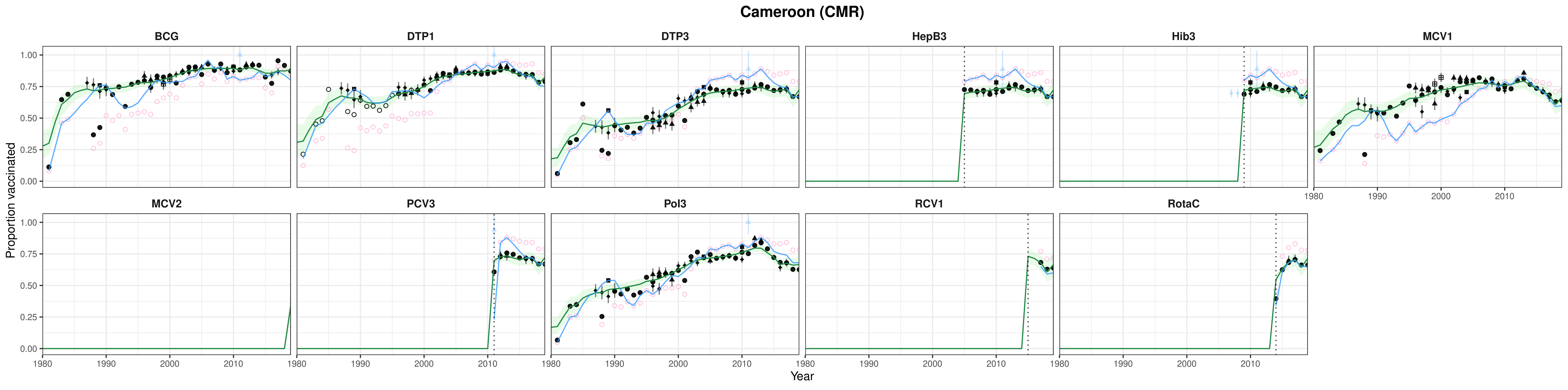

Cameroon (CMR)
BCG
DTP1
DTP3
HepB3
Hib3
MCV1
MCV2
PCV3
Pol3
RCV1
RotaC
Proportion vaccinated
0.00
0.25
0.50
0.75
1.00
1980
1990
2000
2010
Year
Original Country−reported
Outlier
Data
GBD Estimate
WUENIC
Country−reported
DHS
MICS
Other microdata
Other tabulation
Imputed

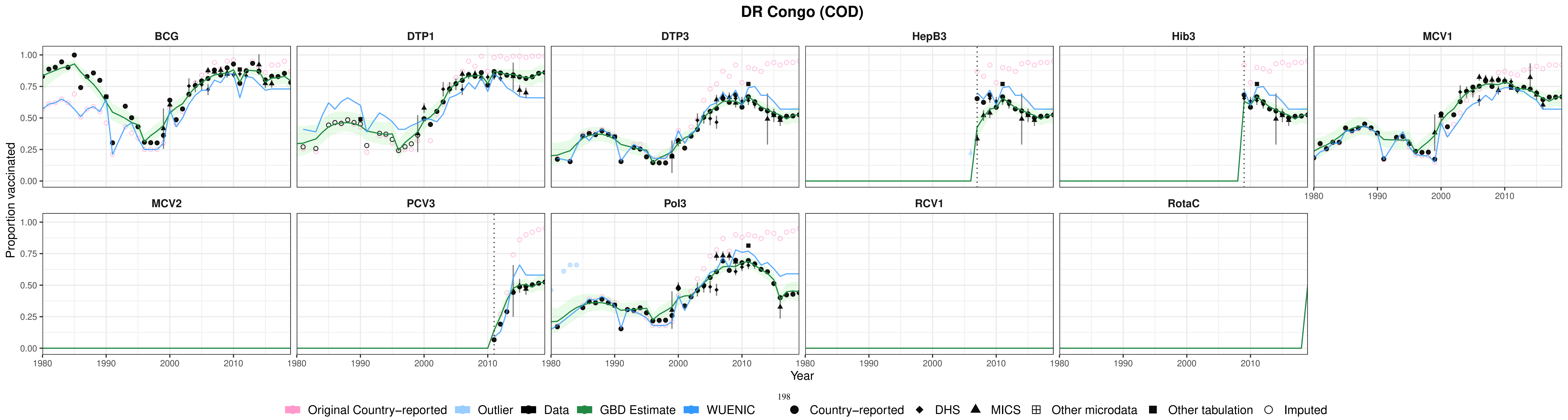
DR Congo (COD)
BCG
DTP1
DTP3
HepB3
Hib3
MCV1
MCV2
PCV3
Pol3
RCV1
RotaC
Proportion vaccinated
Year
Original Country−reported
Outlier
Data
GBD Estimate
WUENIC
Country−reported
DHS
MICS
Other microdata
Other tabulation
Imputed

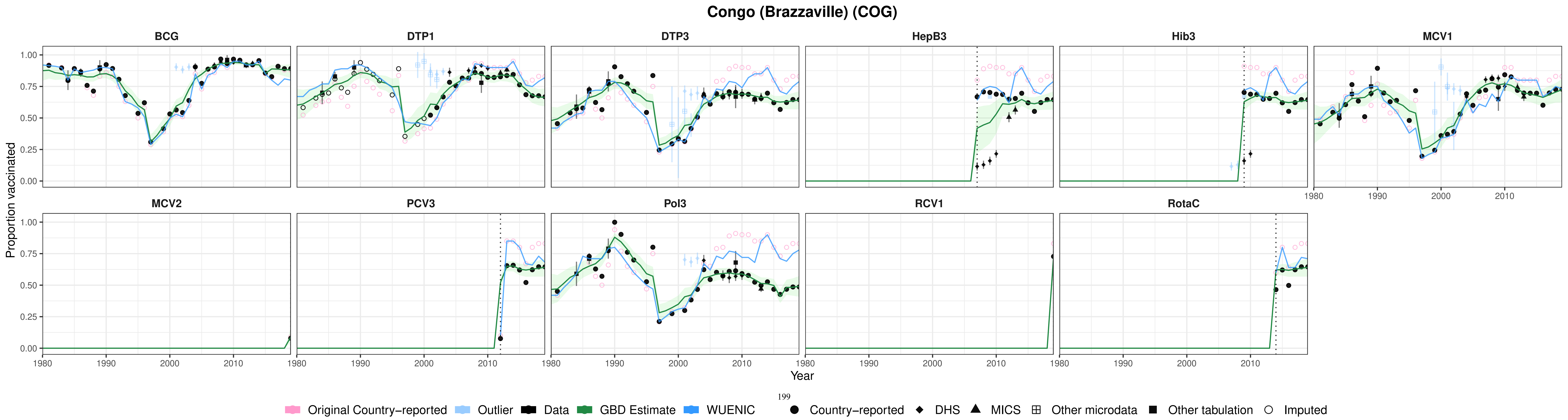

Congo (Brazzaville) (COG)
BCG
DTP1
DTP3
HepB3
Hib3
MCV1
MCV2
PCV3
Pol3
RCV1
RotaC
Proportion vaccinated
Year
Original Country−reported
Outlier
Data
GBD Estimate
WUENIC
Country−reported
DHS
MICS
Other microdata
Other tabulation
Imputed

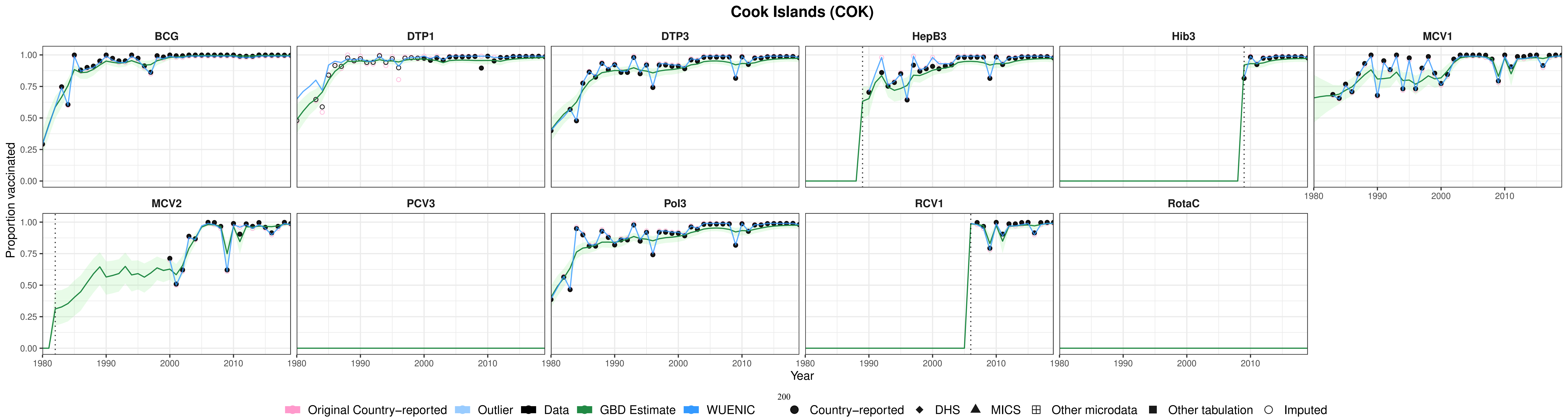
Cook Islands (COK)
BCG
DTP1
DTP3
HepB3
Hib3
MCV1
MCV2
PCV3
Pol3
RCV1
RotaC
Proportion vaccinated
Year
0.00
0.25
0.50
0.75
1.00
1980
1990
2000
2010
Original Country−reported
Outlier
Data
GBD Estimate
WUENIC
Country−reported
DHS
MICS
Other microdata
Other tabulation
Imputed

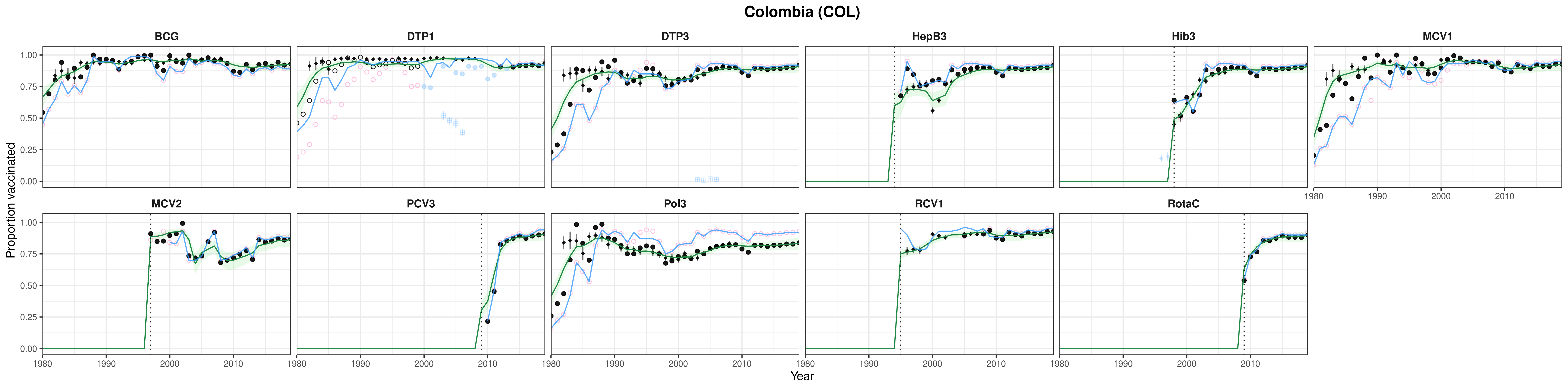
Colombia (COL)
BCG
DTP1
DTP3
HepB3
Hib3
MCV1
MCV2
PCV3
Pol3
RCV1
RotaC
Proportion vaccinated
Year
Original Country−reported
Outlier
Data
GBD Estimate
WUENIC
Country−reported
DHS
MICS
Other microdata
Other tabulation
Imputed

Comoros (COM)

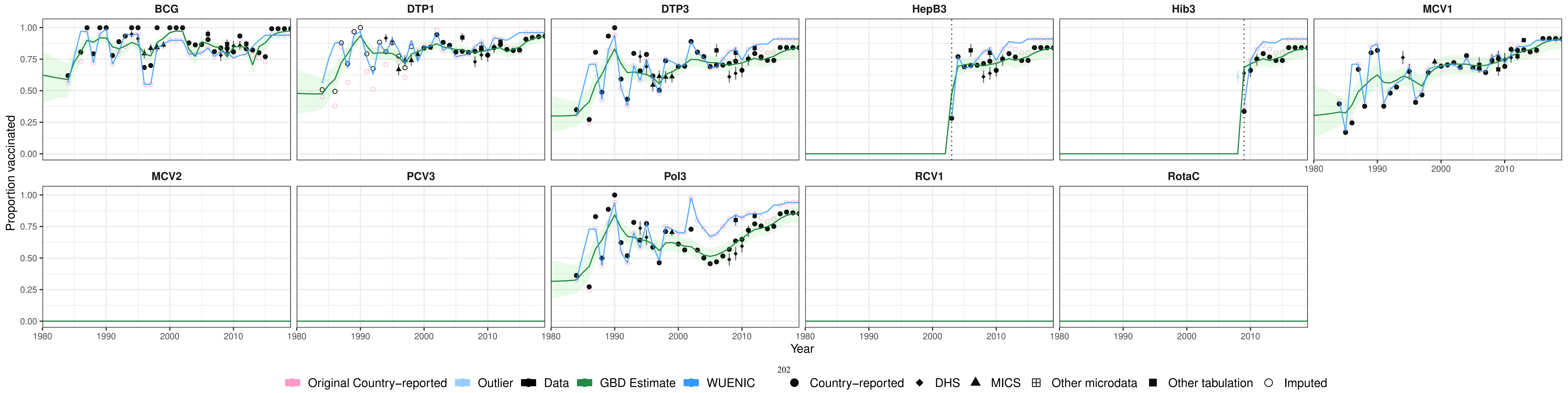

BCG
DTP1
DTP3
HepB3
Hib3
MCV1
MCV2
PCV3
Pol3
RCV1
RotaC
Proportion vaccinated
Year
Original Country-reported
Outlier
Data
GBD Estimate
WUENIC
Country-reported
DHS
MICS
Other microdata
Other tabulation
Imputed

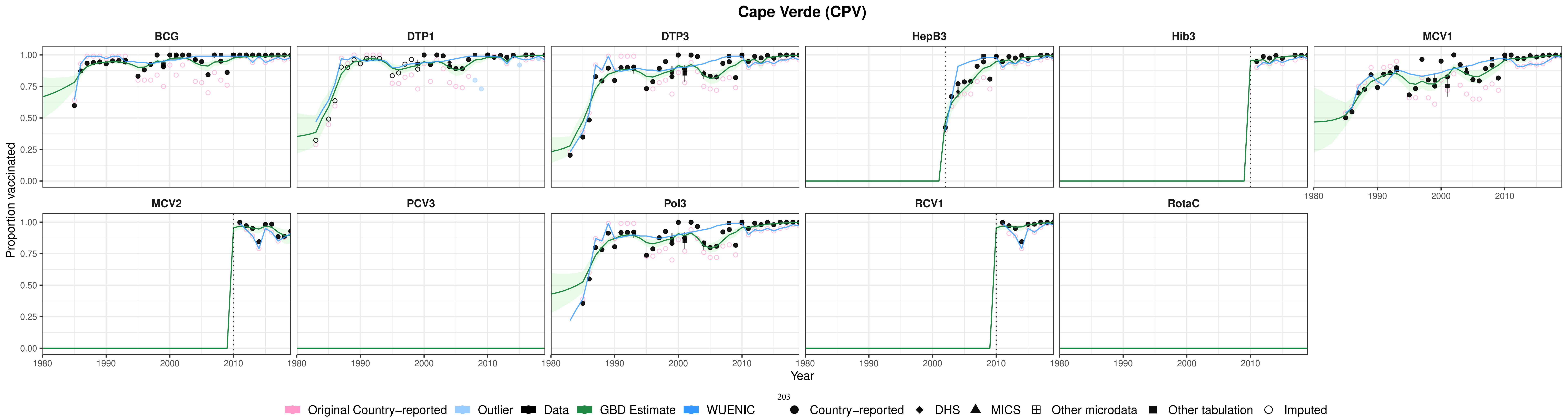
Cape Verde (CPV)
BCG
DTP1
DTP3
HepB3
Hib3
MCV1
MCV2
PCV3
Pol3
RCV1
RotaC
Proportion vaccinated
Year
Original Country−reported
Outlier
Data
GBD Estimate
WUENIC
Country−reported
DHS
MICS
Other microdata
Other tabulation
Imputed

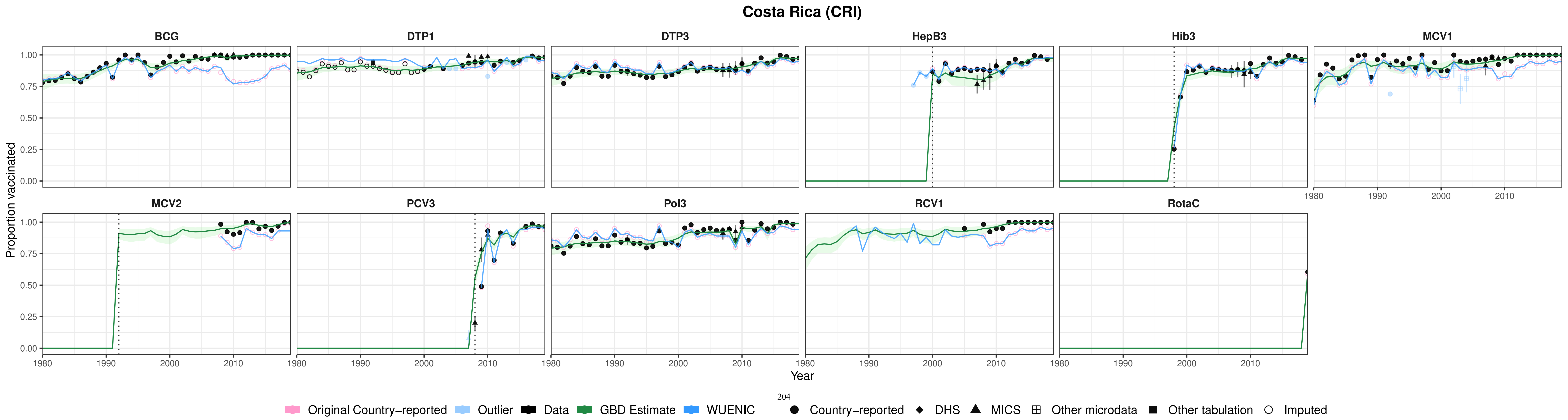

Costa Rica (CRI)
BCG
DTP1
DTP3
HepB3
Hib3
MCV1
MCV2
PCV3
Pol3
RCV1
RotaC
Proportion vaccinated
Year
Original Country−reported
Outlier
Data
GBD Estimate
WUENIC
Country−reported
DHS
MICS
Other microdata
Other tabulation
Imputed

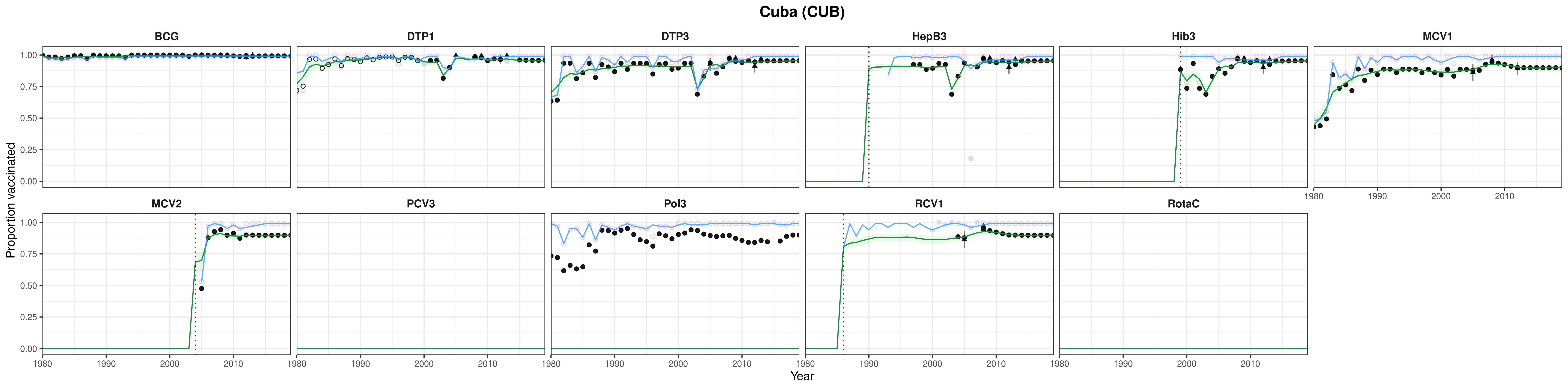
Cuba (CUB)
BCG
DTP1
DTP3
HepB3
Hib3
MCV1
MCV2
PCV3
Pol3
RCV1
RotaC
Proportion vaccinated
Year
0.00
0.25
0.50
0.75
1.00
1980
1990
2000
2010
Original Country−reported
Outlier
Data
GBD Estimate
WUENIC
Country−reported
DHS
MICS
Other microdata
Other tabulation
Imputed

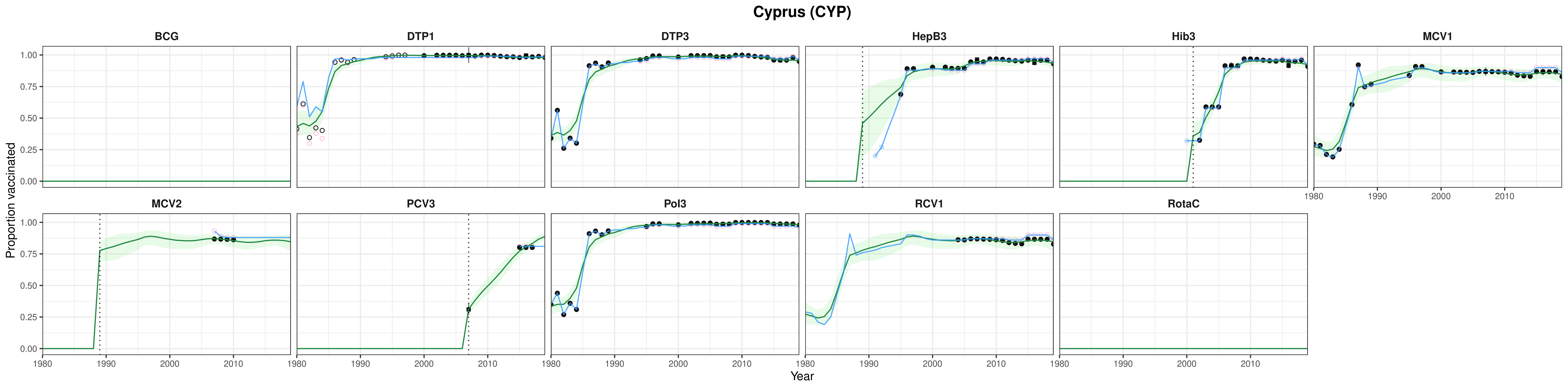

Cyprus (CYP)
BCG
DTP1
DTP3
HepB3
Hib3
MCV1
MCV2
PCV3
Pol3
RCV1
RotaC
Proportion vaccinated
Year
Original Country−reported
Outlier
Data
GBD Estimate
WUENIC
Country−reported
DHS
MICS
Other microdata
Other tabulation
Imputed

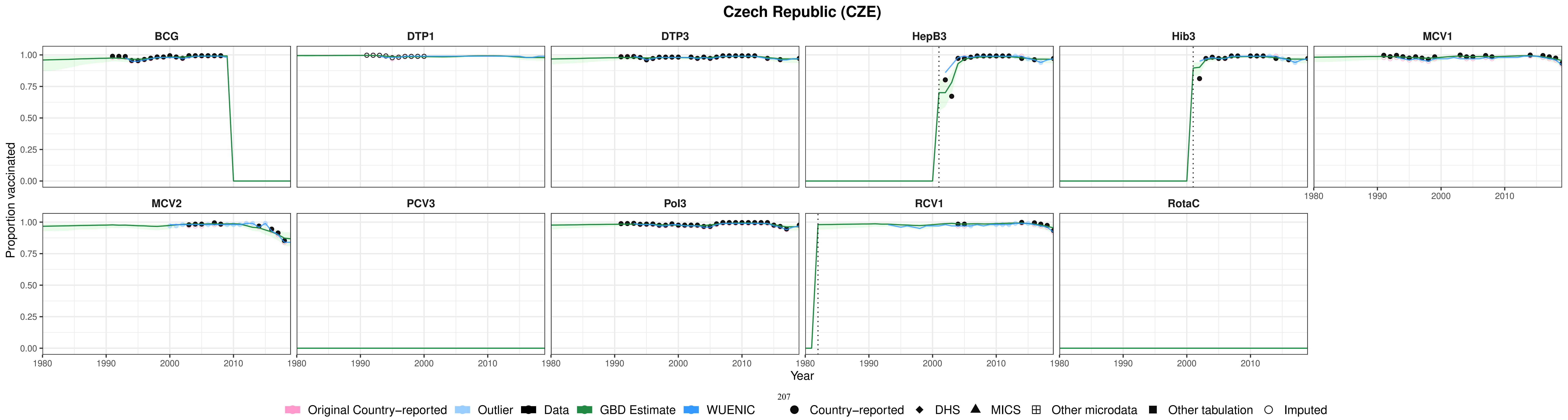

Czech Republic (CZE)
BCG
DTP1
DTP3
HepB3
Hib3
MCV1
MCV2
PCV3
Pol3
RCV1
RotaC
Proportion vaccinated
Year
Original Country−reported
Outlier
Data
GBD Estimate
WUENIC
Country−reported
DHS
MICS
Other microdata
Other tabulation
Imputed

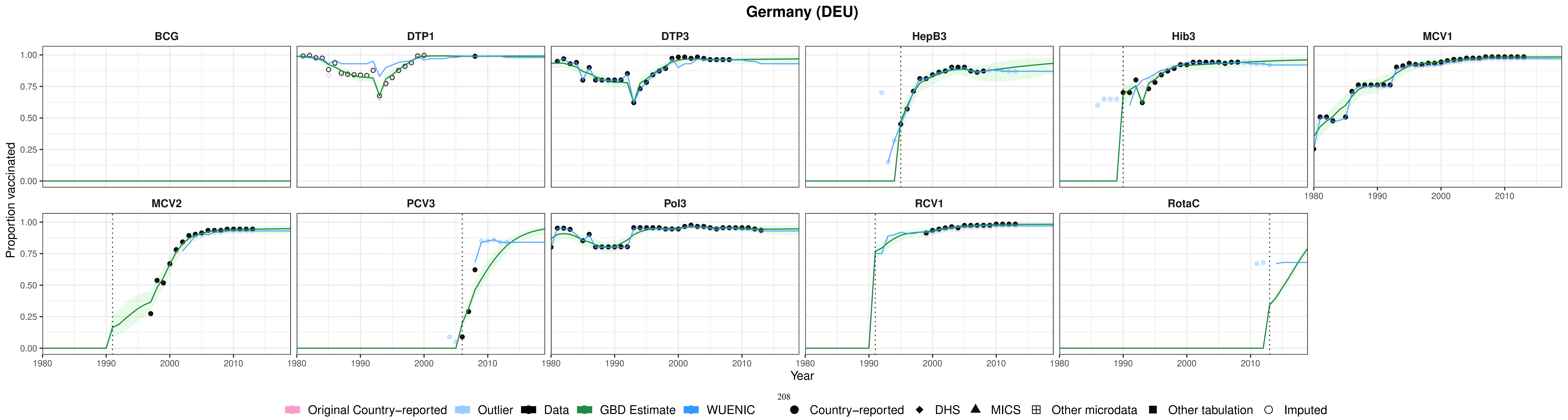
Germany (DEU)
BCG
DTP1
DTP3
HepB3
Hib3
MCV1
MCV2
PCV3
Pol3
RCV1
RotaC
Proportion vaccinated
Year
Original Country−reported
Outlier
Data
GBD Estimate
WUENIC
Country−reported
DHS
MICS
Other microdata
Other tabulation
Imputed

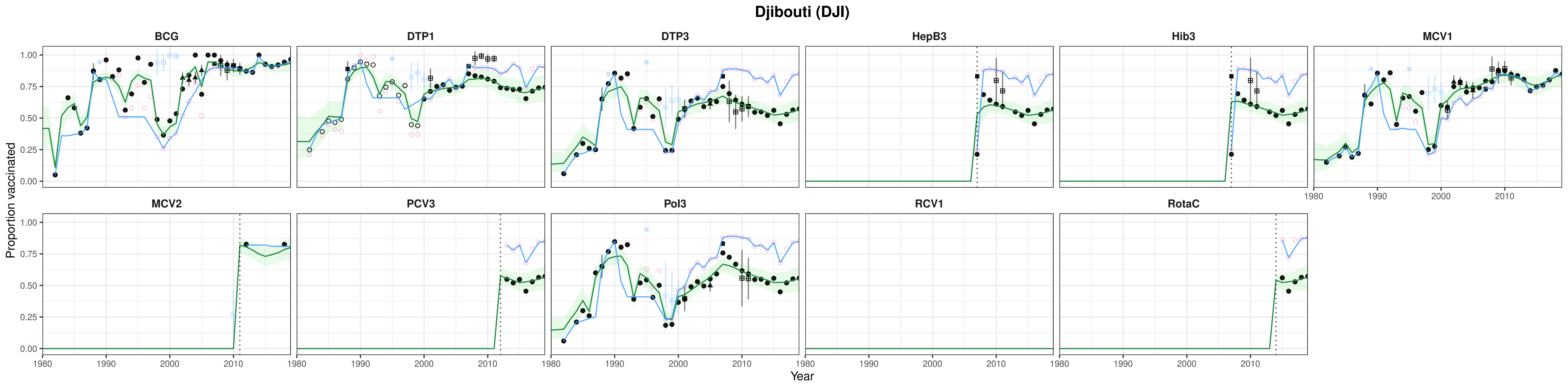
Djibouti (DJI)
BCG
DTP1
DTP3
HepB3
Hib3
MCV1
MCV2
PCV3
Pol3
RCV1
RotaC
Proportion vaccinated
Year
Original Country−reported
Outlier
Data
GBD Estimate
WUENIC
Country−reported
DHS
MICS
Other microdata
Other tabulation
Imputed

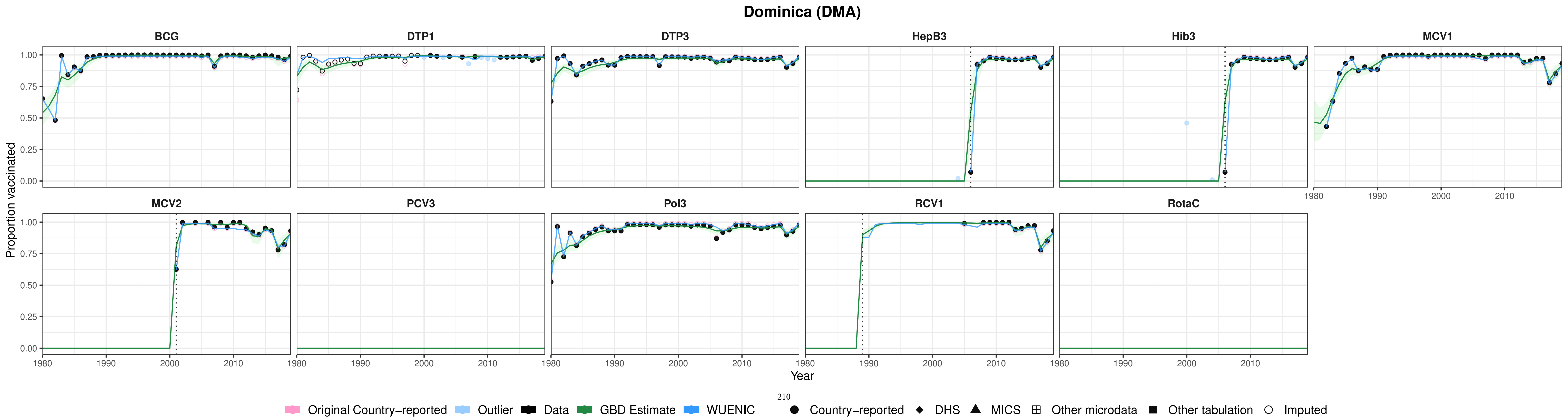
Dominica (DMA)
BCG
DTP1
DTP3
HepB3
Hib3
MCV1
MCV2
PCV3
Pol3
RCV1
RotaC
Proportion vaccinated
Year
Original Country−reported
Outlier
Data
GBD Estimate
WUENIC
Country−reported
DHS
MICS
Other microdata
Other tabulation
Imputed

Denmark (DNK)

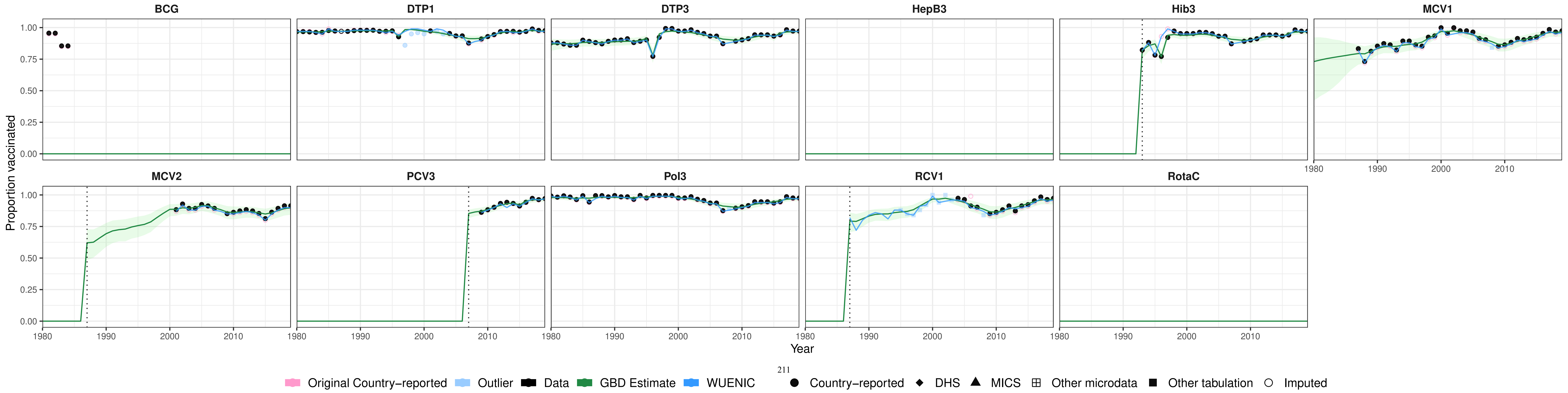
BCG
DTP1
DTP3
HepB3
Hib3
MCV1
MCV2
PCV3
Pol3
RCV1
RotaC
Proportion vaccinated
Year
0.00
0.25
0.50
0.75
1.00
1980
1990
2000
2010
Original Country−reported
Outlier
Data
GBD Estimate
WUENIC
Country−reported
DHS
MICS
Other microdata
Other tabulation
Imputed

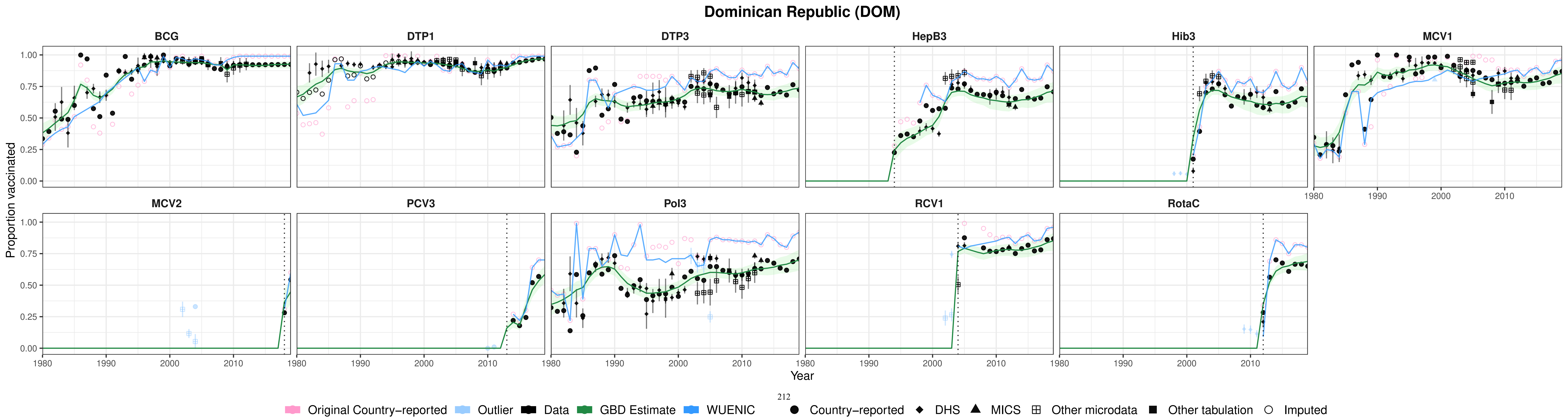
Dominican Republic (DOM)
BCG
DTP1
DTP3
HepB3
Hib3
MCV1
MCV2
PCV3
Pol3
RCV1
RotaC
Proportion vaccinated
Year
Original Country−reported
Outlier
Data
GBD Estimate
WUENIC
Country−reported
DHS
MICS
Other microdata
Other tabulation
Imputed

Algeria (DZA)

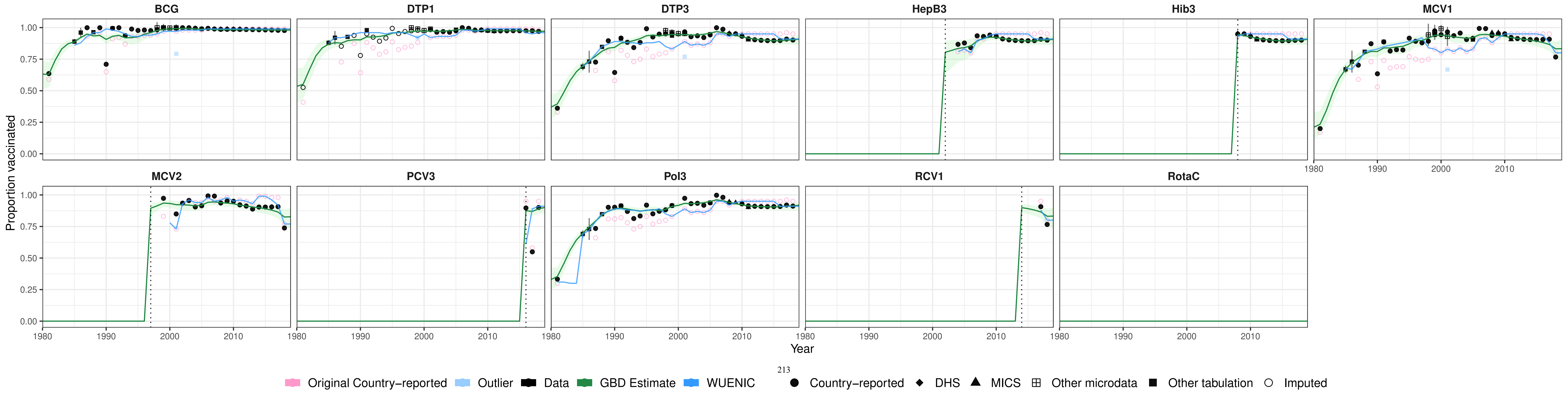

BCG
DTP1
DTP3
HepB3
Hib3
MCV1
MCV2
PCV3
Pol3
RCV1
RotaC
Proportion vaccinated
Year
Original Country−reported
Outlier
Data
GBD Estimate
WUENIC
Country−reported
DHS
MICS
Other microdata
Other tabulation
Imputed

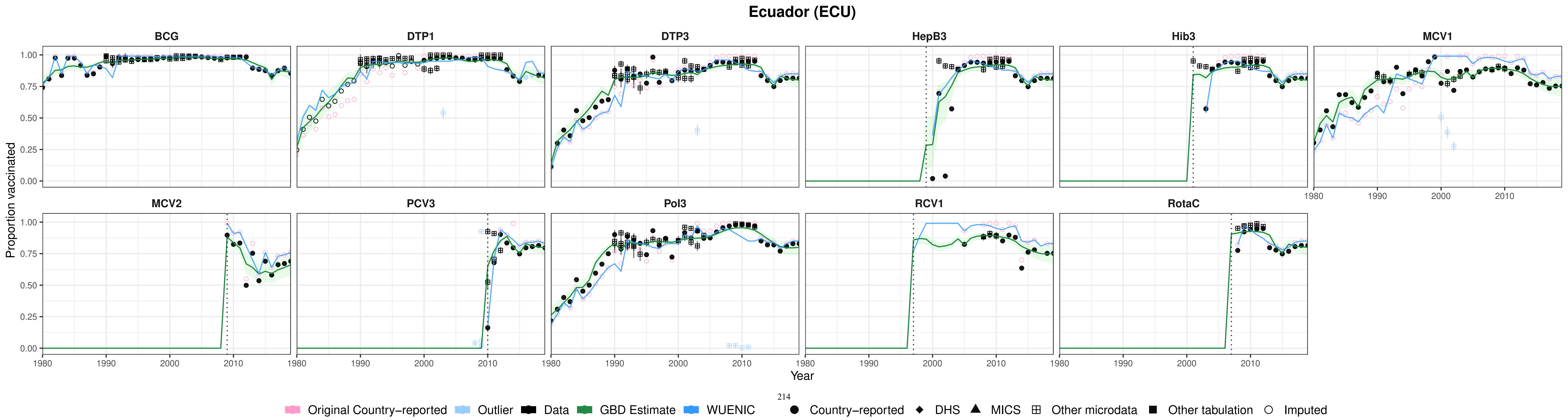
Ecuador (ECU)
BCG
DTP1
DTP3
HepB3
Hib3
MCV1
MCV2
PCV3
Pol3
RCV1
RotaC
Proportion vaccinated
Year
0.00
0.25
0.50
0.75
1.00
1980
1990
2000
2010
Original Country−reported
Outlier
Data
GBD Estimate
WUENIC
Country−reported
DHS
MICS
Other microdata
Other tabulation
Imputed

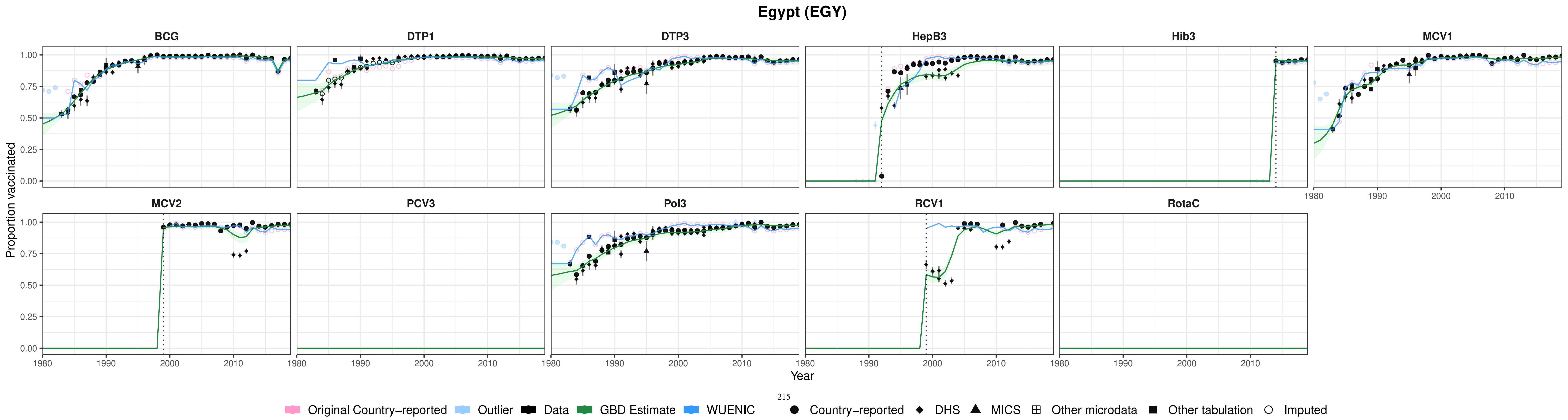
Egypt (EGY)
BCG
DTP1
DTP3
HepB3
Hib3
MCV1
MCV2
PCV3
Pol3
RCV1
RotaC
Proportion vaccinated
Year
Original Country−reported
Outlier
Data
GBD Estimate
WUENIC
Country−reported
DHS
MICS
Other microdata
Other tabulation
Imputed

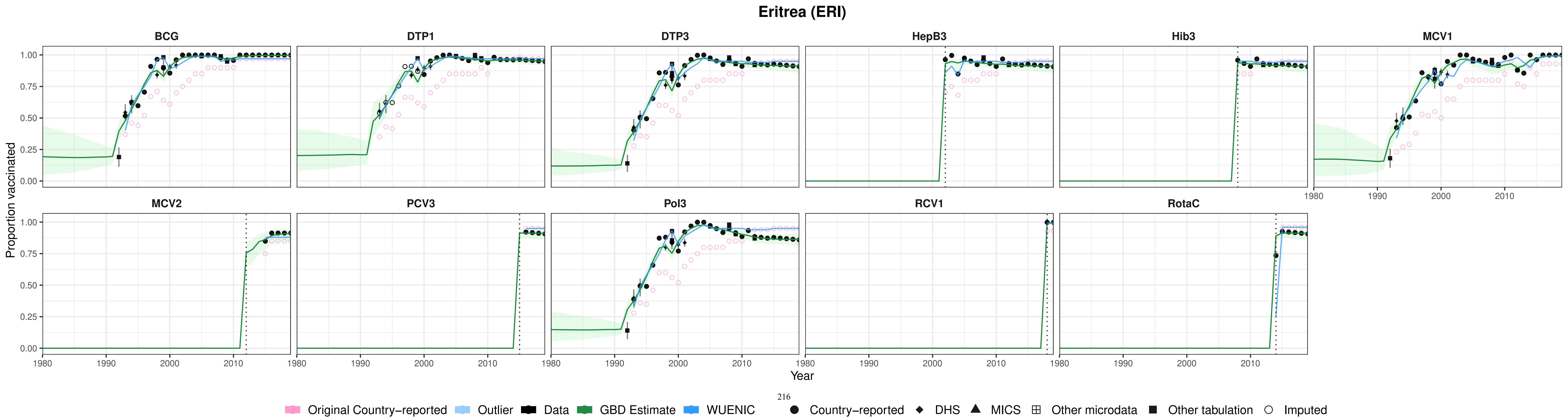
Eritrea (ERI)
BCG
DTP1
DTP3
HepB3
Hib3
MCV1
MCV2
PCV3
Pol3
RCV1
RotaC
Proportion vaccinated
0.00
0.25
0.50
0.75
1.00
1980
1990
2000
2010
Year
Original Country−reported
Outlier
Data
GBD Estimate
WUENIC
Country−reported
DHS
MICS
Other microdata
Other tabulation
Imputed

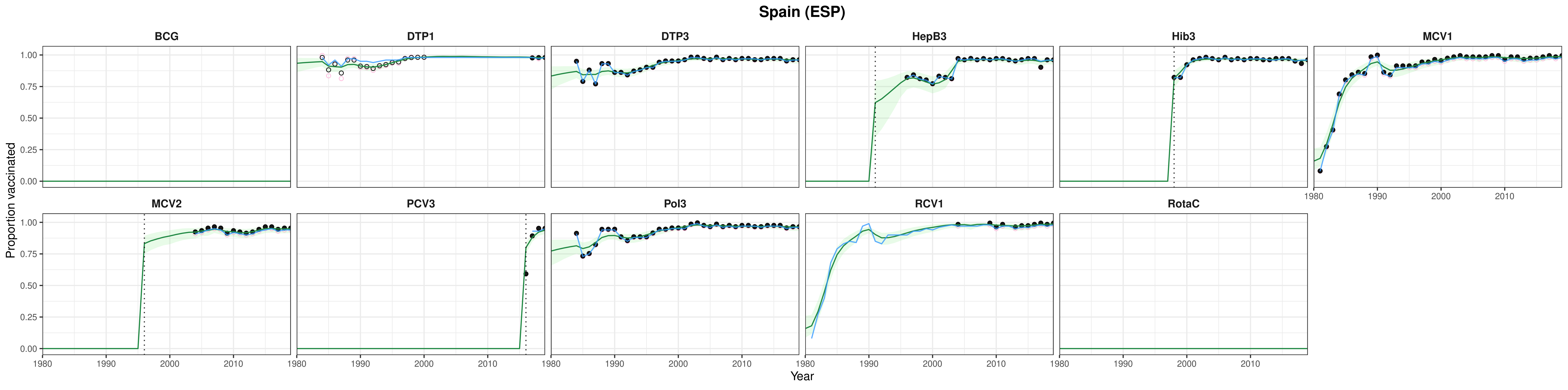
Spain (ESP)
BCG
DTP1
DTP3
HepB3
Hib3
MCV1
MCV2
PCV3
Pol3
RCV1
RotaC
Proportion vaccinated
1.00
0.75
0.50
0.25
0.00
1980
1990
2000
2010
Year
Original Country−reported
Outlier
Data
GBD Estimate
WUENIC
Country−reported
DHS
MICS
Other microdata
Other tabulation
Imputed

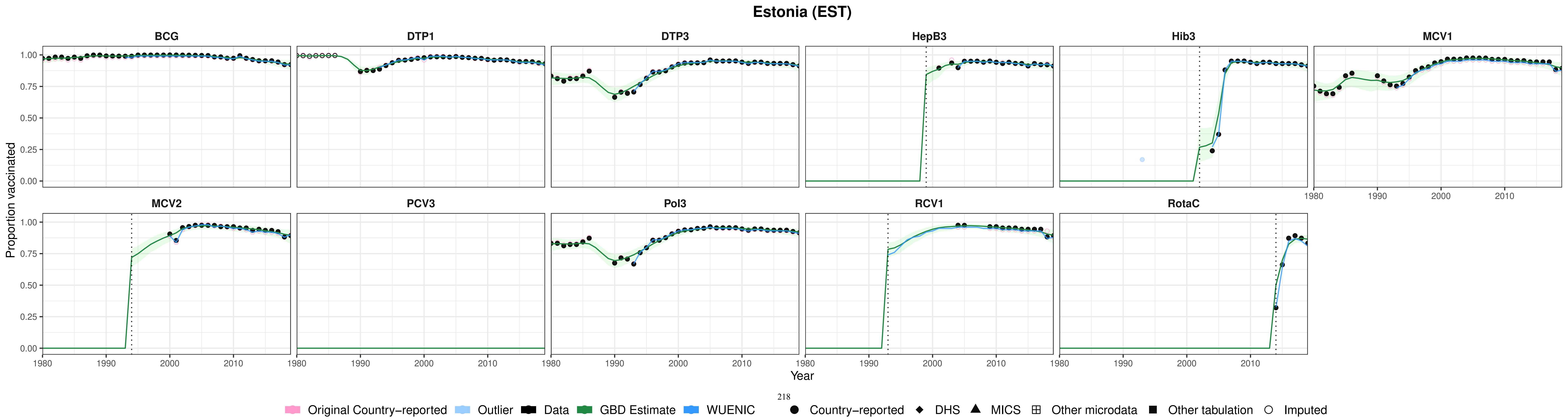
Estonia (EST)
BCG
DTP1
DTP3
HepB3
Hib3
MCV1
MCV2
PCV3
Pol3
RCV1
RotaC
Proportion vaccinated
Year
Original Country−reported
Outlier
Data
GBD Estimate
WUENIC
Country−reported
DHS
MICS
Other microdata
Other tabulation
Imputed

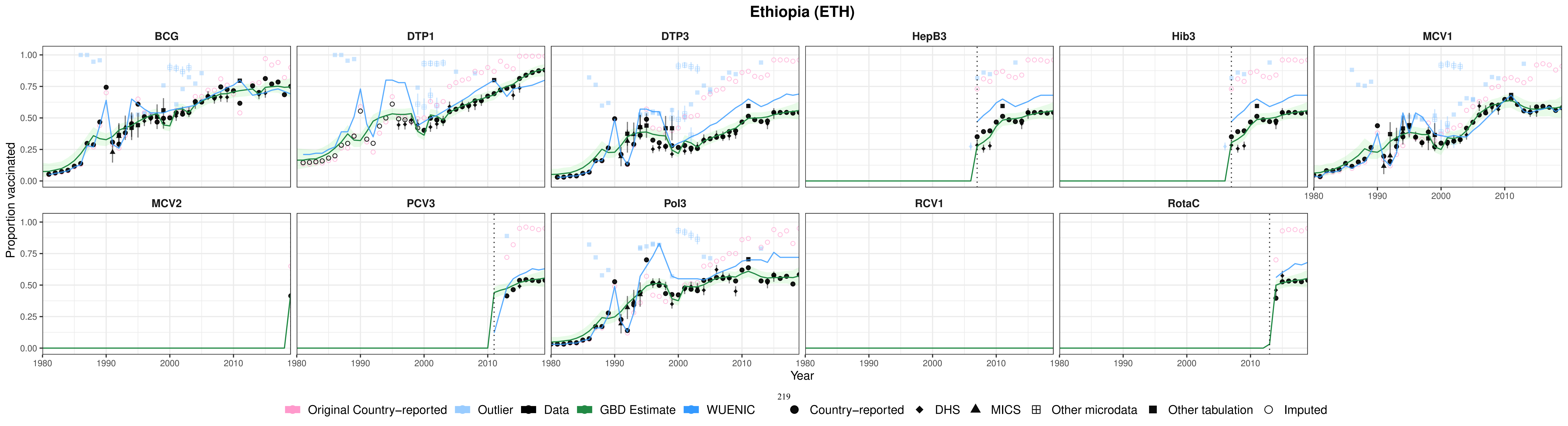

Ethiopia (ETH)
BCG
DTP1
DTP3
HepB3
Hib3
MCV1
MCV2
PCV3
Pol3
RCV1
RotaC
Proportion vaccinated
Year
0.00
0.25
0.50
0.75
1.00
1980
1990
2000
2010
Original Country−reported
Outlier
Data
GBD Estimate
WUENIC
Country−reported
DHS
MICS
Other microdata
Other tabulation
Imputed

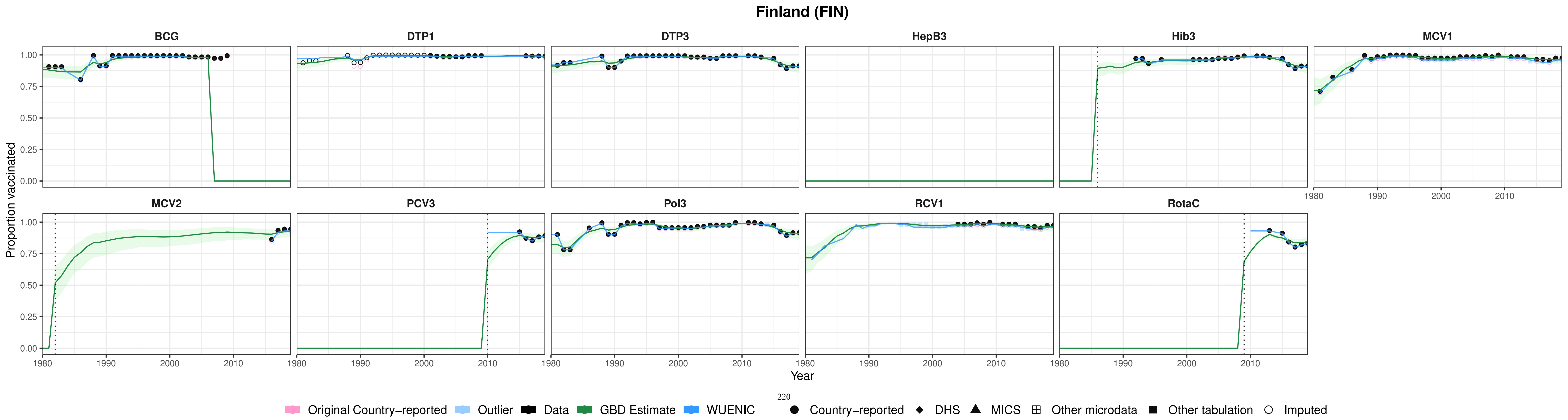

Finland (FIN)
BCG
DTP1
DTP3
HepB3
Hib3
MCV1
MCV2
PCV3
Pol3
RCV1
RotaC
Proportion vaccinated
1.00
0.75
0.50
0.25
0.00
1980
1990
2000
2010
Year
Original Country−reported
Outlier
Data
GBD Estimate
WUENIC
Country−reported
DHS
MICS
Other microdata
Other tabulation
Imputed

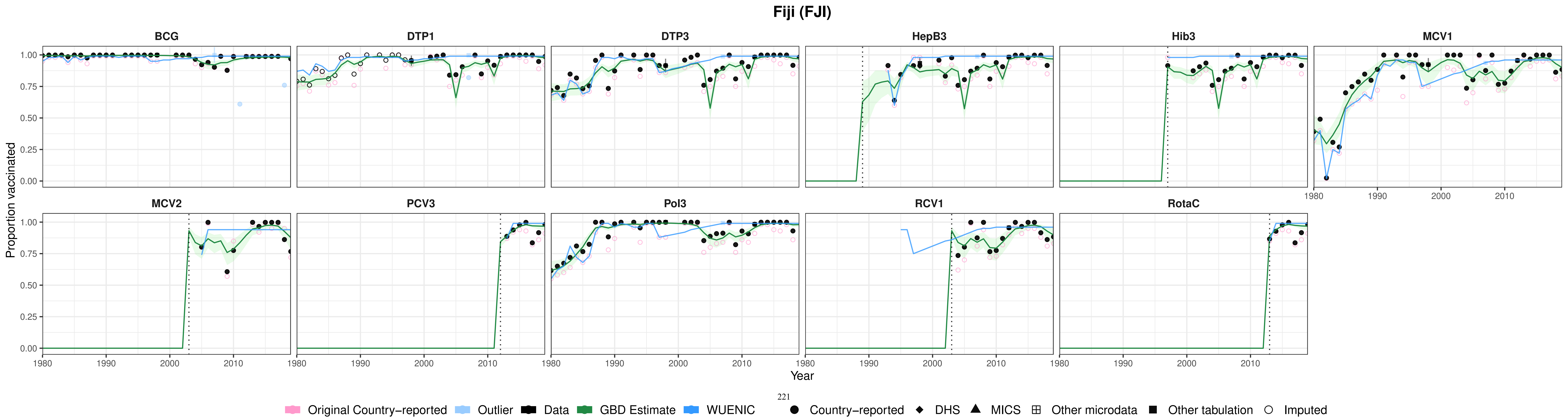

Fiji (FJI)
BCG
DTP1
DTP3
HepB3
Hib3
MCV1
MCV2
PCV3
Pol3
RCV1
RotaC
Proportion vaccinated
Year
1.00
0.75
0.50
0.25
0.00
1980
1990
2000
2010
Original Country−reported
Outlier
Data
GBD Estimate
WUENIC
Country−reported
DHS
MICS
Other microdata
Other tabulation
Imputed

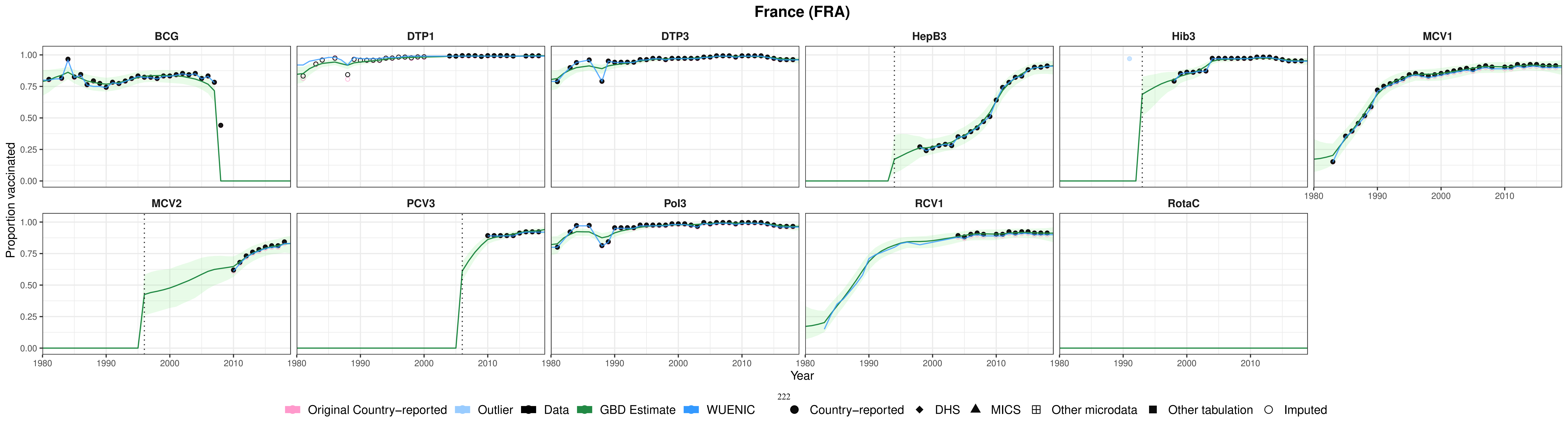
France (FRA)
BCG
DTP1
DTP3
HepB3
Hib3
MCV1
MCV2
PCV3
Pol3
RCV1
RotaC
Proportion vaccinated
Year
Original Country−reported
Outlier
Data
GBD Estimate
WUENIC
Country−reported
DHS
MICS
Other microdata
Other tabulation
Imputed

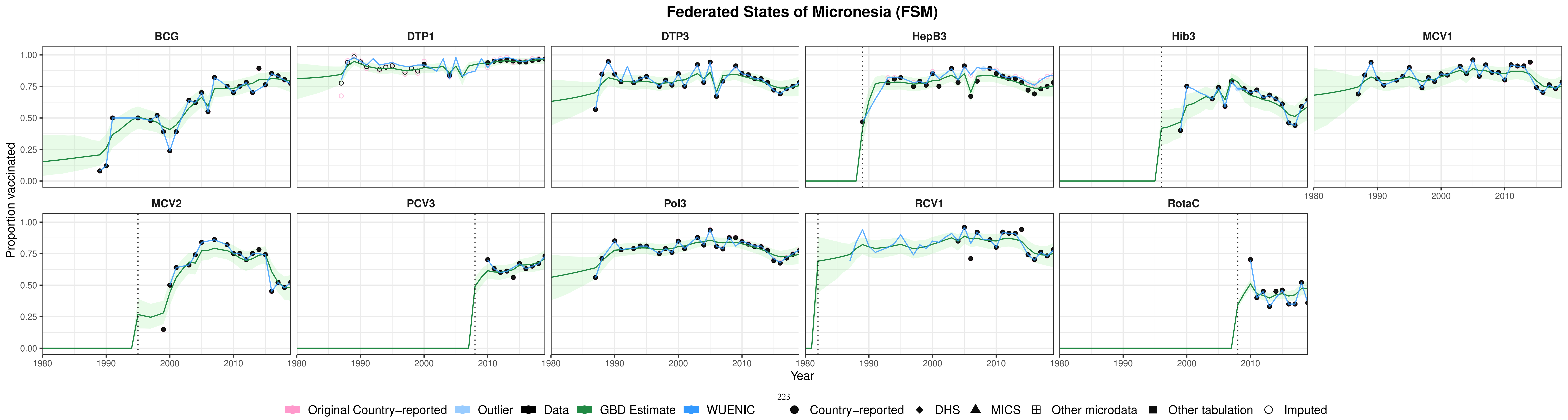

Federated States of Micronesia (FSM)
BCG
DTP1
DTP3
HepB3
Hib3
MCV1
MCV2
PCV3
Pol3
RCV1
RotaC
Proportion vaccinated
Year
Original Country−reported
Outlier
Data
GBD Estimate
WUENIC
Country−reported
DHS
MICS
Other microdata
Other tabulation
Imputed

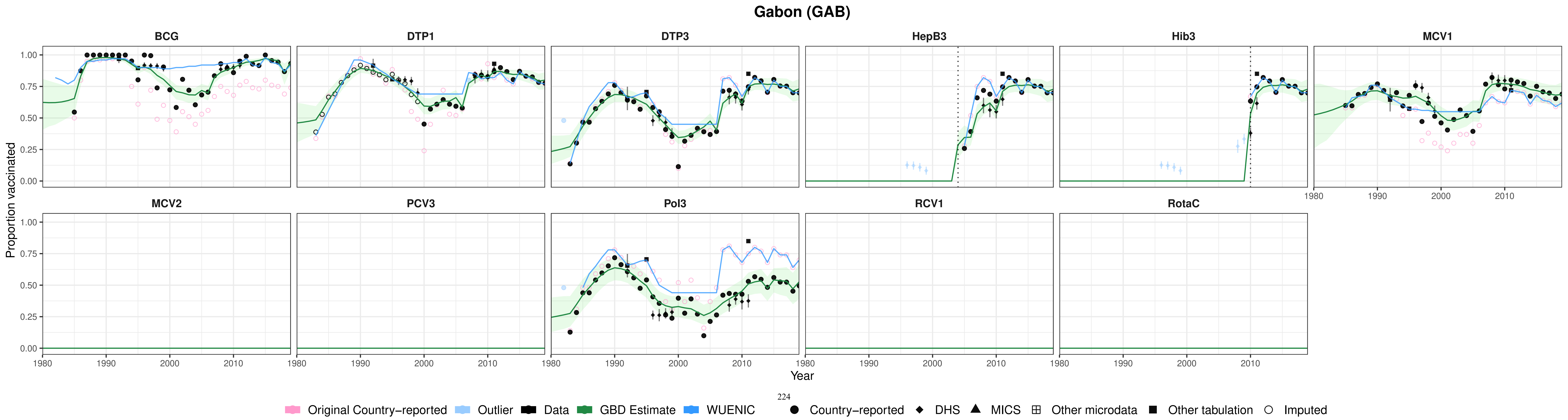

Gabon (GAB)
BCG
DTP1
DTP3
HepB3
Hib3
MCV1
MCV2
PCV3
Pol3
RCV1
RotaC
Proportion vaccinated
Year
Original Country−reported
Outlier
Data
GBD Estimate
WUENIC
Country−reported
DHS
MICS
Other microdata
Other tabulation
Imputed

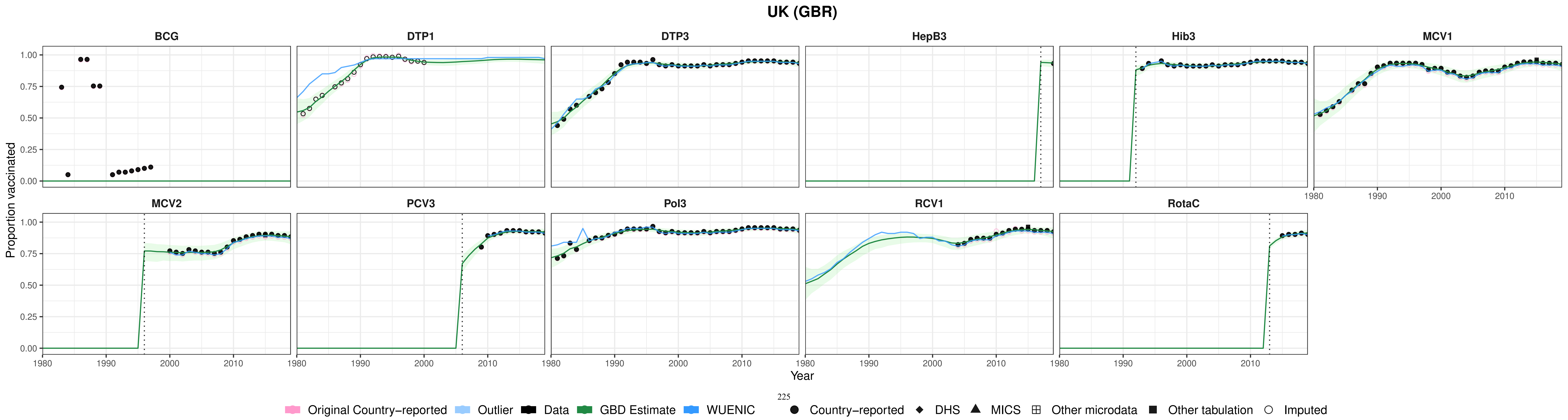

UK (GBR)
BCG
DTP1
DTP3
HepB3
Hib3
MCV1
MCV2
PCV3
Pol3
RCV1
RotaC
Proportion vaccinated
Year
Original Country−reported
Outlier
Data
GBD Estimate
WUENIC
Country−reported
DHS
MICS
Other microdata
Other tabulation
Imputed

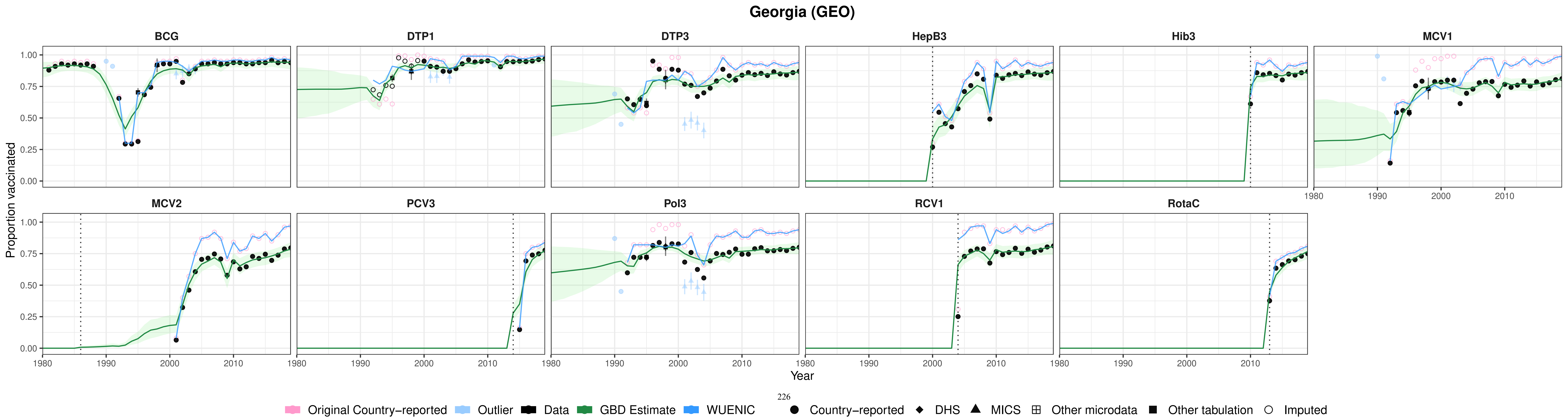

Georgia (GEO)
BCG
DTP1
DTP3
HepB3
Hib3
MCV1
MCV2
PCV3
Pol3
RCV1
RotaC
Proportion vaccinated
Year
Original Country−reported
Outlier
Data
GBD Estimate
WUENIC
Country−reported
DHS
MICS
Other microdata
Other tabulation
Imputed

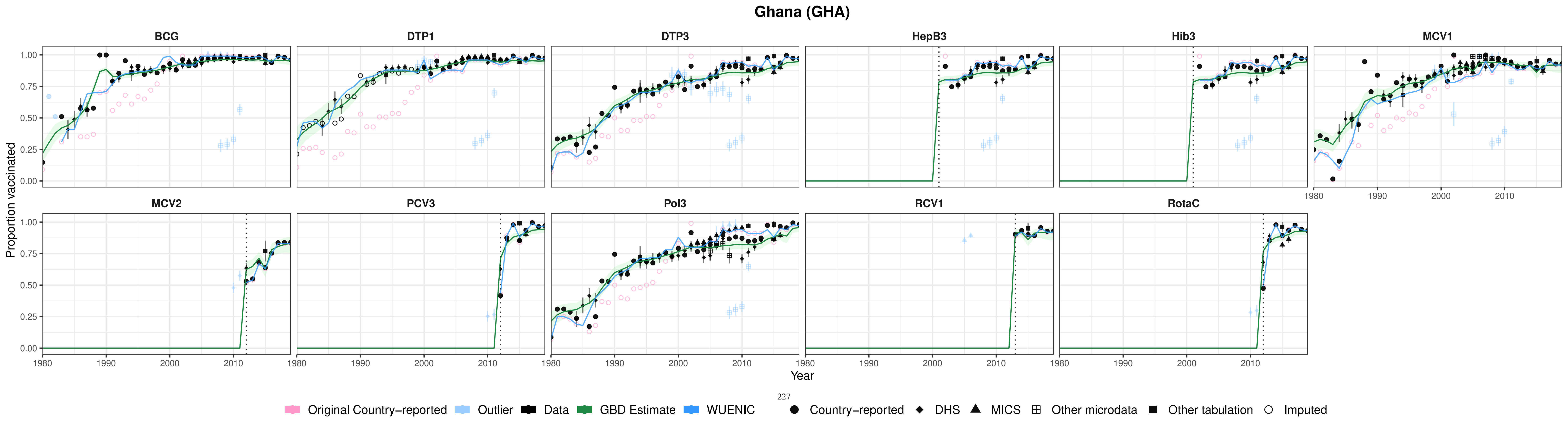
Ghana (GHA)
BCG
DTP1
DTP3
HepB3
Hib3
MCV1
MCV2
PCV3
Pol3
RCV1
RotaC
Proportion vaccinated
Year
1.00
0.75
0.50
0.25
0.00
1980
1990
2000
2010
Original Country−reported
Outlier
Data
GBD Estimate
WUENIC
Country−reported
DHS
MICS
Other microdata
Other tabulation
Imputed

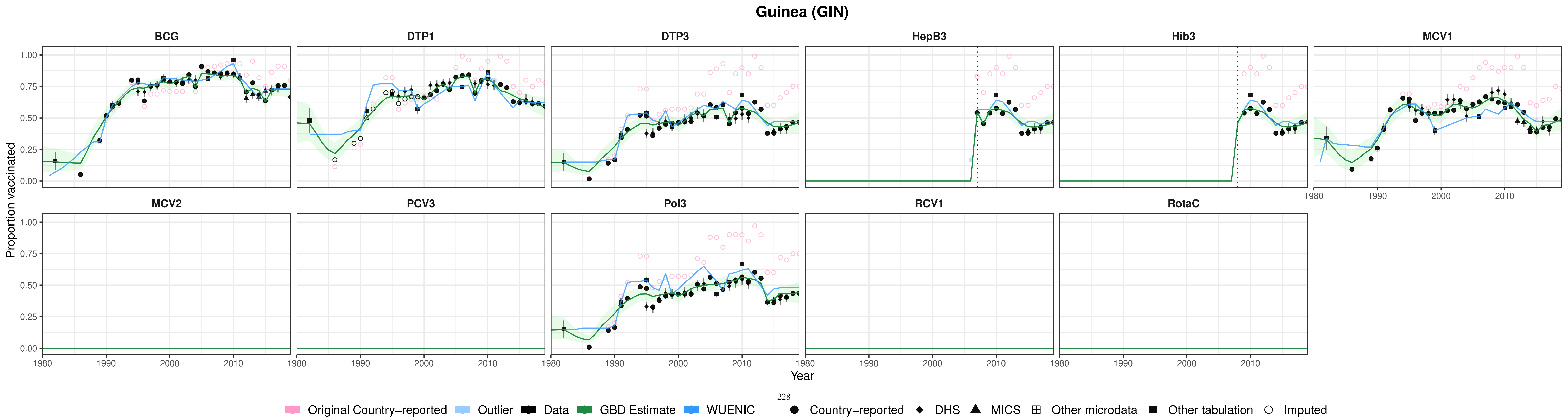

Guinea (GIN)
BCG
DTP1
DTP3
HepB3
Hib3
MCV1
MCV2
PCV3
Pol3
RCV1
RotaC
Proportion vaccinated
0.00
0.25
0.50
0.75
1.00
1980
1990
2000
2010
Year
Original Country−reported
Outlier
Data
GBD Estimate
WUENIC
Country−reported
DHS
MICS
Other microdata
Other tabulation
Imputed

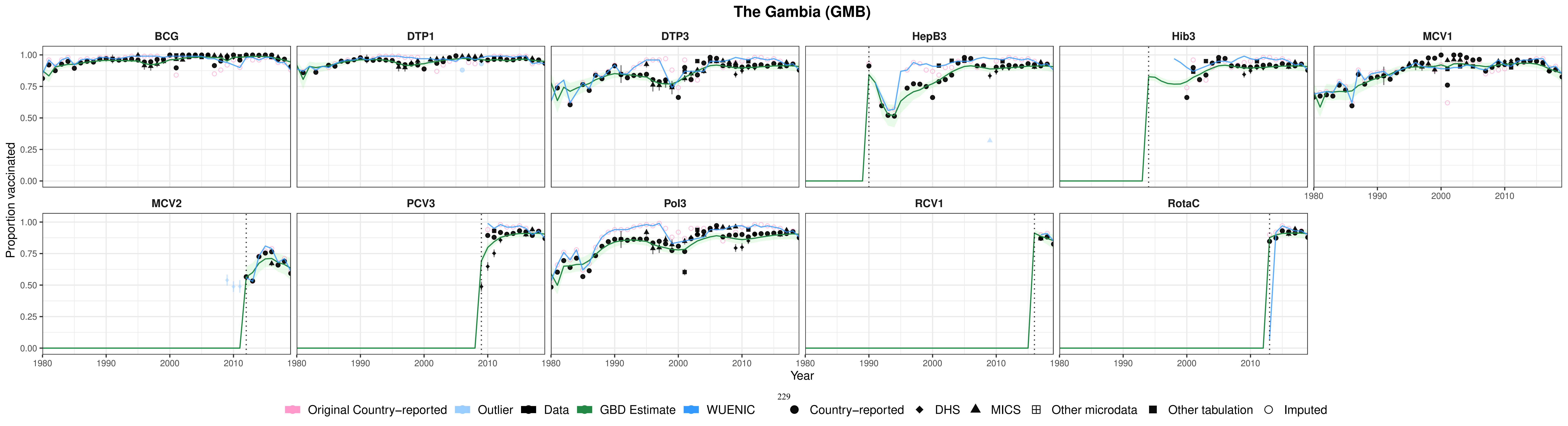
The Gambia (GMB)
BCG
DTP1
DTP3
HepB3
Hib3
MCV1
MCV2
PCV3
Pol3
RCV1
RotaC
Proportion vaccinated
Year
Original Country−reported
Outlier
Data
GBD Estimate
WUENIC
Country−reported
DHS
MICS
Other microdata
Other tabulation
Imputed

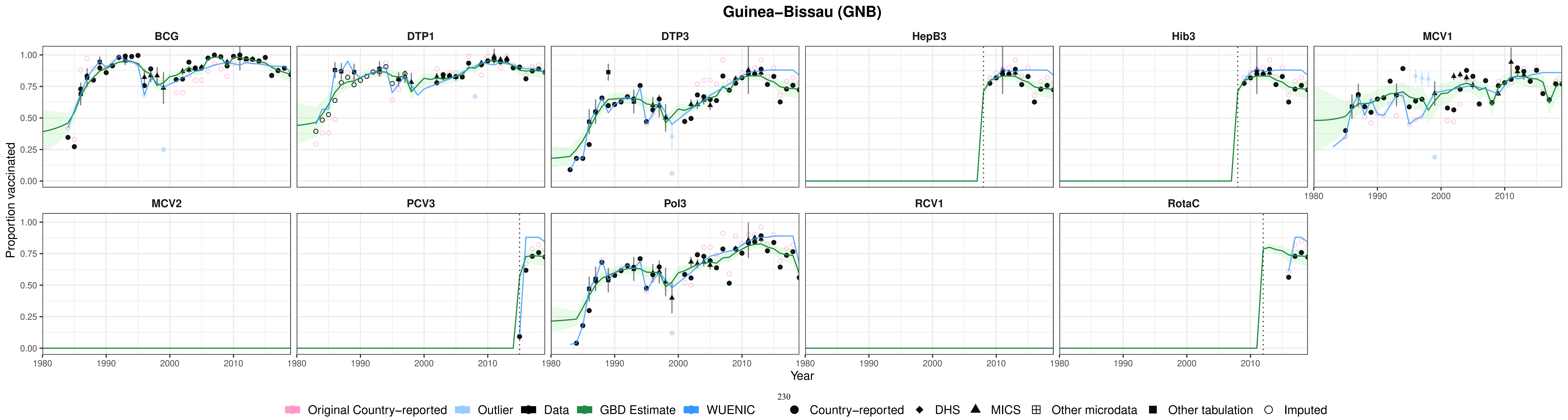

Guinea−Bissau (GNB)
BCG
DTP1
DTP3
HepB3
Hib3
MCV1
MCV2
PCV3
Pol3
RCV1
RotaC
Proportion vaccinated
Year
Original Country−reported
Outlier
Data
GBD Estimate
WUENIC
Country−reported
DHS
MICS
Other microdata
Other tabulation
Imputed

Equatorial Guinea (GNQ)

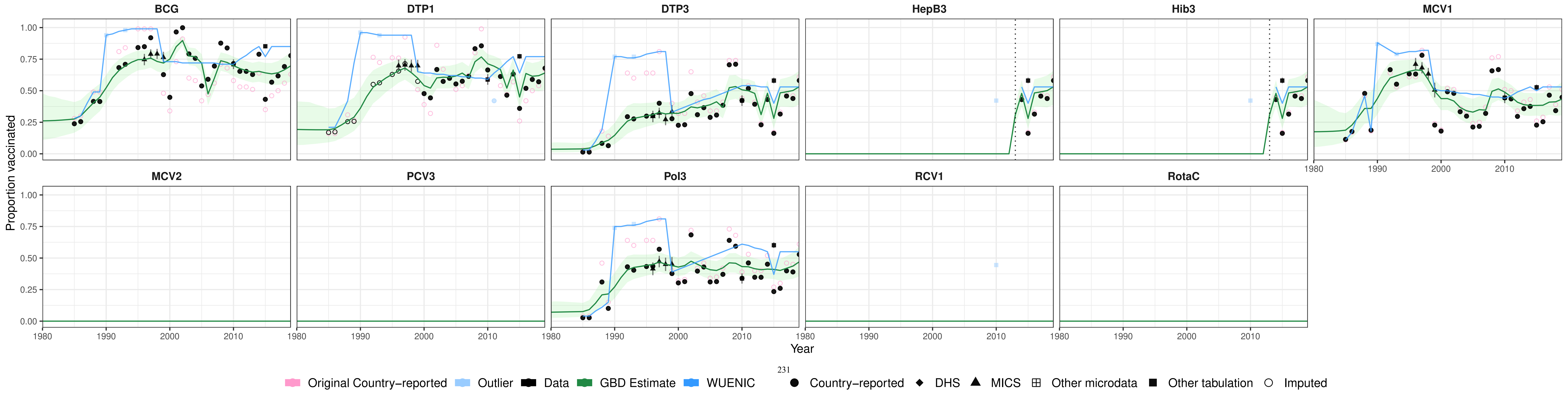

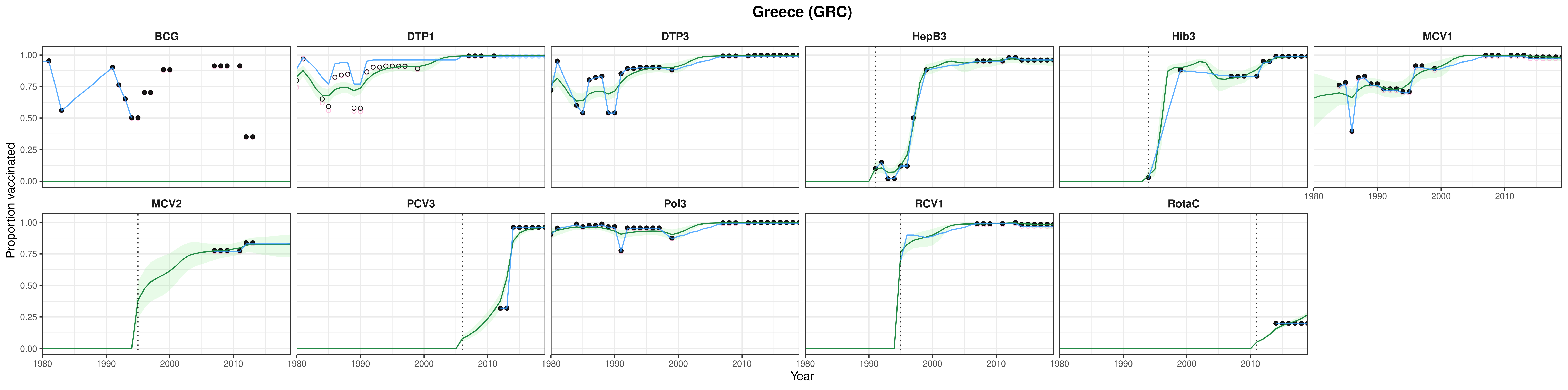

Greece (GRC)
BCG
DTP1
DTP3
HepB3
Hib3
MCV1
MCV2
PCV3
Pol3
RCV1
RotaC
Proportion vaccinated
Year
Original Country−reported
Outlier
Data
GBD Estimate
WUENIC
Country−reported
DHS
MICS
Other microdata
Other tabulation
Imputed

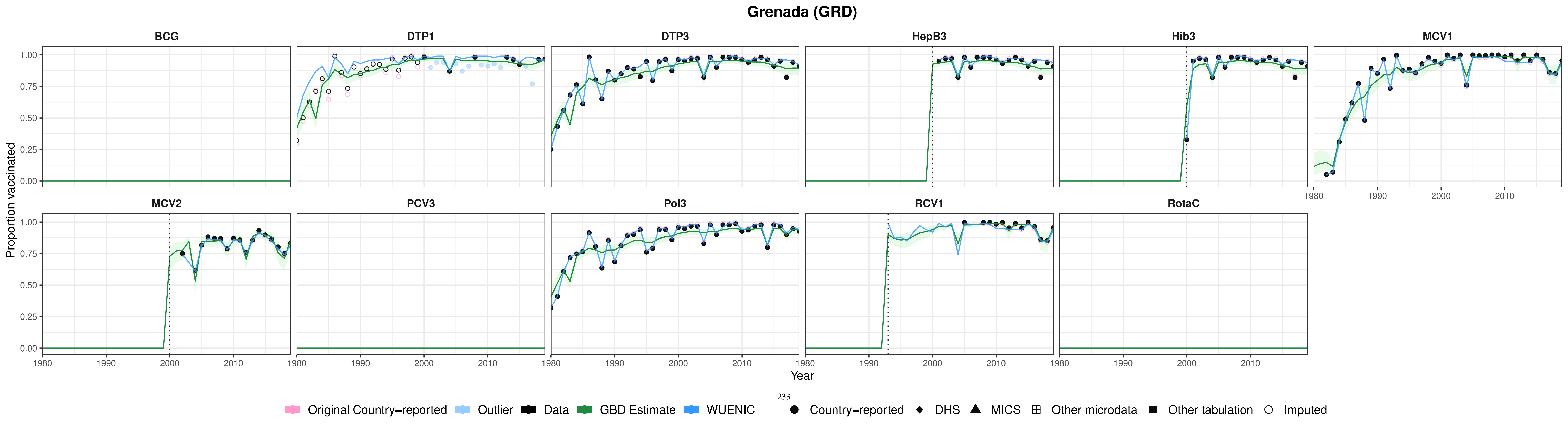
Grenada (GRD)
BCG
DTP1
DTP3
HepB3
Hib3
MCV1
MCV2
PCV3
Pol3
RCV1
RotaC
Proportion vaccinated
Year
Original Country−reported
Outlier
Data
GBD Estimate
WUENIC
Country−reported
DHS
MICS
Other microdata
Other tabulation
Imputed

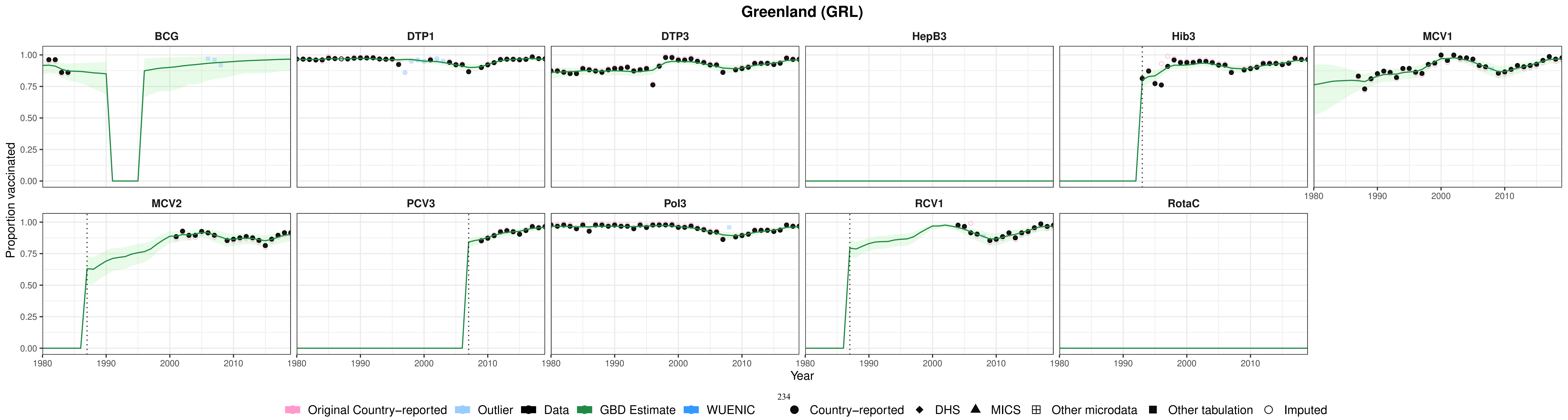

Greenland (GRL)
BCG
DTP1
DTP3
HepB3
Hib3
MCV1
MCV2
PCV3
Pol3
RCV1
RotaC
Proportion vaccinated
Year
Original Country−reported
Outlier
Data
GBD Estimate
WUENIC
Country−reported
DHS
MICS
Other microdata
Other tabulation
Imputed

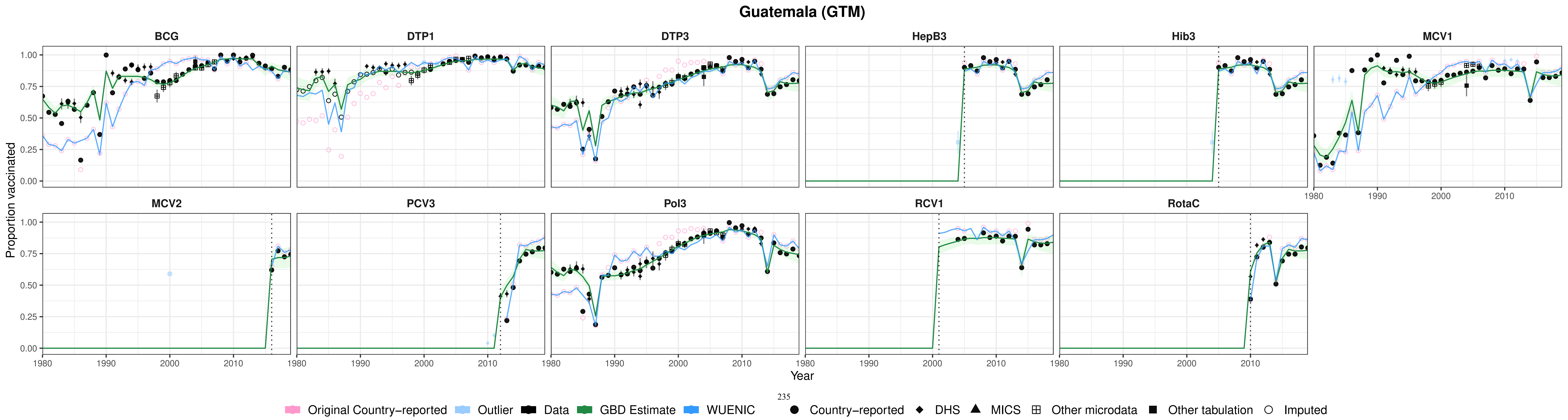
Guatemala (GTM)
BCG
DTP1
DTP3
HepB3
Hib3
MCV1
MCV2
PCV3
Pol3
RCV1
RotaC
Proportion vaccinated
0.00
0.25
0.50
0.75
1.00
1980
1990
2000
2010
Year
Original Country−reported
Outlier
Data
GBD Estimate
WUENIC
Country−reported
DHS
MICS
Other microdata
Other tabulation
Imputed

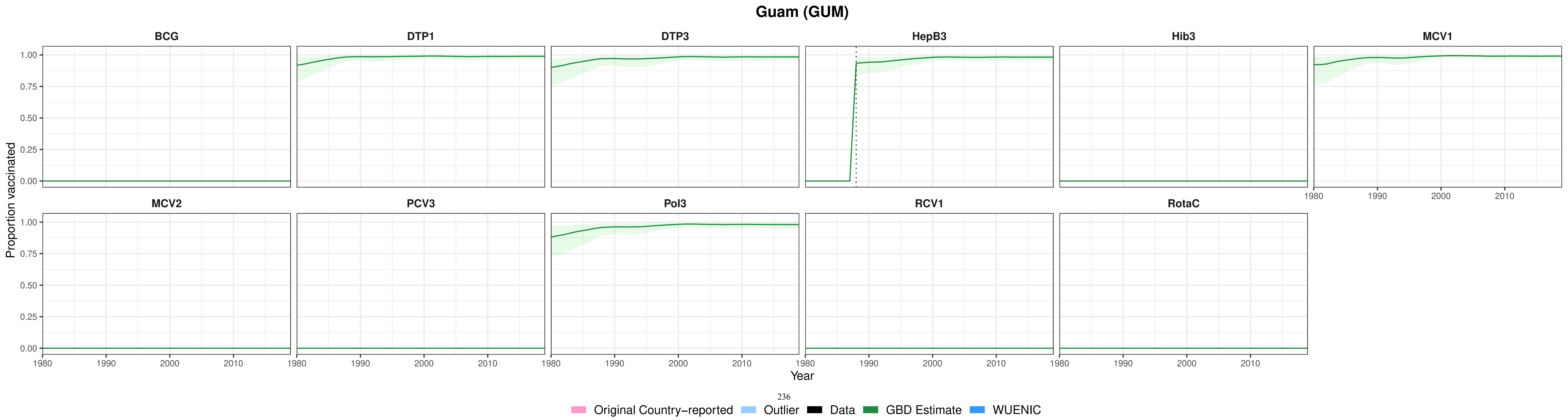

Guam (GUM)
BCG
DTP1
DTP3
HepB3
Hib3
MCV1
MCV2
PCV3
Pol3
RCV1
RotaC
Proportion vaccinated
1.00
0.75
0.50
0.25
0.00
1980
1990
2000
2010
Year
Original Country−reported
Outlier
Data
GBD Estimate
WUENIC

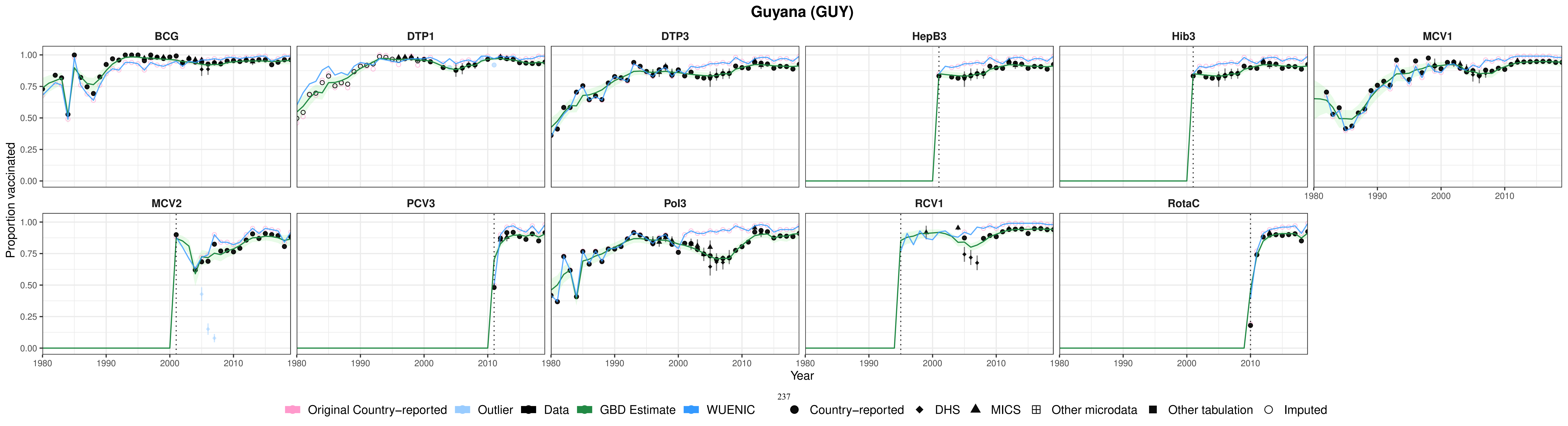

Guyana (GUY)
BCG
DTP1
DTP3
HepB3
Hib3
MCV1
MCV2
PCV3
Pol3
RCV1
RotaC
Proportion vaccinated
0.00
0.25
0.50
0.75
1.00
1980
1990
2000
2010
Year
Original Country−reported
Outlier
Data
GBD Estimate
WUENIC
Country−reported
DHS
MICS
Other microdata
Other tabulation
Imputed

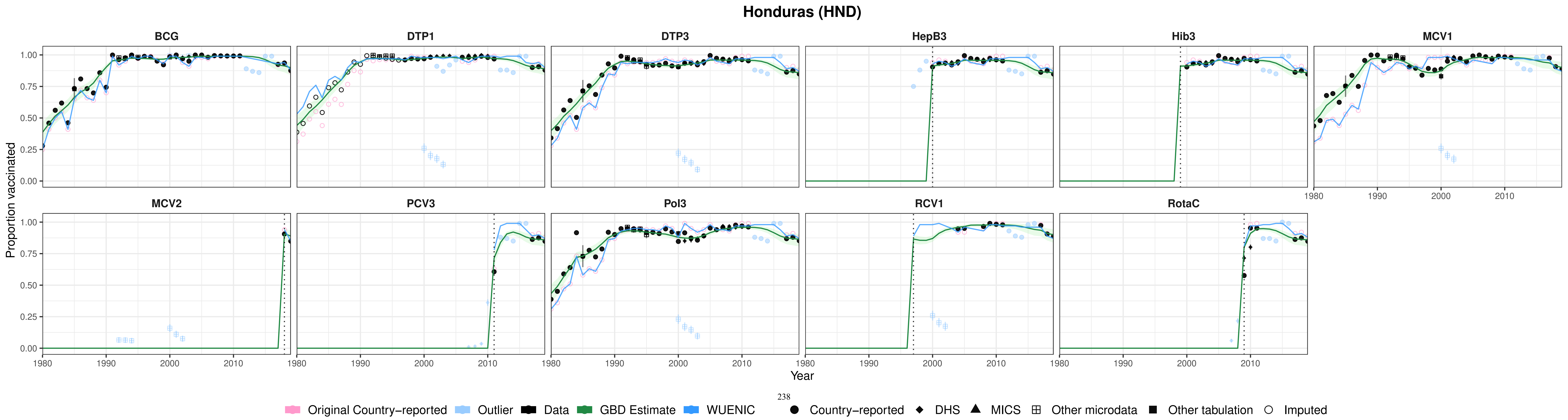
Honduras (HND)
BCG
DTP1
DTP3
HepB3
Hib3
MCV1
MCV2
PCV3
Pol3
RCV1
RotaC
Proportion vaccinated
1.00
0.75
0.50
0.25
0.00
1980
1990
2000
2010
Year
Original Country−reported
Outlier
Data
GBD Estimate
WUENIC
Country−reported
DHS
MICS
Other microdata
Other tabulation
Imputed

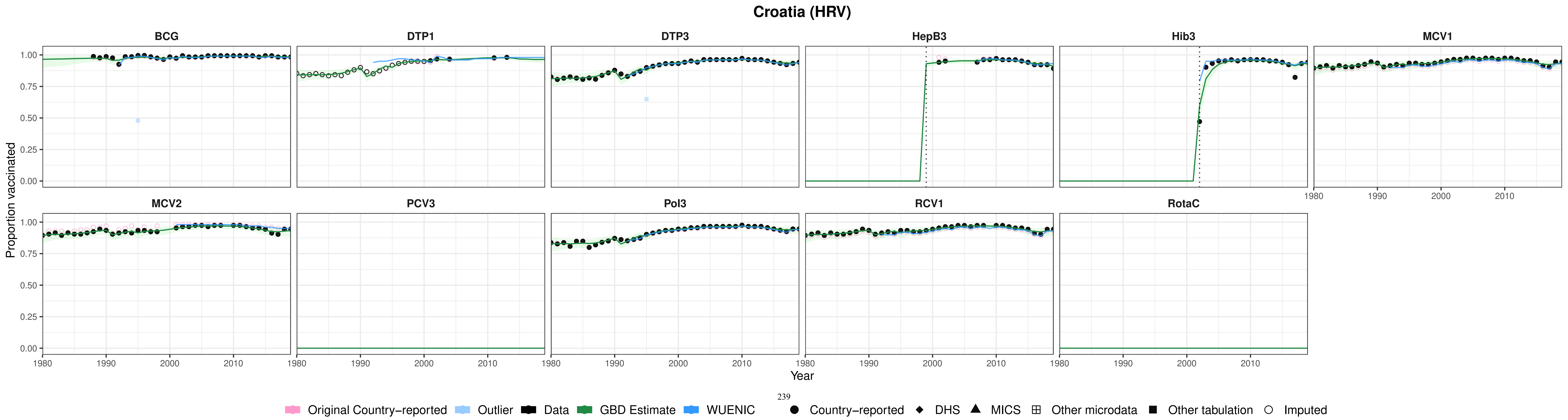
Croatia (HRV)
BCG
DTP1
DTP3
HepB3
Hib3
MCV1
MCV2
PCV3
Pol3
RCV1
RotaC
Proportion vaccinated
Year
1.00
0.75
0.50
0.25
0.00
1980
1990
2000
2010
Original Country−reported
Outlier
Data
GBD Estimate
WUENIC
Country−reported
DHS
MICS
Other microdata
Other tabulation
Imputed

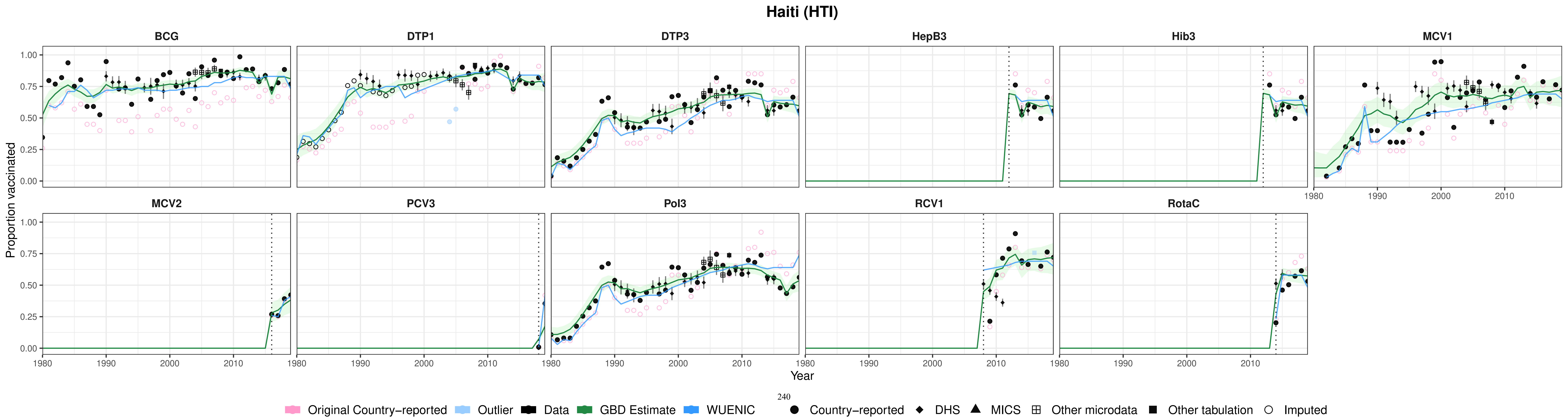

Haiti (HTI)
BCG
DTP1
DTP3
HepB3
Hib3
MCV1
MCV2
PCV3
Pol3
RCV1
RotaC
Proportion vaccinated
0.00
0.25
0.50
0.75
1.00
1980
1990
2000
2010
Year
Original Country−reported
Outlier
Data
GBD Estimate
WUENIC
Country−reported
DHS
MICS
Other microdata
Other tabulation
Imputed

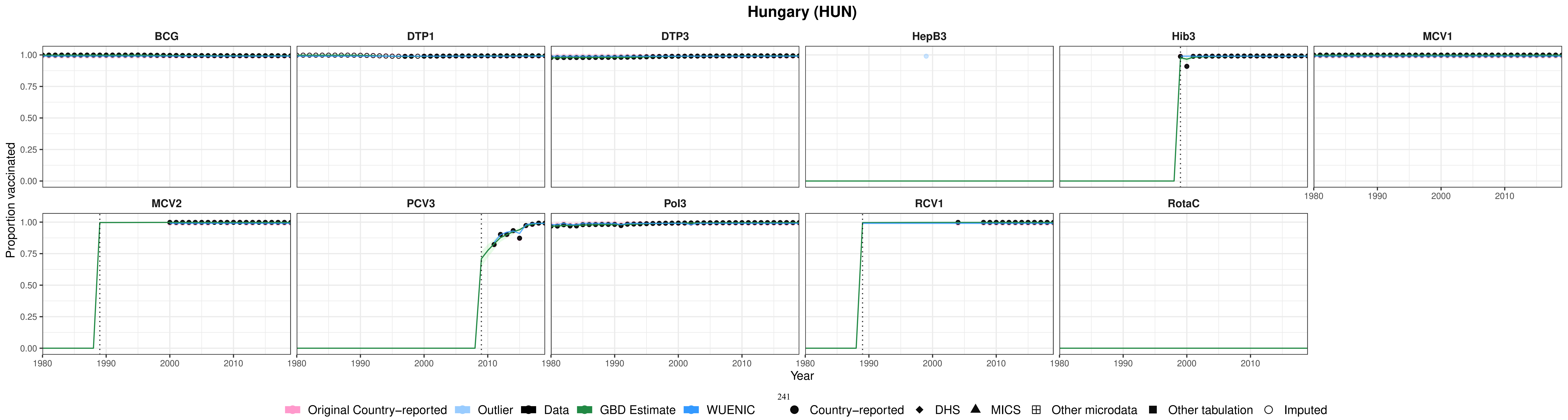
Hungary (HUN)
BCG
DTP1
DTP3
HepB3
Hib3
MCV1
MCV2
PCV3
Pol3
RCV1
RotaC
Proportion vaccinated
1.00
0.75
0.50
0.25
0.00
1980
1990
2000
2010
Year
Original Country−reported
Outlier
Data
GBD Estimate
WUENIC
Country−reported
DHS
MICS
Other microdata
Other tabulation
Imputed

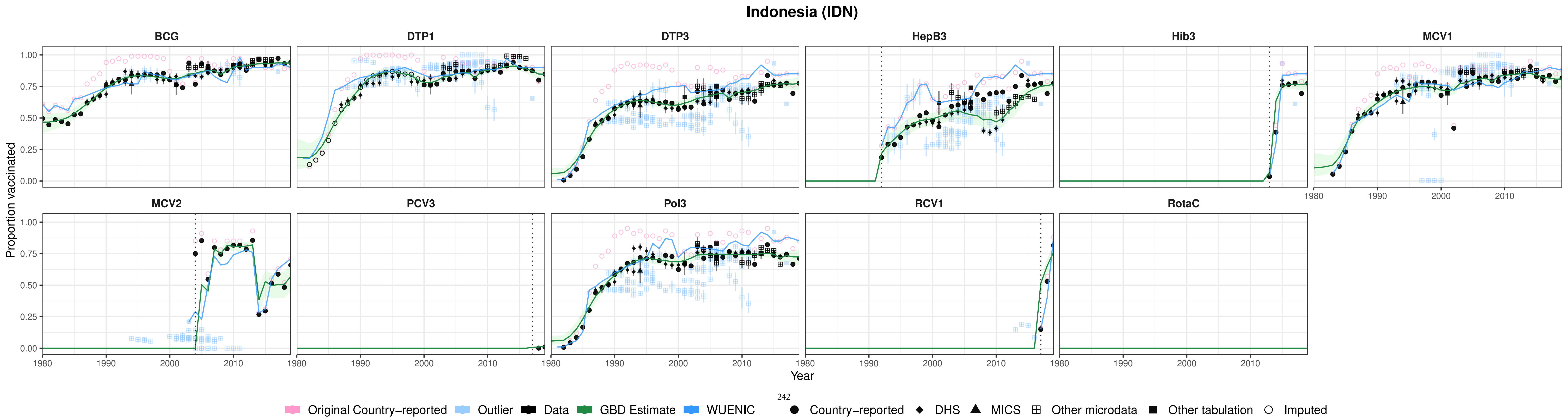

Indonesia (IDN)
BCG
DTP1
DTP3
HepB3
Hib3
MCV1
MCV2
PCV3
Pol3
RCV1
RotaC
Proportion vaccinated
1.00
0.75
0.50
0.25
0.00
1980
1990
2000
2010
Year
Original Country−reported
Outlier
Data
GBD Estimate
WUENIC
Country−reported
DHS
MICS
Other microdata
Other tabulation
Imputed

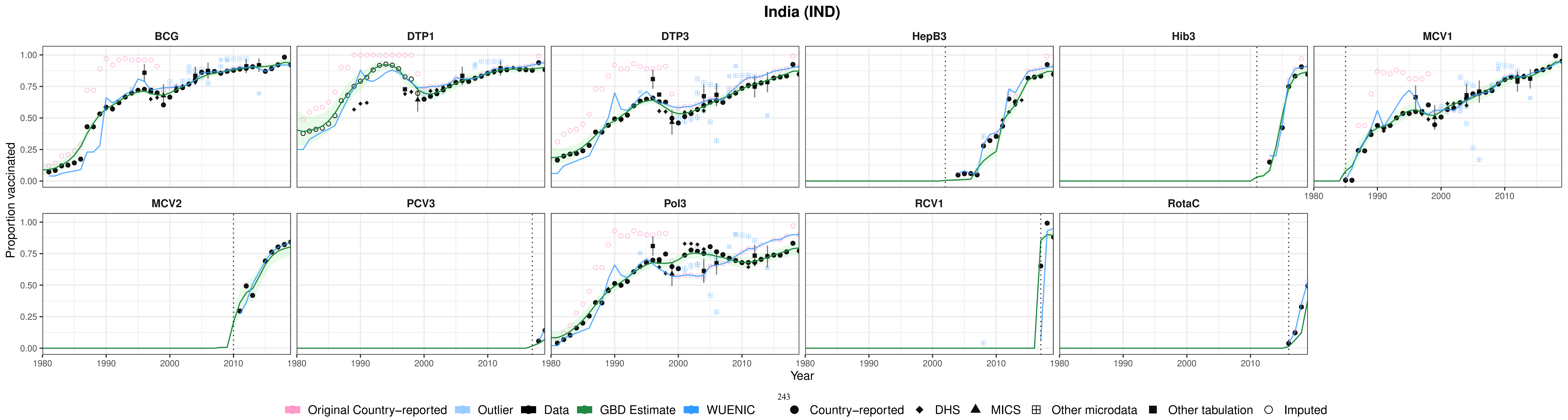

India (IND)
BCG
DTP1
DTP3
HepB3
Hib3
MCV1
MCV2
PCV3
Pol3
RCV1
RotaC
Proportion vaccinated
0.00
0.25
0.50
0.75
1.00
1980
1990
2000
2010
Year
Original Country−reported
Outlier
Data
GBD Estimate
WUENIC
Country−reported
DHS
MICS
Other microdata
Other tabulation
Imputed

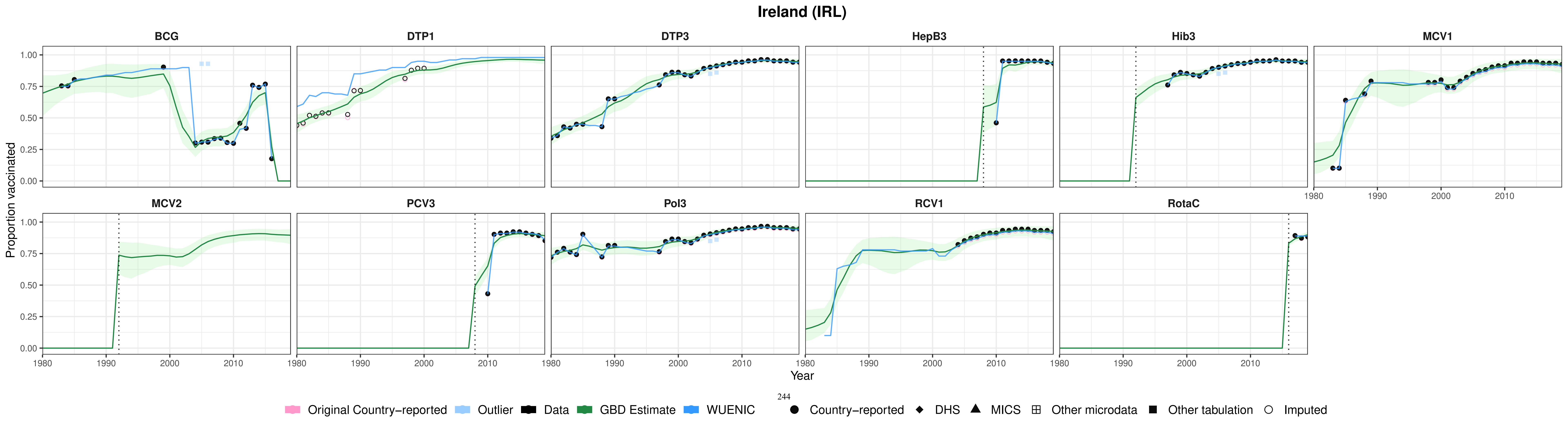

Ireland (IRL)
BCG
DTP1
DTP3
HepB3
Hib3
MCV1
MCV2
PCV3
Pol3
RCV1
RotaC
Proportion vaccinated
Year
Original Country−reported
Outlier
Data
GBD Estimate
WUENIC
Country−reported
DHS
MICS
Other microdata
Other tabulation
Imputed

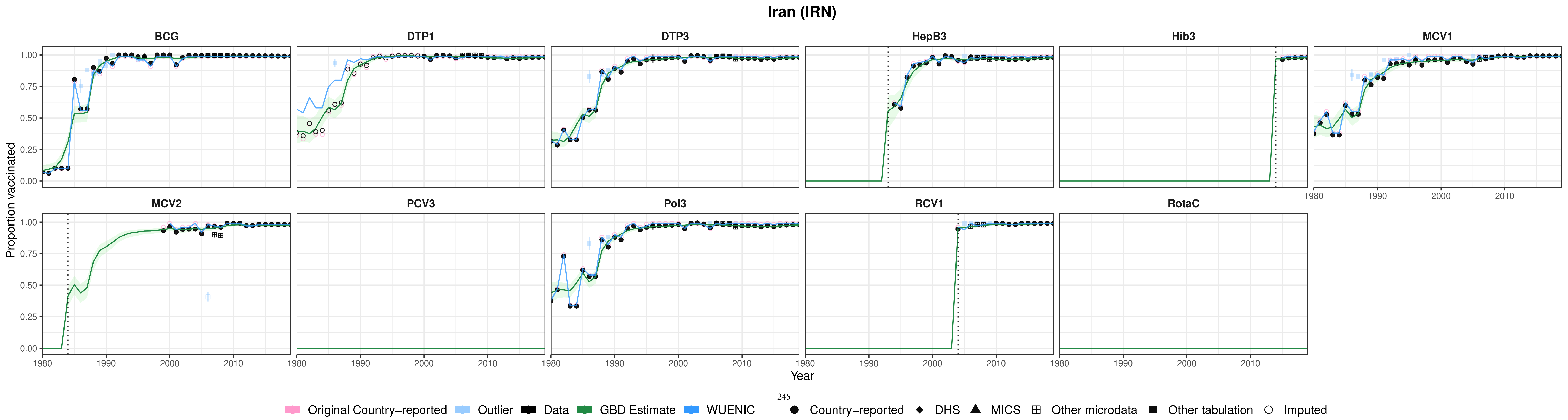

Iran (IRN)
BCG
DTP1
DTP3
HepB3
Hib3
MCV1
MCV2
PCV3
Pol3
RCV1
RotaC
Proportion vaccinated
1.00
0.75
0.50
0.25
0.00
1980
1990
2000
2010
Year
Original Country−reported
Outlier
Data
GBD Estimate
WUENIC
Country−reported
DHS
MICS
Other microdata
Other tabulation
Imputed

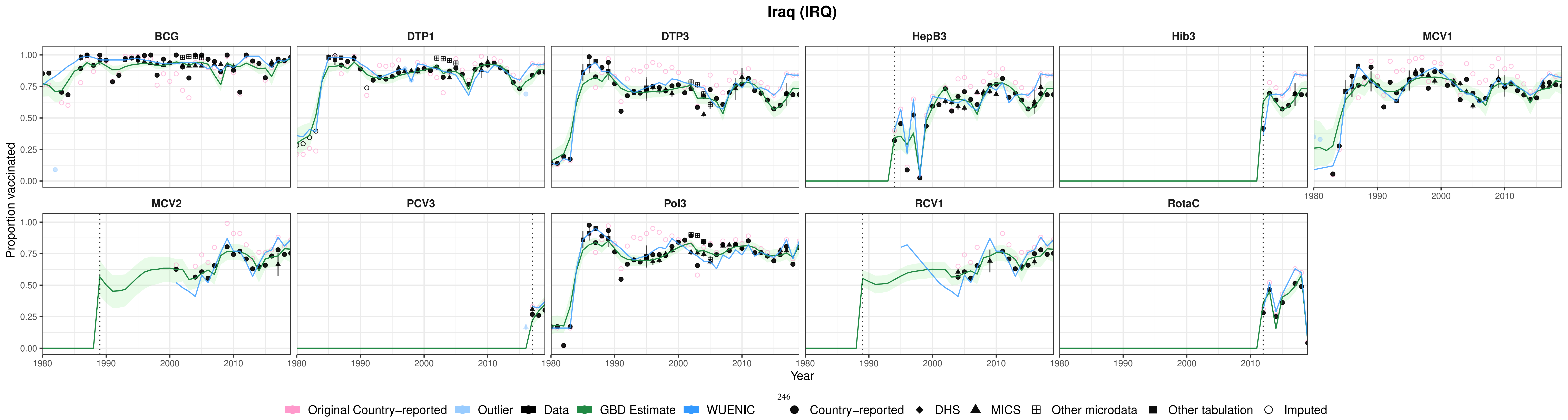
Iraq (IRQ)
BCG
DTP1
DTP3
HepB3
Hib3
MCV1
MCV2
PCV3
Pol3
RCV1
RotaC
Proportion vaccinated
Year
Original Country−reported
Outlier
Data
GBD Estimate
WUENIC
Country−reported
DHS
MICS
Other microdata
Other tabulation
Imputed

Iceland (ISL)

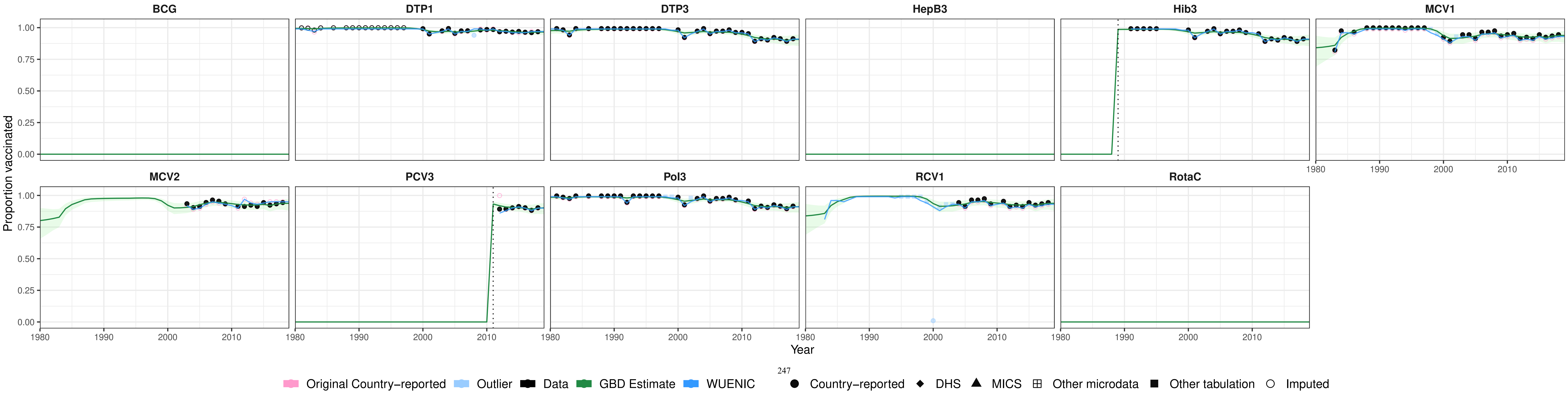

BCG
DTP1
DTP3
HepB3
Hib3
MCV1
MCV2
PCV3
Pol3
RCV1
RotaC
Proportion vaccinated
1.00
0.75
0.50
0.25
0.00
1980
1990
2000
2010
Year
Original Country−reported
Outlier
Data
GBD Estimate
WUENIC
Country−reported
DHS
MICS
Other microdata
Other tabulation
Imputed

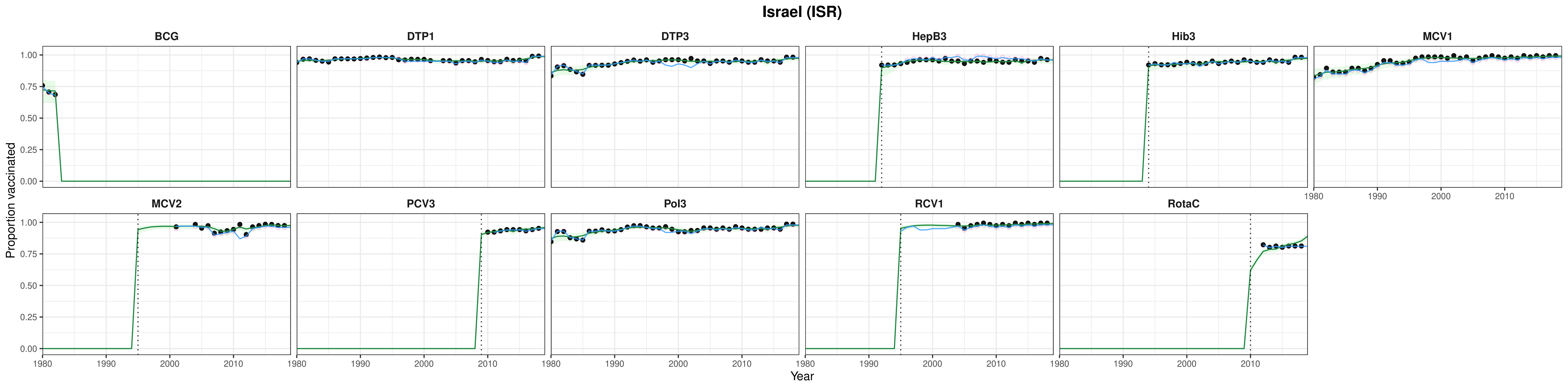
Israel (ISR)
BCG
DTP1
DTP3
HepB3
Hib3
MCV1
MCV2
PCV3
Pol3
RCV1
RotaC
Proportion vaccinated
Year
Original Country−reported
Outlier
Data
GBD Estimate
WUENIC
Country−reported
DHS
MICS
Other microdata
Other tabulation
Imputed

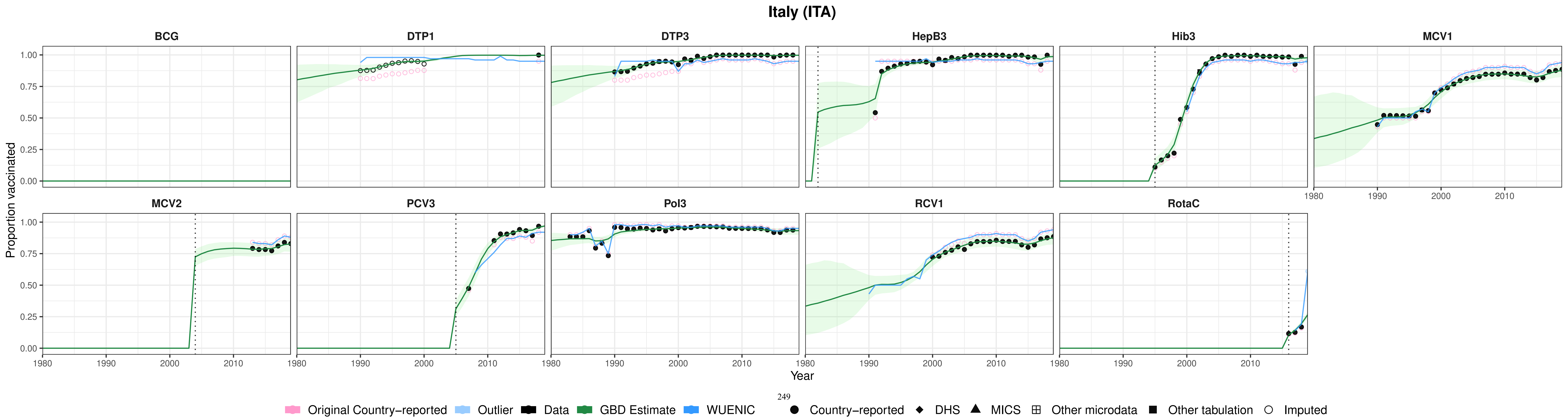

Italy (ITA)
BCG
DTP1
DTP3
HepB3
Hib3
MCV1
MCV2
PCV3
Pol3
RCV1
RotaC
Proportion vaccinated
Year
Original Country−reported
Outlier
Data
GBD Estimate
WUENIC
Country−reported
DHS
MICS
Other microdata
Other tabulation
Imputed

Jamaica (JAM)

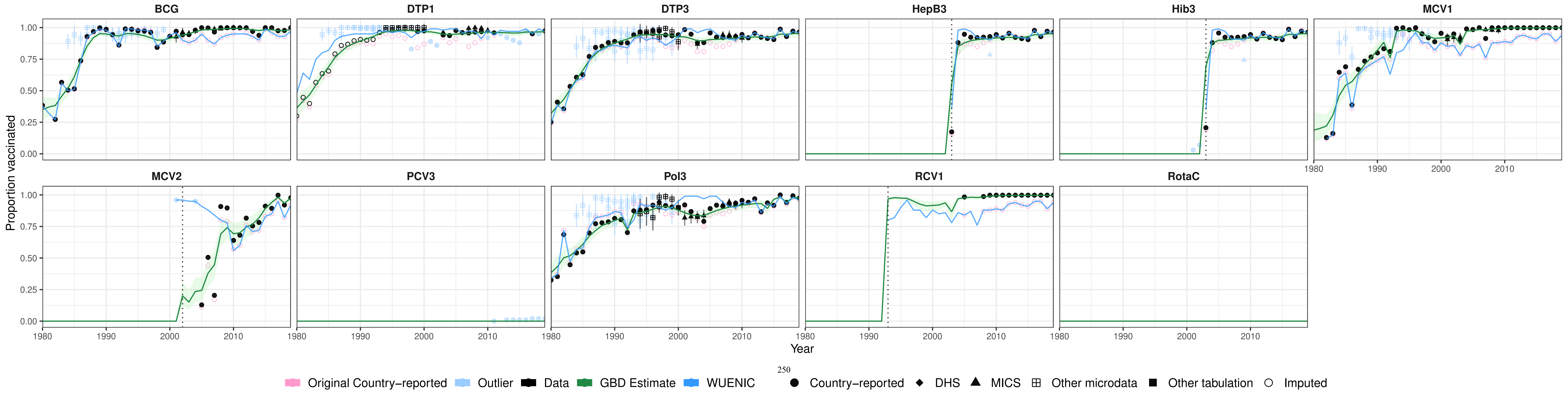

BCG
DTP1
DTP3
HepB3
Hib3
MCV1
MCV2
PCV3
Pol3
RCV1
RotaC
Proportion vaccinated
Year
1.00
0.75
0.50
0.25
0.00
1980
1990
2000
2010
Original Country−reported
Outlier
Data
GBD Estimate
WUENIC
Country−reported
DHS
MICS
Other microdata
Other tabulation
Imputed

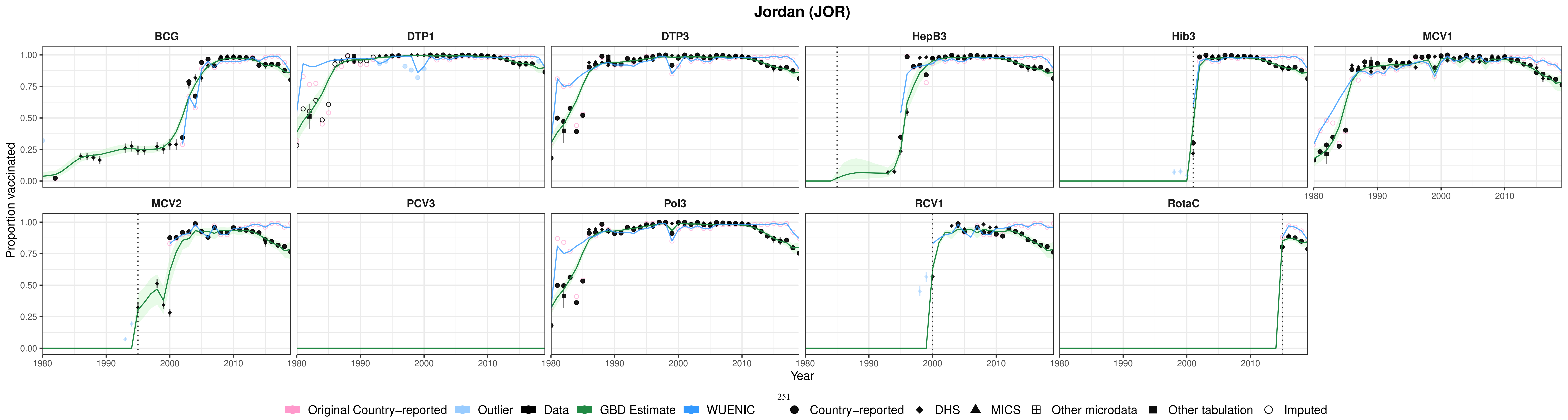
Jordan (JOR)
BCG
DTP1
DTP3
HepB3
Hib3
MCV1
MCV2
PCV3
Pol3
RCV1
RotaC
Proportion vaccinated
Year
Original Country−reported
Outlier
Data
GBD Estimate
WUENIC
Country−reported
DHS
MICS
Other microdata
Other tabulation
Imputed

Japan (JPN)

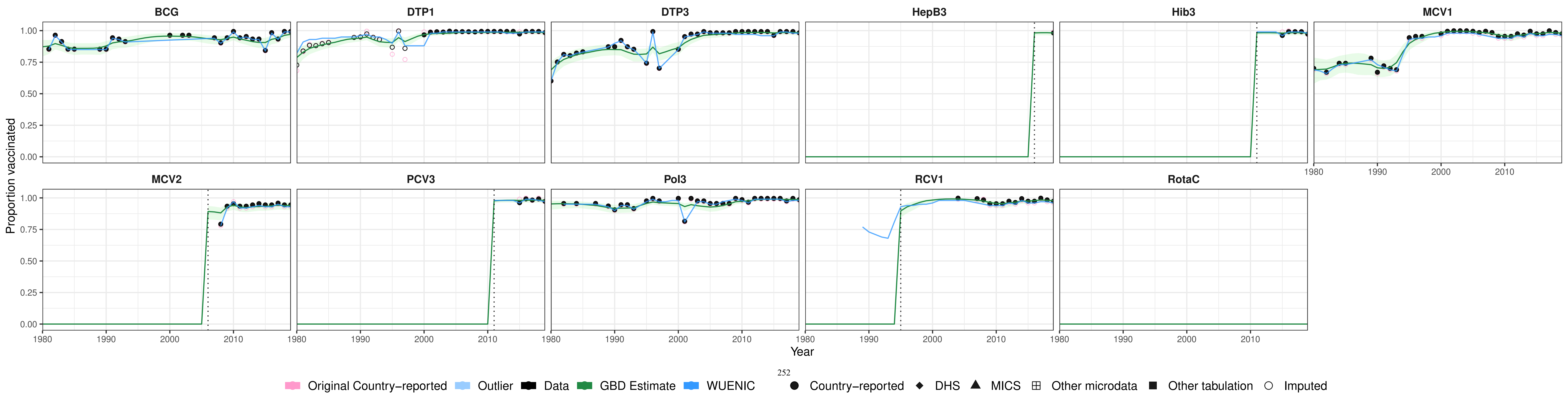

BCG
DTP1
DTP3
HepB3
Hib3
MCV1
MCV2
PCV3
Pol3
RCV1
RotaC
Proportion vaccinated
0.00
0.25
0.50
0.75
1.00
1980
1990
2000
2010
Year
Original Country−reported
Outlier
Data
GBD Estimate
WUENIC
Country−reported
DHS
MICS
Other microdata
Other tabulation
Imputed

Kazakhstan (KAZ)

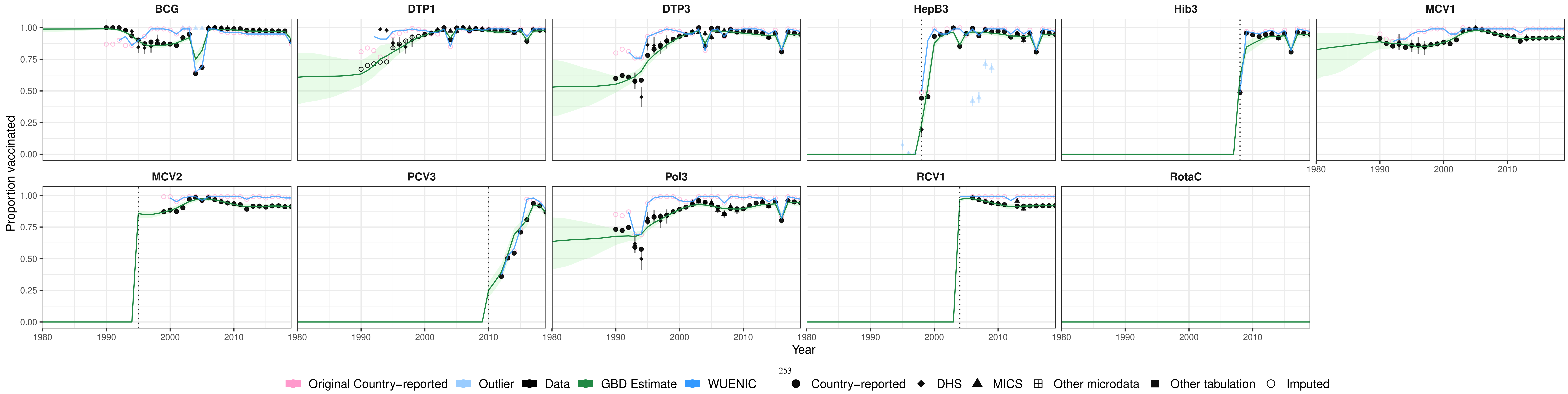

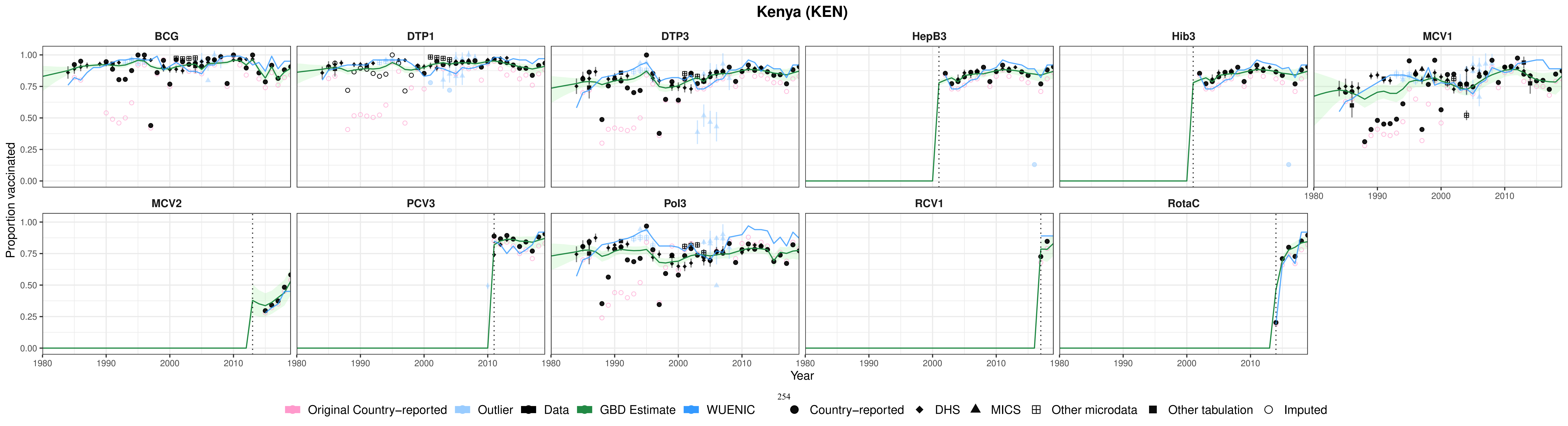
Kenya (KEN)
BCG
DTP1
DTP3
HepB3
Hib3
MCV1
MCV2
PCV3
Pol3
RCV1
RotaC
Proportion vaccinated
0.00
0.25
0.50
0.75
1.00
1980
1990
2000
2010
Year
Original Country−reported
Outlier
Data
GBD Estimate
WUENIC
Country−reported
DHS
MICS
Other microdata
Other tabulation
Imputed

Kyrgyzstan (KGZ)

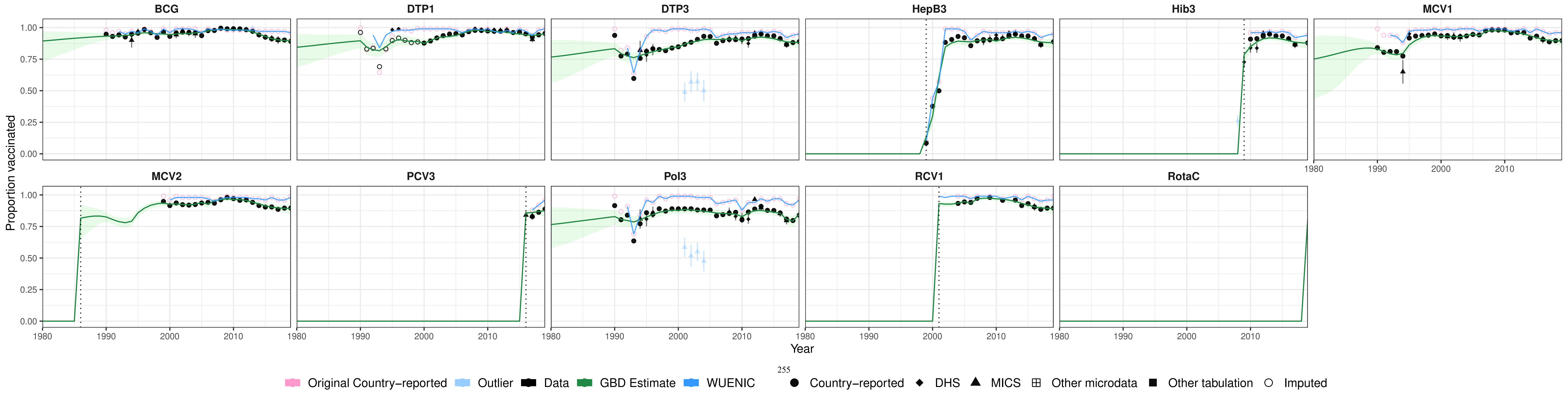

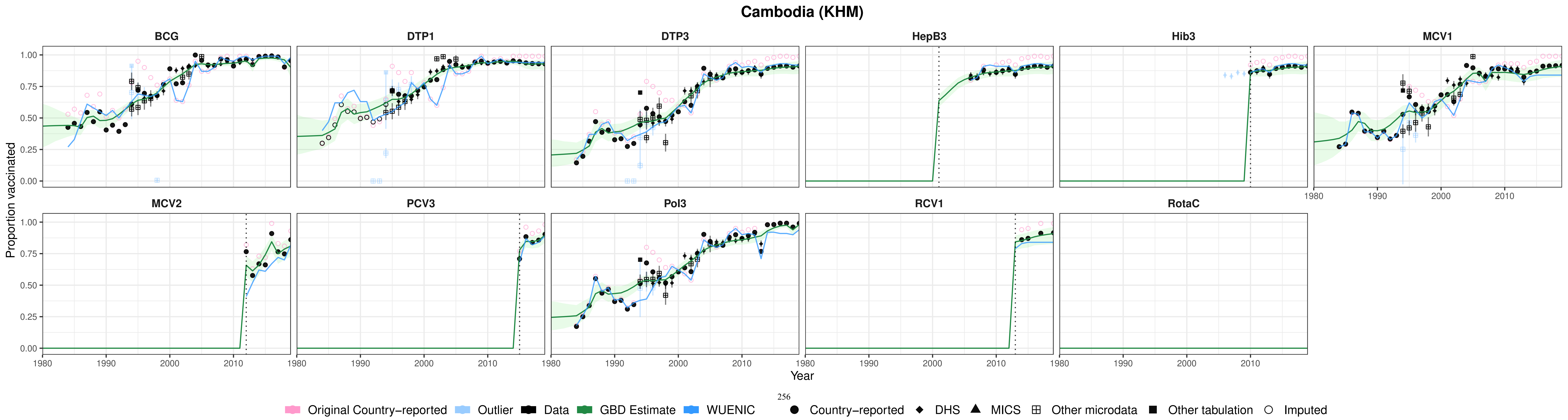

Cambodia (KHM)
BCG
DTP1
DTP3
HepB3
Hib3
MCV1
MCV2
PCV3
Pol3
RCV1
RotaC
Proportion vaccinated
Year
Original Country−reported
Outlier
Data
GBD Estimate
WUENIC
Country−reported
DHS
MICS
Other microdata
Other tabulation
Imputed

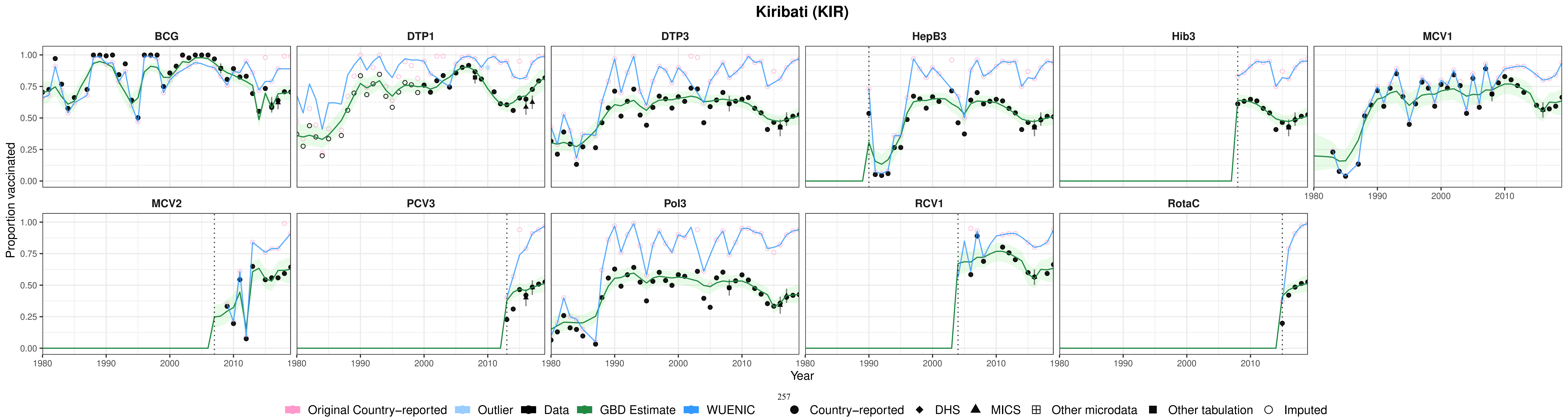
Kiribati (KIR)
BCG
DTP1
DTP3
HepB3
Hib3
MCV1
MCV2
PCV3
Pol3
RCV1
RotaC
Proportion vaccinated
Year
Original Country−reported
Outlier
Data
GBD Estimate
WUENIC
Country−reported
DHS
MICS
Other microdata
Other tabulation
Imputed

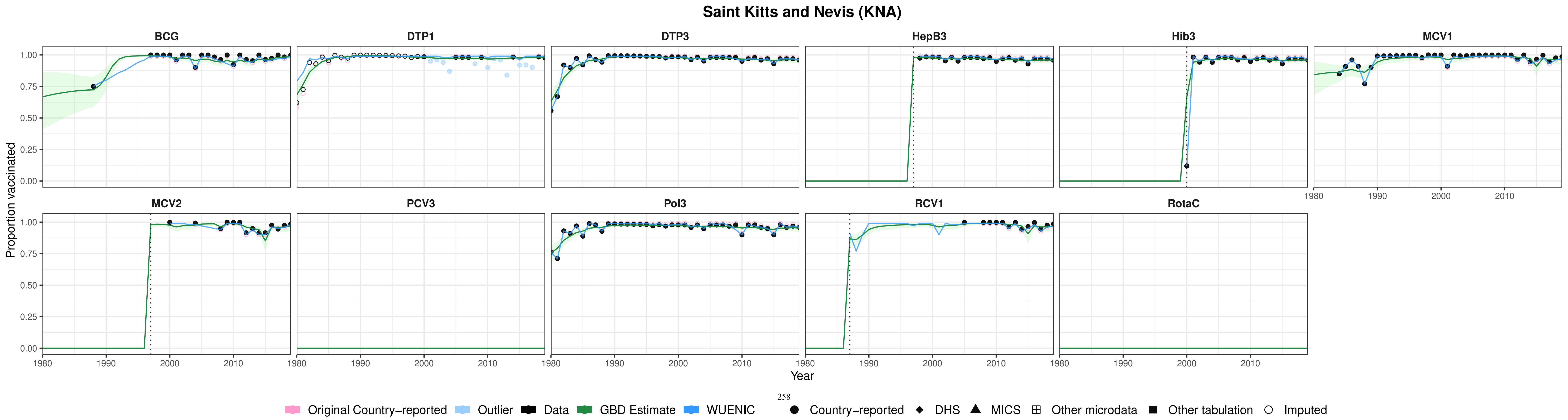
Saint Kitts and Nevis (KNA)
BCG
DTP1
DTP3
HepB3
Hib3
MCV1
MCV2
PCV3
Pol3
RCV1
RotaC
Proportion vaccinated
Year
Original Country−reported
Outlier
Data
GBD Estimate
WUENIC
Country−reported
DHS
MICS
Other microdata
Other tabulation
Imputed

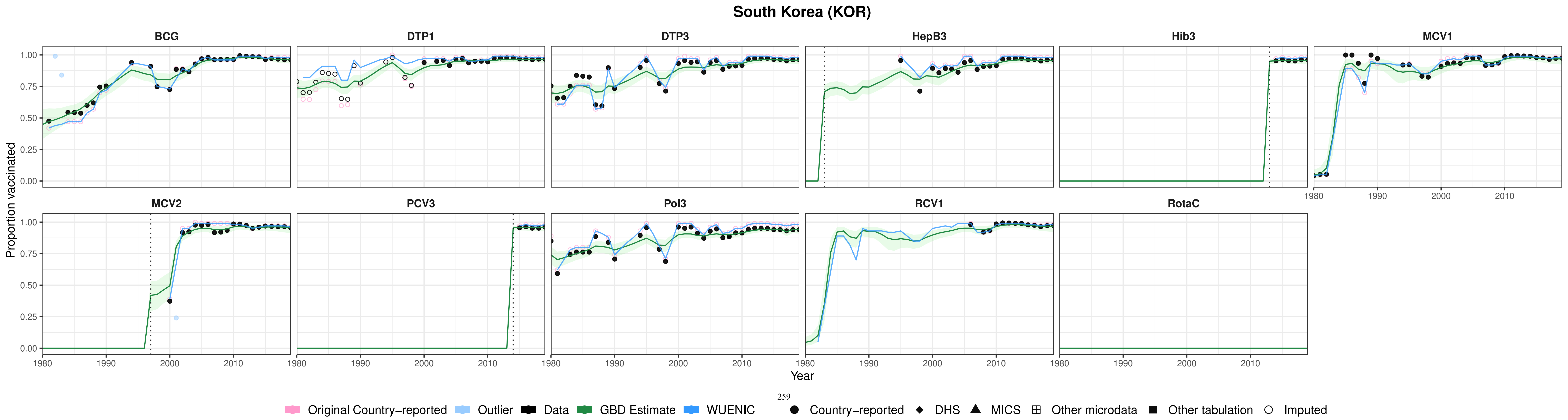
South Korea (KOR)
BCG
DTP1
DTP3
HepB3
Hib3
MCV1
MCV2
PCV3
Pol3
RCV1
RotaC
Proportion vaccinated
Year
Original Country−reported
Outlier
Data
GBD Estimate
WUENIC
Country−reported
DHS
MICS
Other microdata
Other tabulation
Imputed

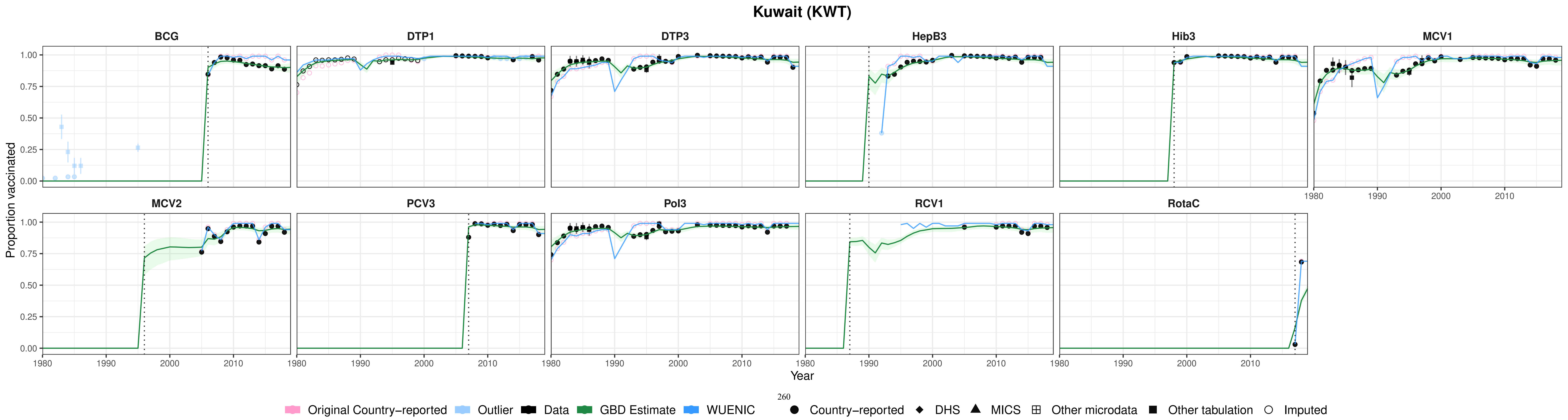

Kuwait (KWT)
BCG
DTP1
DTP3
HepB3
Hib3
MCV1
MCV2
PCV3
Pol3
RCV1
RotaC
Proportion vaccinated
Year
Original Country−reported
Outlier
Data
GBD Estimate
WUENIC
Country−reported
DHS
MICS
Other microdata
Other tabulation
Imputed

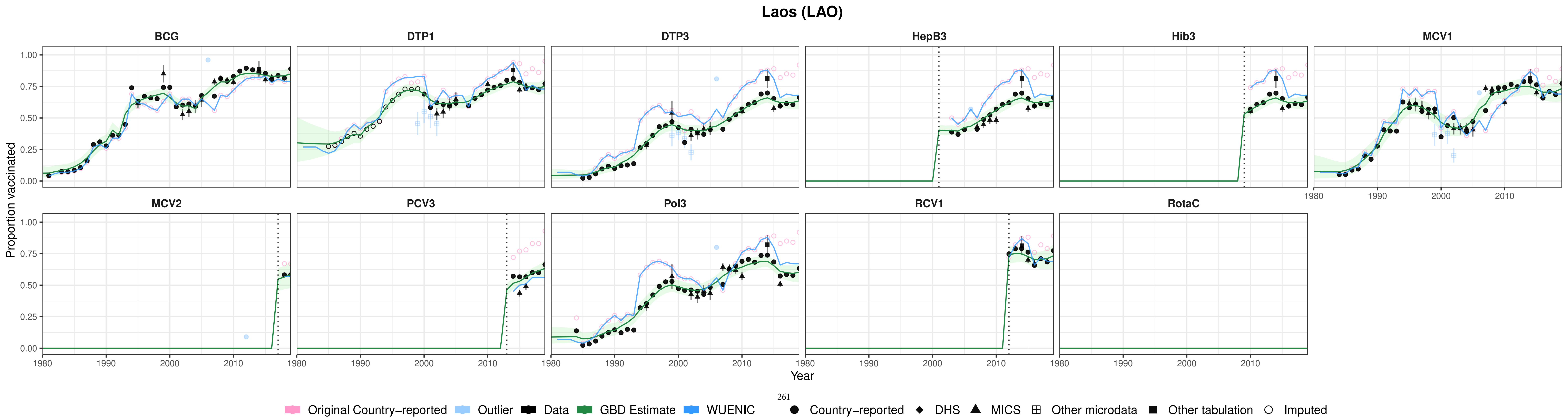

Laos (LAO)
BCG
DTP1
DTP3
HepB3
Hib3
MCV1
MCV2
PCV3
Pol3
RCV1
RotaC
Proportion vaccinated
Year
1.00
0.75
0.50
0.25
0.00
1980
1990
2000
2010
Original Country−reported
Outlier
Data
GBD Estimate
WUENIC
Country−reported
DHS
MICS
Other microdata
Other tabulation
Imputed

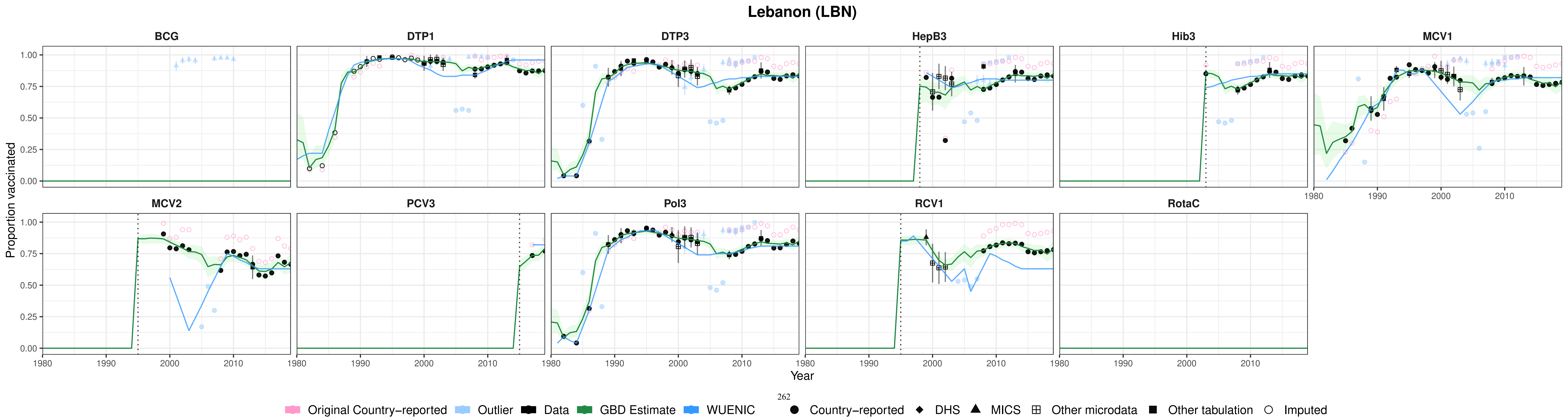

Lebanon (LBN)
BCG
DTP1
DTP3
HepB3
Hib3
MCV1
MCV2
PCV3
Pol3
RCV1
RotaC
Proportion vaccinated
Year
0.00
0.25
0.50
0.75
1.00
1980
1990
2000
2010
Original Country−reported
Outlier
Data
GBD Estimate
WUENIC
Country−reported
DHS
MICS
Other microdata
Other tabulation
Imputed

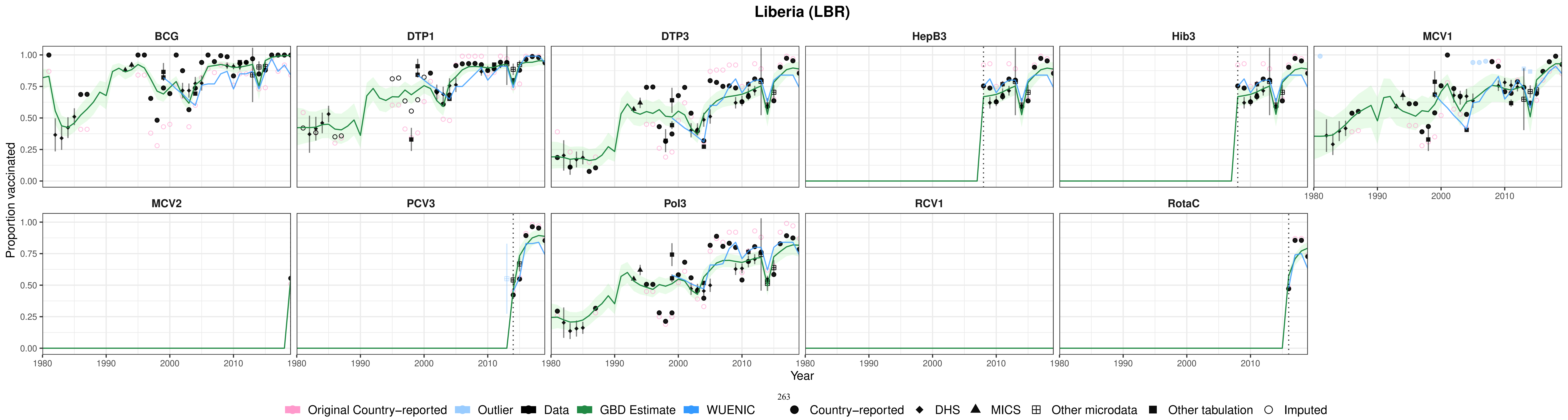

Liberia (LBR)
BCG
DTP1
DTP3
HepB3
Hib3
MCV1
MCV2
PCV3
Pol3
RCV1
RotaC
Proportion vaccinated
Year
Original Country−reported
Outlier
Data
GBD Estimate
WUENIC
Country−reported
DHS
MICS
Other microdata
Other tabulation
Imputed

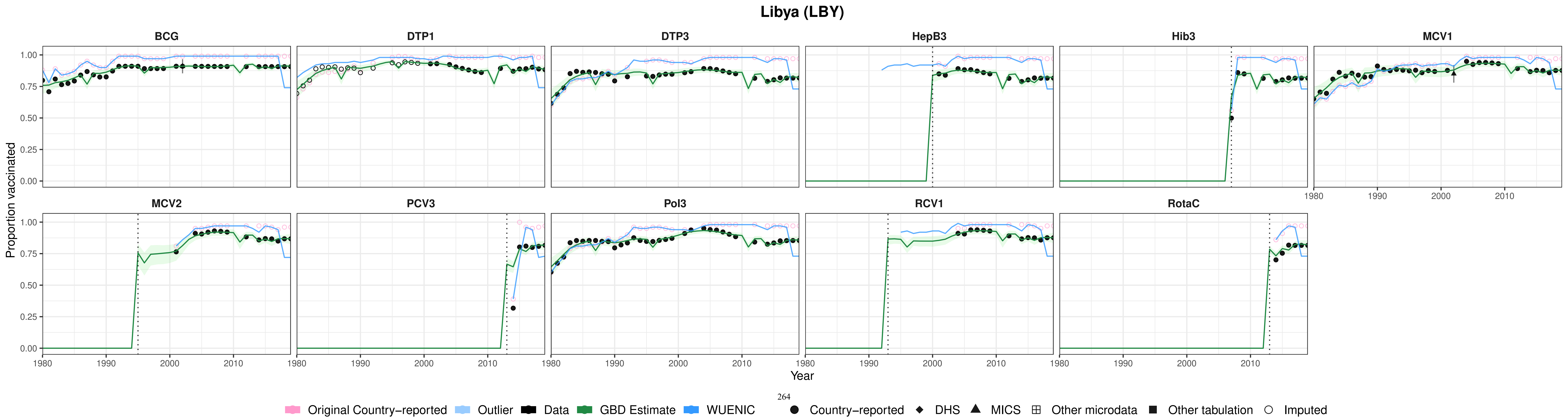
Libya (LBY)
BCG
DTP1
DTP3
HepB3
Hib3
MCV1
MCV2
PCV3
Pol3
RCV1
RotaC
Proportion vaccinated
Year
Original Country−reported
Outlier
Data
GBD Estimate
WUENIC
Country−reported
DHS
MICS
Other microdata
Other tabulation
Imputed

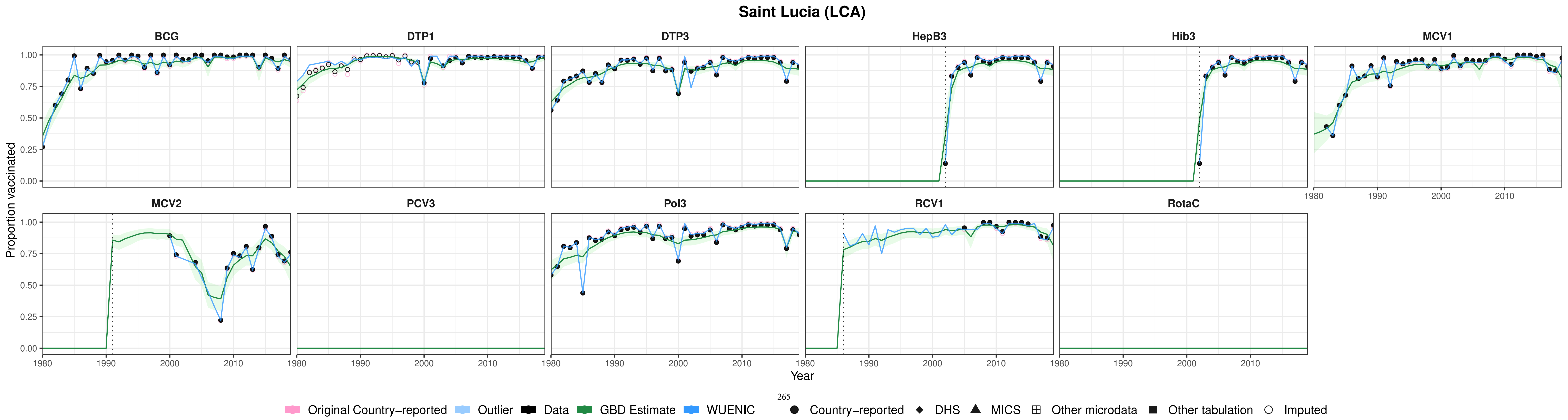
Saint Lucia (LCA)
BCG
DTP1
DTP3
HepB3
Hib3
MCV1
MCV2
PCV3
Pol3
RCV1
RotaC
Proportion vaccinated
Year
Original Country−reported
Outlier
Data
GBD Estimate
WUENIC
Country−reported
DHS
MICS
Other microdata
Other tabulation
Imputed

Sri Lanka (LKA)

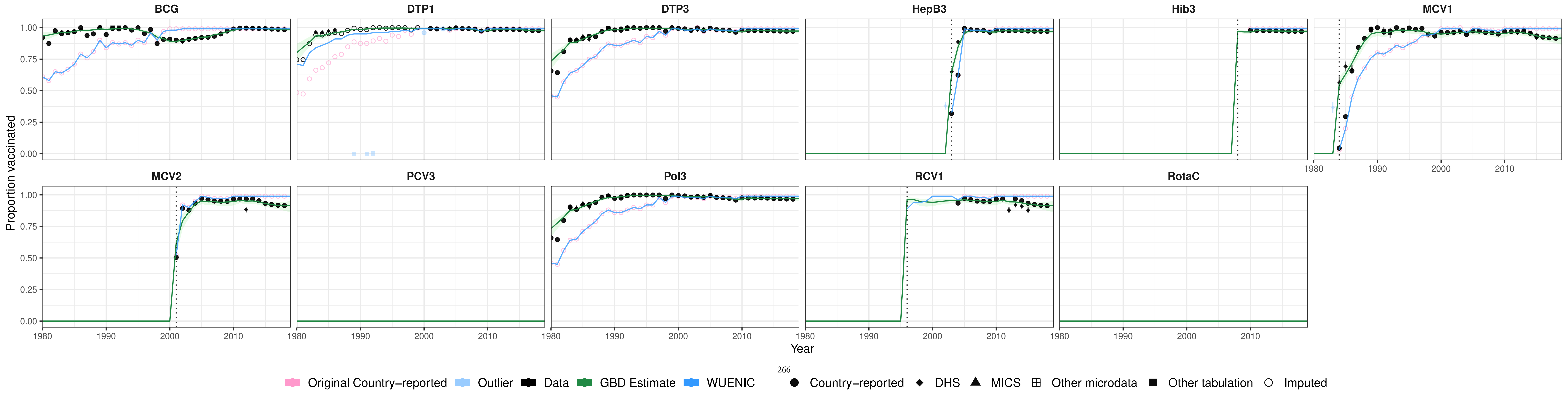

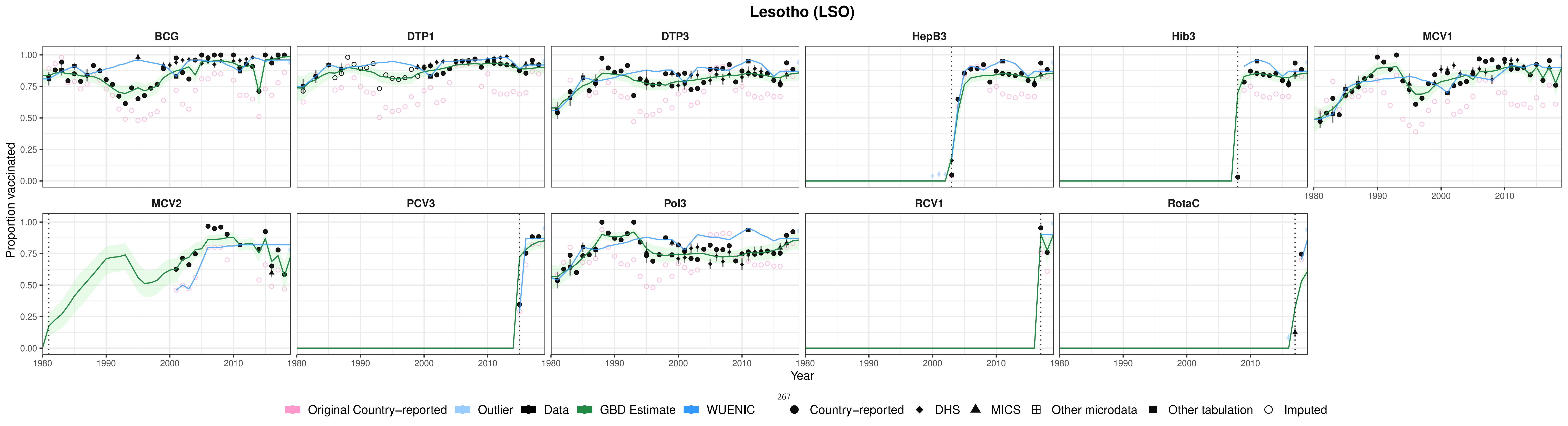
Lesotho (LSO)
BCG
DTP1
DTP3
HepB3
Hib3
MCV1
MCV2
PCV3
Pol3
RCV1
RotaC
Proportion vaccinated
Year
Original Country−reported
Outlier
Data
GBD Estimate
WUENIC
Country−reported
DHS
MICS
Other microdata
Other tabulation
Imputed

Lithuania (LTU)

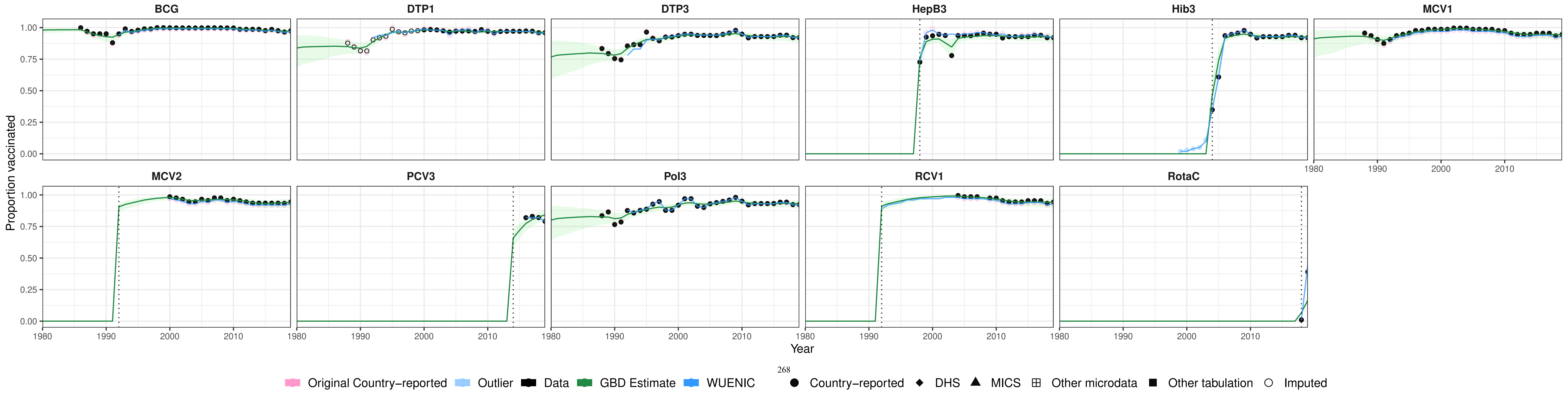

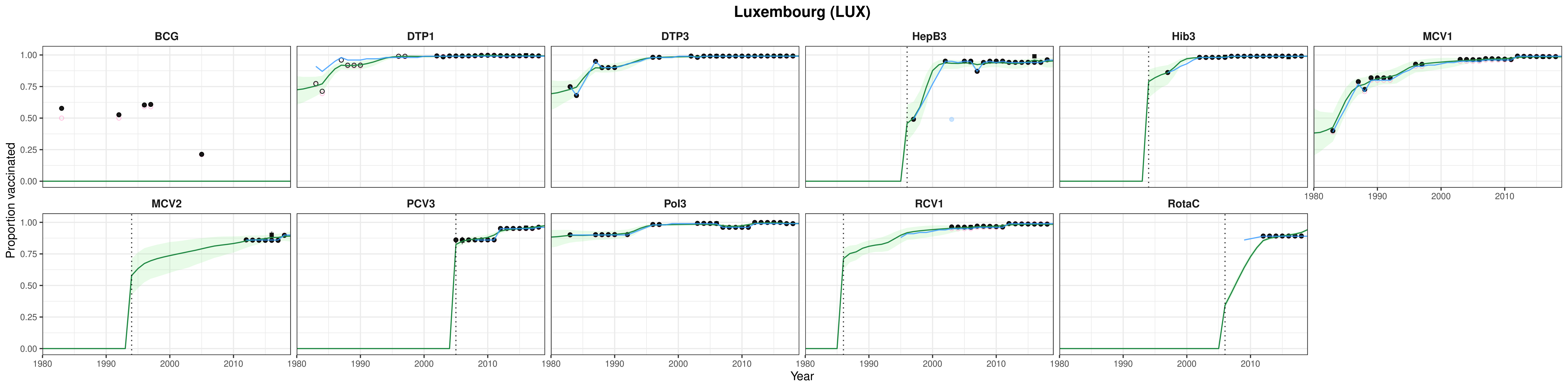

Luxembourg (LUX)
BCG
DTP1
DTP3
HepB3
Hib3
MCV1
MCV2
PCV3
Pol3
RCV1
RotaC
Proportion vaccinated
Year
1.00
0.75
0.50
0.25
0.00
1980
1990
2000
2010
Original Country−reported
Outlier
Data
GBD Estimate
WUENIC
Country−reported
DHS
MICS
Other microdata
Other tabulation
Imputed

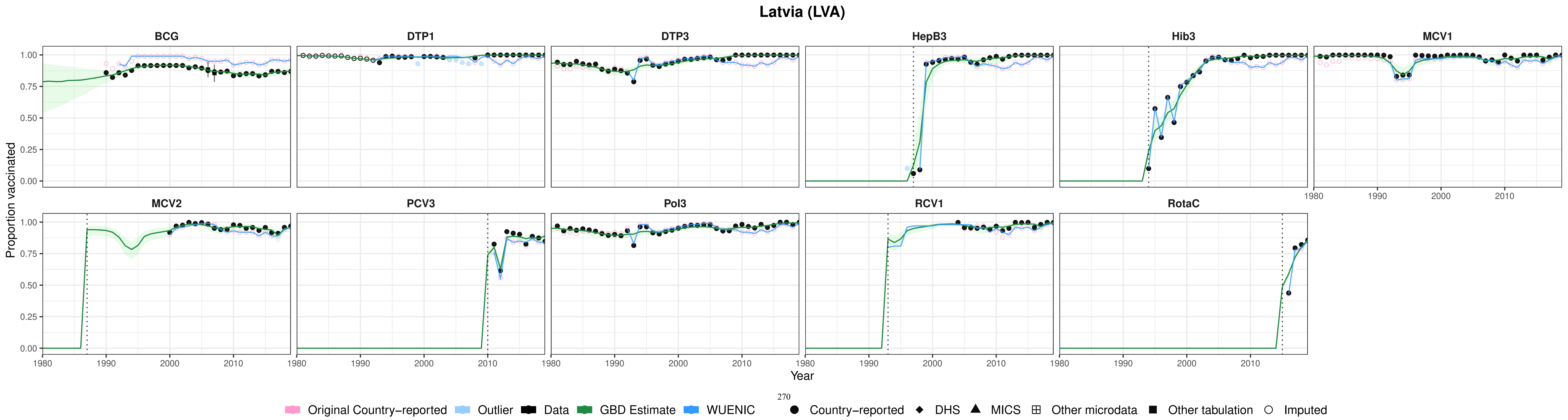

Latvia (LVA)
BCG
DTP1
DTP3
HepB3
Hib3
MCV1
MCV2
PCV3
Pol3
RCV1
RotaC
Proportion vaccinated
Year
Original Country−reported
Outlier
Data
GBD Estimate
WUENIC
Country−reported
DHS
MICS
Other microdata
Other tabulation
Imputed

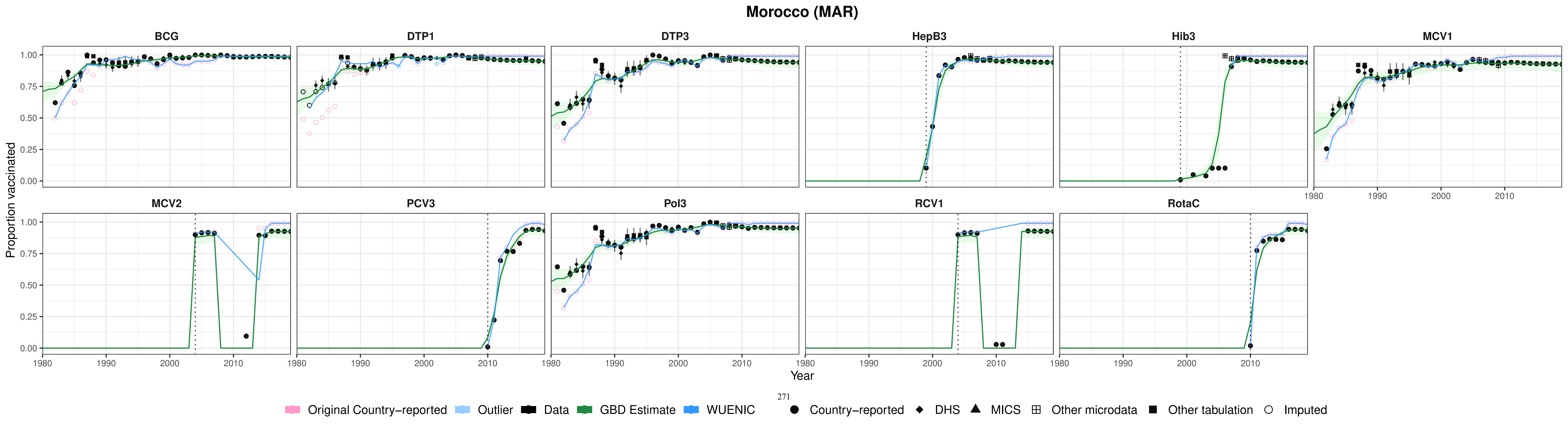
Morocco (MAR)
BCG
DTP1
DTP3
HepB3
Hib3
MCV1
MCV2
PCV3
Pol3
RCV1
RotaC
Proportion vaccinated
Year
Original Country−reported
Outlier
Data
GBD Estimate
WUENIC
Country−reported
DHS
MICS
Other microdata
Other tabulation
Imputed

Monaco (MCO)

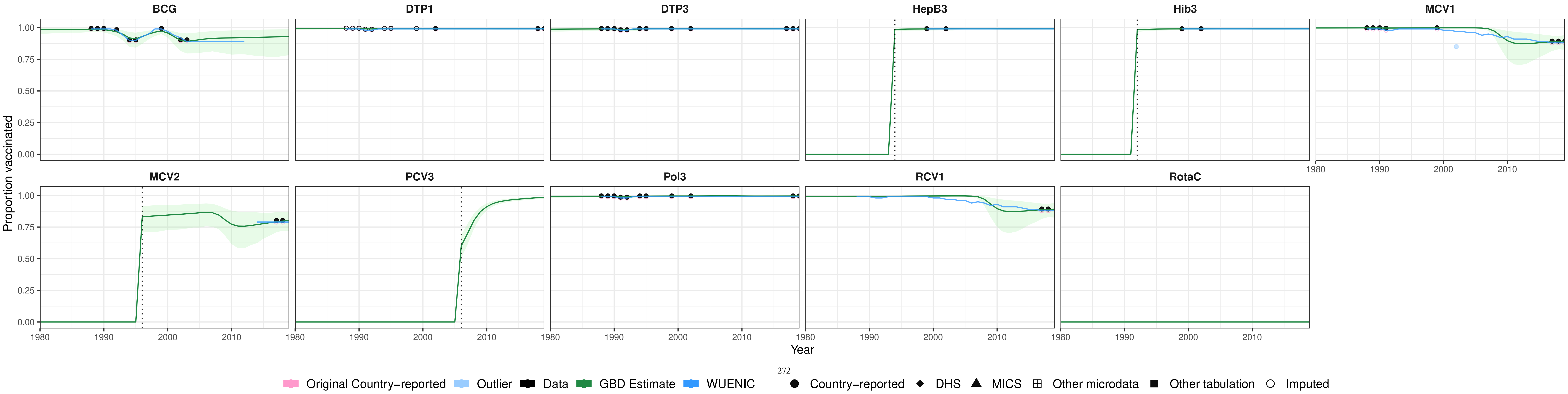
BCG
DTP1
DTP3
HepB3
Hib3
MCV1
MCV2
PCV3
Pol3
RCV1
RotaC
Proportion vaccinated
Year
1.00
0.75
0.50
0.25
0.00
1980
1990
2000
2010
Original Country−reported
Outlier
Data
GBD Estimate
WUENIC
Country−reported
DHS
MICS
Other microdata
Other tabulation
Imputed

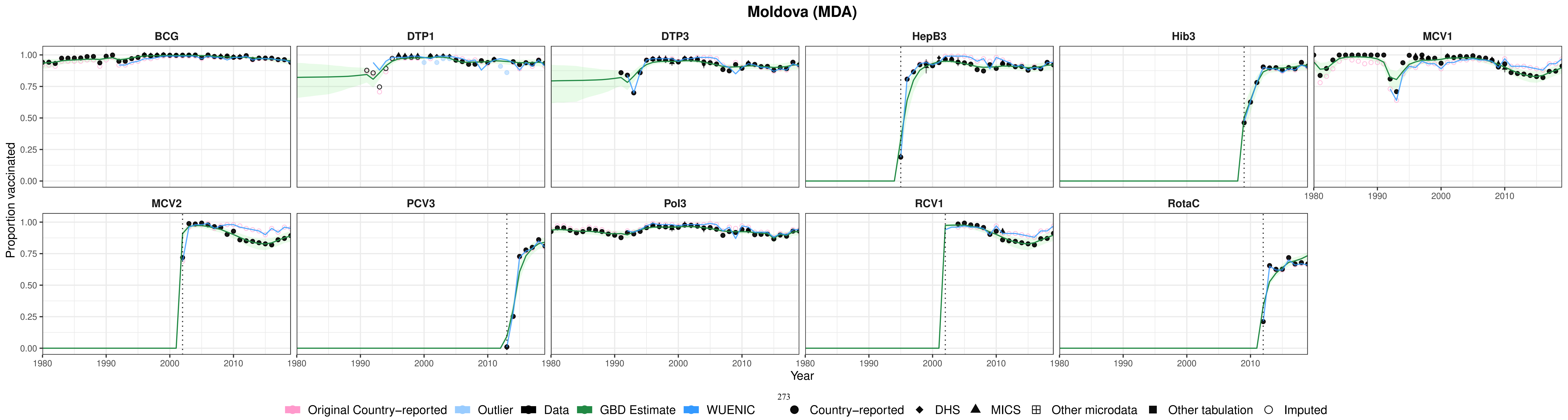

Moldova (MDA)
BCG
DTP1
DTP3
HepB3
Hib3
MCV1
MCV2
PCV3
Pol3
RCV1
RotaC
Proportion vaccinated
Year
0.00
0.25
0.50
0.75
1.00
1980
1990
2000
2010
Original Country−reported
Outlier
Data
GBD Estimate
WUENIC
Country−reported
DHS
MICS
Other microdata
Other tabulation
Imputed

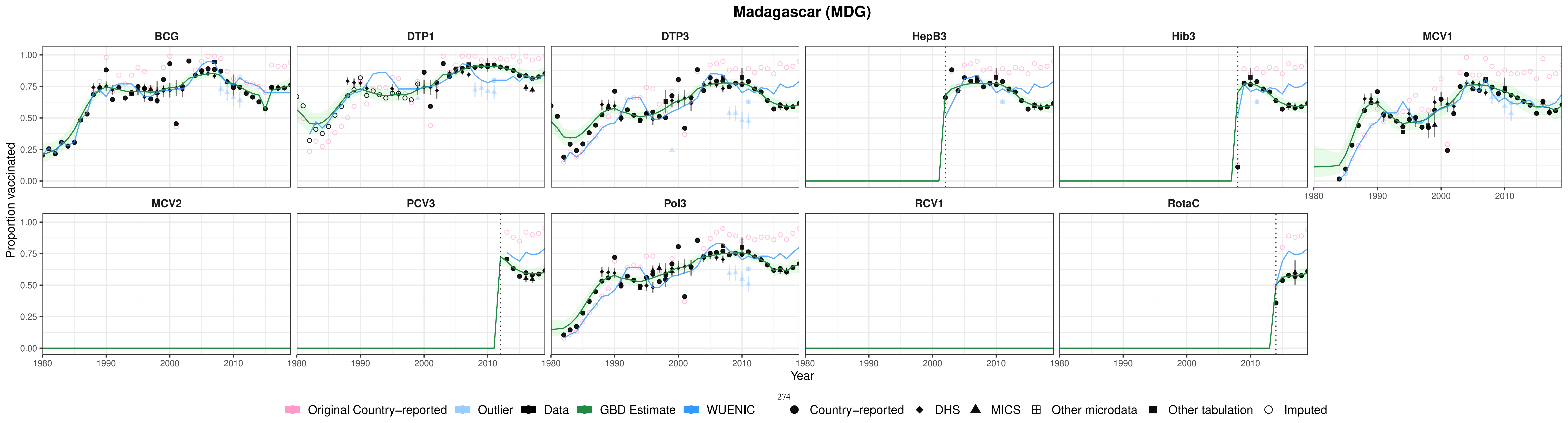

Madagascar (MDG)
BCG
DTP1
DTP3
HepB3
Hib3
MCV1
MCV2
PCV3
Pol3
RCV1
RotaC
Proportion vaccinated
Year
1.00
0.75
0.50
0.25
0.00
1980
1990
2000
2010
Original Country−reported
Outlier
Data
GBD Estimate
WUENIC
Country−reported
DHS
MICS
Other microdata
Other tabulation
Imputed

Maldives (MDV)

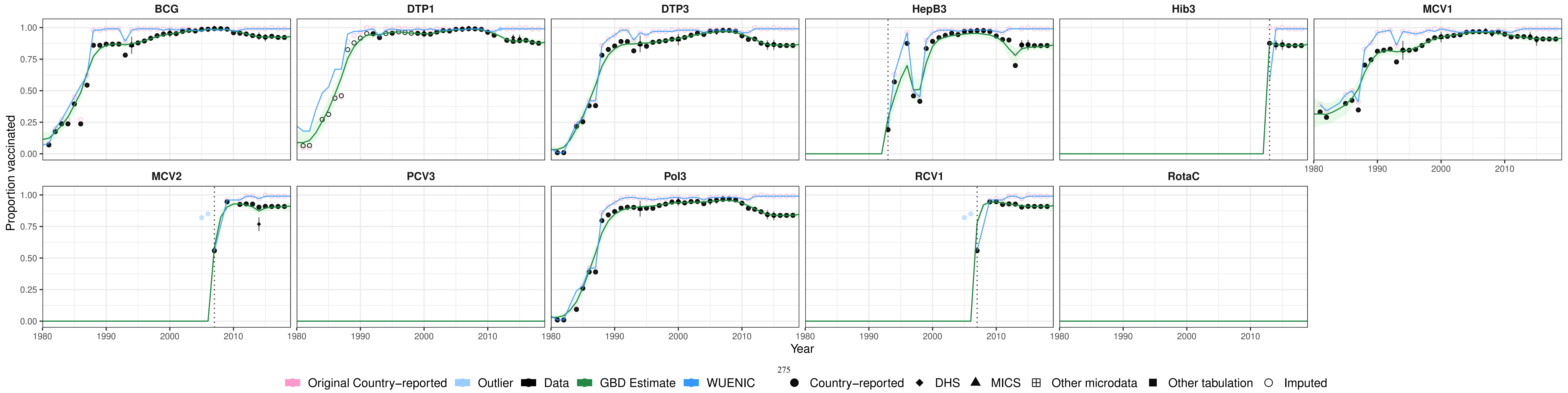

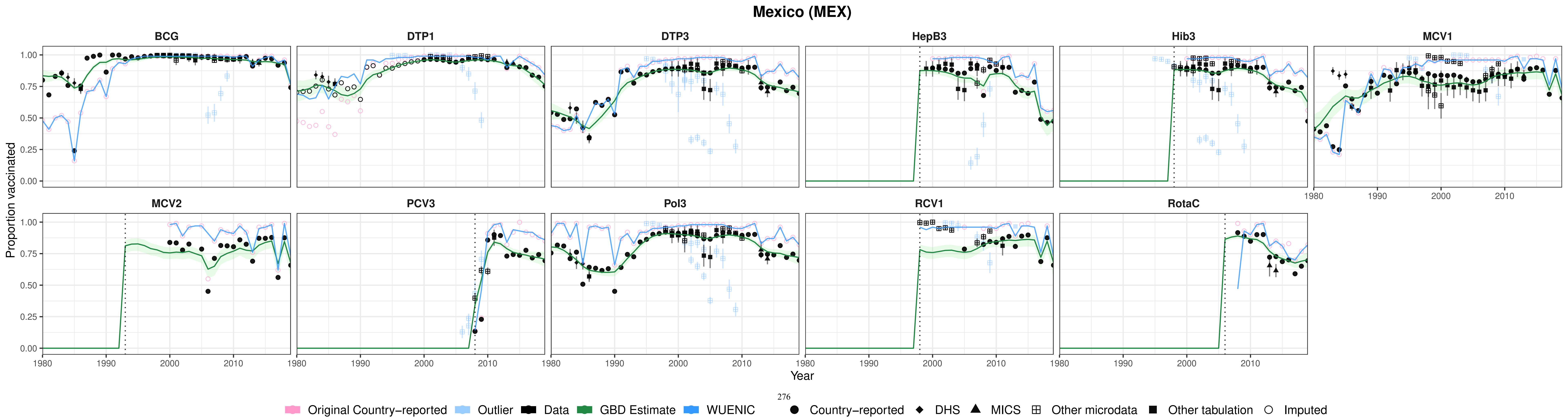
Mexico (MEX)
BCG
DTP1
DTP3
HepB3
Hib3
MCV1
MCV2
PCV3
Pol3
RCV1
RotaC
Proportion vaccinated
Year
Original Country−reported
Outlier
Data
GBD Estimate
WUENIC
Country−reported
DHS
MICS
Other microdata
Other tabulation
Imputed

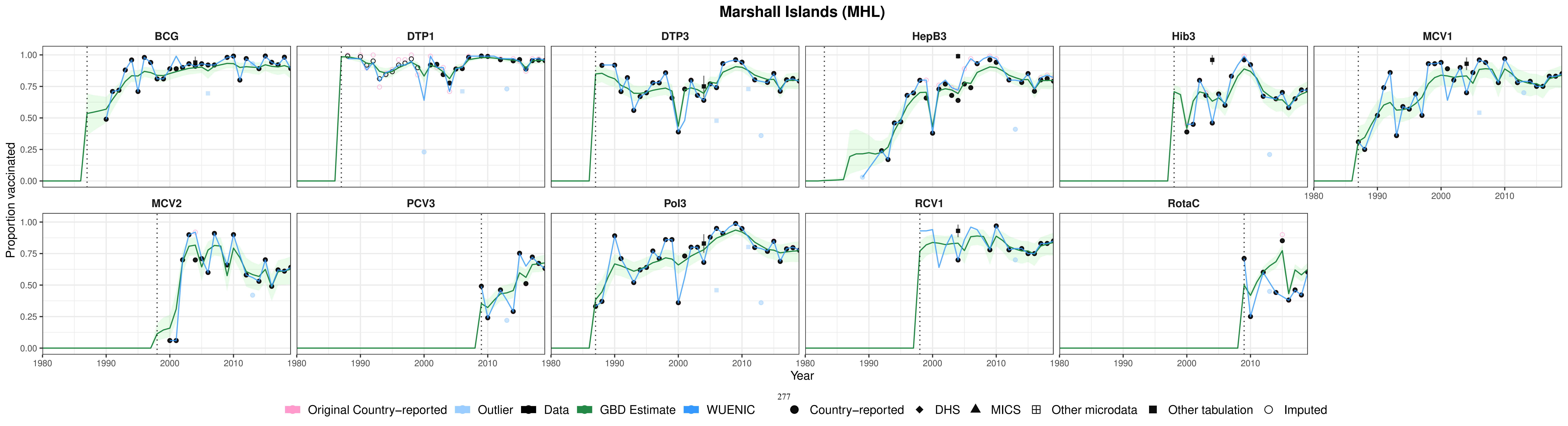

Marshall Islands (MHL)
BCG
DTP1
DTP3
HepB3
Hib3
MCV1
MCV2
PCV3
Pol3
RCV1
RotaC
Proportion vaccinated
Year
Original Country−reported
Outlier
Data
GBD Estimate
WUENIC
Country−reported
DHS
MICS
Other microdata
Other tabulation
Imputed

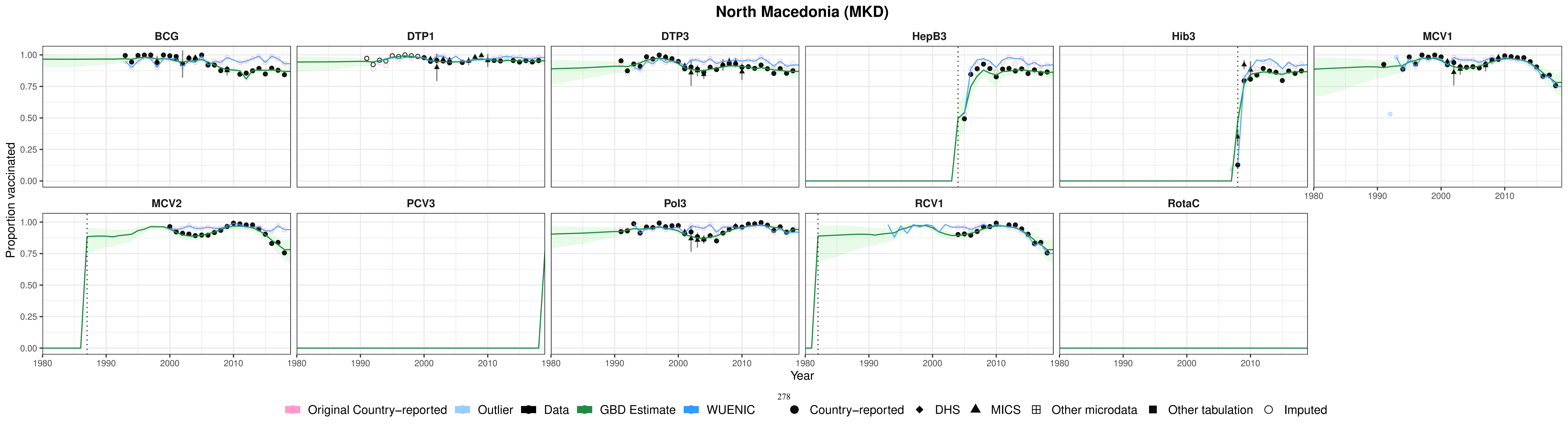

North Macedonia (MKD)
BCG
DTP1
DTP3
HepB3
Hib3
MCV1
MCV2
PCV3
Pol3
RCV1
RotaC
Proportion vaccinated
0.00
0.25
0.50
0.75
1.00
1980
1990
2000
2010
Year
Original Country−reported
Outlier
Data
GBD Estimate
WUENIC
Country−reported
DHS
MICS
Other microdata
Other tabulation
Imputed

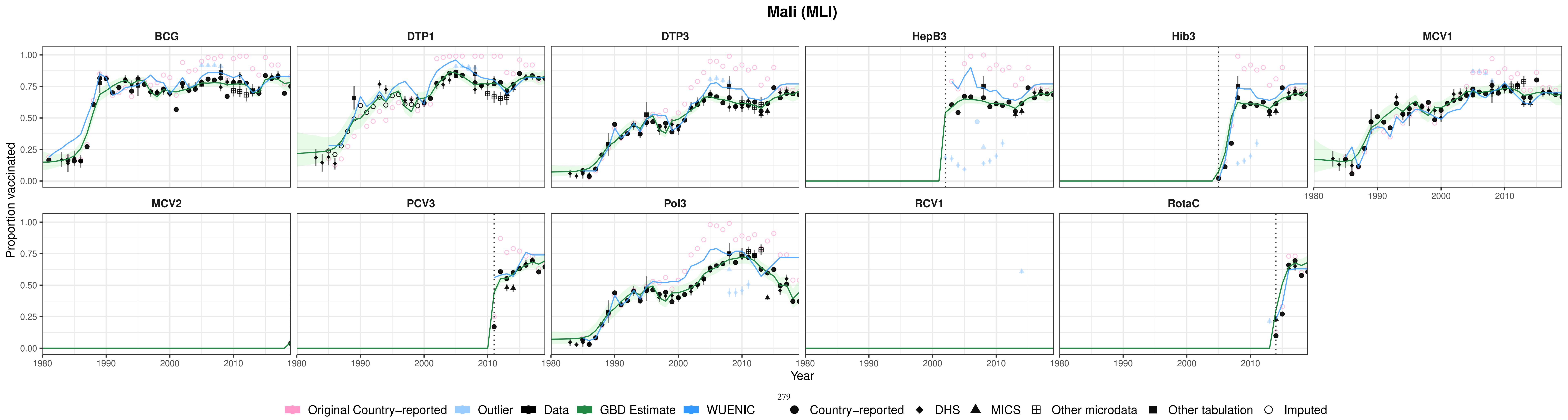
Mali (MLI)
BCG
DTP1
DTP3
HepB3
Hib3
MCV1
MCV2
PCV3
Pol3
RCV1
RotaC
Proportion vaccinated
Year
Original Country−reported
Outlier
Data
GBD Estimate
WUENIC
Country−reported
DHS
MICS
Other microdata
Other tabulation
Imputed

Malta (MLT)

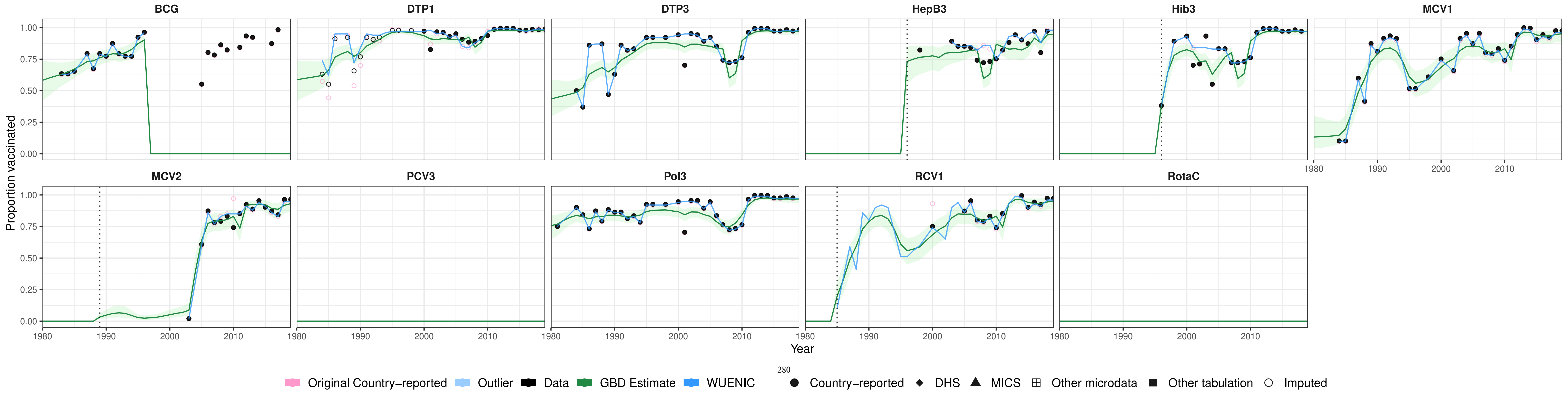

BCG
DTP1
DTP3
HepB3
Hib3
MCV1
MCV2
PCV3
Pol3
RCV1
RotaC
Proportion vaccinated
Year
Original Country−reported
Outlier
Data
GBD Estimate
WUENIC
Country−reported
DHS
MICS
Other microdata
Other tabulation
Imputed

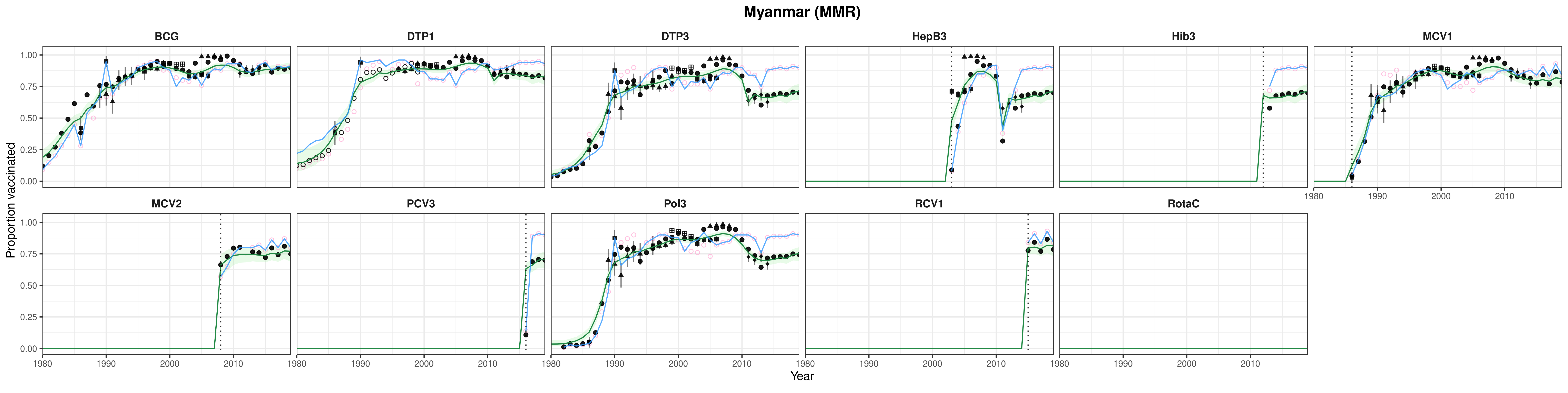

Myanmar (MMR)
BCG
DTP1
DTP3
HepB3
Hib3
MCV1
MCV2
PCV3
Pol3
RCV1
RotaC
Proportion vaccinated
Year
0.00
0.25
0.50
0.75
1.00
1980
1990
2000
2010
Original Country−reported
Outlier
Data
GBD Estimate
WUENIC
Country−reported
DHS
MICS
Other microdata
Other tabulation
Imputed

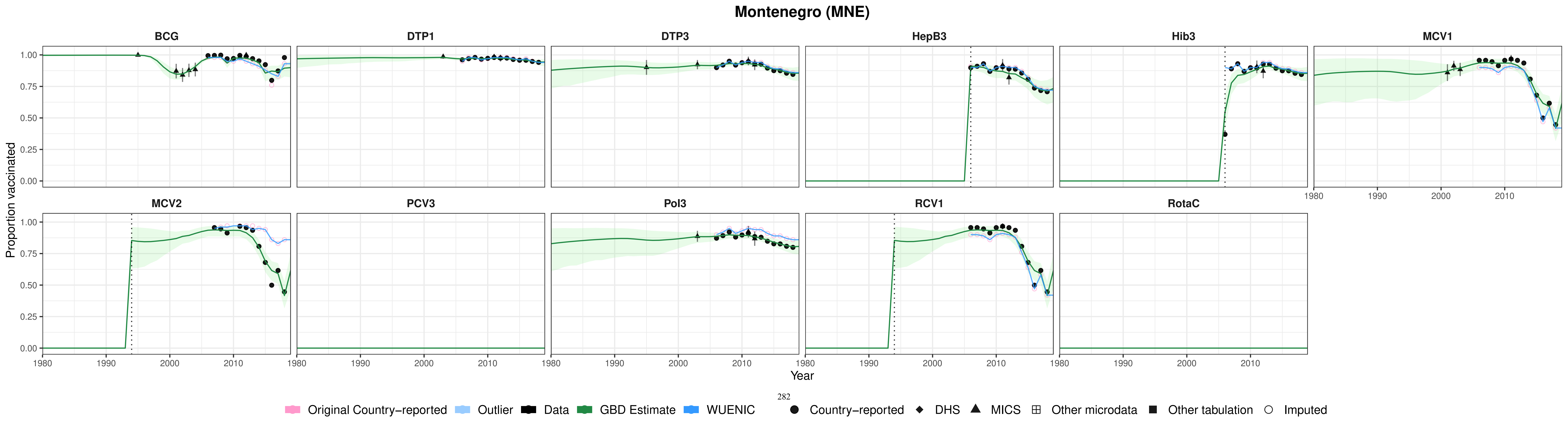

Montenegro (MNE)
BCG
DTP1
DTP3
HepB3
Hib3
MCV1
MCV2
PCV3
Pol3
RCV1
RotaC
Proportion vaccinated
Year
1.00
0.75
0.50
0.25
0.00
1980
1990
2000
2010
Original Country−reported
Outlier
Data
GBD Estimate
WUENIC
Country−reported
DHS
MICS
Other microdata
Other tabulation
Imputed

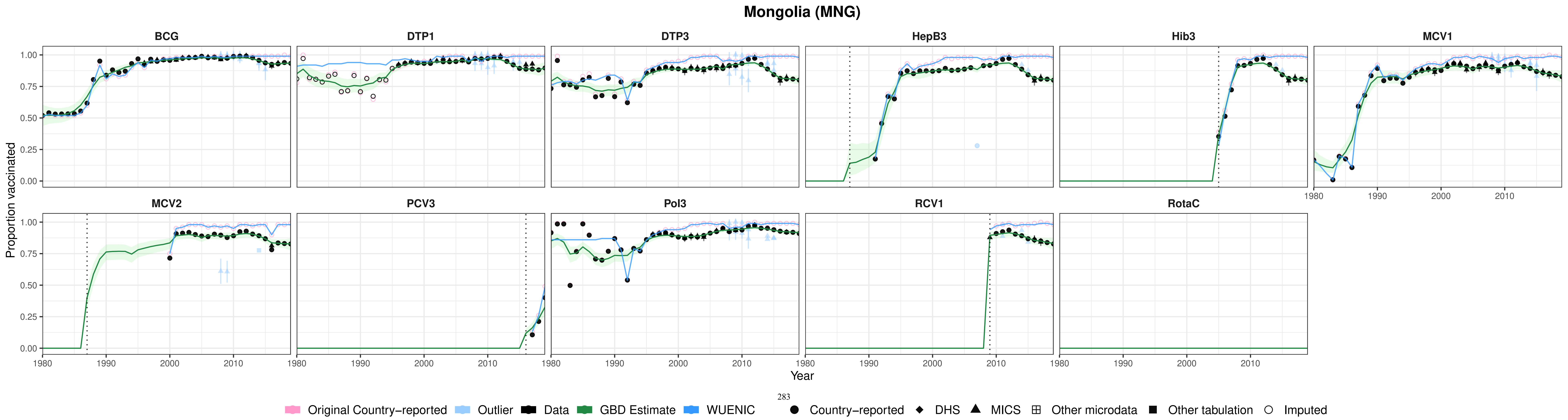
Mongolia (MNG)
BCG
DTP1
DTP3
HepB3
Hib3
MCV1
MCV2
PCV3
Pol3
RCV1
RotaC
Proportion vaccinated
Year
Original Country−reported
Outlier
Data
GBD Estimate
WUENIC
Country−reported
DHS
MICS
Other microdata
Other tabulation
Imputed

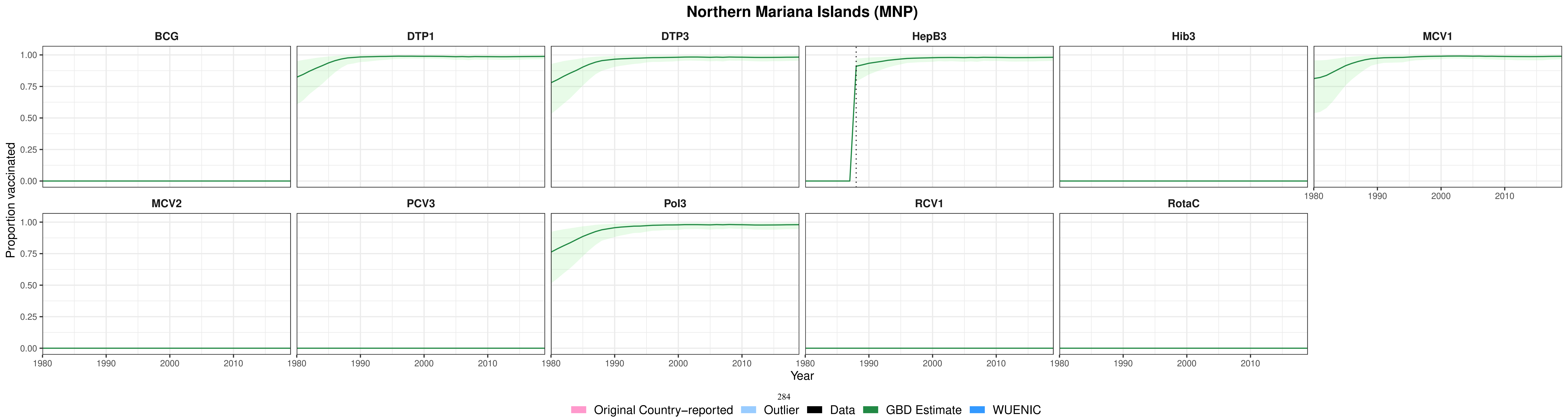

Northern Mariana Islands (MNP)
BCG
DTP1
DTP3
HepB3
Hib3
MCV1
MCV2
PCV3
Pol3
RCV1
RotaC
Proportion vaccinated
0.00
0.25
0.50
0.75
1.00
1980
1990
2000
2010
Year
Original Country−reported
Outlier
Data
GBD Estimate
WUENIC

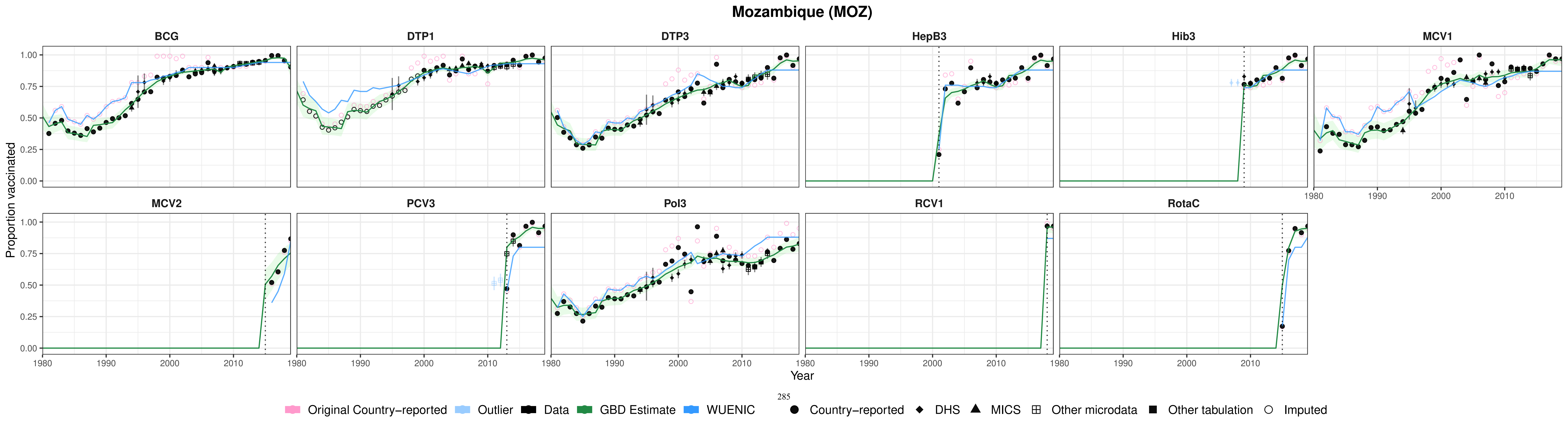

Mozambique (MOZ)
BCG
DTP1
DTP3
HepB3
Hib3
MCV1
MCV2
PCV3
Pol3
RCV1
RotaC
Proportion vaccinated
Year
0.00
0.25
0.50
0.75
1.00
1980
1990
2000
2010
Original Country−reported
Outlier
Data
GBD Estimate
WUENIC
Country−reported
DHS
MICS
Other microdata
Other tabulation
Imputed

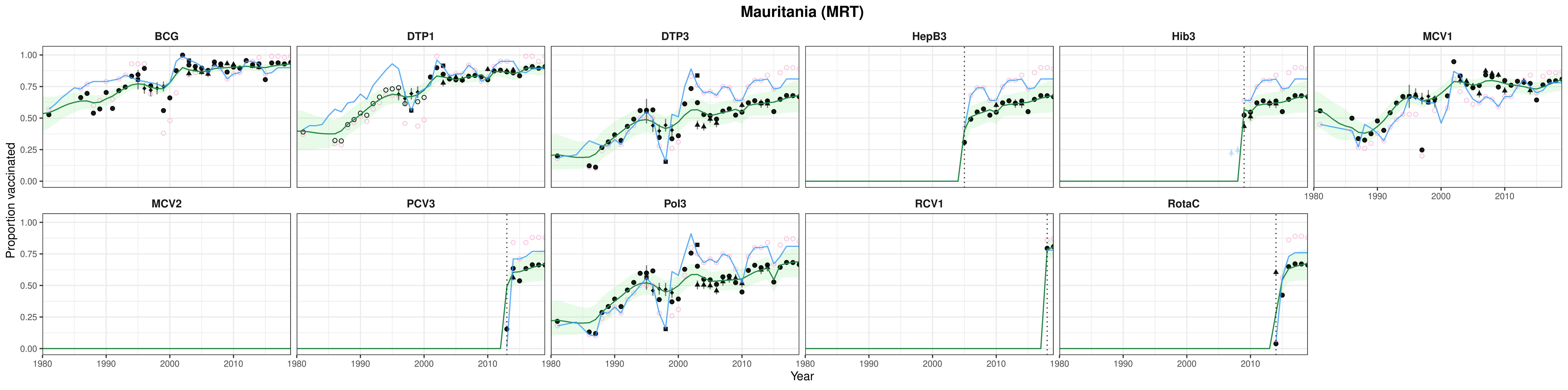

Mauritania (MRT)
BCG
DTP1
DTP3
HepB3
Hib3
MCV1
MCV2
PCV3
Pol3
RCV1
RotaC
Proportion vaccinated
Year
Original Country−reported
Outlier
Data
GBD Estimate
WUENIC
Country−reported
DHS
MICS
Other microdata
Other tabulation
Imputed

Mauritius (MUS)

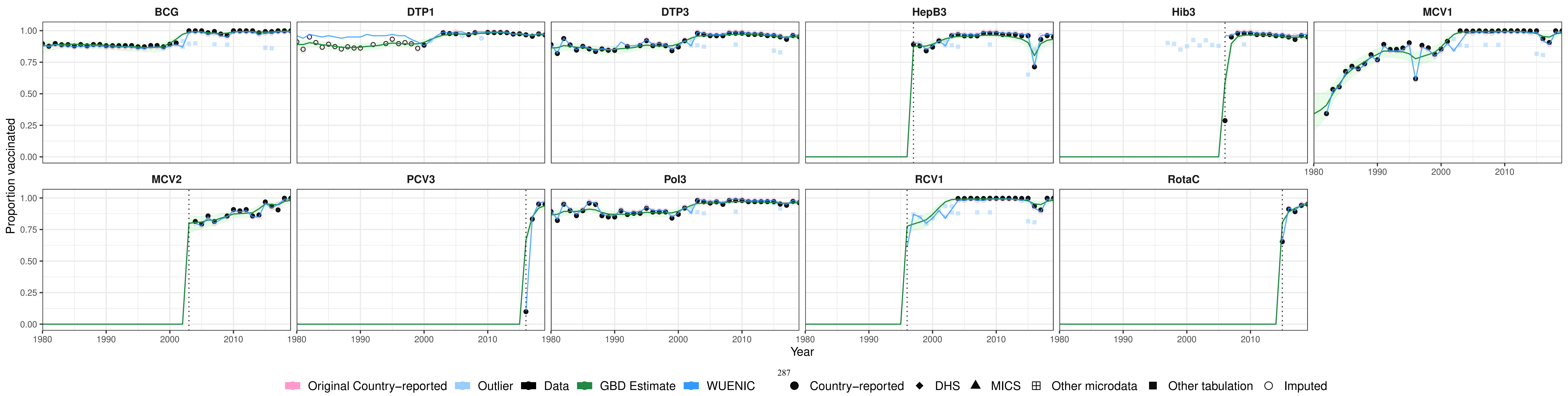

Malawi (MWI)

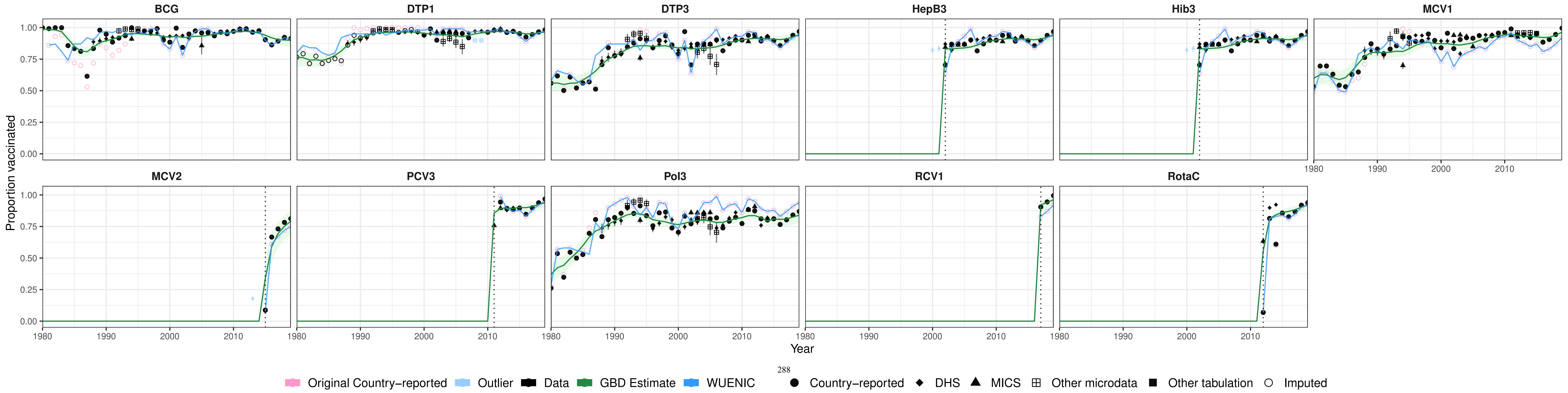

BCG
DTP1
DTP3
HepB3
Hib3
MCV1
MCV2
PCV3
Pol3
RCV1
RotaC
Proportion vaccinated
0.00
0.25
0.50
0.75
1.00
1980
1990
2000
2010
Year
Original Country−reported
Outlier
Data
GBD Estimate
WUENIC
Country−reported
DHS
MICS
Other microdata
Other tabulation
Imputed

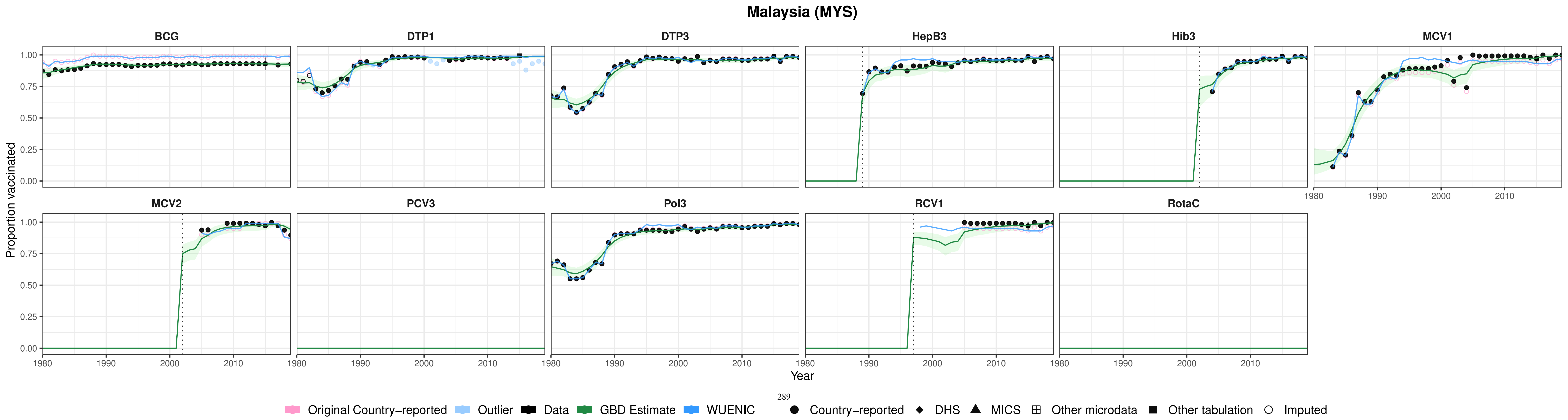

Malaysia (MYS)
BCG
DTP1
DTP3
HepB3
Hib3
MCV1
MCV2
PCV3
Pol3
RCV1
RotaC
Proportion vaccinated
Year
Original Country−reported
Outlier
Data
GBD Estimate
WUENIC
Country−reported
DHS
MICS
Other microdata
Other tabulation
Imputed

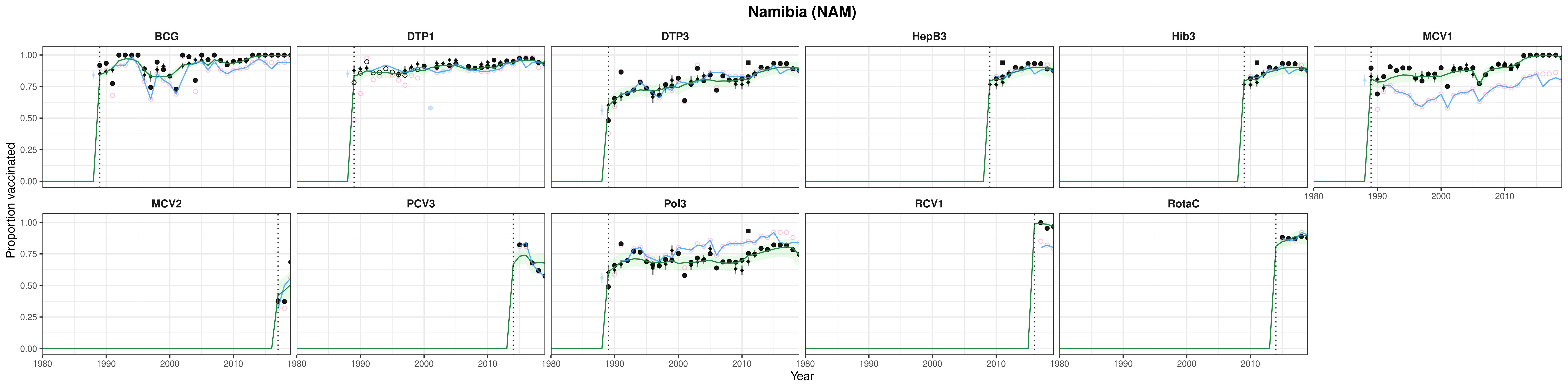

Namibia (NAM)
BCG
DTP1
DTP3
HepB3
Hib3
MCV1
MCV2
PCV3
Pol3
RCV1
RotaC
Proportion vaccinated
Year
Original Country−reported
Outlier
Data
GBD Estimate
WUENIC
Country−reported
DHS
MICS
Other microdata
Other tabulation
Imputed

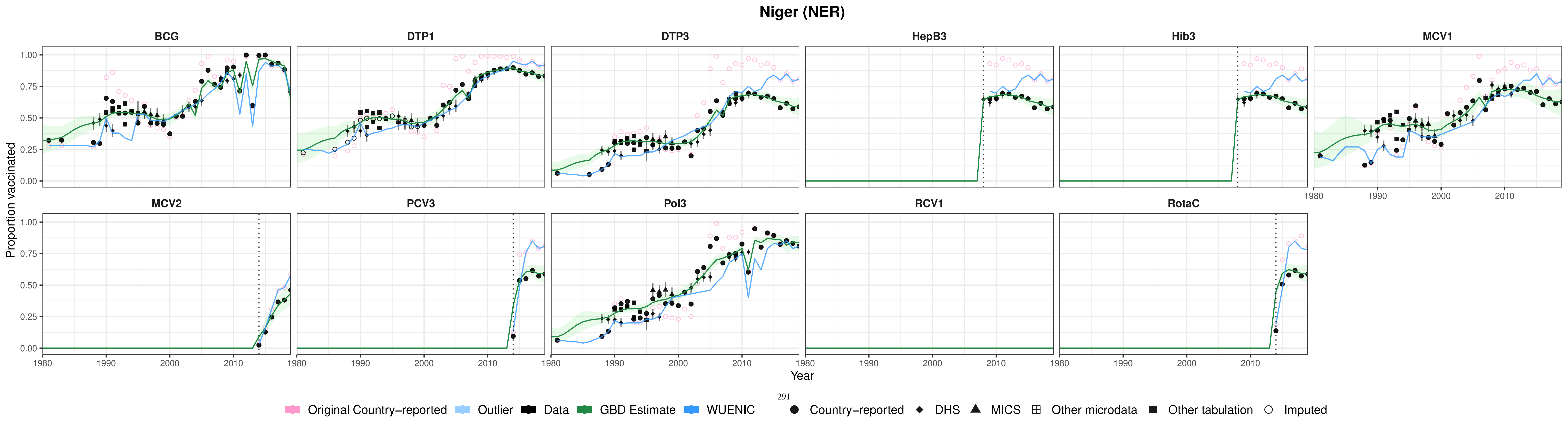
Niger (NER)
BCG
DTP1
DTP3
HepB3
Hib3
MCV1
MCV2
PCV3
Pol3
RCV1
RotaC
Proportion vaccinated
Year
Original Country−reported
Outlier
Data
GBD Estimate
WUENIC
Country−reported
DHS
MICS
Other microdata
Other tabulation
Imputed

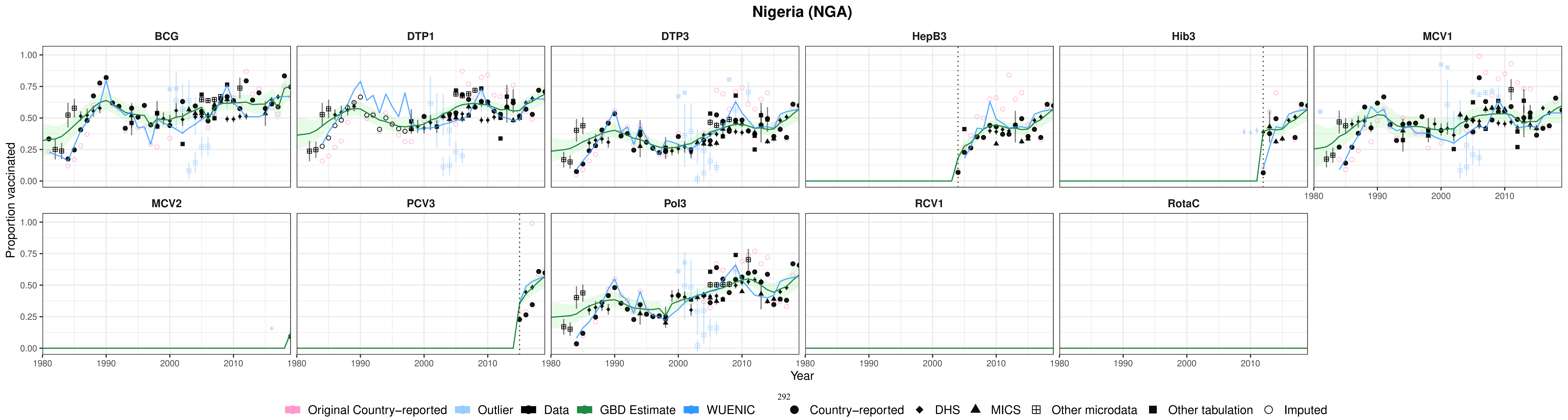

Nigeria (NGA)
BCG
DTP1
DTP3
HepB3
Hib3
MCV1
MCV2
PCV3
Pol3
RCV1
RotaC
Proportion vaccinated
Year
1.00
0.75
0.50
0.25
0.00
1980
1990
2000
2010
Original Country−reported
Outlier
Data
GBD Estimate
WUENIC
Country−reported
DHS
MICS
Other microdata
Other tabulation
Imputed

Nicaragua (NIC)

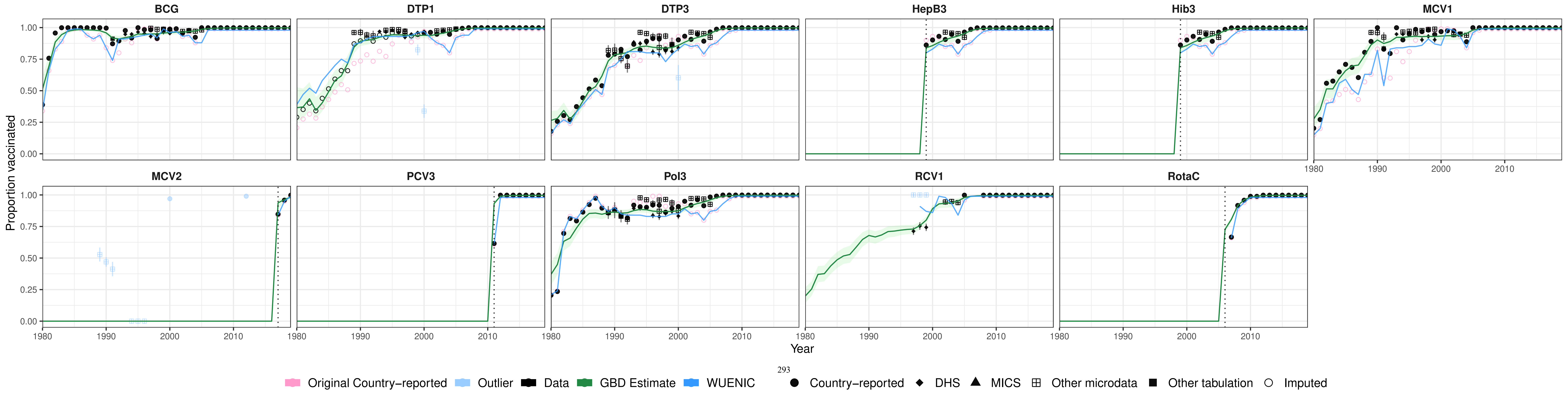

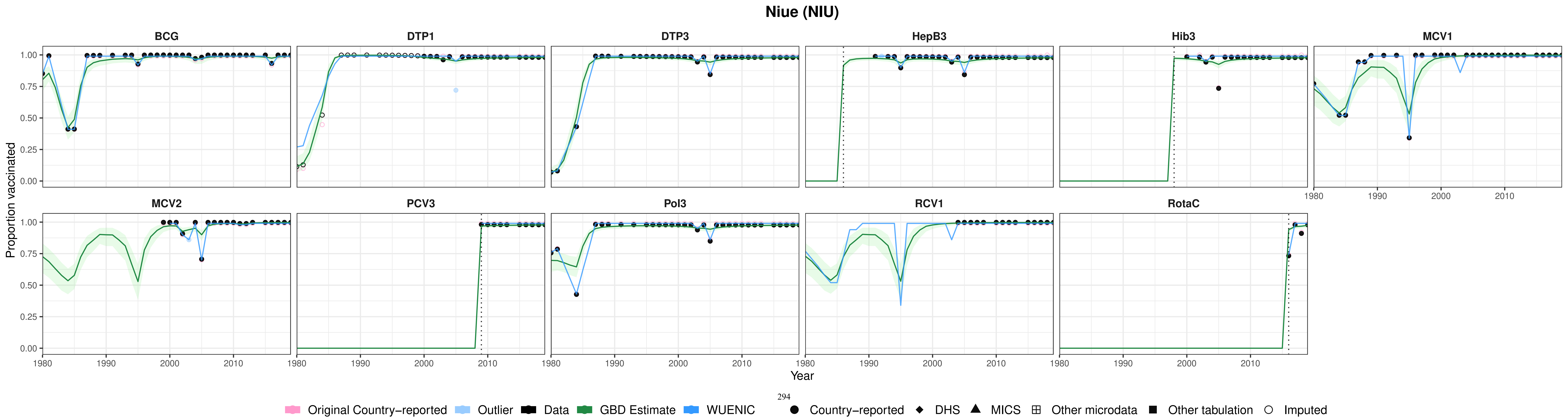
Niue (NIU)
BCG
DTP1
DTP3
HepB3
Hib3
MCV1
MCV2
PCV3
Pol3
RCV1
RotaC
Proportion vaccinated
Year
0.00
0.25
0.50
0.75
1.00
1980
1990
2000
2010
Original Country−reported
Outlier
Data
GBD Estimate
WUENIC
Country−reported
DHS
MICS
Other microdata
Other tabulation
Imputed

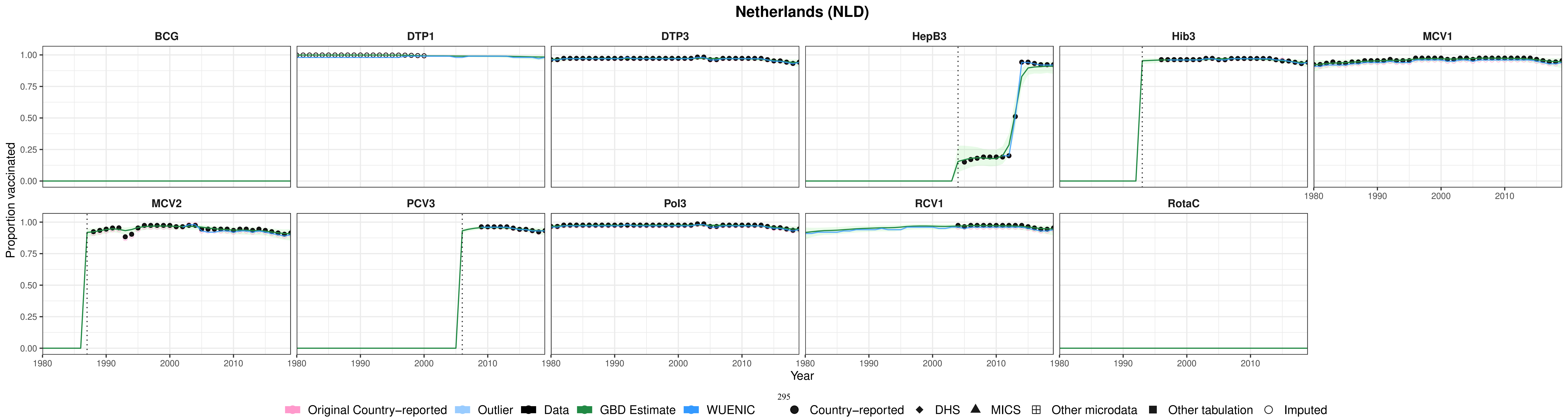
Netherlands (NLD)
BCG
DTP1
DTP3
HepB3
Hib3
MCV1
MCV2
PCV3
Pol3
RCV1
RotaC
Proportion vaccinated
Year
Original Country−reported
Outlier
Data
GBD Estimate
WUENIC
Country−reported
DHS
MICS
Other microdata
Other tabulation
Imputed

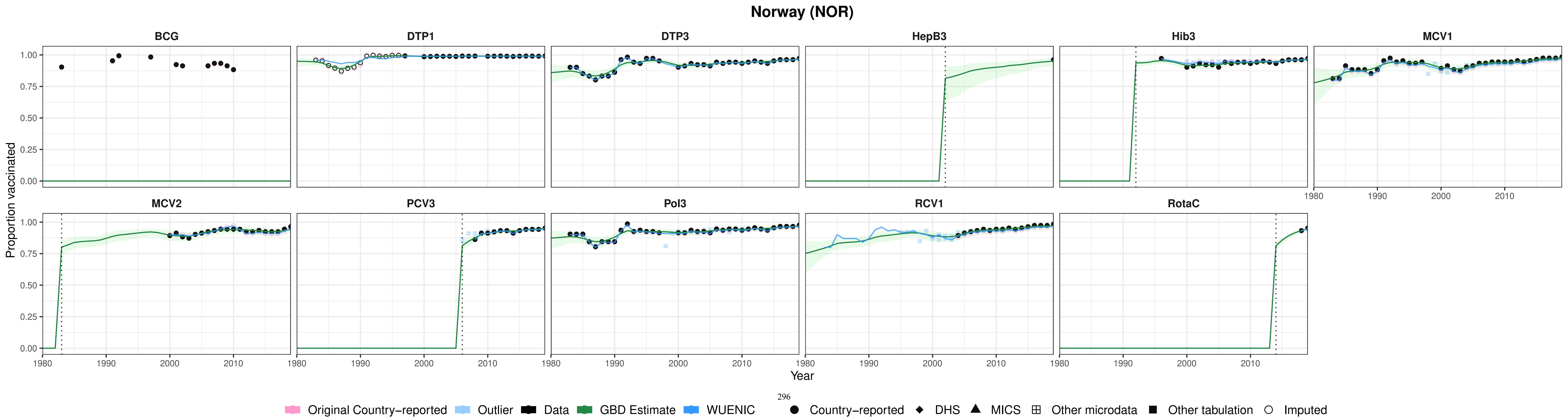

Norway (NOR)
BCG
DTP1
DTP3
HepB3
Hib3
MCV1
MCV2
PCV3
Pol3
RCV1
RotaC
Proportion vaccinated
Year
Original Country−reported
Outlier
Data
GBD Estimate
WUENIC
Country−reported
DHS
MICS
Other microdata
Other tabulation
Imputed

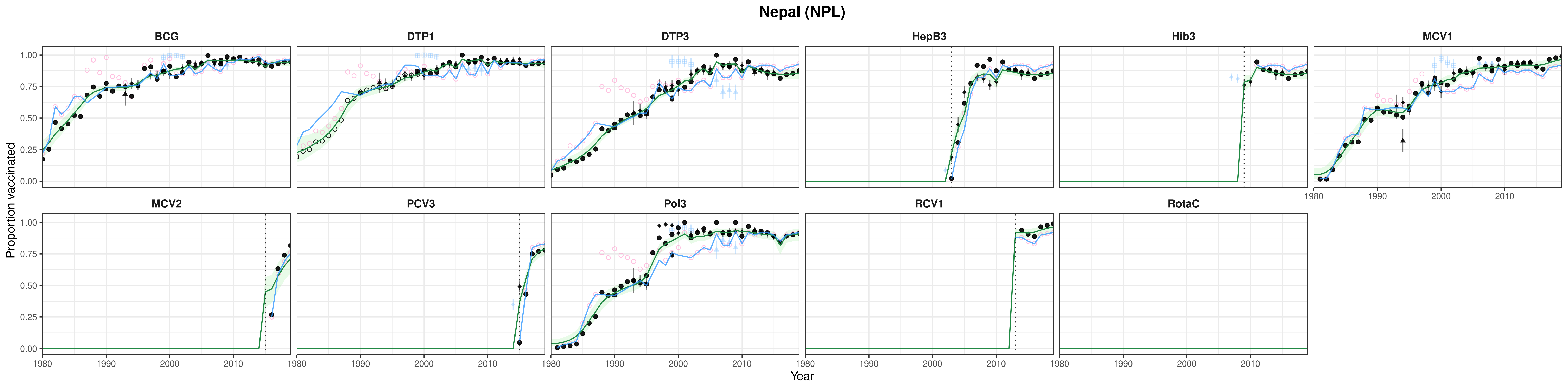

Nepal (NPL)
BCG
DTP1
DTP3
HepB3
Hib3
MCV1
MCV2
PCV3
Pol3
RCV1
RotaC
Proportion vaccinated
Year
0.00
0.25
0.50
0.75
1.00
1980
1990
2000
2010
Original Country−reported
Outlier
Data
GBD Estimate
WUENIC
Country−reported
DHS
MICS
Other microdata
Other tabulation
Imputed

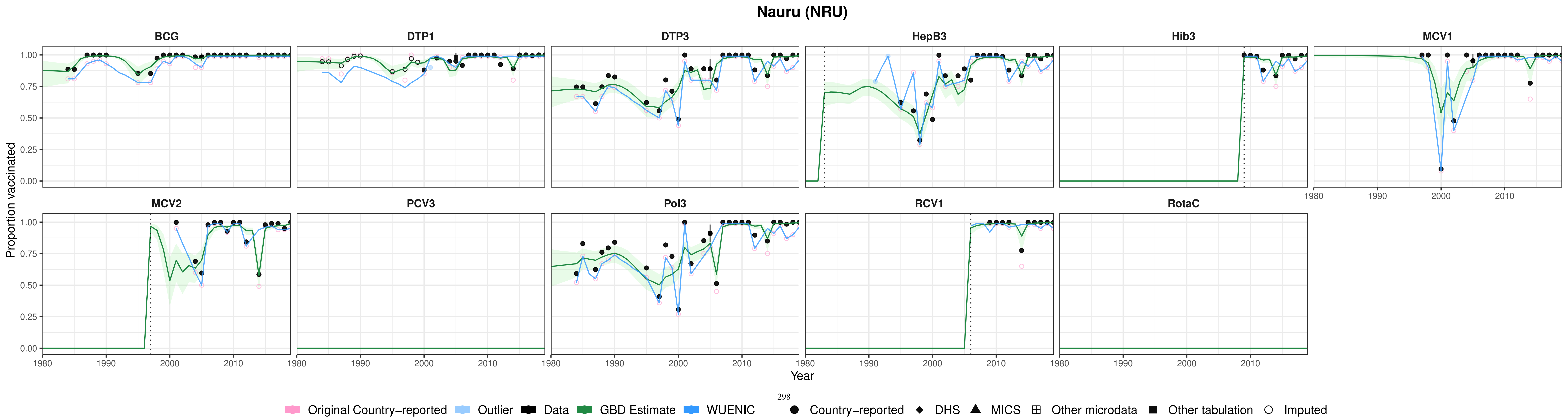

Nauru (NRU)
BCG
DTP1
DTP3
HepB3
Hib3
MCV1
MCV2
PCV3
Pol3
RCV1
RotaC
Proportion vaccinated
Year
0.00
0.25
0.50
0.75
1.00
1980
1990
2000
2010
Original Country−reported
Outlier
Data
GBD Estimate
WUENIC
Country−reported
DHS
MICS
Other microdata
Other tabulation
Imputed

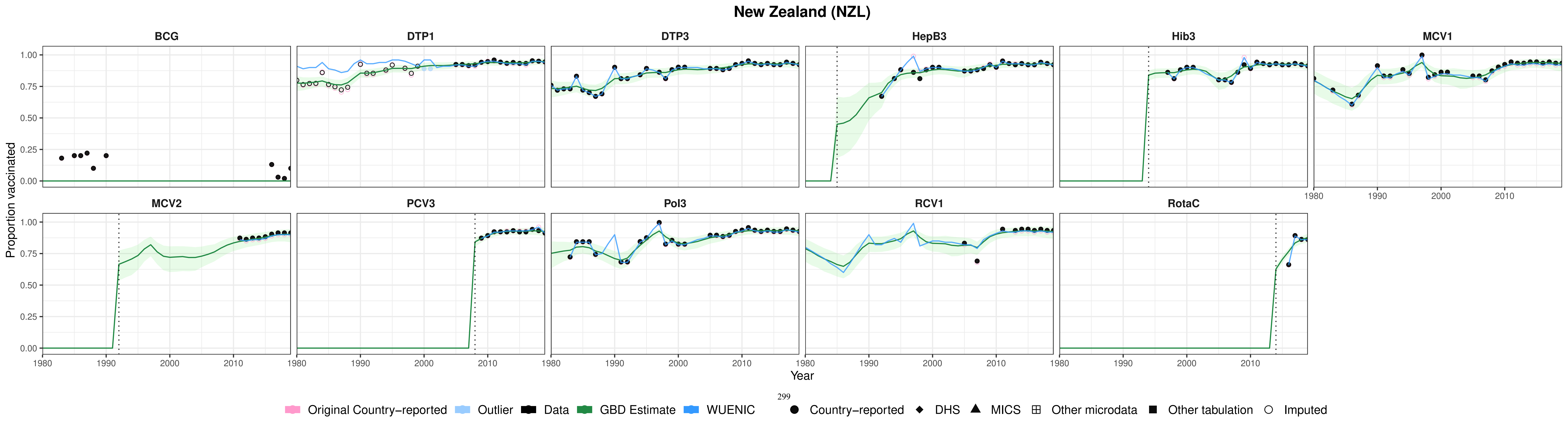
New Zealand (NZL)
BCG
DTP1
DTP3
HepB3
Hib3
MCV1
MCV2
PCV3
Pol3
RCV1
RotaC
Proportion vaccinated
Year
1.00
0.75
0.50
0.25
0.00
1980
1990
2000
2010
Original Country−reported
Outlier
Data
GBD Estimate
WUENIC
Country−reported
DHS
MICS
Other microdata
Other tabulation
Imputed

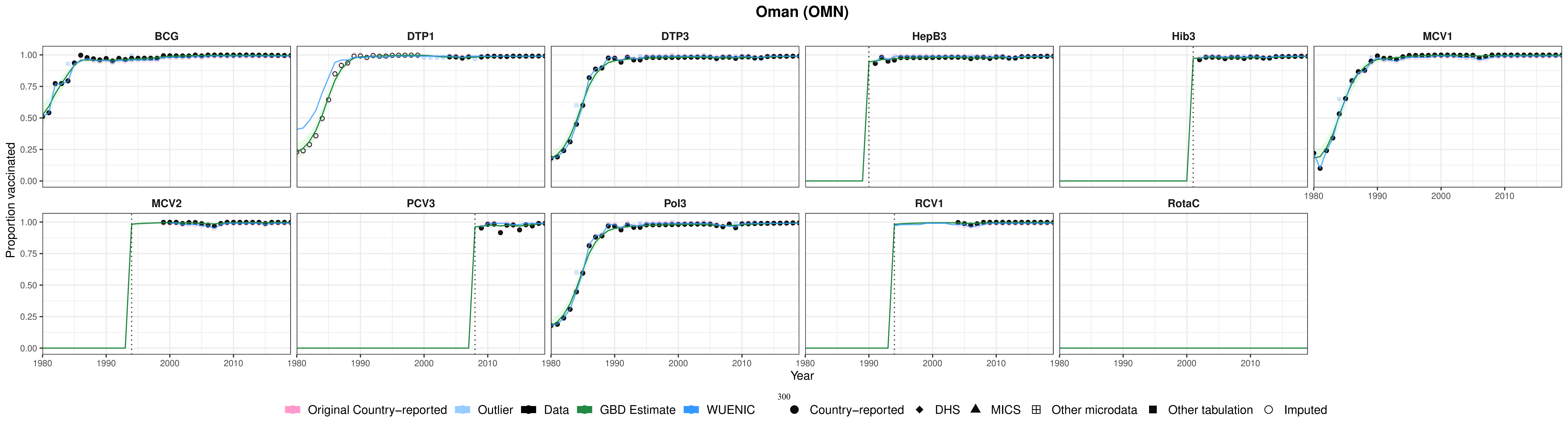

Oman (OMN)
BCG
DTP1
DTP3
HepB3
Hib3
MCV1
MCV2
PCV3
Pol3
RCV1
RotaC
Proportion vaccinated
Year
Original Country−reported
Outlier
Data
GBD Estimate
WUENIC
Country−reported
DHS
MICS
Other microdata
Other tabulation
Imputed

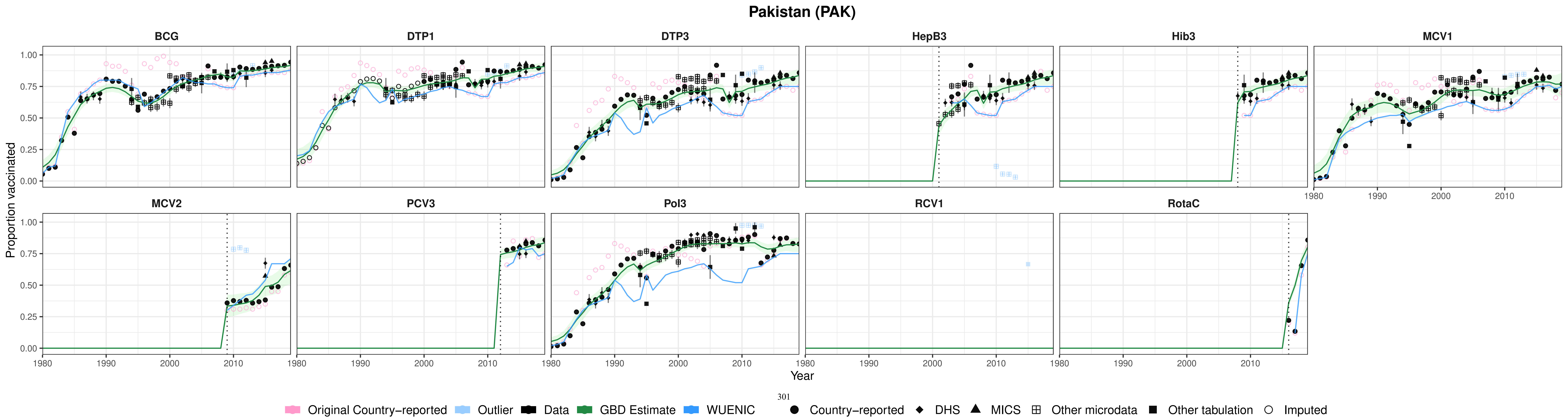

Pakistan (PAK)
BCG
DTP1
DTP3
HepB3
Hib3
MCV1
MCV2
PCV3
Pol3
RCV1
RotaC
Proportion vaccinated
Year
Original Country−reported
Outlier
Data
GBD Estimate
WUENIC
Country−reported
DHS
MICS
Other microdata
Other tabulation
Imputed

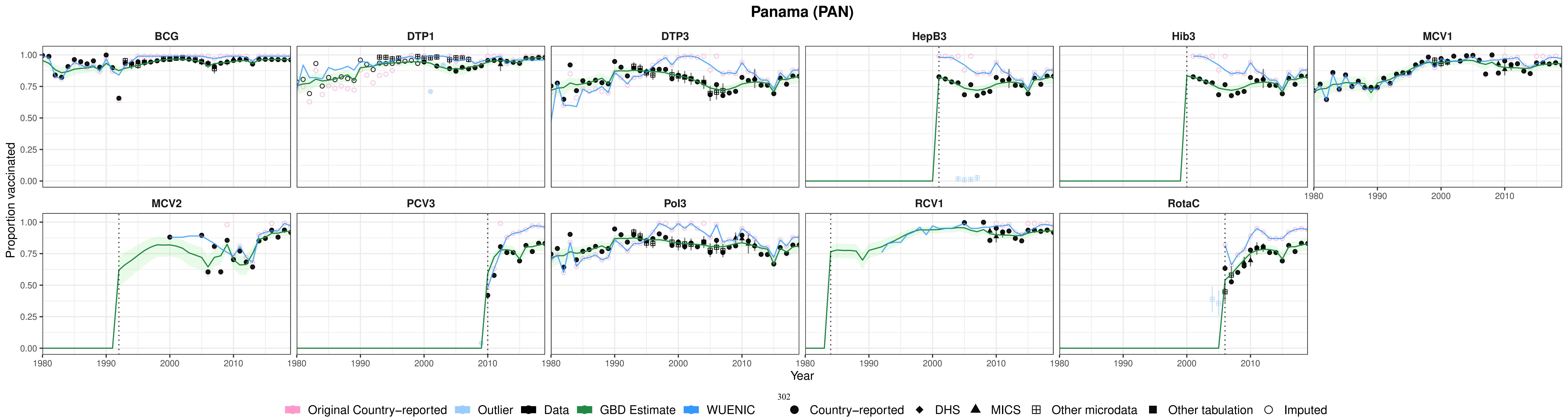

Panama (PAN)
BCG
DTP1
DTP3
HepB3
Hib3
MCV1
MCV2
PCV3
Pol3
RCV1
RotaC
Proportion vaccinated
Year
Original Country−reported
Outlier
Data
GBD Estimate
WUENIC
Country−reported
DHS
MICS
Other microdata
Other tabulation
Imputed

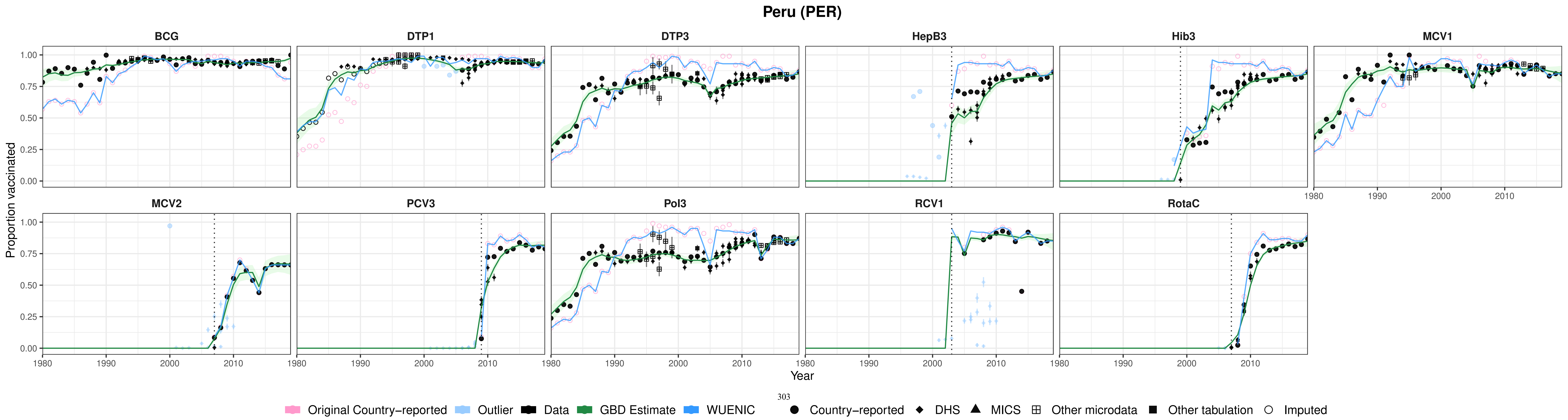
Peru (PER)
BCG
DTP1
DTP3
HepB3
Hib3
MCV1
MCV2
PCV3
Pol3
RCV1
RotaC
Proportion vaccinated
Year
Original Country−reported
Outlier
Data
GBD Estimate
WUENIC
Country−reported
DHS
MICS
Other microdata
Other tabulation
Imputed

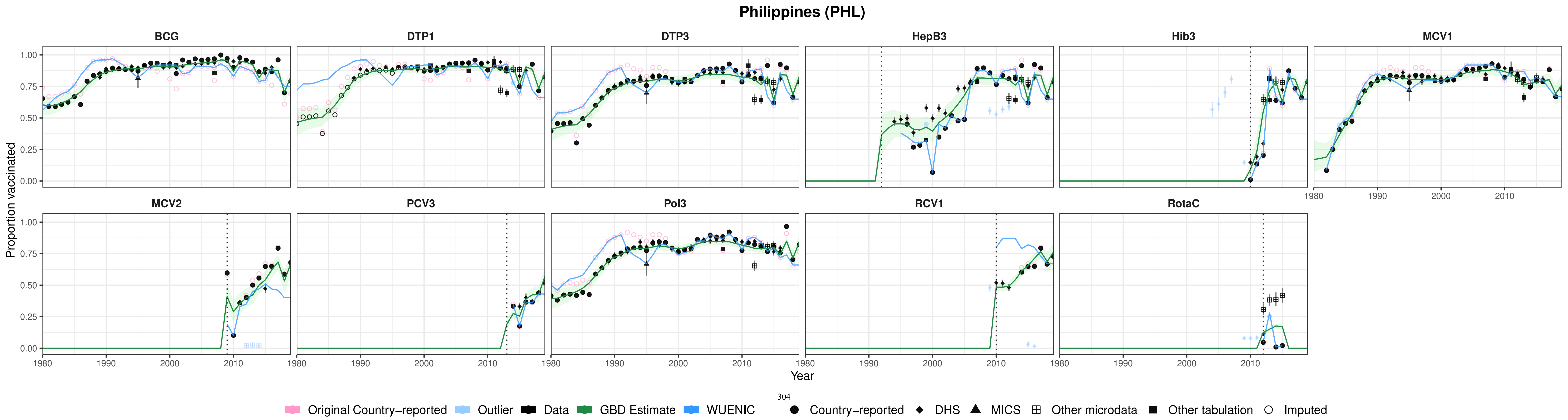
Philippines (PHL)
BCG
DTP1
DTP3
HepB3
Hib3
MCV1
MCV2
PCV3
Pol3
RCV1
RotaC
Proportion vaccinated
Year
Original Country−reported
Outlier
Data
GBD Estimate
WUENIC
Country−reported
DHS
MICS
Other microdata
Other tabulation
Imputed

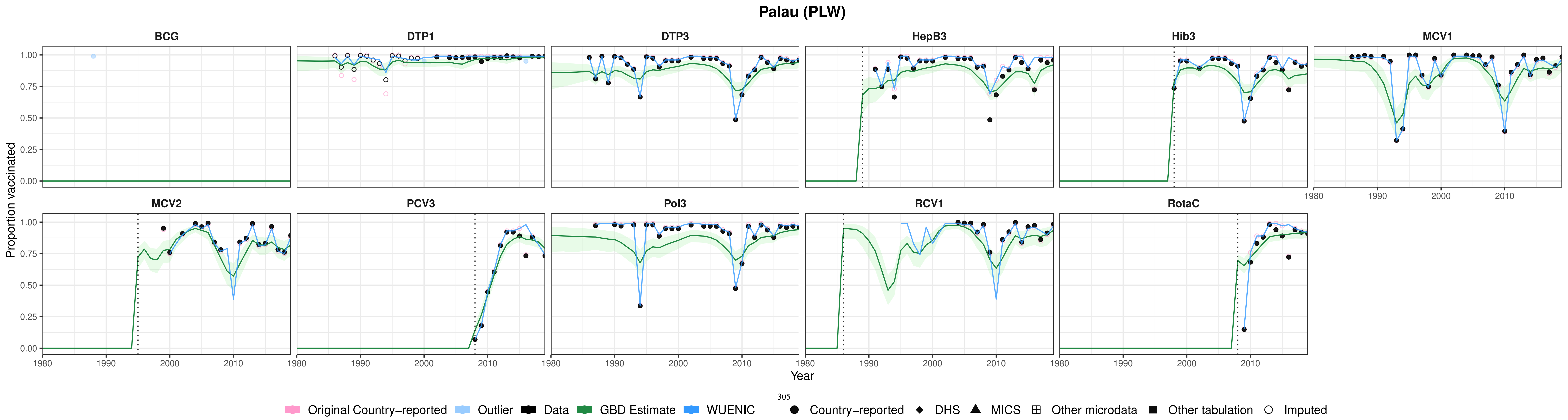

Palau (PLW)
BCG
DTP1
DTP3
HepB3
Hib3
MCV1
MCV2
PCV3
Pol3
RCV1
RotaC
Proportion vaccinated
Year
0.00
0.25
0.50
0.75
1.00
1980
1990
2000
2010
Original Country−reported
Outlier
Data
GBD Estimate
WUENIC
Country−reported
DHS
MICS
Other microdata
Other tabulation
Imputed

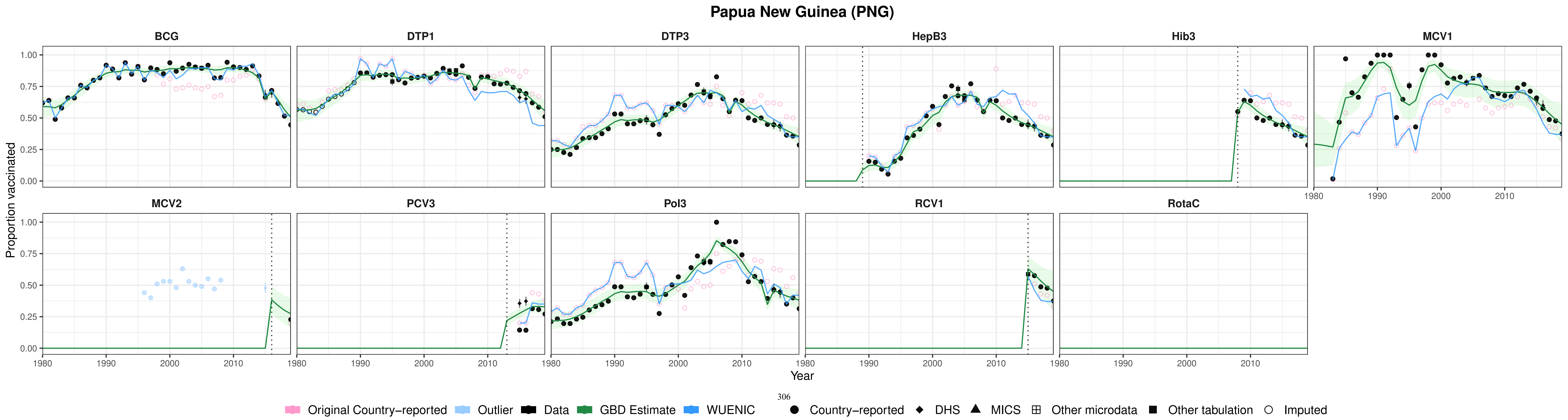

Papua New Guinea (PNG)
BCG
DTP1
DTP3
HepB3
Hib3
MCV1
MCV2
PCV3
Pol3
RCV1
RotaC
Proportion vaccinated
Year
Original Country−reported
Outlier
Data
GBD Estimate
WUENIC
Country−reported
DHS
MICS
Other microdata
Other tabulation
Imputed

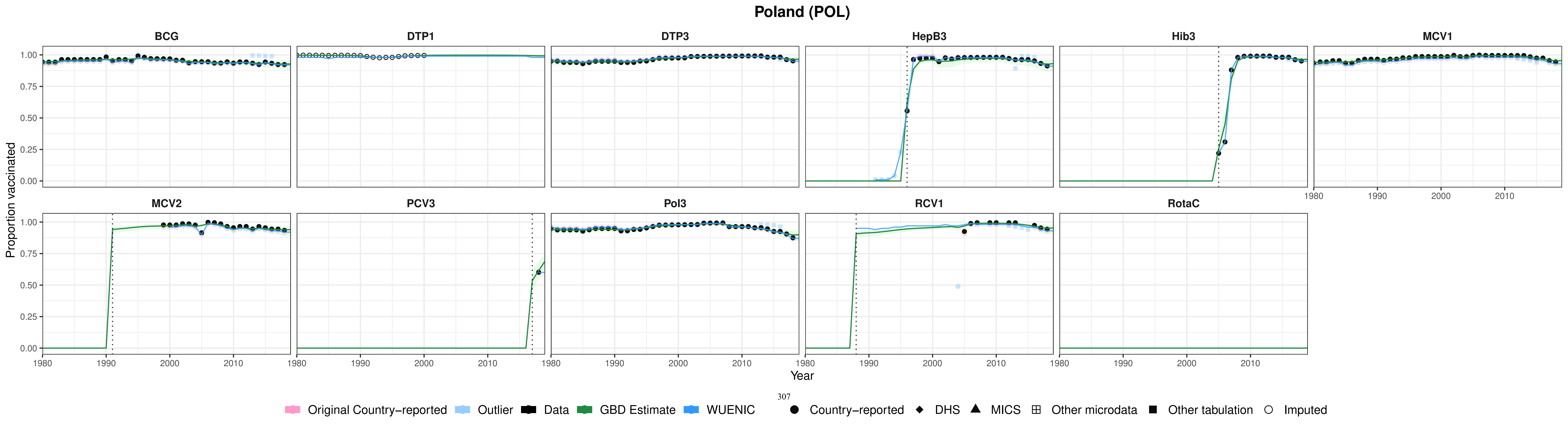
Poland (POL)
BCG
DTP1
DTP3
HepB3
Hib3
MCV1
MCV2
PCV3
Pol3
RCV1
RotaC
Proportion vaccinated
Year
Original Country−reported
Outlier
Data
GBD Estimate
WUENIC
Country−reported
DHS
MICS
Other microdata
Other tabulation
Imputed

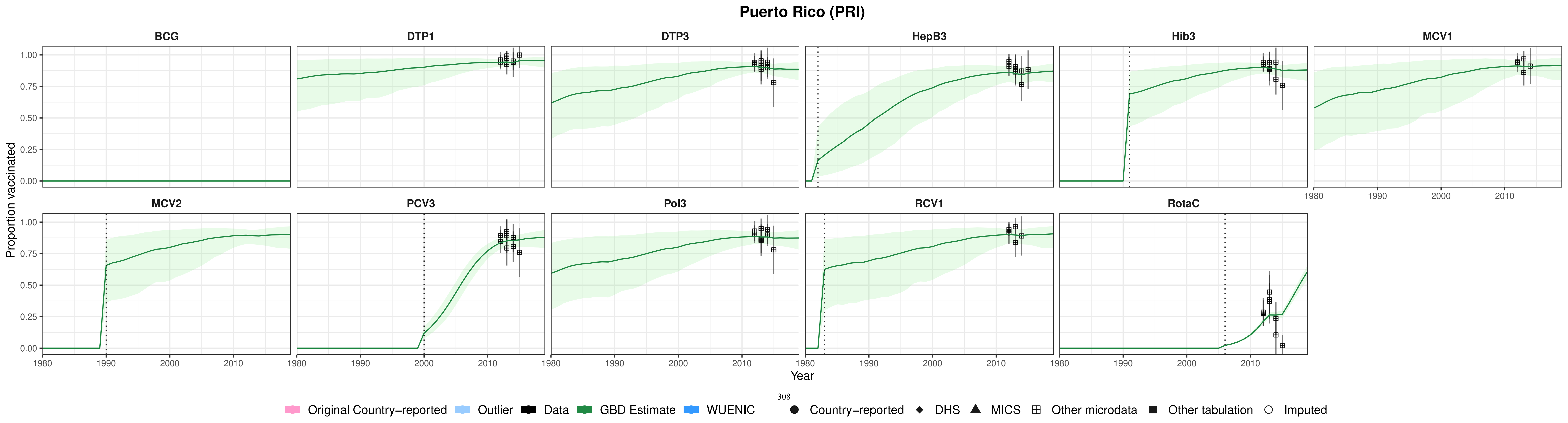
Puerto Rico (PRI)
BCG
DTP1
DTP3
HepB3
Hib3
MCV1
MCV2
PCV3
Pol3
RCV1
RotaC
Proportion vaccinated
Year
0.00
0.25
0.50
0.75
1.00
1980
1990
2000
2010
Original Country−reported
Outlier
Data
GBD Estimate
WUENIC
Country−reported
DHS
MICS
Other microdata
Other tabulation
Imputed

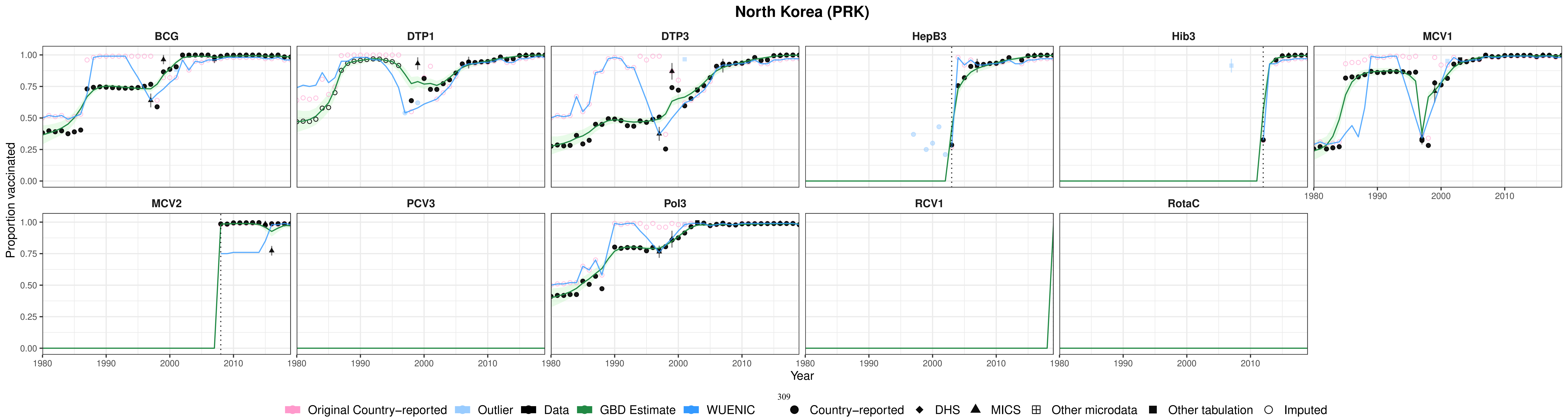
North Korea (PRK)
BCG
DTP1
DTP3
HepB3
Hib3
MCV1
MCV2
PCV3
Pol3
RCV1
RotaC
Proportion vaccinated
Year
Original Country−reported
Outlier
Data
GBD Estimate
WUENIC
Country−reported
DHS
MICS
Other microdata
Other tabulation
Imputed

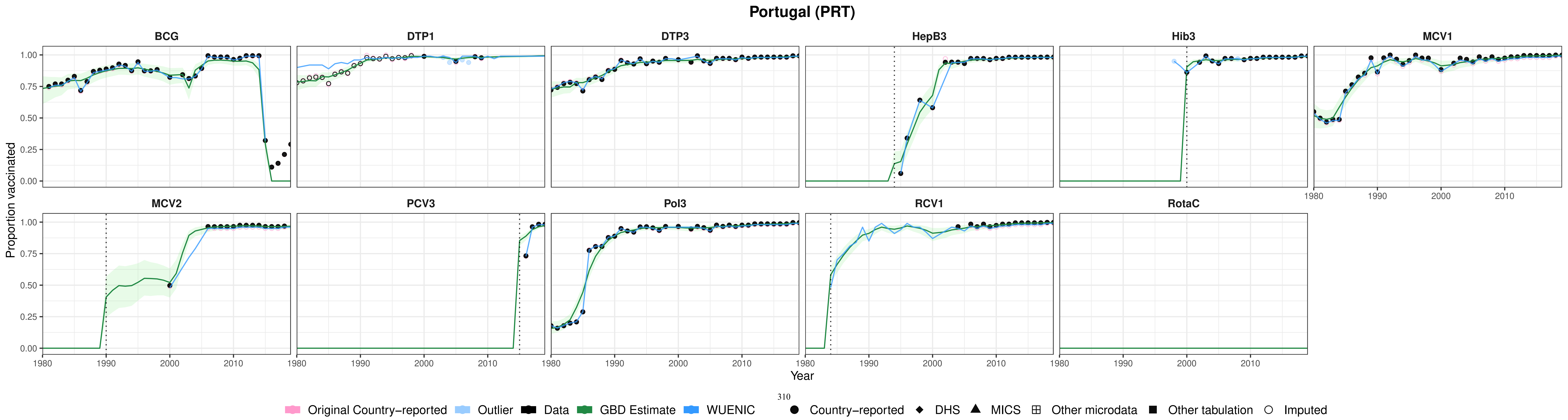

Portugal (PRT)
BCG
DTP1
DTP3
HepB3
Hib3
MCV1
MCV2
PCV3
Pol3
RCV1
RotaC
Proportion vaccinated
Year
Original Country−reported
Outlier
Data
GBD Estimate
WUENIC
Country−reported
DHS
MICS
Other microdata
Other tabulation
Imputed

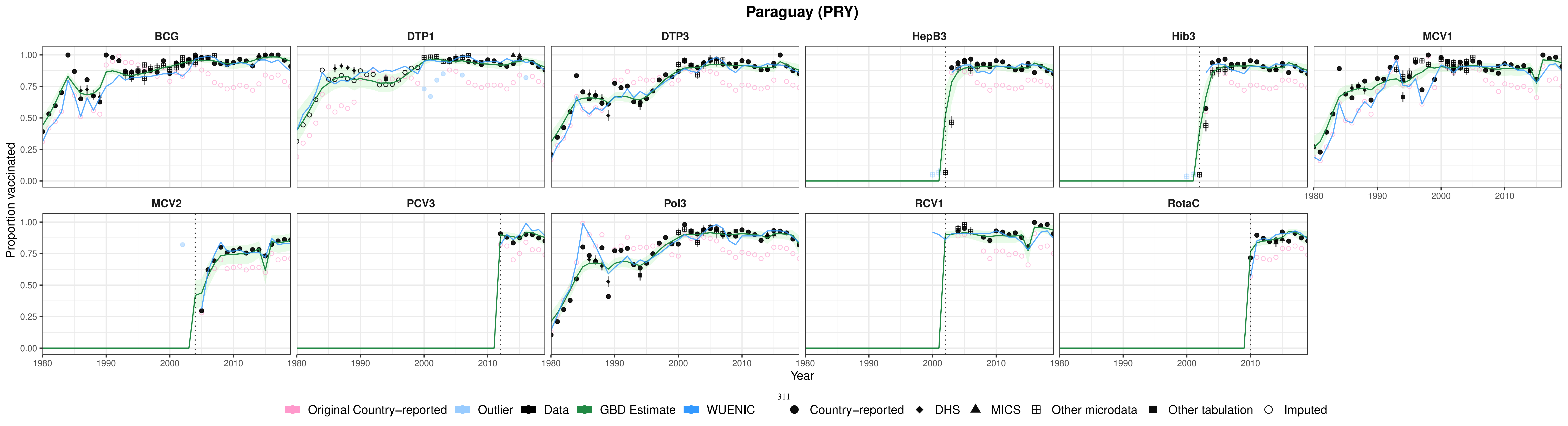

Paraguay (PRY)
BCG
DTP1
DTP3
HepB3
Hib3
MCV1
MCV2
PCV3
Pol3
RCV1
RotaC
Proportion vaccinated
Year
Original Country−reported
Outlier
Data
GBD Estimate
WUENIC
Country−reported
DHS
MICS
Other microdata
Other tabulation
Imputed

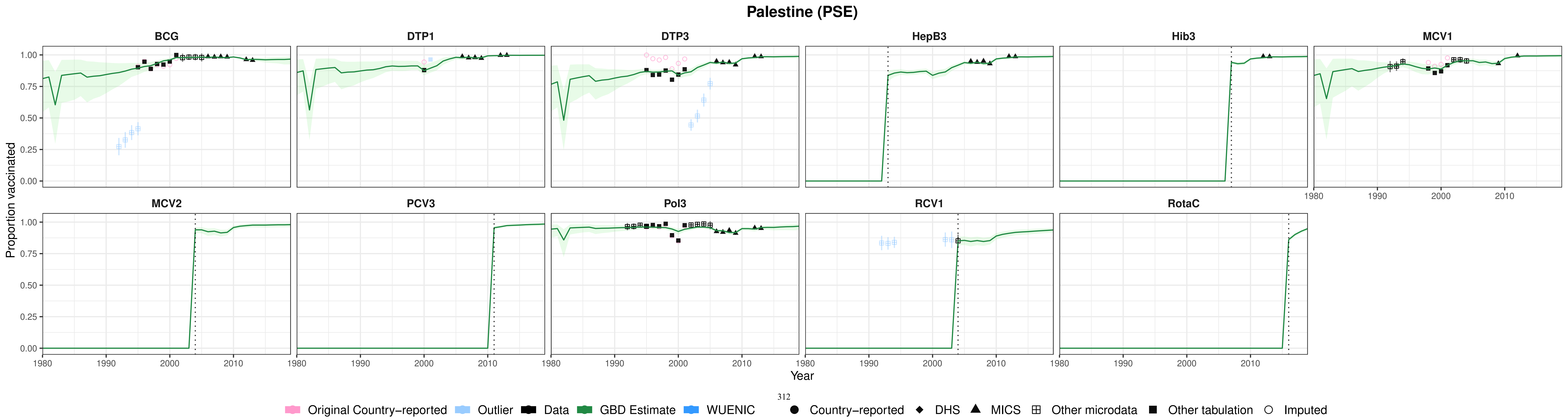

Palestine (PSE)
BCG
DTP1
DTP3
HepB3
Hib3
MCV1
MCV2
PCV3
Pol3
RCV1
RotaC
Proportion vaccinated
Year
Original Country−reported
Outlier
Data
GBD Estimate
WUENIC
Country−reported
DHS
MICS
Other microdata
Other tabulation
Imputed

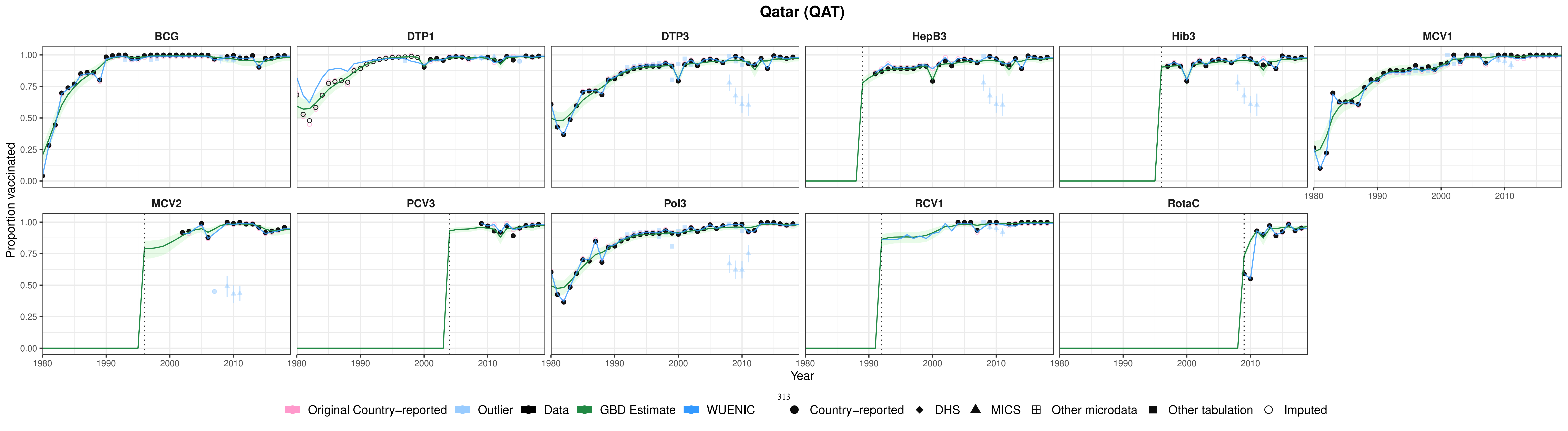
Qatar (QAT)
BCG
DTP1
DTP3
HepB3
Hib3
MCV1
MCV2
PCV3
Pol3
RCV1
RotaC
Proportion vaccinated
Year
Original Country−reported
Outlier
Data
GBD Estimate
WUENIC
Country−reported
DHS
MICS
Other microdata
Other tabulation
Imputed

Romania (ROU)

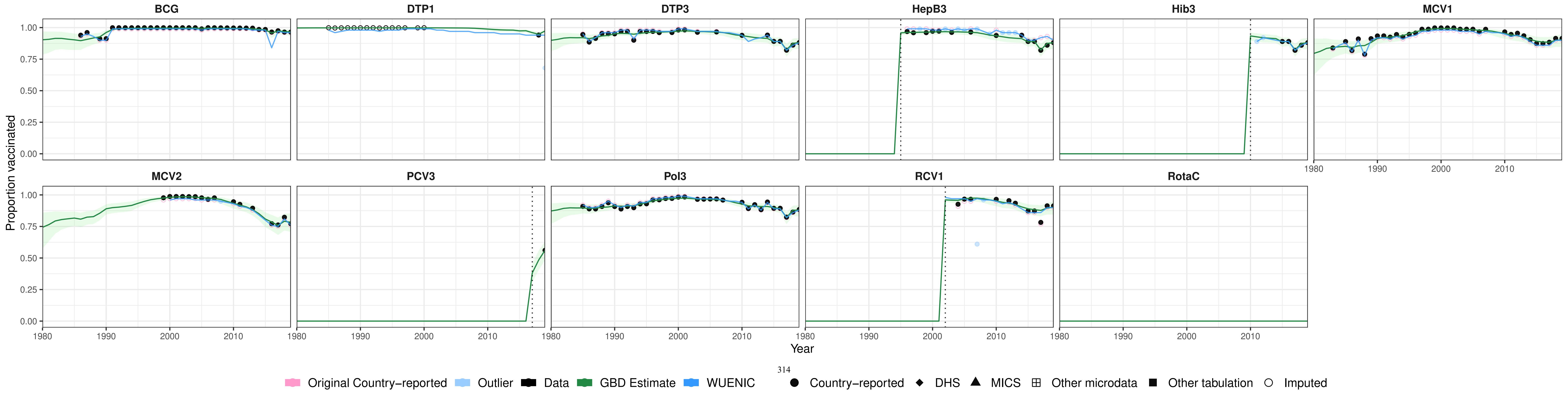

BCG
DTP1
DTP3
HepB3
Hib3
MCV1
MCV2
PCV3
Pol3
RCV1
RotaC
Proportion vaccinated
Year
1.00
0.75
0.50
0.25
0.00
1980
1990
2000
2010
Original Country−reported
Outlier
Data
GBD Estimate
WUENIC
Country−reported
DHS
MICS
Other microdata
Other tabulation
Imputed

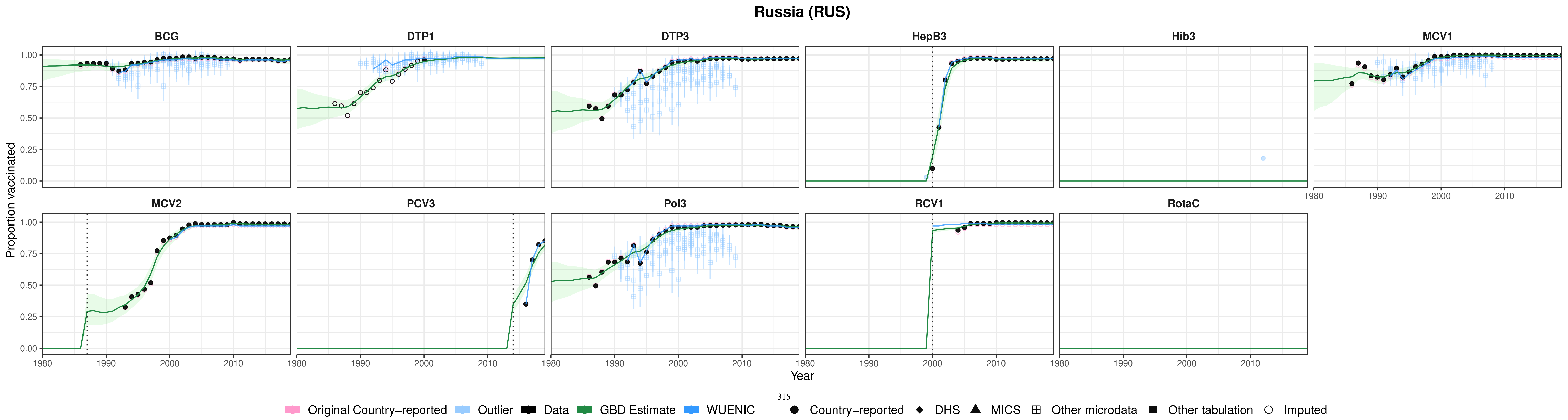

Russia (RUS)
BCG
DTP1
DTP3
HepB3
Hib3
MCV1
MCV2
PCV3
Pol3
RCV1
RotaC
Proportion vaccinated
Year
0.00
0.25
0.50
0.75
1.00
1980
1990
2000
2010
Original Country−reported
Outlier
Data
GBD Estimate
WUENIC
Country−reported
DHS
MICS
Other microdata
Other tabulation
Imputed

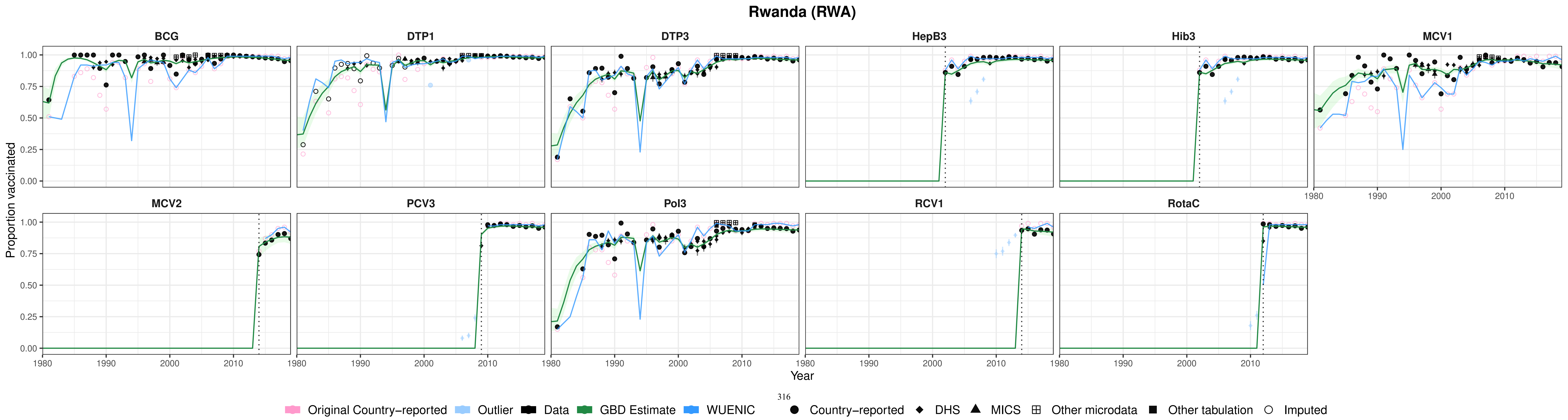
Rwanda (RWA)
BCG
DTP1
DTP3
HepB3
Hib3
MCV1
MCV2
PCV3
Pol3
RCV1
RotaC
Proportion vaccinated
Year
Original Country−reported
Outlier
Data
GBD Estimate
WUENIC
Country−reported
DHS
MICS
Other microdata
Other tabulation
Imputed

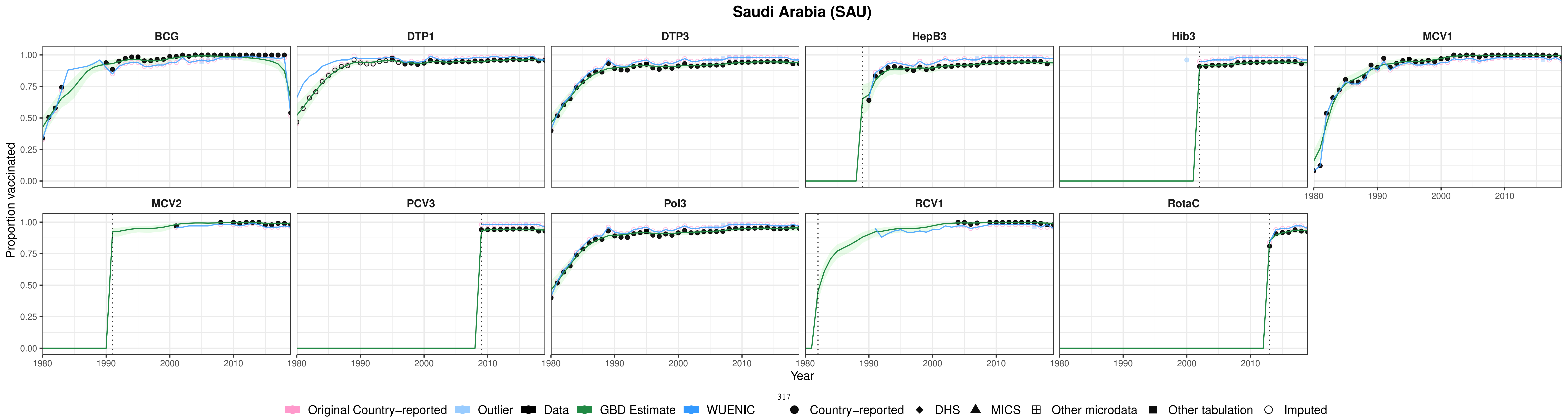

Saudi Arabia (SAU)
BCG
DTP1
DTP3
HepB3
Hib3
MCV1
MCV2
PCV3
Pol3
RCV1
RotaC
Proportion vaccinated
Year
Original Country−reported
Outlier
Data
GBD Estimate
WUENIC
Country−reported
DHS
MICS
Other microdata
Other tabulation
Imputed

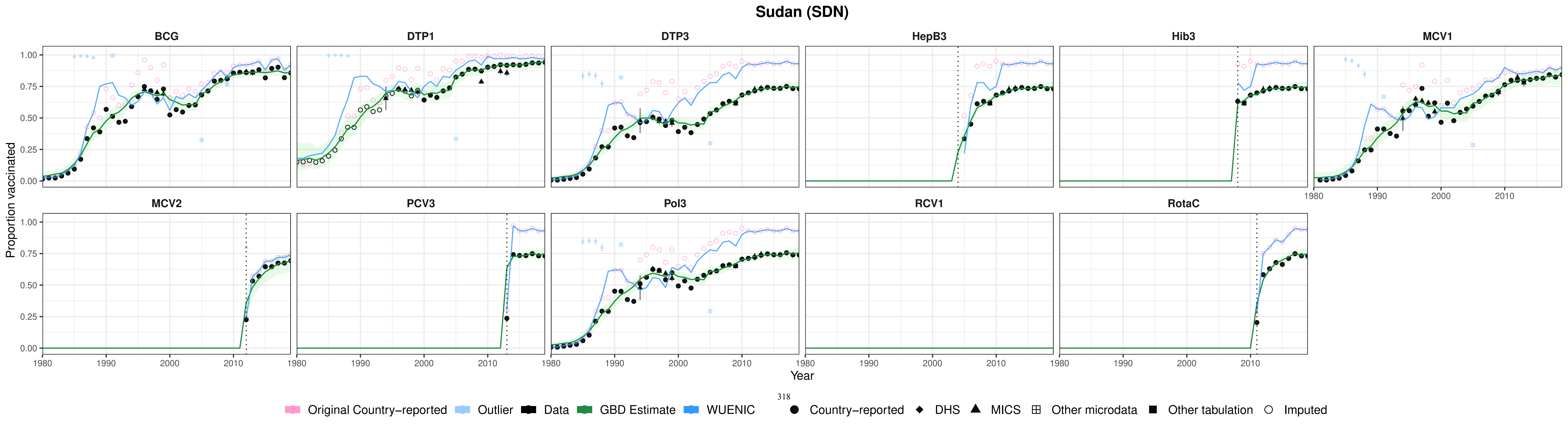
Sudan (SDN)
BCG
DTP1
DTP3
HepB3
Hib3
MCV1
MCV2
PCV3
Pol3
RCV1
RotaC
Proportion vaccinated
Year
0.00
0.25
0.50
0.75
1.00
1980
1990
2000
2010
Original Country−reported
Outlier
Data
GBD Estimate
WUENIC
Country−reported
DHS
MICS
Other microdata
Other tabulation
Imputed

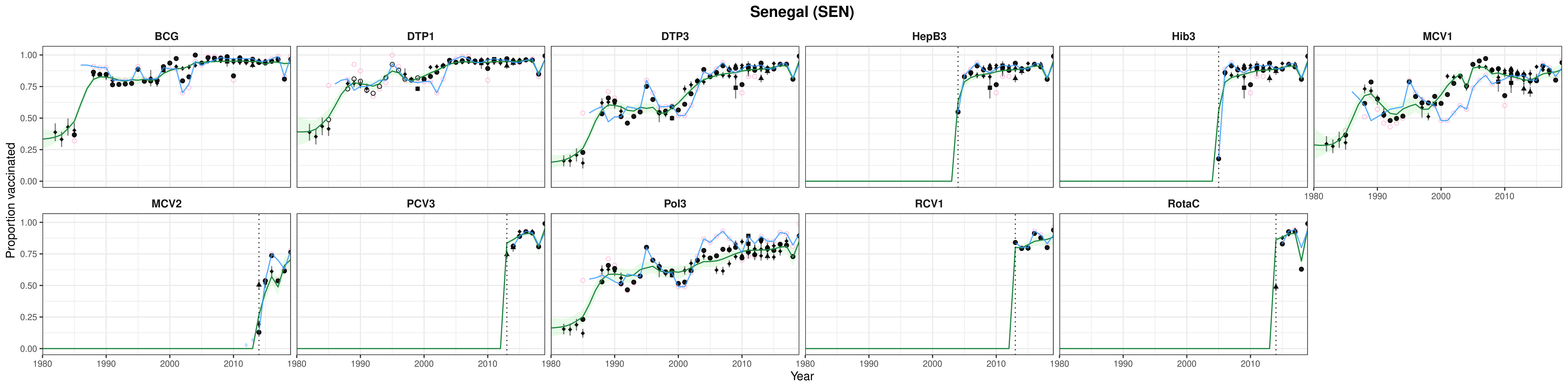
Senegal (SEN)
BCG
DTP1
DTP3
HepB3
Hib3
MCV1
MCV2
PCV3
Pol3
RCV1
RotaC
Proportion vaccinated
Year
Original Country−reported
Outlier
Data
GBD Estimate
WUENIC
Country−reported
DHS
MICS
Other microdata
Other tabulation
Imputed

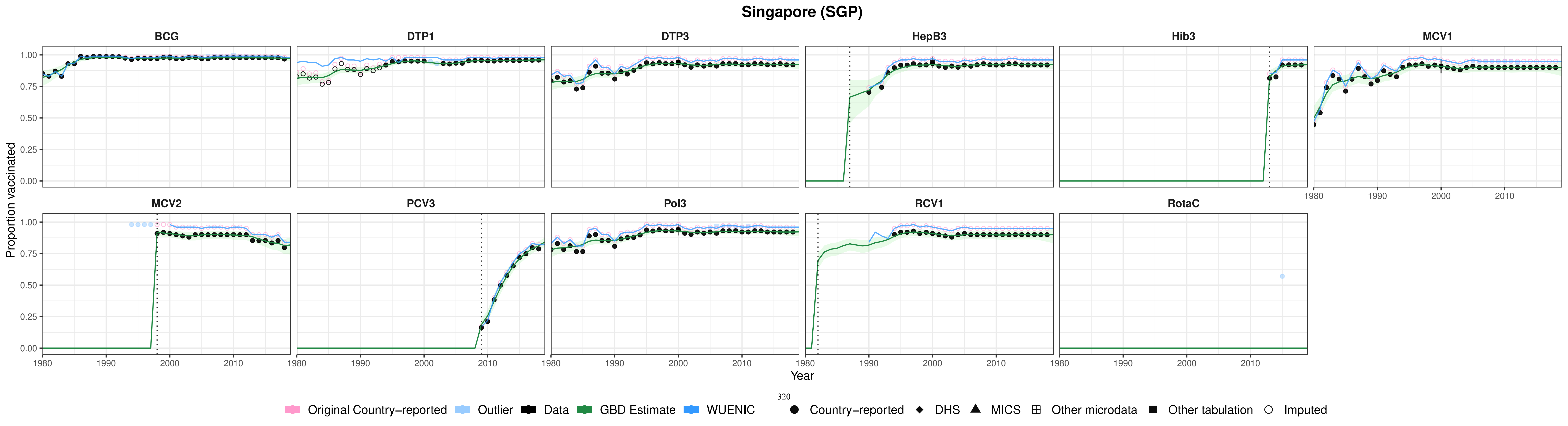

Singapore (SGP)
BCG
DTP1
DTP3
HepB3
Hib3
MCV1
MCV2
PCV3
Pol3
RCV1
RotaC
Proportion vaccinated
Year
Original Country−reported
Outlier
Data
GBD Estimate
WUENIC
Country−reported
DHS
MICS
Other microdata
Other tabulation
Imputed

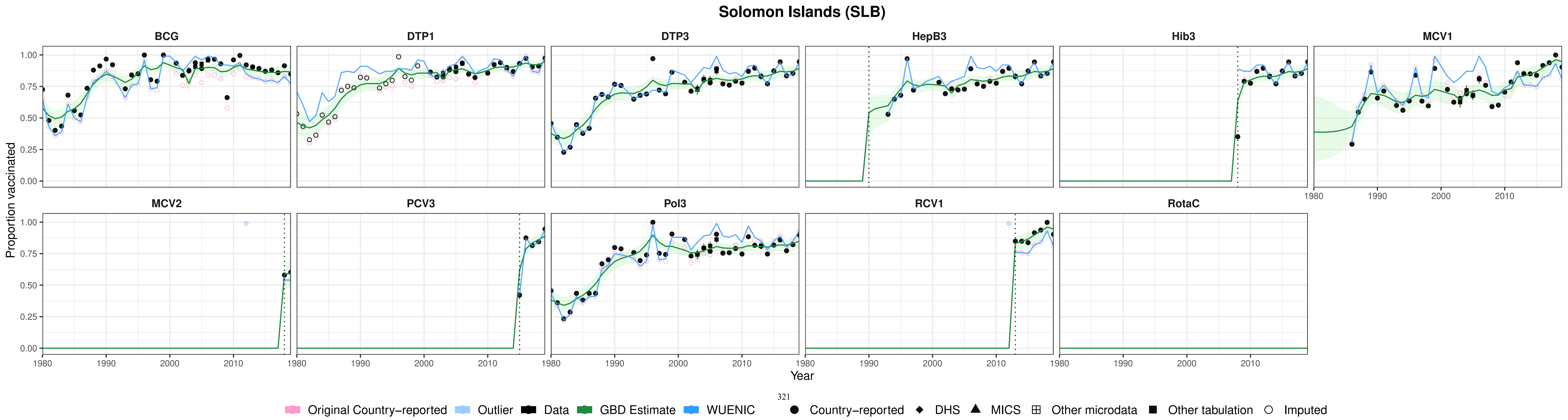

Solomon Islands (SLB)
BCG
DTP1
DTP3
HepB3
Hib3
MCV1
MCV2
PCV3
Pol3
RCV1
RotaC
Proportion vaccinated
Year
0.00
0.25
0.50
0.75
1.00
1980
1990
2000
2010
Original Country−reported
Outlier
Data
GBD Estimate
WUENIC
Country−reported
DHS
MICS
Other microdata
Other tabulation
Imputed

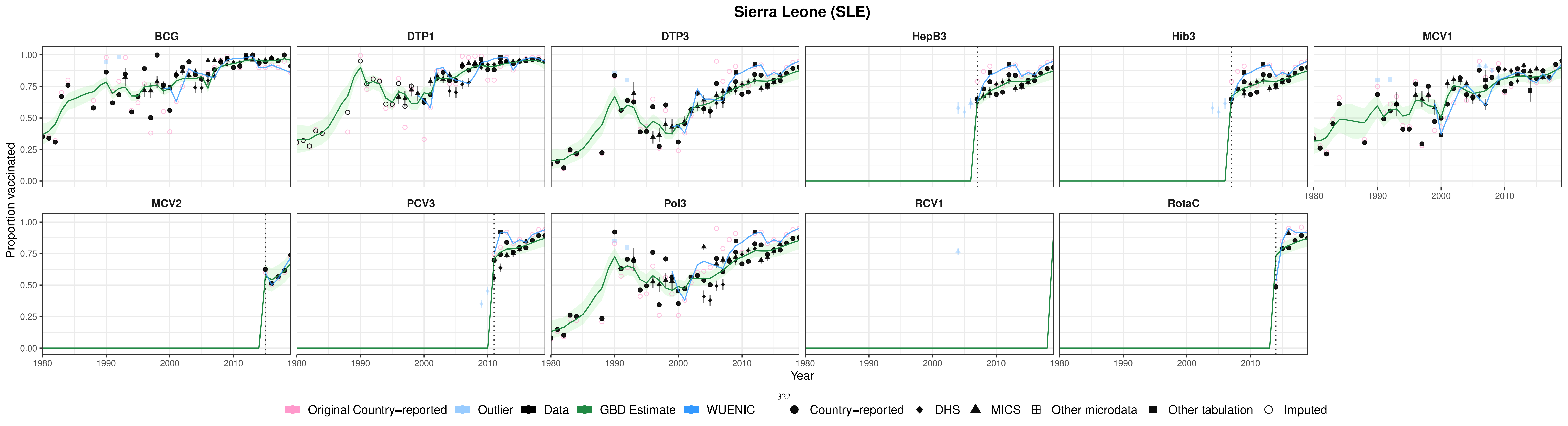
Sierra Leone (SLE)
BCG
DTP1
DTP3
HepB3
Hib3
MCV1
MCV2
PCV3
Pol3
RCV1
RotaC
Proportion vaccinated
0.00
0.25
0.50
0.75
1.00
1980
1990
2000
2010
Year
Original Country−reported
Outlier
Data
GBD Estimate
WUENIC
Country−reported
DHS
MICS
Other microdata
Other tabulation
Imputed

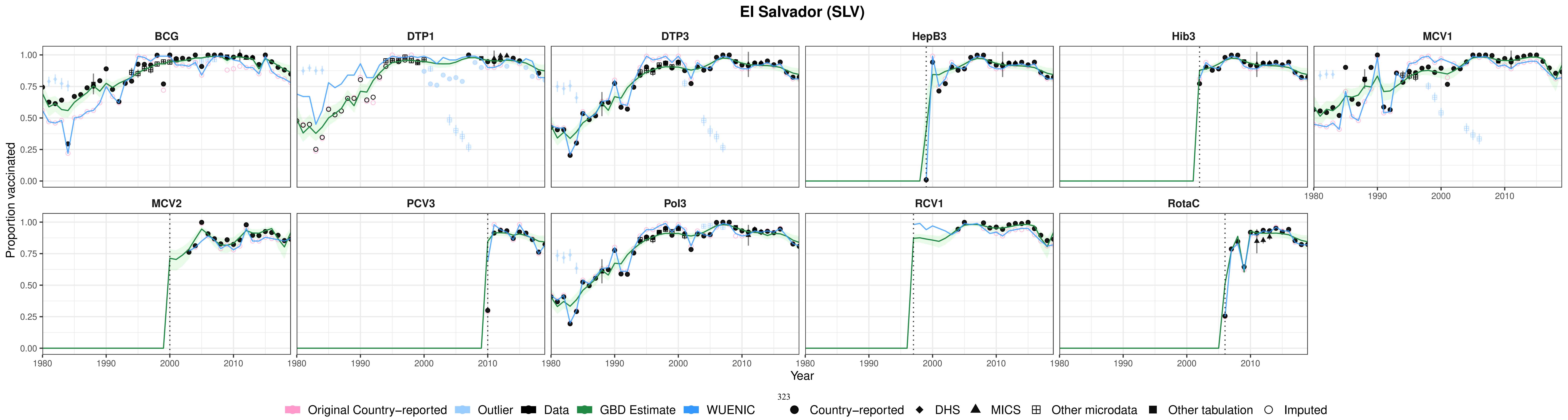
El Salvador (SLV)
BCG
DTP1
DTP3
HepB3
Hib3
MCV1
MCV2
PCV3
Pol3
RCV1
RotaC
Proportion vaccinated
Year
Original Country−reported
Outlier
Data
GBD Estimate
WUENIC
Country−reported
DHS
MICS
Other microdata
Other tabulation
Imputed

San Marino (SMR)

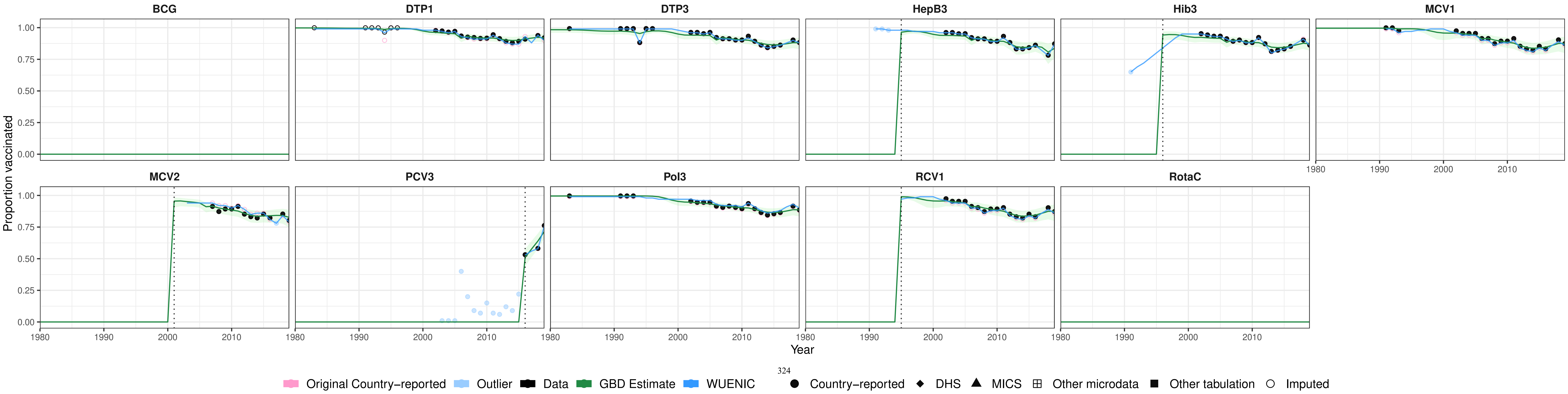

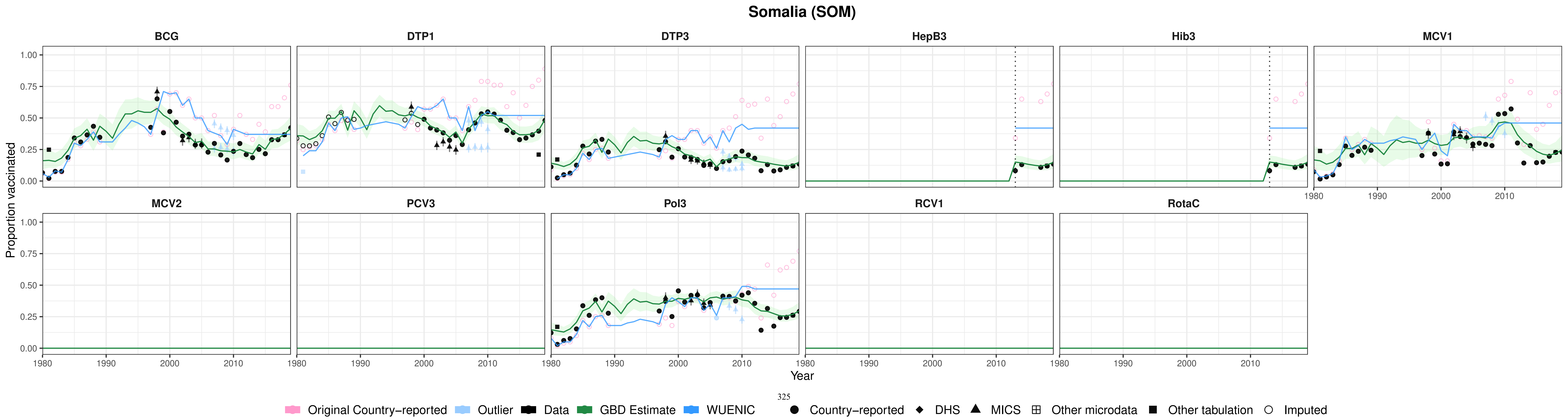

Somalia (SOM)
BCG
DTP1
DTP3
HepB3
Hib3
MCV1
MCV2
PCV3
Pol3
RCV1
RotaC
Proportion vaccinated
0.00
0.25
0.50
0.75
1.00
1980
1990
2000
2010
Year
Original Country−reported
Outlier
Data
GBD Estimate
WUENIC
Country−reported
DHS
MICS
Other microdata
Other tabulation
Imputed

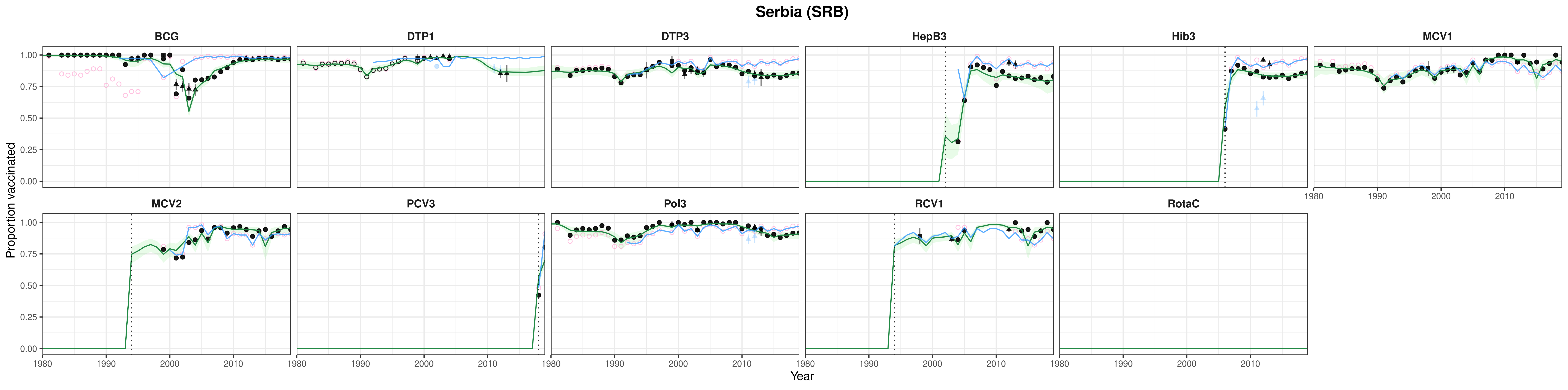

Serbia (SRB)
BCG
DTP1
DTP3
HepB3
Hib3
MCV1
MCV2
PCV3
Pol3
RCV1
RotaC
Proportion vaccinated
Year
Original Country−reported
Outlier
Data
GBD Estimate
WUENIC
Country−reported
DHS
MICS
Other microdata
Other tabulation
Imputed

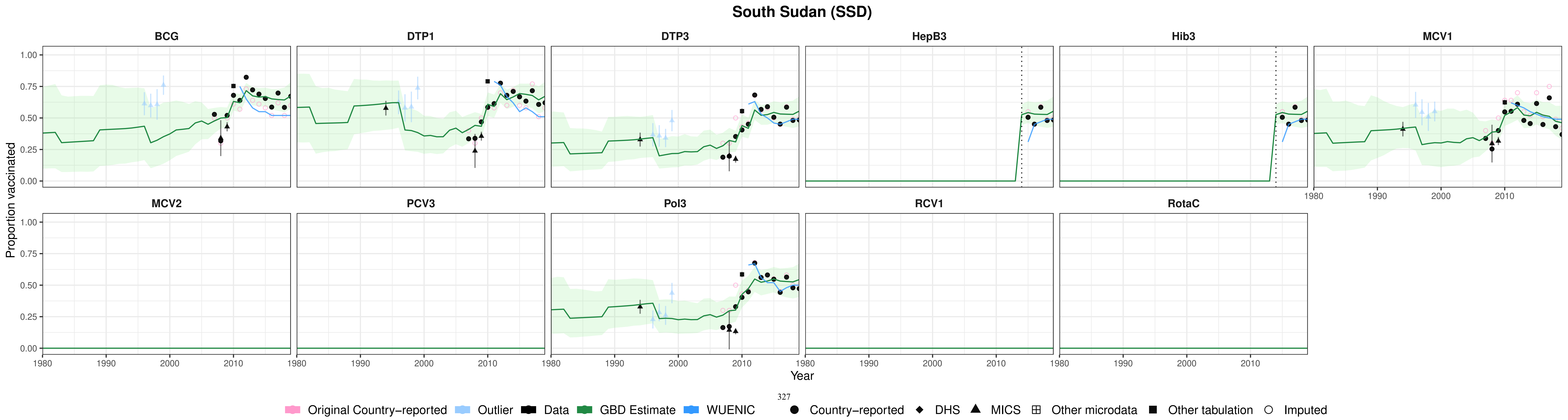
South Sudan (SSD)
BCG
DTP1
DTP3
HepB3
Hib3
MCV1
MCV2
PCV3
Pol3
RCV1
RotaC
Proportion vaccinated
Year
Original Country−reported
Outlier
Data
GBD Estimate
WUENIC
Country−reported
DHS
MICS
Other microdata
Other tabulation
Imputed

São Tomé and PrÍncipe (STP)

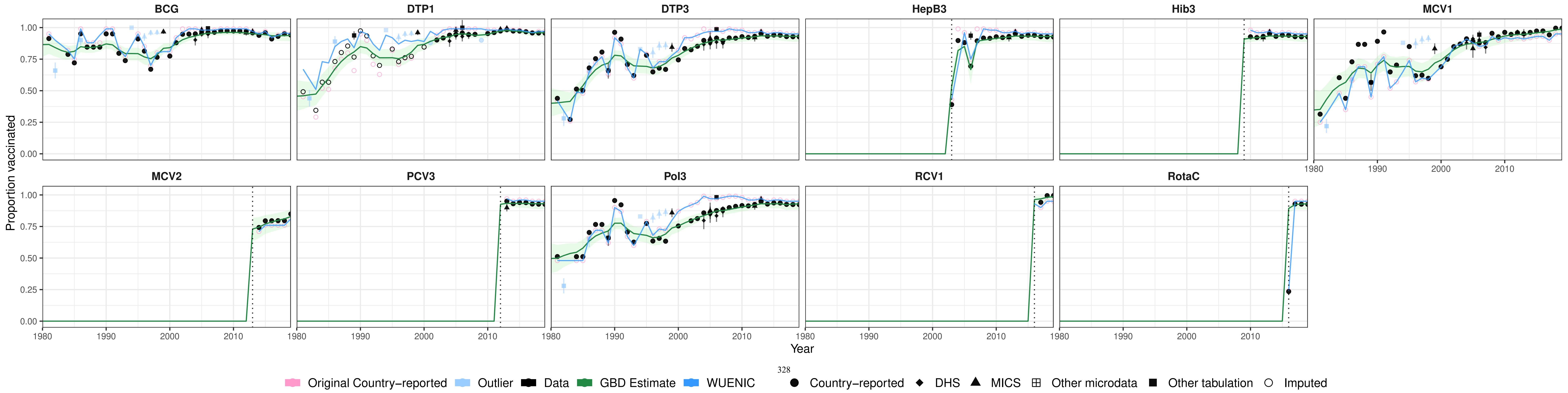

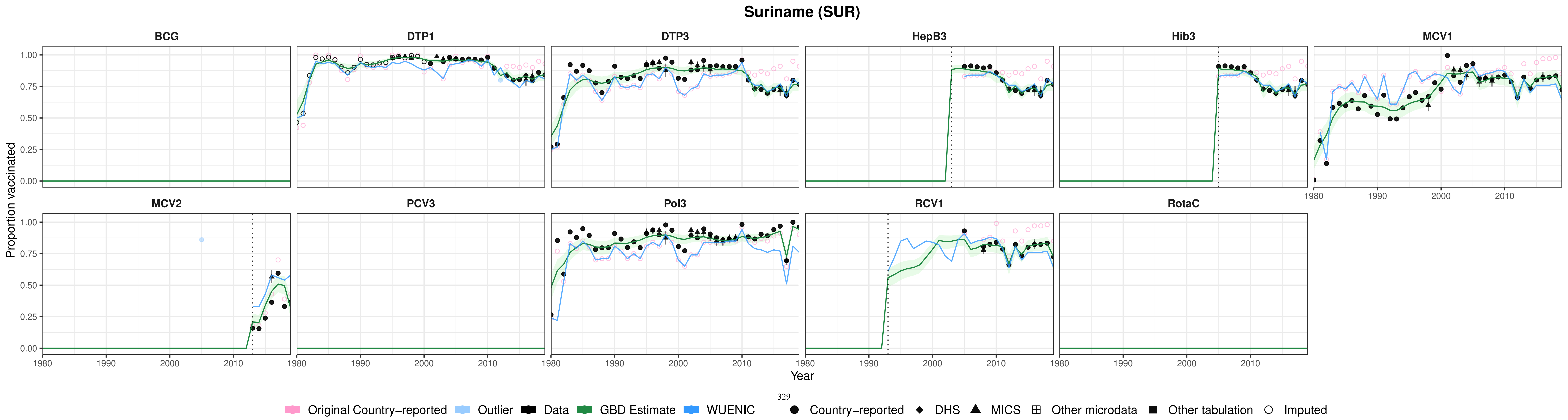

Suriname (SUR)
BCG
DTP1
DTP3
HepB3
Hib3
MCV1
MCV2
PCV3
Pol3
RCV1
RotaC
Proportion vaccinated
Year
Original Country−reported
Outlier
Data
GBD Estimate
WUENIC
Country−reported
DHS
MICS
Other microdata
Other tabulation
Imputed

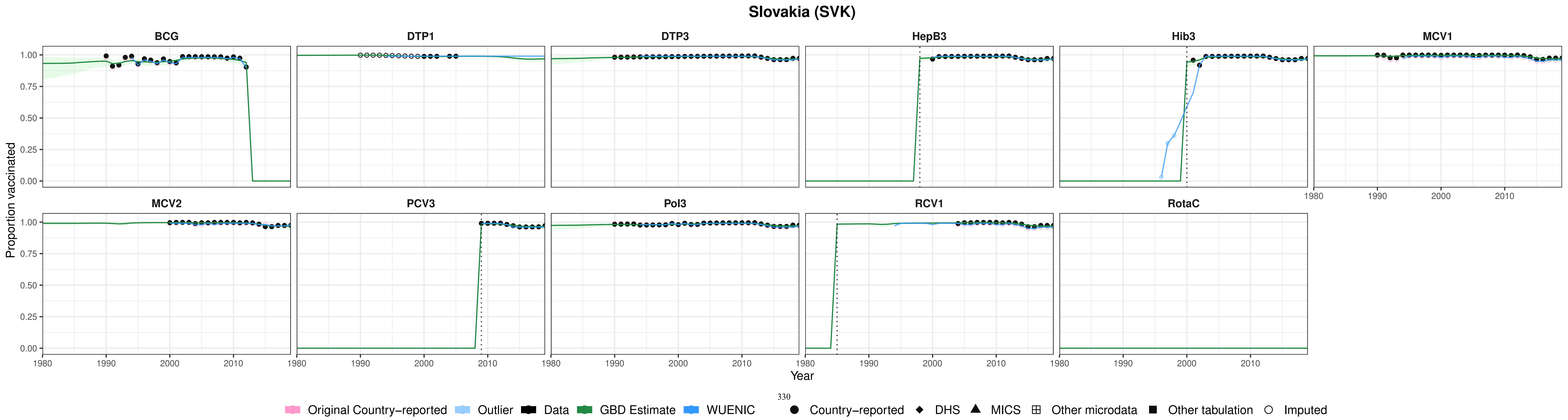
Slovakia (SVK)
BCG
DTP1
DTP3
HepB3
Hib3
MCV1
MCV2
PCV3
Pol3
RCV1
RotaC
Proportion vaccinated
0.00
0.25
0.50
0.75
1.00
1980
1990
2000
2010
Year
Original Country−reported
Outlier
Data
GBD Estimate
WUENIC
Country−reported
DHS
MICS
Other microdata
Other tabulation
Imputed

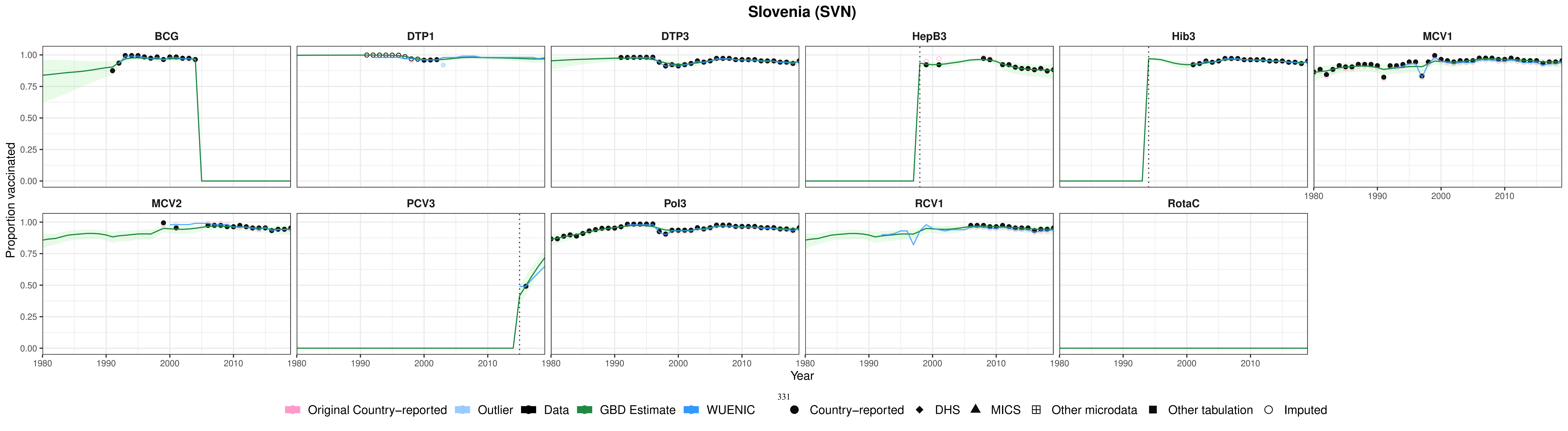

Slovenia (SVN)
BCG
DTP1
DTP3
HepB3
Hib3
MCV1
MCV2
PCV3
Pol3
RCV1
RotaC
Proportion vaccinated
Year
Original Country−reported
Outlier
Data
GBD Estimate
WUENIC
Country−reported
DHS
MICS
Other microdata
Other tabulation
Imputed

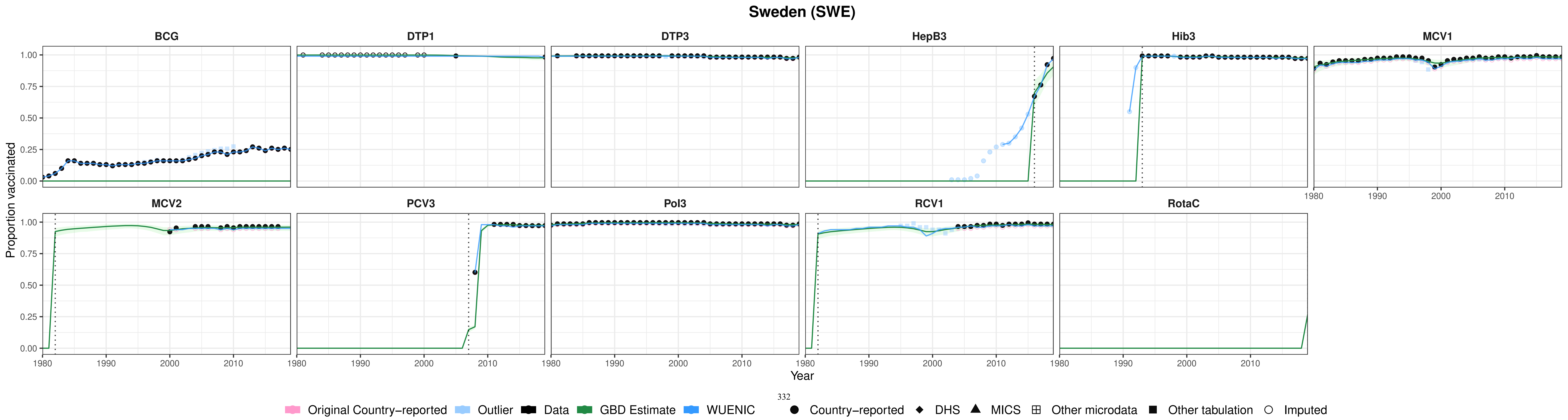
Sweden (SWE)
BCG
DTP1
DTP3
HepB3
Hib3
MCV1
MCV2
PCV3
Pol3
RCV1
RotaC
Proportion vaccinated
Year
Original Country−reported
Outlier
Data
GBD Estimate
WUENIC
Country−reported
DHS
MICS
Other microdata
Other tabulation
Imputed

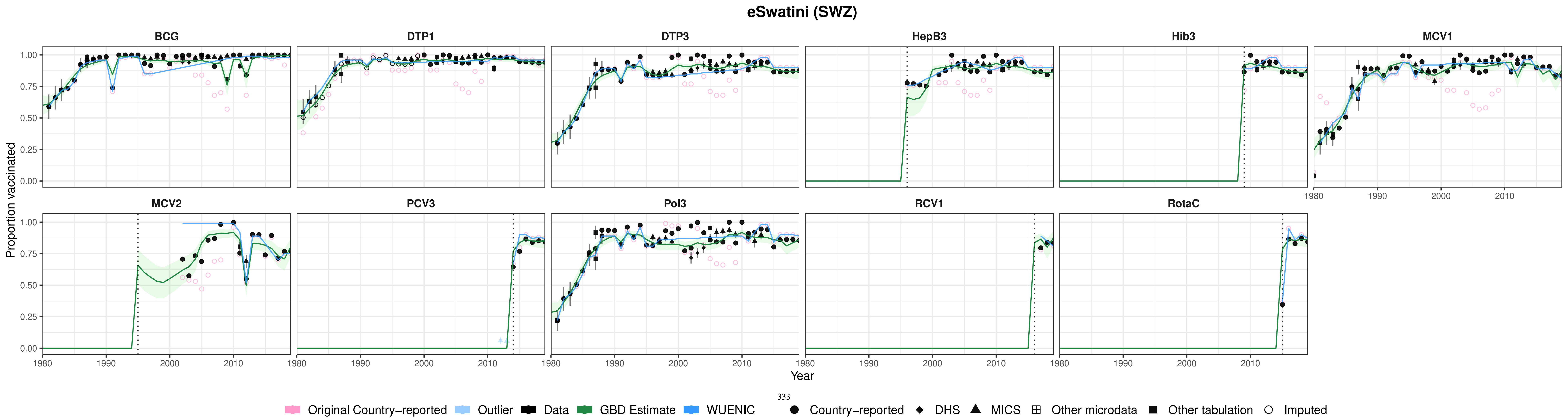

eSwatini (SWZ)
BCG
DTP1
DTP3
HepB3
Hib3
MCV1
MCV2
PCV3
Pol3
RCV1
RotaC
Proportion vaccinated
Year
Original Country−reported
Outlier
Data
GBD Estimate
WUENIC
Country−reported
DHS
MICS
Other microdata
Other tabulation
Imputed

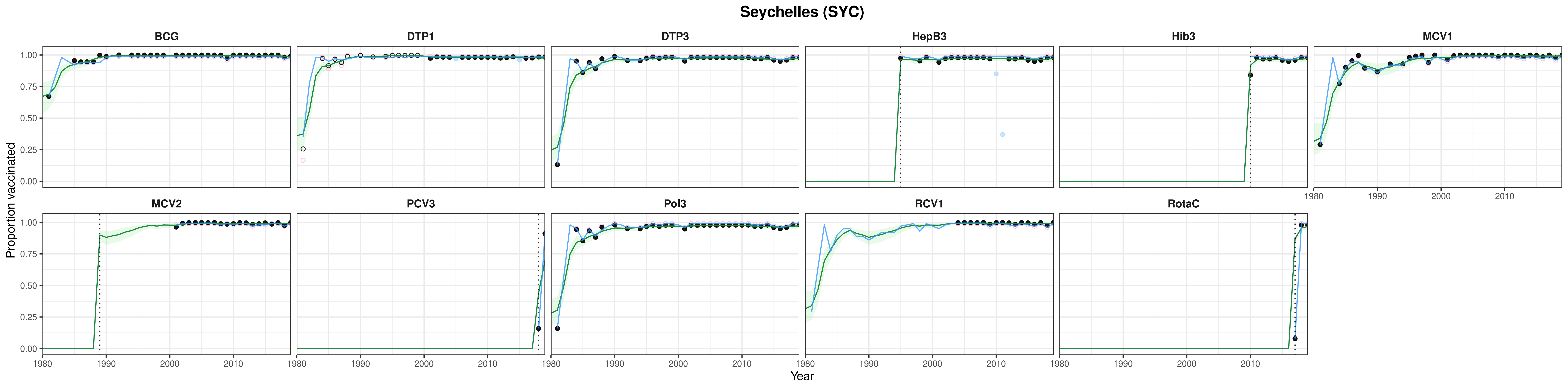
Seychelles (SYC)
BCG
DTP1
DTP3
HepB3
Hib3
MCV1
MCV2
PCV3
Pol3
RCV1
RotaC
Proportion vaccinated
Year
Original Country−reported
Outlier
Data
GBD Estimate
WUENIC
Country−reported
DHS
MICS
Other microdata
Other tabulation
Imputed

Syria (SYR)

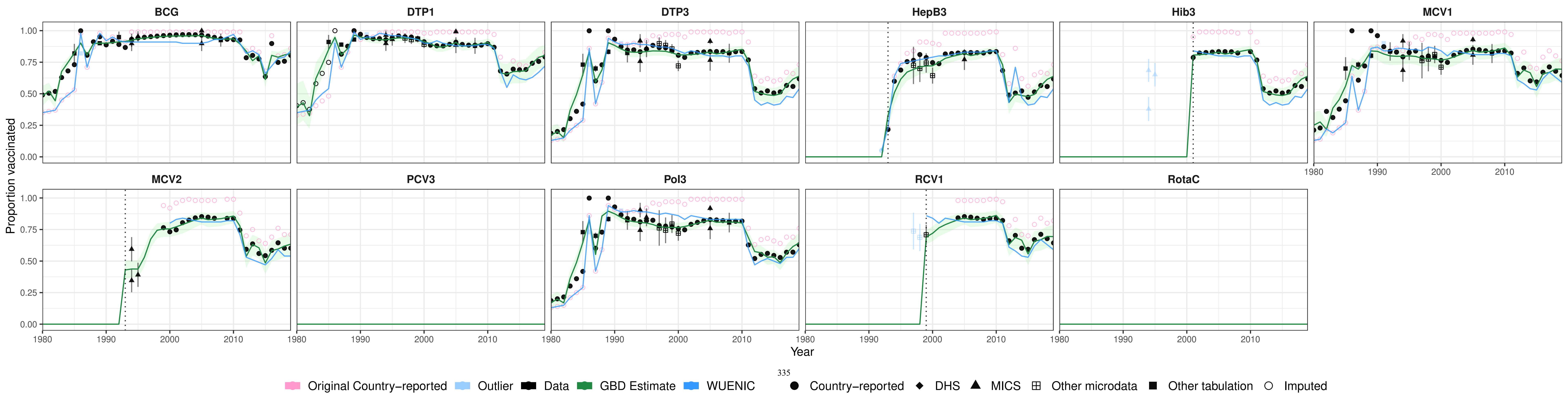

BCG
DTP1
DTP3
HepB3
Hib3
MCV1
MCV2
PCV3
Pol3
RCV1
RotaC
Proportion vaccinated
Year
1.00
0.75
0.50
0.25
0.00
1980
1990
2000
2010
Original Country−reported
Outlier
Data
GBD Estimate
WUENIC
Country−reported
DHS
MICS
Other microdata
Other tabulation
Imputed

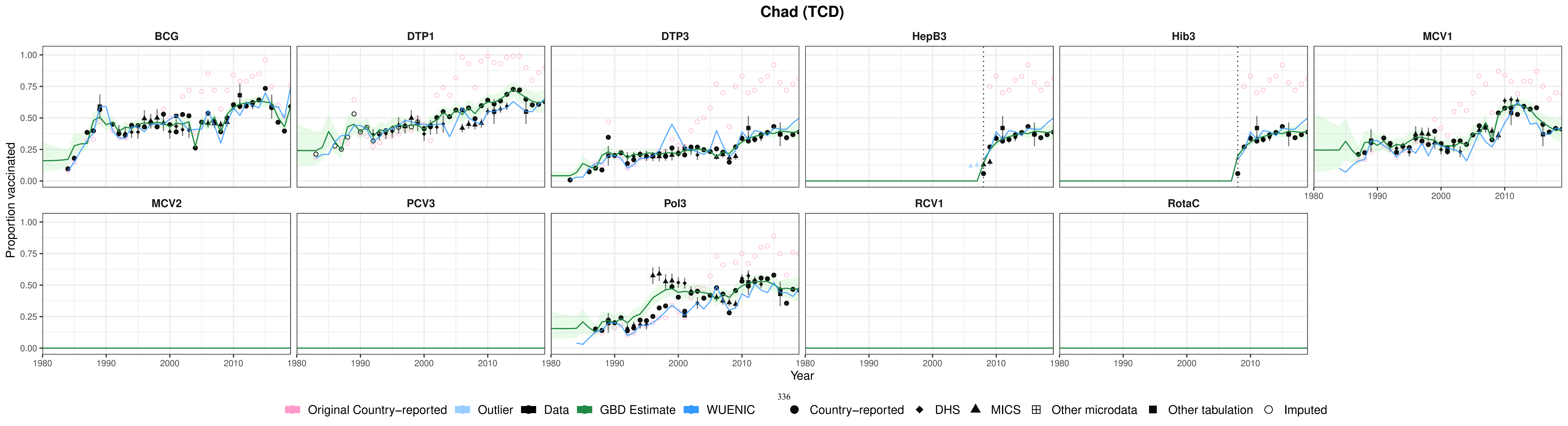
Chad (TCD)
BCG
DTP1
DTP3
HepB3
Hib3
MCV1
MCV2
PCV3
Pol3
RCV1
RotaC
Proportion vaccinated
Year
0.00
0.25
0.50
0.75
1.00
1980
1990
2000
2010
Original Country−reported
Outlier
Data
GBD Estimate
WUENIC
Country−reported
DHS
MICS
Other microdata
Other tabulation
Imputed

Togo (TGO)

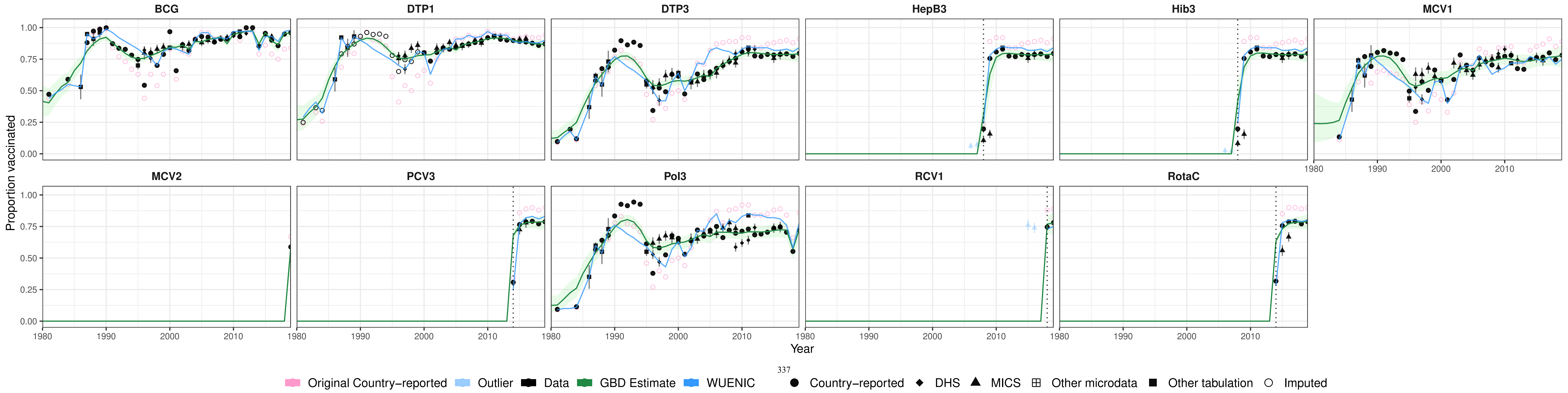

BCG
DTP1
DTP3
HepB3
Hib3
MCV1
MCV2
PCV3
Pol3
RCV1
RotaC
Proportion vaccinated
Year
Original Country−reported
Outlier
Data
GBD Estimate
WUENIC
Country−reported
DHS
MICS
Other microdata
Other tabulation
Imputed

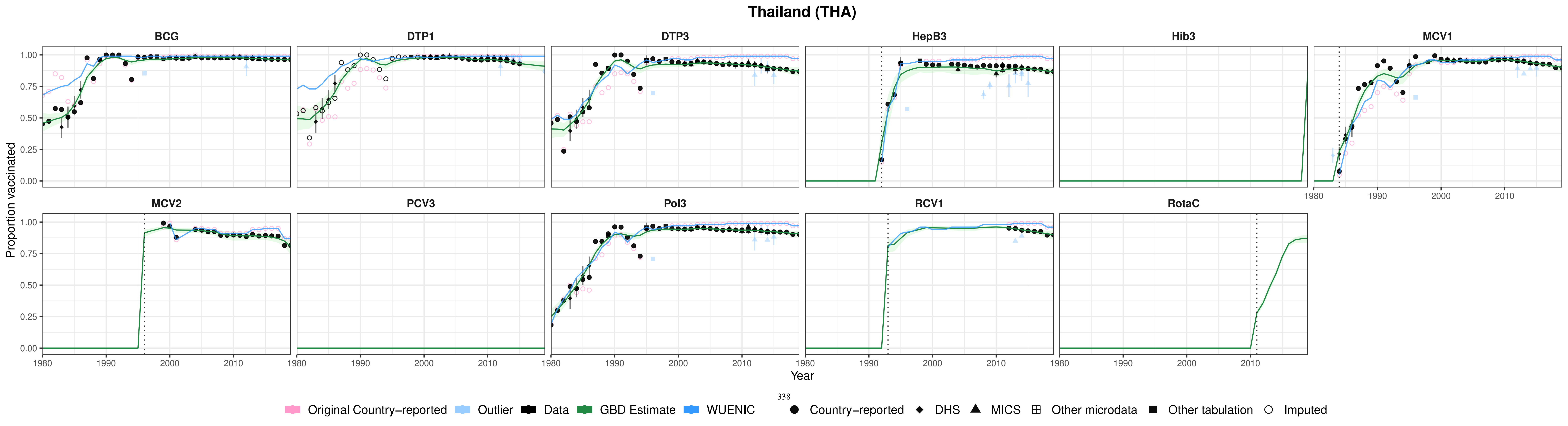
Thailand (THA)
BCG
DTP1
DTP3
HepB3
Hib3
MCV1
MCV2
PCV3
Pol3
RCV1
RotaC
Proportion vaccinated
Year
Original Country−reported
Outlier
Data
GBD Estimate
WUENIC
Country−reported
DHS
MICS
Other microdata
Other tabulation
Imputed

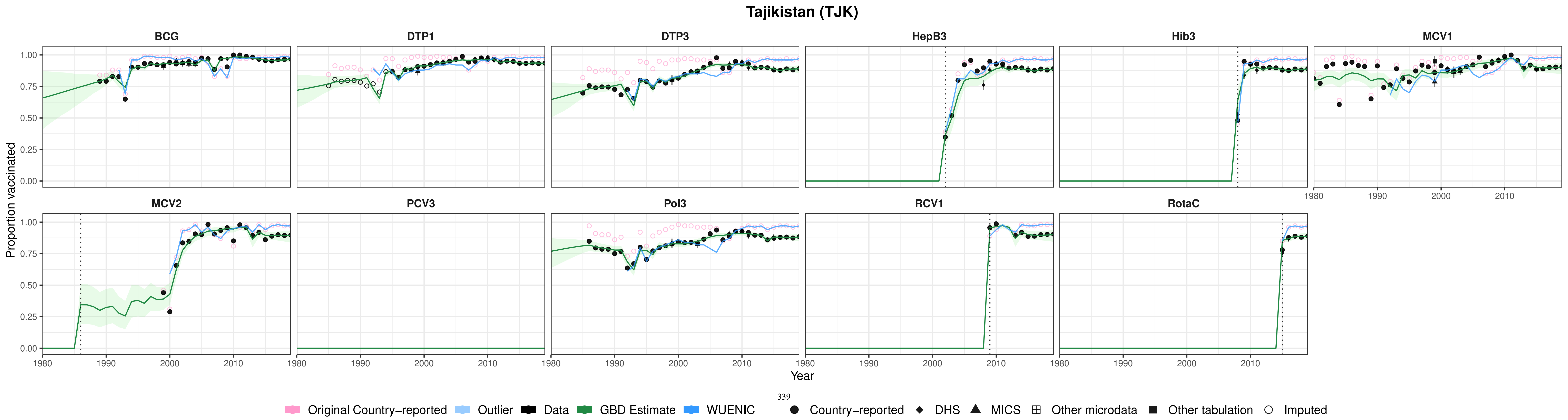
Tajikistan (TJK)
BCG
DTP1
DTP3
HepB3
Hib3
MCV1
MCV2
PCV3
Pol3
RCV1
RotaC
Proportion vaccinated
Year
Original Country−reported
Outlier
Data
GBD Estimate
WUENIC
Country−reported
DHS
MICS
Other microdata
Other tabulation
Imputed

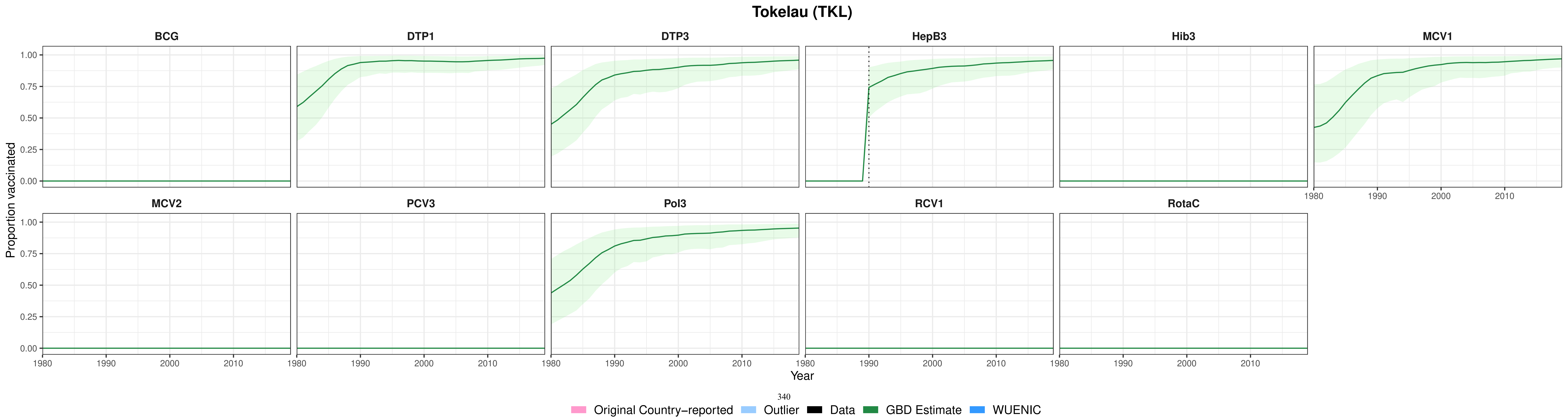
Tokelau (TKL)
BCG
DTP1
DTP3
HepB3
Hib3
MCV1
MCV2
PCV3
Pol3
RCV1
RotaC
Proportion vaccinated
1.00
0.75
0.50
0.25
0.00
1980
1990
2000
2010
Year
Original Country−reported
Outlier
Data
GBD Estimate
WUENIC

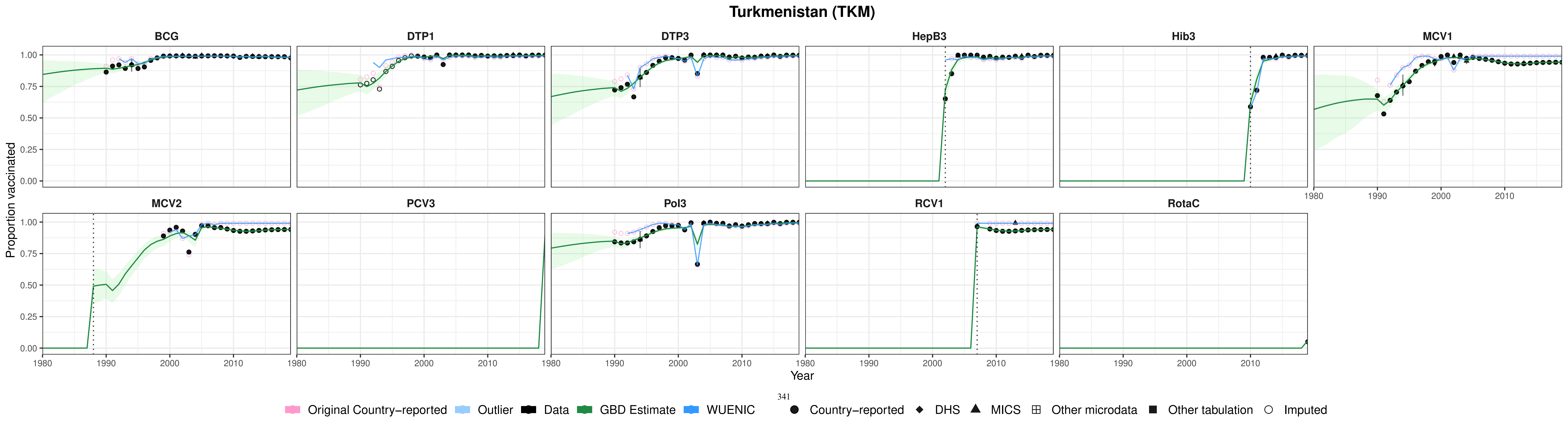
Turkmenistan (TKM)
BCG
DTP1
DTP3
HepB3
Hib3
MCV1
MCV2
PCV3
Pol3
RCV1
RotaC
Proportion vaccinated
0.00
0.25
0.50
0.75
1.00
1980
1990
2000
2010
Year
Original Country−reported
Outlier
Data
GBD Estimate
WUENIC
Country−reported
DHS
MICS
Other microdata
Other tabulation
Imputed

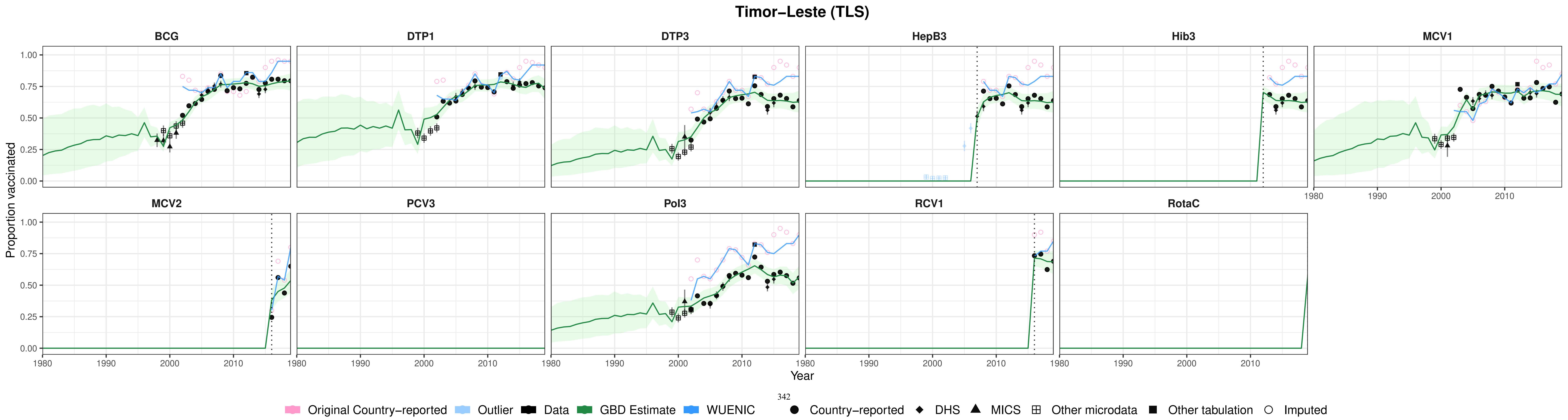

Timor−Leste (TLS)
BCG
DTP1
DTP3
HepB3
Hib3
MCV1
MCV2
PCV3
Pol3
RCV1
RotaC
Proportion vaccinated
Year
Original Country−reported
Outlier
Data
GBD Estimate
WUENIC
Country−reported
DHS
MICS
Other microdata
Other tabulation
Imputed

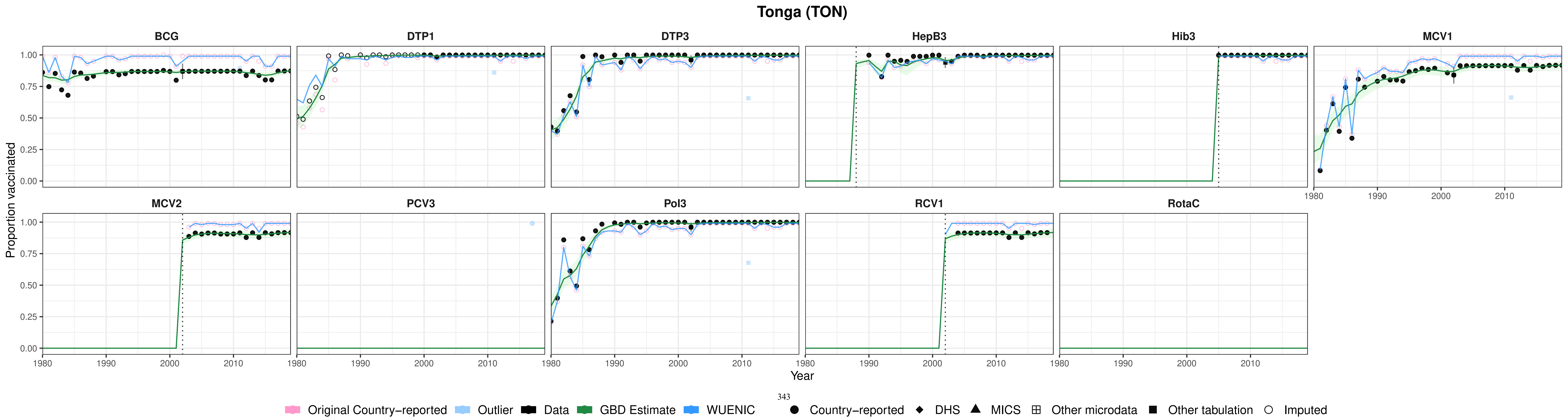
Tonga (TON)
BCG
DTP1
DTP3
HepB3
Hib3
MCV1
MCV2
PCV3
Pol3
RCV1
RotaC
Proportion vaccinated
Year
Original Country−reported
Outlier
Data
GBD Estimate
WUENIC
Country−reported
DHS
MICS
Other microdata
Other tabulation
Imputed

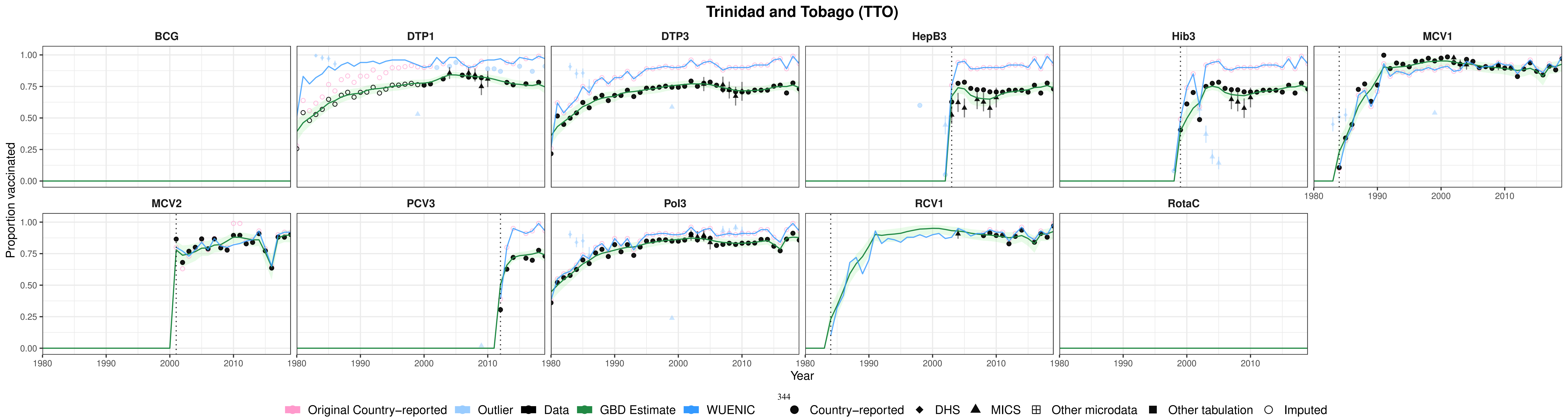

Trinidad and Tobago (TTO)
BCG
DTP1
DTP3
HepB3
Hib3
MCV1
MCV2
PCV3
Pol3
RCV1
RotaC
Proportion vaccinated
Year
Original Country−reported
Outlier
Data
GBD Estimate
WUENIC
Country−reported
DHS
MICS
Other microdata
Other tabulation
Imputed

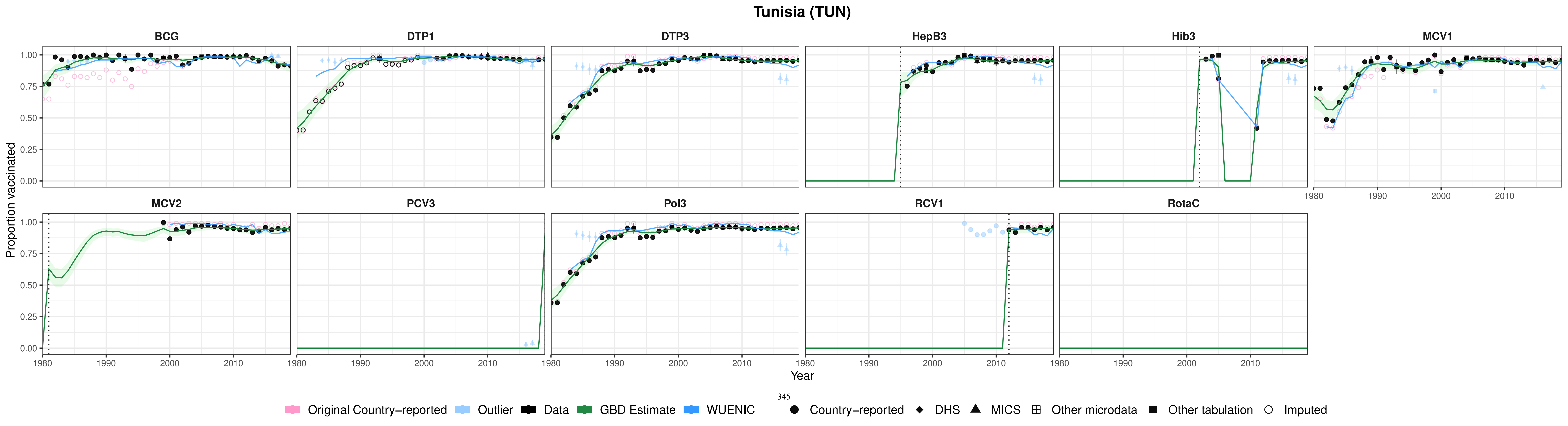
Tunisia (TUN)
BCG
DTP1
DTP3
HepB3
Hib3
MCV1
MCV2
PCV3
Pol3
RCV1
RotaC
Proportion vaccinated
0.00
0.25
0.50
0.75
1.00
1980
1990
2000
2010
Year
Original Country−reported
Outlier
Data
GBD Estimate
WUENIC
Country−reported
DHS
MICS
Other microdata
Other tabulation
Imputed

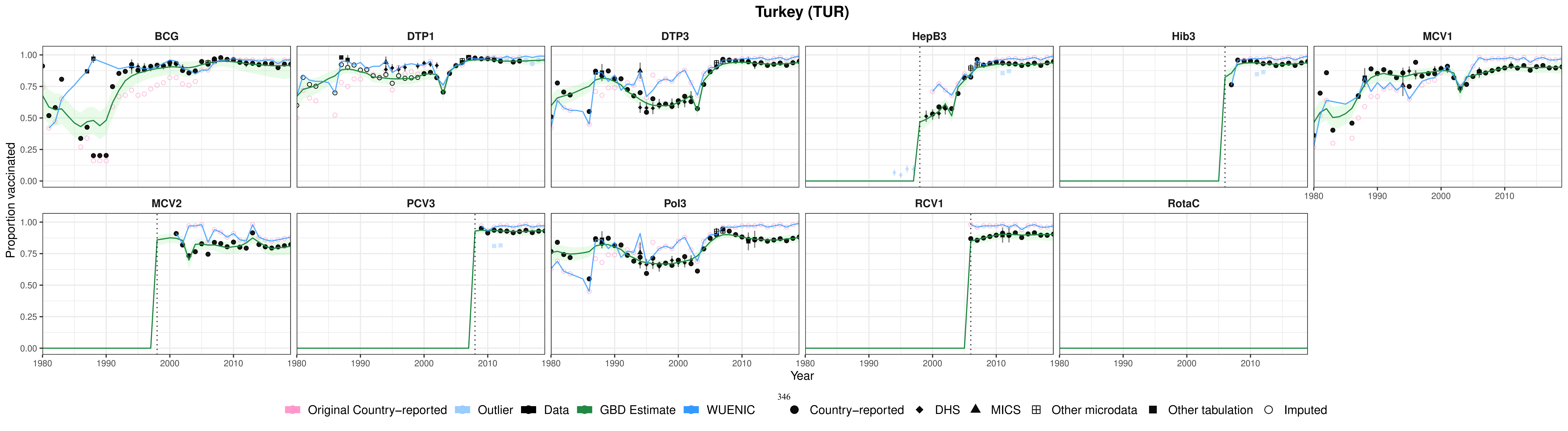
Turkey (TUR)
BCG
DTP1
DTP3
HepB3
Hib3
MCV1
MCV2
PCV3
Pol3
RCV1
RotaC
Proportion vaccinated
0.00
0.25
0.50
0.75
1.00
1980
1990
2000
2010
Year
Original Country−reported
Outlier
Data
GBD Estimate
WUENIC
Country−reported
DHS
MICS
Other microdata
Other tabulation
Imputed

Tuvalu (TUV)

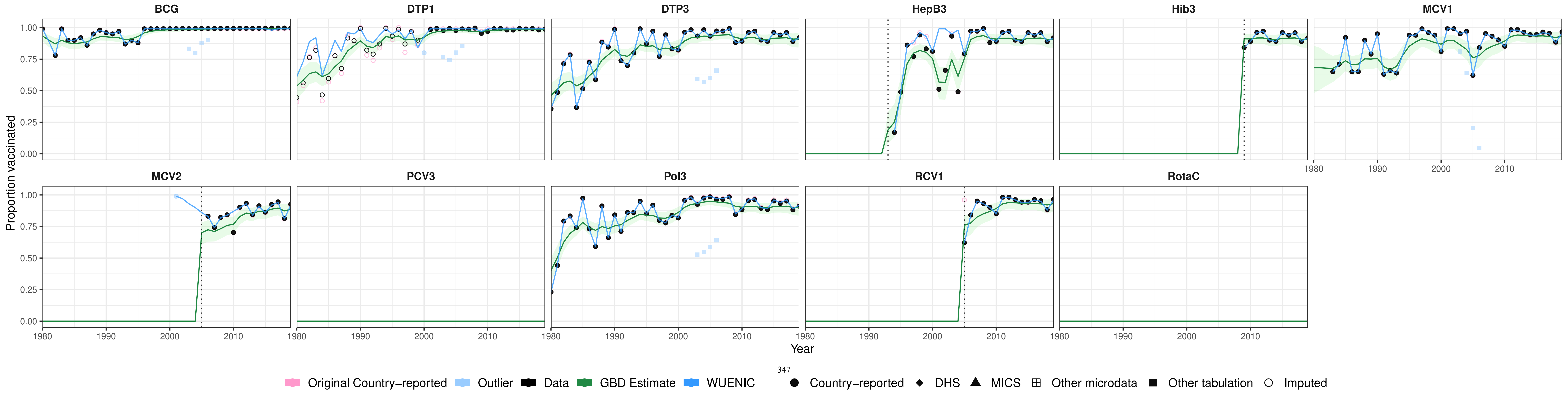

BCG
DTP1
DTP3
HepB3
Hib3
MCV1
MCV2
PCV3
Pol3
RCV1
RotaC
Proportion vaccinated
Year
0.00
0.25
0.50
0.75
1.00
1980
1990
2000
2010
Original Country−reported
Outlier
Data
GBD Estimate
WUENIC
Country−reported
DHS
MICS
Other microdata
Other tabulation
Imputed

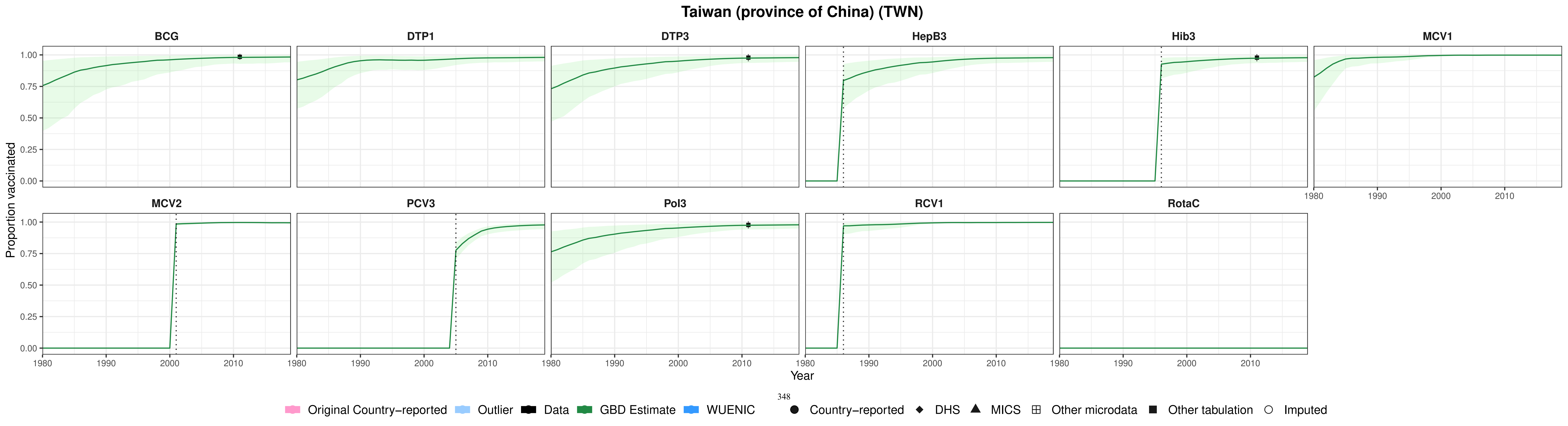
Taiwan (province of China) (TWN)
BCG
DTP1
DTP3
HepB3
Hib3
MCV1
MCV2
PCV3
Pol3
RCV1
RotaC
Proportion vaccinated
1.00
0.75
0.50
0.25
0.00
1980
1990
2000
2010
Year
Original Country−reported
Outlier
Data
GBD Estimate
WUENIC
Country−reported
DHS
MICS
Other microdata
Other tabulation
Imputed

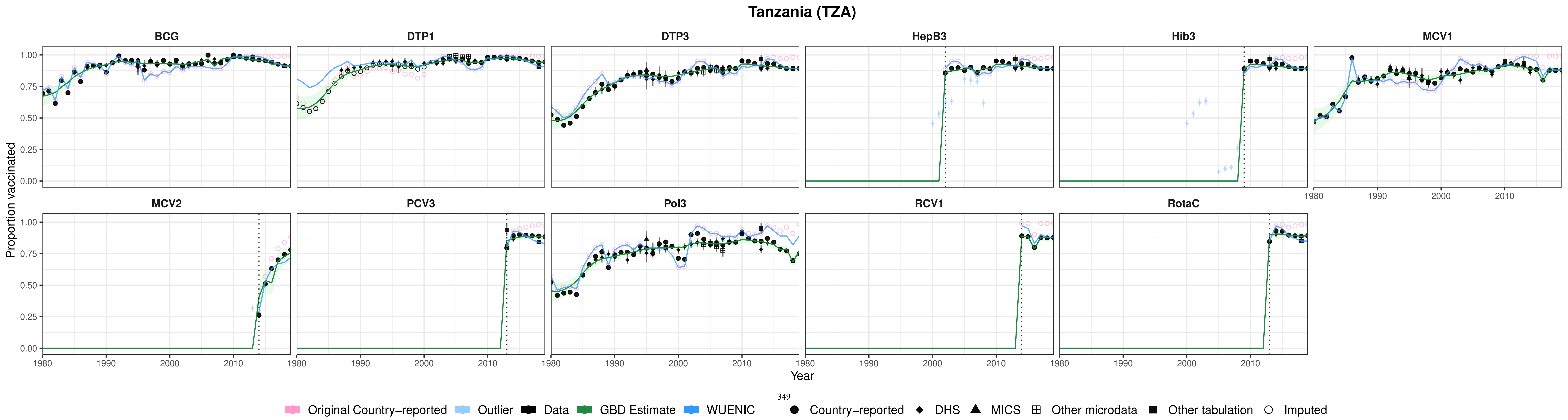
Tanzania (TZA)
BCG
DTP1
DTP3
HepB3
Hib3
MCV1
MCV2
PCV3
Pol3
RCV1
RotaC
Proportion vaccinated
Year
Original Country−reported
Outlier
Data
GBD Estimate
WUENIC
Country−reported
DHS
MICS
Other microdata
Other tabulation
Imputed

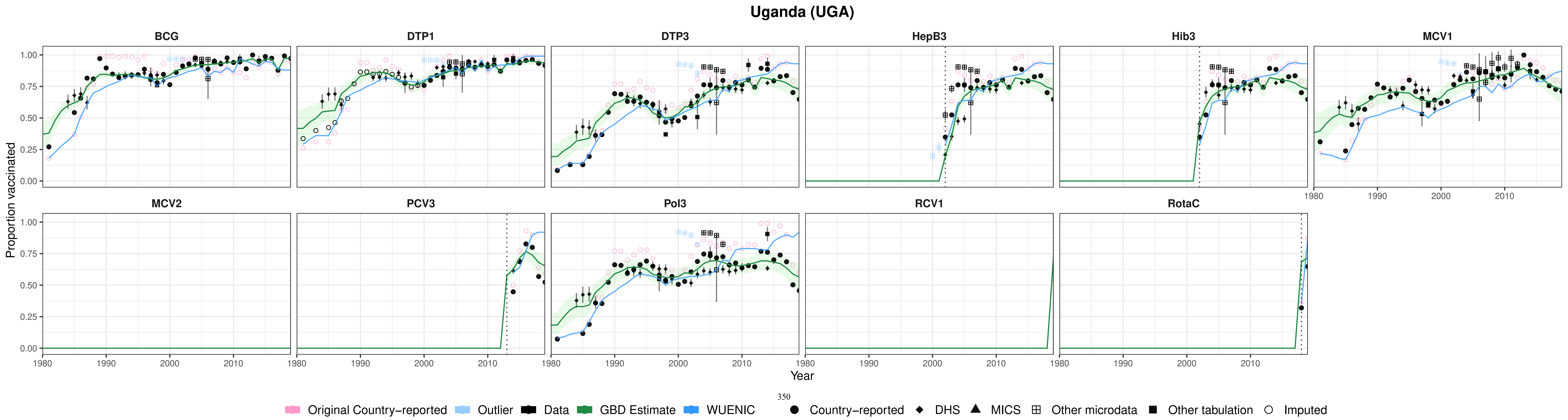

Uganda (UGA)
BCG
DTP1
DTP3
HepB3
Hib3
MCV1
MCV2
PCV3
Pol3
RCV1
RotaC
Proportion vaccinated
0.00
0.25
0.50
0.75
1.00
1980
1990
2000
2010
Year
Original Country−reported
Outlier
Data
GBD Estimate
WUENIC
Country−reported
DHS
MICS
Other microdata
Other tabulation
Imputed

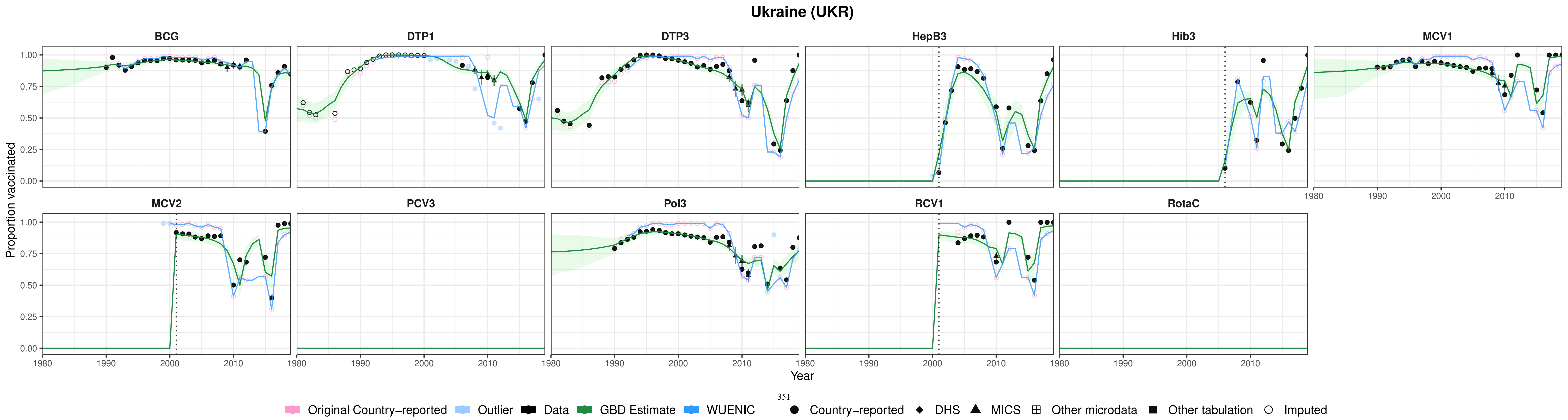

Ukraine (UKR)
BCG
DTP1
DTP3
HepB3
Hib3
MCV1
MCV2
PCV3
Pol3
RCV1
RotaC
Proportion vaccinated
Year
Original Country−reported
Outlier
Data
GBD Estimate
WUENIC
Country−reported
DHS
MICS
Other microdata
Other tabulation
Imputed

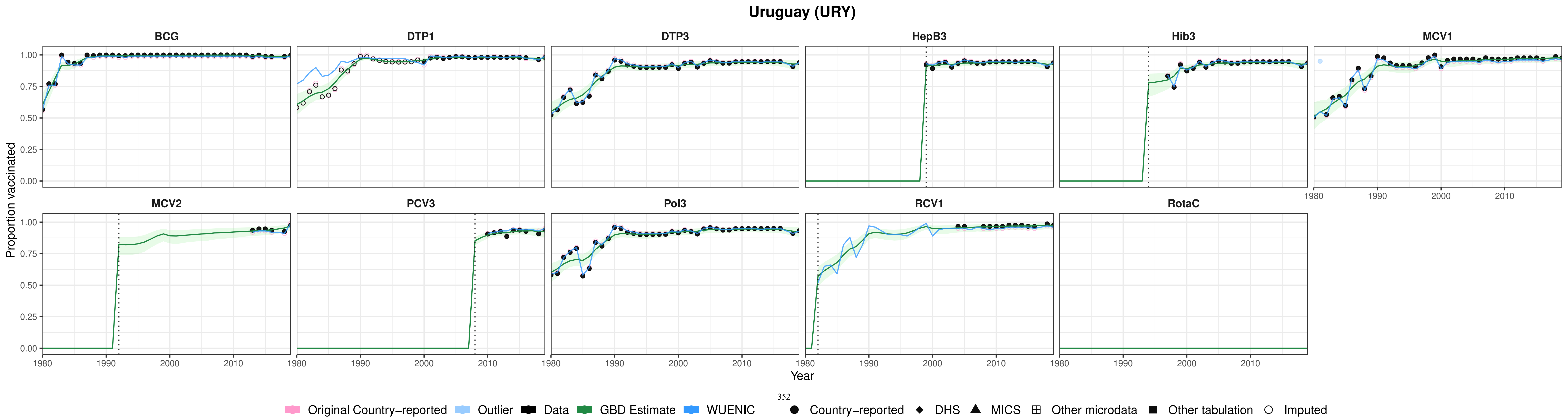

Uruguay (URY)
BCG
DTP1
DTP3
HepB3
Hib3
MCV1
MCV2
PCV3
Pol3
RCV1
RotaC
Proportion vaccinated
Year
1.00
0.75
0.50
0.25
0.00
1980
1990
2000
2010
Original Country−reported
Outlier
Data
GBD Estimate
WUENIC
Country−reported
DHS
MICS
Other microdata
Other tabulation
Imputed

USA (USA)

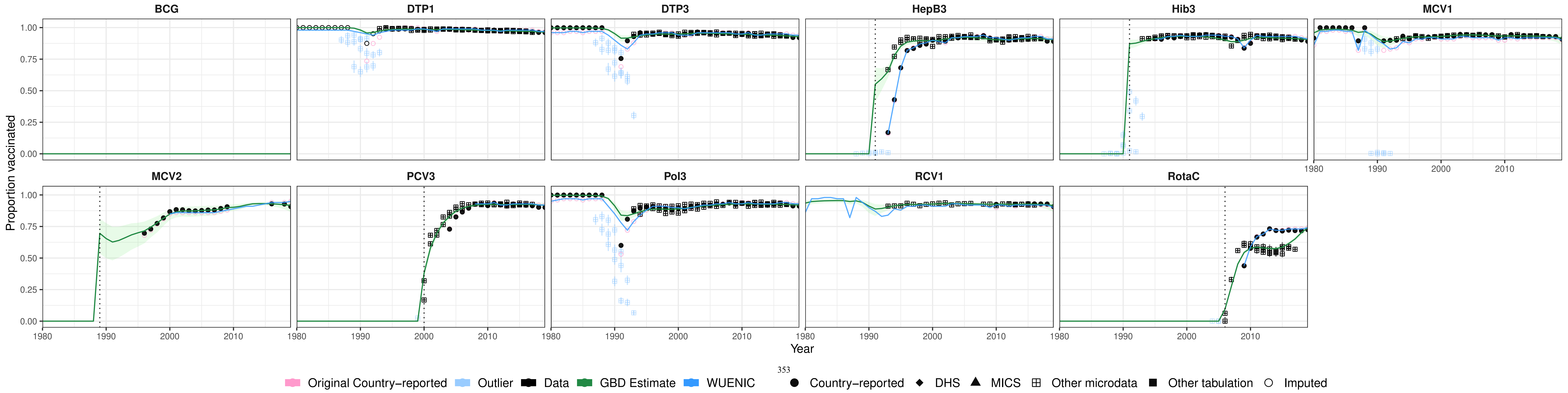

BCG
DTP1
DTP3
HepB3
Hib3
MCV1
MCV2
PCV3
Pol3
RCV1
RotaC
Proportion vaccinated
Year
1.00
0.75
0.50
0.25
0.00
1980
1990
2000
2010
Original Country−reported
Outlier
Data
GBD Estimate
WUENIC
Country−reported
DHS
MICS
Other microdata
Other tabulation
Imputed

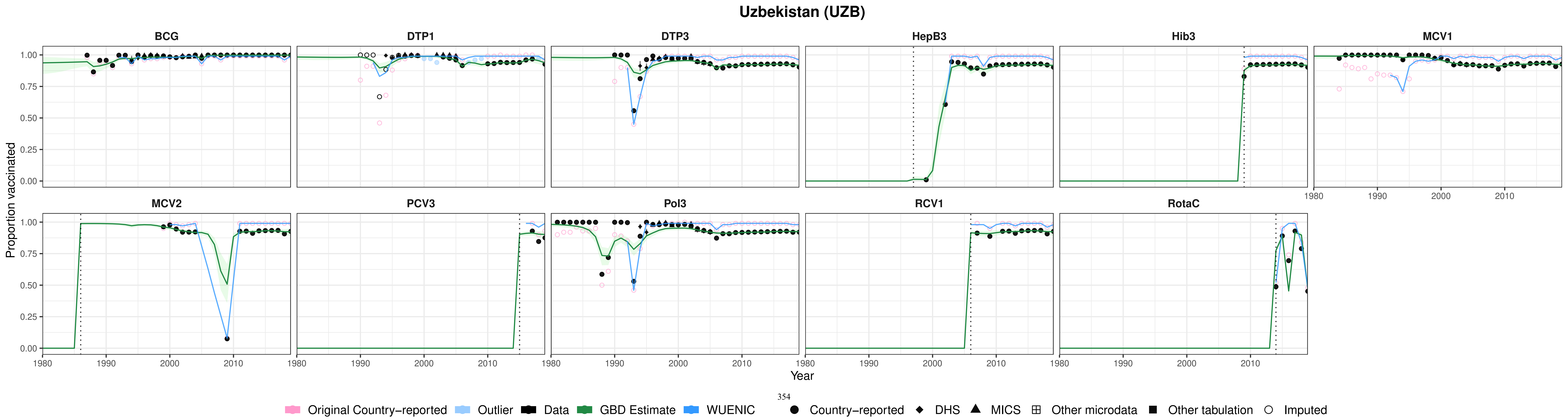

Uzbekistan (UZB)
BCG
DTP1
DTP3
HepB3
Hib3
MCV1
MCV2
PCV3
Pol3
RCV1
RotaC
Proportion vaccinated
Year
Original Country−reported
Outlier
Data
GBD Estimate
WUENIC
Country−reported
DHS
MICS
Other microdata
Other tabulation
Imputed

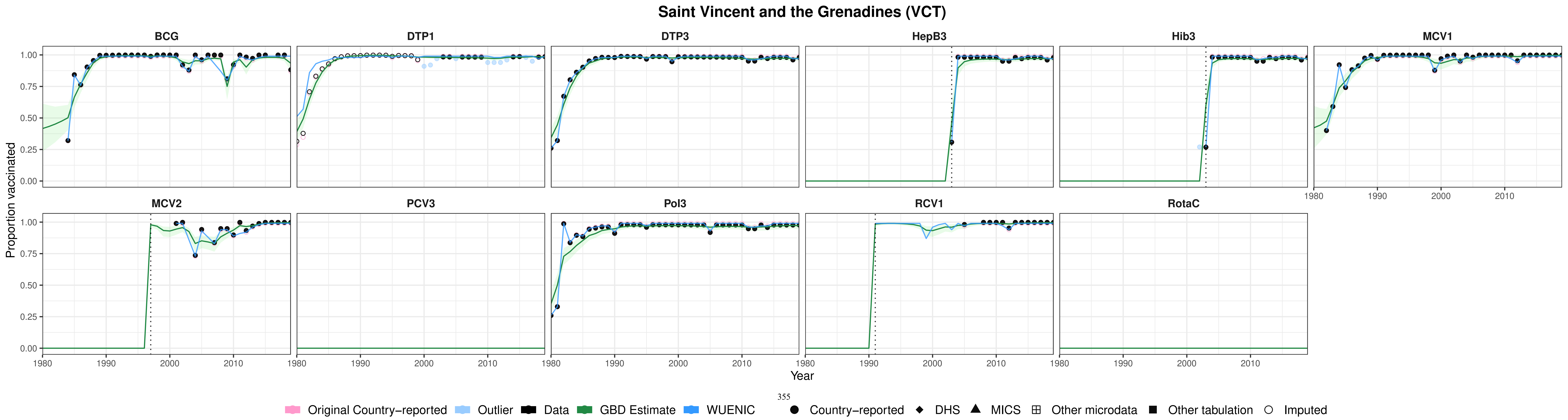

Saint Vincent and the Grenadines (VCT)
BCG
DTP1
DTP3
HepB3
Hib3
MCV1
MCV2
PCV3
Pol3
RCV1
RotaC
Proportion vaccinated
1.00
0.75
0.50
0.25
0.00
1980
1990
2000
2010
Year
Original Country−reported
Outlier
Data
GBD Estimate
WUENIC
Country−reported
DHS
MICS
Other microdata
Other tabulation
Imputed

Venezuela (VEN)

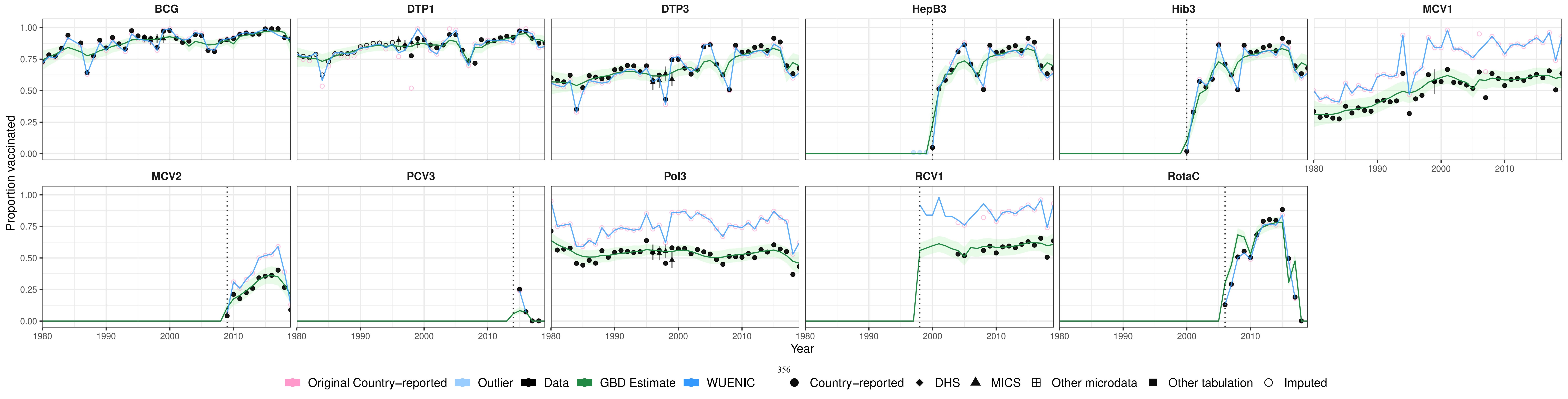

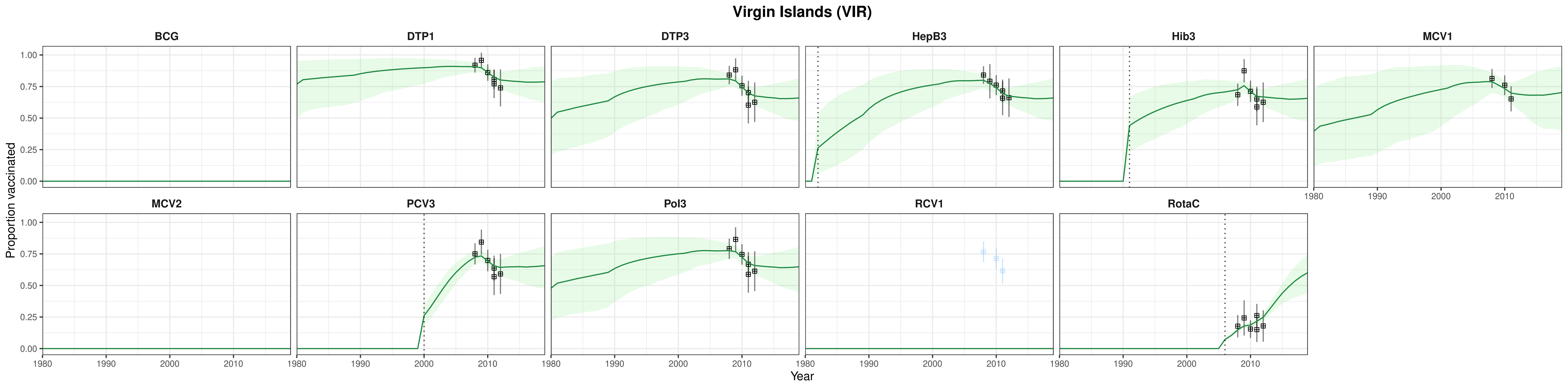

Virgin Islands (VIR)
BCG
DTP1
DTP3
HepB3
Hib3
MCV1
MCV2
PCV3
Pol3
RCV1
RotaC
Proportion vaccinated
Year
1.00
0.75
0.50
0.25
0.00
1980
1990
2000
2010
Original Country−reported
Outlier
Data
GBD Estimate
WUENIC
Country−reported
DHS
MICS
Other microdata
Other tabulation
Imputed

Vietnam (VNM)

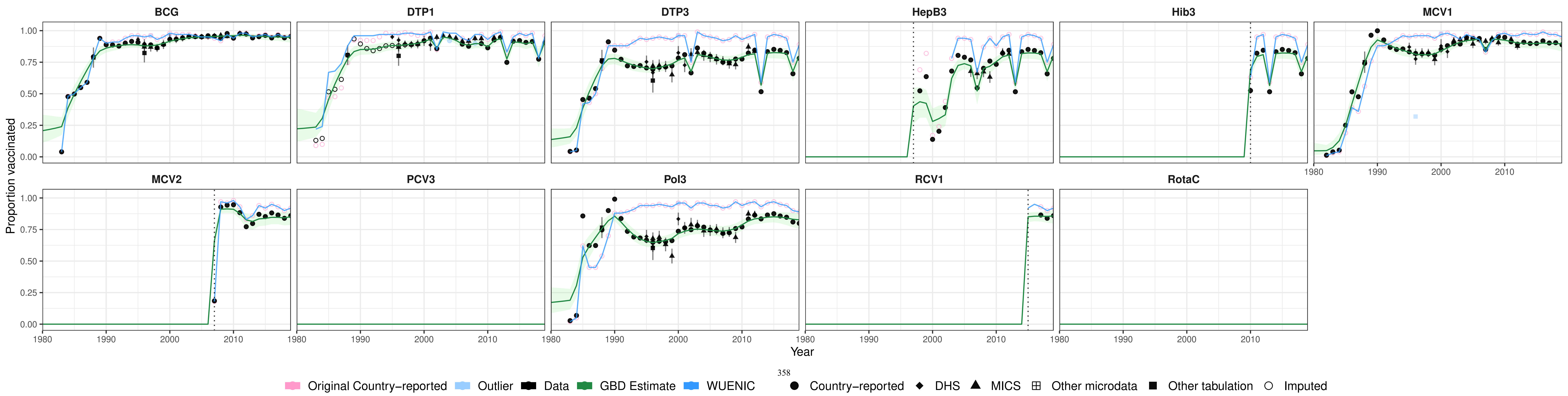
BCG
DTP1
DTP3
HepB3
Hib3
MCV1
MCV2
PCV3
Pol3
RCV1
RotaC
Proportion vaccinated
Year
1.00
0.75
0.50
0.25
0.00
1980
1990
2000
2010
Original Country-reported
Outlier
Data
GBD Estimate
WUENIC
Country-reported
DHS
MICS
Other microdata
Other tabulation
Imputed

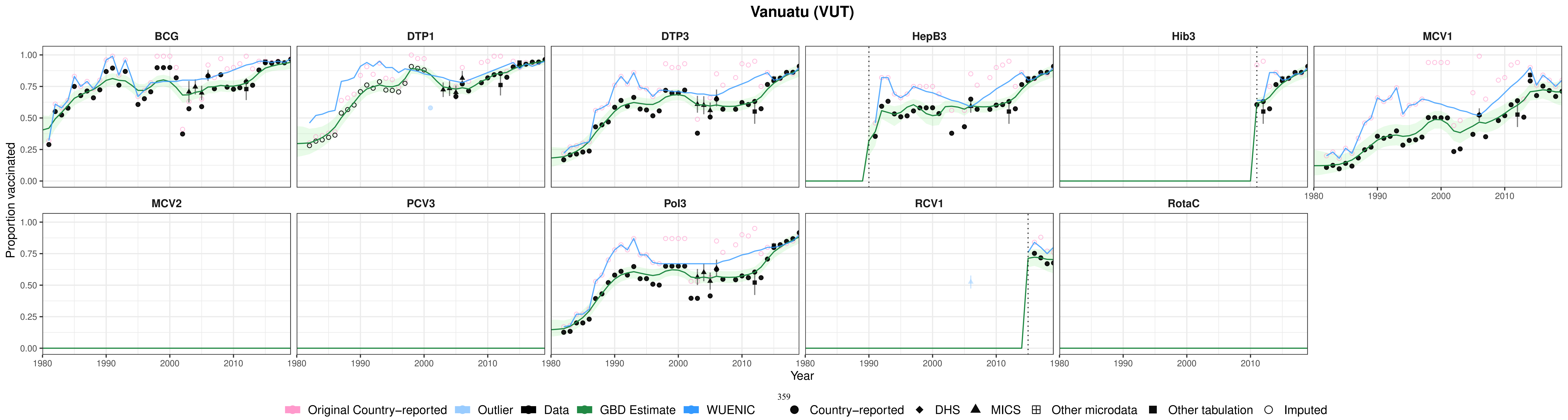
Vanuatu (VUT)
BCG
DTP1
DTP3
HepB3
Hib3
MCV1
MCV2
PCV3
Pol3
RCV1
RotaC
Proportion vaccinated
Year
Original Country−reported
Outlier
Data
GBD Estimate
WUENIC
Country−reported
DHS
MICS
Other microdata
Other tabulation
Imputed

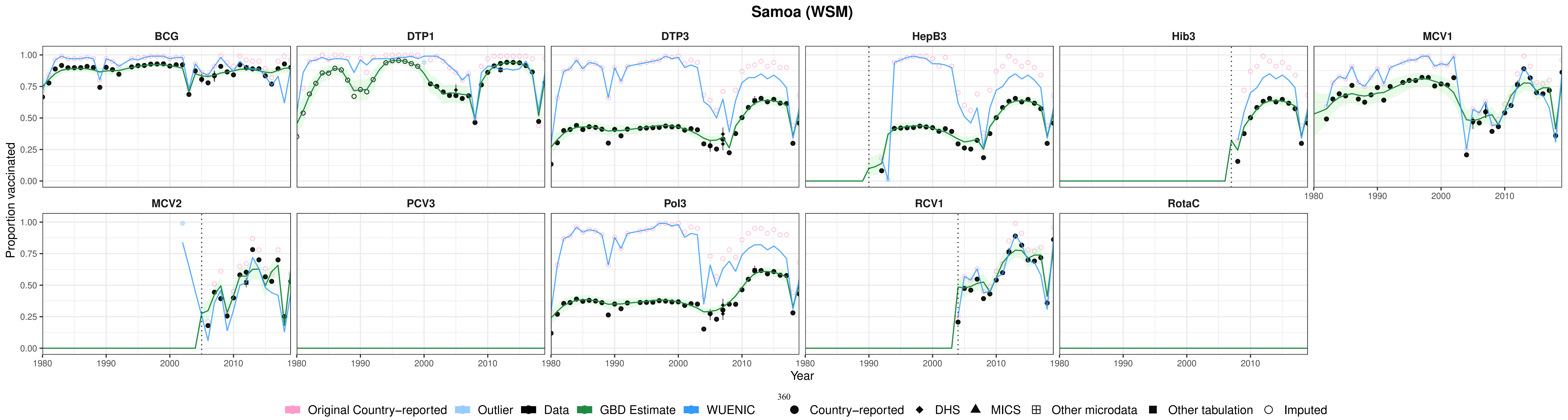
Samoa (WSM)
BCG
DTP1
DTP3
HepB3
Hib3
MCV1
MCV2
PCV3
Pol3
RCV1
RotaC
Proportion vaccinated
Year
Original Country−reported
Outlier
Data
GBD Estimate
WUENIC
Country−reported
DHS
MICS
Other microdata
Other tabulation
Imputed

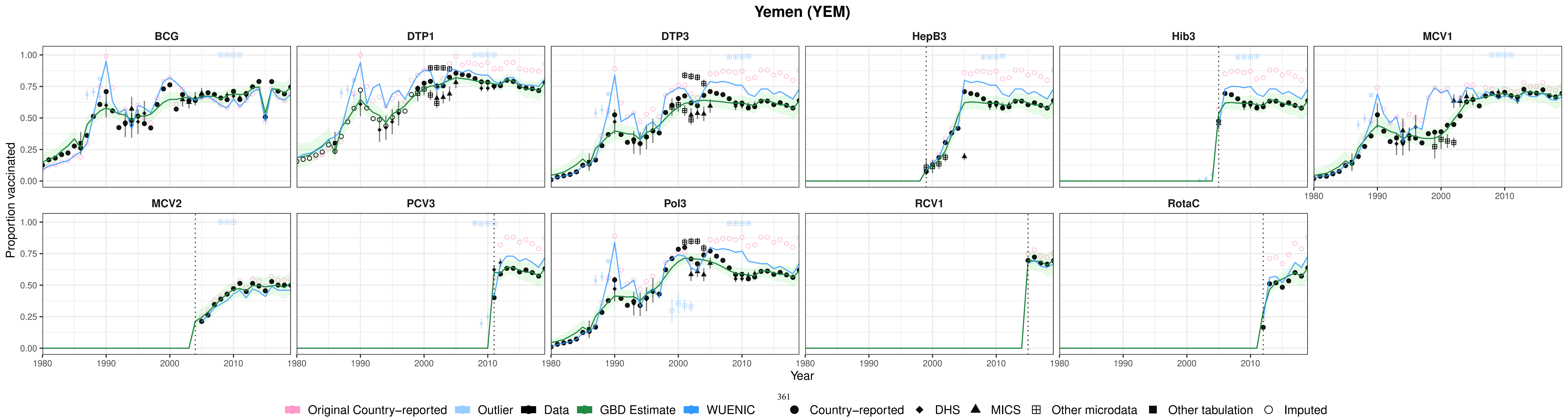
Yemen (YEM)
BCG
DTP1
DTP3
HepB3
Hib3
MCV1
MCV2
PCV3
Pol3
RCV1
RotaC
Proportion vaccinated
Year
Original Country−reported
Outlier
Data
GBD Estimate
WUENIC
Country−reported
DHS
MICS
Other microdata
Other tabulation
Imputed

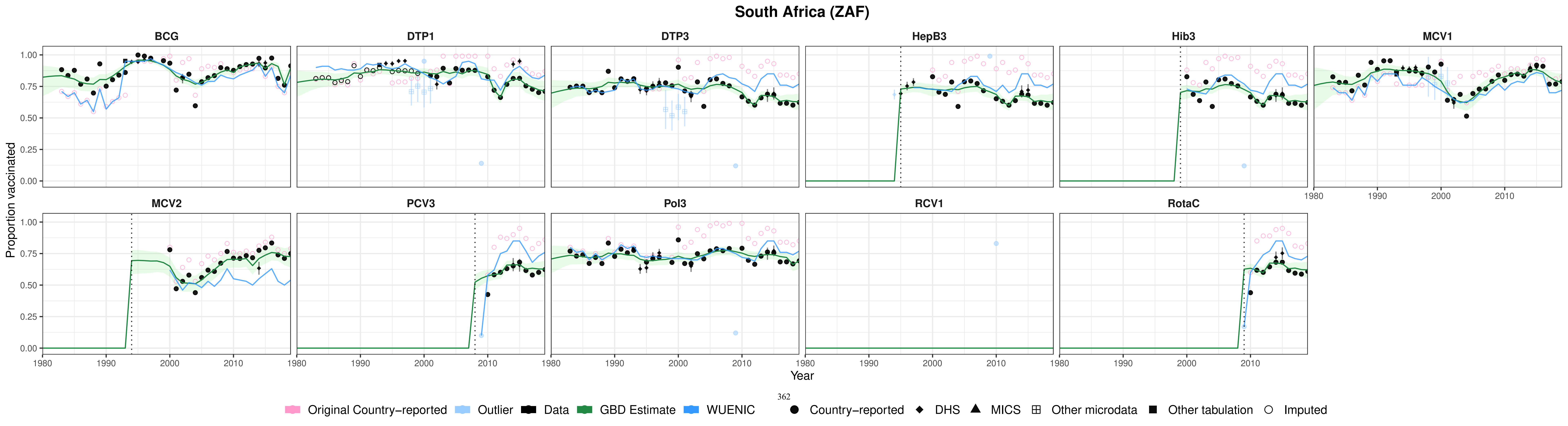
South Africa (ZAF)
BCG
DTP1
DTP3
HepB3
Hib3
MCV1
MCV2
PCV3
Pol3
RCV1
RotaC
Proportion vaccinated
Year
Original Country−reported
Outlier
Data
GBD Estimate
WUENIC
Country−reported
DHS
MICS
Other microdata
Other tabulation
Imputed

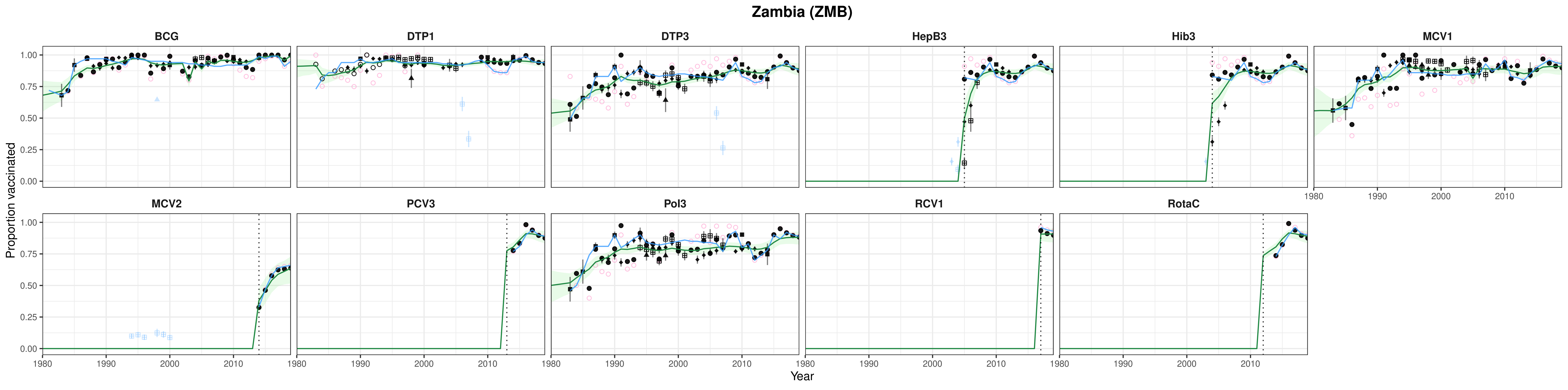
Zambia (ZMB)
BCG
DTP1
DTP3
HepB3
Hib3
MCV1
MCV2
PCV3
Pol3
RCV1
RotaC
Proportion vaccinated
Year
0.00
0.25
0.50
0.75
1.00
1980
1990
2000
2010
Original Country−reported
Outlier
Data
GBD Estimate
WUENIC
Country−reported
DHS
MICS
Other microdata
Other tabulation
Imputed

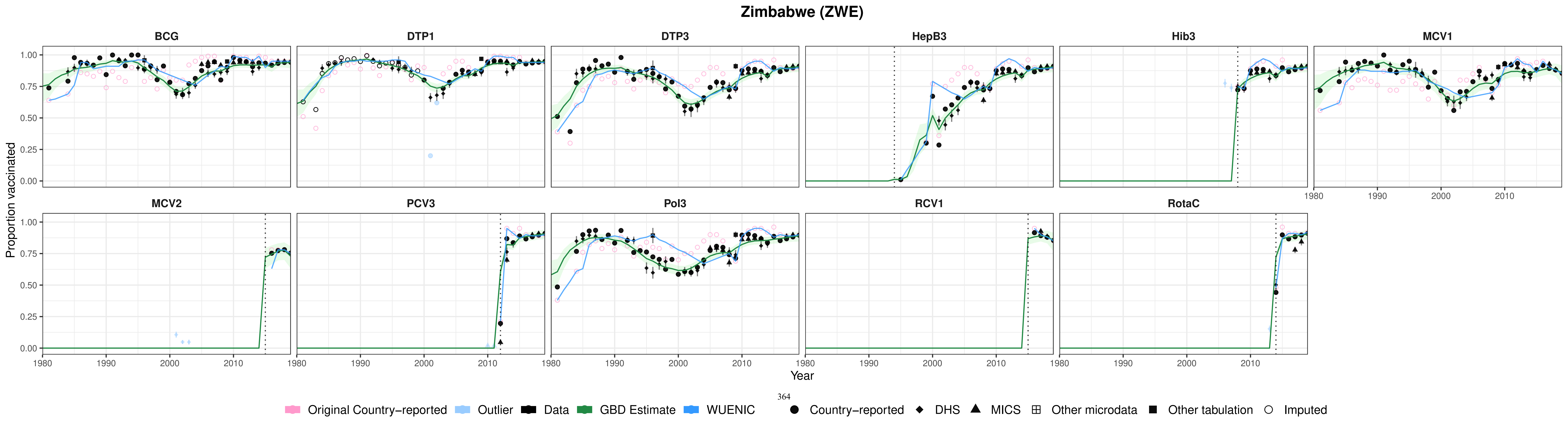
Zimbabwe (ZWE)
BCG
DTP1
DTP3
HepB3
Hib3
MCV1
MCV2
PCV3
Pol3
RCV1
RotaC
Proportion vaccinated
Year
Original Country−reported
Outlier
Data
GBD Estimate
WUENIC
Country−reported
DHS
MICS
Other microdata
Other tabulation
Imputed

Supplementary table 1. Data sources included in vaccine coverage modelling. List of sources with GHDx identification number included in the final analysis is provided. Vaccines with an "I" were included, vaccines with a "E" were excluded, and vaccines with "-" were not present. These sources can also be explored following this link to the GHDx input data sources tool: http://ghdx.healthdata.org/record/ihme-data/gbd-2020-routine-childhood-vaccination-coverage-1980-2019. BCG=Bacillus Calmette-Guérin. DTP1=diphtheria-tetanus-pertussis, first dose. DTP3=diphtheria-tetanus-pertussis, third dose. HepB3=hepatitis B vaccine, third dose. Hib3=*Haemophilus influenzae* type b vaccine, third dose. MCV1=measles-containing vaccine, first dose. MCV2=measles-containing vaccine, second dose. PCV3=pneumococcal conjugate vaccine, third dose. Pol3=polio vaccine, third dose. RCV1=rubella-containing vaccine, first dose. RotaC=completed rotavirus series.

GHDx ID	Country	Series	Year	BCG	DPT1	DPT3	HepB3	Hib3	MCV1	MCV2	PCV3	Pol3	RCV1	RotaC	Citation	Link
454354	Global	WHO and UNICEF Reported Official Target Population, Number of Doses Administered and Official Coverage 1966-2018	1966 - 2018	I	I	I	I	I	I	I	I	I	I	I	United Nations Children's Fund (UNICEF), World Health Organization (WHO). WHO and UNICEF Reported Official Target Population, Number of Doses Administered and Official Coverage 1966-2018. Geneva, Switzerland: World Health Organization (WHO), 2019.	GHDx
561	Afghanistan	Afghanistan Multiple Indicator Cluster Survey 2003	2003	I	I	I	-	-	E	-	-	I	-	-	Central Statistics Organization (Afghanistan), United Nations Children's Fund (UNICEF). Afghanistan Multiple Indicator Cluster Survey 2003.	GHDx
18468	Afghanistan	Afghanistan Health Survey 2006	2006	E	-	-	-	-	I	-	-	I	-	-	Indian Institute of Health Management Research (IIHMR), Johns Hopkins University, Ministry of Public Health (Afghanistan). Afghanistan Health Survey 2006.	GHDx
56830	Afghanistan	Afghanistan Multiple Indicator Cluster Survey 2010-2011	2010 - 2011	I	I	I	-	-	I	-	-	I	-	-	Central Statistics Organization (Afghanistan), United Nations Children's Fund (UNICEF). Afghanistan Multiple Indicator Cluster Survey 2010-2011. New York, United States of America: United Nations Children's Fund (UNICEF), 2013.	GHDx
108905	Afghanistan	Afghanistan National Risk and Vulnerability Assessment 2007-2008	2007 - 2008	I	-	I	-	-	I	-	-	I	-	-	Central Statistics Organization (Afghanistan), Ministry of Rural Rehabilitation and Development (Afghanistan). Afghanistan National Risk and Vulnerability Assessment 2007-2008.	GHDx
141905	Afghanistan	Afghanistan Multiple Indicator Baseline Survey 1997	1997	I	-	I	-	-	I	-	-	I	-	-	CIET International, United Nations Children's Fund (UNICEF). Afghanistan Multiple Indicator Baseline Survey 1997.	GHDx
157018	Afghanistan	Afghanistan Demographic and Health Survey 2015-2016	2015 - 2016	I	I	I	I	I	I	I	I	I	-	-	Central Statistics Organization (Afghanistan), ICF International, Ministry of Public Health (Afghanistan). Afghanistan Demographic and Health Survey 2015-2016. Fairfax, United States of America: ICF International, 2017.	GHDx
279695	Afghanistan	Afghanistan National Immunization Coverage Survey 2013	2013	I	I	I	I	I	I	-	-	I	-	-	Central Statistics Organization (Afghanistan), Health Protection and Research Organisation (Afghanistan), Ministry of Public Health (Afghanistan), United Nations Children's Fund (UNICEF). Afghanistan National Immunization Coverage Survey 2013.	GHDx
280108	Afghanistan	Afghanistan Health Survey 2012	2012	I	-	I	I	I	I	-	-	I	-	-	Indian Institute of Health Management Research (IIHMR), Johns Hopkins University, Ministry of Public Health (Afghanistan). Afghanistan Health Survey 2012.	GHDx
329519	Afghanistan	Progress towards poliomyelitis eradication: Afghanistan, January 2015-August 2016	2015 - 2016	-	-	-	-	-	-	-	-	I	-	-	World Health Organization. Progress towards poliomyelitis eradication: Afghanistan, January 2015-August 2016. WHO Wkly Epidemiol Rec. 2016; 91(44): 517-24.	GHDx
338771	Afghanistan	Afghanistan Health Survey 2015	2015	I	-	I	I	I	I	-	-	I	-	-	Ministry of Public Health (Afghanistan), Royal Tropical Institute. Afghanistan Health Survey 2015.	GHDx
18834	Albania	Albania Demographic and Health Survey 2008-2009	2008 - 2009	I	I	I	I	-	I	-	-	I	-	-	ICF Macro, Institute of Public Health (Albania), Institute of Statistics (Albania). Albania Demographic and Health Survey 2008-2009. Fairfax, United States of America: ICF International, 2009.	GHDx
627	Algeria	Algeria Family Health Survey 2002-2003	2002 - 2003	I	I	I	-	-	I	-	-	-	-	-	National Office of Statistics (Algeria), Ministry of Health, Population and Hospital Reform (Algeria), League of Arab States. Algeria Family Health Survey 2002-2003.	GHDx
634	Algeria	Algeria Maternal and Child Health Survey 1992	1992	I	I	I	-	-	I	-	-	I	-	-	National Office of Statistics (Algeria), League of Arab States. Algeria Maternal and Child Health Survey 1992.	GHDx

GHDx ID	Country	Series	Year	BCG	DPT1	DPT3	HepB3	Hib3	MCV1	MCV2	PCV3	Pol3	RCV1	RotaC	Citation	Link
641	Algeria	Algeria Multiple Indicator Cluster Survey 2006	2006	I	I	I	-	-	I	-	-	I	-	-	Joint United Nations Program on HIV/AIDS (UNAIDS), Ministry of Health, Population and Hospital Reform (Algeria), National Office of Statistics (Algeria), United Nations Children's Fund (UNICEF), United Nations Population Fund (UNFPA). Algeria Multiple Indicator Cluster Survey 2006.	GHDx
26449	Algeria	Algeria Multiple Indicator Cluster Survey 2000	2000	I	E	I	-	-	I	-	-	I	-	-	Ministry of Health and Population (Algeria), National Institute of Public Health (Algeria), National Office of Statistics (Algeria), United Nations Children's Fund (UNICEF). Algeria Multiple Indicator Cluster Survey 2000.	GHDx
210614	Algeria	Algeria Multiple Indicator Cluster Survey 2012-2013	2012 - 2013	I	I	I	-	I	I	-	-	I	-	-	Ministry of Health and Population (Algeria), United Nations Children's Fund (UNICEF). Algeria Multiple Indicator Cluster Survey 2012-2013. New York, United States of America: United Nations Children's Fund (UNICEF), 2018.	GHDx
224847	Algeria	Algeria National Immunization Coverage Survey 1989	1989	I	I	I	-	-	I	-	-	I	-	-	Algeria National Immunization Coverage Survey 1989.	GHDx
674	Angola	Angola Multiple Indicator Cluster Survey 1996	1996	I	-	I	-	-	I	-	-	I	-	-	Living Conditions Monitoring Office (Angola), National Statistics Office (Angola), United Nations Children's Fund (UNICEF). Angola Multiple Indicator Cluster Survey 1996. New York, United States of America: United Nations Children's Fund (UNICEF).	GHDx
687	Angola	Angola Multiple Indicator Cluster Survey 2001	2001	I	I	I	-	-	I	-	-	I	-	-	National Institute of Statistics (Angola), United Nations Children's Fund (UNICEF). Angola Multiple Indicator Cluster Survey 2001. New York, United States of America: United Nations Children's Fund (UNICEF).	GHDx
30394	Angola	Angola Integrated Inquiry into People's Well-Being 2008-2009	2008 - 2009	I	I	I	I	I	I	-	-	I	-	-	National Institute of Statistics (Angola), Oxford Policy Management, United Nations Children's Fund (UNICEF). Angola Integrated Inquiry into People's Well-Being 2008-2009.	GHDx
218555	Angola	Angola Demographic and Health Survey 2015-2016	2015 - 2016	I	I	I	I	I	I	E	I	I	-	I	ICF International, Ministry of Health (Angola), National Institute of Statistics (Angola), United Nations Children's Fund (UNICEF). Angola Demographic and Health Survey 2015-2016. Fairfax, United States of America: ICF International, 2017.	GHDx
18843	Armenia	Armenia Demographic and Health Survey 2000	2000	I	I	I	I	-	I	-	-	I	-	-	Ministry of Health (Armenia), National Statistical Service (NSS), ORC Macro. Armenia Demographic and Health Survey 2000. Fairfax, United States of America: ICF International.	GHDx
31750	Armenia	Armenia Demographic and Health Survey 2010	2010	I	I	I	I	I	I	-	-	I	-	-	ICF Macro, Ministry of Health (Armenia), National Statistical Service of the Republic of Armenia. Armenia Demographic and Health Survey 2010. Fairfax, United States of America: ICF International, 2015.	GHDx
215019	Armenia	Armenia Evaluation of the National Immunization Program 1999	1999	I	-	I	-	-	I	-	-	I	-	-	Ministry of Health (Armenia), United Nations Children's Fund (UNICEF). Armenia Evaluation of the National Immunization Program 1999.	GHDx
218563	Armenia	Armenia Demographic and Health Survey 2015-2016	2015 - 2016	I	I	I	I	I	I	-	I	I	I	I	ICF International, Ministry of Health (Armenia), National Statistical Service of the Republic of Armenia. Armenia Demographic and Health Survey 2015-2016. Fairfax, United States of America: ICF International, 2017.	GHDx
85056	Australia	Australia National Health Survey 2001	2001	-	-	-	-	-	I	-	-	I	-	-	Australian Bureau of Statistics. Australia National Health Survey 2001. Canberra, Australia: Australian Bureau of Statistics.	GHDx
881	Azerbaijan	Azerbaijan Multiple Indicator Cluster Survey 2000	2000	I	I	I	-	-	I	-	-	I	-	-	State Statistics Committee of Azerbaijan, United Nations Children's Fund (UNICEF). Azerbaijan Multiple Indicator Cluster Survey 2000. New York, United States: United Nations Children's Fund (UNICEF).	GHDx
18865	Azerbaijan	Azerbaijan Demographic and Health Survey 2006	2006	I	I	I	I	-	I	-	-	I	I	-	Macro International, Inc, State Statistical Committee of Azerbaijan. Azerbaijan Demographic and Health Survey 2006. Fairfax, United States of America: ICF International.	GHDx
222676	Azerbaijan	Azerbaijan Demographic and Health Survey 2011	2011	I	I	I	I	-	I	-	-	I	I	-	Public Health and Reforms Center, Ministry of Health (PHRC) (Azerbaijan). Azerbaijan Demographic and Health Survey 2011.	GHDx
281740	Azerbaijan	Azerbaijan Maternal and Child Health Survey 2000	2000	I	-	I	-	-	-	-	-	I	-	-	Save the Children USA. Azerbaijan Maternal and Child Health Survey 2000.	GHDx
905	Bahrain	Bahrain Child Health Survey 1989	1989	-	I	I	-	-	I	-	-	I	E	-	Central Statistics Organization (Bahrain), Ministry of Health (Bahrain). Bahrain Child Health Survey 1989.	GHDx
907	Bahrain	Bahrain Family Health Survey 1995	1995	-	I	I	-	-	I	-	-	I	-	-	Ministry of Health (Bahrain), Council of Health Ministers of GCC States. Bahrain Family Health Survey 1995. Manama, Bahrain: Ministry of Health (Bahrain).	GHDx
215053	Bahrain	Bahrain Review of the EPI 1980	1980	-	I	I	-	-	I	-	-	I	-	-	Bahrain Review of the EPI 1980.	GHDx

GHDx ID	Country	Series	Year	BCG	DPT1	DPT3	HepB3	Hib3	MCV1	MCV2	PCV3	Pol3	RCV1	RotaC	Citation	Link
215056	Bahrain	Bahrain Ministry of Health Reported Immunization Coverage Data, In Response to Draft Estimates, June 2001	1988	-	-	I	-	-	I	-	-	I	-	-	Ministry of Health (Bahrain). Bahrain Ministry of Health Reported Immunization Coverage Data, In Response to Draft Estimates, June 2001.	GHDx
951	Bangladesh	Bangladesh Multiple Indicator Cluster Survey 2006	2006	I	I	I	I	-	I	-	-	I	-	-	Bangladesh Bureau of Statistics (BBS), Mitra and Associates, United Nations Children's Fund (UNICEF). Bangladesh Multiple Indicator Cluster Survey 2006. New York, United States of America: United Nations Children's Fund (UNICEF).	GHDx
18878	Bangladesh	Bangladesh Demographic and Health Survey 1996-1997	1996 - 1997	I	I	I	-	-	I	-	-	I	-	-	Macro International, Inc, Mitra and Associates, National Institute of Population Research and Training (NIPORT). Bangladesh Demographic and Health Survey 1996-1997. Fairfax, United States of America: ICF International.	GHDx
18889	Bangladesh	Bangladesh Demographic and Health Survey 1993-1994	1993 - 1994	I	I	I	-	-	I	-	-	I	-	-	Macro International, Inc, Mitra and Associates, National Institute of Population Research and Training (NIPORT). Bangladesh Demographic and Health Survey 1993-1994. Fairfax, United States of America: ICF International.	GHDx
18902	Bangladesh	Bangladesh Demographic and Health Survey 2004	2004	I	I	I	-	-	I	-	-	I	-	-	Mitra and Associates, ORC Macro. Bangladesh Demographic and Health Survey 2004. Fairfax, United States of America: ICF International.	GHDx
18913	Bangladesh	Bangladesh Demographic and Health Survey 2007	2007	I	I	I	I	-	I	-	-	I	-	-	Macro International, Inc, Mitra and Associates, National Institute of Population Research and Training (NIPORT). Bangladesh Demographic and Health Survey 2007. Fairfax, United States of America: ICF International, 2009.	GHDx
22129	Bangladesh	Bangladesh Multiple Indicator Cluster Survey 1996	1996	-	I	I	-	-	-	-	-	I	-	-	Bangladesh Bureau of Statistics (BBS), United Nations Children's Fund (UNICEF). Bangladesh Multiple Indicator Cluster Survey 1996.	GHDx
26826	Bangladesh	Bangladesh Demographic and Health Survey 1999-2000	1999 - 2000	I	I	I	-	-	I	-	-	I	-	-	Macro Systems, Inc, Mitra and Associates, National Institute of Population Research and Training (NIPORT). Bangladesh Demographic and Health Survey 1999-2000. Fairfax, United States of America: ICF International.	GHDx
55956	Bangladesh	Bangladesh Demographic and Health Survey 2011-2012	2011 - 2012	I	I	I	I	I	I	-	-	I	-	-	ICF Macro, Mitra and Associates, National Institute of Population Research and Training (NIPORT). Bangladesh Demographic and Health Survey 2011-2012. Calverton, United States of America: ICF Macro.	GHDx
107342	Bangladesh	Bangladesh EPI Coverage Evaluation Survey 2006	2006	I	I	I	-	-	I	-	-	I	-	-	ACNielsen Bangladesh, Expanded Program on Immunization (Bangladesh), United Nations Children's Fund (UNICEF), World Health Organization (WHO). Bangladesh EPI Coverage Evaluation Survey 2006.	GHDx
108614	Bangladesh	Bangladesh EPI Coverage Evaluation Survey 2005	2005	I	I	I	-	-	I	-	-	I	-	-	ACNielsen, Directorate General of Health Services, Ministry of Health and Family Welfare (Bangladesh), United Nations Children's Fund (UNICEF), World Health Organization (WHO). Bangladesh EPI Coverage Evaluation Survey 2005.	GHDx
157021	Bangladesh	Bangladesh Demographic and Health Survey 2014	2014	I	I	I	I	I	I	-	-	I	-	-	ICF International, Mitra and Associates, National Institute of Population Research and Training (NIPORT). Bangladesh Demographic and Health Survey 2014. Fairfax, United States of America: ICF International, 2015.	GHDx
189500	Bangladesh	Bangladesh Multiple Indicator Cluster Survey 1994	1994	-	I	I	-	-	I	-	-	-	-	-	Bangladesh Bureau of Statistics (BBS), United Nations Children's Fund (UNICEF). Bangladesh Multiple Indicator Cluster Survey 1994.	GHDx
189502	Bangladesh	Bangladesh Multiple Indicator Cluster Survey 1995	1995	-	I	I	-	-	I	-	-	-	-	-	Bangladesh Bureau of Statistics (BBS), United Nations Children's Fund (UNICEF). Bangladesh Multiple Indicator Cluster Survey 1995.	GHDx
215037	Bangladesh	Bangladesh EPI Coverage Evaluation Survey 1991	1991 - 1992	I	I	I	-	-	I	-	-	-	-	-	Directorate General of Health Services, Ministry of Health and Family Welfare (Bangladesh), United Nations Children's Fund (UNICEF), World Health Organization (WHO). Bangladesh EPI Coverage Evaluation Survey 1991.	GHDx
215038	Bangladesh	Bangladesh EPI Coverage Evaluation Survey 1992	1992	I	I	I	-	-	I	-	-	I	-	-	Directorate General of Health Services, Ministry of Health and Family Welfare (Bangladesh), United Nations Children's Fund (UNICEF), World Health Organization (WHO). Bangladesh EPI Coverage Evaluation Survey 1992.	GHDx
215044	Bangladesh	Achieving the Mid-Decade Goals for Children in Bangladesh 1996	1996	-	-	I	-	-	I	-	-	I	-	-	Bangladesh Bureau of Statistics (BBS), United Nations Children's Fund (UNICEF). Achieving the Mid-Decade Goals for Children in Bangladesh 1996.	GHDx

GHDx ID	Country	Series	Year	BCG	DPT1	DPT3	HepB3	Hib3	MCV1	MCV2	PCV3	Pol3	RCV1	RotaC	Citation	Link
215045	Bangladesh	Bangladesh EPI Coverage Evaluation Survey 1997	1997	I	-	I	-	-	I	-	-	I	-	-	Directorate General of Health Services, Ministry of Health and Family Welfare (Bangladesh), United Nations Children's Fund (UNICEF), World Health Organization (WHO). Bangladesh EPI Coverage Evaluation Survey 1997.	GHDx
215046	Bangladesh	Bangladesh EPI Coverage Evaluation Survey 1998	1998	I	-	I	-	-	I	-	-	I	-	-	Directorate General of Health Services, Ministry of Health and Family Welfare (Bangladesh), United Nations Children's Fund (UNICEF), World Health Organization (WHO). Bangladesh EPI Coverage Evaluation Survey 1998.	GHDx
215047	Bangladesh	Bangladesh EPI Coverage Evaluation Survey 2000	2000	I	I	I	-	-	I	-	-	I	-	-	Directorate General of Health Services, Ministry of Health and Family Welfare (Bangladesh), United Nations Children's Fund (UNICEF), World Health Organization (WHO). Bangladesh EPI Coverage Evaluation Survey 2000.	GHDx
215048	Bangladesh	Bangladesh EPI Coverage Evaluation Survey 2002	2002	I	I	I	-	-	I	-	-	I	-	-	Directorate General of Health Services, Ministry of Health and Family Welfare (Bangladesh), United Nations Children's Fund (UNICEF), World Health Organization (WHO). Bangladesh EPI Coverage Evaluation Survey 2002.	GHDx
215049	Bangladesh	Bangladesh EPI Coverage Evaluation Survey 2001	2001	I	I	I	-	-	I	-	-	I	-	-	Directorate General of Health Services, Ministry of Health and Family Welfare (Bangladesh), United Nations Children's Fund (UNICEF), World Health Organization (WHO). Bangladesh EPI Coverage Evaluation Survey 2001.	GHDx
215050	Bangladesh	Bangladesh EPI Coverage Evaluation Survey 2003	2003	I	I	I	-	-	I	-	-	I	-	-	Directorate General of Health Services, Ministry of Health and Family Welfare (Bangladesh), United Nations Children's Fund (UNICEF), World Health Organization (WHO). Bangladesh EPI Coverage Evaluation Survey 2003.	GHDx
215051	Bangladesh	Bangladesh EPI Coverage Evaluation Survey 2007-2008	2007 - 2008	I	I	I	I	-	I	-	-	I	-	-	ACNielsen Bangladesh, Directorate General of Health Services, Ministry of Health and Family Welfare (Bangladesh), United Nations Children's Fund (UNICEF), World Health Organization (WHO). Bangladesh EPI Coverage Evaluation Survey 2007-2008.	GHDx
215052	Bangladesh	Bangladesh EPI Coverage Evaluation Survey 2013	2013	I	I	I	I	I	I	-	-	I	-	-	ACNielsen Bangladesh, Directorate General of Health Services, Ministry of Health and Family Welfare (Bangladesh), United Nations Children's Fund (UNICEF), World Health Organization (WHO). Bangladesh EPI Coverage Evaluation Survey 2013.	GHDx
268016	Bangladesh	Bangladesh EPI Coverage Evaluation Survey 2011	2011	I	I	I	I	I	I	-	-	I	-	-	Directorate General of Health Services, Ministry of Health and Family Welfare (Bangladesh), Nielsen Company Bangladesh, United Nations Children's Fund (UNICEF), World Health Organization (WHO). Bangladesh EPI Coverage Evaluation Survey 2011.	GHDx
275001	Bangladesh	Bangladesh Utilization of Essential Service Delivery Survey 2013	2013	I	I	I	I	I	I	-	-	I	-	-	Associates for Community and Population Research (ACPR), National Institute of Population Research and Training (NIPORT). Bangladesh Utilization of Essential Service Delivery Survey 2013. Dhaka, Bangladesh: National Institute of Population Research and Training (NIPORT).	GHDx
282828	Bangladesh	Bangladesh Health and Morbidity Status Survey 2012	2012	I	I	I	I	I	I	-	-	I	-	-	Bangladesh Bureau of Statistics (BBS), Ministry of Planning (Bangladesh). Bangladesh Health and Morbidity Status Survey 2012. Dhaka, Bangladesh: Bangladesh Bureau of Statistics (BBS).	GHDx
317284	Bangladesh	Bangladesh EPI Coverage Evaluation Survey 2015-2016	2015 - 2016	I	I	I	I	I	I	-	-	I	I	-	Center for Social and Market Research (CSMR) (Bangladesh), Directorate General of Health Services, Ministry of Health and Family Welfare (Bangladesh), United Nations Children's Fund (UNICEF), World Health Organization (WHO). Bangladesh EPI Coverage Evaluation Survey 2015-2016.	GHDx
446877	Bangladesh	Bangladesh Demographic and Health Survey 2017-2018	2017 - 2018	I	I	I	I	I	I	I	I	I	-	-	ICF International, Mitra and Associates, National Institute of Population Research and Training (NIPORT). Bangladesh Demographic and Health Survey 2017-2018. Fairfax, United States of America: ICF International, 2020.	GHDx
1039	Belarus	Belarus Multiple Indicator Cluster Survey 2005	2005	I	I	I	I	-	I	-	-	I	-	-	Ministry of Statistics and Analysis of the Republic of Belarus, United Nations Children's Fund (UNICEF). Belarus Multiple Indicator Cluster Survey 2005. New York, United States of America: United Nations Children's Fund (UNICEF).	GHDx
1089	Belize	Belize Multiple Indicator Cluster Survey 2006	2006	I	I	I	I	I	I	-	-	I	-	-	Statistical Institute of Belize, United Nations Children's Fund (UNICEF). Belize Multiple Indicator Cluster Survey 2006. New York, United States of America: United Nations Children's Fund (UNICEF).	GHDx
76699	Belize	Belize Multiple Indicator Cluster Survey 2011	2011	I	I	I	I	I	I	-	-	I	-	-	Statistical Institute of Belize, United Nations Children's Fund (UNICEF). Belize Multiple Indicator Cluster Survey 2011. New York, United States of America: United Nations Children's Fund (UNICEF), 2013.	GHDx
215057	Belize	From Girls to Women: Growing up Healthy in Belize	1994 - 1995	I	-	I	-	-	I	-	-	I	-	-	Government of Belize. From Girls to Women: Growing up Healthy in Belize. Belmopan, Belize: Government of Belize, 1997.	GHDx

GHDx ID	Country	Series	Year	BCG	DPT1	DPT3	HepB3	Hib3	MCV1	MCV2	PCV3	Pol3	RCV1	RotaC	Citation	Link
264910	Belize	Belize Multiple Indicator Cluster Survey 2015-2016	2015 - 2016	I	I	I	I	I	I	E	-	I	-	-	Government of Belize, Statistical Institute of Belize, UN Resident Coordinator Fund (UN ResCor), United Nations Children's Fund (UNICEF), United Nations Development Programme (UNDP). Belize Multiple Indicator Cluster Survey 2015-2016. New York, United States of America: United Nations Children's Fund (UNICEF), 2018.	GHDx
18938	Benin	Benin Demographic and Health Survey 1996	1996	I	I	I	-	-	I	-	-	I	-	-	Macro International, Inc, National Institute of Statistics and Economic Analysis (INSAE) (Benin). Benin Demographic and Health Survey 1996. Fairfax, United States of America: ICF International.	GHDx
18950	Benin	Benin Demographic and Health Survey 2001	2001	I	I	I	-	-	I	-	-	I	-	-	National Institute of Statistics and Economic Analysis (INSAE) (Benin), ORC Macro. Benin Demographic and Health Survey 2001. Fairfax, United States of America: ICF International.	GHDx
18959	Benin	Benin Demographic and Health Survey 2006	2006	I	I	I	I	I	I	-	-	I	-	-	Macro International, Inc, National Institute of Statistics and Economic Analysis (INSAE) (Benin), National Program Against AIDS (PNLS) (Benin). Benin Demographic and Health Survey 2006. Fairfax, United States of America: ICF International.	GHDx
206075	Benin	Benin Multiple Indicator Cluster Survey 2014	2014	I	I	I	I	I	I	-	I	I	-	-	National Institute of Statistics and Economic Analysis (INSAE) (Benin), United Nations Children's Fund (UNICEF). Benin Multiple Indicator Cluster Survey 2014. New York, United States of America: United Nations Children's Fund (UNICEF), 2017.	GHDx
215029	Benin	Benin EPI Evaluation of Immunisation Coverage and Social Mobilisation 1991	1998	I	-	I	-	-	I	-	-	-	-	-	Benin EPI Evaluation of Immunisation Coverage and Social Mobilisation 1991.	GHDx
215030	Benin	Benin National Immunization Coverage Survey 1995	1995	I	I	I	-	-	I	-	-	I	-	-	Benin National Immunization Coverage Survey 1995.	GHDx
215031	Benin	Benin External EPI Review 2001	2001	I	I	I	-	-	I	-	-	I	-	-	Benin External EPI Review 2001.	GHDx
215032	Benin	Benin External Review of the Extended Programme on Immunisation 2008	2008	I	I	I	I	I	I	-	-	I	-	-	Benin External Review of the Extended Programme on Immunisation 2008.	GHDx
218565	Benin	Benin Demographic and Health Survey 2017-2018	2017 - 2018	I	I	I	I	I	I	-	I	I	-	-	Hubert Koutoukou Maga National University Hospital Center (CNHU-HKM)(Benin), ICF International, National Institute of Statistics and Economic Analysis (INSAE) (Benin), National Malaria Control Program, Ministry of Health (Benin), Permanent Secretariat of the Food Council and Nutrition (SP-CAN)(Benin). Benin Demographic and Health Survey 2017-2018. Fairfax, United States of America: ICF International, 2018.	GHDx
165290	Bhutan	Bhutan Health Survey 2012-2013	2012 - 2013	I	I	I	-	I	I	-	-	I	I	-	Ministry of Health (Bhutan), National Statistics Bureau (Bhutan), United Nations Population Fund (UNFPA). Bhutan Health Survey 2012-2013.	GHDx
1245	Bolivia	Bolivia Household Survey 2000	2000	-	I	I	-	-	-	-	-	-	-	-	National Institute of Statistics (Bolivia), World Bank (WB), Inter-American Development Bank (IDB), United Nations Economic Commission for Latin America and the Caribbean (CEPAL). Bolivia Household Survey 2000. La Paz, Bolivia: National Institute of Statistics (Bolivia).	GHDx
1259	Bolivia	Bolivia Household Survey 2001	2001	-	I	I	-	-	-	-	-	-	-	-	National Institute of Statistics (Bolivia), World Bank (WB), Inter-American Development Bank (IDB), United Nations Economic Commission for Latin America and the Caribbean (CEPAL). Bolivia Household Survey 2001. La Paz, Bolivia: National Institute of Statistics (Bolivia).	GHDx
1278	Bolivia	Bolivia Household Survey 2002	2002	-	I	I	-	-	-	-	-	-	-	-	National Institute of Statistics (Bolivia), World Bank (WB). Bolivia Household Survey 2002. La Paz, Bolivia: National Institute of Statistics (Bolivia).	GHDx
1289	Bolivia	Bolivia Multiple Indicator Cluster Survey 2000	2000	I	I	I	-	-	I	-	-	I	-	-	Population Development and Environment (PODEMA), National Directorate of Epidemiology (Bolivia), United Nations Children's Fund (UNICEF). Bolivia Multiple Indicator Cluster Survey 2000. New York, United States: United Nations Children's Fund (UNICEF).	GHDx
18971	Bolivia	Bolivia Demographic and Health Survey 1998	1998	I	I	I	-	-	I	-	-	I	-	-	Macro International, Inc, National Institute of Statistics (Bolivia). Bolivia Demographic and Health Survey 1998. Fairfax, United States of America: ICF International.	GHDx
18979	Bolivia	Bolivia Demographic and Health Survey 1989	1989	I	I	I	-	-	I	-	-	I	-	-	Macro Systems, Inc.; Institute for Resource Development, National Institute of Statistics (Bolivia). Bolivia Demographic and Health Survey 1989. Fairfax, United States of America: ICF International.	GHDx

GHDx ID	Country	Series	Year	BCG	DPT1	DPT3	HepB3	Hib3	MCV1	MCV2	PCV3	Pol3	RCV1	RotaC	Citation	Link
18990	Bolivia	Bolivia Demographic and Health Survey 1993-1994	1993 - 1994	I	I	I	-	-	I	-	-	I	-	-	Macro International, Inc, National Institute of Statistics (Bolivia). Bolivia Demographic and Health Survey 1993-1994. Fairfax, United States of America: ICF International.	GHDx
19001	Bolivia	Bolivia Demographic and Health Survey 2003-2004	2003 - 2004	I	I	I	-	-	I	-	-	I	-	-	Macro International, Inc, Ministry of Health and Sports (Bolivia), National Institute of Statistics (Bolivia). Bolivia Demographic and Health Survey 2003-2004. Fairfax, United States of America: ICF International.	GHDx
19016	Bolivia	Bolivia Demographic and Health Survey 2008	2008	I	I	I	I	I	I	-	-	I	-	-	Macro International, Inc, Ministry of Health and Sports (Bolivia), National Institute of Statistics (Bolivia). Bolivia Demographic and Health Survey 2008. Fairfax, United States of America: ICF International.	GHDx
32374	Bolivia	Bolivia Household Survey 2003-2004	2003 - 2004	-	I	I	-	-	-	-	-	I	-	-	National Institute of Statistics (Bolivia). Bolivia Household Survey 2003-2004. La Paz, Bolivia: National Institute of Statistics (Bolivia).	GHDx
148344	Bolivia	Bolivia Household Survey 2007	2007	-	I	I	I	I	-	-	-	I	-	-	National Institute of Statistics (Bolivia). Bolivia Household Survey 2007. La Paz, Bolivia: National Institute of Statistics (Bolivia).	GHDx
148345	Bolivia	Bolivia Household Survey 2008	2008	-	I	I	I	I	-	-	-	I	-	-	National Institute of Statistics (Bolivia). Bolivia Household Survey 2008. La Paz, Bolivia: National Institute of Statistics (Bolivia).	GHDx
148346	Bolivia	Bolivia Household Survey 2009	2009	-	I	I	I	I	-	-	-	I	-	-	National Institute of Statistics (Bolivia). Bolivia Household Survey 2009. La Paz, Bolivia: National Institute of Statistics (Bolivia).	GHDx
164634	Bolivia	Bolivia Household Survey 2011	2011	-	I	I	I	I	-	-	-	I	-	-	National Institute of Statistics (Bolivia). Bolivia Household Survey 2011. La Paz, Bolivia: National Institute of Statistics (Bolivia).	GHDx
215060	Bolivia	Bolivia National Immunization Coverage Survey 1987	1987	I	-	I	-	-	I	-	-	I	-	-	Bolivia National Immunization Coverage Survey 1987.	GHDx
215061	Bolivia	Bolivia National Immunization Coverage Survey 1990	1990	I	-	I	-	-	I	-	-	I	-	-	Bolivia National Immunization Coverage Survey 1990.	GHDx
215062	Bolivia	Bolivia Survey of Coverage by MR (15-39 Years) and MMR (Children 12-23 Months) 2006	2006	-	-	-	-	-	I	-	-	-	-	-	Bolivia Survey of Coverage by MR (15-39 Years) and MMR (Children 12-23 Months) 2006.	GHDx
323944	Bolivia	Bolivia Demographic and Health Survey 2016	2016	I	I	I	I	I	I	-	I	I	I	I	Ministry of Health (Bolivia), National Institute of Statistics (Bolivia). Bolivia Demographic and Health Survey 2016. La Paz, Bolivia: National Institute of Statistics (Bolivia), 2017.	GHDx
336885	Bolivia	Immunization: Levels, Trends and Differentials	1986 - 1989	I	I	I	-	-	I	-	-	I	-	-	Boerma, J. Ties, A. Elisabeth Sommerfelt, Shea O. Rutstein, and Guillermo Rojas. 1990. Immunization: Levels, Trends and Differentials. DHS Comparative Studies No. 1. Columbia, Maryland, USA: Institute for Resource Development.	GHDx
1385	Bosnia and Herzegovina	Bosnia and Herzegovina Multiple Indicator Cluster Survey 2006	2006	I	I	I	-	-	I	-	-	I	I	-	Directorate for Economic Planning (Bosnia and Herzegovina), Federal Office of Statistics (Federation of Bosnia and Herzegovina), Institute of Statistics (Republic of Srpska), Ministry of Health (Federation of Bosnia and Herzegovina), Ministry of Health and Social Welfare (Republic of Srpska), Public Health Institute of Federation of Bosnia and Herzegovina, United Nations Children's Fund (UNICEF). Bosnia and Herzegovina Multiple Indicator Cluster Survey 2006. New York, United States of America: United Nations Children's Fund (UNICEF).	GHDx
26989	Bosnia and Herzegovina	Bosnia and Herzegovina Multiple Indicator Cluster Survey 2000	2000	I	I	I	-	-	I	-	-	I	-	-	Agency for Statistics (Bosnia and Herzegovina), Ministry of Health (Federation of Bosnia and Herzegovina), Ministry of Health and Social Welfare (Republic of Srpska), United Nations Children's Fund (UNICEF). Bosnia and Herzegovina Multiple Indicator Cluster Survey 2000. New York, United States of America: United Nations Children's Fund (UNICEF).	GHDx
80731	Bosnia and Herzegovina	Bosnia and Herzegovina Multiple Indicator Cluster Survey 2011-2012	2011 - 2012	I	I	I	I	-	I	-	-	I	I	-	Agency for Statistics (Bosnia and Herzegovina), Federal Ministry of Health (Bosnia and Herzegovina), Ministry of Health and Social Welfare (Republic of Srpska), Public Health Institute of Federation of Bosnia and Herzegovina, United Nations Children's Fund (UNICEF), United Nations Entity for Gender Equality and the Empowerment of Women (UN Women). Bosnia and Herzegovina Multiple Indicator Cluster Survey 2011-2012.	GHDx

GHDx ID	Country	Series	Year	BCG	DPT1	DPT3	HepB3	Hib3	MCV1	MCV2	PCV3	Pol3	RCV1	RotaC	Citation	Link
1404	Botswana	Botswana Multiple Indicator Cluster Survey 2000	2000	I	I	I	-	-	I	-	-	I	-	-	Central Statistics Office (Botswana), United Nations Children's Fund (UNICEF). Botswana Multiple Indicator Cluster Survey 2000. New York, United States of America: United Nations Children's Fund (UNICEF), 2015.	GHDx
19019	Botswana	Botswana Demographic and Health Survey 1988	1988	I	I	I	-	-	I	-	-	I	-	-	Central Statistics Office (Botswana), Macro Systems, Inc.; Institute for Resource Development, Ministry of Health (Botswana). Botswana Demographic and Health Survey 1988. Fairfax, United States of America: ICF International.	GHDx
22125	Botswana	Botswana Family Health Survey 2007-2008	2007 - 2008	I	I	I	I	-	I	-	-	I	-	-	Central Statistics Office (Botswana). Botswana Family Health Survey 2007-2008. Gaborone, Botswana: Central Statistics Office (Botswana), 2009.	GHDx
151536	Botswana	Botswana Core Welfare Indicators Survey 2009-2010	2009 - 2010	-	I	I	I	-	I	-	-	I	-	-	Central Statistics Office (Botswana). Botswana Core Welfare Indicators Survey 2009-2010.	GHDx
215179	Botswana	Botswana EPI Evaluation 1987	1980 - 1987	I	I	I	-	-	I	-	-	I	-	-	Botswana EPI Evaluation 1987.	GHDx
215181	Botswana	Botswana EPI Evaluation 1990	1990	I	I	I	-	-	I	-	-	I	-	-	World Health Organization (WHO). Botswana EPI Evaluation 1990.	GHDx
215182	Botswana	Botswana EPI Coverage Survey 2007	2007	I	I	I	I	-	I	-	-	I	-	-	Ministry of Health (Botswana). Botswana EPI Coverage Survey 2007.	GHDx
19027	Brazil	Brazil Demographic and Health Survey 1986	1986	I	I	I	-	-	I	E	-	I	-	-	Brazilian Society for Family Welfare (BEMFAM), Westinghouse; Institute for Resource Development. Brazil Demographic and Health Survey 1986. Fairfax, United States: ICF International.	GHDx
19035	Brazil	Brazil Demographic and Health Survey 1991	1991	I	I	I	-	-	I	-	-	I	-	-	Brazilian Society for Family Welfare (BEMFAM), Macro International, Inc. Brazil Demographic and Health Survey 1991. Fairfax, United States: ICF International.	GHDx
19046	Brazil	Brazil Demographic and Health Survey 1996	1996	I	I	I	-	-	I	-	-	I	-	-	Brazilian Society for Family Welfare (BEMFAM), Macro International, Inc. Brazil Demographic and Health Survey 1996. Fairfax, United States of America: ICF International.	GHDx
1921	Burkina Faso	Burkina Faso Multiple Indicator Cluster Survey 1996	1996	I	-	I	-	-	I	-	-	I	-	-	Ministry of Communication and Culture (Burkina Faso), Ministry of Economy and Finance (Burkina Faso), Ministry of Health (Burkina Faso), National Institute of Statistics and Demography (Burkina Faso), United Nations Children's Fund (UNICEF). Burkina Faso Multiple Indicator Cluster Survey 1996.	GHDx
1927	Burkina Faso	Burkina Faso Multiple Indicator Cluster Survey 2006	2006	I	I	I	-	-	I	-	-	I	-	-	National Institute of Statistics and Demography (Burkina Faso), United Nations Children's Fund (UNICEF). Burkina Faso Multiple Indicator Cluster Survey 2006. New York, United States of America: United Nations Children's Fund (UNICEF).	GHDx
19064	Burkina Faso	Burkina Faso Demographic and Health Survey 1992-1993	1992 - 1993	I	I	I	-	-	I	-	-	I	-	-	Macro International, Inc, National Institute of Statistics and Demography (Burkina Faso). Burkina Faso Demographic and Health Survey 1992-1993. Fairfax, United States of America: ICF International.	GHDx
19076	Burkina Faso	Burkina Faso Demographic and Health Survey 1998-1999	1998 - 1999	I	I	I	-	-	I	-	-	I	-	-	Macro International, Inc, National Institute of Statistics and Demography (Burkina Faso). Burkina Faso Demographic and Health Survey 1998-1999. Fairfax, United States of America: ICF International.	GHDx
19088	Burkina Faso	Burkina Faso Demographic and Health Survey 2003	2003	I	I	I	-	-	I	-	-	I	-	-	Macro International, Inc, National Institute of Statistics and Demography (Burkina Faso). Burkina Faso Demographic and Health Survey 2003. Fairfax, United States of America: ICF International.	GHDx
19133	Burkina Faso	Burkina Faso Demographic and Health Survey 2010-2011	2010 - 2011	I	I	I	I	I	I	-	-	I	-	-	ICF Macro, Ministry of Health (Burkina Faso), National Institute of Statistics and Demography (Burkina Faso). Burkina Faso Demographic and Health Survey 2010-2011. Fairfax, United States of America: ICF International.	GHDx
26642	Burkina Faso	Burkina Faso Global Fund Household Health Coverage Survey 2008	2008	I	I	I	-	-	I	-	-	I	-	-	Global Fund to Fight Aids Tuberculosis and Malaria (GFATM). Burkina Faso Global Fund Household Health Coverage Survey 2008.	GHDx
215033	Burkina Faso	Burkina Faso EPI Review 2003	2003	I	I	I	-	-	I	-	-	I	-	-	Burkina Faso EPI Review 2003.	GHDx
215034	Burkina Faso	Burkina Faso EPI Review 2009	2009	I	I	I	-	-	I	-	-	I	-	-	Burkina Faso EPI Review 2009.	GHDx

GHDx ID	Country	Series	Year	BCG	DPT1	DPT3	HepB3	Hib3	MCV1	MCV2	PCV3	Pol3	RCV1	RotaC	Citation	Link
236156	Burkina Faso	Burkina Faso Continuous Multisectoral Survey 2014	2014	I	I	I	-	-	I	-	-	I	-	-	National Institute of Statistics and Demography (Burkina Faso), World Bank. Burkina Faso Continuous Multisectoral Survey 2014. Washington DC, United States of America: World Bank.	GHDx
1981	Burundi	Burundi Multiple Indicator Cluster Survey 2005	2005	I	I	I	-	-	I	-	-	I	-	-	United Nations Children's Fund (UNICEF), Burundi Institute of Statistics and Economic Studies, United Nations Population Fund (UNFPA). Burundi Multiple Indicator Cluster Survey 2005. New York, United States: United Nations Children's Fund (UNICEF).	GHDx
1994	Burundi	Burundi Multiple Indicator Cluster Survey 2000	2000	I	I	I	-	-	I	-	-	I	-	-	Burundi Institute of Statistics and Economic Studies, United Nations Children's Fund (UNICEF). Burundi Multiple Indicator Cluster Survey 2000. New York, United States: United Nations Children's Fund (UNICEF).	GHDx
19142	Burundi	Burundi Demographic and Health Survey 1987	1987	I	I	I	-	-	I	-	-	I	-	-	Population Department, Ministry of the Interior (Burundi), Westinghouse; Institute for Resource Development. Burundi Demographic and Health Survey 1987. Columbia, United States: Westinghouse; Institute for Resource Development.	GHDx
30431	Burundi	Burundi Demographic and Health Survey 2010-2011	2010 - 2011	I	I	I	I	I	I	-	-	I	-	-	Burundi Institute of Statistics and Economic Studies, ICF International, Ministry of Public Health and the Fight Against AIDS (Burundi). Burundi Demographic and Health Survey 2010-2011. Fairfax, United States of America: ICF International, 2012.	GHDx
189141	Burundi	Burundi Multiple Indicator Cluster Survey 1996	1996	I	-	I	-	-	I	-	-	I	-	-	Burundi Institute of Statistics and Economic Studies, United Nations Children's Fund (UNICEF). Burundi Multiple Indicator Cluster Survey 1996.	GHDx
215026	Burundi	Burundi Immunization Coverage Survey for Children 12-23 Months 1989	1989	I	I	I	-	-	I	-	-	I	-	-	Burundi Immunization Coverage Survey for Children 12-23 Months 1989.	GHDx
215027	Burundi	Burundi Routine Immunization Coverage Survey and Post Measles Campaign Survey 2012	2012	I	I	I	I	I	I	-	-	I	-	-	Burundi Routine Immunization Coverage Survey and Post Measles Campaign Survey 2012.	GHDx
286766	Burundi	Burundi Demographic and Health Survey 2016-2017	2016 - 2017	I	I	I	I	I	I	I	I	I	-	I	Burundi Institute of Statistics and Economic Studies, ICF International, Ministry of Public Health and the Fight Against AIDS (Burundi). Burundi Demographic and Health Survey 2016-2017. Fairfax, United States of America: ICF International, 2018.	GHDx
19156	Cambodia	Cambodia Demographic and Health Survey 2000	2000	I	I	I	-	-	I	-	-	I	-	-	Macro International, Inc, Ministry of Health (Cambodia), National Institute of Statistics (Cambodia). Cambodia Demographic and Health Survey 2000. Fairfax, United States of America: ICF International.	GHDx
19167	Cambodia	Cambodia Demographic and Health Survey 2005-2006	2005 - 2006	I	I	I	-	-	I	-	-	I	-	-	Macro International, Inc, National Institute of Public Health (Cambodia), National Institute of Statistics (Cambodia). Cambodia Demographic and Health Survey 2005-2006. Fairfax, United States of America: ICF International.	GHDx
19170	Cambodia	Cambodia Special Demographic and Health Survey 1998	1998	E	E	I	-	-	E	-	-	I	-	-	Macro International, Inc, Ministry of Health (Cambodia), National Institute of Public Health (Cambodia). Cambodia Special Demographic and Health Survey 1998. Fairfax, United States of America: ICF International.	GHDx
30379	Cambodia	Cambodia Demographic and Health Survey 2010-2011	2010 - 2011	I	I	I	I	E	I	-	-	I	-	-	ICF Macro, Ministry of Health (Cambodia), National Institute of Statistics (Cambodia). Cambodia Demographic and Health Survey 2010-2011. Fairfax, United States of America: ICF International.	GHDx
30758	Cambodia	Cambodia Socio-Economic Survey 1996	1996	I	I	I	-	-	I	-	-	-	-	-	National Institute of Statistics (Cambodia). Cambodia Socio-Economic Survey 1996. Phnom Penh, Cambodia: National Institute of Statistics (Cambodia).	GHDx
30842	Cambodia	Cambodia Socio-Economic Survey 1999	1999	E	E	I	-	-	I	-	-	I	-	-	National Institute of Statistics (Cambodia), United Nations Development Programme (UNDP), World Bank. Cambodia Socio-Economic Survey 1999. Phnom Penh, Cambodia: National Institute of Statistics (Cambodia).	GHDx
30963	Cambodia	Cambodia Socio-Economic Survey 2003-2005	2003 - 2005	I	I	I	-	-	I	-	-	I	-	-	National Institute of Statistics (Cambodia), Statistics Sweden. Cambodia Socio-Economic Survey 2003-2005. Phnom Penh, Cambodia: National Institute of Statistics (Cambodia).	GHDx
31050	Cambodia	Cambodia Socio-Economic Survey 2006-2007	2006 - 2007	I	I	I	-	-	I	-	-	I	-	-	National Institute of Statistics (Cambodia), Statistics Sweden. Cambodia Socio-Economic Survey 2006-2007. Phnom Penh, Cambodia: National Institute of Statistics (Cambodia).	GHDx
157024	Cambodia	Cambodia Demographic and Health Survey 2014	2014	I	I	I	I	I	I	-	-	I	-	-	ICF International, Ministry of Health (Cambodia), National Institute of Statistics (Cambodia). Cambodia Demographic and Health Survey 2014. Fairfax, United States of America: ICF International, 2017.	GHDx

GHDx ID	Country	Series	Year	BCG	DPT1	DPT3	HepB3	Hib3	MCV1	MCV2	PCV3	Pol3	RCV1	RotaC	Citation	Link
232764	Cambodia	Cambodia Vaccine Coverage Survey of Children 12-23 Months of Age 1995	1995	E	E	I	-	-	I	-	-	I	-	-	Cambodia Vaccine Coverage Survey of Children 12-23 Months of Age 1995.	GHDx
2039	Cameroon	Cameroon Household Survey 2001	2001	I	I	-	-	-	I	-	-	-	-	-	National Institute of Statistics (Cameroon), Directorate of Statistics and National Accounts, Ministry of Economics and Finance (Cameroon), AFRISTAT. Cameroon Household Survey 2001. Yaounde, Cameroon: National Institute of Statistics (Cameroon).	GHDx
2053	Cameroon	Cameroon Multiple Indicator Cluster Survey 2000	2000	I	I	I	-	-	I	-	-	I	-	-	Directorate of Statistics and National Accounts, Ministry of Economics and Finance (Cameroon), United Nations Children's Fund (UNICEF). Cameroon Multiple Indicator Cluster Survey 2000. New York, United States of America: United Nations Children's Fund (UNICEF).	GHDx
2063	Cameroon	Cameroon Multiple Indicator Cluster Survey 2006	2006	I	I	I	-	-	I	-	-	I	-	-	United Nations Children's Fund (UNICEF), National Institute of Statistics (Cameroon). Cameroon Multiple Indicator Cluster Survey 2006. New York, United States: United Nations Children's Fund (UNICEF).	GHDx
19188	Cameroon	Cameroon Demographic and Health Survey 1991	1991	I	I	I	-	-	I	-	-	I	-	-	Macro International, Inc, Ministry of Economy, Planning and Regional Development (Cameroon). Cameroon Demographic and Health Survey 1991. Fairfax, United States of America: ICF International.	GHDx
19198	Cameroon	Cameroon Demographic and Health Survey 1998	1998	I	I	I	-	-	I	-	-	I	-	-	Central Bureau of the Census and Population Studies (Cameroon), Macro International, Inc. Cameroon Demographic and Health Survey 1998. Fairfax, United States of America: ICF International.	GHDx
19211	Cameroon	Cameroon Demographic and Health Survey 2004	2004	I	I	I	-	-	I	-	-	I	-	-	Macro International, Inc, National Institute of Statistics (Cameroon). Cameroon Demographic and Health Survey 2004. Fairfax, United States of America: ICF International.	GHDx
19274	Cameroon	Cameroon Demographic and Health Survey 2011	2011	I	I	I	I	I	I	-	-	I	-	-	ICF International, Ministry of Economy, Planning and Regional Development (Cameroon), Ministry of Public Health (Cameroon), National Institute of Statistics (Cameroon), Pasteur Center of Cameroon. Cameroon Demographic and Health Survey 2011. Fairfax, United States of America: ICF International.	GHDx
217762	Cameroon	Cameroon Immunization Coverage Survey November-December 1990	1987 - 1990	I	I	I	-	-	I	-	-	I	-	-	Cameroon Immunization Coverage Survey November-December 1990.	GHDx
217763	Cameroon	Cameroon National Evaluation of Immunization Coverage among Children Aged 12 to 23 Months 2005	2005	I	I	I	-	-	I	-	-	I	-	-	Cameroon National Evaluation of Immunization Coverage among Children Aged 12 to 23 Months 2005.	GHDx
217764	Cameroon	Cameroon Post-Campaign Vaccination Coverage Survey 2011	2011	I	I	I	I	I	I	-	-	I	-	-	Ministry of Public Health (Cameroon), National Institute of Statistics (Cameroon). Cameroon Post-Campaign Vaccination Coverage Survey 2011.	GHDx
244455	Cameroon	Cameroon Multiple Indicator Cluster Survey 2014	2014	I	I	I	I	I	I	-	I	I	-	-	Ministry of Public Health (Cameroon), National Institute of Statistics (Cameroon), United Nations Children's Fund (UNICEF). Cameroon Multiple Indicator Cluster Survey 2014. New York, United States of America: United Nations Children's Fund (UNICEF), 2017.	GHDx
413167	Cameroon	Cameroon Demographic and Health Survey 2018-2019	2018 - 2019	I	I	I	I	I	I	-	I	I	-	I	ICF International, Ministry of Public Health (Cameroon), National Institute of Statistics (Cameroon). Cameroon Demographic and Health Survey 2018-2019. Fairfax, United States of America: ICF International.	GHDx
191172	Canada	Canada Childhood National Immunization Coverage Survey 2011	2011	-	-	I	-	-	I	-	-	I	-	-	Public Health Agency of Canada, Statistics Canada. Canada Childhood National Immunization Coverage Survey 2011.	GHDx
191908	Canada	Canadian National Report on Immunization 2006	1992 - 2004	-	-	I	-	-	I	-	-	I	-	-	Public Health Agency of Canada. Canadian National Report on Immunization 2006. Ottawa, Canada: Public Health Agency of Canada, 2006.	GHDx
311073	Canada	Canada Health System Review 2013	1980 - 2011	-	-	I	-	-	I	-	-	-	-	-	European Observatory on Health Systems and Policies, World Health Organization (WHO). Canada Health System Review 2013. Brussels, Belgium: European Observatory on Health Systems and Policies, 2013.	GHDx

GHDx ID	Country	Series	Year	BCG	DPT1	DPT3	HepB3	Hib3	MCV1	MCV2	PCV3	Pol3	RCV1	RotaC	Citation	Link
453327	Canada	Canada Childhood National Immunization Coverage Survey 2017-2018	2017 - 2018	-	-	-	I	-	I	-	I	-	-	-	Public Health Agency of Canada, Statistics Canada (StatCan). Canada Childhood National Immunization Coverage Survey 2017-2018.	GHDx
21442	Cape Verde	Cape Verde Demographic and Health Survey 2005	2005	I	I	I	I	-	I	-	-	I	-	-	Macro International, Inc, Ministry of Health (Cape Verde), National Institute of Statistics (Cape Verde). Cape Verde Demographic and Health Survey 2005.	GHDx
217914	Cape Verde	The Health of Children less than five years of age in Cape Verde, Volume I, Epidemiologic Study, 1997	1997	I	-	I	-	-	I	-	-	I	-	-	The Health of Children less than five years of age in Cape Verde, Volume I, Epidemiologic Study, 1997.	GHDx
217915	Cape Verde	Cape Verde National Immunization Coverage Survey 2000	2000	I	I	I	-	-	I	-	-	I	-	-	Ministry of Health (Cape Verde), World Health Organization (WHO). Cape Verde National Immunization Coverage Survey 2000.	GHDx
217916	Cape Verde	Cape Verde National Immunization Coverage Survey 2002	2002	I	-	I	-	-	I	-	-	I	-	-	Ministry of Health (Cape Verde), World Health Organization (WHO). Cape Verde National Immunization Coverage Survey 2002.	GHDx
217918	Cape Verde	Cape Verde National Immunization Coverage Survey 2009	2009	I	I	I	I	-	I	-	-	I	-	-	Ministry of Health (Cape Verde), World Health Organization (WHO). Cape Verde National Immunization Coverage Survey 2009.	GHDx
217920	Cape Verde	Cape Verde Immunization Coverage Survey 2011	2011	I	I	I	I	-	I	-	-	I	-	-	Ministry of Health (Cape Verde), World Health Organization (WHO). Cape Verde Immunization Coverage Survey 2011.	GHDx
2209	Central African Republic	Central African Republic Multiple Indicator Cluster Survey 2000	2000	I	I	I	-	-	I	-	-	I	-	-	Division of Statistics and Economic and Social Studies (Central African Republic), Ministry of Economy, Planning and International Cooperation (Central African Republic), United Nations Children's Fund (UNICEF). Central African Republic Multiple Indicator Cluster Survey 2000. New York, United States of America: United Nations Children's Fund (UNICEF).	GHDx
2223	Central African Republic	Central African Republic Multiple Indicator Cluster Survey 2006	2006	I	I	I	-	-	I	-	-	I	-	-	United Nations Children's Fund (UNICEF). Central African Republic Multiple Indicator Cluster Survey 2006. New York, United States: United Nations Children's Fund (UNICEF).	GHDx
19292	Central African Republic	Central African Republic Demographic and Health Survey 1994-1995	1994 - 1995	I	I	I	-	-	I	-	-	I	-	-	Division of Statistics and Economic and Social Studies (Central African Republic), Macro International, Inc. Central African Republic Demographic and Health Survey 1994-1995. Fairfax, United States of America: ICF International.	GHDx
82832	Central African Republic	Central African Republic Multiple Indicator Cluster Survey 2010-2011	2010 - 2011	I	I	I	I	-	I	-	-	I	-	-	Central African Institute of Statistics, Economic and Social Studies (ICASEES) (Central African Republic), ICF International. Central African Republic Multiple Indicator Cluster Survey 2010-2011. Fairfax, United States of America: ICF International, 2013.	GHDx
215186	Central African Republic	Central African Republic Immunization Coverage Survey February 1991	1989 - 1991	I	I	I	-	-	I	-	-	I	-	-	Central African Republic Immunization Coverage Survey February 1991.	GHDx
215187	Central African Republic	Central African Republic EPI Review 1993	1993	I	I	I	-	-	I	-	-	I	-	-	Central African Republic EPI Review 1993.	GHDx
215189	Central African Republic	Central African Republic External EPI Review 2002	2002	I	I	I	-	-	I	-	-	I	-	-	Government of Central African Republic, United Nations Children's Fund (UNICEF), World Health Organization (WHO). Central African Republic External EPI Review 2002.	GHDx
215247	Central African Republic	Central African Republic EPI Coverage Survey 2012	2012	I	-	I	I	I	I	-	-	E	-	-	Ministry of Public Health, Population, and AIDS Control (Central African Republic). Central African Republic EPI Coverage Survey 2012.	GHDx
2244	Chad	Chad Multiple Indicator Cluster Survey 2000	2000	I	I	I	-	-	I	-	-	I	-	-	United Nations Children's Fund (UNICEF), Census Bureau (Chad), National Institute of Statistical, Economic and Demographic Studies (Chad). Chad Multiple Indicator Cluster Survey 2000. New York, United States: United Nations Children's Fund (UNICEF).	GHDx
19305	Chad	Chad Demographic and Health Survey 1996-1997	1996 - 1997	I	I	I	-	-	I	-	-	I	-	-	Census Bureau (Chad), Macro International, Inc, National Institute for Statistics, Economic and Demographic Studies (INSEED) (Chad). Chad Demographic and Health Survey 1996-1997. Fairfax, United States of America: ICF International.	GHDx

GHDx ID	Country	Series	Year	BCG	DPT1	DPT3	HepB3	Hib3	MCV1	MCV2	PCV3	Pol3	RCV1	RotaC	Citation	Link
19315	Chad	Chad Demographic and Health Survey 2004	2004	I	I	I	-	-	I	-	-	I	-	-	Macro International, Inc, National Institute for Statistics, Economic and Demographic Studies (INSEED) (Chad). Chad Demographic and Health Survey 2004. Fairfax, United States of America: ICF International.	GHDx
76701	Chad	Chad Multiple Indicator Cluster Survey 2010	2010	I	I	I	I	-	I	-	-	I	-	-	Ministry of Planning, Economy, and International Cooperation (Chad), National Institute for Statistics, Economic and Demographic Studies (INSEED) (Chad), United Nations Children's Fund (UNICEF). Chad Multiple Indicator Cluster Survey 2010. New York, United States of America: United Nations Children's Fund (UNICEF), 2014.	GHDx
157025	Chad	Chad Demographic and Health Survey 2014-2015	2014 - 2015	I	I	I	I	I	I	-	-	I	-	-	ICF International, National Institute for Statistics, Economic and Demographic Studies (INSEED) (Chad). Chad Demographic and Health Survey 2014-2015. Fairfax, United States of America: ICF International, 2016.	GHDx
229490	Chad	Chad Vaccination Coverage Survey 2012	2012	I	I	I	I	I	I	-	-	I	-	-	Chad Vaccination Coverage Survey 2012.	GHDx
229566	Chad	Chad EPI Review 2002	2002	I	I	I	-	-	I	-	-	I	-	-	Ministry of Public Health (Chad). Chad EPI Review 2002.	GHDx
229585	Chad	Chad National Immunization Coverage Survey 1990	1990	I	-	I	-	-	I	-	-	I	-	-	Chad National Immunization Coverage Survey 1990.	GHDx
438586	Chad	Chad Vaccine Coverage Survey 2017	2017	I	I	I	I	I	I	-	-	I	-	-	World Health Organization (WHO). Chad Vaccine Coverage Survey 2017.	GHDx
68803	China	China National Health Services Survey 2008	2008	I	-	I	I	-	I	-	-	I	-	-	Ministry of Health (China). China National Health Services Survey 2008.	GHDx
68809	China	China National Health Services Survey 2003	2003	I	-	I	I	-	I	-	-	I	-	-	Ministry of Health (China). China National Health Services Survey 2003.	GHDx
194012	China	China Multiple Indicator Cluster Survey 1995	1995	I	-	I	-	-	I	-	-	I	-	-	State Statistical Bureau (China), United Nations Children's Fund (UNICEF). China Multiple Indicator Cluster Survey 1995.	GHDx
217077	China	The EPI Programme in The People's Republic of China 1986	1985	I	-	I	-	-	I	-	-	I	-	-	The EPI Programme in The People's Republic of China 1986.	GHDx
19324	Colombia	Colombia Demographic and Health Survey 2004-2005	2004 - 2005	I	I	I	I	I	I	-	-	I	I	-	Macro International, Inc, Profamilia (Colombia). Colombia Demographic and Health Survey 2004-2005. Fairfax, United States of America: ICF International, 2005.	GHDx
19333	Colombia	Colombia Demographic and Health Survey 1986	1986	I	I	I	-	-	I	-	-	I	-	-	Ministry of Health (Colombia), Regional Population Center (Colombia), Westinghouse; Institute for Resource Development. Colombia Demographic and Health Survey 1986. Fairfax, United States: ICF International, 1988.	GHDx
19341	Colombia	Colombia Demographic and Health Survey 1990	1990	I	I	I	-	-	I	-	-	I	-	-	Institute for Resource Development, Macro Systems, Profamilia (Colombia). Colombia Demographic and Health Survey 1990. Fairfax, United States: ICF International, 1991.	GHDx
19350	Colombia	Colombia Demographic and Health Survey 1995	1995	I	I	I	-	-	I	-	-	I	-	-	Macro International, Inc, Profamilia (Colombia). Colombia Demographic and Health Survey 1995. Fairfax, United States: ICF International, 1995.	GHDx
19359	Colombia	Colombia Demographic and Health Survey 2000	2000	I	I	I	I	I	I	-	-	I	I	-	Macro International, Inc, Profamilia (Colombia). Colombia Demographic and Health Survey 2000. Fairfax, United States of America: ICF International, 2000.	GHDx
21281	Colombia	Colombia Demographic and Health Survey 2009-2010	2009 - 2010	I	I	I	I	I	I	-	-	I	I	-	ICF Macro, Profamilia (Colombia). Colombia Demographic and Health Survey 2009-2010. Fairfax, United States of America: ICF International, 2011.	GHDx
3114	Comoros	Comoros Multiple Indicator Cluster Survey 2000	2000	I	I	I	-	-	I	-	-	I	-	-	United Nations Development Programme (UNDP), United Nations Children's Fund (UNICEF). Comoros Multiple Indicator Cluster Survey 2000. New York, United States: United Nations Children's Fund (UNICEF).	GHDx
19370	Comoros	Comoros Demographic and Health Survey 1996	1996	I	I	I	-	-	I	-	-	I	-	-	Macro International, Inc, National Centre of Documentation and Scientific Research (Comoros). Comoros Demographic and Health Survey 1996. Fairfax, United States of America: ICF International.	GHDx
76850	Comoros	Comoros Demographic and Health Survey 2012-2013	2012 - 2013	I	I	I	I	I	I	-	-	I	-	-	General Directorate of Statistics and Forecasting (Comoros), ICF International. Comoros Demographic and Health Survey 2012-2013. Fairfax, United States of America: ICF International.	GHDx

GHDx ID	Country	Series	Year	BCG	DPT1	DPT3	HepB3	Hib3	MCV1	MCV2	PCV3	Pol3	RCV1	RotaC	Citation	Link
217775	Comoros	Comoros Immunization Coverage Survey November 2007	2007	I	I	I	I	-	I	-	-	-	-	-	Comoros Immunization Coverage Survey November 2007.	GHDx
217776	Comoros	Comoros Immunization Coverage Survey October 2010	2010	I	I	I	I	-	I	-	-	I	-	-	Comoros Immunization Coverage Survey October 2010.	GHDx
217777	Comoros	Comoros Post Measles Campaign and Routine Immunization Coverage Survey 2013	2013	I	I	I	-	-	I	-	-	I	-	-	Comoros Post Measles Campaign and Routine Immunization Coverage Survey 2013.	GHDx
447887	Comoros	Comoros Post Measles Campaign and Routine Immunization Coverage Survey 2016	2016	-	-	-	-	-	I	-	-	-	-	-	United Nations Children's Fund (UNICEF), World Health Organization (WHO). Comoros Post Measles Campaign and Routine Immunization Coverage Survey 2016.	GHDx
19391	Congo (Brazzaville)	Congo Demographic and Health Survey 2005	2005	I	I	I	-	-	I	-	-	I	-	-	Macro International, Inc, National Center for Statistics and Economic Studies (Congo, Rep.). Congo Demographic and Health Survey 2005. Fairfax, United States of America: ICF International.	GHDx
56151	Congo (Brazzaville)	Congo Demographic and Health Survey 2011-2012	2011 - 2012	I	I	I	I	I	I	-	-	I	-	-	ICF International, Ministry of Health (Congo, Rep.), National Center for Statistics and Economic Studies (Congo, Rep.). Congo Demographic and Health Survey 2011-2012. Fairfax, United States of America: ICF International.	GHDx
217771	Congo (Brazzaville)	Congo EPI Review 1988	1985	I	I	I	-	-	I	-	-	I	-	-	Congo EPI Review 1988.	GHDx
217772	Congo (Brazzaville)	Congo EPI Review 1987	1987	I	I	I	-	-	I	-	-	I	-	-	Congo EPI Review 1987.	GHDx
217773	Congo (Brazzaville)	Congo International EPI Review 1990	1990	I	I	I	-	-	I	-	-	I	-	-	Congo International EPI Review 1990.	GHDx
217774	Congo (Brazzaville)	Congo External EPI Review 2010	2010	I	I	I	E	E	I	-	-	I	-	-	Congo External EPI Review 2010.	GHDx
234733	Congo (Brazzaville)	Congo Multiple Indicator Cluster Survey 2014-2015	2014 - 2015	I	I	I	I	-	I	-	-	I	-	-	National Institute of Statistics (INS) (Congo, Rep.), United Nations Children's Fund (UNICEF). Congo Multiple Indicator Cluster Survey 2014-2015. New York, United States of America: United Nations Children's Fund (UNICEF), 2018.	GHDx
27638	Costa Rica	Costa Rica Reproductive Health Survey 1992-1993	1992 - 1993	I	I	I	-	-	I	-	-	I	-	-	Costa Rica Social Security Institute and Division of Reproductive Health-Centers for Disease Control and Prevention (CDC). Costa Rica 1993 Reproductive Health Survey. Atlanta, United States: Centers for Disease Control and Prevention (CDC).	GHDx
125596	Costa Rica	Costa Rica Multiple Indicator Cluster Survey 2011	2011	I	I	I	I	I	I	-	I	I	-	-	Costa Rican Demographic Association, Ministry of Health (Costa Rica), United Nations Children's Fund (UNICEF). Costa Rica Multiple Indicator Cluster Survey 2011. New York, United States of America: United Nations Children's Fund (UNICEF), 2013.	GHDx
18519	Côte d'Ivoire	Côte d'Ivoire Demographic and Health Survey 1994	1994	I	I	I	-	-	I	-	-	I	-	-	Macro International, Inc, National Institute of Statistics (Côte d'Ivoire). Côte d'Ivoire Demographic and Health Survey 1994. Fairfax, United States of America: ICF International.	GHDx
18531	Côte d'Ivoire	Côte d'Ivoire Demographic and Health Survey 1998-1999	1998 - 1999	I	I	I	-	-	I	-	-	I	-	-	Macro International, Inc, National Institute of Statistics (Côte d'Ivoire). Côte d'Ivoire Demographic and Health Survey 1998-1999. Fairfax, United States of America: ICF International.	GHDx
18533	Côte d'Ivoire	Côte d'Ivoire Demographic and Health Survey 2011-2012	2011 - 2012	I	I	I	I	I	I	-	-	I	-	-	ICF International, Ministry of the Fight Against AIDS (Côte d'Ivoire), National Institute of Statistics (Côte d'Ivoire). Côte d'Ivoire Demographic and Health Survey 2011-2012. Fairfax, United States of America: ICF International.	GHDx
26433	Côte d'Ivoire	Côte d'Ivoire Multiple Indicator Cluster Survey 2006	2006	I	I	I	I	-	I	-	-	I	-	-	United Nations Children's Fund (UNICEF), National Institute of Statistics (Côte d'Ivoire). Côte d'Ivoire Multiple Indicator Cluster Survey 2006. New York, United States: United Nations Children's Fund (UNICEF).	GHDx
26444	Côte d'Ivoire	Côte d'Ivoire Multiple Indicator Cluster Survey 2000	2000	I	-	I	-	-	I	-	-	I	-	-	National School for Statistics and Economics Applied (ENSEA), United Nations Children's Fund (UNICEF), United Nations Educational, Scientific and Cultural Organization (UNESCO). Côte d'Ivoire Multiple Indicator Cluster Survey 2000. New York, United States of America: United Nations Children's Fund (UNICEF).	GHDx

GHDx ID	Country	Series	Year	BCG	DPT1	DPT3	HepB3	Hib3	MCV1	MCV2	PCV3	Pol3	RCV1	RotaC	Citation	Link
26445	Côte d'Ivoire	Côte d'Ivoire Multiple Indicator Cluster Survey 1996	1996	I	I	I	-	-	I	-	-	I	-	-	Ministry of Planning and Industrial Development (Côte d'Ivoire), National Statistics Institute (Côte d'Ivoire), United Nations Children's Fund (UNICEF). Côte d'Ivoire Multiple Indicator Cluster Survey 1996.	GHDx
217174	Côte d'Ivoire	Côte d'Ivoire Immunization Coverage Survey February 1991	1991	-	I	I	-	-	I	-	-	I	-	-	Côte d'Ivoire Immunization Coverage Survey Feburary 1991.	GHDx
217175	Côte d'Ivoire	Côte d'Ivoire External EPI Review 2001	2001	I	I	I	-	-	I	-	-	I	-	-	Côte d'Ivoire External EPI Review 2001.	GHDx
217176	Côte d'Ivoire	Côte d'Ivoire External EPI Review 2010	2010	I	I	I	I	-	I	-	-	I	-	-	Côte d'Ivoire External EPI Review 2010.	GHDx
217760	Côte d'Ivoire	Côte d'Ivoire Vaccination Coverage Survey 2013	2013	I	I	I	I	I	I	-	-	I	-	-	Côte d'Ivoire Vaccination Coverage Survey 2013.	GHDx
218611	Côte d'Ivoire	Côte d'Ivoire Multiple Indicator Cluster Survey 2016	2016	I	E	I	I	I	I	-	I	I	-	E	National Institute of Statistics (Côte d'Ivoire), United Nations Children's Fund (UNICEF). Côte d'Ivoire Multiple Indicator Cluster Survey 2016. New York, United States of America: United Nations Children's Fund (UNICEF), 2018.	GHDx
446845	Côte d'Ivoire	Côte d'Ivoire Measles Vaccination Campaign Evaluation 2014-2015	2014 - 2015	I	I	I	I	I	I	-	-	I	-	-	Ministry of Health and the Fight against AIDS (CÃƒÂ´te d'Ivoire), World Health Organization (WHO). CÃƒÂ´te d'Ivoire Measles Vaccination Campaign Evaluation 2014-2015.	GHDx
3310	Cuba	Cuba Multiple Indicator Cluster Survey 2006	2006	I	I	I	-	-	I	-	-	-	I	-	United Nations Children's Fund (UNICEF), National Bureau of Statistics (Cuba). Cuba Multiple Indicator Cluster Survey 2006. New York, United States: United Nations Children's Fund (UNICEF).	GHDx
60935	Cuba	Cuba Multiple Indicator Cluster Survey 2010-2011	2010 - 2011	I	I	I	I	I	I	-	-	-	I	-	Ministry of Public Health (Cuba), United Nations Children's Fund (UNICEF). Cuba Multiple Indicator Cluster Survey 2010-2011. New York, United States of America: United Nations Children's Fund (UNICEF).	GHDx
169975	Cuba	Cuba Multiple Indicator Cluster Survey 2014	2014	I	I	I	I	I	I	-	-	-	-	-	Ministry of Public Health (Cuba), National Office of Statistics (Cuba), United Nations Children's Fund (UNICEF). Cuba Multiple Indicator Cluster Survey 2014. New York, United States of America: United Nations Children's Fund (UNICEF), 2005.	GHDx
217922	Cyprus	Cyprus Children's Immunization Survey 2009	2009	-	I	I	I	I	I	-	I	I	-	-	Ministry of Health (Cyprus). Cyprus Children's Immunization Survey 2009.	GHDx
453332	Cyprus	Cyprus Child Immunization Survey 2019	2019	-	I	I	I	I	I	-	-	-	-	-	Ministry of Health (Cyprus). Cyprus Child Immunization Survey 2019.	GHDx
3379	Djibouti	Djibouti Immunization, Diarrhoeal Disease, Maternal and Child Mortality Survey 1989	1989	I	I	I	-	-	I	-	-	I	-	-	United Nations Children's Fund (UNICEF). Djibouti Immunization, Diarrhoeal Disease, Maternal and Child Mortality Survey 1989.	GHDx
3392	Djibouti	Djibouti Family Health Survey 2002	2002	E	I	I	-	-	I	-	-	I	-	-	Department of Statistics and Demographic Studies (Djibouti), League of Arab States, Ministry of Health (Djibouti), Pan Arab Project for Family Health (PAPFAM). Djibouti Family Health Survey 2002.	GHDx
3404	Djibouti	Djibouti Multiple Indicator Cluster Survey 2006	2006	I	-	I	-	-	I	-	-	I	-	-	Ministry of Economy, Finance, and Planning in charge of Privatization (Djibouti), Ministry of Health (Djibouti), United Nations Children's Fund (UNICEF). Djibouti Multiple Indicator Cluster Survey 2006. New York, United States of America: United Nations Children's Fund (UNICEF).	GHDx
218033	Djibouti	Djibouti Immunization Coverage Survey 2008	2008	I	I	I	I	I	I	-	-	I	-	-	Ministry of Health (Djibouti). Djibouti Immunization Coverage Survey 2008.	GHDx
218035	Djibouti	Djibouti Family Health Survey 2012	2012	I	I	I	I	I	I	E	-	I	-	-	Department of Statistics and Demographic Studies (Djibouti), League of Arab States, Ministry of Health (Djibouti), Pan Arab Project for Family Health (PAPFAM). Djibouti Family Health Survey 2012.	GHDx
3441	Dominican Republic	Dominican Republic National Multipurpose Household Survey 2007	2007	-	-	-	-	-	I	-	-	-	-	-	National Statistics Office (Dominican Republic). Dominican Republic National Multipurpose Household Survey 2007. Santo Domingo, Dominican Republic: National Statistics Office (Dominican Republic).	GHDx
3455	Dominican Republic	Dominican Republic National Multipurpose Household Survey 2006	2006	I	I	I	I	I	I	E	-	E	I	-	National Statistics Office (Dominican Republic), United Nations Children's Fund (UNICEF). Dominican Republic National Multipurpose Household Survey 2006. Santo Domingo, Dominican Republic: National Statistics Office (Dominican Republic).	GHDx

GHDx ID	Country	Series	Year	BCG	DPT1	DPT3	HepB3	Hib3	MCV1	MCV2	PCV3	Pol3	RCV1	RotaC	Citation	Link
3465	Dominican Republic	Dominican Republic Multiple Indicator Cluster Survey 2006	2006	I	I	I	-	-	I	-	-	I	-	-	National Statistics Office (Dominican Republic), United Nations Children's Fund (UNICEF). Dominican Republic Multiple Indicator Cluster Survey 2006.	GHDx
19400	Dominican Republic	Dominican Republic Demographic and Health Survey 1986	1986	I	I	I	-	-	I	-	-	I	-	-	National Council for Population and Family (Dominican Republic), Westinghouse; Institute for Resource Development. Dominican Republic Demographic and Health Survey 1986. Fairfax, United States: ICF International.	GHDx
19410	Dominican Republic	Dominican Republic Demographic and Health Survey 1991	1991	I	I	I	-	-	I	-	-	I	-	-	Institute for Resource Development, Macro Systems, National Planning Office (Dominican Republic), Profamilia (Colombia). Dominican Republic Demographic and Health Survey 1991. Fairfax, United States of America: ICF International.	GHDx
19421	Dominican Republic	Dominican Republic Demographic and Health Survey 1996	1996	I	I	I	-	-	I	-	-	I	-	-	Center for Social and Demographic Studies (Dominican Republic) (CESDEM), Macro International, Inc, National Planning Office (Dominican Republic), Profamilia (Colombia). Dominican Republic Demographic and Health Survey 1996. Fairfax, United States of America: ICF International.	GHDx
19431	Dominican Republic	Dominican Republic Experimental Demographic and Health Survey 1999	1999	I	I	I	-	-	I	-	-	I	-	-	Center for Social and Demographic Studies (Dominican Republic) (CESDEM), Macro International, Inc. Dominican Republic Experimental Demographic and Health Survey 1999. Fairfax, United States of America: ICF International.	GHDx
19444	Dominican Republic	Dominican Republic Demographic and Health Survey 2002	2002	I	I	I	I	I	I	-	-	I	-	-	Center for Social and Demographic Studies (Dominican Republic) (CESDEM), Macro International, Inc. Dominican Republic Demographic and Health Survey 2002. Fairfax, United States of America: ICF International.	GHDx
19456	Dominican Republic	Dominican Republic Demographic and Health Survey 2007	2007	I	I	I	I	I	I	-	-	I	I	-	Center for Social and Demographic Studies (Dominican Republic) (CESDEM), Macro International, Inc. Dominican Republic Demographic and Health Survey 2007. Fairfax, United States of America: ICF International.	GHDx
21198	Dominican Republic	Dominican Republic Special Demographic and Health Survey 2007	2007	I	I	I	-	-	I	-	-	I	-	-	Center for Social and Demographic Studies (Dominican Republic) (CESDEM), Macro International, Inc. Dominican Republic Special Demographic and Health Survey 2007. Fairfax, United States of America: ICF International.	GHDx
27069	Dominican Republic	Dominican Republic Multiple Indicator Cluster Survey 2000	2000	I	I	I	-	-	E	-	-	I	-	-	Center for Social and Demographic Studies (Dominican Republic) (CESDEM), United Nations Children's Fund (UNICEF). Dominican Republic Multiple Indicator Cluster Survey 2000. New York, United States of America: United Nations Children's Fund (UNICEF).	GHDx
65416	Dominican Republic	Dominican Republic National Multipurpose Household Survey 2009-2010	2009 - 2010	I	I	I	-	-	I	-	-	I	-	-	International Labour Organization (ILO), National Statistics Office (Dominican Republic), United Nations Children's Fund (UNICEF). Dominican Republic National Multipurpose Household Survey 2009-2010. 2011.	GHDx
77819	Dominican Republic	Dominican Republic Demographic and Health Survey 2013	2013	I	I	I	I	I	I	-	-	I	-	I	Center for Social and Demographic Studies (Dominican Republic) (CESDEM), ICF International, Ministry of Public Health and Social Assistance (Dominican Republic). Dominican Republic Demographic and Health Survey 2013. Fairfax, United States of America: ICF International, 2014.	GHDx
165645	Dominican Republic	Dominican Republic Special Demographic and Health Survey 2013	2013	I	I	I	-	-	I	-	-	I	-	-	Center for Social and Demographic Studies (Dominican Republic) (CESDEM), ICF International, National Public Health Laboratory (Dominican Republic). Dominican Republic Special Demographic and Health Survey 2013. Fairfax, United States of America: ICF International, 2015.	GHDx
200697	Dominican Republic	Dominican Republic Multiple Indicator Cluster Survey 2014	2014	-	I	I	I	I	I	-	-	I	I	-	National Statistics Office (Dominican Republic), United Nations Children's Fund (UNICEF). Dominican Republic Multiple Indicator Cluster Survey 2014. New York, United States of America: United Nations Children's Fund (UNICEF), 2016.	GHDx
3161	DR Congo	Democratic Republic of the Congo Multiple Indicator Cluster Survey 2001	2001	I	I	I	-	-	I	-	-	I	-	-	Ministry of Planning and Reconstruction (Congo, DR), United Nations Children's Fund (UNICEF). Congo, DR Multiple Indicator Cluster Survey 2001. New York, United States: United Nations Children's Fund (UNICEF).	GHDx
19381	DR Congo	Democratic Republic of the Congo Demographic and Health Survey 2007	2007	I	I	I	-	-	I	-	-	I	-	-	Macro International, Inc, Ministry of Planning (Congo, DR). Democratic Republic of the Congo Demographic and Health Survey 2007. Fairfax, United States of America: ICF International.	GHDx

GHDx ID	Country	Series	Year	BCG	DPT1	DPT3	HepB3	Hib3	MCV1	MCV2	PCV3	Pol3	RCV1	RotaC	Citation	Link
22308	DR Congo	Zaire Immunization Survey 1991	1991	I	I	I	-	-	I	-	-	I	-	-	Expanded Program on Immunization and Childhood Disease Control (Zaire), Centers for Disease Control and Prevention (CDC), Rotary International, United Nations Children's Fund (UNICEF), World Health Organization (WHO). Zaire Immunization Survey 1991.	GHDx
26998	DR Congo	Democratic Republic of the Congo Multiple Indicator Cluster Survey 2010	2010	I	I	I	I	-	I	-	-	I	-	-	National Statistical Institute (Congo, DR), Ministry of Planning (Congo, DR), United Nations Children's Fund (UNICEF). Congo, DR Multiple Indicator Cluster Survey 2010. New York, United States: United Nations Children's Fund (UNICEF).	GHDx
76878	DR Congo	Democratic Republic of the Congo Demographic and Health Survey 2013-2014	2013 - 2014	I	I	I	I	I	I	-	-	I	-	-	ICF International, Ministry of Planning and Monitoring Implementation of the Revolution of Modernity (Congo, DR), Ministry of Public Health (Congo, DR), National Institute of Statistics (Congo, DR). Democratic Republic of the Congo Demographic and Health Survey 2013-2014. Fairfax, United States of America: ICF International, 2014.	GHDx
217767	DR Congo	Democratic Republic of the Congo Immunization Coverage Survey 2012	2012	I	I	I	I	I	I	-	-	I	-	-	Congo, DR Immunization Coverage Survey 2012.	GHDx
437955	DR Congo	Democratic Republic of the Congo Multiple Indicator Cluster Survey 2017-2018	2017 - 2018	I	I	I	I	I	I	-	I	I	-	-	National Statistical Institute (Congo, DR), Ministry of Planning (Congo, DR), United Nations Children's Fund (UNICEF). Congo, DR Multiple Indicator Cluster Survey 2010. New York, United States: United Nations Children's Fund (UNICEF).	GHDx
27615	Ecuador	Ecuador Reproductive Health Survey 1994	1994	I	I	I	-	-	I	-	-	I	-	-	Center for Studies of Population and Social Development (CEPAR), Division of Reproductive Health-Centers for Disease Control and Prevention (CDC). (1995) Ecuador Reproductive Health Survey 1994. Atlanta, United States: Centers for Disease Control and Prevention (CDC).	GHDx
27621	Ecuador	Ecuador Reproductive Health Survey 1999	1999	I	I	I	-	-	I	-	-	I	-	-	Center for Studies of Population and Social Development (CEPAR) (Ecuador), Division of Reproductive Health-Centers for Disease Control and Prevention (CDC). Ecuador Reproductive Health Survey 1999. Atlanta, United States: Centers for Disease Control and Prevention (CDC), 2001.	GHDx
27630	Ecuador	Ecuador Reproductive Health Survey 2004	2004	I	I	I	-	-	E	-	-	I	-	-	Center for Studies of Population and Social Development (CEPAR) (Ecuador) and Division of Reproductive Health-Centers for Disease Control and Prevention (CDC). (2005) Ecuador Reproductive Health Survey 2004. Quito, Ecuador: CEPAR.	GHDx
46804	Ecuador	Ecuador Living Standards Measurement Survey 1994	1994	I	I	I	-	-	-	-	-	I	-	-	Ecuadorian Professional Training Service (SECAP), World Bank. Ecuador Living Standards Measurement Survey 1994. Washington DC, United States: World Bank.	GHDx
46837	Ecuador	Ecuador Living Standards Measurement Survey 1995	1995	I	I	I	-	-	-	-	-	I	-	-	National Institute of Statistics and Censuses (Ecuador), World Bank. Ecuador Living Standards Measurement Survey 1995.	GHDx
46924	Ecuador	Ecuador Living Conditions Survey 2005-2006	2005 - 2006	I	I	I	I	I	I	-	-	I	-	-	National Institute of Statistics and Censuses (Ecuador), Inter-American Development Bank (IDB). Ecuador Living Conditions Survey 2005-2006. Quito, Ecuador: National Institute of Statistics and Censuses (Ecuador).	GHDx
153674	Ecuador	Ecuador National Health and Nutrition Survey 2012	2012	I	I	I	I	I	I	-	I	E	I	I	Ministry of Public Health (Ecuador), National Institute of Statistics and Censuses (Ecuador). Ecuador National Health and Nutrition Survey 2012.	GHDx
364181	Ecuador	Ecuador Living Conditions Survey 2013-2014	2013 - 2014	-	I	I	I	I	-	-	I	I	-	I	National Institute of Statistics and Censuses (Ecuador). Ecuador Living Conditions Survey 2013-2014. Quito, Ecuador: National Institute of Statistics and Censuses (Ecuador).	GHDx
3583	Egypt	Egypt Maternal and Child Health Survey 1991	1991	I	I	I	-	-	I	-	-	I	-	-	Central Agency for Public Mobilization and Statistics (CAPMAS) (Egypt), League of Arab States. Egypt Maternal and Child Health Survey 1991.	GHDx
3585	Egypt	Egypt Multiple Indicator Cluster Survey 1996	1996	I	-	I	I	-	I	-	-	I	-	-	Social Research Centre, American University in Cairo and United Nations Children's Fund (UNICEF). Egypt Multiple Indicator Cluster Survey 1996. New York, United States: United Nations Children's Fund (UNICEF).	GHDx
19472	Egypt	Egypt Demographic and Health Survey 1988-1989	1988 - 1989	I	I	I	-	-	I	-	-	I	-	-	Central Agency for Public Mobilization and Statistics (CAPMAS) (Egypt), Macro Systems, Inc.; Institute for Resource Development, Population Council (Egypt). Egypt Demographic and Health Survey 1988-1989. Fairfax, United States of America: ICF International.	GHDx
19482	Egypt	Egypt Demographic and Health Survey 1992-1993	1992 - 1993	I	I	I	E	-	I	-	-	I	-	-	Macro International, Inc, Population Council (Egypt). Egypt Demographic and Health Survey 1992-1993. Fairfax, United States of America: ICF International.	GHDx

GHDx ID	Country	Series	Year	BCG	DPT1	DPT3	HepB3	Hib3	MCV1	MCV2	PCV3	Pol3	RCV1	RotaC	Citation	Link
19493	Egypt	Egypt Demographic and Health Survey 1995-1996	1995 - 1996	I	I	I	I	-	I	-	-	I	-	-	Macro International, Inc, Population Council (Egypt). Egypt Demographic and Health Survey 1995-1996. Fairfax, United States of America: ICF International.	GHDx
19501	Egypt	Egypt Interim Demographic and Health Survey 1997-1998	1997 - 1998	I	I	I	I	-	I	-	-	I	-	-	El-Zanaty and Associates, Macro International, Inc. Egypt Interim Demographic and Health Survey 1997-1998.	GHDx
19511	Egypt	Egypt Demographic and Health Survey 2000	2000	I	I	I	I	-	I	-	-	I	-	-	Macro International, Inc, Population Council (Egypt). Egypt Demographic and Health Survey 2000. Fairfax, United States of America: ICF International.	GHDx
19521	Egypt	Egypt Demographic and Health Survey 2005	2005	I	I	I	I	-	I	-	-	I	I	-	El-Zanaty and Associates, Macro International, Inc, Ministry of Health and Population (Egypt), Population Council (Egypt). Egypt Demographic and Health Survey 2005. Fairfax, United States of America: ICF International.	GHDx
19529	Egypt	Egypt Interim Demographic and Health Survey 2003	2003	I	I	I	I	-	I	-	-	I	I	-	El-Zanaty and Associates, Macro International, Inc, Ministry of Health and Population (Egypt), Population Council (Egypt). Egypt Interim Demographic and Health Survey 2003. Fairfax, United States of America: ICF International.	GHDx
26842	Egypt	Egypt Demographic and Health Survey 2008	2008	I	I	I	I	-	I	-	-	I	I	-	El-Zanaty and Associates, Macro International, Inc, Ministry of Health and Population (Egypt). Egypt Demographic and Health Survey 2008. Fairfax, United States of America: ICF International.	GHDx
154897	Egypt	Egypt Demographic and Health Survey 2014	2014	I	I	I	I	E	I	I	-	I	I	-	El-Zanaty and Associates, ICF International, Ministry of Health and Population (Egypt). Egypt Demographic and Health Survey 2014. Fairfax, United States of America: ICF International.	GHDx
224872	Egypt	Egypt National Cluster Survey for Vaccination Coverage 1984	1984	I	I	I	-	-	I	-	-	I	-	-	Egypt National Cluster Survey for Vaccination Coverage 1984.	GHDx
224874	Egypt	Egypt National Cluster Survey for Vaccination Coverage 1987	1987	I	I	I	-	-	I	-	-	I	-	-	United Nations Children's Fund (UNICEF). Egypt National Cluster Survey for Vaccination Coverage 1987.	GHDx
224903	Egypt	Egypt Expanded Programme on Immunization Survey 1991	1991	I	I	I	-	-	I	-	-	I	-	-	Ministry of Health and Population (Egypt), United Nations Children's Fund (UNICEF). Egypt Expanded Programme on Immunization Survey 1991.	GHDx
27572	El Salvador	El Salvador Family Planning/Maternal and Child Health Survey 1988	1988	I	-	I	-	-	I	-	-	I	-	-	Division of Reproductive Health-Centers for Disease Control and Prevention (CDC). El Salvador Family Planning/Maternal and Child Health Survey 1988. Final English Language Report. Atlanta, United States: Centers for Disease Control and Prevention (CDC).	GHDx
27590	El Salvador	El Salvador Reproductive Health Survey 1998	1998	I	I	I	-	-	I	-	-	I	-	-	El Salvador Demographic Association (ADS), Division of Reproductive Health-Centers for Disease Control and Prevention (CDC). El Salvador Reproductive Health Survey 1998. Atlanta, United States: Centers for Disease Control and Prevention (CDC).	GHDx
27599	El Salvador	El Salvador Reproductive Health Survey 2002-2003	2002 - 2003	I	I	I	-	-	E	-	-	I	-	-	Asociación Demográfica Salvadoreña (ADS), Division of Reproductive Health-Centers for Disease Control and Prevention (CDC). (2004) El Salvador Reproductive Health Survey 2002-2003. San Salvador, El Salvador: ADS.	GHDx
200636	El Salvador	El Salvador Multiple Indicator Cluster Survey 2014	2014	I	I	I	I	I	I	-	-	I	-	I	General Administration of Statistics and Censuses (El Salvador), Ministry of Health (El Salvador), United Nations Children's Fund (UNICEF). El Salvador Multiple Indicator Cluster Survey 2014. New York, United States of America: United Nations Children's Fund (UNICEF), 2017.	GHDx
229697	El Salvador	El Salvador National Immunization Coverage Survey 2011	2011	I	I	I	I	I	I	-	-	I	-	-	Ministry of Health (El Salvador). El Salvador National Immunization Coverage Survey 2011.	GHDx
3655	Equatorial Guinea	Equatorial Guinea Multiple Indicator Cluster Survey 2000	2000	I	I	I	-	-	I	-	-	I	-	-	Ministry of Planning, Economic Development and Public Investment (Equatorial Guinea), United Nations Children's Fund (UNICEF). Equatorial Guinea Multiple Indicator Cluster Survey 2000. New York, United States of America: United Nations Children's Fund (UNICEF).	GHDx
76884	Equatorial Guinea	Equatorial Guinea Demographic and Health Survey 2011	2011	I	I	I	E	E	I	-	-	I	E	-	ICF International, Ministry of Health and Social Welfare (Equatorial Guinea), Ministry of Planning, Economic Development and Public Investment (Equatorial Guinea). Equatorial Guinea Demographic and Health Survey 2011. Fairfax, United States of America: ICF International, 2012.	GHDx
453344	Equatorial Guinea	Equatorial Guinea External Review of Expanded	2016	I	I	I	I	I	I	-	-	I	-	-	Equatorial Guinea External Review of Expanded Programme on Immunization National Coverage Survey 2016.	GHDx

GHDx ID	Country	Series	Year	BCG	DPT1	DPT3	HepB3	Hib3	MCV1	MCV2	PCV3	Pol3	RCV1	RotaC	Citation	Link
		Programme on Immunization National Coverage Survey 2016														
19539	Eritrea	Eritrea Demographic and Health Survey 2002	2002	I	I	I	-	-	I	-	-	I	-	-	Macro International, Inc, National Statistics and Evaluation Office (Eritrea). Eritrea Demographic and Health Survey 2002. Fairfax, United States of America: ICF International.	GHDx
19546	Eritrea	Eritrea Demographic and Health Survey 1995-1996	1995 - 1996	I	I	I	-	-	I	-	-	I	-	-	Macro International, Inc, National Statistics Office (Eritrea). Eritrea Demographic and Health Survey 1995-1996. Fairfax, United States of America: ICF International.	GHDx
156685	Eritrea	The Health Sector in Eritrea	1990 - 2000	I	-	I	-	-	I	-	-	I	-	-	Ministry of Health (Eritrea). The Health Sector in Eritrea. Washington DC, United States of America: World Bank, 2004.	GHDx
224896	Eritrea	Eritrea Health and Nutrition Survey 1993	1993	I	-	I	-	-	I	-	-	I	-	-	Ministry of Health (Eritrea). Eritrea Health and Nutrition Survey 1993.	GHDx
224915	Eritrea	Eritrea EPI Coverage Survey 2000	2000	I	I	I	-	-	I	-	-	I	-	-	Ministry of Health (Eritrea), United Nations Children's Fund (UNICEF). Eritrea EPI Coverage Survey 2000.	GHDx
224928	Eritrea	Eritrea Routine Immunization Coverage Survey 2006	2006	I	I	I	I	-	I	-	-	I	-	-	Ministry of Health (Eritrea), United Nations Children's Fund (UNICEF). Eritrea Routine Immunization Coverage Survey 2006.	GHDx
224936	Eritrea	Eritrea EPI Coverage Survey 2009	2009	I	I	I	I	-	I	-	-	I	-	-	Ministry of Health (Eritrea), United Nations Children's Fund (UNICEF). Eritrea EPI Coverage Survey 2009.	GHDx
224938	Eritrea	Eritrea National EPI Coverage Survey 2013	2013	-	-	I	I	I	I	-	-	I	-	-	Ministry of Health (Eritrea), United Nations Children's Fund (UNICEF), World Health Organization (WHO). Eritrea National EPI Coverage Survey 2013.	GHDx
249999	Eritrea	Eritrea Population and Health Survey 2010	2010	I	I	I	-	-	I	-	-	I	-	-	Kenya Medical Research Institute (KEMRI), National Statistics Office (Eritrea), The Fafo Research Foundation. Eritrea Population and Health Survey 2010.	GHDx
12301	eSwatini	Swaziland Family Planning/Maternal and Child Health Survey 1988-1989	1988 - 1989	I	I	I	-	-	I	-	-	I	-	-	Division of Reproductive Health-Centers for Disease Control and Prevention (CDC) and Ministry of Health. (1989) Swaziland Family Planning/Maternal and Child Health Survey 1988. Atlanta, United States: Centers for Disease Control and Prevention (CDC).	GHDx
12320	eSwatini	Swaziland Multiple Indicator Cluster Survey 2000	2000	I	I	I	-	-	I	-	-	I	-	-	Central Statistical Office (Swaziland), United Nations Children's Fund (UNICEF). Swaziland Multiple Indicator Cluster Survey 2000. New York, United States of America: United Nations Children's Fund (UNICEF).	GHDx
20829	eSwatini	Swaziland Demographic and Health Survey 2006-2007	2006 - 2007	I	I	I	I	-	I	-	-	I	-	-	Central Statistical Office (Swaziland), Macro International, Inc. Swaziland Demographic and Health Survey 2006-2007. Fairfax, United States of America: ICF International.	GHDx
30325	eSwatini	Swaziland Multiple Indicator Cluster Survey 2010	2010	I	I	I	I	I	I	-	-	I	-	-	Central Statistical Office (Swaziland), United Nations Children's Fund (UNICEF). Swaziland Multiple Indicator Cluster Survey 2010. New York, United States of America: United Nations Children's Fund (UNICEF).	GHDx
141312	eSwatini	Swaziland National Nutrition Survey 2008	2008	-	-	-	-	-	I	-	-	-	-	-	Ministry of Health (Swaziland). Swaziland National Nutrition Survey 2008.	GHDx
200707	eSwatini	Swaziland Multiple Indicator Cluster Survey 2014	2014	I	I	I	I	I	I	I	E	I	-	-	Central Statistical Office (Swaziland), United Nations Children's Fund (UNICEF), United Nations Educational, Scientific and Cultural Organization (UNESCO), United Nations Population Fund (UNFPA). Swaziland Multiple Indicator Cluster Survey 2014. New York, United States of America: United Nations Children's Fund (UNICEF), 2016.	GHDx
229598	eSwatini	Swaziland Immunization Coverage Survey 2013	2013	I	I	I	I	I	I	I	-	I	-	-	Swaziland Immunization Coverage Survey 2013.	GHDx
229599	eSwatini	Swaziland Measles Post Campaing Evaluation and EPI Coverage Survey 2006	2006	I	I	I	I	-	I	-	-	I	-	-	Swaziland Measles Post Campaing Evaluation and EPI Coverage Survey 2006.	GHDx
229631	eSwatini	Swaziland Report on National EPI Review 2003	2003	I	I	I	-	-	I	-	-	I	-	-	Swaziland Report on National EPI Review 2003.	GHDx
229633	eSwatini	Swaziland Unicef External Evaluation of Expanded	1989	I	I	I	-	-	I	-	-	I	-	-	Swaziland Unicef External Evaluation of Expanded Program on Immunization 1989 - Preliminary Data.	GHDx

GHDx ID	Country	Series	Year	BCG	DPT1	DPT3	HepB3	Hib3	MCV1	MCV2	PCV3	Pol3	RCV1	RotaC	Citation	Link
		Program on Immunization 1989 - Preliminary Data														
229634	eSwatini	Swaziland Unicef External Evaluation of Expanded Program on Immunization 1988	1988	I	I	I	-	-	I	-	-	I	-	-	Swaziland Unicef External Evaluation of Expanded Program on Immunization 1988.	GHDx
229635	eSwatini	Swaziland Unicef External Evaluation of Expanded Program on Immunization 1987	1987	I	I	I	-	-	I	-	-	I	-	-	Swaziland Unicef External Evaluation of Expanded Program on Immunization 1987.	GHDx
229636	eSwatini	Swaziland Unicef External Evaluation of Expanded Program on Immunization 1984	1984	I	I	I	-	-	I	-	-	I	-	-	Swaziland Unicef External Evaluation of Expanded Program on Immunization 1984.	GHDx
229638	eSwatini	Swaziland Unicef External Evaluation of Expanded Program on Immunization 1983	1983	I	I	I	-	-	I	-	-	I	-	-	Swaziland Unicef External Evaluation of Expanded Program on Immunization 1983.	GHDx
229639	eSwatini	Swaziland Unicef External Evaluation of Expanded Program on Immunization 1982	1982	I	I	I	-	-	I	-	-	I	-	-	Swaziland Unicef External Evaluation of Expanded Program on Immunization 1982.	GHDx
19557	Ethiopia	Ethiopia Demographic and Health Survey 2005	2005	I	I	I	-	-	I	-	-	I	-	-	Macro International, Inc, Population and Housing Census Commissions Office (PHCCO). Ethiopia Demographic and Health Survey 2005. Fairfax, United States of America: ICF International.	GHDx
19571	Ethiopia	Ethiopia Demographic and Health Survey 2000	2000	I	I	I	-	-	I	-	-	I	-	-	Central Statistical Agency (Ethiopia), ORC Macro. Ethiopia Demographic and Health Survey 2000. Calverton, United States of America: ORC Macro, 2001.	GHDx
21301	Ethiopia	Ethiopia Demographic and Health Survey 2010-2011	2010 - 2011	I	I	I	I	I	I	-	-	I	-	-	Central Statistical Agency (Ethiopia), ICF Macro, Ministry of Health (Ethiopia). Ethiopia Demographic and Health Survey 2010-2011. Fairfax, United States of America: ICF International.	GHDx
34047	Ethiopia	Ethiopia Welfare Monitoring Survey 1996	1996	I	-	I	-	-	I	-	-	-	-	-	Central Statistical Agency (Ethiopia). Ethiopia Welfare Monitoring Survey 1996.	GHDx
194013	Ethiopia	Ethiopia Multiple Indicator Cluster Survey 1995	1995	I	-	I	-	-	I	-	-	I	-	-	Ministry of Health (Ethiopia), United Nations Children's Fund (UNICEF). Ethiopia Multiple Indicator Cluster Survey 1995.	GHDx
218568	Ethiopia	Ethiopia Demographic and Health Survey 2016	2016	I	I	I	I	I	I	-	I	I	-	I	Central Statistical Agency (Ethiopia), ICF International. Ethiopia Demographic and Health Survey 2016. Fairfax, United States of America: ICF International, 2017.	GHDx
225023	Ethiopia	Ethiopia Immunization Coverage Survey 2012	2012	I	I	I	I	I	I	-	-	I	-	-	Ethiopia Immunization Coverage Survey 2012.	GHDx
258106	Ethiopia	Ethiopia Health and Health Related Indicators 1999-2000	1999 - 2000	I	-	I	-	-	I	-	-	-	-	-	Ministry of Health (Ethiopia). Ethiopia Health and Health Related Indicators 1999-2000. Addis Ababa, Ethiopia: Ministry of Health (Ethiopia), 2000.	GHDx
258108	Ethiopia	Ethiopia Health and Health Related Indicators 2000-2001	2000 - 2001	I	-	I	-	-	I	-	-	-	-	-	Ministry of Health (Ethiopia). Ethiopia Health and Health Related Indicators 2000-2001. Addis Ababa, Ethiopia: Ministry of Health (Ethiopia), 2001.	GHDx
191162	Fiji	Fiji National Immunisation Coverage Survey 2013	2013	I	I	I	I	I	I	-	-	I	I	-	Devi R, Volavola L, Jenkins K, Comrie-Thomson L, Stewart T, Chan G. Fiji National Immunisation Coverage Survey 2013. Fiji J Public Health. 2014; 3(2): 8-13.	GHDx
225373	Fiji	Fiji National EPI Survey 1999	1999	I	I	I	I	-	I	-	-	I	-	-	Fiji National EPI Survey 1999.	GHDx
19579	Gabon	Gabon Demographic and Health Survey 2000-2001	2000 - 2001	I	I	I	E	E	I	-	-	I	-	-	General Directorate of Statistics and Economic Studies (Gabon), Macro International, Inc. Gabon Demographic and Health Survey 2000-2001. Fairfax, United States of America: ICF International.	GHDx

GHDx ID	Country	Series	Year	BCG	DPT1	DPT3	HepB3	Hib3	MCV1	MCV2	PCV3	Pol3	RCV1	RotaC	Citation	Link
76706	Gabon	Gabon Demographic and Health Survey 2012	2012	I	I	I	I	I	I	-	-	I	-	-	General Directorate of Statistics (Gabon), ICF International, Ministry of Economy, Employment and Sustainable Development (Gabon), Ministry of Health (Gabon). Gabon Demographic and Health Survey 2012. Fairfax, United States of America: ICF International, 2013.	GHDx
90712	Gabon	Gabon Multiple Indicator Cluster Survey 1996	1996	I	I	I	-	-	I	-	-	I	-	-	Ministry of Health (Gabon), United Nations Children's Fund (UNICEF). Gabon Multiple Indicator Cluster Survey 1996.	GHDx
226776	Gabon	Gabon EPI External Review 2012	2012	I	I	I	I	I	I	-	-	I	-	-	Ministry of Health (Gabon). Gabon EPI External Review 2012.	GHDx
3960	Georgia	Georgia Multiple Indicator Cluster Survey 1999	1999	I	I	I	-	-	I	-	-	I	-	-	National Center for Disease Control (Georgia), State Department of Statistics of Georgia, United Nations Children's Fund (UNICEF). Georgia Multiple Indicator Cluster Survey 1999.	GHDx
226576	Georgia	Georgia EPI Coverage Survey with add on Questions 1996	1996	I	I	I	-	-	I	-	-	I	-	-	Ministry of Health (Georgia), United Nations Children's Fund (UNICEF). Georgia EPI Coverage Survey with add on Questions 1996.	GHDx
226577	Georgia	Georgia Immunization Programme Evaluation 1999	1999	I	I	I	-	-	I	-	-	I	-	-	Georgia Immunization Programme Evaluation 1999.	GHDx
4681	Ghana	Ghana Multiple Indicator Cluster Survey 1995	1995	I	I	I	-	-	I	-	-	I	-	-	Ministry of Health (Ghana), United Nations Children's Fund (UNICEF). Ghana Multiple Indicator Cluster Survey 1995.	GHDx
4694	Ghana	Ghana Multiple Indicator Cluster Survey 2006	2006	I	I	I	-	-	I	-	-	I	E	-	Ministry of Health (MOH) (Ghana), Ghana Statistical Service and United Nations Children's Fund (UNICEF). Ghana Multiple Indicator Cluster Survey 2006. New York, United States: United Nations Children's Fund (UNICEF).	GHDx
19587	Ghana	Ghana Demographic and Health Survey 1988	1988	I	I	I	-	-	I	-	-	I	-	-	Ghana Statistical Service, Macro Systems, Inc.; Institute for Resource Development. Ghana Demographic and Health Survey 1988. Fairfax, United States of America: ICF International.	GHDx
19604	Ghana	Ghana Demographic and Health Survey 1993-1994	1993 - 1994	I	I	I	-	-	I	-	-	I	-	-	Ghana Statistical Service, Macro International, Inc. Ghana Demographic and Health Survey 1993-1994. Fairfax, United States of America: ICF International.	GHDx
19614	Ghana	Ghana Demographic and Health Survey 1998-1999	1998 - 1999	I	I	I	-	-	I	-	-	I	-	-	Ghana Statistical Service, Macro International, Inc. Ghana Demographic and Health Survey 1998-1999. Fairfax, United States of America: ICF International.	GHDx
19627	Ghana	Ghana Demographic and Health Survey 2003	2003	I	I	I	-	-	I	-	-	I	-	-	Ghana Statistical Service, Macro International, Inc. Ghana Demographic and Health Survey 2003. Fairfax, United States of America: ICF International.	GHDx
21188	Ghana	Ghana Demographic and Health Survey 2008	2008	I	I	I	I	I	I	-	-	I	-	-	Ghana Statistical Service, Macro International, Inc, Ministry of Health (Ghana). Ghana Demographic and Health Survey 2008. Fairfax, United States of America: ICF International.	GHDx
63993	Ghana	Ghana Multiple Indicator Cluster Survey 2011	2011	I	I	I	I	-	I	-	-	I	-	-	Centers for Disease Control and Prevention (CDC), Ghana Statistical Service, Government of Japan, ICF Macro, Ministry of Health (Ghana), Navrongo Health Research Centre, United Nations Children's Fund (UNICEF), United Nations Population Fund (UNFPA), United States Agency for International Development (USAID). Ghana Multiple Indicator Cluster Survey 2011. New York, United States of America: United Nations Children's Fund (UNICEF), 2013.	GHDx
157027	Ghana	Ghana Demographic and Health Survey 2014	2014	I	I	I	I	I	I	I	I	I	-	I	Ghana Health Service, Ghana Statistical Service, ICF International. Ghana Demographic and Health Survey 2014. Fairfax, United States of America: ICF International, 2016.	GHDx
160576	Ghana	Ghana District Multiple Indicator Cluster Survey 2007-2008	2007 - 2008	I	I	I	I	-	I	-	-	I	E	-	Ghana Statistical Service, Ministry of Health (Ghana), United Nations Children's Fund (UNICEF). Ghana District Multiple Indicator Cluster Survey 2007-2008.	GHDx
227921	Ghana	Ghana EPI Cluster Survey 2012	2012	I	I	I	I	I	I	-	-	I	-	-	Ghana EPI Cluster Survey 2012.	GHDx
236205	Ghana	Ghana Socioeconomic Panel Survey 2009-2010	2009 - 2010	-	I	E	-	-	I	-	-	I	-	-	Economic Growth Center, Yale University, Institute of Statistical, Social and Economic Research, University of Ghana. Ghana Socioeconomic Panel Survey 2009-2010. Washington DC, United States: World Bank.	GHDx
437993	Ghana	Ghana Multiple Indicator Cluster Survey 2017-2018	2017 - 2018	I	I	I	I	I	I	-	I	I	-	I	Centers for Disease Control and Prevention (CDC), Ghana Statistical Service, Government of Japan, ICF Macro, Ministry of Health (Ghana), Navrongo Health Research Centre, United Nations Children's Fund (UNICEF), United Nations Population Fund (UNFPA), United States Agency for International Development (USAID). Ghana Multiple Indicator Cluster Survey 2017-2018. New York, United States of America: United Nations Children's Fund (UNICEF), 2013.	GHDx

GHDx ID	Country	Series	Year	BCG	DPT1	DPT3	HepB3	Hib3	MCV1	MCV2	PCV3	Pol3	RCV1	RotaC	Citation	Link
4779	Guatemala	Guatemala Reproductive Health Survey 2008-2009	2008 - 2009	I	I	I	-	-	I	-	-	I	-	-	Guatemala Ministry of Health and Social Assistance, University of Valle and Division of Reproductive Health-Centers for Disease Control and Prevention (CDC). Guatemala Reproductive Health Survey 2008-2009. Atlanta, United States: Centers for Disease Control and Prevention (CDC).	GHDx
19637	Guatemala	Guatemala Demographic and Health Survey 1995	1995	I	I	I	-	-	I	-	-	I	-	-	Macro International, Inc, National Statistics Institute (Guatemala). Guatemala Demographic and Health Survey 1995. Fairfax, United States of America: ICF International.	GHDx
19647	Guatemala	Guatemala Demographic and Health Survey 1987	1987	I	I	I	-	-	E	-	-	I	-	-	Institute of Nutrition of Central America and Panama, Westinghouse; Institute for Resource Development. Guatemala Demographic and Health Survey 1987. Fairfax, United States: ICF International.	GHDx
19656	Guatemala	Guatemala Interim Demographic and Health Survey 1998-1999	1998 - 1999	I	I	I	-	-	I	-	-	I	-	-	Guatemala Ministry of Health and Social Assistance, Macro International, Inc, National Statistics Institute (Guatemala). Guatemala Interim Demographic and Health Survey 1998-1999. Fairfax, United States of America: ICF International.	GHDx
27563	Guatemala	Guatemala Reproductive Health Survey 2002	2002	I	I	I	-	-	I	-	-	I	-	-	Guatemala Ministry of Health and Social Assistance, University of Valle, Division of Reproductive Health-Centers for Disease Control and Prevention (CDC). (2003) Guatemala Reproductive Health Survey 2002. Atlanta, United States: Centers for Disease Control and Prevention (CDC).	GHDx
32127	Guatemala	Guatemala National Survey of Living Conditions 2006	2006	I	-	I	E	E	I	-	-	I	-	-	National Institute of Statistics (Guatemala), National Statistical System (Guatemala), World Bank (WB), United Nations Development Programme (UNDP), United Nations Economic Commission for Latin America and the Caribbean (CEPAL), Rafael Landavar University, United Nations Population Fund (UNFPA), Norwegian Agency for Development (NORAD), Swedish International Development Agency (SIDA), Secretary of Planning and Programming (SEGEPLAN) (Guatemala), Bank of Guatemala. Guatemala National Survey of Living Conditions 2006. Guatemala City, Guatemala: National Statistics Institute (Guatemala).	GHDx
157031	Guatemala	Guatemala Demographic and Health Survey 2014-2015	2014 - 2015	I	I	I	I	I	E	-	I	I	-	I	ICF International, Institute of Nutrition of Central America and Panama, Ministry of Public Health and Social Assistance (Guatemala), National Statistics Institute (Guatemala), Secretary of Planning and Programming of the Presidency (Segeplan) (Guatemala). Guatemala Demographic and Health Survey 2014-2015. Fairfax, United States of America: ICF International, 2017.	GHDx
4787	Guinea	Guinea Multiple Indicator Cluster Survey 1996	1996	I	I	I	-	-	I	-	-	I	-	-	Ministry of Economy, Finance, and Planning (Guinea), National Statistics Directorate (Guinea), United Nations Children's Fund (UNICEF). Guinea Multiple Indicator Cluster Survey 1996.	GHDx
19659	Guinea	Guinea Demographic and Health Survey 1992	1992	I	I	I	-	-	I	-	-	I	-	-	Macro International, Inc, National Statistics Directorate (Guinea). Guinea Demographic and Health Survey 1992.	GHDx
19670	Guinea	Guinea Demographic and Health Survey 1999	1999	I	I	I	-	-	I	-	-	I	-	-	Macro International, Inc, National Statistics Directorate (Guinea). Guinea Demographic and Health Survey 1999. Fairfax, United States of America: ICF International.	GHDx
19683	Guinea	Guinea Demographic and Health Survey 2005	2005	I	I	I	-	-	I	-	-	I	-	-	Macro International, Inc, National Statistics Directorate (Guinea). Guinea Demographic and Health Survey 2005. Fairfax, United States of America: ICF International.	GHDx
69761	Guinea	Guinea Demographic and Health Survey 2012	2012	I	I	I	-	-	I	-	-	I	-	-	ICF Macro, Ministry of Health and Public Hygiene (Guinea), National Institute of Statistics (Guinea). Guinea Demographic and Health Survey 2012. Fairfax, United States of America: ICF International.	GHDx
141983	Guinea	Guinea National Survey on Nutritional Status and Tracking Key Indicators of Child Survival 2007-2008	2007 - 2008	I	I	I	E	-	I	-	-	I	-	-	National Institute of Statistics (Guinea). Guinea National Survey on Nutritional Status and Tracking Key Indicators of Child Survival 2007-2008.	GHDx
233714	Guinea	Guinea EPI External Review 2011	2011	I	I	I	I	I	I	-	-	I	-	-	Ministry of Health and Public Hygiene (Guinea). Guinea EPI External Review 2011.	GHDx
233728	Guinea	Guinea EPI External Review 2000	2000	I	I	I	-	-	I	-	-	I	-	-	Government of Guinea. Guinea EPI External Review 2000.	GHDx
233733	Guinea	Guinea Combating Childhood Communicable Diseases 1983	1983	I	I	I	-	-	I	-	-	I	-	-	Guinea Combating Childhood Communicable Diseases 1983.	GHDx
303458	Guinea	Guinea Multiple Indicator Cluster Survey 2016	2016	I	I	I	I	I	I	-	-	I	-	-	National Institute of Public Health (NPHI) (Guinea), National Institute of Statistics (Guinea), National Malaria Control Program (Guinea), United Nations Children's Fund (UNICEF). Guinea Multiple Indicator Cluster Survey 2016. New York, United States of America: United Nations Children's Fund (UNICEF), 2018.	GHDx

GHDx ID	Country	Series	Year	BCG	DPT1	DPT3	HepB3	Hib3	MCV1	MCV2	PCV3	Pol3	RCV1	RotaC	Citation	Link
396957	Guinea	Guinea Demographic and Health Survey 2018	2018	I	I	I	I	I	I	-	-	I	-	-	Ministry of Health (Guinea), Ministry of Planning and Economic Development (Guinea), National Institute of Statistics (Guinea). Guinea Demographic and Health Survey 2018. Fairfax, United States of America: ICF International, 2019.	GHDx
4808	Guinea-Bissau	Guinea-Bissau Multiple Indicator Cluster Survey 2000	2000	I	I	I	-	-	I	-	-	I	-	-	Secretary State of Planning, National Institute of Statistics and Census (INEC), United Nations Children's Fund (UNICEF). Guinea-Bissau Multiple Indicator Cluster Survey 2000. New York, United States: United Nations Children's Fund (UNICEF).	GHDx
4818	Guinea-Bissau	Guinea-Bissau Multiple Indicator Cluster Survey 2006	2006	I	I	I	-	-	I	-	-	I	-	-	United Nations Children's Fund (UNICEF), Government of Guinea-Bissau. Guinea-Bissau Multiple Indicator Cluster Survey 2006. New York, United States: United Nations Children's Fund (UNICEF).	GHDx
27215	Guinea-Bissau	Guinea-Bissau Multiple Indicator Cluster Survey 2010	2010	I	I	I	-	-	I	-	-	I	-	-	Centers for Disease Control and Prevention (CDC), National Statistics Institute (Guinea-Bissau), United Nations Children's Fund (UNICEF). Guinea-Bissau Multiple Indicator Cluster Survey 2010. New York, United States of America: United Nations Children's Fund (UNICEF), 2018.	GHDx
174049	Guinea-Bissau	Guinea-Bissau Multiple Indicator Cluster Survey 2014	2014	I	I	I	I	I	I	-	-	I	-	-	National Statistics Institute (Guinea-Bissau), United Nations Children's Fund (UNICEF). Guinea-Bissau Multiple Indicator Cluster Survey 2014. New York, United States of America: United Nations Children's Fund (UNICEF), 2016.	GHDx
233666	Guinea-Bissau	Guinea-Bissau EPI Review 1994	1994	I	I	I	-	-	I	-	-	I	-	-	Ministry of Public Health (Guinea-Bissau). Guinea-Bissau EPI Review 1994.	GHDx
233671	Guinea-Bissau	Guinea-Bissau EPI Review 1988	1988	I	I	I	-	-	I	-	-	I	-	-	Guinea-Bissau EPI Review 1988.	GHDx
4916	Guyana	Guyana Multiple Indicator Cluster Survey 2000	2000	I	I	I	-	-	I	-	-	I	I	-	Bureau of Statistics (Guyana), United Nations Children's Fund (UNICEF). Guyana Multiple Indicator Cluster Survey 2000. New York, United States: United Nations Children's Fund (UNICEF)	GHDx
4926	Guyana	Guyana Multiple Indicator Cluster Survey 2006-2007	2006 - 2007	I	-	-	-	-	I	-	-	I	I	-	United Nations Children's Fund (UNICEF), Bureau of Statistics (Guyana). Guyana Multiple Indicator Cluster Survey 2006. New York, United States: United Nations Children's Fund (UNICEF).	GHDx
21348	Guyana	Guyana Demographic and Health Survey 2009	2009	I	I	I	I	I	I	E	-	I	I	-	Bureau of Statistics (Guyana), ICF Macro, Ministry of Health (Guyana). Guyana Demographic and Health Survey 2009. Fairfax, United States of America: ICF International, 2011.	GHDx
200598	Guyana	Guyana Multiple Indicator Cluster Survey 2014	2014	I	I	I	I	I	I	-	I	I	I	I	Bureau of Statistics (Guyana), Ministry of Health (Guyana), United Nations Children's Fund (UNICEF). Guyana Multiple Indicator Cluster Survey 2014. New York, United States of America: United Nations Children's Fund (UNICEF), 2016.	GHDx
19695	Haiti	Haiti Demographic and Health Survey 1994-1995	1994 - 1995	I	I	I	-	-	I	-	-	I	-	-	Haitian Institute of Childhood (IHE), Macro International, Inc. Haiti Demographic and Health Survey 1994-1995. Calverton, United States of America: Macro International, Inc.	GHDx
19708	Haiti	Haiti Demographic and Health Survey 2000	2000	I	I	I	-	-	I	-	-	I	-	-	Haitian Institute of Childhood (IHE), Macro International, Inc. Haiti Demographic and Health Survey 2000. Fairfax, United States of America: ICF International.	GHDx
19720	Haiti	Haiti Demographic and Health Survey 2005-2006	2005 - 2006	I	I	I	-	-	I	-	-	I	-	-	Haitian Institute of Childhood (IHE), Haitian Institute of Statistics and Informatics, Macro International, Inc. Haiti Demographic and Health Survey 2005-2006. Fairfax, United States of America: ICF International.	GHDx
26680	Haiti	Haiti Global Fund Household Survey 2008	2008	I	I	I	-	-	I	-	-	I	-	-	Global Fund to Fight Aids Tuberculosis and Malaria (GFATM). Haiti Global Fund Household Survey 2008.	GHDx
65118	Haiti	Haiti Demographic and Health Survey 2012	2012	I	I	I	-	-	I	-	-	I	I	-	Centers for Disease Control and Prevention (CDC), Haitian Institute of Childhood (IHE), Haitian Institute of Statistics and Informatics, Macro International, Inc. Haiti Demographic and Health Survey 2012. Fairfax, United States of America: ICF International.	GHDx
218574	Haiti	Haiti Demographic and Health Survey 2016-2017	2016 - 2017	I	I	I	I	I	I	-	-	I	-	I	Haitian Institute of Childhood (IHE), Haitian Institute of Statistics and Informatics, ICF International, Ministry of Public Health and Population (Haiti). Haiti Demographic and Health Survey 2016-2017. Fairfax, United States of America: ICF International.	GHDx
233645	Haiti	Haiti National Vaccination Coverage Survey 2009	2009	I	I	I	-	-	I	-	-	I	-	-	World Health Organization (WHO). Haiti National Vaccination Coverage Survey 2009.	GHDx
5017	Honduras	Honduras Epidemiology and Family Health Survey 1987	1987	I	-	I	-	-	I	-	-	I	-	-	Honduras Family Planning Association (ASHOPLAFA), Ministry of Health (Honduras), Family Health International (FHI). (1989): Honduras Epidemiology and Family Health Survey 1987. Tegucigalpa, Honduras: ASHONPLAFA.	GHDx

GHDx ID	Country	Series	Year	BCG	DPT1	DPT3	HepB3	Hib3	MCV1	MCV2	PCV3	Pol3	RCV1	RotaC	Citation	Link
19728	Honduras	Honduras Demographic and Health Survey 2005-2006	2005 - 2006	I	I	I	I	I	I	-	-	I	-	-	Macro International, Inc, National Institute of Statistics (Honduras), Secretary of Health (Honduras). Honduras Demographic and Health Survey 2005-2006. Fairfax, United States of America: ICF International.	GHDx
27542	Honduras	Honduras Reproductive Health Survey 1996	1996	I	I	I	-	-	I	E	-	I	-	-	Ministry of Health (Honduras), Honduras Family Planning Association (ASHONPLAFA) and Division of Reproductive Health-Centers for Disease Control and Prevention (CDC). (1997) Honduras Reproductive Health Survey 1996. Tegucigalpa, Honduras: ASHONPLAFA.	GHDx
27551	Honduras	Honduras Reproductive Health Survey 2001	2001	I	-	I	-	-	I	-	-	I	-	-	Honduras Family Planning Association (ASHONPLAFA), Ministry of Health (Honduras), and Division of Reproductive Health-Centers for Disease Control and Prevention (CDC). Honduras Reproductive Health Survey 2001. Tegucigalpa, Honduras: Honduras Family Planning Association (ASHONPLAFA).	GHDx
95440	Honduras	Honduras Demographic and Health Survey 2011-2012	2011 - 2012	I	I	I	I	I	I	-	E	I	-	I	ICF Macro, National Institute of Statistics (Honduras). Honduras Demographic and Health Survey 2011-2012. Fairfax, United States of America: ICF International.	GHDx
5127	India	India Multiple Indicator Cluster Survey 2000	2000	I	I	I	-	-	I	-	-	I	-	-	United Nations Statistical Division, World Health Organization (WHO), United Nations Educational, Scientific, and Cultural Organization (UNESCO), United Nations Population Fund (UNFPA), World Bank (WB), London School of Hygiene and Tropical Medicine, United Nations Children's Fund (UNICEF). India Multiple Indicator Cluster Survey 2000. New York, United States: United Nations Children's Fund (UNICEF).	GHDx
19787	India	India Demographic and Health Survey 1992-1993	1992 - 1993	I	I	I	-	-	I	-	-	I	-	-	International Institute for Population Sciences (India). India Demographic and Health Survey 1992-1993. Mumbai, India: International Institute for Population Sciences (India).	GHDx
19950	India	India Demographic and Health Survey 1998-1999	1998 - 1999	I	I	I	-	-	I	-	-	I	-	-	International Institute for Population Sciences (India), Macro International, Inc. India Demographic and Health Survey 1998-1999. Calverton, United States of America: Macro International, Inc.	GHDx
19963	India	India Demographic and Health Survey 2005-2006	2005 - 2006	I	I	I	-	-	I	-	-	I	-	-	International Institute for Population Sciences (India), Macro International, Inc. India Demographic and Health Survey 2005-2006. Fairfax, United States of America: ICF International.	GHDx
65418	India	India Coverage Evaluation Survey 2005	2005	I	-	I	-	-	I	-	-	I	-	-	MODE Services (P) Ltd, Ministry of Health and Family Welfare (India), United Nations Children's Fund (UNICEF). India Coverage Evaluation Survey 2005.	GHDx
65420	India	India Coverage Evaluation Survey 2007	2007	I	I	I	-	-	I	-	-	I	-	-	Ministry of Health and Family Welfare (India), United Nations Children's Fund (UNICEF). India Coverage Evaluation Survey 2007.	GHDx
157050	India	India Demographic and Health Survey 2015-2016	2015 - 2016	I	I	I	I	-	I	-	-	I	-	-	ICF International, International Institute for Population Sciences (India), Ministry of Health and Family Welfare (India). India Demographic and Health Survey 2015-2016. Fairfax, United States of America: ICF International, 2018.	GHDx
233610	India	India Evaluation of Routine Immunization Coverage Survey 1997-1998	1997 - 1998	I	-	I	-	-	I	-	-	I	-	-	Ministry of Health and Family Welfare (India), United Nations Children's Fund (UNICEF). India Evaluation of Routine Immunization Coverage Survey 1997-1998.	GHDx
233611	India	India Evaluation of Routine Immunization Coverage Survey 1998-1999	1998 - 1999	I	I	I	-	-	I	-	-	I	-	-	Ministry of Health and Family Welfare (India), United Nations Children's Fund (UNICEF). India Evaluation of Routine Immunization Coverage Survey 1998-1999.	GHDx
285887	India	India Rapid Survey on Children 2013-2014	2013 - 2014	I	I	I	-	-	I	-	-	I	-	-	Ministry of Women and Child Development (India). India Rapid Survey on Children 2013-2014 .	GHDx
317159	India	India National Health Profile 2017	2017	I	-	I	-	-	I	-	-	I	-	-	Central Bureau of Health Intelligence (India), Ministry of Health and Family Welfare (India). India National Health Profile 2017. New Delhi, India: Central Bureau of Health Intelligence (India), 2017.	GHDx
6464	Indonesia	Indonesia Family Life Survey 2007-2008	2007 - 2008	I	I	I	I	-	I	-	-	I	-	-	Center for Population and Policy Studies, Gadjah Mada University (Indonesia), RAND Corporation, SurveyMETER. Indonesia Family Life Survey 2007-2008. Santa Monica, United States of America: RAND Corporation.	GHDx

GHDx ID	Country	Series	Year	BCG	DPT1	DPT3	HepB3	Hib3	MCV1	MCV2	PCV3	Pol3	RCV1	RotaC	Citation	Link
19979	Indonesia	Indonesia Demographic and Health Survey 1991	1991	I	I	I	-	-	I	-	-	I	-	-	Central Bureau of Statistics (Indonesia), Macro International, Inc, Ministry of Health (Indonesia), National Family Planning Coordinating Board (Indonesia). Indonesia Demographic and Health Survey 1991. Fairfax, United States of America: ICF International.	GHDx
19990	Indonesia	Indonesia Demographic and Health Survey 1994	1994	I	I	I	-	-	I	-	-	I	-	-	Central Bureau of Statistics (Indonesia), Macro International, Inc, Ministry of Health (Indonesia), National Family Planning Coordinating Board (Indonesia). Indonesia Demographic and Health Survey 1994. Fairfax, United States of America: ICF International.	GHDx
19999	Indonesia	Indonesia Demographic and Health Survey 1997	1997	I	I	I	-	-	I	-	-	I	-	-	Central Bureau of Statistics (Indonesia), Macro International, Inc, Ministry of Health (Indonesia), National Family Planning Coordinating Board (Indonesia). Indonesia Demographic and Health Survey 1997. Fairfax, United States of America: ICF International.	GHDx
20011	Indonesia	Indonesia Demographic and Health Survey 2002-2003	2002 - 2003	I	I	I	I	-	I	-	-	I	-	-	Macro International, Inc, Ministry of Health (Indonesia), National Family Planning Coordinating Board (Indonesia), Statistics Indonesia. Indonesia Demographic and Health Survey 2002-2003. Fairfax, United States of America: ICF International.	GHDx
20021	Indonesia	Indonesia Demographic and Health Survey 2007	2007	I	I	I	I	-	I	-	-	I	-	-	Macro International, Inc, Ministry of Health (Indonesia), National Family Planning Coordinating Board (Indonesia), Statistics Indonesia. Indonesia Demographic and Health Survey 2007. Fairfax, United States of America: ICF International.	GHDx
76705	Indonesia	Indonesia Demographic and Health Survey 2012	2012	I	I	I	I	-	I	-	-	I	-	-	ICF International, Ministry of Health (Indonesia), National Population and Family Planning Board (Indonesia), Statistics Indonesia. Indonesia Demographic and Health Survey 2012. Fairfax, United States of America: ICF International.	GHDx
114622	Indonesia	Indonesia Basic Health Research 2010	2010	I	-	I	I	-	I	-	-	I	-	-	Agency for Health Research and Development (Indonesia). Indonesia Basic Health Research 2010.	GHDx
141911	Indonesia	Indonesia Multiple Indicator Cluster Survey 1995	1995	I	-	I	-	-	I	-	-	I	-	-	Central Bureau of Statistics (Indonesia), United Nations Children's Fund (UNICEF). Indonesia Multiple Indicator Cluster Survey 1995.	GHDx
233624	Indonesia	Indonesia Immunization Coverage Survey 2007-2008	2007 - 2008	I	I	I	I	-	I	-	-	I	-	-	United Nations Children's Fund (UNICEF), World Health Organization (WHO). Indonesia Immunization Coverage Survey 2007-2008.	GHDx
233625	Indonesia	Indonesia NID and Routine Coverage Survey 2002	2002	I	I	I	I	-	I	-	-	I	-	-	Indonesia NID and Routine Coverage Survey 2002.	GHDx
233632	Indonesia	Indonesia Expanded Program on Immunization 1978	1978	I	I	-	-	-	-	-	-	-	-	-	Indonesia Expanded Program on Immunization 1978.	GHDx
264956	Indonesia	Indonesia Family Life Survey 2014-2015	2014 - 2015	I	I	I	I	-	I	-	-	I	-	-	RAND Corporation, SurveyMETER. Indonesia Family Life Survey 2014-2015. Santa Monica, United States of America: RAND Corporation, 2016.	GHDx
286781	Indonesia	Indonesia Demographic and Health Survey 2017	2017	I	I	I	I	I	I	-	-	I	-	-	Ministry of Health (Indonesia), National Population and Family Planning Board (Indonesia), Statistics Indonesia. Indonesia Demographic and Health Survey 2017. Fairfax, United States of America: ICF International, 2019.	GHDx
395694	Indonesia	Indonesia National Socioeconomic Survey 2017	2017	I	I	I	I	-	I	-	-	I	E	-	Central Bureau of Statistics (Indonesia). Indonesia National Socioeconomic Survey 2017. Jakarta, Indonesia: Central Bureau of Statistics (Indonesia), 2018.	GHDx
81416	Iran	Iran Multiple Indicator Demographic and Health Survey 2010	2010	I	I	I	I	-	I	I	-	I	I	-	Ministry of Health and Medical Education (Iran), Statistical Centre of Iran. Iran Multiple Indicator Demographic and Health Survey 2010.	GHDx
95514	Iran	Iran Multiple Indicator Cluster Survey 1997	1997	I	-	I	-	-	I	-	-	I	-	-	Ministry of Health and Medical Education (Iran), Statistical Centre of Iran, United Nations Children's Fund (UNICEF). Iran Multiple Indicator Cluster Survey 1997.	GHDx
7018	Iraq	Iraq Immunization, Diarrheal Disease, Maternal and Childhood Mortality Survey 1990	1990	I	I	I	-	-	I	-	-	I	-	-	Ministry of Health (Iraq), Ministry of Planning (Iraq), United Nations Children's Fund (UNICEF). Iraq Immunization, Diarrheal Disease, Maternal and Childhood Mortality Survey 1990.	GHDx
7028	Iraq	Iraq Multiple Indicator Cluster Survey 2006	2006	I	I	I	I	-	I	-	-	I	E	-	United Nations Children's Fund (UNICEF), Central Organization for Statistics and Information Technology (Iraq), Kurdistan Regional Statistics Office. Iraq Multiple Indicator Cluster Survey 2006. New York, United States: United Nations Children's Fund (UNICEF).	GHDx

GHDx ID	Country	Series	Year	BCG	DPT1	DPT3	HepB3	Hib3	MCV1	MCV2	PCV3	Pol3	RCV1	RotaC	Citation	Link
7054	Iraq	Iraq Multiple Indicator Cluster Survey 2000	2000	I	I	I	-	-	I	-	-	I	-	-	Central Statistical Organization (Iraq), United Nations Children's Fund (UNICEF). Iraq Multiple Indicator Cluster Survey 2000. New York, United States: United Nations Children's Fund (UNICEF).	GHDx
34524	Iraq	Iraq Household Socioeconomic Survey 2006-2007	2006 - 2007	I	I	I	-	-	-	-	-	I	-	-	Central Organization for Statistics and Information Technology (Iraq), Kurdistan Regional Statistics Office, World Bank. Iraq Household Socioeconomic Survey 2006-2007. Washington DC, United States of America: World Bank.	GHDx
76707	Iraq	Iraq Multiple Indicator Cluster Survey 2011	2011	I	I	I	I	-	I	-	-	I	I	-	Central Organization for Statistics and Information Technology (Iraq), Kurdistan Regional Statistics Office, Ministry of Health (Iraq), United Nations Children's Fund (UNICEF). Iraq Multiple Indicator Cluster Survey 2011. New York, United States of America: United Nations Children's Fund (UNICEF), 2013.	GHDx
90717	Iraq	Iraq Multiple Indicator Cluster Survey 1996	1996	I	-	I	-	-	I	-	-	I	-	-	Central Statistical Organization (Iraq), United Nations Children's Fund (UNICEF). Iraq Multiple Indicator Cluster Survey 1996.	GHDx
232988	Iraq	Iraq Immunization Coverage Survey 1988	1988	I	I	I	-	-	I	-	-	I	-	-	Iraq Immunization Coverage Survey 1988.	GHDx
232989	Iraq	Iraq Evaluation of Immunization Coverage and Diarrhoeal Diseases Program 1987	1987	I	I	I	-	-	I	-	-	I	-	-	Iraq Evaluation of Immunization Coverage and Diarrhoeal Diseases Program 1987.	GHDx
232992	Iraq	Iraq Immunization Coverage Survey 1986	1986	-	I	I	-	-	I	-	-	I	-	-	Iraq Immunization Coverage Survey 1986.	GHDx
337359	Iraq	Situation Analysis of Children and Women in Iraq	1960 - 1999	I	I	I	-	-	I	-	-	I	-	-	United Nations Children's Fund (UNICEF). Situation Analysis of Children and Women in Iraq. 1998.	GHDx
385708	Iraq	Iraq Multiple Indicator Cluster Survey 2018	2018	I	-	I	I	I	I	I	I	I	I	-	Central Statistical Organization (Iraq), United Nations Children's Fund (UNICEF). Iraq Multiple Indicator Cluster Survey 2018. New York, United States of America: United Nations Children's Fund (UNICEF), 2019.	GHDx
232970	Italy	Italy National Infant Vaccination Coverage Survey 2003	2003	-	-	I	I	I	I	-	-	I	-	-	National Institute of Health (Italy). Italy National Infant Vaccination Coverage Survey 2003.	GHDx
232977	Italy	Italy National Children Vaccination Coverage Survey 1998	1998	-	-	I	I	-	I	-	-	I	-	-	National Institute of Health (Italy). Italy National Children Vaccination Coverage Survey 1998.	GHDx
7149	Jamaica	Jamaica Multiple Indicator Cluster Survey 2005	2005	I	I	I	-	-	I	-	-	I	-	-	Statistical Institute of Jamaica (STATIN) and United Nations Children's Fund (UNICEF). Jamaica Multiple Indicator Cluster Survey 2005. New York, United States: United Nations Children's Fund (UNICEF).	GHDx
7222	Jamaica	Jamaica Survey of Living Conditions 2001	2001	-	I	I	-	-	-	-	-	I	-	-	Planning Institute of Jamaica, Statistical Institute of Jamaica. Jamaica Survey of Living Conditions 2001.	GHDx
45852	Jamaica	Jamaica Survey of Living Conditions 1998	1998	-	I	I	-	-	-	-	-	I	-	-	Planning Institute of Jamaica, Statistical Institute of Jamaica. Jamaica Survey of Living Conditions 1998.	GHDx
141336	Jamaica	Jamaica Multiple Indicator Cluster Survey 2011	2011	I	I	I	E	E	I	-	-	I	-	-	Statistical Institute of Jamaica, United Nations Children's Fund (UNICEF). Jamaica Multiple Indicator Cluster Survey 2011. New York, United States of America: United Nations Children's Fund (UNICEF), 2015.	GHDx
7274	Jordan	Jordan EPI/CDD and Child Mortality Survey 1990	1990	-	I	I	-	-	I	-	-	-	-	-	Ministry of Health (Jordan), United Nations Children's Fund (UNICEF). Jordan EPI/CDD and Child Mortality Survey 1990.	GHDx
7276	Jordan	Jordan Family Planning/Maternal and Child Health Survey 1983	1983	-	I	I	-	-	I	-	-	I	-	-	Jordan Department of Statistics and Division of Reproductive Health-Centers for Disease Control and Prevention (CDC). (1984) Jordan Family Planning/Maternal and Child Health Survey 1983. Atlanta, United States: Centers for Disease Control and Prevention (CDC).	GHDx
20051	Jordan	Jordan Demographic and Health Survey 1990	1990	I	I	I	-	-	I	-	-	I	-	-	Department of Statistics (Jordan), Institute for Resource Development, Macro Systems, Ministry of Planning (Jordan). Jordan Demographic and Health Survey 1990. Fairfax, United States of America: ICF International.	GHDx
20060	Jordan	Jordan Demographic and Health Survey 1997	1997	I	I	I	I	-	I	I	-	I	-	-	Department of Statistics (Jordan), Macro International, Inc. Jordan Demographic and Health Survey 1997. Fairfax, United States: ICF International.	GHDx

GHDx ID	Country	Series	Year	BCG	DPT1	DPT3	HepB3	Hib3	MCV1	MCV2	PCV3	Pol3	RCV1	RotaC	Citation	Link
20073	Jordan	Jordan Demographic and Health Survey 2002	2002	I	I	I	I	I	I	I	-	I	I	-	Department of Statistics (Jordan), Macro International, Inc. Jordan Demographic and Health Survey 2002. Fairfax, United States of America: ICF International.	GHDx
20083	Jordan	Jordan Demographic and Health Survey 2007	2007	I	I	I	I	I	I	-	-	I	I	-	Department of Statistics (Jordan), Macro International, Inc. Jordan Demographic and Health Survey 2007. Fairfax, United States of America: ICF International.	GHDx
77517	Jordan	Jordan Demographic and Health Survey 2012	2012	I	I	I	I	I	I	-	-	I	I	-	Department of Statistics (Jordan), ICF International. Jordan Demographic and Health Survey 2012. Fairfax, United States of America: ICF International.	GHDx
356955	Jordan	Jordan Demographic and Health Survey 2017-2018	2017 - 2018	I	I	I	I	I	I	I	-	I	-	I	Department of Statistics (Jordan), ICF International. Jordan Demographic and Health Survey 2017-2018. Fairfax, United States of America: ICF International, 2019.	GHDx
7340	Kazakhstan	Kazakhstan Multiple Indicator Cluster Survey 2006	2006	E	I	I	I	-	I	-	-	I	-	-	Agency of the Republic of Kazakhstan on Statistics and United Nations Children's Fund (UNICEF). Kazakhstan Multiple Indicator Cluster Survey 2006. New York, United States: United Nations Children's Fund (UNICEF).	GHDx
20092	Kazakhstan	Kazakhstan Demographic and Health Survey 1995	1995	I	I	I	-	-	I	-	-	I	-	-	Macro International, Inc, National Institute of Nutrition (Kazakhstan). Kazakhstan Demographic and Health Survey 1995. Fairfax, United States: ICF International.	GHDx
20103	Kazakhstan	Kazakhstan Demographic and Health Survey 1999	1999	I	I	I	I	-	I	-	-	I	-	-	Academy of Preventive Medicine (Kazakhstan), Macro International, Inc. Kazakhstan Demographic and Health Survey 1999. Fairfax, United States of America: ICF International.	GHDx
76702	Kazakhstan	Kazakhstan Multiple Indicator Cluster Survey 2010-2011	2010 - 2011	I	I	I	E	-	I	-	-	I	I	-	Agency of the Republic of Kazakhstan on Statistics, United Nations Children's Fund (UNICEF). Kazakhstan Multiple Indicator Cluster Survey 2010-2011. New York, United States of America: United Nations Children's Fund (UNICEF), 2013.	GHDx
260403	Kazakhstan	Kazakhstan Multiple Indicator Cluster Survey 2015	2015	I	I	I	I	I	I	-	-	I	I	-	Ministry of National Economy (Kazakhstan), United Nations Children's Fund (UNICEF). Kazakhstan Multiple Indicator Cluster Survey 2015. New York, United States of America: United Nations Children's Fund (UNICEF), 2017.	GHDx
7375	Kenya	Kenya Integrated Household Budget Survey 2005-2006	2005 - 2006	I	I	I	-	-	I	-	-	I	-	-	Central Bureau of Statistics (Kenya), UK Department for International Development (DFID), United States Agency for International Development (USAID), European Union (EU), Danish International Development Agency (DANIDA), World Bank (WB), United Nations Development Programme (UNDP). Kenya Integrated Household Budget Survey 2005-2006. Nairobi, Kenya: Central Bureau of Statistics (Kenya).	GHDx
7387	Kenya	Kenya Multiple Indicator Cluster Survey 2000	2000	I	I	I	-	-	I	-	-	I	-	-	Central Bureau of Statistics (Kenya), United Nations Children's Fund (UNICEF). Kenya Multiple Indicator Cluster Survey 2000. New York, United States: United Nations Children's Fund (UNICEF).	GHDx
20109	Kenya	Kenya Demographic and Health Survey 1988-1989	1988 - 1989	I	I	I	-	-	I	-	-	I	-	-	Macro Systems, Inc.; Institute for Resource Development, National Council for Population Development (NCPD). Kenya Demographic and Health Survey 1988-1989. Fairfax, United States of America: ICF International.	GHDx
20120	Kenya	Kenya Demographic and Health Survey 1993	1993	I	I	I	-	-	I	-	-	I	-	-	Central Bureau of Statistics (Kenya), Macro International, Inc, National Council for Population Development (NCPD). Kenya Demographic and Health Survey 1993. Fairfax, United States of America: ICF International.	GHDx
20132	Kenya	Kenya Demographic and Health Survey 1998	1998	I	I	I	-	-	I	-	-	I	-	-	Central Bureau of Statistics (Kenya), Macro International, Inc, National Council for Population Development (NCPD). Kenya Demographic and Health Survey 1998. Fairfax, United States of America: ICF International.	GHDx
20145	Kenya	Kenya Demographic and Health Survey 2003	2003	I	I	I	-	-	I	-	-	I	-	-	Centers for Disease Control and Prevention (CDC), Central Bureau of Statistics (Kenya), Macro International, Inc, Ministry of Health (Kenya), National Council for Population and Development (Kenya). Kenya Demographic and Health Survey 2003. Fairfax, United States of America: ICF International.	GHDx
21365	Kenya	Kenya Demographic and Health Survey 2008-2009	2008 - 2009	I	I	I	I	I	I	-	-	I	-	-	ICF Macro, Kenya Medical Research Institute (KEMRI), Kenya National Bureau of Statistics, Ministry of Public Health and Sanitation (Kenya), National AIDS and STI Control Programme (NASCOP) (Kenya), National Aids Control Council (NACC), National Coordinating Agency for Population and Development (Kenya). Kenya Demographic and Health Survey 2008-2009. Fairfax, United States of America: ICF International.	GHDx
49586	Kenya	Kenya Integrated Household Budget Survey Tabular Data 2005-2006	2005 - 2006	I	-	-	-	-	I	-	-	E	-	-	Kenya National Bureau of Statistics. Kenya Integrated Household Budget Survey Tabular Data 2005-2006. Nairobi, Kenya: Kenya National Bureau of Statistics.	GHDx
157057	Kenya	Kenya Demographic and Health Survey 2014	2014	I	I	I	I	I	I	-	I	I	-	-	ICF International, Kenya Medical Research Institute (KEMRI), Kenya National Bureau of Statistics, Ministry of Health (Kenya), National AIDS Control Council (Kenya), National	GHDx

GHDx ID	Country	Series	Year	BCG	DPT1	DPT3	HepB3	Hib3	MCV1	MCV2	PCV3	Pol3	RCV1	RotaC	Citation	Link
															Council for Population and Development (Kenya). Kenya Demographic and Health Survey 2014. Fairfax, United States of America: ICF International.	
232807	Kenya	Kenya EPI National Immunization Coverage Survey 1992	1992	I	I	I	-	-	I	-	-	I	-	-	Kenya EPI National Immunization Coverage Survey 1992.	GHDx
232815	Kenya	Kenya National Immunization Coverage Survey 1987	1987	-	I	I	-	-	I	-	-	I	-	-	Kenya National Immunization Coverage Survey 1987.	GHDx
287198	Kenya	Kenya Integrated Household Budget Survey 2015-2016	2015 - 2016	-	-	-	-	-	I	-	-	-	-	-	Kenya National Bureau of Statistics. 2015-2016 Kenya Integrated Household Budget Survey. Nairobi, Kenya: Kenya National Bureau of Statistics, 2015.	GHDx
448112	Kenya	Kenya Post Measles-Rubella Supplementary Immunization Activities Coverage Survey 2016	2016	-	-	-	-	-	I	-	-	-	-	-	Centers for Disease Control and Prevention (CDC), Kenya National Bureau of Statistics, Ministry of Health (Kenya), World Health Organization (WHO). Kenya Post Measles-Rubella Supplementary Immunization Activities Coverage Survey 2016.	GHDx
135533	Kiribati	Kiribati Demographic and Health Survey 2009	2009	I	I	I	-	-	I	-	-	I	-	-	Kiribati National Statistics Office, Secretariat of the Pacific Community (SPC). Kiribati Demographic and Health Survey 2009.	GHDx
438014	Kiribati	Kiribati Multiple Indicator Cluster Survey 2018-2019	2018 - 2019	I	I	I	I	I	I	-	I	I	I	-	Kiribati National Statistics Office, Ministry of Health and Medical Services (Kiribati), Secretariat of the Pacific Community (SPC), United Nations Children's Fund (UNICEF), United Nations Population Fund (UNFPA). Kiribati Multiple Indicator Cluster Survey 2018-2019. New York, United States of America: United Nations Children's Fund (UNICEF), 2020.	GHDx
7503	Kuwait	Kuwait Child Health Survey 1987	1987	E	-	I	-	-	I	-	-	I	-	-	Health Ministers Council for GCC States, Ministry of Health (Kuwait). Kuwait Child Health Survey 1987.	GHDx
7504	Kuwait	Kuwait Family Health Survey 1996	1996	E	I	I	-	-	I	-	-	I	-	-	Health Ministers Council for GCC States, Ministry of Health (Kuwait), United Nations Statistics Division (UNSD). Kuwait Family Health Survey 1996.	GHDx
232531	Kuwait	Kuwait Vaccine Assessment Survey 1998	1998	-	-	I	-	-	I	-	-	I	-	-	Kuwait Vaccine Assessment Survey 1998.	GHDx
7540	Kyrgyzstan	Kyrgyzstan Multiple Indicator Cluster Survey 2005-2006	2005 - 2006	I	I	E	-	-	I	-	-	E	-	-	United Nations Children's Fund (UNICEF), National Statistical Committee of the Kyrgyz Republic. Kyrgyzstan Multiple Indicator Cluster Survey 2005-2006. New York, United States: United Nations Children's Fund (UNICEF).	GHDx
20154	Kyrgyzstan	Kyrgyzstan Demographic and Health Survey 1997	1997	I	I	I	-	-	I	-	-	I	-	-	Macro International, Inc, Ministry of Health (Kyrgyzstan), Research Institute of Obstetrics and Pediatrics (Kyrgyzstan). Kyrgyzstan Demographic and Health Survey 1997. Fairfax, United States of America: ICF International.	GHDx
77518	Kyrgyzstan	Kyrgyzstan Demographic and Health Survey 2012	2012	I	I	I	I	I	I	-	-	I	-	-	ICF International, Ministry of Health (Kyrgyzstan), National Statistical Committee of the Kyrgyz Republic. Kyrgyzstan Demographic and Health Survey 2012. Fairfax, United States of America: ICF International.	GHDx
90716	Kyrgyzstan	Kyrgyzstan Multiple Indicator Cluster Survey 1995	1995	I	-	I	-	-	I	-	-	I	-	-	Government of Kyrgyzstan, Ministry of Health (Kyrgyzstan), United Nations Children's Fund (UNICEF). Kyrgyzstan Multiple Indicator Cluster Survey 1995.	GHDx
162283	Kyrgyzstan	Kyrgyzstan Multiple Indicator Cluster Survey 2014	2014	E	I	I	I	I	E	-	-	I	-	-	National Statistical Committee of the Kyrgyz Republic, United Nations Children's Fund (UNICEF). Kyrgyzstan Multiple Indicator Cluster Survey 2014. New York, United States of America: United Nations Children's Fund (UNICEF), 2015.	GHDx
408226	Kyrgyzstan	Kyrgyzstan Multiple Indicator Cluster Survey 2018	2018	I	I	I	I	I	I	-	I	I	I	-	National Statistical Committee of the Kyrgyz Republic, United Nations Children's Fund (UNICEF). Kyrgyzstan Multiple Indicator Cluster Survey 2018. New York, United States of America: United Nations Children's Fund (UNICEF), 2019.	GHDx
7603	Laos	Laos Multiple Indicator Cluster Survey 1996	1996	I	-	I	-	-	I	-	-	I	-	-	Ministry of Education and Sports (Laos), Ministry of Health (Laos), National Statistical Center (Laos), United Nations Children's Fund (UNICEF). Laos Multiple Indicator Cluster Survey 1996. New York, United States: United Nations Children's Fund (UNICEF).	GHDx
7618	Laos	Laos Multiple Indicator Cluster Survey 2000	2000	I	-	I	-	-	I	-	-	I	-	-	Ministry of Health (Laos), National Institute of Public Health (NIOPH), National Statistical Center (Laos), United Nations Children's Fund (UNICEF). Laos Multiple Indicator Cluster Survey 2000. New York, United States of America: United Nations Children's Fund (UNICEF).	GHDx

GHDx ID	Country	Series	Year	BCG	DPT1	DPT3	HepB3	Hib3	MCV1	MCV2	PCV3	Pol3	RCV1	RotaC	Citation	Link
7629	Laos	Laos Multiple Indicator Cluster Survey 2006	2006	I	I	I	-	-	I	-	-	I	-	-	United Nations Children's Fund (UNICEF), Department of Statistics (Laos), Ministry of Health (Laos). Laos Multiple Indicator Cluster Survey 2006. New York, United States: United Nations Children's Fund (UNICEF).Â	GHDx
103973	Laos	Laos Multiple Indicator Cluster Survey 2011-2012	2011 - 2012	I	I	I	I	I	I	-	-	I	-	-	Ministry of Education and Sports (Laos), Ministry of Health (Laos), Ministry of Planning and Investment (Laos). Laos Multiple Indicator Cluster Survey 2011-2012. New York, United States of America: United Nations Children's Fund (UNICEF), 2013.	GHDx
375362	Laos	Laos Multiple Indicator Cluster Survey 2017	2017	I	I	I	I	I	I	-	I	I	I	-	Lao Statistics Bureau, Ministry of Education and Sports (Laos), Ministry of Health (Laos), United Nations Children's Fund (UNICEF). Laos Multiple Indicator Cluster Survey 2017. New York, United States of America: United Nations Children's Fund (UNICEF), 2018.	GHDx
448337	Laos	Laos National Immunization Survey 2015	2015	I	I	I	I	I	I	-	-	I	I	-	Ministry of Health (Cambodia), World Health Organization (WHO). Laos National Immunization Survey 2015.	GHDx
194100	Latvia	Latvia Maternal and Infant Health Care 2008	1950 - 2008	I	-	-	-	-	-	-	-	-	-	-	Ministry of Health of the Republic of Latvia. Latvia Maternal and Infant Health Care 2008. Riga, Latvia: Ministry of Health of the Republic of Latvia, 2009.	GHDx
7678	Lebanon	Lebanon Multiple Indicator Cluster Survey 2000	2000	-	-	I	-	-	I	-	-	I	I	-	Central Administration of Statistics (Lebanon), United Nations Children's Fund (UNICEF). Lebanon Multiple Indicator Cluster Survey 2000. New York, United States: United Nations Children's Fund (UNICEF).	GHDx
44861	Lebanon	Lebanon Family Health Survey 2004	2004	-	I	I	I	-	I	-	-	I	I	-	Central Administration of Statistics (Lebanon), League of Arab States, Ministry of Social Affairs (Lebanon), Pan Arab Project for Family Health (PAPFAM). Lebanon Family Health Survey 2004.	GHDx
138112	Lebanon	Lebanon Multiple Indicator Cluster Survey 2009	2009	-	I	I	I	I	I	-	-	I	-	-	Central Administration of Statistics (Lebanon). Lebanon Multiple Indicator Cluster Survey 2009.	GHDx
232518	Lebanon	Lebanon National EPI/CDD Survey 1996	1996	-	I	I	-	-	I	-	-	I	-	-	Ministry of Public Health (Lebanon), United Nations Children's Fund (UNICEF). Lebanon National EPI/CDD Survey 1996.	GHDx
232519	Lebanon	Lebanon National Health Indicators of Children Survey 1994	1994	-	I	I	-	-	I	-	-	I	-	-	Lebanon National Health Indicators of Children Survey 1994.	GHDx
232520	Lebanon	Lebanon National Immunization Coverage Survey 1992	1992	-	I	I	-	-	I	-	-	I	-	-	Lebanon National Immunization Coverage Survey 1992.	GHDx
232525	Lebanon	Lebanon National EPI CDD IMR Survey 1990	1990	-	-	I	-	-	I	-	-	I	-	-	Lebanon National EPI CDD IMR Survey 1990.	GHDx
401878	Lebanon	Vaccination coverage in Lebanon following the Syrian crisis: results from the district-based immunization coverage evaluation survey 2016	2016	-	I	I	I	I	I	I	-	I	-	-	Mansour Z, Hamadeh R, Rady A, Danovaro-Holliday MC, Fahmy K, Said R, Brandt L, Warrak R, Ammar W. Vaccination coverage in Lebanon following the Syrian crisis: results from the district-based immunization coverage evaluation survey 2016. BMC Public Health. 2019; 19(1): 58.	GHDx
7721	Lesotho	Lesotho Multiple Indicator Cluster Survey 2000	2000	I	I	I	-	-	I	-	-	I	-	-	Bureau of Statistics (Lesotho), United Nations Children's Fund (UNICEF). Lesotho Multiple Indicator Cluster Survey 2000. New York, United States of America: United Nations Children's Fund (UNICEF).	GHDx
20167	Lesotho	Lesotho Demographic and Health Survey 2004-2005	2004 - 2005	I	I	I	I	-	I	-	-	I	-	-	Bureau of Statistics (Lesotho), Macro International, Inc, Ministry of Health and Social Welfare (Lesotho). Lesotho Demographic and Health Survey 2004-2005. Fairfax, United States of America: ICF International.	GHDx
21382	Lesotho	Lesotho Demographic and Health Survey 2009-2010	2009 - 2010	I	I	I	-	-	I	-	-	I	-	-	ICF Macro, Ministry of Health and Social Welfare (Lesotho). Lesotho Demographic and Health Survey 2009-2010. Fairfax, United States of America: ICF International.	GHDx
142213	Lesotho	Lesotho Multiple Indicator Cluster Survey 1996	1996	I	-	I	-	-	I	-	-	I	-	-	Bureau of Statistics (Lesotho), Ministry of Health (Lesotho), United Nations Children's Fund (UNICEF). Lesotho Multiple Indicator Cluster Survey 1996.	GHDx
157058	Lesotho	Lesotho Demographic and Health Survey 2014	2014	I	I	I	-	-	I	-	-	I	-	-	ICF International, Ministry of Health and Social Welfare (Lesotho). Lesotho Demographic and Health Survey 2014. Fairfax, United States of America: ICF International.	GHDx

GHDx ID	Country	Series	Year	BCG	DPT1	DPT3	HepB3	Hib3	MCV1	MCV2	PCV3	Pol3	RCV1	RotaC	Citation	Link
232277	Lesotho	Lesotho Post SIAs and Routine Immunization Coverage Survey 2013	2013	I	I	I	I	I	I	I	-	I	-	-	Government of Lesotho. Lesotho Post SIAs and Routine Immunization Coverage Survey 2013.	GHDx
232279	Lesotho	Lesotho National Nutrition and EPI Cluster Survey 2002	2002	I	I	I	-	-	I	-	-	I	-	-	Government of Lesotho, Irish Aid, United Nations Children's Fund (UNICEF). Lesotho National Nutrition and EPI Cluster Survey 2002.	GHDx
232403	Lesotho	Lesotho EPI Coverage Survey 1982	1982	I	I	I	-	-	I	-	-	I	-	-	Ministry of Health (Lesotho). Lesotho EPI Coverage Survey 1982.	GHDx
232404	Lesotho	Lesotho EPI Coverage Survey 1984	1984	I	I	I	-	-	I	-	-	I	-	-	Ministry of Health (Lesotho). Lesotho EPI Coverage Survey 1984.	GHDx
232443	Lesotho	Lesotho EPI Coverage Survey 1986	1986	I	I	I	-	-	I	-	-	I	-	-	Ministry of Health (Lesotho). Lesotho EPI Coverage Survey 1986.	GHDx
232445	Lesotho	Lesotho EPI Coverage Survey 1988	1988	I	I	I	-	-	I	-	-	I	-	-	Ministry of Health (Lesotho). Lesotho EPI Coverage Survey 1988.	GHDx
427778	Lesotho	Lesotho Multiple Indicator Cluster Survey 2018	2018	I	I	I	I	I	I	I	-	I	-	I	Bureau of Statistics (Lesotho), United Nations Children's Fund (UNICEF). Lesotho Multiple Indicator Cluster Survey 2018. New York, United States of America: United Nations Children's Fund (UNICEF), 2019.	GHDx
7751	Liberia	Liberia Multiple Indicator Cluster Survey 1995	1995	I	-	I	-	-	I	-	-	I	-	-	Bureau of Statistics (Liberia), United Nations Children's Fund (UNICEF). Liberia Multiple Indicator Cluster Survey 1995.	GHDx
20180	Liberia	Liberia Demographic and Health Survey 1986	1986	I	I	I	-	-	I	-	-	I	-	-	Ministry of Planning and Economic Affairs (Liberia), Westinghouse; Institute for Resource Development. Liberia Demographic and Health Survey 1986. Fairfax, United States of America: ICF International.	GHDx
20191	Liberia	Liberia Demographic and Health Survey 2006-2007	2006 - 2007	I	I	I	-	-	I	-	-	I	-	-	Liberia Institute for Statistics and Geo-information Services (LISGIS), Macro International, Inc. Liberia Demographic and Health Survey 2006-2007. Fairfax, United States of America: ICF International.	GHDx
77385	Liberia	Liberia Demographic and Health Survey 2013	2013	I	I	I	I	I	I	-	-	I	-	-	ICF International, Liberia Institute for Statistics and Geo-information Services (LISGIS), National AIDS and STI Control Program (NACP), Ministry of Health and Social Welfare (Liberia). Liberia Demographic and Health Survey 2013. Fairfax, United States of America: ICF International.	GHDx
90711	Liberia	Liberia Demographic and Health Survey 1999-2000	1999 - 2000	-	I	I	-	-	I	-	-	-	-	-	Ministry of Planning and Economic Affairs (Liberia), United Nations Population Fund (UNFPA), University of Liberia. Liberia Demographic and Health Survey 1999-2000.	GHDx
141586	Liberia	Liberia National Nutrition Survey 1999-2000	1999 - 2000	I	I	I	-	-	I	-	-	I	-	-	Christian Health Association of Liberia, Ministry of Health and Social Welfare (Liberia), United Nations Children's Fund (UNICEF). Liberia National Nutrition Survey 1999-2000.	GHDx
232467	Liberia	Liberia Routine Immunization Survey 2012	2012	I	I	I	I	I	I	-	-	I	-	-	Liberia Routine Immunization Survey 2012.	GHDx
232470	Liberia	Liberia EPI Cluster Survey 2005	2005	I	I	I	-	-	I	-	-	I	-	-	United Nations Children's Fund (UNICEF), World Health Organization (WHO). Liberia EPI Cluster Survey 2005.	GHDx
232515	Liberia	Liberia Integrated Management of Childhood Illness Household Baseline Survey 2000	2000	I	I	I	-	-	I	-	-	I	-	-	Liberia Integrated Management of Childhood Illness Household Baseline Survey 2000.	GHDx
286768	Liberia	Liberia Malaria Indicator Survey 2016	2016	I	I	I	I	I	I	-	I	I	-	-	ICF International, Liberia Institute for Statistics and Geo-information Services (LISGIS), National Malaria Control Program (Liberia). Liberia Malaria Indicator Survey 2016. Fairfax, United States of America: ICF International, 2017.	GHDx
124079	Libya	Libya Multiple Indicator Cluster Survey 2003	2003	I	I	-	-	-	I	-	-	-	-	-	Public Commission for Health Care Planning (Libya), United Nations Children's Fund (UNICEF). Libya Multiple Indicator Cluster Survey 2003.	GHDx
195061	Luxembourg	Luxembourg Immunization Coverage Survey 2007-2008	2007 - 2008	-	-	I	I	I	I	-	I	I	I	-	Free University of Brussels (ULB), Ministry of Health (Luxembourg). Luxembourg Immunization Coverage Survey 2007-2008.	GHDx

GHDx ID	Country	Series	Year	BCG	DPT1	DPT3	HepB3	Hib3	MCV1	MCV2	PCV3	Pol3	RCV1	RotaC	Citation	Link
453457	Luxembourg	Luxembourg Immunization Coverage Survey 2018	2018	-	I	I	I	I	I	I	I	-	-	-	Ministry of Health (Luxembourg). Luxembourg Immunization Coverage Survey 2018.	GHDx
7843	Macedonia	Macedonia Multiple Indicator Cluster Survey 2005	2005	I	I	I	-	-	I	-	-	I	-	-	State Statistical Office (Macedonia) and United Nations Children's Fund (UNICEF). Macedonia Multiple Indicator Cluster Survey 2005. New York, United States: United Nations Children's Fund (UNICEF).	GHDx
91324	Macedonia	Macedonia Multiple Indicator Cluster Survey 2011	2011	I	I	I	-	I	I	-	-	I	-	-	Institute of Public Health (Macedonia), Ipsos Strategic Puls, Ministry of Education and Science (Macedonia), Ministry of Labor and Social Policy (Macedonia), United Nations Children's Fund (UNICEF). Macedonia Multiple Indicator Cluster Survey 2011. New York, United States of America: United Nations Children's Fund (UNICEF), 2013.	GHDx
124337	Macedonia	Macedonia Multiple Indicator Cluster Survey 1999	1999	I	-	I	-	-	-	-	-	-	-	-	Clinic for Children's Diseases, Clinic Center Skopje (Macedonia), Institute for Mother and Child Health, Health Home Skopje (Macedonia), Ministry of Health (Macedonia), National Nutrition Institute (Italy), Republic Institute for Health Protection (Macedonia), United Nations Children's Fund (UNICEF). Macedonia Multiple Indicator Cluster Survey 1999.	GHDx
7855	Madagascar	Madagascar Multiple Indicator Cluster Survey 1995	1995	I	-	I	-	-	I	-	-	I	-	-	National Institute of Statistics (Madagascar), United Nations Children's Fund (UNICEF). Madagascar Multiple Indicator Cluster Survey 1995.	GHDx
7876	Madagascar	Madagascar Household Priority Survey 1999	1999	I	-	I	-	-	I	-	-	I	-	-	National Institute of Statistics (Madagascar). Madagascar Household Priority Survey 1999.	GHDx
20202	Madagascar	Madagascar Demographic and Health Survey 1992	1992	I	I	I	-	-	I	-	-	I	-	-	Department of Applied Research for Development (Madagascar), Macro International, Inc. Madagascar Demographic and Health Survey 1992. Calverton, United States of America: Macro International, Inc.	GHDx
20212	Madagascar	Madagascar Demographic and Health Survey 1997	1997	I	I	I	-	-	I	-	-	I	-	-	Macro International, Inc, National Institute of Statistics (Madagascar). Madagascar Demographic and Health Survey 1997. Fairfax, United States of America: ICF International.	GHDx
20223	Madagascar	Madagascar Demographic and Health Survey 2003-2004	2003 - 2004	I	I	I	-	-	I	-	-	I	-	-	Macro International, Inc, National Institute of Statistics (Madagascar). Madagascar Demographic and Health Survey 2003-2004. Fairfax, United States of America: ICF International.	GHDx
21409	Madagascar	Madagascar Demographic and Health Survey 2008-2009	2008 - 2009	I	I	I	-	-	I	-	-	I	-	-	ICF Macro, National Institute of Statistics (Madagascar). Madagascar Demographic and Health Survey 2008-2009. Fairfax, United States of America: ICF International.	GHDx
27020	Madagascar	Madagascar Multiple Indicator Cluster Survey 2000	2000	I	E	E	-	-	I	-	-	I	-	-	National Institute of Statistics (Madagascar), United Nations Children's Fund (UNICEF). Madagascar Multiple Indicator Cluster Survey 2000. New York, United States of America: United Nations Children's Fund (UNICEF).	GHDx
231845	Madagascar	Madagascar Immunization Coverage Evaluation 2011	2011	I	I	I	I	I	I	-	-	I	-	-	Madagascar Immunization Coverage Evaluation 2011.	GHDx
231848	Madagascar	Madagascar Immunization Coverage Survey 2008	2008	I	I	I	I	-	I	-	-	I	-	-	Madagascar Immunization Coverage Survey 2008.	GHDx
399853	Madagascar	Madagascar Multiple Indicator Cluster Survey 2018	2018	I	I	I	I	I	I	-	I	I	-	I	National Institute of Statistics (Madagascar), United Nations Children's Fund (UNICEF). Madagascar Multiple Indicator Cluster Survey 2018. 2019.	GHDx
7907	Malawi	Malawi Multiple Indicator Cluster Survey 1995	1995	I	-	I	-	-	I	-	-	I	-	-	National Statistical Office of Malawi, United Nations Children's Fund (UNICEF). Malawi Multiple Indicator Cluster Survey 1995.	GHDx
7919	Malawi	Malawi Multiple Indicator Cluster Survey 2006	2006	I	I	I	I	I	I	-	-	I	-	-	United Nations Children's Fund (UNICEF), National Statistics Office (Malawi). Malawi Multiple Indicator Cluster Survey 2006. New York, United States: United Nations Children's Fund (UNICEF).	GHDx
20235	Malawi	Malawi Demographic and Health Survey 1992	1992	I	I	I	-	-	I	-	-	I	-	-	Macro International, Inc, National Statistical Office of Malawi. Malawi Demographic and Health Survey 1992. Fairfax, United States of America: ICF International.	GHDx
20252	Malawi	Malawi Demographic and Health Survey 2000	2000	I	I	I	-	-	I	-	-	I	-	-	Macro International, Inc, National Statistical Office of Malawi. Malawi Demographic and Health Survey 2000. Fairfax, United States of America: ICF International.	GHDx
20263	Malawi	Malawi Demographic and Health Survey 2004-2005	2004 - 2005	I	I	I	I	I	I	-	-	I	-	-	Macro International, Inc, National Statistical Office of Malawi. Malawi Demographic and Health Survey 2004-2005. Fairfax, United States of America: ICF International.	GHDx

GHDx ID	Country	Series	Year	BCG	DPT1	DPT3	HepB3	Hib3	MCV1	MCV2	PCV3	Pol3	RCV1	RotaC	Citation	Link
21393	Malawi	Malawi Demographic and Health Survey 2010	2010	I	I	I	I	I	I	-	-	I	-	-	ICF Macro, National Statistical Office of Malawi. Malawi Demographic and Health Survey 2010. Fairfax, United States of America: ICF International.	GHDx
26683	Malawi	Malawi Global Fund Household Health Coverage Survey 2007-2008	2007 - 2008	-	I	I	-	-	-	-	-	I	-	-	Ministry of Economic Planning and Development (Malawi), National Statistical Office of Malawi. Malawi Global Fund Household Health Coverage Survey 2007-2008.	GHDx
80790	Malawi	Malawi Knowledge, Attitudes and Practices in Health Survey 1996	1996	I	I	I	-	-	I	-	-	I	-	-	Macro International, Inc, National Statistical Office of Malawi. Malawi Knowledge, Attitudes and Practices in Health Survey 1996. Fairfax, United States of America: ICF International.	GHDx
161662	Malawi	Malawi Multiple Indicator Cluster Survey 2013-2014	2013 - 2014	I	I	I	I	I	I	-	I	I	-	I	National Statistical Office of Malawi, United Nations Children's Fund (UNICEF). Malawi Multiple Indicator Cluster Survey 2013-2014. New York, United States of America: United Nations Children's Fund (UNICEF), 2015.	GHDx
218581	Malawi	Malawi Demographic and Health Survey 2015-2016	2015 - 2016	I	I	I	I	I	I	E	I	I	-	I	Emory University and Centers for Disease Control & Prevention Collaboration, ICF International, Ministry of Health (Malawi), National Statistical Office of Malawi. Malawi Demographic and Health Survey 2015-2016. Fairfax, United States of America: ICF International, 2017.	GHDx
327852	Malawi	Malawi Integrated Household Survey 2016-2017	2016 - 2017	-	-	-	-	-	I	-	-	-	-	-	National Statistical Office. Malawi - Fourth Integrated Household Survey 2016-2017, Ref. MWI_2016_IHS-IV_v02_M. Dataset downloaded from [http://microdata.worldbank.org/index.php/catalog/2936] on [December 18, 2017].	GHDx
284410	Malaysia	Malaysia National Health and Morbidity Survey 2016	2016	I	I	I	I	I	I	-	-	I	I	-	Institute for Public Health, Ministry of Health (Malaysia), National Institutes of Health (NIH). Malaysia National Health and Morbidity Survey 2016.	GHDx
8033	Maldives	Maldives Multiple Indicator Cluster Survey 2001	2001	I	I	I	-	-	I	-	-	I	-	-	Ministry of Health (Maldives), Republic of Maldives, United Nations Children's Fund (UNICEF). Maldives Multiple Indicator Cluster Survey 2001.	GHDx
21311	Maldives	Maldives Demographic and Health Survey 2009	2009	I	I	I	I	-	I	-	-	I	-	-	ICF Macro, Ministry of Health (Maldives). Maldives Demographic and Health Survey 2009. Fairfax, United States of America: ICF International.	GHDx
90714	Maldives	Maldives Multiple Indicator Cluster Survey 1995	1995	I	-	I	-	-	I	-	-	I	-	-	United Nations Children's Fund (UNICEF). Maldives Multiple Indicator Cluster Survey 1995.	GHDx
218582	Maldives	Maldives Demographic and Health Survey 2016-2017	2016 - 2017	I	I	I	I	I	I	I	-	I	-	-	ICF International, Ministry of Health (Maldives). Maldives Demographic and Health Survey 2016-2017. Fairfax, United States of America: ICF International, 2019.	GHDx
8043	Mali	Mali Multiple Indicator Cluster Survey 1996	1996	I	-	I	-	-	I	-	-	I	-	-	National Directorate of Statistics and Informatics (DNSI) (Mali), United Nations Children's Fund (UNICEF). Mali Multiple Indicator Cluster Survey 1996.	GHDx
20274	Mali	Mali Demographic and Health Survey 2006	2006	I	I	I	E	-	I	-	-	I	-	-	Macro International, Inc, Ministry of Health (Mali), National Directorate of Statistics and Informatics (DNSI) (Mali). Mali Demographic and Health Survey 2006. Fairfax, United States of America: ICF International.	GHDx
20283	Mali	Mali Demographic and Health Survey 1987	1987	I	I	I	-	-	I	-	-	I	-	-	Sahel Institute, Westinghouse; Institute for Resource Development. Mali Demographic and Health Survey 1987. Fairfax, United States of America: ICF International.	GHDx
20301	Mali	Mali Demographic and Health Survey 1995-1996	1995 - 1996	I	I	I	-	-	I	-	-	I	-	-	Macro International, Inc, National Directorate of Statistics and Informatics (DNSI) (Mali), Planning and Statistics Unit, Ministry of Health (Mali). Mali Demographic and Health Survey 1995-1996. Fairfax, United States of America: ICF International.	GHDx
20315	Mali	Mali Demographic and Health Survey 2001	2001	I	I	I	-	-	I	-	-	I	-	-	Macro International, Inc, National Directorate of Statistics and Informatics (DNSI) (Mali), Planning and Statistics Unit, Ministry of Health (Mali). Mali Demographic and Health Survey 2001. Fairfax, United States of America: ICF International.	GHDx
77388	Mali	Mali Demographic and Health Survey 2012-2013	2012 - 2013	I	I	I	E	E	I	-	-	E	-	-	ICF International, INFO-STAT (Mali), Ministry of Health (Mali), National Institute of Statistics (INSTAT) (Mali), Planning and Statistics Unit, Ministry of Health (Mali). Mali Demographic and Health Survey 2012-2013. Fairfax, United States of America: ICF International, 2014.	GHDx
231841	Mali	Mali Evaluation of Routine Immunization Coverage 2009-2010	2009 - 2010	I	I	I	I	I	I	-	-	I	-	-	Ministry of Health (Mali). Mali Evaluation of Routine Immunization Coverage 2009-2010.	GHDx

GHDx ID	Country	Series	Year	BCG	DPT1	DPT3	HepB3	Hib3	MCV1	MCV2	PCV3	Pol3	RCV1	RotaC	Citation	Link
231842	Mali	Mali External EPI Review 2006	2006	I	I	I	-	-	I	-	-	I	-	-	Mali External EPI Review 2006.	GHDx
231844	Mali	Mali Immunization Coverage Survey 1990	1990	I	I	I	-	-	I	-	-	I	-	-	**CITATION MISSING**	GHDx
248224	Mali	Mali Multiple Indicator Cluster Survey 2015	2015	I	I	I	I	I	I	-	I	I	E	I	Ministry of Health (Mali), Ministry of Planning (Mali), National Institute of Statistics (INSTAT) (Mali), United Nations Children's Fund (UNICEF). Mali Multiple Indicator Cluster Survey 2015. New York, United States of America: United Nations Children's Fund (UNICEF), 2017.	GHDx
260407	Mali	Mali Agricultural Integrated Economic Survey 2014-2015	2014 - 2015	I	I	I	-	-	I	-	-	I	-	-	Ministry of Rural Development (Mali), National Institute of Statistics (INSTAT) (Mali), World Bank. Mali Agricultural Integrated Economic Survey 2014-2015. Washington DC, United States of America: World Bank.	GHDx
398033	Mali	Mali Demographic and Health Survey 2018	2018	I	I	I	I	I	I	-	I	I	-	I	ICF International, National Institute of Statistics (INSTAT) (Mali). Mali Demographic and Health Survey 2018. Fairfax, United States of America: ICF International, 2019.	GHDx
24152	Marshall Islands	Marshall Islands Community Survey 2006	2006	I	-	I	I	I	I	-	-	I	I	-	Economic Policy, Planning and Statistics Office (Marshall Islands). Marshall Islands Community Survey 2006.	GHDx
8102	Mauritania	Mauritania Multiple Indicator Cluster Survey 1996	1996	I	-	I	-	-	I	-	-	I	-	-	United Nations Children's Fund (UNICEF). Mauritania Multiple Indicator Cluster Survey 1996.	GHDx
8115	Mauritania	Mauritania Multiple Indicator Cluster Survey 2007	2007	I	I	I	-	-	I	-	-	I	-	-	National Office of Statistics (Mauritania), United Nations Children's Fund (UNICEF). Mauritania Multiple Indicator Cluster Survey 2007. New York, United States of America: United Nations Children's Fund (UNICEF).	GHDx
20322	Mauritania	Mauritania Demographic and Health Survey 2000-2001	2000 - 2001	I	I	I	-	-	I	-	-	I	-	-	Macro International, Inc, National Office of Statistics (Mauritania). Mauritania Demographic and Health Survey 2000-2001. Fairfax, United States of America: ICF International.	GHDx
152783	Mauritania	Mauritania Multiple Indicator Cluster Survey 2011	2011	I	I	I	I	I	I	-	-	I	-	-	National Office of Statistics (Mauritania), United Nations Children's Fund (UNICEF). Mauritania Multiple Indicator Cluster Survey 2011. New York, United States of America: United Nations Children's Fund (UNICEF), 2015.	GHDx
231813	Mauritania	Mauritania Immunization Coverage and Social Mobilization Survey 2004	2004	I	I	I	-	-	I	-	-	I	-	-	Mauritania Immunization Coverage and Social Mobilization Survey 2004.	GHDx
267343	Mauritania	Mauritania Multiple Indicator Cluster Survey 2015	2015	I	I	I	I	I	I	-	I	I	-	I	National Office of Statistics (Mauritania), United Nations Children's Fund (UNICEF). Mauritania Multiple Indicator Cluster Survey 2015. New York, United States of America: United Nations Children's Fund (UNICEF), 2018.	GHDx
440420	Mauritania	Mauritania National Vaccine Coverage Survey 1999	1999	-	I	I	-	-	I	-	-	I	-	-	World Health Organization (WHO). Mauritania National Vaccine Coverage Survey 1999.	GHDx
8442	Mexico	Mexico Family Life Survey 2002	2002	I	I	E	-	I	I	-	-	I	I	-	Center for Research and Teaching in Economics (CIDE) (Mexico), Ibero-American University, National Institute of Perinatology (Mexico), National Institute of Statistics and Geography (INEGI) (Mexico). Mexico Family Life Survey 2002.	GHDx
8618	Mexico	Mexico National Survey of Health and Nutrition 2005-2006	2005 - 2006	I	I	I	-	I	I	-	-	I	I	-	National Institute of Public Health (Mexico). Mexico National Survey of Health and Nutrition 2005-2006. Cuernavaca, Mexico: National Institute of Public Health (Mexico).	GHDx
8684	Mexico	Mexico National Performance Evaluation Survey 2002-2003	2002 - 2003	-	I	E	-	-	I	-	-	-	-	-	National Institute of Public Health (Mexico), Secretariat of Health (Mexico), World Health Organization (WHO). Mexico National Performance Evaluation Survey 2002-2003.	GHDx
20326	Mexico	Mexico Demographic and Health Survey 1987	1987	I	I	I	-	-	I	-	-	I	-	-	Macro Systems, Inc.; Institute for Resource Development, Secretariat of Health (Mexico). Mexico Demographic and Health Survey 1987. Fairfax, United States: ICF International.	GHDx
81748	Mexico	Mexico National Survey of Health and Nutrition 2011-2012	2011 - 2012	I	I	I	I	I	I	-	I	I	I	-	National Institute of Public Health (Mexico). Mexico National Survey of Health and Nutrition 2011-2012. Cuernavaca, Mexico: National Institute of Public Health (Mexico).	GHDx
129896	Mexico	Mexico INEGI Information Bank - Proportion of Children	1990	-	-	-	-	-	I	-	-	-	-	-	National Institute of Statistics and Geography (INEGI) (Mexico). Mexico INEGI Information Bank - Proportion of Children Under 1 Year Vaccinated Against Measles. Mexico City, Mexico: National Institute of Statistics and Geography (INEGI) (Mexico).	GHDx

GHDx ID	Country	Series	Year	BCG	DPT1	DPT3	HepB3	Hib3	MCV1	MCV2	PCV3	Pol3	RCV1	RotaC	Citation	Link
		Under 1 Year Vaccinated Against Measles														
130481	Mexico	Mexico Vaccinations for Under 1-year and Age 1-4 years 1998-2012	1998 - 2012	I	-	I	I	I	I	-	I	I	I	-	Secretariat of Health (Mexico). Mexico Vaccinations for Under 1-year and Age 1-4 years 1998-2012.	GHDx
264590	Mexico	Mexico Multiple Indicator Cluster Survey 2015	2015	I	I	I	-	I	I	-	-	I	-	I	National Institute of Public Health (Mexico), United Nations Children's Fund (UNICEF). Mexico Multiple Indicator Cluster Survey 2015. New York, United States of America: United Nations Children's Fund (UNICEF), 2017.	GHDx
8734	Moldova	Moldova Multiple Indicator Cluster Survey 2000	2000	I	I	I	I	-	I	-	-	I	-	-	National Scientific and Applied Center for Preventive Medicine (NCPM) (Moldova), United Nations Children's Fund (UNICEF). Moldova Multiple Indicator Cluster Survey 2000. New York, United States: United Nations Children's Fund (UNICEF).	GHDx
20339	Moldova	Moldova Demographic and Health Survey 2005	2005	I	I	I	I	-	I	-	-	I	-	-	Macro International, Inc, National Scientific and Applied Center for Preventive Medicine (Moldova). Moldova Demographic and Health Survey 2005. Fairfax, United States of America: ICF International.	GHDx
140200	Moldova	Moldova Multiple Indicator Cluster Survey 2012	2012	I	I	I	I	-	I	-	-	I	I	-	Ministry of Health (Moldova), National Bureau of Statistics (Moldova), United Nations Children's Fund (UNICEF). Moldova Multiple Indicator Cluster Survey 2012. New York, United States of America: United Nations Children's Fund (UNICEF), 2015.	GHDx
8777	Mongolia	Mongolia Multiple Indicator Cluster Survey 2005	2005	I	I	I	-	-	I	-	-	I	-	-	National Statistical Office of Mongolia, United Nations Children's Fund (UNICEF). Mongolia Multiple Indicator Cluster Survey 2005. New York, United States of America: United Nations Children's Fund (UNICEF).	GHDx
8788	Mongolia	Mongolia Multiple Indicator Cluster Survey 2000	2000	I	I	I	-	-	I	-	-	I	-	-	National Statistical Office of Mongolia. Mongolia Multiple Indicator Cluster Survey 2000. New York, United States of America: United Nations Children's Fund (UNICEF).	GHDx
76704	Mongolia	Mongolia Multiple Indicator Cluster Survey 2010	2010	I	I	I	-	-	I	-	-	I	I	-	National Statistical Office of Mongolia, United Nations Children's Fund (UNICEF). Mongolia Multiple Indicator Cluster Survey 2010. New York, United States of America: United Nations Children's Fund (UNICEF), 2013.	GHDx
150866	Mongolia	Mongolia Multiple Indicator Cluster Survey 2013	2013	I	I	I	I	I	I	-	-	I	-	-	Government of Mongolia, National Statistical Office of Mongolia, United Nations Children's Fund (UNICEF), United Nations Population Fund (UNFPA). Mongolia Multiple Indicator Cluster Survey 2013. New York, United States of America: United Nations Children's Fund (UNICEF), 2016.	GHDx
427952	Mongolia	Mongolia Multiple Indicator Cluster Survey 2018	2018	I	I	I	I	I	I	I	-	I	I	-	Government of Mongolia, National Statistical Office of Mongolia, United Nations Children's Fund (UNICEF), United Nations Population Fund (UNFPA). Mongolia Multiple Indicator Cluster Survey 2018. New York, United States of America: United Nations Children's Fund (UNICEF), 2019.	GHDx
150526	Montenegro	Montenegro Multiple Indicator Cluster Survey 2013	2013	I	I	I	I	I	I	-	-	I	I	-	Statistical Office of Montenegro. Montenegro Multiple Indicator Cluster Survey 2013. New York, United States of America: United Nations Children's Fund (UNICEF), 2015.	GHDx
8829	Morocco	Morocco Maternal and Child Health Survey 1996-1997	1996 - 1997	I	I	I	-	-	I	-	-	I	-	-	Ministry of Health (Morocco), League of Arab States. Morocco Maternal and Child Health Survey 1996-1997.	GHDx
20347	Morocco	Morocco Demographic and Health Survey 1987	1987	I	I	I	-	-	I	-	-	I	-	-	Ministry of Public Health (Morocco), Westinghouse; Institute for Resource Development. Morocco Demographic and Health Survey 1987. Fairfax, United States of America: ICF International.	GHDx
20361	Morocco	Morocco Demographic and Health Survey 2003-2004	2003 - 2004	I	I	I	-	-	I	-	-	I	-	-	League of Arab States, Macro International, Inc, Ministry of Health (Morocco). Morocco Demographic and Health Survey 2003-2004. Fairfax, United States of America: ICF International.	GHDx
20371	Morocco	Morocco Demographic and Health Survey 1992	1992	I	I	I	-	-	I	-	-	I	-	-	Macro International, Inc, Ministry of Public Health (Morocco). Morocco Demographic and Health Survey 1992. Fairfax, United States of America: ICF International.	GHDx
126909	Morocco	Morocco National Survey on Population and Family Health 2010-2011	2010 - 2011	I	I	I	I	I	I	-	-	I	-	-	Ministry of Health (Morocco), Pan Arab Project for Family Health (PAPFAM), United Nations Children's Fund (UNICEF), United Nations Population Fund (UNFPA), World Health Organization (WHO). Morocco National Survey on Population and Family Health 2010-2011.	GHDx

GHDx ID	Country	Series	Year	BCG	DPT1	DPT3	HepB3	Hib3	MCV1	MCV2	PCV3	Pol3	RCV1	RotaC	Citation	Link
231861	Morocco	Morocco National Immunization Coverage Survey 1989	1989	I	I	I	-	-	I	-	-	I	-	-	Morocco National Immunisation Coverage Survey 1989.	GHDx
231862	Morocco	Morocco National Immunization Coverage Survey 1988	1988	I	I	I	-	-	I	-	-	I	-	-	Morocco National Immunisation Coverage Survey 1988.	GHDx
423654	Morocco	Morocco National Survey on Population and Family Health 2017-2018	2017 - 2018	I	I	I	I	I	I	-	-	I	-	-	Ministry of Health (Morocco), Pan Arab Project for Family Health (PAPFAM), United Nations Children's Fund (UNICEF), United Nations Population Fund (UNFPA), World Health Organization (WHO). Morocco National Survey on Population and Family Health 2017-2018.	GHDx
8881	Mozambique	Mozambique Multiple Indicator Cluster Survey 1995	1995	I	-	I	-	-	I	-	-	I	-	-	Ministry of Health (Mozambique), National Institute of Statistics (INE) (Mozambique), United Nations Children's Fund (UNICEF). Mozambique Multiple Indicator Cluster Survey 1995.	GHDx
20382	Mozambique	Mozambique Demographic and Health Survey 1997	1997	I	I	I	-	-	I	-	-	I	-	-	Macro International, Inc, National Institute of Statistics (INE) (Mozambique). Mozambique Demographic and Health Survey 1997. Fairfax, United States of America: ICF International.	GHDx
20394	Mozambique	Mozambique Demographic and Health Survey 2003-2004	2003 - 2004	I	I	I	-	-	I	-	-	I	-	-	Macro International, Inc, National Institute of Statistics (INE) (Mozambique). Mozambique Demographic and Health Survey 2003-2004. Fairfax, United States of America: ICF International.	GHDx
27031	Mozambique	Mozambique Multiple Indicator Cluster Survey 2008-2009	2008 - 2009	I	I	I	-	-	I	-	-	I	-	-	United Nations Children's Fund (UNICEF), National Statistics Institute (Mozambique). Mozambique Multiple Indicator Cluster Survey 2008-2009. New York, United States: United Nations Children's Fund (UNICEF).	GHDx
55975	Mozambique	Mozambique Demographic and Health Survey 2011	2011	I	I	I	I	I	I	-	-	I	-	-	ICF Macro, Manhica Health Research Center (CISM), Ministry of Health (Mozambique), National Institute of Statistics (INE) (Mozambique). Mozambique Demographic and Health Survey 2011. Fairfax, United States of America: ICF International.	GHDx
157060	Mozambique	Mozambique AIDS Indicator Survey 2015	2015	I	I	I	-	-	I	-	I	I	-	-	Centers for Disease Control and Prevention (CDC), ICF International, Ministry of Health (Mozambique), National Institute of Health (Mozambique), National Institute of Statistics (INE) (Mozambique). Mozambique AIDS Indicator Survey 2015. Fairfax, United States of America: ICF International, 2018.	GHDx
8922	Myanmar	Myanmar Multiple Indicator Cluster Survey 1995	1995	I	-	I	-	-	I	-	-	I	-	-	Ministry of Health (Myanmar), United Nations Children's Fund (UNICEF). Myanmar Multiple Indicator Cluster Survey 1995.	GHDx
8932	Myanmar	Myanmar Multiple Indicator Cluster Survey 2000	2000	I	I	I	-	-	I	-	-	I	-	-	Ministry of Health (Myanmar), United Nations Children's Fund (UNICEF). Myanmar Multiple Indicator Cluster Survey 2000. New York, United States of America: United Nations Children's Fund (UNICEF).	GHDx
40237	Myanmar	Myanmar Fertility and Reproductive Health Survey 2006-2007	2006 - 2007	I	-	I	I	-	I	-	-	I	-	-	Ministry of Immigration and Population (Myanmar), United Nations Population Fund (UNFPA). Myanmar Fertility and Reproductive Health Survey 2006-2007.	GHDx
90696	Myanmar	Myanmar Multiple Indicator Cluster Survey 2009-2010	2009 - 2010	I	I	I	I	-	I	-	-	I	-	-	Ministry of Health (Myanmar), Ministry of National Planning and Economic Development (Myanmar), United Nations Children's Fund (UNICEF). Myanmar Multiple Indicator Cluster Survey 2009-2010.	GHDx
141910	Myanmar	Myanmar Multiple Indicator Cluster Survey 2003	2003	I	I	I	-	-	I	-	-	I	-	-	Ministry of Health (Myanmar), United Nations Children's Fund (UNICEF). Myanmar Multiple Indicator Cluster Survey 2003.	GHDx
157061	Myanmar	Myanmar Demographic and Health Survey 2015-2016	2015 - 2016	I	I	I	I	-	I	-	-	I	-	-	ICF International, Ministry of Health and Sports (Myanmar). Myanmar Demographic and Health Survey 2015-2016. Fairfax, United States of America: ICF International, 2017.	GHDx
231817	Myanmar	Monitoring Progress Towards the Goals of National Program of Action for Myanmar's Children 1997	1997	I	-	I	-	-	I	-	-	I	-	-	Monitoring Progress Towards the Goals of National Program of Action for Myanmar's Children 1997.	GHDx
231821	Myanmar	Myanmar EPI Coverage Evaluation Survey 1991	1991	I	I	I	-	-	I	-	-	I	-	-	United Nations Children's Fund (UNICEF). Myanmar EPI Coverage Evaluation Survey 1991.	GHDx

GHDx ID	Country	Series	Year	BCG	DPT1	DPT3	HepB3	Hib3	MCV1	MCV2	PCV3	Pol3	RCV1	RotaC	Citation	Link
231824	Myanmar	Myanmar EPI Review 1986-1987	1986 - 1987	I	I	I	-	-	I	-	-	I	-	-	Government of Myanmar, United Nations Children's Fund (UNICEF), World Health Organization (WHO). Myanmar EPI Review 1986-1987.	GHDx
20404	Namibia	Namibia Demographic and Health Survey 1992	1992	I	I	I	-	-	I	-	-	I	-	-	Central Statistics Office (Namibia), Macro International, Inc, Ministry of Health and Social Services (Namibia). Namibia Demographic and Health Survey 1992. Fairfax, United States of America: ICF International.	GHDx
20417	Namibia	Namibia Demographic and Health Survey 2000	2000	I	I	I	-	-	I	-	-	I	-	-	Macro International, Inc, Ministry of Health and Social Services (Namibia), National Planning Commission (Namibia). Namibia Demographic and Health Survey 2000. Fairfax, United States of America: ICF International.	GHDx
20428	Namibia	Namibia Demographic and Health Survey 2006-2007	2006 - 2007	I	I	I	-	-	I	-	-	I	-	-	Macro International, Inc, Ministry of Health and Social Services (Namibia). Namibia Demographic and Health Survey 2006-2007. Fairfax, United States of America: ICF International.	GHDx
150382	Namibia	Namibia Demographic and Health Survey 2013	2013	I	I	I	I	I	I	-	-	I	-	-	ICF International, Ministry of Health and Social Services (Namibia), Namibia Institute of Pathology, Namibia Statistics Agency. Namibia Demographic and Health Survey 2013. Fairfax, United States of America: ICF International.	GHDx
231812	Namibia	Namibia Post Measles Supplemental Immunization and EPI Coverage Survey 2012	2012	I	I	I	I	I	I	-	-	I	-	-	Namibia Post Measles Supplemental Immunization and EPI Coverage Survey 2012.	GHDx
21436	Nauru	Nauru Demographic and Health Survey 2007	2007	I	I	I	-	-	I	-	-	I	-	-	Nauru Bureau of Statistics, Secretariat of the Pacific Community (SPC), Macro International, Inc., Nauru Demographic and Health Survey 2007. Noumea, New Caledonia, France: Secretariat of the Pacific Community (SPC).	GHDx
9002	Nepal	Nepal Fertility, Family Planning, and Health Survey 1991-1992	1991 - 1992	I	I	I	-	-	I	-	-	I	-	-	Ministry of Health (Nepal). Nepal Fertility, Family Planning, and Health Survey 1991-1992.	GHDx
20437	Nepal	Nepal Demographic and Health Survey 1996	1996	I	I	I	-	-	I	-	-	I	-	-	Macro International, Inc, Ministry of Health and Population (Nepal), New ERA. Nepal Demographic and Health Survey 1996. Fairfax, United States of America: ICF International.	GHDx
20450	Nepal	Nepal Demographic and Health Survey 2001	2001	I	I	I	-	-	I	-	-	I	-	-	Macro International, Inc, Ministry of Health and Population (Nepal), New ERA. Nepal Demographic and Health Survey 2001. Fairfax, United States of America: ICF International.	GHDx
20462	Nepal	Nepal Demographic and Health Survey 2006	2006	I	I	I	I	-	I	-	-	I	-	-	Macro International, Inc, Ministry of Health and Population (Nepal), New ERA. Nepal Demographic and Health Survey 2006. Fairfax, United States of America: ICF International.	GHDx
21240	Nepal	Nepal Demographic and Health Survey 2011	2011	I	I	I	I	I	I	-	-	I	-	-	ICF Macro, Ministry of Health and Population (Nepal), New ERA. Nepal Demographic and Health Survey 2011. Fairfax, United States of America: ICF International.	GHDx
25030	Nepal	Nepal Intercensal Household Information Monitoring and Evaluation System 2000	2000	I	I	I	-	-	I	-	-	I	-	-	Central Bureau of Statistics (Nepal). Nepal Intercensal Household Information Monitoring and Evaluation System 2000.	GHDx
141917	Nepal	Nepal Multiple Indicator Cluster Survey 1995-1998	1995 - 1998	I	I	I	-	-	I	-	-	I	-	-	Community Information and Epidemiological Technologies (CIET), National Planning Commission (Nepal), New ERA, United Nations Children's Fund (UNICEF). Nepal Multiple Indicator Cluster Survey 1995-1998. New York, United States: United Nations Children's Fund (UNICEF).	GHDx
153587	Nepal	Nepal - Chitwan Valley Family Study 1996-1997	1996 - 1997	I	I	I	-	-	I	-	-	I	-	-	Institute for Social Research, University of Michigan. Nepal - Chitwan Valley Family Study 1996-1997.	GHDx
162317	Nepal	Nepal Multiple Indicator Cluster Survey 2014	2014	I	I	I	I	I	I	-	-	I	-	-	Central Bureau of Statistics (Nepal), United Nations Children's Fund (UNICEF). Nepal Multiple Indicator Cluster Survey 2014. New York, United States of America: United Nations Children's Fund (UNICEF), 2015.	GHDx
231789	Nepal	Nepal Immunization Coverage Survey 2009	2009	I	I	I	-	-	I	-	-	I	-	-	Ministry of Health and Population (Nepal). Nepal Immunization Coverage Survey 2009.	GHDx
286782	Nepal	Nepal Demographic and Health Survey 2016-2017	2016 - 2017	I	I	I	I	I	I	-	I	I	-	-	ICF International, Ministry of Health (Nepal), New ERA. Nepal Demographic and Health Survey 2016-2017. Fairfax, United States of America: ICF International, 2017.	GHDx

GHDx ID	Country	Series	Year	BCG	DPT1	DPT3	HepB3	Hib3	MCV1	MCV2	PCV3	Pol3	RCV1	RotaC	Citation	Link
9270	Nicaragua	Nicaragua Reproductive Health Survey 2006-2007	2006 - 2007	I	I	I	I	I	I	-	-	I	I	-	Division of Reproductive Health, Centers for Disease Control and Prevention (CDC), National Institute for Development Information (Nicaragua). Nicaragua Reproductive Health Survey 2006-2007. Managua, Nicaragua: National Institute for Development Information (Nicaragua).	GHDx
9278	Nicaragua	Nicaragua Reproductive Health Survey 1992-1993	1992 - 1993	I	-	I	-	-	I	-	-	I	-	-	Profamilia and Division of Reproductive Health-Centers for Disease Control and Prevention (CDC). (1993) Nicaragua Reproductive Health Survey 1992-1993. Profamilia, Managua, Nicaragua.	GHDx
9310	Nicaragua	Nicaragua Living Standards Measurement Survey 1993	1993	-	I	I	-	-	I	E	-	I	-	-	National Institute of Statistics and Censuses (Nicaragua), World Bank. Nicaragua Living Standards Measurement Survey 1993.	GHDx
9370	Nicaragua	Nicaragua Living Standards Measurement Survey 1998-1999	1998 - 1999	-	I	I	-	-	I	E	-	I	-	-	National Institute of Statistics and Censuses (Nicaragua), World Bank. Nicaragua Living Standards Measurement Survey 1998-1999.	GHDx
9422	Nicaragua	Nicaragua Living Standards Measurement Survey 2001	2001	I	I	I	-	-	I	-	-	I	E	-	National Institute of Statistics and Censuses (Nicaragua), World Bank. Nicaragua Living Standards Measurement Survey 2001.	GHDx
20478	Nicaragua	Nicaragua Demographic and Health Survey 1997-1998	1997 - 1998	I	I	I	-	-	I	-	-	I	-	-	Macro International, Inc, Ministry of Health (Nicaragua), National Institute of Statistics and Censuses (Nicaragua). Nicaragua Demographic and Health Survey 1997-1998. Fairfax, United States of America: ICF International.	GHDx
20487	Nicaragua	Nicaragua Demographic and Health Survey 2001	2001	I	I	I	-	-	I	-	-	I	I	-	Macro International, Inc, Ministry of Health (Nicaragua), National Institute of Statistics and Censuses (Nicaragua). Nicaragua Demographic and Health Survey 2001. Fairfax, United States of America: ICF International.	GHDx
9439	Niger	Niger Multiple Indicator Cluster Survey 2000	2000	I	I	I	-	-	I	-	-	I	-	-	Government of Niger, Macro International, Inc, United Nations Children's Fund (UNICEF). Niger Multiple Indicator Cluster Survey 2000. New York, United States of America: United Nations Children's Fund (UNICEF).	GHDx
9465	Niger	Niger Continuous Survey on Economic and Social Conditions 1995-1996	1995	I	I	I	-	-	I	-	-	I	-	-	Department of Statistics and National Accounts (Niger), United Nations Department of Economic and Social Development. Niger Continuous Survey on Economic and Social Conditions 1995-1996. Niamey, Niger: Department of Statistics and National Accounts (Niger).	GHDx
9469	Niger	Niger Continuous Survey on Economic and Social Conditions 1994	1994	I	I	I	-	-	I	-	-	I	-	-	Department of Statistics and National Accounts (Niger), United Nations Department of Economic and Social Development. Niger Continuous Survey on Economic and Social Conditions 1994. Niamey, Niger: Department of Statistics and National Accounts (Niger).	GHDx
9477	Niger	Niger Multiple Indicator Cluster Survey 1996	1996	I	-	I	-	-	I	-	-	I	-	-	Centers for Disease Control and Prevention (CDC), Government of Niger, United Nations Children's Fund (UNICEF), United Nations Population Fund (UNFPA). Niger Multiple Indicator Cluster Survey 1996.	GHDx
20499	Niger	Niger Demographic and Health Survey 2006	2006	I	I	I	-	-	I	-	-	I	-	-	Department of Statistics and National Accounts (Niger), Macro International, Inc. Niger Demographic and Health Survey 2006. Fairfax, United States of America: ICF International.	GHDx
20518	Niger	Niger Demographic and Health Survey 1992	1992	I	I	I	-	-	I	-	-	I	-	-	Department of Statistics and National Accounts (Niger), Macro International, Inc. Niger Demographic and Health Survey 1992. Fairfax, United States of America: ICF International.	GHDx
20537	Niger	Niger Demographic and Health Survey 1998	1998	I	I	I	-	-	I	-	-	I	-	-	CARE International, Macro International, Inc. Niger Demographic and Health Survey 1998. Fairfax, United States of America: ICF International.	GHDx
74393	Niger	Niger Demographic and Health Survey 2012	2012	I	I	I	I	I	I	-	-	I	-	-	ICF International, Ministry of Public Health (Niger), National Institute of Statistics (Niger). Niger Demographic and Health Survey 2012. Fairfax, United States of America: ICF International.	GHDx
160040	Niger	Niger Nutrition and Child Survival Survey 2010	2010	I	I	I	-	-	I	-	-	I	-	-	Ministry of Health (Niger), National Institute of Statistics (Niger). Niger Nutrition and Child Survival Survey 2010.	GHDx
160053	Niger	Niger Nutrition and Child Survival Survey 2009	2009	I	I	I	-	-	I	-	-	-	-	-	Ministry of Public Health (Niger), National Institute of Statistics (Niger). Niger Nutrition and Child Survival Survey 2009.	GHDx
160198	Niger	Niger Nutrition and Child Survival Survey 2008	2008	-	I	I	-	-	I	-	-	-	-	-	Ministry of Public Health (Niger), National Institute of Statistics (Niger). Niger Nutrition and Child Survival Survey 2008.	GHDx
9504	Nigeria	Nigeria Multiple Indicator Cluster Survey 1995	1995	I	-	I	-	-	I	-	-	I	-	-	Federal Office of Statistics (Nigeria), United Nations Children's Fund (UNICEF). Nigeria Multiple Indicator Cluster Survey 1995.	GHDx
9506	Nigeria	Nigeria Multiple Indicator Cluster Survey 1999	1999	I	I	I	-	-	I	-	-	I	-	-	National Bureau of Statistics (Nigeria), United Nations Children's Fund (UNICEF). Nigeria Multiple Indicator Cluster Survey 1999. Abuja, Nigeria: National Bureau of Statistics (Nigeria).	GHDx

GHDx ID	Country	Series	Year	BCG	DPT1	DPT3	HepB3	Hib3	MCV1	MCV2	PCV3	Pol3	RCV1	RotaC	Citation	Link
9516	Nigeria	Nigeria Multiple Indicator Cluster Survey 2007	2007	I	I	I	-	-	I	-	-	I	-	-	United Nations Children's Fund (UNICEF), National Bureau of Statistics (Nigeria). Nigeria Multiple Indicator Cluster Survey 2007. New York, United States: United Nations Children's Fund (UNICEF).	GHDx
20552	Nigeria	Nigeria Demographic and Health Survey 1990	1990	I	I	I	-	-	I	-	-	I	-	-	Federal Office of Statistics (Nigeria), Institute for Resource Development, Macro Systems. Nigeria Demographic and Health Survey 1990. Fairfax, United States of America: ICF International.	GHDx
20555	Nigeria	Nigeria Demographic and Health Survey 1999	1999	I	I	I	-	-	I	-	-	I	-	-	Macro International, Inc, National Population Commission of Nigeria. Nigeria Demographic and Health Survey 1999. Calverton, United States of America: Macro International, Inc.	GHDx
20567	Nigeria	Nigeria Demographic and Health Survey 2003	2003	I	I	I	-	-	I	-	-	I	-	-	Department for International Development (DFiD) (United Kingdom), National Population Commission of Nigeria, ORC Macro, United Nations Children's Fund (UNICEF), United Nations Population Fund (UNFPA). Nigeria Demographic and Health Survey 2003. Fairfax, United States of America: ICF International.	GHDx
20573	Nigeria	Nigeria - Ondo Special Demographic and Health Survey 1986-1987	1986 - 1987	I	I	I	-	-	I	-	-	I	-	-	Federal Ministry of Health (Nigeria), Macro Systems, Inc.; Institute for Resource Development, National Population Bureau (Nigeria). Nigeria - Ondo Special Demographic and Health Survey 1986-1987. Fairfax, United States of America: ICF International.	GHDx
21433	Nigeria	Nigeria Demographic and Health Survey 2008	2008	I	I	I	-	-	I	-	-	I	-	-	Macro International, Inc, National Population Commission of Nigeria. Nigeria Demographic and Health Survey 2008. Fairfax, United States of America: ICF International, 2009.	GHDx
33188	Nigeria	Nigeria Annual Abstract of Statistics 2009	2003 - 2008	I	I	I	-	-	I	-	-	I	-	-	National Bureau of Statistics (Nigeria). Nigeria Annual Abstract of Statistics 2009. Abuja, Nigeria: National Bureau of Statistics (Nigeria).	GHDx
58333	Nigeria	Nigeria Integrated Baseline Survey 1995	1995	I	-	I	-	-	I	-	-	-	-	-	Basic Support for Institutionalizing Child Survival (BASICS), Centers for Disease Control and Prevention (CDC), Johns Hopkins University, United States Agency for International Development (USAID). Nigeria Integrated Baseline Survey 1995.	GHDx
76703	Nigeria	Nigeria Multiple Indicator Cluster Survey 2011	2011	I	I	I	I	-	I	-	-	I	-	-	National Bureau of Statistics (Nigeria), United Nations Children's Fund (UNICEF). Nigeria Multiple Indicator Cluster Survey 2011. New York, United States of America: United Nations Children's Fund (UNICEF), 2013.	GHDx
77390	Nigeria	Nigeria Demographic and Health Survey 2013	2013	I	I	I	I	I	I	-	-	I	-	-	ICF International, National Population Commission of Nigeria. Nigeria Demographic and Health Survey 2013. Fairfax, United States of America: ICF International.	GHDx
151719	Nigeria	Nigeria Living Standards Survey 2008-2010	2008 - 2010	I	I	I	-	-	E	-	-	I	-	-	National Bureau of Statistics (Nigeria). Nigeria Living Standards Survey 2008-2010. Abuja, Nigeria: National Bureau of Statistics (Nigeria).	GHDx
151732	Nigeria	Nigeria Standardized Monitoring and Assessment of Relief and Transitions Survey, Round V 2013	2013	-	I	I	-	-	I	-	-	-	-	-	National Bureau of Statistics (Nigeria), United Nations Children's Fund (UNICEF). Nigeria Standardized Monitoring and Assessment of Relief and Transitions Survey, Round V 2013.	GHDx
151733	Nigeria	Nigeria Standardized Monitoring and Assessment of Relief and Transitions Survey, Round V 2013 - Women and Children Tabulations	2013	-	I	I	-	-	I	-	-	-	-	-	National Bureau of Statistics (Nigeria), United Nations Children's Fund (UNICEF). Nigeria Standardized Monitoring and Assessment of Relief and Transitions Survey, Round V 2013 - Women and Children Tabulations.	GHDx
151797	Nigeria	Nigeria General Household Survey 2012-2013	2012 - 2013	I	I	E	-	-	I	-	-	I	-	-	National Bureau of Statistics (Nigeria). Nigeria General Household Survey 2012-2013. Washington DC, United States of America: World Bank.	GHDx
218613	Nigeria	Nigeria Multiple Indicator Cluster Survey with National Immunization Coverage Survey Supplement 2016-2017	2016 - 2017	I	I	I	I	I	I	-	-	I	-	-	National Agency for the Control of AIDS (Nigeria), National Bureau of Statistics (Nigeria), National Primary Health Care Development Agency (NPHCDA) (Nigeria), United Nations Children's Fund (UNICEF). Nigeria Multiple Indicator Cluster Survey with National Immunization Coverage Survey Supplement 2016-2017. New York, United States of America: United Nations Children's Fund (UNICEF), 2018.	GHDx
231794	Nigeria	Nigeria National Immunization Coverage Survey 2010	2010	I	I	I	-	-	I	-	-	I	-	-	National Primary Health Care Development Agency (NPHCDA) (Nigeria). Nigeria National Immunization Coverage Survey 2010.	GHDx

GHDx ID	Country	Series	Year	BCG	DPT1	DPT3	HepB3	Hib3	MCV1	MCV2	PCV3	Pol3	RCV1	RotaC	Citation	Link
231801	Nigeria	Nigeria National Immunization Coverage Survey 2006	2006	I	I	I	I	-	I	-	-	I	-	-	World Health Organization (WHO). Nigeria National Immunization Coverage Survey 2006.	GHDx
231803	Nigeria	Nigeria National Immunization Coverage Survey 2003	2003	I	I	I	-	-	I	-	-	I	-	-	World Health Organization (WHO). Nigeria National Immunization Coverage Survey 2003.	GHDx
253019	Nigeria	Nigeria Report on Children, Adolescents and Youth	1991 - 1999	I	I	I	-	-	I	-	-	I	-	-	National Population Commission of Nigeria. Nigeria Report on Children, Adolescents and Youth. Abuja, Nigeria: National Population Commission of Nigeria.	GHDx
274707	Nigeria	Nigeria National Nutrition and Health Survey 2015	2015	-	I	I	-	-	I	-	-	-	-	-	National Bureau of Statistics (Nigeria). Nigeria National Nutrition and Health Survey 2015.Â v1.0.	GHDx
274708	Nigeria	Nigeria National Nutrition and Health Survey 2014	2014	-	I	I	-	-	I	-	-	-	-	-	National Bureau of Statistics (Nigeria). National Nutrition and Health Survey 2014-v1.0.	GHDx
408484	Nigeria	Nigeria Demographic and Health Survey 2018	2018	I	I	I	I	I	I	E	I	I	-	-	Federal Ministry of Health (Nigeria), ICF International, National Population Commission (NPC). Nigeria Demographic and Health Survey 2018. Fairfax, United States of America: ICF International, 2020.	GHDx
7465	North Korea	North Korea Multiple Indicator Cluster Survey 1998	1998	I	-	I	-	-	I	-	-	I	-	-	European Union (EU), Government of North Korea, United Nations Children's Fund (UNICEF), World Food Programme (WFP). North Korea Multiple Indicator Cluster Survey 1998.	GHDx
7467	North Korea	North Korea Multiple Indicator Cluster Survey 2000	2000	I	I	I	-	-	I	-	-	I	-	-	Central Bureau of Statistics (North Korea), United Nations Children's Fund (UNICEF). North Korea Multiple Indicator Cluster Survey 2000.	GHDx
58950	North Korea	North Korea EPI Coverage Evaluation Survey 2008	2008	I	I	I	I	E	I	-	-	I	-	-	Ministry of Public Health (North Korea), United Nations Children's Fund (UNICEF), World Health Organization (WHO). North Korea EPI Coverage Evaluation Survey 2008.	GHDx
58954	North Korea	North Korea Nutrition Assessment 2004	2004	I	-	I	-	-	I	-	-	I	-	-	Central Bureau of Statistics (North Korea), United Nations Children's Fund (UNICEF), World Food Programme (WFP). North Korea Nutrition Assessment 2004.	GHDx
355051	North Korea	North Korea Multiple Indicator Cluster Survey 2017	2017	I	I	I	I	I	I	I	-	I	-	-	Central Bureau of Statistics (North Korea), United Nations Children's Fund (UNICEF). North Korea Multiple Indicator Cluster Survey 2017.	GHDx
9585	Pakistan	Pakistan Fertility and Family Planning Survey 1996-1997	1996 - 1997	I	I	I	-	-	I	-	-	I	-	-	Centre for Population Studies, London School of Hygiene and Tropical Medicine, National Institute of Population Studies (Pakistan). Pakistan Fertility and Family Planning Survey 1996-1997.	GHDx
9602	Pakistan	Pakistan Integrated Household Survey 1995-1996	1995 - 1996	I	I	I	-	-	I	-	-	I	-	-	Pakistan Bureau of Statistics. Pakistan Integrated Household Survey 1995-1996.	GHDx
9658	Pakistan	Pakistan Integrated Household Survey/ Household Integrated Economic Survey 1998-1999	1998 - 1999	I	I	I	-	-	I	-	-	I	-	-	Federal Bureau of Statistics (Pakistan). Pakistan Integrated Household Survey 1998-1999. Islamabad, Pakistan: Federal Bureau of Statistics (Pakistan).	GHDx
9720	Pakistan	Pakistan Integrated Household Survey/ Household Integrated Economic Survey 2001-2002	2001 - 2002	I	I	I	-	-	I	-	-	I	-	-	Federal Bureau of Statistics (Pakistan). Pakistan Integrated Household Survey 2001-2002. Islamabad, Pakistan: Federal Bureau of Statistics (Pakistan).	GHDx
9951	Pakistan	Pakistan Social and Living Standards Measurement Survey/ Household Integrated Economic Survey 2004-2005	2004 - 2005	I	I	I	-	-	I	-	-	I	-	-	Federal Bureau of Statistics (Pakistan). Pakistan Social and Living Standards Measurement Survey 2004-2005. Islamabad, Pakistan: Federal Bureau of Statistics (Pakistan).	GHDx
20584	Pakistan	Pakistan Demographic and Health Survey 1990-1991	1990 - 1991	I	I	I	-	-	I	-	-	I	-	-	Institute for Resource Development, Macro Systems, National Institute of Population Studies (Pakistan). Pakistan Demographic and Health Survey 1990-1991. Fairfax, United States: ICF International.	GHDx

GHDx ID	Country	Series	Year	BCG	DPT1	DPT3	HepB3	Hib3	MCV1	MCV2	PCV3	Pol3	RCV1	RotaC	Citation	Link
20595	Pakistan	Pakistan Demographic and Health Survey 2006-2007	2006 - 2007	I	I	I	I	-	I	-	-	I	-	-	Macro International, Inc, National Institute of Population Studies (Pakistan). Pakistan Demographic and Health Survey 2006-2007. Fairfax, United States of America: ICF International.	GHDx
24818	Pakistan	Pakistan Social and Living Standards Measurement Survey/ Household Integrated Economic Survey 2005-2006	2005 - 2006	I	I	I	I	-	I	-	-	I	-	-	Federal Bureau of Statistics (Pakistan). Pakistan Social and Living Standards Measurement Survey 2005-2006. Islamabad, Pakistan: Federal Bureau of Statistics (Pakistan).	GHDx
30634	Pakistan	Pakistan Social and Living Standards Measurement Survey/ Household Integrated Economic Survey 2007-2008	2007 - 2008	I	I	I	I	-	I	-	-	I	-	-	Federal Bureau of Statistics (Pakistan). Pakistan Social and Living Standards Measurement Survey 2007-2008. Islamabad, Pakistan: Federal Bureau of Statistics (Pakistan).	GHDx
77521	Pakistan	Pakistan Demographic and Health Survey 2012-2013	2012 - 2013	I	I	I	I	I	I	-	-	I	-	-	ICF International, National Institute of Population Studies (Pakistan), Pakistan Bureau of Statistics. Pakistan Demographic and Health Survey 2012-2013. Fairfax, United States of America: ICF International.	GHDx
141521	Pakistan	Pakistan National Nutrition Survey 2011	2011	I	-	I	I	I	I	-	-	I	-	-	Aga Khan University, Ministry of National Health Services, Regulations & Coordination (Pakistan), Ministry of Planning and Development (Pakistan), Pakistan Medical Research Council, United Nations Children's Fund (UNICEF). Pakistan National Nutrition Survey 2011.	GHDx
165908	Pakistan	Pakistan Social and Living Standards Measurement Survey 2008-2009	2008 - 2009	I	I	I	-	-	I	-	-	I	-	-	Pakistan Bureau of Statistics. Pakistan Social and Living Standards Measurement Survey 2008-2009. Islamabad, Pakistan: Pakistan Bureau of Statistics.	GHDx
228002	Pakistan	Pakistan Social and Living Standards Measurement Survey 2010-2011	2010 - 2011	I	I	I	-	-	I	-	-	I	-	-	Pakistan Bureau of Statistics. Pakistan Social and Living Standards Measurement Survey 2010-2011. Islamabad, Pakistan: Pakistan Bureau of Statistics.	GHDx
231783	Pakistan	Pakistan EPI Coverage Evaluation Survey 2005	2005	I	I	I	I	-	I	-	-	I	-	-	Ministry of Health (Pakistan). Pakistan EPI Coverage Evaluation Survey 2005.	GHDx
238338	Pakistan	Pakistan Social and Living Standards Measurement Survey/ Household Integrated Economic Survey 2013-2014	2013 - 2014	I	I	I	I	I	I	-	-	I	-	-	Pakistan Bureau of Statistics. Pakistan Social and Living Standards Measurement Survey/ Household Integrated Economic Survey 2013-2014.	GHDx
286783	Pakistan	Pakistan Demographic and Health Survey 2017-2018	2017 - 2018	I	I	I	I	I	I	I	I	I	E	-	ICF International, Ministry of National Health Services, Regulations & Coordination (Pakistan), National Institute of Population Studies (Pakistan). Pakistan Demographic and Health Survey 2017-2018. Fairfax, United States of America: ICF International, 2018.	GHDx
400526	Pakistan	Pakistan - Punjab Multiple Indicator Cluster Survey 2017-2018	2017 - 2018	I	I	I	I	I	I	I	I	I	-	-	Bureau of Statistics Punjab (Pakistan), United Nations Children's Fund (UNICEF). Pakistan - Punjab Multiple Indicator Cluster Survey 2017-2018. New York, United States of America: United Nations Children's Fund (UNICEF), 2019.	GHDx
9999	Palestine	Palestine Family Health Survey 2006-2007	2006 - 2007	I	I	E	-	-	I	-	-	I	I	-	League of Arab States, Palestinian Central Bureau of Statistics, United Nations Children's Fund (UNICEF). Palestine Family Health Survey 2006-2007.	GHDx
125591	Palestine	Palestine Multiple Indicator Cluster Survey 2010	2010	I	I	I	I	-	I	-	-	I	-	-	Ministry of Health (Palestine), Palestinian Central Bureau of Statistics, United Nations Children's Fund (UNICEF), United Nations Population Fund (UNFPA). Palestine Multiple Indicator Cluster Survey 2010. New York, United States of America: United Nations Children's Fund (UNICEF), 2014.	GHDx
134754	Palestine	Palestine Health Status Annual Report 2001	1997 - 2001	I	E	I	-	-	I	-	-	I	-	-	Ministry of Health (Palestine). Palestine Health Status Annual Report 2001. Nablus, Palestine: Ministry of Health (Palestine), 2002.	GHDx
136572	Palestine	Palestine Health Status Annual Report 2000	2000	I	I	I	-	-	I	-	-	I	-	-	Ministry of Health (Palestine). Palestine Health Status Annual Report 2000. Nablus, Palestine: Ministry of Health (Palestine), 2001.	GHDx
142186	Palestine	Palestine - West Bank and Gaza Strip Multiple	1996	E	-	-	-	-	I	-	-	I	E	-	Palestine - West Bank and Gaza Strip Health Survey 1996	GHDx

GHDx ID	Country	Series	Year	BCG	DPT1	DPT3	HepB3	Hib3	MCV1	MCV2	PCV3	Pol3	RCV1	RotaC	Citation	Link
		Indicator Cluster Survey 1996 - UNICEF														
161590	Palestine	Palestine Multiple Indicator Cluster Survey 2014	2014	I	I	I	I	I	I	-	-	I	-	-	Ministry of Health (Palestine), Palestinian Central Bureau of Statistics, United Nations Children's Fund (UNICEF). Palestine Multiple Indicator Cluster Survey 2014. New York, United States of America: United Nations Children's Fund (UNICEF), 2015.	GHDx
10063	Panama	Panama Family Planning/Maternal and Child Health Survey 1979	1979	I	-	I	-	-	I	-	-	I	-	-	Ministry of Health and Division of Reproductive Health-Centers for Disease Control and Prevention (CDC). Panama Family Planning/Maternal and Child Health Survey 1979. Ministry of Health, Ciudad de Panama, Panama, 1981.	GHDx
10224	Panama	Panama Living Standard Measurement Survey 2003	2003	I	I	I	-	-	I	-	-	I	-	-	Census and Statistics Directorate (Panama), Ministry of Economy and Finance (Panama), World Bank. Panama Living Standard Measurement Survey 2003. Washington DC, United States of America: World Bank.	GHDx
10277	Panama	Panama Living Standards Measurement Survey 1997	1997	I	I	I	-	-	-	-	-	I	-	-	Ministry of Planning and Economic Policy (Panama), World Bank. Panama Living Standards Measurement Survey 1997. Washington DC, United States: World Bank.	GHDx
46517	Panama	Panama Living Standard Measurement Survey 2008	2008	I	I	I	E	-	-	-	-	I	-	I	Census and Statistics Directorate (Panama), Ministry of Economy and Finance (Panama), World Bank. Panama Living Standard Measurement Survey 2008. Washington DC, United States of America: World Bank.	GHDx
161587	Panama	Panama Multiple Indicator Cluster Survey 2013	2013	I	I	I	I	I	I	-	-	I	I	I	National Institute of Statistics and Census (Panama), United Nations Children's Fund (UNICEF). Panama Multiple Indicator Cluster Survey 2013. New York, United States of America: United Nations Children's Fund (UNICEF).	GHDx
20599	Papua New Guinea	Papua New Guinea Demographic and Health Survey 1996-1997	1996 - 1997	I	I	I	-	-	I	-	-	I	-	-	National Statistical Office (Papua New Guinea). Papua New Guinea Demographic and Health Survey 1996-1997.	GHDx
44870	Papua New Guinea	Papua New Guinea Demographic and Health Survey 2006-2007	2006 - 2007	I	I	I	I	-	I	-	-	I	-	-	National Statistical Office (Papua New Guinea), National Statistics Office (Philippines). Papua New Guinea Demographic and Health Survey 2006-2007.	GHDx
231774	Papua New Guinea	Papua New Guinea National Immunization Coverage Survey 2005-2006	2005 - 2006	I	I	I	I	-	I	-	-	I	-	-	National Department of Health (Papua New Guinea). Papua New Guinea National Immunization Coverage Survey 2005-2006.	GHDx
426238	Papua New Guinea	Papua New Guinea Demographic and Health Survey 2016-2018	2016 - 2018	I	I	I	I	I	I	E	I	I	I	-	ICF International, National Statistical Office (Papua New Guinea). Papua New Guinea Demographic and Health Survey 2016-2018. Fairfax, United States of America: ICF International, 2019.	GHDx
10350	Paraguay	Paraguay Integrated Household Survey 2000-2001	2000 - 2001	I	-	-	-	-	I	-	-	-	-	-	General Directorate of Statistics, Surveys and Censuses (Paraguay). Paraguay Integrated Household Survey 2000-2001. Asuncion, Paraguay: General Directorate of Statistics, Surveys and Censuses (Paraguay).	GHDx
10357	Paraguay	Paraguay Integrated Household Survey 1997-1998	1997 - 1998	I	-	-	-	-	I	-	-	-	-	-	General Directorate of Statistics, Surveys and Censuses (Paraguay). Paraguay Integrated Household Survey 1997-1998. Asuncion, Paraguay: General Directorate of Statistics, Surveys and Censuses (Paraguay).	GHDx
10364	Paraguay	Paraguay Reproductive Health Survey 1995-1996	1995 - 1996	I	I	I	-	-	I	-	-	I	-	-	Division of Reproductive Health-Centers for Disease Control and Prevention (CDC). Paraguay Reproductive Health Survey 1995-1996. Asuncion, Paraguay, Paraguayan Center for Population Studies (CEPEP).	GHDx
10370	Paraguay	Paraguay Reproductive Health Survey 2004	2004	I	I	I	I	I	I	-	-	I	-	-	Division of Reproductive Health-Centers for Disease Control and Prevention (CDC). (2005): Paraguay Reproductive Health Survey 2004. Asuncion, Paraguay, Paraguayan Center for Population Studies (CEPEP).	GHDx
20608	Paraguay	Paraguay Demographic and Health Survey 1990	1990	I	I	I	-	-	I	-	-	I	-	-	Macro Systems, Inc.; Institute for Resource Development, Paraguayan Center for Population Studies (CEPEP). Paraguay Demographic and Health Survey 1990. Fairfax, United States: ICF International.	GHDx
27525	Paraguay	Paraguay Reproductive Health Survey 2008	2008	I	I	I	I	I	I	-	-	I	I	-	Paraguay Center for Population Studies (CEPEP). Paraguay Reproductive Health Survey 2008. AsunciÃƒÂ³n, Paraguay: Paraguayan Center for Population Studies (CEPEP).	GHDx
41830	Paraguay	Paraguay Permanent Household Survey 2005	2005	I	-	-	-	-	I	-	-	-	-	-	Department of Statistics, Surveys and Censuses (Paraguay). Paraguay Permanent Household Survey 2005. AsunciÃƒÂ³n, Paraguay: Department of Statistics, Surveys and Censuses (Paraguay).	GHDx

GHDx ID	Country	Series	Year	BCG	DPT1	DPT3	HepB3	Hib3	MCV1	MCV2	PCV3	Pol3	RCV1	RotaC	Citation	Link
41837	Paraguay	Paraguay Permanent Household Survey 2006	2006	I	-	-	-	-	I	-	-	-	-	-	Department of Statistics, Surveys and Censuses (Paraguay). Paraguay Permanent Household Survey 2006. AsunciÃƒÂ³n, Paraguay: Department of Statistics, Surveys and Censuses (Paraguay).	GHDx
231759	Paraguay	Paraguay National Vaccination Coverage Survey of Children Aged 12-35 Months 2011	2011	I	I	I	I	I	I	-	-	I	-	-	Paraguay National Vaccination Coverage Survey of Children Aged 12-35 Months 2011.	GHDx
324470	Paraguay	Paraguay Multiple Indicator Cluster Survey 2016	2016	I	I	I	I	I	I	-	-	I	-	E	General Directorate of Statistics, Surveys and Censuses (Paraguay), Ministry of Public Health and Social Welfare (Paraguay), United Nations Children's Fund (UNICEF). Paraguay Multiple Indicator Cluster Survey 2016. New York, United States of America: United Nations Children's Fund (UNICEF), 2017.	GHDx
20626	Peru	Peru Demographic and Health Survey 1991-1992	1991 - 1992	I	I	I	-	-	I	-	-	I	-	-	Macro International, Inc, National Institute of Statistics (Peru), PRISMA (Peru). Peru Demographic and Health Survey 1991-1992. Fairfax, United States of America: ICF International.	GHDx
20638	Peru	Peru Demographic and Health Survey 1996	1996	I	I	I	-	-	I	-	-	I	-	-	Macro International, Inc, National Institute of Statistics (Peru). Peru Demographic and Health Survey 1996. Fairfax, United States of America: ICF International.	GHDx
20649	Peru	Peru Demographic and Health Survey 2000	2000	I	I	I	E	I	I	-	-	I	-	-	Macro International, Inc, National Institute of Statistics (Peru). Peru Demographic and Health Survey 2000. Fairfax, United States of America: ICF International.	GHDx
43845	Peru	Peru National Household Survey, Second Quarter 1998	1998	I	I	I	-	-	I	-	-	I	-	-	National Institute of Statistics and Informatics (INEI) (Peru). Peru National Household Survey, Second Quarter 1998. Lima, Peru: National Institute of Statistics and Informatics (INEI) (Peru).	GHDx
44315	Peru	Peru National Household Survey, Second Quarter 2000	2000	-	I	I	-	-	-	-	-	I	-	-	National Institute of Statistics and Informatics (INEI) (Peru). Peru National Household Survey, Second Quarter 2000. Lima, Peru: National Institute of Statistics and Informatics (INEI) (Peru).	GHDx
129521	Peru	Peru Continuous Demographic and Health Survey 2004-2008	2004 - 2008	I	I	I	-	-	I	-	-	I	-	-	Peru Continuous Demographic and Health Survey 2004-2008	GHDx
209930	Peru	Peru Continuous Demographic and Health Survey 2014	2014	I	I	I	-	-	I	-	-	I	-	-	ICF International, Ministry of Health (Peru), National Institute of Statistics and Informatics (Peru), National Police of Peru (PNP). Peru Continuous Demographic and Health Survey 2014. Fairfax, United States of America: ICF International, 2015.	GHDx
210182	Peru	Peru Continuous Demographic and Health Survey 2014 - INEI	2014	I	I	I	-	-	I	-	-	I	-	-	ICF International, Ministry of Health (Peru), National Institute of Statistics and Informatics (Peru), National Police of Peru (PNP). Peru Continuous Demographic and Health Survey 2014 - INEI. Lima, Peru: National Institute of Statistics and Informatics (Peru), 2015.	GHDx
210231	Peru	Peru Continuous Demographic and Health Survey 2013 - INEI	2013	I	I	I	-	-	I	-	-	I	-	-	ICF International, National Institute of Statistics and Informatics (Peru). Peru Continuous Demographic and Health Survey 2013 - INEI. Lima, Peru: National Institute of Statistics and Informatics (Peru), 2014.	GHDx
270404	Peru	Peru Continuous Demographic and Health Survey 2009	2009	I	I	I	I	I	I	I	E	I	E	E	National Institute of Statistics and Informatics (Peru), ORC Macro. Peru Continuous Demographic and Health Survey 2009. Fairfax, United States of America: ICF International.	GHDx
270469	Peru	Peru Continuous Demographic and Health Survey 2010	2010	I	I	I	I	I	I	E	I	I	E	I	National Institute of Statistics and Informatics (Peru). Peru Continuous Demographic and Health Survey 2010. Fairfax, United States of America: ICF International.	GHDx
270470	Peru	Peru Continuous Demographic and Health Survey 2011	2011	I	I	I	I	I	I	E	I	I	E	I	Macro International, Inc, National Institute of Statistics and Informatics (Peru). Peru Continuous Demographic and Health Survey 2011. Fairfax, United States of America: ICF International.	GHDx
270471	Peru	Peru Continuous Demographic and Health Survey 2012	2012	I	I	I	I	I	I	E	I	I	E	I	Macro International, Inc, National Institute of Statistics and Informatics (Peru). Peru Continuous Demographic and Health Survey 2012. Fairfax, United States of America: ICF International.	GHDx
275090	Peru	Peru Continuous Demographic and Health Survey 2003-2008	2003 - 2008	I	I	I	I	I	E	E	E	I	E	-	Ministry of Economy and Finance (Peru), National Institute of Statistics and Informatics (Peru), ORC Macro. Peru Continuous Demographic and Health Survey 2003-2008. Fairfax, United States of America: ICF International.	GHDx

GHDx ID	Country	Series	Year	BCG	DPT1	DPT3	HepB3	Hib3	MCV1	MCV2	PCV3	Pol3	RCV1	RotaC	Citation	Link
358824	Peru	Peru Demographic and Family Health Survey 2017	2017	I	I	I	-	-	I	-	-	I	-	-	National Center for Food and Nutrition, National Institute of Health (Peru), National Institute of Statistics and Informatics (Peru), National Police of Peru (PNP). Peru Demographic and Family Health Survey 2017. Lima, Peru: National Institute of Statistics and Informatics (Peru).	GHDx
407869	Peru	Peru Demographic and Family Health Survey 2018	2018	I	I	I	-	-	I	-	-	I	-	-	National Institute of Statistics and Informatics (Peru). Peru Demographic and Family Health Survey 2018. Lima, Peru: National Institute of Statistics and Informatics (Peru), 2019.	GHDx
10937	Philippines	Philippines Multiple Indicator Cluster Survey 1996	1996	I	-	I	-	-	I	-	-	I	-	-	National Statistics Office (Philippines), United Nations Children's Fund (UNICEF). Philippines Multiple Indicator Cluster Survey 1996.	GHDx
20674	Philippines	Philippines Demographic and Health Survey 1993	1993	I	I	I	-	-	I	-	-	I	-	-	Macro International, Inc, National Statistics Office (Philippines). Philippines Demographic and Health Survey 1993. Fairfax, United States of America: ICF International.	GHDx
20683	Philippines	Philippines Demographic and Health Survey 1998	1998	I	I	I	I	-	I	-	-	I	-	-	Department of Health (Philippines), Macro International, Inc, National Statistics Office (Philippines). Philippines Demographic and Health Survey 1998. Fairfax, United States of America: ICF International.	GHDx
20699	Philippines	Philippines Demographic and Health Survey 2003	2003	I	I	I	I	-	I	-	-	I	-	-	Macro International, Inc, National Statistics Office (Philippines). Philippines Demographic and Health Survey 2003. Fairfax, United States of America: ICF International.	GHDx
21421	Philippines	Philippines Demographic and Health Survey 2008	2008	I	I	I	I	E	I	-	-	I	-	-	Macro International, Inc, National Statistics Office (Philippines). Philippines Demographic and Health Survey 2008. Fairfax, United States of America: ICF International, 2010.	GHDx
27826	Philippines	Philippines Field Health Service Information System (FHSIS) Annual Report 2007	2007	I	I	I	I	-	I	-	-	I	-	-	National Epidemiology Center, Department of Health (Philippines). Philippines Field Health Service Information System (FHSIS) Annual Report 2007. Manila, Philippines: National Epidemiology Center, Department of Health (Philippines).	GHDx
142943	Philippines	Philippines Demographic and Health Survey 2013	2013	I	I	I	E	I	I	-	-	I	I	I	ICF International, Philippines Statistics Authority. Philippines Demographic and Health Survey 2013. Fairfax, United States of America: ICF International, 2014.	GHDx
248530	Philippines	Philippines Sub-Regional Multiple Indicator Survey 2012	2012	-	I	I	-	-	I	-	-	-	-	-	National Statistics Office (Philippines), United Nations Children's Fund (UNICEF). Philippines Sub-Regional Multiple Indicator Survey 2012.	GHDx
248532	Philippines	Philippines Multiple Indicator Survey 2014	2014	-	I	I	I	I	I	-	-	-	-	-	National Statistics Office (Philippines), United Nations Children's Fund (UNICEF), University of the Philippines, Manila. Philippines Multiple Indicator Survey 2014.	GHDx
264955	Philippines	Philippines Multiple Indicator Survey 2016	2016	-	I	I	I	I	I	E	-	I	-	I	Demographic Reseach and Development Foundation Inc. (Philippines), National Statistics Office (Philippines), United Nations Children's Fund (UNICEF). Philippines Multiple Indicator Survey 2016.	GHDx
285194	Philippines	Philippines Field Health Services Information System (FHSIS) Annual Report 2015	2015	-	I	I	I	I	I	-	I	I	I	-	National Epidemiology Center, Department of Health (Philippines). Philippines Field Health Services Information System (FHSIS) Annual Report 2015. Manila, Philippines: National Epidemiology Center, Department of Health (Philippines).	GHDx
337557	Philippines	Philippines Maternal and Child Survey 2002	2002	I	I	I	-	-	I	-	-	I	-	-	Department of Health (Philippines), Philippines Statistics Authority. Philippines Maternal and Child Survey 2002. Manila, Philippines: Philippines Statistics Authority.	GHDx
337877	Philippines	Philippines Demographic and Health Survey 2017	2017	I	I	I	I	I	I	I	I	I	E	-	ICF International, Philippines Statistics Authority, United States Agency for International Development (USAID). Philippines Demographic and Health Survey 2017. Fairfax, United States of America: ICF International, 2018.	GHDx
338568	Philippines	Philipines Maternal and Child Health Survey 2000	2000	I	I	I	I	-	I	-	-	I	-	-	National Statistics Office (Philippines), United States Census Bureau (USCB). Philipines Maternal and Child Health Survey 2000.	GHDx
11324	Rwanda	Rwanda Integrated Living Conditions Survey 2005-2006	2005 - 2006	I	I	-	-	-	-	-	-	-	-	-	National Institute of Statistics of Rwanda (NISR), Oxford Policy Management. Rwanda Integrated Living Conditions Survey 2005-2006. Kigali, Rwanda: National Institute of Statistics of Rwanda (NISR).	GHDx
20711	Rwanda	Rwanda Demographic and Health Survey 1992	1992	I	I	I	-	-	I	-	-	I	-	-	Macro International, Inc, National Office of Population (Rwanda). Rwanda Demographic and Health Survey 1992. Fairfax, United States of America: ICF International.	GHDx
20722	Rwanda	Rwanda Demographic and Health Survey 2000	2000	I	I	I	-	-	I	-	-	I	-	-	Macro International, Inc, National Office of Population (Rwanda). Rwanda Demographic and Health Survey 2000. Fairfax, United States of America: ICF International.	GHDx
20740	Rwanda	Rwanda Demographic and Health Survey 2005	2005	I	I	I	-	-	I	-	-	I	-	-	Macro International, Inc, National Institute of Statistics of Rwanda. Rwanda Demographic and Health Survey 2005. Fairfax, United States of America: ICF International.	GHDx

GHDx ID	Country	Series	Year	BCG	DPT1	DPT3	HepB3	Hib3	MCV1	MCV2	PCV3	Pol3	RCV1	RotaC	Citation	Link
21222	Rwanda	Rwanda Interim Demographic and Health Survey 2007-2008	2007 - 2008	I	I	I	-	-	I	-	-	I	-	-	Macro International, Inc, Ministry of Health (Rwanda), National Institute of Statistics of Rwanda. Rwanda Interim Demographic and Health Survey 2007-2008. Fairfax, United States of America: ICF International.	GHDx
26930	Rwanda	Rwanda Multiple Indicator Cluster Survey 2000	2000	I	I	I	-	-	I	-	-	I	-	-	Department of Statistics (Rwanda), United Nations Children's Fund (UNICEF). Rwanda Multiple Indicator Cluster Survey 2000. New York, United States: United Nations Children's Fund (UNICEF).	GHDx
56040	Rwanda	Rwanda Demographic and Health Survey 2010-2011	2010 - 2011	I	I	I	I	I	I	-	I	I	-	-	ICF Macro, Ministry of Health (Rwanda), National Institute of Statistics of Rwanda. Rwanda Demographic and Health Survey 2010-2011. Fairfax, United States of America: ICF International.	GHDx
151437	Rwanda	Rwanda Integrated Household Living Conditions Survey 2010-2011	2010 - 2011	I	I	I	-	-	I	-	-	I	-	-	National Institute of Statistics of Rwanda. Rwanda Integrated Household Living Conditions Survey 2010-2011. Kigali, Rwanda: National Institute of Statistics of Rwanda.	GHDx
157063	Rwanda	Rwanda Demographic and Health Survey 2014-2015	2014 - 2015	I	I	I	I	I	I	-	I	I	E	I	ICF International, Ministry of Health (Rwanda), National Institute of Statistics of Rwanda. Rwanda Demographic and Health Survey 2014-2015. Fairfax, United States of America: ICF International, 2016.	GHDx
231641	Rwanda	Rwanda Integrated Post Measles-Rubella Campaign and Routine Immunization Coverage Evaluation Survey 2013	2013	I	I	I	I	I	I	-	I	I	-	-	Government of Rwanda, World Health Organization (WHO). Rwanda Integrated Post Measles-Rubella Campaign and Routine Immunization Coverage Evaluation Survey 2013.	GHDx
231648	Rwanda	Rwanda National Immunization Coverage Survey 2007	2007	I	I	I	I	I	I	-	-	I	-	-	Ministry of Health (Rwanda). Rwanda National Immunization Coverage Survey 2007.	GHDx
21313	Samoa	Samoa Demographic and Health Survey 2009	2009	I	I	I	-	-	I	-	-	I	-	-	ICF Macro, Ministry of Health (Samoa), Samoa Bureau of Statistics. Samoa Demographic and Health Survey 2009.	GHDx
267785	Samoa	Samoa Demographic and Health Survey 2014	2014	I	I	I	-	-	I	I	-	I	I	-	Ministry of Finance (Samoa), Ministry of Health (Samoa), Ministry of Women, Community and Social Development (Samoa). Samoa Demographic and Health Survey 2014.	GHDx
11434	São Tomé and Príncipe	Sao Tome and Principe Multiple Indicator Cluster Survey 2006	2006	I	I	I	-	-	I	-	-	I	-	-	National Institute of Statistics (Sao Tome and Principe), United Nations Children's Fund (UNICEF). Sao Tome and Principe Multiple Indicator Cluster Survey 2006. New York, United States of America: United Nations Children's Fund (UNICEF).	GHDx
26866	São Tomé and Príncipe	Sao Tome and Principe Demographic and Health Survey 2008-2009	2008 - 2009	I	I	I	-	-	I	-	-	I	-	-	ICF Macro, Ministry of Health (Sao Tome and Principe), National Institute of Statistics (Sao Tome and Principe). Sao Tome and Principe Demographic and Health Survey 2008-2009. Fairfax, United States of America: ICF International.	GHDx
27055	São Tomé and Príncipe	Sao Tome and Principe Multiple Indicator Cluster Survey 2000	2000	I	I	I	-	-	I	-	-	I	-	-	National Institute of Statistics (Sao Tome and Principe), United Nations Children's Fund (UNICEF). Sao Tome and Principe Multiple Indicator Cluster Survey 2000. New York, United States of America: United Nations Children's Fund (UNICEF).	GHDx
214640	São Tomé and Príncipe	Sao Tome and Principe Multiple Indicator Cluster Survey 2014	2014	I	I	I	I	I	I	-	I	I	-	-	Global Fund to Fight Aids Tuberculosis and Malaria (GFATM), ICF International, National Center for Endemic Diseases (CNE) (Sao Tome and Principe), National Institute of Statistics (Sao Tome and Principe), United Nations Children's Fund (UNICEF), United Nations Development Programme (UNDP). Sao Tome and Principe Multiple Indicator Cluster Survey 2014. New York, United States of America: United Nations Children's Fund (UNICEF), 2016.	GHDx
229647	São Tomé and Príncipe	Sao Tome and Principe National Vaccine Coverage Survey 2007	2007	I	I	I	I	-	I	-	-	I	-	-	World Health Organization (WHO). Sao Tome and Principe National Vaccine Coverage Survey 2007.	GHDx
229674	São Tomé and Príncipe	Sao Tome and Principe Vaccination Coverage Survey 1990	1990	-	I	I	-	-	I	-	-	I	-	-	Sao Tome and Principe Vaccination Coverage Survey 1990.	GHDx
11468	Saudi Arabia	Saudi Arabia Family Health Survey 1996-1997	1996 - 1997	I	I	I	-	-	I	-	-	I	-	-	Arab Fund for Economic and Social Development (AFESD), Arab Gulf Program for Development (AGFUND), Gulf-Co-operation Council (GCC), Ministry of Health (Saudi Arabia), United Nations Children's Fund (UNICEF), United Nations Population Fund (UNFPA), United Nations Statistics Division (UNSD), World Health Organization (WHO). Saudi Arabia Family Health Survey 1996-1997.	GHDx

GHDx ID	Country	Series	Year	BCG	DPT1	DPT3	HepB3	Hib3	MCV1	MCV2	PCV3	Pol3	RCV1	RotaC	Citation	Link
20749	Senegal	Senegal Demographic and Health Survey 1986	1986	I	I	I	-	-	I	-	-	I	-	-	Department of Statistics (Senegal), Westinghouse; Institute for Resource Development. Senegal Demographic and Health Survey 1986. Fairfax, United States of America: ICF International.	GHDx
20767	Senegal	Senegal Demographic and Health Survey 1992-1993	1992 - 1993	I	I	I	-	-	I	-	-	I	-	-	Directorate of Forecasting and Statistics, Ministry of the Economy, Finance and Planning (Senegal), Macro International, Inc. Senegal Demographic and Health Survey 1992-1993. Fairfax, United States of America: ICF International.	GHDx
20786	Senegal	Senegal Demographic and Health Survey 1999-2000	1999 - 2000	I	I	I	-	-	I	-	-	I	-	-	Groupe SERDHA, Macro International, Inc, Ministry of Health and Prevention (Senegal). Senegal Demographic and Health Survey 1999-2000. Fairfax, United States of America: ICF International.	GHDx
26855	Senegal	Senegal Demographic and Health Survey 2005	2005	I	I	I	-	-	I	-	-	I	-	-	Ministry of Health and Prevention (Senegal), Research Center for Human Development (Senegal). Senegal Demographic and Health Survey 2005. Fairfax, United States of America: ICF International.	GHDx
56063	Senegal	Senegal Demographic and Health Survey 2010-2011	2010 - 2011	I	I	I	I	I	I	-	-	I	-	-	Center for Research in Human Development (CRDH), Cheikh Anta Diop University, Hospital Aristide Le Dantec, ICF Macro, National Agency of Statistics and Demography (Senegal). Senegal Demographic and Health Survey 2010-2011. Fairfax, United States of America: ICF International.	GHDx
77392	Senegal	Senegal Continuous Service Provision Assessment 2012-2013	2012 - 2013	I	-	I	I	I	I	-	-	I	-	-	ICF International, Ministry of Health and Social Action (Senegal), National Agency of Statistics and Demography (Senegal). Senegal Continuous Service Provision Assessment 2012-2013. Fairfax, United States of America: ICF International, 2015.	GHDx
111432	Senegal	Senegal Continuous Demographic and Health Survey 2012-2013	2012 - 2013	I	I	I	I	I	I	-	-	I	-	-	ICF International, Ministry of Health and Social Action (Senegal), National Agency of Statistics and Demography (Senegal). Senegal Continuous Demographic and Health Survey 2012-2013. Fairfax, United States of America: ICF International.	GHDx
191270	Senegal	Senegal Continuous Demographic and Health Survey 2014	2014	I	I	I	I	I	I	-	-	I	-	-	Cheikh Anta Diop University, ICF International, National Agency of Statistics and Demography (Senegal). Senegal Continuous Demographic and Health Survey 2014. Fairfax, United States of America: ICF International.	GHDx
218592	Senegal	Senegal Continuous Demographic and Health Survey 2015	2015	I	I	I	I	I	I	-	-	I	-	-	Cheikh Anta Diop University, ICF International, National Agency of Statistics and Demography (Senegal). Senegal Continuous Demographic and Health Survey 2015. Fairfax, United States of America: ICF International, 2016.	GHDx
231585	Senegal	Senegal National EPI Coverage Survey 2013	2013	I	I	I	I	I	I	-	-	I	-	-	Maternal and Child Health Integrated Program (MCHIP). Senegal National EPI Coverage Survey 2013.	GHDx
231622	Senegal	Senegal EPI External Review 2010	2010	I	I	I	I	I	I	-	-	-	-	-	Ministry of Health and Prevention (Senegal). Senegal EPI External Review 2010.	GHDx
231637	Senegal	Senegal External EPI Review 2000	2000	I	I	I	-	-	I	-	-	I	-	-	Ministry of Health and Prevention (Senegal). Senegal External EPI Review 2000.	GHDx
286772	Senegal	Senegal Continuous Demographic and Health Survey 2016	2016	I	I	I	-	-	I	I	-	I	-	-	ICF International, Ministry of Health and Social Action (Senegal), National Agency of Statistics and Demography (Senegal). Senegal Continuous Demographic and Health Survey 2016. Fairfax, United States of America: ICF International, 2017.	GHDx
287639	Senegal	Senegal - Dakar Urban Multiple Indicator Cluster Survey 2015-2016	2015 - 2016	I	I	I	I	I	I	I	I	I	-	I	National Agency of Statistics and Demography (Senegal), United Nations Children's Fund (UNICEF). Senegal - Dakar Urban Multiple Indicator Cluster Survey 2015-2016. New York, United States of America: United Nations Children's Fund (UNICEF), 2018.	GHDx
353526	Senegal	Senegal Continuous Demographic and Health Survey 2017	2017	I	I	I	I	I	I	I	I	I	-	I	ICF International, Ministry of Health and Social Action (Senegal), National Agency of Statistics and Demography (Senegal), Unit for the Fight Against Malnutrition (Senegal). Senegal Continuous Demographic and Health Survey 2017. Fairfax, United States of America: ICF International, 2018.	GHDx
450419	Senegal	Senegal Continuous Demographic and Health Survey 2018	2018	I	I	I	I	I	I	I	I	I	-	I	Directorate of Forecasting and Statistics, Ministry of the Economy, Finance and Planning (Senegal), ICF International, Ministry of Health and Social Action (Senegal), United States Agency for International Development (USAID). Senegal Continuous Demographic and Health Survey 2018. Fairfax, United States of America: ICF International, 2020.	GHDx
8819	Serbia	Serbia and Montenegro - Montenegro Multiple Indicator Cluster Survey 2005-2006	2005 - 2006	I	I	I	-	-	I	-	-	I	-	-	United Nations Children's Fund (UNICEF), Statistical Office of Montenegro. Montenegro Multiple Indicator Cluster Survey 2005. New York, United States: United Nations Children's Fund (UNICEF).	GHDx

GHDx ID	Country	Series	Year	BCG	DPT1	DPT3	HepB3	Hib3	MCV1	MCV2	PCV3	Pol3	RCV1	RotaC	Citation	Link
11551	Serbia	Serbia and Montenegro - Serbia Multiple Indicator Cluster Survey 2005-2006	2005 - 2006	I	I	I	-	-	I	-	-	I	I	-	United Nations Children's Fund (UNICEF), Statistical Office of the Republic of Serbia (SORS), Strategic Marketing Research Agency (SMMRI). Serbia Multiple Indicator Cluster Survey 2005-2006. New York, United States: United Nations Children's Fund (UNICEF).	GHDx
27022	Serbia	Yugoslavia, Federal Republic Multiple Indicator Cluster Survey 1996	1996	I	-	I	-	-	-	-	-	-	-	-	Institute for Public Health (Montenegro), Institute for Public Health (Pristina), Institute of Public Health of Serbia, Ministry of Health (FR Yugoslavia), Mother Theresa Charity, United Nations Children's Fund (UNICEF). Yugoslavia, Federal Republic Multiple Indicator Cluster Survey 1996.	GHDx
27024	Serbia	Yugoslavia, Federal Republic Multiple Indicator Cluster Survey 2000	2000	I	I	I	-	-	I	-	-	I	I	-	Institute for Public Health (Montenegro), Institute of Public Health of Serbia, United Nations Children's Fund (UNICEF), World Health Organization (WHO). Yugoslavia, Federal Republic Multiple Indicator Cluster Survey 2000.	GHDx
150870	Serbia	Serbia Multiple Indicator Cluster Survey 2014	2014	I	I	I	I	I	I	-	-	I	I	-	Statistical Office of the Republic of Serbia. Serbia Multiple Indicator Cluster Survey 2014. New York, United States: United Nations Children's Fund (UNICEF), 2015.	GHDx
11639	Sierra Leone	Sierra Leone Multiple Indicator Cluster Survey 2000	2000	I	I	I	-	-	I	-	-	I	-	-	Central Statistics Office (Sierra Leone), United Nations Children's Fund (UNICEF). Sierra Leone Multiple Indicator Cluster Survey 2000. New York, United States of America: United Nations Children's Fund (UNICEF).	GHDx
11649	Sierra Leone	Sierra Leone Multiple Indicator Cluster Survey 2005	2005	I	I	I	-	-	I	-	-	I	E	-	United Nations Children's Fund (UNICEF), Statistics Sierra Leone. Sierra Leone Multiple Indicator Cluster Survey 2005. New York, United States: United Nations Children's Fund (UNICEF).	GHDx
21258	Sierra Leone	Sierra Leone Demographic and Health Survey 2008	2008	I	I	I	I	I	I	-	-	I	-	-	Macro International, Inc, Statistics Sierra Leone. Sierra Leone Demographic and Health Survey 2008. Fairfax, United States of America: ICF International.	GHDx
76700	Sierra Leone	Sierra Leone Multiple Indicator Cluster Survey 2010	2010	I	I	I	I	I	E	-	-	I	-	-	Statistics Sierra Leone, United Nations Children's Fund (UNICEF). Sierra Leone Multiple Indicator Cluster Survey 2010. New York, United States of America: United Nations Children's Fund (UNICEF).	GHDx
131467	Sierra Leone	Sierra Leone Demographic and Health Survey 2013	2013	I	I	I	I	I	I	-	I	I	-	-	ICF International, Ministry of Health and Sanitation (Sierra Leone), Statistics Sierra Leone. Sierra Leone Demographic and Health Survey 2013. Fairfax, United States of America: ICF International, 2014.	GHDx
173776	Sierra Leone	Sierra Leone Multiple Indicator Cluster Survey 1995	1995	I	-	I	-	-	I	-	-	I	-	-	Central Statistics Office (Sierra Leone), Government of Sierra Leone, United Nations Children's Fund (UNICEF). Sierra Leone Multiple Indicator Cluster Survey 1995.	GHDx
218619	Sierra Leone	Sierra Leone Multiple Indicator Cluster Survey 2017	2017	I	I	I	I	I	I	-	I	I	-	I	Statistics Sierra Leone, United Nations Children's Fund (UNICEF). Sierra Leone Multiple Indicator Cluster Survey 2017. New York, United States of America: United Nations Children's Fund (UNICEF), 2018.	GHDx
229705	Sierra Leone	Sierra Leone Routine Immunization Coverage Survey 2013	2013	I	I	I	I	I	I	-	I	I	-	-	Government of Sierra Leone. Sierra Leone Routine Immunization Coverage Survey 2013.	GHDx
229711	Sierra Leone	Sierra Leone Immunization Cluster Coverage Survey 2010	2010	I	I	I	I	I	I	-	-	I	-	-	Sierra Leone Immunization Cluster Coverage Survey 2010.	GHDx
231381	Sierra Leone	Sierra Leone EPI National Coverage Evaluation Survey 2001	2001	I	I	I	-	-	I	-	-	I	-	-	Sierra Leone EPI National Coverage Evaluation Survey 2001.	GHDx
274896	Sierra Leone	Sierra Leone Nutrition Survey 2010	2010	-	-	-	-	-	I	-	-	-	-	-	Ministry of Health (Sierra Leone), Statistics Sierra Leone. Sierra Leone Nutrition Survey 2010.	GHDx
375813	Sierra Leone	Sierra Leone National Nutrition Survey 2017	2017	-	-	-	-	-	I	-	-	-	-	-	Action Against Hunger (ACF), Ministry of Health and Sanitation (Sierra Leone), Statistics Sierra Leone. Sierra Leone National Nutrition Survey 2017.	GHDx
157111	Singapore	Singapore State of Health 2001	1968 - 2001	I	-	I	I	-	I	-	-	I	-	-	Ministry of Health (Singapore). Singapore State of Health 2001. Singapore, Singapore: Ministry of Health (Singapore).	GHDx
21446	Solomon Islands	Solomon Islands Demographic and Health Survey 2006-2007	2006 - 2007	I	I	I	-	-	I	-	-	I	-	-	Macro International, Inc, Ministry of Health (Solomon Islands), Secretariat of the Pacific Community (SPC), Solomon Islands National Statistics Office (SINSO). Solomon Islands Demographic and Health Survey 2006-2007.	GHDx

GHDx ID	Country	Series	Year	BCG	DPT1	DPT3	HepB3	Hib3	MCV1	MCV2	PCV3	Pol3	RCV1	RotaC	Citation	Link
322237	Solomon Islands	Solomon Islands Demographic and Health Survey 2015	2015	I	I	I	I	I	I	-	-	I	-	-	Department of Foreign Affairs and Trade (Australia), Ministry of Health and Medical Services (Solomon Islands), Secretariat of the Pacific Community (SPC), Solomon Islands National Statistics Office (SINSO), Solomon Islands Resource Facility (SIRF), United Nations Children's Fund (UNICEF). Solomon Islands Demographic and Health Survey 2015.	GHDx
11759	Somalia	Somalia Multiple Indicator Cluster Survey 1999	1999	I	I	I	-	-	I	-	-	I	-	-	United Nations Children's Fund (UNICEF). Somalia Multiple Indicator Cluster Survey 1999.	GHDx
11774	Somalia	Somalia Multiple Indicator Cluster Survey 2006	2006	I	I	I	-	-	I	-	-	I	-	-	Pan Arab Project for Family Health (PAPFAM), United Nations Children's Fund (UNICEF). Somalia Multiple Indicator Cluster Survey 2006. New York, United States of America: United Nations Children's Fund (UNICEF).	GHDx
151893	Somalia	Somalia Family Health Survey 1983	1983	I	E	I	-	-	I	-	-	I	-	-	Ministry of Health (Somalia), Ministry of National Planning (Somalia), Westinghouse Health Systems, Inc. Somalia Family Health Survey 1983.	GHDx
454381	Somalia	Somalia Health and Demographic Survey 2018-2019	2018 - 2019	I	I	I	I	I	I	-	-	I	-	-	Directorate of National Statistics (Somalia), Office for National Statistics (ONS) (United Kingdom), Statistics Norway, Statistics Sweden, United Nations Population Fund (UNFPA). Somalia Health and Demographic Survey 2018-2019. New York City, United States of America: United Nations Population Fund (UNFPA), 2020.	GHDx
20796	South Africa	South Africa Demographic and Health Survey 1998	1998	I	I	I	I	-	I	-	-	I	-	-	Department of Health (South Africa), Macro International, Inc, South African Medical Research Council. South Africa Demographic and Health Survey 1998. Fairfax, United States of America: ICF International.	GHDx
20798	South Africa	South Africa Demographic and Health Survey 2003-2004	2003 - 2004	I	I	I	-	-	I	-	-	I	-	-	Department of Health (South Africa), Macro International, Inc, South African Medical Research Council. South Africa Demographic and Health Survey 2003-2004.	GHDx
139810	South Africa	South Africa Anthropometric, Vitamin A, Iron and Immunisation Coverage Status in Children Aged 6 to 71 Months 1994	1994	I	I	I	-	-	I	-	-	I	-	-	South African Vitamin A Consultative Group (SAVACG). South Africa Anthropometric, Vitamin A, Iron and Immunisation Coverage Status in Children Aged 6 to 71 Months 1994.	GHDx
157064	South Africa	South Africa Demographic and Health Survey 2016	2016	I	I	I	I	I	I	I	I	I	-	I	Department of Health (South Africa), ICF International, South African Medical Research Council, Statistics South Africa. South Africa Demographic and Health Survey 2016. Fairfax, United States of America: ICF International, 2019.	GHDx
232705	South Korea	South Korea National Vaccine Coverage Survey 2008-2009	2008 - 2009	-	-	-	-	-	-	-	-	I	-	-	South Korea National Vaccine Coverage Survey 2008-2009.	GHDx
229684	South Sudan	South Sudan EPI Coverage Survey 2011-2012	2011 - 2012	I	I	I	-	-	I	-	-	I	-	-	Ministry of Health (South Sudan). South Sudan EPI Coverage Survey 2011-2012.	GHDx
18815	Sri Lanka	Sri Lanka Demographic and Health Survey 2006-2007	2006 - 2007	I	I	I	I	-	I	-	-	I	-	-	Department of Census and Statistics (Sri Lanka), Macro International, Inc. Sri Lanka Demographic and Health Survey 2006-2007. 2009.	GHDx
20806	Sri Lanka	Sri Lanka Demographic and Health Survey 1987	1987	I	I	I	-	-	I	-	-	I	-	-	Department of Census and Statistics (Sri Lanka), Westinghouse; Institute for Resource Development. Sri Lanka Demographic and Health Survey 1987. Fairfax, United States of America: ICF International.	GHDx
90705	Sri Lanka	Sri Lanka Demographic and Health Survey 1993	1993	I	E	I	-	-	I	-	-	I	-	-	Department of Census and Statistics (Sri Lanka). Sri Lanka Demographic and Health Survey 1993. 2009.	GHDx
326837	Sri Lanka	Sri Lanka Demographic and Health Survey 2016	2016	I	I	I	I	I	I	I	-	I	I	-	Department of Census and Statistics (Sri Lanka), ICF International. Sri Lanka Demographic and Health Survey 2016.	GHDx
12243	Sudan	Sudan Multiple Indicator Cluster Survey 2000	2000	I	I	I	-	-	I	-	-	I	-	-	Central Bureau of Statistics (Sudan), Federal Ministry of Health (Sudan), United Nations Children's Fund (UNICEF). Sudan Multiple Indicator Cluster Survey 2000. New York, United States of America: United Nations Children's Fund (UNICEF).	GHDx
32189	Sudan	Sudan - South Multiple Indicator Cluster Survey 2010	2010	I	I	I	-	-	I	-	-	I	-	-	Central Bureau of Statistics (Sudan), Federal Ministry of Health (Sudan), Government of Sudan, Ministry of Health (South Sudan), Southern Sudan Centre for Census, Statistics and Evaluation. Sudan - South Multiple Indicator Cluster Survey 2010. New York, United States of America: United Nations Children's Fund (UNICEF), 2015.	GHDx

GHDx ID	Country	Series	Year	BCG	DPT1	DPT3	HepB3	Hib3	MCV1	MCV2	PCV3	Pol3	RCV1	RotaC	Citation	Link
141914	Sudan	Sudan Multiple Indicator Cluster Survey 1995	1995	-	I	I	-	-	I	-	-	I	-	-	Central Bureau of Statistics (Sudan), Federal Ministry of Health (Sudan), United Nations Children's Fund (UNICEF), University of Khartoum. Sudan Multiple Indicator Cluster Survey 1995.	GHDx
153643	Sudan	Sudan - North Multiple Indicator Cluster Survey 2010	2010	I	I	I	I	I	I	-	-	I	-	-	Central Bureau of Statistics (Sudan), Ministry of Health (South Sudan). Sudan - North Multiple Indicator Cluster Survey 2010. New York, United States of America: United Nations Children's Fund (UNICEF), 2015.	GHDx
200617	Sudan	Sudan Multiple Indicator Cluster Survey 2014	2014	I	I	I	I	I	I	E	-	I	-	-	Central Bureau of Statistics (Sudan), Federal Ministry of Health (Sudan), United Nations Children's Fund (UNICEF). Sudan Multiple Indicator Cluster Survey 2014. New York, United States of America: United Nations Children's Fund (UNICEF), 2016.	GHDx
12280	Suriname	Suriname Multiple Indicator Cluster Survey 1999-2000	1999 - 2000	-	I	I	-	-	I	-	-	I	-	-	General Bureau of Statistics (Suriname), Pan American Health Organization (PAHO), United Nations Children's Fund (UNICEF), United Nations Development Programme (UNDP). Suriname Multiple Indicator Cluster Survey 1999-2000. New York, United States of America: United Nations Children's Fund (UNICEF).	GHDx
12289	Suriname	Suriname Multiple Indicator Cluster Survey 2006	2006	-	I	I	-	-	I	-	-	I	-	-	General Statistical Office (Suriname), United Nations Children's Fund (UNICEF). Suriname Multiple Indicator Cluster Survey 2006. New York, United States of America: United Nations Children's Fund (UNICEF).	GHDx
81203	Suriname	Suriname Multiple Indicator Cluster Survey 2010	2010	-	-	-	-	-	I	-	-	I	I	-	General Bureau of Statistics (Suriname), Ministry of Planning and Development Cooperation (Suriname), Ministry of Social Affairs and Housing (Suriname), United Nations Children's Fund (UNICEF). Suriname Multiple Indicator Cluster Survey 2010. New York, United States of America: United Nations Children's Fund (UNICEF), 2013.	GHDx
427983	Suriname	Suriname Multiple Indicator Cluster Survey 2018	2018	-	I	I	I	I	I	I	-	I	I	-	General Bureau of Statistics (Suriname), Ministry of Planning and Development Cooperation (Suriname), Ministry of Social Affairs and Housing (Suriname), United Nations Children's Fund (UNICEF). Suriname Multiple Indicator Cluster Survey 2018. New York, United States of America: United Nations Children's Fund (UNICEF), 2019.	GHDx
10023	Syria	Palestinians in Syria Multiple Indicator Cluster Survey 2006	2006	I	I	I	I	-	I	-	-	I	-	-	General Administration for Palestine Arab Refugees (GAPAR), Palestinian Central Bureau of Statistics, Pan Arab Project for Family Health (PAPFAM), United Nations Children's Fund (UNICEF). Palestinians in Syria Multiple Indicator Cluster Survey 2006.	GHDx
12377	Syria	Syria EPI/CDD and Child Mortality Survey 1990	1990	I	I	I	-	-	I	-	-	I	-	-	United Nations Children's Fund (UNICEF), World Health Organization (WHO). Syria EPI/CDD and Child Mortality Survey 1990.	GHDx
12379	Syria	Syria Family Health Survey 2001	2001	-	I	I	I	-	I	-	-	I	I	-	Central Bureau of Statistics (Syria), League of Arab States. Syria Family Health Survey 2001.	GHDx
12388	Syria	Syria Maternal and Child Health Survey 1993	1993	I	-	I	-	-	I	-	-	I	-	-	Central Bureau of Statistics (Syria), League of Arab States. Syria Maternal and Child Health Survey 1993.	GHDx
12389	Syria	Syria Multiple Indicator Cluster Survey 1995	1995	I	I	I	-	E	I	I	-	I	-	-	United Nations Children's Fund (UNICEF). Syria Multiple Indicator Cluster Survey 1995.	GHDx
12399	Syria	Syria Multiple Indicator Cluster Survey 2006	2006	I	I	I	-	-	I	-	-	I	-	-	United Nations Children's Fund (UNICEF), Central Bureau of Statistics (Syria), Ministry of Health (Syria), Pan Arab Project for Family Health (PAPFAM). Syria Multiple Indicator Cluster Survey 2006. New York, United States: United Nations Children's Fund (UNICEF).	GHDx
126911	Syria	Syria Family Health Survey 2009	2009	I	I	I	-	-	I	-	-	I	-	-	Central Bureau of Statistics (Syria), League of Arab States. Syria Family Health Survey 2009.	GHDx
229594	Syria	Syria EPI/CDD Review 1988	1988	I	I	I	-	-	I	-	-	I	-	-	Syria EPI/CDD Review 1988.	GHDx
229595	Syria	Syria Immunization Campaign 1986	1986	I	I	I	-	-	I	-	-	I	-	-	Syria Immunization Campaign 1986.	GHDx
300048	Taiwan (province of China)	Taiwan Statistics of Communicable Diseases and Surveillance Report 2015	2015	I	-	I	-	I	-	-	-	I	-	-	Centers for Disease Control, R.O.C (Taiwan). Taiwan Statistics of Communicable Diseases and Surveillance Report 2015. Taipei, Taiwan (Province of China): Centers for Disease Control, R.O.C (Taiwan), 2016.	GHDx
12595	Tajikistan	Tajikistan Multiple Indicator Cluster Survey 2000	2000	I	I	I	-	-	I	-	-	I	-	-	National State Statistical Agency (Tajikistan), United Nations Children's Fund (UNICEF). Tajikistan Multiple Indicator Cluster Survey 2000. New York, United States: United Nations Children's Fund (UNICEF).	GHDx

GHDx ID	Country	Series	Year	BCG	DPT1	DPT3	HepB3	Hib3	MCV1	MCV2	PCV3	Pol3	RCV1	RotaC	Citation	Link
12608	Tajikistan	Tajikistan Multiple Indicator Cluster Survey 2005	2005	I	I	I	-	-	I	-	-	I	-	-	United Nations Children's Fund (UNICEF), State Committee on Statistics of the Republic of Tajikistan. Tajikistan Multiple Indicator Cluster Survey 2005. New York, United States: United Nations Children's Fund (UNICEF).	GHDx
74460	Tajikistan	Tajikistan Demographic and Health Survey 2012	2012	I	I	I	I	I	I	-	-	I	-	-	ICF International, Ministry of Health (Tajikistan), Statistical Agency under the President of the Republic of Tajikistan. Tajikistan Demographic and Health Survey 2012. Fairfax, United States of America: ICF International, 2013.	GHDx
156672	Tajikistan	Tajikistan Health Care Systems in Transition 2000	1990 - 1998	-	-	-	-	-	I	-	-	-	-	-	European Observatory on Health Systems and Policies, World Health Organization Regional Office for Europe (WHO/Europe). Tajikistan Health Care Systems in Transition 2000. Copenhagen, Denmark: World Health Organization Regional Office for Europe (WHO/Europe), 2000.	GHDx
341838	Tajikistan	Tajikistan Demographic and Health Survey 2017	2017	I	I	I	I	I	I	-	-	I	-	I	ICF International, Statistical Agency under the President of the Republic of Tajikistan. Tajikistan Demographic and Health Survey 2017. Fairfax, United States of America: ICF International, 2018.	GHDx
20841	Tanzania	Tanzania Demographic and Health Survey 1991-1992	1991 - 1992	I	I	I	-	-	I	-	-	I	-	-	Bureau of Statistics (Tanzania), Macro International, Inc, Ministry of Health (Tanzania). Tanzania Demographic and Health Survey 1991-1992. Fairfax, United States of America: ICF International.	GHDx
20852	Tanzania	Tanzania Demographic and Health Survey 1996	1996	I	I	I	-	-	I	-	-	I	-	-	Bureau of Statistics (Tanzania), Macro International, Inc, Planning Commission (Tanzania). Tanzania Demographic and Health Survey 1996. Fairfax, United States of America: ICF International.	GHDx
20865	Tanzania	Tanzania Demographic and Health Survey 1999	1999	I	I	I	-	-	I	-	-	I	-	-	Macro International, Inc, National Bureau of Statistics (Tanzania). Tanzania Demographic and Health Survey 1999. Fairfax, United States of America: ICF International.	GHDx
20875	Tanzania	Tanzania Demographic and Health Survey 2004-2005	2004 - 2005	I	I	I	E	E	I	-	-	I	-	-	Macro International, Inc, National Bureau of Statistics (Tanzania). Tanzania Demographic and Health Survey 2004-2005. Fairfax, United States of America: ICF International.	GHDx
21331	Tanzania	Tanzania Demographic and Health Survey 2009-2010	2009 - 2010	I	I	I	E	E	I	-	-	I	-	-	ICF Macro, National Bureau of Statistics (Tanzania). Tanzania Demographic and Health Survey 2009-2010. Fairfax, United States of America: ICF International.	GHDx
27297	Tanzania	Tanzania National Panel Survey 2008-2009	2008 - 2009	-	I	I	-	-	-	-	-	I	-	-	National Bureau of Statistics (Tanzania). Tanzania Living Standards Measurement Study - Integrated Survey on Agriculture 2008-2009. Dar es Salaam, Tanzania: National Bureau of Statistics (Tanzania).	GHDx
50513	Tanzania	Tanzania National Panel Survey 2008-2009	1999 - 2000	I	I	I	-	-	I	-	-	I	-	-	MEASURE Evaluation Project, Carolina Population Center, University of North Carolina, Ministry of Health (Tanzania), National Bureau of Statistics (Tanzania). Tanzania Reproductive and Child Health Survey 1999-2000. Chapel Hill, United States of America: MEASURE Evaluation Project, Carolina Population Center, University of North Carolina.	GHDx
194016	Tanzania	Tanzania Multiple Indicator Cluster Survey 1996	1996	I	-	I	-	-	I	-	-	I	-	-	Government of Tanzania, Revolutionary Government of Zanzibar, United Nations Children's Fund (UNICEF). Tanzania Multiple Indicator Cluster Survey 1996.	GHDx
218593	Tanzania	Tanzania Demographic and Health Survey 2015-2016	2015 - 2016	I	I	I	I	I	I	E	I	I	-	I	ICF International, Ministry of Health (Zanzibar), Ministry of Health, Community Development, Gender, Elderly and Children (MoHCDEC) (Tanzania), National Bureau of Statistics (Tanzania), Office of the Chief Government Statistician (OCGS) (Zanzibar). Tanzania Demographic and Health Survey 2015-2016. Fairfax, United States of America: ICF International, 2016.	GHDx
228513	Tanzania	Tanzania Integrated Measles and Routine Immunization: Post Campaign Coverage Evaluation Survey 2011	2011	I	I	I	I	I	I	-	-	I	-	-	Tanzania Integrated Measles and Routine Immunization: Post Campaign Coverage Evaluation Survey 2011.	GHDx
448257	Tanzania	Tanzania Post Integrated Measles Rubella Campaign Evaluation and Routine Immunization Coverage Survey 2014	2014	I	I	I	I	I	I	-	I	I	-	-	Ministry of Health and Social Welfare (Tanzania), Muhimbili University of Health and Allied Sciences (Tanzania), National Bureau of Statistics (Tanzania), World Health Organization (WHO). Tanzania Post Integrated Measles Rubella Campaign Evaluation and Routine Immunization Coverage Survey 2014.	GHDx

GHDx ID	Country	Series	Year	BCG	DPT1	DPT3	HepB3	Hib3	MCV1	MCV2	PCV3	Pol3	RCV1	RotaC	Citation	Link
453458	Tanzania	Tanzania Post Measles-Rubella Campaign Evaluation 2019	2019	I	I	I	I	I	I	-	I	I	-	I	Ministry of Health (Tanzania). Tanzania Post Measles-Rubella Campaign Evaluation 2019.	GHDx
12732	Thailand	Thailand Multiple Indicator Cluster Survey 2005-2006	2005 - 2006	I	I	I	I	-	I	-	-	I	-	-	National Statistical Office (Thailand), United Nations Children's Fund (UNICEF). Thailand Multiple Indicator Cluster Survey 2005-2006. New York, United States of America: United Nations Children's Fund (UNICEF).	GHDx
20887	Thailand	Thailand Demographic and Health Survey 1987	1987	I	I	I	-	-	I	-	-	I	-	-	Chulalongkorn University, Institute of Population Studies (Thailand), Westinghouse; Institute for Resource Development. Thailand Demographic and Health Survey 1987. Fairfax, United States: ICF International.	GHDx
148649	Thailand	Thailand Multiple Indicator Cluster Survey 2012	2012	I	I	I	I	-	I	-	-	I	-	-	College of Population Studies, Chulalongkorn University (Thailand), Institute for Population and Social Research, Mahidol University (Thailand), International Health Policy Program (Thailand), Ministry of Education (Thailand), Ministry of Public Health (Thailand), Ministry of Social Development and Human Security (MSDHS) (Thailand), National Health Security Office (Thailand), National Statistical Office (Thailand), Thai Health Promotion Foundation, United Nations Children's Fund (UNICEF). Thailand Multiple Indicator Cluster Survey 2012. New York, United States of America: United Nations Children's Fund (UNICEF), 2016.	GHDx
228965	Thailand	Thailand Immunization Coverage Survey 1999	1999	I	I	I	I	-	I	-	-	I	-	-	Thailand Immunization Coverage Survey 1999.	GHDx
229348	Thailand	Thailand UNICEF Reporting Form for Immunization and Case Reporting 1996	1996	I	-	I	I	-	I	-	-	I	-	-	Thailand UNICEF Reporting Form for Immunization and Case Reporting 1996.	GHDx
229377	Thailand	Thailand EPI Coverage Survey 1990	1990	I	-	I	-	-	I	-	-	I	-	-	ASEAN Institute for Health Development (AIHD) (Thailand). Thailand EPI Coverage Survey 1990.	GHDx
296646	Thailand	Thailand Multiple Indicator Cluster Survey 2015-2016	2015 - 2016	I	I	I	I	-	I	-	-	I	-	-	National Health Security Office (Thailand), National Statistical Office (Thailand), United Nations Children's Fund (UNICEF). Thailand Multiple Indicator Cluster Survey 2015-2016. New York, United States of America: United Nations Children's Fund (UNICEF), 2018.	GHDx
3922	The Gambia	Gambia Multiple Indicator Cluster Survey 2000	2000	I	I	I	-	-	I	-	-	I	-	-	Central Statistics Department (Gambia), United Nations Children's Fund (UNICEF). Gambia Multiple Indicator Cluster Survey 2000. New York, United States: United Nations Children's Fund (UNICEF).	GHDx
3935	The Gambia	Gambia Multiple Indicator Cluster Survey 2005-2006	2005 - 2006	I	I	I	-	-	I	-	-	I	-	-	Gambia Bureau of Statistics (GBOS), United Nations Children's Fund (UNICEF). Gambia Multiple Indicator Cluster Survey 2005-2006. New York, United States of America: United Nations Children's Fund (UNICEF).	GHDx
77384	The Gambia	Gambia Demographic and Health Survey 2013	2013	I	I	I	I	I	I	E	I	I	-	-	Gambia Bureau of Statistics (GBOS), ICF International, Ministry of Health and Social Welfare (The Gambia). Gambia Demographic and Health Survey 2013. Fairfax, United States of America: ICF International, 2015.	GHDx
91506	The Gambia	Gambia Multiple Indicator Cluster Survey 2010	2010	I	I	I	E	-	I	-	-	I	-	-	Gambia Bureau of Statistics (GBOS), United Nations Children's Fund (UNICEF). Gambia Multiple Indicator Cluster Survey 2010. New York, United States of America: United Nations Children's Fund (UNICEF), 2018.	GHDx
142212	The Gambia	Gambia Multiple Indicator Cluster Survey 1996	1996	I	-	I	-	-	I	-	-	I	-	-	Central Statistics Department (Gambia), United Nations Children's Fund (UNICEF). Gambia Multiple Indicator Cluster Survey 1996.	GHDx
233678	The Gambia	Gambia EPI Cluster Survey 2012	2012	I	I	I	I	I	I	-	I	I	-	-	United Nations Children's Fund (UNICEF). Gambia EPI Cluster Survey 2012.	GHDx
233680	The Gambia	Gambia Immunization Survey 2006	2006	I	-	I	I	I	I	-	-	I	-	-	Gambia Immunization Survey 2006.	GHDx
233682	The Gambia	Gambia Immunization Survey 2005	2005	I	-	I	I	I	I	-	-	I	-	-	Gambia Immunization Survey 2005.	GHDx
233696	The Gambia	Gambia Immunization Coverage Survey 2002	2002	I	-	I	I	I	I	-	-	I	-	-	Gambia Immunization Coverage Survey 2002.	GHDx
233704	The Gambia	Gambia EPI Coverage Evaluation Survey 1992	1992	I	I	I	-	-	I	-	-	I	-	-	Gambia EPI Coverage Evaluation Survey 1992.	GHDx

GHDx ID	Country	Series	Year	BCG	DPT1	DPT3	HepB3	Hib3	MCV1	MCV2	PCV3	Pol3	RCV1	RotaC	Citation	Link
424884	The Gambia	Gambia Multiple Indicator Cluster Survey 2018	2018	I	I	I	I	I	I	I	I	I	I	I	Gambia Bureau of Statistics (GBOS), United Nations Children's Fund (UNICEF). Gambia Multiple Indicator Cluster Survey 2018. New York, United States of America: United Nations Children's Fund (UNICEF), 2019.	GHDx
12865	Timor-Leste	Timor-Leste Multiple Indicator Cluster Survey 2002	2002	I	-	I	-	-	I	-	-	I	-	-	Insan Hitawasana Sejahtera, National Statistics Directorate (Timor-Leste), United Nations Children's Fund (UNICEF). Timor-Leste Multiple Indicator Cluster Survey 2002. Dili, Timor-Leste: National Statistics Directorate (Timor-Leste).	GHDx
20888	Timor-Leste	Timor-Leste Demographic and Health Survey 2003	2003	I	I	I	E	-	I	-	-	I	-	-	ACIL Australia Pty Ltd., Australian National University, Ministry of Health (Timor-Leste), National Statistics Directorate (Timor-Leste), University of Newcastle (Australia). Timor-Leste Demographic and Health Survey 2003. Newcastle, Australia: University of Newcastle (Australia).	GHDx
21274	Timor-Leste	Timor-Leste Demographic and Health Survey 2009-2010	2009 - 2010	I	I	I	I	-	I	-	-	I	-	-	ICF Macro, Ministry of Finance (Timor-Leste), National Statistics Directorate (Timor-Leste). Timor-Leste Demographic and Health Survey 2009-2010. Fairfax, United States of America: ICF International.	GHDx
286211	Timor-Leste	Timor-Leste Food and Nutrition Survey 2013	2013	I	I	I	-	-	I	-	-	I	-	-	Ministry of Health (Timor-Leste). Timor-Leste Food and Nutrition Survey 2013.	GHDx
286785	Timor-Leste	Timor-Leste Demographic and Health Survey 2016	2016	I	I	I	I	I	I	-	-	I	-	-	ICF International, National Statistics Directorate (Timor-Leste). Timor-Leste Demographic and Health Survey 2016. Fairfax, United States of America: ICF International, 2018.	GHDx
12886	Togo	Togo Multiple Indicator Cluster Survey 2000	2000	I	I	I	-	-	I	-	-	I	-	-	United Nations Children's Fund (UNICEF). Togo Multiple Indicator Cluster Survey 2000. New York, United States of America: United Nations Children's Fund (UNICEF).	GHDx
12896	Togo	Togo Multiple Indicator Cluster Survey 2006	2006	I	I	I	-	-	I	-	-	I	-	-	Directorate General of Statistics and National Accounting (Togo), United Nations Children's Fund (UNICEF). Togo Multiple Indicator Cluster Survey 2006. New York, United States: United Nations Children's Fund (UNICEF).	GHDx
20909	Togo	Togo Demographic and Health Survey 1998	1998	I	I	I	-	-	I	-	-	I	-	-	Department of Statistics (Togo), Macro International, Inc. Togo Demographic and Health Survey 1998. Fairfax, United States of America: ICF International.	GHDx
40021	Togo	Togo Multiple Indicator Cluster Survey 2010	2010	I	I	I	I	I	I	-	-	I	-	-	Directorate General of Statistics and National Accounting (Togo), United Nations Children's Fund (UNICEF). Togo Multiple Indicator Cluster Survey 2010. New York, United States: United Nations Children's Fund (UNICEF).	GHDx
77515	Togo	Togo Demographic and Health Survey 2013-2014	2013 - 2014	I	I	I	-	-	I	-	-	I	-	-	Directorate General of Statistics and National Accounts (Togo), ICF International, Ministry of Health (Togo), Ministry of Planning, Development and Zoning (Togo). Togo Demographic and Health Survey 2013-2014. Fairfax, United States of America: ICF International, 2015.	GHDx
189153	Togo	Togo Multiple Indicator Cluster Survey 1996	1996	I	-	I	-	-	I	-	-	I	-	-	Demographic Research Unit, University of Benin (Togo), Department of Statistics (Togo), Ministry of Interior and Security (Togo), Ministry of Planning and Territorial Development (Togo), United Nations Children's Fund (UNICEF). Togo Multiple Indicator Cluster Survey 1996. New York, United States: United Nations Children's Fund (UNICEF).	GHDx
229452	Togo	Togo EPI Review 2012-2013	2012 - 2013	I	I	I	I	I	I	-	-	I	-	-	GAVI Alliance. Togo EPI Review 2012-2013.	GHDx
229459	Togo	Togo External EPI Review 2001	2001	I	I	I	-	-	I	-	-	I	-	-	Global Alliance for Vaccines and Immunization (GAVI). Togo External EPI Review 2001.	GHDx
229463	Togo	Togo National EPI Cost Assessment of Fully Vaccinating Children through the Anti-Malaria Program 1990	1990	I	I	I	-	-	I	-	-	I	-	-	Togo National EPI Cost Assessment of Fully Vaccinating Children through the Anti-Malaria Program 1990.	GHDx
229484	Togo	Togo National EPI Assessment 1988	1988	I	I	I	-	-	I	-	-	I	-	-	Togo National EPI Assessment 1988.	GHDx
429991	Togo	Togo Multiple Indicator Cluster Survey 2017	2017	I	I	I	I	I	I	-	I	I	E	I	Directorate General of Statistics and National Accounting (Togo), United Nations Children's Fund (UNICEF). Togo Multiple Indicator Cluster Survey 2010. New York, United States: United Nations Children's Fund (UNICEF).	GHDx
228787	Tonga	Tonga Evaluation of Immunization Program 2003	2003	I	-	I	I	-	I	-	-	I	-	-	Tonga Evaluation of Immunization Program 2003.	GHDx

GHDx ID	Country	Series	Year	BCG	DPT1	DPT3	HepB3	Hib3	MCV1	MCV2	PCV3	Pol3	RCV1	RotaC	Citation	Link
12950	Trinidad and Tobago	Trinidad and Tobago Multiple Indicator Cluster Survey 2006	2006	-	I	I	I	E	I	-	-	I	I	-	Central Statistical Office (Trinidad and Tobago) and United Nations Children's Fund (UNICEF). Trinidad and Tobago Multiple Indicator Cluster Survey 2006. New York, United States: United Nations Children's Fund (UNICEF).	GHDx
332558	Trinidad and Tobago	Trinidad and Tobago Multiple Indicator Cluster Survey 2011	2011	-	I	I	I	I	I	-	E	E	-	-	Central Statistical Office (Trinidad and Tobago), Ministry of Social Development and Family Services (Trinidad and Tobago), United Nations Children's Fund (UNICEF). Trinidad and Tobago Multiple Indicator Cluster Survey 2011. New York, United States of America: United Nations Children's Fund (UNICEF), 2018.	GHDx
12970	Tunisia	Tunisia Maternal and Child Health Survey 1994-1995	1994 - 1995	I	I	I	-	-	I	-	-	I	-	-	Ministry of Public Health (Tunisia), National Office for Family and Population (Tunisia), League of Arab States. Tunisia Maternal and Child Health Survey 1994-1995.	GHDx
12983	Tunisia	Tunisia Multiple Indicator Cluster Survey 2000	2000	I	-	I	I	-	E	-	-	I	-	-	Ministry of Public Health (Tunisia), United Nations Children's Fund (UNICEF). Tunisia Multiple Indicator Cluster Survey 2000.	GHDx
12985	Tunisia	Tunisia Multiple Indicator Cluster Survey 2006	2006	I	-	I	I	I	I	-	-	-	-	-	Ministry of Public Health (Tunisia), National Office for Family and Population, Ministry of Public Health (Tunisia), United Nations Children's Fund (UNICEF). Tunisia Multiple Indicator Cluster Survey 2006.	GHDx
76709	Tunisia	Tunisia Multiple Indicator Cluster Survey 2011-2012	2011 - 2012	I	I	I	I	-	I	-	-	I	-	-	Ministry of Regional Development and Planning (Tunisia), National Institute of Statistics (Tunisia), United Nations Children's Fund (UNICEF). Tunisia Multiple Indicator Cluster Survey 2011-2012. New York, United States of America: United Nations Children's Fund (UNICEF), 2014.	GHDx
228740	Tunisia	Tunisia National Immunization Coverage Survey 1990	1990	-	-	-	-	-	I	-	-	-	-	-	Tunisia National Immunization Coverage Survey 1990.	GHDx
20947	Turkey	Turkey Demographic and Health Survey 1998	1998	I	I	I	E	-	I	-	-	I	-	-	Institute of Population Studies, Hacettepe University, Macro International, Inc. Turkey Demographic and Health Survey 1998. Fairfax, United States: ICF International.	GHDx
20954	Turkey	Turkey Demographic and Health Survey 2003-2004	2003 - 2004	I	I	I	I	-	I	-	-	I	-	-	Institute of Population Studies, Hacettepe University, Ministry of Health (Turkey). Turkey Demographic and Health Survey 2003-2004. Fairfax, United States of America: ICF International.	GHDx
32421	Turkey	Turkey Demographic and Health Survey 2008	2008	I	I	I	I	-	I	-	-	I	-	-	Institute of Population Studies, Hacettepe University, Ministry of Health (Turkey), State Planning Organization (Turkey), Turkish Statistical Institute. Turkey Demographic and Health Survey 2008. Fairfax, United States: ICF International, 2019.	GHDx
142116	Turkey	Turkey Multiple Indicator Cluster Survey 1995	1995	I	I	I	-	-	I	-	-	I	-	-	State Institute of Statistics (Turkey), United Nations Children's Fund (UNICEF). Turkey Multiple Indicator Cluster Survey 1995. New York, United States: United Nations Children's Fund (UNICEF).	GHDx
223669	Turkey	Turkey Demographic and Health Survey 2013-2014	2013 - 2014	I	I	I	E	E	I	-	E	I	I	-	Institute of Population Studies, Hacettepe University, Ministry of Development (Turkey), Ministry of Health (Turkey). Turkey Demographic and Health Survey 2013-2014. Fairfax, United States of America: ICF International, 2019.	GHDx
228654	Turkey	Turkey Immunisation Coverage Survey 1989	1989	I	I	I	-	-	I	-	-	I	-	-	Turkey Immunisation Coverage Survey 1989.	GHDx
228736	Turkey	Turkey National EPI Program Review 1988	1988	I	I	I	-	-	I	-	-	I	-	-	Turkey National EPI Program Review 1988.	GHDx
13064	Turkmenistan	Turkmenistan Multiple Indicator Cluster Survey 2006	2006	I	I	I	I	-	I	-	-	I	-	-	Ministry of Foreign Affairs (Turkmenistan), Ministry of Health and Medical Industry (Turkmenistan), National Institute of State Statistics and Information (Turkmenistan), United Nations Children's Fund (UNICEF). Turkmenistan Multiple Indicator Cluster Survey 2006. New York, United States of America: United Nations Children's Fund (UNICEF), 2016.	GHDx
20956	Turkmenistan	Turkmenistan Demographic and Health Survey 2000	2000	I	I	I	-	-	I	-	-	I	-	-	Gurbansoltan Eje Clinical Research Center for Maternal and Child Health (GECRCMCH), Macro International, Inc, Ministry of Health and Medical Industry (Turkmenistan). Turkmenistan Demographic and Health Survey 2000.	GHDx
90713	Turkmenistan	Turkmenistan Multiple Indicator Cluster Survey 1995	1995	I	-	I	-	-	I	-	-	I	-	-	Ministry of Education (Turkmenistan), Ministry of Health and Medical Industry (Turkmenistan), State Committee on Statistics of Turkmenistan, United Nations Children's Fund (UNICEF). Turkmenistan Multiple Indicator Cluster Survey 1995.	GHDx
264583	Turkmenistan	Turkmenistan Multiple Indicator Cluster Survey 2015-2016	2015 - 2016	I	I	I	I	I	I	-	-	I	I	-	State Committee on Statistics of Turkmenistan, United Nations Children's Fund (UNICEF). Turkmenistan Multiple Indicator Cluster Survey 2015-2016. New York, United States of America: United Nations Children's Fund (UNICEF), 2017.	GHDx

GHDx ID	Country	Series	Year	BCG	DPT1	DPT3	HepB3	Hib3	MCV1	MCV2	PCV3	Pol3	RCV1	RotaC	Citation	Link
13084	Uganda	Uganda AIDS Indicator Survey 2004-2005	2004 - 2005	-	-	-	I	-	-	-	-	-	-	-	Division of Reproductive Health, Centers for Disease Control and Prevention (CDC), Ministry of Health (Uganda). Uganda AIDS Indicator Survey 2004-2005.	GHDx
20964	Uganda	Uganda Demographic and Health Survey 1988-1989	1988 - 1989	I	I	I	-	-	I	-	-	I	-	-	Macro Systems, Inc.; Institute for Resource Development, Makerere University, Ministry of Health (Uganda). Uganda Demographic and Health Survey 1988-1989. Fairfax, United States of America: ICF International.	GHDx
20976	Uganda	Uganda Demographic and Health Survey 1995	1995	I	I	I	-	-	I	-	-	I	-	-	Macro International, Inc, Statistics Department (Uganda). Uganda Demographic and Health Survey 1995. Fairfax, United States of America: ICF International.	GHDx
20993	Uganda	Uganda Demographic and Health Survey 2000-2001	2000 - 2001	I	I	I	-	-	I	-	-	I	-	-	Macro International, Inc, Uganda Bureau of Statistics. Uganda Demographic and Health Survey 2000-2001. Fairfax, United States of America: ICF International.	GHDx
21014	Uganda	Uganda Demographic and Health Survey 2006	2006	I	I	I	I	I	I	-	-	I	-	-	Macro International, Inc, Uganda Bureau of Statistics. Uganda Demographic and Health Survey 2006. Fairfax, United States of America: ICF International.	GHDx
23289	Uganda	Uganda Child Verbal Autopsy Study 2007	2007	I	I	I	I	I	I	-	-	I	-	-	MEASURE Evaluation Project, Carolina Population Center, University of North Carolina, Macro International, Inc, Ministry of Health (Uganda), Uganda Bureau of Statistics. Uganda Child Verbal Autopsy Study 2007. Calverton, United States of America: Macro International, Inc.	GHDx
50471	Uganda	Uganda Delivery of Improved Services for Health, Facility and Household Evaluation Surveys 1999	1999	I	-	I	-	-	I	-	-	I	-	-	MEASURE Evaluation Project, Carolina Population Center, University of North Carolina, Pathfinder International, Macro International, Inc., Ministry of Health (Uganda), Center for Communication Programs, Bloomberg School of Public Health, Johns Hopkins, Program for International Training in Health, University of North Carolina (INTRAH), Management Sciences for Health (MSH). Uganda Delivery of Improved Services for Health, Facility and Household Evaluation Surveys 1999. Chapel Hill, United States: MEASURE Evaluation Project, Carolina Population Center, University of North Carolina.	GHDx
56021	Uganda	Uganda Demographic and Health Survey 2011	2011	I	I	I	I	I	I	-	-	I	-	-	ICF Macro, Uganda Bureau of Statistics. Uganda Demographic and Health Survey 2011. Fairfax, United States of America: ICF International.	GHDx
81004	Uganda	Uganda Living Standards Measurement Survey - Integrated Survey on Agriculture 2009-2010	2009 - 2010	-	I	-	-	-	I	-	-	-	-	-	Uganda Bureau of Statistics. Uganda Living Standards Measurement Survey - Integrated Survey on Agriculture 2009-2010. Washington DC, United States of America: World Bank.	GHDx
93320	Uganda	Uganda National Service Delivery Survey 2008	2008	I	I	I	I	I	I	-	-	I	-	-	Ministry of Public Service (Uganda), Uganda Bureau of Statistics. Uganda National Service Delivery Survey 2008.	GHDx
142934	Uganda	Uganda Living Standards Measurement Survey - Integrated Survey on Agriculture 2010-2011	2010 - 2011	-	I	-	-	-	I	-	-	-	-	-	Uganda Bureau of Statistics. Uganda Living Standards Measurement Survey - Integrated Survey on Agriculture 2010-2011. Washington DC, United States of America: World Bank.	GHDx
142935	Uganda	Uganda Living Standards Measurement Survey - Integrated Survey on Agriculture 2011-2012	2011 - 2012	-	I	-	-	-	I	-	-	-	-	-	Uganda Bureau of Statistics. Uganda Living Standards Measurement Survey - Integrated Survey on Agriculture 2011-2012. Washington DC, United States of America: World Bank.	GHDx
228366	Uganda	Uganda Routine Immunization Coverage Survey 2012	2012	I	I	I	-	-	I	-	-	-	-	-	Uganda Routine Immunization Coverage Survey 2012.	GHDx
228440	Uganda	Uganda EPI Plus Coverage Survey 2005	2005	I	I	I	I	I	I	-	-	I	-	-	Ministry of Health (Uganda). Uganda EPI Plus Coverage Survey 2005.	GHDx
228482	Uganda	Uganda Immunization Coverage Validation Survey 1998-1999	1998 - 1999	I	I	I	-	-	I	-	-	I	-	-	World Health Organization (WHO). Uganda Immunization Coverage Validation Survey 1998-1999.	GHDx
264959	Uganda	Uganda Living Standards Measurement Survey - Integrated Survey on Agriculture 2013-2014	2013 - 2014	-	I	-	-	-	I	-	-	-	-	-	Government of the Netherlands, Uganda Bureau of Statistics, World Bank. Uganda Living Standards Measurement Survey - Integrated Survey on Agriculture 2013-2014. Washington DC, United States of America: World Bank.	GHDx

GHDx ID	Country	Series	Year	BCG	DPT1	DPT3	HepB3	Hib3	MCV1	MCV2	PCV3	Pol3	RCV1	RotaC	Citation	Link
281653	Uganda	Uganda Lot Quality Assurance Sampling Survey 2004	2004	-	I	I	-	-	-	-	-	-	-	-	John Snow, Inc., Uganda Program for Human and Holistic Development (UPHOLD). Uganda Lot Quality Assurance Sampling Survey 2004.	GHDx
286780	Uganda	Uganda Demographic and Health Survey 2016	2016	I	I	I	I	I	I	-	I	I	-	-	ICF International, Uganda Bureau of Statistics. Uganda Demographic and Health Survey 2016. Fairfax, United States of America: ICF International, 2018.	GHDx
342461	Uganda	Uganda National Service Delivery Survey 2015	2015 - 2016	I	I	I	-	-	I	-	-	I	-	-	Ministry of Public Service (Uganda), Uganda Bureau of Statistics. Uganda National Service Delivery Survey 2015.	GHDx
317790	UK	United Kingdom - England NHS Immunization Statistics 2015-2016	1988 - 2016	-	-	-	-	-	I	I	-	-	I	-	Health and Social Care Information Centre (HSCIC), NHS, Public Health England. United Kingdom - England NHS Immunization Statistics 2015-2016. Leeds, United Kingdom: Health and Social Care Information Centre (HSCIC), NHS, 2016.	GHDx
132739	Ukraine	Ukraine Multiple Indicator Cluster Survey 2012	2012	I	I	I	-	-	I	-	-	I	I	-	StatInform Consulting, State Statistics Service (Ukraine), Ukrainian Center for Social Reforms (UCSR), United Nations Children's Fund (UNICEF). Ukraine Multiple Indicator Cluster Survey 2012. New York, United States of America: United Nations Children's Fund (UNICEF), 2014.	GHDx
13224	United Arab Emirates	United Arab Emirates Child Health Survey 1987	1987	I	I	I	-	-	I	-	-	I	-	-	Ministry of Health (United Arab Emirates). United Arab Emirates Child Health Survey 1987.	GHDx
13226	United Arab Emirates	United Arab Emirates Family Health Survey 1995	1995	I	I	I	-	-	I	-	-	I	-	-	Health Ministers Council for GCC States, Ministry of Health (United Arab Emirates). United Arab Emirates Family Health Survey 1995.	GHDx
215001	United Arab Emirates	United Arab Emirates Review of the EPI 1986	1986	I	I	I	-	-	I	-	-	I	-	-	United Arab Emirates Review of the EPI 1986.	GHDx
215017	United Arab Emirates	United Arab Emirates Survey of Immunization Coverage and the EPI Information System 1990-1991	1990 - 1991	-	-	-	-	-	I	-	-	-	-	-	United Arab Emirates Survey of Immunization Coverage and the EPI Information System 1990-1991.	GHDx
13389	USA	United States National Immunization Survey-Child 2008-2009	2008 - 2009	-	I	I	I	I	I	-	I	I	I	I	National Center for Health Statistics, Centers for Disease Control and Prevention, National Center for Immunization and Respiratory Diseases, CDC (NCIRD), National Opinion Research Center (NORC) (United States). United States National Immunization Survey-Child 2008-2009. Hyattsville, United States of America: National Center for Health Statistics, Centers for Disease Control and Prevention.	GHDx
84694	USA	United States National Immunization Survey-Child 2007-2008	2007 - 2008	-	I	I	I	I	I	-	I	I	I	I	National Center for Health Statistics, Centers for Disease Control and Prevention, National Center for Immunization and Respiratory Diseases, CDC (NCIRD), National Opinion Research Center (NORC) (United States). United States National Immunization Survey-Child 2007-2008. Hyattsville, United States of America: National Center for Health Statistics, Centers for Disease Control and Prevention.	GHDx
84707	USA	United States National Immunization Survey-Child 2006-2007	2006 - 2007	-	I	I	I	I	I	-	I	I	I	E	National Center for Health Statistics, Centers for Disease Control and Prevention, National Center for Immunization and Respiratory Diseases, CDC (NCIRD), National Opinion Research Center (NORC) (United States). United States National Immunization Survey-Child 2006-2007. Hyattsville, United States of America: National Center for Health Statistics, Centers for Disease Control and Prevention.	GHDx
84716	USA	United States National Immunization Survey-Child 2005-2006	2005 - 2006	-	I	I	I	I	I	-	I	I	I	-	National Center for Health Statistics, Centers for Disease Control and Prevention, National Center for Immunization and Respiratory Diseases, CDC (NCIRD), National Opinion Research Center (NORC) (United States). United States National Immunization Survey-Child 2005-2006. Hyattsville, United States of America: National Center for Health Statistics, Centers for Disease Control and Prevention.	GHDx
84725	USA	United States National Immunization Survey-Child 2004-2005	2004 - 2005	-	I	I	I	I	I	-	I	I	I	-	Abt Associates Inc., National Center for Health Statistics, Centers for Disease Control and Prevention, National Center for Immunization and Respiratory Diseases, CDC (NCIRD). United States National Immunization Survey-Child 2004-2005. Hyattsville, United States of America: National Center for Health Statistics, Centers for Disease Control and Prevention.	GHDx
84734	USA	United States National Immunization Survey-Child 2003-2004	2003 - 2004	-	I	I	I	I	I	-	I	I	I	-	Abt Associates Inc., National Center for Health Statistics, Centers for Disease Control and Prevention, National Center for Immunization and Respiratory Diseases, CDC (NCIRD). United	GHDx

GHDx ID	Country	Series	Year	BCG	DPT1	DPT3	HepB3	Hib3	MCV1	MCV2	PCV3	Pol3	RCV1	RotaC	Citation	Link
															States National Immunization Survey-Child 2003-2004. Hyattsville, United States of America: National Center for Health Statistics, Centers for Disease Control and Prevention.	
84743	USA	United States National Immunization Survey-Child 2002-2003	2002 - 2003	-	I	I	I	I	I	-	I	I	I	-	National Center for Health Statistics, Centers for Disease Control and Prevention, National Center for Immunization and Respiratory Diseases, CDC (NCIRD), National Opinion Research Center (NORC) (United States). United States National Immunization Survey-Child 2002-2003. Hyattsville, United States of America: National Center for Health Statistics, Centers for Disease Control and Prevention.	GHDx
84752	USA	United States National Immunization Survey-Child 2001-2002	2001 - 2002	-	I	I	I	I	I	-	I	I	I	-	Abt Associates Inc., National Center for Health Statistics, Centers for Disease Control and Prevention, National Center for Immunization and Respiratory Diseases, CDC (NCIRD). United States National Immunization Survey-Child 2001-2002. Hyattsville, United States of America: National Center for Health Statistics, Centers for Disease Control and Prevention.	GHDx
84761	USA	United States National Immunization Survey-Child 2000-2001	2000 - 2001	-	I	I	I	I	I	-	-	I	I	-	Abt Associates Inc., National Center for Health Statistics, Centers for Disease Control and Prevention, National Center for Immunization and Respiratory Diseases, CDC (NCIRD). United States National Immunization Survey-Child 2000-2001. Hyattsville, United States of America: National Center for Health Statistics, Centers for Disease Control and Prevention.	GHDx
84771	USA	United States National Immunization Survey-Child 1999-2000	1999 - 2000	-	I	I	I	I	I	-	-	I	I	-	Abt Associates Inc., National Center for Health Statistics, Centers for Disease Control and Prevention, National Center for Immunization and Respiratory Diseases, CDC (NCIRD). United States National Immunization Survey-Child 1999-2000. Hyattsville, United States of America: National Center for Health Statistics, Centers for Disease Control and Prevention.	GHDx
84780	USA	United States National Immunization Survey-Child 1998-1999	1998 - 1999	-	I	I	I	I	I	-	-	I	I	-	Abt Associates Inc., National Center for Health Statistics, Centers for Disease Control and Prevention, National Center for Immunization and Respiratory Diseases, CDC (NCIRD). United States National Immunization Survey-Child 1998-1999. Hyattsville, United States of America: National Center for Health Statistics, Centers for Disease Control and Prevention.	GHDx
84789	USA	United States National Immunization Survey-Child 1996-1998	1996 - 1998	-	I	I	I	I	I	-	-	I	I	-	Abt Associates Inc., National Center for Health Statistics, Centers for Disease Control and Prevention, National Center for Immunization and Respiratory Diseases, CDC (NCIRD). United States National Immunization Survey-Child 1996-1998. Hyattsville, United States of America: National Center for Health Statistics, Centers for Disease Control and Prevention.	GHDx
84799	USA	United States National Immunization Survey-Child 1996-1997	1996 - 1997	-	I	I	I	I	I	-	-	I	I	-	Abt Associates Inc., National Center for Health Statistics, Centers for Disease Control and Prevention, National Center for Immunization and Respiratory Diseases, CDC (NCIRD). United States National Immunization Survey-Child 1996-1997. Hyattsville, United States of America: National Center for Health Statistics, Centers for Disease Control and Prevention.	GHDx
84808	USA	United States National Immunization Survey-Child 1995-1996	1995 - 1996	-	I	I	I	I	I	-	-	I	I	-	Abt Associates Inc., National Center for Health Statistics, Centers for Disease Control and Prevention, National Center for Immunization and Respiratory Diseases, CDC (NCIRD). United States National Immunization Survey-Child 1995-1996. Hyattsville, United States of America: National Center for Health Statistics, Centers for Disease Control and Prevention.	GHDx
135819	USA	United States National Immunization Survey-Child 2010-2011	2010 - 2011	-	I	I	I	I	I	-	I	I	I	I	NORC at the University of Chicago, National Center for Health Statistics, Centers for Disease Control and Prevention, National Center for Immunization and Respiratory Diseases, CDC (NCIRD). United States National Immunization Survey-Child 2010-2011. Hyattsville, United States of America: National Center for Health Statistics, Centers for Disease Control and Prevention.	GHDx
135820	USA	United States National Immunization Survey-Child 2011-2012	2011 - 2012	-	I	I	I	I	I	-	I	I	I	I	NORC at the University of Chicago, National Center for Health Statistics, Centers for Disease Control and Prevention, National Center for Immunization and Respiratory Diseases, CDC (NCIRD). United States National Immunization Survey-Child 2011-2012. Hyattsville, United States of America: National Center for Health Statistics, Centers for Disease Control and Prevention.	GHDx
137220	USA	United States National Immunization Survey-Child 2012-2013	2012 - 2013	-	I	I	I	I	I	-	I	I	I	I	NORC at the University of Chicago, National Center for Health Statistics, Centers for Disease Control and Prevention, National Center for Immunization and Respiratory Diseases, CDC (NCIRD). United States National Immunization Survey-Child 2012-2013. Hyattsville, United States of America: National Center for Health Statistics, Centers for Disease Control and Prevention.	GHDx
224280	USA	United States National Immunization Survey-Child 2013-2014	2013 - 2014	-	I	I	I	I	I	-	I	I	I	I	NORC at the University of Chicago, National Center for Health Statistics, Centers for Disease Control and Prevention, National Center for Immunization and Respiratory Diseases, CDC (NCIRD). United States National Immunization Survey-Child 2013-2014. Atlanta, United States of America: Centers for Disease Control and Prevention (CDC).	GHDx

GHDx ID	Country	Series	Year	BCG	DPT1	DPT3	HepB3	Hib3	MCV1	MCV2	PCV3	Pol3	RCV1	RotaC	Citation	Link
224282	USA	United States National Immunization Survey-Child 2014-2015	2014 - 2015	-	I	I	I	I	I	-	I	I	I	I	NORC at the University of Chicago, National Center for Health Statistics, Centers for Disease Control and Prevention, National Center for Immunization and Respiratory Diseases, CDC (NCIRD). United States National Immunization Survey-Child 2014-2015. Atlanta, United States of America: Centers for Disease Control and Prevention (CDC), 2015.	GHDx
317937	USA	United States - Texas HIV Surveillance Annual Report 2014	2005 - 2014	I	-	I	I	I	I	I	I	I	I	I	Texas Department of State Health Services. United States - Texas HIV Surveillance Annual Report 2014. Austin, TX, United States of America: Texas Department of State Health Services, 2015.	GHDx
354855	USA	United States National Immunization Survey-Child 2015-2016	2015 - 2016	-	I	I	I	I	I	-	I	I	I	I	NORC at the University of Chicago, National Center for Health Statistics, Centers for Disease Control and Prevention, National Center for Immunization and Respiratory Diseases, CDC (NCIRD). United States National Immunization Survey-Child 2015-2016. Atlanta, United States of America: Centers for Disease Control and Prevention (CDC), 2016.	GHDx
355985	USA	United States National Immunization Survey-Child 2016-2017	2016 - 2017	-	I	I	I	I	I	-	I	I	I	I	NORC at the University of Chicago, National Center for Health Statistics, Centers for Disease Control and Prevention, National Center for Immunization and Respiratory Diseases, CDC (NCIRD). United States National Immunization Survey-Child 2016-2017. Atlanta, United States of America: Centers for Disease Control and Prevention (CDC), 2017.	GHDx
356126	USA	United States National Immunization Survey-Child 2017-2018	2017 - 2018	-	I	I	I	I	I	-	I	I	I	I	NORC at the University of Chicago, National Center for Health Statistics, Centers for Disease Control and Prevention, National Center for Immunization and Respiratory Diseases, CDC (NCIRD). United States National Immunization Survey-Child 2017-2018. Atlanta, United States of America: Centers for Disease Control and Prevention (CDC), 2018.	GHDx
429869	USA	United States National Immunization Survey-Child 2018-2019	2018 - 2019	-	I	I	I	I	I	-	I	I	I	I	NORC at the University of Chicago, National Center for Immunization and Respiratory Diseases, CDC (NCIRD). United States National Immunization Survey-Child 2018-2019. Atlanta, United States of America: Centers for Disease Control and Prevention (CDC), 2018.	GHDx
13436	Uzbekistan	Uzbekistan Multiple Indicator Cluster Survey 2000	2000	I	I	I	-	-	I	-	-	I	-	-	United Nations Children's Fund (UNICEF), Ministry of Macroeconomics and Statistics (Uzbekistan). Uzbekistan Multiple Indicator Cluster Survey 2000. New York, United States: United Nations Children's Fund (UNICEF).	GHDx
13445	Uzbekistan	Uzbekistan Multiple Indicator Cluster Survey 2006	2006	I	I	I	-	-	I	-	-	I	-	-	United Nations Children's Fund (UNICEF), State Committee of the Republic of Uzbekistan on Statistics. Uzbekistan Multiple Indicator Cluster Survey 2006. New York, United States: United Nations Children's Fund (UNICEF).	GHDx
21033	Uzbekistan	Uzbekistan Demographic and Health Survey 1996	1996	I	I	I	-	-	I	-	-	I	-	-	Institute of Obstetrics and Gynecology, Ministry of Health (Uzbekistan), Macro International, Inc, Ministry of Health (Uzbekistan). Uzbekistan Demographic and Health Survey 1996. Fairfax, United States: ICF International.	GHDx
13465	Vanuatu	Vanuatu Multiple Indicator Cluster Survey 2007-2008	2007 - 2008	I	I	I	I	-	I	-	-	I	E	-	Ministry of Health (Vanuatu), United Nations Children's Fund (UNICEF). Vanuatu Multiple Indicator Cluster Survey 2007-2008. New York, United States of America: United Nations Children's Fund (UNICEF).	GHDx
229477	Vanuatu	Vanuatu Demographic and Health Survey 2013	2013	I	I	I	I	I	I	-	-	I	-	-	Ministry of Health (Vanuatu), Secretariat of the Pacific Community (SPC), Vanuatu National Statistics Office. Vanuatu Demographic and Health Survey 2013.	GHDx
453459	Vanuatu	Vanuatu Vaccination Coverage Survey 2016	2016	I	I	I	I	I	I	-	-	I	-	-	Ministry of Health (Vanuatu). Vanuatu Vaccination Coverage Survey 2016.	GHDx
13516	Venezuela	Venezuela Multiple Indicator Cluster Survey 2000	2000	I	I	I	-	-	I	-	-	I	-	-	National Institute of Statistics (Venezuela), United Nations Children's Fund (UNICEF). Venezuela Multiple Indicator Cluster Survey 2000. New York, United States: United Nations Children's Fund (UNICEF).	GHDx
13708	Vietnam	Vietnam Multiple Indicator Cluster Survey 2000	2000	I	I	I	-	-	I	-	-	I	-	-	General Statistics Office (Vietnam), United Nations Children's Fund (UNICEF). Vietnam Multiple Indicator Cluster Survey 2000. New York, United States of America: United Nations Children's Fund (UNICEF).	GHDx
13719	Vietnam	Vietnam Multiple Indicator Cluster Survey 2006	2006	I	I	I	-	-	I	-	-	I	-	-	General Statistics Office (Vietnam), United Nations Children's Fund (UNICEF). Vietnam Multiple Indicator Cluster Survey 2006. New York, United States of America: United Nations Children's Fund (UNICEF).	GHDx
21049	Vietnam	Vietnam Demographic and Health Survey 1997	1997	I	I	I	-	-	I	-	-	I	-	-	Macro International, Inc, National Committee for Population and Family Planning (Vietnam). Vietnam Demographic and Health Survey 1997. Calverton, United States of America: Macro International, Inc.	GHDx
21058	Vietnam	Vietnam Demographic and Health Survey 2002	2002	I	I	I	-	-	I	-	-	I	-	-	General Statistics Office (Vietnam), Macro International, Inc. Vietnam Demographic and Health Survey 2002. Fairfax, United States of America: ICF International.	GHDx

GHDx ID	Country	Series	Year	BCG	DPT1	DPT3	HepB3	Hib3	MCV1	MCV2	PCV3	Pol3	RCV1	RotaC	Citation	Link
57999	Vietnam	Vietnam Multiple Indicator Cluster Survey 2010-2011	2010 - 2011	I	I	I	I	-	I	-	-	I	-	-	General Statistics Office (Vietnam), United Nations Children's Fund (UNICEF). Vietnam Multiple Indicator Cluster Survey 2010-2011. New York, United States of America: United Nations Children's Fund (UNICEF).	GHDx
152735	Vietnam	Vietnam Multiple Indicator Cluster Survey 2013-2014	2013 - 2014	I	I	I	I	-	I	-	-	I	-	-	General Statistics Office (Vietnam), United Nations Children's Fund (UNICEF). Vietnam Multiple Indicator Cluster Survey 2013-2014. New York, United States of America: United Nations Children's Fund (UNICEF), 2015.	GHDx
189110	Vietnam	Vietnam Multiple Indicator Cluster Survey 1996	1996	I	I	I	-	-	E	-	-	I	-	-	General Statistics Office (Vietnam), United Nations Children's Fund (UNICEF). Vietnam Multiple Indicator Cluster Survey 1996.	GHDx
228334	Vietnam	Vietnam EPI Review 1989	1989	I	I	I	-	-	I	-	-	I	-	-	Vietnam EPI Review 1989.	GHDx
13795	Yemen	Yemen Family Health Survey 2003	2003	-	I	I	I	-	I	-	-	E	-	-	Central Statistical Organization (Yemen), League of Arab States, Ministry of Public Health and Population (Yemen), Pan Arab Project for Family Health (PAPFAM). Yemen Family Health Survey 2003.	GHDx
13816	Yemen	Yemen Multiple Indicator Cluster Survey 2006	2006	I	I	I	I	I	I	-	-	I	-	-	Ministry of Health (Yemen) and United Nations Children's Fund (UNICEF). Yemen Multiple Indicator Cluster Survey 2006. New York, United States: United Nations Children's Fund (UNICEF).	GHDx
21070	Yemen	Yemen Demographic and Health Survey 1997	1997	I	I	I	-	-	I	-	-	I	-	-	Central Statistical Organization (Yemen), Macro International, Inc. Yemen Demographic and Health Survey 1997.	GHDx
22882	Yemen	Yemen Household Budget Survey 2005-2006	2005 - 2006	-	I	I	-	-	-	-	-	I	-	-	Central Statistical Organization (Yemen). Yemen Household Budget Survey 2005-2006. Sana'a, Yemen: Central Statistical Organization (Yemen).	GHDx
112500	Yemen	Yemen Demographic and Health Survey 2013	2013	I	I	I	I	I	I	-	I	I	-	-	Central Statistical Organization (Yemen), ICF International, Ministry of Public Health and Population (Yemen). Yemen Demographic and Health Survey 2013. Fairfax, United States of America: ICF International.	GHDx
142000	Yemen	Yemen Multiple Indicator Cluster Survey 1996	1996	I	I	-	-	-	I	-	-	-	-	-	Central Statistical Organization (Yemen), Ministry of Planning and Development (Yemen), United Nations Children's Fund (UNICEF). Yemen Multiple Indicator Cluster Survey 1996.	GHDx
228257	Yemen	Yemen Arab Republic Joint Government/WHO/Unicef EPI Coverage Review 1987	1987	I	I	I	-	-	I	-	-	I	-	-	Government of Yemen Arab Republic, World Health Organization (WHO). Yemen Arab Republic Joint Government/WHO/Unicef EPI Coverage Review 1987.	GHDx
14015	Zambia	Zambia Living Conditions Monitoring Survey 1998	1998	-	I	I	-	-	I	E	-	I	-	-	Central Statistical Office (Zambia), London School of Hygiene and Tropical Medicine. Zambia Living Conditions Monitoring Survey 1998. Lusaka, Zambia: Central Statistical Office (Zambia).	GHDx
14027	Zambia	Zambia Living Conditions Monitoring Survey 2002-2003	2002 - 2003	-	I	I	-	-	I	E	-	I	-	-	Central Statistical Office (Zambia). Zambia Living Conditions Monitoring Survey 2002-2003. Lusaka, Zambia: Central Statistical Office (Zambia).	GHDx
14111	Zambia	Zambia Multiple Indicator Cluster Survey 1995	1995	I	-	I	-	-	I	-	-	I	-	-	Centers for Disease Control and Prevention (CDC), Central Statistical Office (Zambia), Food Security, Health and Nutrition Information System (Zambia), Food and Agriculture Organization of the United Nations (FAO), United Nations Children's Fund (UNICEF). Zambia Multiple Indicator Cluster Survey 1995.	GHDx
14122	Zambia	Zambia Multiple Indicator Cluster Survey 1999	1999	E	I	I	-	-	I	-	-	I	-	-	Central Statistical Office (Zambia), Food Security, Health and Nutrition Information System (Zambia), United Nations Children's Fund (UNICEF). Zambia Multiple Indicator Cluster Survey 1999. New York, United States of America: United Nations Children's Fund (UNICEF).	GHDx
21079	Zambia	Zambia Demographic and Health Survey 1992	1992	I	I	I	-	-	I	-	-	I	-	-	Central Statistical Office (Zambia), Macro International, Inc, University of Zambia. Zambia Demographic and Health Survey 1992. Fairfax, United States of America: ICF International.	GHDx
21090	Zambia	Zambia Demographic and Health Survey 1996-1997	1996 - 1997	I	I	I	-	-	I	-	-	I	-	-	Central Statistical Office (Zambia), Macro International, Inc, Ministry of Health (Zambia). Zambia Demographic and Health Survey 1996-1997. Fairfax, United States of America: ICF International.	GHDx
21102	Zambia	Zambia Demographic and Health Survey 2001-2002	2001 - 2002	I	I	I	-	-	I	-	-	I	-	-	Central Board of Health (Zambia), Central Statistical Office (Zambia), Macro International, Inc. Zambia Demographic and Health Survey 2001-2002. Fairfax, United States of America: ICF International.	GHDx
21117	Zambia	Zambia Demographic and Health Survey 2007	2007	I	I	I	I	I	I	-	-	I	-	-	Central Statistical Office (Zambia), Macro International, Inc. Zambia Demographic and Health Survey 2007. Fairfax, United States of America: ICF International.	GHDx

GHDx ID	Country	Series	Year	BCG	DPT1	DPT3	HepB3	Hib3	MCV1	MCV2	PCV3	Pol3	RCV1	RotaC	Citation	Link
26702	Zambia	Zambia Global Fund Household Health Coverage Survey 2008	2008	I	I	I	I	-	I	-	-	I	-	-	Central Statistical Office (Zambia). Zambia Global Fund Household Health Coverage Survey 2008. Lusaka, Zambia: Central Statistical Office (Zambia).	GHDx
77516	Zambia	Zambia Demographic and Health Survey 2013-2014	2013 - 2014	I	I	I	I	I	I	-	-	I	-	-	Central Statistical Office (Zambia), ICF International, Ministry of Health (Zambia), Tropical Diseases Research Centre, University Teaching Hospital (Zambia), University of Zambia. Zambia Demographic and Health Survey 2013-2014. Fairfax, United States of America: ICF International.	GHDx
228195	Zambia	Zambia Expanded Program on Immunization Survey Using the Cluster Survey Methodology 2011	2011	I	I	I	I	I	I	-	-	I	-	-	Zambia Expanded Program on Immunization Survey using the cluster survey methodology 2011.	GHDx
228196	Zambia	Zambia EPI Cluster Survey Report 2001	2001	I	I	I	-	-	I	-	-	I	-	-	Ministry of Health (Zambia), World Health Organization (WHO). Zambia EPI Cluster Survey Report 2001.	GHDx
228217	Zambia	Zambia Immunization Coverage Survey 1991	1991	I	I	I	-	-	I	-	-	I	-	-	Ministry of Health (Zambia), World Health Organization (WHO). Zambia Immunization Coverage Survey 1991.	GHDx
284177	Zambia	Zambia Living Conditions Monitoring Survey 2015	2015	I	I	I	-	-	I	-	-	I	-	-	Central Statistical Office (Zambia), World Bank. Zambia Living Conditions Monitoring Survey 2015.	GHDx
21126	Zimbabwe	Zimbabwe Demographic and Health Survey 1988-1989	1988 - 1989	I	I	I	-	-	I	-	-	I	-	-	Central Statistical Office (Zimbabwe), Macro Systems, Inc.; Institute for Resource Development. Zimbabwe Demographic and Health Survey 1988-1989. Fairfax, United States of America: ICF International.	GHDx
21139	Zimbabwe	Zimbabwe Demographic and Health Survey 1994	1994	I	I	I	-	-	I	-	-	I	-	-	Central Statistical Office (Zimbabwe), Macro International, Inc. Zimbabwe Demographic and Health Survey 1994. Fairfax, United States of America: ICF International.	GHDx
21151	Zimbabwe	Zimbabwe Demographic and Health Survey 1999	1999	I	I	I	-	-	I	-	-	I	-	-	Central Statistical Office (Zimbabwe), Macro International, Inc. Zimbabwe Demographic and Health Survey 1999. Fairfax, United States of America: ICF International.	GHDx
21163	Zimbabwe	Zimbabwe Demographic and Health Survey 2005-2006	2005 - 2006	I	I	I	I	-	I	E	-	I	-	-	Central Statistical Office (Zimbabwe), Macro International, Inc. Zimbabwe Demographic and Health Survey 2005-2006. Fairfax, United States of America: ICF International.	GHDx
35493	Zimbabwe	Zimbabwe Multiple Indicator Monitoring Survey 2009	2009	I	I	I	I	-	I	-	-	I	-	-	Central Statistical Office (Zimbabwe). Zimbabwe Multiple Indicator Monitoring Survey 2009. New York, United States of America: United Nations Children's Fund (UNICEF).	GHDx
55992	Zimbabwe	Zimbabwe Demographic and Health Survey 2010-2011	2010 - 2011	I	I	I	I	I	I	-	-	I	-	-	ICF Macro, Zimbabwe National Statistics Agency. Zimbabwe Demographic and Health Survey 2010-2011. Calverton, United States of America: ICF Macro, 2012.	GHDx
141997	Zimbabwe	Zimbabwe National Maternal and Child Health Family Planning Survey 1997	1997	I	I	I	-	-	I	-	-	I	-	-	Zimbabwe National Maternal and Child Health Family Planning Survey 1997	GHDx
152720	Zimbabwe	Zimbabwe Multiple Indicator Cluster Survey 2014	2014	I	I	I	I	I	I	-	I	I	-	-	United Nations Children's Fund (UNICEF), Zimbabwe National Statistics Agency. Zimbabwe Multiple Indicator Cluster Survey 2014. New York, United States of America: United Nations Children's Fund (UNICEF), 2015.	GHDx
157066	Zimbabwe	Zimbabwe Demographic and Health Survey 2015	2015	I	I	I	I	I	I	-	I	I	-	I	ICF International, National Microbiology Reference Laboratory, Harare Central Hospital (NMRL) (Zimbabwe), Zimbabwe National Statistics Agency. Zimbabwe Demographic and Health Survey 2015. Fairfax, United States of America: ICF International, 2016.	GHDx
228136	Zimbabwe	Zimbabwe Routine Immunization Coverage Survey 2010	2010	I	I	I	-	-	I	-	-	I	-	-	Ministry of Health and Child Welfare (Zimbabwe). Zimbabwe Routine Immunization Coverage Survey 2010.	GHDx
431951	Zimbabwe	Zimbabwe Multiple Indicator Cluster Survey 2019	2019	I	I	I	I	I	I	-	I	I	I	I	United Nations Children's Fund (UNICEF), Zimbabwe National Statistics Agency. Zimbabwe Multiple Indicator Cluster Survey 2019. New York, United States of America: United Nations Children's Fund (UNICEF), 2020.	GHDx

Supplementary table 2. Data sources excluded from vaccine coverage modelling. Citations, series, country, years, and antigens are provided for each excluded dataset along with rationale for their exclusion. BCG=Bacillus Calmette-Guérin. DTP1=diphtheria-tetanus-pertussis, first dose. DTP3=diphtheria-tetanus-pertussis, third dose. HepB3=hepatitis B vaccine, third dose. Hib3=*Haemophilus influenzae* type b vaccine, third dose. MCV1=measles-containing vaccine, first dose. MCV2=measles-containing vaccine, second dose. PCV3=pneumococcal conjugate vaccine, third dose. Pol3=Pol vaccine, third dose. RCV1=rubella-containing vaccine, first dose. RotaC=completed rotavirus series.

Country	Series	Year(s)	Cohort	Vaccine	Citation	Rationale for exclusion
Afghanistan	Afghanistan Multiple Indicator Cluster Survey 2003	2003	all	MCV1	Central Statistics Organization (Afghanistan), United Nations Children's Fund (UNICEF). Afghanistan Multiple Indicator Cluster Survey 2003.	MCV estimates are systematically high compared to admin estimates and estimates from other established surveys series (Afghanistan Health Survey 2006). MCV removed from analysis.
Afghanistan	Afghanistan Health Survey 2006	2006	all	BCG	Indian Institute of Health Management Research (IIHMR), Johns Hopkins University, Ministry of Public Health (Afghanistan). Afghanistan Health Survey 2006.	BCG estimates include implausibly high coverage rates of 100%. BCG removed from analysis.
Afghanistan	Afghanistan Demographic and Health Survey 2015-2016	2015-2016	select	PCV3	Central Statistics Organization (Afghanistan), ICF International, Ministry of Public Health (Afghanistan). Afghanistan Demographic and Health Survey 2015-2016. Fairfax, United States of America: ICF International, 2017.	Older age-cohorts precede introduction of PCV3 to national immunization schedule. Select age-cohorts removed from analysis for PCV3.
Afghanistan	EPI Coverage Situation in Women and Children of Afghanistan, Report of Post NID's, Routine Coverage and Acceleration Campaign Survey in Afghanistan 1999	1999	all	BCG, DPT1, DPT3, MCV1, Pol3	EPI Coverage Situation in Women and Children of Afghanistan, Report of Post NID's, Routine Coverage and Acceleration Campaign Survey in Afghanistan 1999.	Estimates considered implausible. Survey estimates are systematically high compared to admin estimates and estimates from other established surveys series (Afghanistan Multiple Indicator Cluster Survey 2003). Survey removed from analysis.
Albania	Albania Multiple Indicator Cluster Survey 2000	2000	all	BCG, DPT1, DPT3, MCV1, Pol3	National Institute of Statistics (Albania), United Nations Children's Fund (UNICEF). Albania Multiple Indicator Cluster Survey 2000. New York, United States: United Nations Children's Fund (UNICEF).	Estimates considered implausible. Survey estimates are systematically low compared to admin estimates and estimates from other established surveys series. Survey removed from analysis.
Albania	Albania Multiple Indicator Cluster Survey 2005	2005	all	BCG, DPT1, DPT3, MCV1, Pol3	National Institute of Statistics (Albania), United Nations Children's Fund (UNICEF). Albania Multiple Indicator Cluster Survey 2005. New York, United States: United Nations Children's Fund (UNICEF).	Estimates considered implausible. Survey estimates are systematically low compared to admin estimates and estimates from other established surveys series. Survey removed from analysis.
Albania	Albania Demographic and Health Survey 2017-2018	2017-2018	all	BCG, DPT1, DPT3, HepB3, PCV3, MCV1, PCV3, Pol3	Albania Institute of Public Health (IPH), Albania Institute of Statistics (INSTAT), ICF International. Albania Demographic and Health Survey 2017-2018. Fairfax, United States of America: ICF International, 2019.	Survey data is based off health facility vaccination registries. Absence of maternal recall introduces potential bias; survey removed from analysis.
Algeria	Algeria Multiple Indicator Cluster Survey 2000	2000	all	DPT1	Ministry of Health and Population (Algeria), National Institute of Public Health (Algeria), National Office of Statistics (Algeria), United Nations Children's Fund (UNICEF). Algeria Multiple Indicator Cluster Survey 2000.	Survey reports higher DPT third dose coverage than first dose coverage. DPT1 removed from analysis due to reliability concerns.
Angola	Angola Integrated Inquiry into People's Well-Being 2008-2009	2008-2009	select	HepB3, PCV3	National Institute of Statistics (Angola), Oxford Policy Management, United Nations Children's Fund (UNICEF). Angola Integrated Inquiry into People's Well-Being 2008-2009.	Older age-cohorts precede introduction of PCV3 and HepB3 to national immunization schedule. Select age-cohorts removed from analysis for PCV3 and HepB3.
Angola	Angola Core Welfare Indicators Questionnaire Survey 2011	2011	all	BCG, DPT1, DPT3,	Ministry of Planning and Territorial Development (Angola), National Institute of Statistics (Angola). Angola	Estimates considered implausible. Survey estimates are systematically low compared to admin estimates and

Country	Series	Year(s)	Cohort	Vaccine	Citation	Rationale for exclusion
				MCV1, Pol3	Core Welfare Indicators Questionnaire Survey 2011. Luanda, Angola: National Institute of Statistics (Angola).	estimates from other established surveys series. Survey removed from analysis.
Angola	Angola Demographic and Health Survey 2015-2016	2015-2016	select	MCV2, RotaC	ICF International, Ministry of Health (Angola), National Institute of Statistics (Angola), United Nations Children's Fund (UNICEF). Angola Demographic and Health Survey 2015-2016. Fairfax, United States of America: ICF International, 2017.	Older age-cohorts precede introduction of MCV2 and RotaC to national immunization schedule. Select age-cohorts removed from analysis for MCV2 and RotaC.
Armenia	Armenia Demographic and Health Survey 2000	2000	select	HepB3	Ministry of Health (Armenia), National Statistical Service (NSS), ORC Macro. Armenia Demographic and Health Survey 2000. Fairfax, United States of America: ICF International.	Older age-cohorts precede introduction of HepB3 to national immunization schedule. Select age-cohorts removed from analysis for HepB3.
Armenia	Armenia Demographic and Health Survey 2005	2005	all	BCG, DPT1, DPT3, HepB3, MCV1, Pol3	MOH Center for Health Information and Statistics, Macro International, Inc, National Statistical Service (NSS). Armenia Demographic and Health Survey 2005. Fairfax, United States of America: ICF International.	Survey removed from analysis due to small sample size and reliability concerns.
Armenia	Armenia Demographic and Health Survey 2010	2010	select	Hib3	ICF Macro, Ministry of Health (Armenia), National Statistical Service of the Republic of Armenia. Armenia Demographic and Health Survey 2010. Fairfax, United States of America: ICF International, 2015.	Older age-cohorts precede introduction of Hib3 to national immunization schedule. Select age-cohorts removed from analysis for Hib3.
Armenia	Armenia Demographic and Health Survey 2015-2016	2015-2016	select	PCV3	ICF International, Ministry of Health (Armenia), National Statistical Service of the Republic of Armenia. Armenia Demographic and Health Survey 2015-2016. Fairfax, United States of America: ICF International, 2017.	Older age-cohorts precede introduction of PCV3 to national immunization schedule. Select age-cohorts removed from analysis for PCV3.
Australia	Australia National Health Survey 2001	2001	select	Pol3	Australian Bureau of Statistics. Australia National Health Survey 2001. Canberra, Australia: Australian Bureau of Statistics.	Pol3 estimates do not follow a reasonable age structure. Older children have significantly higher coverage than reported in either administrative data or other survey series, suggesting survey is capturing results of "catch-up" immunization. Select age-cohorts removed from analysis for Pol3.
Bahrain	Bahrain Child Health Survey 1989	1989	select	DPT1, DPT3, MCV1, Pol3, RCV1	Central Statistics Organization (Bahrain), Ministry of Health (Bahrain). Bahrain Child Health Survey 1989.	Estimates considered implausible. Survey estimates do not follow a reasonable age structure. Older children have significantly higher coverage than reported in either administrative data or other survey series, suggesting survey is capturing results of "catch-up" immunization. Select age-cohorts removed from analysis.
Bahrain	Bahrain Diarrhoeal Diseases Morbidity, Mortality and Treatment Rates and Immunization Coverage 1986	1986	all	DPT1, DPT3, MCV1, Pol3	Ministry of Health (Bahrain). Bahrain Diarrhoeal Diseases Morbidity, Mortality and Treatment Rates and Immunization Coverage 1986.	Estimates considered implausible. Survey estimates are systematically low compared to admin estimates and estimates from other established surveys series. Survey removed from analysis.
Bangladesh	Bangladesh Demographic and Health Survey 2011-2012	2011-2012	select	Hib3	ICF Macro, Mitra and Associates, National Institute of Population Research and Training (NIPORT). Bangladesh Demographic and Health Survey 2011-2012. Calverton, United States of America: ICF Macro.	Older age-cohorts precede introduction of Hib3 to national immunization schedule. Select age-cohorts removed from analysis for Hib3.
Belarus	Belarus Multiple Indicator Cluster Survey 2005	2005	select	BCG, DPT1, DPT3, Pol3	Ministry of Statistics and Analysis of the Republic of Belarus, United Nations Children's Fund (UNICEF). Belarus Multiple Indicator Cluster Survey 2005. New York, United States of America: United Nations Children's Fund (UNICEF).	Estimates considered implausible. Survey estimates do not follow a reasonable age structure. Older children have significantly higher coverage than reported in either administrative data or other survey series, suggesting survey is capturing results of "catch-up" immunization. Select age-cohorts removed from analysis.
Belize	Belize Multiple Indicator Cluster Survey 2015-2016	2015-2016	all	MCV2	Government of Belize, Statistical Institute of Belize, UN Resident Coordinator Fund (UN ResCor), United Nations Children's Fund (UNICEF), United Nations Development	MCV2 estimates are implausibly low compared to admin estimates and estimates from other established surveys series. MCV2 removed from analysis.

Country	Series	Year(s)	Cohort	Vaccine	Citation	Rationale for exclusion
					Programme (UNDP). Belize Multiple Indicator Cluster Survey 2015-2016. New York, United States of America: United Nations Children's Fund (UNICEF), 2018.	
Benin	Benin Demographic and Health Survey 2006	2006	select	Hib3	Macro International, Inc, National Institute of Statistics and Economic Analysis (INSAE) (Benin), National Program Against AIDS (PNLS) (Benin). Benin Demographic and Health Survey 2006. Fairfax, United States of America: ICF International.	Older age-cohorts precede introduction of Hib3 to national immunization schedule. Select age-cohorts removed from analysis for Hib3.
Benin	Benin Demographic and Health Survey 2011-2012	2011-2012	all	BCG, DPT1, DPT3, HepB3, PCV3, MCV1, Pol3	ICF International, National Institute of Statistics and Economic Analysis (INSAE) (Benin), National Program Against AIDS (PNLS) (Benin). Benin Demographic and Health Survey 2011-2012. Fairfax, United States of America: ICF International, 2014.	Estimates considered implausible. Survey estimates are inconsistent with admin estimates and estimates from other established survey series (Benin Demographic and Health Survey 2006). Survey removed from analysis.
Bhutan	Bhutan National EPI Coverage Evaluation Survey 2002	2002	all	BCG, DPT1, DPT3, HepB3, MCV1, Pol3	Government of Bhutan, United Nations Children's Fund (UNICEF), World Health Organization (WHO). Bhutan National EPI Coverage Evaluation Survey 2002.	Survey estimates include implausibly high coverage rates of 100%. Survey removed from analysis.
Bolivia	Bolivia Integrated Household Survey November 1989	1989	all	BCG, DPT1, DPT3, MCV1	National Institute of Statistics (Bolivia). Bolivia Integrated Household Survey November 1989. La Paz, Bolivia: National Institute of Statistics (Bolivia).	Estimates considered implausible. Survey estimates are inconsistent with admin estimates and estimates from other established survey series (Bolivia Demographic and Health Survey 1989). Survey removed from analysis.
Bolivia	Bolivia Integrated Household Survey February 1989	1989	all	BCG, DPT1, DPT3, MCV1	National Institute of Statistics (Bolivia). Bolivia Integrated Household Survey February 1989. La Paz, Bolivia: National Institute of Statistics (Bolivia).	Estimates considered implausible. Survey estimates are inconsistent with admin estimates and estimates from other established survey series (Bolivia Demographic and Health Survey 1989). Survey removed from analysis.
Bolivia	Bolivia Demographic and Health Survey 2016	2016	select	PCV3	Ministry of Health (Bolivia), National Institute of Statistics (Bolivia). Bolivia Demographic and Health Survey 2016. La Paz, Bolivia: National Institute of Statistics (Bolivia), 2017.	Older age-cohorts precede introduction of PCV to national immunization schedule. Select age-cohorts removed from analysis for PCV.
Brazil	Brazil Demographic and Health Survey 1986	1986	select	BCG, DPT1, DPT3, MCV1, MCV2, Pol3	Brazilian Society for Family Welfare (BEMFAM), Westinghouse; Institute for Resource Development. Brazil Demographic and Health Survey 1986. Fairfax, United States: ICF International.	Estimates considered implausible. Survey estimates do not follow a reasonable age structure. Older children have significantly higher coverage than reported in either administrative data or other survey series, suggesting survey is capturing results of "catch-up" immunization. Select age-cohorts removed from analysis.
Brazil	Brazil Demographic and Health Survey 1991	1991	select	BCG, DPT1, DPT3, MCV1, Pol3	Brazilian Society for Family Welfare (BEMFAM), Macro International, Inc. Brazil Demographic and Health Survey 1991. Fairfax, United States: ICF International.	Estimates considered implausible. Survey estimates do not follow a reasonable age structure. Older children have significantly higher coverage than reported in either administrative data or other survey series, suggesting survey is capturing results of "catch-up" immunization. Select age-cohorts removed from analysis.
Brazil	Brazil Demographic and Health Survey 1996	1996	select	BCG, DPT1, DPT3, MCV1, Pol3	Brazilian Society for Family Welfare (BEMFAM), Macro International, Inc. Brazil Demographic and Health Survey 1996. Fairfax, United States of America: ICF International.	Estimates considered implausible. Survey estimates do not follow a reasonable age structure. Older children have significantly higher coverage than reported in either administrative data or other survey series, suggesting survey is capturing results of "catch-up" immunization. Select age-cohorts removed from analysis.
Brazil	Brazil National Demographic and Health Survey of Children and Women 2006-2007	2006-2007	all	DPT1	Brazilian Center for Analysis and Planning (CEBRAP), Brazilian Institute of Public Opinion and Statistics (IBOPE), Ministry of Health (Brazil). Brazil National Demographic and Health Survey of Children and Women	DPT1 estimates are implausibly low compared to admin estimates and estimates from other established surveys series. DPT1 removed from analysis.

Country	Series	Year(s)	Cohort	Vaccine	Citation	Rationale for exclusion
					2006-2007. Rio de Janeiro, Brazil: Ministry of Health (Brazil).	
Burundi	Burundi Priority Survey 1998-1999	1998-1999	all	BCG	Burundi Institute of Statistics and Economic Studies. Burundi Priority Survey 1998-1999.	BCG estimates include implausibly high coverage rates of 100%. BCG removed from analysis.
Cambodia	Cambodia Demographic and Health Survey 2010-2011	2010-2011	all	Hib3	ICF Macro, Ministry of Health (Cambodia), National Institute of Statistics (Cambodia). Cambodia Demographic and Health Survey 2010-2011. Fairfax, United States of America: ICF International.	Survey precedes introduction of Hib3 to national immunization schedule. Hib3 removed from analysis.
Cambodia	Cambodia Socio-Economic Survey 1996	1996	select	DPT1, DPT3	National Institute of Statistics (Cambodia). Cambodia Socio-Economic Survey 1996. Phnom Penh, Cambodia: National Institute of Statistics (Cambodia).	DPT1 and DPT3 estimates include implausibly low coverage rates of 0% for some age-cohorts. Select age cohorts removed from analysis for DPT1 and DPT3.
Cambodia	Cambodia Socio-Economic Survey 1999	1999	all	BCG, DPT1	National Institute of Statistics (Cambodia), United Nations Development Programme (UNDP), World Bank. Cambodia Socio-Economic Survey 1999. Phnom Penh, Cambodia: National Institute of Statistics (Cambodia).	Estimates considered implausible. Survey estimates are systematically high compared to admin estimates and estimates from other established surveys series. Survey removed from analysis.
Cambodia	Cambodia Socio-Economic Survey 1997	1997	all	BCG, DPT1, DPT3, MCV1, Pol3	National Institute of Statistics (Cambodia), World Bank. Cambodia Socio-Economic Survey 1997. Phnom Penh, Cambodia: National Institute of Statistics (Cambodia).	Survey estimates include implausibly high coverage rates of 100%. Survey removed from analysis.
Cambodia	Cambodia Vaccine Coverage Survey of Children 12-23 Months of Age 1995	1995	all	BCG, DPT1	Cambodia Vaccine Coverage Survey of Children 12-23 Months of Age 1995.	BCG and DPT1 estimates are systematically high compared to admin estimates and estimates from other established surveys series (Cambodia Special Demographic and Health Survey 1998). BCG and DPT1 removed from analysis.
Cameroon	Cameroon Demographic and Health Survey 2011	2011	select	Hib3	ICF International, Ministry of Economy, Planning and Regional Development (Cameroon), Ministry of Public Health (Cameroon), National Institute of Statistics (Cameroon), Pasteur Center of Cameroon. Cameroon Demographic and Health Survey 2011. Fairfax, United States of America: ICF International.	Older age-cohorts precede introduction of Hib3 to national immunization schedule. Select age-cohorts removed from analysis for Hib3.
Cameroon	Cameroon Multiple Indicator Cluster Survey 2014	2014	select	BCG, DPT1, DPT3, HepB3, PCV3, PCV3, Pol3	Ministry of Public Health (Cameroon), National Institute of Statistics (Cameroon), United Nations Children's Fund (UNICEF). Cameroon Multiple Indicator Cluster Survey 2014. New York, United States of America: United Nations Children's Fund (UNICEF), 2017.	Survey estimates include implausibly high coverage rates of 100% for some age cohorts. Select age cohorts removed from analysis.
Cape Verde	Cape Verde Survey on DTP3 and Measles Coverage 2009	2009	all	DPT3, MCV1	Cape Verde Survey on DTP3 and Measles Coverage 2009.	Survey estimates include implausibly high coverage rates of 100%. Survey removed from analysis.
Central African Republic	Central African Republic Multiple Indicator Cluster Survey 2010-2011	2010-2011	select	HepB3	Central African Institute of Statistics, Economic and Social Studies (ICASEES) (Central African Republic), ICF International. Central African Republic Multiple Indicator Cluster Survey 2010-2011. Fairfax, United States of America: ICF International, 2013.	Older age-cohorts precede introduction of HepB to national immunization schedule. Select age cohorts removed from analysis for HepB.
Central African Republic	Central African Republic Public Expenditure Review 2012	1986-2010	all	BCG, DPT3, HepB3, PCV3, MCV1, Pol3	World Bank. Central African Republic Public Expenditure Review 2012. Washington DC, United States: World Bank, 2012.	Estimates considered implausible. Survey estimates are systematically high compared to admin estimates and estimates from other established surveys series. Survey removed from analysis.
Central African Republic	Central African Republic EPI Coverage Survey 2012	2012	all	Pol3	Ministry of Public Health, Population, and AIDS Control (Central African Republic). Central African Republic EPI Coverage Survey 2012.	Pol3 estimates are implausibly high compared to admin estimates and estimates from other established surveys series (Central African Republic Multiple Indicator Cluster Survey 2010-2011). Pol3 removed from analysis.

Country	Series	Year(s)	Cohort	Vaccine	Citation	Rationale for exclusion
Chad	Chad Multiple Indicator Cluster Survey 2010	2010	select	HepB3	Ministry of Planning, Economy, and International Cooperation (Chad), National Institute for Statistics, Economic and Demographic Studies (INSEED) (Chad), United Nations Children's Fund (UNICEF). Chad Multiple Indicator Cluster Survey 2010. New York, United States of America: United Nations Children's Fund (UNICEF), 2014.	Older age-cohorts precede introduction of HepB to national immunization schedule. Select age-cohorts removed from analysis for HepB.
China	China Health Statistics Yearbook 2005	2004	all	BCG, DPT3, MCV1, Pol3	Ministry of Health (China). China Health Statistics Yearbook 2005.	Estimates considered implausible. Survey estimates are systematically high compared to admin estimates and estimates from other established survey series. Survey removed from analysis.
China	China Health Statistics Yearbook 2007	2006	all	BCG, DPT3, MCV1, Pol3	Ministry of Health (China). China Health Statistics Yearbook 2007.	Estimates considered implausible. Survey estimates are systematically high compared to admin estimates and estimates from other established survey series. Survey removed from analysis.
China	China Health Statistics Yearbook 2009	2008	all	BCG, DPT3, MCV1, Pol3	Ministry of Health (China). China Health Statistics Yearbook 2009.	Estimates considered implausible. Survey estimates are systematically high compared to admin estimates and estimates from other established survey series. Survey removed from analysis.
China	Survey on the immunization status of category B vaccine among children aged 1 to 2 years in China	2009-2011	all	DPT3, PCV3, PCV3	Zheng Jing-shan, Cao Lei, Guo Shi-cheng. Survey on the immunization status of category B vaccine among children aged 1 to 2 years in China. Chin J Vaccines Immun. 2012; 18(3): 2337.	Survey removed from analysis due to small sample size and reliability concerns.
Colombia	Colombia National Health Survey 2007-2008	2007-2008	all	DPT1, DPT3	Administrative Department of Science, Technology, and Innovation (Colombia), Center for Development Projects, Pontifical Xavierian University, Ministry of Social Protection (Colombia), Specialized Information Systems. Colombia National Health Survey 2007-2008.	Survey estimates include implausibly low coverage rates of 0%. Survey removed from analysis.
Colombia	Colombia Demographic and Health Survey 2000	2000	select	Hib3	Macro International, Inc, Profamilia (Colombia). Colombia Demographic and Health Survey 2000. Fairfax, United States of America: ICF International, 2000.	Older age-cohorts precede introduction of Hib3 to national immunization schedule. Select age-cohorts removed from analysis for Hib3.
Comoros	Comoros Demographic and Health Survey 2012-2013	2012-2013	select	Hib3	General Directorate of Statistics and Forecasting (Comoros), ICF International. Comoros Demographic and Health Survey 2012-2013. Fairfax, United States of America: ICF International.	Older age-cohorts precede introduction of Hib3 to national immunization schedule. Select age-cohorts removed from analysis for Hib3.
Congo (Brazzaville)	Congo Demographic and Health Survey 2005	2005	select	BCG, DPT1, DPT3, MCV1, Pol3	Macro International, Inc, National Center for Statistics and Economic Studies (Congo, Rep.). Congo Demographic and Health Survey 2005. Fairfax, United States of America: ICF International.	Estimates considered implausible. Survey estimates do not follow a reasonable age structure. Older children have significantly higher coverage than reported in either administrative data or other survey series, suggesting survey is capturing results of "catch-up" immunization. Select age-cohorts removed from analysis.
Congo (Brazzaville)	Congo World Health Survey 2003	2003	all	DPT1, DPT3, MCV1	World Health Organization (WHO). Congo World Health Survey 2003. Geneva, Switzerland: World Health Organization (WHO), 2005.	Estimates considered implausible. Survey estimates are inconsistent with admin estimates and estimates from other established survey series. Survey removed from analysis.
Congo (Brazzaville)	Congo Demographic and Health Survey 2011-2012	2011-2012	select	Hib3	ICF International, Ministry of Health (Congo, Rep.), National Center for Statistics and Economic Studies (Congo, Rep.). Congo Demographic and Health Survey 2011-2012. Fairfax, United States of America: ICF International.	Older age-cohorts precede introduction of Hib3 to national immunization schedule. Select age-cohorts removed from analysis for Hib3.
Congo (Brazzaville)	Congo External EPI Review 2010	2010	all	HepB3, PCV3	Congo External EPI Review 2010.	Estimates considered implausible. Survey estimates are systematically high compared to admin estimates and estimates from other established surveys series (Congo Demographic and Health Survey 2011-2012). Survey removed from analysis.

Country	Series	Year(s)	Cohort	Vaccine	Citation	Rationale for exclusion
Costa Rica	Costa Rica Multiple Indicator Cluster Survey 2011	2011	select	PCV3	Costa Rican Demographic Association, Ministry of Health (Costa Rica), United Nations Children's Fund (UNICEF). Costa Rica Multiple Indicator Cluster Survey 2011. New York, United States of America: United Nations Children's Fund (UNICEF), 2013.	Older age-cohorts precede introduction of PCV3 to national immunization schedule. Select age-cohorts removed from analysis for PCV3.
Costa Rica	Costa Rica National Health Survey 2006	2006	all	DPT1, MCV1	Central American Population Center, University of Costa Rica. Costa Rica National Health Survey 2006. San Jose, Costa Rica: Central American Population Center, University of Costa Rica.	Estimates considered implausible. Survey estimates are systematically low compared to admin estimates and estimates from other established surveys series (Costa Rica Survey of Family Health Services and Expenses 2008). Survey removed from analysis.
Côte d'Ivoire	Côte d'Ivoire Demographic and Health Survey 2011-2012	2011-2012	select	Hib3	ICF International, Ministry of the Fight Against AIDS (Côte d'Ivoire), National Institute of Statistics (Côte d'Ivoire). Côte d'Ivoire Demographic and Health Survey 2011-2012. Fairfax, United States of America: ICF International.	Older age-cohorts precede introduction of Hib to national immunization schedule. Select age cohorts removed from analysis for Hib.
Côte d'Ivoire	Côte d'Ivoire Immunization Coverage Survey 1987	1987	all	DPT1, DPT3, MCV1, Pol3	Côte d'Ivoire Immunization Coverage Survey 1987.	Estimates considered implausible. Survey estimates are systematically high compared to admin estimates and estimates from other established surveys series (Côte d'Ivoire Demographic and Health Survey 1994). Survey removed from analysis.
Côte d'Ivoire	Côte d'Ivoire Multiple Indicator Cluster Survey 2016	2016	all	DPT1, RotaC	National Institute of Statistics (Côte d'Ivoire), United Nations Children's Fund (UNICEF). Côte d'Ivoire Multiple Indicator Cluster Survey 2016. New York, United States of America: United Nations Children's Fund (UNICEF), 2018.	Survey estimates include implausibly high coverage rates of 100%. Survey removed from analysis.
Croatia	Croatia Multiple Indicator Cluster Survey 1996	1996	all	BCG, DPT3	Children's Hospital Zagreb, National Institute of Health (Italy), United Nations Children's Fund (UNICEF). Croatia Multiple Indicator Cluster Survey 1996.	Estimates considered implausible. Survey estimates are systematically low compared to admin estimates and estimates from other established surveys series. Survey removed from analysis.
DR Congo	Democratic Republic of the Congo Multiple Indicator Cluster Survey 2010	2010	select	HepB3	National Statistical Institute (Congo, DR), Ministry of Planning (Congo, DR), United Nations Children's Fund (UNICEF). Congo, DR Multiple Indicator Cluster Survey 2010. New York, United States: United Nations Children's Fund (UNICEF).	Older age-cohorts precede introduction of HepB to national immunization schedule. Select age cohorts removed from analysis for HepB.
Denmark	Denmark Act No. 707, Act to Amend the Law Prohibiting the Sale of Tobacco to Persons Under the Age of 18, and the Sale of Alcohol to Persons Under the Age of 16, 2010	2010	all	BCG, DPT1, DPT3, MCV1, Pol3	Ministry of Interior and Health (Denmark). Denmark Act No. 707, Act to Amend the Law Prohibiting the Sale of Tobacco to Persons Under the Age of 18, and the Sale of Alcohol to Persons Under the Age of 16, 2010. Copenhagen, Denmark: Government of Denmark.	Estimates considered implausible. Survey estimates are systematically low compared to admin estimates and estimates from other established surveys series. Survey removed from analysis.
Djibouti	Djibouti Household Survey - Social Indicators 1996	1996	all	BCG, DPT1, DPT3, MCV1, Pol3	National Directorate of Statistics, Ministry of Commerce and Tourism, World Bank (WB). Djibouti Household Survey - Social Indicators 1996. Djibouti, Djibouti: National Directorate of Statistics, Ministry of Commerce and Tourism.	Estimates considered implausible. Survey estimates are systematically high compared to admin estimates and estimates from other established surveys series. Survey removed from analysis.
Djibouti	Djibouti Family Health Survey 2002	2002	select	BCG, DPT1, DPT3, MCV1, Pol3	Department of Statistics and Demographic Studies (Djibouti), League of Arab States, Ministry of Health (Djibouti), Pan Arab Project for Family Health (PAPFAM). Djibouti Family Health Survey 2002.	Estimates considered implausible. Survey estimates do not follow a reasonable age structure. Older children have significantly higher coverage than reported in either administrative data or other survey series, suggesting survey is capturing results of "catch-up" immunization. Select age-cohorts removed from analysis.
Djibouti	Djibouti Family Health Survey 2012	2012	all	MCV2	Department of Statistics and Demographic Studies (Djibouti), League of Arab States, Ministry of Health	Survey precedes introduction of MCV2 to national immunization schedule. MCV2 removed from analysis.

Country	Series	Year(s)	Cohort	Vaccine	Citation	Rationale for exclusion
					(Djibouti), Pan Arab Project for Family Health (PAPFAM). Djibouti Family Health Survey 2012.	
Dominican Republic	Dominican Republic National Multipurpose Household Survey 2006	2006	select	MCV2, Pol3, RCV1	National Statistics Office (Dominican Republic), United Nations Children's Fund (UNICEF). Dominican Republic National Multipurpose Household Survey 2006. Santo Domingo, Dominican Republic: National Statistics Office (Dominican Republic).	MCV2, Pol3, and RCV1 estimates are systematically low compared to admin estimates and estimates from other established surveys series. MCV2, Pol3, and RCV1 removed from analysis.
Dominican Republic	Dominican Republic Demographic and Health Survey 2002	2002	select	Hib3	Center for Social and Demographic Studies (Dominican Republic) (CESDEM), Macro International, Inc. Dominican Republic Demographic and Health Survey 2002. Fairfax, United States of America: ICF International.	Older age-cohorts precede introduction of Hib3 to national immunization schedule. Select age-cohorts removed from analysis for Hib3.
Dominican Republic	Dominican Republic Demographic and Health Survey 2007	2007	select	RCV1	Center for Social and Demographic Studies (Dominican Republic) (CESDEM), Macro International, Inc. Dominican Republic Demographic and Health Survey 2007. Fairfax, United States of America: ICF International.	Older age-cohorts precede introduction of RCV1 to national immunization schedule. Select age-cohorts removed from analysis for RCV1.
Dominican Republic	Dominican Republic Multiple Indicator Cluster Survey 2000	2000	all	MCV1	Center for Social and Demographic Studies (Dominican Republic) (CESDEM), United Nations Children's Fund (UNICEF). Dominican Republic Multiple Indicator Cluster Survey 2000. New York, United States of America: United Nations Children's Fund (UNICEF).	MCV1 estimates are implausibly low compared to admin estimates and estimates from other established survey series. MCV1 removed from analysis.
Dominican Republic	Dominican Republic Demographic and Health Survey 2013	2013	select	RotaC	Center for Social and Demographic Studies (Dominican Republic) (CESDEM), ICF International, Ministry of Public Health and Social Assistance (Dominican Republic). Dominican Republic Demographic and Health Survey 2013. Fairfax, United States of America: ICF International, 2014.	Older age-cohorts precede introduction of rotavirus vaccine to national immunization schedule. Rotavirus vaccine removed from analysis.
Ecuador	Ecuador Reproductive Health Survey 2004	2004	select	DPT1, DPT3, MCV1	Center for Studies of Population and Social Development (CEPAR) (Ecuador) and Division of Reproductive Health-Centers for Disease Control and Prevention (CDC). (2005) Ecuador Reproductive Health Survey 2004. Quito, Ecuador: CEPAR.	DPT1, DPT3, and MCV1 estimates considered implausible for some age-cohorts due to sudden drop in coverage. Coverage trends are inconsistent with admin estimates and estimates from other established survey series (Ecuador Living Conditions Survey 2005-2006). Select age-cohorts removed from analysis for DPT1, DPT3, and MCV1.
Ecuador	Ecuador National Health and Nutrition Survey 2012	2012	select	PCV3, Pol3	Ministry of Public Health (Ecuador), National Institute of Statistics and Censuses (Ecuador). Ecuador National Health and Nutrition Survey 2012.	Pol3 estimates considered implausible due to sudden drop in coverage inconsistent with admin estimates and estimates from other established survey series (Ecuador Living Conditions Survey 2013-2014). Older age-cohorts precede introduction of PCV3 to national immunization schedule. Pol3 and select age-cohorts of PCV3 removed from analysis.
Ecuador	Ecuador Living Conditions Survey 2013-2014	2013-2014	select	PCV3	National Institute of Statistics and Censuses (Ecuador). Ecuador Living Conditions Survey 2013-2014. Quito, Ecuador: National Institute of Statistics and Censuses (Ecuador).	Older age-cohorts precede introduction of PCV3 to national immunization schedule. Select age-cohorts removed from analysis for PCV3.
Egypt	Egypt Demographic and Health Survey 1992-1993	1992-1993	all	HepB3	Macro International, Inc, Population Council (Egypt). Egypt Demographic and Health Survey 1992-1993. Fairfax, United States of America: ICF International.	Survey precedes introduction of HepB3 to national immunization schedule. HepB3 removed from analysis.
Egypt	Egypt Demographic and Health Survey 1995-1996	1995-1996	select	HepB3	Macro International, Inc, Population Council (Egypt). Egypt Demographic and Health Survey 1995-1996. Fairfax, United States of America: ICF International.	Older age-cohorts precede introduction of HepB to national immunization schedule. Select age cohorts removed from analysis for HepB.
Egypt	Egypt Demographic and Health Survey 2014	2014	all	PCV3	El-Zanaty and Associates, ICF International, Ministry of Health and Population (Egypt). Egypt Demographic and	Survey precedes introduction of PCV3 to national immunization schedule. PCV3 removed from analysis.

Country	Series	Year(s)	Cohort	Vaccine	Citation	Rationale for exclusion
					Health Survey 2014. Fairfax, United States of America: ICF International.	
El Salvador	El Salvador Demographic and Health Survey 1985	1985	all	BCG, DPT1, DPT3, MCV1, Pol3	Salvadoran Demographic Association (ADS), Westinghouse; Institute for Resource Development. El Salvador Demographic and Health Survey 1985. Fairfax, United States: ICF International.	Estimates considered implausible. Survey estimates are systematically high compared to admin estimates and estimates from other established surveys series. Survey removed from analysis.
El Salvador	El Salvador Reproductive Health Survey 2002-2003	2002-2003	select	DPT1, DPT3, MCV1	Asociación Demográfica Salvadoreña (ADS), Division of Reproductive Health-Centers for Disease Control and Prevention (CDC). (2004) El Salvador Reproductive Health Survey 2002-2003. San Salvador, El Salvador: ADS.	Estimates considered implausible. Survey estimates are systematically low compared to admin estimates and estimates from other established surveys series. Survey removed from analysis.
El Salvador	El Salvador Reproductive Health Survey 2008	2008	all	BCG, DPT1, DPT3, MCV1, Pol3	Asociación Demográfica Salvadoreña (ADS), Division of Reproductive Health-Centers for Disease Control and Prevention (CDC). (2009) El Salvador Reproductive Health Survey 2008. San Salvador, El Salvador: ADS.	Estimates considered implausible. Survey estimates are systematically low compared to admin estimates and estimates from other established surveys series. Survey removed from analysis.
Equatorial Guinea	Equatorial Guinea Demographic and Health Survey 2011	2011	all	HepB3, PCV3, RCV1	ICF International, Ministry of Health and Social Welfare (Equatorial Guinea), Ministry of Planning, Economic Development and Public Investment (Equatorial Guinea). Equatorial Guinea Demographic and Health Survey 2011. Fairfax, United States of America: ICF International, 2012.	Survey precedes introduction of BCG, PCV, and RCV to national immunization schedule. BCG, PCV, and RCV removed from analysis.
Equatorial Guinea	Equatorial Guinea National Immunization Coverage Survey 1994	1994	all	BCG, DPT1, DPT3, MCV1, Pol3	Equatorial Guinea National Immunization Coverage Survey 1994.	Estimates considered implausible. Survey estimates are systematically high compared to admin estimates and estimates from other established surveys series (Equatorial Guinea Multiple Indicator Cluster Survey 2000). Survey removed from analysis.
eSwatini	Swaziland Multiple Indicator Cluster Survey 2014	2014	all	PCV3	Central Statistical Office (Swaziland), United Nations Children's Fund (UNICEF), United Nations Educational, Scientific and Cultural Organization (UNESCO), United Nations Population Fund (UNFPA). Swaziland Multiple Indicator Cluster Survey 2014. New York, United States of America: United Nations Children's Fund (UNICEF), 2016.	Survey precedes introduction of PCV3 to national immunization schedule. PCV3 removed from analysis.
Ethiopia	Ethiopia Health and Nutrition Survey 1998	1998	all	BCG, DPT3, MCV1, Pol3	Central Statistical Agency (Ethiopia). Ethiopia Health and Nutrition Survey 1998.	Estimates considered implausible. Survey estimates are systematically high compared to admin estimates and estimates from other established surveys series (Ethiopia Demographic and Health Survey 2000). Survey removed from analysis.
Ethiopia	Ethiopia National Fertility and Family Survey 1990-1991	1990-1991	all	BCG, DPT1, DPT3, MCV1, Pol3	Central Statistical Agency (Ethiopia). Ethiopia National Fertility and Family Survey 1990-1991. Addis Ababa, Ethiopia: Central Statistical Agency (Ethiopia).	Survey estimates include implausibly high coverage rates of 100%. Survey removed from analysis.
Ethiopia	Ethiopia Demographic and Health Survey 2010-2011	2010-2011	select	HepB3, Hib3	Central Statistical Agency (Ethiopia), ICF Macro, Ministry of Health (Ethiopia). Ethiopia Demographic and Health Survey 2010-2011. Fairfax, United States of America: ICF International.	Survey precedes introduction of Hepb3 and Hib3 to national immunization schedule. Hepb3 and Hib3 removed from analysis.
Ethiopia	Ethiopia World Health Survey 2003	2003	all	DPT1, DPT3, MCV1	World Health Organization (WHO). Ethiopia World Health Survey 2003. Geneva, Switzerland: World Health Organization (WHO), 2005.	Estimates considered implausible. Survey estimates are systematically high compared to admin estimates and estimates from other established surveys series (Ethiopia Demographic and Health Survey 2005). Survey removed from analysis.

Country	Series	Year(s)	Cohort	Vaccine	Citation	Rationale for exclusion
Ethiopia	Ethiopia Health and Health Related Indicators 2003-2004	2003-2004	all	BCG, DPT3, MCV1	Ministry of Health (Ethiopia). Ethiopia Health and Health Related Indicators 2003-2004. Addis Ababa, Ethiopia: Ministry of Health (Ethiopia), 2004.	Estimates considered implausible. Survey estimates are systematically high compared to admin estimates and estimates from other established surveys series (Ethiopia Demographic and Health Survey 2005). Survey removed from analysis.
Ethiopia	Ethiopia Health and Health Related Indicators 2006-2007	2006-2007	all	DPT3, MCV1	Ministry of Health (Ethiopia). Ethiopia Health and Health Related Indicators 2006-2007. Addis Ababa, Ethiopia: Ministry of Health (Ethiopia), 2007.	Estimates considered implausible. Survey estimates are systematically high compared to admin estimates and estimates from other established surveys series (Ethiopia Demographic and Health Survey 2005). Survey removed from analysis.
Ethiopia	Ethiopia Welfare Monitoring Survey 1998	1998	all	BCG, DPT3, MCV1, Pol3	Central Statistical Agency (Ethiopia). Ethiopia Welfare Monitoring Survey 1998.	Estimates considered implausible. Survey estimates are systematically high compared to admin estimates and estimates from other established surveys series (Ethiopia Demographic and Health Survey 2000). Survey removed from analysis.
Ethiopia	Ethiopia Welfare Monitoring Survey 2004	2004	all	BCG, DPT1, DPT3, MCV1, Pol3	Central Statistical Agency (Ethiopia). Ethiopia Welfare Monitoring Survey 2004.	Estimates considered implausible. Survey estimates are systematically high compared to admin estimates and estimates from other established surveys series (Ethiopia Demographic and Health Survey 2005). Survey removed from analysis.
Ethiopia	Ethiopia Health and Health Related Indicators 2009-2010	2009-2010	all	DPT1, DPT3, HepB3, PCV3, MCV1	Ministry of Health (Ethiopia). Ethiopia Health and Health Related Indicators 2009-2010. Addis Ababa, Ethiopia: Ministry of Health (Ethiopia).	Estimates considered implausible. Survey estimates are systematically high compared to admin estimates and estimates from other established surveys series (Ethiopia Demographic and Health Survey 2010-2011). Survey removed from analysis.
Ethiopia	Ethiopia National EPI Coverage Survey 2001	2001	all	BCG, DPT1, DPT3, MCV1, Pol3	Ethiopia National EPI Coverage Survey 2001.	Estimates considered implausible. Survey estimates are systematically high compared to admin estimates and estimates from other established surveys series (Ethiopia Demographic and Health Survey 2000). Survey removed from analysis.
Ethiopia	Ethiopia Health and Health Related Indicators 2002-2003	2002-2003	all	BCG, DPT3, MCV1	Ministry of Health (Ethiopia). Ethiopia Health and Health Related Indicators 2002-2003. Addis Ababa, Ethiopia: Ministry of Health (Ethiopia), 2004.	Estimates considered implausible. Survey estimates are systematically high compared to admin estimates and estimates from other established surveys series (Ethiopia Demographic and Health Survey 2000, Ethiopia Demographic and Health Survey 2005). Survey removed from analysis.
Ethiopia	Ethiopia Health and Health Related Indicators 2004-2005	2004-2005	all	BCG, DPT3, MCV1	Ministry of Health (Ethiopia). Ethiopia Health and Health Related Indicators 2004-2005. Addis Ababa, Ethiopia: Ministry of Health (Ethiopia), 2005.	Estimates considered implausible. Survey estimates are systematically high compared to admin estimates and estimates from other established surveys series (Ethiopia Demographic and Health Survey 2005). Estimates removed from analysis.
Ethiopia	Ethiopia Health and Health Related Indicators 2005-2006	2005-2006	all	BCG, DPT3, MCV1, Pol3	Ministry of Health (Ethiopia). Ethiopia Health and Health Related Indicators 2005-2006. Addis Ababa, Ethiopia: Ministry of Health (Ethiopia), 2006.	Estimates considered implausible. Survey estimates are systematically high compared to admin estimates and estimates from other established surveys series (Ethiopia Demographic and Health Survey 2005). Survey removed from analysis.
Ethiopia	Ethiopia Health and Health Related Indicators 2008-2009	2008-2009	all	DPT3, HepB3, PCV3, MCV1	Ministry of Health (Ethiopia). Ethiopia Health and Health Related Indicators 2008-2009. Addis Ababa, Ethiopia: Ministry of Health (Ethiopia).	Estimates considered implausible. Survey estimates are systematically high compared to admin estimates and estimates from other established surveys series (Ethiopia Demographic and Health Survey 2010-2011). Survey removed from analysis.
Ethiopia	Ethiopia Health and Health Related Indicators 2010-2011	2010-2011	all	DPT3, HepB3, PCV3, MCV1	Ministry of Health (Ethiopia). Ethiopia Health and Health Related Indicators 2010-2011. Addis Ababa, Ethiopia: Ministry of Health (Ethiopia), 2012.	Estimates considered implausible. Survey estimates are systematically high compared to admin estimates and estimates from other established surveys series (Ethiopia

Country	Series	Year(s)	Cohort	Vaccine	Citation	Rationale for exclusion
						Demographic and Health Survey 2010-2011). Survey removed from analysis.
Ethiopia	Ethiopia Young Lives: Household and Child Survey Round 5 2016 - UK Data Service	2016	all	BCG, DPT3, HepB3, PCV3, MCV1, PCV3, Pol3	Boyden, J., Duc, L. Thuc, Galab, S., Penny, M., Sanchez, A., Woldehanna, T. (2018). Young Lives: an International Study of Childhood Poverty: Round 5, 2016. [data collection]. UK Data Service. SN: 8357, http://doi.org/10.5255/UKDA-SN-8357-1.	Estimates considered implausible. Survey estimates are systematically high compared to admin estimates and estimates from other established surveys series (Ethiopia Demographic and Health Survey 2016). Survey removed from analysis.
Gabon	Gabon Demographic and Health Survey 2000-2001	2000-2001	all	HepB3, Hib3	General Directorate of Statistics and Economic Studies (Gabon), Macro International, Inc. Gabon Demographic and Health Survey 2000-2001. Fairfax, United States of America: ICF International.	Survey precedes introduction of HepB and Hib3 to national immunization schedule. HepB and Hib3 removed from analysis.
Gabon	Gabon Demographic and Health Survey 2012	2012	select	Hib3	General Directorate of Statistics (Gabon), ICF International, Ministry of Economy, Employment and Sustainable Development (Gabon), Ministry of Health (Gabon). Gabon Demographic and Health Survey 2012. Fairfax, United States of America: ICF International, 2013.	Survey precedes introduction of Hib3 to national immunization schedule. Hib3 removed from analysis.
The Gambia	Gambia Demographic and Health Survey 2013	2013	all	MCV2	Gambia Bureau of Statistics (GBOS), ICF International, Ministry of Health and Social Welfare (The Gambia). Gambia Demographic and Health Survey 2013. Fairfax, United States of America: ICF International, 2015.	Survey precedes introduction of MCV2 to national immunization schedule. MCV2 removed from analysis.
The Gambia	Gambia Multiple Indicator Cluster Survey 2010	2010	all	HepB3	Gambia Bureau of Statistics (GBOS), United Nations Children's Fund (UNICEF). Gambia Multiple Indicator Cluster Survey 2010. New York, United States of America: United Nations Children's Fund (UNICEF), 2018.	HepB3 estimates are systematically low compared to admin estimates and estimates from other established surveys series (Gambia Demographic and Health Survey 2013). HepB3 removed from analysis.
Georgia	Georgia Multiple Indicator Cluster Survey 2005	2005	all	BCG, DPT1, DPT3, MCV1, Pol3	National Center for Disease Control (Georgia), State Department of Statistics of Georgia, United Nations Children's Fund (UNICEF). Georgia Multiple Indicator Cluster Survey 2005. New York, United States of America: United Nations Children's Fund (UNICEF).	Estimates considered implausible. Survey estimates are systematically low compared to admin estimates and estimates from other established surveys series. Survey removed from analysis.
Ghana	Ghana Multiple Indicator Cluster Survey 2006	2006	all	RCV1	Ministry of Health (MOH) (Ghana), Ghana Statistical Service and United Nations Children's Fund (UNICEF). Ghana Multiple Indicator Cluster Survey 2006. New York, United States: United Nations Children's Fund (UNICEF).	Survey precedes introduction of RCV1 to national immunization schedule. RCV1 removed from analysis.
Ghana	Ghana World Health Survey 2003	2003	all	DPT1, DPT3, MCV1	World Health Organization (WHO). Ghana World Health Survey 2003. Geneva, Switzerland: World Health Organization (WHO), 2005.	Estimates considered implausible. Survey estimates are inconsistent with admin estimates and estimates from other established survey series (Ghana Demographic and Health Survey 2003). Survey removed from analysis.
Ghana	Ghana Demographic and Health Survey 2014	2014	select	MCV2, PCV3, RotaC	Ghana Health Service, Ghana Statistical Service, ICF International. Ghana Demographic and Health Survey 2014. Fairfax, United States of America: ICF International, 2016.	Older age-cohorts precede introduction of MCV2, PCV3, and RotaC to national immunization schedule. Select age-cohorts removed from analysis for MCV2, PCV3, and RotaC.
Ghana	Ghana District Multiple Indicator Cluster Survey 2007-2008	2007-2008	all	RCV1	Ghana Statistical Service, Ministry of Health (Ghana), United Nations Children's Fund (UNICEF). Ghana District Multiple Indicator Cluster Survey 2007-2008.	Survey precedes introduction of RCV1 to national immunization schedule. RCV1 removed from analysis.
Ghana	Ghana Living Standards Measurement Survey 2012-2013	2012-2013	all	BCG, DPT1, DPT3, HepB3, PCV3,	Ghana Statistical Service, World Bank. Ghana Living Standards Measurement Survey 2012-2013. Accra, Ghana: Ghana Statistical Service.	Estimates considered implausible. Survey estimates are systematically low compared to admin estimates and estimates from other established surveys series (Ghana Multiple Indicator Cluster Survey 2011). Survey removed from analysis.

Country	Series	Year(s)	Cohort	Vaccine	Citation	Rationale for exclusion
				MCV1, Pol3		
Ghana	Ghana Socioeconomic Panel Survey 2009-2010	2009-2010	all	DPT3	Economic Growth Center, Yale University, Institute of Statistical, Social and Economic Research, University of Ghana. Ghana Socioeconomic Panel Survey 2009-2010. Washington DC, United States: World Bank.	DPT3 estimates are systematically low compared to admin estimates and estimates from other established surveys series (Ghana Multiple Indicator Cluster Survey 2011). DPT3 removed from analysis.
Guatemala	Guatemala Demographic and Health Survey 1987	1987	all	MCV1	Institute of Nutrition of Central America and Panama, Westinghouse; Institute for Resource Development. Guatemala Demographic and Health Survey 1987. Fairfax, United States: ICF International.	MCV1 estimates are systematically low compared to admin estimates and estimates from other established surveys series (Guatemala Demographic and Health Survey 1995). MCV1 removed from analysis.
Guatemala	Guatemala National Survey of Living Conditions 2006	2006	all	HepB3, Hib3	National Institute of Statistics (Guatemala), National Statistical System (Guatemala), World Bank (WB), United Nations Development Programme (UNDP), United Nations Economic Commission for Latin America and the Caribbean (CEPAL), Rafael Landavar University, United Nations Population Fund (UNFPA), Norwegian Agency for Development (NORAD), Swedish International Development Agency (SIDA), Secretary of Planning and Programming (SEGEPLAN) (Guatemala), Bank of Guatemala. Guatemala National Survey of Living Conditions 2006. Guatemala City, Guatemala: National Statistics Institute (Guatemala).	Survey precedes introduction of HepB3 and Hib3 to national immunization schedule. HepB3 and Hib3 removed from analysis.
Guatemala	Guatemala Demographic and Health Survey 2014-2015	2014-2015	select	MCV1, PCV3	ICF International, Institute of Nutrition of Central America and Panama, Ministry of Public Health and Social Assistance (Guatemala), National Statistics Institute (Guatemala), Secretary of Planning and Programming of the Presidency (Segeplan) (Guatemala). Guatemala Demographic and Health Survey 2014-2015. Fairfax, United States of America: ICF International, 2017.	MCV1 estimates are systematically high compared to admin estimates and estimates from other established surveys series. Older age-cohorts precede introduction of PCV3 to national immunization schedule. MCV1 and select age-cohorts of PCV3 removed from analysis.
Guinea	Guinea National Survey on Nutritional Status and Tracking Key Indicators of Child Survival 2007-2008	2007-2008	all	HepB3	National Institute of Statistics (Guinea). Guinea National Survey on Nutritional Status and Tracking Key Indicators of Child Survival 2007-2008.	Survey precedes introduction of HepB3 to national immunization schedule. HepB3 removed from analysis.
Guinea-Bissau	Guinea-Bissau Multiple Indicator Cluster Survey 2000	2000	select	DPT3, MCV1	Secretary State of Planning, National Institute of Statistics and Census (INEC), United Nations Children's Fund (UNICEF). Guinea-Bissau Multiple Indicator Cluster Survey 2000. New York, United States: United Nations Children's Fund (UNICEF).	MCV1 estimates do not follow a reasonable age structure. Older children have significantly higher coverage than reported in either administrative data or other survey series, suggesting survey is capturing results of "catch-up" immunization. Select DPT3 age-cohorts are also removed from analysis due to implausibly low coverage compared to admin estimates and estimates from other established survey series (Guinea-Bissau Multiple Indicator Cluster Survey 2006).
Guyana	Guyana Demographic and Health Survey 2009	2009	all	MCV2	Bureau of Statistics (Guyana), ICF Macro, Ministry of Health (Guyana). Guyana Demographic and Health Survey 2009. Fairfax, United States of America: ICF International, 2011.	MCV2 estimates are systematically low compared to admin estimates and estimates from other established surveys series (Guyana Multiple Indicator Cluster Survey 2006-2007). MCV2 removed from analysis.
Honduras	Honduras Survey of Living Conditions 2004	2004	all	BCG, DPT1, DPT3, MCV1, MCV2, Pol3, RCV1	National Institute of Statistics (Honduras). Honduras Survey of Living Conditions 2004. Tegucigalpa, Honduras: National Institute of Statistics (Honduras).	Estimates considered implausible. Survey estimates are inconsistent with admin estimates and estimates from other established survey series (Honduras Demographic and Health Survey 2005-2006). Survey removed from analysis.

Country	Series	Year(s)	Cohort	Vaccine	Citation	Rationale for exclusion
Honduras	Honduras Reproductive Health Survey 1996	1996	all	MCV2	Ministry of Health (Honduras), Honduras Family Planning Association (ASHONPLAFA) and Division of Reproductive Health-Centers for Disease Control and Prevention (CDC). (1997) Honduras Reproductive Health Survey 1996. Tegucigalpa, Honduras: ASHONPLAFA.	Survey precedes introduction of MCV2 to national immunization schedule. MCV2 removed from analysis.
Honduras	Honduras Demographic and Health Survey 2011-2012	2011-2012	select	PCV3, RotaC	ICF Macro, National Institute of Statistics (Honduras). Honduras Demographic and Health Survey 2011-2012. Fairfax, United States of America: ICF International.	Older age-cohorts precede introduction of PCV3 and RotaC to national immunization schedule. Select age-cohorts removed from analysis for PCV3 and RotaC.
India	India National Sample Survey Round 52 1995-1996	1995-1996	all	BCG, DPT3, MCV1, Pol3	Ministry of Statistics and Programme Implementation (India). India National Sample Survey Round 52 1995-1996. New Delhi, India: Ministry of Statistics and Programme Implementation (India).	Estimates considered implausible. Survey estimates are systematically high compared to admin estimates and estimates from other established surveys series (India Demographic and Health Survey 1992-1993). Survey removed from analysis.
India	India District Level Household Survey 2007-2008	2007-2008	all	BCG, DPT1, DPT3, MCV1, Pol3	International Institute for Population Sciences (India). India District Level Household Survey 2007-2008. Mumbai, India: International Institute for Population Sciences (India), 2010.	Estimates considered implausible. Survey estimates are systematically high compared to admin estimates and estimates from other established surveys series (India Demographic and Health Survey 2005-2006). Survey removed from analysis.
India	India Human Development Survey 2004-2005	2004-2005	all	BCG, DPT1, DPT3, MCV1, Pol3	Desai, Sonalde, Reeve Vanneman, and National Council of Applied Economic Research, New Delhi. India Human Development Survey (IHDS), 2005. ICPSR22626-v8. Ann Arbor, MI: Inter-university Consortium for Political and Social Research [distributor], 2010-06-29. doi:10.3886/ICPSR22626.v8.	Estimates considered implausible. Survey estimates are systematically low compared to admin estimates and estimates from other established surveys series (India Demographic and Health Survey 2005-2006). Survey removed from analysis.
India	India Family Welfare Statistics 2011	1980-2011	all	BCG, DPT3, MCV1, Pol3	Ministry of Health and Family Welfare (India). India Family Welfare Statistics 2011. New Delhi, India: Ministry of Health and Family Welfare (India), 2011.	Estimates considered implausible. Survey estimates are systematically high compared to admin estimates and estimates from other established surveys series (India Demographic and Health Survey 2005-2006). Survey removed from analysis.
India	India Coverage Evaluation Survey 2009-2010	2009-2010	all	BCG, DPT1, DPT3, HepB3, MCV1, Pol3, RCV1	Ministry of Health and Family Welfare (India), ORG Centre for Social Research (ORG CSR), United Nations Children's Fund (UNICEF). India Coverage Evaluation Survey 2009-2010.	Estimates considered implausible. Survey estimates are systematically high compared to admin estimates and estimates from other established surveys series (India Demographic and Health Survey 2005-2006). Survey removed from analysis.
India	India Concurrent Evaluation of National Rural Health Mission 2009	2009	all	BCG, DPT1, DPT3, MCV1, Pol3	International Institute for Population Sciences (India), Ministry of Health and Family Welfare (India). India Concurrent Evaluation of National Rural Health Mission 2009.	Estimates considered implausible. Survey estimates are systematically high compared to admin estimates and estimates from other established surveys series (India Demographic and Health Survey 2005-2006). Survey removed from analysis.
Indonesia	Indonesia National Socioeconomic Survey 2005	2005	all	DPT1, DPT3, HepB3, MCV1, MCV2, Pol3	Statistics Indonesia. Indonesia National Socioeconomic Survey 2005.	Estimates considered implausible. Survey estimates are inconsistent with admin estimates and estimates from other established survey series (Indonesia Demographic and Health Survey 2007). Survey removed from analysis.
Indonesia	Indonesia National Socioeconomic Survey 2006	2006	all	DPT1, DPT3, HepB3, MCV1, MCV2, Pol3	Statistics Indonesia. Indonesia National Socioeconomic Survey 2006.	Estimates considered implausible. Survey estimates are inconsistent with admin estimates and estimates from other established survey series (Indonesia Demographic and Health Survey 2007). Survey removed from analysis.

Country	Series	Year(s)	Cohort	Vaccine	Citation	Rationale for exclusion
Indonesia	Indonesia Family Life Survey 1997	1997	all	BCG, DPT1, DPT3, HepB3, MCV1, Pol3	Macro International, Inc, RAND Corporation, University of California, Los Angeles (UCLA), University of Indonesia. Indonesia Family Life Survey 1997.	Survey estimates include implausibly high coverage rates of 100%. Survey removed from analysis.
Indonesia	Indonesia Family Life Survey 2000	2000	all	BCG, DPT1, DPT3, HepB3, MCV1, Pol3	Center for Population and Policy Studies, Gadjah Mada University (Indonesia), RAND Corporation. Indonesia Family Life Survey 2000. Santa Monica, United States of America: RAND Corporation.	Survey estimates include implausibly high coverage rates of 100%. Survey removed from analysis.
Indonesia	Indonesia National Socioeconomic Survey 1992	1992	all	DPT1, DPT3, Pol3	Central Bureau of Statistics (Indonesia). Indonesia National Socioeconomic Survey 1992.	Estimates considered implausible. Survey estimates are inconsistent with admin estimates and estimates from other established survey series (Indonesia Demographic and Health Survey 1994). Survey removed from analysis.
Indonesia	Indonesia National Socioeconomic Survey 1993	1993	all	DPT1, DPT3, Pol3	Central Bureau of Statistics (Indonesia). Indonesia National Socioeconomic Survey 1993.	Estimates considered implausible. Survey estimates are inconsistent with admin estimates and estimates from other established survey series (Indonesia Demographic and Health Survey 1994). Survey removed from analysis.
Indonesia	Indonesia National Socioeconomic Survey 1994	1994	all	DPT1, DPT3, Pol3	Central Bureau of Statistics (Indonesia). Indonesia National Socioeconomic Survey 1994.	Estimates considered implausible. Survey estimates are inconsistent with admin estimates and estimates from other established survey series (Indonesia Demographic and Health Survey 1994). Survey removed from analysis.
Indonesia	Indonesia National Socioeconomic Survey 1995	1995	all	DPT1, DPT3, Pol3	Central Bureau of Statistics (Indonesia), Ministry of Health (Indonesia), United Nations Children's Fund (UNICEF). Indonesia National Socioeconomic Survey 1995.	Estimates considered implausible. Survey estimates are inconsistent with admin estimates and estimates from other established survey series (Indonesia Demographic and Health Survey 1994). Survey removed from analysis.
Indonesia	Indonesia National Socioeconomic Survey 1996	1996	all	DPT1, DPT3, Pol3	Central Bureau of Statistics (Indonesia), Ministry of Health (Indonesia), United Nations Children's Fund (UNICEF). Indonesia National Socioeconomic Survey 1996.	Estimates considered implausible. Survey estimates are inconsistent with admin estimates and estimates from other established survey series (Indonesia Demographic and Health Survey 1994, Indonesia Demographic and Health Survey 1997). Survey removed from analysis.
Indonesia	Indonesia National Socioeconomic Survey 1997	1997	all	DPT1, DPT3, Pol3	Central Bureau of Statistics (Indonesia), Ministry of Health (Indonesia), United Nations Children's Fund (UNICEF). Indonesia National Socioeconomic Survey 1997.	Estimates considered implausible. Survey estimates are inconsistent with admin estimates and estimates from other established survey series (Indonesia Demographic and Health Survey 1994, Indonesia Demographic and Health Survey 1997). Survey removed from analysis.
Indonesia	Indonesia National Socioeconomic Survey 1998	1998	all	DPT1, DPT3, MCV1, MCV2, Pol3	Central Bureau of Statistics (Indonesia), Ministry of Health (Indonesia), World Bank. Indonesia National Socioeconomic Survey 1998.	Estimates considered implausible. Survey estimates are inconsistent with admin estimates and estimates from other established survey series (Indonesia Demographic and Health Survey 1997). Survey removed from analysis.
Indonesia	Indonesia National Socioeconomic Survey 1999	1999	all	DPT1, DPT3, MCV1, MCV2, Pol3	Central Bureau of Statistics (Indonesia), Ministry of Health (Indonesia), World Bank. Indonesia National Socioeconomic Survey 1999.	Estimates considered implausible. Survey estimates are inconsistent with admin estimates and estimates from other established survey series (Indonesia Demographic and Health Survey 1997, Indonesia Demographic and Health Survey 2002-2003). Survey removed from analysis.
Indonesia	Indonesia National Socioeconomic Survey 2001	2001	all	MCV1	Central Bureau of Statistics (Indonesia), Ministry of Health (Indonesia), World Bank. Indonesia National Socioeconomic Survey 2001.	Estimates considered implausible. Survey estimates are inconsistent with admin estimates and estimates from other established survey series (Indonesia Demographic and Health Survey 1997, Indonesia Demographic and Health Survey 2002-2003). Survey removed from analysis.

Country	Series	Year(s)	Cohort	Vaccine	Citation	Rationale for exclusion
Indonesia	Indonesia National Socioeconomic Survey 2004	2004	all	DPT1, DPT3, HepB3, MCV1, MCV2, Pol3	Statistics Indonesia. Indonesia National Socioeconomic Survey 2004.	Estimates considered implausible. Survey estimates are inconsistent with admin estimates and estimates from other established survey series (Indonesia Demographic and Health Survey 2002-2003). Survey removed from analysis.
Indonesia	Indonesia National Socioeconomic Survey 2007	2007	all	DPT1, DPT3, HepB3, MCV1, MCV2, Pol3	Statistics Indonesia. Indonesia National Socioeconomic Survey 2007.	Estimates considered implausible. Survey estimates are inconsistent with admin estimates and estimates from other established survey series (Indonesia Demographic and Health Survey 2002-2003, Indonesia Demographic and Health Survey 2007). Survey removed from analysis.
Indonesia	Indonesia National Socioeconomic Survey 2010	2010	all	DPT1, DPT3, HepB3, MCV1, MCV2, Pol3	Statistics Indonesia. Indonesia National Socioeconomic Survey 2010.	Estimates considered implausible. Survey estimates are inconsistent with admin estimates and estimates from other established survey series (Indonesia Demographic and Health Survey 2007, Indonesia Demographic and Health Survey 2012). Survey removed from analysis.
Indonesia	Indonesia National Socioeconomic Survey 2008	2008	all	DPT1, DPT3, HepB3, MCV1, MCV2, Pol3	Statistics Indonesia. Indonesia National Socioeconomic Survey 2008.	Estimates considered implausible. Survey estimates are inconsistent with admin estimates and estimates from other established survey series (Indonesia Demographic and Health Survey 2007, Indonesia Demographic and Health Survey 2012). Survey removed from analysis.
Indonesia	Indonesia National Socioeconomic Survey 2009	2009	all	DPT1, DPT3, HepB3, MCV1, MCV2, Pol3	Statistics Indonesia. Indonesia National Socioeconomic Survey 2009.	Estimates considered implausible. Survey estimates are inconsistent with admin estimates and estimates from other established survey series (Indonesia Demographic and Health Survey 2007, Indonesia Demographic and Health Survey 2012). Survey removed from analysis.
Indonesia	Indonesia National Socioeconomic Survey - Poverty Program Evaluation 2006	2006	all	DPT1, DPT3, HepB3, MCV1, MCV2, Pol3	Statistics Indonesia. Indonesia National Socioeconomic Survey - Poverty Program Evaluation 2006. Jakarta, Indonesia: Statistics Indonesia.	Estimates considered implausible. Survey estimates are inconsistent with admin estimates and estimates from other established survey series (Indonesia Demographic and Health Survey 2007). Survey removed from analysis.
Indonesia	Indonesia National Socioeconomic Survey 2011	2011	all	DPT1, DPT3, HepB3, MCV1, Pol3	Statistics Indonesia. Indonesia National Socioeconomic Survey 2011.	Estimates considered implausible. Survey estimates are inconsistent with admin estimates and estimates from other established survey series (Indonesia Demographic and Health Survey 2007, Indonesia Demographic and Health Survey 2012). Survey removed from analysis.
Indonesia	Indonesia National Socioeconomic Survey 2012	2012	all	DPT1, DPT3, HepB3, MCV1, Pol3	Central Bureau of Statistics (Indonesia). Indonesia National Socioeconomic Survey 2012. Jakarta, Indonesia: Central Bureau of Statistics (Indonesia).	Estimates considered implausible. Survey estimates are inconsistent with admin estimates and estimates from other established survey series (Indonesia Demographic and Health Survey 2012). Survey removed from analysis.
Indonesia	Indonesia National Socioeconomic Survey 2013	2013	all	DPT1, DPT3, HepB3, MCV1, MCV2, Pol3	Statistics Indonesia. Indonesia National Socioeconomic Survey 2013. Jakarta, Indonesia: Statistics Indonesia.	Estimates considered implausible. Survey estimates are inconsistent with admin estimates and estimates from other established survey series (Indonesia Demographic and Health Survey 2012). Survey removed from analysis.
Indonesia	Indonesia Family Life Survey East 2012	2012	all	BCG, DPT1, DPT3,	National Team for the Acceleration of Poverty Reduction (TNP2K) (Indonesia), SurveyMETER, University of	Estimates considered implausible. Survey estimates do not follow a reasonable age structure. Older children have significantly higher coverage than reported in either

Country	Series	Year(s)	Cohort	Vaccine	Citation	Rationale for exclusion
				HepB3, MCV1, Pol3	Southern California, World Bank. Indonesia Family Life Survey East 2012.	administrative data or other survey series, suggesting survey is capturing results of "catch-up" immunization. Survey removed from analysis.
Indonesia	Indonesia Health Profile 2016	1991-2016	all	BCG, DPT1, DPT3, HepB3, PCV3, MCV1, Pol3	Ministry of Health (Indonesia). Indonesia Health Profile 2016. Jakarta, Indonesia: Ministry of Health (Indonesia), 2017.	Estimates considered implausible. Survey estimates are systematically high compared to admin estimates and estimates from other established surveys series (Indonesia Demographic and Health Survey 1994, Indonesia Demographic and Health Survey 1997, Indonesia Demographic and Health Survey 2002-2003, Indonesia Demographic and Health Survey 2007, Indonesia Demographic and Health Survey 2012). Survey removed from analysis.
Indonesia	Indonesia National Socioeconomic Survey 2017	2017	all	RCV1	Central Bureau of Statistics (Indonesia). Indonesia National Socioeconomic Survey 2017. Jakarta, Indonesia: Central Bureau of Statistics (Indonesia), 2018.	Survey precedes introduction of RCV1 to national immunization schedule. RCV1 removed from analysis.
Indonesia	Indonesia Basic Health Research 2018	2018	all	BCG, DPT1, DPT3, HepB3, PCV3, MCV1, Pol3	Agency of Health Research and Development (Indonesia). Indonesia Basic Health Research 2018.	Estimates considered implausible. Survey estimates are inconsistent with admin estimates and estimates from other established survey series (Indonesia Demographic and Health Survey 2017). Survey removed from analysis.
Iran	Iran Multiple Indicator Demographic and Health Survey 2010	2010	select	MCV2	Ministry of Health and Medical Education (Iran), Statistical Centre of Iran. Iran Multiple Indicator Demographic and Health Survey 2010.	Oldest age-cohort for MCV2 removed from analysis due to implausible drop in coverage.
Iran	Iran Communicable Diseases and Vaccination Coverage Surveillance 2006-2010	2006-2010	all	BCG, DPT3, HepB3, MCV1, Pol3, RCV1	Ministry of Health and Medical Education (Iran). Iran Communicable Diseases and Vaccination Coverage Surveillance 2006-2010 .	Survey estimates include implausibly high coverage rates of 100%. Survey removed from analysis.
Iraq	Iraq Multiple Indicator Cluster Survey 2018	2018	select	PCV3	Central Statistical Organization (Iraq), United Nations Children's Fund (UNICEF). Iraq Multiple Indicator Cluster Survey 2018. New York, United States of America: United Nations Children's Fund (UNICEF), 2019.	Older age-cohorts precede introduction of PCV3 to national immunization schedule. Select age-cohorts removed from analysis for PCV3.
Jamaica	Jamaica Survey of Living Conditions 1988	1988-1998	all	BCG, DPT1, DPT3, MCV1, Pol3	Planning Institute of Jamaica, Statistical Institute of Jamaica. Jamaica Survey of Living Conditions 1988. Kingston, Jamaica: Planning Institute of Jamaica.	Estimates considered implausible. Survey estimates are systematically high compared to admin estimates and estimates from other established surveys series (Jamaica Multiple Indicator Cluster Survey 2005). Survey removed from analysis.
Jamaica	Jamaica Survey of Living Conditions 1991	1991	all	BCG, DPT1, DPT3, MCV1, Pol3	Planning Institute of Jamaica, Statistical Institute of Jamaica. Jamaica Survey of Living Conditions 1991.	Estimates considered implausible. Survey estimates are systematically high compared to admin estimates and estimates from other established surveys series (Jamaica Multiple Indicator Cluster Survey 2005). Survey removed from analysis.
Jamaica	Jamaica Survey of Living Conditions 1993	1993	all	BCG, DPT1, DPT3, MCV1, Pol3	Planning Institute of Jamaica, Statistical Institute of Jamaica. Jamaica Survey of Living Conditions 1993.	Estimates considered implausible. Survey estimates are systematically high compared to admin estimates and estimates from other established surveys series (Jamaica Multiple Indicator Cluster Survey 2005). Survey removed from analysis.
Jamaica	Jamaica Survey of Living Conditions 1995	1995	all	BCG, DPT1, DPT3,	Planning Institute of Jamaica, Statistical Institute of Jamaica. Jamaica Survey of Living Conditions 1995.	Estimates considered implausible. Survey estimates are systematically high compared to admin estimates and estimates from other established surveys series (Jamaica

Country	Series	Year(s)	Cohort	Vaccine	Citation	Rationale for exclusion
				MCV1, Pol3		Multiple Indicator Cluster Survey 2005). Survey removed from analysis.
Jamaica	Jamaica Survey of Living Conditions 1996	1996	all	BCG, DPT1, DPT3, MCV1, Pol3	Planning Institute of Jamaica, Statistical Institute of Jamaica. Jamaica Survey of Living Conditions 1996.	Estimates considered implausible. Survey estimates are systematically high compared to admin estimates and estimates from other established surveys series (Jamaica Multiple Indicator Cluster Survey 2005). Survey removed from analysis.
Jamaica	Jamaica Survey of Living Conditions 1997	1997	all	BCG, DPT1, DPT3, MCV1, Pol3	Planning Institute of Jamaica, Statistical Institute of Jamaica. Jamaica Survey of Living Conditions 1997.	Estimates considered implausible. Survey estimates are systematically high compared to admin estimates and estimates from other established surveys series (Jamaica Multiple Indicator Cluster Survey 2005). Survey removed from analysis.
Jamaica	Jamaica Survey of Living Conditions 2000	2000	all	BCG, DPT1, DPT3, MCV1, Pol3	Planning Institute of Jamaica, Statistical Institute of Jamaica. Jamaica Survey of Living Conditions 2000.	Estimates considered implausible. Survey estimates are systematically high compared to admin estimates and estimates from other established surveys series (Jamaica Multiple Indicator Cluster Survey 2005). Survey removed from analysis.
Jamaica	Jamaica Multiple Indicator Cluster Survey 2011	2011	all	HepB3, PCV3	Statistical Institute of Jamaica, United Nations Children's Fund (UNICEF). Jamaica Multiple Indicator Cluster Survey 2011. New York, United States of America: United Nations Children's Fund (UNICEF), 2015.	Estimates considered implausible. Survey estimates are systematically low compared to admin estimates and estimates from other established surveys series. Survey removed from analysis.
Jordan	Jordan Demographic and Health Survey 1997	1997	select	MCV2	Department of Statistics (Jordan), Macro International, Inc. Jordan Demographic and Health Survey 1997. Fairfax, United States: ICF International.	Older age-cohorts precede introduction of MCV2 to national immunization schedule. Select age-cohorts removed from analysis for MCV2.
Jordan	Jordan Demographic and Health Survey 2002	2002	select	Hib3, RCV1	Department of Statistics (Jordan), Macro International, Inc. Jordan Demographic and Health Survey 2002. Fairfax, United States of America: ICF International.	Older age-cohorts precede introduction of Hib3 and RCV to national immunization schedule. Select age-cohorts removed from analysis for Hib3 and RCV.
Kazakhstan	Kazakhstan Multiple Indicator Cluster Survey 2006	2006	all	BCG	Agency of the Republic of Kazakhstan on Statistics and United Nations Children's Fund (UNICEF). Kazakhstan Multiple Indicator Cluster Survey 2006. New York, United States: United Nations Children's Fund (UNICEF).	BCG coverage rates capture results of "catch-up" immunization during stockout years, as confirmed by WUENIC historical estimate reports. Estimates not representative of coverage from national routine immunization schedule. BCG removed from analysis.
Kazakhstan	Kazakhstan Demographic and Health Survey 1999	1999	select	HepB3	Academy of Preventive Medicine (Kazakhstan), Macro International, Inc. Kazakhstan Demographic and Health Survey 1999. Fairfax, United States of America: ICF International.	Survey precedes introduction of HepB to national immunization schedule. HepB removed from analysis.
Kazakhstan	Kazakhstan Multiple Indicator Cluster Survey 2010-2011	2010-2011	all	HepB3	Agency of the Republic of Kazakhstan on Statistics, United Nations Children's Fund (UNICEF). Kazakhstan Multiple Indicator Cluster Survey 2010-2011. New York, United States of America: United Nations Children's Fund (UNICEF), 2013.	HepB3 estimates considered implausibly low and display unlikely cohort trend inconsistent with admin estimates. HepB3 removed from analysis.
Kenya	Kenya Welfare Monitoring Survey III 1997	1997	all	BCG, DPT1, DPT3, MCV1, Pol3	Central Bureau of Statistics (Kenya). Kenya Welfare Monitoring Survey III 1997. Nairobi, Kenya: Kenya National Bureau of Statistics.	Estimates considered implausible. Survey estimates are systematically high compared to admin estimates and estimates from other established surveys series (Kenya Demographic and Health Survey 1998). Survey removed from analysis.
Kenya	Kenya Integrated Household Budget Survey Tabular Data 2005-2006	2005-2006	all	Pol3	Kenya National Bureau of Statistics. Kenya Integrated Household Budget Survey Tabular Data 2005-2006. Nairobi, Kenya: Kenya National Bureau of Statistics.	Pol3 estimates are systematically high compared to admin estimates and estimates from other established surveys series (Kenya Demographic and Health Survey 2008-2009). Pol3 removed from analysis.
Kenya	Kenya Demographic and Health Survey 2014	2014	select	PCV3	ICF International, Kenya Medical Research Institute (KEMRI), Kenya National Bureau of Statistics, Ministry of Health (Kenya), National AIDS Control Council (Kenya), National Council for Population and Development (Kenya). Kenya Demographic and Health	Survey precedes introduction of PCV to national immunization schedule. PCV removed from analysis.

Country	Series	Year(s)	Cohort	Vaccine	Citation	Rationale for exclusion
					Survey 2014. Fairfax, United States of America: ICF International.	
Kenya	Kenya Multiple Indicator Cluster Survey 1996	1996	all	BCG, DPT1, DPT3, MCV1, Pol3	Kenya National Bureau of Statistics, United Nations Children's Fund (UNICEF). Kenya Multiple Indicator Cluster Survey 1996.	Estimates considered implausible. Survey estimates are systematically high compared to admin estimates and estimates from other established surveys series (Kenya Demographic and Health Survey 1998). Survey removed from analysis.
Kuwait	Kuwait Child Health Survey 1987	1987	all	BCG	Health Ministers Council for GCC States, Ministry of Health (Kuwait). Kuwait Child Health Survey 1987.	Survey precedes introduction of BCG to national immunization schedule. BCG removed from analysis.
Kuwait	Kuwait Family Health Survey 1996	1996	all	BCG	Health Ministers Council for GCC States, Ministry of Health (Kuwait), United Nations Statistics Division (UNSD). Kuwait Family Health Survey 1996.	Survey precedes introduction of BCG to national immunization schedule. BCG removed from analysis.
Kyrgyzstan	Kyrgyzstan Multiple Indicator Cluster Survey 2005-2006	2005-2006	all	DPT3, Pol3	United Nations Children's Fund (UNICEF), National Statistical Committee of the Kyrgyz Republic. Kyrgyzstan Multiple Indicator Cluster Survey 2005-2006. New York, United States: United Nations Children's Fund (UNICEF).	Estimates considered implausible. Survey estimates are systematically low compared to admin estimates and estimates from other established surveys series (Kyrgyzstan Demographic and Health Survey 2012). Survey removed from analysis.
Kyrgyzstan	Kyrgyzstan Demographic and Health Survey 2012	2012	select	Hib3	ICF International, Ministry of Health (Kyrgyzstan), National Statistical Committee of the Kyrgyz Republic. Kyrgyzstan Demographic and Health Survey 2012. Fairfax, United States of America: ICF International.	Older age-cohorts precede introduction of Hib3 to national immunization schedule. Select age-cohorts removed from analysis for Hib3.
Kyrgyzstan	Kyrgyzstan Multiple Indicator Cluster Survey 2014	2014	all	BCG, MCV1	National Statistical Committee of the Kyrgyz Republic, United Nations Children's Fund (UNICEF). Kyrgyzstan Multiple Indicator Cluster Survey 2014. New York, United States of America: United Nations Children's Fund (UNICEF), 2015.	BCG and MCV1 estimates are systematically low compared to admin estimates and estimates from other established surveys series (Kyrgyzstan Multiple Indicator Cluster Survey 2018). BCG and MCV1 removed from analysis.
Lesotho	Lesotho Demographic and Health Survey 2004-2005	2004-2005	select	HepB3	Bureau of Statistics (Lesotho), Macro International, Inc, Ministry of Health and Social Welfare (Lesotho). Lesotho Demographic and Health Survey 2004-2005. Fairfax, United States of America: ICF International.	Older age-cohorts precede introduction of HepB3 to national immunization schedule. Select age-cohorts removed from analysis for HepB3.
Lesotho	Lesotho Multiple Indicator Cluster Survey 2018	2018	select	RotaC	Bureau of Statistics (Lesotho), United Nations Children's Fund (UNICEF). Lesotho Multiple Indicator Cluster Survey 2018. New York, United States of America: United Nations Children's Fund (UNICEF), 2019.	Older age-cohorts precede introduction of rotavirus vaccine to national immunization schedule. Select age-cohorts removed from analysis for rotavirus vaccine.
Liberia	Liberia Malaria Indicator Survey 2016	2016	select	PCV3	ICF International, Liberia Institute for Statistics and Geo-information Services (LISGIS), National Malaria Control Program (Liberia). Liberia Malaria Indicator Survey 2016. Fairfax, United States of America: ICF International, 2017.	Older age-cohorts precede introduction of PCV3 to national immunization schedule. Select age-cohorts removed from analysis for PCV3.
Macedonia	Macedonia Multiple Indicator Cluster Survey 2011	2011	select	PCV3	Institute of Public Health (Macedonia), Ipsos Strategic Puls, Ministry of Education and Science (Macedonia), Ministry of Labor and Social Policy (Macedonia), United Nations Children's Fund (UNICEF). Macedonia Multiple Indicator Cluster Survey 2011. New York, United States of America: United Nations Children's Fund (UNICEF), 2013.	Older age-cohorts precede introduction of PCV3 to national immunization schedule. Select age-cohorts removed from analysis for PCV3.
Madagascar	Madagascar Multiple Indicator Cluster Survey 2000	2000	all	DPT1, DPT3	National Institute of Statistics (Madagascar), United Nations Children's Fund (UNICEF). Madagascar Multiple Indicator Cluster Survey 2000. New York, United States of America: United Nations Children's Fund (UNICEF).	Estimates considered implausible. Survey estimates are systematically low compared to admin estimates and estimates from other established surveys series (Madagascar Demographic and Health Survey 2003-2004). Survey removed from analysis.
Madagascar	Madagascar National Survey on Monitoring the Millennium Development Goals 2012-2013	2012-2013	all	BCG, DPT1, DPT3, HepB3,	African Development Bank (AfDB), National Institute of Statistics (Madagascar), UNICEF Madagascar, United Nations Population Fund (UNFPA), United Nations Women's Fund (UNIFEM), World Food Programme	Estimates considered implausible. Survey estimates are systematically low compared to admin estimates and estimates from other established surveys series (Madagascar Demographic and Health Survey 2008,

Country	Series	Year(s)	Cohort	Vaccine	Citation	Rationale for exclusion
				PCV3, MCV1, Pol3	(WFP). Madagascar National Survey on Monitoring the Millennium Development Goals 2012-2013.	Madagascar Multiple Indicator Cluster Survey 2018). Survey removed from analysis.
Malawi	Malawi Demographic and Health Survey 2004-2005	2004-2005	select	HepB3, Hib3	Macro International, Inc, National Statistical Office of Malawi. Malawi Demographic and Health Survey 2004-2005. Fairfax, United States of America: ICF International.	Older age-cohorts precede introduction of HepB3 and Hib3 to national immunization schedule. Select age-cohorts removed from analysis for HepB3 and Hib3.
Malawi	Malawi Demographic and Health Survey 2015-2016	2015-2016	all	MCV2	Emory University and Centers for Disease Control & Prevention Collaboration, ICF International, Ministry of Health (Malawi), National Statistical Office of Malawi. Malawi Demographic and Health Survey 2015-2016. Fairfax, United States of America: ICF International, 2017.	Older age-cohorts precede introduction of MCV2 to national immunization schedule. Select age-cohorts removed from analysis for MCV2.
Mali	Mali Demographic and Health Survey 2006	2006	all	HepB3	Macro International, Inc, Ministry of Health (Mali), National Directorate of Statistics and Informatics (DNSI) (Mali). Mali Demographic and Health Survey 2006. Fairfax, United States of America: ICF International.	HepB3 estimates are systematically low compared to admin estimates and estimates from other established surveys series. HepB3 removed from analysis.
Mali	Mali Demographic and Health Survey 2012-2013	2012-2013	all	HepB3, Hib3, Pol3	ICF International, INFO-STAT (Mali), Ministry of Health (Mali), National Institute of Statistics (INSTAT) (Mali), Planning and Statistics Unit, Ministry of Health (Mali). Mali Demographic and Health Survey 2012-2013. Fairfax, United States of America: ICF International, 2014.	HepB3, hib3, and Pol3 estimates are systematically low compared to admin estimates and estimates from other established surveys series. HepB3, hib3, and Pol3 removed from analysis.
Mali	Mali Multiple Indicator Cluster Survey 2015	2015	select	RCV1, RotaC	Ministry of Health (Mali), Ministry of Planning (Mali), National Institute of Statistics (INSTAT) (Mali), United Nations Children's Fund (UNICEF). Mali Multiple Indicator Cluster Survey 2015. New York, United States of America: United Nations Children's Fund (UNICEF), 2017.	Survey precedes introduction of RCV1 and some age-cohorts of rotavirus vaccine to national immunization schedule. RCV1 and select age-cohorts of rotavirus vaccines removed from analysis.
Mali	Mali Multiple Indicator Cluster Survey 2009-2010	2009-2010	all	BCG, DPT1, DPT3, HepB3, MCV1, Pol3	Ministry of Health (Mali), National Institute of Statistics (INSTAT) (Mali), United Nations Children's Fund (UNICEF). Mali Multiple Indicator Cluster Survey 2009-2010. New York, United States of America: United Nations Children's Fund (UNICEF), 2017.	Estimates considered implausible. Survey estimates are systematically high compared to admin estimates and estimates from other established surveys series. Survey removed from analysis.
Marshall Islands	Marshall Islands Demographic and Health Survey 2007	2007	all	BCG, DPT1, DPT3, MCV1, Pol3	Economic Policy, Planning and Statistics Office (Marshall Islands), Secretariat of the Pacific Community (SPC). Marshall Islands Demographic and Health Survey 2007.	Estimates considered implausible. Survey estimates are systematically low compared to admin estimates and estimates from other established surveys series. Survey removed from analysis.
Mauritania	Mauritania Multiple Indicator Cluster Survey 2011	2011	select	Hib3	National Office of Statistics (Mauritania), United Nations Children's Fund (UNICEF). Mauritania Multiple Indicator Cluster Survey 2011. New York, United States of America: United Nations Children's Fund (UNICEF), 2015.	Older age-cohorts precede introduction of Hib3 to national immunization schedule. Select age-cohorts removed from analysis for Hib3.
Mexico	Mexico Family Life Survey 2002	2002	all	DPT3	Center for Research and Teaching in Economics (CIDE) (Mexico), Ibero-American University, National Institute of Perinatology (Mexico), National Institute of Statistics and Geography (INEGI) (Mexico). Mexico Family Life Survey 2002.	Estimates considered implausible. Survey estimates are systematically low compared to admin estimates and estimates from other established surveys series. Survey removed from analysis.
Mexico	Mexico National Health Survey 1999-2000	1999-2000	all	DPT1, DPT3, PCV3, MCV1, Pol3	National Institute of Public Health (Mexico). Mexico National Health Survey 1999-2000.	Estimates considered implausible. Survey estimates are systematically high compared to admin estimates and estimates from other established surveys series. Survey removed from analysis.

Country	Series	Year(s)	Cohort	Vaccine	Citation	Rationale for exclusion
Mexico	Mexico National Performance Evaluation Survey 2002-2003	2002-2003	all	DPT3	National Institute of Public Health (Mexico), Secretariat of Health (Mexico), World Health Organization (WHO). Mexico National Performance Evaluation Survey 2002-2003.	Estimates considered implausible. Survey estimates are systematically low compared to admin estimates and estimates from other established surveys series. Survey removed from analysis.
Mexico	Mexico National Survey of Demographic Dynamics 2006	2006	all	DPT1, DPT3, PCV3, Pol3	National Institute of Statistics, Geography, and Informatics (Mexico), National Institute for Public Health (Mexico), National Population Council (Mexico). Mexico National Survey of Demographic Dynamics 2006. Aguascalientes, Mexico: National Institute of Statistics, Geography, and Informatics (Mexico).	Estimates considered implausible. Survey estimates are systematically low compared to admin estimates and estimates from other established surveys series. Survey removed from analysis.
Mexico	Mexico Family Life Survey 2005-2006	2005-2006	all	BCG, DPT1, DPT3, PCV3, MCV1, Pol3, RCV1	California Center for Population Research (CCPR), University of California Los Angeles (UCLA), Center for Research and Teaching in Economics (CIDE) (Mexico), Ibero-American University, National Institute of Public Health (Mexico). Mexico Family Life Survey 2005-2006.	Estimates considered implausible. Survey estimates are inconsistent with admin estimates and estimates from other established survey series. Survey removed from analysis.
Mexico	Mexico National Survey of Health and Nutrition 2011-2012	2011-2012	select	PCV3	National Institute of Public Health (Mexico). Mexico National Survey of Health and Nutrition 2011-2012. Cuernavaca, Mexico: National Institute of Public Health (Mexico).	Older age-cohorts precede introduction of PCV3 to national immunization schedule. Select age-cohorts removed from analysis for PCV3.
Mexico	Mexico Family Life Survey 2008-2013	2008-2013	all	BCG, DPT1, DPT3, HepB3, PCV3, MCV1, PCV3, Pol3, RCV1	Center for Research and Teaching in Economics (CIDE) (Mexico), Duke University, Ibero-American University, National Institute of Public Health (Mexico), University of California, Los Angeles (UCLA). Mexico Family Life Survey 2008-2013.	Estimates considered implausible. Survey estimates are inconsistent with admin estimates and estimates from other established survey series. Survey removed from analysis.
Morocco	Morocco National Survey on Population and Family Health 2010-2011	2010-2011	select	DPT1	Ministry of Health (Morocco), Pan Arab Project for Family Health (PAPFAM), United Nations Children's Fund (UNICEF), United Nations Population Fund (UNFPA), World Health Organization (WHO). Morocco National Survey on Population and Family Health 2010-2011.	Survey reports DPT3 coverage greater than DPT1 coverage for some age-cohorts. Select age-cohorts removed for DPT1 due to reliability concerns.
Mozambique	Mozambique Demographic and Health Survey 2011	2011	select	Hib3	ICF Macro, Manhica Health Research Center (CISM), Ministry of Health (Mozambique), National Institute of Statistics (INE) (Mozambique). Mozambique Demographic and Health Survey 2011. Fairfax, United States of America: ICF International.	Older age-cohorts precede introduction of Hib3 to national immunization schedule. Select age-cohorts removed from analysis for Hib3.
Mozambique	Mozambique AIDS Indicator Survey 2015	2015	select	PCV3	Centers for Disease Control and Prevention (CDC), ICF International, Ministry of Health (Mozambique), National Institute of Health (Mozambique), National Institute of Statistics (INE) (Mozambique). Mozambique AIDS Indicator Survey 2015. Fairfax, United States of America: ICF International, 2018.	Older age-cohorts precede introduction of PCV3 to national immunization schedule. Select age-cohorts removed from analysis for PCV3.
Namibia	Namibia Demographic and Health Survey 1992	1992	select	BCG, DPT1, DPT3, MCV1, Pol3	Central Statistics Office (Namibia), Macro International, Inc, Ministry of Health and Social Services (Namibia). Namibia Demographic and Health Survey 1992. Fairfax, United States of America: ICF International.	Older age-cohorts precede introduction of BCG, DPT1, DPT3, MCV1, and Pol3 to national immunization schedule. Select age-cohorts removed from analysis for BCG, DPT1, DPT3, MCV1, and Pol3.
Nepal	Nepal Demographic and Health Survey 2006	2006	select	HepB3	Macro International, Inc, Ministry of Health and Population (Nepal), New ERA. Nepal Demographic and	Older age-cohorts precede introduction of HepB3 to national immunization schedule. Select age-cohorts removed from analysis for HepB3.

Country	Series	Year(s)	Cohort	Vaccine	Citation	Rationale for exclusion
					Health Survey 2006. Fairfax, United States of America: ICF International.	
Nepal	Nepal Demographic and Health Survey 2011	2011	select	PCV3	ICF Macro, Ministry of Health and Population (Nepal), New ERA. Nepal Demographic and Health Survey 2011. Fairfax, United States of America: ICF International.	Older age-cohorts precede introduction of Hib3 to national immunization schedule. Select age-cohorts removed from analysis for HepB3.
Nepal	Nepal Living Standards Measurement Survey 2003-2004	2003-2004	all	BCG, DPT1, DPT3, MCV1, Pol3	Central Bureau of Statistics (Nepal), World Bank. Nepal Living Standards Measurement Survey 2003-2004. Kathmandu, Nepal: Central Bureau of Statistics (Nepal).	Estimates considered implausible. Survey estimates are systematically high compared to admin estimates and estimates from other established surveys series (Nepal Demographic and Health Survey 2006). Survey removed from analysis.
Nepal	Nepal Demographic and Health Survey 2016-2017	2016-2017	select	PCV3	ICF International, Ministry of Health (Nepal), New ERA. Nepal Demographic and Health Survey 2016-2017. Fairfax, United States of America: ICF International, 2017.	Survey precedes introduction of PCV3 to national immunization schedule. PCV3 removed from analysis.
Nicaragua	Nicaragua Living Standards Measurement Survey 1993	1993	all	MCV2	National Institute of Statistics and Censuses (Nicaragua), World Bank. Nicaragua Living Standards Measurement Survey 1993.	Survey precedes introduction of second measles dose to national immunization schedule. MCV2 removed from analysis.
Nicaragua	Nicaragua Living Standards Measurement Survey 1998-1999	1998-1999	all	MCV2	National Institute of Statistics and Censuses (Nicaragua), World Bank. Nicaragua Living Standards Measurement Survey 1998-1999.	Survey precedes introduction of second measles dose to national immunization schedule. MCV2 removed from analysis.
Nicaragua	Nicaragua Living Standards Measurement Survey 2001	2001	select	DPT1, DPT3, RCV1	National Institute of Statistics and Censuses (Nicaragua), World Bank. Nicaragua Living Standards Measurement Survey 2001.	Survey precedes introduction of RCV1 to national immunization schedule and reports DPT3 coverage greater than DPT1 coverage for some age-cohorts. RCV1 and select age-cohorts of DPT1 removed from analysis.
Nigeria	Nigeria Demographic and Health Survey 2013	2013	select	Hib3	ICF International, National Population Commission of Nigeria. Nigeria Demographic and Health Survey 2013. Fairfax, United States of America: ICF International.	Older age-cohorts precede introduction of Hib3 to national immunization schedule. Select age-cohorts removed from analysis for Hib3.
Nigeria	Nigeria Living Standards Survey 2008-2010	2008-2010	select	DPT1, MCV1	National Bureau of Statistics (Nigeria). Nigeria Living Standards Survey 2008-2010. Abuja, Nigeria: National Bureau of Statistics (Nigeria).	DPT1 and MCV1 estimates are systematically high compared to admin estimates and estimates from other established surveys series (Nigeria Multiple Indicator Cluster Survey 2011). DPT1 and MCV1 removed from analysis.
Nigeria	Nigeria General Household Survey 2012-2013	2012-2013	all	DPT3	National Bureau of Statistics (Nigeria). Nigeria General Household Survey 2012-2013. Washington DC, United States of America: World Bank.	DPT3 estimates are systematically high compared to admin estimates and estimates from other established surveys series (Nigeria Demographic and Health Survey 2013). DPT3 removed from analysis.
Nigeria	Nigeria General Household Survey 2010-2011	2010-2011	all	BCG, DPT1, DPT3, MCV1, Pol3	National Bureau of Statistics (Nigeria). Nigeria General Household Survey 2010-2011. Abuja, Nigeria: National Bureau of Statistics (Nigeria).	Survey estimates are systematically high compared to admin estimates and estimates from other established survey series (Nigeria Demographic and Health Survey 2013). Survey removed from analysis.
Nigeria	Nigeria Annual Report and Statement of Accounts 2002	1991-2002	all	BCG, DPT3, MCV1, Pol3	Central Bank of Nigeria. Nigeria Annual Report and Statement of Accounts 2002. Abuja, Nigeria: Central Bank of Nigeria, 2003.	Estimates considered implausible. Survey estimates are systematically high compared to admin estimates and estimates from other established surveys series (Nigeria Multiple Indicator Cluster Survey 1999). Survey removed from analysis.
Nigeria	Nigeria Annual Report and Statement of Accounts 2001	1991-2001	all	BCG, DPT3, MCV1, Pol3	Central Bank of Nigeria. Nigeria Annual Report and Statement of Accounts 2001. Abuja, Nigeria: Central Bank of Nigeria, 2002.	Estimates considered implausible. Survey estimates are systematically high compared to admin estimates and estimates from other established surveys series (Nigeria Multiple Indicator Cluster Survey 1999). Survey removed from analysis.
Nigeria	Nigeria Demographic and Health Survey 2018	2018	select	MCV2	Federal Ministry of Health (Nigeria), ICF International, National Population Commission (NPC). Nigeria Demographic and Health Survey 2018. Fairfax, United States of America: ICF International, 2020.	Survey precedes introduction of MCV2 to national immunization schedule. MCV2 removed from analysis.

Country	Series	Year(s)	Cohort	Vaccine	Citation	Rationale for exclusion
North Korea	North Korea EPI Coverage Evaluation Survey 2008	2008	all	Hib3	Ministry of Public Health (North Korea), United Nations Children's Fund (UNICEF), World Health Organization (WHO). North Korea EPI Coverage Evaluation Survey 2008.	Survey precedes introduction of PCV3 to national immunization schedule. PCV3 removed from analysis.
Pakistan	Pakistan Social and Living Standards Measurement Survey 2014-2015	2014-2015	all	BCG, DPT1, DPT3, HepB3, MCV1, MCV2, Pol3	Pakistan Bureau of Statistics. Pakistan Social and Living Standards Measurement Survey 2014-2015. Islamabad, Pakistan: Pakistan Bureau of Statistics, 2016.	Estimates considered implausible. Survey estimates are inconsistent with admin estimates and estimates from other established survey series (Pakistan Demographic and Health Survey 2017-2018). Survey removed from analysis.
Pakistan	Pakistan Demographic and Health Survey 2017-2018	2017-2018	all	RCV1	ICF International, Ministry of National Health Services, Regulations & Coordination (Pakistan), National Institute of Population Studies (Pakistan). Pakistan Demographic and Health Survey 2017-2018. Fairfax, United States of America: ICF International, 2018.	Survey precedes introduction of RCV1 to national immunization schedule. RCV1 removed from analysis.
Palestine	Palestine Family Health Survey 2006-2007	2006-2007	select	DPT3, RCV1	League of Arab States, Palestinian Central Bureau of Statistics, United Nations Children's Fund (UNICEF). Palestine Family Health Survey 2006-2007.	DPT3 estimates are systematically low compared to admin estimates and estimates from other established surveys series (Palestine Multiple Indicator Cluster Survey 2010). Older age cohorts precede introduction of RCV1 to national immunization schedule. DPT3 and select age-cohorts of RCV1 removed from analysis.
Palestine	Palestine - West Bank and Gaza Strip Multiple Indicator Cluster Survey 1996 - UNICEF	1996	all	BCG, RCV1	Palestine - West Bank and Gaza Strip Health Survey 1996	Estimates considered implausible. Survey estimates are systematically low compared to admin estimates and estimates from other established surveys series (West Bank and Gaza Strip Multiple Indicator Cluster Survey 2000). Survey removed from analysis.
Panama	Panama Living Standard Measurement Survey 2008	2008	select	HepB3, RotaC	Census and Statistics Directorate (Panama), Ministry of Economy and Finance (Panama), World Bank. Panama Living Standard Measurement Survey 2008. Washington DC, United States of America: World Bank.	HepB3 estimates include implausibly low coverage rates of 0%. Older age-cohorts precede introduction of rotavirus vaccine to national immunization schedule. HepB and select age-cohorts of RotaC removed from analysis.
Papua New Guinea	Papua New Guinea Demographic and Health Survey 2016-2018	2016-2018	all	MCV2	ICF International, National Statistical Office (Papua New Guinea). Papua New Guinea Demographic and Health Survey 2016-2018. Fairfax, United States of America: ICF International, 2019.	Survey precedes introduction of MCV2 to national immunization schedule. MCV2 removed from analysis.
Paraguay	Paraguay Reproductive Health Survey 2004	2004	select	HepB3, Hib3	Division of Reproductive Health-Centers for Disease Control and Prevention (CDC). (2005): Paraguay Reproductive Health Survey 2004. Asuncion, Paraguay, Paraguayan Center for Population Studies (CEPEP).	Older age-cohorts precede introduction of HepB3 and Hib3 to national immunization schedule. Select age-cohorts removed from analysis for HepB3 and Hib3.
Peru	Peru Demographic and Health Survey 2000	2000	select	HepB3, Hib3	Macro International, Inc, National Institute of Statistics (Peru). Peru Demographic and Health Survey 2000. Fairfax, United States of America: ICF International.	HepB3 and older age-cohorts of Hib3 precede introduction to national immunization schedule. HepB3 and select age-cohorts of Hib3 removed from analysis.
Peru	Peru Continuous Demographic and Health Survey 2009	2009	select	MCV2, PCV3, RCV1, RotaC	National Institute of Statistics and Informatics (Peru), ORC Macro. Peru Continuous Demographic and Health Survey 2009. Fairfax, United States of America: ICF International.	PCV3, RCV1, RotaC and older age-cohorts of MCV2 precede introduction to national immunization schedule. PCV3, RCV1, RotaC and select age-cohorts of MCV2 removed from analysis.
Peru	Peru Continuous Demographic and Health Survey 2010	2010	select	MCV2, PCV3, RCV1, RotaC	National Institute of Statistics and Informatics (Peru). Peru Continuous Demographic and Health Survey 2010. Fairfax, United States of America: ICF International.	MCV2, RCV1 and older age-cohorts of RotaC and PCV3 precede introduction to national immunization schedule. MCV2, RCV1 and select age-cohorts of RotaC and PCV3 removed from analysis.
Peru	Peru Continuous Demographic and Health Survey 2011	2011	select	MCV2, PCV3, RCV1	Macro International, Inc, National Institute of Statistics and Informatics (Peru). Peru Continuous Demographic and Health Survey 2011. Fairfax, United States of America: ICF International.	MCV2, RCV1 and older age-cohorts of PCV3 precede introduction to national immunization schedule. MCV2, RCV1 and select age-cohorts of PCV3 removed from analysis.

Country	Series	Year(s)	Cohort	Vaccine	Citation	Rationale for exclusion
Peru	Peru Continuous Demographic and Health Survey 2012	2012	select	MCV2, PCV3, RCV1	Macro International, Inc, National Institute of Statistics and Informatics (Peru). Peru Continuous Demographic and Health Survey 2012. Fairfax, United States of America: ICF International.	MCV2, RCV1 and older age-cohorts of PCV3 precede introduction to national immunization schedule. MCV2, RCV1 and select age-cohorts of PCV3 removed from analysis.
Peru	Peru Continuous Demographic and Health Survey 2003-2008	2003-2008	select	HepB3, MCV1, MCV2, PCV3, RCV1	Ministry of Economy and Finance (Peru), National Institute of Statistics and Informatics (Peru), ORC Macro. Peru Continuous Demographic and Health Survey 2003-2008. Fairfax, United States of America: ICF International.	Required survey indicators for RCV1 and MCV1 missing from survey data. Survey precedes introduction of MCV2 and rotavirus vaccine to national immunization schedule. RCV1, MCV1, MCV2, and RotaC removed from analysis.
Philippines	Philippines Demographic and Health Survey 2008	2008	all	Hib3	Macro International, Inc, National Statistics Office (Philippines). Philippines Demographic and Health Survey 2008. Fairfax, United States of America: ICF International, 2010.	Survey precedes introduction of Hib3 to national immunization schedule. Hib3 removed from analysis.
Philippines	Philippines Demographic and Health Survey 2013	2013	select	HepB3, PCV3, RCV1, RotaC	ICF International, Philippines Statistics Authority. Philippines Demographic and Health Survey 2013. Fairfax, United States of America: ICF International, 2014.	HepB3 estimates are implausibly low compared to admin estimates and estimates from other established survey series (Philippines Multiple Indicator Survey 2016). Older age-cohorts precede introduction of rotavirus vaccine and RCV1 to national immunization schedule. HepB3 and select age-cohorts of RotaC and RCV1 removed from analysis.
Philippines	Philippines Multiple Indicator Survey 2016	2016	all	MCV2	Demographic Research and Development Foundation Inc. (Philippines), National Statistics Office (Philippines), United Nations Children's Fund (UNICEF). Philippines Multiple Indicator Survey 2016.	MCV2 estimates include implausibly low coverage rates of 0%. MCV2 removed from analysis.
Philippines	Philippines Demographic and Health Survey 2017	2017	select	RCV1	ICF International, Philippines Statistics Authority, United States Agency for International Development (USAID). Philippines Demographic and Health Survey 2017. Fairfax, United States of America: ICF International, 2018.	Survey precedes introduction of RCV1 to national immunization schedule. RCV1 removed from analysis.
Qatar	Qatar Multiple Indicator Cluster Survey 2012	2012	all	BCG, DPT1, DPT3, HepB3, PCV3, MCV1, MCV2, Pol3, RCV1	Ministry of Development Planning and Statistics (Qatar), Qatar Foundation for Education, Science and Community Development, Qatar Statistics Authority, Supreme Council of Health (Qatar), United Nations Children's Fund (UNICEF). Qatar Multiple Indicator Cluster Survey 2012. New York, United States of America: United Nations Children's Fund (UNICEF), 2018.	Estimates considered implausible. Survey estimates are systematically low compared to admin estimates and estimates from other established surveys series. Survey removed from analysis.
Rwanda	Rwanda Demographic and Health Survey 2010-2011	2010-2011	select	HepB3, Hib3, PCV3	ICF Macro, Ministry of Health (Rwanda), National Institute of Statistics of Rwanda. Rwanda Demographic and Health Survey 2010-2011. Fairfax, United States of America: ICF International.	HepB3, Hib3, and PCV3 estimates do not follow a reasonable age structure. Older children have significantly higher coverage than reported in either administrative data or other survey series, suggesting survey is capturing results of "catch-up" immunization. Select age-cohorts removed from analysis for HepB3, Hib3, and PCV3.
Rwanda	Rwanda Demographic and Health Survey 2014-2015	2014-2015	select	RCV1, RotaC	ICF International, Ministry of Health (Rwanda), National Institute of Statistics of Rwanda. Rwanda Demographic and Health Survey 2014-2015. Fairfax, United States of America: ICF International, 2016.	RCV1 and older age-cohorts of rotavirus vaccine precede introduction to national immunization schedule. RCV1 and select age-cohorts of rotavirus vaccine removed from analysis.
São Tomé and Príncipe	Sao Tome and Principe Multiple Indicator Cluster Survey 2000	2000	select	BCG, DPT1, DPT3, MCV1, Pol3	National Institute of Statistics (Sao Tome and Principe), United Nations Children's Fund (UNICEF). Sao Tome and Principe Multiple Indicator Cluster Survey 2000. New York, United States of America: United Nations Children's Fund (UNICEF).	Estimates considered implausible. Survey estimates do not follow a reasonable age structure. Older children have significantly higher coverage than reported in either administrative data or other survey series, suggesting survey is capturing results of "catch-up" immunization. Select age-cohorts removed from analysis.

Country	Series	Year(s)	Cohort	Vaccine	Citation	Rationale for exclusion
Senegal	Senegal Continuous Demographic and Health Survey 2016	2016	select	MCV2	ICF International, Ministry of Health and Social Action (Senegal), National Agency of Statistics and Demography (Senegal). Senegal Continuous Demographic and Health Survey 2016. Fairfax, United States of America: ICF International, 2017.	Older age-cohorts precede introduction of MCV2 to national immunization schedule. Select age-cohorts removed from analysis for MCV2.
Sierra Leone	Sierra Leone Multiple Indicator Cluster Survey 2005	2005	all	RCV1	United Nations Children's Fund (UNICEF), Statistics Sierra Leone. Sierra Leone Multiple Indicator Cluster Survey 2005. New York, United States: United Nations Children's Fund (UNICEF).	Survey precedes introduction of RCV1 to national immunization schedule. RCV1 removed from analysis.
Sierra Leone	Sierra Leone Demographic and Health Survey 2008	2008	select	HepB3, Hib3	Macro International, Inc, Statistics Sierra Leone. Sierra Leone Demographic and Health Survey 2008. Fairfax, United States of America: ICF International.	Older age-cohorts precede introduction of HepB3 and Hib3 to national immunization schedule. Select age-cohorts removed from analysis for HepB3 and Hib3.
Sierra Leone	Sierra Leone Multiple Indicator Cluster Survey 2010	2010	select	HepB3, MCV1	Statistics Sierra Leone, United Nations Children's Fund (UNICEF). Sierra Leone Multiple Indicator Cluster Survey 2010. New York, United States of America: United Nations Children's Fund (UNICEF).	Older age-cohorts precede introduction of HepB3 to national immunization schedule. MCV1 estimates from survey microdata don't match coverage from survey report. MCV1 and select age-cohorts of HepB3 removed from analysis.
Sierra Leone	Sierra Leone Demographic and Health Survey 2013	2013	select	PCV3	ICF International, Ministry of Health and Sanitation (Sierra Leone), Statistics Sierra Leone. Sierra Leone Demographic and Health Survey 2013. Fairfax, United States of America: ICF International, 2014.	Older age-cohorts precede introduction of PCV3 to national immunization schedule. Select age-cohorts removed from analysis for PCV3.
Sierra Leone	Sierra Leone National EPI Coverage Evaluation Survey 1993	1993	all	BCG, DPT3, MCV1, Pol3	Sierra Leone National EPI Coverage Evaluation Survey 1993.	Estimates considered implausible. Survey estimates are systematically high compared to admin estimates and estimates from other established surveys series (Sierra Leone Multiple Indicator Survey 2000). Survey removed from analysis.
Sierra Leone	Sierra Leone National EPI Coverage Evaluation Survey 1991	1991	all	BCG, DPT3, MCV1, Pol3	Sierra Leone National EPI Coverage Evaluation Survey 1991.	Estimates considered implausible. Survey estimates are systematically high compared to admin estimates and estimates from other established surveys series (Sierra Leone Multiple Indicator Survey 2000). Survey removed from analysis.
Somalia	Somalia Family Health Survey 1983	1983	all	DPT1	Ministry of Health (Somalia), Ministry of National Planning (Somalia), Westinghouse Health Systems, Inc. Somalia Family Health Survey 1983.	Survey reports higher DPT3 coverage than DPT1 coverage. DPT1 removed from analysis due to reliability concerns.
South Africa	South Africa Demographic and Health Survey 1998	1998	select	HepB3	Department of Health (South Africa), Macro International, Inc, South African Medical Research Council. South Africa Demographic and Health Survey 1998. Fairfax, United States of America: ICF International.	Older age-cohorts precede introduction of HepB3 to national immunization schedule. Select age-cohorts removed from analysis for HepB3.
South Africa	South Africa World Health Survey 2002-2003	2002-2003	all	DPT1, DPT3, MCV1	World Health Organization (WHO). South Africa World Health Survey 2002-2003. Geneva, Switzerland: World Health Organization (WHO), 2005.	Estimates considered implausible. Survey estimates are inconsistent with admin estimates and estimates from other established survey series (South Africa Demographic and Health Survey 2003-2004). Survey removed from analysis.
Sri Lanka	Sri Lanka Demographic and Health Survey 2006-2007	2006-2007	select	HepB3	Department of Census and Statistics (Sri Lanka), Macro International, Inc. Sri Lanka Demographic and Health Survey 2006-2007. 2009.	HepB3 estimates do not follow a reasonable age structure. Older children have significantly higher coverage than reported in either administrative data or other survey series, suggesting survey is capturing results of "catch-up" immunization. Select age cohorts removed from analysis for HepB3.
Sri Lanka	Sri Lanka Demographic and Health Survey 1987	1987	select	MCV1	Department of Census and Statistics (Sri Lanka), Westinghouse; Institute for Resource Development. Sri Lanka Demographic and Health Survey 1987. Fairfax, United States of America: ICF International.	Older age-cohorts precede introduction of MCV1 to national immunization schedule. Select age-cohorts removed from analysis for MCV1.

Country	Series	Year(s)	Cohort	Vaccine	Citation	Rationale for exclusion
Sri Lanka	Sri Lanka Demographic and Health Survey 1993	1993	all	DPT1	Department of Census and Statistics (Sri Lanka). Sri Lanka Demographic and Health Survey 1993. 2009.	Survey reports higher DPT3 coverage than DPT1 coverage. DPT1 removed from analysis due to reliability concerns.
Sudan	Sudan Maternal and Child Health Survey 1992-1993	1992-1993	all	BCG, DPT3, MCV1, Pol3	Federal Ministry of Health (Sudan), League of Arab States. Sudan Maternal and Child Health Survey 1992-1993.	Estimates considered implausible. Survey estimates are systematically high compared to admin estimates and estimates from other established surveys series (Sudan Multiple Indicator Survey 2000). Survey removed from analysis.
Sudan	Sudan Demographic and Health Survey 1989-1990	1989-1990	all	BCG, DPT1, DPT3, MCV1, Pol3	Ministry of Finance and Economic Planning, Department of Statistics, Macro Systems, Inc.; Institute for Resource Development. Sudan Demographic and Health Survey 1989-1990. Columbia, United States: Macro Systems, Inc.	Estimates considered implausible. Survey estimates are systematically high compared to admin estimates and estimates from other established surveys series. Survey removed from analysis.
Sudan	Sudan Family Health Survey 2006	2006	all	BCG, DPT1, DPT3, MCV1, Pol3	Ministry of Health (Southern Sudan), Federal Ministry of Health (Sudan), Southern Sudan Centre for Census, Statistics and Evaluation (SSCCSE), Central Bureau of Statistics (Sudan). Sudan Family Health Survey 2006.	Estimates considered implausible. Survey estimates are systematically low compared to admin estimates and estimates from other established surveys series (Sudan Multiple Indicator Cluster Survey 2010). Survey removed from analysis.
Sudan	Sudan Multiple Indicator Cluster Survey 2014	2014	all	MCV2	Central Bureau of Statistics (Sudan), Federal Ministry of Health (Sudan), United Nations Children's Fund (UNICEF). Sudan Multiple Indicator Cluster Survey 2014. New York, United States of America: United Nations Children's Fund (UNICEF), 2016.	Survey reports implausibly high MCV2 coverage in introduction year. Select age-cohorts removed from analysis for MCV2.
Syria	Syria Family Health Survey 2001	2001	select	RCV1	Central Bureau of Statistics (Syria), League of Arab States. Syria Family Health Survey 2001.	Older age-cohorts precede introduction of RCV1 to national immunization schedule. Select age-cohorts removed from analysis for RCV1.
Syria	Syria Multiple Indicator Cluster Survey 1995	1995	all	Hib3	United Nations Children's Fund (UNICEF). Syria Multiple Indicator Cluster Survey 1995.	Survey precedes introduction of Hib3 to national immunization schedule. Hib3 removed from analysis.
Tanzania	Tanzania Demographic and Health Survey 2004-2005	2004-2005	all	HepB3, PCV3	Macro International, Inc, National Bureau of Statistics (Tanzania). Tanzania Demographic and Health Survey 2004-2005. Fairfax, United States of America: ICF International.	Survey precedes introduction of HepB and PCV in national immunization schedule. HepB and PCV removed from analysis.
Tanzania	Tanzania Demographic and Health Survey 2009-2010	2009-2010	all	HepB3, Hib3	ICF Macro, National Bureau of Statistics (Tanzania). Tanzania Demographic and Health Survey 2009-2010. Fairfax, United States of America: ICF International.	Estimates considered implausible. Survey estimates are systematically low compared to admin estimates and estimates from other established surveys series. Survey removed from analysis.
Tanzania	Tanzania Demographic and Health Survey 2015-2016	2015-2016	all	MCV2	ICF International, Ministry of Health (Zanzibar), Ministry of Health, Community Development, Gender, Elderly and Children (MoHCDEC) (Tanzania), National Bureau of Statistics (Tanzania), Office of the Chief Government Statistician (OCGS) (Zanzibar). Tanzania Demographic and Health Survey 2015-2016. Fairfax, United States of America: ICF International, 2016.	Older age-cohorts precede introduction of MCV2 to national immunization schedule. Select age-cohorts removed from analysis for MCV2.
Thailand	Thailand Demographic and Health Survey 1987	1987	select	MCV1	Chulalongkorn University, Institute of Population Studies (Thailand), Westinghouse; Institute for Resource Development. Thailand Demographic and Health Survey 1987. Fairfax, United States: ICF International.	Older age-cohorts precede introduction of MCV1 to national immunization schedule. Select age-cohorts removed from analysis for MCV1.
Thailand	Thailand Multiple Indicator Cluster Survey 2012	2012	select	HepB3	College of Population Studies, Chulalongkorn University (Thailand), Institute for Population and Social Research, Mahidol University (Thailand), International Health Policy Program (Thailand), Ministry of Education (Thailand), Ministry of Public Health (Thailand), Ministry of Social Development and Human Security (MSDHS) (Thailand), National Health Security Office (Thailand), National Statistical Office (Thailand), Thai Health	Older age-cohorts for HepB3 are implausibly low compared to admin estimates and estimates from other established surveys series (Thailand Multiple Indicator Cluster Survey 2012). Select age-cohorts removed from analysis for HepB3.

Country	Series	Year(s)	Cohort	Vaccine	Citation	Rationale for exclusion
					Promotion Foundation, United Nations Children's Fund (UNICEF). Thailand Multiple Indicator Cluster Survey 2012. New York, United States of America: United Nations Children's Fund (UNICEF), 2016.	
Timor-Leste	Timor-Leste Demographic and Health Survey 2003	2003	all	HepB3	ACIL Australia Pty Ltd., Australian National University, Ministry of Health (Timor-Leste), National Statistics Directorate (Timor-Leste), University of Newcastle (Australia). Timor-Leste Demographic and Health Survey 2003. Newcastle, Australia: University of Newcastle (Australia).	Survey precedes introduction of HepB in national immunization schedule. HepB removed from analysis.
Timor-Leste	Timor-Leste Demographic and Health Survey 2009-2010	2009-2010	select	HepB3	ICF Macro, Ministry of Finance (Timor-Leste), National Statistics Directorate (Timor-Leste). Timor-Leste Demographic and Health Survey 2009-2010. Fairfax, United States of America: ICF International.	Older age-cohorts precede introduction of HepB3 to national immunization schedule. Select age-cohorts removed from analysis for HepB3.
Togo	Togo Multiple Indicator Cluster Survey 2010	2010	select	HepB3, Hib3	Directorate General of Statistics and National Accounting (Togo), United Nations Children's Fund (UNICEF). Togo Multiple Indicator Cluster Survey 2010. New York, United States: United Nations Children's Fund (UNICEF).	Older age-cohorts precede introduction of HepB3 and Hib3 to national immunization schedule. Select age-cohorts removed from analysis for HepB3 and Hib3.
Togo	Togo Multiple Indicator Cluster Survey 2017	2017	all	RCV1	Directorate General of Statistics and National Accounting (Togo), United Nations Children's Fund (UNICEF). Togo Multiple Indicator Cluster Survey 2010. New York, United States: United Nations Children's Fund (UNICEF).	Survey precedes introduction of RCV1 to national immunization schedule. RCV1 removed from analysis.
Tonga	Tonga Demographic and Health Survey 2012	2012	all	BCG, DPT1, DPT3, MCV1, Pol3	Macro International, Inc, Ministry of Health (Tonga), Secretariat of the Pacific Community (SPC), Tonga Department of Statistics. Tonga Demographic and Health Survey 2012.	Estimates considered implausible. Survey estimates are systematically low compared to admin estimates and estimates from other established surveys series. Survey removed from analysis.
Trinidad and Tobago	Trinidad and Tobago Multiple Indicator Cluster Survey 2000	2000	all	DPT1, DPT3, MCV1, Pol3	Central Statistical Office (Trinidad and Tobago), United Nations Children's Fund (UNICEF). Trinidad and Tobago Multiple Indicator Cluster Survey 2000. New York, United States of America: United Nations Children's Fund (UNICEF).	Estimates considered implausible. Survey estimates are systematically low compared to admin estimates and estimates from other established surveys series (Trinidad and Tobago Multiple Indicator Cluster Survey 2006). Survey removed from analysis.
Trinidad and Tobago	Trinidad and Tobago Multiple Indicator Cluster Survey 2006	2006	select	HepB3, Hib3	Central Statistical Office (Trinidad and Tobago) and United Nations Children's Fund (UNICEF). Trinidad and Tobago Multiple Indicator Cluster Survey 2006. New York, United States: United Nations Children's Fund (UNICEF).	Older age-cohorts of HepB3 and all age-cohorts of Hib3 precede introduction to national immunization schedule. Hib3 and select age-cohorts of HepB3 removed from analysis.
Trinidad and Tobago	Trinidad and Tobago Demographic and Health Survey 1987	1987	all	DPT1, DPT3, MCV1, Pol3	Family Planning Association (Trinidad and Tobago), Westinghouse; Institute for Resource Development. Trinidad and Tobago Demographic and Health Survey 1987. Fairfax, United States: ICF International.	Estimates considered implausible. Survey estimates are systematically high compared to admin estimates and estimates from other established surveys series. Survey removed from analysis.
Trinidad and Tobago	Trinidad and Tobago Multiple Indicator Cluster Survey 2011	2011	all	PCV3, Pol3	Central Statistical Office (Trinidad and Tobago), Ministry of Social Development and Family Services (Trinidad and Tobago), United Nations Children's Fund (UNICEF). Trinidad and Tobago Multiple Indicator Cluster Survey 2011. New York, United States of America: United Nations Children's Fund (UNICEF), 2018.	Survey precedes introduction of PCV to national immunization schedule. Pol3 estimates are implausibly high compared to admin estimates. PCV and Pol3 removed from analysis.
Tunisia	Tunisia Multiple Indicator Cluster Survey 2000	2000	all	MCV1	Ministry of Public Health (Tunisia), United Nations Children's Fund (UNICEF). Tunisia Multiple Indicator Cluster Survey 2000.	MCV1 estimates are systematically low compared to admin estimates and estimates from other established surveys series. MCV1 removed from analysis.
Tunisia	Tunisia Demographic and Health Survey 1988	1988	all	BCG, DPT1, DPT3, MCV1, Pol3	Macro Systems, Inc.; Institute for Resource Development, National Office for Family and Population, Ministry of Public Health (Tunisia). Tunisia Demographic and Health Survey 1988. Fairfax, United States of America: ICF International.	Survey estimates are implausibly high compared to admin estimates and estimates from other established survey series. Survey removed from analysis.

Country	Series	Year(s)	Cohort	Vaccine	Citation	Rationale for exclusion
Tunisia	Tunisia Multiple Indicator Cluster Survey 2018	2018	all	BCG, DPT1, DPT3, HepB3, PCV3, MCV1, PCV3, Pol3	National Institute of Statistics (Tunisia), United Nations Children's Fund (UNICEF). Tunisia Multiple Indicator Cluster Survey 2018. New York, United States of America: United Nations Children's Fund (UNICEF), 2019.	Estimates considered implausible. Survey estimates are systematically low compared to admin estimates and estimates from other established surveys series. Survey removed from analysis.
Turkey	Turkey Demographic and Health Survey 1998	1998	all	HepB3	Institute of Population Studies, Hacettepe University, Macro International, Inc. Turkey Demographic and Health Survey 1998. Fairfax, United States: ICF International.	Survey precedes introduction of HepB to national immunization schedule. HepB removed from analysis.
Turkey	Turkey Demographic and Health Survey 2013-2014	2013-2014	all	HepB3, Hib3, PCV3	Institute of Population Studies, Hacettepe University, Ministry of Development (Turkey), Ministry of Health (Turkey). Turkey Demographic and Health Survey 2013-2014. Fairfax, United States of America: ICF International, 2019.	Estimates considered implausible. Survey estimates are systematically low compared to admin estimates and estimates from other established surveys series (Turkey Demographic and Health Survey 2008). Survey removed from analysis.
Tuvalu	Tuvalu Demographic and Health Survey 2007	2007	all	BCG, DPT1, DPT3, MCV1, Pol3	Macro International, Inc, Secretariat of the Pacific Community (SPC), Tuvalu Central Statistics Division. Tuvalu Demographic and Health Survey 2007.	Estimates considered implausible. Survey estimates are systematically low compared to admin estimates and estimates from other established surveys series. Survey removed from analysis.
Uganda	Uganda AIDS Indicator Survey 2004-2005	2004-2005	select	HepB3	Division of Reproductive Health, Centers for Disease Control and Prevention (CDC), Ministry of Health (Uganda). Uganda AIDS Indicator Survey 2004-2005.	Older age-cohorts precede introduction of HepB3 to national immunization schedule. Select age-cohorts removed from analysis for HepB3.
United Arab Emirates	United Arab Emirates Child Health Survey 1987	1987	select	BCG, DPT1, DPT3, MCV1, Pol3	Ministry of Health (United Arab Emirates). United Arab Emirates Child Health Survey 1987.	Estimates considered implausible. Survey estimates do not follow a reasonable age structure. Older children have significantly higher coverage than reported in either administrative data or other survey series, suggesting survey is capturing results of "catch-up" immunization. Select age cohorts removed from analysis.
USA	United States National Health Interview Survey 1991	1991	all	DPT1, DPT3, PCV3, MCV1, Pol3	National Center for Health Statistics (NCHS), Centers for Disease Control and Prevention (CDC), US Census Bureau. United States National Health Interview Survey 1991. Hyattsville, United States: National Center for Health Statistics (NCHS), Centers for Disease Control and Prevention (CDC).	Survey estimates are systematically low and suggest an implausible drop in coverage in the 1980's and 1990's. Survey removed from analysis.
USA	United States National Health Interview Survey 1992	1992	all	DPT1, DPT3, HepB3, PCV3, MCV1, Pol3	National Center for Health Statistics (NCHS), Centers for Disease Control and Prevention (CDC), US Census Bureau. United States National Health Interview Survey 1992. Hyattsville, United States: National Center for Health Statistics (NCHS), Centers for Disease Control and Prevention (CDC).	Survey estimates are systematically low and suggest an implausible drop in coverage in the 1980's and 1990's. Survey removed from analysis.
USA	United States National Health Interview Survey 1993	1993	all	DPT1, DPT3, PCV3, MCV1, Pol3	National Center for Health Statistics (NCHS), Centers for Disease Control and Prevention (CDC), US Census Bureau. United States National Health Interview Survey 1993. Hyattville, United States: National Center for Health Statistics (NCHS), Centers for Disease Control and Prevention (CDC).	Survey estimates are systematically low and suggest an implausible drop in coverage in the 1980's and 1990's. Survey removed from analysis.
USA	United States National Health Interview Survey 1994	1994	all	DPT1, DPT3, HepB3, PCV3, MCV1, Pol3	National Center for Health Statistics (NCHS), Centers for Disease Control and Prevention (CDC), US Census Bureau. United States National Health Interview Survey 1994. Hyattsville, United States: National Center for Health Statistics (NCHS), Centers for Disease Control and Prevention (CDC).	Survey estimates are systematically low and suggest an implausible drop in coverage in the 1980's and 1990's. Survey removed from analysis.

Country	Series	Year(s)	Cohort	Vaccine	Citation	Rationale for exclusion
USA	United States National Immunization Survey-Child 2007-2008	2007-2008	select	RotaC	National Center for Health Statistics, Centers for Disease Control and Prevention, National Center for Immunization and Respiratory Diseases, CDC (NCIRD), National Opinion Research Center (NORC) (United States). United States National Immunization Survey-Child 2007-2008. Hyattsville, United States of America: National Center for Health Statistics, Centers for Disease Control and Prevention.	Older age-cohorts precede introduction of rotavirus vaccine to national immunization schedule. Select age-cohorts removed from analysis for rotavirus vaccine.
USA	United States National Immunization Survey-Child 2006-2007	2006-2007	all	RotaC	National Center for Health Statistics, Centers for Disease Control and Prevention, National Center for Immunization and Respiratory Diseases, CDC (NCIRD), National Opinion Research Center (NORC) (United States). United States National Immunization Survey-Child 2006-2007. Hyattsville, United States of America: National Center for Health Statistics, Centers for Disease Control and Prevention.	Survey precedes introduction of rotavirus vaccine to national immunization schedule. Rotavirus vaccine removed from analysis.
USA	United States National Immunization Survey-Child 2001-2002	2001-2002	select	PCV3, RotaC	Abt Associates Inc., National Center for Health Statistics, Centers for Disease Control and Prevention, National Center for Immunization and Respiratory Diseases, CDC (NCIRD). United States National Immunization Survey-Child 2001-2002. Hyattsville, United States of America: National Center for Health Statistics, Centers for Disease Control and Prevention.	Older age-cohorts of PCV3 and all age-cohorts of RotaC precede introduction to national immunization schedule. PCV3 and select age-cohorts of RotaC removed from analysis.
USA	United States National Immunization Survey-Child 2010-2011	2010-2011	select	RCV1	NORC at the University of Chicago, National Center for Health Statistics, Centers for Disease Control and Prevention, National Center for Immunization and Respiratory Diseases, CDC (NCIRD). United States National Immunization Survey-Child 2010-2011. Hyattsville, United States of America: National Center for Health Statistics, Centers for Disease Control and Prevention.	Older age-cohorts precede introduction of RCV1 to national immunization schedule. Select age-cohorts removed from analysis for RCV1.
USA	United States National Immunization Survey-Child 2012-2013	2012-2013	select	RCV1	NORC at the University of Chicago, National Center for Health Statistics, Centers for Disease Control and Prevention, National Center for Immunization and Respiratory Diseases, CDC (NCIRD). United States National Immunization Survey-Child 2012-2013. Hyattsville, United States of America: National Center for Health Statistics, Centers for Disease Control and Prevention.	Older age-cohorts precede introduction of RCV1 to national immunization schedule. Select age-cohorts removed from analysis for RCV1.
USA	United States National Immunization Survey-Child 2013-2014	2013-2014	select	RCV1	NORC at the University of Chicago, National Center for Health Statistics, Centers for Disease Control and Prevention, National Center for Immunization and Respiratory Diseases, CDC (NCIRD). United States National Immunization Survey-Child 2013-2014. Atlanta, United States of America: Centers for Disease Control and Prevention (CDC).	Older age-cohorts precede introduction of RCV1 to national immunization schedule. Select age-cohorts removed from analysis for RCV1.
Vanuatu	Vanuatu Multiple Indicator Cluster Survey 2007-2008	2007-2008	all	RCV1	Ministry of Health (Vanuatu), United Nations Children's Fund (UNICEF). Vanuatu Multiple Indicator Cluster Survey 2007-2008. New York, United States of America: United Nations Children's Fund (UNICEF).	Survey precedes introduction of RCV1 to national immunization schedule. RCV1 removed from analysis.
Vietnam	Vietnam Multiple Indicator Cluster Survey 1996	1996	all	MCV1	General Statistics Office (Vietnam), United Nations Children's Fund (UNICEF). Vietnam Multiple Indicator Cluster Survey 1996.	MCV1 estimates are systematically low compared to admin estimates and estimates from other established surveys series (Vietnam Demographic and Health Survey 1997). MCV1 removed from analysis.

Country	Series	Year(s)	Cohort	Vaccine	Citation	Rationale for exclusion
Vietnam	Vietnam Children Indicators 2001	2001	all	BCG, DPT3, MCV1, Pol3	Vietnam Children Indicators 2001.	Estimates considered implausible. Survey estimates are systematically high compared to admin estimates and estimates from other established surveys series (Vietnam Demographic and Health Survey 2002). Survey removed from analysis.
Yemen	Yemen Family Health Survey 2003	2003	all	Pol3	Central Statistical Organization (Yemen), League of Arab States, Ministry of Public Health and Population (Yemen), Pan Arab Project for Family Health (PAPFAM). Yemen Family Health Survey 2003.	Pol3 estimates are systematically low compared to admin estimates and estimates from other established surveys series (Yemen Household Budget Survey 2005-2006). Pol3 removed from analysis.
Yemen	Yemen Multiple Indicator Cluster Survey 2006	2006	select	Hib3	Ministry of Health (Yemen) and United Nations Children's Fund (UNICEF). Yemen Multiple Indicator Cluster Survey 2006. New York, United States: United Nations Children's Fund (UNICEF).	Older age-cohorts precede introduction of Hib3 to national immunization schedule. Select age-cohorts removed from analysis for Hib3.
Yemen	Yemen Demographic and Health Survey 1991-1992	1991-1992	all	BCG, DPT1, DPT3, MCV1, Pol3	Central Statistical Organization (Yemen), League of Arab States, Macro International, Inc. Yemen Demographic and Health Survey 1991-1992. Fairfax, United States: ICF International.	Estimates considered implausible. Survey estimates are systematically high compared to admin estimates and estimates from other established surveys series. Survey removed from analysis.
Yemen	Yemen Demographic and Health Survey 2013	2013	select	PCV3	Central Statistical Organization (Yemen), ICF International, Ministry of Public Health and Population (Yemen). Yemen Demographic and Health Survey 2013. Fairfax, United States of America: ICF International.	Older age-cohorts precede introduction of PCV3 to national immunization schedule. Select age-cohorts removed from analysis for PCV3.
Yemen	Yemen Immunization, Diarrhoeal Disease, Maternal and Child Mortality Survey 1990	1990	all	BCG, DPT1, DPT3, MCV1, Pol3	Yemen Immunization, Diarrhoeal Disease, Maternal and Child Mortality Survey 1990.	Estimates considered implausible. Survey estimates are systematically high compared to admin estimates and estimates from other established surveys series. Survey removed from analysis.
Yemen	Yemen National Social Protection Monitoring Survey 2012-2013	2012-2013	all	BCG, DPT1, DPT3, HepB3, PCV3, MCV1, MCV2, PCV3, Pol3	Ministry of Planning and International Cooperation (Yemen), International Policy Center for Inclusive Growth, Interaction in Development (Yemen), UNICEF Yemen. Yemen National Social Protection Monitoring Survey 2012-2013.	Survey estimates include implausibly high coverage rates of 100%. Survey removed from analysis.
Zambia	Zambia Living Conditions Monitoring Survey 1998	1998	all	MCV2	Central Statistical Office (Zambia), London School of Hygiene and Tropical Medicine. Zambia Living Conditions Monitoring Survey 1998. Lusaka, Zambia: Central Statistical Office (Zambia).	Survey precedes introduction of MCV2 to national immunization schedule. MCV2 removed from analysis.
Zambia	Zambia Living Conditions Monitoring Survey 2002-2003	2002-2003	all	MCV2	Central Statistical Office (Zambia). Zambia Living Conditions Monitoring Survey 2002-2003. Lusaka, Zambia: Central Statistical Office (Zambia).	Survey precedes introduction of MCV2 to national immunization schedule. MCV2 removed from analysis.
Zambia	Zambia Multiple Indicator Cluster Survey 1999	1999	all	BCG	Central Statistical Office (Zambia), Food Security, Health and Nutrition Information System (Zambia), United Nations Children's Fund (UNICEF). Zambia Multiple Indicator Cluster Survey 1999. New York, United States of America: United Nations Children's Fund (UNICEF).	BCG estimates are implausibly low compared to admin estimates and estimates from other established surveys series for some age-cohorts (Zambia Living Conditions Monitoring Survey 1998). Select age-cohorts removed from analysis for BCG.
Zambia	Zambia Demographic and Health Survey 2007	2007	select	HepB3, Hib3	Central Statistical Office (Zambia), Macro International, Inc. Zambia Demographic and Health Survey 2007. Fairfax, United States of America: ICF International.	Older age-cohorts precede introduction of HepB3 and Hib3 to national immunization schedule. Select age-cohorts removed from analysis for HepB3 and Hib3.
Zambia	Zambia Global Fund Household Health Coverage Survey 2008	2008	select	DPT1, DPT3, HepB3	Central Statistical Office (Zambia). Zambia Global Fund Household Health Coverage Survey 2008. Lusaka, Zambia: Central Statistical Office (Zambia).	DPT3 estimates are implausibly low compared to admin estimates and estimates from other established surveys series for some age-cohorts (Zambia Demographic and Health Survey 2007). Older age-cohorts precede

Country	Series	Year(s)	Cohort	Vaccine	Citation	Rationale for exclusion
						introduction of HepB3 to national immunization schedule. Select age-cohorts removed from analysis for HepB3 and DPT3.
Zimbabwe	Zimbabwe Demographic and Health Survey 2005-2006	2005-2006	all	MCV2	Central Statistical Office (Zimbabwe), Macro International, Inc. Zimbabwe Demographic and Health Survey 2005-2006. Fairfax, United States of America: ICF International.	Survey precedes introduction of MCV2 to national immunization schedule. MCV2 removed from analysis.
Zimbabwe	Zimbabwe Demographic and Health Survey 2010-2011	2010-2011	select	Hib3	ICF Macro, Zimbabwe National Statistics Agency. Zimbabwe Demographic and Health Survey 2010-2011. Calverton, United States of America: ICF Macro, 2012.	Older age-cohorts precede introduction of Hib3 to national immunization schedule. Select age-cohorts removed from analysis for Hib3.
Zimbabwe	Zimbabwe Multiple Indicator Cluster Survey 2014	2014	select	PCV3	United Nations Children's Fund (UNICEF), Zimbabwe National Statistics Agency. Zimbabwe Multiple Indicator Cluster Survey 2014. New York, United States of America: United Nations Children's Fund (UNICEF), 2015.	Older age-cohorts precede introduction of PCV3 to national immunization schedule. Select age-cohorts removed from analysis for PCV3.
Zimbabwe	Zimbabwe Demographic and Health Survey 2015	2015	select	RotaC	ICF International, National Microbiology Reference Laboratory, Harare Central Hospital (NMRL) (Zimbabwe), Zimbabwe National Statistics Agency. Zimbabwe Demographic and Health Survey 2015. Fairfax, United States of America: ICF International, 2016.	Older age-cohorts precede introduction of RotaC to national immunization schedule. Select age-cohorts removed from analysis for RotaC.

Supplementary table 3. Supplementary ancillary data used in vaccine coverage modelling. These data supplement the country-reported data obtained from the JRF (see supplementary section 2.5). The Ancillary Source Type field denotes which kind of data the source provides (Introduction, meaning vaccine-specific introduction; Removal, meaning vaccine-specific phase-out or non-introduction; and Disruption, meaning vaccine-specific acute disruption to vaccine delivery). These sources can also be explored following this link to the GHDx input data sources tool: http://ghdx.healthdata.org/record/ihme-data/gbd-2020-routine-childhood-vaccination-coverage-1980-2019. BCG=Bacillus Calmette-Guérin. DTP1=diphtheria-tetanus-pertussis, first dose. DTP3=diphtheria-tetanus-pertussis, third dose. HepB3=hepatitis B vaccine, third dose. Hib3=*Haemophilus influenzae* type b vaccine, third dose. MCV1=measles-containing vaccine, first dose. MCV2=measles-containing vaccine, second dose. PCV3=pneumococcal conjugate vaccine, third dose. Pol3=polio vaccine, third dose. RCV1=rubella-containing vaccine, first dose. RotaC=completed rotavirus series.

GHDx id	Country	Series	Citation	Vaccine	Link	Ancillary Source Type
467154	Algeria	Hepatitis B vaccination in Africa: mission accomplished?	François G, Dochez C, Mphahlele MJ, Burnett R, Hal GV, Meheus A. Hepatitis B vaccination in Africa: mission accomplished?. South Afr J Epidemiol Infect. 2008; 23(1): 24-8	HepB3	GHDx	Introduction
466979	American Samoa	Progress Toward Hepatitis B Control and Elimination of Mother-to-Child Transmission of Hepatitis B Virus — Western Pacific Region, 2005–2017	Woodring J, Pastore R, Brink A, Ishikawa N, Takashima Y, Tohme RA. Progress Toward Hepatitis B Control and Elimination of Mother-to-Child Transmission of Hepatitis B Virus — Western Pacific Region, 2005–2017. MMWR Morb Mort Wkly. 2019; 68(8): 195-200.	HepB3	GHDx	Introduction
467639	Austria	Measles and rubella country profile - Austria (2019)	Centers for Disease Control and Prevention (CDC). Current Trends Measles -- Puerto Rico, 1993, and the Measles Elimination Program. MMWR Morb Mort Wkly. 1994; 43(09): 171-3.	MCV2, RCV1	GHDx	Introduction
468045	Bermuda	Bermuda EPI Country Report 2019	Pan American Health Organization (PAHO). Bermuda EPI Country Report 2019. Washington, D.C., United States of America: Pan American Health Organization (PAHO).	Hib3, HepB3, RCV1, PCV3	GHDx	Introduction
467993	Bhutan	Bhutan Expanded Programme on Immunization (EPI) Factsheet 2020	Ministry of Health (Bhutan), World Health Organization Regional Office for South-East Asia (SEARO). Bhutan Expanded Programme on Immunization (EPI) Factsheet 2020. New Delhi, India: World Health Organization Regional Office for South-East Asia (SEARO), 2020.	MCV2	GHDx	Introduction
467635	Brazil	Brazil EPI Country Report 2018	Pan American Health Organization (PAHO). Brazil EPI Country Report 2018. Washington, D.C., United States of America: Pan American Health Organization (PAHO), 2019.	HepB3, MCV2, RCV1	GHDx	Introduction
467154	Côte d'Ivoire	Hepatitis B vaccination in Africa: mission accomplished?	François G, Dochez C, Mphahlele MJ, Burnett R, Hal GV, Meheus A. Hepatitis B vaccination in Africa: mission accomplished?. South Afr J Epidemiol Infect. 2008; 23(1): 24-8	HepB3	GHDx	Introduction
467035	Canada	Canada EPI Country Report 2019	Pan American Health Organization (PAHO). Canada EPI Country Report 2019. Washington, D.C., United States of America: Pan American Health Organization (PAHO), 2020.	HepB3, RCV1, RotaC	GHDx	Introduction
466799	China	Effectiveness of the live attenuated rotavirus vaccine produced by a domestic manufacturer in China studied using a population-based case-control design	Zhen SS, Li Y, Wang SM, Zhang XJ, Hao ZY, Chen Y, Wang D, Zhang YH, Zhang ZY, Ma JC, Zhou P, Zhang Z, Jiang ZW, Zhao YL, Wang XY. Effectiveness of the live attenuated rotavirus vaccine produced by a domestic manufacturer in China studied using a population-based case-control design. Emerg Microbes Infect. 2015; 4(10): e64.	RotaC	GHDx	Introduction
467145	China	Streptococcus pneumoniae and Haemophilus influenzae type b carriage in Chinese children aged 12-18 months in Shanghai, China: a cross-sectional study	Hu J, Sun X, Huang Z, Wagner AL, Carlson B, Yang J, Tang S, Li Y, Boulton ML, Yuan Z. Streptococcus pneumoniae and Haemophilus influenzae type b carriage in Chinese children aged 12-18 months in Shanghai, China: a cross-sectional study. BMC Infect Dis. 2016; 16: 149.	Hib3, PCV3	GHDx	Introduction
467613	Estonia	Measles and rubella country profile - Estonia (2019)	Centers for Disease Control and Prevention (CDC). Current Trends Measles -- Puerto Rico, 1993, and the Measles Elimination Program. MMWR Morb Mort Wkly. 1994; 43(09): 171-3.	RCV1	GHDx	Introduction
467150	France	Impact of Haemophilus influenzae type b vaccination on the incidence of invasive Haemophilus influenzae disease in France, 15 years after its introduction	Georges S, Lepoutre A, Dabernat H, Levy-Bruhl D. Impact of Haemophilus influenzae type b vaccination on the incidence of invasive Haemophilus influenzae disease in France, 15 years after its introduction. Epidemiol Infect. 2013; 141(9): 1787-96.	Hib3	GHDx	Introduction
467513	Germany	Measles and rubella country profile - Germany (2019)	Centers for Disease Control and Prevention (CDC). Current Trends Measles -- Puerto Rico, 1993, and the Measles Elimination Program. MMWR Morb Mort Wkly. 1994; 43(09): 171-3.	MCV2, RCV1	GHDx	Introduction
466979	Guam	Progress Toward Hepatitis B Control and Elimination of Mother-to-Child Transmission of Hepatitis B Virus — Western Pacific Region, 2005–2017	Woodring J, Pastore R, Brink A, Ishikawa N, Takashima Y, Tohme RA. Progress Toward Hepatitis B Control and Elimination of Mother-to-Child Transmission of Hepatitis B Virus — Western Pacific Region, 2005–2017. MMWR Morb Mort Wkly. 2019; 68(8): 195-200.	HepB3	GHDx	Introduction
467154	Guinea	Hepatitis B vaccination in Africa: mission accomplished?	François G, Dochez C, Mphahlele MJ, Burnett R, Hal GV, Meheus A. Hepatitis B vaccination in Africa: mission accomplished?. South Afr J Epidemiol Infect. 2008; 23(1): 24-8	HepB3	GHDx	Introduction
466984	India	Pneumonia vaccines to be introduced in 5 states	PTI. Pneumonia vaccines to be introduced in 5 states. The Indian Express [Internet]. 2016 Nov 8.	PCV3	GHDx	Introduction

GHDx id	Country	Series	Citation	Vaccine	Link	Ancillary Source Type
467994	India	India Expanded Programme on Immunization (EPI) Factsheet 2020	Ministry of Health and Family Welfare (India), World Health Organization Regional Office for South-East Asia (SEARO). India Expanded Programme on Immunization (EPI) Factsheet 2020. New Delhi, India: World Health Organization Regional Office for South-East Asia (SEARO), 2020.	MCV1	GHDx	Introduction
466869	Indonesia	Indonesia EPI Fact Sheet 2016	World Health Organization Regional Office for South-East Asia (SEARO). Indonesia EPI Fact Sheet 2016. New Delhi, India: World Health Organization Regional Office for South-East Asia (SEARO), 2016.	MCV2, HepB3	GHDx	Introduction
467149	Israel	Decreased immune response to hepatitis B eight years after routine vaccination in Israel	Gold Y, Somech R, Mandel D, Peled Y, Reif S. Decreased immune response to hepatitis B eight years after routine vaccination in Israel. Acta Paediatr. 2003; 92(10): 1158-62.	HepB3	GHDx	Introduction
466985	Italy	Measles and rubella country profile - Italy (2019)	Centers for Disease Control and Prevention (CDC). Current Trends Measles -- Puerto Rico, 1993, and the Measles Elimination Program. MMWR Morb Mort Wkly. 1994; 43(09): 171-3.	MCV2	GHDx	Introduction
467210	Jamaica	Jamaica EPI Country Report 2019	Pan American Health Organization (PAHO). Jamaica EPI Country Report 2019. Washington, D.C., United States of America: Pan American Health Organization (PAHO).	HepB3	GHDx	Introduction
466888	Latvia	National Immunization Program in Latvia 2015	Centre for Disease Prevention and Control (Latvia). National Immunization Program in Latvia 2015. 2015.	HepB3	GHDx	Introduction
467640	Latvia	Measles and rubella country profile - Latvia (2019)	Centers for Disease Control and Prevention (CDC). Current Trends Measles -- Puerto Rico, 1993, and the Measles Elimination Program. MMWR Morb Mort Wkly. 1994; 43(09): 171-3.	MCV2	GHDx	Introduction
466889	Lithuania	Lithuania National Immunization Program: Coverage and Impact 2015	Lithuania National Immunization Program: Coverage and Impact 2015.	Hib3	GHDx	Introduction
466856	Maldives	Maldives Immunization Handbook for Health Care Professionals	Health Protection Agency (Maldives), World Health Organization (WHO). Maldives Immunization Handbook for Health Care Professionals. Sosun Magu, Maldives: Health Protection Agency (Maldives), 2015.	HepB3	GHDx	Introduction
467996	Maldives	Maldives Expanded Programme on Immunization (EPI) Factsheet 2020	Ministry of Health (Maldives), World Health Organization Regional Office for South-East Asia (SEARO). Maldives Expanded Programme on Immunization (EPI) Factsheet 2020. New Delhi, India: World Health Organization Regional Office for South-East Asia (SEARO), 2020.	Hib3	GHDx	Introduction
467154	Mali	Hepatitis B vaccination in Africa: mission accomplished?	François G, Dochez C, Mphahlele MJ, Burnett R, Hal GV, Meheus A. Hepatitis B vaccination in Africa: mission accomplished?. South Afr J Epidemiol Infect. 2008; 23(1): 24-8	HepB3	GHDx	Introduction
467154	Mauritius	Hepatitis B vaccination in Africa: mission accomplished?	François G, Dochez C, Mphahlele MJ, Burnett R, Hal GV, Meheus A. Hepatitis B vaccination in Africa: mission accomplished?. South Afr J Epidemiol Infect. 2008; 23(1): 24-8	HepB3	GHDx	Introduction
467997	Myanmar	Myanmar Expanded Programme on Immunization (EPI) Factsheet 2020	Ministry of Health and Sports (Myanmar), World Health Organization Regional Office for South-East Asia (SEARO). Myanmar Expanded Programme on Immunization (EPI) Factsheet 2020. New Delhi, India: World Health Organization Regional Office for South-East Asia (SEARO), 2020.	MCV1	GHDx	Introduction
466793	Nauru	Epidemiology of hepatitis B infection in the Western Pacific and South East Asia	Gust ID. Epidemiology of hepatitis B infection in the Western Pacific and South East Asia. Gut. 1996; 38 Suppl 2: S18-23.	HepB3	GHDx	Introduction
466978	Nauru	Progress Toward the 2012 Measles Elimination Goal --- Western Pacific Region, 1990--2008	Centers for Disease Control and Prevention (CDC). Progress Toward the 2012 Measles Elimination Goal --- Western Pacific Region, 1990--2008. MMWR Morb Mort Wkly. 2009; 58(24): 229-73.	MCV2	GHDx	Introduction
467154	Niger	Hepatitis B vaccination in Africa: mission accomplished?	François G, Dochez C, Mphahlele MJ, Burnett R, Hal GV, Meheus A. Hepatitis B vaccination in Africa: mission accomplished?. South Afr J Epidemiol Infect. 2008; 23(1): 24-8	HepB3	GHDx	Introduction
467154	Nigeria	Hepatitis B vaccination in Africa: mission accomplished?	François G, Dochez C, Mphahlele MJ, Burnett R, Hal GV, Meheus A. Hepatitis B vaccination in Africa: mission accomplished?. South Afr J Epidemiol Infect. 2008; 23(1): 24-8	HepB3	GHDx	Introduction
466979	Northern Mariana Islands	Progress Toward Hepatitis B Control and Elimination of Mother-to-Child Transmission of Hepatitis B Virus — Western Pacific Region, 2005–2017	Woodring J, Pastore R, Brink A, Ishikawa N, Takashima Y, Tohme RA. Progress Toward Hepatitis B Control and Elimination of Mother-to-Child Transmission of Hepatitis B Virus — Western Pacific Region, 2005–2017. MMWR Morb Mort Wkly. 2019; 68(8): 195-200.	HepB3	GHDx	Introduction
466893	Norway	Evaluation of a Hepatitis B Selective Vaccination Programme in Norway	Norwegian Institute of Public Health. Evaluation of a Hepatitis B Selective Vaccination Programme in Norway.	HepB3	GHDx	Introduction
466979	Palau	Progress Toward Hepatitis B Control and Elimination of Mother-to-Child Transmission of Hepatitis B Virus — Western Pacific Region, 2005–2017	Woodring J, Pastore R, Brink A, Ishikawa N, Takashima Y, Tohme RA. Progress Toward Hepatitis B Control and Elimination of Mother-to-Child Transmission of Hepatitis B Virus — Western Pacific Region, 2005–2017. MMWR Morb Mort Wkly. 2019; 68(8): 195-200.	HepB3	GHDx	Introduction
466857	Palestine	Vaccine-preventable Diseases in Palestine: Monitoring, Prevention and Recommendations	Vaccine-preventable Diseases in Palestine: Monitoring, Prevention and Recommendations.	PCV3, Hib3, RCV1, RCV2	GHDx	Introduction

GHDx id	Country	Series	Citation	Vaccine	Link	Ancillary Source Type
466894	Palestine	Palestine WHO and UNICEF Estimates of Immunization Coverage 2016 Revision	United Nations Children's Fund (UNICEF), World Health Organization (WHO). Palestine WHO and UNICEF Estimates of Immunization Coverage 2016 Revision. New York, United States of America: United Nations Children's Fund (UNICEF), 2017.	MCV2	GHDx	Introduction
467742	Palestine	Evaluation of the routine hepatitis B immunization programme in Palestine, 1996	Kuhail S, el-Khodary R, Ahmed F. Evaluation of the routine hepatitis B immunization programme in Palestine, 1996. East Mediterr Health J. 2000; 6(5-6): 864-9.	HepB3	GHDx	Introduction
468012	Palestine	The Number of Children Hospitalized for Diarrhea Drops Dramatically in Gaza after Rotavirus Vaccine Introduction	Ministry of Health (Palestine). The Number of Children Hospitalized for Diarrhea Drops Dramatically in Gaza after Rotavirus Vaccine Introduction.	RotaC	GHDx	Introduction
467147	Peru	Progress in vaccination towards hepatitis B control and elimination in the Region of the Americas	Ropero Álvarez AM, Pérez-Vilar S, Pacis-Tirso C, Contreras M, El Omeiri N, Ruiz-Matus C, Velandia-González M. Progress in vaccination towards hepatitis B control and elimination in the Region of the Americas. BMC Public Health. 2017; 17(1): 325.	HepB3	GHDx	Introduction
467203	Peru	Peru EPI Country Report 2018	Pan American Health Organization (PAHO). Peru EPI Country Report 2018. Washington, D.C., United States of America: Pan American Health Organization (PAHO).	PCV3	GHDx	Introduction
467158	Poland	A cohesive European policy for hepatitis B vaccination, are we there yet?	Lernout T, Hendrickx G, Vorsters A, Mosina L, Emiroglu N, Van Damme P. A cohesive European policy for hepatitis B vaccination, are we there yet?. Clin Microbiol Infect. 2014; 20: 19-24.	HepB3	GHDx	Introduction
466795	Portugal	Emergence of nonencapsulated and encapsulated non-b-type invasive Haemophilus influenzae isolates in Portugal (1989-2001)	Bajanca P, Caniça M, Multicenter Study Group. Emergence of nonencapsulated and encapsulated non-b-type invasive Haemophilus influenzae isolates in Portugal (1989-2001). J Clin Microbiol. 2004; 42(2): 807-10.	Hib3	GHDx	Introduction
466974	Puerto Rico	Current Trends Measles -- Puerto Rico, 1993, and the Measles Elimination Program	Centers for Disease Control and Prevention (CDC). Current Trends Measles -- Puerto Rico, 1993, and the Measles Elimination Program. MMWR Morb Mort Wkly. 1994; 43(09): 171-3.	RCV1, MCV2	GHDx	Introduction
467211	Saint Lucia	Saint Lucia EPI Country Report 2019	Pan American Health Organization (PAHO). Saint Lucia EPI Country Report 2019. Washington, D.C., United States of America: Pan American Health Organization (PAHO).	MCV2	GHDx	Introduction
466979	Singapore	Progress Toward Hepatitis B Control and Elimination of Mother-to-Child Transmission of Hepatitis B Virus — Western Pacific Region, 2005–2017	Woodring J, Pastore R, Brink A, Ishikawa N, Takashima Y, Tohme RA. Progress Toward Hepatitis B Control and Elimination of Mother-to-Child Transmission of Hepatitis B Virus — Western Pacific Region, 2005–2017. MMWR Morb Mort Wkly. 2019; 68(8): 195-200.	HepB3	GHDx	Introduction
467044	Singapore	Communicable Diseases Surveillance in Singapore 2014	Ministry of Health (Singapore). Communicable Diseases Surveillance in Singapore 2014. Singapore, Singapore: Ministry of Health (Singapore).	MCV2	GHDx	Introduction
467427	Slovakia	National analysis of bacterial meningitis in Slovakia, 1997-2007	Hudeckova H, Jesenak M, Maria A, Svihrova V, Banovcin P. National analysis of bacterial meningitis in Slovakia, 1997-2007. Public Health Rep. 2010; 125(1): 129-36.	Hib3	GHDx	Introduction
467515	Spain	Measles and rubella country profile - Spain (2019)	Centers for Disease Control and Prevention (CDC). Current Trends Measles -- Puerto Rico, 1993, and the Measles Elimination Program. MMWR Morb Mort Wkly. 1994; 43(09): 171-3.	MCV2	GHDx	Introduction
467995	Sri Lanka	Sri Lanka Expanded Programme on Immunization (EPI) Factsheet 2020	Ministry of Health (Sri Lanka), World Health Organization Regional Office for South-East Asia (SEARO). Sri Lanka Expanded Programme on Immunization (EPI) Factsheet 2020. New Delhi, India: World Health Organization Regional Office for South-East Asia (SEARO), 2020.	RCV1	GHDx	Introduction
468027	Sri Lanka	Sustaining Vaccination Coverage: Continued National Commitment to Primary Health Care with a Strong Focus on Community Engagement - Case Study Sri Lanka	United Nations Children's Fund (UNICEF). Sustaining Vaccination Coverage: Continued National Commitment to Primary Health Care with a Strong Focus on Community Engagement - Case Study Sri Lanka. New York, United States of America: United Nations Children's Fund (UNICEF), 2019.	MCV1	GHDx	Introduction
467514	Sweden	Measles and rubella country profile - Sweden (2019)	Centers for Disease Control and Prevention (CDC). Current Trends Measles -- Puerto Rico, 1993, and the Measles Elimination Program. MMWR Morb Mort Wkly. 1994; 43(09): 171-3.	RCV1	GHDx	Introduction
467949	Sweden	Health Promotion and Prevention Work With Hepatitis in Sweden	Health Promotion and Prevention Work With Hepatitis in Sweden. Östersund, Sweden: Public Health Agency of Sweden, 2019.	HepB3	GHDx	Introduction
467641	Syria	Synthesis Report: New Vaccine Adoption In Lower-Middle-Income Countries	Synthesis Report: New Vaccine Adoption In Lower-Middle-Income Countries.	HepB3	GHDx	Introduction
418161	Taiwan (province of China)	Effectiveness of 2 rotavirus vaccines against rotavirus disease in Taiwanese infants	Chang WC, Yen C, Wu FT, Huang YC, Lin JS, Huang FC, Yu HT, Chi CL, Lin HY, Tate JE, Parashar UD, Wu HS, Hsiung CA. Effectiveness of 2 rotavirus vaccines against rotavirus disease in Taiwanese infants. Pediatr Infect Dis J. 2014; 33(3): e81-6.	RotaC	GHDx	Introduction
466797	Taiwan (province of China)	Pneumococcal disease and use of pneumococcal vaccines in Taiwan	Wei SH, Chiang CS, Chen CL, Chiu CH. Pneumococcal disease and use of pneumococcal vaccines in Taiwan. Clin Exp Vaccine Res. 2015; 4(2): 121-9.	PCV3	GHDx	Introduction

GHDx id	Country	Series	Citation	Vaccine	Link	Ancillary Source Type
467746	Taiwan (province of China)	Nationwide hepatitis B vaccination program in Taiwan: effectiveness in the 20 years after it was launched	Chien YC, Jan CF, Kuo HS, Chen CJ. Nationwide hepatitis B vaccination program in Taiwan: effectiveness in the 20 years after it was launched. Epidemiol Rev. 2006; 28: 126-35.	HepB3	GHDx	Introduction
467752	Taiwan (province of China)	Epidemiology of measles in Taiwan: dynamics of transmission and timeliness of reporting during an epidemic in 1988-9	Lee MS, King CC, Chen CJ, Yang SY, Ho MS. Epidemiology of measles in Taiwan: dynamics of transmission and timeliness of reporting during an epidemic in 1988-9. Epidemiol Infect. 1995; 114(2): 345-59.	MCV2	GHDx	Introduction
467756	Taiwan (province of China)	Humoral immunity to mumps in a highly vaccinated population in Taiwan	Ho YH, Tsai CC, Tsai YW, Wang YC, Lin TY, Lee DJ, Chen CJ. Humoral immunity to mumps in a highly vaccinated population in Taiwan. J Microbiol Immunol Infect. 2019; 52(3): 379-385.	RCV1	GHDx	Introduction
467998	Thailand	Thailand Expanded Programme on Immunization (EPI) Factsheet 2020	Ministry of Public Health (Thailand), World Health Organization Regional Office for South-East Asia (SEARO). Thailand Expanded Programme on Immunization (EPI) Factsheet 2020. New Delhi, India: World Health Organization Regional Office for South-East Asia (SEARO), 2020.	HepB3, RCV1, MCV1	GHDx	Introduction
466979	Tokelau	Progress Toward Hepatitis B Control and Elimination of Mother-to-Child Transmission of Hepatitis B Virus — Western Pacific Region, 2005–2017	Woodring J, Pastore R, Brink A, Ishikawa N, Takashima Y, Tohme RA. Progress Toward Hepatitis B Control and Elimination of Mother-to-Child Transmission of Hepatitis B Virus — Western Pacific Region, 2005–2017. MMWR Morb Mort Wkly. 2019; 68(8): 195-200.	HepB3	GHDx	Introduction
467622	Trinidad and Tobago	Trinidad and Tobago EPI Country Report 2019	Pan American Health Organization (PAHO). Trinidad and Tobago EPI Country Report 2019. Washington, D.C., United States of America: Pan American Health Organization (PAHO), 2020.	HepB3, MCV1	GHDx	Introduction
468036	Trinidad and Tobago	Trinidad and Tobago's Second Periodic Report under the Convention on the Rights of the Child	Trinidad and Tobago's Second Periodic Report under the Convention on the Rights of the Child.	Hib3	GHDx	Introduction
467975	Tunisia	Case Studies of IPV Introduction: Albania, Nigeria, and Tunisia	Case Studies of IPV Introduction: Albania, Nigeria, and Tunisia. Baltimore, United States of America: International Vaccine Access Center, Johns Hopkins Bloomberg School of Public Health, 2018.	Hib3	GHDx	Introduction
467636	Turkey	Measles and rubella country profile - Turkey (2019)	Centers for Disease Control and Prevention (CDC). Current Trends Measles -- Puerto Rico, 1993, and the Measles Elimination Program. MMWR Morb Mort Wkly. 1994; 43(09): 171-3.	MCV2	GHDx	Introduction
466885	Tuvalu	Strengthening Development Partner Support to Immunisation Programs in the Pacific	Strengthening Development Partner Support to Immunisation Programs in the Pacific. Australia: Australian Government Department of Foreign Affairs and Trade (DFAT).	MCV2	GHDx	Introduction
467637	Ukraine	Measles and rubella country profile - Ukraine (2019)	Centers for Disease Control and Prevention (CDC). Current Trends Measles -- Puerto Rico, 1993, and the Measles Elimination Program. MMWR Morb Mort Wkly. 1994; 43(09): 171-3.	MCV2, RCV1	GHDx	Introduction
467638	Uzbekistan	Measles and rubella country profile - Uzbekistan (2019)	Centers for Disease Control and Prevention (CDC). Current Trends Measles -- Puerto Rico, 1993, and the Measles Elimination Program. MMWR Morb Mort Wkly. 1994; 43(09): 171-3.	RCV1	GHDx	Introduction
466896	Vanuatu	WHO WPRO Hepatitis B Control Country Profile 2017	World Health Organization Regional Office for the Western Pacific (WPRO-WHO). WHO WPRO Hepatitis B Control Country Profile 2017. Manila, Philippines: World Health Organization Regional Office for the Western Pacific (WPRO-WHO), 2018.	HepB3	GHDx	Introduction
468024	American Samoa	Expanded Programme on Immunization (EPI) Immunization schedules in the WHO Western Pacific Region, 1995	World Health Organization. Expanded Programme on Immunization (EPI) Immunization schedules in the WHO Western Pacific Region, 1995. WHO Wkly Epidemiol Rec. 1996; 71(18): 133-137.	BCG	GHDx	Removal
468043	Antigua and Barbuda	Health in the Americas 1998 Edition	Pan American Health Organization (PAHO). Health in the Americas 1998 Edition. Washington, D.C., United States of America: Pan American Health Organization (PAHO), 1998.	BCG	GHDx	Removal
437645	The Bahamas	Bahamas Expanded Program on Immunization (EPI) Immunization Schedule 2018	Ministry of Health (Bahamas). Bahamas Expanded Program on Immunization (EPI) Immunization Schedule 2018. Bahamas: Ministry of Health (Bahamas).	BCG	GHDx	Removal
467070	Belgium	The BCG World Atlas 3rd Edition 2020	Aeras (United States), McGill University (Canada), McGill University Health Centre (Canada), University of Ottawa. The BCG World Atlas 3rd Edition 2020. Canada: McGill University (Canada), 2020.	BCG	GHDx	Removal
468045	Bermuda	Bermuda EPI Country Report 2019	Pan American Health Organization (PAHO). Bermuda EPI Country Report 2019. Washington, D.C., United States of America: Pan American Health Organization (PAHO).	BCG	GHDx	Removal
467070	Canada	The BCG World Atlas 3rd Edition 2020	Aeras (United States), McGill University (Canada), McGill University Health Centre (Canada), University of Ottawa. The BCG World Atlas 3rd Edition 2020. Canada: McGill University (Canada), 2020.	BCG	GHDx	Removal
467070	Cyprus	The BCG World Atlas 3rd Edition 2020	Aeras (United States), McGill University (Canada), McGill University Health Centre (Canada), University of Ottawa. The BCG World Atlas 3rd Edition 2020. Canada: McGill University (Canada), 2020.	BCG	GHDx	Removal

GHDx id	Country	Series	Citation	Vaccine	Link	Ancillary Source Type
467070	Czech Republic	The BCG World Atlas 3rd Edition 2020	Aeras (United States), McGill University (Canada), McGill University Health Centre (Canada), University of Ottawa. The BCG World Atlas 3rd Edition 2020. Canada: McGill University (Canada), 2020.	BCG	GHDx	Removal
467070	Denmark	The BCG World Atlas 3rd Edition 2020	Aeras (United States), McGill University (Canada), McGill University Health Centre (Canada), University of Ottawa. The BCG World Atlas 3rd Edition 2020. Canada: McGill University (Canada), 2020.	BCG	GHDx	Removal
467070	Finland	The BCG World Atlas 3rd Edition 2020	Aeras (United States), McGill University (Canada), McGill University Health Centre (Canada), University of Ottawa. The BCG World Atlas 3rd Edition 2020. Canada: McGill University (Canada), 2020.	BCG	GHDx	Removal
467070	France	The BCG World Atlas 3rd Edition 2020	Aeras (United States), McGill University (Canada), McGill University Health Centre (Canada), University of Ottawa. The BCG World Atlas 3rd Edition 2020. Canada: McGill University (Canada), 2020.	BCG	GHDx	Removal
467070	Germany	The BCG World Atlas 3rd Edition 2020	Aeras (United States), McGill University (Canada), McGill University Health Centre (Canada), University of Ottawa. The BCG World Atlas 3rd Edition 2020. Canada: McGill University (Canada), 2020.	BCG	GHDx	Removal
467070	Greece	The BCG World Atlas 3rd Edition 2020	Aeras (United States), McGill University (Canada), McGill University Health Centre (Canada), University of Ottawa. The BCG World Atlas 3rd Edition 2020. Canada: McGill University (Canada), 2020.	BCG	GHDx	Removal
467070	Greenland	The BCG World Atlas 3rd Edition 2020	Aeras (United States), McGill University (Canada), McGill University Health Centre (Canada), University of Ottawa. The BCG World Atlas 3rd Edition 2020. Canada: McGill University (Canada), 2020.	BCG	GHDx	Removal
468047	Grenada	TB Vaccine no longer in use in Grenada may help fight COVID-19	TB Vaccine no longer in use in Grenada may help fight COVID-19.	BCG	GHDx	Removal
468024	Guam	Expanded Programme on Immunization (EPI) Immunization schedules in the WHO Western Pacific Region, 1995	World Health Organization. Expanded Programme on Immunization (EPI) Immunization schedules in the WHO Western Pacific Region, 1995. WHO Wkly Epidemiol Rec. 1996; 71(18): 133-137.	BCG	GHDx	Removal
467070	Iceland	The BCG World Atlas 3rd Edition 2020	Aeras (United States), McGill University (Canada), McGill University Health Centre (Canada), University of Ottawa. The BCG World Atlas 3rd Edition 2020. Canada: McGill University (Canada), 2020.	BCG	GHDx	Removal
467070	Israel	The BCG World Atlas 3rd Edition 2020	Aeras (United States), McGill University (Canada), McGill University Health Centre (Canada), University of Ottawa. The BCG World Atlas 3rd Edition 2020. Canada: McGill University (Canada), 2020.	BCG	GHDx	Removal
467070	Italy	The BCG World Atlas 3rd Edition 2020	Aeras (United States), McGill University (Canada), McGill University Health Centre (Canada), University of Ottawa. The BCG World Atlas 3rd Edition 2020. Canada: McGill University (Canada), 2020.	BCG	GHDx	Removal
467070	Lebanon	The BCG World Atlas 3rd Edition 2020	Aeras (United States), McGill University (Canada), McGill University Health Centre (Canada), University of Ottawa. The BCG World Atlas 3rd Edition 2020. Canada: McGill University (Canada), 2020.	BCG	GHDx	Removal
467070	Luxembourg	The BCG World Atlas 3rd Edition 2020	Aeras (United States), McGill University (Canada), McGill University Health Centre (Canada), University of Ottawa. The BCG World Atlas 3rd Edition 2020. Canada: McGill University (Canada), 2020.	BCG	GHDx	Removal
467070	Netherlands	The BCG World Atlas 3rd Edition 2020	Aeras (United States), McGill University (Canada), McGill University Health Centre (Canada), University of Ottawa. The BCG World Atlas 3rd Edition 2020. Canada: McGill University (Canada), 2020.	BCG	GHDx	Removal
467070	New Zealand	The BCG World Atlas 3rd Edition 2020	Aeras (United States), McGill University (Canada), McGill University Health Centre (Canada), University of Ottawa. The BCG World Atlas 3rd Edition 2020. Canada: McGill University (Canada), 2020.	BCG	GHDx	Removal
468024	Northern Mariana Islands	Expanded Programme on Immunization (EPI) Immunization schedules in the WHO Western Pacific Region, 1995	World Health Organization. Expanded Programme on Immunization (EPI) Immunization schedules in the WHO Western Pacific Region, 1995. WHO Wkly Epidemiol Rec. 1996; 71(18): 133-137.	BCG	GHDx	Removal
467070	Norway	The BCG World Atlas 3rd Edition 2020	Aeras (United States), McGill University (Canada), McGill University Health Centre (Canada), University of Ottawa. The BCG World Atlas 3rd Edition 2020. Canada: McGill University (Canada), 2020.	BCG	GHDx	Removal

GHDx id	Country	Series	Citation	Vaccine	Link	Ancillary Source Type
467070	Slovakia	The BCG World Atlas 3rd Edition 2020	Aeras (United States), McGill University (Canada), McGill University Health Centre (Canada), University of Ottawa. The BCG World Atlas 3rd Edition 2020. Canada: McGill University (Canada), 2020.	BCG	GHDx	Removal
467070	Slovenia	The BCG World Atlas 3rd Edition 2020	Aeras (United States), McGill University (Canada), McGill University Health Centre (Canada), University of Ottawa. The BCG World Atlas 3rd Edition 2020. Canada: McGill University (Canada), 2020.	BCG	GHDx	Removal
467070	Spain	The BCG World Atlas 3rd Edition 2020	Aeras (United States), McGill University (Canada), McGill University Health Centre (Canada), University of Ottawa. The BCG World Atlas 3rd Edition 2020. Canada: McGill University (Canada), 2020.	BCG	GHDx	Removal
467070	Sweden	The BCG World Atlas 3rd Edition 2020	Aeras (United States), McGill University (Canada), McGill University Health Centre (Canada), University of Ottawa. The BCG World Atlas 3rd Edition 2020. Canada: McGill University (Canada), 2020.	BCG	GHDx	Removal
467070	Switzerland	The BCG World Atlas 3rd Edition 2020	Aeras (United States), McGill University (Canada), McGill University Health Centre (Canada), University of Ottawa. The BCG World Atlas 3rd Edition 2020. Canada: McGill University (Canada), 2020.	BCG	GHDx	Removal
468048	Trinidad and Tobago	No Risk to Children From Halt of BCG Vaccine	No Risk to Children From Halt of BCG Vaccine.	BCG	GHDx	Removal
467070	UK	The BCG World Atlas 3rd Edition 2020	Aeras (United States), McGill University (Canada), McGill University Health Centre (Canada), University of Ottawa. The BCG World Atlas 3rd Edition 2020. Canada: McGill University (Canada), 2020.	BCG	GHDx	Removal
467070	USA	The BCG World Atlas 3rd Edition 2020	Aeras (United States), McGill University (Canada), McGill University Health Centre (Canada), University of Ottawa. The BCG World Atlas 3rd Edition 2020. Canada: McGill University (Canada), 2020.	BCG	GHDx	Removal
468049	Virgin Islands	United States Immunization Against Infectious Disease 1968	United States Immunization Against Infectious Disease 1968.	BCG	GHDx	Removal
467303	Bahrain	Bahrain WHO and UNICEF Estimates of Immunization Coverage 2019 Revision	United Nations Children's Fund (UNICEF), World Health Organization (WHO). Bahrain WHO and UNICEF Estimates of Immunization Coverage 2019 Revision. Geneva, Switzerland: World Health Organization (WHO), 2020.	RotaC	GHDx	Disruption
467304	Brazil	Brazil WHO and UNICEF Estimates of Immunization Coverage 2019 Revision	United Nations Children's Fund (UNICEF), World Health Organization (WHO). Brazil WHO and UNICEF Estimates of Immunization Coverage 2019 Revision. Geneva, Switzerland: World Health Organization (WHO), 2020.	RotaC	GHDx	Disruption
467306	Côte d'Ivoire	Cote d'Ivoire WHO and UNICEF Estimates of Immunization Coverage 2019 Revision	United Nations Children's Fund (UNICEF), World Health Organization (WHO). Cote d'Ivoire WHO and UNICEF Estimates of Immunization Coverage 2019 Revision. Geneva, Switzerland: World Health Organization (WHO), 2020.	BCG, MCV1, Pol3	GHDx	Disruption
467305	Central African Republic	Central African Republic WHO and UNICEF Estimates of Immunization Coverage 2019 Revision	United Nations Children's Fund (UNICEF), World Health Organization (WHO). Central African Republic WHO and UNICEF Estimates of Immunization Coverage 2019 Revision. Geneva, Switzerland: World Health Organization (WHO), 2020.	Pol3	GHDx	Disruption
467422	Chad	Chad WHO and UNICEF Estimates of Immunization Coverage 2019 Revision	United Nations Children's Fund (UNICEF), World Health Organization (WHO). Chad WHO and UNICEF Estimates of Immunization Coverage 2019 Revision. Geneva, Switzerland: World Health Organization (WHO), 2020.	MCV1	GHDx	Disruption
467372	Cook Islands	Cook Islands WHO and UNICEF Estimates of Immunization Coverage 2019 Revision	United Nations Children's Fund (UNICEF), World Health Organization (WHO). Cook Islands WHO and UNICEF Estimates of Immunization Coverage 2019 Revision. Geneva, Switzerland: World Health Organization (WHO), 2020.	MCV2	GHDx	Disruption
467374	Costa Rica	Costa Rica WHO and UNICEF Estimates of Immunization Coverage 2019 Revision	United Nations Children's Fund (UNICEF), World Health Organization (WHO). Costa Rica WHO and UNICEF Estimates of Immunization Coverage 2019 Revision. Geneva, Switzerland: World Health Organization (WHO), 2020.	PCV3, Pol3	GHDx	Disruption
467309	DR Congo	Republic of Congo WHO and UNICEF Estimates of Immunization Coverage 2019 Revision	United Nations Children's Fund (UNICEF), World Health Organization (WHO). Republic of Congo WHO and UNICEF Estimates of Immunization Coverage 2019 Revision. Geneva, Switzerland: World Health Organization (WHO), 2020.	BCG	GHDx	Disruption
467419	El Salvador	El Salvador WHO and UNICEF Estimates of Immunization Coverage 2019 Revision	United Nations Children's Fund (UNICEF), World Health Organization (WHO). El Salvador WHO and UNICEF Estimates of Immunization Coverage 2019 Revision. Geneva, Switzerland: World Health Organization (WHO), 2020.	RotaC	GHDx	Disruption
467408	Equatorial Guinea	Equatorial Guinea WHO and UNICEF Estimates of Immunization Coverage 2019 Revision	United Nations Children's Fund (UNICEF), World Health Organization (WHO). Equatorial Guinea WHO and UNICEF Estimates of Immunization Coverage 2019 Revision. Geneva, Switzerland: World Health Organization (WHO), 2020.	DTP3	GHDx	Disruption

GHDx id	Country	Series	Citation	Vaccine	Link	Ancillary Source Type
467375	Georgia	Georgia WHO and UNICEF Estimates of Immunization Coverage 2019 Revision	United Nations Children's Fund (UNICEF), World Health Organization (WHO). Georgia WHO and UNICEF Estimates of Immunization Coverage 2019 Revision. Geneva, Switzerland: World Health Organization (WHO), 2020.	HepB3	GHDx	Disruption
467409	Guatemala	Guatemala WHO and UNICEF Estimates of Immunization Coverage 2019 Revision	United Nations Children's Fund (UNICEF), World Health Organization (WHO). Guatemala WHO and UNICEF Estimates of Immunization Coverage 2019 Revision. Geneva, Switzerland: World Health Organization (WHO), 2020.	RotaC	GHDx	Disruption
467410	Indonesia	Indonesia WHO and UNICEF Estimates of Immunization Coverage 2019 Revision	United Nations Children's Fund (UNICEF), World Health Organization (WHO). Indonesia WHO and UNICEF Estimates of Immunization Coverage 2019 Revision. Geneva, Switzerland: World Health Organization (WHO), 2020.	MCV2	GHDx	Disruption
467072	Ireland	Ireland Dáil Éireann Parliamentary Questions - April 16, 2019	Houses of the Oireachtas (Ireland). Ireland Dáil Éireann Parliamentary Questions - April 16, 2019.	BCG	GHDx	Disruption
467071	Kiribati	Systems Strengthening for Effective Coverage of New Vaccines in the Pacific Project: Report and Recommendation of the President	Asian Development Bank (ADB). Systems Strengthening for Effective Coverage of New Vaccines in the Pacific Project: Report and Recommendation of the President. Manila, Philippines: Asian Development Bank (ADB), 2018.	BCG, PCV3, Pol3, MCV1	GHDx	Disruption
467412	Latvia	Latvia WHO and UNICEF Estimates of Immunization Coverage 2019 Revision	United Nations Children's Fund (UNICEF), World Health Organization (WHO). Latvia WHO and UNICEF Estimates of Immunization Coverage 2019 Revision. Geneva, Switzerland: World Health Organization (WHO), 2020.	PCV3	GHDx	Disruption
467037	Lebanon	Israel’s Second Lebanon War Remains a Resounding Failure	Haaretz. Israel’s Second Lebanon War Remains a Resounding Failure. Tel Aviv, Israel: Haaretz, 2016.	DTP1, DTP3, HepB3, Hib3, MCV1, MCV2, Pol3, RCV1	GHDx	Disruption
467411	Liberia	Liberia WHO and UNICEF Estimates of Immunization Coverage 2019 Revision	United Nations Children's Fund (UNICEF), World Health Organization (WHO). Liberia WHO and UNICEF Estimates of Immunization Coverage 2019 Revision. Geneva, Switzerland: World Health Organization (WHO), 2020.	BCG, DTP1, DTP3, HepB3, Hib3, MCV1, Pol3	GHDx	Disruption
467071	Marshall Islands	Systems Strengthening for Effective Coverage of New Vaccines in the Pacific Project: Report and Recommendation of the President	Asian Development Bank (ADB). Systems Strengthening for Effective Coverage of New Vaccines in the Pacific Project: Report and Recommendation of the President. Manila, Philippines: Asian Development Bank (ADB), 2018.	DTP1, DTP3	GHDx	Disruption
467414	Marshall Islands	Marshall Islands WHO and UNICEF Estimates of Immunization Coverage 2019 Revision	United Nations Children's Fund (UNICEF), World Health Organization (WHO). Marshall Islands WHO and UNICEF Estimates of Immunization Coverage 2019 Revision. Geneva, Switzerland: World Health Organization (WHO), 2020.	MCV1, MCV2, RotaC	GHDx	Disruption
467413	Mexico	Mexico WHO and UNICEF Estimates of Immunization Coverage 2019 Revision	United Nations Children's Fund (UNICEF), World Health Organization (WHO). Mexico WHO and UNICEF Estimates of Immunization Coverage 2019 Revision. Geneva, Switzerland: World Health Organization (WHO), 2020.	MCV2	GHDx	Disruption
467415	Myanmar	Myanmar WHO and UNICEF Estimates of Immunization Coverage 2019 Revision	United Nations Children's Fund (UNICEF), World Health Organization (WHO). Myanmar WHO and UNICEF Estimates of Immunization Coverage 2019 Revision. Geneva, Switzerland: World Health Organization (WHO), 2020.	HepB3	GHDx	Disruption
467416	Niger	Niger WHO and UNICEF Estimates of Immunization Coverage 2019 Revision	United Nations Children's Fund (UNICEF), World Health Organization (WHO). Niger WHO and UNICEF Estimates of Immunization Coverage 2019 Revision. Geneva, Switzerland: World Health Organization (WHO), 2020.	BCG, Pol3, Pol3	GHDx	Disruption
467071	Niue	Systems Strengthening for Effective Coverage of New Vaccines in the Pacific Project: Report and Recommendation of the President	Asian Development Bank (ADB). Systems Strengthening for Effective Coverage of New Vaccines in the Pacific Project: Report and Recommendation of the President. Manila, Philippines: Asian Development Bank (ADB), 2018.	PCV3	GHDx	Disruption
467071	Papua New Guinea	Systems Strengthening for Effective Coverage of New Vaccines in the Pacific Project: Report and Recommendation of the President	Asian Development Bank (ADB). Systems Strengthening for Effective Coverage of New Vaccines in the Pacific Project: Report and Recommendation of the President. Manila, Philippines: Asian Development Bank (ADB), 2018.	BCG	GHDx	Disruption
467417	Peru	Peru WHO and UNICEF Estimates of Immunization Coverage 2019 Revision	United Nations Children's Fund (UNICEF), World Health Organization (WHO). Peru WHO and UNICEF Estimates of Immunization Coverage 2019 Revision. Geneva, Switzerland: World Health Organization (WHO), 2020.	Pol3	GHDx	Disruption
467418	Philippines	Philippines WHO and UNICEF Estimates of Immunization Coverage 2019 Revision	United Nations Children's Fund (UNICEF), World Health Organization (WHO). Philippines WHO and UNICEF Estimates of Immunization Coverage 2019 Revision. Geneva, Switzerland: World Health Organization (WHO), 2020.	DTP1, DTP3, HepB3, Hib3	GHDx	Disruption

GHDx id	Country	Series	Citation	Vaccine	Link	Ancillary Source Type
467425	Saint Vincent and the Grenadines	Saint Vincent and the Grenadines WHO and UNICEF Estimates of Immunization Coverage 2019 Revision	United Nations Children's Fund (UNICEF), World Health Organization (WHO). Saint Vincent and the Grenadines WHO and UNICEF Estimates of Immunization Coverage 2019 Revision. Geneva, Switzerland: World Health Organization (WHO), 2020.	BCG	GHDx	Disruption
467039	Samoa	Samoa measles outbreak: WHO blames anti-vaccine scare as death toll hits 39	Samoa measles outbreak: WHO blames anti-vaccine scare as death toll hits 39. The Guardian [Internet]. 2019 Nov 27; UK.	MCV1, MCV2, DTP1, DTP3, HepB3, Hib3, RCV1	GHDx	Disruption
467426	Samoa	Samoa WHO and UNICEF Estimates of Immunization Coverage 2019 Revision	United Nations Children's Fund (UNICEF), World Health Organization (WHO). Samoa WHO and UNICEF Estimates of Immunization Coverage 2019 Revision. Geneva, Switzerland: World Health Organization (WHO), 2020.	BCG, DTP1, MCV2	GHDx	Disruption
467421	Syria	Syrian Arab Republic WHO and UNICEF Estimates of Immunization Coverage 2019 Revision	United Nations Children's Fund (UNICEF), World Health Organization (WHO). Syrian Arab Republic WHO and UNICEF Estimates of Immunization Coverage 2019 Revision. Geneva, Switzerland: World Health Organization (WHO), 2020.	DTP3, HepB3, Hib3, MCV1, MCV2, RCV1	GHDx	Disruption
467423	Togo	Togo WHO and UNICEF Estimates of Immunization Coverage 2019 Revision	United Nations Children's Fund (UNICEF), World Health Organization (WHO). Togo WHO and UNICEF Estimates of Immunization Coverage 2019 Revision. Geneva, Switzerland: World Health Organization (WHO), 2020.	BCG	GHDx	Disruption
467424	Ukraine	Ukraine WHO and UNICEF Estimates of Immunization Coverage 2019 Revision	United Nations Children's Fund (UNICEF), World Health Organization (WHO). Ukraine WHO and UNICEF Estimates of Immunization Coverage 2019 Revision. Geneva, Switzerland: World Health Organization (WHO), 2020.	BCG, DTP3, Pol3, MCV1, MCV2	GHDx	Disruption
467073	Vietnam	Safety of Quinvaxem (DTwP-HepB-Hib) pentavalent vaccine	World Health Organization (WHO). Safety of Quinvaxem (DTwP-HepB-Hib) pentavalent vaccine. Geneva, Switzerland: World Health Organization (WHO).	DTP3	GHDx	Disruption

Supplementary table 4. ST-GPR covariate weights. Tables a-c describe the covariates used in each ST-GPR linear model component. Asterisks denote covariate significance (p-value) in each model. ST-GPR=spatiotemporal Gaussian process regression. BCG=Bacillus Calmette-Guérin. DTP1=diphtheria-tetanus-pertussis, first dose. DTP3=diphtheria-tetanus-pertussis, third dose. HepB3=hepatitis B vaccine, third dose. Hib3=*Haemophilus influenzae* type b vaccine, third dose. MCV1=measles-containing vaccine, first dose. MCV2=measles-containing vaccine, second dose. PCV3=pneumococcal conjugate vaccine, third dose. Pol3=polio vaccine, third dose. RCV1=rubella-containing vaccine, first dose. RotaC=completed rotavirus series. GBD=Global Burden of Disease.

a. Direct vaccines

Covariate Name	Covariate Description	DTP3	MCV1	Polio3	BCG	DTP1,2 conditional probability
Healthcare Access and Quality (HAQ) Index	Index indicator of health-care performance based on causes considered amenable to health-care and scaled to a 0-100 score per GBD location-year.	0.087***	0.104***	0.081***	0.095***	-0.020***
Mortality rate due to war and terrorism	Composite measure of acute war and terrorism events produced for GBD, applied in log space.	-0.314***	-0.264***	-0.248***	-0.266***	-0.305***
Vaccine stockouts and delivery disruptions (Stockouts)	Custom-calculated, scaled capture of country-reported interruptions in vaccine delivery. Details of construction and application described in supplementary section 3.7.	-3.72***	-3.93***	-3.84***	-3.85***	-1.91**

b. Ratio vaccines

Covariate Name	Covariate Description	Hib3-DTP3 ratio	HepB3-DTP3 ratio	PCV3-DTP3 ratio	RotaC-DTP3 ratio	MCV2-MCV1 ratio	RCV1-MCV1 ratio
HAQ	As described above.	0.032*	0.031*	0.008	0.003	0.019	0.012
Stockouts	As described above.	0.256	-5.96***	-4.14*	-9.46***	-4.70**	-1.13
Years since introduction	Count of years since vaccine addition to national schedule.	0.129*	0.403***	0.354***	0.412***	0.184	0.062
DTP3 coverage proportion	Proportion of target population with third-dose diphtheria-tetanus-pertussis vaccination.	0.326	4.86***	n/a	n/a	n/a	n/a
DTP3 coverage proportion * Years since introduction	Interaction between DTP3 vaccine coverage and years since vaccine introduction.	-0.034	-0.305***	n/a	n/a	n/a	n/a
MCV1 coverage proportion	Proportion of target population with first-dose measles vaccination.	n/a	n/a	n/a	n/a	4.33	-0.401
MCV1 coverage proportion * Years since introduction	Interaction between MCV1 vaccine coverage and years since vaccine introduction.	n/a	n/a	n/a	n/a	-0.162	-0.022

c. DTP1 administrative data imputation

Covariate Name	Covariate Description	DTP1-3 absolute dropout
Socio-demographic Index (SDI)	Composite indicator combining estimates of average educational attainment, total fertility rates among women under 25 years of age, and lag-distributed income.	0.149***

*p-value < 0.01; **p-value < 1e-5; ***p-value < 1e-10

Supplementary table 5. ST-GPR hyperparameters. Descriptions and values of each ST-GPR hyperparameter are provided for each ST-GPR model. Parameter values are included separately by location data density as applicable. ST-GPR=spatiotemporal Gaussian process regression. BCG=Bacillus Calmette-Guérin. DTP1=diphtheria-tetanus-pertussis, first dose. DTP3=diphtheria-tetanus-pertussis, third dose. HepB3=hepatitis B vaccine, third dose. Hib3=*Haemophilus influenzae* type b vaccine, third dose. MCV1=measles-containing vaccine, first dose. MCV2=measles-containing vaccine, second dose. PCV3=pneumococcal conjugate vaccine, third dose. Pol3=polio vaccine, third dose. RCV1=rubella-containing vaccine, first dose. RotaC=completed rotavirus series.

Parameter name	Parameter description	Model	Data density	Parameter value
st_lambda	Controls the amount of smoothing over time in the spacetime model fit. The common range of *st_lambda* is 0.05 to 0.5. A lower lambda indicates that more strength is being borrowed from points further apart in time (more smoothing) than a higher lambda.	DTP1,3 drop-out	No data	0.5
			More than 0	
		Administrative Bias Models (BCG, DTP3, MCV1, Pol3)	No data	0.5
			More than 0	
		Direct Vaccine Coverage (BCG, DTP1,2 conditional, DTP3, MCV1, Pol3)	No data	0.35
			1 to 4 data	0.4
			More than 4	0.5
		Ratios of Vaccine Coverage (HepB3, Hib3, MCV2, PCV3, RCV1, RotaC)	No data	0.35
			1 to 4 data	0.4
			More than 4	0.75
st_zeta	Controls the amount of smoothing over space in the spacetime model fit. The common range of *st_zeta* is 0.001 to 0.1. A lower zeta indicates that less strength is being borrowed from points further away (less smoothing) than a higher zeta.	DTP1,3 drop-out	No data	0.01
			More than 0	0
		Administrative Bias Models (BCG, DTP3, MCV1, Pol3)	No data	0.1
			More than 0	0
		Direct Vaccine Coverage (BCG, DTP1,2 conditional, DTP3, MCV1, Pol3)	No data	0.01
			1 to 4 data	1E-11
			More than 4	0
		Ratios of Vaccine Coverage (HepB3, Hib3, MCV2, PCV3, RCV1, RotaC)	No data	0.01
			1 to 4 data	1E-11
			More than 4	0
gpr_scale	Controls how correlated estimates are over time in the GPR model fit. The common range of *gpr_scale* is 5 to 20. A lower scale indicates a smaller distance over which estimates are correlated compared to a higher scale.	DTP1,3 drop-out	No data	10
			More than 0	
		Administrative Bias Models (BCG, DTP3, MCV1, Pol3)	No data	10
			More than 0	
		Direct Vaccine Coverage (BCG, DTP1,2 conditional, DTP3, Pol3)	No data	20
			1 to 4 data	
			More than 4	
		Direct Vaccine Coverage (MCV1)	No data	10
			1 to 4 data	
			More than 4	
		Ratios of Vaccine Coverage (HepB3, Hib3, MCV2, PCV3, RCV1, RotaC)	No data	10
			1 to 4 data	
			More than 4	
gpr_amp_cutoff	Controls that only locations at or above a certain data density are included in the amplitude calculation, which is the factor explaining how far the GPR estimates deviate from the spacetime estimates. The common range of gpr_amp_cutoff is 5 to 20. A lower cutoff indicates that locations with fewer data can still inform the amplitude compared to a higher cutoff.	DTP1,3 drop-out	No data	5
			More than 0	
		Administrative Bias Models (BCG, DTP3, MCV1, Pol3)	No data	5
			More than 0	
		Direct Vaccine Coverage (BCG, DTP1,2 conditional, DTP3, MCV1, Pol3)	No data	10
			1 to 4 data	
			More than 4	
		Ratios of Vaccine Coverage (HepB3, Hib3, MCV2, PCV3, RCV1, RotaC)	No data	10
			1 to 4 data	
			More than 4	

Supplementary table 6. ST-GPR administrative bias excluded ratios. Citations, series, country, years, and vaccines provided for each excluded survey-to-country-reported bias ratio along with rationale for their exclusion. ST-GPR=spatio-temporal Gaussian process regression. BCG=Bacillus Calmette-Guérin. DTP3=diphtheria-tetanus-pertussis, third dose. MCV1=measles-containing vaccine, first dose. Pol3=polio vaccine, third dose.

Country	Series	Year(s)	Cohort	Vaccine	Citation	Rationale for exclusion
Angola	Angola Multiple Indicator Cluster Survey 2001	2001	select	Pol3	National Institute of Statistics (Angola), United Nations Children's Fund (UNICEF). Angola Multiple Indicator Cluster Survey 2001. New York, United States of America: United Nations Children's Fund (UNICEF).	Survey-to-administrative data bias adjustment ratios greater than 2 considered implausible.
Bolivia	Bolivia Demographic and Health Survey 1989	1989	all	BCG1	Macro Systems, Inc.; Institute for Resource Development, National Institute of Statistics (Bolivia). Bolivia Demographic and Health Survey 1989. Fairfax, United States of America: ICF International.	Survey-to-administrative data bias adjustment ratios greater than 2 considered implausible.
Bolivia	Bolivia National Immunization Coverage Survey 1990	1990	all	BCG1	Bolivia National Immunization Coverage Survey 1990.	Survey-to-administrative data bias adjustment ratios greater than 2 considered implausible.
Brazil	Brazil Demographic and Health Survey 1996	1996	select	MCV1	Brazilian Society for Family Welfare (BEMFAM), Macro International, Inc. Brazil Demographic and Health Survey 1996. Fairfax, United States of America: ICF International.	Survey-to-administrative data bias adjustment ratios greater than 2 considered implausible.
Burkina Faso	Burkina Faso Demographic and Health Survey 1992-1993	1992-1993	select	DPT3, Pol3	Macro International, Inc, National Institute of Statistics and Demography (Burkina Faso). Burkina Faso Demographic and Health Survey 1992-1993. Fairfax, United States of America: ICF International.	Survey-to-administrative data bias adjustment ratios greater than 2 considered implausible.
Burkina Faso	Burkina Faso Demographic and Health Survey 2003	2003	select	MCV1	Macro International, Inc, National Institute of Statistics and Demography (Burkina Faso). Burkina Faso Demographic and Health Survey 2003. Fairfax, United States of America: ICF International.	Survey-to-administrative data bias adjustment ratios greater than 2 considered implausible.
Cameroon	Cameroon Demographic and Health Survey 1991	1991	select	BCG1, DPT3, MCV1, Pol3	Macro International, Inc, Ministry of Economy, Planning and Regional Development (Cameroon). Cameroon Demographic and Health Survey 1991. Fairfax, United States of America: ICF International.	Survey-to-administrative data bias adjustment ratios greater than 2 considered implausible.
Cameroon	Cameroon Immunization Coverage Survey November-December 1990	1987-1990	all	BCG1, DPT3	Cameroon Immunization Coverage Survey November-December 1990.	Survey-to-administrative data bias adjustment ratios greater than 2 considered implausible.
Central African Republic	Central African Republic Multiple Indicator Cluster Survey 2006	2006	select	Pol3	United Nations Children's Fund (UNICEF). Central African Republic Multiple Indicator Cluster Survey 2006. New York, United States: United Nations Children's Fund (UNICEF).	Survey-to-administrative data bias adjustment ratios greater than 2 considered implausible.
Chad	Chad Multiple Indicator Cluster Survey 2000	2000	select	Pol3	United Nations Children's Fund (UNICEF), Census Bureau (Chad), National Institute of Statistical, Economic and Demographic Studies (Chad). Chad Multiple Indicator Cluster Survey 2000. New York, United States: United Nations Children's Fund (UNICEF).	Survey-to-administrative data bias adjustment ratios greater than 2 considered implausible.
Chad	Chad Demographic and Health Survey 2004	2004	select	Pol3	Macro International, Inc, National Institute for Statistics, Economic and Demographic Studies (INSEED) (Chad). Chad Demographic and Health Survey 2004. Fairfax, United States of America: ICF International.	Survey-to-administrative data bias adjustment ratios greater than 2 considered implausible.
Colombia	Colombia Demographic and Health Survey 1986	1986	select	DPT3, MCV1, Pol3	Ministry of Health (Colombia), Regional Population Center (Colombia), Westinghouse; Institute for Resource Development. Colombia Demographic and Health Survey 1986. Fairfax, United States: ICF International, 1988.	Survey-to-administrative data bias adjustment ratios greater than 2 considered implausible.
Colombia	Colombia Demographic and Health Survey 1990	1990	select	MCV1	Institute for Resource Development, Macro Systems, Profamilia (Colombia). Colombia Demographic and Health Survey 1990. Fairfax, United States: ICF International, 1991.	Survey-to-administrative data bias adjustment ratios greater than 2 considered implausible.
DR Congo	Democratic Republic of the Congo Multiple Indicator Cluster Survey 2001	2001	select	MCV1	Ministry of Planning and Reconstruction (Congo, DR), United Nations Children's Fund (UNICEF). Congo, DR Multiple Indicator Cluster Survey 2001. New York, United States: United Nations Children's Fund (UNICEF).	Survey-to-administrative data bias adjustment ratios greater than 2 considered implausible.
Dominican Republic	Dominican Republic Demographic and Health Survey 1986	1986	select	DPT3, Pol3	National Council for Population and Family (Dominican Republic), Westinghouse; Institute for Resource Development. Dominican Republic Demographic and Health Survey 1986. Fairfax, United States: ICF International.	Survey-to-administrative data bias adjustment ratios greater than 2 considered implausible.

Country	Series	Year(s)	Cohort	Vaccine	Citation	Rationale for exclusion
Dominican Republic	Dominican Republic Demographic and Health Survey 1991	1991	select	MCV1	Institute for Resource Development, Macro Systems, National Planning Office (Dominican Republic), Profamilia (Colombia). Dominican Republic Demographic and Health Survey 1991. Fairfax, United States of America: ICF International.	Survey-to-administrative data bias adjustment ratios greater than 2 considered implausible.
Equatorial Guinea	Equatorial Guinea Multiple Indicator Cluster Survey 2000	2000	select	MCV1	Ministry of Planning, Economic Development and Public Investment (Equatorial Guinea), United Nations Children's Fund (UNICEF). Equatorial Guinea Multiple Indicator Cluster Survey 2000. New York, United States of America: United Nations Children's Fund (UNICEF).	Survey-to-administrative data bias adjustment ratios greater than 2 considered implausible.
Eritrea	Eritrea Demographic and Health Survey 1995-1996	1995-1996	select	MCV1	Macro International, Inc, National Statistics Office (Eritrea). Eritrea Demographic and Health Survey 1995-1996. Fairfax, United States of America: ICF International.	Survey-to-administrative data bias adjustment ratios greater than 2 considered implausible.
Ethiopia	Ethiopia Welfare Monitoring Survey 1996	1996	select	DPT3	Central Statistical Agency (Ethiopia). Ethiopia Welfare Monitoring Survey 1996.	Survey-to-administrative data bias adjustment ratios greater than 2 considered implausible.
Ethiopia	Ethiopia Multiple Indicator Cluster Survey 1995	1995	select	DPT3, Pol3	Ministry of Health (Ethiopia), United Nations Children's Fund (UNICEF). Ethiopia Multiple Indicator Cluster Survey 1995.	Survey-to-administrative data bias adjustment ratios greater than 2 considered implausible.
Gabon	Gabon Demographic and Health Survey 2000-2001	2000-2001	select	MCV1	General Directorate of Statistics and Economic Studies (Gabon), Macro International, Inc. Gabon Demographic and Health Survey 2000-2001. Fairfax, United States of America: ICF International.	Survey-to-administrative data bias adjustment ratios greater than 2 considered implausible.
Ghana	Ghana Demographic and Health Survey 1988	1988	select	DPT3, MCV1, Pol3	Ghana Statistical Service, Macro Systems, Inc.; Institute for Resource Development. Ghana Demographic and Health Survey 1988. Fairfax, United States of America: ICF International.	Survey-to-administrative data bias adjustment ratios greater than 2 considered implausible.
Guatemala	Guatemala Demographic and Health Survey 1987	1987	select	BCG1, DPT3, Pol3	Institute of Nutrition of Central America and Panama, Westinghouse; Institute for Resource Development. Guatemala Demographic and Health Survey 1987. Fairfax, United States: ICF International.	Survey-to-administrative data bias adjustment ratios greater than 2 considered implausible.
Guinea-Bissau	Guinea-Bissau Multiple Indicator Cluster Survey 2006	2006	select	MCV1	United Nations Children's Fund (UNICEF), Government of Guinea-Bissau. Guinea-Bissau Multiple Indicator Cluster Survey 2006. New York, United States: United Nations Children's Fund (UNICEF).	Survey-to-administrative data bias adjustment ratio considered implausible.
Haiti	Haiti Demographic and Health Survey 1994-1995	1994-1995	select	MCV1	Haitian Institute of Childhood (IHE), Macro International, Inc. Haiti Demographic and Health Survey 1994-1995. Calverton, United States of America: Macro International, Inc.	Survey-to-administrative data bias adjustment ratios greater than 2 considered implausible.
Haiti	Haiti Demographic and Health Survey 2000	2000	select	MCV1	Haitian Institute of Childhood (IHE), Macro International, Inc. Haiti Demographic and Health Survey 2000. Fairfax, United States of America: ICF International.	Survey-to-administrative data bias adjustment ratios greater than 2 considered implausible.
Haiti	Haiti Demographic and Health Survey 2005-2006	2005-2006	select	MCV1	Haitian Institute of Childhood (IHE), Haitian Institute of Statistics and Informatics, Macro International, Inc. Haiti Demographic and Health Survey 2005-2006. Fairfax, United States of America: ICF International.	Survey-to-administrative data bias adjustment ratios greater than 2 considered implausible.
Haiti	Haiti Global Fund Household Survey 2008	2008	select	BCG1	Global Fund to Fight Aids Tuberculosis and Malaria (GFATM). Haiti Global Fund Household Survey 2008.	Survey-to-administrative data bias adjustment ratios greater than 2 considered implausible.
Kenya	Kenya Multiple Indicator Cluster Survey 2000	2000	select	MCV1	Central Bureau of Statistics (Kenya), United Nations Children's Fund (UNICEF). Kenya Multiple Indicator Cluster Survey 2000. New York, United States: United Nations Children's Fund (UNICEF).	Survey-to-administrative data bias adjustment ratios greater than 2 considered implausible.
Kenya	Kenya Demographic and Health Survey 1993	1993	select	BCG1, DPT3, MCV1, Pol3	Central Bureau of Statistics (Kenya), Macro International, Inc, National Council for Population Development (NCPD). Kenya Demographic and Health Survey 1993. Fairfax, United States of America: ICF International.	Survey-to-administrative data bias adjustment ratios greater than 2 considered implausible.
Kenya	Kenya Demographic and Health Survey 1998	1998	select	BCG1, DPT3, MCV1, Pol3	Central Bureau of Statistics (Kenya), Macro International, Inc, National Council for Population Development (NCPD). Kenya Demographic and Health Survey 1998. Fairfax, United States of America: ICF International.	Survey-to-administrative data bias adjustment ratios greater than 2 considered implausible.
Kenya	Kenya EPI National Immunization Coverage Survey 1992	1992	all	DPT3, MCV1	Kenya EPI National Immunization Coverage Survey 1992.	Survey-to-administrative data bias adjustment ratios greater than 2 considered implausible.
Lesotho	Lesotho Multiple Indicator Cluster Survey 1996	1996	all	BCG1	Bureau of Statistics (Lesotho), Ministry of Health (Lesotho), United Nations Children's Fund (UNICEF). Lesotho Multiple Indicator Cluster Survey 1996.	Survey-to-administrative data bias adjustment ratios greater than 2 considered implausible.

Country	Series	Year(s)	Cohort	Vaccine	Citation	Rationale for exclusion
Liberia	Liberia Integrated Management of Childhhod Illness Household Baseline Survey 2000	2000	all	BCG1, DPT3, MCV1, Pol3	Liberia Integrated Management of Childhhod Illness Household Baseline Survey 2000.	Survey-to-administrative data bias adjustment ratios greater than 2 considered implausible.
Madagascar	Madagascar Demographic and Health Survey 2003-2004	2003-2004	select	MCV1	Macro International, Inc, National Institute of Statistics (Madagascar). Madagascar Demographic and Health Survey 2003-2004. Fairfax, United States of America: ICF International.	Survey-to-administrative data bias adjustment ratios greater than 2 considered implausible.
Mali	Mali Demographic and Health Survey 1987	1987	select	MCV1	Sahel Institute, Westinghouse; Institute for Resource Development. Mali Demographic and Health Survey 1987. Fairfax, United States of America: ICF International.	Survey-to-administrative data bias adjustment ratios greater than 2 considered implausible.
Marshall Islands	Marshall Islands Community Survey 2006	2006	all	BCG1, DPT3, MCV1, Pol3	Economic Policy, Planning and Statistics Office (Marshall Islands). Marshall Islands Community Survey 2006.	Survey-to-administrative data bias adjustment ratio considered implausible.
Mauritania	Mauritania Demographic and Health Survey 2000-2001	2000-2001	select	MCV1	Macro International, Inc, National Office of Statistics (Mauritania). Mauritania Demographic and Health Survey 2000-2001. Fairfax, United States of America: ICF International.	Survey-to-administrative data bias adjustment ratios greater than 2 considered implausible.
Mexico	Mexico Demographic and Health Survey 1987	1987	select	BCG1, MCV1	Macro Systems, Inc.; Institute for Resource Development, Secretariat of Health (Mexico). Mexico Demographic and Health Survey 1987. Fairfax, United States: ICF International.	Survey-to-administrative data bias adjustment ratios greater than 2 considered implausible.
Niger	Niger Demographic and Health Survey 1992	1992	select	DPT3, MCV1, Pol3	Department of Statistics and National Accounts (Niger), Macro International, Inc. Niger Demographic and Health Survey 1992. Fairfax, United States of America: ICF International.	Survey-to-administrative data bias adjustment ratios greater than 2 considered implausible.
Nigeria	Nigeria Demographic and Health Survey 1990	1990	select	MCV1	Federal Office of Statistics (Nigeria), Institute for Resource Development, Macro Systems. Nigeria Demographic and Health Survey 1990. Fairfax, United States of America: ICF International.	Survey-to-administrative data bias adjustment ratios greater than 2 considered implausible.
Nigeria	Nigeria - Ondo Special Demographic and Health Survey 1986-1987	1986-1987	select	BCG1, DPT3, MCV1, Pol3	Federal Ministry of Health (Nigeria), Macro Systems, Inc.; Institute for Resource Development, National Population Bureau (Nigeria). Nigeria - Ondo Special Demographic and Health Survey 1986-1987. Fairfax, United States of America: ICF International.	Survey-to-administrative data bias adjustment ratios greater than 2 considered implausible.
Philippines	Philippines Demographic and Health Survey 2003	2003	select	BCG1, DPT3, MCV1	Macro International, Inc, National Statistics Office (Philippines). Philippines Demographic and Health Survey 2003. Fairfax, United States of America: ICF International.	Survey-to-administrative data bias adjustment ratios greater than 2 considered implausible.
Philippines	Philippines Demographic and Health Survey 2008	2008	select	BCG1, DPT3, MCV1, Pol3	Macro International, Inc, National Statistics Office (Philippines). Philippines Demographic and Health Survey 2008. Fairfax, United States of America: ICF International, 2010.	Survey-to-administrative data bias adjustment ratios greater than 2 considered implausible.
Philippines	Philippines Demographic and Health Survey 2017	2017	select	Pol3, DPT3, BCG1	ICF International, Philippines Statistics Authority, United States Agency for International Development (USAID). Philippines Demographic and Health Survey 2017. Fairfax, United States of America: ICF International, 2018.	Survey-to-administrative data bias adjustment ratios greater than 2 considered implausible.
Sierra Leone	Sierra Leone Multiple Indicator Cluster Survey 2000	2000	select	MCV1	Central Statistics Office (Sierra Leone), United Nations Children's Fund (UNICEF). Sierra Leone Multiple Indicator Cluster Survey 2000. New York, United States of America: United Nations Children's Fund (UNICEF).	Survey-to-administrative data bias adjustment ratios greater than 2 considered implausible.
Somalia	Somalia Family Health Survey 1983	1983	all	BCG1, DPT3, Pol3	Ministry of Health (Somalia), Ministry of National Planning (Somalia), Westinghouse Health Systems, Inc.. Somalia Family Health Survey 1983.	Survey-to-administrative data bias adjustment ratios greater than 2 considered implausible.
Sri Lanka	Sri Lanka Demographic and Health Survey 1987	1987	select	MCV1	Department of Census and Statistics (Sri Lanka), Westinghouse; Institute for Resource Development. Sri Lanka Demographic and Health Survey 1987. Fairfax, United States of America: ICF International.	Survey-to-administrative data bias adjustment ratios greater than 2 considered implausible.
Syria	Syria Immunization Campaign 1986	1986	all	DPT3, MCV1, Pol3	Syria Immunization Campaign 1986.	Survey-to-administrative data bias adjustment ratios greater than 2 considered implausible.

Country	Series	Year(s)	Cohort	Vaccine	Citation	Rationale for exclusion
Thailand	Thailand Demographic and Health Survey 1987	1987	select	MCV1	Chulalongkorn University, Institute of Population Studies (Thailand), Westinghouse; Institute for Resource Development. Thailand Demographic and Health Survey 1987. Fairfax, United States: ICF International.	Survey-to-administrative data bias adjustment ratios greater than 2 considered implausible.
Togo	Togo Multiple Indicator Cluster Survey 2000	2000	select	BCG1, DPT3, MCV1, Pol3	United Nations Children's Fund (UNICEF). Togo Multiple Indicator Cluster Survey 2000. New York, United States of America: United Nations Children's Fund (UNICEF).	Survey-to-administrative data bias adjustment ratio considered implausible.
Togo	Togo Multiple Indicator Cluster Survey 2000	2000	select	BCG1, DPT3, MCV1, Pol3	United Nations Children's Fund (UNICEF). Togo Multiple Indicator Cluster Survey 2000. New York, United States of America: United Nations Children's Fund (UNICEF).	Survey-to-administrative data bias adjustment ratios greater than 2 considered implausible.
Togo	Togo Demographic and Health Survey 1998	1998	select	BCG1, DPT3, MCV1, Pol3	Department of Statistics (Togo), Macro International, Inc. Togo Demographic and Health Survey 1998. Fairfax, United States of America: ICF International.	Survey-to-administrative data bias adjustment ratio considered implausible.
Togo	Togo Demographic and Health Survey 1998	1998	select	BCG1, DPT3, MCV1, Pol3	Department of Statistics (Togo), Macro International, Inc. Togo Demographic and Health Survey 1998. Fairfax, United States of America: ICF International.	Survey-to-administrative data bias adjustment ratios greater than 2 considered implausible.
Turkey	Turkey Immunisation Coverage Survey 1989	1989	all	BCG1	Turkey Immunisation Coverage Survey 1989.	Survey-to-administrative data bias adjustment ratios greater than 2 considered implausible.
Turkey	Turkey National EPI Program Review 1988	1988	all	BCG1	Turkey National EPI Program Review 1988.	Survey-to-administrative data bias adjustment ratios greater than 2 considered implausible.
Uganda	Uganda Demographic and Health Survey 1988-1989	1988-1989	select	DPT3, MCV1, Pol3	Macro Systems, Inc.; Institute for Resource Development, Makerere University, Ministry of Health (Uganda). Uganda Demographic and Health Survey 1988-1989. Fairfax, United States of America: ICF International.	Survey-to-administrative data bias adjustment ratios greater than 2 considered implausible.
Vanuatu	Vanuatu Multiple Indicator Cluster Survey 2007-2008	2007-2008	select	BCG1, DPT3, Pol3	Ministry of Health (Vanuatu), United Nations Children's Fund (UNICEF). Vanuatu Multiple Indicator Cluster Survey 2007-2008. New York, United States of America: United Nations Children's Fund (UNICEF).	Survey-to-administrative data bias adjustment ratio considered implausible.

Supplementary table 7. National vaccination coverage in select years, by location and vaccine. Estimated coverage proportions and 95% uncertainty intervals for each country and vaccine in select years are provided. Vaccines not included in the national schedule in each respective year are indicated with a dash. BCG=Bacillus Calmette-Guérin. DTP1=diphtheria-tetanus-pertussis, first dose. DTP3=diphtheria-tetanus-pertussis, third dose. HepB3=hepatitis B vaccine, third dose. Hib3=*Haemophilus influenzae* type b vaccine, third dose. MCV1=measles-containing vaccine, first dose. MCV2=measles-containing vaccine, second dose. PCV3=pneumococcal conjugate vaccine, third dose. Pol3=polio vaccine, third dose. RCV1=rubella-containing vaccine, first dose. RotaC=completed rotavirus series.

Country	Vaccine	1980	1990	2000	2010	2019
Afghanistan (AFG)	BCG	22.2% (14.2-31.6%)	34.5% (28.6-40.8%)	47.7% (42.3-53.1%)	65.7% (62.1-69.2%)	78.0% (72.2-83.3%)
	DTP1	30.0% (20.2-42.1%)	47.3% (39.9-55.3%)	46.6% (40.0-52.8%)	65.8% (61.5-70.1%)	84.9% (78.9-90.2%)
	DTP3	2.8% (1.1-5.8%)	10.9% (6.8-16.6%)	18.0% (13.9-22.1%)	40.6% (37.6-43.9%)	60.8% (54.4-67.5%)
	HepB3	-	-	-	40.4% (37.3-43.6%)	60.7% (54.3-67.3%)
	Hib3	-	-	-	40.6% (37.5-43.8%)	60.7% (54.3-67.4%)
	MCV1	8.9% (4.4-15.1%)	24.0% (17.8-31.4%)	35.5% (29.1-42.1%)	56.1% (52.5-59.8%)	58.3% (48.9-67.1%)
	MCV2	-	-	-	38.2% (33.7-42.9%)	41.1% (33.2-49.4%)
	PCV3	-	-	-	-	59.4% (53.1-66.0%)
	Pol3	3.5% (1.6-6.3%)	13.6% (9.4-19.2%)	33.0% (28.4-37.8%)	54.6% (51.6-57.5%)	69.8% (63.0-75.7%)
	RCV1	-	-	-	-	-
	RotaC	-	-	-	-	49.6% (44.1-55.1%)
Angola (AGO)	BCG	28.0% (15.7-43.0%)	51.1% (45.3-57.3%)	70.2% (66.0-74.1%)	68.5% (63.9-72.8%)	81.0% (74.1-86.8%)
	DTP1	25.6% (12.2-47.5%)	38.0% (29.3-48.2%)	53.7% (47.1-60.5%)	82.0% (75.1-88.0%)	75.8% (64.1-86.0%)
	DTP3	6.9% (3.2-13.3%)	18.8% (14.8-23.3%)	29.6% (25.9-33.0%)	40.1% (36.1-44.2%)	40.3% (33.7-47.3%)
	HepB3	-	-	-	40.0% (36.0-44.2%)	40.3% (33.6-47.3%)
	Hib3	-	-	-	40.0% (36.0-44.2%)	40.3% (33.6-47.3%)
	MCV1	31.1% (13.2-53.8%)	36.1% (28.2-44.9%)	64.3% (57.6-70.8%)	74.5% (67.0-81.0%)	53.5% (42.7-63.8%)
	MCV2	-	-	-	-	31.2% (23.3-40.2%)
	PCV3	-	-	-	-	40.1% (33.5-47.1%)
	Pol3	16.4% (7.3-30.1%)	25.8% (19.4-33.1%)	57.4% (51.2-63.5%)	57.6% (51.9-64.1%)	41.0% (31.1-50.9%)
	RCV1	-	-	-	-	53.4% (42.6-63.7%)
	RotaC	-	-	-	-	40.0% (33.4-47.0%)
Albania (ALB)	BCG	93.1% (87.4-96.9%)	90.3% (86.8-93.2%)	94.1% (90.7-96.5%)	99.0% (98.0-99.6%)	99.7% (99.2-99.9%)
	DTP1	96.0% (92.6-98.2%)	93.3% (89.9-95.7%)	98.9% (97.9-99.5%)	99.6% (99.3-99.9%)	99.8% (99.6-100.0%)
	DTP3	94.5% (89.8-97.3%)	91.4% (87.7-94.2%)	98.5% (97.2-99.2%)	99.6% (99.1-99.8%)	99.8% (99.6-100.0%)
	HepB3	-	-	95.8% (91.9-98.0%)	99.4% (98.7-99.7%)	99.7% (99.2-99.9%)
	Hib3	-	-	-	99.5% (98.9-99.7%)	99.7% (99.4-99.9%)
	MCV1	93.6% (86.7-97.7%)	92.1% (87.5-95.6%)	95.0% (91.5-97.4%)	99.8% (99.4-100.0%)	98.9% (97.2-99.7%)
	MCV2	-	-	-	99.6% (99.2-99.8%)	98.8% (97.1-99.6%)
	PCV3	-	-	-	-	99.3% (99.0-99.5%)
	Pol3	91.4% (86.4-95.2%)	92.6% (89.6-94.9%)	98.3% (97.1-99.1%)	99.0% (98.1-99.6%)	99.8% (99.5-99.9%)
	RCV1	-	-	-	99.7% (99.3-99.9%)	98.8% (97.1-99.6%)
	RotaC	-	-	-	-	47.0% (44.0-50.1%)
Andorra (AND)	BCG	-	-	-	-	-
	DTP1	-	85.4% (72.9-93.6%)	95.9% (93.8-97.5%)	98.4% (97.2-99.1%)	98.8% (97.5-99.5%)
	DTP3	-	83.1% (69.5-92.0%)	94.8% (92.5-96.6%)	98.3% (97.1-99.0%)	98.7% (97.4-99.5%)
	HepB3	-	-	81.0% (75.1-86.3%)	95.9% (93.4-97.6%)	97.3% (94.8-98.8%)
	Hib3	-	-	83.2% (77.6-88.0%)	97.4% (95.9-98.4%)	98.3% (96.9-99.2%)
	MCV1	-	86.3% (64.6-96.6%)	96.4% (93.5-98.4%)	98.8% (97.6-99.6%)	99.6% (98.9-99.9%)
	MCV2	-	-	78.6% (67.0-87.8%)	84.8% (79.6-88.9%)	94.8% (91.4-97.1%)
	PCV3	-	-	-	78.6% (72.3-84.0%)	96.2% (94.6-97.4%)
	Pol3	-	85.5% (74.0-93.7%)	94.1% (91.4-96.2%)	98.8% (97.8-99.4%)	99.1% (98.1-99.7%)
	RCV1	-	86.1% (64.5-96.3%)	96.3% (93.4-98.2%)	98.7% (97.5-99.4%)	99.5% (98.8-99.8%)
	RotaC	-	-	-	-	-
	BCG	24.8% (18.1-32.5%)	98.2% (97.0-99.1%)	96.2% (91.8-98.6%)	96.5% (93.0-98.3%)	94.2% (88.5-97.6%)

Country	Vaccine	1980	1990	2000	2010	2019
United Arab Emirates (ARE)	DTP1	43.7% (33.9-55.1%)	94.6% (92.9-96.0%)	92.9% (90.7-94.8%)	90.9% (88.0-93.3%)	94.6% (91.8-96.8%)
	DTP3	34.6% (27.0-43.1%)	92.2% (90.0-94.0%)	88.6% (85.9-91.1%)	90.4% (87.3-92.8%)	94.5% (91.7-96.7%)
	HepB3	-	91.9% (89.7-93.7%)	86.6% (82.5-89.9%)	90.2% (87.2-92.6%)	93.9% (91.1-96.2%)
	Hib3	-	-	87.8% (84.9-90.5%)	90.3% (87.2-92.7%)	94.4% (91.5-96.5%)
	MCV1	42.4% (33.6-52.3%)	80.9% (75.9-85.2%)	88.4% (85.1-91.3%)	89.5% (85.3-93.1%)	93.5% (90.6-95.9%)
	MCV2	-	80.1% (75.1-84.4%)	87.2% (83.6-90.1%)	89.4% (85.2-92.9%)	93.5% (90.6-95.8%)
	PCV3	-	-	-	90.2% (87.1-92.7%)	94.4% (91.6-96.6%)
	Pol3	34.7% (27.1-43.0%)	92.5% (90.4-94.3%)	89.4% (86.5-91.8%)	91.9% (88.8-94.2%)	93.9% (90.5-96.4%)
	RCV1	-	79.6% (74.6-83.8%)	87.5% (84.2-90.4%)	89.4% (85.2-92.9%)	93.4% (90.5-95.8%)
	RotaC	-	-	-	-	86.6% (83.7-88.7%)
Argentina (ARG)	BCG	64.2% (55.9-71.9%)	98.4% (97.1-99.2%)	99.6% (98.9-99.9%)	99.5% (98.8-99.9%)	93.8% (87.8-97.5%)
	DTP1	55.8% (47.1-64.5%)	88.3% (85.2-91.0%)	91.8% (88.9-94.1%)	94.5% (92.5-96.3%)	94.0% (89.9-96.9%)
	DTP3	46.9% (39.1-54.5%)	80.6% (76.6-84.0%)	85.4% (81.3-88.9%)	91.8% (89.0-94.3%)	88.1% (82.2-92.8%)
	HepB3	-	-	53.2% (36.4-69.4%)	91.5% (88.7-94.0%)	87.8% (82.0-92.5%)
	Hib3	-	-	84.5% (80.4-87.9%)	91.6% (88.9-94.1%)	87.9% (82.1-92.5%)
	MCV1	65.9% (56.7-74.7%)	93.2% (89.2-96.1%)	93.5% (88.6-96.7%)	98.6% (97.0-99.4%)	93.4% (86.9-97.2%)
	MCV2	-	-	60.2% (51.6-68.8%)	93.4% (90.0-95.8%)	93.2% (86.6-97.0%)
	PCV3	-	-	-	-	87.6% (81.8-92.2%)
	Pol3	38.5% (30.0-46.2%)	85.4% (81.8-88.8%)	88.6% (85.0-91.7%)	92.7% (90.0-94.9%)	85.5% (78.9-90.8%)
	RCV1	-	-	93.3% (88.4-96.6%)	98.5% (96.8-99.3%)	93.3% (86.8-97.1%)
	RotaC	-	-	-	-	72.3% (67.3-76.5%)
Armenia (ARM)	BCG	88.9% (69.1-97.4%)	90.8% (84.5-95.3%)	93.8% (91.8-95.5%)	98.0% (96.5-98.9%)	98.7% (96.9-99.6%)
	DTP1	91.6% (80.8-97.8%)	90.1% (84.3-94.5%)	98.6% (97.9-99.2%)	97.7% (96.5-98.5%)	96.0% (93.6-98.0%)
	DTP3	85.5% (70.5-94.4%)	83.6% (76.7-89.3%)	97.0% (95.9-97.9%)	93.8% (91.7-95.6%)	92.0% (87.8-95.0%)
	HepB3	-	-	49.1% (32.5-64.6%)	92.8% (90.2-94.8%)	91.8% (87.5-94.7%)
	Hib3	-	-	-	92.8% (90.3-94.7%)	91.8% (87.7-94.8%)
	MCV1	90.3% (72.6-97.9%)	93.2% (88.3-96.3%)	88.5% (85.1-91.4%)	97.4% (95.7-98.5%)	93.0% (88.4-96.2%)
	MCV2	-	93.0% (88.1-96.1%)	88.3% (85.0-91.2%)	97.3% (95.6-98.4%)	92.9% (88.4-96.1%)
	PCV3	-	-	-	-	91.8% (87.7-94.8%)
	Pol3	84.2% (69.6-93.3%)	83.9% (79.2-87.8%)	95.4% (93.9-96.7%)	94.9% (93.0-96.5%)	93.2% (89.8-95.8%)
	RCV1	-	-	-	97.3% (95.6-98.4%)	92.9% (88.3-96.1%)
	RotaC	-	-	-	-	91.9% (87.8-94.9%)
American Samoa (ASM)	BCG	-	-	-	-	-
	DTP1	77.6% (53.8-93.1%)	96.7% (89.4-99.5%)	97.2% (92.1-99.5%)	96.6% (89.7-99.3%)	97.3% (91.9-99.6%)
	DTP3	71.1% (43.6-89.5%)	92.0% (79.7-97.8%)	94.7% (86.5-98.7%)	95.2% (86.8-98.9%)	95.7% (87.4-99.0%)
	HepB3	-	-	94.0% (85.5-98.1%)	94.9% (86.4-98.6%)	95.4% (87.3-98.8%)
	Hib3	-	-	-	-	-
	MCV1	72.7% (41.9-93.0%)	92.6% (77.8-98.7%)	96.4% (89.3-99.4%)	96.3% (87.9-99.3%)	96.9% (90.0-99.4%)
	MCV2	-	-	-	-	-
	PCV3	-	-	-	-	-
	Pol3	68.9% (42.6-89.0%)	90.0% (76.3-97.1%)	94.1% (85.2-98.5%)	94.9% (86.3-98.7%)	95.3% (87.8-98.9%)
	RCV1	-	-	-	-	-
	RotaC	-	-	-	-	-
Antigua and Barbuda (ATG)	BCG	-	-	-	-	-
	DTP1	67.7% (60.1-75.9%)	99.0% (98.3-99.5%)	97.0% (95.4-98.2%)	97.5% (96.0-98.5%)	98.2% (96.1-99.3%)
	DTP3	63.5% (55.6-71.3%)	96.7% (95.1-97.9%)	96.4% (94.6-97.7%)	97.4% (95.9-98.4%)	93.9% (90.2-96.5%)
	HepB3	-	-	95.6% (93.3-97.3%)	97.0% (95.3-98.1%)	93.7% (89.9-96.4%)
	Hib3	-	-	93.9% (91.3-95.9%)	96.7% (95.1-97.9%)	93.8% (90.1-96.3%)
	MCV1	47.8% (27.0-68.4%)	91.0% (86.4-94.5%)	97.6% (94.7-99.2%)	99.3% (98.1-99.8%)	93.4% (86.8-97.2%)
	MCV2	-	-	50.3% (34.3-65.8%)	87.7% (83.0-91.7%)	84.1% (76.8-89.8%)
	PCV3	-	-	-	-	-
	Pol3	45.1% (36.7-54.0%)	96.8% (95.2-98.0%)	96.4% (94.4-97.7%)	97.3% (95.9-98.3%)	93.8% (90.4-96.2%)
	RCV1	-	90.8% (86.3-94.3%)	97.5% (94.5-99.0%)	99.2% (98.0-99.7%)	93.3% (86.7-97.1%)
	RotaC	-	-	-	-	-

Country	Vaccine	1980	1990	2000	2010	2019
Australia (AUS)	BCG	-	-	-	-	-
	DTP1	41.6% (31.9-52.0%)	96.2% (94.1-97.7%)	89.0% (86.0-91.6%)	90.0% (87.3-92.5%)	93.2% (90.0-95.7%)
	DTP3	38.8% (29.2-49.4%)	95.1% (92.5-97.0%)	88.3% (85.1-91.0%)	89.9% (87.2-92.4%)	93.1% (89.7-95.7%)
	HepB3	-	-	88.1% (84.8-90.8%)	89.7% (87.1-92.2%)	93.0% (89.4-95.5%)
	Hib3	-	-	88.1% (84.9-90.8%)	89.7% (87.1-92.2%)	92.9% (89.5-95.4%)
	MCV1	70.5% (50.7-86.6%)	91.0% (85.0-95.5%)	93.6% (91.1-95.6%)	98.2% (95.4-99.4%)	99.5% (98.4-99.9%)
	MCV2	-	-	92.3% (89.6-94.5%)	90.8% (86.4-94.2%)	97.6% (95.6-98.8%)
	PCV3	-	-	-	89.1% (86.4-91.5%)	93.0% (89.5-95.6%)
	Pol3	25.1% (14.3-37.1%)	72.7% (62.7-81.0%)	90.2% (87.5-92.3%)	93.6% (90.7-95.9%)	96.3% (93.4-98.1%)
	RCV1	-	90.8% (84.8-95.2%)	93.5% (90.9-95.5%)	98.1% (95.3-99.3%)	99.4% (98.4-99.8%)
	RotaC	-	-	-	79.4% (76.7-81.8%)	91.5% (88.0-94.0%)
Austria (AUT)	BCG	89.2% (80.8-95.0%)	-	-	-	-
	DTP1	99.9% (99.7-100.0%)	99.9% (99.7-100.0%)	99.4% (98.7-99.7%)	93.2% (89.8-95.8%)	93.3% (89.5-96.2%)
	DTP3	89.3% (83.7-93.6%)	90.2% (87.0-92.7%)	82.1% (77.6-85.9%)	86.2% (81.4-90.2%)	89.0% (83.1-93.3%)
	HepB3	-	-	33.5% (22.1-47.2%)	86.0% (81.3-90.1%)	88.9% (83.1-93.3%)
	Hib3	-	-	78.1% (72.6-82.8%)	86.1% (81.3-90.1%)	88.9% (83.0-93.2%)
	MCV1	24.4% (14.2-37.1%)	61.8% (54.4-68.7%)	75.6% (67.8-82.1%)	82.8% (72.1-90.1%)	95.8% (89.1-98.9%)
	MCV2	-	-	34.1% (26.0-42.3%)	68.0% (57.8-77.1%)	85.8% (77.8-91.5%)
	PCV3	-	-	-	-	79.6% (73.7-84.8%)
	Pol3	89.6% (84.5-93.8%)	90.2% (87.1-92.8%)	81.5% (76.8-85.8%)	85.8% (80.9-89.8%)	87.4% (80.7-92.2%)
	RCV1	24.3% (14.2-37.0%)	61.6% (54.3-68.5%)	75.4% (67.7-81.9%)	82.6% (72.0-90.0%)	95.7% (89.0-98.8%)
	RotaC	-	-	-	67.8% (63.6-71.6%)	88.4% (82.6-92.7%)
Azerbaijan (AZE)	BCG	68.2% (41.2-88.7%)	77.5% (72.1-82.5%)	73.5% (71.4-75.5%)	96.7% (95.5-97.7%)	96.0% (92.8-98.0%)
	DTP1	32.7% (16.0-53.8%)	39.2% (30.1-51.5%)	59.3% (49.6-69.2%)	90.3% (86.9-93.2%)	86.3% (81.3-91.0%)
	DTP3	26.9% (12.0-48.3%)	31.3% (24.7-38.4%)	33.0% (29.9-36.5%)	81.6% (78.4-84.5%)	81.5% (75.7-86.5%)
	HepB3	-	-	-	80.2% (76.1-83.4%)	81.3% (75.5-86.4%)
	Hib3	-	-	-	-	81.3% (75.5-86.2%)
	MCV1	56.5% (25.9-84.5%)	65.5% (58.3-72.1%)	65.5% (62.7-68.1%)	88.0% (85.8-89.9%)	88.1% (83.8-91.7%)
	MCV2	-	-	-	87.8% (85.6-89.7%)	87.7% (83.5-91.1%)
	PCV3	-	-	-	-	81.4% (75.5-86.3%)
	Pol3	41.6% (21.8-63.0%)	48.8% (43.0-55.0%)	42.6% (40.2-44.9%)	85.7% (83.5-87.7%)	84.7% (80.5-88.3%)
	RCV1	-	-	-	87.9% (85.7-89.8%)	88.0% (83.8-91.6%)
	RotaC	-	-	-	-	-
Burundi (BDI)	BCG	54.0% (44.1-63.0%)	84.0% (81.6-86.3%)	88.4% (86.5-90.1%)	98.2% (97.4-98.8%)	96.5% (93.5-98.4%)
	DTP1	45.0% (35.4-56.5%)	83.2% (79.7-86.6%)	82.1% (78.9-85.2%)	98.1% (97.3-98.7%)	97.9% (96.4-99.0%)
	DTP3	33.6% (26.1-41.5%)	71.4% (67.6-74.8%)	70.3% (68.0-72.7%)	96.0% (94.6-97.1%)	95.8% (92.8-97.7%)
	HepB3	-	-	-	95.9% (94.5-97.0%)	95.6% (92.4-97.6%)
	Hib3	-	-	-	95.9% (94.5-97.0%)	95.7% (92.6-97.6%)
	MCV1	32.8% (21.7-45.9%)	73.0% (66.2-79.0%)	82.8% (79.8-85.6%)	95.6% (93.6-97.0%)	91.0% (85.2-95.1%)
	MCV2	-	-	-	-	77.1% (68.3-84.1%)
	PCV3	-	-	-	-	95.7% (92.6-97.6%)
	Pol3	36.9% (29.6-45.0%)	72.6% (69.1-75.8%)	73.3% (70.0-76.2%)	90.1% (88.1-92.0%)	90.6% (86.8-93.7%)
	RCV1	-	-	-	-	90.9% (85.1-95.0%)
	RotaC	-	-	-	-	95.7% (92.7-97.6%)
Belgium (BEL)	BCG	-	-	-	-	-
	DTP1	98.1% (96.1-99.2%)	94.6% (92.4-96.4%)	93.4% (90.6-95.6%)	99.0% (98.2-99.6%)	99.3% (98.4-99.8%)
	DTP3	94.1% (89.8-97.0%)	90.3% (86.9-93.1%)	89.2% (85.2-92.4%)	98.3% (96.8-99.2%)	98.3% (96.3-99.4%)
	HepB3	-	-	54.4% (45.9-62.8%)	96.7% (94.3-98.4%)	97.0% (93.4-98.8%)
	Hib3	-	-	76.6% (69.6-82.7%)	98.0% (96.6-99.0%)	97.2% (94.7-98.6%)
	MCV1	49.6% (32.5-65.3%)	75.7% (68.4-82.0%)	82.1% (75.6-87.7%)	95.8% (91.7-98.2%)	96.8% (91.6-99.1%)
	MCV2	-	-	61.5% (48.5-71.9%)	83.8% (78.4-88.0%)	85.7% (79.5-90.6%)
	PCV3	-	-	-	85.5% (81.8-88.6%)	95.2% (93.3-96.8%)
	Pol3	95.4% (92.3-97.5%)	94.3% (91.7-96.4%)	95.9% (93.4-97.6%)	98.8% (97.8-99.5%)	98.5% (96.7-99.5%)
	RCV1	-	75.6% (68.3-81.8%)	82.0% (75.5-87.5%)	95.7% (91.6-98.1%)	96.7% (91.5-99.0%)

Country	Vaccine	1980	1990	2000	2010	2019
	RotaC	-	-	-	77.1% (74.8-79.2%)	90.4% (88.4-91.8%)
Benin (BEN)	BCG	-	85.6% (82.0-88.7%)	89.2% (87.4-90.9%)	91.3% (89.2-93.2%)	84.1% (79.1-88.3%)
	DTP1	-	73.4% (67.9-79.3%)	88.1% (85.2-90.5%)	86.7% (83.7-89.5%)	86.3% (80.3-90.9%)
	DTP3	-	58.9% (52.9-64.6%)	71.8% (68.0-75.1%)	76.2% (72.4-79.5%)	81.7% (74.6-87.3%)
	HepB3	-	-	-	75.8% (71.8-79.0%)	81.6% (74.4-87.2%)
	Hib3	-	-	-	76.1% (72.3-79.4%)	81.6% (74.6-87.3%)
	MCV1	-	65.7% (59.3-72.1%)	71.6% (67.9-75.4%)	75.8% (71.0-79.9%)	75.3% (67.0-83.2%)
	MCV2	-	-	-	-	-
	PCV3	-	-	-	-	81.3% (74.2-86.8%)
	Pol3	-	50.1% (42.8-56.7%)	66.9% (62.5-71.2%)	74.5% (70.2-78.7%)	78.2% (70.0-84.6%)
	RCV1	-	-	-	-	75.2% (66.9-83.2%)
	RotaC	-	-	-	-	78.4% (71.6-83.8%)
Burkina Faso (BFA)	BCG	19.4% (11.9-28.7%)	81.0% (78.7-83.2%)	80.2% (78.1-82.1%)	96.6% (95.5-97.6%)	98.6% (96.8-99.5%)
	DTP1	29.6% (14.5-52.5%)	72.1% (65.1-78.9%)	78.1% (72.9-83.3%)	93.2% (91.4-94.8%)	95.9% (92.9-98.0%)
	DTP3	6.4% (2.9-11.3%)	45.7% (41.7-49.8%)	54.6% (51.1-58.0%)	90.4% (88.2-92.4%)	92.4% (87.3-95.9%)
	HepB3	-	-	-	90.0% (87.7-92.0%)	92.2% (87.2-95.7%)
	Hib3	-	-	-	90.2% (88.0-92.2%)	92.3% (87.1-95.8%)
	MCV1	44.9% (30.8-59.7%)	63.5% (57.9-68.6%)	63.5% (58.6-68.2%)	90.2% (86.2-93.1%)	95.1% (88.5-98.6%)
	MCV2	-	-	-	-	67.8% (58.1-77.2%)
	PCV3	-	-	-	-	92.3% (87.3-95.8%)
	Pol3	5.8% (2.8-10.4%)	45.9% (41.9-49.7%)	57.0% (53.7-60.2%)	93.0% (90.7-94.8%)	95.3% (91.0-97.7%)
	RCV1	-	-	-	-	95.0% (88.4-98.5%)
	RotaC	-	-	-	-	92.3% (87.3-95.8%)
Bangladesh (BGD)	BCG	1.5% (0.6-3.0%)	81.0% (78.5-83.6%)	93.3% (92.2-94.3%)	96.5% (95.5-97.3%)	98.0% (96.2-99.1%)
	DTP1	22.2% (11.0-37.8%)	79.3% (75.1-83.2%)	91.2% (89.4-92.8%)	97.1% (96.2-97.9%)	99.1% (98.2-99.6%)
	DTP3	1.8% (0.8-3.6%)	61.3% (57.4-64.8%)	79.8% (77.6-81.8%)	91.2% (89.2-92.7%)	94.7% (91.5-97.1%)
	HepB3	-	-	-	90.8% (88.8-92.5%)	94.6% (91.3-96.9%)
	Hib3	-	-	-	91.1% (89.1-92.6%)	94.6% (91.3-96.9%)
	MCV1	2.0% (0.4-5.3%)	63.0% (57.8-68.3%)	76.9% (73.4-80.5%)	87.2% (84.0-89.8%)	92.9% (87.2-96.6%)
	MCV2	-	-	-	-	85.2% (77.8-90.4%)
	PCV3	-	-	-	-	94.6% (91.4-96.9%)
	Pol3	2.4% (1.1-4.8%)	55.8% (51.0-60.6%)	81.4% (79.0-83.6%)	91.5% (89.4-93.2%)	93.4% (89.7-96.2%)
	RCV1	-	-	-	-	92.8% (87.1-96.5%)
	RotaC	-	-	-	-	-
Bulgaria (BGR)	BCG	99.5% (98.3-99.9%)	99.8% (99.3-99.9%)	99.1% (97.8-99.7%)	98.6% (97.0-99.4%)	96.7% (93.7-98.7%)
	DTP1	99.8% (99.5-99.9%)	99.7% (99.4-99.8%)	94.8% (92.9-96.3%)	95.9% (94.3-97.1%)	95.1% (92.1-97.2%)
	DTP3	97.5% (95.7-98.7%)	97.6% (96.6-98.4%)	93.0% (90.6-94.9%)	94.0% (92.0-95.7%)	92.1% (87.8-95.3%)
	HepB3	-	-	90.9% (87.5-93.6%)	93.6% (91.3-95.4%)	79.3% (67.1-87.9%)
	Hib3	-	-	-	92.9% (90.5-94.9%)	91.4% (87.1-94.7%)
	MCV1	96.9% (92.9-98.9%)	98.8% (97.4-99.6%)	92.7% (88.5-95.7%)	97.0% (94.4-98.7%)	94.3% (89.2-97.7%)
	MCV2	-	97.7% (96.2-98.8%)	88.9% (84.3-92.5%)	96.0% (93.3-97.7%)	89.9% (84.1-94.3%)
	PCV3	-	-	-	80.1% (75.6-84.1%)	88.4% (84.3-91.7%)
	Pol3	97.3% (95.3-98.5%)	95.1% (93.1-96.7%)	93.5% (91.2-95.5%)	94.3% (92.2-95.9%)	92.3% (88.0-95.1%)
	RCV1	-	-	92.6% (88.3-95.6%)	96.9% (94.3-98.5%)	94.2% (89.1-97.6%)
	RotaC	-	-	-	-	39.7% (36.6-42.9%)
Bahrain (BHR)	BCG	-	-	-	-	-
	DTP1	64.1% (53.6-74.5%)	97.1% (95.7-98.2%)	94.3% (92.5-95.9%)	94.4% (92.8-95.8%)	95.1% (92.8-96.8%)
	DTP3	58.7% (48.7-68.7%)	92.8% (90.9-94.5%)	91.8% (90.1-93.5%)	93.5% (91.9-94.9%)	94.5% (92.1-96.3%)
	HepB3	-	-	91.4% (89.5-93.1%)	93.4% (91.7-94.8%)	94.4% (92.0-96.1%)
	Hib3	-	-	90.0% (87.8-91.9%)	93.3% (91.6-94.7%)	94.4% (92.0-96.2%)
	MCV1	41.9% (30.8-54.8%)	90.5% (86.7-93.4%)	99.2% (98.1-99.7%)	99.8% (99.4-100.0%)	99.9% (99.6-100.0%)
	MCV2	-	89.8% (85.9-92.7%)	97.8% (96.3-98.8%)	99.3% (98.7-99.6%)	99.7% (99.4-99.9%)
	PCV3	-	-	-	93.0% (91.4-94.5%)	93.8% (91.5-95.6%)
	Pol3	59.1% (49.5-68.5%)	92.7% (90.9-94.4%)	91.8% (90.0-93.4%)	93.7% (92.0-95.1%)	94.9% (92.7-96.7%)

Country	Vaccine	1980	1990	2000	2010	2019
	RCV1	-	90.3% (86.5-93.2%)	99.1% (98.0-99.6%)	99.7% (99.4-99.9%)	99.8% (99.5-99.9%)
	RotaC	-	-	-	37.3% (34.7-40.2%)	94.3% (92.0-96.1%)
The Bahamas (BHS)	BCG	-	-	-	-	-
	DTP1	51.9% (42.4-61.0%)	89.7% (86.5-92.6%)	93.4% (91.2-95.4%)	97.8% (96.5-98.7%)	93.8% (89.7-96.6%)
	DTP3	46.4% (37.8-54.6%)	87.5% (83.9-90.5%)	92.8% (90.3-95.0%)	95.6% (93.3-97.2%)	88.2% (81.9-92.6%)
	HepB3	-	-	-	93.2% (88.0-96.0%)	88.0% (81.8-92.5%)
	Hib3	-	-	89.2% (85.0-92.2%)	95.2% (93.0-96.8%)	88.1% (81.8-92.5%)
	MCV1	62.5% (45.0-77.6%)	96.2% (94.0-97.7%)	92.5% (88.1-95.8%)	94.5% (90.3-97.0%)	88.3% (80.4-93.5%)
	MCV2	-	-	-	78.4% (71.3-84.3%)	80.2% (72.0-86.2%)
	PCV3	-	-	-	4.9% (3.5-6.7%)	88.1% (81.8-92.5%)
	Pol3	45.7% (37.9-53.8%)	85.2% (81.4-88.3%)	89.7% (86.4-92.2%)	95.1% (93.0-96.8%)	88.6% (83.4-92.7%)
	RCV1	-	-	92.3% (88.0-95.6%)	94.4% (90.2-96.9%)	88.2% (80.3-93.4%)
	RotaC	-	-	-	2.6% (2.3-2.9%)	78.2% (72.5-82.3%)
Bosnia and Herzegovina (BIH)	BCG	27.8% (8.8-55.8%)	33.3% (23.7-44.1%)	95.5% (93.6-96.9%)	98.1% (96.8-98.9%)	96.3% (92.2-98.5%)
	DTP1	80.9% (59.7-94.5%)	81.7% (71.9-88.8%)	94.6% (92.8-96.1%)	94.6% (92.8-96.1%)	90.9% (85.8-94.7%)
	DTP3	68.4% (43.4-86.5%)	73.0% (62.5-81.5%)	90.7% (88.1-92.8%)	90.3% (87.5-92.7%)	77.7% (68.4-84.8%)
	HepB3	-	-	-	87.8% (83.9-91.1%)	77.5% (68.2-84.7%)
	Hib3	-	-	-	80.6% (74.4-85.3%)	52.8% (40.6-64.7%)
	MCV1	45.8% (16.5-78.6%)	55.5% (37.4-72.0%)	74.7% (69.2-79.6%)	91.3% (86.8-94.8%)	68.3% (54.3-80.2%)
	MCV2	28.5% (10.2-50.7%)	47.0% (31.0-63.0%)	69.5% (63.1-74.8%)	89.5% (84.9-93.0%)	68.2% (54.2-80.2%)
	PCV3	-	-	-	-	-
	Pol3	72.4% (49.9-88.4%)	78.5% (70.2-85.5%)	89.4% (87.1-91.3%)	90.8% (88.1-93.0%)	77.4% (69.6-84.5%)
	RCV1	45.6% (16.4-78.1%)	55.3% (37.3-71.8%)	74.5% (69.0-79.4%)	91.0% (86.6-94.5%)	68.2% (54.2-80.1%)
	RotaC	-	-	-	-	-
Belarus (BLR)	BCG	92.1% (84.4-96.9%)	93.1% (90.3-95.2%)	99.6% (99.3-99.8%)	98.5% (97.4-99.2%)	97.2% (94.6-98.8%)
	DTP1	88.8% (77.2-96.1%)	89.2% (84.7-92.8%)	99.5% (99.1-99.7%)	97.8% (96.4-98.8%)	98.2% (96.4-99.2%)
	DTP3	85.9% (71.8-94.4%)	87.0% (82.1-90.9%)	96.6% (95.0-97.7%)	96.2% (94.1-97.8%)	97.7% (95.5-99.0%)
	HepB3	-	-	73.0% (61.8-82.1%)	94.3% (90.4-96.7%)	97.0% (94.5-98.6%)
	Hib3	-	-	-	9.4% (6.4-13.5%)	77.9% (69.0-84.9%)
	MCV1	56.5% (43.0-68.9%)	93.1% (89.5-95.8%)	98.7% (97.4-99.4%)	98.3% (96.7-99.2%)	98.0% (95.6-99.4%)
	MCV2	-	92.3% (88.7-95.2%)	96.8% (94.8-98.1%)	98.1% (96.5-99.0%)	97.9% (95.5-99.3%)
	PCV3	-	-	-	-	3.9% (2.6-5.5%)
	Pol3	80.7% (74.7-85.9%)	88.4% (84.9-91.5%)	98.1% (97.2-98.7%)	96.0% (94.0-97.4%)	98.0% (96.3-99.1%)
	RCV1	-	-	98.5% (97.3-99.3%)	98.2% (96.6-99.1%)	97.9% (95.5-99.3%)
	RotaC	-	-	-	-	-
Belize (BLZ)	BCG	71.1% (63.2-78.4%)	89.9% (87.2-92.3%)	91.4% (88.9-93.4%)	95.6% (93.8-96.9%)	94.0% (90.1-96.8%)
	DTP1	56.5% (46.4-67.3%)	89.1% (85.7-92.0%)	89.6% (86.3-92.5%)	92.7% (90.3-94.7%)	90.1% (86.1-93.7%)
	DTP3	46.1% (37.6-54.2%)	79.2% (75.0-82.8%)	76.6% (72.7-79.9%)	77.2% (74.4-79.9%)	83.4% (79.0-87.5%)
	HepB3	-	-	70.2% (63.1-75.6%)	77.0% (74.2-79.7%)	83.2% (78.9-87.4%)
	Hib3	-	-	-	77.1% (74.3-79.7%)	83.3% (78.9-87.4%)
	MCV1	30.8% (21.8-40.3%)	75.9% (70.0-81.9%)	86.7% (82.1-91.0%)	94.7% (91.9-96.9%)	89.6% (83.0-94.0%)
	MCV2	-	-	-	90.8% (87.4-93.7%)	87.7% (80.7-92.2%)
	PCV3	-	-	-	-	-
	Pol3	51.1% (43.5-59.1%)	80.7% (76.3-84.4%)	76.1% (72.4-79.3%)	75.6% (72.9-78.3%)	85.4% (81.5-89.1%)
	RCV1	-	-	86.6% (81.9-90.8%)	94.6% (91.8-96.8%)	89.5% (82.9-93.9%)
	RotaC	-	-	-	-	-
Bermuda (BMU)	BCG	-	-	-	-	-
	DTP1	90.4% (75.9-97.6%)	98.2% (94.4-99.7%)	99.2% (97.5-99.9%)	99.3% (97.8-99.9%)	99.4% (98.1-99.9%)
	DTP3	88.3% (70.5-96.9%)	97.0% (91.6-99.3%)	98.8% (96.3-99.7%)	99.0% (97.2-99.8%)	99.2% (97.7-99.8%)
	HepB3	-	-	98.2% (95.3-99.4%)	98.9% (97.2-99.7%)	99.1% (97.6-99.8%)
	Hib3	-	95.7% (90.4-98.4%)	98.4% (96.1-99.4%)	99.0% (97.2-99.7%)	99.2% (97.7-99.8%)
	MCV1	83.5% (57.5-96.1%)	98.2% (93.9-99.7%)	99.7% (98.9-99.9%)	99.8% (99.4-100.0%)	99.8% (99.4-100.0%)
	MCV2	-	-	-	-	-
	PCV3	-	-	-	90.6% (87.2-93.3%)	99.1% (97.7-99.8%)

Country	Vaccine	1980	1990	2000	2010	2019
	Pol3	87.5% (72.1-96.1%)	96.2% (89.9-98.9%)	98.2% (95.4-99.6%)	98.7% (96.7-99.7%)	99.2% (97.9-99.8%)
	RCV1	83.2% (57.3-95.7%)	97.9% (93.7-99.4%)	99.5% (98.7-99.8%)	99.7% (99.3-99.9%)	99.8% (99.3-99.9%)
	RotaC	-	-	-	-	-
Bolivia (BOL)	BCG	34.2% (25.8-42.5%)	67.9% (63.5-71.8%)	93.0% (90.4-95.2%)	98.6% (97.4-99.3%)	86.4% (77.5-92.8%)
	DTP1	34.8% (22.6-52.6%)	70.6% (62.1-79.1%)	87.3% (83.2-91.1%)	92.7% (89.6-95.2%)	91.8% (85.5-96.0%)
	DTP3	20.0% (14.1-28.2%)	46.7% (41.8-51.1%)	74.8% (71.6-77.9%)	89.1% (85.4-92.1%)	86.4% (78.9-92.2%)
	HepB3	-	-	74.0% (70.3-77.3%)	88.9% (85.1-92.0%)	86.3% (78.7-92.1%)
	Hib3	-	-	73.3% (69.9-76.6%)	88.9% (85.2-92.0%)	86.3% (78.8-92.1%)
	MCV1	17.3% (11.1-24.9%)	63.3% (58.0-68.5%)	85.9% (82.4-88.7%)	87.9% (82.8-92.3%)	88.4% (79.9-94.3%)
	MCV2	-	-	-	-	43.9% (34.0-53.4%)
	PCV3	-	-	-	-	86.2% (78.8-92.0%)
	Pol3	25.4% (18.5-32.9%)	50.5% (46.2-54.9%)	69.1% (65.1-72.9%)	90.6% (87.3-93.2%)	86.4% (79.3-91.8%)
	RCV1	-	-	85.7% (82.2-88.6%)	87.8% (82.7-92.2%)	88.4% (79.8-94.1%)
	RotaC	-	-	-	80.8% (77.4-83.7%)	86.3% (78.8-92.1%)
Brazil (BRA)	BCG	65.4% (57.4-72.5%)	75.0% (71.5-78.2%)	93.4% (92.1-94.6%)	93.6% (92.3-94.7%)	88.1% (84.3-91.4%)
	DTP1	58.1% (49.6-66.9%)	76.7% (71.9-81.1%)	98.3% (97.2-99.0%)	97.5% (96.2-98.5%)	82.6% (76.1-87.9%)
	DTP3	48.0% (39.9-56.1%)	64.2% (60.4-67.9%)	96.0% (94.4-97.4%)	97.3% (95.8-98.3%)	75.2% (67.9-81.6%)
	HepB3	-	0.2% (0.0-0.6%)	82.6% (73.5-89.0%)	95.9% (93.0-97.6%)	75.1% (67.8-81.4%)
	Hib3	-	-	89.9% (83.5-93.9%)	97.1% (95.6-98.2%)	75.0% (67.7-81.3%)
	MCV1	71.2% (61.3-80.2%)	93.9% (91.8-95.6%)	99.5% (98.7-99.9%)	99.8% (99.5-100.0%)	99.9% (99.6-100.0%)
	MCV2	-	-	92.6% (88.3-95.3%)	58.9% (51.8-65.8%)	63.9% (54.4-72.2%)
	PCV3	-	-	-	51.8% (44.3-59.2%)	74.7% (67.3-81.0%)
	Pol3	64.2% (55.3-72.2%)	72.3% (68.8-75.7%)	99.3% (99.0-99.6%)	99.4% (99.0-99.7%)	77.6% (69.9-84.5%)
	RCV1	-	-	98.2% (97.8-98.6%)	99.1% (98.7-99.4%)	91.7% (89.4-93.4%)
	RotaC	-	-	-	84.2% (82.4-85.8%)	74.9% (67.6-81.2%)
Barbados (BRB)	BCG	-	-	-	-	-
	DTP1	65.2% (57.2-73.0%)	88.0% (84.6-90.9%)	91.1% (88.1-93.7%)	88.6% (84.9-91.4%)	93.9% (89.7-96.6%)
	DTP3	59.9% (51.8-67.3%)	83.5% (79.4-87.1%)	88.0% (84.3-91.0%)	87.6% (83.9-90.7%)	91.1% (85.9-94.8%)
	HepB3	-	-	34.8% (16.2-55.9%)	87.4% (83.8-90.4%)	91.0% (85.8-94.6%)
	Hib3	-	-	40.1% (25.3-55.6%)	87.5% (83.8-90.6%)	91.0% (85.8-94.7%)
	MCV1	42.4% (31.7-53.8%)	88.8% (83.6-92.7%)	91.6% (86.8-95.3%)	90.6% (86.1-94.1%)	98.5% (97.1-99.4%)
	MCV2	-	-	28.2% (17.0-42.9%)	90.4% (85.9-93.9%)	86.0% (79.2-91.6%)
	PCV3	-	-	-	81.7% (77.5-85.3%)	90.8% (85.6-94.4%)
	Pol3	78.6% (70.2-85.7%)	81.9% (76.5-86.6%)	87.4% (82.3-91.3%)	88.9% (84.6-92.6%)	91.6% (85.4-95.8%)
	RCV1	-	88.6% (83.5-92.5%)	91.5% (86.7-95.2%)	90.5% (86.1-94.0%)	98.4% (97.0-99.2%)
	RotaC	-	-	-	-	-
Brunei (BRN)	BCG	98.1% (96.1-99.3%)	92.9% (88.8-95.7%)	98.7% (97.4-99.5%)	98.4% (96.6-99.3%)	99.7% (99.2-99.9%)
	DTP1	92.9% (88.5-96.3%)	97.8% (96.2-99.0%)	95.6% (93.4-97.2%)	98.0% (96.4-99.1%)	98.1% (96.4-99.1%)
	DTP3	88.9% (83.0-93.3%)	94.6% (92.1-96.4%)	94.9% (92.5-96.7%)	95.8% (93.6-97.4%)	98.0% (96.2-99.0%)
	HepB3	-	92.3% (87.1-95.2%)	94.7% (92.3-96.5%)	95.0% (92.6-96.8%)	97.8% (96.0-98.9%)
	Hib3	-	-	-	95.6% (93.5-97.1%)	97.8% (96.0-98.9%)
	MCV1	73.7% (62.1-83.4%)	98.4% (96.0-99.5%)	98.9% (97.5-99.7%)	96.7% (93.4-98.4%)	99.0% (97.3-99.8%)
	MCV2	-	98.2% (95.8-99.3%)	98.7% (97.4-99.5%)	95.7% (92.5-97.6%)	98.7% (97.1-99.5%)
	PCV3	-	-	-	-	-
	Pol3	89.1% (83.5-93.2%)	95.6% (93.6-97.2%)	96.7% (95.0-97.9%)	96.8% (95.2-98.0%)	97.9% (96.2-99.0%)
	RCV1	-	98.0% (95.5-99.0%)	98.6% (97.2-99.4%)	96.6% (93.3-98.3%)	98.9% (97.2-99.7%)
	RotaC	-	-	-	-	-
Bhutan (BTN)	BCG	49.1% (40.1-57.9%)	83.3% (78.5-87.6%)	93.1% (89.7-95.8%)	99.5% (98.7-99.8%)	99.1% (97.5-99.8%)
	DTP1	20.2% (12.6-32.2%)	89.7% (85.6-93.0%)	95.9% (93.5-97.7%)	98.4% (96.6-99.4%)	99.9% (99.8-100.0%)
	DTP3	11.9% (7.6-17.1%)	83.2% (79.0-87.0%)	91.1% (87.3-94.0%)	97.9% (95.4-99.1%)	99.8% (99.6-100.0%)
	HepB3	-	-	90.7% (86.9-93.7%)	97.7% (95.2-99.0%)	99.7% (99.3-99.9%)
	Hib3	-	-	-	-	99.7% (99.4-99.9%)
	MCV1	21.2% (14.4-29.4%)	71.7% (64.8-78.2%)	79.3% (72.8-85.6%)	97.7% (95.1-99.1%)	99.6% (98.6-99.9%)
	MCV2	-	-	-	90.5% (86.5-93.9%)	95.0% (91.9-97.2%)

Country	Vaccine	1980	1990	2000	2010	2019
	PCV3	-	-	-	-	27.5% (21.0-34.6%)
	Pol3	10.4% (6.9-14.8%)	83.7% (79.5-87.3%)	95.4% (93.5-97.0%)	97.3% (94.7-98.9%)	99.1% (97.9-99.7%)
	RCV1	-	-	-	97.6% (94.9-99.0%)	99.4% (98.5-99.8%)
	RotaC	-	-	-	-	-
Botswana (BWA)	BCG	88.5% (82.8-93.1%)	91.5% (88.1-94.1%)	92.2% (88.9-94.7%)	95.0% (93.1-96.5%)	90.0% (84.7-94.0%)
	DTP1	87.6% (81.5-92.9%)	94.9% (93.0-96.6%)	94.8% (92.5-96.6%)	97.8% (96.6-98.8%)	92.1% (85.8-96.4%)
	DTP3	77.9% (71.5-83.5%)	92.3% (90.0-94.2%)	87.8% (85.2-90.1%)	94.6% (93.0-95.9%)	79.0% (72.6-84.5%)
	HepB3	-	-	61.4% (50.3-70.2%)	85.7% (78.6-90.7%)	78.8% (72.4-84.3%)
	Hib3	-	-	-	-	78.9% (72.5-84.4%)
	MCV1	72.7% (62.8-81.1%)	86.4% (80.5-91.1%)	86.7% (81.2-90.7%)	94.6% (90.5-97.2%)	88.3% (79.0-94.1%)
	MCV2	-	-	-	-	74.6% (64.5-82.0%)
	PCV3	-	-	-	-	77.7% (71.3-83.1%)
	Pol3	79.5% (73.2-85.0%)	89.3% (86.5-91.8%)	88.1% (85.5-90.5%)	95.4% (93.9-96.7%)	91.3% (87.5-94.1%)
	RCV1	-	-	-	-	84.5% (75.7-90.4%)
	RotaC	-	-	-	-	73.7% (67.6-78.8%)
Central African Republic (CAF)	BCG	37.3% (29.0-46.3%)	94.8% (92.9-96.3%)	65.1% (60.3-69.3%)	63.2% (58.1-68.0%)	60.7% (50.4-70.1%)
	DTP1	33.6% (25.5-43.4%)	78.7% (74.6-82.5%)	60.5% (55.9-65.1%)	72.0% (66.9-76.6%)	74.3% (66.1-82.0%)
	DTP3	18.0% (12.6-25.3%)	61.4% (56.3-66.4%)	36.0% (32.1-39.8%)	40.5% (36.3-45.0%)	45.6% (37.0-54.4%)
	HepB3	-	-	-	39.7% (35.6-44.0%)	45.6% (36.9-54.3%)
	Hib3	-	-	-	40.4% (36.3-44.9%)	45.6% (36.9-54.3%)
	MCV1	12.8% (6.2-23.0%)	54.9% (46.8-63.3%)	44.9% (37.7-51.9%)	53.5% (46.3-60.2%)	39.9% (28.4-52.7%)
	MCV2	-	-	-	-	-
	PCV3	-	-	-	-	45.5% (36.8-54.3%)
	Pol3	18.7% (12.0-26.4%)	61.1% (54.5-67.6%)	45.1% (39.5-50.9%)	44.8% (38.1-51.7%)	41.4% (31.5-52.7%)
	RCV1	-	-	-	-	-
	RotaC	-	-	-	-	-
Canada (CAN)	BCG	-	-	-	-	-
	DTP1	90.8% (80.8-97.0%)	96.1% (93.7-97.8%)	97.5% (95.9-98.7%)	94.1% (91.4-96.5%)	92.2% (88.5-95.4%)
	DTP3	77.9% (61.3-89.6%)	85.1% (80.2-89.1%)	92.2% (89.3-94.5%)	84.7% (81.4-87.7%)	87.7% (83.2-91.7%)
	HepB3	-	-	10.4% (2.2-28.6%)	51.0% (42.6-59.4%)	70.9% (60.1-79.3%)
	Hib3	-	81.8% (75.5-86.6%)	91.7% (88.8-94.0%)	84.4% (81.2-87.5%)	87.6% (83.2-91.6%)
	MCV1	69.0% (41.3-90.1%)	89.5% (84.0-93.6%)	95.1% (92.0-97.2%)	93.7% (91.1-95.7%)	90.7% (84.7-94.9%)
	MCV2	-	-	87.1% (79.9-92.2%)	88.1% (83.0-92.0%)	87.5% (81.8-92.2%)
	PCV3	-	-	-	55.6% (50.9-60.1%)	80.2% (75.7-84.5%)
	Pol3	87.6% (75.1-95.0%)	92.1% (86.7-95.8%)	94.1% (90.8-96.5%)	95.9% (93.4-97.6%)	98.0% (95.8-99.3%)
	RCV1	-	89.3% (83.8-93.4%)	94.9% (91.9-97.0%)	93.6% (91.0-95.6%)	90.5% (84.6-94.8%)
	RotaC	-	-	-	-	77.7% (73.6-81.5%)
Switzerland (CHE)	BCG	-	-	-	-	-
	DTP1	93.4% (86.6-97.8%)	93.8% (91.7-95.5%)	94.7% (93.1-96.2%)	95.7% (93.9-97.2%)	98.1% (96.8-99.0%)
	DTP3	88.3% (76.8-94.9%)	89.7% (86.2-92.4%)	92.3% (89.6-94.4%)	95.5% (93.7-97.1%)	96.2% (93.5-98.0%)
	HepB3	-	-	-	-	71.3% (62.0-79.2%)
	Hib3	-	73.9% (58.4-84.1%)	89.1% (85.3-91.9%)	95.2% (93.3-96.7%)	95.2% (92.3-97.1%)
	MCV1	68.1% (42.3-88.2%)	85.2% (79.0-89.7%)	83.0% (77.6-87.7%)	93.3% (89.6-96.0%)	96.1% (92.0-98.5%)
	MCV2	-	-	61.3% (48.7-72.0%)	84.4% (79.4-88.7%)	90.8% (85.6-94.5%)
	PCV3	-	-	-	49.1% (43.2-54.7%)	86.8% (83.4-89.7%)
	Pol3	97.2% (93.7-99.0%)	97.0% (95.0-98.2%)	95.0% (93.0-96.6%)	96.4% (94.6-97.7%)	96.6% (94.1-98.3%)
	RCV1	63.7% (39.6-82.8%)	81.3% (75.3-85.7%)	80.7% (75.2-85.3%)	90.7% (86.9-93.5%)	96.0% (91.9-98.4%)
	RotaC	-	-	-	-	-
Chile (CHL)	BCG	95.6% (91.7-98.0%)	97.7% (95.9-98.9%)	94.3% (91.1-96.6%)	96.4% (94.2-97.8%)	97.2% (94.7-98.7%)
	DTP1	98.4% (96.9-99.4%)	98.6% (97.8-99.2%)	97.6% (96.3-98.5%)	93.8% (91.2-95.7%)	98.9% (97.9-99.5%)
	DTP3	93.3% (89.2-96.2%)	91.8% (89.1-94.1%)	92.6% (90.1-94.7%)	92.8% (90.0-95.0%)	95.3% (92.2-97.4%)
	HepB3	-	-	-	92.5% (89.7-94.7%)	95.1% (92.0-97.3%)
	Hib3	-	-	92.5% (89.9-94.6%)	92.7% (89.8-94.8%)	95.2% (92.0-97.3%)
	MCV1	95.3% (91.0-98.1%)	95.6% (92.4-97.7%)	96.8% (94.0-98.7%)	93.0% (89.0-95.9%)	95.1% (90.4-98.0%)

Country	Vaccine	1980	1990	2000	2010	2019
	MCV2	-	-	96.6% (93.9-98.5%)	84.2% (79.0-88.7%)	93.1% (88.3-96.4%)
	PCV3	-	-	-	-	94.4% (91.3-96.6%)
	Pol3	92.9% (89.0-95.8%)	91.8% (89.2-94.1%)	92.8% (90.2-94.7%)	92.9% (90.2-95.0%)	95.5% (92.5-97.5%)
	RCV1	-	95.3% (92.2-97.5%)	96.7% (93.9-98.5%)	92.9% (88.9-95.8%)	95.0% (90.4-97.9%)
	RotaC	-	-	-	-	-
China (CHN)	BCG	46.7% (32.3-61.1%)	94.6% (92.2-96.3%)	98.5% (97.0-99.4%)	99.5% (98.7-99.9%)	99.8% (99.1-100.0%)
	DTP1	69.3% (59.7-78.6%)	91.8% (89.8-93.6%)	93.1% (90.7-95.0%)	95.1% (93.2-96.6%)	97.0% (95.0-98.3%)
	DTP3	65.4% (55.0-74.9%)	86.7% (83.7-89.1%)	92.3% (89.6-94.4%)	94.3% (92.1-95.9%)	96.8% (94.7-98.2%)
	HepB3	-	-	65.5% (54.5-75.0%)	93.9% (91.6-95.5%)	96.7% (94.5-98.0%)
	Hib3	-	-	30.9% (24.1-37.9%)	51.8% (43.5-59.9%)	10.5% (6.0-16.2%)
	MCV1	81.8% (65.4-92.5%)	80.9% (76.5-84.8%)	96.6% (94.1-98.3%)	95.6% (92.6-97.8%)	96.3% (92.7-98.4%)
	MCV2	-	76.3% (70.8-81.0%)	94.4% (91.5-96.4%)	95.2% (92.4-96.9%)	95.5% (92.4-97.0%)
	PCV3	-	-	-	1.1% (0.8-1.5%)	7.3% (4.3-10.9%)
	Pol3	81.8% (75.2-87.0%)	89.5% (87.4-91.5%)	96.4% (95.1-97.5%)	95.7% (94.3-97.0%)	98.0% (96.6-98.9%)
	RCV1	-	-	95.2% (92.8-96.7%)	94.6% (91.7-96.4%)	95.8% (92.6-97.3%)
	RotaC	-	-	0.6% (0.5-0.6%)	10.2% (7.6-13.2%)	13.2% (7.7-20.1%)
Côte d'Ivoire (CIV)	BCG	24.4% (12.6-40.5%)	73.3% (67.5-78.7%)	87.5% (85.1-89.5%)	75.8% (71.9-79.3%)	95.9% (92.8-97.9%)
	DTP1	45.5% (29.2-66.4%)	69.1% (62.3-75.8%)	83.5% (79.6-87.2%)	85.6% (82.2-88.6%)	94.1% (90.0-97.1%)
	DTP3	22.9% (12.9-35.6%)	47.6% (42.0-53.4%)	66.2% (61.9-70.4%)	70.4% (66.9-73.9%)	77.6% (71.5-83.3%)
	HepB3	-	-	-	70.3% (66.7-73.8%)	77.5% (71.3-83.2%)
	Hib3	-	-	-	70.4% (66.7-73.8%)	77.6% (71.4-83.2%)
	MCV1	36.7% (16.0-63.0%)	55.3% (47.4-62.9%)	69.0% (62.6-74.9%)	63.4% (57.6-68.9%)	74.5% (64.7-82.5%)
	MCV2	-	-	-	-	-
	PCV3	-	-	-	-	77.3% (71.2-82.9%)
	Pol3	15.8% (7.2-28.9%)	51.4% (46.0-57.7%)	71.4% (67.7-75.2%)	73.3% (69.6-76.8%)	70.1% (63.6-76.1%)
	RCV1	-	-	-	-	74.2% (64.5-82.1%)
	RotaC	-	-	-	-	65.0% (59.7-69.9%)
Cameroon (CMR)	BCG	27.8% (17.7-40.7%)	73.5% (69.9-76.7%)	82.2% (79.7-84.4%)	89.5% (86.6-92.0%)	87.9% (82.4-92.1%)
	DTP1	30.8% (19.2-45.5%)	64.9% (60.1-69.9%)	74.9% (71.3-78.5%)	86.9% (84.2-89.4%)	82.5% (76.7-87.3%)
	DTP3	17.8% (10.6-26.6%)	45.0% (40.0-50.0%)	57.2% (52.8-61.2%)	73.2% (69.2-77.1%)	72.5% (64.9-78.7%)
	HepB3	-	-	-	73.1% (69.1-77.0%)	72.4% (64.8-78.6%)
	Hib3	-	-	-	73.1% (69.1-77.0%)	72.4% (64.8-78.6%)
	MCV1	26.8% (15.7-40.9%)	55.5% (49.6-61.2%)	72.4% (67.8-76.7%)	75.7% (70.6-80.4%)	66.7% (56.1-76.0%)
	MCV2	-	-	-	-	34.1% (21.3-47.9%)
	PCV3	-	-	-	-	72.3% (64.8-78.5%)
	Pol3	16.9% (10.8-25.1%)	47.4% (43.4-51.2%)	62.5% (58.8-65.9%)	76.3% (73.1-79.4%)	66.5% (59.8-72.8%)
	RCV1	-	-	-	-	66.6% (56.1-75.9%)
	RotaC	-	-	-	-	71.6% (64.2-77.7%)
DR Congo (COD)	BCG	83.5% (71.8-91.3%)	62.6% (55.7-69.4%)	53.9% (47.9-59.8%)	88.2% (85.0-90.9%)	79.4% (71.3-86.1%)
	DTP1	29.7% (19.2-43.8%)	43.7% (35.9-52.9%)	48.0% (40.8-56.0%)	77.3% (73.3-81.0%)	86.1% (77.7-92.5%)
	DTP3	20.1% (12.4-30.6%)	33.2% (27.9-38.9%)	30.2% (25.4-35.3%)	60.6% (56.3-64.6%)	52.4% (45.1-59.7%)
	HepB3	-	-	-	58.0% (53.1-62.1%)	52.3% (45.0-59.6%)
	Hib3	-	-	-	60.5% (56.3-64.5%)	52.3% (45.0-59.6%)
	MCV1	23.6% (15.6-32.9%)	37.6% (32.3-43.8%)	47.5% (41.3-53.9%)	78.4% (74.8-81.9%)	67.0% (58.2-74.5%)
	MCV2	-	-	-	-	-
	PCV3	-	-	-	-	52.3% (45.0-59.6%)
	Pol3	21.0% (11.7-32.5%)	33.0% (27.6-39.0%)	39.7% (33.7-45.8%)	67.9% (63.8-72.0%)	44.7% (36.7-53.0%)
	RCV1	-	-	-	-	-
	RotaC	-	-	-	-	50.4% (43.5-57.6%)
Congo (Brazzaville) (COG)	BCG	87.5% (79.5-92.9%)	85.2% (81.6-88.3%)	49.5% (43.6-55.4%)	94.1% (92.3-95.5%)	88.2% (81.8-92.7%)
	DTP1	60.1% (48.3-73.2%)	86.0% (81.7-89.7%)	51.5% (42.2-61.7%)	82.3% (78.3-86.4%)	66.5% (59.0-73.5%)
	DTP3	47.9% (37.4-59.0%)	78.2% (73.4-82.3%)	36.1% (30.5-41.7%)	69.1% (65.7-72.6%)	64.3% (56.9-71.3%)
	HepB3	-	-	-	53.1% (37.7-63.7%)	64.0% (56.5-71.1%)
	Hib3	-	-	-	65.6% (60.7-70.0%)	64.2% (56.7-71.2%)

Country	Vaccine	1980	1990	2000	2010	2019
	MCV1	45.2% (29.4-61.1%)	72.9% (66.3-79.1%)	34.0% (26.7-42.3%)	77.2% (72.1-81.8%)	72.5% (62.3-81.0%)
	MCV2	-	-	-	-	9.0% (4.3-15.7%)
	PCV3	-	-	-	-	64.2% (56.8-71.2%)
	Pol3	46.6% (34.7-59.3%)	88.0% (84.5-91.1%)	34.9% (28.9-41.8%)	58.9% (55.0-62.8%)	49.1% (40.8-57.3%)
	RCV1	-	-	-	-	72.4% (62.2-80.8%)
	RotaC	-	-	-	-	64.2% (56.8-71.2%)
Cook Islands (COK)	BCG	29.4% (20.8-40.5%)	95.1% (92.6-97.0%)	98.1% (96.2-99.2%)	99.4% (98.3-99.9%)	99.7% (98.7-99.9%)
	DTP1	48.6% (37.7-60.7%)	95.3% (92.7-97.2%)	96.8% (94.6-98.3%)	95.6% (92.6-97.8%)	98.1% (95.8-99.3%)
	DTP3	40.6% (31.2-50.1%)	87.7% (84.0-90.4%)	89.1% (85.2-92.3%)	93.2% (90.0-95.9%)	97.2% (94.2-98.8%)
	HepB3	-	65.3% (52.4-74.8%)	87.9% (83.6-91.2%)	93.0% (89.7-95.6%)	97.0% (94.1-98.8%)
	Hib3	-	-	-	93.1% (89.8-95.7%)	97.1% (94.1-98.7%)
	MCV1	65.9% (46.1-84.4%)	80.9% (73.2-87.2%)	81.8% (74.8-87.8%)	97.5% (95.8-98.7%)	99.3% (98.0-99.8%)
	MCV2	-	56.5% (42.4-68.9%)	62.7% (54.3-70.8%)	96.5% (94.7-98.0%)	98.4% (96.9-99.2%)
	PCV3	-	-	-	-	-
	Pol3	40.4% (31.7-49.7%)	83.9% (79.1-87.9%)	88.7% (84.3-92.3%)	93.3% (89.7-96.2%)	97.4% (94.6-99.0%)
	RCV1	-	-	-	97.4% (95.7-98.6%)	99.1% (97.9-99.7%)
	RotaC	-	-	-	-	-
Colombia (COL)	BCG	66.5% (59.2-73.4%)	94.4% (93.1-95.4%)	95.5% (94.3-96.5%)	92.2% (89.5-94.2%)	92.5% (87.4-95.9%)
	DTP1	58.6% (47.8-69.4%)	94.2% (92.2-95.9%)	94.5% (91.9-96.5%)	92.6% (89.8-95.1%)	92.3% (88.2-95.6%)
	DTP3	40.7% (33.3-48.9%)	86.3% (83.6-88.6%)	80.4% (77.6-82.8%)	88.5% (85.4-91.2%)	90.9% (86.3-94.6%)
	HepB3	-	-	63.9% (57.3-69.9%)	88.2% (85.0-90.9%)	90.7% (86.0-94.4%)
	Hib3	-	-	60.3% (56.1-64.1%)	88.3% (85.1-91.0%)	90.7% (86.2-94.5%)
	MCV1	34.8% (26.4-44.2%)	93.5% (90.8-95.6%)	92.6% (89.7-95.1%)	90.0% (85.8-93.0%)	92.5% (87.2-95.8%)
	MCV2	-	-	92.0% (89.1-94.5%)	70.0% (62.5-76.5%)	86.3% (80.5-91.0%)
	PCV3	-	-	-	37.6% (30.5-44.8%)	90.4% (85.8-94.2%)
	Pol3	41.4% (34.0-48.7%)	83.9% (81.4-86.3%)	72.3% (69.6-74.9%)	81.3% (78.1-84.7%)	83.5% (78.1-88.2%)
	RCV1	-	-	85.7% (82.7-88.4%)	89.8% (85.7-92.9%)	92.4% (87.1-95.8%)
	RotaC	-	-	-	74.5% (71.6-77.2%)	89.6% (85.1-93.3%)
Comoros (COM)	BCG	62.4% (40.1-81.1%)	91.8% (87.9-94.6%)	96.1% (94.4-97.4%)	84.6% (80.4-88.1%)	97.4% (94.5-99.0%)
	DTP1	48.0% (31.4-66.7%)	93.6% (90.7-95.9%)	83.2% (78.3-87.5%)	81.9% (77.5-85.4%)	93.2% (88.7-96.3%)
	DTP3	30.0% (17.0-46.1%)	83.0% (78.0-87.4%)	67.9% (61.8-73.5%)	72.0% (66.7-76.6%)	84.1% (76.0-90.2%)
	HepB3	-	-	-	71.6% (66.4-76.3%)	84.0% (75.9-90.1%)
	Hib3	-	-	-	71.2% (66.2-76.0%)	84.0% (75.9-90.0%)
	MCV1	30.4% (12.7-55.1%)	62.5% (52.7-71.3%)	69.6% (62.0-76.7%)	74.9% (69.3-80.0%)	91.1% (82.6-96.3%)
	MCV2	-	-	-	-	-
	PCV3	-	-	-	-	-
	Pol3	31.6% (18.3-48.4%)	84.2% (79.3-88.3%)	60.8% (54.3-67.4%)	65.4% (59.3-70.7%)	84.9% (76.7-91.2%)
	RCV1	-	-	-	-	-
	RotaC	-	-	-	-	-
Cape Verde (CPV)	BCG	66.7% (49.1-82.3%)	94.3% (93.1-95.5%)	97.0% (95.7-97.9%)	97.5% (95.7-98.7%)	99.3% (98.0-99.9%)
	DTP1	35.3% (21.6-53.9%)	95.2% (92.1-97.7%)	94.6% (92.5-96.3%)	98.1% (96.5-99.1%)	99.6% (99.2-99.9%)
	DTP3	23.2% (14.3-34.7%)	86.4% (84.1-88.5%)	91.5% (89.5-93.2%)	95.7% (93.6-97.3%)	99.6% (99.1-99.9%)
	HepB3	-	-	-	94.8% (92.4-96.6%)	99.4% (98.8-99.8%)
	Hib3	-	-	-	95.5% (93.4-97.2%)	99.5% (99.0-99.8%)
	MCV1	46.8% (22.8-74.3%)	81.1% (76.0-85.8%)	81.2% (76.1-85.1%)	95.8% (92.9-97.8%)	99.7% (99.0-99.9%)
	MCV2	-	-	-	95.5% (92.5-97.5%)	89.7% (82.9-94.2%)
	PCV3	-	-	-	-	-
	Pol3	42.9% (28.0-59.6%)	87.0% (84.6-89.0%)	90.8% (88.6-92.6%)	95.8% (93.6-97.4%)	99.6% (99.1-99.9%)
	RCV1	-	-	-	95.6% (92.6-97.6%)	99.5% (98.8-99.8%)
	RotaC	-	-	-	-	-
Costa Rica (CRI)	BCG	78.8% (71.6-84.6%)	89.7% (86.7-92.3%)	93.5% (90.0-96.0%)	99.2% (98.3-99.7%)	99.8% (99.4-100.0%)
	DTP1	85.8% (79.4-90.8%)	91.4% (88.5-93.9%)	89.0% (85.6-91.8%)	95.2% (92.7-97.5%)	98.0% (96.0-99.2%)
	DTP3	81.5% (75.3-86.5%)	87.2% (84.4-89.8%)	87.1% (83.7-89.9%)	90.6% (87.7-93.1%)	97.2% (94.6-98.7%)
	HepB3	-	-	84.8% (80.6-88.3%)	85.9% (79.6-90.0%)	97.0% (94.3-98.5%)

Country	Vaccine	1980	1990	2000	2010	2019
	Hib3	-	-	83.4% (79.6-86.8%)	89.0% (85.9-91.8%)	97.0% (94.4-98.6%)
	MCV1	71.4% (61.5-80.5%)	92.0% (87.3-95.5%)	88.7% (82.6-93.7%)	96.8% (93.3-98.7%)	99.8% (99.5-100.0%)
	MCV2	-	-	88.3% (82.2-93.2%)	95.1% (91.5-97.3%)	99.5% (99.1-99.8%)
	PCV3	-	-	-	87.1% (83.9-89.8%)	96.8% (94.2-98.3%)
	Pol3	79.3% (73.4-84.7%)	85.0% (81.9-87.7%)	87.3% (83.5-90.6%)	95.8% (93.7-97.2%)	98.8% (97.6-99.5%)
	RCV1	71.2% (61.2-80.2%)	91.8% (87.1-95.2%)	88.6% (82.5-93.5%)	96.4% (92.9-98.3%)	99.7% (99.4-99.9%)
	RotaC	-	-	-	-	60.7% (57.5-63.6%)
Cuba (CUB)	BCG	99.0% (97.6-99.7%)	98.9% (97.4-99.6%)	99.8% (99.5-99.9%)	99.5% (99.0-99.7%)	99.1% (98.0-99.7%)
	DTP1	77.2% (68.4-85.5%)	96.0% (93.6-98.0%)	94.8% (92.2-97.2%)	95.8% (93.9-97.3%)	95.7% (93.0-97.6%)
	DTP3	70.0% (62.6-76.7%)	90.1% (87.5-92.3%)	90.6% (88.3-92.7%)	94.4% (92.6-95.9%)	95.5% (92.6-97.4%)
	HepB3	-	89.2% (85.9-91.7%)	90.4% (88.0-92.4%)	94.1% (92.3-95.8%)	95.3% (92.5-97.2%)
	Hib3	-	-	79.4% (72.9-84.4%)	94.0% (92.2-95.6%)	95.3% (92.5-97.3%)
	MCV1	44.3% (35.0-54.4%)	87.0% (83.7-89.7%)	86.4% (82.8-89.4%)	92.1% (89.3-94.2%)	90.0% (86.1-92.9%)
	MCV2	-	-	-	89.2% (86.3-91.9%)	89.9% (86.0-92.8%)
	PCV3	-	-	-	-	-
	Pol3	-	-	-	-	-
	RCV1	-	86.8% (83.5-89.5%)	86.3% (82.7-89.3%)	92.0% (89.2-94.1%)	89.9% (86.0-92.8%)
	RotaC	-	-	-	-	-
Cyprus (CYP)	BCG	-	-	-	-	-
	DTP1	43.2% (34.2-54.6%)	95.7% (92.8-97.9%)	99.5% (99.0-99.8%)	99.5% (99.0-99.7%)	98.2% (96.5-99.3%)
	DTP3	36.1% (29.3-43.4%)	92.4% (88.1-95.3%)	98.6% (97.2-99.4%)	99.4% (98.9-99.7%)	95.5% (92.5-97.6%)
	HepB3	-	50.6% (23.9-74.0%)	89.3% (82.9-94.1%)	95.5% (91.7-97.6%)	93.3% (88.7-96.2%)
	Hib3	-	-	-	95.2% (92.3-97.1%)	92.3% (88.2-95.3%)
	MCV1	27.4% (19.2-36.6%)	79.7% (69.9-87.5%)	86.8% (79.4-92.0%)	85.5% (80.1-90.0%)	84.7% (76.9-90.7%)
	MCV2	-	79.3% (69.4-87.2%)	86.6% (79.3-91.9%)	85.4% (80.1-89.9%)	84.6% (76.8-90.7%)
	PCV3	-	-	-	49.6% (43.2-55.9%)	88.8% (85.4-91.6%)
	Pol3	33.4% (26.4-41.0%)	92.0% (87.8-95.2%)	98.3% (96.7-99.3%)	99.7% (99.3-99.9%)	98.2% (95.7-99.4%)
	RCV1	27.3% (19.2-36.5%)	79.5% (69.7-87.3%)	86.6% (79.3-91.9%)	85.4% (80.0-89.9%)	84.6% (76.8-90.6%)
	RotaC	-	-	-	-	-
Czech Republic (CZE)	BCG	96.0% (87.1-99.3%)	97.4% (94.2-99.0%)	98.3% (97.0-99.2%)	-	-
	DTP1	99.5% (98.1-99.9%)	99.6% (99.1-99.9%)	98.7% (97.7-99.3%)	99.3% (98.7-99.7%)	97.9% (95.4-99.4%)
	DTP3	96.8% (92.4-99.0%)	97.8% (95.6-99.0%)	97.9% (96.5-98.8%)	99.0% (98.1-99.5%)	96.7% (93.6-98.6%)
	HepB3	-	-	-	98.7% (97.6-99.3%)	96.6% (93.5-98.5%)
	Hib3	-	-	-	98.7% (97.8-99.3%)	96.6% (93.5-98.5%)
	MCV1	98.2% (94.3-99.7%)	98.8% (96.2-99.7%)	98.1% (95.6-99.4%)	98.9% (96.8-99.7%)	95.5% (90.9-98.3%)
	MCV2	96.7% (92.8-98.7%)	97.6% (94.9-98.9%)	97.4% (94.7-98.7%)	98.7% (96.5-99.5%)	86.7% (80.5-91.9%)
	PCV3	-	-	-	-	-
	Pol3	97.6% (94.2-99.3%)	98.3% (96.5-99.3%)	97.5% (95.8-98.6%)	99.3% (98.7-99.6%)	96.4% (93.4-98.2%)
	RCV1	-	98.5% (96.0-99.5%)	98.0% (95.4-99.2%)	98.8% (96.7-99.6%)	95.4% (90.8-98.2%)
	RotaC	-	-	-	-	-
Germany (DEU)	BCG	-	-	-	-	-
	DTP1	98.9% (97.6-99.6%)	82.4% (78.9-86.0%)	99.0% (98.2-99.6%)	99.0% (97.5-99.7%)	99.1% (97.3-99.9%)
	DTP3	93.3% (88.8-96.3%)	78.2% (74.1-82.1%)	95.9% (93.9-97.4%)	96.3% (92.7-98.3%)	96.8% (92.3-99.1%)
	HepB3	-	-	82.8% (76.3-87.6%)	88.5% (78.8-94.3%)	93.3% (84.7-97.7%)
	Hib3	-	69.0% (60.6-76.0%)	91.8% (87.8-94.4%)	94.5% (90.5-97.1%)	96.1% (91.7-98.5%)
	MCV1	35.4% (26.6-45.6%)	76.3% (70.1-81.8%)	94.5% (90.7-97.0%)	98.3% (95.9-99.5%)	98.4% (95.2-99.7%)
	MCV2	-	-	66.7% (59.0-72.9%)	94.1% (90.7-96.5%)	94.8% (90.8-97.4%)
	PCV3	-	-	-	62.9% (55.5-69.6%)	94.6% (90.2-97.0%)
	Pol3	86.9% (80.7-91.3%)	80.7% (76.2-84.7%)	95.3% (93.2-97.0%)	95.3% (92.6-97.2%)	94.6% (89.0-97.8%)
	RCV1	-	-	93.4% (89.6-95.9%)	98.1% (95.6-99.2%)	98.3% (95.1-99.6%)
	RotaC	-	-	-	-	79.2% (75.3-81.9%)
Djibouti (DJI)	BCG	41.7% (25.9-60.0%)	82.5% (77.6-86.5%)	43.0% (37.1-49.2%)	87.6% (82.6-91.6%)	93.4% (87.4-97.0%)
	DTP1	31.5% (16.9-53.3%)	89.9% (85.0-94.0%)	69.0% (61.6-75.8%)	80.8% (73.3-87.5%)	74.0% (63.0-84.2%)
	DTP3	13.6% (6.7-23.7%)	73.0% (66.1-79.1%)	54.9% (47.6-61.8%)	60.7% (54.9-66.5%)	56.7% (46.4-66.2%)

Country	Vaccine	1980	1990	2000	2010	2019
	HepB3	-	-	-	59.8% (53.8-65.5%)	56.5% (46.2-66.1%)
	Hib3	-	-	-	60.4% (54.7-66.3%)	56.6% (46.3-66.2%)
	MCV1	17.2% (7.3-31.9%)	75.4% (68.5-81.4%)	60.2% (52.5-67.2%)	84.1% (78.0-89.0%)	84.1% (74.2-90.7%)
	MCV2	-	-	-	-	80.1% (70.2-87.5%)
	PCV3	-	-	-	-	56.6% (46.3-66.2%)
	Pol3	14.7% (7.3-24.6%)	72.8% (66.2-79.0%)	41.1% (34.4-48.7%)	61.0% (54.4-67.0%)	55.2% (45.0-65.3%)
	RCV1	-	-	-	-	-
	RotaC	-	-	-	-	56.6% (46.3-66.2%)
Dominica (DMA)	BCG	54.2% (44.3-63.6%)	99.0% (97.9-99.6%)	99.7% (99.3-99.9%)	99.3% (98.4-99.8%)	97.8% (94.0-99.3%)
	DTP1	83.0% (75.5-89.6%)	95.1% (92.7-96.8%)	99.3% (98.6-99.7%)	98.8% (97.8-99.4%)	98.1% (96.6-99.1%)
	DTP3	77.5% (69.5-84.1%)	94.1% (91.5-96.1%)	97.5% (96.0-98.6%)	96.9% (95.2-98.0%)	97.0% (95.0-98.4%)
	HepB3	-	-	-	96.5% (94.9-97.8%)	96.9% (94.9-98.3%)
	Hib3	-	-	-	96.7% (95.1-97.7%)	96.9% (94.8-98.3%)
	MCV1	46.7% (31.4-63.9%)	93.3% (89.4-96.3%)	99.7% (99.1-99.9%)	99.6% (99.0-99.9%)	91.5% (85.6-95.3%)
	MCV2	-	-	-	98.8% (98.1-99.3%)	91.1% (85.3-95.0%)
	PCV3	-	-	-	-	-
	Pol3	67.0% (58.5-74.8%)	94.2% (91.4-96.0%)	97.1% (95.5-98.2%)	95.9% (94.0-97.3%)	96.7% (94.6-98.2%)
	RCV1	-	93.1% (89.2-96.1%)	99.5% (99.0-99.8%)	99.5% (98.9-99.8%)	91.4% (85.5-95.2%)
	RotaC	-	-	-	-	-
Denmark (DNK)	BCG	-	-	-	-	-
	DTP1	96.6% (94.6-98.0%)	97.5% (96.5-98.4%)	97.1% (95.8-98.1%)	93.0% (90.5-95.1%)	97.3% (95.3-98.6%)
	DTP3	86.9% (81.5-91.1%)	88.8% (85.6-91.5%)	96.8% (95.4-97.9%)	90.4% (87.0-92.9%)	96.9% (94.7-98.4%)
	HepB3	-	-	-	-	-
	Hib3	-	-	93.9% (91.0-95.9%)	90.2% (86.9-92.8%)	96.8% (94.5-98.3%)
	MCV1	73.1% (42.0-92.3%)	83.4% (78.2-88.1%)	96.9% (94.4-98.4%)	86.6% (81.1-91.2%)	96.6% (92.6-98.7%)
	MCV2	-	69.3% (58.9-77.8%)	88.6% (83.1-92.8%)	86.2% (80.6-90.8%)	89.8% (84.2-93.6%)
	PCV3	-	-	-	88.5% (85.5-91.2%)	96.7% (94.6-98.2%)
	Pol3	98.1% (96.3-99.1%)	98.2% (96.9-99.1%)	98.0% (96.7-98.9%)	90.6% (87.3-93.3%)	97.1% (94.9-98.5%)
	RCV1	-	83.3% (78.0-87.9%)	96.8% (94.2-98.3%)	86.4% (80.9-91.1%)	96.5% (92.5-98.6%)
	RotaC	-	-	-	-	-
Dominican Republic (DOM)	BCG	38.1% (30.6-45.9%)	69.0% (65.3-72.3%)	94.6% (92.8-96.0%)	92.1% (90.3-93.8%)	92.7% (89.1-95.2%)
	DTP1	66.7% (58.3-75.5%)	86.3% (83.1-89.3%)	93.6% (91.8-95.2%)	88.2% (86.2-90.2%)	96.8% (93.9-98.6%)
	DTP3	44.1% (35.7-53.5%)	63.1% (58.9-67.2%)	65.2% (61.3-69.1%)	72.8% (69.1-76.1%)	73.6% (65.6-80.5%)
	HepB3	-	-	44.1% (37.8-50.1%)	63.7% (56.4-69.1%)	71.3% (62.4-78.6%)
	Hib3	-	-	-	63.3% (54.9-70.0%)	66.9% (58.0-75.2%)
	MCV1	28.0% (19.8-38.6%)	86.3% (81.7-90.3%)	91.2% (86.8-94.7%)	77.8% (73.2-82.0%)	85.3% (77.3-91.3%)
	MCV2	-	-	-	-	44.9% (35.3-55.2%)
	PCV3	-	-	-	-	58.7% (51.7-65.6%)
	Pol3	34.7% (26.0-44.4%)	62.8% (57.5-68.1%)	48.4% (43.5-53.3%)	59.8% (55.2-64.1%)	70.0% (61.5-78.3%)
	RCV1	-	-	-	77.4% (72.8-81.7%)	85.2% (77.2-91.2%)
	RotaC	-	-	-	-	68.8% (61.4-75.4%)
Algeria (DZA)	BCG	63.0% (51.4-73.8%)	90.7% (87.0-93.6%)	98.9% (97.7-99.5%)	98.3% (97.0-99.1%)	98.1% (96.1-99.2%)
	DTP1	53.4% (40.4-68.2%)	90.3% (87.9-92.4%)	97.1% (95.9-98.0%)	97.6% (96.7-98.3%)	97.1% (95.0-98.6%)
	DTP3	37.0% (27.6-47.3%)	84.3% (81.3-87.1%)	94.2% (92.2-95.9%)	92.8% (90.9-94.3%)	90.9% (86.3-93.9%)
	HepB3	-	-	-	92.3% (90.2-93.9%)	90.8% (86.1-93.9%)
	Hib3	-	-	-	92.7% (90.8-94.1%)	90.7% (86.2-93.8%)
	MCV1	21.1% (11.6-33.1%)	81.1% (76.4-85.2%)	94.0% (90.8-96.4%)	93.1% (90.5-95.3%)	83.3% (73.8-90.4%)
	MCV2	-	-	93.3% (90.0-95.7%)	93.0% (90.3-95.1%)	82.7% (73.2-89.8%)
	PCV3	-	-	-	-	90.1% (85.6-93.2%)
	Pol3	33.0% (24.6-42.3%)	88.9% (87.1-90.6%)	91.8% (89.7-93.6%)	92.2% (90.7-93.5%)	91.9% (87.9-94.7%)
	RCV1	-	-	-	-	83.3% (73.7-90.3%)
	RotaC	-	-	-	-	-
Ecuador (ECU)	BCG	77.3% (69.9-83.6%)	95.4% (94.4-96.3%)	97.4% (96.5-98.2%)	97.0% (95.8-97.9%)	86.3% (80.5-90.7%)
	DTP1	26.7% (17.2-41.6%)	90.1% (87.2-92.8%)	95.1% (92.5-97.1%)	96.2% (94.3-97.8%)	81.8% (75.7-87.5%)

Country	Vaccine	1980	1990	2000	2010	2019
	DTP3	14.0% (9.8-18.7%)	80.6% (77.8-83.2%)	84.1% (81.3-86.6%)	92.9% (91.1-94.4%)	80.2% (73.6-85.9%)
	HepB3	-	-	28.9% (10.9-51.3%)	92.1% (89.8-94.0%)	80.0% (73.5-85.8%)
	Hib3	-	-	-	91.8% (90.0-93.6%)	80.0% (73.5-85.7%)
	MCV1	30.4% (22.4-39.1%)	80.2% (76.0-84.0%)	82.3% (78.2-86.2%)	89.1% (86.0-91.8%)	75.2% (66.7-82.8%)
	MCV2	-	-	-	83.7% (79.4-87.3%)	66.2% (57.4-74.6%)
	PCV3	-	-	-	65.1% (58.3-71.4%)	79.7% (73.2-85.4%)
	Pol3	26.3% (20.4-33.4%)	83.1% (80.7-85.2%)	85.1% (82.6-87.4%)	95.5% (94.0-96.9%)	80.0% (73.5-85.7%)
	RCV1	-	-	82.2% (78.1-86.1%)	89.0% (85.9-91.7%)	75.1% (66.7-82.7%)
	RotaC	-	-	-	92.7% (90.9-94.3%)	80.1% (73.5-85.8%)
Egypt (EGY)	BCG	45.1% (36.1-54.2%)	88.3% (87.3-89.1%)	98.8% (98.3-99.1%)	99.1% (98.6-99.5%)	96.8% (94.4-98.4%)
	DTP1	66.3% (54.9-77.8%)	89.8% (87.6-92.2%)	98.0% (96.9-98.8%)	98.4% (97.3-99.2%)	97.1% (94.4-98.7%)
	DTP3	52.1% (42.3-61.9%)	79.3% (77.1-81.3%)	92.5% (90.4-94.1%)	97.3% (95.5-98.5%)	95.9% (92.5-98.1%)
	HepB3	-	-	84.3% (80.4-87.5%)	95.4% (92.5-97.3%)	95.7% (92.2-97.9%)
	Hib3	-	-	-	-	95.8% (92.4-98.0%)
	MCV1	29.9% (17.2-44.4%)	82.1% (80.0-84.0%)	97.5% (96.1-98.6%)	96.5% (94.6-98.0%)	98.5% (96.0-99.6%)
	MCV2	-	-	96.4% (94.7-97.7%)	90.1% (86.2-93.1%)	97.7% (95.1-99.0%)
	PCV3	-	-	-	-	-
	Pol3	57.7% (46.2-69.4%)	79.8% (76.6-82.8%)	91.8% (89.1-93.9%)	97.3% (95.1-98.5%)	97.6% (94.6-99.1%)
	RCV1	-	-	56.6% (51.8-61.3%)	90.7% (88.3-92.7%)	98.4% (95.9-99.5%)
	RotaC	-	-	-	-	-
Eritrea (ERI)	BCG	19.3% (4.9-44.0%)	19.1% (12.1-27.7%)	90.5% (87.2-93.2%)	97.5% (94.6-99.0%)	99.7% (99.1-99.9%)
	DTP1	20.2% (8.2-41.1%)	20.8% (12.9-33.0%)	89.4% (85.9-92.6%)	95.3% (92.5-97.2%)	95.1% (91.4-97.7%)
	DTP3	11.9% (4.3-26.4%)	12.5% (8.1-18.1%)	83.8% (79.9-87.3%)	93.0% (89.8-95.7%)	91.3% (86.0-95.1%)
	HepB3	-	-	-	92.6% (89.3-95.2%)	91.2% (85.8-95.0%)
	Hib3	-	-	-	92.9% (89.7-95.5%)	91.2% (85.9-95.0%)
	MCV1	17.3% (4.2-45.9%)	15.5% (7.5-27.9%)	86.2% (82.3-89.9%)	92.0% (87.4-95.4%)	99.6% (98.7-99.9%)
	MCV2	-	-	-	-	91.0% (84.6-95.1%)
	PCV3	-	-	-	-	91.2% (85.9-95.1%)
	Pol3	14.7% (5.6-29.2%)	14.8% (9.8-20.8%)	84.8% (81.0-88.0%)	90.3% (86.7-92.9%)	86.6% (80.9-91.3%)
	RCV1	-	-	-	-	99.5% (98.6-99.8%)
	RotaC	-	-	-	-	91.2% (85.9-95.1%)
Spain (ESP)	BCG	-	-	-	-	-
	DTP1	93.4% (86.2-97.7%)	91.3% (88.3-93.8%)	98.3% (97.2-99.2%)	98.5% (97.2-99.4%)	97.9% (96.0-99.1%)
	DTP3	83.3% (72.4-91.1%)	86.4% (83.0-89.5%)	95.6% (93.4-97.2%)	96.8% (94.8-98.1%)	96.1% (93.0-98.1%)
	HepB3	-	-	76.9% (69.6-83.1%)	96.3% (94.3-97.8%)	95.7% (92.4-97.8%)
	Hib3	-	-	92.4% (89.3-95.0%)	96.6% (94.6-98.0%)	95.8% (92.7-97.7%)
	MCV1	15.9% (8.7-26.0%)	94.6% (91.6-96.6%)	96.1% (92.5-98.3%)	97.9% (95.3-99.3%)	98.8% (97.0-99.7%)
	MCV2	-	-	89.2% (82.7-93.8%)	92.6% (88.6-95.3%)	94.9% (91.5-97.1%)
	PCV3	-	-	-	-	93.8% (90.7-95.9%)
	Pol3	77.3% (65.8-86.3%)	89.6% (86.4-92.1%)	95.7% (93.7-97.3%)	97.0% (95.0-98.3%)	96.4% (93.2-98.2%)
	RCV1	15.9% (8.7-26.0%)	94.3% (91.4-96.4%)	96.0% (92.4-98.2%)	97.8% (95.1-99.1%)	98.7% (96.9-99.6%)
	RotaC	-	-	-	-	-
Estonia (EST)	BCG	96.8% (93.3-98.8%)	99.2% (98.2-99.7%)	99.8% (99.6-100.0%)	97.0% (95.0-98.4%)	92.6% (88.0-96.0%)
	DTP1	99.4% (98.8-99.8%)	87.8% (84.3-90.8%)	97.5% (96.5-98.2%)	96.4% (95.0-97.6%)	93.2% (89.5-95.9%)
	DTP3	81.8% (75.8-87.0%)	68.8% (62.7-74.0%)	91.4% (88.8-93.7%)	94.3% (92.1-96.2%)	91.5% (86.9-94.9%)
	HepB3	-	-	87.0% (79.4-91.4%)	94.0% (91.7-95.9%)	91.3% (86.8-94.7%)
	Hib3	-	-	-	94.0% (91.8-95.9%)	91.4% (86.7-94.8%)
	MCV1	72.2% (62.8-80.4%)	79.9% (72.2-86.0%)	94.5% (91.0-96.9%)	96.1% (92.9-98.1%)	89.7% (83.0-94.1%)
	MCV2	-	-	89.3% (84.4-93.0%)	95.9% (92.7-98.0%)	89.5% (82.8-93.9%)
	PCV3	-	-	-	-	-
	Pol3	82.8% (77.0-87.3%)	69.4% (63.7-74.3%)	91.7% (89.1-93.8%)	94.9% (92.9-96.6%)	92.3% (87.7-95.0%)
	RCV1	-	-	94.3% (90.8-96.8%)	96.0% (92.8-98.0%)	89.6% (82.9-94.0%)
	RotaC	-	-	-	-	86.5% (82.0-89.7%)
Ethiopia (ETH)	BCG	7.5% (3.2-14.4%)	32.7% (27.6-37.8%)	43.7% (39.4-47.8%)	70.7% (66.1-75.0%)	74.7% (66.5-81.4%)

Country	Vaccine	1980	1990	2000	2010	2019
	DTP1	16.5% (9.3-26.0%)	32.1% (27.1-37.2%)	36.3% (32.3-40.5%)	66.9% (62.9-71.0%)	88.4% (82.8-92.8%)
	DTP3	5.2% (2.2-9.8%)	22.8% (18.6-27.3%)	21.8% (18.7-25.4%)	46.1% (41.9-50.6%)	56.0% (48.6-62.8%)
	HepB3	-	-	-	43.4% (38.7-48.0%)	55.8% (48.4-62.6%)
	Hib3	-	-	-	44.1% (39.8-48.8%)	55.9% (48.5-62.7%)
	MCV1	6.3% (2.6-12.6%)	22.8% (17.5-28.2%)	25.0% (20.5-29.8%)	63.2% (57.8-68.3%)	59.0% (50.3-67.5%)
	MCV2	-	-	-	-	43.4% (33.8-52.7%)
	PCV3	-	-	-	-	55.6% (48.3-62.5%)
	Pol3	4.8% (2.4-8.9%)	24.8% (20.6-29.5%)	37.6% (33.7-41.3%)	59.2% (54.8-63.2%)	57.3% (50.3-64.4%)
	RCV1	-	-	-	-	-
	RotaC	-	-	-	-	55.3% (48.0-62.1%)
Finland (FIN)	BCG	88.6% (81.3-93.7%)	93.9% (90.3-96.7%)	98.7% (97.4-99.5%)	-	-
	DTP1	93.3% (88.6-96.6%)	96.1% (93.6-98.0%)	99.5% (98.9-99.8%)	98.9% (98.0-99.4%)	98.8% (97.1-99.7%)
	DTP3	91.2% (85.4-95.1%)	93.6% (90.2-96.0%)	98.7% (97.6-99.4%)	98.7% (97.7-99.3%)	90.6% (85.8-94.2%)
	HepB3	-	-	-	-	-
	Hib3	-	90.3% (85.2-93.7%)	95.3% (91.8-97.5%)	98.5% (97.5-99.1%)	90.4% (85.7-94.0%)
	MCV1	71.9% (58.8-82.9%)	97.9% (95.4-99.2%)	97.2% (94.5-98.9%)	98.9% (97.4-99.7%)	96.6% (93.1-98.7%)
	MCV2	-	85.2% (73.9-92.3%)	88.3% (80.9-93.8%)	91.9% (85.7-95.8%)	92.8% (88.1-95.9%)
	PCV3	-	-	-	70.5% (62.1-77.4%)	88.6% (83.8-92.1%)
	Pol3	82.4% (74.7-88.6%)	94.1% (91.1-96.3%)	95.0% (92.4-96.9%)	98.8% (98.0-99.3%)	90.7% (85.7-94.3%)
	RCV1	71.7% (58.6-82.6%)	97.7% (95.2-99.0%)	97.1% (94.4-98.7%)	98.8% (97.3-99.5%)	96.5% (93.0-98.6%)
	RotaC	-	-	-	76.6% (74.5-78.6%)	84.5% (80.1-87.9%)
Fiji (FJI)	BCG	99.4% (98.0-99.9%)	99.6% (98.8-99.9%)	99.5% (98.8-99.8%)	96.0% (94.2-97.4%)	97.9% (95.6-99.1%)
	DTP1	78.7% (71.6-84.8%)	94.4% (91.6-96.3%)	95.0% (92.4-97.0%)	94.0% (91.2-96.1%)	97.9% (95.6-99.1%)
	DTP3	71.3% (63.1-77.8%)	89.6% (85.3-92.7%)	89.5% (85.0-93.1%)	92.4% (88.9-95.1%)	96.9% (93.8-98.7%)
	HepB3	-	68.3% (47.5-82.0%)	87.9% (83.4-91.6%)	92.1% (88.5-94.7%)	96.8% (93.5-98.5%)
	Hib3	-	-	83.9% (77.7-88.5%)	92.2% (88.8-94.9%)	96.8% (93.6-98.5%)
	MCV1	40.6% (29.0-53.0%)	89.1% (82.0-94.0%)	94.0% (87.9-97.4%)	79.4% (69.6-87.0%)	90.0% (80.6-95.6%)
	MCV2	-	-	-	78.7% (69.0-86.1%)	87.7% (78.5-93.5%)
	PCV3	-	-	-	-	96.7% (93.6-98.4%)
	Pol3	61.8% (54.6-69.3%)	96.7% (94.9-98.0%)	99.0% (98.2-99.5%)	89.6% (85.2-93.2%)	98.0% (96.2-99.2%)
	RCV1	-	-	-	79.3% (69.5-86.9%)	89.9% (80.5-95.4%)
	RotaC	-	-	-	-	96.8% (93.7-98.5%)
France (FRA)	BCG	79.0% (67.5-88.1%)	76.8% (70.9-81.9%)	83.5% (77.8-88.0%)	-	-
	DTP1	84.6% (77.0-90.6%)	94.4% (92.1-96.1%)	98.4% (97.3-99.1%)	99.1% (98.4-99.5%)	99.3% (98.5-99.7%)
	DTP3	80.5% (71.7-87.8%)	92.2% (89.3-94.6%)	97.0% (95.1-98.3%)	98.9% (98.1-99.4%)	96.3% (93.2-98.2%)
	HepB3	-	-	27.1% (21.2-33.6%)	63.5% (56.0-69.9%)	91.4% (84.5-95.4%)
	Hib3	-	-	84.6% (79.6-88.9%)	97.1% (95.2-98.3%)	95.4% (92.1-97.5%)
	MCV1	17.3% (7.0-33.4%)	68.8% (62.0-75.2%)	85.4% (79.7-89.7%)	90.5% (85.7-94.1%)	91.3% (84.1-96.0%)
	MCV2	-	-	47.7% (31.9-62.8%)	64.7% (56.4-72.8%)	83.0% (75.3-89.0%)
	PCV3	-	-	-	86.2% (82.6-89.4%)	94.1% (90.9-96.1%)
	Pol3	82.1% (73.6-88.7%)	91.4% (88.1-94.1%)	97.9% (96.3-98.9%)	99.2% (98.5-99.6%)	96.6% (93.4-98.4%)
	RCV1	17.2% (7.0-33.3%)	68.7% (61.9-75.0%)	85.3% (79.5-89.6%)	90.4% (85.7-94.0%)	91.2% (84.0-95.9%)
	RotaC	-	-	-	-	-
Federated States of Micronesia (FSM)	BCG	15.3% (4.1-36.7%)	26.1% (16.9-37.3%)	40.8% (33.5-48.2%)	73.5% (67.1-79.6%)	79.4% (70.4-86.7%)
	DTP1	81.3% (64.8-93.3%)	93.0% (89.6-95.7%)	89.9% (86.0-93.1%)	93.0% (89.6-95.7%)	96.3% (92.9-98.6%)
	DTP3	63.2% (43.9-80.1%)	80.7% (74.6-85.8%)	79.8% (74.6-84.6%)	83.3% (78.4-87.5%)	75.6% (67.2-82.8%)
	HepB3	-	63.6% (51.6-72.9%)	79.6% (74.3-84.3%)	82.8% (78.0-87.0%)	75.5% (67.1-82.7%)
	Hib3	-	-	59.9% (48.8-68.9%)	67.9% (59.0-75.1%)	59.0% (48.4-68.5%)
	MCV1	68.1% (39.2-89.7%)	80.7% (72.7-86.7%)	82.7% (75.3-88.4%)	85.2% (78.5-90.6%)	75.2% (64.4-83.5%)
	MCV2	-	-	44.1% (33.8-54.4%)	74.9% (67.6-81.6%)	48.1% (37.6-58.0%)
	PCV3	-	-	-	61.4% (55.2-67.0%)	70.8% (62.9-78.0%)
	Pol3	56.2% (38.1-74.2%)	77.7% (72.3-82.6%)	80.6% (76.0-84.8%)	82.8% (78.7-86.6%)	74.4% (66.7-81.1%)
	RCV1	-	80.4% (72.4-86.3%)	82.5% (75.0-88.2%)	85.0% (78.5-90.4%)	75.1% (64.3-83.4%)
	RotaC	-	-	-	51.0% (47.2-54.8%)	47.2% (41.6-52.3%)

Country	Vaccine	1980	1990	2000	2010	2019
Gabon (GAB)	BCG	62.4% (40.6-81.0%)	97.9% (96.5-98.8%)	75.7% (70.4-80.5%)	89.9% (86.4-92.8%)	94.3% (88.7-97.4%)
	DTP1	46.0% (30.5-62.1%)	89.3% (85.8-92.2%)	59.4% (52.9-65.9%)	81.9% (77.8-85.6%)	80.0% (72.5-86.3%)
	DTP3	23.4% (13.5-36.1%)	68.6% (62.6-74.1%)	34.5% (29.4-39.8%)	61.7% (56.2-66.7%)	73.1% (63.9-81.1%)
	HepB3	-	-	-	55.6% (49.0-61.3%)	72.9% (63.7-80.8%)
	Hib3	-	-	-	53.3% (46.5-59.2%)	72.9% (63.6-80.8%)
	MCV1	52.4% (25.6-76.9%)	71.6% (64.3-78.4%)	51.8% (44.9-58.9%)	77.2% (71.8-82.4%)	68.7% (57.8-78.3%)
	MCV2	-	-	-	-	-
	PCV3	-	-	-	-	-
	Pol3	24.5% (12.8-40.2%)	63.8% (53.9-72.2%)	33.0% (24.7-41.9%)	45.4% (36.5-54.3%)	52.7% (39.2-66.6%)
	RCV1	-	-	-	-	-
	RotaC	-	-	-	-	-
UK (GBR)	BCG	-	-	-	-	-
	DTP1	54.7% (45.0-65.8%)	92.4% (89.8-94.7%)	94.4% (92.1-96.2%)	95.9% (93.7-98.0%)	96.1% (92.7-98.7%)
	DTP3	45.2% (36.0-54.9%)	85.4% (81.6-88.5%)	91.7% (89.1-94.0%)	94.0% (91.9-95.8%)	93.8% (90.2-96.4%)
	HepB3	-	-	-	-	93.6% (89.9-96.2%)
	Hib3	-	-	91.6% (89.0-93.8%)	93.9% (91.7-95.6%)	93.7% (90.1-96.2%)
	MCV1	51.6% (38.4-65.7%)	88.7% (83.9-92.3%)	88.1% (83.7-91.9%)	90.0% (85.4-93.3%)	92.8% (87.2-96.2%)
	MCV2	-	-	76.1% (69.0-81.8%)	83.6% (78.5-87.7%)	88.5% (82.5-92.7%)
	PCV3	-	-	-	87.3% (84.5-89.9%)	92.3% (88.7-94.9%)
	Pol3	71.7% (63.5-78.9%)	90.9% (87.9-93.2%)	92.1% (89.4-94.3%)	94.3% (92.0-96.0%)	94.1% (90.7-96.6%)
	RCV1	51.1% (38.2-64.5%)	83.1% (76.2-87.5%)	87.1% (83.4-90.2%)	89.9% (85.3-93.2%)	92.6% (87.1-95.7%)
	RotaC	-	-	-	-	91.4% (87.9-94.0%)
Georgia (GEO)	BCG	89.5% (82.0-94.6%)	76.4% (68.7-82.6%)	88.7% (85.0-91.7%)	93.5% (90.2-96.0%)	94.0% (88.5-97.3%)
	DTP1	72.6% (49.6-90.2%)	74.1% (63.2-83.8%)	91.9% (88.6-94.6%)	95.3% (92.6-97.3%)	96.6% (92.9-98.7%)
	DTP3	59.3% (35.0-80.8%)	64.6% (53.1-74.8%)	80.1% (75.8-84.0%)	83.8% (79.3-87.7%)	86.3% (80.0-91.4%)
	HepB3	-	-	33.3% (23.9-44.4%)	82.3% (77.8-86.4%)	86.1% (79.7-91.2%)
	Hib3	-	-	-	74.7% (67.0-80.8%)	86.1% (79.7-91.1%)
	MCV1	31.6% (10.0-64.5%)	36.1% (20.2-56.0%)	78.2% (73.6-82.3%)	78.2% (73.3-82.7%)	80.8% (74.2-86.4%)
	MCV2	-	1.5% (0.6-3.3%)	18.0% (11.7-26.2%)	70.1% (64.2-75.6%)	78.6% (72.2-84.0%)
	PCV3	-	-	-	-	77.5% (71.2-83.0%)
	Pol3	59.8% (36.6-80.8%)	68.1% (58.9-76.9%)	78.5% (75.4-81.5%)	76.8% (73.2-80.3%)	79.6% (74.4-84.2%)
	RCV1	-	-	-	78.1% (73.2-82.6%)	80.7% (74.1-86.3%)
	RotaC	-	-	-	-	76.8% (70.8-81.3%)
Ghana (GHA)	BCG	21.8% (15.1-30.0%)	88.5% (86.4-90.3%)	90.5% (88.7-92.2%)	96.3% (94.9-97.5%)	95.2% (91.8-97.6%)
	DTP1	32.6% (23.1-43.0%)	74.1% (69.0-79.5%)	87.5% (84.5-90.2%)	95.3% (93.5-96.6%)	95.2% (92.3-97.4%)
	DTP3	23.4% (16.7-30.7%)	59.8% (55.4-64.3%)	77.9% (74.4-81.1%)	85.7% (82.8-88.4%)	94.7% (91.3-97.0%)
	HepB3	-	-	-	85.6% (82.7-88.2%)	94.5% (91.1-96.9%)
	Hib3	-	-	-	85.5% (82.6-88.2%)	94.5% (91.2-97.0%)
	MCV1	31.0% (22.1-41.0%)	67.7% (61.3-73.5%)	84.7% (80.4-88.2%)	93.4% (90.4-95.9%)	92.2% (85.3-96.3%)
	MCV2	-	-	-	-	83.1% (75.7-88.6%)
	PCV3	-	-	-	-	94.5% (91.1-96.9%)
	Pol3	21.3% (15.2-28.4%)	59.9% (54.9-64.6%)	76.6% (72.8-80.2%)	82.0% (78.4-85.2%)	95.6% (92.1-97.7%)
	RCV1	-	-	-	-	92.1% (85.3-96.2%)
	RotaC	-	-	-	-	93.0% (89.6-95.3%)
Guinea (GIN)	BCG	15.3% (7.0-28.2%)	51.0% (44.1-58.3%)	76.9% (72.3-81.0%)	83.9% (80.3-87.1%)	72.4% (64.4-79.5%)
	DTP1	46.0% (29.8-63.5%)	42.4% (35.5-49.9%)	64.4% (60.1-69.1%)	80.9% (77.7-83.9%)	58.9% (50.6-66.5%)
	DTP3	14.4% (7.1-25.5%)	27.5% (21.2-34.5%)	44.1% (39.2-49.0%)	57.9% (53.3-62.4%)	46.0% (37.6-54.7%)
	HepB3	-	-	-	57.6% (53.1-62.0%)	46.0% (37.6-54.6%)
	Hib3	-	-	-	57.8% (53.2-62.3%)	46.0% (37.6-54.6%)
	MCV1	33.9% (18.7-52.0%)	32.7% (25.3-41.0%)	52.0% (45.6-57.6%)	63.2% (58.3-67.8%)	48.9% (39.2-58.9%)
	MCV2	-	-	-	-	-
	PCV3	-	-	-	-	-
	Pol3	14.4% (6.9-25.6%)	27.6% (21.0-34.8%)	41.4% (36.3-46.5%)	56.3% (51.2-60.9%)	44.1% (35.8-52.5%)
	RCV1	-	-	-	-	-

Country	Vaccine	1980	1990	2000	2010	2019
	RotaC	-	-	-	-	-
The Gambia (GMB)	BCG	87.6% (82.9-91.4%)	95.7% (94.0-97.0%)	96.1% (95.0-97.0%)	98.3% (97.4-98.9%)	94.4% (90.0-97.2%)
	DTP1	92.1% (86.6-96.2%)	96.1% (94.3-97.5%)	93.0% (90.7-94.8%)	95.6% (94.3-96.8%)	94.9% (91.6-97.0%)
	DTP3	78.3% (69.5-85.6%)	85.2% (81.8-88.0%)	78.9% (76.1-81.6%)	89.7% (87.5-91.5%)	90.8% (86.3-94.2%)
	HepB3	-	84.3% (81.0-87.2%)	77.2% (73.9-80.0%)	89.0% (86.4-91.0%)	90.7% (86.1-94.0%)
	Hib3	-	-	78.8% (76.0-81.5%)	89.6% (87.4-91.4%)	90.7% (86.3-94.1%)
	MCV1	70.6% (61.6-78.8%)	81.6% (76.6-86.1%)	92.1% (89.5-94.3%)	92.6% (89.9-94.9%)	85.3% (78.4-90.5%)
	MCV2	-	-	-	-	63.4% (53.9-71.3%)
	PCV3	-	-	-	79.9% (76.3-82.8%)	90.4% (86.0-93.8%)
	Pol3	59.3% (50.8-66.8%)	84.1% (80.4-87.4%)	77.9% (74.2-81.4%)	86.1% (82.6-89.0%)	90.3% (85.4-94.0%)
	RCV1	-	-	-	-	85.2% (78.2-90.4%)
	RotaC	-	-	-	-	90.7% (86.2-94.1%)
Guinea-Bissau (GNB)	BCG	39.1% (23.0-56.7%)	89.4% (86.3-92.1%)	82.8% (78.5-86.2%)	98.1% (97.2-98.8%)	84.5% (77.7-89.9%)
	DTP1	44.1% (28.6-62.2%)	82.0% (77.8-85.9%)	81.5% (76.0-86.2%)	95.2% (93.0-97.1%)	86.5% (80.5-91.6%)
	DTP3	18.0% (10.3-27.8%)	65.1% (60.3-69.7%)	57.1% (52.3-62.0%)	81.8% (78.3-85.1%)	72.8% (64.5-79.6%)
	HepB3	-	-	-	81.7% (78.2-85.0%)	72.7% (64.5-79.5%)
	Hib3	-	-	-	81.7% (78.2-85.0%)	72.7% (64.5-79.5%)
	MCV1	48.0% (22.7-75.4%)	64.5% (55.7-72.7%)	69.3% (60.1-77.6%)	78.2% (71.6-84.4%)	78.5% (67.6-86.8%)
	MCV2	-	-	-	-	-
	PCV3	-	-	-	-	72.7% (64.4-79.5%)
	Pol3	21.5% (12.4-33.6%)	59.9% (55.2-64.5%)	59.8% (55.1-64.8%)	78.5% (74.5-82.2%)	59.3% (50.6-67.8%)
	RCV1	-	-	-	-	-
	RotaC	-	-	-	-	72.7% (64.5-79.5%)
Equatorial Guinea (GNQ)	BCG	26.1% (10.9-47.8%)	52.3% (41.2-62.4%)	75.0% (67.3-82.0%)	69.9% (61.6-77.1%)	69.7% (57.1-80.5%)
	DTP1	19.3% (7.0-41.6%)	36.2% (23.8-51.9%)	54.1% (45.7-62.5%)	71.3% (62.5-79.0%)	64.1% (50.9-76.0%)
	DTP3	3.6% (1.1-8.9%)	14.7% (7.7-24.5%)	32.0% (24.0-40.7%)	50.7% (41.3-59.3%)	53.2% (38.9-65.9%)
	HepB3	-	-	-	-	53.1% (38.9-65.8%)
	Hib3	-	-	-	-	53.1% (38.9-65.8%)
	MCV1	17.5% (5.3-42.9%)	43.8% (31.2-57.4%)	43.7% (34.6-53.5%)	48.9% (40.3-57.7%)	43.4% (30.4-56.9%)
	MCV2	-	-	-	-	-
	PCV3	-	-	-	-	-
	Pol3	7.1% (2.5-15.6%)	27.2% (18.0-38.3%)	42.9% (35.7-50.3%)	43.2% (35.8-51.3%)	47.0% (35.5-59.2%)
	RCV1	-	-	-	-	-
	RotaC	-	-	-	-	-
Greece (GRC)	BCG	-	-	-	-	-
	DTP1	82.9% (76.6-88.3%)	73.7% (69.0-78.2%)	91.7% (87.0-95.0%)	99.6% (99.0-99.9%)	99.9% (99.6-100.0%)
	DTP3	76.4% (68.4-83.0%)	71.4% (66.3-76.1%)	91.1% (86.3-94.6%)	99.6% (99.0-99.8%)	99.9% (99.6-100.0%)
	HepB3	-	-	90.2% (85.4-93.8%)	95.7% (91.7-98.0%)	95.7% (91.0-98.3%)
	Hib3	-	-	91.0% (86.1-94.4%)	86.0% (81.3-90.0%)	98.9% (97.8-99.5%)
	MCV1	65.8% (42.5-85.5%)	75.4% (69.1-81.2%)	91.2% (82.8-96.1%)	99.8% (99.4-100.0%)	98.2% (95.0-99.5%)
	MCV2	-	-	61.5% (46.3-75.5%)	78.9% (73.5-83.4%)	82.9% (72.6-90.5%)
	PCV3	-	-	-	23.7% (18.5-29.4%)	96.4% (95.1-97.4%)
	Pol3	91.8% (86.8-95.4%)	93.0% (90.0-95.3%)	91.6% (86.7-95.2%)	99.7% (99.3-99.9%)	99.9% (99.6-100.0%)
	RCV1	-	-	90.0% (81.9-94.9%)	99.0% (98.5-99.3%)	98.1% (94.9-99.4%)
	RotaC	-	-	-	-	26.9% (24.8-29.2%)
Grenada (GRD)	BCG	-	-	-	-	-
	DTP1	41.6% (32.2-53.1%)	85.0% (80.2-89.1%)	96.8% (94.6-98.3%)	95.2% (92.4-97.1%)	96.1% (92.1-98.7%)
	DTP3	35.5% (27.5-44.3%)	80.0% (74.9-84.5%)	92.8% (89.7-95.3%)	95.0% (92.0-97.0%)	89.5% (83.8-93.9%)
	HepB3	-	-	92.6% (89.5-95.1%)	94.8% (91.8-96.8%)	89.4% (83.6-93.8%)
	Hib3	-	-	59.8% (45.3-72.5%)	94.8% (91.8-96.8%)	89.4% (83.7-93.8%)
	MCV1	11.4% (4.5-22.5%)	79.9% (72.6-85.6%)	94.2% (89.7-97.1%)	98.6% (96.8-99.5%)	95.6% (92.4-98.0%)
	MCV2	-	-	72.9% (61.9-81.4%)	85.8% (81.5-89.6%)	86.2% (80.1-91.0%)
	PCV3	-	-	-	-	-
	Pol3	40.7% (33.1-48.3%)	77.9% (73.2-81.9%)	91.0% (87.7-93.3%)	94.2% (91.6-96.2%)	93.7% (90.0-96.3%)

Country	Vaccine	1980	1990	2000	2010	2019
	RCV1	-	-	94.1% (89.6-97.0%)	98.5% (96.6-99.4%)	95.5% (92.3-97.9%)
	RotaC	-	-	-	-	-
Greenland (GRL)	BCG	91.6% (84.3-96.0%)	85.0% (68.5-94.8%)	90.1% (71.2-98.1%)	94.7% (83.7-99.2%)	96.7% (87.6-99.5%)
	DTP1	96.4% (94.6-98.0%)	97.2% (96.1-98.1%)	95.1% (93.1-96.6%)	92.4% (89.8-94.6%)	96.6% (94.2-98.1%)
	DTP3	86.3% (80.8-90.8%)	87.4% (83.9-90.3%)	94.8% (92.6-96.3%)	89.6% (86.1-92.3%)	96.1% (93.5-97.9%)
	HepB3	-	-	-	-	-
	Hib3	-	-	92.0% (89.2-94.4%)	89.5% (86.1-92.2%)	96.0% (93.4-97.7%)
	MCV1	76.3% (51.5-92.4%)	83.1% (77.2-87.5%)	97.1% (94.7-98.4%)	86.8% (81.2-91.2%)	96.9% (93.1-98.9%)
	MCV2	-	69.1% (58.2-77.7%)	88.8% (83.3-92.7%)	86.4% (80.9-90.8%)	90.2% (84.8-94.0%)
	PCV3	-	-	-	87.8% (84.4-90.5%)	95.9% (93.3-97.7%)
	Pol3	96.8% (94.6-98.2%)	96.8% (95.5-97.8%)	96.4% (94.9-97.5%)	89.7% (86.7-92.3%)	96.3% (93.8-98.0%)
	RCV1	-	83.0% (77.0-87.4%)	96.9% (94.6-98.3%)	86.7% (81.2-91.1%)	96.8% (93.0-98.8%)
	RotaC	-	-	-	-	-
Guatemala (GTM)	BCG	65.0% (57.1-72.0%)	87.2% (84.9-89.3%)	77.9% (74.9-80.9%)	97.4% (96.1-98.3%)	86.5% (79.4-91.8%)
	DTP1	73.8% (64.4-82.9%)	80.7% (75.3-85.8%)	87.8% (84.7-90.9%)	96.8% (95.4-98.0%)	89.0% (83.5-93.8%)
	DTP3	60.3% (51.9-67.7%)	64.6% (59.8-68.9%)	80.5% (76.8-83.7%)	92.2% (89.6-94.3%)	77.6% (70.6-83.7%)
	HepB3	-	-	-	92.1% (89.4-94.2%)	77.5% (70.5-83.6%)
	Hib3	-	-	-	92.1% (89.5-94.2%)	77.5% (70.5-83.7%)
	MCV1	28.4% (19.7-39.3%)	91.4% (87.3-94.6%)	78.6% (73.3-83.1%)	87.8% (82.7-92.0%)	84.2% (74.9-90.3%)
	MCV2	-	-	-	-	73.0% (64.1-80.8%)
	PCV3	-	-	-	-	77.5% (70.5-83.5%)
	Pol3	64.1% (57.6-70.8%)	57.4% (53.8-60.9%)	80.8% (78.0-83.3%)	93.1% (91.3-94.6%)	74.2% (67.3-80.1%)
	RCV1	-	-	-	87.7% (82.6-91.9%)	84.1% (74.8-90.2%)
	RotaC	-	-	-	61.0% (58.1-63.8%)	77.5% (70.5-83.6%)
Guam (GUM)	BCG	-	-	-	-	-
	DTP1	91.8% (77.6-98.1%)	98.7% (95.4-99.8%)	99.1% (97.0-99.9%)	98.8% (96.4-99.8%)	98.9% (96.8-99.8%)
	DTP3	90.1% (74.4-97.3%)	97.2% (91.6-99.3%)	98.4% (95.6-99.7%)	98.4% (95.3-99.6%)	98.4% (95.8-99.6%)
	HepB3	-	94.3% (85.9-98.3%)	98.1% (95.1-99.4%)	98.3% (95.2-99.5%)	98.3% (95.6-99.5%)
	Hib3	-	-	-	-	-
	MCV1	92.3% (76.1-98.4%)	97.9% (93.5-99.6%)	99.2% (97.6-99.9%)	99.0% (96.7-99.8%)	99.1% (96.9-99.8%)
	MCV2	-	-	-	-	-
	PCV3	-	-	-	-	-
	Pol3	88.1% (72.3-96.8%)	96.3% (90.7-99.1%)	98.2% (95.4-99.6%)	98.2% (94.9-99.5%)	98.1% (94.6-99.5%)
	RCV1	-	-	-	-	-
	RotaC	-	-	-	-	-
Guyana (GUY)	BCG	73.8% (64.5-81.8%)	87.6% (82.9-91.5%)	96.4% (94.5-97.7%)	94.5% (92.8-96.0%)	95.6% (92.5-97.6%)
	DTP1	54.6% (46.1-64.7%)	89.3% (86.3-92.2%)	95.7% (93.9-97.2%)	96.6% (95.3-97.7%)	93.5% (90.4-96.0%)
	DTP3	42.4% (34.9-50.2%)	79.6% (75.4-83.2%)	86.0% (83.4-88.6%)	90.1% (87.9-92.0%)	91.3% (88.1-94.0%)
	HepB3	-	-	-	89.9% (87.8-91.9%)	91.2% (88.0-93.9%)
	Hib3	-	-	-	90.0% (87.8-91.9%)	91.2% (88.0-93.9%)
	MCV1	65.3% (48.5-80.3%)	72.5% (65.6-79.1%)	91.6% (88.0-94.3%)	90.6% (87.7-93.3%)	94.3% (90.5-97.0%)
	MCV2	-	-	-	78.7% (73.8-82.9%)	86.6% (81.4-90.7%)
	PCV3	-	-	-	-	90.7% (87.4-93.4%)
	Pol3	46.0% (38.3-54.8%)	80.1% (75.8-83.7%)	82.6% (79.2-85.4%)	81.6% (78.1-84.6%)	90.8% (86.3-94.2%)
	RCV1	-	-	91.4% (87.8-94.1%)	90.3% (87.5-93.0%)	94.2% (90.4-96.8%)
	RotaC	-	-	-	45.9% (42.7-48.8%)	91.2% (88.0-93.9%)
Honduras (HND)	BCG	38.6% (31.3-46.7%)	90.2% (87.5-92.5%)	97.5% (96.7-98.2%)	99.0% (98.3-99.4%)	89.9% (83.9-94.3%)
	DTP1	44.0% (36.2-52.2%)	94.5% (92.8-96.0%)	97.2% (96.0-98.3%)	97.8% (96.6-98.7%)	88.8% (83.7-93.1%)
	DTP3	39.8% (32.6-47.0%)	92.4% (90.5-94.1%)	91.4% (89.8-92.8%)	95.9% (94.5-96.9%)	86.0% (80.5-90.4%)
	HepB3	-	-	91.2% (89.6-92.7%)	95.7% (94.3-96.8%)	85.9% (80.4-90.2%)
	Hib3	-	-	91.2% (89.7-92.7%)	95.8% (94.4-96.8%)	85.9% (80.4-90.3%)
	MCV1	46.9% (38.0-56.5%)	97.4% (95.5-98.6%)	87.4% (84.8-89.6%)	98.0% (96.0-99.1%)	90.0% (83.8-94.5%)
	MCV2	-	-	-	-	89.0% (82.7-93.5%)
	PCV3	-	-	-	-	85.9% (80.5-90.3%)

Country	Vaccine	1980	1990	2000	2010	2019
	Pol3	43.2% (35.6-50.7%)	90.2% (87.7-92.4%)	87.9% (85.8-89.9%)	95.9% (94.4-97.1%)	86.4% (80.7-90.9%)
	RCV1	-	-	87.0% (84.5-89.3%)	97.8% (95.9-99.0%)	89.9% (83.7-94.4%)
	RotaC	-	-	-	91.1% (89.8-92.3%)	85.9% (80.5-90.3%)
Croatia (HRV)	BCG	96.5% (90.0-99.2%)	97.4% (95.1-98.8%)	97.6% (95.6-98.8%)	99.2% (98.3-99.7%)	98.4% (96.5-99.5%)
	DTP1	84.3% (78.6-88.8%)	89.6% (86.9-91.9%)	95.3% (93.5-96.8%)	97.7% (96.6-98.6%)	96.5% (93.6-98.5%)
	DTP3	81.3% (75.2-86.6%)	87.4% (84.3-90.1%)	93.8% (91.5-95.6%)	96.4% (94.8-97.6%)	93.7% (90.3-96.3%)
	HepB3	-	-	93.6% (91.3-95.5%)	96.2% (94.6-97.4%)	91.0% (85.6-94.5%)
	Hib3	-	-	-	95.7% (93.9-97.1%)	93.4% (89.9-96.0%)
	MCV1	89.6% (83.3-94.0%)	93.6% (90.5-95.9%)	94.4% (91.0-96.8%)	96.9% (94.6-98.6%)	93.4% (88.7-96.7%)
	MCV2	89.5% (83.2-93.9%)	93.5% (90.4-95.8%)	94.3% (90.9-96.7%)	96.8% (94.4-98.5%)	93.3% (88.6-96.6%)
	PCV3	-	-	-	-	-
	Pol3	82.8% (76.8-87.6%)	87.2% (84.2-90.0%)	94.4% (92.3-96.1%)	96.7% (95.1-97.9%)	94.2% (90.4-96.8%)
	RCV1	89.5% (83.1-93.9%)	93.5% (90.4-95.8%)	94.3% (90.8-96.7%)	96.8% (94.5-98.5%)	93.3% (88.6-96.6%)
	RotaC	-	-	-	-	-
Haiti (HTI)	BCG	52.5% (42.3-62.5%)	76.6% (72.4-80.2%)	76.9% (73.2-80.1%)	86.2% (83.2-88.8%)	81.1% (73.7-87.6%)
	DTP1	24.9% (16.6-35.7%)	74.3% (68.9-79.7%)	79.3% (74.9-83.4%)	86.4% (83.1-89.3%)	78.5% (70.4-85.6%)
	DTP3	11.2% (7.4-16.2%)	51.8% (47.1-56.8%)	57.2% (52.9-61.7%)	68.7% (64.8-72.4%)	59.6% (51.4-67.5%)
	HepB3	-	-	-	-	59.1% (50.9-66.9%)
	Hib3	-	-	-	-	59.4% (51.2-67.3%)
	MCV1	10.5% (2.8-24.0%)	56.5% (46.2-66.7%)	70.5% (60.7-78.8%)	68.2% (59.3-76.2%)	72.0% (58.4-83.6%)
	MCV2	-	-	-	-	38.3% (28.2-48.6%)
	PCV3	-	-	-	-	17.5% (12.7-22.5%)
	Pol3	10.9% (6.5-17.0%)	51.8% (46.6-57.0%)	54.2% (49.8-58.9%)	63.3% (59.2-67.1%)	53.6% (45.1-62.0%)
	RCV1	-	-	-	62.0% (53.5-70.0%)	71.9% (58.4-83.5%)
	RotaC	-	-	-	-	57.3% (49.5-64.9%)
Hungary (HUN)	BCG	99.8% (99.4-100.0%)	99.8% (99.5-100.0%)	99.6% (98.8-99.9%)	99.3% (98.4-99.8%)	99.3% (98.2-99.8%)
	DTP1	99.8% (99.6-99.9%)	99.7% (99.5-99.8%)	98.9% (98.2-99.4%)	99.2% (98.4-99.7%)	99.3% (98.4-99.7%)
	DTP3	97.7% (96.2-98.7%)	98.0% (97.0-98.7%)	98.9% (98.1-99.4%)	99.2% (98.4-99.6%)	99.3% (98.3-99.7%)
	HepB3	-	-	-	-	-
	Hib3	-	-	96.6% (94.2-98.0%)	99.0% (98.2-99.5%)	99.2% (98.2-99.6%)
	MCV1	99.8% (99.5-100.0%)	99.8% (99.5-100.0%)	99.9% (99.6-100.0%)	99.9% (99.6-100.0%)	99.9% (99.5-100.0%)
	MCV2	-	99.7% (99.3-99.9%)	99.8% (99.5-99.9%)	99.7% (99.4-99.9%)	99.8% (99.4-99.9%)
	PCV3	-	-	-	77.5% (71.9-82.3%)	99.1% (98.2-99.6%)
	Pol3	96.7% (95.0-98.1%)	98.0% (96.9-98.7%)	99.2% (98.5-99.6%)	99.6% (99.0-99.8%)	99.6% (99.1-99.9%)
	RCV1	-	99.6% (99.3-99.8%)	99.7% (99.5-99.9%)	99.7% (99.5-99.9%)	99.8% (99.5-99.9%)
	RotaC	-	-	-	-	-
Indonesia (IDN)	BCG	46.7% (38.2-55.0%)	73.7% (71.3-75.9%)	81.1% (78.8-83.4%)	90.4% (88.5-92.0%)	93.6% (89.5-96.6%)
	DTP1	18.8% (9.5-33.7%)	73.9% (69.6-78.2%)	78.2% (74.0-82.0%)	87.9% (85.1-90.6%)	84.7% (79.4-89.6%)
	DTP3	5.9% (3.0-10.2%)	56.7% (53.9-59.6%)	60.4% (56.9-63.5%)	70.3% (67.5-72.9%)	77.5% (71.8-82.8%)
	HepB3	-	-	49.3% (43.6-53.9%)	46.9% (40.2-53.3%)	76.8% (71.2-82.2%)
	Hib3	-	-	-	-	77.3% (71.7-82.5%)
	MCV1	10.4% (3.5-22.0%)	62.0% (57.8-65.8%)	72.3% (67.6-76.2%)	83.9% (80.6-87.2%)	80.9% (72.3-87.7%)
	MCV2	-	-	-	80.9% (77.1-84.2%)	56.9% (45.3-68.0%)
	PCV3	-	-	-	-	1.5% (1.0-2.1%)
	Pol3	5.7% (2.7-10.0%)	57.7% (54.2-61.2%)	68.5% (64.7-72.0%)	74.2% (71.2-77.3%)	72.4% (65.7-78.4%)
	RCV1	-	-	-	-	76.1% (67.8-82.6%)
	RotaC	-	-	-	-	-
India (IND)	BCG	8.8% (4.7-14.6%)	57.1% (55.1-59.0%)	69.8% (67.4-72.1%)	88.1% (85.9-90.0%)	93.7% (90.1-96.5%)
	DTP1	40.5% (27.9-56.2%)	78.6% (71.8-85.9%)	69.7% (65.0-74.3%)	85.1% (81.8-88.4%)	90.2% (86.0-93.3%)
	DTP3	18.5% (12.3-27.0%)	48.1% (45.3-51.1%)	53.4% (50.4-56.3%)	73.1% (70.0-76.3%)	87.2% (82.4-90.8%)
	HepB3	-	-	-	23.5% (19.8-26.8%)	87.0% (82.1-90.6%)
	Hib3	-	-	-	-	86.6% (81.9-90.2%)
	MCV1	-	42.1% (39.1-45.3%)	55.1% (50.5-59.5%)	79.9% (75.4-83.7%)	94.6% (89.4-97.6%)
	MCV2	-	-	-	20.5% (14.2-27.9%)	80.3% (72.5-86.0%)

Country	Vaccine	1980	1990	2000	2010	2019
	PCV3	-	-	-	-	7.0% (5.1-9.3%)
	Pol3	8.2% (4.3-13.9%)	49.3% (45.0-53.3%)	70.1% (66.1-73.9%)	68.1% (63.7-72.3%)	79.5% (73.2-85.0%)
	RCV1	-	-	-	-	89.4% (85.7-92.0%)
	RotaC	-	-	-	-	38.4% (35.4-41.6%)
Ireland (IRL)	BCG	69.6% (51.4-84.0%)	83.2% (69.8-92.9%)	75.5% (61.7-86.5%)	38.5% (31.5-45.3%)	-
	DTP1	45.5% (38.1-52.8%)	69.1% (63.0-75.0%)	88.0% (84.9-90.9%)	95.5% (93.2-97.4%)	95.9% (92.7-98.1%)
	DTP3	35.5% (28.6-42.8%)	61.9% (54.9-68.3%)	84.5% (80.9-88.1%)	94.2% (91.9-96.0%)	94.7% (91.6-97.0%)
	HepB3	-	-	-	62.3% (43.8-76.9%)	93.7% (89.9-96.3%)
	Hib3	-	-	84.2% (80.6-87.7%)	94.0% (91.7-95.9%)	94.6% (91.5-96.9%)
	MCV1	15.0% (5.3-30.3%)	77.8% (66.2-87.4%)	77.4% (70.3-83.7%)	91.7% (86.3-95.6%)	92.7% (85.4-96.9%)
	MCV2	-	-	73.2% (66.1-79.8%)	89.3% (83.5-93.5%)	89.6% (82.5-94.4%)
	PCV3	-	-	-	65.2% (58.0-71.4%)	89.2% (85.7-92.1%)
	Pol3	73.3% (66.0-79.7%)	79.8% (74.3-84.9%)	84.6% (80.6-88.3%)	94.6% (92.4-96.3%)	95.1% (92.1-97.4%)
	RCV1	15.0% (5.3-30.3%)	77.6% (66.0-87.3%)	77.3% (70.2-83.7%)	91.6% (86.2-95.4%)	92.6% (85.3-96.8%)
	RotaC	-	-	-	-	90.0% (86.9-92.2%)
Iran (IRN)	BCG	8.3% (5.2-12.6%)	95.0% (92.9-96.6%)	98.1% (96.7-99.0%)	99.1% (98.2-99.6%)	99.0% (97.5-99.7%)
	DTP1	39.4% (29.7-52.1%)	92.1% (88.8-95.2%)	98.5% (97.5-99.2%)	97.8% (96.8-98.7%)	98.1% (96.6-99.0%)
	DTP3	32.4% (25.8-39.6%)	88.5% (85.5-90.9%)	97.6% (96.5-98.4%)	97.0% (96.0-97.9%)	97.9% (96.5-98.9%)
	HepB3	-	-	96.4% (94.9-97.6%)	96.6% (95.5-97.5%)	97.8% (96.3-98.8%)
	Hib3	-	-	-	-	97.8% (96.3-98.8%)
	MCV1	42.9% (33.9-52.3%)	83.1% (78.6-87.2%)	95.8% (94.0-97.2%)	98.6% (97.4-99.3%)	99.1% (97.8-99.8%)
	MCV2	-	80.5% (75.8-85.0%)	95.0% (93.1-96.4%)	97.8% (96.8-98.4%)	97.7% (96.2-98.6%)
	PCV3	-	-	-	-	-
	Pol3	43.8% (36.0-52.2%)	87.9% (85.1-90.5%)	97.4% (96.2-98.2%)	97.0% (95.8-97.9%)	97.8% (96.4-98.9%)
	RCV1	-	-	-	98.1% (97.2-98.7%)	98.8% (97.6-99.3%)
	RotaC	-	-	-	-	-
Iraq (IRQ)	BCG	77.2% (66.9-85.4%)	91.3% (87.9-94.0%)	92.8% (90.5-94.7%)	91.1% (88.5-93.2%)	96.7% (94.1-98.4%)
	DTP1	29.9% (20.2-42.0%)	89.8% (86.6-92.5%)	88.0% (85.1-90.4%)	92.3% (90.0-94.2%)	88.4% (83.3-92.6%)
	DTP3	15.9% (9.7-23.6%)	79.2% (74.0-83.8%)	73.9% (69.5-77.8%)	77.9% (73.3-82.0%)	73.2% (65.4-79.6%)
	HepB3	-	-	58.5% (48.5-67.1%)	77.6% (72.8-81.7%)	73.1% (65.4-79.5%)
	Hib3	-	-	-	-	73.0% (65.3-79.4%)
	MCV1	26.1% (10.3-48.7%)	76.2% (69.0-82.8%)	82.1% (75.4-87.1%)	77.2% (70.4-82.9%)	78.9% (68.4-86.9%)
	MCV2	-	50.2% (36.1-63.0%)	63.5% (52.3-72.1%)	76.9% (70.1-82.6%)	78.6% (68.2-86.6%)
	PCV3	-	-	-	-	34.8% (28.2-41.2%)
	Pol3	17.4% (11.2-25.5%)	79.0% (74.3-83.0%)	79.9% (76.2-83.1%)	81.0% (77.1-84.6%)	81.5% (75.4-86.6%)
	RCV1	-	52.9% (46.3-60.0%)	62.7% (55.9-68.4%)	75.8% (69.3-81.3%)	78.5% (68.2-86.6%)
	RotaC	-	-	-	-	4.6% (3.9-5.3%)
Iceland (ISL)	BCG	-	-	-	-	-
	DTP1	99.4% (98.6-99.8%)	99.9% (99.7-99.9%)	98.5% (97.4-99.3%)	98.0% (97.0-98.8%)	96.5% (94.0-98.3%)
	DTP3	97.7% (95.0-99.0%)	98.8% (97.8-99.5%)	96.9% (94.8-98.4%)	94.9% (92.7-96.6%)	90.7% (85.5-94.6%)
	HepB3	-	-	-	-	-
	Hib3	-	98.7% (97.7-99.3%)	96.8% (94.7-98.2%)	94.8% (92.5-96.5%)	90.7% (85.3-94.5%)
	MCV1	84.1% (69.0-93.6%)	99.6% (99.0-99.9%)	93.7% (89.6-96.7%)	94.1% (90.5-96.7%)	93.9% (88.0-97.4%)
	MCV2	80.1% (65.7-90.1%)	97.6% (95.5-98.8%)	92.3% (87.9-95.5%)	93.5% (89.9-96.0%)	93.8% (87.9-97.3%)
	PCV3	-	-	-	-	90.2% (85.0-94.0%)
	Pol3	98.5% (96.7-99.5%)	98.7% (97.4-99.4%)	96.6% (94.4-98.1%)	94.8% (92.4-96.6%)	91.1% (86.3-94.8%)
	RCV1	83.8% (68.8-93.3%)	99.4% (98.8-99.7%)	93.6% (89.5-96.6%)	94.0% (90.4-96.6%)	93.8% (87.9-97.3%)
	RotaC	-	-	-	-	-
Israel (ISR)	BCG	72.4% (61.9-80.7%)	-	-	-	-
	DTP1	95.0% (92.5-97.0%)	97.3% (96.2-98.2%)	96.3% (94.3-97.6%)	95.5% (93.5-97.1%)	98.7% (97.5-99.4%)
	DTP3	86.2% (80.8-90.9%)	93.0% (90.6-95.0%)	96.0% (94.0-97.4%)	95.2% (93.0-96.8%)	97.4% (95.2-98.7%)
	HepB3	-	-	95.8% (93.7-97.3%)	95.0% (92.7-96.6%)	96.2% (93.0-98.1%)
	Hib3	-	-	93.3% (90.1-95.5%)	95.0% (92.9-96.7%)	97.3% (95.1-98.6%)
	MCV1	83.5% (75.5-89.5%)	91.8% (87.7-94.9%)	97.9% (95.9-99.1%)	97.9% (95.5-99.2%)	99.2% (97.8-99.8%)

Country	Vaccine	1980	1990	2000	2010	2019
	MCV2	-	-	96.9% (94.7-98.3%)	94.5% (91.5-96.6%)	97.5% (95.5-98.8%)
	PCV3	-	-	-	92.0% (89.7-94.0%)	96.0% (93.9-97.4%)
	Pol3	87.3% (81.8-91.3%)	93.9% (91.6-95.8%)	94.2% (91.9-96.0%)	95.2% (93.2-96.9%)	97.6% (95.7-98.9%)
	RCV1	-	-	97.8% (95.7-99.0%)	97.8% (95.3-99.1%)	99.1% (97.7-99.7%)
	RotaC	-	-	-	62.1% (59.2-64.9%)	89.1% (86.8-90.7%)
Italy (ITA)	BCG	-	-	-	-	-
	DTP1	80.2% (62.3-92.2%)	87.9% (82.7-92.1%)	95.1% (93.7-96.2%)	99.8% (99.6-99.9%)	99.7% (99.3-99.9%)
	DTP3	78.1% (58.7-91.5%)	86.9% (81.2-91.4%)	94.8% (93.3-95.9%)	99.8% (99.5-99.9%)	99.7% (99.3-99.9%)
	HepB3	-	62.9% (47.3-75.4%)	94.3% (92.6-95.7%)	99.4% (98.7-99.8%)	98.7% (96.9-99.6%)
	Hib3	-	-	61.4% (55.7-67.1%)	99.3% (98.7-99.6%)	97.8% (95.4-99.0%)
	MCV1	33.6% (10.5-66.9%)	48.3% (38.5-58.4%)	70.7% (65.3-75.9%)	84.9% (80.7-88.8%)	87.8% (82.4-92.4%)
	MCV2	-	-	-	79.3% (73.2-84.1%)	82.7% (76.5-87.8%)
	PCV3	-	-	-	79.0% (73.9-83.6%)	97.0% (95.9-97.9%)
	Pol3	85.4% (77.2-91.6%)	90.6% (88.1-92.8%)	95.3% (94.1-96.3%)	95.0% (93.3-96.5%)	93.2% (89.8-96.1%)
	RCV1	33.3% (10.4-66.1%)	48.0% (38.3-58.0%)	70.5% (65.1-75.5%)	84.8% (80.6-88.5%)	87.6% (82.3-91.8%)
	RotaC	-	-	-	-	26.6% (24.2-29.1%)
Jamaica (JAM)	BCG	35.1% (26.7-44.5%)	95.0% (92.9-96.7%)	91.6% (88.8-93.8%)	99.4% (98.8-99.7%)	98.5% (96.2-99.6%)
	DTP1	36.3% (28.5-45.6%)	89.5% (85.9-92.3%)	96.7% (94.9-98.0%)	96.4% (94.6-97.9%)	96.7% (94.0-98.4%)
	DTP3	32.0% (24.8-39.5%)	87.8% (83.8-90.9%)	92.6% (90.0-94.8%)	92.1% (89.4-94.3%)	95.4% (91.9-97.5%)
	HepB3	-	-	-	91.8% (89.0-94.0%)	95.2% (91.8-97.4%)
	Hib3	-	-	-	91.8% (89.1-94.0%)	95.3% (91.8-97.4%)
	MCV1	18.7% (9.1-32.9%)	82.1% (76.0-87.4%)	91.8% (87.6-95.1%)	99.5% (98.7-99.8%)	99.8% (99.5-100.0%)
	MCV2	-	-	-	69.3% (59.9-77.6%)	97.5% (95.7-98.7%)
	PCV3	-	-	-	-	-
	Pol3	38.4% (30.2-46.9%)	79.8% (75.6-83.5%)	87.9% (84.9-90.4%)	91.9% (88.4-94.4%)	97.7% (95.7-98.9%)
	RCV1	-	-	91.7% (87.5-94.9%)	99.3% (98.5-99.7%)	99.7% (99.4-99.9%)
	RotaC	-	-	-	-	-
Jordan (JOR)	BCG	3.7% (1.6-7.1%)	22.2% (18.8-25.9%)	31.2% (28.2-34.2%)	97.5% (96.2-98.3%)	85.7% (80.2-90.0%)
	DTP1	38.7% (30.3-49.5%)	96.0% (94.7-97.1%)	99.3% (98.9-99.6%)	98.6% (97.9-99.0%)	89.9% (85.6-93.5%)
	DTP3	30.1% (24.2-36.1%)	93.9% (92.6-95.0%)	98.2% (97.4-98.9%)	97.7% (96.8-98.4%)	86.1% (82.2-89.3%)
	HepB3	-	6.5% (2.0-15.7%)	95.3% (91.3-97.4%)	97.5% (96.5-98.3%)	86.0% (82.1-89.2%)
	Hib3	-	-	-	97.4% (96.5-98.2%)	86.0% (82.2-89.2%)
	MCV1	17.8% (11.5-25.7%)	91.2% (88.1-93.6%)	96.9% (94.6-98.4%)	96.5% (94.2-97.9%)	78.3% (70.8-84.5%)
	MCV2	-	-	61.4% (49.1-72.7%)	93.6% (90.5-96.0%)	78.2% (70.7-84.4%)
	PCV3	-	-	-	-	-
	Pol3	31.9% (26.7-37.7%)	92.9% (91.2-94.3%)	98.7% (98.1-99.2%)	97.6% (96.7-98.3%)	79.8% (75.0-83.8%)
	RCV1	-	-	62.9% (58.0-67.0%)	92.7% (90.3-94.6%)	78.2% (70.7-84.4%)
	RotaC	-	-	-	-	84.3% (80.5-87.4%)
Japan (JPN)	BCG	87.2% (79.5-92.6%)	87.9% (82.2-91.7%)	95.7% (92.3-98.0%)	94.9% (92.3-96.9%)	97.1% (94.4-98.7%)
	DTP1	78.7% (71.3-85.6%)	94.1% (91.4-96.3%)	96.9% (94.8-98.4%)	98.6% (97.1-99.5%)	98.5% (96.5-99.5%)
	DTP3	68.8% (61.1-75.7%)	85.0% (80.5-88.8%)	86.4% (80.7-90.8%)	98.4% (96.6-99.3%)	98.3% (96.1-99.4%)
	HepB3	-	-	-	-	98.2% (95.9-99.3%)
	Hib3	-	-	-	-	97.9% (95.6-99.1%)
	MCV1	69.0% (58.0-78.6%)	70.5% (63.1-77.0%)	98.6% (96.8-99.5%)	95.8% (92.1-98.0%)	98.0% (95.2-99.4%)
	MCV2	-	-	-	94.1% (90.5-96.4%)	94.0% (90.4-96.6%)
	PCV3	-	-	-	-	98.0% (95.6-99.1%)
	Pol3	95.2% (90.7-97.8%)	91.6% (87.7-94.5%)	95.5% (93.3-97.2%)	97.3% (95.4-98.6%)	98.6% (97.0-99.5%)
	RCV1	-	-	98.3% (96.6-99.0%)	95.6% (92.0-97.8%)	97.8% (95.1-98.9%)
	RotaC	-	-	-	-	-
Kazakhstan (KAZ)	BCG	98.9% (96.8-99.8%)	99.2% (98.5-99.6%)	86.7% (84.9-88.4%)	98.2% (97.2-98.9%)	90.7% (84.5-95.0%)
	DTP1	60.8% (39.1-80.0%)	63.4% (54.2-74.2%)	94.1% (92.1-95.8%)	97.2% (96.0-98.2%)	97.9% (96.0-99.1%)
	DTP3	53.0% (30.3-74.8%)	55.3% (48.7-61.9%)	92.5% (90.5-94.3%)	95.7% (94.0-96.9%)	94.6% (91.6-96.9%)
	HepB3	-	-	87.7% (83.9-90.7%)	95.2% (93.3-96.6%)	94.5% (91.4-96.7%)
	Hib3	-	-	-	87.5% (82.6-91.4%)	94.5% (91.4-96.7%)

Country	Vaccine	1980	1990	2000	2010	2019
	MCV1	82.6% (58.9-95.7%)	88.9% (83.2-92.9%)	88.3% (85.9-90.5%)	93.0% (91.2-94.6%)	92.1% (89.4-94.2%)
	MCV2	-	-	88.2% (85.8-90.3%)	92.9% (91.0-94.5%)	91.4% (88.7-93.6%)
	PCV3	-	-	-	25.2% (19.5-31.4%)	86.1% (82.1-89.3%)
	Pol3	63.6% (41.5-82.6%)	67.8% (60.9-74.0%)	88.9% (86.7-90.8%)	89.5% (87.3-91.3%)	94.0% (91.0-96.4%)
	RCV1	-	-	-	92.9% (91.1-94.5%)	92.0% (89.3-94.0%)
	RotaC	-	-	-	-	-
Kenya (KEN)	BCG	82.9% (71.2-91.3%)	92.3% (90.7-93.6%)	88.9% (87.2-90.4%)	95.8% (94.5-96.8%)	89.4% (84.0-93.7%)
	DTP1	86.2% (76.7-93.3%)	88.7% (86.3-91.1%)	90.0% (87.7-92.1%)	94.8% (93.2-96.2%)	91.0% (86.3-94.6%)
	DTP3	73.6% (61.7-83.3%)	79.5% (76.6-82.1%)	75.5% (72.9-77.9%)	87.6% (84.8-90.0%)	87.2% (81.4-91.8%)
	HepB3	-	-	-	87.4% (84.6-89.8%)	86.9% (81.2-91.7%)
	Hib3	-	-	-	87.4% (84.6-89.8%)	87.1% (81.3-91.7%)
	MCV1	67.2% (43.3-86.5%)	70.4% (64.0-76.0%)	79.3% (74.9-83.4%)	87.8% (82.6-91.8%)	83.3% (73.3-90.7%)
	MCV2	-	-	-	-	53.2% (42.1-64.0%)
	PCV3	-	-	-	-	87.1% (81.3-91.7%)
	Pol3	73.1% (61.9-83.1%)	76.9% (73.2-80.2%)	68.9% (65.8-72.1%)	77.7% (74.5-80.9%)	77.5% (70.0-83.6%)
	RCV1	-	-	-	-	83.1% (73.2-89.9%)
	RotaC	-	-	-	-	84.4% (78.8-88.9%)
Kyrgyzstan (KGZ)	BCG	89.4% (72.7-97.0%)	93.2% (90.0-95.6%)	94.4% (92.8-95.8%)	98.3% (97.5-99.0%)	89.4% (86.0-92.1%)
	DTP1	84.4% (68.4-95.1%)	89.5% (83.7-94.7%)	87.5% (84.7-90.3%)	97.0% (95.3-98.3%)	95.1% (92.0-97.5%)
	DTP3	76.6% (56.2-90.4%)	83.3% (77.4-87.9%)	84.6% (82.0-86.8%)	89.7% (87.3-91.8%)	88.2% (83.7-91.5%)
	HepB3	-	-	29.9% (22.1-38.2%)	89.3% (86.9-91.5%)	88.1% (83.6-91.4%)
	Hib3	-	-	-	84.8% (81.3-87.8%)	87.6% (83.3-90.9%)
	MCV1	75.0% (42.9-93.3%)	83.1% (79.1-86.7%)	93.2% (90.8-95.1%)	97.0% (95.2-98.3%)	89.4% (84.7-92.9%)
	MCV2	-	82.6% (78.7-86.3%)	92.6% (90.1-94.5%)	96.4% (94.7-97.7%)	89.2% (84.6-92.8%)
	PCV3	-	-	-	-	87.5% (83.2-90.8%)
	Pol3	76.5% (57.3-90.3%)	82.9% (76.6-88.1%)	87.7% (84.7-90.1%)	82.8% (79.8-85.7%)	84.9% (80.6-88.5%)
	RCV1	-	-	-	96.9% (95.2-98.2%)	89.3% (84.6-92.8%)
	RotaC	-	-	-	-	81.4% (77.4-84.6%)
Cambodia (KHM)	BCG	43.5% (26.6-61.3%)	48.1% (42.4-53.4%)	79.0% (76.2-81.6%)	93.4% (91.1-95.3%)	90.0% (82.2-95.2%)
	DTP1	35.4% (20.8-52.7%)	54.3% (47.0-61.4%)	76.9% (72.4-81.6%)	93.6% (91.3-95.6%)	92.6% (87.4-96.2%)
	DTP3	20.7% (11.0-33.1%)	39.0% (32.4-45.3%)	59.0% (53.9-63.7%)	86.6% (82.9-90.3%)	91.0% (84.9-95.2%)
	HepB3	-	-	-	86.5% (82.8-90.2%)	90.8% (84.8-95.0%)
	Hib3	-	-	-	86.5% (82.7-90.2%)	90.9% (84.8-95.0%)
	MCV1	31.0% (12.4-54.7%)	39.9% (31.4-48.1%)	63.8% (57.0-69.9%)	86.2% (79.9-91.0%)	91.0% (82.6-96.2%)
	MCV2	-	-	-	-	81.1% (72.5-87.7%)
	PCV3	-	-	-	-	89.6% (83.6-93.8%)
	Pol3	24.4% (14.0-37.2%)	43.4% (37.5-49.3%)	61.5% (57.3-65.7%)	86.0% (82.1-89.2%)	98.1% (96.5-99.1%)
	RCV1	-	-	-	-	90.9% (82.5-96.1%)
	RotaC	-	-	-	-	-
Kiribati (KIR)	BCG	70.2% (59.7-79.2%)	93.2% (90.2-95.4%)	82.2% (74.7-88.4%)	84.0% (78.9-88.3%)	70.3% (63.5-76.5%)
	DTP1	36.0% (26.6-46.6%)	77.4% (69.3-85.1%)	75.2% (70.1-80.5%)	73.2% (67.9-78.7%)	81.5% (72.0-89.6%)
	DTP3	30.3% (22.2-39.0%)	60.6% (54.8-66.2%)	65.7% (60.6-70.7%)	63.1% (58.1-67.6%)	51.6% (44.8-58.5%)
	HepB3	-	30.8% (18.9-43.0%)	65.1% (59.9-69.7%)	62.1% (57.1-66.7%)	51.1% (44.3-58.0%)
	Hib3	-	-	-	62.9% (57.7-67.4%)	51.5% (44.7-58.4%)
	MCV1	19.9% (7.7-38.2%)	65.0% (55.3-73.2%)	71.4% (63.5-78.9%)	76.9% (69.1-83.6%)	63.5% (52.3-73.2%)
	MCV2	-	-	-	32.2% (23.3-42.3%)	62.7% (51.4-72.4%)
	PCV3	-	-	-	-	51.4% (44.6-58.3%)
	Pol3	15.0% (8.8-22.8%)	55.5% (49.3-61.6%)	56.4% (50.6-62.2%)	53.3% (48.2-58.3%)	42.8% (35.5-50.7%)
	RCV1	-	-	-	76.8% (69.0-83.4%)	63.4% (52.2-73.2%)
	RotaC	-	-	-	-	51.5% (44.7-58.4%)
Saint Kitts and Nevis (KNA)	BCG	66.7% (41.0-86.5%)	82.6% (72.2-90.2%)	98.4% (96.9-99.3%)	94.4% (90.9-96.9%)	98.9% (97.4-99.7%)
	DTP1	68.3% (60.4-76.1%)	99.6% (99.3-99.8%)	98.0% (96.7-98.8%)	96.8% (95.2-97.9%)	97.7% (95.9-98.9%)
	DTP3	63.1% (55.3-71.3%)	98.4% (97.3-99.1%)	97.8% (96.5-98.7%)	96.7% (95.1-97.8%)	96.0% (93.3-97.8%)
	HepB3	-	-	97.6% (96.2-98.6%)	96.5% (94.9-97.7%)	95.9% (93.2-97.7%)

Country	Vaccine	1980	1990	2000	2010	2019
	Hib3	-	-	66.7% (46.8-83.1%)	96.5% (94.9-97.7%)	95.9% (93.2-97.7%)
	MCV1	84.3% (68.0-94.4%)	94.4% (90.8-97.1%)	97.7% (95.4-99.1%)	99.2% (97.9-99.7%)	96.8% (92.9-98.9%)
	MCV2	-	-	97.6% (95.2-98.9%)	98.8% (97.5-99.4%)	96.7% (92.7-98.7%)
	PCV3	-	-	-	-	-
	Pol3	75.5% (67.9-81.8%)	97.4% (96.2-98.4%)	97.2% (95.8-98.3%)	95.3% (93.4-96.9%)	95.2% (92.0-97.3%)
	RCV1	-	94.2% (90.5-96.9%)	97.6% (95.2-98.9%)	99.1% (97.8-99.6%)	96.7% (92.8-98.8%)
	RotaC	-	-	-	-	-
South Korea (KOR)	BCG	44.8% (33.2-57.1%)	74.0% (67.5-79.7%)	80.3% (74.8-85.1%)	97.3% (95.5-98.5%)	96.1% (93.1-98.1%)
	DTP1	73.9% (66.1-81.0%)	79.2% (73.3-84.1%)	89.9% (86.5-92.7%)	95.5% (93.5-97.2%)	96.4% (93.7-98.2%)
	DTP3	69.9% (61.6-77.0%)	75.1% (69.1-80.5%)	88.8% (85.0-91.8%)	93.2% (90.4-95.5%)	95.9% (92.8-98.0%)
	HepB3	-	74.5% (68.5-79.9%)	83.1% (76.8-87.8%)	93.0% (90.3-95.3%)	95.7% (92.7-97.9%)
	Hib3	-	-	-	-	95.7% (92.6-97.9%)
	MCV1	4.6% (1.9-8.9%)	93.0% (88.1-96.6%)	89.9% (85.1-93.5%)	96.6% (93.4-98.5%)	96.9% (92.4-99.0%)
	MCV2	-	-	49.5% (38.6-60.4%)	96.2% (93.1-98.1%)	95.6% (91.3-97.8%)
	PCV3	-	-	-	-	95.7% (92.7-97.9%)
	Pol3	74.0% (66.3-80.6%)	77.9% (72.0-82.7%)	90.0% (86.6-92.7%)	91.5% (89.0-93.6%)	94.2% (90.8-96.5%)
	RCV1	4.6% (1.9-8.9%)	92.7% (87.8-96.3%)	89.8% (85.0-93.4%)	96.5% (93.3-98.4%)	96.8% (92.3-98.8%)
	RotaC	-	-	-	-	-
Kuwait (KWT)	BCG	-	-	-	94.7% (92.7-96.4%)	90.1% (85.8-93.5%)
	DTP1	82.7% (76.4-88.1%)	92.3% (89.8-94.5%)	97.5% (96.1-98.5%)	97.7% (95.9-98.7%)	97.6% (95.0-99.3%)
	DTP3	79.5% (72.3-85.3%)	90.6% (87.6-93.1%)	96.9% (95.3-98.1%)	97.6% (95.8-98.7%)	94.3% (90.1-97.0%)
	HepB3	-	83.0% (73.2-89.5%)	95.6% (92.5-97.2%)	97.4% (95.7-98.6%)	94.2% (90.1-96.9%)
	Hib3	-	-	96.8% (95.1-97.9%)	97.5% (95.7-98.6%)	94.2% (90.1-96.9%)
	MCV1	60.8% (50.1-70.5%)	82.8% (76.4-88.0%)	97.0% (94.4-98.5%)	96.6% (94.3-98.1%)	95.8% (91.7-98.2%)
	MCV2	-	-	80.3% (69.6-88.1%)	95.6% (93.5-97.2%)	94.5% (90.3-97.1%)
	PCV3	-	-	-	97.4% (95.7-98.5%)	94.3% (90.1-96.9%)
	Pol3	80.4% (73.8-85.7%)	91.6% (88.9-94.0%)	93.7% (91.2-95.7%)	96.3% (94.6-97.6%)	96.6% (94.0-98.3%)
	RCV1	-	80.0% (73.9-85.2%)	94.8% (92.3-96.4%)	96.5% (94.2-98.0%)	95.7% (91.6-98.1%)
	RotaC	-	-	-	-	47.5% (44.4-51.0%)
Laos (LAO)	BCG	6.2% (2.8-11.8%)	31.6% (26.3-36.9%)	66.3% (61.9-70.7%)	81.4% (77.5-84.9%)	85.0% (78.4-90.2%)
	DTP1	30.3% (15.0-50.8%)	36.3% (30.1-43.2%)	69.1% (64.6-73.7%)	71.6% (67.6-75.3%)	75.3% (69.2-81.0%)
	DTP3	4.5% (1.7-9.7%)	11.0% (7.5-15.4%)	42.7% (38.4-47.0%)	56.3% (52.0-60.4%)	63.6% (56.9-69.8%)
	HepB3	-	-	-	56.2% (52.0-60.3%)	63.6% (56.8-69.7%)
	Hib3	-	-	-	56.2% (52.0-60.4%)	63.6% (56.8-69.8%)
	MCV1	7.6% (2.0-20.7%)	27.4% (20.8-35.7%)	46.5% (39.6-53.7%)	71.5% (65.2-77.9%)	73.4% (63.2-82.5%)
	MCV2	-	-	-	-	58.4% (47.6-68.6%)
	PCV3	-	-	-	-	63.3% (56.5-69.5%)
	Pol3	8.9% (3.7-16.9%)	14.2% (10.1-19.9%)	48.7% (43.3-54.0%)	64.4% (59.4-69.2%)	60.2% (52.2-67.8%)
	RCV1	-	-	-	-	73.3% (63.1-82.5%)
	RotaC	-	-	-	-	-
Lebanon (LBN)	BCG	-	-	-	-	-
	DTP1	32.8% (18.1-55.0%)	89.7% (86.5-92.4%)	94.6% (92.9-96.1%)	91.0% (88.4-93.3%)	89.1% (85.3-92.6%)
	DTP3	16.0% (8.5-27.6%)	85.3% (81.3-88.7%)	87.7% (84.5-90.3%)	78.0% (75.2-80.8%)	85.3% (80.9-89.0%)
	HepB3	-	-	70.5% (59.9-78.5%)	77.8% (74.9-80.7%)	85.2% (80.9-88.9%)
	Hib3	-	-	-	78.0% (75.0-80.7%)	85.2% (80.9-88.9%)
	MCV1	44.6% (20.3-70.4%)	58.4% (51.9-64.3%)	85.5% (81.8-88.9%)	82.3% (78.3-85.6%)	79.2% (72.4-84.8%)
	MCV2	-	-	84.1% (80.4-87.6%)	73.6% (68.9-77.8%)	67.8% (60.1-74.8%)
	PCV3	-	-	-	-	79.3% (74.6-83.5%)
	Pol3	20.8% (11.9-32.3%)	84.8% (80.8-88.0%)	87.1% (84.0-89.8%)	78.4% (75.5-81.0%)	85.0% (80.7-88.7%)
	RCV1	-	-	77.3% (73.4-80.9%)	82.2% (78.2-85.5%)	79.1% (72.3-84.7%)
	RotaC	-	-	-	-	-
Liberia (LBR)	BCG	81.9% (72.3-88.9%)	67.7% (56.8-78.3%)	74.2% (68.9-79.2%)	90.0% (85.6-93.6%)	99.6% (99.0-99.9%)
	DTP1	42.3% (28.2-58.1%)	36.2% (26.3-47.5%)	75.6% (69.5-81.5%)	89.7% (85.8-92.9%)	95.2% (91.3-98.0%)
	DTP3	19.1% (9.7-31.3%)	23.4% (15.3-33.3%)	55.4% (48.4-63.1%)	68.8% (61.4-75.3%)	88.9% (81.4-94.3%)

Country	Vaccine	1980	1990	2000	2010	2019
	HepB3	-	-	-	68.7% (61.3-75.2%)	88.8% (81.3-94.2%)
	Hib3	-	-	-	68.7% (61.3-75.2%)	88.8% (81.4-94.2%)
	MCV1	35.5% (17.2-57.3%)	47.7% (29.3-66.6%)	71.0% (60.3-80.0%)	73.3% (62.4-82.5%)	92.9% (84.1-97.5%)
	MCV2	-	-	-	-	55.7% (41.8-68.6%)
	PCV3	-	-	-	-	88.7% (81.3-94.2%)
	Pol3	24.4% (14.8-35.5%)	35.2% (27.0-44.5%)	54.8% (49.1-60.4%)	68.0% (61.9-73.2%)	81.7% (72.5-88.1%)
	RCV1	-	-	-	-	-
	RotaC	-	-	-	-	79.4% (72.7-84.2%)
Libya (LBY)	BCG	75.8% (69.0-82.1%)	86.3% (83.5-88.9%)	90.2% (88.1-91.9%)	91.8% (90.0-93.5%)	91.1% (88.6-93.2%)
	DTP1	72.5% (65.4-79.2%)	89.4% (86.3-91.9%)	93.3% (91.3-95.1%)	87.8% (85.2-90.0%)	88.6% (85.3-91.3%)
	DTP3	65.3% (57.2-72.8%)	84.4% (80.4-87.6%)	86.0% (82.9-88.7%)	86.2% (83.5-88.6%)	82.1% (78.1-85.7%)
	HepB3	-	-	83.8% (78.1-87.4%)	86.1% (83.3-88.5%)	82.0% (78.0-85.6%)
	Hib3	-	-	-	85.4% (82.6-87.8%)	82.0% (77.9-85.5%)
	MCV1	65.5% (56.1-74.4%)	87.3% (82.4-91.3%)	86.7% (82.3-90.4%)	92.7% (89.5-95.3%)	87.7% (83.5-91.6%)
	MCV2	-	-	75.8% (67.5-82.0%)	91.7% (88.5-94.3%)	86.8% (82.6-90.6%)
	PCV3	-	-	-	-	81.7% (77.7-85.3%)
	Pol3	64.5% (56.5-71.7%)	83.7% (80.4-86.6%)	89.6% (86.7-91.9%)	89.4% (87.0-91.5%)	85.8% (82.3-88.9%)
	RCV1	-	-	84.9% (80.5-88.8%)	92.6% (89.4-95.1%)	87.6% (83.4-91.5%)
	RotaC	-	-	-	-	82.0% (78.0-85.6%)
Saint Lucia (LCA)	BCG	35.5% (26.2-45.6%)	92.1% (88.2-95.0%)	93.1% (88.8-96.0%)	97.9% (95.9-99.1%)	94.9% (89.8-97.8%)
	DTP1	72.2% (64.1-80.0%)	96.9% (95.2-98.3%)	78.1% (71.8-83.8%)	97.6% (96.1-98.6%)	97.7% (95.4-99.2%)
	DTP3	62.6% (54.9-70.1%)	90.2% (87.0-92.8%)	69.5% (63.0-75.8%)	95.1% (92.7-96.8%)	88.7% (83.5-93.0%)
	HepB3	-	-	-	94.9% (92.4-96.7%)	88.6% (83.3-92.9%)
	Hib3	-	-	-	94.9% (92.4-96.7%)	88.6% (83.4-92.9%)
	MCV1	37.1% (21.9-54.7%)	85.1% (79.1-90.0%)	91.3% (86.3-95.1%)	96.7% (93.3-98.6%)	81.4% (68.6-90.4%)
	MCV2	-	-	90.3% (85.4-94.3%)	65.9% (57.3-74.0%)	64.7% (51.8-75.1%)
	PCV3	-	-	-	-	-
	Pol3	62.2% (53.6-69.7%)	89.3% (85.7-92.3%)	82.9% (77.9-87.2%)	94.9% (92.3-96.7%)	91.8% (87.4-95.0%)
	RCV1	-	84.9% (78.9-89.8%)	91.1% (86.2-94.9%)	96.6% (93.2-98.5%)	81.3% (68.5-90.4%)
	RotaC	-	-	-	-	-
Sri Lanka (LKA)	BCG	93.3% (89.3-96.2%)	98.7% (97.8-99.3%)	89.2% (87.5-90.9%)	97.9% (97.1-98.6%)	98.6% (97.1-99.5%)
	DTP1	80.1% (72.9-87.1%)	98.6% (97.9-99.1%)	99.3% (98.8-99.6%)	97.4% (96.4-98.2%)	97.7% (96.3-98.7%)
	DTP3	73.4% (66.9-79.1%)	98.2% (97.4-98.8%)	98.8% (98.1-99.3%)	96.7% (95.4-97.7%)	97.3% (95.6-98.4%)
	HepB3	-	-	-	96.5% (95.2-97.5%)	97.2% (95.4-98.3%)
	Hib3	-	-	-	96.6% (95.2-97.5%)	97.2% (95.5-98.3%)
	MCV1	-	96.2% (93.2-98.1%)	94.9% (91.7-97.1%)	95.2% (92.2-97.2%)	91.8% (86.4-95.5%)
	MCV2	-	-	-	94.6% (91.6-96.7%)	91.6% (86.2-95.3%)
	PCV3	-	-	-	-	-
	Pol3	73.3% (66.7-79.2%)	98.0% (97.1-98.7%)	98.9% (98.3-99.3%)	96.6% (95.4-97.6%)	96.9% (95.4-98.1%)
	RCV1	-	-	94.1% (91.1-96.4%)	94.8% (91.8-96.8%)	91.6% (86.2-95.3%)
	RotaC	-	-	-	-	-
Lesotho (LSO)	BCG	83.5% (76.1-89.3%)	77.3% (71.3-82.6%)	85.2% (80.4-88.9%)	93.7% (90.7-95.9%)	98.6% (97.0-99.5%)
	DTP1	73.9% (63.5-83.8%)	88.2% (84.1-91.7%)	86.7% (82.8-90.6%)	93.4% (90.4-96.0%)	90.2% (84.9-94.8%)
	DTP3	57.9% (49.3-66.3%)	84.5% (80.0-88.1%)	78.9% (74.9-82.3%)	84.5% (81.0-87.6%)	85.7% (79.5-91.0%)
	HepB3	-	-	-	84.3% (80.6-87.3%)	85.6% (79.5-90.9%)
	Hib3	-	-	-	84.3% (80.7-87.2%)	85.6% (79.4-90.9%)
	MCV1	49.0% (38.2-60.0%)	89.4% (83.9-93.4%)	78.9% (74.2-83.5%)	89.9% (84.9-93.9%)	90.5% (84.2-95.3%)
	MCV2	-	70.9% (58.4-80.6%)	61.9% (51.0-70.7%)	87.9% (82.9-91.8%)	73.3% (63.4-81.8%)
	PCV3	-	-	-	-	85.2% (79.0-90.4%)
	Pol3	56.9% (47.0-66.5%)	88.2% (83.8-91.8%)	74.4% (69.0-79.7%)	73.7% (68.2-78.8%)	85.8% (78.0-91.6%)
	RCV1	-	-	-	-	90.4% (84.1-95.2%)
	RotaC	-	-	-	-	61.2% (56.6-65.5%)
Lithuania (LTU)	BCG	98.0% (95.3-99.5%)	92.8% (88.7-95.6%)	99.7% (99.3-99.9%)	99.4% (98.5-99.8%)	97.1% (94.3-98.8%)
	DTP1	83.9% (68.6-94.0%)	83.8% (79.2-88.2%)	98.1% (97.0-98.9%)	96.9% (95.3-98.1%)	96.3% (93.5-98.1%)

Country	Vaccine	1980	1990	2000	2010	2019
	DTP3	76.8% (59.0-89.4%)	78.2% (73.4-82.7%)	93.3% (91.0-95.3%)	94.5% (92.3-96.3%)	92.7% (88.0-95.7%)
	HepB3	-	-	90.9% (87.7-93.5%)	93.8% (91.2-95.7%)	92.5% (87.9-95.6%)
	Hib3	-	-	-	94.2% (92.0-96.0%)	92.5% (88.0-95.6%)
	MCV1	91.1% (75.4-97.9%)	90.7% (85.7-94.1%)	98.6% (96.9-99.6%)	96.9% (94.2-98.5%)	94.3% (89.6-97.4%)
	MCV2	-	-	98.1% (96.3-99.1%)	96.1% (93.3-97.7%)	94.1% (89.4-97.2%)
	PCV3	-	-	-	-	84.2% (79.2-88.1%)
	Pol3	80.1% (63.7-91.1%)	81.2% (76.4-85.8%)	92.0% (89.4-94.3%)	94.5% (92.4-96.3%)	93.1% (88.8-96.0%)
	RCV1	-	-	98.5% (96.7-99.4%)	96.8% (94.1-98.4%)	94.2% (89.5-97.3%)
	RotaC	-	-	-	-	16.6% (14.9-18.6%)
Luxembourg (LUX)	BCG	-	-	-	-	-
	DTP1	72.4% (60.6-83.2%)	92.2% (88.6-95.2%)	98.8% (97.9-99.4%)	99.7% (99.3-99.9%)	99.3% (98.5-99.7%)
	DTP3	69.3% (56.3-80.0%)	90.4% (86.4-93.4%)	98.6% (97.4-99.2%)	99.1% (98.4-99.5%)	99.2% (98.4-99.6%)
	HepB3	-	-	87.6% (79.2-93.5%)	93.9% (90.4-96.3%)	95.3% (90.1-98.0%)
	Hib3	-	-	97.0% (95.1-98.2%)	98.9% (98.1-99.4%)	99.0% (98.2-99.5%)
	MCV1	38.2% (20.3-57.8%)	81.2% (75.9-85.9%)	94.4% (88.9-97.7%)	97.0% (95.1-98.5%)	98.6% (96.9-99.5%)
	MCV2	-	-	73.7% (60.0-84.4%)	83.2% (76.1-88.9%)	89.1% (84.7-92.8%)
	PCV3	-	-	-	88.1% (85.4-90.4%)	97.4% (96.4-98.2%)
	Pol3	88.3% (79.9-94.1%)	90.4% (86.7-93.4%)	98.3% (97.1-99.2%)	97.3% (95.7-98.4%)	99.1% (98.0-99.6%)
	RCV1	-	81.0% (75.7-85.7%)	94.2% (88.8-97.6%)	96.9% (94.9-98.3%)	98.5% (96.8-99.4%)
	RotaC	-	-	-	72.8% (70.6-75.0%)	94.3% (93.3-95.0%)
Latvia (LVA)	BCG	78.7% (53.2-93.2%)	83.4% (78.1-87.9%)	91.8% (90.4-93.0%)	85.3% (82.7-87.7%)	87.2% (83.6-90.6%)
	DTP1	99.5% (98.7-99.8%)	97.0% (95.4-98.1%)	98.4% (97.5-99.1%)	99.8% (99.7-99.9%)	99.9% (99.7-100.0%)
	DTP3	93.5% (88.9-96.4%)	87.0% (83.6-89.9%)	94.1% (91.8-95.9%)	99.7% (99.3-99.9%)	99.8% (99.6-100.0%)
	HepB3	-	-	89.0% (80.8-93.4%)	97.5% (93.0-99.3%)	99.7% (99.3-99.9%)
	Hib3	-	-	76.2% (69.3-81.6%)	98.9% (98.0-99.5%)	99.7% (99.5-99.9%)
	MCV1	99.0% (97.0-99.8%)	98.6% (97.0-99.5%)	98.0% (94.9-99.3%)	97.2% (94.3-98.7%)	99.5% (98.7-99.9%)
	MCV2	-	93.4% (88.7-96.5%)	93.4% (89.3-96.4%)	96.5% (93.6-98.0%)	95.6% (92.5-97.6%)
	PCV3	-	-	-	73.9% (67.4-79.7%)	89.1% (85.8-91.7%)
	Pol3	95.1% (91.7-97.4%)	89.6% (86.3-92.4%)	94.4% (92.3-96.2%)	95.9% (93.7-97.5%)	99.5% (98.9-99.8%)
	RCV1	-	-	97.4% (94.4-98.8%)	96.4% (93.6-98.0%)	99.4% (98.6-99.8%)
	RotaC	-	-	-	-	85.9% (84.5-87.3%)
Morocco (MAR)	BCG	70.9% (60.8-79.2%)	92.0% (90.2-93.5%)	97.8% (96.5-98.7%)	98.7% (97.5-99.4%)	98.2% (96.0-99.3%)
	DTP1	62.6% (51.8-73.5%)	88.7% (85.4-91.6%)	97.1% (95.4-98.4%)	96.8% (94.6-98.3%)	95.0% (91.0-97.7%)
	DTP3	51.2% (40.2-61.2%)	81.1% (76.7-84.9%)	94.1% (90.8-96.7%)	96.1% (93.3-97.9%)	94.2% (89.7-97.1%)
	HepB3	-	-	42.8% (31.8-54.3%)	95.3% (92.2-97.4%)	94.0% (89.5-97.0%)
	Hib3	-	-	2.2% (0.9-4.6%)	95.5% (92.8-97.4%)	94.0% (89.5-97.0%)
	MCV1	37.5% (22.3-55.9%)	82.1% (76.5-86.9%)	92.0% (87.0-95.7%)	93.4% (88.4-96.7%)	92.7% (85.7-96.7%)
	MCV2	-	-	-	-	92.6% (85.5-96.6%)
	PCV3	-	-	-	8.4% (6.4-10.9%)	93.8% (89.2-96.8%)
	Pol3	52.9% (43.7-62.2%)	81.4% (77.2-85.1%)	94.0% (90.8-96.4%)	96.1% (93.3-98.0%)	95.2% (90.8-97.9%)
	RCV1	-	-	-	-	92.6% (85.6-96.6%)
	RotaC	-	-	-	20.5% (18.6-22.6%)	93.9% (89.4-96.8%)
Monaco (MCO)	BCG	98.5% (95.2-99.8%)	98.4% (96.3-99.5%)	95.8% (92.1-98.1%)	91.9% (78.7-97.8%)	93.0% (78.2-98.8%)
	DTP1	99.4% (98.4-99.9%)	99.3% (98.4-99.8%)	99.2% (98.2-99.7%)	99.2% (98.1-99.8%)	99.2% (98.0-99.8%)
	DTP3	98.8% (96.9-99.7%)	98.9% (97.5-99.6%)	99.2% (98.1-99.7%)	99.2% (98.0-99.8%)	99.2% (97.9-99.8%)
	HepB3	-	-	99.0% (97.8-99.6%)	99.1% (98.0-99.7%)	99.1% (97.9-99.8%)
	Hib3	-	-	99.0% (97.9-99.6%)	99.1% (98.0-99.7%)	99.1% (97.9-99.8%)
	MCV1	99.8% (99.2-100.0%)	99.8% (99.3-100.0%)	99.9% (99.5-100.0%)	89.7% (75.0-97.5%)	89.2% (82.6-94.1%)
	MCV2	-	-	84.5% (72.9-92.2%)	77.2% (61.5-87.9%)	80.2% (72.1-87.2%)
	PCV3	-	-	-	91.2% (88.1-93.8%)	98.5% (97.3-99.2%)
	Pol3	99.3% (98.2-99.8%)	99.3% (98.4-99.8%)	99.5% (98.9-99.9%)	99.6% (98.8-99.9%)	99.5% (98.8-99.9%)
	RCV1	99.1% (98.6-99.4%)	99.4% (98.9-99.6%)	99.6% (99.2-99.7%)	89.6% (74.9-97.3%)	89.1% (82.5-94.0%)
	RotaC	-	-	-	-	-
Moldova (MDA)	BCG	93.6% (88.9-96.8%)	97.3% (95.3-98.6%)	99.6% (99.2-99.8%)	98.2% (97.3-98.9%)	95.3% (92.0-97.6%)

Country	Vaccine	1980	1990	2000	2010	2019
	DTP1	82.2% (65.8-93.6%)	83.9% (76.3-90.0%)	97.8% (96.5-99.0%)	94.7% (92.4-96.6%)	93.4% (89.7-96.0%)
	DTP3	79.4% (61.5-92.3%)	81.4% (73.4-87.8%)	95.0% (93.4-96.3%)	90.7% (88.2-92.8%)	92.2% (88.2-95.2%)
	HepB3	-	-	93.1% (90.1-95.1%)	90.5% (88.1-92.7%)	92.1% (88.1-95.0%)
	Hib3	-	-	-	64.0% (53.9-72.4%)	91.8% (87.8-94.8%)
	MCV1	94.6% (89.8-97.4%)	96.8% (94.1-98.5%)	96.5% (93.7-98.4%)	90.3% (87.2-92.9%)	89.8% (85.0-93.5%)
	MCV2	-	-	-	90.2% (87.0-92.7%)	89.1% (84.4-92.9%)
	PCV3	-	-	-	-	84.1% (79.5-87.7%)
	Pol3	93.7% (90.5-95.8%)	90.7% (87.9-93.2%)	96.2% (95.1-97.2%)	92.4% (90.5-94.0%)	92.6% (89.3-95.1%)
	RCV1	-	-	-	90.2% (87.1-92.7%)	89.7% (85.0-93.4%)
	RotaC	-	-	-	-	73.5% (70.0-76.3%)
Madagascar (MDG)	BCG	20.9% (14.3-29.6%)	74.6% (70.5-78.2%)	74.3% (70.5-77.9%)	77.3% (73.1-81.4%)	75.9% (69.3-81.8%)
	DTP1	56.6% (47.0-67.1%)	73.1% (68.4-77.9%)	74.6% (70.8-78.4%)	91.1% (88.4-93.4%)	84.7% (78.0-90.6%)
	DTP3	47.3% (38.5-56.6%)	57.5% (53.7-61.4%)	63.9% (60.0-67.6%)	77.5% (73.9-80.8%)	60.2% (54.1-66.0%)
	HepB3	-	-	-	77.4% (73.8-80.7%)	60.1% (54.1-65.9%)
	Hib3	-	-	-	77.3% (73.7-80.5%)	60.1% (54.1-65.9%)
	MCV1	11.2% (3.0-28.1%)	61.2% (54.8-67.2%)	58.0% (51.7-64.4%)	71.2% (64.6-77.3%)	59.9% (50.6-68.6%)
	MCV2	-	-	-	-	-
	PCV3	-	-	-	-	60.1% (54.0-65.9%)
	Pol3	14.8% (9.0-22.4%)	57.7% (53.7-61.6%)	64.0% (60.3-67.6%)	75.7% (72.1-79.3%)	65.8% (59.6-71.8%)
	RCV1	-	-	-	-	-
	RotaC	-	-	-	-	59.5% (53.5-65.3%)
Maldives (MDV)	BCG	11.3% (6.9-17.2%)	85.9% (83.9-87.8%)	95.1% (93.4-96.3%)	97.4% (96.2-98.2%)	92.8% (89.5-95.3%)
	DTP1	8.9% (4.0-18.3%)	90.4% (87.0-93.6%)	95.2% (93.6-96.6%)	97.0% (95.9-98.0%)	88.3% (84.8-91.5%)
	DTP3	3.3% (1.8-5.6%)	83.3% (80.4-85.8%)	91.3% (89.3-93.0%)	94.7% (93.3-95.8%)	86.6% (83.0-89.8%)
	HepB3	-	-	85.1% (80.1-88.8%)	92.4% (89.3-94.5%)	86.1% (82.4-89.3%)
	Hib3	-	-	-	-	86.5% (82.9-89.7%)
	MCV1	31.5% (20.2-44.9%)	79.4% (75.6-82.7%)	92.6% (90.4-94.3%)	94.8% (92.4-96.5%)	91.5% (86.9-94.9%)
	MCV2	-	-	-	92.9% (90.5-94.9%)	91.2% (86.7-94.5%)
	PCV3	-	-	-	-	-
	Pol3	2.9% (1.7-4.8%)	84.7% (81.9-87.7%)	94.0% (92.5-95.4%)	93.6% (92.0-94.9%)	84.5% (81.0-87.7%)
	RCV1	-	-	-	94.4% (92.2-96.2%)	91.4% (86.8-94.8%)
	RotaC	-	-	-	-	-
Mexico (MEX)	BCG	83.5% (78.2-88.2%)	94.2% (92.0-95.9%)	98.5% (97.6-99.0%)	96.4% (95.5-97.2%)	76.3% (68.8-83.3%)
	DTP1	71.3% (62.8-80.1%)	73.0% (68.1-77.8%)	95.8% (94.4-96.9%)	95.7% (94.2-97.0%)	76.2% (70.2-81.9%)
	DTP3	55.8% (48.4-62.2%)	63.7% (59.0-68.1%)	88.6% (86.8-90.3%)	88.2% (86.2-90.0%)	70.6% (64.4-76.6%)
	HepB3	-	-	87.5% (84.8-89.6%)	85.1% (82.3-87.5%)	49.4% (40.3-57.5%)
	Hib3	-	-	88.5% (86.6-90.2%)	87.9% (86.0-89.8%)	62.4% (54.9-69.5%)
	MCV1	40.9% (29.8-52.9%)	74.5% (67.7-80.4%)	76.0% (70.9-80.3%)	84.7% (80.4-88.8%)	68.0% (56.1-79.1%)
	MCV2	-	-	75.6% (70.7-79.9%)	77.0% (70.7-82.3%)	67.5% (55.5-78.5%)
	PCV3	-	-	-	76.3% (73.1-79.1%)	70.6% (64.3-76.5%)
	Pol3	80.3% (76.7-83.6%)	60.5% (57.0-63.8%)	90.4% (88.9-91.8%)	88.2% (86.4-89.9%)	71.9% (66.2-77.6%)
	RCV1	-	-	75.9% (70.9-80.2%)	84.6% (80.3-88.6%)	67.9% (56.0-79.0%)
	RotaC	-	-	-	86.7% (84.8-88.5%)	70.1% (63.9-76.0%)
Marshall Islands (MHL)	BCG	-	57.0% (46.2-66.7%)	85.3% (80.3-89.5%)	93.1% (89.5-95.7%)	89.8% (81.2-94.8%)
	DTP1	-	96.7% (94.1-98.4%)	83.2% (77.7-87.7%)	97.9% (96.8-98.8%)	95.6% (92.4-97.7%)
	DTP3	-	81.1% (74.5-86.9%)	43.4% (37.1-50.3%)	90.2% (86.8-93.1%)	79.9% (72.7-86.3%)
	HepB3	-	22.4% (10.7-36.4%)	42.9% (36.5-49.7%)	90.0% (86.5-93.0%)	79.8% (72.6-86.2%)
	Hib3	-	-	41.6% (35.4-48.5%)	87.0% (82.6-90.4%)	70.9% (61.6-79.2%)
	MCV1	-	53.0% (42.5-63.4%)	83.9% (75.8-90.2%)	88.9% (81.1-94.3%)	83.9% (73.5-91.7%)
	MCV2	-	-	15.8% (8.8-26.4%)	79.4% (71.0-86.6%)	62.6% (50.5-72.7%)
	PCV3	-	-	-	32.2% (26.9-38.4%)	67.6% (60.8-74.2%)
	Pol3	-	66.8% (58.0-74.6%)	66.0% (58.7-73.2%)	92.1% (88.6-94.9%)	77.2% (68.0-84.8%)
	RCV1	-	-	83.8% (75.7-90.1%)	88.8% (81.0-94.2%)	83.8% (73.4-91.6%)
	RotaC	-	-	-	41.8% (38.7-44.9%)	62.6% (56.6-67.9%)

Country	Vaccine	1980	1990	2000	2010	2019
North Macedonia (MKD)	BCG	96.6% (89.9-99.5%)	96.7% (92.3-99.1%)	96.5% (94.5-98.0%)	88.1% (85.7-90.1%)	86.9% (81.9-91.0%)
	DTP1	94.3% (86.5-98.7%)	94.9% (91.1-97.5%)	97.1% (95.8-98.1%)	96.2% (94.7-97.5%)	95.3% (91.7-97.8%)
	DTP3	88.9% (76.1-96.0%)	91.0% (85.3-94.9%)	93.0% (91.0-94.8%)	90.6% (88.5-92.6%)	87.0% (81.5-91.2%)
	HepB3	-	-	-	84.0% (74.8-89.1%)	86.3% (80.9-90.6%)
	Hib3	-	-	-	84.6% (79.5-88.3%)	86.7% (81.2-91.0%)
	MCV1	88.7% (67.9-98.0%)	90.4% (81.3-95.9%)	95.5% (92.8-97.5%)	96.9% (94.5-98.4%)	78.2% (67.2-87.2%)
	MCV2	-	88.8% (80.0-94.5%)	94.6% (91.6-96.5%)	96.8% (94.3-98.2%)	78.1% (67.2-87.1%)
	PCV3	-	-	-	-	78.2% (71.9-83.1%)
	Pol3	90.4% (78.8-96.5%)	92.5% (88.1-95.8%)	93.0% (90.8-94.7%)	95.7% (94.0-97.1%)	94.2% (89.7-96.9%)
	RCV1	-	90.2% (81.1-95.7%)	95.4% (92.7-97.4%)	96.8% (94.4-98.3%)	78.1% (67.2-87.1%)
	RotaC	-	-	-	-	-
Mali (MLI)	BCG	15.0% (8.6-23.7%)	71.7% (66.8-76.3%)	71.2% (67.9-74.2%)	76.8% (74.3-79.3%)	78.1% (71.1-83.9%)
	DTP1	21.9% (11.4-38.9%)	49.6% (41.7-57.3%)	65.7% (60.9-70.9%)	77.0% (73.3-80.8%)	83.1% (77.1-88.1%)
	DTP3	7.2% (3.5-12.7%)	31.0% (26.4-35.7%)	49.0% (45.5-52.7%)	61.5% (58.9-64.0%)	71.4% (65.0-77.2%)
	HepB3	-	-	-	61.4% (58.7-63.9%)	71.3% (64.8-77.1%)
	Hib3	-	-	-	61.2% (58.5-63.7%)	71.3% (64.8-77.1%)
	MCV1	17.2% (6.6-33.7%)	45.5% (38.9-52.0%)	58.0% (52.6-63.2%)	73.5% (69.6-77.6%)	69.1% (59.8-78.1%)
	MCV2	-	-	-	-	4.8% (2.3-8.8%)
	PCV3	-	-	-	-	69.3% (62.9-75.0%)
	Pol3	7.2% (3.0-13.2%)	31.5% (25.2-38.1%)	44.0% (38.5-49.3%)	72.3% (67.1-76.4%)	44.4% (35.6-53.8%)
	RCV1	-	-	-	-	-
	RotaC	-	-	-	-	68.0% (61.9-73.6%)
Malta (MLT)	BCG	58.2% (42.2-73.8%)	77.6% (72.3-82.6%)	-	-	-
	DTP1	58.7% (41.7-74.1%)	80.1% (74.4-85.6%)	93.5% (90.4-95.9%)	97.2% (95.6-98.5%)	97.9% (95.5-99.2%)
	DTP3	43.4% (29.3-58.3%)	68.3% (61.9-74.6%)	86.9% (82.2-91.0%)	89.6% (85.9-92.9%)	97.2% (94.3-98.8%)
	HepB3	-	-	77.7% (66.4-85.4%)	86.8% (82.3-90.4%)	94.7% (90.7-97.3%)
	Hib3	-	-	82.5% (77.4-87.0%)	89.3% (85.5-92.6%)	97.0% (94.2-98.7%)
	MCV1	13.3% (4.1-30.0%)	79.0% (70.8-86.2%)	68.5% (58.2-77.9%)	83.3% (75.7-89.1%)	95.2% (88.8-98.4%)
	MCV2	-	4.7% (2.1-8.9%)	5.1% (2.6-8.9%)	83.0% (75.5-88.7%)	93.1% (87.0-96.7%)
	PCV3	-	-	-	-	-
	Pol3	75.7% (65.0-84.0%)	84.0% (79.7-87.5%)	86.7% (82.2-90.6%)	84.2% (78.7-88.6%)	96.8% (93.4-98.7%)
	RCV1	-	78.9% (70.7-86.1%)	68.5% (58.1-77.8%)	83.2% (75.6-89.0%)	95.1% (88.7-98.3%)
	RotaC	-	-	-	-	-
Myanmar (MMR)	BCG	18.7% (13.5-25.0%)	74.1% (70.7-77.5%)	89.7% (87.2-91.8%)	90.0% (87.4-92.3%)	90.2% (84.7-93.9%)
	DTP1	14.2% (7.7-25.2%)	78.2% (73.9-82.2%)	88.9% (86.6-91.0%)	89.7% (87.1-91.9%)	82.4% (76.2-87.6%)
	DTP3	5.4% (3.1-8.8%)	68.2% (64.1-72.1%)	82.5% (79.5-85.2%)	80.4% (77.0-83.9%)	71.1% (64.1-77.1%)
	HepB3	-	-	-	79.2% (75.7-82.7%)	70.9% (64.0-76.9%)
	Hib3	-	-	-	-	71.0% (64.0-77.1%)
	MCV1	-	63.9% (59.2-68.5%)	87.5% (83.9-90.7%)	88.5% (84.1-92.5%)	81.4% (72.9-87.6%)
	MCV2	-	-	-	73.7% (66.2-80.1%)	77.1% (68.7-83.7%)
	PCV3	-	-	-	-	70.8% (63.9-76.9%)
	Pol3	3.6% (1.6-6.7%)	68.1% (64.0-71.9%)	86.6% (84.2-88.8%)	82.5% (79.4-85.4%)	75.2% (69.5-80.3%)
	RCV1	-	-	-	-	81.3% (72.8-87.6%)
	RotaC	-	-	-	-	-
Montenegro (MNE)	BCG	99.7% (98.9-100.0%)	99.8% (99.3-100.0%)	86.6% (81.3-90.8%)	97.4% (95.9-98.6%)	89.9% (82.2-94.6%)
	DTP1	96.9% (89.7-99.6%)	97.8% (93.9-99.6%)	97.8% (96.0-99.0%)	97.0% (96.0-97.9%)	94.4% (91.0-96.6%)
	DTP3	87.8% (73.3-96.0%)	91.0% (83.1-95.9%)	90.4% (86.0-93.8%)	93.1% (91.1-95.0%)	85.5% (79.3-90.6%)
	HepB3	-	-	-	87.3% (81.6-91.0%)	73.4% (62.3-82.5%)
	Hib3	-	-	-	86.6% (80.4-90.7%)	85.4% (79.2-90.5%)
	MCV1	83.8% (59.7-96.7%)	87.0% (65.5-97.0%)	86.3% (76.8-93.2%)	93.6% (88.8-96.9%)	62.0% (47.3-75.1%)
	MCV2	-	-	86.1% (76.6-92.9%)	93.5% (88.6-96.8%)	61.9% (47.2-75.0%)
	PCV3	-	-	-	-	-
	Pol3	82.8% (60.9-95.1%)	86.8% (71.8-95.1%)	86.7% (79.1-92.2%)	89.4% (86.7-91.6%)	80.7% (74.2-86.0%)
	RCV1	-	-	86.1% (76.7-93.0%)	93.5% (88.7-96.8%)	61.9% (47.2-75.0%)

Country	Vaccine	1980	1990	2000	2010	2019
	RotaC	-	-	-	-	-
Mongolia (MNG)	BCG	52.1% (43.6-60.5%)	84.1% (79.9-87.8%)	96.1% (95.0-97.0%)	97.7% (96.7-98.4%)	93.5% (91.7-95.1%)
	DTP1	85.1% (77.9-92.4%)	75.4% (70.1-80.8%)	93.2% (91.1-95.1%)	97.0% (95.6-98.1%)	89.9% (84.6-94.7%)
	DTP3	79.1% (72.4-84.9%)	72.0% (66.9-76.4%)	88.7% (86.5-90.8%)	92.9% (90.9-94.4%)	80.5% (76.4-84.0%)
	HepB3	-	18.9% (10.8-29.7%)	87.2% (83.8-89.9%)	92.7% (90.7-94.3%)	80.4% (76.2-83.8%)
	Hib3	-	-	-	92.5% (90.6-94.1%)	80.4% (76.3-83.8%)
	MCV1	15.4% (8.3-25.3%)	82.4% (77.4-87.0%)	89.4% (86.3-92.2%)	90.3% (87.1-93.1%)	83.2% (77.9-87.7%)
	MCV2	-	76.3% (69.7-82.3%)	83.5% (78.5-87.4%)	89.4% (86.0-92.2%)	82.8% (77.6-87.2%)
	PCV3	-	-	-	-	32.7% (26.6-38.9%)
	Pol3	85.1% (78.3-90.1%)	73.6% (68.7-78.2%)	88.9% (87.0-90.7%)	94.5% (92.9-95.9%)	91.7% (89.0-94.1%)
	RCV1	-	-	-	90.2% (86.9-93.0%)	83.1% (77.8-87.6%)
	RotaC	-	-	-	-	-
Northern Mariana Islands (MNP)	BCG	-	-	-	-	-
	DTP1	82.3% (60.6-95.1%)	98.4% (94.4-99.8%)	98.9% (97.1-99.8%)	98.6% (96.3-99.7%)	98.8% (96.7-99.8%)
	DTP3	77.9% (53.1-92.9%)	96.6% (90.6-99.2%)	98.2% (95.1-99.6%)	98.2% (95.4-99.5%)	98.3% (95.4-99.6%)
	HepB3	-	93.4% (84.5-97.9%)	97.8% (94.5-99.4%)	98.0% (95.2-99.4%)	98.1% (95.2-99.5%)
	Hib3	-	-	-	-	-
	MCV1	81.2% (53.7-95.7%)	97.4% (92.8-99.5%)	99.0% (96.6-99.8%)	98.8% (96.1-99.8%)	98.9% (96.7-99.8%)
	MCV2	-	-	-	-	-
	PCV3	-	-	-	-	-
	Pol3	76.2% (51.3-92.4%)	95.6% (88.2-98.7%)	97.8% (93.9-99.4%)	97.9% (94.7-99.5%)	98.0% (94.6-99.5%)
	RCV1	-	-	-	-	-
	RotaC	-	-	-	-	-
Mozambique (MOZ)	BCG	51.4% (41.3-61.3%)	45.6% (40.8-50.4%)	83.1% (81.3-84.9%)	91.3% (89.7-92.8%)	89.4% (82.6-94.2%)
	DTP1	71.7% (61.6-81.4%)	55.7% (49.4-61.6%)	85.9% (82.6-89.0%)	85.7% (83.0-88.2%)	96.2% (93.7-98.0%)
	DTP3	54.9% (45.3-64.1%)	40.9% (36.0-45.7%)	66.0% (62.9-69.2%)	74.9% (71.9-77.9%)	95.3% (91.9-97.3%)
	HepB3	-	-	-	74.6% (71.6-77.6%)	95.1% (91.8-97.1%)
	Hib3	-	-	-	74.8% (71.8-77.7%)	95.2% (91.8-97.2%)
	MCV1	40.4% (26.8-55.1%)	40.4% (33.6-47.5%)	76.8% (72.4-80.8%)	83.9% (78.8-87.8%)	96.2% (91.0-98.7%)
	MCV2	-	-	-	-	75.6% (65.3-83.6%)
	PCV3	-	-	-	-	95.2% (91.8-97.2%)
	Pol3	39.6% (29.4-50.3%)	39.6% (34.0-44.8%)	64.2% (60.0-68.5%)	68.5% (64.8-72.1%)	82.5% (76.6-87.9%)
	RCV1	-	-	-	-	96.1% (90.9-98.6%)
	RotaC	-	-	-	-	95.1% (91.7-97.0%)
Mauritania (MRT)	BCG	53.6% (39.1-67.0%)	65.2% (59.7-70.4%)	78.0% (74.4-81.5%)	90.4% (87.3-92.8%)	93.0% (88.8-96.0%)
	DTP1	39.7% (24.2-56.4%)	52.7% (43.9-62.5%)	72.1% (66.1-78.1%)	80.4% (75.4-85.0%)	91.0% (84.7-95.4%)
	DTP3	20.5% (9.2-37.3%)	35.1% (24.5-47.1%)	46.9% (38.1-56.9%)	54.7% (45.6-63.1%)	68.0% (54.7-80.2%)
	HepB3	-	-	-	54.5% (45.4-62.9%)	67.9% (54.7-80.1%)
	Hib3	-	-	-	54.6% (45.5-63.0%)	67.9% (54.7-80.1%)
	MCV1	55.3% (40.0-71.8%)	43.4% (36.0-50.9%)	69.6% (63.3-75.2%)	77.4% (72.2-82.3%)	80.5% (71.8-87.8%)
	MCV2	-	-	-	-	-
	PCV3	-	-	-	-	67.1% (54.1-79.1%)
	Pol3	22.1% (10.2-37.5%)	37.5% (27.3-48.6%)	49.8% (41.4-58.8%)	55.2% (46.9-63.3%)	68.7% (55.7-80.2%)
	RCV1	-	-	-	-	80.5% (71.7-87.7%)
	RotaC	-	-	-	-	67.4% (54.2-79.5%)
Mauritius (MUS)	BCG	88.0% (82.2-92.5%)	88.5% (84.7-91.4%)	89.9% (86.0-93.0%)	99.0% (97.8-99.6%)	99.6% (98.9-99.9%)
	DTP1	89.0% (83.8-92.8%)	86.9% (83.2-89.9%)	90.7% (87.7-93.3%)	98.1% (97.1-98.8%)	96.6% (94.3-98.1%)
	DTP3	86.3% (80.2-90.7%)	85.1% (81.2-88.4%)	89.5% (86.1-92.4%)	97.2% (95.9-98.2%)	95.1% (92.2-97.3%)
	HepB3	-	-	89.3% (85.9-92.2%)	96.4% (94.2-97.7%)	93.3% (89.4-95.9%)
	Hib3	-	-	-	96.7% (95.3-97.7%)	95.0% (92.0-97.2%)
	MCV1	34.0% (20.0-51.3%)	81.2% (75.6-86.2%)	87.0% (80.9-91.6%)	99.5% (98.6-99.9%)	98.4% (96.3-99.4%)
	MCV2	-	-	-	87.3% (83.6-90.8%)	98.2% (96.2-99.3%)
	PCV3	-	-	-	-	94.0% (90.9-96.2%)
	Pol3	86.5% (81.4-90.8%)	86.9% (83.6-89.8%)	89.6% (86.4-92.3%)	97.2% (95.9-98.2%)	96.2% (93.6-97.9%)

Country	Vaccine	1980	1990	2000	2010	2019
	RCV1	-	-	86.9% (80.8-91.5%)	99.4% (98.5-99.8%)	98.2% (96.2-99.3%)
	RotaC	-	-	-	-	94.8% (91.8-96.9%)
Malawi (MWI)	BCG	98.9% (97.5-99.6%)	89.6% (87.7-91.2%)	92.5% (90.9-93.9%)	96.7% (95.4-97.6%)	89.9% (84.4-93.9%)
	DTP1	76.6% (68.6-83.9%)	91.8% (89.6-93.8%)	95.4% (94.1-96.6%)	96.3% (95.1-97.4%)	96.5% (94.0-98.1%)
	DTP3	56.0% (48.7-64.0%)	77.6% (74.8-80.3%)	83.9% (81.6-86.0%)	90.3% (87.9-92.2%)	94.0% (90.0-96.6%)
	HepB3	-	-	-	90.1% (87.7-92.1%)	93.8% (89.9-96.5%)
	Hib3	-	-	-	90.1% (87.8-92.0%)	93.9% (90.0-96.4%)
	MCV1	59.8% (49.4-69.2%)	80.4% (76.4-84.1%)	87.2% (83.6-90.1%)	94.3% (91.4-96.4%)	96.3% (92.4-98.6%)
	MCV2	-	-	-	-	79.1% (68.7-87.2%)
	PCV3	-	-	-	-	93.8% (89.9-96.4%)
	Pol3	37.0% (29.3-45.7%)	79.4% (75.5-82.8%)	76.5% (73.0-79.7%)	82.1% (78.9-85.2%)	84.0% (77.6-88.9%)
	RCV1	-	-	-	-	96.2% (92.3-98.5%)
	RotaC	-	-	-	-	92.0% (88.2-94.5%)
Malaysia (MYS)	BCG	85.8% (81.1-89.8%)	92.7% (91.4-93.7%)	92.5% (91.2-93.9%)	93.1% (91.9-94.2%)	92.7% (90.0-94.9%)
	DTP1	78.6% (71.8-84.9%)	91.7% (88.7-94.2%)	98.1% (97.0-98.9%)	97.6% (96.2-98.5%)	98.8% (97.4-99.5%)
	DTP3	65.7% (57.9-72.8%)	86.5% (82.5-89.8%)	96.2% (94.3-97.6%)	95.7% (93.6-97.4%)	97.8% (95.9-99.0%)
	HepB3	-	79.3% (72.8-84.4%)	91.7% (87.7-94.5%)	95.3% (93.0-96.9%)	97.1% (94.6-98.5%)
	Hib3	-	-	-	94.6% (92.1-96.4%)	97.7% (95.8-98.8%)
	MCV1	13.1% (5.0-26.3%)	74.5% (67.3-80.8%)	85.8% (79.2-91.3%)	96.6% (92.0-99.0%)	99.5% (98.5-99.9%)
	MCV2	-	-	-	96.4% (91.9-98.9%)	94.1% (90.6-96.5%)
	PCV3	-	-	-	-	-
	Pol3	64.6% (57.1-71.6%)	85.1% (81.7-88.3%)	94.2% (92.1-95.9%)	96.0% (94.1-97.4%)	98.2% (96.7-99.2%)
	RCV1	-	-	85.7% (79.1-91.1%)	96.5% (91.9-98.9%)	99.4% (98.4-99.8%)
	RotaC	-	-	-	-	-
Namibia (NAM)	BCG	-	86.4% (83.2-89.4%)	82.8% (79.0-86.2%)	93.6% (90.9-95.7%)	99.8% (99.4-99.9%)
	DTP1	-	85.1% (79.9-89.5%)	87.6% (84.0-90.9%)	90.9% (87.8-93.4%)	94.1% (89.3-97.1%)
	DTP3	-	64.3% (57.9-70.0%)	74.2% (68.8-79.0%)	81.2% (76.2-85.2%)	89.6% (82.3-94.5%)
	HepB3	-	-	-	81.1% (76.1-85.1%)	89.4% (82.2-94.3%)
	Hib3	-	-	-	81.1% (76.1-85.1%)	89.5% (82.2-94.4%)
	MCV1	-	78.6% (74.0-82.7%)	83.1% (78.4-87.0%)	90.0% (86.2-93.0%)	97.7% (93.6-99.5%)
	MCV2	-	-	-	-	50.8% (39.8-61.4%)
	PCV3	-	-	-	-	67.8% (59.4-74.6%)
	Pol3	-	64.7% (57.8-72.3%)	67.4% (61.4-72.7%)	69.8% (64.0-75.2%)	74.3% (63.2-83.3%)
	RCV1	-	-	-	-	96.7% (92.4-98.4%)
	RotaC	-	-	-	-	89.4% (82.1-94.3%)
Niger (NER)	BCG	32.2% (21.6-45.1%)	51.1% (47.7-54.6%)	49.1% (44.6-53.7%)	88.0% (85.4-90.4%)	64.6% (51.4-75.5%)
	DTP1	24.5% (14.2-38.2%)	46.6% (42.9-50.6%)	46.6% (42.4-50.8%)	86.1% (83.9-88.2%)	83.4% (77.9-88.3%)
	DTP3	8.6% (4.2-15.6%)	28.2% (25.6-31.2%)	29.6% (26.5-32.6%)	67.6% (64.9-70.3%)	59.1% (51.5-66.5%)
	HepB3	-	-	-	67.5% (64.8-70.2%)	59.0% (51.5-66.4%)
	Hib3	-	-	-	67.5% (64.8-70.2%)	59.0% (51.4-66.4%)
	MCV1	22.6% (10.0-39.9%)	42.0% (36.8-47.2%)	41.0% (34.1-48.1%)	72.5% (67.2-77.4%)	62.9% (51.0-74.2%)
	MCV2	-	-	-	-	43.4% (33.8-52.7%)
	PCV3	-	-	-	-	59.0% (51.4-66.4%)
	Pol3	9.0% (4.2-16.4%)	27.7% (24.3-31.3%)	41.9% (37.7-46.3%)	79.1% (75.7-82.2%)	83.6% (76.4-89.2%)
	RCV1	-	-	-	-	-
	RotaC	-	-	-	-	59.0% (51.5-66.4%)
Nigeria (NGA)	BCG	32.1% (19.7-45.2%)	63.9% (56.4-70.6%)	48.5% (42.1-55.2%)	62.0% (56.1-67.9%)	75.3% (63.0-84.5%)
	DTP1	36.4% (25.3-50.6%)	57.1% (51.4-62.9%)	45.0% (40.3-50.3%)	60.3% (56.3-64.5%)	68.9% (60.9-76.3%)
	DTP3	23.6% (15.2-34.4%)	40.2% (34.6-45.9%)	27.1% (22.8-31.6%)	45.0% (41.1-49.2%)	57.2% (47.9-65.8%)
	HepB3	-	-	-	44.4% (40.5-48.6%)	57.1% (47.8-65.7%)
	Hib3	-	-	-	-	57.1% (47.8-65.6%)
	MCV1	25.4% (10.2-46.0%)	52.5% (42.0-62.8%)	40.4% (32.7-48.8%)	53.5% (46.7-60.1%)	59.6% (45.9-71.8%)
	MCV2	-	-	-	-	12.5% (6.3-21.0%)
	PCV3	-	-	-	-	57.0% (47.8-65.6%)

Country	Vaccine	1980	1990	2000	2010	2019
	Pol3	24.5% (15.2-37.3%)	38.4% (32.1-45.1%)	37.3% (32.1-42.7%)	54.0% (48.7-59.2%)	58.1% (47.7-68.3%)
	RCV1	-	-	-	-	-
	RotaC	-	-	-	-	-
Nicaragua (NIC)	BCG	51.3% (40.8-61.8%)	95.8% (94.0-97.2%)	96.1% (94.3-97.3%)	99.8% (99.4-99.9%)	99.8% (99.5-100.0%)
	DTP1	36.5% (25.6-52.9%)	89.3% (85.5-92.7%)	93.9% (91.7-95.8%)	99.8% (99.5-99.9%)	99.9% (99.6-100.0%)
	DTP3	26.5% (19.3-34.0%)	78.6% (74.9-81.7%)	85.9% (82.5-88.7%)	99.7% (99.4-99.9%)	99.8% (99.6-100.0%)
	HepB3	-	-	85.8% (82.4-88.5%)	99.6% (99.1-99.8%)	99.7% (99.3-99.9%)
	Hib3	-	-	85.8% (82.4-88.6%)	99.6% (99.2-99.8%)	99.7% (99.5-99.9%)
	MCV1	27.6% (20.1-36.8%)	90.2% (86.9-92.8%)	93.3% (90.0-95.5%)	99.7% (99.2-99.9%)	99.8% (99.5-100.0%)
	MCV2	-	-	-	-	98.3% (96.9-99.1%)
	PCV3	-	-	-	-	99.7% (99.5-99.9%)
	Pol3	37.0% (28.4-47.0%)	86.7% (83.6-89.3%)	87.5% (84.4-90.1%)	99.7% (99.4-99.9%)	99.8% (99.5-100.0%)
	RCV1	19.8% (14.3-26.7%)	67.9% (62.5-72.8%)	89.2% (86.0-91.6%)	99.6% (99.0-99.8%)	99.7% (99.4-99.9%)
	RotaC	-	-	-	98.3% (98.0-98.6%)	99.8% (99.5-99.9%)
Niue (NIU)	BCG	80.3% (71.2-88.3%)	95.9% (92.2-98.1%)	99.0% (97.6-99.7%)	99.0% (97.6-99.7%)	99.1% (97.6-99.7%)
	DTP1	11.5% (7.0-19.7%)	99.8% (99.5-99.9%)	98.0% (96.7-98.8%)	97.4% (95.9-98.5%)	98.0% (96.2-99.0%)
	DTP3	7.1% (4.4-11.0%)	98.0% (96.5-98.9%)	97.2% (95.5-98.4%)	97.2% (95.7-98.3%)	97.9% (96.1-99.0%)
	HepB3	-	97.3% (95.5-98.5%)	96.7% (94.7-98.0%)	97.0% (95.5-98.2%)	97.8% (95.9-98.9%)
	Hib3	-	-	97.0% (95.2-98.1%)	97.0% (95.4-98.1%)	97.8% (96.0-98.9%)
	MCV1	73.2% (59.9-83.8%)	90.3% (81.3-95.8%)	97.9% (94.7-99.4%)	99.8% (99.3-100.0%)	99.9% (99.5-100.0%)
	MCV2	72.7% (59.6-83.2%)	90.0% (81.0-95.5%)	97.1% (93.8-98.7%)	99.0% (98.2-99.5%)	99.7% (99.3-99.9%)
	PCV3	-	-	-	97.1% (95.5-98.2%)	97.8% (96.0-98.9%)
	Pol3	69.6% (60.6-77.5%)	96.6% (94.6-98.0%)	96.7% (95.0-97.9%)	97.0% (95.5-98.1%)	97.7% (96.0-98.8%)
	RCV1	73.0% (59.8-83.5%)	90.1% (81.0-95.6%)	97.7% (94.6-99.3%)	99.7% (99.1-99.9%)	99.7% (99.4-99.9%)
	RotaC	-	-	-	-	97.4% (95.6-98.5%)
Netherlands (NLD)	BCG	-	-	-	-	-
	DTP1	99.9% (99.7-100.0%)	99.9% (99.8-100.0%)	99.3% (98.8-99.6%)	99.1% (97.9-99.7%)	98.3% (95.9-99.7%)
	DTP3	96.3% (93.8-98.0%)	97.2% (95.7-98.2%)	97.2% (95.7-98.2%)	97.0% (95.5-98.1%)	94.0% (90.6-96.5%)
	HepB3	-	-	-	17.9% (11.8-24.9%)	91.0% (85.0-94.8%)
	Hib3	-	-	96.1% (94.0-97.4%)	96.9% (95.4-98.0%)	93.9% (90.5-96.4%)
	MCV1	92.1% (86.6-96.0%)	95.3% (92.5-97.4%)	97.0% (94.3-98.5%)	97.3% (94.8-98.8%)	94.7% (89.6-97.6%)
	MCV2	-	94.1% (91.0-96.3%)	96.8% (94.2-98.4%)	93.7% (90.2-96.1%)	90.8% (85.4-94.5%)
	PCV3	-	-	-	95.8% (94.2-97.0%)	93.2% (89.8-95.8%)
	Pol3	96.7% (94.2-98.3%)	97.5% (96.0-98.6%)	97.6% (96.0-98.6%)	97.4% (96.0-98.4%)	94.4% (91.4-96.8%)
	RCV1	91.8% (86.3-95.7%)	95.1% (92.3-97.1%)	96.8% (94.2-98.4%)	97.2% (94.7-98.7%)	94.6% (89.4-97.5%)
	RotaC	-	-	-	-	-
Norway (NOR)	BCG	-	-	-	-	-
	DTP1	95.0% (90.6-97.8%)	95.0% (93.1-96.6%)	98.8% (97.9-99.4%)	99.1% (98.5-99.5%)	99.0% (98.0-99.5%)
	DTP3	86.0% (77.5-92.3%)	88.9% (85.4-91.7%)	91.8% (88.7-94.3%)	94.0% (91.8-95.8%)	96.5% (94.1-98.2%)
	HepB3	-	-	-	90.2% (81.7-94.3%)	95.1% (91.4-97.4%)
	Hib3	-	-	91.7% (88.4-94.1%)	93.9% (91.6-95.7%)	96.4% (93.9-98.1%)
	MCV1	77.9% (60.0-90.4%)	90.4% (86.3-93.8%)	90.0% (84.8-93.5%)	94.3% (91.1-96.7%)	97.7% (95.1-99.2%)
	MCV2	-	87.1% (82.2-90.9%)	89.6% (84.7-93.1%)	94.1% (90.9-96.5%)	94.3% (91.4-96.4%)
	PCV3	-	-	-	91.3% (89.0-93.4%)	95.1% (92.5-96.8%)
	Pol3	87.3% (78.9-92.9%)	88.1% (84.5-90.9%)	91.8% (88.9-94.2%)	94.2% (91.6-96.0%)	96.7% (94.4-98.3%)
	RCV1	75.2% (58.8-85.0%)	86.4% (81.6-90.1%)	89.2% (84.3-92.6%)	94.2% (91.0-96.5%)	97.1% (95.0-98.4%)
	RotaC	-	-	-	-	94.5% (92.1-96.2%)
Nepal (NPL)	BCG	24.9% (18.2-32.3%)	74.0% (71.9-76.0%)	87.4% (85.6-89.0%)	95.9% (94.9-96.8%)	94.3% (91.2-96.5%)
	DTP1	22.7% (14.7-34.9%)	69.8% (65.1-74.7%)	86.5% (84.2-88.6%)	92.0% (90.2-93.5%)	93.1% (89.9-95.8%)
	DTP3	8.8% (5.5-13.3%)	43.8% (40.6-46.8%)	75.6% (73.1-77.9%)	86.2% (83.8-88.5%)	85.8% (80.8-90.0%)
	HepB3	-	-	-	79.3% (73.6-83.6%)	85.5% (80.7-89.8%)
	Hib3	-	-	-	80.2% (75.7-83.9%)	85.7% (80.7-89.9%)
	MCV1	5.2% (1.9-10.8%)	56.4% (52.0-60.7%)	77.9% (74.4-81.2%)	90.8% (87.5-93.5%)	96.5% (92.2-98.7%)
	MCV2	-	-	-	-	71.2% (60.0-80.0%)

Country	Vaccine	1980	1990	2000	2010	2019
	PCV3	-	-	-	-	79.1% (74.1-83.2%)
	Pol3	4.1% (2.0-8.0%)	44.2% (39.5-49.5%)	88.1% (84.5-91.2%)	92.6% (89.5-95.2%)	90.9% (84.9-94.9%)
	RCV1	-	-	-	-	96.4% (92.0-98.6%)
	RotaC	-	-	-	-	-
Nauru (NRU)	BCG	87.6% (74.1-95.3%)	99.2% (98.6-99.6%)	99.0% (98.1-99.5%)	99.7% (99.3-99.9%)	99.8% (99.4-100.0%)
	DTP1	94.9% (86.5-98.6%)	98.8% (97.8-99.5%)	93.8% (90.8-96.3%)	98.7% (97.9-99.2%)	99.9% (99.7-100.0%)
	DTP3	71.4% (58.2-83.1%)	76.6% (70.0-82.6%)	73.5% (66.6-79.5%)	98.5% (97.7-99.1%)	99.3% (98.5-99.7%)
	HepB3	-	74.9% (67.7-81.1%)	68.5% (60.6-75.4%)	98.1% (96.9-98.9%)	99.1% (98.2-99.6%)
	Hib3	-	-	-	98.3% (97.4-99.0%)	99.2% (98.4-99.6%)
	MCV1	99.4% (98.1-99.9%)	99.3% (97.5-99.9%)	54.1% (33.7-73.1%)	98.9% (97.3-99.7%)	99.7% (98.9-99.9%)
	MCV2	-	-	53.5% (33.4-71.9%)	97.5% (95.8-98.6%)	98.9% (97.9-99.4%)
	PCV3	-	-	-	-	-
	Pol3	64.8% (48.8-78.6%)	75.4% (67.9-81.4%)	62.8% (54.6-70.0%)	98.6% (97.7-99.3%)	99.5% (98.8-99.8%)
	RCV1	-	-	-	98.8% (97.2-99.6%)	99.6% (98.8-99.8%)
	RotaC	-	-	-	-	-
New Zealand (NZL)	BCG	-	-	-	-	-
	DTP1	77.7% (71.3-83.2%)	85.6% (82.0-88.7%)	91.1% (88.0-93.7%)	94.0% (91.6-95.9%)	94.8% (91.5-97.2%)
	DTP3	73.3% (66.1-79.8%)	81.2% (76.6-85.1%)	88.2% (84.3-91.4%)	92.4% (89.6-94.7%)	92.7% (88.6-95.9%)
	HepB3	-	66.0% (48.1-77.9%)	88.1% (84.1-91.3%)	91.7% (88.5-94.0%)	92.6% (88.5-95.8%)
	Hib3	-	-	88.1% (84.1-91.3%)	90.9% (87.7-93.5%)	91.8% (87.7-95.0%)
	MCV1	79.6% (69.3-87.6%)	83.8% (76.8-89.4%)	83.5% (76.8-88.9%)	91.1% (86.1-94.6%)	93.2% (86.9-97.2%)
	MCV2	-	-	72.0% (60.3-80.7%)	83.4% (77.0-88.3%)	90.3% (83.9-94.4%)
	PCV3	-	-	-	89.3% (86.4-91.7%)	92.4% (88.3-95.6%)
	Pol3	75.2% (63.2-84.8%)	71.0% (64.4-77.0%)	83.3% (78.3-87.5%)	92.5% (89.4-94.9%)	93.1% (88.8-96.1%)
	RCV1	78.9% (68.6-86.8%)	83.3% (76.3-88.5%)	83.2% (76.6-88.6%)	90.4% (85.9-94.0%)	92.5% (86.7-96.3%)
	RotaC	-	-	-	-	87.7% (83.7-90.7%)
Oman (OMN)	BCG	52.3% (43.2-60.8%)	95.9% (93.4-97.7%)	98.9% (97.6-99.6%)	99.8% (99.4-100.0%)	99.5% (98.4-99.9%)
	DTP1	23.0% (16.8-30.2%)	98.5% (97.7-99.1%)	99.7% (99.4-99.9%)	98.6% (97.6-99.2%)	99.0% (98.0-99.6%)
	DTP3	17.9% (13.2-23.5%)	95.4% (93.6-96.7%)	98.0% (97.0-98.8%)	97.9% (96.5-98.8%)	98.9% (97.8-99.5%)
	HepB3	-	94.5% (92.0-96.2%)	97.9% (96.8-98.6%)	97.7% (96.3-98.6%)	98.8% (97.7-99.5%)
	Hib3	-	-	-	97.7% (96.4-98.6%)	98.8% (97.7-99.4%)
	MCV1	18.2% (12.1-26.1%)	96.4% (93.8-98.0%)	99.7% (99.1-99.9%)	99.7% (99.2-99.9%)	99.9% (99.6-100.0%)
	MCV2	-	-	99.5% (98.9-99.8%)	99.6% (99.0-99.8%)	99.7% (99.4-99.9%)
	PCV3	-	-	-	97.4% (96.1-98.3%)	98.8% (97.7-99.4%)
	Pol3	17.7% (13.2-23.0%)	94.9% (93.1-96.3%)	98.0% (97.0-98.8%)	97.9% (96.7-98.8%)	99.2% (98.3-99.7%)
	RCV1	-	-	99.5% (98.8-99.7%)	99.6% (99.0-99.8%)	99.8% (99.5-99.9%)
	RotaC	-	-	-	-	-
Pakistan (PAK)	BCG	10.8% (6.3-16.6%)	73.6% (70.0-76.8%)	71.3% (68.6-73.7%)	82.3% (79.2-85.0%)	91.7% (86.5-95.1%)
	DTP1	17.3% (10.9-25.3%)	76.1% (72.3-79.7%)	75.8% (72.7-78.8%)	80.8% (77.7-84.0%)	91.1% (86.9-94.3%)
	DTP3	4.9% (2.5-8.4%)	54.2% (49.1-59.2%)	68.5% (64.8-72.0%)	71.1% (66.9-75.3%)	83.8% (76.9-89.1%)
	HepB3	-	-	-	70.9% (66.6-75.0%)	83.7% (76.8-89.0%)
	Hib3	-	-	-	71.0% (66.9-75.2%)	83.7% (76.8-89.1%)
	MCV1	6.2% (2.4-12.5%)	61.1% (53.8-68.2%)	66.7% (61.3-71.5%)	68.7% (63.1-73.5%)	76.9% (66.3-85.9%)
	MCV2	-	-	-	34.0% (25.5-42.6%)	61.6% (50.9-70.8%)
	PCV3	-	-	-	-	83.7% (76.8-89.0%)
	Pol3	5.3% (2.8-8.8%)	53.9% (48.8-59.1%)	78.0% (74.5-81.0%)	82.7% (79.1-86.0%)	82.7% (76.3-88.1%)
	RCV1	-	-	-	-	-
	RotaC	-	-	-	-	80.4% (73.8-85.5%)
Panama (PAN)	BCG	95.6% (92.3-97.7%)	93.4% (91.1-95.5%)	96.5% (95.3-97.4%)	94.9% (93.5-96.1%)	96.4% (94.3-98.0%)
	DTP1	75.6% (67.8-83.2%)	89.9% (86.9-92.7%)	94.3% (91.3-96.7%)	94.3% (91.3-96.9%)	97.9% (95.5-99.3%)
	DTP3	72.0% (64.6-79.1%)	87.2% (84.1-90.2%)	83.5% (81.3-85.8%)	77.0% (73.6-79.9%)	82.9% (77.3-87.8%)
	HepB3	-	-	-	76.8% (73.5-79.8%)	82.8% (77.2-87.6%)
	Hib3	-	-	83.4% (81.1-85.7%)	76.9% (73.5-79.8%)	82.8% (77.1-87.7%)
	MCV1	70.9% (61.3-79.0%)	77.7% (71.7-83.0%)	94.1% (90.9-96.3%)	90.1% (86.7-93.0%)	93.1% (88.9-95.9%)

Country	Vaccine	1980	1990	2000	2010	2019
	MCV2	-	-	81.9% (73.0-88.0%)	70.2% (63.4-76.4%)	92.7% (88.7-95.7%)
	PCV3	-	-	-	59.3% (54.1-64.6%)	82.8% (77.2-87.7%)
	Pol3	71.7% (63.6-79.2%)	86.9% (83.4-89.9%)	83.4% (81.1-85.7%)	83.6% (80.6-86.2%)	81.1% (75.2-86.4%)
	RCV1	-	77.6% (71.5-82.8%)	93.9% (90.8-96.2%)	90.0% (86.6-92.9%)	93.0% (88.8-95.9%)
	RotaC	-	-	-	75.3% (72.0-78.2%)	82.8% (77.2-87.7%)
Peru (PER)	BCG	82.4% (74.2-88.7%)	92.1% (90.8-93.2%)	96.6% (95.7-97.3%)	93.5% (92.9-94.1%)	97.4% (96.1-98.3%)
	DTP1	38.1% (29.2-49.4%)	87.6% (84.8-90.1%)	97.0% (95.9-98.0%)	93.1% (92.2-93.8%)	94.1% (91.3-96.3%)
	DTP3	27.5% (21.0-33.8%)	73.1% (70.3-75.9%)	81.5% (79.2-83.5%)	80.0% (78.4-81.6%)	85.0% (80.5-88.9%)
	HepB3	-	-	-	77.1% (74.1-79.6%)	84.9% (80.3-88.7%)
	Hib3	-	-	28.4% (22.8-34.5%)	78.2% (76.2-80.0%)	84.9% (80.4-88.8%)
	MCV1	36.2% (28.2-45.3%)	87.2% (83.2-90.4%)	89.7% (86.4-92.9%)	90.3% (88.6-92.1%)	85.5% (77.5-91.5%)
	MCV2	-	-	-	50.5% (43.9-56.8%)	66.8% (58.2-74.6%)
	PCV3	-	-	-	52.5% (48.1-56.6%)	81.7% (77.3-85.5%)
	Pol3	26.9% (20.8-33.7%)	72.1% (68.9-75.3%)	72.4% (69.8-75.1%)	82.7% (81.1-84.3%)	86.0% (81.0-90.1%)
	RCV1	-	-	-	90.2% (88.5-91.9%)	85.4% (77.4-91.3%)
	RotaC	-	-	-	49.8% (48.1-51.5%)	84.9% (80.5-88.8%)
Philippines (PHL)	BCG	60.4% (52.2-68.8%)	86.3% (83.5-88.7%)	90.4% (87.8-92.6%)	87.2% (82.8-91.0%)	82.6% (75.0-88.2%)
	DTP1	46.4% (36.6-57.7%)	83.4% (78.9-87.2%)	88.0% (84.7-90.7%)	90.2% (87.4-92.7%)	86.2% (80.2-91.1%)
	DTP3	40.8% (31.9-51.4%)	73.5% (68.8-78.1%)	79.1% (74.5-83.1%)	82.4% (78.3-86.2%)	82.9% (76.1-88.3%)
	HepB3	-	-	39.5% (30.9-48.2%)	81.3% (76.9-85.1%)	82.8% (75.9-88.2%)
	Hib3	-	-	-	9.2% (4.7-16.7%)	82.3% (75.2-87.8%)
	MCV1	17.1% (7.3-32.7%)	79.7% (74.8-84.0%)	80.3% (75.7-84.3%)	86.8% (82.3-90.5%)	76.6% (66.9-84.5%)
	MCV2	-	-	-	28.9% (21.2-37.7%)	67.2% (57.8-75.0%)
	PCV3	-	-	-	-	57.3% (50.4-63.8%)
	Pol3	39.3% (31.3-47.8%)	72.2% (68.3-75.8%)	78.8% (75.6-81.6%)	81.7% (78.6-84.7%)	84.1% (78.7-88.6%)
	RCV1	-	-	-	48.5% (45.0-51.9%)	76.3% (66.7-83.7%)
	RotaC	-	-	-	-	-
Palau (PLW)	BCG	-	-	-	-	-
	DTP1	95.3% (87.1-99.0%)	94.7% (90.8-97.8%)	93.9% (90.2-96.6%)	97.5% (94.5-99.1%)	98.4% (96.5-99.5%)
	DTP3	86.1% (72.6-94.1%)	87.6% (82.6-91.5%)	90.9% (86.4-94.4%)	72.4% (66.0-78.6%)	94.0% (89.5-96.9%)
	HepB3	-	73.3% (61.3-82.5%)	90.6% (85.8-94.1%)	71.7% (65.1-78.1%)	92.2% (87.4-95.7%)
	Hib3	-	-	89.7% (85.1-93.2%)	70.7% (64.1-76.7%)	85.1% (77.5-90.6%)
	MCV1	96.6% (90.2-99.4%)	85.4% (71.7-93.8%)	85.4% (75.3-92.1%)	63.4% (52.1-74.0%)	93.5% (86.7-97.7%)
	MCV2	-	-	78.3% (68.0-85.8%)	57.2% (46.1-67.2%)	81.8% (73.4-87.8%)
	PCV3	-	-	-	42.4% (35.9-49.2%)	79.2% (73.7-84.2%)
	Pol3	89.4% (75.9-96.3%)	86.2% (76.8-92.5%)	85.5% (78.7-91.0%)	73.0% (65.3-79.9%)	94.0% (89.4-97.0%)
	RCV1	-	85.2% (71.5-93.6%)	85.3% (75.2-92.0%)	63.4% (52.0-73.9%)	93.4% (86.5-97.6%)
	RotaC	-	-	-	71.9% (65.6-78.1%)	91.7% (87.4-94.6%)
Papua New Guinea (PNG)	BCG	59.0% (50.4-67.0%)	85.5% (81.8-88.7%)	89.1% (85.6-92.1%)	89.8% (86.2-92.6%)	50.5% (42.1-58.6%)
	DTP1	56.3% (46.4-66.2%)	83.7% (78.9-87.9%)	82.6% (79.0-86.3%)	81.0% (77.1-84.6%)	55.6% (47.2-64.4%)
	DTP3	24.3% (18.0-31.9%)	46.9% (42.1-51.9%)	60.0% (55.3-64.6%)	60.2% (55.5-64.8%)	35.2% (28.2-42.8%)
	HepB3	-	12.3% (7.7-18.1%)	51.9% (44.3-58.2%)	60.0% (55.2-64.6%)	35.2% (28.2-42.8%)
	Hib3	-	-	-	60.1% (55.4-64.7%)	35.2% (28.2-42.8%)
	MCV1	29.3% (10.8-55.2%)	93.6% (88.5-97.1%)	88.0% (79.0-93.9%)	70.2% (58.7-79.5%)	45.0% (29.8-61.5%)
	MCV2	-	-	-	-	27.3% (16.1-40.0%)
	PCV3	-	-	-	-	32.8% (26.2-39.8%)
	Pol3	21.4% (14.7-29.9%)	43.2% (37.3-49.4%)	50.1% (44.2-56.3%)	68.6% (62.6-74.0%)	38.0% (29.7-46.9%)
	RCV1	-	-	-	-	44.9% (29.7-61.5%)
	RotaC	-	-	-	-	-
Poland (POL)	BCG	94.4% (90.2-97.1%)	96.5% (94.4-98.0%)	96.2% (94.0-97.8%)	93.8% (91.2-95.9%)	92.9% (87.5-96.2%)
	DTP1	99.8% (99.4-99.9%)	99.3% (98.9-99.6%)	99.6% (99.3-99.8%)	99.8% (99.4-100.0%)	99.3% (97.7-99.9%)
	DTP3	94.7% (91.6-96.8%)	94.4% (92.4-95.9%)	97.7% (96.5-98.6%)	98.9% (98.1-99.4%)	96.6% (93.6-98.4%)
	HepB3	-	-	96.1% (93.7-97.6%)	97.3% (94.8-98.6%)	93.1% (87.3-96.6%)
	Hib3	-	-	-	98.5% (97.6-99.1%)	96.5% (93.4-98.3%)

Country	Vaccine	1980	1990	2000	2010	2019
	MCV1	94.0% (89.1-96.9%)	96.0% (93.2-98.0%)	98.7% (96.9-99.6%)	99.2% (98.1-99.8%)	95.4% (90.5-98.3%)
	MCV2	-	-	97.1% (95.0-98.2%)	94.1% (90.7-96.5%)	94.0% (88.9-96.8%)
	PCV3	-	-	-	-	69.0% (62.3-75.3%)
	Pol3	94.1% (91.1-96.4%)	94.1% (92.1-95.7%)	97.7% (96.5-98.6%)	96.4% (94.7-97.6%)	90.0% (84.5-94.0%)
	RCV1	-	91.5% (88.5-93.5%)	95.6% (93.9-96.8%)	99.0% (97.9-99.4%)	95.3% (90.4-97.9%)
	RotaC	-	-	-	-	-
Puerto Rico (PRI)	BCG	-	-	-	-	-
	DTP1	80.9% (55.0-95.7%)	85.3% (63.8-97.0%)	90.2% (76.0-97.6%)	94.1% (89.5-97.0%)	95.4% (89.9-98.3%)
	DTP3	61.9% (33.1-85.7%)	72.6% (44.6-91.6%)	83.1% (65.2-93.8%)	90.5% (84.5-94.5%)	88.7% (79.9-94.5%)
	HepB3	-	45.4% (17.2-71.4%)	73.9% (50.8-89.3%)	85.5% (76.0-91.5%)	87.2% (78.2-93.2%)
	Hib3	-	-	81.2% (63.7-92.0%)	89.7% (83.8-93.9%)	88.1% (79.3-93.9%)
	MCV1	57.8% (24.4-86.0%)	71.5% (39.5-93.0%)	82.3% (55.6-96.0%)	90.8% (81.1-96.5%)	91.7% (80.2-97.9%)
	MCV2	-	65.7% (36.0-85.9%)	80.0% (53.7-93.7%)	89.2% (79.6-94.9%)	90.4% (78.9-96.6%)
	PCV3	-	-	11.9% (7.7-16.7%)	77.3% (70.9-82.8%)	88.0% (79.2-93.8%)
	Pol3	59.3% (30.6-83.5%)	69.4% (42.3-89.0%)	80.2% (61.0-92.5%)	88.2% (81.0-93.1%)	87.4% (78.1-93.6%)
	RCV1	-	69.3% (38.6-89.8%)	80.6% (54.1-94.1%)	89.6% (79.7-95.2%)	90.7% (79.2-96.8%)
	RotaC	-	-	-	10.6% (9.4-11.9%)	61.3% (54.5-66.0%)
North Korea (PRK)	BCG	36.6% (29.1-44.1%)	75.3% (73.5-76.9%)	89.0% (86.1-91.7%)	99.1% (98.0-99.7%)	98.4% (95.7-99.7%)
	DTP1	47.0% (38.6-56.4%)	95.6% (92.7-97.5%)	76.9% (73.0-80.7%)	95.3% (92.9-97.0%)	99.9% (99.7-99.9%)
	DTP3	27.6% (20.8-35.5%)	48.6% (45.7-51.7%)	65.4% (61.6-68.7%)	94.2% (91.3-96.3%)	99.8% (99.5-99.9%)
	HepB3	-	-	-	93.7% (90.9-95.9%)	99.6% (99.1-99.8%)
	Hib3	-	-	-	-	99.5% (99.2-99.8%)
	MCV1	24.0% (16.7-32.4%)	86.9% (84.0-89.4%)	77.7% (73.6-81.1%)	99.1% (97.8-99.8%)	99.4% (98.2-99.9%)
	MCV2	-	-	-	98.8% (97.5-99.5%)	97.2% (95.0-98.5%)
	PCV3	-	-	-	-	-
	Pol3	40.0% (32.4-47.4%)	77.0% (74.8-79.0%)	88.6% (86.9-90.2%)	98.3% (97.3-98.9%)	98.6% (97.2-99.3%)
	RCV1	-	-	-	-	99.0% (97.8-99.5%)
	RotaC	-	-	-	-	-
Portugal (PRT)	BCG	73.4% (61.0-83.1%)	87.4% (82.0-91.8%)	83.9% (76.4-89.6%)	95.0% (91.5-97.3%)	-
	DTP1	78.5% (71.9-84.1%)	93.4% (91.0-95.3%)	98.6% (97.6-99.3%)	98.3% (97.0-99.2%)	99.4% (98.5-99.8%)
	DTP3	73.0% (66.1-79.2%)	89.1% (85.9-91.8%)	95.6% (93.3-97.3%)	97.2% (95.5-98.4%)	98.9% (97.7-99.6%)
	HepB3	-	-	67.9% (57.1-76.8%)	96.7% (94.5-98.0%)	98.0% (95.5-99.2%)
	Hib3	-	-	90.4% (85.6-93.8%)	96.9% (95.1-98.1%)	98.8% (97.6-99.5%)
	MCV1	51.9% (42.1-61.7%)	91.3% (86.5-94.7%)	91.3% (85.0-95.5%)	97.6% (95.0-99.1%)	99.7% (99.2-100.0%)
	MCV2	-	40.6% (24.4-56.9%)	52.1% (40.2-63.3%)	96.7% (94.0-98.3%)	96.7% (94.5-98.3%)
	PCV3	-	-	-	-	97.3% (96.0-98.2%)
	Pol3	15.7% (11.3-21.3%)	89.1% (86.2-91.7%)	95.8% (93.5-97.4%)	97.3% (95.4-98.6%)	99.3% (98.4-99.7%)
	RCV1	-	91.1% (86.3-94.5%)	91.2% (84.9-95.3%)	97.5% (94.9-99.0%)	99.6% (99.1-99.9%)
	RotaC	-	-	-	-	-
Paraguay (PRY)	BCG	43.9% (35.0-52.3%)	86.4% (84.0-88.5%)	90.8% (88.7-92.5%)	93.1% (88.8-96.2%)	95.0% (89.3-98.0%)
	DTP1	40.2% (29.8-55.0%)	81.2% (74.1-88.9%)	95.1% (92.1-97.5%)	92.7% (89.8-95.1%)	90.3% (84.3-94.9%)
	DTP3	30.9% (23.6-39.0%)	66.6% (63.1-70.1%)	87.5% (84.4-90.2%)	91.6% (88.7-94.1%)	87.8% (81.5-92.7%)
	HepB3	-	-	-	91.2% (87.9-93.7%)	87.6% (81.2-92.5%)
	Hib3	-	-	-	91.4% (88.4-93.9%)	87.7% (81.4-92.5%)
	MCV1	26.4% (18.6-35.8%)	76.3% (69.6-82.1%)	90.6% (86.4-93.6%)	89.2% (83.5-93.7%)	93.7% (86.7-97.7%)
	MCV2	-	-	-	74.3% (66.5-81.2%)	85.4% (77.7-91.2%)
	PCV3	-	-	-	-	87.7% (81.3-92.6%)
	Pol3	21.1% (15.2-28.7%)	67.2% (62.8-71.1%)	87.6% (84.3-90.6%)	90.7% (87.2-93.7%)	82.3% (74.0-89.1%)
	RCV1	-	-	-	89.1% (83.4-93.6%)	93.6% (86.6-97.6%)
	RotaC	-	-	-	76.6% (73.6-79.3%)	87.7% (81.4-92.6%)
Palestine (PSE)	BCG	81.2% (54.6-95.4%)	84.5% (69.6-93.9%)	96.1% (94.3-97.4%)	98.4% (97.9-98.8%)	96.7% (92.5-98.8%)
	DTP1	86.1% (68.0-96.4%)	87.3% (78.3-94.8%)	87.6% (83.8-92.1%)	99.3% (98.6-99.7%)	99.7% (99.3-100.0%)
	DTP3	76.7% (55.2-91.5%)	81.6% (71.9-88.7%)	84.0% (81.0-86.7%)	97.0% (96.0-97.7%)	98.9% (97.3-99.6%)
	HepB3	-	-	83.8% (80.8-86.4%)	96.8% (95.8-97.6%)	98.8% (97.3-99.5%)

Country	Vaccine	1980	1990	2000	2010	2019
	Hib3	-	-	-	96.8% (95.8-97.6%)	98.8% (97.3-99.5%)
	MCV1	83.7% (58.4-96.5%)	89.4% (81.8-94.5%)	88.8% (85.3-91.7%)	97.1% (95.8-98.1%)	99.4% (98.3-99.9%)
	MCV2	-	-	-	95.7% (94.0-97.2%)	98.0% (96.1-99.1%)
	PCV3	-	-	-	-	98.5% (97.0-99.2%)
	Pol3	94.4% (87.5-98.0%)	95.4% (92.4-97.6%)	92.6% (90.1-94.7%)	94.8% (93.4-95.9%)	96.8% (93.7-98.6%)
	RCV1	-	-	-	89.0% (86.2-91.3%)	93.8% (91.8-95.4%)
	RotaC	-	-	-	-	94.9% (93.4-95.7%)
Qatar (QAT)	BCG	20.4% (13.5-27.6%)	95.4% (92.7-97.2%)	99.5% (98.8-99.8%)	96.8% (93.7-98.6%)	98.0% (95.3-99.3%)
	DTP1	59.4% (50.1-68.6%)	89.9% (86.8-92.6%)	90.9% (87.0-93.8%)	97.3% (95.7-98.4%)	98.6% (97.0-99.4%)
	DTP3	49.9% (41.3-58.0%)	82.0% (78.0-85.4%)	80.5% (74.8-85.7%)	95.3% (92.9-97.0%)	97.4% (94.9-98.8%)
	HepB3	-	81.7% (77.7-85.2%)	80.2% (74.7-85.4%)	95.1% (92.7-96.9%)	97.3% (94.8-98.7%)
	Hib3	-	-	80.3% (74.7-85.6%)	95.0% (92.7-96.8%)	97.3% (94.7-98.7%)
	MCV1	23.0% (15.7-31.8%)	81.5% (75.4-86.4%)	91.7% (87.0-95.1%)	99.0% (97.5-99.7%)	99.9% (99.6-100.0%)
	MCV2	-	-	83.5% (77.0-88.7%)	98.4% (96.8-99.2%)	94.4% (91.0-96.8%)
	PCV3	-	-	-	95.1% (92.7-96.9%)	97.3% (94.7-98.7%)
	Pol3	49.5% (41.4-57.0%)	82.2% (78.4-85.8%)	91.7% (88.7-94.1%)	96.0% (93.7-97.6%)	98.4% (96.7-99.4%)
	RCV1	-	-	91.5% (86.9-95.0%)	98.9% (97.4-99.6%)	99.8% (99.5-99.9%)
	RotaC	-	-	-	85.4% (83.0-87.4%)	96.5% (93.9-97.9%)
Romania (ROU)	BCG	90.3% (78.5-97.1%)	96.1% (93.4-98.0%)	99.6% (99.0-99.9%)	99.4% (98.6-99.8%)	96.6% (93.5-98.6%)
	DTP1	99.7% (99.1-99.9%)	99.8% (99.7-99.9%)	99.8% (99.6-99.9%)	98.8% (97.6-99.6%)	97.3% (95.8-98.4%)
	DTP3	89.9% (81.2-95.6%)	95.1% (93.1-96.6%)	97.2% (95.6-98.3%)	93.6% (90.0-96.3%)	88.1% (83.0-91.9%)
	HepB3	-	-	96.5% (94.3-98.0%)	93.5% (89.8-96.2%)	88.0% (82.9-91.8%)
	Hib3	-	-	-	93.4% (89.7-96.0%)	88.0% (82.9-91.8%)
	MCV1	79.5% (62.3-91.4%)	91.4% (87.2-94.6%)	99.2% (98.1-99.7%)	95.4% (91.2-97.8%)	90.4% (84.6-94.8%)
	MCV2	74.3% (57.4-86.0%)	89.0% (84.5-92.6%)	98.1% (96.6-99.0%)	93.4% (89.2-96.1%)	78.2% (70.7-85.3%)
	PCV3	-	-	-	-	56.2% (48.2-63.4%)
	Pol3	87.3% (77.0-93.3%)	91.3% (88.6-93.4%)	97.4% (96.1-98.3%)	92.7% (90.1-95.0%)	87.9% (82.6-91.9%)
	RCV1	-	-	-	95.2% (91.1-97.7%)	90.2% (84.4-94.6%)
	RotaC	-	-	-	-	-
Russia (RUS)	BCG	90.8% (79.2-97.1%)	91.1% (87.5-93.7%)	96.7% (94.4-98.1%)	96.8% (94.7-98.1%)	96.1% (92.8-98.1%)
	DTP1	57.6% (41.1-73.5%)	66.7% (61.9-71.2%)	94.8% (92.8-96.3%)	97.7% (96.2-98.8%)	97.7% (95.6-99.0%)
	DTP3	55.0% (37.5-71.6%)	64.7% (59.6-69.4%)	93.3% (90.9-95.2%)	97.0% (95.3-98.1%)	97.1% (94.8-98.6%)
	HepB3	-	-	19.4% (11.5-29.1%)	96.8% (95.2-98.0%)	97.0% (94.7-98.5%)
	Hib3	-	-	-	-	-
	MCV1	79.3% (55.5-93.5%)	82.6% (77.2-87.3%)	98.1% (95.8-99.2%)	99.7% (99.1-99.9%)	99.5% (98.5-99.9%)
	MCV2	-	28.5% (19.2-39.1%)	86.3% (81.3-90.0%)	98.8% (98.2-99.3%)	98.2% (97.0-99.0%)
	PCV3	-	-	-	-	82.0% (78.1-85.6%)
	Pol3	52.9% (36.4-68.6%)	66.1% (61.6-70.6%)	94.4% (92.5-96.1%)	97.8% (96.6-98.7%)	96.7% (94.0-98.3%)
	RCV1	-	-	93.4% (91.3-94.9%)	99.2% (98.9-99.5%)	99.2% (98.4-99.5%)
	RotaC	-	-	-	-	-
Rwanda (RWA)	BCG	63.1% (49.3-75.2%)	88.3% (86.2-90.3%)	95.1% (94.0-96.0%)	98.5% (97.8-99.0%)	95.6% (93.0-97.6%)
	DTP1	36.7% (25.8-51.2%)	86.8% (84.2-89.2%)	95.4% (94.1-96.6%)	98.6% (98.1-99.1%)	98.0% (96.5-99.0%)
	DTP3	27.9% (19.2-38.2%)	80.8% (78.7-83.0%)	87.6% (86.2-88.9%)	96.9% (95.9-97.8%)	96.1% (93.6-97.8%)
	HepB3	-	-	-	95.2% (93.5-96.4%)	95.8% (93.2-97.6%)
	Hib3	-	-	-	95.7% (94.4-96.7%)	95.9% (93.3-97.6%)
	MCV1	56.6% (41.7-70.0%)	81.1% (77.9-84.1%)	87.2% (84.6-89.5%)	96.1% (94.5-97.3%)	92.1% (87.9-95.2%)
	MCV2	-	-	-	-	88.0% (82.7-91.9%)
	PCV3	-	-	-	95.2% (94.1-96.1%)	96.0% (93.4-97.7%)
	Pol3	21.1% (13.5-32.1%)	81.6% (78.6-84.3%)	86.6% (84.9-88.1%)	93.1% (91.3-94.6%)	93.9% (91.0-96.2%)
	RCV1	-	-	-	-	92.0% (87.8-95.1%)
	RotaC	-	-	-	-	96.0% (93.5-97.7%)
Saudi Arabia (SAU)	BCG	42.5% (34.3-51.0%)	92.6% (88.2-95.8%)	97.1% (94.2-98.8%)	98.5% (96.3-99.5%)	65.0% (53.2-76.4%)
	DTP1	52.0% (43.1-60.9%)	93.9% (92.0-95.7%)	93.5% (91.5-95.2%)	95.2% (93.6-96.5%)	96.2% (94.1-97.9%)
	DTP3	45.8% (37.7-53.5%)	89.7% (87.2-91.9%)	91.2% (88.9-93.1%)	93.9% (92.1-95.3%)	93.8% (91.1-95.9%)

Country	Vaccine	1980	1990	2000	2010	2019
	HepB3	-	68.5% (57.1-77.8%)	89.7% (86.0-92.1%)	93.8% (91.9-95.2%)	93.7% (90.9-95.9%)
	Hib3	-	-	-	93.8% (92.0-95.1%)	93.7% (91.0-95.9%)
	MCV1	15.9% (9.6-24.5%)	90.4% (86.2-93.9%)	97.2% (94.3-98.7%)	99.8% (99.3-100.0%)	99.3% (97.9-99.9%)
	MCV2	-	-	97.1% (94.2-98.6%)	99.5% (99.0-99.8%)	99.0% (97.6-99.7%)
	PCV3	-	-	-	93.8% (92.0-95.2%)	93.7% (91.0-95.9%)
	Pol3	46.0% (38.3-53.4%)	89.6% (86.9-91.7%)	91.5% (89.4-93.3%)	94.7% (93.2-96.0%)	95.4% (92.8-97.2%)
	RCV1	-	90.2% (86.0-93.7%)	97.1% (94.2-98.6%)	99.7% (99.1-99.8%)	99.1% (97.7-99.6%)
	RotaC	-	-	-	-	93.4% (90.6-95.5%)
Sudan (SDN)	BCG	3.6% (1.6-7.0%)	47.6% (41.7-53.0%)	64.6% (60.7-68.2%)	84.4% (81.2-87.1%)	86.2% (80.8-90.7%)
	DTP1	16.4% (7.5-31.1%)	50.7% (43.2-58.7%)	68.3% (62.6-74.4%)	90.2% (86.5-93.3%)	94.5% (89.9-97.7%)
	DTP3	2.4% (1.2-4.3%)	34.5% (29.6-39.6%)	46.1% (42.6-49.6%)	68.2% (65.6-70.7%)	74.9% (69.9-79.7%)
	HepB3	-	-	-	67.9% (65.3-70.5%)	74.8% (69.9-79.6%)
	Hib3	-	-	-	68.1% (65.6-70.7%)	74.8% (69.8-79.6%)
	MCV1	2.6% (0.7-6.7%)	34.2% (27.6-41.1%)	55.5% (49.4-61.3%)	76.2% (71.3-80.6%)	84.4% (76.5-90.2%)
	MCV2	-	-	-	-	69.6% (60.4-77.2%)
	PCV3	-	-	-	-	74.8% (69.8-79.6%)
	Pol3	2.6% (1.3-4.7%)	37.2% (32.1-42.4%)	56.7% (53.0-60.2%)	70.1% (67.2-73.0%)	75.6% (70.1-80.5%)
	RCV1	-	-	-	-	-
	RotaC	-	-	-	-	74.8% (69.8-79.6%)
Senegal (SEN)	BCG	33.3% (25.3-41.8%)	82.5% (80.3-84.4%)	87.7% (85.9-89.4%)	95.0% (94.1-95.8%)	93.6% (90.6-95.7%)
	DTP1	39.1% (27.9-51.9%)	77.2% (73.9-80.2%)	83.3% (80.2-85.9%)	94.2% (93.2-95.1%)	96.6% (94.9-97.8%)
	DTP3	15.0% (10.1-21.0%)	60.0% (56.9-63.2%)	62.0% (59.0-64.8%)	87.3% (85.6-88.8%)	95.5% (93.2-97.1%)
	HepB3	-	-	-	86.7% (84.8-88.4%)	95.3% (93.0-97.0%)
	Hib3	-	-	-	86.3% (84.3-88.1%)	95.3% (93.1-96.9%)
	MCV1	28.8% (17.2-43.3%)	64.1% (59.6-68.6%)	68.5% (63.3-73.0%)	84.1% (81.6-86.5%)	88.5% (82.4-93.2%)
	MCV2	-	-	-	-	70.6% (62.9-77.3%)
	PCV3	-	-	-	-	95.4% (93.2-97.0%)
	Pol3	16.3% (9.6-24.5%)	59.0% (53.7-63.9%)	60.5% (55.7-64.9%)	76.8% (73.9-79.8%)	85.1% (79.8-89.4%)
	RCV1	-	-	-	-	88.4% (82.3-93.1%)
	RotaC	-	-	-	-	95.4% (93.1-97.0%)
Singapore (SGP)	BCG	82.2% (75.3-88.1%)	98.1% (97.0-98.9%)	97.6% (96.4-98.4%)	97.7% (96.6-98.5%)	97.3% (95.1-98.7%)
	DTP1	81.5% (75.4-86.6%)	87.8% (85.0-90.2%)	94.4% (92.9-95.9%)	95.5% (94.0-96.7%)	96.1% (93.9-97.7%)
	DTP3	78.2% (72.0-83.5%)	85.0% (81.9-87.7%)	93.1% (91.3-94.7%)	92.4% (90.3-94.2%)	92.5% (88.9-95.2%)
	HepB3	-	71.9% (59.3-80.3%)	92.3% (90.2-94.1%)	92.1% (89.8-94.0%)	92.3% (88.7-95.0%)
	Hib3	-	-	-	-	92.2% (88.6-95.0%)
	MCV1	49.8% (40.5-59.5%)	81.9% (76.6-86.2%)	90.9% (88.0-93.4%)	89.9% (86.2-92.9%)	90.1% (83.2-94.8%)
	MCV2	-	-	90.8% (87.9-93.3%)	89.7% (86.1-92.7%)	81.9% (74.2-87.7%)
	PCV3	-	-	-	25.8% (21.5-30.5%)	84.3% (80.4-87.7%)
	Pol3	78.1% (72.0-83.8%)	85.3% (82.1-88.1%)	93.0% (91.3-94.5%)	92.4% (90.5-94.1%)	92.2% (88.7-95.0%)
	RCV1	-	81.8% (76.5-86.1%)	90.8% (87.9-93.3%)	89.8% (86.2-92.8%)	90.0% (83.1-94.7%)
	RotaC	-	-	-	-	-
Solomon Islands (SLB)	BCG	57.7% (47.8-66.9%)	84.7% (79.2-88.9%)	91.9% (88.4-94.3%)	90.0% (86.3-93.1%)	86.5% (78.8-92.2%)
	DTP1	46.3% (36.5-57.7%)	76.2% (70.3-81.7%)	85.6% (81.2-89.4%)	87.8% (84.3-90.7%)	94.8% (90.9-97.7%)
	DTP3	37.6% (29.8-46.8%)	68.9% (63.0-74.0%)	77.2% (72.3-81.8%)	80.9% (76.7-84.6%)	89.3% (83.6-93.7%)
	HepB3	-	54.3% (36.9-66.2%)	76.5% (71.5-81.2%)	80.7% (76.5-84.4%)	89.2% (83.4-93.6%)
	Hib3	-	-	-	80.1% (76.0-83.8%)	89.2% (83.4-93.5%)
	MCV1	38.8% (14.5-67.9%)	68.7% (60.0-76.6%)	71.4% (62.7-79.2%)	72.3% (64.4-79.5%)	94.7% (88.3-98.1%)
	MCV2	-	-	-	-	59.3% (47.8-70.2%)
	PCV3	-	-	-	-	89.0% (83.3-93.4%)
	Pol3	38.4% (29.9-46.8%)	68.8% (62.3-74.5%)	79.2% (74.7-83.8%)	79.8% (75.4-83.9%)	85.0% (78.7-89.8%)
	RCV1	-	-	-	-	94.6% (88.2-97.9%)
	RotaC	-	-	-	-	-
Sierra Leone (SLE)	BCG	36.6% (28.4-45.4%)	78.5% (74.2-82.5%)	73.6% (70.4-76.4%)	93.0% (91.0-94.6%)	95.8% (92.5-98.1%)
	DTP1	32.7% (22.1-44.8%)	90.2% (85.5-93.6%)	69.9% (65.4-75.0%)	90.8% (88.3-93.0%)	92.9% (88.3-96.3%)

Country	Vaccine	1980	1990	2000	2010	2019
	DTP3	15.9% (9.5-25.0%)	67.2% (60.0-75.0%)	44.9% (40.1-49.9%)	75.3% (71.5-79.1%)	87.4% (80.8-92.1%)
	HepB3	-	-	-	73.8% (69.8-77.7%)	87.2% (80.6-91.9%)
	Hib3	-	-	-	75.2% (71.3-79.0%)	87.3% (80.6-92.0%)
	MCV1	31.7% (21.3-43.2%)	59.4% (50.3-68.2%)	50.1% (43.9-56.2%)	81.3% (76.1-85.6%)	90.3% (81.6-95.6%)
	MCV2	-	-	-	-	67.1% (57.3-75.7%)
	PCV3	-	-	-	-	87.1% (80.5-91.8%)
	Pol3	13.1% (7.5-21.7%)	72.6% (65.3-79.6%)	48.8% (43.0-54.7%)	72.1% (67.3-76.9%)	85.3% (78.4-90.5%)
	RCV1	-	-	-	-	90.1% (81.5-95.5%)
	RotaC	-	-	-	-	86.9% (80.3-91.6%)
El Salvador (SLV)	BCG	69.4% (61.7-77.0%)	77.5% (73.2-81.3%)	94.8% (93.3-96.1%)	98.4% (97.2-99.2%)	86.3% (78.3-91.8%)
	DTP1	49.0% (39.6-60.3%)	71.2% (65.8-77.4%)	94.8% (92.6-96.8%)	95.7% (93.7-97.4%)	87.3% (81.6-92.2%)
	DTP3	43.9% (36.2-51.8%)	66.9% (62.7-70.8%)	90.4% (88.2-92.2%)	93.5% (91.3-95.4%)	84.5% (78.2-89.5%)
	HepB3	-	-	84.4% (73.1-90.0%)	93.3% (90.9-95.2%)	84.4% (77.9-89.5%)
	Hib3	-	-	-	93.4% (91.2-95.3%)	84.4% (78.0-89.5%)
	MCV1	59.0% (48.8-68.5%)	83.2% (77.8-87.8%)	85.8% (81.5-89.6%)	95.6% (91.9-97.8%)	92.1% (87.0-95.7%)
	MCV2	-	-	71.3% (61.7-78.5%)	83.2% (77.5-88.0%)	91.9% (86.9-95.6%)
	PCV3	-	-	-	84.7% (81.1-87.9%)	84.2% (78.0-89.3%)
	Pol3	40.7% (33.3-48.4%)	67.4% (63.2-71.6%)	91.0% (89.0-92.7%)	93.9% (91.8-95.5%)	83.9% (77.9-89.0%)
	RCV1	-	-	85.7% (81.3-89.5%)	95.3% (91.7-97.5%)	92.0% (86.9-95.6%)
	RotaC	-	-	-	92.6% (90.4-94.5%)	84.4% (78.1-89.5%)
San Marino (SMR)	BCG	-	-	-	-	-
	DTP1	99.8% (99.3-100.0%)	99.5% (98.8-99.9%)	97.4% (95.4-98.7%)	91.8% (89.2-94.1%)	91.9% (87.9-95.1%)
	DTP3	98.4% (95.9-99.6%)	97.2% (94.5-98.8%)	95.0% (91.8-97.3%)	90.4% (87.4-92.9%)	88.1% (83.0-92.1%)
	HepB3	-	-	94.7% (91.5-97.1%)	89.3% (85.8-92.3%)	83.7% (76.4-89.4%)
	Hib3	-	-	92.9% (89.0-95.8%)	88.3% (84.7-91.1%)	87.2% (81.8-91.4%)
	MCV1	99.6% (98.7-99.9%)	99.6% (98.9-99.9%)	95.8% (90.2-98.6%)	88.6% (84.0-92.2%)	87.6% (79.9-92.9%)
	MCV2	-	-	-	88.5% (83.9-92.1%)	82.7% (74.6-88.8%)
	PCV3	-	-	-	-	71.5% (66.1-77.0%)
	Pol3	99.5% (98.8-99.9%)	99.6% (98.9-99.9%)	95.3% (91.9-97.5%)	90.3% (87.2-92.6%)	88.6% (83.4-92.6%)
	RCV1	-	-	95.6% (90.1-98.5%)	88.4% (83.8-92.1%)	87.5% (79.8-92.8%)
	RotaC	-	-	-	-	-
Somalia (SOM)	BCG	16.1% (9.4-25.7%)	39.4% (29.0-49.9%)	49.0% (41.9-55.7%)	23.1% (17.2-29.8%)	41.9% (32.1-51.2%)
	DTP1	36.0% (28.1-45.4%)	49.8% (42.5-57.9%)	48.6% (43.4-53.7%)	51.8% (45.8-58.1%)	48.9% (39.2-58.2%)
	DTP3	14.2% (9.8-19.5%)	28.7% (21.8-35.0%)	25.1% (21.0-30.0%)	18.5% (14.3-24.1%)	14.8% (9.5-21.4%)
	HepB3	-	-	-	-	14.8% (9.5-21.4%)
	Hib3	-	-	-	-	14.8% (9.5-21.4%)
	MCV1	16.8% (8.2-28.1%)	25.9% (15.3-40.4%)	28.3% (20.3-36.4%)	46.9% (37.3-56.1%)	24.5% (15.3-36.6%)
	MCV2	-	-	-	-	-
	PCV3	-	-	-	-	-
	Pol3	14.7% (10.3-20.7%)	33.2% (26.6-40.7%)	39.5% (34.9-44.1%)	38.4% (32.8-43.6%)	29.6% (23.5-36.5%)
	RCV1	-	-	-	-	-
	RotaC	-	-	-	-	-
Serbia (SRB)	BCG	99.6% (98.7-99.9%)	99.2% (98.1-99.8%)	92.8% (90.5-94.6%)	93.4% (91.0-95.3%)	96.7% (93.5-98.7%)
	DTP1	92.5% (88.0-95.7%)	90.0% (86.9-92.3%)	97.0% (95.9-97.8%)	90.9% (88.1-93.5%)	87.7% (83.5-91.8%)
	DTP3	86.8% (80.0-92.2%)	85.3% (81.1-88.4%)	90.1% (87.6-92.3%)	87.1% (84.4-89.6%)	85.2% (80.9-88.7%)
	HepB3	-	-	-	82.2% (74.7-86.8%)	80.3% (71.1-85.9%)
	Hib3	-	-	-	86.4% (83.6-89.0%)	85.1% (80.7-88.6%)
	MCV1	90.5% (81.5-95.6%)	82.7% (77.5-87.1%)	87.6% (83.7-90.7%)	98.5% (97.2-99.2%)	95.3% (90.1-98.2%)
	MCV2	-	-	79.0% (73.0-84.0%)	94.5% (91.3-96.6%)	94.9% (89.6-97.8%)
	PCV3	-	-	-	-	70.3% (65.1-75.2%)
	Pol3	98.7% (97.7-99.4%)	88.5% (84.1-92.1%)	98.0% (96.8-98.8%)	96.6% (94.8-97.8%)	90.9% (87.1-93.7%)
	RCV1	-	-	87.5% (83.6-90.6%)	98.3% (97.1-99.1%)	95.2% (90.0-98.1%)
	RotaC	-	-	-	-	-
South Sudan (SSD)	BCG	38.0% (9.7-74.5%)	40.8% (12.2-76.5%)	37.4% (16.5-62.8%)	63.1% (55.9-70.0%)	67.5% (57.0-78.3%)

Country	Vaccine	1980	1990	2000	2010	2019
	DTP1	58.3% (30.7-85.0%)	59.8% (37.9-80.5%)	35.7% (21.6-51.8%)	60.9% (51.5-70.3%)	67.2% (55.4-78.7%)
	DTP3	30.1% (11.3-55.4%)	31.9% (15.5-51.0%)	21.9% (11.0-35.3%)	43.9% (35.1-52.9%)	55.5% (43.2-67.2%)
	HepB3	-	-	-	-	55.4% (43.2-67.1%)
	Hib3	-	-	-	-	55.4% (43.1-67.1%)
	MCV1	37.7% (11.3-73.0%)	40.2% (18.3-66.4%)	30.2% (11.0-55.7%)	52.0% (43.7-60.6%)	46.2% (32.8-59.7%)
	MCV2	-	-	-	-	-
	PCV3	-	-	-	-	-
	Pol3	30.5% (11.4-55.7%)	32.9% (16.2-54.2%)	22.6% (11.5-37.4%)	42.7% (32.3-53.7%)	54.4% (40.5-67.2%)
	RCV1	-	-	-	-	-
	RotaC	-	-	-	-	-
São Tomé and Príncipe (STP)	BCG	86.5% (77.0-92.5%)	87.1% (83.3-90.3%)	84.1% (80.1-87.8%)	96.2% (94.2-97.7%)	93.8% (89.1-96.9%)
	DTP1	45.7% (34.0-58.5%)	85.0% (79.7-90.2%)	86.7% (81.9-91.5%)	94.6% (92.2-96.6%)	95.9% (92.6-98.1%)
	DTP3	40.1% (29.5-52.2%)	78.1% (73.9-82.0%)	78.0% (73.8-82.0%)	91.7% (88.7-94.2%)	93.2% (88.6-96.1%)
	HepB3	-	-	-	91.5% (88.4-93.9%)	93.0% (88.4-96.0%)
	Hib3	-	-	-	91.6% (88.6-94.0%)	93.1% (88.5-96.0%)
	MCV1	34.7% (20.8-51.4%)	70.2% (62.8-77.3%)	74.2% (67.1-80.2%)	93.4% (88.0-96.8%)	98.7% (96.6-99.7%)
	MCV2	-	-	-	-	82.9% (75.6-88.6%)
	PCV3	-	-	-	-	93.1% (88.5-96.0%)
	Pol3	49.6% (37.8-61.9%)	77.6% (73.0-81.9%)	75.3% (70.4-79.8%)	90.5% (86.9-93.1%)	92.9% (88.1-96.0%)
	RCV1	-	-	-	-	98.6% (96.5-99.6%)
	RotaC	-	-	-	-	93.0% (88.4-95.9%)
Suriname (SUR)	BCG	-	-	-	-	-
	DTP1	52.6% (42.1-64.3%)	93.2% (90.2-95.3%)	96.4% (94.7-97.6%)	94.3% (92.3-96.0%)	84.2% (79.1-88.5%)
	DTP3	35.4% (27.4-44.6%)	82.3% (77.4-86.7%)	87.6% (83.7-90.7%)	87.4% (83.7-90.3%)	76.9% (71.4-82.0%)
	HepB3	-	-	-	83.7% (77.7-87.6%)	76.7% (71.2-81.8%)
	Hib3	-	-	-	85.5% (81.5-88.7%)	76.8% (71.3-81.9%)
	MCV1	16.5% (9.1-26.6%)	59.4% (52.1-66.1%)	78.6% (72.0-84.1%)	81.6% (75.8-86.6%)	72.4% (61.4-81.6%)
	MCV2	-	-	-	-	31.4% (24.3-39.6%)
	PCV3	-	-	-	-	-
	Pol3	48.1% (38.1-58.3%)	83.4% (78.4-87.8%)	86.9% (82.6-90.3%)	88.5% (84.8-91.6%)	94.2% (91.5-96.2%)
	RCV1	-	-	78.5% (71.8-84.0%)	81.5% (75.7-86.5%)	72.3% (61.3-81.5%)
	RotaC	-	-	-	-	-
Slovakia (SVK)	BCG	93.2% (80.4-98.5%)	95.0% (91.0-97.6%)	94.9% (91.9-97.1%)	96.4% (93.8-98.2%)	-
	DTP1	99.7% (98.9-100.0%)	99.8% (99.6-99.9%)	98.7% (97.9-99.3%)	98.9% (98.1-99.4%)	96.8% (94.4-98.4%)
	DTP3	97.0% (92.4-99.1%)	98.0% (96.6-98.9%)	98.6% (97.7-99.2%)	98.9% (98.1-99.4%)	96.8% (94.3-98.4%)
	HepB3	-	-	97.7% (95.5-98.7%)	98.7% (97.9-99.3%)	96.7% (94.0-98.3%)
	Hib3	-	-	94.4% (90.5-96.9%)	98.7% (97.9-99.2%)	96.7% (94.2-98.2%)
	MCV1	99.3% (97.7-99.9%)	99.3% (98.3-99.8%)	99.7% (99.1-99.9%)	99.7% (99.1-99.9%)	97.0% (93.8-98.8%)
	MCV2	99.1% (97.4-99.7%)	99.2% (98.2-99.7%)	99.6% (99.0-99.8%)	99.5% (99.0-99.8%)	96.9% (93.7-98.7%)
	PCV3	-	-	-	98.7% (98.0-99.3%)	96.7% (94.1-98.2%)
	Pol3	97.4% (93.7-99.2%)	98.1% (96.8-99.1%)	98.3% (97.3-99.1%)	99.2% (98.6-99.6%)	97.1% (94.9-98.6%)
	RCV1	-	98.6% (97.6-99.2%)	99.3% (98.7-99.6%)	99.6% (99.0-99.8%)	96.9% (93.7-98.7%)
	RotaC	-	-	-	-	-
Slovenia (SVN)	BCG	83.9% (62.2-96.1%)	89.9% (82.6-94.9%)	97.0% (94.8-98.5%)	-	-
	DTP1	99.7% (98.9-100.0%)	99.8% (99.6-100.0%)	96.3% (94.8-97.5%)	97.8% (96.1-99.0%)	96.9% (93.9-99.2%)
	DTP3	95.3% (88.4-98.5%)	97.4% (95.3-98.8%)	92.4% (89.5-94.6%)	96.1% (94.1-97.5%)	94.4% (91.0-96.7%)
	HepB3	-	-	92.2% (89.3-94.5%)	94.9% (92.2-96.7%)	87.8% (80.4-92.9%)
	Hib3	-	-	92.2% (89.3-94.4%)	95.9% (93.9-97.4%)	94.3% (90.8-96.6%)
	MCV1	86.1% (78.8-91.3%)	90.0% (85.5-93.4%)	94.8% (91.5-97.2%)	96.5% (93.5-98.2%)	94.7% (90.1-97.6%)
	MCV2	85.7% (78.5-91.1%)	89.8% (85.3-93.2%)	94.7% (91.3-97.2%)	96.3% (93.4-98.1%)	94.6% (90.0-97.4%)
	PCV3	-	-	-	-	72.0% (65.7-77.5%)
	Pol3	86.5% (81.1-90.7%)	95.7% (93.6-97.1%)	93.2% (90.4-95.4%)	96.4% (94.5-97.7%)	94.8% (91.7-97.0%)
	RCV1	85.7% (78.4-91.0%)	89.7% (85.3-93.2%)	94.7% (91.3-97.1%)	96.3% (93.4-98.1%)	94.6% (90.0-97.5%)
	RotaC	-	-	-	-	-

Country	Vaccine	1980	1990	2000	2010	2019
Sweden (SWE)	BCG	-	-	-	-	-
	DTP1	99.9% (99.8-100.0%)	99.9% (99.8-100.0%)	99.9% (99.8-100.0%)	98.9% (98.1-99.5%)	97.8% (96.0-99.0%)
	DTP3	99.2% (98.0-99.7%)	99.2% (98.3-99.7%)	99.2% (98.4-99.6%)	98.2% (97.0-99.0%)	97.8% (95.9-99.0%)
	HepB3	-	-	-	-	90.6% (83.7-95.1%)
	Hib3	-	-	98.3% (97.0-99.1%)	98.0% (96.9-98.9%)	97.4% (95.5-98.7%)
	MCV1	90.6% (84.0-94.9%)	96.9% (94.3-98.5%)	93.5% (89.7-96.2%)	98.0% (95.9-99.2%)	98.3% (95.8-99.5%)
	MCV2	-	96.5% (93.9-98.2%)	93.3% (89.6-96.0%)	95.6% (93.0-97.5%)	96.0% (93.0-97.8%)
	PCV3	-	-	-	97.4% (96.3-98.3%)	97.4% (95.6-98.7%)
	Pol3	98.0% (96.0-99.1%)	99.5% (99.0-99.8%)	99.5% (99.0-99.8%)	98.5% (97.3-99.3%)	98.1% (96.5-99.2%)
	RCV1	-	95.0% (92.3-96.8%)	92.4% (88.7-95.2%)	97.8% (95.7-99.0%)	98.1% (95.7-99.3%)
	RotaC	-	-	-	-	27.1% (24.7-29.7%)
eSwatini (SWZ)	BCG	60.1% (52.1-67.9%)	96.2% (94.5-97.5%)	94.8% (92.5-96.7%)	96.2% (94.2-97.6%)	99.5% (98.8-99.9%)
	DTP1	51.4% (40.1-65.0%)	93.9% (91.7-95.8%)	96.4% (95.1-97.6%)	97.2% (95.9-98.2%)	93.8% (89.5-96.7%)
	DTP3	30.7% (24.3-37.7%)	87.4% (84.2-90.1%)	92.0% (90.0-93.7%)	93.6% (91.4-95.2%)	86.9% (80.9-91.2%)
	HepB3	-	-	88.4% (84.9-91.1%)	93.3% (91.2-95.0%)	86.5% (80.7-90.7%)
	Hib3	-	-	-	93.4% (91.3-95.1%)	86.6% (80.7-90.9%)
	MCV1	24.6% (17.4-33.7%)	88.0% (82.9-92.2%)	86.3% (81.4-90.0%)	94.1% (91.0-96.4%)	87.9% (80.9-93.1%)
	MCV2	-	-	55.2% (42.9-66.6%)	91.9% (88.6-94.3%)	80.5% (72.8-86.8%)
	PCV3	-	-	-	-	85.1% (79.2-89.2%)
	Pol3	28.4% (21.6-36.2%)	89.0% (85.3-92.0%)	82.2% (78.1-85.7%)	91.7% (89.0-93.9%)	86.1% (79.8-91.2%)
	RCV1	-	-	-	-	87.6% (80.6-92.8%)
	RotaC	-	-	-	-	86.0% (80.1-90.2%)
Seychelles (SYC)	BCG	67.2% (53.5-78.7%)	98.7% (97.4-99.4%)	99.7% (99.0-99.9%)	99.4% (98.3-99.9%)	98.9% (96.6-99.8%)
	DTP1	36.1% (23.8-51.0%)	98.9% (98.1-99.5%)	99.1% (98.4-99.6%)	97.8% (96.4-98.8%)	97.6% (95.6-98.8%)
	DTP3	24.7% (15.7-35.5%)	96.4% (94.5-97.9%)	97.3% (95.7-98.5%)	97.6% (95.9-98.6%)	97.2% (95.0-98.6%)
	HepB3	-	-	96.4% (94.4-97.8%)	97.2% (95.4-98.4%)	97.1% (94.7-98.5%)
	Hib3	-	-	-	91.9% (87.1-95.0%)	97.0% (94.7-98.4%)
	MCV1	31.6% (20.1-46.1%)	88.3% (82.2-93.1%)	97.9% (95.5-99.2%)	99.5% (98.6-99.9%)	99.4% (98.2-99.9%)
	MCV2	-	88.1% (82.1-92.9%)	97.8% (95.3-99.1%)	99.1% (98.1-99.6%)	99.3% (98.1-99.7%)
	PCV3	-	-	-	-	69.9% (62.7-76.6%)
	Pol3	28.0% (18.3-39.0%)	95.5% (93.4-97.1%)	97.0% (95.3-98.1%)	97.5% (96.2-98.5%)	97.2% (95.2-98.6%)
	RCV1	31.5% (20.1-46.0%)	88.1% (82.1-92.9%)	97.8% (95.3-99.1%)	99.4% (98.5-99.8%)	99.3% (98.1-99.8%)
	RotaC	-	-	-	-	96.7% (94.5-98.0%)
Syria (SYR)	BCG	48.3% (40.4-56.3%)	90.0% (88.2-91.8%)	95.7% (94.2-97.0%)	93.5% (92.0-94.9%)	81.7% (76.0-87.0%)
	DTP1	39.5% (24.9-58.0%)	95.0% (92.9-96.8%)	91.4% (87.6-94.4%)	90.7% (87.6-93.7%)	80.4% (71.0-88.8%)
	DTP3	17.1% (12.2-23.9%)	88.3% (85.5-90.7%)	80.8% (77.1-84.1%)	85.1% (81.9-87.9%)	64.0% (56.0-72.2%)
	HepB3	-	-	72.1% (66.0-77.2%)	83.9% (80.5-87.0%)	63.9% (55.8-72.0%)
	Hib3	-	-	-	85.0% (81.8-87.8%)	64.0% (55.9-72.1%)
	MCV1	25.1% (15.8-35.3%)	83.6% (76.8-89.2%)	76.7% (70.2-82.3%)	86.3% (80.4-90.4%)	69.5% (58.0-78.7%)
	MCV2	-	-	75.5% (69.1-81.1%)	85.7% (79.9-89.7%)	63.4% (52.9-72.6%)
	PCV3	-	-	-	-	-
	Pol3	17.0% (11.6-23.1%)	87.5% (84.3-89.9%)	76.5% (72.5-80.3%)	80.1% (76.3-83.5%)	64.5% (55.8-72.3%)
	RCV1	-	-	72.0% (65.7-77.5%)	86.2% (80.3-90.3%)	69.4% (57.9-78.6%)
	RotaC	-	-	-	-	-
Chad (TCD)	BCG	16.1% (7.1-31.0%)	44.6% (39.5-50.1%)	42.9% (39.0-46.6%)	58.6% (54.8-62.2%)	59.5% (51.5-67.7%)
	DTP1	24.1% (11.1-45.2%)	39.0% (32.5-46.5%)	41.1% (35.8-46.4%)	62.5% (56.8-68.9%)	63.4% (54.1-73.1%)
	DTP3	4.2% (1.8-8.1%)	20.2% (16.8-23.6%)	21.3% (19.2-23.7%)	31.0% (28.4-33.6%)	39.5% (32.8-46.1%)
	HepB3	-	-	-	30.4% (27.9-33.0%)	39.4% (32.7-46.1%)
	Hib3	-	-	-	30.8% (28.3-33.4%)	39.4% (32.7-46.0%)
	MCV1	24.5% (7.0-53.4%)	28.2% (21.8-35.5%)	27.5% (22.8-32.6%)	57.3% (51.8-62.9%)	40.8% (30.7-51.9%)
	MCV2	-	-	-	-	-
	PCV3	-	-	-	-	-
	Pol3	15.5% (6.9-29.2%)	20.1% (14.7-26.3%)	44.2% (38.6-49.5%)	49.4% (43.7-54.4%)	47.1% (37.2-56.7%)
	RCV1	-	-	-	-	-

Country	Vaccine	1980	1990	2000	2010	2019
	RotaC	-	-	-	-	-
Togo (TGO)	BCG	41.5% (29.4-54.2%)	92.3% (89.9-94.1%)	84.7% (82.3-86.9%)	94.8% (93.4-96.0%)	95.5% (92.4-97.7%)
	DTP1	27.0% (17.6-38.9%)	90.2% (88.1-92.2%)	78.3% (75.3-81.3%)	91.1% (89.3-92.7%)	86.8% (82.0-91.0%)
	DTP3	12.3% (7.4-19.0%)	74.6% (70.7-78.2%)	58.0% (54.4-61.7%)	78.8% (75.8-81.5%)	79.3% (72.6-84.8%)
	HepB3	-	-	-	76.5% (71.9-80.1%)	79.1% (72.4-84.7%)
	Hib3	-	-	-	77.5% (74.2-80.6%)	79.2% (72.5-84.8%)
	MCV1	24.1% (8.6-49.1%)	77.4% (70.3-83.1%)	59.7% (53.7-65.2%)	75.7% (71.1-79.9%)	77.9% (68.0-85.9%)
	MCV2	-	-	-	-	58.5% (46.2-69.4%)
	PCV3	-	-	-	-	78.9% (72.3-84.5%)
	Pol3	12.5% (6.6-20.7%)	74.4% (69.0-79.5%)	62.1% (57.1-66.7%)	69.8% (64.9-73.9%)	72.8% (64.6-79.9%)
	RCV1	-	-	-	-	77.8% (67.9-85.8%)
	RotaC	-	-	-	-	78.9% (72.3-84.5%)
Thailand (THA)	BCG	46.0% (39.0-53.8%)	97.1% (96.1-97.9%)	96.8% (95.7-97.7%)	97.7% (97.0-98.3%)	96.3% (94.3-97.8%)
	DTP1	49.3% (39.8-60.0%)	96.2% (94.8-97.2%)	97.7% (96.4-98.7%)	96.1% (94.6-97.4%)	91.1% (87.1-94.9%)
	DTP3	41.3% (34.0-49.6%)	95.1% (93.6-96.3%)	92.4% (90.3-94.3%)	92.3% (90.7-93.7%)	87.2% (83.4-90.3%)
	HepB3	-	-	90.0% (85.8-92.8%)	87.2% (82.9-90.1%)	87.0% (83.2-90.1%)
	Hib3	-	-	-	-	87.0% (83.2-90.1%)
	MCV1	-	83.2% (80.0-86.2%)	95.6% (93.8-97.0%)	96.2% (94.9-97.2%)	90.3% (85.9-93.6%)
	MCV2	-	-	95.2% (93.4-96.6%)	89.4% (85.3-92.5%)	80.6% (74.1-85.5%)
	PCV3	-	-	-	-	-
	Pol3	24.8% (19.8-30.8%)	90.1% (88.1-91.9%)	94.3% (92.7-95.5%)	93.6% (92.4-94.7%)	90.7% (87.7-93.0%)
	RCV1	-	-	95.4% (93.6-96.8%)	96.1% (94.8-97.1%)	90.2% (85.8-93.5%)
	RotaC	-	-	-	-	86.9% (83.2-90.0%)
Tajikistan (TJK)	BCG	65.8% (40.9-87.3%)	81.1% (76.1-85.5%)	93.2% (91.9-94.4%)	98.2% (97.5-98.8%)	96.2% (94.1-97.6%)
	DTP1	71.9% (58.1-84.4%)	80.6% (76.8-84.1%)	91.3% (89.3-93.0%)	96.3% (95.3-97.2%)	93.4% (91.1-95.5%)
	DTP3	64.6% (50.0-78.3%)	75.5% (71.8-78.9%)	81.9% (79.8-83.9%)	92.2% (90.6-93.7%)	89.0% (85.8-91.8%)
	HepB3	-	-	-	89.0% (85.6-91.4%)	88.8% (85.6-91.6%)
	Hib3	-	-	-	89.2% (86.6-91.4%)	88.8% (85.6-91.6%)
	MCV1	79.9% (71.3-86.8%)	81.0% (75.0-86.1%)	85.9% (82.1-89.0%)	96.6% (93.9-98.2%)	90.2% (84.5-94.1%)
	MCV2	-	32.4% (18.4-47.0%)	42.8% (34.5-51.9%)	94.5% (91.7-96.6%)	89.4% (83.7-93.4%)
	PCV3	-	-	-	-	-
	Pol3	76.9% (63.6-87.2%)	77.8% (74.3-81.1%)	82.8% (80.6-84.8%)	91.1% (89.4-92.7%)	88.1% (85.2-90.9%)
	RCV1	-	-	-	96.5% (93.8-98.1%)	90.1% (84.4-94.0%)
	RotaC	-	-	-	-	88.9% (85.7-91.7%)
Tokelau (TKL)	BCG	-	-	-	-	-
	DTP1	59.0% (31.5-84.4%)	94.0% (82.4-99.2%)	95.1% (85.7-99.2%)	95.6% (87.7-99.1%)	97.4% (92.1-99.5%)
	DTP3	45.0% (19.3-73.4%)	84.1% (64.0-95.4%)	90.2% (73.7-97.4%)	93.8% (83.5-98.5%)	95.9% (88.6-99.0%)
	HepB3	-	74.2% (50.4-90.3%)	89.3% (73.3-96.8%)	93.5% (83.2-98.3%)	95.7% (88.5-98.9%)
	Hib3	-	-	-	-	-
	MCV1	42.5% (14.7-76.8%)	83.5% (57.7-96.4%)	92.3% (78.1-98.5%)	94.6% (83.6-99.0%)	96.9% (90.0-99.5%)
	MCV2	-	-	-	-	-
	PCV3	-	-	-	-	-
	Pol3	43.8% (18.8-70.8%)	81.0% (60.1-94.2%)	89.7% (75.9-97.0%)	93.4% (82.5-98.2%)	95.3% (87.9-98.8%)
	RCV1	-	-	-	-	-
	RotaC	-	-	-	-	-
Turkmenistan (TKM)	BCG	84.5% (61.3-95.5%)	89.4% (85.4-92.9%)	98.5% (97.8-99.1%)	98.8% (97.9-99.3%)	98.1% (96.5-99.1%)
	DTP1	72.1% (52.0-88.5%)	77.8% (71.1-84.2%)	98.2% (97.4-98.9%)	99.2% (98.5-99.7%)	99.7% (99.4-99.9%)
	DTP3	66.9% (43.6-84.7%)	74.0% (67.3-79.6%)	97.0% (95.9-97.9%)	98.0% (96.5-99.0%)	99.6% (99.1-99.9%)
	HepB3	-	-	-	97.6% (95.8-98.7%)	99.4% (98.7-99.8%)
	Hib3	-	-	-	61.9% (52.1-71.3%)	99.4% (98.8-99.7%)
	MCV1	56.7% (24.3-84.3%)	64.9% (56.1-72.6%)	96.7% (95.3-97.7%)	93.6% (91.6-95.1%)	94.2% (91.6-96.2%)
	MCV2	-	50.6% (39.5-60.9%)	88.9% (84.4-92.5%)	93.4% (91.3-95.0%)	94.1% (91.5-96.1%)
	PCV3	-	-	-	-	90.3% (86.8-93.2%)
	Pol3	79.3% (62.4-91.6%)	84.8% (80.5-88.6%)	95.4% (93.9-96.6%)	96.6% (94.3-98.0%)	99.6% (99.2-99.9%)

Country	Vaccine	1980	1990	2000	2010	2019
	RCV1	-	-	-	93.5% (91.5-95.0%)	94.1% (91.5-96.1%)
	RotaC	-	-	-	-	6.0% (5.3-6.6%)
Timor-Leste (TLS)	BCG	20.2% (4.6-49.1%)	35.8% (15.5-61.4%)	42.2% (38.1-46.8%)	76.9% (72.2-81.4%)	79.4% (71.8-85.4%)
	DTP1	30.5% (10.6-62.3%)	44.3% (23.6-66.9%)	49.1% (40.2-58.1%)	76.2% (70.9-80.6%)	73.2% (65.1-80.8%)
	DTP3	12.2% (3.4-28.6%)	24.3% (10.4-44.1%)	31.4% (24.0-38.8%)	68.4% (62.5-73.4%)	62.8% (54.3-71.6%)
	HepB3	-	-	-	67.8% (62.0-72.9%)	62.7% (54.2-71.5%)
	Hib3	-	-	-	-	62.7% (54.2-71.6%)
	MCV1	15.9% (3.9-40.0%)	33.4% (10.4-64.0%)	36.5% (30.0-43.2%)	69.7% (62.8-76.2%)	68.6% (59.0-77.5%)
	MCV2	-	-	-	-	53.7% (44.1-62.4%)
	PCV3	-	-	-	-	-
	Pol3	14.2% (4.5-32.6%)	26.1% (11.7-45.0%)	32.6% (25.1-40.7%)	60.4% (54.3-66.1%)	56.7% (47.1-65.4%)
	RCV1	-	-	-	-	68.5% (58.9-77.5%)
	RotaC	-	-	-	-	57.8% (50.1-65.9%)
Tonga (TON)	BCG	83.8% (80.1-87.2%)	85.9% (83.7-88.0%)	86.6% (84.7-88.3%)	87.0% (85.2-88.7%)	87.5% (84.4-90.1%)
	DTP1	49.2% (40.5-59.2%)	99.0% (98.2-99.5%)	99.7% (99.4-99.9%)	99.7% (99.3-99.9%)	99.8% (99.5-100.0%)
	DTP3	40.4% (32.7-47.9%)	97.4% (95.6-98.6%)	98.9% (97.8-99.6%)	99.7% (99.2-99.9%)	99.8% (99.5-100.0%)
	HepB3	-	95.5% (92.7-97.3%)	97.8% (96.1-98.9%)	99.4% (98.7-99.8%)	99.7% (99.2-99.9%)
	Hib3	-	-	-	99.5% (99.1-99.8%)	99.7% (99.3-99.9%)
	MCV1	23.4% (13.4-36.4%)	79.1% (73.1-84.2%)	87.3% (83.2-90.9%)	91.3% (88.4-93.7%)	91.9% (87.8-95.2%)
	MCV2	-	-	-	90.8% (87.9-93.2%)	91.8% (87.7-95.0%)
	PCV3	-	-	-	-	-
	Pol3	33.3% (25.8-41.0%)	97.8% (96.4-98.8%)	99.3% (98.7-99.7%)	99.7% (99.4-99.9%)	99.8% (99.5-100.0%)
	RCV1	-	-	-	91.2% (88.3-93.6%)	91.8% (87.7-95.1%)
	RotaC	-	-	-	-	-
Trinidad and Tobago (TTO)	BCG	-	-	-	-	-
	DTP1	39.2% (31.3-47.5%)	69.6% (65.4-73.8%)	77.7% (74.4-80.5%)	81.8% (79.0-84.7%)	74.6% (69.3-79.1%)
	DTP3	35.9% (28.3-43.7%)	67.1% (62.9-71.2%)	76.1% (72.5-79.0%)	71.5% (68.1-74.6%)	74.0% (68.5-78.7%)
	HepB3	-	-	-	66.9% (62.0-71.0%)	73.7% (68.3-78.5%)
	Hib3	-	-	49.6% (40.7-57.7%)	68.9% (65.3-72.0%)	73.8% (68.4-78.5%)
	MCV1	-	81.2% (73.6-86.9%)	95.2% (90.7-97.9%)	89.2% (84.5-93.0%)	92.7% (86.8-96.4%)
	MCV2	-	-	-	88.1% (83.4-92.0%)	89.8% (83.9-93.8%)
	PCV3	-	-	-	-	73.9% (68.5-78.5%)
	Pol3	44.6% (37.2-52.4%)	77.8% (73.8-81.4%)	86.2% (83.1-89.0%)	83.2% (79.8-86.4%)	87.8% (83.8-91.3%)
	RCV1	-	81.0% (73.4-86.7%)	95.1% (90.5-97.7%)	89.1% (84.4-92.9%)	92.6% (86.7-96.4%)
	RotaC	-	-	-	-	-
Tunisia (TUN)	BCG	75.2% (64.9-83.4%)	97.9% (96.2-98.9%)	96.4% (94.6-97.6%)	98.3% (97.4-99.0%)	91.4% (86.4-94.8%)
	DTP1	41.9% (33.0-54.2%)	92.5% (89.9-95.4%)	97.5% (96.4-98.5%)	97.7% (96.5-98.7%)	96.2% (94.1-97.8%)
	DTP3	36.4% (29.1-43.4%)	88.9% (86.4-91.0%)	96.2% (95.1-97.1%)	95.8% (94.7-96.8%)	95.6% (93.5-97.2%)
	HepB3	-	-	88.2% (85.2-90.8%)	95.0% (93.4-96.3%)	95.5% (93.4-97.1%)
	Hib3	-	-	-	-	95.3% (93.2-97.0%)
	MCV1	67.4% (57.3-76.7%)	93.1% (89.5-95.6%)	92.8% (88.8-95.8%)	95.1% (92.8-96.9%)	95.3% (91.5-97.6%)
	MCV2	-	92.9% (89.3-95.5%)	92.7% (88.6-95.7%)	94.9% (92.6-96.7%)	94.7% (91.0-97.1%)
	PCV3	-	-	-	-	90.3% (87.3-92.8%)
	Pol3	37.8% (30.7-45.6%)	88.7% (86.0-91.0%)	94.6% (93.0-95.9%)	95.4% (94.2-96.4%)	95.4% (92.9-97.2%)
	RCV1	-	-	-	-	95.2% (91.4-97.5%)
	RotaC	-	-	-	-	-
Turkey (TUR)	BCG	67.9% (52.4-81.1%)	48.1% (36.6-59.1%)	90.6% (84.4-95.0%)	94.6% (89.3-97.7%)	91.8% (81.5-97.2%)
	DTP1	67.1% (57.6-76.2%)	86.6% (83.2-89.8%)	84.7% (80.1-88.8%)	96.2% (94.5-97.5%)	96.1% (93.0-98.2%)
	DTP3	59.6% (50.5-68.3%)	78.9% (74.6-82.8%)	62.4% (58.5-66.1%)	94.2% (91.9-96.1%)	94.0% (90.3-96.8%)
	HepB3	-	-	52.1% (47.6-56.5%)	92.5% (89.2-94.9%)	93.9% (90.2-96.7%)
	Hib3	-	-	-	93.8% (91.4-95.7%)	93.9% (90.2-96.7%)
	MCV1	46.3% (36.6-56.8%)	84.2% (78.3-89.1%)	87.7% (83.8-91.0%)	89.7% (86.1-92.9%)	90.5% (85.2-94.7%)
	MCV2	-	-	87.5% (83.6-90.8%)	80.5% (75.2-85.2%)	80.9% (74.4-86.7%)
	PCV3	-	-	-	93.1% (90.7-95.0%)	93.4% (89.7-96.1%)

Country	Vaccine	1980	1990	2000	2010	2019
	Pol3	75.7% (68.1-82.5%)	80.4% (76.9-83.5%)	68.1% (65.1-71.0%)	88.2% (85.6-90.5%)	87.9% (84.1-91.1%)
	RCV1	-	-	-	89.6% (86.0-92.8%)	90.4% (85.1-94.6%)
	RotaC	-	-	-	-	-
Tuvalu (TUV)	BCG	93.4% (89.2-96.4%)	92.7% (89.7-95.1%)	98.3% (96.6-99.3%)	99.2% (97.8-99.8%)	99.5% (98.5-99.9%)
	DTP1	53.7% (43.1-65.3%)	89.5% (85.2-93.0%)	92.4% (88.2-95.8%)	97.5% (95.3-98.9%)	98.5% (96.2-99.6%)
	DTP3	46.3% (37.1-55.9%)	82.6% (77.8-86.5%)	85.2% (78.9-90.4%)	91.3% (86.6-95.1%)	91.0% (84.2-95.7%)
	HepB3	-	-	75.4% (66.9-82.2%)	90.9% (86.2-94.5%)	90.8% (84.0-95.5%)
	Hib3	-	-	-	91.0% (86.4-94.8%)	90.9% (84.0-95.6%)
	MCV1	68.2% (48.1-85.5%)	75.7% (68.1-82.1%)	86.9% (78.4-92.8%)	89.4% (82.8-93.9%)	93.2% (86.9-97.2%)
	MCV2	-	-	-	76.8% (69.3-82.8%)	88.7% (81.0-93.5%)
	PCV3	-	-	-	-	-
	Pol3	40.2% (31.4-50.1%)	75.5% (69.8-80.7%)	86.2% (81.1-90.4%)	90.3% (85.1-93.8%)	90.2% (83.5-94.6%)
	RCV1	-	-	-	89.3% (82.7-93.8%)	93.1% (86.8-97.1%)
	RotaC	-	-	-	-	-
Taiwan (province of China) (TWN)	BCG	75.6% (39.6-95.3%)	91.5% (72.6-98.7%)	96.1% (86.1-99.5%)	98.0% (93.2-99.7%)	98.3% (94.1-99.8%)
	DTP1	80.2% (57.3-94.6%)	95.4% (85.5-99.3%)	95.7% (88.0-98.9%)	97.6% (93.8-99.4%)	98.0% (94.8-99.5%)
	DTP3	73.1% (46.9-91.4%)	89.6% (75.0-97.2%)	95.1% (86.2-98.7%)	97.4% (93.5-99.3%)	97.8% (94.4-99.5%)
	HepB3	-	86.8% (71.8-95.4%)	94.4% (85.6-98.2%)	97.4% (93.4-99.2%)	97.8% (94.4-99.4%)
	Hib3	-	-	94.6% (85.8-98.3%)	97.3% (93.3-99.2%)	97.8% (94.4-99.4%)
	MCV1	82.3% (55.3-95.9%)	98.1% (93.3-99.7%)	99.6% (98.8-99.9%)	99.8% (99.4-100.0%)	99.8% (99.3-100.0%)
	MCV2	-	-	-	99.6% (99.2-99.8%)	99.4% (98.8-99.7%)
	PCV3	-	-	-	94.3% (90.3-96.6%)	97.7% (94.3-99.4%)
	Pol3	76.4% (51.9-92.7%)	90.4% (75.6-97.2%)	95.3% (88.1-98.7%)	97.4% (94.0-99.2%)	97.8% (94.8-99.4%)
	RCV1	-	97.7% (92.9-99.3%)	99.4% (98.5-99.7%)	99.6% (99.2-99.8%)	99.7% (99.3-99.9%)
	RotaC	-	-	-	-	-
Tanzania (TZA)	BCG	67.2% (58.9-74.9%)	92.3% (90.8-93.6%)	92.9% (91.4-94.1%)	98.0% (97.3-98.5%)	91.4% (89.3-93.0%)
	DTP1	57.6% (48.7-68.2%)	88.3% (85.2-91.1%)	91.8% (89.9-93.8%)	97.2% (96.1-98.0%)	94.4% (91.8-96.6%)
	DTP3	48.0% (40.8-55.7%)	77.6% (75.4-79.8%)	84.0% (82.2-85.8%)	92.8% (91.3-94.0%)	89.5% (86.6-91.7%)
	HepB3	-	-	-	92.7% (91.1-93.9%)	89.3% (86.5-91.6%)
	Hib3	-	-	-	92.7% (91.2-93.9%)	89.4% (86.6-91.6%)
	MCV1	48.0% (37.6-57.5%)	81.0% (77.2-84.3%)	82.9% (79.4-86.0%)	91.3% (88.0-93.9%)	88.2% (84.1-91.7%)
	MCV2	-	-	-	-	75.7% (68.8-81.5%)
	PCV3	-	-	-	-	88.8% (86.0-91.1%)
	Pol3	45.5% (37.7-53.5%)	73.0% (70.2-76.0%)	79.6% (76.9-82.3%)	86.1% (83.4-88.5%)	77.2% (73.3-81.0%)
	RCV1	-	-	-	-	88.1% (84.0-91.6%)
	RotaC	-	-	-	-	89.3% (86.5-91.5%)
Uganda (UGA)	BCG	37.0% (26.1-48.9%)	84.6% (81.7-87.2%)	83.9% (81.1-86.3%)	94.2% (92.1-95.9%)	97.3% (94.8-98.8%)
	DTP1	41.7% (28.0-58.1%)	83.1% (77.9-87.3%)	78.5% (73.7-83.0%)	91.3% (88.3-93.8%)	93.6% (88.9-96.9%)
	DTP3	19.3% (11.3-30.2%)	61.7% (55.3-67.8%)	53.4% (47.6-59.0%)	77.5% (71.7-82.6%)	72.8% (61.8-81.9%)
	HepB3	-	-	-	76.9% (71.1-82.1%)	72.7% (61.7-81.7%)
	Hib3	-	-	-	77.3% (71.5-82.3%)	72.7% (61.7-81.7%)
	MCV1	38.3% (22.3-56.7%)	68.2% (60.3-75.6%)	66.2% (59.5-72.9%)	84.3% (78.8-88.5%)	73.1% (61.8-82.5%)
	MCV2	-	-	-	-	-
	PCV3	-	-	-	-	65.2% (54.8-73.7%)
	Pol3	18.3% (10.6-29.3%)	59.0% (51.5-66.1%)	56.8% (50.9-63.0%)	65.4% (58.7-71.3%)	56.3% (45.0-67.9%)
	RCV1	-	-	-	-	73.0% (61.7-82.4%)
	RotaC	-	-	-	-	71.7% (60.9-80.5%)
Ukraine (UKR)	BCG	87.3% (68.6-96.8%)	91.0% (86.0-94.9%)	96.2% (94.5-97.5%)	91.5% (89.5-93.4%)	85.5% (79.3-90.8%)
	DTP1	57.4% (46.9-68.0%)	90.3% (86.3-93.2%)	99.4% (98.9-99.7%)	86.6% (83.6-89.2%)	96.4% (94.3-97.9%)
	DTP3	50.1% (39.8-60.7%)	84.6% (78.9-88.9%)	95.7% (93.6-97.3%)	72.7% (68.5-76.6%)	93.6% (90.6-96.1%)
	HepB3	-	-	-	54.6% (42.5-64.3%)	90.6% (84.0-94.4%)
	Hib3	-	-	-	65.2% (57.7-71.0%)	92.1% (88.6-94.7%)
	MCV1	86.1% (64.9-97.2%)	89.8% (82.1-95.0%)	93.1% (89.0-95.9%)	79.3% (73.4-84.0%)	99.0% (97.8-99.6%)
	MCV2	-	-	-	67.0% (59.3-73.6%)	95.3% (91.4-97.5%)

Country	Vaccine	1980	1990	2000	2010	2019
	PCV3	-	-	-	-	-
	Pol3	76.4% (57.1-90.3%)	81.9% (75.1-87.5%)	90.8% (88.4-93.0%)	69.7% (65.8-73.6%)	77.3% (69.5-83.5%)
	RCV1	-	-	-	79.1% (73.3-83.7%)	97.1% (93.3-98.8%)
	RotaC	-	-	-	-	-
Uruguay (URY)	BCG	60.4% (51.6-69.7%)	99.3% (98.4-99.8%)	99.7% (99.3-100.0%)	99.8% (99.4-100.0%)	99.2% (97.6-99.8%)
	DTP1	60.7% (52.5-69.0%)	96.7% (95.0-97.9%)	95.9% (94.1-97.3%)	98.0% (96.8-98.8%)	97.7% (95.1-99.0%)
	DTP3	55.2% (47.5-62.7%)	90.3% (87.5-92.6%)	91.6% (88.9-94.0%)	94.3% (91.7-96.2%)	93.0% (88.4-96.0%)
	HepB3	-	-	91.5% (88.7-93.9%)	94.1% (91.5-96.0%)	92.9% (88.4-95.9%)
	Hib3	-	-	89.2% (85.9-92.1%)	94.1% (91.6-96.1%)	92.9% (88.3-95.9%)
	MCV1	51.2% (40.5-62.4%)	91.1% (86.5-94.3%)	95.1% (91.4-97.5%)	96.5% (92.8-98.5%)	97.6% (94.1-99.3%)
	MCV2	-	-	89.1% (83.1-93.7%)	92.1% (87.6-95.5%)	97.0% (93.6-98.8%)
	PCV3	-	-	-	89.8% (87.0-92.1%)	92.9% (88.3-95.9%)
	Pol3	60.2% (52.4-67.4%)	89.9% (86.9-92.3%)	92.0% (89.3-94.3%)	94.5% (91.9-96.4%)	92.8% (88.4-96.1%)
	RCV1	-	90.9% (86.3-94.2%)	94.9% (91.3-97.4%)	96.4% (92.7-98.4%)	97.5% (94.0-99.2%)
	RotaC	-	-	-	-	-
USA (USA)	BCG	-	-	-	-	-
	DTP1	100.0% (99.9-100.0%)	98.1% (96.7-99.1%)	98.5% (98.0-98.9%)	97.8% (97.2-98.2%)	96.5% (94.9-97.8%)
	DTP3	99.8% (99.4-99.9%)	95.2% (93.2-96.8%)	94.9% (94.0-95.7%)	94.8% (93.9-95.5%)	92.9% (90.5-95.0%)
	HepB3	-	-	89.6% (87.5-91.4%)	90.4% (88.2-92.2%)	90.4% (86.7-93.2%)
	Hib3	-	-	93.2% (92.0-94.2%)	91.5% (90.0-92.7%)	90.5% (87.7-93.1%)
	MCV1	95.8% (90.5-98.4%)	91.4% (85.9-95.0%)	93.1% (91.5-94.6%)	93.4% (91.8-94.7%)	91.8% (86.6-95.4%)
	MCV2	-	65.4% (49.8-77.7%)	84.9% (80.3-88.4%)	90.2% (87.3-92.5%)	91.7% (86.5-95.2%)
	PCV3	-	-	38.0% (33.9-42.4%)	92.5% (91.4-93.4%)	91.9% (89.5-94.0%)
	Pol3	99.7% (99.4-99.9%)	91.4% (88.2-94.0%)	89.0% (87.6-90.3%)	93.0% (91.7-94.1%)	92.0% (89.2-94.3%)
	RCV1	93.5% (89.9-95.6%)	91.2% (85.8-94.6%)	92.8% (91.2-94.3%)	92.7% (91.3-94.0%)	91.7% (86.4-95.2%)
	RotaC	-	-	-	57.9% (56.6-59.1%)	74.1% (71.8-76.2%)
Uzbekistan (UZB)	BCG	93.7% (84.9-98.3%)	92.6% (89.3-95.3%)	98.2% (97.2-98.8%)	99.8% (99.6-99.9%)	99.9% (99.5-100.0%)
	DTP1	98.3% (96.0-99.5%)	98.3% (97.2-99.0%)	98.9% (98.0-99.5%)	92.8% (91.0-94.5%)	93.7% (90.8-96.6%)
	DTP3	98.0% (95.4-99.4%)	98.0% (96.8-98.9%)	95.3% (93.7-96.6%)	92.1% (90.4-93.6%)	91.8% (88.7-94.3%)
	HepB3	-	-	8.3% (3.6-15.8%)	91.4% (89.2-93.1%)	91.6% (88.4-94.0%)
	Hib3	-	-	-	91.5% (89.7-93.1%)	91.7% (88.6-94.1%)
	MCV1	99.1% (96.9-99.8%)	99.4% (98.6-99.8%)	95.8% (94.2-97.1%)	91.7% (89.6-93.7%)	92.5% (89.1-95.0%)
	MCV2	-	98.8% (97.8-99.4%)	95.5% (93.8-96.7%)	88.5% (85.2-91.3%)	92.4% (88.9-95.0%)
	PCV3	-	-	-	-	90.2% (87.2-92.7%)
	Pol3	98.2% (96.3-99.3%)	84.9% (80.3-88.6%)	95.2% (93.6-96.5%)	91.7% (90.0-93.1%)	92.5% (89.9-94.8%)
	RCV1	-	-	-	91.6% (89.4-93.6%)	92.4% (89.0-94.9%)
	RotaC	-	-	-	-	46.9% (43.8-49.9%)
Saint Vincent and the Grenadines (VCT)	BCG	41.6% (23.4-61.4%)	98.6% (97.4-99.3%)	98.7% (97.5-99.4%)	94.3% (91.0-96.6%)	93.4% (87.7-96.9%)
	DTP1	39.1% (31.4-47.4%)	99.4% (99.1-99.7%)	98.3% (97.1-99.0%)	97.5% (96.0-98.5%)	98.2% (96.7-99.1%)
	DTP3	34.2% (27.0-42.0%)	97.9% (96.7-98.8%)	97.8% (96.4-98.8%)	97.3% (95.6-98.4%)	97.4% (95.3-98.7%)
	HepB3	-	-	-	96.7% (94.5-98.1%)	97.2% (95.2-98.5%)
	Hib3	-	-	-	96.9% (95.3-98.0%)	97.2% (95.2-98.5%)
	MCV1	42.2% (25.7-59.6%)	97.8% (95.3-99.2%)	93.4% (88.5-96.7%)	99.1% (97.8-99.8%)	99.7% (99.2-99.9%)
	MCV2	-	-	93.0% (88.1-96.3%)	93.7% (90.8-96.0%)	99.6% (99.0-99.8%)
	PCV3	-	-	-	-	-
	Pol3	35.2% (27.0-44.5%)	94.7% (91.8-96.8%)	97.2% (95.1-98.5%)	96.5% (93.9-97.9%)	97.1% (94.4-98.8%)
	RCV1	-	-	93.2% (88.4-96.6%)	99.0% (97.7-99.6%)	99.6% (99.1-99.8%)
	RotaC	-	-	-	-	-
Venezuela (VEN)	BCG	74.4% (66.2-81.9%)	83.0% (77.6-87.2%)	92.7% (90.1-94.7%)	87.1% (82.5-91.4%)	87.3% (78.0-93.6%)
	DTP1	77.0% (68.9-84.3%)	83.4% (79.0-87.2%)	87.2% (83.8-90.5%)	87.4% (83.2-91.0%)	88.7% (82.8-93.4%)
	DTP3	56.9% (47.0-65.6%)	63.7% (57.6-69.1%)	66.7% (61.4-71.5%)	78.4% (73.1-83.4%)	69.7% (60.1-78.2%)
	HepB3	-	-	23.6% (8.9-43.7%)	78.3% (72.9-83.2%)	69.6% (60.0-78.2%)
	Hib3	-	-	10.2% (5.4-17.4%)	78.3% (72.9-83.2%)	69.6% (60.0-78.2%)
	MCV1	31.4% (21.1-42.5%)	40.0% (32.6-48.0%)	60.4% (54.2-66.6%)	58.5% (51.9-65.0%)	60.8% (51.6-69.7%)

Country	Vaccine	1980	1990	2000	2010	2019
	MCV2	-	-	-	17.5% (12.2-24.1%)	20.5% (13.7-27.8%)
	PCV3	-	-	-	-	-
	Pol3	63.9% (56.9-70.5%)	52.9% (47.9-58.4%)	56.0% (51.8-60.0%)	52.9% (47.2-58.1%)	45.9% (37.7-54.9%)
	RCV1	-	-	59.7% (53.5-65.8%)	58.4% (51.8-64.9%)	60.7% (51.5-69.6%)
	RotaC	-	-	-	52.9% (48.8-57.1%)	-
Virgin Islands (VIR)	BCG	-	-	-	-	-
	DTP1	76.9% (48.9-94.8%)	85.2% (63.4-96.9%)	89.9% (77.2-97.1%)	86.2% (81.2-90.5%)	78.8% (62.0-91.1%)
	DTP3	49.9% (20.2-78.9%)	66.9% (39.0-87.1%)	78.6% (61.0-90.9%)	74.7% (69.6-79.3%)	66.0% (48.0-81.7%)
	HepB3	-	57.4% (30.9-79.8%)	76.3% (58.7-89.1%)	74.2% (68.9-78.8%)	66.0% (48.0-81.7%)
	Hib3	-	-	63.9% (45.4-79.4%)	71.0% (65.2-76.3%)	65.7% (47.8-81.3%)
	MCV1	39.6% (11.5-73.1%)	56.6% (22.4-86.5%)	72.7% (43.8-92.0%)	73.4% (66.4-79.9%)	70.3% (39.6-90.8%)
	MCV2	-	-	-	-	-
	PCV3	-	-	26.1% (18.2-34.2%)	69.3% (64.4-73.8%)	65.7% (47.8-81.4%)
	Pol3	47.8% (21.4-72.3%)	63.5% (36.9-85.2%)	75.1% (58.5-88.4%)	72.4% (66.8-77.4%)	64.8% (44.5-80.8%)
	RCV1	-	-	-	-	-
	RotaC	-	-	-	18.7% (16.7-20.9%)	60.2% (43.9-74.5%)
Vietnam (VNM)	BCG	20.7% (11.2-33.4%)	86.2% (82.6-89.2%)	91.6% (89.6-93.4%)	96.0% (94.1-97.4%)	94.8% (90.6-97.5%)
	DTP1	22.2% (12.0-38.3%)	85.0% (80.8-88.8%)	91.4% (88.4-93.9%)	87.3% (83.7-90.5%)	91.6% (86.3-95.7%)
	DTP3	13.7% (7.3-22.6%)	78.0% (73.4-81.8%)	76.6% (73.3-79.8%)	79.0% (75.4-82.2%)	80.5% (74.2-86.1%)
	HepB3	-	-	28.0% (18.8-38.4%)	75.4% (70.7-79.3%)	80.3% (74.0-86.0%)
	Hib3	-	-	-	69.3% (61.9-75.2%)	80.3% (74.1-85.9%)
	MCV1	4.8% (1.5-11.1%)	92.6% (89.5-95.1%)	84.0% (80.5-87.2%)	92.5% (89.3-94.8%)	89.6% (83.5-94.0%)
	MCV2	-	-	-	91.1% (87.8-93.6%)	85.2% (78.6-89.9%)
	PCV3	-	-	-	-	-
	Pol3	17.2% (8.7-29.2%)	85.6% (81.8-88.8%)	71.8% (67.7-75.3%)	78.7% (74.1-82.6%)	82.7% (75.5-88.6%)
	RCV1	-	-	-	-	85.8% (79.7-90.3%)
	RotaC	-	-	-	-	-
Vanuatu (VUT)	BCG	40.5% (29.0-52.1%)	79.8% (74.6-84.2%)	78.8% (73.7-83.1%)	75.3% (70.6-79.7%)	94.1% (88.9-97.4%)
	DTP1	29.7% (19.0-43.6%)	68.6% (62.9-74.4%)	87.6% (83.4-91.3%)	81.0% (76.0-85.3%)	95.2% (92.2-97.3%)
	DTP3	18.3% (11.1-27.0%)	55.3% (50.3-60.9%)	68.5% (64.1-72.9%)	60.6% (56.2-64.8%)	88.2% (82.6-92.3%)
	HepB3	-	31.9% (19.8-42.9%)	51.9% (43.8-59.8%)	60.0% (55.6-64.3%)	88.1% (82.5-92.2%)
	Hib3	-	-	-	-	88.1% (82.5-92.3%)
	MCV1	12.2% (4.3-24.9%)	32.5% (24.6-40.9%)	48.6% (41.5-55.5%)	53.7% (45.7-61.0%)	70.7% (59.6-80.4%)
	MCV2	-	-	-	-	-
	PCV3	-	-	-	-	-
	Pol3	14.7% (9.1-22.6%)	54.8% (49.4-59.9%)	61.9% (57.2-66.6%)	57.3% (52.6-61.8%)	88.2% (83.1-92.2%)
	RCV1	-	-	-	-	70.3% (59.4-80.0%)
	RotaC	-	-	-	-	-
Samoa (WSM)	BCG	75.0% (67.3-81.1%)	87.3% (84.2-90.0%)	91.4% (89.1-93.4%)	87.3% (84.8-89.6%)	89.8% (85.8-93.1%)
	DTP1	45.3% (34.9-57.2%)	72.1% (63.8-80.4%)	85.2% (78.5-90.9%)	86.3% (81.1-90.5%)	81.1% (70.3-90.9%)
	DTP3	26.8% (20.1-33.7%)	40.1% (35.9-44.5%)	42.8% (39.5-46.0%)	50.6% (46.5-54.3%)	53.6% (46.0-61.4%)
	HepB3	-	10.0% (3.7-18.9%)	41.9% (38.6-45.2%)	50.3% (46.3-54.0%)	53.5% (45.9-61.2%)
	Hib3	-	-	-	50.4% (46.4-54.0%)	53.5% (45.9-61.2%)
	MCV1	53.2% (34.7-72.7%)	69.7% (62.1-76.4%)	77.4% (70.4-83.3%)	56.1% (48.3-64.0%)	79.1% (68.5-87.2%)
	MCV2	-	-	-	41.4% (34.2-49.3%)	61.8% (51.2-71.7%)
	PCV3	-	-	-	-	-
	Pol3	24.0% (18.2-30.6%)	35.2% (31.3-39.1%)	36.8% (33.8-39.8%)	48.1% (44.5-51.5%)	50.3% (42.8-57.5%)
	RCV1	-	-	-	56.0% (48.2-63.9%)	79.0% (68.5-87.2%)
	RotaC	-	-	-	-	-
Yemen (YEM)	BCG	15.1% (9.3-22.4%)	58.0% (52.4-63.4%)	64.8% (58.8-70.4%)	69.1% (64.0-73.8%)	74.2% (64.7-82.3%)
	DTP1	18.5% (10.4-30.6%)	64.6% (57.0-72.0%)	73.1% (68.5-77.6%)	78.4% (74.1-82.7%)	78.1% (69.9-85.1%)
	DTP3	4.5% (2.0-8.8%)	39.7% (32.7-47.2%)	58.6% (52.4-64.3%)	61.9% (55.8-67.8%)	63.5% (53.6-73.0%)
	HepB3	-	-	12.0% (8.4-16.0%)	61.8% (55.6-67.7%)	63.4% (53.5-73.0%)
	Hib3	-	-	-	61.8% (55.7-67.6%)	63.4% (53.5-73.0%)

Country	Vaccine	1980	1990	2000	2010	2019
	MCV1	4.4% (1.5-10.0%)	44.1% (36.6-51.7%)	36.4% (30.1-42.8%)	69.5% (63.5-75.2%)	70.1% (59.6-79.3%)
	MCV2	-	-	-	46.5% (39.5-53.0%)	51.8% (41.1-61.2%)
	PCV3	-	-	-	-	63.2% (53.3-72.6%)
	Pol3	4.4% (2.0-8.0%)	41.3% (35.8-47.0%)	68.8% (64.0-73.4%)	58.9% (53.8-64.0%)	61.3% (52.5-69.7%)
	RCV1	-	-	-	-	70.0% (59.5-79.2%)
	RotaC	-	-	-	-	63.4% (53.5-72.9%)
South Africa (ZAF)	BCG	82.3% (67.3-91.7%)	80.4% (75.4-84.7%)	88.4% (85.7-90.7%)	86.0% (81.9-89.7%)	89.9% (85.0-93.9%)
	DTP1	78.2% (66.6-87.7%)	85.4% (81.8-88.8%)	86.4% (82.6-89.5%)	84.5% (79.6-89.8%)	71.4% (63.5-79.6%)
	DTP3	69.7% (57.6-80.2%)	77.7% (73.7-81.6%)	76.8% (73.2-80.4%)	70.1% (66.1-73.9%)	62.8% (56.1-69.5%)
	HepB3	-	-	72.7% (67.0-77.0%)	68.6% (64.0-72.7%)	62.7% (56.1-69.4%)
	Hib3	-	-	71.6% (65.4-76.2%)	70.0% (66.0-73.8%)	62.8% (56.1-69.4%)
	MCV1	75.8% (56.4-90.2%)	87.0% (81.1-91.9%)	79.1% (73.3-84.4%)	82.6% (77.1-87.2%)	78.2% (69.3-85.3%)
	MCV2	-	-	64.9% (56.7-71.8%)	70.6% (63.9-76.2%)	72.6% (63.9-80.2%)
	PCV3	-	-	-	57.4% (52.7-62.3%)	62.7% (56.0-69.4%)
	Pol3	70.7% (59.2-81.3%)	74.7% (70.5-78.4%)	72.9% (68.8-76.6%)	75.4% (71.4-79.2%)	69.3% (63.0-75.7%)
	RCV1	-	-	-	-	-
	RotaC	-	-	-	63.4% (59.7-66.9%)	62.1% (55.5-68.7%)
Zambia (ZMB)	BCG	68.1% (55.0-79.8%)	91.7% (89.3-93.6%)	92.8% (91.0-94.5%)	95.0% (93.1-96.5%)	99.5% (98.7-99.8%)
	DTP1	91.0% (82.0-96.7%)	91.4% (89.3-93.2%)	93.3% (91.5-94.7%)	94.9% (93.1-96.2%)	94.0% (90.6-96.7%)
	DTP3	53.9% (42.2-65.8%)	79.1% (76.2-81.9%)	78.8% (76.0-81.4%)	87.5% (84.7-90.0%)	89.0% (83.4-93.5%)
	HepB3	-	-	-	86.9% (83.8-89.6%)	88.9% (83.3-93.3%)
	Hib3	-	-	-	87.0% (84.1-89.5%)	88.9% (83.3-93.4%)
	MCV1	55.8% (34.6-75.0%)	78.9% (74.9-82.6%)	87.6% (84.3-90.5%)	88.8% (85.5-92.0%)	90.9% (84.1-95.4%)
	MCV2	-	-	-	-	63.7% (51.6-72.9%)
	PCV3	-	-	-	-	88.9% (83.3-93.4%)
	Pol3	50.1% (36.6-61.9%)	76.2% (72.6-79.7%)	78.7% (75.5-81.5%)	80.4% (76.9-83.7%)	88.0% (82.3-92.7%)
	RCV1	-	-	-	-	90.8% (84.1-95.3%)
	RotaC	-	-	-	-	88.9% (83.3-93.4%)
Zimbabwe (ZWE)	BCG	75.2% (62.7-85.5%)	93.3% (90.0-95.6%)	74.8% (70.8-78.8%)	91.5% (89.3-93.3%)	94.0% (89.3-96.8%)
	DTP1	61.4% (50.8-72.6%)	96.0% (94.4-97.2%)	79.7% (76.4-82.9%)	91.6% (89.7-93.2%)	94.6% (91.8-96.7%)
	DTP3	49.4% (38.7-59.9%)	89.4% (86.1-92.0%)	66.7% (62.4-70.6%)	81.5% (78.4-84.2%)	90.7% (86.3-94.1%)
	HepB3	-	-	51.9% (45.9-57.8%)	81.0% (77.8-83.8%)	90.5% (86.2-93.9%)
	Hib3	-	-	-	81.4% (78.3-84.2%)	90.6% (86.2-94.0%)
	MCV1	72.3% (55.7-84.3%)	92.9% (88.2-96.0%)	70.8% (64.5-76.6%)	85.2% (80.7-89.0%)	85.4% (76.9-91.7%)
	MCV2	-	-	-	-	72.8% (63.1-80.8%)
	PCV3	-	-	-	-	90.6% (86.2-94.0%)
	Pol3	58.1% (46.6-69.6%)	85.6% (81.8-89.0%)	61.6% (57.7-65.3%)	82.2% (79.4-84.8%)	89.0% (84.4-92.7%)
	RCV1	-	-	-	-	85.3% (76.8-91.6%)
	RotaC	-	-	-	-	90.5% (86.1-93.8%)

Supplementary table 8. Concordance correlation coefficients, by vaccine, for WUENIC- and GBD-based coverage estimates. Reported coefficient values are based on concordance estimated between matched location-year estimates from 1980 to 2019 from each source. WUENIC=WHO-UNICEF Estimates of National Immunization Coverage. GBD=Global Burden of Disease. CCC=Concordance correlation coefficient. BCG=Bacillus Calmette-Guérin. DTP1=diphtheria-tetanus-pertussis, first dose. DTP3=diphtheria-tetanus-pertussis, third dose. HepB3=hepatitis B vaccine, third dose. Hib3=*Haemophilus influenzae* type b vaccine, third dose. MCV1=measles-containing vaccine, first dose. MCV2=measles-containing vaccine, second dose. PCV3=pneumococcal conjugate vaccine, third dose. Pol3=polio vaccine, third dose. RCV1=rubella-containing vaccine, first dose. RotaC=completed rotavirus series.

Vaccine	CCC
BCG	0.91
DTP1	0.90
DTP3	0.92
HepB3	0.84
Hib3	0.85
MCV1	0.92
MCV2	0.85
PCV3	0.87
Pol3	0.85
RCV1	0.74
RotaC	0.80
